# Identifying Molecular Effects of Diet through Systems Biology: Influence of Herring Diet on Sterol Metabolism and Protein Turnover in Mice

**DOI:** 10.1371/journal.pone.0012361

**Published:** 2010-08-24

**Authors:** Intawat Nookaew, Britt G. Gabrielsson, Agneta Holmäng, Ann-Sofie Sandberg, Jens Nielsen

**Affiliations:** 1 Life Sciences/Systems Biology, Department of Chemical and Biological Engineering, Chalmers University of Technology, Gothenburg, Sweden; 2 Life Sciences/Food Science, Department of Chemical and Biological Engineering, Chalmers University of Technology, Gothenburg, Sweden; 3 Department of Physiology, Institute of Neuroscience and Physiology, University of Gothenburg, Gothenburg, Sweden; New Mexico State University, United States of America

## Abstract

**Background:**

Changes in lifestyle have resulted in an epidemic development of obesity-related diseases that challenge the healthcare systems worldwide. To develop strategies to tackle this problem the focus is on diet to prevent the development of obesity-associated diseases such as cardiovascular disease (CVD). This will require methods for linking nutrient intake with specific metabolic processes in different tissues.

**Methodology/Principal Finding:**

Low-density lipoprotein receptor-deficient (*Ldlr* −/−) mice were fed a high fat/high sugar diet to mimic a westernized diet, being a major reason for development of obesity and atherosclerosis. The diets were supplemented with either beef or herring, and matched in macronutrient contents. Body composition, plasma lipids and aortic lesion areas were measured. Transcriptomes of metabolically important tissues, e.g. liver, muscle and adipose tissue were analyzed by an integrated approach with metabolic networks to directly map the metabolic effects of diet in these different tissues. Our analysis revealed a reduction in sterol metabolism and protein turnover at the transcriptional level in herring-fed mice.

**Conclusion:**

This study shows that an integrated analysis of transcriptome data using metabolic networks resulted in the identification of signature pathways. This could not have been achieved using standard clustering methods. In particular, this systems biology analysis could enrich the information content of biomedical or nutritional data where subtle changes in several tissues together affects body metabolism or disease progression. This could be applied to improve diets for subjects exposed to health risks associated with obesity.

## Introduction

**Figure 1:**
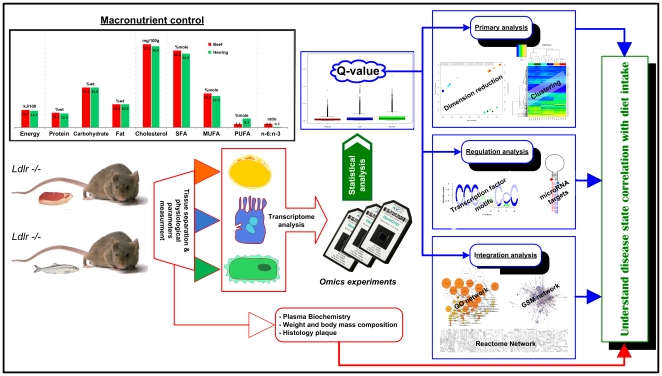
In order to obtain molecular insight into the influence of diet on the metabolism in different tissues, mice were fed with different diets under marcronutrient control. In the study *Ldlr* −/− mice were used, as this allowed for evaluation of how diet influences the development of atherosclerosis. The mice were fed with either a beef-based (B) diet or a herring-based (H) diet. The body weights were monitored weekly, and at the end of the study body composition was measured and aortic plaques were detected by *en face* histology. Furthermore, metabolically important tissues such as liver, muscle and adipose tissue were collected and genome-wide transcription analysis was performed on these samples. After statistical analysis of the data there was performed, in parallel, a standard clustering and dimension reduction analysis with the objective to identify gross patterns within the samples. In the integrated analysis different types of biological network graphs were used. Through this analysis specific metabolic pathways activated in the specific tissues in response to the diet were identified. This information was integrated together with histological data in order to gain new fundamental insight into the molecular effects of diet on whole body metabolism.

More than 40% of adults in the USA are obese and it is expected that by 2030 close to 200 million subjects (corresponding to about 33%) in Europe will be obese. Many of these will develop dyslipidemia, hypertension and glucose intolerance, imposing increased costs for the health care systems. These obesity-related disorders are mainly caused by sedentary lifestyle habits and changes to energy-dense foods with high content of refined carbohydrates and saturated fats. It is therefore of interest to identify nutritional strategies that could reduce the prevalence of these disorders, as this would shift focus from treatment to prevention of diseases. Presently, there is a large interest in the action of isolated bioactive food compounds that have health benefits, e.g. resveratrol and long-chain n-3 polyunsaturated fatty acids (LC n-3 PUFAs) [1], [2]. Although there are numerous studies on the mechanisms of action of such compounds, it is difficult to translate or to explain the known health effects of specific food items. To address this issue we developed a novel concept for data analyses of dietary studies, by directly linking specific food intake with metabolic activities in different tissues. Our approach was to simultaneously analyze the transcriptional responses in three metabolically important tissues; liver, muscle and adipose tissue. Therefore, we could relate responses at the tissue level to whole body metabolic events. A further advantage of our study is using diets that simulated a Westernized diet commonly consumed in modern society, as this allowed us to evaluate the integrative effects of all the components in typical meals. An overview of the concept is illustrated in Figure 1.

Our concept allows direct mapping of the dietary effects on molecular mechanisms in the three tissues individually, and by further linking concerted dietary effects in all three tissues combined, gaining insight into how nutrition influences whole body metabolism. This type of integrated analysis has previously been proposed to be how systems biology methodology could advance nutrigenomics [3]. Through integrated analysis, mapping transcriptome data on metabolic networks and other types of interaction networks we further show that it is possible to identify complete pathway signatures in response to diet. The use of transcriptional profiles in combination with metabolic models has previously been used to identify signature pathways in yeast [4] and in human tissues [5]. Important to our concept is that we perform integrated analysis in three major metabolically important tissues, as this reflects whole body metabolic responses to changes in the diet. In addition, this approach could easily be adapted to incorporate and integrate plasma measurements for identification of biomarkers that reflect specific tissue metabolic activities and can ultimately be used for clinical evaluations.

## Results

### Experimental design

We designed an experiment aimed to evaluate the influence of herring *versus* beef based diet on the development of atherosclerosis. We used the low-density lipoprotein receptor-deficient (*Ldlr−/−*) mouse [6], as this model has a dietary dependence on the development of atherosclerotic plaques [7]. Furthermore, the *Ldlr−/−* mouse is susceptible to diet-induced obesity with concomitant insulin resistance [7], [8] and as such mimics the situation in the Westernized world. Epidemiological studies show that a high dietary intake of fish reduces the incidence of CVD [9], and these effects are usually attributed to the long chain (LC) n-3 polyunsaturated fatty acids (PUFAs) [10]. Indeed, previous studies show that LC n-3 PUFAs supplemented diet reduced plaque formation and hepatic steatosis in the *Ldlr−/−* mouse model [10], [11]. However, the effect of fish intake on risk factors associated to CVD has previously not been investigated in mice. This is of interest since fish protein reduced blood lipid levels in rats, and also affected hepatic expression of genes involved in cholesterol metabolism [12]. Furthermore, a recent clinical study showed that combined treatment with LC n-3 PUFAs and taurine, both present in fish muscle, was more efficient in lowering blood lipid levels than LC n-3 PUFAs alone [13]. In our study, *Ldlr−/−* male mice were given a 16-week high fat/high sucrose diet, supplemented with either minced herring fillets or minced beef, to identify metabolic pathways that were differentially affected in three tissues important for whole body glucose and lipid metabolism; liver, skeletal muscle and white adipose tissue (WAT). The macronutrient composition of the two diets was identical except for the source of protein and fat (Table S1).

### Animal phenotypes

Three animals from each diet-group were selected to represent the whole diet group with respect to weight-gain, body composition and blood lipid levels (for details see Table S2). The plasma total cholesterol and triacylglyceride levels were lower in the mice fed herring compared to those given beef at both time-points (week 8 p-value <0.001 both; week 16 p-value  = 0.017 and 0.002, cholesterol and triglycerides, respectively). Body composition, measured at week 15, showed a trend to increased lean body mass in the herring-fed mice in comparison with the beef-fed (p-value  = 0.050). At week 16, the plaque areas in the aortic arch, assessed by *en face* histology, were significantly lower in the herring-fed mice (p-value <0.001; 0.6±0.1 *versus* 7.4±0.7% area, herring and beef, respectively).

### Primary analysis of transcriptome data

**Figure 2:**
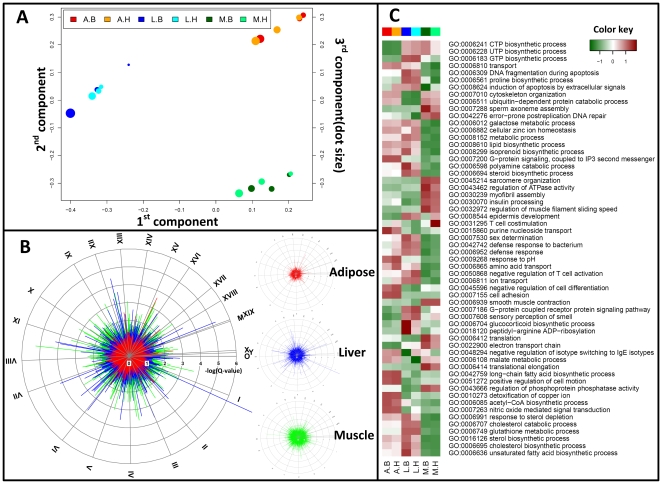
Analysis of transcriptome data. Three mice from each diet group were selected for transcriptome analysis. Liver, muscle and adipose tissue were obtained from these mice, mRNA was extracted from these tissues and the resulting samples were analyzed. **A.** After normalization Single Value Decomposition (SVD) of the data were performed. This analysis points to a very clear separation of the three tissues analyzed, showing that the tissue effect is larger than the diet effect as expected. The SVD analysis points to good consistency between the samples from the three different mice, giving good statistical power for further analysis of the data. **B.** Circular mapping plot of Q-values (p-values obtained from a Student t-test and corrected for multiple testing) according to the transcript loci arrangement on the different chromosomes for each of the three tissues. The plot shows the distribution of Q-values in response to diet. The three smaller plots to the right indicate the Q-values for the three different tissues and were overlaid in the figure to the left (more details in Text S1 and Figure S6, for simple boxplot of Q-values see Figure S2). **C.** For each tissue the reporter Biological Process GO-terms were identified according to the influence of the diets. The reporter GO-terms of cellular component and molecular function category are given in Figure S4. Normalized X-score for all the genes in each GO-term was identified (more details in Text S1). This was done for each of the three tissues in each of the two groups of mice, resulting in a total of 6 categories for each GO-term (3 categories for each GO-terms when consider only tissues factor see Figure S3). The figure illustrates the X-score for each GO-term. The analysis corrects for the size of the group and reporter GO terms with a large number of genes therefore represents a global response, whereas GO terms with few genes represents specific transcriptional changes.

We first performed a singular value decomposition (SVD) of the transcriptome data to evaluate the quality of the microarray experiments (Figure 2A). As expected, the largest separation of the data was based on differences in the three tissues and the tissue effect masked the diet effect. Nevertheless, in each tissue there was a large number of genes that had significantly changed expression in response to diet (Figure 2B). This was also supported by hierarchical clustering of significantly changed genes based on diet effect (Text S1 and Figure S1). As seen in Figure 2B, the most distinct effect of diet, i.e. highest Q-values in the logarithmic scale, was in liver where 344 genes had significantly changed expression. However, larger number of genes (859) in the muscle satisfied the Q-value cut-off for significance of less than 0.05. The gene expression in WAT was less affected by diet, where only 48 genes had significantly changed expression.

### Integrated data analysis

**Figure 3:**
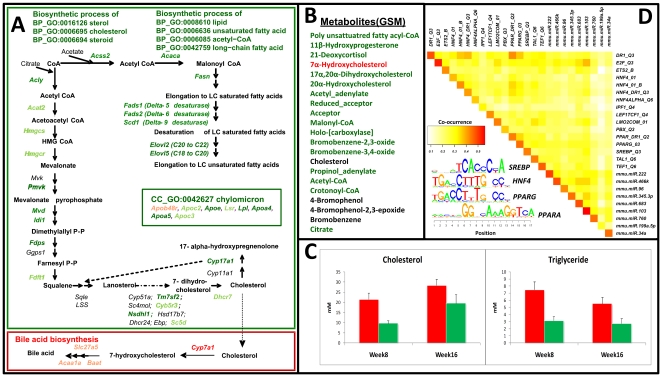
Mapping of metabolic activities in the liver (green and red indicate down- and upregulated based on herring diet, respectively). **A.** Overview of genes involved in sterol and lipid biosynthesis that are downregulated in response to herring diet. Besides identification of a key reporter GO terms it is also seen that most genes in the biosynthetic pathway towards sterols and fatty acids are downregulated. **B.** The downregulation (panel A) is further supported by the identification of several reporter metabolites of the cholesterol and fatty acid biosynthesis. **C.** Measurements of cholesterol and triacylglyceride in the plasma. It is seen that the levels of both are down in the mice fed with the herring diet, and this effect is seen both after 8 and 16 weeks of feeding. **D.** For all downregulated genes identified in the reporter GO terms (panel A) there was searched for enrichment of transcription factors and microRNAs. The heat map shows identified transcription factors and microRNAs and their co-occurrence matrix. It is observed that most regulatory effects are due to a single factor. For some of the identified transcription factors the corresponding consensus binding sites were identified, and this resulted in identification of consensus binding sites for Srebf (*Srebp*), *Hnf4*, *Pparg* and *Ppara*. The Ppar systems are important lipid-activated nuclear receptors involved in lipid and glucose metabolism; *Pparg* is an important transcription factor in adipocytes and Ppara in hepatocytes. *Hnf4a* is an important regulator of coordinated nuclear receptor-mediated response to xenobiotics through interaction with *Cars/Pxr* and through *Hnf1* it activates the expression of a large number of liver-specific genes, including those involved in glucose, cholesterol, and fatty acid metabolism. The most frequent binding site for microRNAs is the site mmu.miR.103 which implies its contribution to transcriptional inhibition of hepatic lipid synthesis (see Text S1and Figure S7).

**Figure 4:**
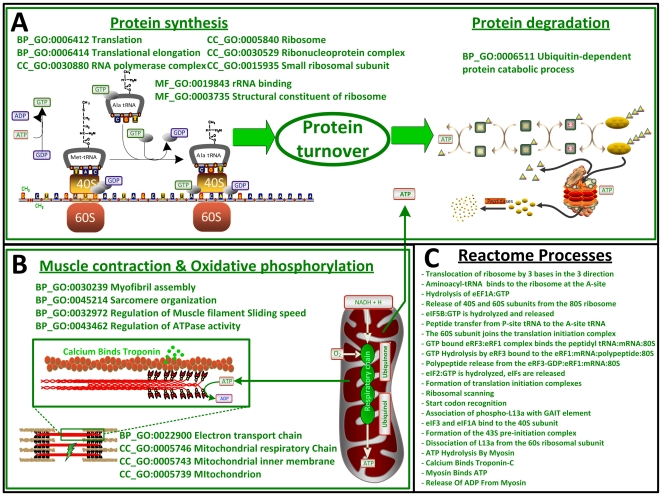
Mapping of metabolic activities in the muscle (green and red indicate down- and upregulated based on herring diet, respectively). **A.** Reporter GO terms resulted in the identification of several key processes involved in protein biosynthesis and protein degradation, and genes associated with these processes are downregulated in response to the herring diet. This points a reduced protein turn-over in response to a herring diet. **B.** Reporter GO terms also show that there is downregulation of genes associated with oxidative stress and muscle contraction in response to a herring diet. This indicates more efficient energy utilization, and the reduced oxidative stress may cause reduced protein misfolding and hence reduced protein turn-over. **C.** Identification of reporter Reactome processes points to the same overall function and allows for identification of even more specific processes affected by the diet, e.g. start site recognition and binding of activated tRNAs to the ribosome.

In order to identify key biological processes affected by diet in liver, skeletal muscle and WAT, we performed integrated analysis using three biological networks to capture different levels of information. The first analysis provides a global view of the response to diet, identifying significant Gene Ontology (GO)-terms (Figure 2C). We applied the reporter algorithm [4], [14], rather than using the traditional hypogeometric test, since it has the advantage in using the Q-value for all transcripts. The reporter algorithm made it possible to identify key biological processes affected by diet in the three tissues (Figure 2C; for GO “cellular component” and “molecular function” see Figure S4). In liver, the GO biological processes that were affected by diet were related to lipid/sterol metabolism, e.g. “lipid biosynthetic process” (99 transcripts), “sterol biosynthetic process” (25 transcripts) and “cholesterol biosynthetic process” (23 transcripts). Similarly, in muscle and WAT, the GO biological processes affected by diet were translation (321 transcripts, muscle), cell adhesion (419 transcripts, WAT) and defense response to bacterium (77 transcripts, WAT). To further characterize dietary effects on metabolism by the reporter algorithm [4], [14], we also determined key metabolites, using a generic genome-scale metabolic model (GSMM) for mouse [15], and key Reactome processes, using curated evidences from the Reactome database [16]. From this analysis, metabolites related to fatty acid/sterol biosynthesis were identified as the major responses in liver (Figure 3B). Furthermore, specific biological processes related to protein turnover were identified in muscle (Figure 4C). To further identify molecular mechanisms that were triggered by diet, we screened for overrepresentation of regulatory targets, either in the promoter regions or microRNA targets, to identify putative regulatory drivers for the previously identified changes. Hereby, we were able to identify known transcription factors (TFs) and microRNAs that could explain the transcriptional differences in liver and muscle arising from the influence of diet as shown in Figure 3D and Figure S7 (see Text S2 for complete results).

### White adipose tissue

Even though only few genes showed significantly changed expression in WAT, it is of interest that the biological process “defense response to bacterium” (GO:0042742) was affected by diet. WAT has lately been recognized to produce a number of immune-related proteins that both have paracrine metabolic functions but also can contribute to the elevated plasma levels of acute-phase proteins associated with the metabolic syndrome [17]. However, members of this GO class belong predominantly to the defensin family, which are evolutionary conserved small antimicrobial peptides. In *Drosophila*, defensins are synthesized in the fat body and regulated by the Toll pathway [18]. The role of Toll-like receptors signaling in atherosclerosis [19] and type 2 diabetes [20] is gaining interest since these receptors appear to be a link between nutritional and inflammatory responses [21]. Similar as in liver, biosynthesis of cholesterol (GO:0006695) was lower in WAT from the herring-fed mice, and this could be related to the observed reduced adipocyte size in the herring-fed animals, since larger adipocytes require more cholesterol for the triacylglyceride droplet [22].

### Liver

The biological processes that were affected by the diet in liver were predominantly related to lipid or sterol metabolism and were downregulated in mice fed herring diet relative to the beef diet (Figure 3A). These biological processes include genes encoding protein involved in elongation/desaturation of fatty acids and sterol biosynthesis. This was also reflected from the integrated analysis of transcriptome data using the GSMM or the Reactome processes, where the vast majority of identified metabolites identified were related to biosynthesis of n-6 PUFAs and to a lesser extent n-3 PUFAs (Figure 3B). There was an overrepresentation of response elements of several TFs that are known regulators of lipid/sterol metabolic pathways, as well as microRNA targets, especially the mmu-miR-103 family is found to play a prominent role (Figure 3D, see Text S1 for more details). We also found an increased hepatic expression of *Cyp7a1* as a consequence of the herring diet. This gene encodes the key enzyme for bile acid biosynthesis, and this could be a partial explanation to the lower total cholesterol levels in herring-fed mice (Figure 3C).

### Muscle

There were some unexpected effects of the diet in skeletal muscle (Figure 4). The herring-fed mice appeared to have lower protein turnover in skeletal muscle (Figure 4A). This was reflected in lower protein synthesis (indicated by GO-terms associated with ribosomes and translational processes), lower protein degradation (indicated by the GO-terms related with protein catabolic process by ubiquitination). This was in agreement with the Reactome processes analysis that showed that protein synthesis as well as degradation was downregulated (Figure 4C). There was also a reduced expression of genes related muscle contraction and oxidative phosphorylation in the herring-fed mice (Figure 4B).

### Gene co-expression modules

**Figure 5:**
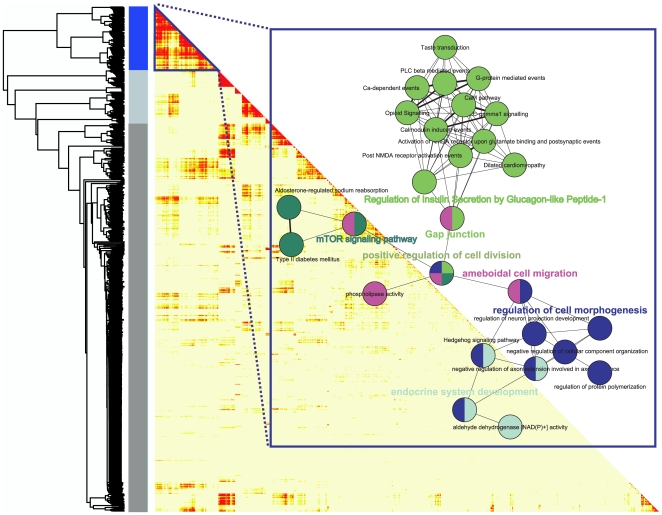
Connectivity (topological overlap) matrix for the most differentially expressed genes by the diets in the three tissues. Based on a two-way ANNOVA, 881 genes were identified to be significantly responding to changes in diet, and these genes were used for the analysis. The rows and columns of the half lower heatmap represent genes in a symmetric fashion. The connectivity strengths were signified by the color intensity, red representing the strongest connection and light yellow representing no connection. The blue color bar delineates the highest interconnected genes module. Within the rectangular frame, the functional terms that show significant enrichment within the blue module is depicted. The colors of the circles indicate the same functional module.

Concerted dietary effects in all three tissues were identified using the approach of Zhang et al. [23] and the results are summarized in Figure 5. One significant gene co-expression module was identified (blue module). In this module, the strongest connected functional groups were G-protein coupled receptor (GPCR) signal transduction and calcium signaling via phospholipase C (PLC) (light green symbols; Figure 5). PLC catalyzes a reaction resulting in the formation of two second messengers; inositol 1,4,5-trisphosphate (IP3) and diacylglycerol (DAG). IP3 mobilizes intracellularly stored calcium while DAG activates protein kinase C isoforms which are involved in regulatory functions. Taste and opioid receptors are GPCRs, whereas activation of the NMDA receptor triggers intracellular calcium signaling events, involving IP3, DAG and calmodulin. Fewer connections were found in mTOR signaling pathway (dark green) and regulation of cell morphogenesis (dark blue).

## Discussion

The herring-fed mice had lower requirement for *de novo* synthesis of PUFAs as the diet contained a surplus of these fatty acids, but surprisingly there was a decreased expression of genes involved in cholesterol and steroid biosynthesis as well (Figure 3D). Our data show that despite the comparable cholesterol content in the two diets, the herring-fed mice had lower plasma levels of total cholesterol, which is consistent with the downregulation of sterol biosynthesis (Figure 3C and Table S2). However, this group of proteins is known to be transcriptionally regulated by the sterol regulatory element-binding factor-2 (*Srebf2*), which is activated by cleavage from the endoplasmic reticulum (ER) membrane when the membrane cholesterol levels are decreased [24]. Increased levels of PUFAs in the ER membrane may affect cholesterol retention in this membrane and that could lead to reduced activation of Srebf2. It is, also possible that the effects of dietary LC n-3 PUFAs on Srebf2 and cholesterol biosynthesis are mediated by Ppara as previously shown in wild-type and *Ppara*−/− mice [24].

Our findings of increased cholesterol clearance through bile acid biosynthesis adds further explains why circulating cholesterol levels were reduced after herring-diet despite similar cholesterol content in the two diets. Other studies using this mouse model also found that the plasma cholesterol levels depends on the lipid composition in the diet [11], [25]. The molecular mechanisms for LC n-3 PUFAs effects on lipid homeostasis and atherosclerosis in these mice have been suggested to involve reduced vascular inflammatory response [25] or an impaired clearance of circulating lipoproteins [26]. Decreased plasma VLDL levels was associated with reduced atherosclerosis in the *Ldlr*−/− mouse [27], which would support the latter hypothesis. Our data suggest differential effects of diet on hepatic lipid metabolism namely downregulation of cholesterol biosynthesis in herring-fed mice and an enrichment of chylomicrons in livers from the beef-fed mice, reflecting a disturbed clearance of circulating lipoprotein particles in mice fed a beef-based diet. We hypothesize that dietary herring ameliorates hepatic lipid handling, resulting in improved blood lipid profile and consequently less plaque formation.

Our findings in the skeletal muscle are consistent with a study where rats fed fish oil resulted in reduced protein turnover in this tissue [28]. Furthermore, changes in the fatty acid composition of skeletal and cardiac muscles, caused by fish oil consumption, affected physical properties of the cell membranes and subsequently muscle function in a rat model [29], [30]. Our analysis showed that there was lower expression of genes related to muscle contraction and oxidative phosphorylation in herring-fed mice compared to those fed beef (Figure 4B). Reduced oxidative phosphorylation could imply lower mitochondria content and thereby dietary effects on skeletal muscle with a switch from type I to type II fibers in the herring-fed mice. It would also explain the reduction in contractility which is more a feature of slow-twitch type I fibers. The enrichment analysis of regulatory motifs revealed overrepresentation of *Nrf1* and *Mef2* regulatory elements (see Text S1 and Figure S8) which also supports a switch in fiber type [31]. However, in this context it is of interest to note that in migrating birds, dietary LC n-3 PUFAs increase the oxidative capacity of muscle to a similar extent as endurance training [32]. In rats and humans, an increased LC n-3 PUFAs content of the cell membranes was shown to improve the efficiency of oxidative phosphorylation, resulting in improved energy production in skeletal muscle [29], [30]. Consequently, we hypothesize that a herring-based diet leads to suppression of protein turnover and hereby reduces atrophy resulting in increased muscle mass. In addition, there was a border-line significance showing increased lean body mass in the herring-fed mice compared to the beef-fed mice (p-value  = 0.05). Our findings are also consistent with clinical studies showing that supplementation of LC n-3 PUFAs resulted in better preservation of body mass in cancer patients [33], [34]. Thus, our results point to a functional explanation of these gross observations.

Analysis of concerted dietary effects across the three tissues revealed a common theme, namely calcium handling. The effects on skeletal muscle protein turnover could be related to this finding since dysregulation of intracellular calcium levels is considered to be a major cause of ER stress leading to unfolded protein response [35]. In skeletal muscle, it has been suggested that dietary fish oil conserves muscle-cell energy metabolism via maintaining sarcoplasmic calcium homeostasis [36]. Changes in cell morphogenesis suggest dietary effects on tissue remodeling. In line with this, mTOR is a nutrient/ATP sensor that regulates pathways controlling ribosome biogenesis and cell growth [37]. In particular, in skeletal muscle mTOR activates phosphatidylinositol 3-kinase and intracellular calcium-related events affecting cell growth, differentiation and survival [38]. These findings could be relevant to reduced plaque formation in mice fed herring since altered sarcoplasmic reticulum calcium handling in vascular smooth muscle cells has suggested to precede the development of atherosclerotic lesions in mice [39].

In conclusion, our integrated analysis of the effect of diet on metabolic function in different tissues shows some very clear effects that have implications for disease development. We propose a mechanistic explanation for the lowered plasma cholesterol levels in response to herring diet, and we further find that a herring diet had a positive effect on protein handling, which could be caused by lower ER stress resulting in less protein misfolding and hence reduced protein turnover. The integrated analysis of transcriptome data using metabolic networks resulted in the identification of signature pathways/processes that could not have been found by standard clustering technique. The core of our concept is to extrapolate differences in the signature pathways/processes, linking them together and combining this with analysis of concerted effects in different tissues to identify mechanisms behind common complex disorders and the effects of diet.

## Materials and Methods

### Animal experiments

The study was approved by the local Animal Ethics Committee at University of Gothenburg, Gothenburg, Sweden. *Ldlr*−*/−* mice were chosen for this study (Text S1). Seven-week old *Ldlr−/−*male mice (JAX stock no 002207) were obtained from Charles River Laboratories (Sulzfeld, Germany). The mice were allowed to acclimatize to the conditions in our animal facility (constant humidity, temperature and 12 h dark/light cycle) for one week before start of the experiment. The mice were given high fat/high sucrose supplemented with either minced herring fillets (*Clupea harengus*) or minced beef (14 mice per diet group). The total fat and cholesterol contents of the two diets were matched (Table S1). The animals were kept on the diets for 16 weeks and body weights were recorded weekly. At week 8, tail vein blood samples were taken and analyzed for plasma content of triglycerides and total cholesterol levels by enzymatically assay with Konelab autoanalyzer version 2.0 (Vantaa, Finland). At week 15, anesthetized (Isofluran, Baxter, Deerfield, IL, USA) mice were scanned by Lunar PIXImus densitometer (Lunar Corp, Madison, WI, USA) to analyze body composition [40]. The mice were killed by overdose of sodium pentobarbital at week 16. The aortas were dissected out, prepared and analyzed by *en face* histology [41]. Liver, skeletal muscle (gastrocnemoius) and epididymal white adipose tissue (WAT) were frozen in liquid nitrogen. Statistical analysis of phenotypes was performed by the SPSS software version 16.0, using the Mann-Whitney U-test. A p-value less than 0.05 was considered statistically significant.

### Transcriptome experiment

Total RNA from liver, skeletal muscle and adipose tissue was isolated from selected three mice from each group using the RNeasy Lipid Tissue Mini kit (liver and WATs; Qiagen, Hilden, Germany) or the RNeasy Mini kit (skeletal muscle; Qiagen), following the manufacturers instruction. 1 µg of total RNA was processed and hybridized on Affymetrix MoGene 1.0 ST (Affymetrix, Santa Clara, CA, USA.) arrays according to the Affymetrix GeneChip Expression Analysis Technical Manual (Affymetrix, Santa Clara, CA, USA.). cDNA was quantified in a spectrophotometer and its quality was evaluated using an Agilent 2100 Bioanalyzer (Agilent Technologies, Waldbronn, Germany) using RNA 6000 Nano LabChip kits (Agilent Technologies). A GeneChip Fluidics Station FS-400 and a GeneChip Scanner 3000 7G were used for hybridization and scanning, respectively. The scanned images (.DAT files) were converted into.CEL files by using the Command console software (Affymetrix). The CEL files were used for further data analysis.

### Analysis of transcriptome data

The CEL-files from all three tissues (18 files) were normalized together to allow for comparison of all expression values. The expression signals were processed by the method of Probe Logarithmic Intensity Error (PLIER) with quantile normalization [42]. Perfect mach probe only (PM-only) was used to calculate the noise and detection limits. All the transcriptome data are available at following statistical analysis using Student's *t*-test and two-way analysis of variance, with correction for multiple testing. In parallel, a Singular Value Decomposition (SVD) and a standard clustering analysis were employed to identify gross patterns of the transcriptome. All analysis were performed using R suite and Bioconductor packages. See more details in Text S1. All the transcriptome data are available at Gene Expression Omnibus (GEO) under accession number GSE22532.

### Integrated analysis

The statistical values were mapped and the reporter algorithm [4] was applied using three different types of biological networks derived from Gene Ontologies (GO) [43], a genome-scale metabolic model (GSMM) of *Mus musculus* [15] and biological evidences from the Reactome database [16], for extraction of biological responses according to influence of different diets on each specific tissue. The multiway comparison of selected significant features from previous integrated analysis were performed and visualized as heat maps of the X-score (normalized accumulative expression values). The regulation analysis was performed on the bioinformatics predictions of TF binding sites and microRNA targets. The statistical values of binding site/target enrichment of each TF/microRNA were calculated by the Fisher's exact test. The pair wise co-occurrence of selected binding sites/targets were presented as a half heat map plot. The influence of diet on multi-tissue fashion were performed over the differentially expressed genes (based on the Q-value of the diet factor derived from 2-way ANOVA) by gene co-expression network module analysis [23]. The strongly connected genes in the co-expression module were further evaluated their related functions by modular enrichment analysis [44]. All analysis were performed using R suite and Cytoscape software [45]. See more details in Text S1 and Figure S5.

## Supporting Information

Text S1 Supplementary data
(0.56 MB DOC)

Text S2 Supplementary file
(2.25 MB PDF)

Figure S1 Primary analysis of the transcriptome data. (A) Bar plot of eigen values of each eigen component that is indicative of the relative variance capture capability of each eigen component. (B) Heat map plot of loading scores of each eigen component. (C) Unsupervised hierarchical clustering of the group of significant genes (Q-value <0.05). Column row colors: red - WAT, beef diet; orange - WAT, herring diet; blue - liver, beef diet; cyan - liver, herring diet; green - muscle, beef diet; light green - muscle, herring diet.
(7.71 MB TIF)

Figure S2 Boxplot of negative logarithm of Q-value derived from A) 2-way ANOVA of tissue, diet and interaction factor, B)from student t-test of transcripts in each of the three tissues.
(0.90 MB TIF)

Figure S3 Multiways heatmap of the X-scores for the different GOs in the three different tissues. The GOs were selected based on reporter p-values <0.001. Column row colors represent tissue by orange, blue and green refer to WAT, liver and muscle, respectively. (A) Biological process, (B) Cellular compartment, (C) Molecular function. The number of genes participated in each GO term are given in Supplementary file.
(2.51 MB TIF)

Figure S4 Multiway heatmap of GOs in response to diet in different tissues. GOs were selected based on reporter p-values <0.001. Column row colors: red - WAT, beef diet; orange - WAT, herring diet; blue - liver, beef diet; cyan - liver, herring diet; green - muscle, beef diet; light green - muscle, herring diet. (A) Cellular compartment, (B) Molecular function. The number of genes participated in each GO term are given in Supplementary file.
(2.16 MB TIF)

Figure S5 The heat map of topological overlap matrix and its connectivity clustering. The colour intensity signifies the connection strength between two genes, with red representing the strongest connection and light yellow representing no connection. The side colors represent the indentified modules.
(0.57 MB TIF)

Figure S6 Circular mapping plot of Q-values (more details in legend of Figure 2 in the main text)
(0.93 MB TIF)

Figure S7 Cumulative distribution of changes for transcripts containing binding target of microRNA mmu-miR-103 and mmu-miR-107 (green line) compared to transcripts without the binding target (black line). The log2 fold changes were calculated by the ratio of the average transcriptional values of herring-fed mice to beef-fed mice. The p-value is calculated between ‘contain site’ group and ‘no site’ group by one-side Kolmogorov-Smirnov(KS) test
(0.74 MB TIF)

Figure S8 Co-occurrence matrix heatmap of overepresented transcript factors and regulatory microRNAs and the response elements of Egr1, Elk1, Nrf1 and Foxn1. In this analysis, there were no significant overepresented microRNA.
(1.01 MB TIF)

Table S1 Macronutrient and fatty acid composition of diets. Data for the fatty acids are shown as mean ± SD, n = 3. Macronutrient contents were calculated from public available food composition data at the National Food Institute, Technical University of Denmark (http://www.foodcomp.dk/v7/fcdb_default.asp).
(0.13 MB PDF)

Table S2 Physiological characteristics of all mice and those selected for microarray analysis. Data are shown as mean ± SD. Significant difference according to the Mann-Whitney U-test is shown as * (p-value <0.05)
(0.17 MB PDF)
