# Supplementary material for: Identifying Molecular Effects of Diet through Systems Biology: Influence of Herring Diet on Sterol Metabolism and Protein Turnover in Mice
Source: PLoS One. 2010 Aug 24;5(8):e12361. doi: 10.1371/journal.pone.0012361 (PMC2927425; doi:10.1371/journal.pone.0012361)
Supplement: Text S1 — Supplementary data (0.56 MB DOC) [file pone.0012361.s001.doc]

Supplementary Data for (Text S1.)

**Identifying molecular effects of diet through systems biology : Influence of herring diet on sterol metabolism and protein turnover in mice**

I. Nookaew^1,#^, B.G. Gabrielsson^2,#^, A. Holmäng^3^, A.-S. Sandberg^2^, J. Nielsen^1^

^1^ Life sciences/Systems Biology, Department of Chemical and Biological Engineering, Chalmers University of Technology, SE-412 96 Gothenburg, Sweden

^2^ Life sciences/Food Science, Department of Chemical and Biological Engineering, Chalmers University of Technology, SE-412 96 Gothenburg, Sweden

^3^ Department of Physiology, Institute of Neuroscience and Physiology, University of Gothenburg, SE-405 30, Gothenburg, Sweden

^#^ These authors contributed equally to this work

*Corresponding author:

Professor Jens Nielsen

Phone: +46 (0) 31 772 3804 , Fax : +46 (0) 31 772 3801

[nielsenj@chalmers.se](mailto:nielsenj@chalmers.se)

**Supplementary data 1 – Animal selection for the experiment and physiological characteristics**

Two of the most commonly studied mouse models of atherosclerosis are the apolipoprotein E-deficient (*ApoE-/-*) mice [1] and the low density lipoprotein receptor-deficient (*Ldlr-/-*) mice [2]. These mice mainly differ in that the *Ldlr-/-* mouse has a dietary dependence on the development of atherosclerosis [3], whereas the *ApoE-/-* mouse spontaneously develops plaques although increased levels of dietary cholesterol enhances the process [4]. Furthermore, the *Ldlr-/-*, but not the *ApoE-/-*, mouse, is susceptible to diet-induced obesity with concomitant insulin resistance [5,6] and as such mimics the clinical situation in the Westernized world.

Epidemiological studies show that a high dietary intake of fish reduces cardiovascular disease (CVD) [7], and these effects are usually attributed to the long-chain n-3 polyunsaturated fatty acids (LC n-3 PUFAs) [8] present in fish. Indeed, previous studies show that dietary LC n-3 PUFAs reduced plaque formation and hepatic steatosis in the *Ldlr-/-* mouse model [9,10]. However, the effects of intake of whole fish fillets on risk factors associated to CVD have previously not been investigated in mice. In the rat, fish protein reduced blood lipid levels, and also affected hepatic genes involved in cholesterol metabolism [11]. Furthermore, a recent clinical study showed that a supplement of LC n-3 PUFAs combined with taurine was more efficient in lowering blood lipid levels than LC n-3 PUFAs alone [12].

In this study, *Ldlr-/-* male mice were given a 16-week high fat/high sucrose diet, supplemented with either minced herring fillets or minced beef, to identify metabolic pathways that were differentially affected in three tissues important for whole body glucose and lipid homeostasis; liver, skeletal muscle and WAT.

**Supplementary data 2 – Analysis of transcriptome data**

**Dimensionality reduction and clustering**

Primary analyses of the transcriptome data was performed and visualized by an unsupervised method of Singular Value Decomposition (SVD). It is possible to investigate the intrinsic variability of the high dimensional transcriptome data among the three tissues in response to the different diets with the dimensionality reduction capability of SVD, based on left eigenvectors called eigenarrays [13]. SVD was performed on the matrix of the transcriptome data consisting of ~26,000 rows (transcripts) and 18 columns (arrays). The first three eigenarray components were used to illustrate the sample discrimination in a pseudo-three dimension plot (the third dimension is presented by the dot size). Furthermore, based on the significant level Q-value at a cut-off of 0.05, unsupervised hierarchical clustering by complete linkage method was performed on the group of significant expressed genes to visualize the consistency of the genes to each group of experiments. Figure S1 panel A and B illustrate that the first 3 eigen components capture most of the variance in the data set as indicated by the eigen values and the loading scores. Unsupervised hierarchical clustering of the group of significant genes indicated that tissue and diet are well discriminated as shown in Figure S1 panel C.

**Statistical analysis of gene expression**

2-Way ANOVA was formulated to evaluate the differentially expressed genes with respect to tissue factor (WAT, liver and muscle) and diet factor (beef and herring fed) as well as the interaction of the two factors. As the influence of tissue factor on gene expression is greater than the influence of diet as noticed by dimensionality reduction analysis, evaluation of significant level of genes expression between beef-fed and herring-fed mice in the specific tissues was performed separately using linear models together with empirical Bayes [14]. The calculated p-values of all statistical analyses were transformed to Q-value by correcting for multiple testing using the method of Benjamini and Hochberg [15]. Changed gene expression with a Q-value < 0.05 was considered significant.

The distribution of Q-values derived from the 2-way ANOVA is illustrated in Figure S2Aand it is clearly seen that the tissue factor is the dominant factor in the system. The interaction between the tissue and diet factors also influences the system, and the effect of diet is therefore best captured by considering differential expression between the two diets for each specific tissue. The distribution of Q-values derived from a student t-test for the specific tissues is shown in Figure 2B

**Supplemenatry data 3 – Integrated analysis**

**Integrated analysis of transcriptome data and biological networks**

To uncover the biological processes adaptations in response to the diet, the transcriptome data were analyzed in the context of biological networks using the reporter algorithm [16,17]. For this, we considered three kinds of well annotatated biological information: gene ontologies (GO) [18], a genome-scale metabolic model (GSMM) [19] and biological evidences from the Reactome database [20]. The three different types of biological information were transformed to bipartite graphs, i.e. graphs with GO-gene interactions, metabolite-gene interactions and reactome-gene interactions. The Q-values for each gene were then mapped on the graphs and the reporter algorithm was applied to indentify features that were significantly changed in response to the different diets.

The results from this integrated analysis of the three biological networks are given in supplementary file 1. The results contain a large number of neighbors for each feature. The reporter p-values, which are derived from the integration of the networks and the Q-values, were calculated for all three types of features.

**Multiways comparison and visualization**

According to the reporter algorithm a feature
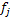
 contains
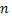
members of transcription levels, whose normalized expression values are represented as a vector of transription levels
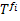
. Considering, a specific experiment
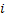
, the empirical cummulative distribution function of the vector is expressed as (1)


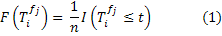


The total expression score
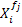
of the feature
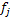
 is then estimated by the difference between the integral of the uniform distribution
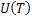
 and the integral of the empirical cummulative distribution function in the interval of normalized expression values as specified by (2)


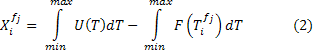


Finally the expression score is normalized with the mean
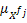
 and the standard deviation
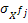
 to give what we call the X-score (
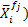
 given as (3)


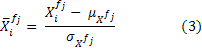


The X-scores are used to indicate the significance of the features. The results were plotted as heatmap for visualization.

The results of the integrated analysis on GO-terms for the different tissues were illustrated in Figure S3, It is recognized that GO catagories associated with specific tissues are identified using this approach, which validates the approach. For example, the GOs that are related with cholesterol and lipid processes and functions are dominant in liver as shown in the blue box of Figure S3 panel A and C, and the GOs that are associated with muscle are found as having highest expression in the muscle.

The additional results of integrated analysis of GO terms in response to diet in the three tissues are lillustrated in Figure S4 in a similar way as Figure 2.

**Enrichment analysis of transcription factor motifs and microRNA targets**

The information of transcription factor (TF) binding sites motif targets, which is derived from vertebrate transcription factors binding sites from position specific scoring matrix (PSSM) from TRANSFAC version 9.4, were retrieved from ECRbase (version 102) [21]. The microRNA targets predictions of mouse were retrieved and compiled from 9 different algorithms, i.e. TargetScan (version 5.1) [22,23], miRanda (release Sept2008) [24,25], PITA (version 6) [26], microcosm (vesion 5) [27], Pictar (release March2007) [28], DIANA-microT (version 3) [29], EIMMo (vesion 3) [30] miRDB (version 3) [31], RNA22 (release May2008) [32]. For each group of genes to be evaluated for TF and microRNA enrichment we evaluated their significant value derived from a one tail Fisher’s exact test. With a p-value cut-off < 0.001, all significant TF and microRNA were drawn as half heatmap of pair wise co-occurrence to illustrate the possible relationships of binding motifs. The diagonal of the heatmap represents the occurrence of each F or microRNA in the tested group. The selected consensus motifs were drawn as logo plot [33] by using SeqLogo package from bioconductor version 2.4.

**Gene co-expression network module analysis**

The integration of transcriptome data over the three tissues is an important analysis to reveal whether the key metabolic effects are coordinated in a multi-tissue fashion. To evaluated this based on the influence of diet, the gene that have Qvalue < 0.075 derived from a 2-way ANOVA (see Supplementary data 2) were chosen for this analysis. The gene co-expression modules were identified using the weighted gene co-expression network analysis (WGCNA) frame work [34] that is available in the R package (named WCGNA) [35]. To identify the modules, the static tree cut method with a minimum size of the module being 40 nodes was performed. The result of the analysis is shown as a heatmap of topological overlap matrix indicating co-expression modules.

In Figure S5, it is clearly seen that the strongest connected gene co-expression module among the three tissues derived from the diet effect are the blue module. The gene members in the blue module were further evaluated of their biological roles by modular enrichment analysis of GO and biological pathways retrieved from KEGG, Reactome and BioCarta. The analysis was performed and visualized using Cytoscape [36] equipped with ClueGO pluging [37].

**Supplementary data 4 – Additional results**

**Circular mapping plot of gene expression**

The logarithm of Q-vales of all transcripts were sorted according to their loci arrangement then plotted in clock-wise direction. **O** is the beginning point of the plot and refers to the first transcript of chromosome 1. The area between **I** and **II** is belong to the transcripts located on chromosome2 (II) as shown in Figure S6.

**Enrichment of the microRNAs in liver implies their role in lipid/sterol metabolism**

According to Figure 3D, mmu-miR-103 has the most frequent binding target (occurrence score > 0.5 with a p-value = 1.99e-4). The mmu-miR-103 is encoded from introns of the pantothenate kinase (a key enzyme of Coenzyme A biosynthesis) genes *Pank2* and *Pank3*. It has a microRNA paralogue named mmu-miR-107 (slightly significant in the enrichment analysis with a p-value = 3.82e-3, see Supplementary file 1), which is encoded from an intron of *Pank1*. These microRNAs are highly conserved in vertebrates and also have high potential role in the inhibition of lipid biosynthesis and metabolism [38]. To explore the effect of mmu-miR-103 and mmu-miR-107 in liver, cumulative distributions of changes in the average transcript levels of herring-fed mice versus beef-fed mice is illustrated in Figure S7.

The efficiency of mmu-miR-103/7 on the transcriptional down-regulation of the genes containing the site on their UTR(s) is significantly observed in herring-fed mice with a p-value < 5e-10 using a one-side KS test (p-value = 3.88e-8 and 2.3e-5 when target site of mmu-miR-103 and mmu-miR-107 is separately considered, respectively). This implies that mmu-miR-103/7 contributes to reduce expression of genes involved in cholesterol and triglyceride biosynthetic in livers from the herring-fed mice, which could be a reason to explain the reduced plasma triglycerides and cholesterol levels. Recently, Li *et al* reported that mmu-miR-103, mmu-miR-107 as well as mmu-miR-34a (also found in our enrichment analysis, see Figure 3D), are associated with changes in hepatic energy and lipid related processes [39].

**Enrichment analysis of DNA motifs of transcription factors and microRNA in muscle**

Genes, related with the reporter GO terms; muscle contraction and oxidative phosphorylation(shown in Figure 4), were analyzed for enrichment of transcription factors and microRNAs. The heat maps shows identified transcription factors and microRNAs and their. Figure S8 illustrates the heatmap of identified transcription factors and microRNAs, and their co-occurrence.

From the enrichment analysis as illustrated in Figure S7, the consensus binding sites of transcription factor *Nrf1*, *Foxn1*, *Egr1* and *Elk1* were overrepresented in these analyses. Nrf1 is a key transcription activator for respiratory and mitochondria biogenesis being induced by Ppargc1a (PGC-1a) in muscle [40]. The co-activator also works in the combination with *Mef2* transcription factor to switch to type I fibers [40]. From the analysis, MEF2_02 binding site, which belong to Mef2, is border-line significant, p-value = 0.0066, using a cut-off of p<0.001 (see Supplementary file). Foxn1, Erg1 and Elk1 have been reported to influence skeletal muscle development [41,42,43].

**References**

1. Nakashima Y, Plump AS, Raines EW, Breslow JL, Ross R (1994) ApoE-deficient mice develop lesions of all phases of atherosclerosis throughout the arterial tree. Arteriosclerosis and Thrombosis 14: 133-140.

2. Ishibashi S, Goldstein JL, Brown MS, Herz J, Burns DK (1994) Massive xanthomatosis and atherosclerosis in cholesterol-fed low density lipoprotein receptor-negative mice. J Clin Invest 93: 1885-1893.

3. Teupser D, Persky AD, Breslow JL (2003) Induction of Atherosclerosis by Low-Fat, Semisynthetic Diets in LDL Receptor-Deficient C57BL/6J and FVB/NJ Mice: Comparison of Lesions of the Aortic Root, Brachiocephalic Artery, and Whole Aorta (En Face Measurement). Arterioscler Thromb Vasc Biol 23: 1907-1913.

4. Getz GS, Reardon CA (2006) Diet and Murine Atherosclerosis. Arterioscler Thromb Vasc Biol 26: 242-249.

5. Schreyer SA, Vick C, Lystig TC, Mystkowski P, LeBoeuf RC (2002) LDL receptor but not apolipoprotein E deficiency increases diet-induced obesity and diabetes in mice. Am J Physiol Endocrinol Metab 282: E207-214.

6. Wu L, Vikramadithyan R, Yu S, Pau C, Hu Y, et al. (2006) Addition of dietary fat to cholesterol in the diets of LDL receptor knockout mice: effects on plasma insulin, lipoproteins, and atherosclerosis. J Lipid Res 47: 2215-2222.

7. Bang HO, Dyerberg J, Sinclair HM (1980) The composition of the Eskimo food in north Western Greenland. American Journal of Clinical Nutrition 33: 2657-2661

8. Bays HE, Tighe AP, Sadovsky R, Davidson MH (2008) Prescription omega-3 fatty acids and their lipid effects: physiologic mechanisms of action and clinical implications. Expert Rev Cardiovasc Ther 6: 391-409.

9. Zampolli A, Bysted A, Leth T, Mortensen A, De Caterina R, et al. (2006) Contrasting effect of fish oil supplementation on the development of atherosclerosis in murine models. Atherosclerosis 184: 78.

10. Saraswathi V, Gao L, Morrow JD, Chait A, Niswender KD, et al. (2007) Fish Oil Increases Cholesterol Storage in White Adipose Tissue with Concomitant Decreases in Inflammation, Hepatic Steatosis, and Atherosclerosis in Mice. J Nutr 137: 1776-1782.

11. Shukla A, Bettzieche A, Hirche F, Brandsch C, Stangl GI, et al. (2006) Dietary fish protein alters blood lipid concentrations and hepatic genes involved in cholesterol homeostasis in the rat model. British Journal of Nutrition 96: 674-682.

12. Elvevoll EO, Eilertsen K-E, Brox J, Dragnes BT, Falkenberg P, et al. (2008) Seafood diets: Hypolipidemic and antiatherogenic effects of taurine and n-3 fatty acids. Atherosclerosis 200: 396.

13. Alter O, Brown PO, Botstein D (2000) Singular value decomposition for genome-wide expression data processing and modeling. Proc Natl Acad Sci U S A 97: 10101-10106.

14. Smyth GK (2004) Linear models and empirical bayes methods for assessing differential expression in microarray experiments. Stat Appl Genet Mol Biol 3: Article3.

15. Benjamini Y, Hochberg Y (1995) Controlling the False Discovery Rate - a Practical and Powerful Approach to Multiple Testing. Journal of the Royal Statistical Society Series B-Methodological 57: 289-300.

16. Patil KR, Nielsen J (2005) Uncovering transcriptional regulation of metabolism by using metabolic network topology. Proc Natl Acad Sci U S A 102: 2685-2689.

17. Oliveira AP, Patil KR, Nielsen J (2008) Architecture of transcriptional regulatory circuits is knitted over the topology of bio-molecular interaction networks. BMC Syst Biol 2: 17.

18. Ashburner M, Ball CA, Blake JA, Botstein D, Butler H, et al. (2000) Gene ontology: tool for the unification of biology. The Gene Ontology Consortium. Nat Genet 25: 25-29.

19. Quek LE, Nielsen LK (2008) On the reconstruction of the Mus musculus genome-scale metabolic network model. Genome Inform 21: 89-100.

20. Vastrik I, D'Eustachio P, Schmidt E, Gopinath G, Croft D, et al. (2007) Reactome: a knowledge base of biologic pathways and processes. Genome Biol 8: R39.

21. Loots G, Ovcharenko I (2007) ECRbase: database of evolutionary conserved regions, promoters, and transcription factor binding sites in vertebrate genomes. Bioinformatics 23: 122-124.

22. Friedman RC, Farh KK, Burge CB, Bartel DP (2009) Most mammalian mRNAs are conserved targets of microRNAs. Genome Res 19: 92-105.

23. Grimson A, Farh KK, Johnston WK, Garrett-Engele P, Lim LP, et al. (2007) MicroRNA targeting specificity in mammals: determinants beyond seed pairing. Mol Cell 27: 91-105.

24. John B, Enright AJ, Aravin A, Tuschl T, Sander C, et al. (2004) Human MicroRNA targets. PLoS Biol 2: e363.

25. Betel D, Wilson M, Gabow A, Marks DS, Sander C (2008) The microRNA.org resource: targets and expression. Nucleic Acids Res 36: D149-153.

26. Kertesz M, Iovino N, Unnerstall U, Gaul U, Segal E (2007) The role of site accessibility in microRNA target recognition. Nat Genet 39: 1278-1284.

27. Griffiths-Jones S, Saini HK, van Dongen S, Enright AJ (2008) miRBase: tools for microRNA genomics. Nucleic Acids Res 36: D154-158.

28. Krek A, Grun D, Poy MN, Wolf R, Rosenberg L, et al. (2005) Combinatorial microRNA target predictions. Nat Genet 37: 495-500.

29. Maragkakis M, Alexiou P, Papadopoulos GL, Reczko M, Dalamagas T, et al. (2009) Accurate microRNA target prediction correlates with protein repression levels. BMC Bioinformatics 10: 295.

30. Gaidatzis D, van Nimwegen E, Hausser J, Zavolan M (2007) Inference of miRNA targets using evolutionary conservation and pathway analysis. BMC Bioinformatics 8: 69.

31. Wang X (2008) miRDB: a microRNA target prediction and functional annotation database with a wiki interface. RNA 14: 1012-1017.

32. Miranda KC, Huynh T, Tay Y, Ang YS, Tam WL, et al. (2006) A pattern-based method for the identification of MicroRNA binding sites and their corresponding heteroduplexes. Cell 126: 1203-1217.

33. Schneider TD, Stephens RM (1990) Sequence logos: a new way to display consensus sequences. Nucleic Acids Res 18: 6097-6100.

34. Emilsson V, Thorleifsson G, Zhang B, Leonardson AS, Zink F, et al. (2008) Genetics of gene expression and its effect on disease. Nature 452: 423-428.

35. Langfelder P, Horvath S (2008) WGCNA: an R package for weighted correlation network analysis. BMC Bioinformatics 9: 559.

36. Shannon P, Markiel A, Ozier O, Baliga NS, Wang JT, et al. (2003) Cytoscape: a software environment for integrated models of biomolecular interaction networks. Genome Res 13: 2498-2504.

37. Bindea G, Mlecnik B, Hackl H, Charoentong P, Tosolini M, et al. (2009) ClueGO: a Cytoscape plug-in to decipher functionally grouped gene ontology and pathway annotation networks. Bioinformatics 25: 1091-1093.

38. Wilfred BR, Wang WX, Nelson PT (2007) Energizing miRNA research: a review of the role of miRNAs in lipid metabolism, with a prediction that miR-103/107 regulates human metabolic pathways. Mol Genet Metab 91: 209-217.

39. Li SJ, Chen X, Zhang HJ, Liang XY, Xiang Y, et al. (2009) Differential expression of microRNAs in mouse liver under aberrant energy metabolic status. Journal of Lipid Research 50: 1756-1765.

40. Lin J, Wu H, Tarr PT, Zhang CY, Wu Z, et al. (2002) Transcriptional co-activator PGC-1 alpha drives the formation of slow-twitch muscle fibres. Nature 418: 797-801.

41. Morrison J, Partridge T, Bou-Gharios G (2005) Nude mutation influences limb skeletal muscle development. Matrix Biol 23: 535-542.

42. Irrcher I, Hood DA (2004) Regulation of Egr-1, SRF, and Sp1 mRNA expression in contracting skeletal muscle cells. J Appl Physiol 97: 2207-2213.

43. Khurana A, Dey CS (2002) Involvement of Elk-1 in L6E9 skeletal muscle differentiation. FEBS Lett 527: 119-124.
