# Supplementary figures and images for: Identifying Molecular Effects of Diet through Systems Biology: Influence of Herring Diet on Sterol Metabolism and Protein Turnover in Mice
Source: PLoS One. 2010 Aug 24;5(8):e12361. doi: 10.1371/journal.pone.0012361 (PMC2927425; doi:10.1371/journal.pone.0012361)

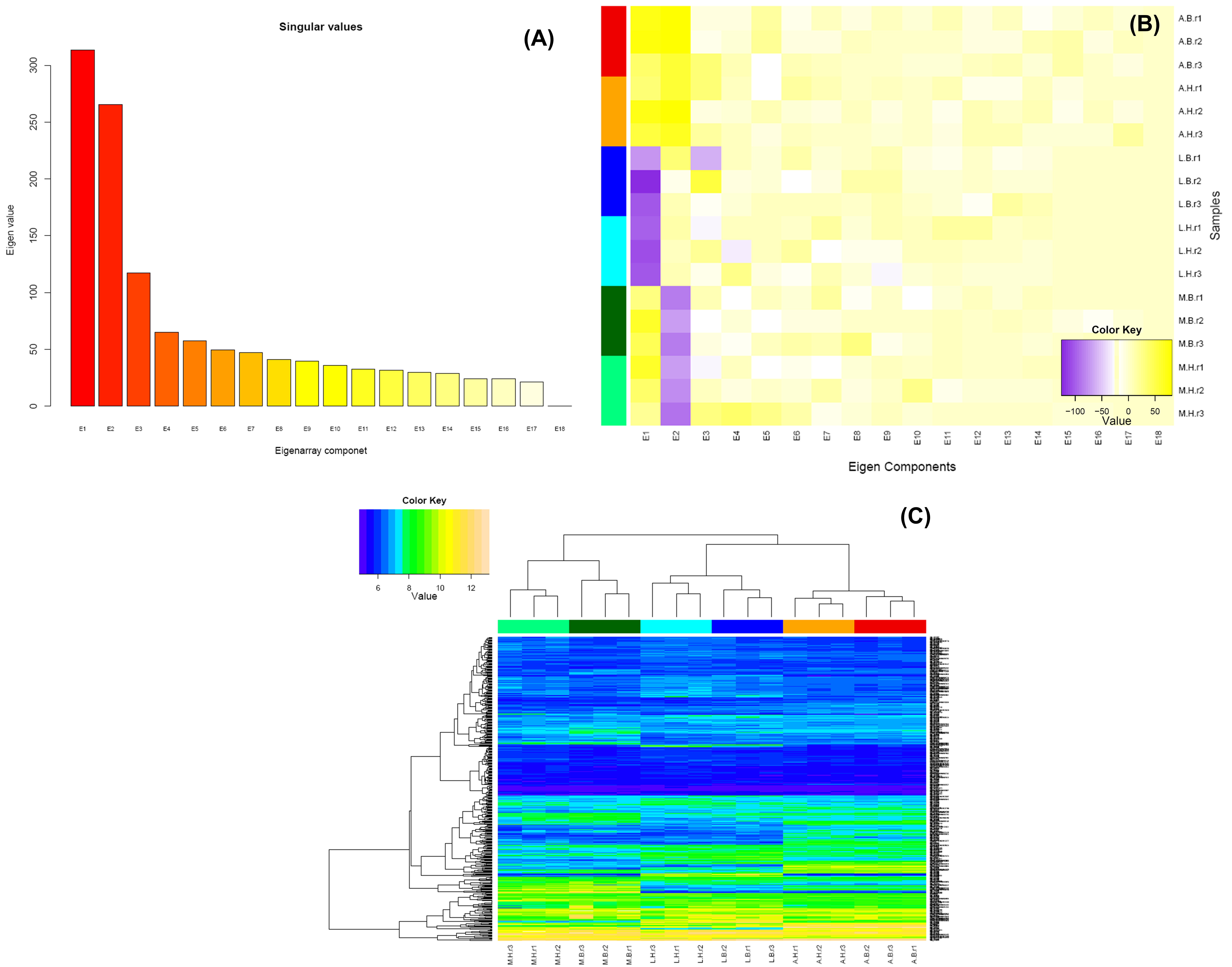

Supplement: Figure S1 — Primary analysis of the transcriptome data. (A) Bar plot of eigen values of each eigen component that is indicative of the relative variance capture capability of each eigen component. (B) Heat map plot of loading scores of each eigen component. (C) Unsupervised hierarchical clustering of the group of significant genes (Q-value <0.05). Column row colors: red - WAT, beef diet; orange - WAT, herring diet; blue - liver, beef diet; cyan - liver, herring diet; green - muscle, beef diet; light green - muscle, herring diet. (7.71 MB TIF) [file pone.0012361.s003.tif]

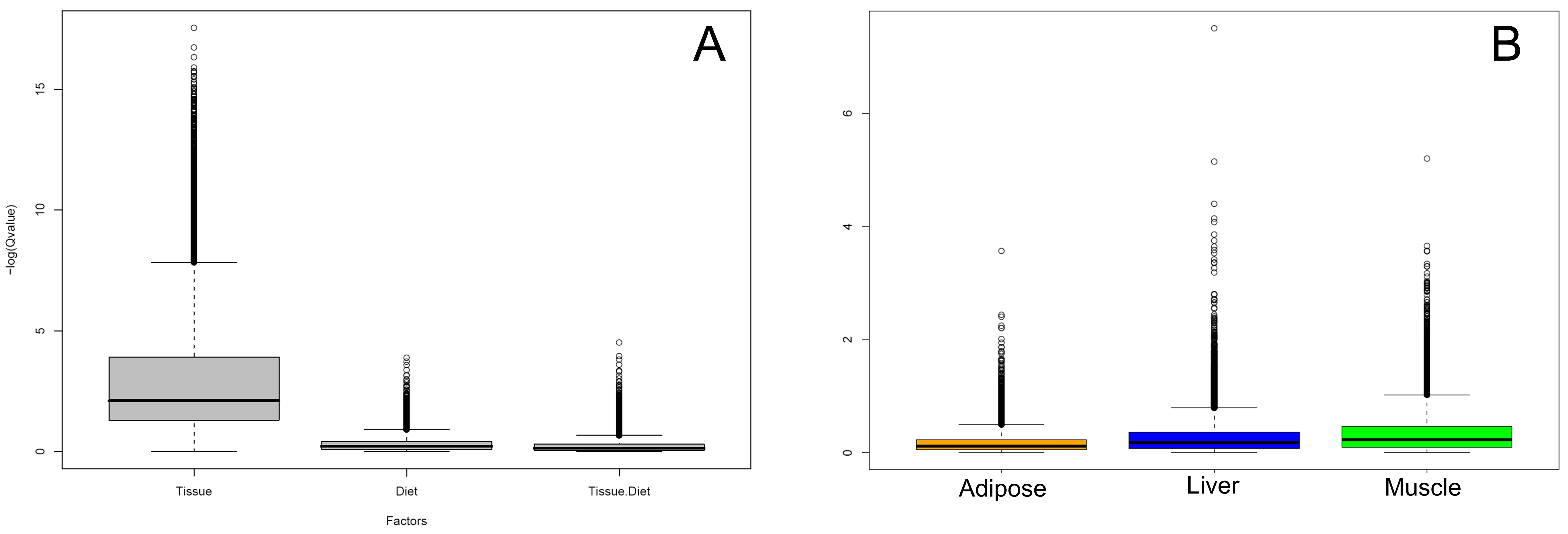

Supplement: Figure S2 — Boxplot of negative logarithm of Q-value derived from A) 2-way ANOVA of tissue, diet and interaction factor, B)from student t-test of transcripts in each of the three tissues. (0.90 MB TIF) [file pone.0012361.s004.tif]

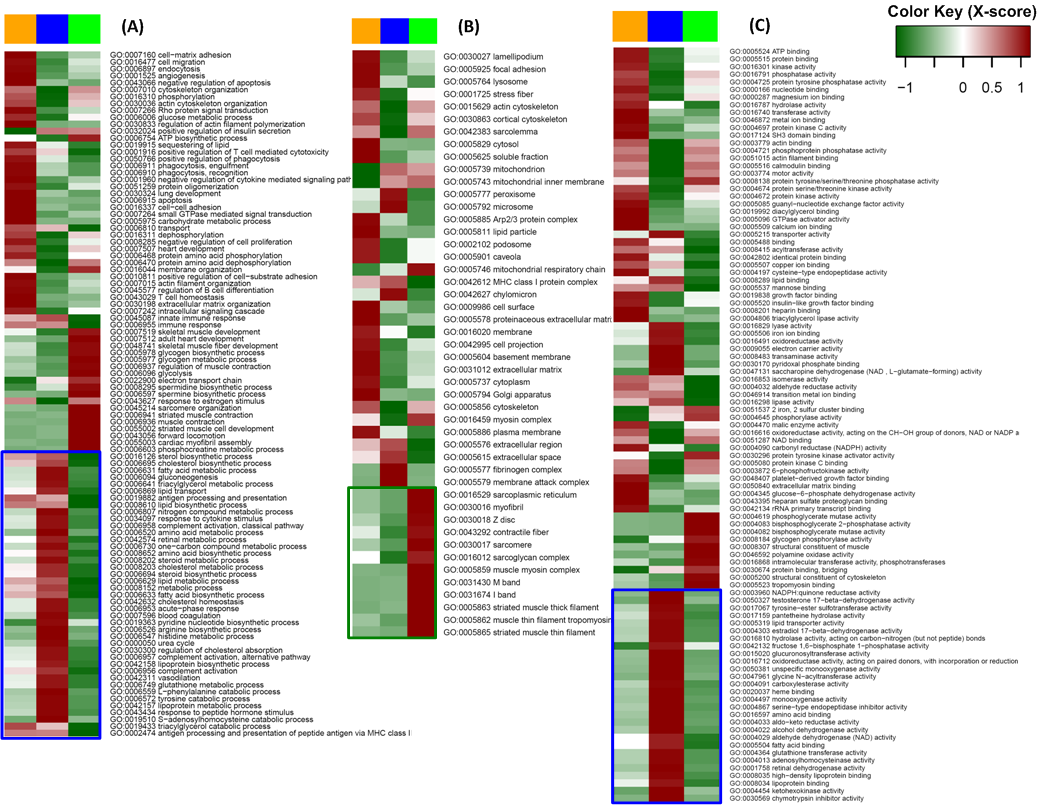

Supplement: Figure S3 — Multiways heatmap of the X-scores for the different GOs in the three different tissues. The GOs were selected based on reporter p-values <0.001. Column row colors represent tissue by orange, blue and green refer to WAT, liver and muscle, respectively. (A) Biological process, (B) Cellular compartment, (C) Molecular function. The number of genes participated in each GO term are given in Supplementary file. (2.51 MB TIF) [file pone.0012361.s005.tif]

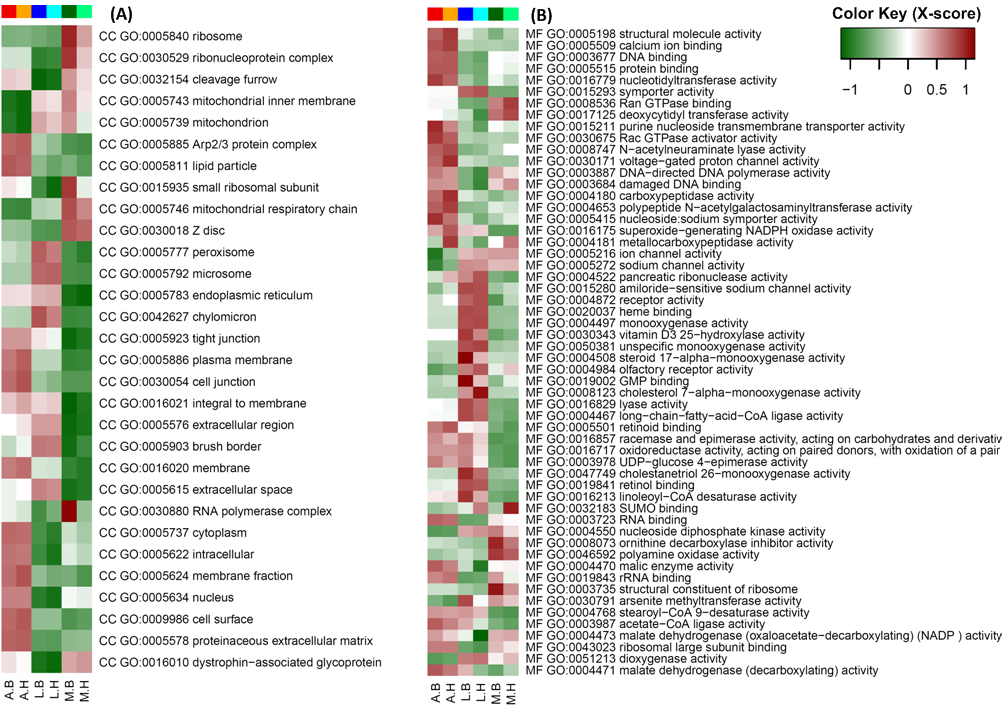

Supplement: Figure S4 — Multiway heatmap of GOs in response to diet in different tissues. GOs were selected based on reporter p-values <0.001. Column row colors: red - WAT, beef diet; orange - WAT, herring diet; blue - liver, beef diet; cyan - liver, herring diet; green - muscle, beef diet; light green - muscle, herring diet. (A) Cellular compartment, (B) Molecular function. The number of genes participated in each GO term are given in Supplementary file. (2.16 MB TIF) [file pone.0012361.s006.tif]

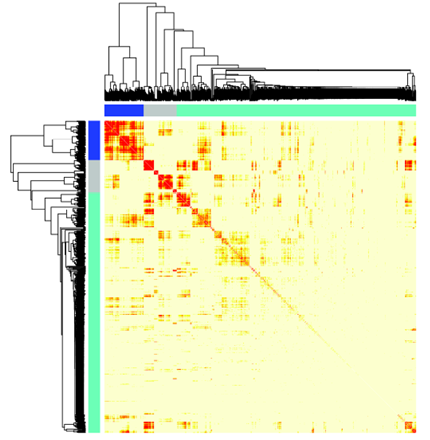

Supplement: Figure S5 — The heat map of topological overlap matrix and its connectivity clustering. The colour intensity signifies the connection strength between two genes, with red representing the strongest connection and light yellow representing no connection. The side colors represent the indentified modules. (0.57 MB TIF) [file pone.0012361.s007.tif]

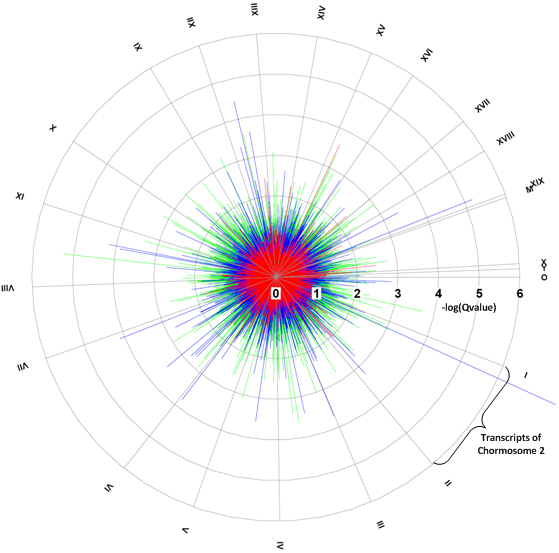

Supplement: Figure S6 — Circular mapping plot of Q-values (more details in legend of Figure 2 in the main text) (0.93 MB TIF) [file pone.0012361.s008.tif]

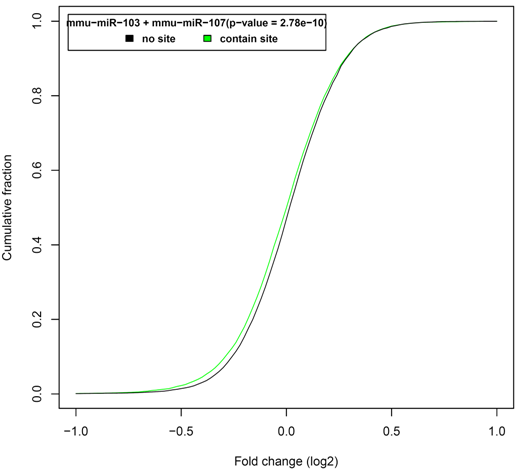

Supplement: Figure S7 — Cumulative distribution of changes for transcripts containing binding target of microRNA mmu-miR-103 and mmu-miR-107 (green line) compared to transcripts without the binding target (black line). The log2 fold changes were calculated by the ratio of the average transcriptional values of herring-fed mice to beef-fed mice. The p-value is calculated between ‘contain site’ group and ‘no site’ group by one-side Kolmogorov-Smirnov(KS) test (0.74 MB TIF) [file pone.0012361.s009.tif]

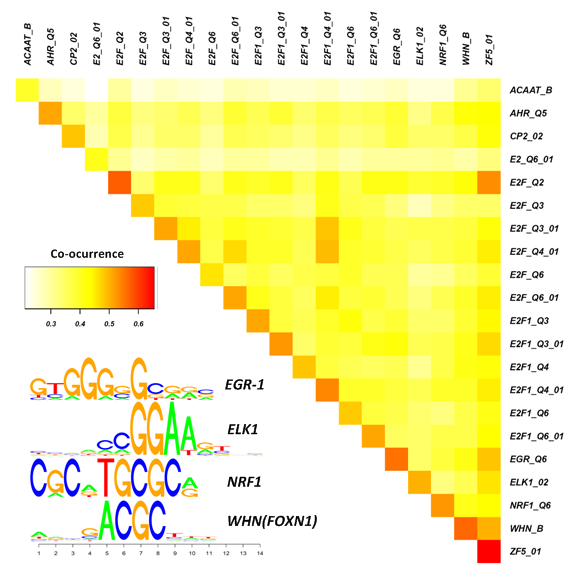

Supplement: Figure S8 — Co-occurrence matrix heatmap of overepresented transcript factors and regulatory microRNAs and the response elements of Egr1, Elk1, Nrf1 and Foxn1. In this analysis, there were no significant overepresented microRNA. (1.01 MB TIF) [file pone.0012361.s010.tif]
