# Supplementary material for: Identifying Molecular Effects of Diet through Systems Biology: Influence of Herring Diet on Sterol Metabolism and Protein Turnover in Mice
Source: PLoS One. 2010 Aug 24;5(8):e12361. doi: 10.1371/journal.pone.0012361 (PMC2927425; doi:10.1371/journal.pone.0012361)
Supplement: Table S1 — Macronutrient and fatty acid composition of diets. Data for the fatty acids are shown as mean ± SD, n = 3. Macronutrient contents were calculated from public available food composition data at the National Food Institute, Technical University of Denmark (http://www.foodcomp.dk/v7/fcdb_default.asp). (0.13 MB PDF) [file pone.0012361.s011.pdf]

Diet	Energy (kJ/100 g)	Protein (g/100 g)	Carbohydrate (g/100 g)	Fat (g/100 g)	SFA (mole%)	MUFA (mole%)	PUFA (mole%)	n-6/n-3 ratio	Cholesterol (mg/100 g)
Beef	1545	13.1	34.8	20.3	66.8±3.2	29.8±3.4	3.3±0.2	3.6±1.2	72.1
Herring	1466	12.5	34.8	20.3	64.0±1.7	27.5±1.8	8.2±0.1	0.50±0.03	70.4
