# Supplementary material for: Identifying Molecular Effects of Diet through Systems Biology: Influence of Herring Diet on Sterol Metabolism and Protein Turnover in Mice
Source: PLoS One. 2010 Aug 24;5(8):e12361. doi: 10.1371/journal.pone.0012361 (PMC2927425; doi:10.1371/journal.pone.0012361)
Supplement: Table S2 — Physiological characteristics of all mice and those selected for microarray analysis. Data are shown as mean ± SD. Significant difference according to the Mann-Whitney U-test is shown as * (p-value <0.05) (0.17 MB PDF) [file pone.0012361.s012.pdf]

Diet	n	Body weights (g)			Body composition (%)		Total cholesterol (mM)		Triglycerides (mM)	
		**w 0**	**w 8**	**w 16**	**LBM**	**FFM**	**w 8**	**w 16**	**w 8**	**w 16**
All:										
Herring	9	21.4±0.47	34.6±1.5	43.2±1.2	59.5±1.0	40.5±1.0	9.6±0.9	19.4±2.9	3.1±0.4	2.7±0.5
Beef	10	21.9±0.41	33.3±0.7	42.8±1.0	56.3±1.3	43.7±1.3	21.4±2.0*	28.4±1.8*	7.4±0.7*	5.5±0.6*
Array:										
Herring	3	21.2±1.4	36.6±3.0	43.6±2.3	58.1±0.7	42.0±0.7	11.3±1.8	25.3±8.0	4.2±0.5	3.6±1.0
Beef	3	21.3±0.3	34.0±1.1	42.7±1.5	55.0±2.5	45.1±2.6	21.6±2.1	30.3±3.4	7.6±1.1	6.4±1.4
