# Supplementary material for: Identifying Molecular Effects of Diet through Systems Biology: Influence of Herring Diet on Sterol Metabolism and Protein Turnover in Mice
Source: PLoS One. 2010 Aug 24;5(8):e12361. doi: 10.1371/journal.pone.0012361 (PMC2927425; doi:10.1371/journal.pone.0012361)
Supplement: Text S2 — Supplementary file (2.25 MB PDF) [file pone.0012361.s002.pdf]

Note: This Supplemenatry file has 3 parts

1. The results of the integration analysis
2. Number of nieghbour of biological networs
3. The results of the enrichment anlysis

1. Integrated Analysis Results

Liver

Gene ontology(GO)

		Reporter P-value	
**#Feature**	**No-direction**	**Down regulated**	**Up regulated**
CC_GO:0016020_membrane	0.627825	1	2.37501E-08
CC_GO:0016021_integral+to+membrane	0.927123	1	1.05638E-12
CC_GO:0005634_nucleus	0.6993	0.999313	0.00097904
MF_GO:0005515_protein+binding	0.961003	0.999909	0.0300381
CC_GO:0005737_cytoplasm	0.175292	0.810382	0.00360632
MF_GO:0004872_receptor+activity	0.994105	1	2.36268E-05
MF_GO:0046872_metal+ion+binding	0.109421	0.345394	0.0708092
CC_GO:0005886_plasma+membrane	0.721949	0.999983	5.28322E-05
MF_GO:0008270_zinc+ion+binding	0.475416	0.769393	0.146563
BP_GO:0006355_regulation+of+transcription,+DNA-dependent	0.956713	0.998659	0.15531
BP_GO:0007186_G-protein+coupled+receptor+protein+signaling+pathway	0.99439	1	6.35027E-07
MF_GO:0003677_DNA+binding	0.93926	0.989683	0.358949
MF_GO:0000166_nucleotide+binding	0.642031	0.775813	0.344636
BP_GO:0006810_transport	0.197787	0.940457	0.000881853
BP_GO:0006350_transcription	0.96751	0.999088	0.174065
BP_GO:0008150_biological_process	0.657432	0.862393	0.254612
CC_GO:0005576_extracellular+region	0.701547	0.999921	0.00026449
CC_GO:0005575_cellular_component	0.191103	0.466994	0.101718
MF_GO:0003674_molecular_function	0.61556	0.880748	0.176972
MF_GO:0016787_hydrolase+activity	0.962584	0.998837	0.160459
MF_GO:0016740_transferase+activity	0.452394	0.687331	0.201345
CC_GO:0005622_intracellular	0.0524095	0.0131196	0.568568
MF_GO:0005524_ATP+binding	0.869952	0.707665	0.880849
BP_GO:0007608_sensory+perception+of+smell	0.957552	1	2.80913E-06
MF_GO:0004984_olfactory+receptor+activity	0.920075	1	1.06365E-06
BP_GO:0007165_signal+transduction	0.944069	0.99991	0.023152
CC_GO:0005739_mitochondrion	0.834775	0.391143	0.960378
BP_GO:0007275_multicellular+organismal+development	0.907876	0.998988	0.0589374

MF_GO:0005509_calcium+ion+binding	0.89717	0.986869	0.265673
CC_GO:0005783_endoplasmic+reticulum	0.0187783	0.908785	9.31349E-06
MF_GO:0003676_nucleic+acid+binding	0.539381	0.954774	0.0244174
MF_GO:0016301_kinase+activity	0.925654	0.941222	0.612734
MF_GO:0003700_transcription+factor+activity	0.932004	0.991297	0.324186
MF_GO:0004871_signal+transducer+activity	0.932759	0.999991	0.00206034
MF_GO:0003824_catalytic+activity	0.00232043	0.00563488	0.092729
MF_GO:0005488_binding	0.39843	0.497582	0.33509
BP_GO:0008152_metabolic+process	0.000110642	0.000499444	0.0385212
CC_GO:0005794_Golgi+apparatus	0.785023	0.975734	0.132255
BP_GO:0006468_protein+amino+acid+phosphorylation	0.898275	0.937687	0.497321
MF_GO:0016491_oxidoreductase+activity	0.00449931	0.0124726	0.083938
BP_GO:0006811_ion+transport	0.811759	0.993761	0.0840617
BP_GO:0055114_oxidation+reduction	0.00219824	0.00546362	0.0840333
MF_GO:0004672_protein+kinase+activity	0.863091	0.88454	0.571561
MF_GO:0003723_RNA+binding	0.942767	0.999956	0.0117823
MF_GO:0004930_G-protein+coupled+receptor+activity	0.98675	0.999999	0.0247046
BP_GO:0030154_cell+differentiation	0.777113	0.999144	0.00321766
BP_GO:0015031_protein+transport	0.377623	0.874046	0.0239357
BP_GO:0006511_ubiquitin-dependent+protein+catabolic+process	0.961461	0.998304	0.236064
BP_GO:0045449_regulation+of+transcription	0.937546	0.999151	0.0992524
MF_GO:0043565_sequence-specific+DNA+binding	0.94921	0.997736	0.274595
MF_GO:0008233_peptidase+activity	0.999922	1	0.164255
BP_GO:0007049_cell+cycle	0.241478	0.266767	0.367298
BP_GO:0007155_cell+adhesion	0.773833	0.996161	0.0153069
CC_GO:0005856_cytoskeleton	0.0183101	0.036346	0.13944
MF_GO:0004674_protein+serine/threonine+kinase+activity	0.863437	0.850004	0.66493
BP_GO:0006508_proteolysis	0.995376	0.999975	0.113225
BP_GO:0006915_apoptosis	0.214747	0.484895	0.119085
CC_GO:0030054_cell+junction	0.418911	0.893285	0.0353736
MF_GO:0000287_magnesium+ion+binding	0.417944	0.363286	0.542825
CC_GO:0005615_extracellular+space	0.93293	0.974695	0.511364
CC_GO:0005887_integral+to+plasma+membrane	0.241713	0.620246	0.0903204
CC_GO:0030529_ribonucleoprotein+complex	0.902413	0.873276	0.764094
BP_GO:0006412_translation	0.993494	0.911655	0.984786
MF_GO:0005525_GTP+binding	0.00612302	0.0228274	0.0649333
BP_GO:0045944_positive+regulation+of+transcription+from+RNA+polymera	0.71121	0.981626	0.0356309
MF_GO:0005216_ion+channel+activity	0.858292	0.991557	0.175693
CC_GO:0005829_cytosol	0.975264	0.982743	0.708769
CC_GO:0005743_mitochondrial+inner+membrane	0.479018	0.28004	0.709703
BP_GO:0006955_immune+response	0.502962	0.488062	0.519062
MF_GO:0005506_iron+ion+binding	0.00979964	0.0528191	0.0443913
MF_GO:0016874_ligase+activity	0.359536	0.325571	0.492088
MF_GO:0003779_actin+binding	0.0834479	0.0600404	0.423011
CC_GO:0005578_proteinaceous+extracellular+matrix	0.446497	0.833242	0.0867513
BP_GO:0006397_mRNA+processing	0.802804	0.99863	0.0213413
MF_GO:0005215_transporter+activity	0.0114478	0.25211	0.00396711

BP_GO:0007242_intracellular+signaling+cascade	0.122919	0.105222	0.412329
CC_GO:0005840_ribosome	0.827885	0.334262	0.95308
CC_GO:0045202_synapse	0.345102	0.922677	0.0193177
BP_GO:0006629_lipid+metabolic+process	0.0100854	0.03141	0.0817602
CC_GO:0005624_membrane+fraction	0.330572	0.473231	0.273062
BP_GO:0051301_cell+division	0.467142	0.4551	0.50571
MF_GO:0030528_transcription+regulator+activity	0.592754	0.915844	0.100026
BP_GO:0050896_response+to+stimulus	0.603377	0.982422	0.0280049
BP_GO:0007264_small+GTPase+mediated+signal+transduction	0.003268	0.00680409	0.0900579
CC_GO:0005874_microtubule	0.0133427	0.0389429	0.089258
CC_GO:0031410_cytoplasmic+vesicle	0.487345	0.833036	0.120549
BP_GO:0008380_RNA+splicing	0.635691	0.996256	0.0132157
MF_GO:0016757_transferase+activity,+transferring+glycosyl+groups	0.335881	0.457195	0.3009
BP_GO:0007283_spermatogenesis	0.849712	0.99276	0.11262
CC_GO:0005667_transcription+factor+complex	0.709905	0.840802	0.372233
BP_GO:0006974_response+to+DNA+damage+stimulus	0.058743	0.22378	0.0594719
BP_GO:0000122_negative+regulation+of+transcription+from+RNA+polymer	0.626583	0.680415	0.476225
MF_GO:0003735_structural+constituent+of+ribosome	0.856487	0.268129	0.976756
BP_GO:0006281_DNA+repair	0.0965015	0.14855	0.217265
MF_GO:0009055_electron+carrier+activity	0.00165311	0.027135	0.012601
BP_GO:0007399_nervous+system+development	0.479397	0.811069	0.104269
CC_GO:0005768_endosome	0.0553172	0.182861	0.0795921
CC_GO:0042995_cell+projection	0.110022	0.484557	0.015684
MF_GO:0005198_structural+molecule+activity	0.979929	0.991497	0.551835
BP_GO:0006886_intracellular+protein+transport	0.953716	0.990249	0.346441
MF_GO:0005125_cytokine+activity	0.919561	0.969388	0.514586
CC_GO:0005694_chromosome	0.995294	0.706252	0.999845
MF_GO:0005096_GTPase+activator+activity	0.18953	0.0333843	0.859287
BP_GO:0005975_carbohydrate+metabolic+process	0.914974	0.726875	0.931292
MF_GO:0008415_acyltransferase+activity	0.273301	0.337521	0.330165
MF_GO:0005529_sugar+binding	0.152401	0.275909	0.185842
MF_GO:0008236_serine-type+peptidase+activity	0.927213	0.999938	0.00712695
BP_GO:0006813_potassium+ion+transport	0.686681	0.78603	0.421481
MF_GO:0004713_protein+tyrosine+kinase+activity	0.752597	0.806584	0.49327
MF_GO:0008237_metallopeptidase+activity	0.881546	0.995798	0.0912429
BP_GO:0007067_mitosis	0.507528	0.617627	0.364208
CC_GO:0005792_microsome	7.10284E-05	0.0407465	0.000126027
CC_GO:0005764_lysosome	0.11496	0.107532	0.353561
BP_GO:0016192_vesicle-mediated+transport	0.645178	0.9806	0.0249536
MF_GO:0016563_transcription+activator+activity	0.914719	0.954055	0.496468
BP_GO:0016568_chromatin+modification	0.83643	0.913931	0.417536
BP_GO:0001701_in+utero+embryonic+development	0.993479	0.972067	0.944217
MF_GO:0004252_serine-type+endopeptidase+activity	0.987485	0.999994	0.0370691
CC_GO:0009897_external+side+of+plasma+membrane	0.577985	0.772804	0.273413
MF_GO:0008083_growth+factor+activity	0.161706	0.876479	0.00454048
MF_GO:0030145_manganese+ion+binding	0.571084	0.385359	0.740291
BP_GO:0007507_heart+development	0.940181	0.974431	0.492475

MF_GO:0005244_voltage-gated+ion+channel+activity	0.778597	0.95916	0.253439
BP_GO:0008284_positive+regulation+of+cell+proliferation	0.818172	0.992903	0.100399
BP_GO:0008283_cell+proliferation	0.915903	0.974886	0.388481
BP_GO:0006954_inflammatory+response	0.550323	0.607603	0.446473
CC_GO:0005681_spliceosome	0.851263	0.9992	0.054081
CC_GO:0031225_anchored+to+membrane	0.127967	0.0640866	0.501414
CC_GO:0045211_postsynaptic+membrane	0.474734	0.80821	0.132942
CC_GO:0005882_intermediate+filament	0.924927	0.976693	0.462868
MF_GO:0004721_phosphoprotein+phosphatase+activity	0.953411	0.905777	0.853326
MF_GO:0042803_protein+homodimerization+activity	0.21656	0.396943	0.172084
MF_GO:0017111_nucleoside-triphosphatase+activity	0.538773	0.544851	0.503491
BP_GO:0008285_negative+regulation+of+cell+proliferation	0.314909	0.58361	0.156641
MF_GO:0003774_motor+activity	0.0566268	0.0245379	0.538539
BP_GO:0016055_Wnt+receptor+signaling+pathway	0.713717	0.920254	0.186382
MF_GO:0005085_guanyl-nucleotide+exchange+factor+activity	0.292794	0.105493	0.793314
BP_GO:0006464_protein+modification+process	0.18633	0.57239	0.0665122
MF_GO:0016829_lyase+activity	0.00437107	0.000985203	0.378804
BP_GO:0006814_sodium+ion+transport	0.215148	0.801979	0.0120683
MF_GO:0008168_methyltransferase+activity	0.757341	0.92541	0.285292
MF_GO:0030955_potassium+ion+binding	0.877372	0.953033	0.469668
MF_GO:0008289_lipid+binding	0.0674539	0.0450165	0.397086
MF_GO:0003924_GTPase+activity	0.149686	0.525949	0.0183837
MF_GO:0005516_calmodulin+binding	0.221099	0.211466	0.41657
MF_GO:0003682_chromatin+binding	0.182947	0.464562	0.0982823
MF_GO:0016853_isomerase+activity	0.417245	0.413468	0.471303
MF_GO:0016791_phosphatase+activity	0.737861	0.895053	0.251515
MF_GO:0016564_transcription+repressor+activity	0.789301	0.891618	0.393725
MF_GO:0015293_symporter+activity	0.322568	0.987872	0.000735954
BP_GO:0006260_DNA+replication	0.0221218	0.00105703	0.825152
BP_GO:0006470_protein+amino+acid+dephosphorylation	0.98929	0.990397	0.753138
BP_GO:0042981_regulation+of+apoptosis	0.101499	0.235041	0.130327
MF_GO:0005102_receptor+binding	0.748013	0.985182	0.0977772
MF_GO:0008234_cysteine-type+peptidase+activity	0.999931	0.993962	0.999104
BP_GO:0006816_calcium+ion+transport	0.557326	0.921953	0.129535
BP_GO:0006457_protein+folding	0.142245	0.464394	0.0816999
BP_GO:0006897_endocytosis	0.496547	0.717052	0.17371
CC_GO:0005730_nucleolus	0.838534	0.96095	0.343012
MF_GO:0020037_heme+binding	0.000260537	0.00385552	0.0125972
MF_GO:0004866_endopeptidase+inhibitor+activity	0.903305	0.38873	0.987306
BP_GO:0009887_organ+morphogenesis	0.968948	0.98539	0.602071
BP_GO:0006357_regulation+of+transcription+from+RNA+polymerase+II+prc	0.942981	0.935633	0.720143
BP_GO:0019236_response+to+pheromone	0.929925	0.960032	0.621251
BP_GO:0043066_negative+regulation+of+apoptosis	0.0192654	0.503247	0.00104912
MF_GO:0005179_hormone+activity	0.846446	0.998888	0.0790153
MF_GO:0031402_sodium+ion+binding	0.188774	0.743261	0.0179379
MF_GO:0004222_metalloendopeptidase+activity	0.862332	0.995242	0.0864515
BP_GO:0008610_lipid+biosynthetic+process	3.06106E-06	2.70177E-07	0.173533

MF_GO:0016503_pheromone+receptor+activity	0.935254	0.956122	0.657623
CC_GO:0005777_peroxisome	0.0479376	0.0756025	0.199093
BP_GO:0009952_anterior/posterior+pattern+formation	0.765314	0.923842	0.317195
MF_GO:0004386_helicase+activity	0.836374	0.180254	0.998031
BP_GO:0022900_electron+transport+chain	0.394354	0.316127	0.517951
MF_GO:0004725_protein+tyrosine+phosphatase+activity	0.956587	0.991521	0.349266
BP_GO:0001525_angiogenesis	0.664791	0.98577	0.0138038
BP_GO:0016311_dephosphorylation	0.849882	0.972791	0.158219
MF_GO:0004842_ubiquitin-protein+ligase+activity	0.559561	0.933416	0.0883047
CC_GO:0043234_protein+complex	0.927164	0.870607	0.821361
MF_GO:0005550_pheromone+binding	0.915621	0.849356	0.819466
MF_GO:0004867_serine-type+endopeptidase+inhibitor+activity	0.676823	0.509841	0.755683
BP_GO:0007601_visual+perception	0.504418	0.901133	0.0500133
MF_GO:0004518_nuclease+activity	0.142529	0.263474	0.169234
MF_GO:0004497_monooxygenase+activity	0.000201163	0.0152305	0.00246072
BP_GO:0045941_positive+regulation+of+transcription	0.976833	0.951041	0.866764
BP_GO:0016042_lipid+catabolic+process	0.254215	0.560896	0.115487
BP_GO:0006952_defense+response	0.646529	0.931944	0.0942842
MF_GO:0008201_heparin+binding	0.55665	0.600613	0.447356
BP_GO:0042127_regulation+of+cell+proliferation	0.409751	0.776889	0.103963
BP_GO:0045087_innate+immune+response	0.351845	0.292048	0.546117
BP_GO:0006950_response+to+stress	0.591355	0.92042	0.0933976
BP_GO:0007154_cell+communication	0.57885	0.8874	0.0619718
BP_GO:0006334_nucleosome+assembly	0.990189	0.359793	0.999917
BP_GO:0045786_negative+regulation+of+cell+cycle	0.252462	0.362877	0.254168
BP_GO:0006935_chemotaxis	0.819934	0.750262	0.734841
BP_GO:0006917_induction+of+apoptosis	0.2629	0.24384	0.439741
MF_GO:0005249_voltage-gated+potassium+channel+activity	0.711446	0.860896	0.334046
CC_GO:0000786_nucleosome	0.950197	0.152552	0.999875
CC_GO:0005929_cilium	0.392674	0.708696	0.141404
BP_GO:0042742_defense+response+to+bacterium	0.674676	0.938754	0.08326
CC_GO:0009986_cell+surface	0.76132	0.759834	0.610756
CC_GO:0005923_tight+junction	0.376123	0.643005	0.137332
BP_GO:0007169_transmembrane+receptor+protein+tyrosine+kinase+signali	0.253409	0.275931	0.374204
BP_GO:0007218_neuropeptide+signaling+pathway	0.309271	0.806092	0.0528965
BP_GO:0006631_fatty+acid+metabolic+process	0.157224	0.224539	0.247828
BP_GO:0030198_extracellular+matrix+organization	0.543769	0.881837	0.11038
CC_GO:0030424_axon	0.734154	0.73304	0.587227
BP_GO:0007229_integrin-mediated+signaling+pathway	0.797088	0.91574	0.299056
MF_GO:0046982_protein+heterodimerization+activity	0.450018	0.616967	0.293179
BP_GO:0007411_axon+guidance	0.457227	0.840036	0.108054
MF_GO:0046983_protein+dimerization+activity	0.973923	0.995338	0.518541
MF_GO:0016887_ATPase+activity	0.425312	0.493976	0.354399
CC_GO:0043025_cell+soma	0.696415	0.771871	0.444549
MF_GO:0042802_identical+protein+binding	0.786544	0.624788	0.796402
BP_GO:0001558_regulation+of+cell+growth	0.0771702	0.310579	0.0479369
MF_GO:0005267_potassium+channel+activity	0.828405	0.957947	0.338926

MF_GO:0017124_SH3+domain+binding	0.00958199	0.0639769	0.0351811
MF_GO:0016798_hydrolase+activity,+acting+on+glycosyl+bonds	0.331451	0.419178	0.325719
BP_GO:0030036_actin+cytoskeleton+organization	0.347415	0.500638	0.232342
BP_GO:0030324_lung+development	0.461208	0.606305	0.279599
CC_GO:0005741_mitochondrial+outer+membrane	0.369769	0.182365	0.709579
BP_GO:0007166_cell+surface+receptor+linked+signal+transduction	0.552815	0.580307	0.485561
BP_GO:0007018_microtubule-based+movement	0.00188716	0.0088509	0.0480238
BP_GO:0007626_locomotory+behavior	0.625977	0.883842	0.159098
BP_GO:0007605_sensory+perception+of+sound	0.493392	0.949213	0.0349213
MF_GO:0016779_nucleotidyltransferase+activity	0.385768	0.338328	0.530442
MF_GO:0031404_chloride+ion+binding	0.757763	0.77781	0.576426
CC_GO:0031012_extracellular+matrix	0.253082	0.382106	0.252043
CC_GO:0016324_apical+plasma+membrane	0.643296	0.857035	0.234035
BP_GO:0007420_brain+development	0.543074	0.864517	0.143639
MF_GO:0003743_translation+initiation+factor+activity	0.834959	0.984652	0.197168
CC_GO:0005604_basement+membrane	0.193301	0.19146	0.380749
MF_GO:0008134_transcription+factor+binding	0.409158	0.653614	0.159395
BP_GO:0016481_negative+regulation+of+transcription	0.975381	0.98308	0.635027
BP_GO:0006364_rRNA+processing	0.295135	0.700777	0.138155
BP_GO:0043065_positive+regulation+of+apoptosis	0.679089	0.155748	0.95876
BP_GO:0001501_skeletal+system+development	0.921995	0.991274	0.380241
MF_GO:0003713_transcription+coactivator+activity	0.771493	0.887698	0.285958
BP_GO:0006916_anti-apoptosis	0.537994	0.71999	0.305723
BP_GO:0045893_positive+regulation+of+transcription,+DNA-dependent	0.855066	0.747465	0.813827
BP_GO:0065002_intracellular+protein+transmembrane+transport	0.834301	0.947953	0.359737
BP_GO:0030900_forebrain+development	0.784089	0.754587	0.656445
MF_GO:0051082_unfolded+protein+binding	0.143253	0.594278	0.0363299
BP_GO:0007268_synaptic+transmission	0.916368	0.98981	0.17134
BP_GO:0007156_homophilic+cell+adhesion	0.153669	0.212652	0.258041
CC_GO:0005625_soluble+fraction	0.233612	0.0323327	0.865037
MF_GO:0016702_oxidoreductase+activity,+acting+on+single+donors+with+i	0.677012	0.615413	0.649704
MF_GO:0003714_transcription+corepressor+activity	0.651708	0.619931	0.596781
BP_GO:0001764_neuron+migration	0.606144	0.686565	0.429265
MF_GO:0050660_FAD+binding	0.0842675	0.225287	0.0772056
MF_GO:0005089_Rho+guanyl-nucleotide+exchange+factor+activity	0.419102	0.126121	0.942529
CC_GO:0019717_synaptosome	0.0942706	0.74742	0.0111613
CC_GO:0008076_voltage-gated+potassium+channel+complex	0.521065	0.428445	0.631487
MF_GO:0005262_calcium+channel+activity	0.542361	0.908972	0.135247
MF_GO:0005254_chloride+channel+activity	0.666053	0.805459	0.385167
MF_GO:0008565_protein+transporter+activity	0.783181	0.874894	0.454239
BP_GO:0001503_ossification	0.228538	0.404653	0.17349
MF_GO:0019992_diacylglycerol+binding	0.00698195	0.00285028	0.385111
BP_GO:0035023_regulation+of+Rho+protein+signal+transduction	0.392283	0.106091	0.942529
CC_GO:0015629_actin+cytoskeleton	0.0994759	0.14506	0.224678
MF_GO:0003777_microtubule+motor+activity	0.0249134	0.0640698	0.106988
MF_GO:0008026_ATP-dependent+helicase+activity	0.997212	0.896472	0.996821
MF_GO:0004888_transmembrane+receptor+activity	0.333583	0.355274	0.410517

MF_GO:0004714_transmembrane+receptor+protein+tyrosine+kinase+activi	0.693971	0.850634	0.292895
MF_GO:0005507_copper+ion+binding	0.395674	0.771264	0.150085
BP_GO:0009790_embryonic+development	0.191891	0.36929	0.162306
BP_GO:0030326_embryonic+limb+morphogenesis	0.554544	0.800385	0.177025
BP_GO:0009791_post-embryonic+development	0.634307	0.961777	0.0676677
MF_GO:0004519_endonuclease+activity	0.0391464	0.0698972	0.166752
BP_GO:0007409_axonogenesis	0.590091	0.670835	0.400955
BP_GO:0042254_ribosome+biogenesis	0.00226254	0.087228	0.00378827
BP_GO:0008033_tRNA+processing	0.486189	0.975635	0.0278551
CC_GO:0005746_mitochondrial+respiratory+chain	0.922879	0.481272	0.95815
BP_GO:0007219_Notch+signaling+pathway	0.792983	0.935543	0.304914
BP_GO:0042472_inner+ear+morphogenesis	0.770605	0.954648	0.203826
BP_GO:0045892_negative+regulation+of+transcription,+DNA-dependent	0.164194	0.283539	0.184645
BP_GO:0006694_steroid+biosynthetic+process	0.000156932	8.21135E-05	0.321031
BP_GO:0009058_biosynthetic+process	0.723216	0.713817	0.587326
BP_GO:0007389_pattern+specification+process	0.330019	0.721541	0.135962
BP_GO:0045454_cell+redox+homeostasis	0.665081	0.827645	0.414537
MF_GO:0004221_ubiquitin+thiolesterase+activity	0.994904	0.981279	0.93575
BP_GO:0030182_neuron+differentiation	0.0548182	0.248538	0.0556203
CC_GO:0030027_lamellipodium	0.224766	0.409233	0.132362
BP_GO:0051726_regulation+of+cell+cycle	0.258278	0.36295	0.280421
CC_GO:0008021_synaptic+vesicle	0.275106	0.477366	0.219362
MF_GO:0030170_pyridoxal+phosphate+binding	0.452213	0.576124	0.276425
BP_GO:0006633_fatty+acid+biosynthetic+process	0.0132951	0.0131597	0.20424
BP_GO:0016337_cell-cell+adhesion	0.177996	0.743838	0.0179665
BP_GO:0008104_protein+localization	0.115643	0.452481	0.0249111
BP_GO:0010468_regulation+of+gene+expression	0.266113	0.86813	0.0104584
BP_GO:0006417_regulation+of+translation	0.497844	0.876603	0.0424551
BP_GO:0006486_protein+amino+acid+glycosylation	0.083118	0.641099	0.00693083
BP_GO:0008202_steroid+metabolic+process	0.0174393	0.070685	0.0486865
CC_GO:0016459_myosin+complex	0.488772	0.203485	0.910737
BP_GO:0007519_skeletal+muscle+development	0.712621	0.816555	0.464942
MF_GO:0004197_cysteine-type+endopeptidase+activity	0.7847	0.283787	0.989677
MF_GO:0035091_phosphoinositide+binding	0.366392	0.538897	0.180883
BP_GO:0051246_regulation+of+protein+metabolic+process	0.0962438	0.390683	0.0673596
BP_GO:0006812_cation+transport	0.800487	0.776453	0.665324
BP_GO:0006821_chloride+transport	0.670703	0.716773	0.508046
BP_GO:0015992_proton+transport	0.980902	0.975512	0.814518
MF_GO:0019787_small+conjugating+protein+ligase+activity	0.0850066	0.401792	0.0521597
CC_GO:0005643_nuclear+pore	0.99023	0.993157	0.708008
BP_GO:0043687_post-translational+protein+modification	0.128722	0.469731	0.0709203
MF_GO:0003707_steroid+hormone+receptor+activity	0.649203	0.597323	0.624031
BP_GO:0006869_lipid+transport	0.0165568	0.00298831	0.78242
BP_GO:0007596_blood+coagulation	0.479715	0.413936	0.629868
BP_GO:0008203_cholesterol+metabolic+process	0.0120681	0.0257251	0.12743
BP_GO:0008360_regulation+of+cell+shape	0.687451	0.616157	0.674537
BP_GO:0007050_cell+cycle+arrest	0.0722922	0.306723	0.0525053

MF_GO:0004879_ligand-dependent+nuclear+receptor+activity	0.64609	0.592559	0.628617
CC_GO:0005875_microtubule+associated+complex	0.0172705	0.108105	0.028551
BP_GO:0045165_cell+fate+commitment	0.0532854	0.227519	0.0566351
CC_GO:0005789_endoplasmic+reticulum+membrane	0.528139	0.920935	0.0806201
CC_GO:0005654_nucleoplasm	0.734205	0.877838	0.291943
BP_GO:0007126_meiosis	0.656274	0.916131	0.148847
BP_GO:0050909_sensory+perception+of+taste	0.743882	0.888729	0.318137
BP_GO:0006754_ATP+biosynthetic+process	0.65295	0.518802	0.737635
BP_GO:0006979_response+to+oxidative+stress	0.329316	0.252766	0.546673
MF_GO:0015078_hydrogen+ion+transmembrane+transporter+activity	0.985894	0.985751	0.800809
CC_GO:0048471_perinuclear+region+of+cytoplasm	0.694406	0.815502	0.424405
BP_GO:0030097_hemopoiesis	0.596487	0.685498	0.389577
CC_GO:0000785_chromatin	0.775578	0.957219	0.284288
BP_GO:0007267_cell-cell+signaling	0.571996	0.78981	0.242131
BP_GO:0051028_mRNA+transport	0.480068	0.822404	0.102444
MF_GO:0008017_microtubule+binding	0.794322	0.865621	0.435795
CC_GO:0005635_nuclear+envelope	0.296509	0.223186	0.531821
CC_GO:0016023_cytoplasmic+membrane-bounded+vesicle	0.0309292	0.199983	0.0281434
BP_GO:0048704_embryonic+skeletal+system+morphogenesis	0.414514	0.738597	0.190705
BP_GO:0006887_exocytosis	0.0771123	0.307068	0.0409316
CC_GO:0000775_chromosome,+centromeric+region	0.940722	0.779483	0.932308
BP_GO:0009953_dorsal/ventral+pattern+formation	0.814312	0.716823	0.765795
MF_GO:0004812_aminoacyl-tRNA+ligase+activity	0.977945	0.975254	0.798378
BP_GO:0006096_glycolysis	0.899474	0.987973	0.0395663
BP_GO:0006006_glucose+metabolic+process	0.998015	0.999813	0.434465
BP_GO:0046777_protein+amino+acid+autophosphorylation	0.326036	0.731596	0.0465231
CC_GO:0045121_membrane+raft	0.714304	0.939327	0.155037
MF_GO:0051536_iron-sulfur+cluster+binding	0.628923	0.788142	0.37414
CC_GO:0000139_Golgi+membrane	0.713773	0.987265	0.0584927
CC_GO:0030425_dendrite	0.974044	0.977727	0.708235
MF_GO:0004527_exonuclease+activity	0.338234	0.743965	0.0469384
BP_GO:0016477_cell+migration	0.245396	0.668823	0.0597489
CC_GO:0005938_cell+cortex	0.203081	0.267558	0.283171
BP_GO:0001568_blood+vessel+development	0.0963231	0.484467	0.0422361
BP_GO:0006874_cellular+calcium+ion+homeostasis	0.329903	0.665646	0.138629
BP_GO:0001822_kidney+development	0.155948	0.573615	0.0438217
BP_GO:0043524_negative+regulation+of+neuron+apoptosis	0.128514	0.872884	0.00398618
CC_GO:0005813_centrosome	0.787614	0.804235	0.561955
BP_GO:0009615_response+to+virus	0.288476	0.385151	0.305729
BP_GO:0006836_neurotransmitter+transport	0.710611	0.993384	0.0165275
BP_GO:0007010_cytoskeleton+organization	4.51179E-05	0.000633122	0.0128239
BP_GO:0006396_RNA+processing	0.707411	0.515182	0.771379
CC_GO:0030018_Z+disc	0.742224	0.405487	0.891543
BP_GO:0007243_protein+kinase+cascade	0.764786	0.883924	0.286688
CC_GO:0045177_apical+part+of+cell	0.525073	0.403161	0.709352
BP_GO:0051216_cartilage+development	0.224714	0.738306	0.0333902
MF_GO:0031072_heat+shock+protein+binding	0.405452	0.819439	0.105425

BP_GO:0019221_cytokine-mediated+signaling+pathway	0.213658	0.324042	0.244436
BP_GO:0009968_negative+regulation+of+signal+transduction	0.462639	0.40104	0.54905
CC_GO:0005802_trans-Golgi+network	0.482539	0.494091	0.480033
CC_GO:0016323_basolateral+plasma+membrane	0.147675	0.353126	0.0679609
MF_GO:0005097_Rab+GTPase+activator+activity	0.0295501	0.0435489	0.204708
BP_GO:0043010_camera-type+eye+development	0.257278	0.762127	0.0542356
MF_GO:0003702_RNA+polymerase+II+transcription+factor+activity	0.315016	0.0286143	0.936809
BP_GO:0007179_transforming+growth+factor+beta+receptor+signaling+pat	0.536572	0.306559	0.792228
BP_GO:0006139_nucleobase,+nucleoside,+nucleotide+and+nucleic+acid+me	0.14056	0.271856	0.120221
MF_GO:0008092_cytoskeletal+protein+binding	0.620698	0.563838	0.619215
MF_GO:0004715_non-membrane+spanning+protein+tyrosine+kinase+activi	0.66267	0.60939	0.628857
MF_GO:0008009_chemokine+activity	0.455107	0.302364	0.615446
MF_GO:0004175_endopeptidase+activity	0.894663	0.958017	0.534766
BP_GO:0006310_DNA+recombination	0.480159	0.188928	0.81763
MF_GO:0005230_extracellular+ligand-gated+ion+channel+activity	0.278727	0.561382	0.162569
MF_GO:0030594_neurotransmitter+receptor+activity	0.278727	0.561382	0.162569
BP_GO:0007160_cell-matrix+adhesion	0.846794	0.905137	0.461811
MF_GO:0016773_phosphotransferase+activity,+alcohol+group+as+acceptor	0.464131	0.488309	0.452419
BP_GO:0042593_glucose+homeostasis	0.671152	0.545752	0.730143
BP_GO:0008344_adult+locomotory+behavior	0.936639	0.987519	0.480231
BP_GO:0001570_vasculogenesis	0.91775	0.981086	0.4681
BP_GO:0042475_odontogenesis+of+dentine-containing+tooth	0.374759	0.662298	0.206118
BP_GO:0006418_tRNA+aminoacylation+for+protein+translation	0.962525	0.924883	0.855765
BP_GO:0051056_regulation+of+small+GTPase+mediated+signal+transductio	0.665908	0.702302	0.514565
MF_GO:0008138_protein+tyrosine/serine/threonine+phosphatase+activity	0.826291	0.821511	0.627764
BP_GO:0015986_ATP+synthesis+coupled+proton+transport	0.945218	0.92075	0.799362
BP_GO:0008643_carbohydrate+transport	0.597275	0.837084	0.291973
BP_GO:0001756_somitogenesis	0.223493	0.598324	0.0703895
MF_GO:0005351_sugar:hydrogen+symporter+activity	0.562085	0.801617	0.291973
BP_GO:0008654_phospholipid+biosynthetic+process	0.821542	0.756385	0.729153
MF_GO:0019904_protein+domain+specific+binding	0.335918	0.764064	0.0482421
BP_GO:0006865_amino+acid+transport	0.382845	0.874279	0.0870857
BP_GO:0001889_liver+development	0.792267	0.850617	0.463183
BP_GO:0007368_determination+of+left/right+symmetry	0.77172	0.821592	0.52556
BP_GO:0007417_central+nervous+system+development	0.226043	0.268931	0.330294
MF_GO:0003755_peptidyl-prolyl+cis-trans+isomerase+activity	0.691003	0.608426	0.6641
BP_GO:0042391_regulation+of+membrane+potential	0.610494	0.737505	0.364568
CC_GO:0001726_ruffle	0.287867	0.53106	0.108137
BP_GO:0000902_cell+morphogenesis	0.290319	0.574955	0.12457
BP_GO:0032313_regulation+of+Rab+GTPase+activity	0.041749	0.0445158	0.30045
CC_GO:0005834_heterotrimeric+G-protein+complex	0.555393	0.843414	0.123379
BP_GO:0007286_spermatid+development	0.826097	0.981265	0.143897
MF_GO:0003899_DNA-directed+RNA+polymerase+activity	0.299749	0.708915	0.0935007
MF_GO:0004091_carboxylesterase+activity	0.45216	0.707257	0.21347
BP_GO:0006730_one-carbon+compound+metabolic+process	0.266339	0.0972291	0.698891
MF_GO:0004180_carboxypeptidase+activity	0.288594	0.262397	0.462942
MF_GO:0004896_cytokine+receptor+activity	0.767944	0.709554	0.687913

BP_GO:0000187_activation+of+MAPK+activity	0.22448	0.949826	0.00332472
BP_GO:0000226_microtubule+cytoskeleton+organization	0.55327	0.397249	0.730081
MF_GO:0008146_sulfotransferase+activity	0.306928	0.756371	0.107724
BP_GO:0019882_antigen+processing+and+presentation	0.901235	0.268437	0.997607
BP_GO:0007517_muscle+development	0.191074	0.375029	0.196357
BP_GO:0018108_peptidyl-tyrosine+phosphorylation	0.966926	0.955247	0.783174
CC_GO:0030117_membrane+coat	0.887182	0.915607	0.545909
BP_GO:0007017_microtubule-based+process	0.0929852	0.418308	0.0140063
BP_GO:0016567_protein+ubiquitination	0.918806	0.964396	0.393602
BP_GO:0030216_keratinocyte+differentiation	0.326425	0.80692	0.0292022
MF_GO:0050381_unspecific+monooxygenase+activity	0.000134316	0.00753685	0.00318652
MF_GO:0016820_hydrolase+activity,+acting+on+acid+anhydrides,+catalyzin	0.920349	0.863842	0.81357
BP_GO:0001843_neural+tube+closure	0.483123	0.546399	0.41807
BP_GO:0042493_response+to+drug	0.986127	0.998285	0.205019
BP_GO:0030163_protein+catabolic+process	0.783196	0.841834	0.494432
MF_GO:0051287_NAD+binding	0.914456	0.865399	0.792295
BP_GO:0030334_regulation+of+cell+migration	0.301144	0.332067	0.386253
BP_GO:0000165_MAPKKK+cascade	0.472558	0.904727	0.0864871
BP_GO:0050885_neuromuscular+process+controlling+balance	0.878015	0.9514	0.302231
CC_GO:0030173_integral+to+Golgi+membrane	0.240329	0.713807	0.0258477
MF_GO:0004693_cyclin-dependent+protein+kinase+activity	0.280216	0.200026	0.56266
BP_GO:0030879_mammary+gland+development	0.639623	0.941183	0.154634
CC_GO:0005925_focal+adhesion	0.278164	0.760008	0.0362886
MF_GO:0043169_cation+binding	0.164866	0.0999887	0.52986
CC_GO:0005905_coated+pit	0.623102	0.821164	0.244964
BP_GO:0003007_heart+morphogenesis	0.283711	0.233605	0.45904
BP_GO:0048706_embryonic+skeletal+system+development	0.285843	0.497533	0.205773
MF_GO:0008080_N-acetyltransferase+activity	0.563544	0.581913	0.505473
BP_GO:0043473_pigmentation	0.973475	0.99843	0.24636
CC_GO:0019898_extrinsic+to+membrane	0.508391	0.483912	0.551124
MF_GO:0003887_DNA-directed+DNA+polymerase+activity	0.00169561	0.00275447	0.133392
CC_GO:0030141_secretory+granule	0.636838	0.683667	0.507085
CC_GO:0019861_flagellum	0.0184431	0.252224	0.00801721
BP_GO:0006506_GPI+anchor+biosynthetic+process	0.0298082	0.639722	0.00243547
BP_GO:0008544_epidermis+development	0.687506	0.861989	0.244036
BP_GO:0031424_keratinization	0.8578	0.95935	0.329473
MF_GO:0000155_two-component+sensor+activity	0.891738	0.947869	0.390445
BP_GO:0009987_cellular+process	0.141763	0.156224	0.320955
BP_GO:0030183_B+cell+differentiation	0.309181	0.695873	0.0715011
MF_GO:0019901_protein+kinase+binding	0.845545	0.975103	0.136231
BP_GO:0035264_multicellular+organism+growth	0.355962	0.768004	0.039633
BP_GO:0006413_translational+initiation	0.338381	0.753558	0.143493
BP_GO:0006366_transcription+from+RNA+polymerase+II+promoter	0.0321666	0.0464794	0.196226
MF_GO:0003746_translation+elongation+factor+activity	0.800484	0.683189	0.771838
MF_GO:0005319_lipid+transporter+activity	0.0106573	0.0050171	0.442105
MF_GO:0016712_oxidoreductase+activity,+acting+on+paired+donors,+with-	0.00267857	0.232134	0.0017032
BP_GO:0001666_response+to+hypoxia	0.878301	0.947094	0.466362

MF_GO:0004722_protein+serine/threonine+phosphatase+activity	0.943638	0.942285	0.727914
MF_GO:0015662_ATPase+activity,+coupled+to+transmembrane+movement	0.230692	0.183631	0.490963
MF_GO:0042626_ATPase+activity,+coupled+to+transmembrane+movement	0.849657	0.956656	0.148129
CC_GO:0043005_neuron+projection	0.276184	0.0485882	0.890995
BP_GO:0040007_growth	0.367528	0.614611	0.169258
BP_GO:0045596_negative+regulation+of+cell+differentiation	0.669931	0.662584	0.561084
MF_GO:0004177_aminopeptidase+activity	0.383913	0.50193	0.316772
MF_GO:0005044_scavenger+receptor+activity	0.526065	0.371234	0.723634
CC_GO:0000151_ubiquitin+ligase+complex	0.0699308	0.448072	0.0213339
BP_GO:0016358_dendrite+development	0.128841	0.462811	0.0524473
MF_GO:0003684_damaged+DNA+binding	0.0859429	0.0354817	0.584743
MF_GO:0008430_selenium+binding	0.481532	0.467417	0.502696
CC_GO:0042383_sarcolemma	0.222815	0.239857	0.361726
MF_GO:0005200_structural+constituent+of+cytoskeleton	0.35121	0.125993	0.760556
BP_GO:0001702_gastrulation+with+mouth+forming+second	0.703555	0.530618	0.821644
BP_GO:0006605_protein+targeting	0.971718	0.991817	0.422477
BP_GO:0019538_protein+metabolic+process	0.599617	0.573214	0.568633
BP_GO:0048705_skeletal+system+morphogenesis	0.767473	0.977908	0.150437
MF_GO:0005496_steroid+binding	0.34835	0.831013	0.0211438
BP_GO:0048754_branching+morphogenesis+of+a+tube	0.594351	0.659727	0.433253
MF_GO:0016881_acid-amino+acid+ligase+activity	0.970559	0.940252	0.858171
MF_GO:0003705_RNA+polymerase+II+transcription+factor+activity,+enhanc	0.732055	0.799717	0.482816
BP_GO:0007281_germ+cell+development	0.116062	0.180676	0.220219
BP_GO:0040018_positive+regulation+of+multicellular+organism+growth	0.990376	0.982808	0.868174
BP_GO:0008542_visual+learning	0.407639	0.483454	0.357576
BP_GO:0045665_negative+regulation+of+neuron+differentiation	0.43577	0.79878	0.103301
BP_GO:0006333_chromatin+assembly+or+disassembly	0.460641	0.751511	0.198529
BP_GO:0001890_placenta+development	0.0630353	0.422501	0.0376888
CC_GO:0030286_dynein+complex	0.355916	0.496792	0.275485
CC_GO:0016529_sarcoplasmic+reticulum	0.533807	0.405815	0.649112
BP_GO:0006958_complement+activation,+classical+pathway	0.863604	0.924727	0.455893
MF_GO:0051015_actin+filament+binding	0.780092	0.573072	0.898617
CC_GO:0008305_integrin+complex	0.933079	0.925333	0.714936
BP_GO:0032496_response+to+lipopolysaccharide	0.884555	0.590989	0.942345
BP_GO:0006606_protein+import+into+nucleus	0.376494	0.579413	0.192723
MF_GO:0045028_purinergic+nucleotide+receptor+activity,+G-protein+coupl	0.816336	0.965213	0.0754033
BP_GO:0048469_cell+maturation	0.75182	0.838506	0.317792
BP_GO:0006338_chromatin+remodeling	0.522131	0.235176	0.881828
BP_GO:0007628_adult+walking+behavior	0.697731	0.620527	0.667034
CC_GO:0005921_gap+junction	0.124037	0.676372	0.010426
MF_GO:0003725_double-stranded+RNA+binding	0.858697	0.818642	0.716561
MF_GO:0003729_mRNA+binding	0.648489	0.936377	0.138868
BP_GO:0030317_sperm+motility	0.44287	0.649472	0.324002
BP_GO:0005977_glycogen+metabolic+process	0.93976	0.917624	0.769495
BP_GO:0010811_positive+regulation+of+cell-substrate+adhesion	0.110951	0.152939	0.252395
BP_GO:0016126_sterol+biosynthetic+process	3.29125E-08	3.74026E-11	0.6717
BP_GO:0006826_iron+ion+transport	0.0309559	0.0628761	0.14065

CC_GO:0005911_cell-cell+junction	0.524718	0.666882	0.282921
BP_GO:0001658_ureteric+bud+branching	0.331729	0.515148	0.252263
MF_GO:0016758_transferase+activity,+transferring+hexosyl+groups	0.748087	0.640892	0.731085
BP_GO:0001656_metanephros+development	0.316439	0.553316	0.22456
BP_GO:0006914_autophagy	0.541824	0.511801	0.55191
BP_GO:0030030_cell+projection+organization	0.37389	0.484956	0.277867
CC_GO:0001669_acrosome	0.675908	0.846275	0.343516
MF_GO:0004364_glutathione+transferase+activity	0.00261499	0.00999169	0.0610125
MF_GO:0008483_transaminase+activity	0.658461	0.451668	0.81059
CC_GO:0030863_cortical+cytoskeleton	0.645346	0.809876	0.27602
BP_GO:0006953_acute-phase+response	0.100483	0.698426	0.00470277
BP_GO:0001649_osteoblast+differentiation	0.325955	0.377558	0.372282
BP_GO:0008217_regulation+of+blood+pressure	0.654686	0.69857	0.517284
CC_GO:0005769_early+endosome	0.702842	0.709973	0.549665
BP_GO:0045444_fat+cell+differentiation	0.804375	0.987609	0.151214
BP_GO:0030073_insulin+secretion	0.437742	0.43771	0.481936
BP_GO:0009954_proximal/distal+pattern+formation	0.292068	0.030646	0.914164
MF_GO:0004601_peroxidase+activity	0.545562	0.438534	0.598696
MF_GO:0015297_antiporter+activity	0.319749	0.677225	0.116677
BP_GO:0000160_two-component+signal+transduction+system+(phosphorel	0.766676	0.890281	0.285951
BP_GO:0050731_positive+regulation+of+peptidyl-tyrosine+phosphorylation	0.354877	0.670641	0.102145
BP_GO:0048663_neuron+fate+commitment	0.331341	0.813138	0.0791828
BP_GO:0007266_Rho+protein+signal+transduction	0.280048	0.836323	0.0310535
BP_GO:0006695_cholesterol+biosynthetic+process	6.14419E-08	4.24985E-09	0.55627
BP_GO:0007015_actin+filament+organization	0.57949	0.725391	0.411988
CC_GO:0005770_late+endosome	0.976864	0.9644	0.849295
BP_GO:0001755_neural+crest+cell+migration	0.54785	0.809125	0.251313
BP_GO:0019835_cytolysis	0.987225	0.981658	0.852834
BP_GO:0009636_response+to+toxin	0.947299	0.957407	0.707133
BP_GO:0051258_protein+polymerization	0.0404206	0.170921	0.0344237
BP_GO:0009117_nucleotide+metabolic+process	0.379497	0.591583	0.259084
CC_GO:0031224_intrinsic+to+membrane	0.238963	0.130267	0.606082
MF_GO:0016788_hydrolase+activity,+acting+on+ester+bonds	0.287957	0.492188	0.192218
BP_GO:0040014_regulation+of+multicellular+organism+growth	0.886837	0.973983	0.39031
BP_GO:0001569_patterning+of+blood+vessels	0.571367	0.371126	0.741668
MF_GO:0004553_hydrolase+activity,+hydrolyzing+O-glycosyl+compounds	0.510236	0.63475	0.361922
CC_GO:0030131_clathrin+adaptor+complex	0.541884	0.693671	0.25913
BP_GO:0030178_negative+regulation+of+Wnt+receptor+signaling+pathway	0.458105	0.39268	0.552819
BP_GO:0007189_G-protein+signaling,+adenylate+cyclase+activating+pathwa	0.660775	0.795628	0.356843
BP_GO:0007610_behavior	0.706612	0.97947	0.0889791
BP_GO:0007059_chromosome+segregation	0.232919	0.604458	0.0795466
BP_GO:0048666_neuron+development	0.522065	0.971042	0.0560526
BP_GO:0009566_fertilization	0.159149	0.395689	0.142973
CC_GO:0045095_keratin+filament	0.951656	0.958334	0.690507
BP_GO:0042632_cholesterol+homeostasis	0.765897	0.723417	0.666179
MF_GO:0004857_enzyme+inhibitor+activity	0.395231	0.769931	0.150659
CC_GO:0030426_growth+cone	0.686445	0.471302	0.833457

MF_GO:0016772_transferase+activity,+transferring+phosphorus-containing-	0.731181	0.799386	0.498244
MF_GO:0004114_3',5'-cyclic-nucleotide+phosphodiesterase+activity	0.134148	0.132941	0.357924
BP_GO:0015758_glucose+transport	0.922797	0.91381	0.732021
BP_GO:0006879_cellular+iron+ion+homeostasis	0.104112	0.238291	0.142712
MF_GO:0016831_carboxy-lyase+activity	0.728214	0.654644	0.685092
BP_GO:0008286_insulin+receptor+signaling+pathway	0.95863	0.990098	0.369266
BP_GO:0006829_zinc+ion+transport	0.797572	0.838549	0.492719
BP_GO:0042552_myelination	0.750987	0.846106	0.434295
MF_GO:0005083_small+GTPase+regulator+activity	0.841046	0.496357	0.942255
BP_GO:0000082_G1/S+transition+of+mitotic+cell+cycle	0.143211	0.373275	0.131803
MF_GO:0018024_histone-lysine+N-methyltransferase+activity	0.921993	0.933204	0.605708
BP_GO:0006986_response+to+unfolded+protein	0.898098	0.995755	0.202296
BP_GO:0007369_gastrulation	0.51671	0.494143	0.533374
BP_GO:0006919_caspase+activation	0.560076	0.568454	0.511862
BP_GO:0006259_DNA+metabolic+process	0.0986857	0.201132	0.156451
BP_GO:0008584_male+gonad+development	0.144874	0.572213	0.0229096
BP_GO:0001892_embryonic+placenta+development	0.999419	0.99904	0.922446
CC_GO:0005839_proteasome+core+complex	0.997702	0.996532	0.925431
MF_GO:0004298_threonine-type+endopeptidase+activity	0.997702	0.996532	0.925431
MF_GO:0005272_sodium+channel+activity	0.72551	0.723669	0.594584
MF_GO:0004890_GABA-A+receptor+activity	0.242247	0.474027	0.155109
BP_GO:0008652_amino+acid+biosynthetic+process	0.408625	0.125921	0.889018
MF_GO:0016616_oxidoreductase+activity,+acting+on+the+CH-OH+group+of	0.0997318	0.137191	0.246057
MF_GO:0005201_extracellular+matrix+structural+constituent	0.273588	0.631093	0.110592
BP_GO:0030335_positive+regulation+of+cell+migration	0.878807	0.879205	0.656376
BP_GO:0007265_Ras+protein+signal+transduction	0.530708	0.816848	0.177466
MF_GO:0004181_metallocarboxypeptidase+activity	0.39302	0.501213	0.328741
BP_GO:0007005_mitochondrion+organization	0.746832	0.939753	0.218578
BP_GO:0006909_phagocytosis	0.409581	0.281658	0.748191
CC_GO:0005913_cell-cell+adherens+junction	0.918878	0.907152	0.718618
BP_GO:0021987_cerebral+cortex+development	0.233268	0.72129	0.0706349
BP_GO:0001657_ureteric+bud+development	0.573296	0.839618	0.167031
BP_GO:0030855_epithelial+cell+differentiation	0.588054	0.702074	0.336586
MF_GO:0008373_sialyltransferase+activity	0.0797793	0.350776	0.0366414
BP_GO:0043588_skin+development	0.629432	0.616929	0.564325
MF_GO:0016627_oxidoreductase+activity,+acting+on+the+CH-CH+group+of	0.691884	0.54763	0.81683
BP_GO:0001707_mesoderm+formation	0.997461	0.997091	0.811595
CC_GO:0005740_mitochondrial+envelope	0.599146	0.55611	0.603794
BP_GO:0048468_cell+development	0.801639	0.983787	0.120062
BP_GO:0006913_nucleocytoplasmic+transport	0.936906	0.998896	0.0465584
CC_GO:0005819_spindle	0.184509	0.509534	0.103113
BP_GO:0031175_neurite+development	0.793837	0.626913	0.810485
MF_GO:0003697_single-stranded+DNA+binding	0.394821	0.466142	0.392778
BP_GO:0048511_rhythmic+process	0.390273	0.421307	0.422323
BP_GO:0001541_ovarian+follicle+development	0.579231	0.852026	0.156716
CC_GO:0000119_mediator+complex	0.624434	0.262162	0.88888
BP_GO:0007223_Wnt+receptor+signaling+pathway,+calcium+modulating+p	0.781942	0.954006	0.22505

CC_GO:0009434_microtubule-based+flagellum	0.796941	0.851101	0.578611
BP_GO:0007606_sensory+perception+of+chemical+stimulus	0.784096	0.905186	0.377185
BP_GO:0034097_response+to+cytokine+stimulus	0.556371	0.646414	0.441163
BP_GO:0006941_striated+muscle+contraction	0.624331	0.481099	0.681106
MF_GO:0005520_insulin-like+growth+factor+binding	0.0113678	0.0590405	0.0425708
BP_GO:0006099_tricarboxylic+acid+cycle	0.616122	0.669841	0.491567
BP_GO:0051016_barbed-end+actin+filament+capping	0.154868	0.120848	0.473673
BP_GO:0030199_collagen+fibril+organization	0.690462	0.799517	0.425982
MF_GO:0004129_cytochrome-c+oxidase+activity	0.296497	0.365835	0.340939
MF_GO:0050662_coenzyme+binding	0.0637708	0.0197281	0.544819
BP_GO:0051260_protein+homooligomerization	0.532174	0.838106	0.191614
BP_GO:0032312_regulation+of+ARF+GTPase+activity	0.521448	0.600989	0.319136
MF_GO:0008060_ARF+GTPase+activator+activity	0.521448	0.600989	0.319136
MF_GO:0005178_integrin+binding	0.238433	0.274384	0.350469
BP_GO:0048536_spleen+development	0.895688	0.99419	0.135305
BP_GO:0048538_thymus+development	0.477431	0.828235	0.128985
BP_GO:0042102_positive+regulation+of+T+cell+proliferation	0.945566	0.867383	0.879454
MF_GO:0005245_voltage-gated+calcium+channel+activity	0.656176	0.772687	0.389978
BP_GO:0019233_sensory+perception+of+pain	0.884285	0.963396	0.290834
BP_GO:0007492_endoderm+development	0.919136	0.882266	0.771593
BP_GO:0007612_learning	0.639441	0.865383	0.215295
BP_GO:0042733_embryonic+digit+morphogenesis	0.674722	0.807134	0.338808
CC_GO:0001750_photoreceptor+outer+segment	0.0498273	0.102291	0.136424
MF_GO:0004869_cysteine+protease+inhibitor+activity	0.828521	0.349124	0.895157
CC_GO:0005922_connexon+complex	0.337428	0.875332	0.0287926
BP_GO:0007602_phototransduction	0.869874	0.676418	0.927983
BP_GO:0007613_memory	0.537154	0.674424	0.34244
BP_GO:0007623_circadian+rhythm	0.491378	0.769703	0.198344
BP_GO:0044267_cellular+protein+metabolic+process	0.308855	0.592656	0.173604
CC_GO:0001533_cornified+envelope	0.381258	0.766803	0.0479847
BP_GO:0006641_triacylglycerol+metabolic+process	0.630747	0.317854	0.837731
CC_GO:0001725_stress+fiber	0.965317	0.96514	0.693865
BP_GO:0006094_gluconeogenesis	0.816559	0.748721	0.748495
MF_GO:0019838_growth+factor+binding	0.0338062	0.0241106	0.335417
BP_GO:0030001_metal+ion+transport	0.826588	0.98791	0.116493
BP_GO:0006469_negative+regulation+of+protein+kinase+activity	0.125144	0.253916	0.159747
MF_GO:0005540_hyaluronic+acid+binding	0.933073	0.859113	0.861061
BP_GO:0007157_heterophilic+cell+adhesion	0.810107	0.962828	0.061557
BP_GO:0030218_erythrocyte+differentiation	0.423866	0.836531	0.0371283
BP_GO:0030155_regulation+of+cell+adhesion	0.208138	0.249137	0.320152
BP_GO:0009408_response+to+heat	0.597275	0.616915	0.506827
MF_GO:0004089_carbonate+dehydratase+activity	0.377621	0.150487	0.681728
BP_GO:0030217_T+cell+differentiation	0.112622	0.108006	0.402396
BP_GO:0007569_cell+aging	0.393443	0.284643	0.55182
BP_GO:0006820_anion+transport	0.0223492	0.00353211	0.415902
MF_GO:0016849_phosphorus-oxygen+lyase+activity	0.184383	0.362256	0.165525
BP_GO:0007585_respiratory+gaseous+exchange	0.737857	0.470861	0.828414

CC_GO:0000118_histone+deacetylase+complex	0.564923	0.703487	0.282526
BP_GO:0043410_positive+regulation+of+MAPKKK+cascade	0.305411	0.645574	0.110836
MF_GO:0005544_calcium-dependent+phospholipid+binding	0.0368085	0.0787482	0.134888
CC_GO:0016605_PML+body	0.621139	0.833067	0.255533
BP_GO:0009411_response+to+UV	0.140099	0.161743	0.306214
BP_GO:0048589_developmental+growth	0.743644	0.543747	0.778588
BP_GO:0030509_BMP+signaling+pathway	0.584381	0.701659	0.366484
BP_GO:0050679_positive+regulation+of+epithelial+cell+proliferation	0.768469	0.952329	0.204949
MF_GO:0008378_galactosyltransferase+activity	0.664817	0.702951	0.512712
BP_GO:0045597_positive+regulation+of+cell+differentiation	0.539781	0.727726	0.289065
MF_GO:0005328_neurotransmitter:sodium+symporter+activity	0.663127	0.95463	0.0910794
BP_GO:0006289_nucleotide-excision+repair	0.0963205	0.057931	0.536513
BP_GO:0035116_embryonic+hindlimb+morphogenesis	0.608411	0.739788	0.324256
MF_GO:0030414_protease+inhibitor+activity	0.872671	0.601568	0.950538
MF_GO:0019843_rRNA+binding	0.308111	0.692329	0.121543
MF_GO:0005126_hematopoietin/interferon-class+(D200-domain)+cytokine+	0.631873	0.774076	0.40179
BP_GO:0006936_muscle+contraction	0.204433	0.139511	0.48864
BP_GO:0006749_glutathione+metabolic+process	0.0266359	0.256968	0.00763642
BP_GO:0016049_cell+growth	0.125814	0.264444	0.147023
BP_GO:0018105_peptidyl-serine+phosphorylation	0.638652	0.733031	0.340735
BP_GO:0042110_T+cell+activation	0.740509	0.696586	0.651763
MF_GO:0016769_transferase+activity,+transferring+nitrogenous+groups	0.492236	0.776461	0.103288
BP_GO:0001932_regulation+of+protein+amino+acid+phosphorylation	0.521653	0.531513	0.476043
MF_GO:0031418_L-ascorbic+acid+binding	0.763651	0.679979	0.728981
BP_GO:0048535_lymph+node+development	0.128265	0.237448	0.169768
MF_GO:0051539_4+iron,+4+sulfur+cluster+binding	0.968737	0.969921	0.715495
MF_GO:0008324_cation+transmembrane+transporter+activity	0.574312	0.420836	0.693256
BP_GO:0002053_positive+regulation+of+mesenchymal+cell+proliferation	0.759496	0.82375	0.462776
BP_GO:0008016_regulation+of+heart+contraction	0.905607	0.848343	0.793464
BP_GO:0030890_positive+regulation+of+B+cell+proliferation	0.303684	0.730271	0.107871
BP_GO:0006644_phospholipid+metabolic+process	0.928186	0.901326	0.79114
CC_GO:0015630_microtubule+cytoskeleton	0.731021	0.77046	0.469011
BP_GO:0009190_cyclic+nucleotide+biosynthetic+process	0.176396	0.362256	0.147219
BP_GO:0007528_neuromuscular+junction+development	0.216636	0.292516	0.272135
BP_GO:0015711_organic+anion+transport	0.00631821	0.240411	0.00330191
BP_GO:0007566_embryo+implantation	0.834502	0.709975	0.791139
BP_GO:0032526_response+to+retinoic+acid	0.0532345	0.165519	0.097344
BP_GO:0046677_response+to+antibiotic	0.633481	0.588778	0.608075
BP_GO:0006885_regulation+of+pH	0.508005	0.863788	0.115739
CC_GO:0030176_integral+to+endoplasmic+reticulum+membrane	0.458649	0.918298	0.0432878
BP_GO:0033077_T+cell+differentiation+in+the+thymus	0.490674	0.674692	0.272636
MF_GO:0004402_histone+acetyltransferase+activity	0.407771	0.215897	0.835766
BP_GO:0001947_heart+looping	0.797422	0.930561	0.381448
BP_GO:0021766_hippocampus+development	0.692789	0.762692	0.465077
MF_GO:0004623_phospholipase+A2+activity	0.863684	0.938826	0.467855
BP_GO:0030901_midbrain+development	0.496841	0.79506	0.147894
BP_GO:0007276_gamete+generation	0.398104	0.717437	0.103669

BP_GO:0060079_regulation+of+excitatory+postsynaptic+membrane+potent	0.757542	0.660249	0.734547
MF_GO:0005261_cation+channel+activity	0.906216	0.995238	0.233415
BP_GO:0048646_anatomical+structure+formation	0.607472	0.505406	0.638488
BP_GO:0007565_female+pregnancy	0.479117	0.809892	0.0649341
MF_GO:0050840_extracellular+matrix+binding	0.411185	0.242838	0.74763
BP_GO:0001816_cytokine+production	0.861585	0.791226	0.77361
MF_GO:0008137_NADH+dehydrogenase+(ubiquinone)+activity	0.944747	0.819442	0.905034
BP_GO:0006928_cell+motion	0.898009	0.96966	0.286756
BP_GO:0008299_isoprenoid+biosynthetic+process	0.00306928	0.000335701	0.795171
BP_GO:0051092_positive+regulation+of+NF-kappaB+transcription+factor+ac	0.527663	0.342058	0.748118
MF_GO:0016706_oxidoreductase+activity,+acting+on+paired+donors,+with-	0.511276	0.408725	0.659309
MF_GO:0004709_MAP+kinase+kinase+kinase+activity	0.132566	0.113302	0.405725
MF_GO:0019001_guanyl+nucleotide+binding	0.231379	0.63329	0.0166247
BP_GO:0007611_learning+or+memory	0.4272	0.623132	0.204883
BP_GO:0009653_anatomical+structure+morphogenesis	0.603959	0.652196	0.465986
MF_GO:0004629_phospholipase+C+activity	0.0866519	0.335586	0.0318628
BP_GO:0051276_chromosome+organization	0.724063	0.697563	0.621606
BP_GO:0060021_palate+development	0.346004	0.305946	0.473754
CC_GO:0000793_condensed+chromosome	0.599267	0.426646	0.773757
BP_GO:0031069_hair+follicle+morphogenesis	0.6494	0.91154	0.0501149
BP_GO:0007595_lactation	0.973509	0.991845	0.502596
MF_GO:0008013_beta-catenin+binding	0.943949	0.943621	0.661992
BP_GO:0042113_B+cell+activation	0.581582	0.160483	0.86417
BP_GO:0001942_hair+follicle+development	0.0919506	0.252571	0.0780097
MF_GO:0015171_amino+acid+transmembrane+transporter+activity	0.616595	0.849578	0.316264
MF_GO:0016493_C-C+chemokine+receptor+activity	0.732614	0.954247	0.107553
BP_GO:0007254_JNK+cascade	0.438619	0.797766	0.0574339
CC_GO:0005669_transcription+factor+TFIID+complex	0.257911	0.415651	0.210305
BP_GO:0006888_ER+to+Golgi+vesicle-mediated+transport	0.0740454	0.153799	0.152305
MF_GO:0003712_transcription+cofactor+activity	0.740356	0.746777	0.53976
BP_GO:0000184_nuclear-transcribed+mRNA+catabolic+process,+nonsense-r	0.314744	0.336416	0.407862
CC_GO:0000795_synaptonemal+complex	0.757063	0.654873	0.73716
CC_GO:0005762_mitochondrial+large+ribosomal+subunit	0.828399	0.317519	0.924362
BP_GO:0007269_neurotransmitter+secretion	0.247085	0.459382	0.187159
CC_GO:0001673_male+germ+cell+nucleus	0.563691	0.668511	0.352329
MF_GO:0017137_Rab+GTPase+binding	0.221759	0.096752	0.681244
MF_GO:0004970_ionotropic+glutamate+receptor+activity	0.95247	0.825425	0.921178
MF_GO:0005164_tumor+necrosis+factor+receptor+binding	0.317739	0.293658	0.454171
BP_GO:0007339_binding+of+sperm+to+zona+pellucida	0.904062	0.896985	0.726651
BP_GO:0001580_detection+of+chemical+stimulus+involved+in+sensory+per	0.454025	0.351132	0.62697
CC_GO:0042612_MHC+class+I+protein+complex	0.137447	0.0660098	0.73064
BP_GO:0001974_blood+vessel+remodeling	0.932491	0.984238	0.231257
CC_GO:0031594_neuromuscular+junction	0.829134	0.925111	0.33186
BP_GO:0032880_regulation+of+protein+localization	0.323359	0.477836	0.23044
BP_GO:0030032_lamellipodium+assembly	0.885008	0.969105	0.132221
BP_GO:0050776_regulation+of+immune+response	0.99182	0.929456	0.972918
BP_GO:0010332_response+to+gamma+radiation	0.490429	0.344265	0.62625

BP_GO:0030336_negative+regulation+of+cell+migration	0.79167	0.896178	0.33897
MF_GO:0004653_polypeptide+N-acetylgalactosaminyltransferase+activity	0.410194	0.306821	0.541462
BP_GO:0042130_negative+regulation+of+T+cell+proliferation	0.116761	0.174037	0.230435
BP_GO:0007224_smoothened+signaling+pathway	0.770165	0.754525	0.618709
BP_GO:0030318_melanocyte+differentiation	0.349994	0.534847	0.263646
BP_GO:0000723_telomere+maintenance	0.439843	0.346317	0.682379
BP_GO:0007249_I-kappaB+kinase/NF-kappaB+cascade	0.0727697	0.117811	0.195976
MF_GO:0005543_phospholipid+binding	0.334625	0.627486	0.176423
BP_GO:0001502_cartilage+condensation	0.239039	0.516776	0.176257
BP_GO:0035108_limb+morphogenesis	0.779221	0.984592	0.242602
BP_GO:0001708_cell+fate+specification	0.547865	0.579054	0.499266
BP_GO:0042221_response+to+chemical+stimulus	0.0115181	0.150477	0.0190213
BP_GO:0030574_collagen+catabolic+process	0.676085	0.848852	0.350216
BP_GO:0000059_protein+import+into+nucleus,+docking	0.995979	0.985611	0.933251
CC_GO:0016363_nuclear+matrix	0.40972	0.719579	0.130613
BP_GO:0030308_negative+regulation+of+cell+growth	0.0495965	0.443972	0.014213
MF_GO:0004889_nicotinic+acetylcholine-activated+cation-selective+channe	0.441072	0.611244	0.356124
BP_GO:0008306_associative+learning	0.854295	0.822738	0.701553
CC_GO:0005932_microtubule+basal+body	0.758329	0.925394	0.136499
MF_GO:0005003_ephrin+receptor+activity	0.380666	0.133004	0.752723
BP_GO:0030534_adult+behavior	0.447659	0.721698	0.165116
MF_GO:0005234_extracellular-glutamate-gated+ion+channel+activity	0.911857	0.671332	0.921178
CC_GO:0015935_small+ribosomal+subunit	0.91095	0.748639	0.899283
CC_GO:0005863_striated+muscle+thick+filament	0.542082	0.104707	0.906461
BP_GO:0006520_amino+acid+metabolic+process	0.241235	0.382655	0.16506
MF_GO:0004697_protein+kinase+C+activity	0.135863	0.131183	0.475041
BP_GO:0007512_adult+heart+development	0.861067	0.396244	0.969331
MF_GO:0046914_transition+metal+ion+binding	0.0134635	0.00720581	0.354753
CC_GO:0005581_collagen	0.220978	0.527751	0.0965991
BP_GO:0006637_acyl-CoA+metabolic+process	0.31184	0.423958	0.311274
MF_GO:0051537_2+iron,+2+sulfur+cluster+binding	0.484632	0.258921	0.683877
BP_GO:0030301_cholesterol+transport	0.889394	0.678792	0.945323
BP_GO:0001934_positive+regulation+of+protein+amino+acid+phosphorylati	0.4021	0.478019	0.372921
MF_GO:0005539_glycosaminoglycan+binding	0.142689	0.250062	0.199563
MF_GO:0046873_metal+ion+transmembrane+transporter+activity	0.926144	0.98791	0.175078
BP_GO:0048286_alveolus+development	0.926685	0.959545	0.467151
BP_GO:0008219_cell+death	0.146124	0.455752	0.0773477
BP_GO:0007040_lysosome+organization	0.830612	0.943986	0.277676
BP_GO:0044237_cellular+metabolic+process	0.329107	0.216664	0.602775
BP_GO:0016525_negative+regulation+of+angiogenesis	0.883655	0.656348	0.918582
BP_GO:0048066_pigmentation+during+development	0.750912	0.95258	0.164991
BP_GO:0019370_leukotriene+biosynthetic+process	0.992148	0.954719	0.958102
BP_GO:0042445_hormone+metabolic+process	0.25452	0.673799	0.0925236
MF_GO:0050661_NADP+binding	0.210432	0.205504	0.438271
BP_GO:0051789_response+to+protein+stimulus	0.424473	0.223736	0.673681
CC_GO:0005912_adherens+junction	0.664635	0.690674	0.458566
BP_GO:0048873_homeostasis+of+number+of+cells+within+a+tissue	0.764658	0.896146	0.297494

CC_GO:0005903_brush+border	0.74727	0.674182	0.688085
MF_GO:0046961_proton-transporting+ATPase+activity,+rotational+mechani	0.830174	0.788501	0.701823
MF_GO:0004860_protein+kinase+inhibitor+activity	0.918126	0.874791	0.784709
BP_GO:0031016_pancreas+development	0.766273	0.926863	0.264462
MF_GO:0005484_SNAP+receptor+activity	0.590889	0.643147	0.413209
BP_GO:0016043_cellular+component+organization	0.863408	0.882308	0.42254
BP_GO:0060041_retina+development+in+camera-type+eye	0.788683	0.818172	0.541879
MF_GO:0005057_receptor+signaling+protein+activity	0.0520544	0.0241797	0.313155
MF_GO:0016818_hydrolase+activity,+acting+on+acid+anhydrides,+in+phosp	0.936177	0.972996	0.466167
BP_GO:0042060_wound+healing	0.793356	0.840942	0.471953
BP_GO:0035115_embryonic+forelimb+morphogenesis	0.990285	0.845484	0.992896
BP_GO:0048593_camera-type+eye+morphogenesis	0.25353	0.402607	0.190524
CC_GO:0000776_kinetochore	0.959147	0.910546	0.870352
BP_GO:0006302_double-strand+break+repair	0.724278	0.826492	0.474806
BP_GO:0016998_cell+wall+catabolic+process	0.248465	0.319578	0.303807
MF_GO:0003995_acyl-CoA+dehydrogenase+activity	0.256908	0.176731	0.718661
BP_GO:0031290_retinal+ganglion+cell+axon+guidance	0.353168	0.29688	0.479773
BP_GO:0042384_cilium+assembly	0.338605	0.672165	0.034097
MF_GO:0016566_specific+transcriptional+repressor+activity	0.842977	0.717764	0.840311
CC_GO:0000781_chromosome,+telomeric+region	0.26962	0.340165	0.201284
BP_GO:0030902_hindbrain+development	0.163332	0.0607134	0.688581
BP_GO:0001824_blastocyst+development	0.750416	0.882373	0.350363
BP_GO:0007422_peripheral+nervous+system+development	0.346721	0.747133	0.0521641
MF_GO:0004864_protein+phosphatase+inhibitor+activity	0.63836	0.510293	0.688469
BP_GO:0050680_negative+regulation+of+epithelial+cell+proliferation	0.298942	0.221555	0.599111
BP_GO:0048167_regulation+of+synaptic+plasticity	0.807906	0.939271	0.18871
BP_GO:0000398_nuclear+mRNA+splicing,+via+spliceosome	0.660703	0.895448	0.312183
MF_GO:0004190_aspartic-type+endopeptidase+activity	0.653707	0.875851	0.215262
BP_GO:0021983_pituitary+gland+development	0.744977	0.79557	0.512366
MF_GO:0005212_structural+constituent+of+eye+lens	0.206426	0.714706	0.0492906
BP_GO:0048839_inner+ear+development	0.406538	0.824192	0.022571
BP_GO:0001829_trophectodermal+cell+differentiation	0.617007	0.501925	0.666865
BP_GO:0007214_gamma-aminobutyric+acid+signaling+pathway	0.331724	0.666456	0.159153
CC_GO:0042734_presynaptic+membrane	0.376219	0.272918	0.621134
MF_GO:0008227_amine+receptor+activity	0.918414	0.983932	0.462788
CC_GO:0022626_cytosolic+ribosome	0.917557	0.757469	0.937463
BP_GO:0042157_lipoprotein+metabolic+process	0.0411334	0.0134352	0.930778
BP_GO:0002474_antigen+processing+and+presentation+of+peptide+antiger	0.142105	0.0631614	0.73064
CC_GO:0030017_sarcomere	0.223233	0.435609	0.180219
BP_GO:0019886_antigen+processing+and+presentation+of+exogenous+pep	0.837367	0.309808	0.958369
BP_GO:0006839_mitochondrial+transport	0.0154039	0.243228	0.00627558
BP_GO:0006071_glycerol+metabolic+process	0.216505	0.305482	0.272266
BP_GO:0007178_transmembrane+receptor+protein+serine/threonine+kinas	0.861175	0.630312	0.874737
BP_GO:0006635_fatty+acid+beta-oxidation	0.375572	0.356759	0.48497
CC_GO:0005778_peroxisomal+membrane	0.910073	0.829657	0.858781
BP_GO:0048514_blood+vessel+morphogenesis	0.765552	0.588629	0.819293
BP_GO:0001759_induction+of+an+organ	0.877657	0.862977	0.68457

BP_GO:0042098_T+cell+proliferation	0.791617	0.522545	0.86278
CC_GO:0008287_protein+serine/threonine+phosphatase+complex	0.987377	0.987508	0.769962
BP_GO:0016485_protein+processing	0.257044	0.757889	0.0211356
BP_GO:0001958_endochondral+ossification	0.43925	0.782832	0.127978
BP_GO:0006471_protein+amino+acid+ADP-ribosylation	0.189767	0.362843	0.158676
MF_GO:0004707_MAP+kinase+activity	0.916346	0.95143	0.534412
MF_GO:0004437_inositol+or+phosphatidylinositol+phosphatase+activity	0.692912	0.567337	0.899328
MF_GO:0016747_transferase+activity,+transferring+acyl+groups+other+thai	0.578323	0.424605	0.749019
CC_GO:0005614_interstitial+matrix	0.0364953	0.305095	0.0267577
MF_GO:0004435_phosphoinositide+phospholipase+C+activity	0.155461	0.546852	0.0318628
MF_GO:0005247_voltage-gated+chloride+channel+activity	0.109991	0.355395	0.074107
BP_GO:0051402_neuron+apoptosis	0.120345	0.142255	0.265825
MF_GO:0004198_calcium-dependent+cysteine-type+endopeptidase+activity	0.992715	0.990145	0.869702
MF_GO:0017048_Rho+GTPase+binding	0.902286	0.894488	0.641762
MF_GO:0008514_organic+anion+transmembrane+transporter+activity	0.01051	0.0182259	0.120482
BP_GO:0006665_sphingolipid+metabolic+process	0.331757	0.793918	0.099333
MF_GO:0004835_tubulin-tyrosine+ligase+activity	0.15444	0.123791	0.469962
BP_GO:0006700_C21-steroid+hormone+biosynthetic+process	0.163211	0.0499111	0.655922
BP_GO:0009409_response+to+cold	0.252434	0.376321	0.201197
MF_GO:0005242_inward+rectifier+potassium+channel+activity	0.85588	0.893975	0.627529
MF_GO:0005070_SH3/SH2+adaptor+activity	0.61865	0.252144	0.947641
BP_GO:0001782_B+cell+homeostasis	0.845036	0.885554	0.618809
BP_GO:0008624_induction+of+apoptosis+by+extracellular+signals	0.369258	0.250527	0.533981
BP_GO:0009966_regulation+of+signal+transduction	0.334645	0.0892616	0.770809
BP_GO:0001662_behavioral+fear+response	0.709815	0.856283	0.308028
MF_GO:0008553_hydrogen-exporting+ATPase+activity,+phosphorylative+m	0.655531	0.879076	0.0352217
MF_GO:0008022_protein+C-terminus+binding	0.00861761	0.0813673	0.0122184
BP_GO:0030514_negative+regulation+of+BMP+signaling+pathway	0.338408	0.471833	0.302037
CC_GO:0019897_extrinsic+to+plasma+membrane	0.441582	0.5338	0.341365
BP_GO:0032012_regulation+of+ARF+protein+signal+transduction	0.938604	0.853176	0.880013
MF_GO:0005086_ARF+guanyl-nucleotide+exchange+factor+activity	0.938604	0.853176	0.880013
BP_GO:0001975_response+to+amphetamine	0.65182	0.808814	0.30209
BP_GO:0006284_base-excision+repair	0.205102	0.87405	0.018698
CC_GO:0014069_postsynaptic+density	0.362628	0.759924	0.0379951
BP_GO:0042491_auditory+receptor+cell+differentiation	0.094892	0.190299	0.124173
BP_GO:0035176_social+behavior	0.216927	0.28718	0.292217
BP_GO:0007338_single+fertilization	0.0943117	0.198498	0.15723
BP_GO:0006414_translational+elongation	0.00474347	0.00614949	0.102998
MF_GO:0015380_anion+exchanger+activity	0.407045	0.522329	0.35972
BP_GO:0031018_endocrine+pancreas+development	0.550504	0.543929	0.525389
BP_GO:0048169_regulation+of+long-term+neuronal+synaptic+plasticity	0.143838	0.542093	0.0403582
CC_GO:0030057_desmosome	0.301794	0.279521	0.466252
BP_GO:0002009_morphogenesis+of+an+epithelium	0.275772	0.457798	0.215608
BP_GO:0045214_sarcomere+organization	0.980882	0.431311	0.998586
BP_GO:0050766_positive+regulation+of+phagocytosis	0.473654	0.336779	0.639931
MF_GO:0005537_mannose+binding	0.608339	0.572596	0.607583
BP_GO:0016044_membrane+organization	0.0385104	0.384856	0.00653266

MF_GO:0003950_NAD++ADP-ribosyltransferase+activity	0.117103	0.115837	0.384896
BP_GO:0030168_platelet+activation	0.862129	0.930549	0.332753
BP_GO:0031668_cellular+response+to+extracellular+stimulus	0.403858	0.458393	0.353743
MF_GO:0016805_dipeptidase+activity	0.729025	0.784045	0.519818
CC_GO:0005626_insoluble+fraction	0.689687	0.837917	0.406167
BP_GO:0006898_receptor-mediated+endocytosis	0.172381	0.0587004	0.513228
CC_GO:0030175_filopodium	0.243556	0.174188	0.561289
MF_GO:0004467_long-chain-fatty-acid-CoA+ligase+activity	0.58464	0.320071	0.861684
MF_GO:0005518_collagen+binding	0.79207	0.597736	0.810736
BP_GO:0030099_myeloid+cell+differentiation	0.26137	0.0379761	0.779948
MF_GO:0030165_PDZ+domain+binding	0.0742885	0.0409971	0.566917
MF_GO:0008047_enzyme+activator+activity	0.977395	0.401916	0.997025
BP_GO:0030593_neutrophil+chemotaxis	0.835563	0.819431	0.686603
BP_GO:0007026_negative+regulation+of+microtubule+depolymerization	0.0725145	0.163579	0.123419
MF_GO:0004407_histone+deacetylase+activity	0.442118	0.788119	0.0388401
BP_GO:0006833_water+transport	0.0400381	0.0218738	0.392852
BP_GO:0006171_cAMP+biosynthetic+process	0.278618	0.722857	0.0450677
BP_GO:0007274_neuromuscular+synaptic+transmission	0.324036	0.820676	0.0138848
CC_GO:0030315_T-tubule	0.0748149	0.137827	0.15632
BP_GO:0002062_chondrocyte+differentiation	0.405952	0.774998	0.0590928
BP_GO:0009611_response+to+wounding	0.845766	0.747252	0.799185
BP_GO:0007076_mitotic+chromosome+condensation	0.781778	0.926309	0.220715
MF_GO:0004143_diacylglycerol+kinase+activity	0.458348	0.122062	0.825396
BP_GO:0050900_leukocyte+migration	0.36026	0.659191	0.166143
BP_GO:0007031_peroxisome+organization	0.319755	0.245376	0.61173
CC_GO:0005891_voltage-gated+calcium+channel+complex	0.659899	0.680755	0.528367
BP_GO:0001836_release+of+cytochrome+c+from+mitochondria	0.282683	0.422329	0.28171
BP_GO:0050905_neuromuscular+process	0.9451	0.886032	0.85385
BP_GO:0008045_motor+axon+guidance	0.54908	0.537741	0.532254
BP_GO:0050880_regulation+of+blood+vessel+size	0.975493	0.995113	0.543181
BP_GO:0007405_neuroblast+proliferation	0.946839	0.99067	0.355651
BP_GO:0048856_anatomical+structure+development	0.686227	0.771242	0.437761
BP_GO:0050808_synapse+organization	0.745197	0.683148	0.714047
MF_GO:0003993_acid+phosphatase+activity	0.45211	0.509505	0.400425
BP_GO:0044419_interspecies+interaction+between+organisms	0.988242	0.966117	0.932387
BP_GO:0008272_sulfate+transport	0.399649	0.438251	0.418381
BP_GO:0030282_bone+mineralization	0.237492	0.806834	0.0234422
BP_GO:0015671_oxygen+transport	0.792541	0.53643	0.937276
MF_GO:0005344_oxygen+transporter+activity	0.792541	0.53643	0.937276
MF_GO:0030552_cAMP+binding	0.822637	0.910115	0.365812
BP_GO:0050852_T+cell+receptor+signaling+pathway	0.465568	0.563345	0.366696
BP_GO:0030878_thyroid+gland+development	0.940516	0.896765	0.861463
CC_GO:0000794_condensed+nuclear+chromosome	0.558991	0.733283	0.252615
BP_GO:0006367_transcription+initiation+from+RNA+polymerase+II+promot	0.160988	0.0765973	0.639603
BP_GO:0048477_oogenesis	0.72556	0.89375	0.151905
BP_GO:0019827_stem+cell+maintenance	0.272935	0.5312	0.165155
BP_GO:0001654_eye+development	0.0382878	0.148852	0.0756844

BP_GO:0042462_eye+photoreceptor+cell+development	0.0946008	0.276226	0.0698988
BP_GO:0001964_startle+response	0.714955	0.903215	0.285189
MF_GO:0005184_neuropeptide+hormone+activity	0.663185	0.872764	0.270867
BP_GO:0001709_cell+fate+determination	0.449881	0.610221	0.313015
CC_GO:0016607_nuclear+speck	0.863032	0.930403	0.458044
BP_GO:0035249_synaptic+transmission,+glutamatergic	0.521031	0.312953	0.795993
BP_GO:0045666_positive+regulation+of+neuron+differentiation	0.644331	0.641279	0.561434
BP_GO:0043434_response+to+peptide+hormone+stimulus	0.556926	0.294329	0.805793
BP_GO:0019915_sequestering+of+lipid	0.0798522	0.022668	0.743827
CC_GO:0030016_myofibril	0.638408	0.320429	0.777586
CC_GO:0005811_lipid+particle	0.00303941	0.000825272	0.485262
BP_GO:0043029_T+cell+homeostasis	0.786148	0.737374	0.683141
BP_GO:0009749_response+to+glucose+stimulus	0.82499	0.7197	0.770595
MF_GO:0004806_triacylglycerol+lipase+activity	0.514338	0.305544	0.744762
CC_GO:0005605_basal+lamina	0.56556	0.601223	0.481204
BP_GO:0018107_peptidyl-threonine+phosphorylation	0.0831155	0.132534	0.200749
BP_GO:0048844_artery+morphogenesis	0.664887	0.633368	0.603426
BP_GO:0016064_immunoglobulin+mediated+immune+response	0.341199	0.169693	0.646893
BP_GO:0043123_positive+regulation+of+I-kappaB+kinase/NF-kappaB+casca	0.191558	0.146389	0.428017
BP_GO:0050853_B+cell+receptor+signaling+pathway	0.487531	0.495717	0.485962
BP_GO:0006541_glutamine+metabolic+process	0.466531	0.347994	0.65831
MF_GO:0004012_phospholipid-translocating+ATPase+activity	0.0231383	0.0449998	0.146846
CC_GO:0030139_endocytic+vesicle	0.191501	0.281557	0.228601
BP_GO:0043113_receptor+clustering	0.23616	0.265654	0.35964
BP_GO:0006479_protein+amino+acid+methylation	0.132765	0.0546742	0.458211
BP_GO:0055010_ventricular+cardiac+muscle+morphogenesis	0.0909589	0.294953	0.0886426
BP_GO:0007204_elevation+of+cytosolic+calcium+ion+concentration	0.831942	0.876712	0.580317
BP_GO:0051085_chaperone+cofactor-dependent+protein+folding	0.594481	0.040816	0.936251
BP_GO:0035265_organ+growth	0.23702	0.224954	0.414893
BP_GO:0016331_morphogenesis+of+embryonic+epithelium	0.304971	0.411409	0.309468
CC_GO:0000792_heterochromatin	0.697933	0.953981	0.0688491
MF_GO:0005158_insulin+receptor+binding	0.956776	0.969962	0.622464
BP_GO:0042474_middle+ear+morphogenesis	0.246575	0.140348	0.505741
BP_GO:0016571_histone+methylation	0.177855	0.178591	0.383044
BP_GO:0017148_negative+regulation+of+translation	0.862698	0.866802	0.539678
BP_GO:0006904_vesicle+docking+during+exocytosis	0.334016	0.475055	0.277546
CC_GO:0017053_transcriptional+repressor+complex	0.593885	0.460423	0.686649
CC_GO:0000159_protein+phosphatase+type+2A+complex	0.975623	0.997247	0.193334
BP_GO:0007498_mesoderm+development	0.531583	0.645762	0.347876
BP_GO:0045669_positive+regulation+of+osteoblast+differentiation	0.116578	0.328975	0.0750001
BP_GO:0007259_JAK-STAT+cascade	0.412309	0.681887	0.146236
CC_GO:0005871_kinesin+complex	0.602936	0.369471	0.864474
BP_GO:0006493_protein+amino+acid+O-linked+glycosylation	0.987773	0.985343	0.818342
BP_GO:0010001_glial+cell+differentiation	0.215466	0.28104	0.294176
BP_GO:0035050_embryonic+heart+tube+development	0.604139	0.654158	0.504491
BP_GO:0006164_purine+nucleotide+biosynthetic+process	0.794009	0.526829	0.845696
BP_GO:0046330_positive+regulation+of+JNK+cascade	0.104041	0.147389	0.242269

BP_GO:0043161_proteasomal+ubiquitin-dependent+protein+catabolic+proc	0.240153	0.738726	0.0715601
BP_GO:0000209_protein+polyubiquitination	0.129229	0.384684	0.0613843
MF_GO:0015450_P-P-bond-hydrolysis-driven+protein+transmembrane+tran	0.72142	0.948091	0.0501328
BP_GO:0030968_endoplasmic+reticulum+unfolded+protein+response	0.866161	0.882344	0.623303
MF_GO:0005149_interleukin-1+receptor+binding	0.0969625	0.0468144	0.714908
BP_GO:0050850_positive+regulation+of+calcium-mediated+signaling	0.870442	0.982206	0.0833274
BP_GO:0007205_activation+of+protein+kinase+C+activity	0.521015	0.239815	0.782886
CC_GO:0045120_pronucleus	0.335087	0.297549	0.456878
BP_GO:0045860_positive+regulation+of+protein+kinase+activity	0.522687	0.715951	0.19081
CC_GO:0005788_endoplasmic+reticulum+lumen	0.235684	0.920963	0.00754692
MF_GO:0003690_double-stranded+DNA+binding	0.791082	0.841203	0.456282
BP_GO:0045190_isotype+switching	0.247691	0.714112	0.0237886
BP_GO:0006298_mismatch+repair	0.444812	0.62269	0.247568
MF_GO:0000049_tRNA+binding	0.953023	0.972707	0.626258
BP_GO:0045595_regulation+of+cell+differentiation	0.21288	0.280191	0.292903
CC_GO:0044424_intracellular+part	0.270066	0.237141	0.48051
MF_GO:0004993_serotonin+receptor+activity	0.192229	0.831329	0.0406538
CC_GO:0000178_exosome+(RNase+complex)	0.676276	0.812623	0.366537
CC_GO:0000300_peripheral+to+membrane+of+membrane+fraction	0.810137	0.945561	0.0757994
MF_GO:0008527_taste+receptor+activity	0.324683	0.236888	0.68766
BP_GO:0030833_regulation+of+actin+filament+polymerization	0.0205419	0.00695992	0.300966
BP_GO:0006911_phagocytosis,+engulfment	0.0364955	0.0125379	0.496045
BP_GO:0006807_nitrogen+compound+metabolic+process	0.0782584	0.102278	0.263697
MF_GO:0030674_protein+binding,+bridging	0.888297	0.970933	0.468224
CC_GO:0031941_filamentous+actin	0.545412	0.802786	0.349381
MF_GO:0008484_sulfuric+ester+hydrolase+activity	0.0983314	0.0711434	0.4207
BP_GO:0048738_cardiac+muscle+development	0.406859	0.548248	0.12182
MF_GO:0010181_FMN+binding	0.867347	0.965908	0.104445
BP_GO:0050830_defense+response+to+Gram-positive+bacterium	0.926897	0.926897	#N/A
MF_GO:0048037_cofactor+binding	0.597631	0.51934	0.683721
BP_GO:0015914_phospholipid+transport	0.00587309	0.00851703	0.198029
BP_GO:0046716_muscle+maintenance	0.418575	0.660988	0.141934
BP_GO:0042573_retinoic+acid+metabolic+process	0.735745	0.295982	0.91044
BP_GO:0030048_actin+filament-based+movement	0.757937	0.748167	0.598249
BP_GO:0007162_negative+regulation+of+cell+adhesion	0.199748	0.195849	0.395894
CC_GO:0000299_integral+to+membrane+of+membrane+fraction	0.740367	0.949883	0.19937
BP_GO:0051017_actin+filament+bundle+formation	0.0761129	0.0528258	0.460466
MF_GO:0015250_water+channel+activity	0.0158109	0.00387861	0.392852
BP_GO:0031663_lipopolysaccharide-mediated+signaling+pathway	0.58982	0.464822	0.689434
MF_GO:0004675_transmembrane+receptor+protein+serine/threonine+kina	0.917054	0.873197	0.784924
MF_GO:0005024_transforming+growth+factor+beta+receptor+activity	0.917054	0.873197	0.784924
MF_GO:0004016_adenylate+cyclase+activity	0.280838	0.638825	0.0759759
BP_GO:0043085_positive+regulation+of+catalytic+activity	0.288183	0.903918	0.0119907
MF_GO:0017110_nucleoside-diphosphatase+activity	0.489164	0.564039	0.398263
MF_GO:0050897_cobalt+ion+binding	0.0476392	0.0700316	0.216896
BP_GO:0048598_embryonic+morphogenesis	0.557192	0.60139	0.442932
BP_GO:0007200_G-protein+signaling,+coupled+to+IP3+second+messenger+	0.32352	0.430748	0.298633

MF_GO:0008603_cAMP-dependent+protein+kinase+regulator+activity	0.578043	0.578043	#N/A
CC_GO:0033177_proton-transporting+two-sector+ATPase+complex,+proton	0.539416	0.692133	0.399465
BP_GO:0046847_filopodium+formation	0.827201	0.87772	0.510261
BP_GO:0042771_DNA+damage+response,+signal+transduction+by+p53+clas	0.461203	0.207649	0.689299
BP_GO:0051145_smooth+muscle+cell+differentiation	0.578448	0.890708	0.0969528
MF_GO:0016790_thiolester+hydrolase+activity	0.69828	0.828539	0.474143
BP_GO:0032869_cellular+response+to+insulin+stimulus	0.979425	0.918438	0.938645
CC_GO:0031105_septin+complex	0.240509	0.149943	0.634625
BP_GO:0048015_phosphoinositide-mediated+signaling	0.276096	0.31172	0.368287
MF_GO:0004683_calmodulin-dependent+protein+kinase+activity	0.00763784	0.0218198	0.0882549
MF_GO:0008235_metalloexopeptidase+activity	0.504396	0.795723	0.187126
BP_GO:0048701_embryonic+cranial+skeleton+morphogenesis	0.361353	0.292311	0.507499
CC_GO:0005942_phosphoinositide+3-kinase+complex	0.470432	0.619971	0.260897
MF_GO:0004926_non-G-protein+coupled+7TM+receptor+activity	0.963559	0.99089	0.530055
BP_GO:0007190_activation+of+adenylate+cyclase+activity	0.430178	0.898447	0.0489698
CC_GO:0005884_actin+filament	0.712222	0.559905	0.840165
BP_GO:0050728_negative+regulation+of+inflammatory+response	0.375135	0.370315	0.456546
BP_GO:0048821_erythrocyte+development	0.0836369	0.252336	0.0937565
BP_GO:0001837_epithelial+to+mesenchymal+transition	0.336591	0.477386	0.266224
MF_GO:0046933_hydrogen+ion+transporting+ATP+synthase+activity,+rotat	0.972017	0.884216	0.943974
MF_GO:0004428_inositol+or+phosphatidylinositol+kinase+activity	0.453563	0.667094	0.176522
BP_GO:0009888_tissue+development	0.375125	0.528076	0.267355
CC_GO:0005902_microvillus	0.424013	0.474378	0.408125
BP_GO:0016197_endosome+transport	0.42475	0.492232	0.38647
BP_GO:0006809_nitric+oxide+biosynthetic+process	0.934972	0.781352	0.971649
BP_GO:0040015_negative+regulation+of+multicellular+organism+growth	0.191395	0.26816	0.254135
CC_GO:0031227_intrinsic+to+endoplasmic+reticulum+membrane	0.20343	0.333219	0.232826
MF_GO:0015269_calcium-activated+potassium+channel+activity	0.266734	0.0720443	0.750721
BP_GO:0030539_male+genitalia+development	0.529661	0.495998	0.548535
CC_GO:0005720_nuclear+heterochromatin	0.160947	0.799804	0.0174789
BP_GO:0055007_cardiac+muscle+cell+differentiation	0.882602	0.84159	0.741318
MF_GO:0001619_lysosphingolipid+and+lysophosphatidic+acid+receptor+act	0.411787	0.850482	0.184953
BP_GO:0007340_acrosome+reaction	0.528128	0.611685	0.39829
BP_GO:0010165_response+to+X-ray	0.518447	0.552756	0.483338
BP_GO:0008543_fibroblast+growth+factor+receptor+signaling+pathway	0.863173	0.97734	0.120551
BP_GO:0042417_dopamine+metabolic+process	0.861681	0.978609	0.353829
CC_GO:0043197_dendritic+spine	0.308266	0.384642	0.315685
BP_GO:0018298_protein-chromophore+linkage	0.924217	0.888605	0.786101
BP_GO:0016070_RNA+metabolic+process	0.560015	0.590667	0.447652
BP_GO:0016573_histone+acetylation	0.599857	0.786234	0.351584
BP_GO:0006342_chromatin+silencing	0.855871	0.745058	0.813448
CC_GO:0030672_synaptic+vesicle+membrane	0.360743	0.302408	0.515345
BP_GO:0006968_cellular+defense+response	0.318826	0.345764	0.399885
CC_GO:0005814_centriole	0.325142	0.430531	0.301489
BP_GO:0007530_sex+determination	0.783483	0.723931	0.764882
BP_GO:0007548_sex+differentiation	0.646613	0.579553	0.640268
BP_GO:0002088_lens+development+in+camera-type+eye	0.60932	0.863875	0.214644

MF_GO:0019905_syntaxin+binding	0.949979	0.949979	#N/A
BP_GO:0007416_synaptogenesis	0.885604	0.976193	0.26642
CC_GO:0005758_mitochondrial+intermembrane+space	0.975339	0.88214	0.954817
BP_GO:0001522_pseudouridine+synthesis	0.522028	0.94681	0.0809812
MF_GO:0009982_pseudouridine+synthase+activity	0.522028	0.94681	0.0809812
BP_GO:0048265_response+to+pain	0.515556	0.796976	0.197145
BP_GO:0031589_cell-substrate+adhesion	0.928333	0.905164	0.746093
BP_GO:0006957_complement+activation,+alternative+pathway	0.819106	0.979483	0.0727219
MF_GO:0008307_structural+constituent+of+muscle	0.721794	0.432772	0.815478
BP_GO:0019363_pyridine+nucleotide+biosynthetic+process	0.493582	0.704133	0.198085
BP_GO:0048741_skeletal+muscle+fiber+development	0.705263	0.824581	0.513985
BP_GO:0006937_regulation+of+muscle+contraction	0.6297	0.550848	0.643473
BP_GO:0006825_copper+ion+transport	0.691498	0.714781	0.555937
BP_GO:0007159_leukocyte+adhesion	0.97615	0.994745	0.245402
MF_GO:0016290_palmitoyl-CoA+hydrolase+activity	0.316808	0.767631	0.0495602
BP_GO:0032760_positive+regulation+of+tumor+necrosis+factor+production	0.797642	0.912048	0.430906
BP_GO:0043087_regulation+of+GTPase+activity	0.0109782	0.0434587	0.0582933
CC_GO:0045263_proton-transporting+ATP+synthase+complex,+coupling+fa	0.942511	0.709938	0.943453
BP_GO:0009072_aromatic+amino+acid+family+metabolic+process	0.173149	0.276956	0.178117
MF_GO:0003954_NADH+dehydrogenase+activity	0.560741	0.336088	0.649006
BP_GO:0055085_transmembrane+transport	0.603385	0.753038	0.413154
MF_GO:0022891_substrate-specific+transmembrane+transporter+activity	0.603385	0.753038	0.413154
BP_GO:0001676_long-chain+fatty+acid+metabolic+process	0.845423	0.941705	0.377179
BP_GO:0042744_hydrogen+peroxide+catabolic+process	0.853411	0.521465	0.905583
BP_GO:0048813_dendrite+morphogenesis	0.803756	0.698887	0.76267
MF_GO:0005355_glucose+transmembrane+transporter+activity	0.485669	0.527873	0.464818
MF_GO:0004935_adrenoceptor+activity	0.379761	0.457884	0.318176
MF_GO:0016209_antioxidant+activity	0.188578	0.230823	0.246447
BP_GO:0022008_neurogenesis	0.498262	0.782668	0.0578638
BP_GO:0046676_negative+regulation+of+insulin+secretion	0.811949	0.823701	0.626743
CC_GO:0001772_immunological+synapse	0.832021	0.954713	0.370502
BP_GO:0009116_nucleoside+metabolic+process	0.156645	0.195116	0.285376
BP_GO:0042640_anagen	0.158931	0.0906051	0.421486
MF_GO:0008143_poly(A)+binding	0.973619	0.964343	0.825725
CC_GO:0012506_vesicle+membrane	0.998084	0.998836	0.504499
BP_GO:0006691_leukotriene+metabolic+process	0.978223	0.902519	0.945868
BP_GO:0042755_eating+behavior	0.484014	0.726498	0.255124
BP_GO:0006309_DNA+fragmentation+during+apoptosis	0.000705538	0.000157135	0.180627
BP_GO:0006783_heme+biosynthetic+process	0.146989	0.11005	0.437437
MF_GO:0016291_acyl-CoA+thioesterase+activity	0.0542816	0.349558	0.0298256
MF_GO:0015075_ion+transmembrane+transporter+activity	0.467719	0.83037	0.235239
MF_GO:0019903_protein+phosphatase+binding	0.638622	0.55331	0.700734
BP_GO:0043330_response+to+exogenous+dsRNA	0.796583	0.597341	0.843973
BP_GO:0002026_regulation+of+the+force+of+heart+contraction	0.49727	0.0949408	0.995467
MF_GO:0005385_zinc+ion+transmembrane+transporter+activity	0.593168	0.679804	0.388705
BP_GO:0051899_membrane+depolarization	0.445628	0.328391	0.599265
BP_GO:0030100_regulation+of+endocytosis	0.4004	0.418133	0.429584

BP_GO:0048010_vascular+endothelial+growth+factor+receptor+signaling+p	0.0604991	0.279489	0.0412178
CC_GO:0005761_mitochondrial+ribosome	0.873413	0.777782	0.802866
BP_GO:0001516_prostaglandin+biosynthetic+process	0.740552	0.82099	0.567562
BP_GO:0046488_phosphatidylinositol+metabolic+process	0.455915	0.548473	0.37303
BP_GO:0019722_calcium-mediated+signaling	0.075662	0.37362	0.0304169
BP_GO:0000045_autophagic+vacuole+formation	0.856166	0.741146	0.83013
BP_GO:0008630_DNA+damage+response,+signal+transduction+resulting+in	0.768162	0.757469	0.60738
BP_GO:0050821_protein+stabilization	0.500828	0.484576	0.52532
BP_GO:0010467_gene+expression	0.541696	0.723767	0.287209
BP_GO:0015672_monovalent+inorganic+cation+transport	0.733552	0.728529	0.608124
BP_GO:0006801_superoxide+metabolic+process	0.16203	0.643725	0.0577645
BP_GO:0046928_regulation+of+neurotransmitter+secretion	0.0676211	0.281009	0.0626295
BP_GO:0006857_oligopeptide+transport	0.978087	0.983105	0.503107
BP_GO:0009880_embryonic+pattern+specification	0.711249	0.773268	0.449342
CC_GO:0005876_spindle+microtubule	0.220274	0.637374	0.124742
BP_GO:0001833_inner+cell+mass+cell+proliferation	0.976263	0.912945	0.929398
BP_GO:0006352_transcription+initiation	0.95864	0.998507	0.303744
BP_GO:0001556_oocyte+maturation	0.386629	0.48323	0.3222
CC_GO:0016604_nuclear+body	0.22475	0.715198	0.101022
BP_GO:0002052_positive+regulation+of+neuroblast+proliferation	0.697415	0.835643	0.292003
MF_GO:0004602_glutathione+peroxidase+activity	0.846679	0.515449	0.884389
MF_GO:0031625_ubiquitin+protein+ligase+binding	0.998426	0.994917	0.946107
CC_GO:0032420_stereocilium	0.0269552	0.215155	0.0102793
BP_GO:0006446_regulation+of+translational+initiation	0.360872	0.490862	0.315454
CC_GO:0005657_replication+fork	0.50436	0.438994	0.567297
CC_GO:0005892_nicotinic+acetylcholine-gated+receptor-channel+complex	0.551063	0.814947	0.237562
BP_GO:0045494_photoreceptor+cell+maintenance	0.137772	0.0269119	0.738174
MF_GO:0008509_anion+transmembrane+transporter+activity	0.122402	0.35233	0.084542
MF_GO:0001664_G-protein-coupled+receptor+binding	0.373114	0.665799	0.15006
BP_GO:0006183_GTP+biosynthetic+process	0.79807	0.587344	0.815488
MF_GO:0015035_protein+disulfide+oxidoreductase+activity	0.342715	0.114759	0.558692
BP_GO:0006182_cGMP+biosynthetic+process	0.224895	0.26314	0.337682
BP_GO:0007257_activation+of+JNK+activity	0.288903	0.350457	0.333834
BP_GO:0048566_embryonic+gut+development	0.11838	0.286638	0.142829
BP_GO:0019226_transmission+of+nerve+impulse	0.901442	0.860568	0.764266
BP_GO:0007631_feeding+behavior	0.986555	0.997282	0.419002
BP_GO:0010212_response+to+ionizing+radiation	0.428354	0.184149	0.793049
MF_GO:0043022_ribosome+binding	0.624544	0.897131	0.266808
BP_GO:0006402_mRNA+catabolic+process	0.511812	0.838294	0.122555
BP_GO:0060052_neurofilament+cytoskeleton+organization	0.325763	0.658717	0.0735771
BP_GO:0048266_behavioral+response+to+pain	0.395	0.624035	0.109685
MF_GO:0004843_ubiquitin-specific+protease+activity	0.835309	0.867074	0.571648
MF_GO:0030983_mismatched+DNA+binding	0.523031	0.736272	0.247568
BP_GO:0021549_cerebellum+development	0.729023	0.69432	0.632439
BP_GO:0043523_regulation+of+neuron+apoptosis	0.865205	0.971362	0.530416
MF_GO:0008271_secondary+active+sulfate+transmembrane+transporter+a	0.511034	0.616666	0.418381
MF_GO:0004983_neuropeptide+Y+receptor+activity	0.474334	0.505402	0.468747

BP_GO:0016265_death	0.721123	0.804106	0.406254
BP_GO:0030238_male+sex+determination	0.294758	0.863768	0.0314245
CC_GO:0015934_large+ribosomal+subunit	0.655633	0.310533	0.970412
BP_GO:0000050_urea+cycle	0.214096	0.00996908	0.972597
BP_GO:0005978_glycogen+biosynthetic+process	0.767248	0.790264	0.576549
BP_GO:0016310_phosphorylation	0.594346	0.256239	0.910057
MF_GO:0048407_platelet-derived+growth+factor+binding	0.0217714	0.024866	0.201983
BP_GO:0001916_positive+regulation+of+T+cell+mediated+cytotoxicity	0.569534	0.631115	0.39966
BP_GO:0050730_regulation+of+peptidyl-tyrosine+phosphorylation	0.253189	0.138258	0.587096
BP_GO:0000910_cytokinesis	0.270059	0.555111	0.104312
MF_GO:0016811_hydrolase+activity,+acting+on+carbon-nitrogen+(but+not+	0.234167	0.49456	0.168367
BP_GO:0042135_neurotransmitter+catabolic+process	0.131318	0.339332	0.111733
BP_GO:0006487_protein+amino+acid+N-linked+glycosylation	0.926372	0.724885	0.952281
BP_GO:0060048_cardiac+muscle+contraction	0.0497765	0.39531	0.0148372
MF_GO:0004017_adenylate+kinase+activity	0.0802393	0.150996	0.165614
MF_GO:0019205_nucleobase,+nucleoside,+nucleotide+kinase+activity	0.105661	0.150996	0.240238
MF_GO:0017127_cholesterol+transporter+activity	0.369897	0.448688	0.361786
BP_GO:0045766_positive+regulation+of+angiogenesis	0.162119	0.243949	0.213659
MF_GO:0016651_oxidoreductase+activity,+acting+on+NADH+or+NADPH	0.560059	0.393658	0.671691
BP_GO:0035137_hindlimb+morphogenesis	0.964674	0.976439	0.548603
MF_GO:0005542_folic+acid+binding	0.632017	0.614944	0.57136
BP_GO:0046622_positive+regulation+of+organ+growth	0.325992	0.495337	0.276127
MF_GO:0016538_cyclin-dependent+protein+kinase+regulator+activity	0.734396	0.403938	0.923636
BP_GO:0030512_negative+regulation+of+transforming+growth+factor+beta	0.602282	0.66514	0.334166
MF_GO:0003854_3-beta-hydroxy-delta5-steroid+dehydrogenase+activity	0.320426	0.376575	0.344178
BP_GO:0008632_apoptotic+program	0.399624	0.251509	0.694056
BP_GO:0043407_negative+regulation+of+MAP+kinase+activity	0.0671584	0.0238993	0.700898
BP_GO:0046641_positive+regulation+of+alpha-beta+T+cell+proliferation	0.207522	0.446083	0.142065
BP_GO:0045582_positive+regulation+of+T+cell+differentiation	0.469124	0.331991	0.644324
BP_GO:0035162_embryonic+hemopoiesis	0.915277	0.92474	0.636077
MF_GO:0005391_sodium:potassium-exchanging+ATPase+activity	0.552024	0.452478	0.692136
BP_GO:0006270_DNA+replication+initiation	0.807276	0.399354	0.918252
MF_GO:0046332_SMAD+binding	0.242453	0.345526	0.273169
MF_GO:0008191_metalloendopeptidase+inhibitor+activity	0.89063	0.948093	0.577885
CC_GO:0005759_mitochondrial+matrix	0.608881	0.847186	0.166087
MF_GO:0019841_retinol+binding	0.6856	0.506544	0.737166
BP_GO:0006878_cellular+copper+ion+homeostasis	0.722326	0.90695	0.276649
MF_GO:0008253_5'-nucleotidase+activity	0.334643	0.159705	0.62462
BP_GO:0006548_histidine+catabolic+process	0.180243	0.251099	0.246784
BP_GO:0045060_negative+thymic+T+cell+selection	0.638284	0.302522	0.825861
BP_GO:0006800_oxygen+and+reactive+oxygen+species+metabolic+process	0.889799	0.954599	0.552833
CC_GO:0005771_multivesicular+body	0.998845	0.907735	0.997144
BP_GO:0009395_phospholipid+catabolic+process	0.380686	0.538079	0.2543
MF_GO:0004702_receptor+signaling+protein+serine/threonine+kinase+activ	0.964477	0.990627	0.625983
BP_GO:0045453_bone+resorption	0.0921762	0.271818	0.0463848
BP_GO:0030889_negative+regulation+of+B+cell+proliferation	0.0799691	0.301574	0.0446024
BP_GO:0006744_ubiquinone+biosynthetic+process	0.139184	0.123417	0.37019

MF_GO:0016303_1-phosphatidylinositol-3-kinase+activity	0.416292	0.576995	0.260897
BP_GO:0019752_carboxylic+acid+metabolic+process	0.395448	0.511214	0.308976
MF_GO:0008375_acetylglucosaminyltransferase+activity	0.869937	0.340985	0.994333
BP_GO:0046854_phosphoinositide+phosphorylation	0.461312	0.533129	0.358957
BP_GO:0051607_defense+response+to+virus	0.844952	0.714565	0.863188
MF_GO:0008195_phosphatidate+phosphatase+activity	0.917549	0.769692	0.896122
CC_GO:0035253_ciliary+rootlet	0.37978	0.420208	0.403231
BP_GO:0021954_central+nervous+system+neuron+development	0.691471	0.879803	0.352599
MF_GO:0015198_oligopeptide+transporter+activity	0.982636	0.987366	0.503107
BP_GO:0017156_calcium+ion-dependent+exocytosis	0.313889	0.455592	0.247626
BP_GO:0021952_central+nervous+system+projection+neuron+axonogenesis	0.579253	0.395089	0.693868
CC_GO:0008180_signalosome	0.253108	0.253108	#N/A
MF_GO:0008276_protein+methyltransferase+activity	0.243539	0.845843	0.0326395
MF_GO:0015485_cholesterol+binding	0.45742	0.568778	0.361786
CC_GO:0005776_autophagic+vacuole	0.628764	0.796245	0.196975
BP_GO:0006376_mRNA+splice+site+selection	0.685701	0.953905	0.0613473
BP_GO:0042542_response+to+hydrogen+peroxide	0.134738	0.490301	0.0721622
BP_GO:0008629_induction+of+apoptosis+by+intracellular+signals	0.538664	0.709728	0.318576
BP_GO:0016051_carbohydrate+biosynthetic+process	0.968515	0.974315	0.774475
MF_GO:0003841_1-acylglycerol-3-phosphate+O-acyltransferase+activity	0.458114	0.897739	0.101092
BP_GO:0000186_activation+of+MAPKK+activity	0.596806	0.768281	0.413893
BP_GO:0007032_endosome+organization	0.119346	0.149188	0.272607
BP_GO:0000188_inactivation+of+MAPK+activity	0.477696	0.369255	0.693896
MF_GO:0030234_enzyme+regulator+activity	0.628673	0.855168	0.0994481
CC_GO:0042470_melanosome	0.506914	0.333527	0.800367
BP_GO:0030325_adrenal+gland+development	0.0335589	0.0226305	0.567896
BP_GO:0051209_release+of+sequestered+calcium+ion+into+cytosol	0.979825	0.91375	0.939163
BP_GO:0050673_epithelial+cell+proliferation	0.44987	0.832145	0.102934
CC_GO:0005641_nuclear+envelope+lumen	0.779434	0.714665	0.717193
BP_GO:0031295_T+cell+costimulation	0.613158	0.411148	0.792974
BP_GO:0030316_osteoclast+differentiation	0.130893	0.573545	0.0473151
BP_GO:0033198_response+to+ATP	0.66215	0.835654	0.0656662
BP_GO:0001967_suckling+behavior	0.193255	0.669974	0.0599587
BP_GO:0045671_negative+regulation+of+osteoclast+differentiation	0.334281	0.39369	0.343322
BP_GO:0006306_DNA+methylation	0.538035	0.928992	0.118112
BP_GO:0045668_negative+regulation+of+osteoblast+differentiation	0.284819	0.395686	0.238423
CC_GO:0033178_proton-transporting+two-sector+ATPase+complex,+catalyt	0.650766	0.575654	0.656202
BP_GO:0043525_positive+regulation+of+neuron+apoptosis	0.670255	0.585937	0.676217
BP_GO:0048148_behavioral+response+to+cocaine	0.415533	0.497207	0.377597
BP_GO:0048872_homeostasis+of+number+of+cells	0.441583	0.496385	0.404662
BP_GO:0031047_gene+silencing+by+RNA	0.354374	0.327801	0.516969
MF_GO:0004003_ATP-dependent+DNA+helicase+activity	0.988055	0.976327	0.860811
BP_GO:0043616_keratinocyte+proliferation	0.937551	0.805609	0.950061
MF_GO:0016780_phosphotransferase+activity,+for+other+substituted+phos	0.158678	0.256851	0.224378
CC_GO:0000922_spindle+pole	0.823999	0.310936	0.9896
BP_GO:0050804_regulation+of+synaptic+transmission	0.991446	0.996861	0.605157
MF_GO:0019825_oxygen+binding	0.7907	0.596202	0.95916

BP_GO:0002087_regulation+of+respiratory+gaseous+exchange+by+neurolo{	0.703696	0.614384	0.683827
MF_GO:0003796_lysozyme+activity	0.247399	0.292636	0.339494
BP_GO:0014032_neural+crest+cell+development	0.783805	0.285273	0.8837
BP_GO:0016601_Rac+protein+signal+transduction	0.373082	0.357499	0.524892
BP_GO:0000077_DNA+damage+checkpoint	0.628167	0.665936	0.504753
BP_GO:0001508_regulation+of+action+potential	0.690633	0.828085	0.316977
CC_GO:0043231_intracellular+membrane-bounded+organelle	0.728297	0.458065	0.931645
MF_GO:0015299_solute:hydrogen+antiporter+activity	0.558952	0.609042	0.465569
CC_GO:0043198_dendritic+shaft	0.406386	0.91386	0.0299747
CC_GO:0031461_cullin-RING+ubiquitin+ligase+complex	0.99324	0.994917	0.797643
CC_GO:0030126_COPI+vesicle+coat	0.990026	0.971116	0.947239
BP_GO:0048146_positive+regulation+of+fibroblast+proliferation	0.998827	0.99996	0.71181
MF_GO:0030553_cGMP+binding	0.217519	0.486247	0.128729
CC_GO:0005680_anaphase-promoting+complex	0.99514	0.999695	0.356863
CC_GO:0016272_prefoldin+complex	0.457639	0.490928	0.446825
MF_GO:0004383_guanylate+cyclase+activity	0.124916	0.205305	0.209996
CC_GO:0005844_polysome	0.310475	0.32178	0.39038
BP_GO:0021915_neural+tube+development	0.331527	0.24873	0.635278
MF_GO:0005452_inorganic+anion+exchanger+activity	0.240052	0.473348	0.187414
BP_GO:0007586_digestion	0.0258264	0.0971268	0.0734622
BP_GO:0006959_humoral+immune+response	0.193944	0.371895	0.176223
BP_GO:0050877_neurological+system+process	0.807701	0.807745	0.586531
BP_GO:0016486_peptide+hormone+processing	0.724661	0.859923	0.225333
BP_GO:0016339_calcium-dependent+cell-cell+adhesion	0.424802	0.474947	0.388079
BP_GO:0050910_detection+of+mechanical+stimulus+involved+in+sensory+p	0.178901	0.693439	0.0689745
BP_GO:0000724_double-strand+break+repair+via+homologous+recombinat	0.946521	0.170209	0.990298
MF_GO:0016494_C-X-C+chemokine+receptor+activity	0.958575	0.994311	0.28298
MF_GO:0009881_photoreceptor+activity	0.863253	0.888605	0.568963
BP_GO:0006228_UTP+biosynthetic+process	0.80977	0.598632	0.815488
BP_GO:0006241_CTP+biosynthetic+process	0.80977	0.598632	0.815488
MF_GO:0004550_nucleoside+diphosphate+kinase+activity	0.80977	0.598632	0.815488
MF_GO:0004526_ribonuclease+P+activity	0.260669	0.264463	0.348036
MF_GO:0008417_fucosyltransferase+activity	0.851013	0.864616	0.597319
CC_GO:0016585_chromatin+remodeling+complex	0.821091	0.93675	0.447533
BP_GO:0002076_osteoblast+development	0.481134	0.621398	0.248682
BP_GO:0001542_ovulation+from+ovarian+follicle	0.466816	0.528413	0.403386
BP_GO:0006476_protein+amino+acid+deacetylation	0.914048	0.859904	0.810441
BP_GO:0007141_male+meiosis+I	0.686039	0.893513	0.321746
CC_GO:0005832_chaperonin-containing+T-complex	0.0420383	0.177459	0.0718641
CC_GO:0042788_polysomal+ribosome	0.826909	0.826909	#N/A
CC_GO:0031674_I+band	0.81328	0.68831	0.778973
CC_GO:0042627_chylomicron	0.00365385	4.01209E-05	0.928725
MF_GO:0004303_estradiol+17-beta-dehydrogenase+activity	0.165833	0.740138	0.0218611
CC_GO:0043292_contractile+fiber	0.2237	0.389532	0.21363
CC_GO:0005901_caveola	0.752714	0.940875	0.183874
MF_GO:0015020_glucuronosyltransferase+activity	0.5272	0.277159	0.707003
MF_GO:0016298_lipase+activity	0.868983	0.950243	0.384396

MF_GO:0005080_protein+kinase+C+binding	0.7471	0.727165	0.611997
BP_GO:0045059_positive+thymic+T+cell+selection	0.649397	0.38277	0.90071
BP_GO:0009435_NAD+biosynthetic+process	0.542418	0.404242	0.736962
BP_GO:0042572_retinol+metabolic+process	0.493888	0.613717	0.404103
BP_GO:0050798_activated+T+cell+proliferation	0.990489	0.998333	0.649293
BP_GO:0043409_negative+regulation+of+MAPKKK+cascade	0.762812	0.86495	0.316028
BP_GO:0045576_mast+cell+activation	0.198505	0.0970157	0.615554
CC_GO:0030864_cortical+actin+cytoskeleton	0.10847	0.0995474	0.359941
BP_GO:0030031_cell+projection+assembly	0.30576	0.176649	0.583212
BP_GO:0045579_positive+regulation+of+B+cell+differentiation	0.712289	0.714605	0.555368
MF_GO:0043621_protein+self-association	0.539406	0.551052	0.476477
BP_GO:0009165_nucleotide+biosynthetic+process	0.321308	0.248527	0.59826
CC_GO:0005765_lysosomal+membrane	0.162116	0.0333375	0.669892
BP_GO:0000060_protein+import+into+nucleus,+translocation	0.232943	0.297014	0.307622
BP_GO:0050796_regulation+of+insulin+secretion	0.723951	0.484569	0.810706
MF_GO:0004745_retinol+dehydrogenase+activity	0.173963	0.723314	0.0498667
MF_GO:0008121_ubiquinol-cytochrome-c+reductase+activity	0.839283	0.699638	0.810571
BP_GO:0045086_positive+regulation+of+interleukin-2+biosynthetic+process	0.461322	0.557691	0.388976
BP_GO:0002504_antigen+processing+and+presentation+of+peptide+or+poly	0.908179	0.685786	0.895582
CC_GO:0042613_MHC+class+II+protein+complex	0.908179	0.685786	0.895582
BP_GO:0043011_myeloid+dendritic+cell+differentiation	0.588801	0.249432	0.790491
BP_GO:0018345_protein+palmitoylation	0.553176	0.469323	0.578939
MF_GO:0016409_palmitoyltransferase+activity	0.553176	0.469323	0.578939
BP_GO:0042104_positive+regulation+of+activated+T+cell+proliferation	0.273278	0.370382	0.203129
CC_GO:0046658_anchored+to+plasma+membrane	0.478743	0.212342	0.709241
MF_GO:0005021_vascular+endothelial+growth+factor+receptor+activity	0.409157	0.374264	0.579008
CC_GO:0005952_cAMP-dependent+protein+kinase+complex	0.629239	0.629239	#N/A
BP_GO:0007173_epidermal+growth+factor+receptor+signaling+pathway	0.0460164	0.00434582	0.208833
MF_GO:0030675_Rac+GTPase+activator+activity	0.635281	0.821094	0.072745
BP_GO:0045765_regulation+of+angiogenesis	0.130847	0.222358	0.124331
MF_GO:0051018_protein+kinase+A+binding	0.06142	0.100643	0.162543
CC_GO:0005881_cytoplasmic+microtubule	0.715878	0.575989	0.731132
BP_GO:0006835_dicarboxylic+acid+transport	0.857837	0.899693	0.538751
BP_GO:0030500_regulation+of+bone+mineralization	0.189609	0.272531	0.238641
BP_GO:0001933_negative+regulation+of+protein+amino+acid+phosphorylat	0.137112	0.0468564	0.551585
BP_GO:0050870_positive+regulation+of+T+cell+activation	0.527921	0.234456	0.742119
MF_GO:0017017_MAP+kinase+tyrosine/serine/threonine+phosphatase+acti	0.570142	0.309016	0.888525
CC_GO:0043296_apical+junction+complex	0.0748672	0.0936651	0.27888
BP_GO:0001952_regulation+of+cell-matrix+adhesion	0.71469	0.76481	0.454124
MF_GO:0050750_low-density+lipoprotein+receptor+binding	0.27137	0.253734	0.398787
BP_GO:0001678_cellular+glucose+homeostasis	0.697168	0.856057	0.0881154
MF_GO:0008199_ferric+iron+binding	0.369819	0.171713	0.683579
BP_GO:0006996_organelle+organization	0.355542	0.697897	0.101315
MF_GO:0016307_phosphatidylinositol+phosphate+kinase+activity	0.417433	0.548473	0.265071
BP_GO:0006940_regulation+of+smooth+muscle+contraction	0.447603	0.613489	0.278556
BP_GO:0008637_apoptotic+mitochondrial+changes	0.775735	0.536089	0.869063
BP_GO:0016079_synaptic+vesicle+exocytosis	0.34627	0.895229	0.0705741

BP_GO:0009267_cellular+response+to+starvation	0.766843	0.901415	0.0881154
BP_GO:0050884_neuromuscular+process+controlling+posture	0.525209	0.494064	0.586761
MF_GO:0042277_peptide+binding	0.338316	0.589637	0.165135
BP_GO:0007188_G-protein+signaling,+coupled+to+cAMP+nucleotide+secon	0.375329	0.489009	0.351864
BP_GO:0006779_porphyrin+biosynthetic+process	0.636273	0.541495	0.697662
BP_GO:0006939_smooth+muscle+contraction	0.017251	0.000919704	0.715242
BP_GO:0042100_B+cell+proliferation	0.316007	0.629393	0.113439
MF_GO:0016918_retinal+binding	0.847338	0.781911	0.768835
BP_GO:0044262_cellular+carbohydrate+metabolic+process	0.00136927	0.00319505	0.103928
BP_GO:0021702_cerebellar+Purkinje+cell+differentiation	0.853546	0.835105	0.654661
BP_GO:0042554_superoxide+release	0.781365	0.943204	0.117646
MF_GO:0015377_cation:chloride+symporter+activity	0.881327	0.805683	0.856258
MF_GO:0008536_Ran+GTPase+binding	0.554839	0.663057	0.325374
CC_GO:0016581_NuRD+complex	0.441307	0.54838	0.345426
BP_GO:0051605_protein+maturation+via+proteolysis	0.764606	0.718568	0.667066
BP_GO:0060216_definitive+hemopoiesis	0.884123	0.786009	0.901102
BP_GO:0000302_response+to+reactive+oxygen+species	0.163083	0.0592753	0.407855
BP_GO:0001841_neural+tube+formation	0.767582	0.850781	0.44121
CC_GO:0031966_mitochondrial+membrane	0.077626	0.291783	0.0848227
BP_GO:0050770_regulation+of+axonogenesis	0.266934	0.317038	0.337367
MF_GO:0048306_calcium-dependent+protein+binding	0.0774486	0.129121	0.18903
BP_GO:0035136_forelimb+morphogenesis	0.588968	0.626638	0.456618
BP_GO:0019800_peptide+cross-linking+via+chondroitin+4-sulfate+glycosam	0.300894	0.205047	0.649437
MF_GO:0008889_glycerophosphodiester+phosphodiesterase+activity	0.593947	0.57279	0.567121
BP_GO:0046907_intracellular+transport	0.877064	0.798518	0.808936
BP_GO:0007193_G-protein+signaling,+adenylate+cyclase+inhibiting+pathwa	0.454061	0.482971	0.456885
BP_GO:0007625_grooming+behavior	0.844076	0.940335	0.360041
BP_GO:0007130_synaptonemal+complex+assembly	0.50669	0.46743	0.542197
MF_GO:0019894_kinesin+binding	0.65378	0.726893	0.399942
BP_GO:0021953_central+nervous+system+neuron+differentiation	0.108729	0.139403	0.254165
BP_GO:0006626_protein+targeting+to+mitochondrion	0.565381	0.814616	0.314081
BP_GO:0009755_hormone-mediated+signaling	0.963549	0.93305	0.839579
CC_GO:0005815_microtubule+organizing+center	0.454523	0.21575	0.734181
CC_GO:0031519_PcG+protein+complex	0.391211	0.710658	0.172195
CC_GO:0009925_basal+plasma+membrane	0.973856	0.998336	0.26779
BP_GO:0021537_telencephalon+development	0.20311	0.117755	0.653277
BP_GO:0007191_dopamine+receptor,+adenylate+cyclase+activating+pathwa	0.895674	0.918245	0.600868
BP_GO:0042325_regulation+of+phosphorylation	0.396374	0.426419	0.426327
BP_GO:0001659_temperature+homeostasis	0.607146	0.600902	0.545643
CC_GO:0005801_cis-Golgi+network	0.291695	0.574137	0.127773
BP_GO:0032729_positive+regulation+of+interferon-gamma+production	0.393145	0.602804	0.160154
BP_GO:0006983_ER+overload+response	0.806583	0.808603	0.662607
BP_GO:0008340_determination+of+adult+life+span	0.403266	0.334817	0.598669
BP_GO:0030850_prostate+gland+development	0.653538	0.383933	0.803556
BP_GO:0002027_regulation+of+heart+rate	0.927561	0.801648	0.901174
BP_GO:0060113_inner+ear+receptor+cell+differentiation	0.267231	0.0821709	0.695749
MF_GO:0047555_3',5'-cyclic-GMP+phosphodiesterase+activity	0.709816	0.509454	0.808762

BP_GO:0021904_dorsoventral+neural+tube+patterning	0.243914	0.535267	0.142404
MF_GO:0004945_angiotensin+type+II+receptor+activity	0.396922	0.505945	0.327915
BP_GO:0008361_regulation+of+cell+size	0.599446	0.76307	0.11838
MF_GO:0005246_calcium+channel+regulator+activity	0.748961	0.544297	0.829879
BP_GO:0045176_apical+protein+localization	0.731533	0.731533	#N/A
CC_GO:0005763_mitochondrial+small+ribosomal+subunit	0.553857	0.278727	0.643
BP_GO:0001655_urogenital+system+development	0.574559	0.52549	0.589084
MF_GO:0008601_protein+phosphatase+type+2A+regulator+activity	0.984682	0.994575	0.265941
MF_GO:0042393_histone+binding	0.481041	0.682529	0.0820094
BP_GO:0030432_peristalsis	0.928478	0.972228	0.349688
BP_GO:0009617_response+to+bacterium	0.883165	0.919652	0.366112
MF_GO:0008094_DNA-dependent+ATPase+activity	0.298021	0.210317	0.522258
BP_GO:0007212_dopamine+receptor+signaling+pathway	0.362162	0.319596	0.51186
CC_GO:0000228_nuclear+chromosome	0.699388	0.433075	0.97304
BP_GO:0045471_response+to+ethanol	0.425809	0.451644	0.443483
BP_GO:0007129_synapsis	0.706454	0.535032	0.752042
BP_GO:0048168_regulation+of+neuronal+synaptic+plasticity	0.00431452	0.0618794	0.0129836
MF_GO:0008158_hedgehog+receptor+activity	0.95537	0.877713	0.89877
CC_GO:0000790_nuclear+chromatin	0.681361	0.47268	0.856176
BP_GO:0042220_response+to+cocaine	0.908394	0.902301	0.663005
CC_GO:0005677_chromatin+silencing+complex	0.766311	0.630159	0.810441
CC_GO:0043209_myelin+sheath	0.253118	0.798595	0.0378199
BP_GO:0048240_sperm+capacitation	0.70928	0.769312	0.517513
BP_GO:0006032_chitin+catabolic+process	0.0352478	0.0618985	0.153979
MF_GO:0004568_chitinase+activity	0.0352478	0.0618985	0.153979
BP_GO:0006611_protein+export+from+nucleus	0.928199	0.948215	0.670739
BP_GO:0042074_cell+migration+involved+in+gastrulation	0.934098	0.892528	0.805554
BP_GO:0007435_salivary+gland+morphogenesis	0.8223	0.924837	0.260278
BP_GO:0042438_melanin+biosynthetic+process	0.880154	0.985613	0.183534
BP_GO:0016579_protein+deubiquitination	0.8095	0.802853	0.609422
BP_GO:0015991_ATP+hydrolysis+coupled+proton+transport	0.840524	0.835906	0.642952
BP_GO:0016446_somatic+hypermutation+of+immunoglobulin+genes	0.0450959	0.646836	0.00277213
MF_GO:0050699_WW+domain+binding	0.0670638	0.348105	0.0101339
BP_GO:0001539_ciliary+or+flagellar+motility	0.569534	0.953983	0.0754468
MF_GO:0004522_pancreatic+ribonuclease+activity	0.347413	0.696606	0.185535
BP_GO:0007140_male+meiosis	0.334509	0.48443	0.258638
BP_GO:0045109_intermediate+filament+organization	0.728636	0.719599	0.596401
BP_GO:0007131_reciprocal+meiotic+recombination	0.669835	0.924414	0.128178
MF_GO:0005248_voltage-gated+sodium+channel+activity	0.517069	0.0363033	0.968299
CC_GO:0022625_cytosolic+large+ribosomal+subunit	0.895325	0.316997	0.987863
CC_GO:0005885_Arp2/3+protein+complex	0.00495679	0.000288599	0.731659
MF_GO:0016810_hydrolase+activity,+acting+on+carbon-nitrogen+(but+not+	0.0701085	0.0915896	0.260264
BP_GO:0042311_vasodilation	0.778526	0.801359	0.539007
BP_GO:0006910_phagocytosis,+recognition	0.565425	0.478272	0.623719
BP_GO:0051259_protein+oligomerization	0.928203	0.766934	0.943631
BP_GO:0042692_muscle+cell+differentiation	0.67332	0.918814	0.269298
CC_GO:0046581_intercellular+canaliculus	0.867764	0.856848	0.633802

MF_GO:0004029_aldehyde+dehydrogenase+(NAD)+activity	0.921205	0.838179	0.907249
BP_GO:0006013_mannose+metabolic+process	0.992163	0.991927	0.763964
BP_GO:0008206_bile+acid+metabolic+process	0.639183	0.89421	0.0953206
MF_GO:0008508_bile+acid:sodium+symporter+activity	0.347852	0.356151	0.421592
BP_GO:0006656_phosphatidylcholine+biosynthetic+process	0.820466	0.85131	0.527586
BP_GO:0007520_myoblast+fusion	0.960107	0.991348	0.313902
MF_GO:0004365_glyceraldehyde-3-phosphate+dehydrogenase+(phosphoryl	0.969234	0.992189	0.16471
MF_GO:0008943_glyceraldehyde-3-phosphate+dehydrogenase+activity	0.969234	0.992189	0.16471
BP_GO:0045785_positive+regulation+of+cell+adhesion	0.521098	0.23884	0.753419
BP_GO:0030866_cortical+actin+cytoskeleton+organization	0.218636	0.15198	0.67861
BP_GO:0007044_cell-substrate+junction+assembly	0.172062	0.355092	0.118444
BP_GO:0010259_multicellular+organismal+aging	0.187626	0.331343	0.166163
BP_GO:0008277_regulation+of+G-protein+coupled+receptor+protein+signal	0.19024	0.211284	0.339572
BP_GO:0009086_methionine+biosynthetic+process	0.19332	0.129777	0.564893
BP_GO:0046034_ATP+metabolic+process	0.972827	0.872235	0.989539
BP_GO:0030148_sphingolipid+biosynthetic+process	0.356761	0.104746	0.81295
MF_GO:0005100_Rho+GTPase+activator+activity	0.00716349	0.0201051	0.072745
MF_GO:0004630_phospholipase+D+activity	0.761648	0.824537	0.443246
BP_GO:0006108_malate+metabolic+process	0.00339258	0.00173339	0.498897
BP_GO:0043353_enucleate+erythrocyte+differentiation	0.603345	0.551347	0.647395
BP_GO:0006044_N-acetylglucosamine+metabolic+process	0.121579	0.276997	0.105943
BP_GO:0006805_xenobiotic+metabolic+process	0.853562	0.90438	0.461114
BP_GO:0030225_macrophage+differentiation	0.656401	0.334333	0.772604
BP_GO:0006672_ceramide+metabolic+process	0.141821	0.444151	0.0973532
BP_GO:0042310_vasoconstriction	0.928907	0.928191	0.838765
BP_GO:0007250_activation+of+NF-kappaB-inducing+kinase+activity	0.481358	0.3832	0.649183
BP_GO:0006729_tetrahydrobiopterin+biosynthetic+process	0.276511	0.254474	0.415778
BP_GO:0050772_positive+regulation+of+axonogenesis	0.734632	0.853224	0.312991
BP_GO:0016575_histone+deacetylation	0.236319	0.718266	0.0388401
MF_GO:0051920_peroxiredoxin+activity	0.0585357	#N/A	0.0585357
BP_GO:0007270_nerve-nerve+synaptic+transmission	0.863087	0.887241	0.471059
BP_GO:0019222_regulation+of+metabolic+process	0.93112	0.915992	0.725239
MF_GO:0005545_phosphatidylinositol+binding	0.161713	0.539626	0.0522378
MF_GO:0017153_sodium:dicarboxylate+symporter+activity	0.853248	0.904288	0.538751
BP_GO:0006750_glutathione+biosynthetic+process	0.413202	0.573922	0.291199
BP_GO:0002011_morphogenesis+of+an+epithelial+sheet	0.819099	0.875664	0.453441
BP_GO:0006546_glycine+catabolic+process	0.600927	0.488072	0.664932
MF_GO:0045296_cadherin+binding	0.666931	0.70011	0.491349
MF_GO:0005501_retinoid+binding	0.662792	0.831184	0.456797
BP_GO:0030838_positive+regulation+of+actin+filament+polymerization	0.0434467	0.0561144	0.261198
BP_GO:0045843_negative+regulation+of+striated+muscle+development	0.83206	0.952428	0.201068
MF_GO:0003956_NAD(P)+-protein-arginine+ADP-ribosyltransferase+activity	0.0452846	0.0293977	0.42918
CC_GO:0045261_proton-transporting+ATP+synthase+complex,+catalytic+co	0.921498	0.870903	0.805548
BP_GO:0048147_negative+regulation+of+fibroblast+proliferation	0.816851	0.908555	0.667523
BP_GO:0017157_regulation+of+exocytosis	0.390413	0.447853	0.392307
BP_GO:0046328_regulation+of+JNK+cascade	0.233874	0.162031	0.689852
BP_GO:0032755_positive+regulation+of+interleukin-6+production	0.920136	0.955048	0.480089

BP_GO:0021756_striatum+development	0.196297	0.118147	0.45801
BP_GO:0048854_brain+morphogenesis	0.0716473	0.091387	0.21685
BP_GO:0006584_catecholamine+metabolic+process	0.621741	0.749567	0.380953
MF_GO:0031177_phosphopantetheine+binding	0.146315	0.0608983	0.683721
MF_GO:0008528_peptide+receptor+activity,+G-protein+coupled	0.335305	0.501873	0.256265
BP_GO:0001963_synaptic+transmission,+dopaminergic	0.19474	0.550146	0.0721099
MF_GO:0017075_syntaxin-1+binding	0.545535	0.91809	0.075978
BP_GO:0002064_epithelial+cell+development	0.518313	0.716493	0.27746
BP_GO:0007413_axonal+fasciculation	0.280239	0.211893	0.469187
BP_GO:0006098_pentose-phosphate+shunt	0.620138	0.614644	0.538318
BP_GO:0007271_synaptic+transmission,+cholinergic	0.587408	0.559975	0.564393
MF_GO:0004931_ATP-gated+cation+channel+activity	0.738701	0.934449	0.0223172
MF_GO:0015385_sodium:hydrogen+antiporter+activity	0.538975	0.720378	0.300025
BP_GO:0048070_regulation+of+pigmentation+during+development	0.242392	0.187361	0.483235
BP_GO:0045410_positive+regulation+of+interleukin-6+biosynthetic+process	0.682838	0.653251	0.605529
BP_GO:0031032_actomyosin+structure+organization	0.5402	0.627364	0.412784
BP_GO:0045084_positive+regulation+of+interleukin-12+biosynthetic+proces	0.505774	0.527434	0.448391
BP_GO:0009416_response+to+light+stimulus	0.714661	0.900457	0.166198
MF_GO:0004622_lysophospholipase+activity	0.562389	0.733066	0.245223
BP_GO:0060088_auditory+receptor+cell+stereocilium+organization	0.0368045	0.356097	0.0105081
BP_GO:0008625_induction+of+apoptosis+via+death+domain+receptors	0.162781	0.151097	0.4177
BP_GO:0002063_chondrocyte+development	0.239258	0.725042	0.0728258
BP_GO:0043281_regulation+of+caspase+activity	0.465033	0.27819	0.707388
BP_GO:0009314_response+to+radiation	0.0728455	0.814141	0.0111248
BP_GO:0042551_neuron+maturation	0.980305	0.978782	0.833895
CC_GO:0000780_condensed+nuclear+chromosome,+centromeric+region	0.841963	0.72049	0.829795
BP_GO:0030279_negative+regulation+of+ossification	0.328444	0.553243	0.270954
MF_GO:0008440_inositol+trisphosphate+3-kinase+activity	0.190366	0.375323	0.127597
BP_GO:0035239_tube+morphogenesis	0.670489	0.804364	0.175469
BP_GO:0006817_phosphate+transport	0.57238	0.729537	0.265954
BP_GO:0002360_T+cell+lineage+commitment	0.546064	0.572358	0.48675
MF_GO:0005160_transforming+growth+factor+beta+receptor+binding	0.354243	0.295079	0.520399
BP_GO:0006924_activated+T+cell+apoptosis	0.777464	0.815439	0.592282
MF_GO:0015077_monovalent+inorganic+cation+transmembrane+transport	0.7159	0.593059	0.726048
MF_GO:0001609_adenosine+receptor+activity,+G-protein+coupled	0.242198	0.356565	0.272077
BP_GO:0045880_positive+regulation+of+smoothened+signaling+pathway	0.688147	0.883367	0.265568
BP_GO:0040011_locomotion	0.23195	0.382957	0.113239
MF_GO:0005041_low-density+lipoprotein+receptor+activity	0.973406	0.965879	0.742428
CC_GO:0016328_lateral+plasma+membrane	0.569607	0.532499	0.604607
BP_GO:0010033_response+to+organic+substance	0.989044	0.995071	0.398671
BP_GO:0043249_erythrocyte+maturation	0.49348	0.515392	0.472494
BP_GO:0014059_regulation+of+dopamine+secretion	0.668521	0.830547	0.464825
MF_GO:0003727_single-stranded+RNA+binding	0.979929	0.954866	0.894281
MF_GO:0003678_DNA+helicase+activity	0.715799	0.220901	0.877305
BP_GO:0006268_DNA+unwinding+during+replication	0.472576	0.143702	0.722875
BP_GO:0043491_protein+kinase+B+signaling+cascade	0.929931	0.881429	0.813962
BP_GO:0060080_regulation+of+inhibitory+postsynaptic+membrane+potenti	0.0737286	0.0922361	0.280224

BP_GO:0060037_pharyngeal+system+development	0.372515	0.460966	0.325195
MF_GO:0016165_lipoxygenase+activity	0.847612	0.821134	0.693872
BP_GO:0043488_regulation+of+mRNA+stability	0.719501	0.935847	0.192881
CC_GO:0000800_lateral+element	0.65697	0.608996	0.625392
MF_GO:0016500_protein-hormone+receptor+activity	0.373294	0.423692	0.397234
MF_GO:0000149_SNARE+binding	0.21222	0.748529	0.0230835
MF_GO:0004579_dolichyl-diphosphooligosaccharide-protein+glycotransfera	0.168794	0.768411	0.0909802
BP_GO:0006349_genetic+imprinting	0.60473	0.654222	0.47938
BP_GO:0008585_female+gonad+development	0.976359	0.930813	0.91473
MF_GO:0004767_sphingomyelin+phosphodiesterase+activity	0.507403	0.507403	#N/A
CC_GO:0015030_Cajal+body	0.296582	0.470494	0.279243
MF_GO:0015280_amiloride-sensitive+sodium+channel+activity	0.413376	0.685379	0.120398
BP_GO:0006303_double-strand+break+repair+via+nonhomologous+end+joi	0.870379	0.850588	0.680562
CC_GO:0001739_sex+chromatin	0.191137	0.13102	0.426763
BP_GO:0048304_positive+regulation+of+isotype+switching+to+IgG+isotypes	0.918112	0.9593	0.279623
CC_GO:0042105_alpha-beta+T+cell+receptor+complex	0.694095	0.798135	0.42538
MF_GO:0003906_DNA-(apurinic+or+apyrimidinic+site)+lyase+activity	0.390001	0.289123	0.508469
CC_GO:0000242_pericentriolar+material	0.276775	0.666699	0.123724
MF_GO:0017136_NAD-dependent+histone+deacetylase+activity	0.815266	0.693781	0.810441
BP_GO:0060134_prepulse+inhibition	0.802932	0.765432	0.673965
BP_GO:0007616_long-term+memory	0.86561	0.964188	0.218019
MF_GO:0008188_neuropeptide+receptor+activity	0.09878	0.283241	0.122573
MF_GO:0003711_transcription+elongation+regulator+activity	0.726051	0.503534	0.818255
BP_GO:0009948_anterior/posterior+axis+specification	0.456043	0.337887	0.585702
MF_GO:0050733_RS+domain+binding	0.194446	0.806934	0.0585439
CC_GO:0032391_photoreceptor+connecting+cilium	0.0217044	0.0199563	0.377858
BP_GO:0019229_regulation+of+vasoconstriction	0.577329	0.339002	0.781772
CC_GO:0031526_brush+border+membrane	0.709411	0.741806	0.566962
MF_GO:0004000_adenosine+deaminase+activity	0.550127	0.601828	0.382676
BP_GO:0007342_fusion+of+sperm+to+egg+plasma+membrane	0.130409	0.442199	0.0866474
BP_GO:0042176_regulation+of+protein+catabolic+process	0.18869	0.309174	0.193799
BP_GO:0060136_embryonic+process+involved+in+female+pregnancy	0.978445	0.734733	0.983583
CC_GO:0005786_signal+recognition+particle,+endoplasmic+reticulum+targe	0.252606	#N/A	0.252606
BP_GO:0032259_methylation	0.340064	0.62202	0.16145
MF_GO:0016251_general+RNA+polymerase+II+transcription+factor+activity	0.349954	0.287569	0.490312
BP_GO:0032355_response+to+estradiol+stimulus	0.917962	0.880169	0.789118
BP_GO:0046620_regulation+of+organ+growth	0.578893	0.747603	0.247665
BP_GO:0045954_positive+regulation+of+natural+killer+cell+mediated+cytot	0.0593508	0.0381227	0.45389
BP_GO:0006559_L-phenylalanine+catabolic+process	0.0242245	0.0242245	#N/A
CC_GO:0005859_muscle+myosin+complex	0.36469	0.173976	0.894933
BP_GO:0042158_lipoprotein+biosynthetic+process	0.464621	0.464621	#N/A
CC_GO:0016012_sarcoglycan+complex	0.309882	0.553065	0.147276
MF_GO:0008035_high-density+lipoprotein+binding	0.756145	0.396004	0.98907
BP_GO:0055003_cardiac+myofibril+assembly	0.818366	0.472831	0.912289
BP_GO:0006956_complement+activation	0.991306	0.993775	0.59453
BP_GO:0042574_retinal+metabolic+process	0.159751	0.159751	#N/A
MF_GO:0016868_intramolecular+transferase+activity,+phosphotransferases	0.514069	0.345422	0.672969

MF_GO:0005523_tropomyosin+binding	0.231328	0.367845	0.241621
BP_GO:0042953_lipoprotein+transport	0.0239936	0.0239936	#N/A
BP_GO:0002237_response+to+molecule+of+bacterial+origin	0.385316	0.379215	0.489677
BP_GO:0006693_prostaglandin+metabolic+process	0.21292	0.497069	0.131606
BP_GO:0046513_ceramide+biosynthetic+process	0.488377	0.552813	0.35645
MF_GO:0043499_eukaryotic+cell+surface+binding	0.717208	0.913278	0.291921
MF_GO:0030151_molybdenum+ion+binding	0.811625	0.711086	0.771926
BP_GO:0002021_response+to+dietary+excess	0.506203	0.680725	0.32736
BP_GO:0043406_positive+regulation+of+MAP+kinase+activity	0.535418	0.738273	0.113239
BP_GO:0060347_heart+trabecula+formation	0.193679	0.126816	0.546756
BP_GO:0030041_actin+filament+polymerization	0.340205	0.43996	0.251019
BP_GO:0010765_positive+regulation+of+sodium+ion+transport	0.885643	0.772551	0.899695
BP_GO:0032868_response+to+insulin+stimulus	0.641379	0.733375	0.400047
MF_GO:0003756_protein+disulfide+isomerase+activity	0.0674398	0.158271	0.116971
MF_GO:0004115_3',5'-cyclic-AMP+phosphodiesterase+activity	0.0628017	0.0556465	0.424248
MF_GO:0017114_wide-spectrum+protease+inhibitor+activity	0.940769	0.946366	0.588728
BP_GO:0008015_blood+circulation	0.248711	0.344658	0.270965
BP_GO:0017015_regulation+of+transforming+growth+factor+beta+receptor	0.267259	0.737747	0.00160396
BP_GO:0051604_protein+maturation	0.252384	0.737436	0.102785
MF_GO:0008308_voltage-gated+anion+channel+activity	0.1777	0.0374578	0.820841
MF_GO:0016813_hydrolase+activity,+acting+on+carbon-nitrogen+(but+not+	0.317802	0.197425	0.649179
BP_GO:0048041_focal+adhesion+formation	0.521578	0.7456	0.0892257
BP_GO:0050860_negative+regulation+of+T+cell+receptor+signaling+pathwa	0.0439286	0.0713608	0.182973
BP_GO:0030856_regulation+of+epithelial+cell+differentiation	0.641715	0.560128	0.661223
BP_GO:0015813_L-glutamate+transport	0.352701	0.867856	0.0494033
MF_GO:0017154_semaphorin+receptor+activity	0.131341	0.111763	0.413461
MF_GO:0030246_carbohydrate+binding	0.561496	0.500987	0.585899
MF_GO:0048185_activin+binding	0.74895	0.534164	0.806317
BP_GO:0009312_oligosaccharide+biosynthetic+process	0.479521	0.760519	0.286758
BP_GO:0048009_insulin-like+growth+factor+receptor+signaling+pathway	0.348819	0.292545	0.498722
BP_GO:0045445_myoblast+differentiation	0.936528	0.987847	0.0971705
MF_GO:0016814_hydrolase+activity,+acting+on+carbon-nitrogen+(but+not+	0.119032	0.254298	0.0788217
BP_GO:0007588_excretion	0.886687	0.990917	0.606287
MF_GO:0042605_peptide+antigen+binding	0.413429	0.209217	0.691526
BP_GO:0006882_cellular+zinc+ion+homeostasis	0.113844	0.932513	4.24092E-05
CC_GO:0033017_sarcoplasmic+reticulum+membrane	0.998796	0.999219	0.783886
BP_GO:0050790_regulation+of+catalytic+activity	0.265212	0.818035	0.0363261
CC_GO:0030130_clathrin+coat+of+trans-Golgi+network+vesicle	0.302589	0.16524	0.521895
BP_GO:0030098_lymphocyte+differentiation	0.936464	0.915396	0.815384
MF_GO:0001784_phosphotyrosine+binding	0.344446	0.344446	#N/A
MF_GO:0004047_aminomethyltransferase+activity	0.615635	0.488072	0.709717
BP_GO:0046902_regulation+of+mitochondrial+membrane+permeability	0.106499	0.0299619	0.422496
MF_GO:0004571_mannosyl-oligosaccharide+1,2-alpha-mannosidase+activity	0.901077	0.821565	0.823557
CC_GO:0030496_midbody	0.0978808	0.0352642	0.624366
MF_GO:0005388_calcium-transporting+ATPase+activity	0.415176	0.269558	0.515933
BP_GO:0045885_positive+regulation+of+survival+gene+product+expression	0.662991	0.664484	0.567858
CC_GO:0000307_cyclin-dependent+protein+kinase+holoenzyme+complex	0.119868	0.0879699	0.451318

MF_GO:0004396_hexokinase+activity	0.356198	0.356198	#N/A
MF_GO:0005154_epidermal+growth+factor+receptor+binding	0.410756	0.711671	0.251259
BP_GO:0006122_mitochondrial+electron+transport,+ubiquinol+to+cytochro	0.879856	0.853378	0.708459
CC_GO:0005954_calcium-+and+calmodulin-dependent+protein+kinase+com	0.26248	0.521005	0.0468473
BP_GO:0045599_negative+regulation+of+fat+cell+differentiation	0.420606	0.830025	0.178924
BP_GO:0048048_embryonic+eye+morphogenesis	0.903347	0.816656	0.836009
BP_GO:0006790_sulfur+metabolic+process	0.309804	0.170512	0.598829
BP_GO:0045664_regulation+of+neuron+differentiation	0.788145	0.5709	0.787261
BP_GO:0016338_calcium-independent+cell-cell+adhesion	0.676413	0.47923	0.807195
BP_GO:0048103_somatic+stem+cell+division	0.220669	0.408257	0.157295
BP_GO:0006353_transcription+termination	0.97433	0.960385	0.814558
BP_GO:0006970_response+to+osmotic+stress	0.879403	0.422692	0.989499
BP_GO:0031647_regulation+of+protein+stability	0.581007	0.46359	0.648474
CC_GO:0044444_cytoplasmic+part	0.610515	0.526308	0.705832
BP_GO:0030517_negative+regulation+of+axon+extension	0.019977	0.373793	0.011049
BP_GO:0022904_respiratory+electron+transport+chain	0.776985	0.935007	0.205863
MF_GO:0016847_1-aminocyclopropane-1-carboxylate+synthase+activity	0.700461	0.758208	0.390442
BP_GO:0009396_folic+acid+and+derivative+biosynthetic+process	0.465168	0.208801	0.840359
MF_GO:0008331_high+voltage-gated+calcium+channel+activity	0.766199	0.799472	0.461112
BP_GO:0019953_sexual+reproduction	0.587807	0.565594	0.560225
BP_GO:0043388_positive+regulation+of+DNA+binding	0.807009	0.720861	0.792843
BP_GO:0006573_valine+metabolic+process	0.494655	0.45937	0.548439
MF_GO:0004540_ribonuclease+activity	0.52277	0.430558	0.576927
BP_GO:0006072_glycerol-3-phosphate+metabolic+process	0.0833406	0.13572	0.200325
BP_GO:0050930_induction+of+positive+chemotaxis	0.502323	0.613248	0.345871
BP_GO:0018149_peptide+cross-linking	0.66012	0.380081	0.764815
MF_GO:0003810_protein-glutamine+gamma-glutamyltransferase+activity	0.66012	0.380081	0.764815
BP_GO:0042743_hydrogen+peroxide+metabolic+process	0.617935	#N/A	0.617935
CC_GO:0005587_collagen+type+IV	0.723107	0.445503	0.835288
BP_GO:0008354_germ+cell+migration	0.62914	0.54631	0.626253
BP_GO:0048745_smooth+muscle+development	0.679849	0.555436	0.798017
BP_GO:0001578_microtubule+bundle+formation	0.286015	0.128328	0.734371
BP_GO:0035121_tail+morphogenesis	0.23895	0.414099	0.215766
CC_GO:0033179_proton-transporting+V-type+ATPase,+V0+domain	0.532278	0.493684	0.538287
BP_GO:0045732_positive+regulation+of+protein+catabolic+process	0.388876	0.741522	0.0799351
BP_GO:0051592_response+to+calcium+ion	0.948705	0.917092	0.816483
MF_GO:0016175_superoxide-generating+NADPH+oxidase+activity	0.943204	0.943204	#N/A
BP_GO:0051924_regulation+of+calcium+ion+transport	0.259615	0.391292	0.262567
MF_GO:0000062_acyl-CoA+binding	0.825972	0.632701	0.938588
BP_GO:0006461_protein+complex+assembly	0.769959	0.490029	0.857843
MF_GO:0008239_dipeptidyl-peptidase+activity	0.486106	0.384547	0.716465
MF_GO:0004128_cytochrome-b5+reductase+activity	0.0594439	0.0086093	0.569899
BP_GO:0043526_neuroprotection	0.945014	0.679424	0.982635
BP_GO:0045648_positive+regulation+of+erythrocyte+differentiation	0.893716	0.893716	#N/A
MF_GO:0004703_G-protein+coupled+receptor+kinase+activity	0.437456	0.437456	#N/A
MF_GO:0016407_acetyltransferase+activity	0.909559	0.689484	0.9852
BP_GO:0045995_regulation+of+embryonic+development	0.237757	0.143017	0.523058

MF_GO:0008046_axon+guidance+receptor+activity	0.218803	0.26649	0.317646
BP_GO:0043154_negative+regulation+of+caspase+activity	0.0984576	0.0713335	0.546792
BP_GO:0042415_norepinephrine+metabolic+process	0.66816	0.690258	0.547426
BP_GO:0007163_establishment+or+maintenance+of+cell+polarity	0.94516	0.961235	0.488958
BP_GO:0016925_protein+sumoylation	0.0303842	0.126908	0.0653364
MF_GO:0019789_SUMO+ligase+activity	0.0303842	0.126908	0.0653364
BP_GO:0000038_very-long-chain+fatty+acid+metabolic+process	0.00385912	0.00993321	0.0930581
MF_GO:0017022_myosin+binding	0.211967	0.104141	0.804099
MF_GO:0001948_glycoprotein+binding	0.47987	0.662097	0.312384
MF_GO:0004659_prenyltransferase+activity	0.146263	0.0709807	0.760069
BP_GO:0007406_negative+regulation+of+neuroblast+proliferation	0.994919	0.992939	0.791167
BP_GO:0042446_hormone+biosynthetic+process	0.08609	0.187687	0.148096
BP_GO:0002262_myeloid+cell+homeostasis	0.373892	0.524207	0.303218
BP_GO:0001776_leukocyte+homeostasis	0.287498	0.561281	0.171814
BP_GO:0030203_glycosaminoglycan+metabolic+process	0.9835	0.987397	0.586761
BP_GO:0045039_protein+import+into+mitochondrial+inner+membrane	0.417636	0.717296	0.314081
CC_GO:0042719_mitochondrial+intermembrane+space+protein+transporter	0.417636	0.717296	0.314081
BP_GO:0050829_defense+response+to+Gram-negative+bacterium	0.836873	0.836873	#N/A
MF_GO:0016308_1-phosphatidylinositol-4-phosphate+5-kinase+activity	0.548473	0.548473	#N/A
BP_GO:0006221_pyrimidine+nucleotide+biosynthetic+process	0.130764	0.627618	0.0542286
BP_GO:0007568_aging	0.540707	0.637778	0.417851
BP_GO:0060351_cartilage+development+involved+in+endochondral+bone+	0.603647	0.864884	0.419049
BP_GO:0007398_ectoderm+development	0.692087	0.60067	0.745448
MF_GO:0005112_Notch+binding	0.961841	0.895648	0.902187
BP_GO:0016188_synaptic+vesicle+maturation	0.501996	0.672827	0.329918
BP_GO:0045778_positive+regulation+of+ossification	0.530099	0.667117	0.31573
BP_GO:0006612_protein+targeting+to+membrane	0.788001	0.674446	0.828714
MF_GO:0030374_ligand-dependent+nuclear+receptor+transcription+coactiv	0.609398	0.600183	0.548907
BP_GO:0048568_embryonic+organ+development	0.46936	0.583878	0.253987
BP_GO:0045598_regulation+of+fat+cell+differentiation	0.987268	0.958912	0.943985
BP_GO:0008343_adult+feeding+behavior	0.914619	0.96774	0.218272
BP_GO:0045670_regulation+of+osteoclast+differentiation	0.0421212	0.222328	0.0280267
BP_GO:0043583_ear+development	0.441048	0.497769	0.419281
BP_GO:0045840_positive+regulation+of+mitosis	0.760787	0.902933	0.578015
MF_GO:0004977_melanocortin+receptor+activity	0.966513	0.995997	0.47628
BP_GO:0043278_response+to+morphine	0.810923	0.832571	0.565018
BP_GO:0006561_proline+biosynthetic+process	0.768218	0.558447	0.813415
BP_GO:0021846_cell+proliferation+in+forebrain	0.211649	0.120013	0.516969
BP_GO:0019933_cAMP-mediated+signaling	0.783111	0.940663	0.197563
CC_GO:0000123_histone+acetyltransferase+complex	0.514644	0.41854	0.709189
BP_GO:0018279_protein+amino+acid+N-linked+glycosylation+via+asparagin	0.44386	0.768411	0.314861
BP_GO:0006406_mRNA+export+from+nucleus	0.959013	0.99286	0.325921
BP_GO:0060070_Wnt+receptor+signaling+pathway+through+beta-catenin	0.72049	0.569211	0.851592
CC_GO:0035097_histone+methyltransferase+complex	0.493867	0.387148	0.727368
CC_GO:0043195_terminal+button	0.625744	0.800979	0.26127
BP_GO:0015904_tetracycline+transport	0.811835	0.898356	0.492007
MF_GO:0015520_tetracycline:hydrogen+antiporter+activity	0.811835	0.898356	0.492007

MF_GO:0031202_RNA+splicing+factor+activity,+transesterification+mechan	0.974318	0.966187	0.863402
CC_GO:0033180_proton-transporting+V-type+ATPase,+V1+domain	0.940797	0.824576	0.917303
BP_GO:0015807_L-amino+acid+transport	0.966683	0.89187	0.912928
MF_GO:0015179_L-amino+acid+transmembrane+transporter+activity	0.966683	0.89187	0.912928
BP_GO:0007346_regulation+of+mitotic+cell+cycle	0.493098	0.372787	0.618108
MF_GO:0008408_3'-5'+exonuclease+activity	0.0948228	0.146616	0.194019
BP_GO:0045429_positive+regulation+of+nitric+oxide+biosynthetic+process	0.324845	0.435015	0.316155
MF_GO:0004908_interleukin-1+receptor+activity	0.0571786	0.210944	0.0548251
BP_GO:0048667_cell+morphogenesis+involved+in+neuron+differentiation	0.37185	0.250833	0.649689
BP_GO:0035058_sensory+cilium+assembly	0.204071	0.546449	0.136672
BP_GO:0007617_mating+behavior	0.84579	0.90519	0.330832
CC_GO:0042101_T+cell+receptor+complex	0.602513	0.877725	0.115958
BP_GO:0060017_parathyroid+gland+development	0.597221	0.87495	0.403689
BP_GO:0040016_embryonic+cleavage	0.244873	0.786775	0.0101216
BP_GO:0030111_regulation+of+Wnt+receptor+signaling+pathway	0.74843	0.300838	0.882901
BP_GO:0000278_mitotic+cell+cycle	0.519675	0.209256	0.973355
BP_GO:0033365_protein+localization+in+organelle	0.47633	0.422288	0.569488
CC_GO:0016514_SWI/SNF+complex	0.857747	0.876196	0.586998
BP_GO:0032088_negative+regulation+of+NF-kappaB+transcription+factor+a	0.506326	0.489858	0.51929
BP_GO:0048599_oocyte+development	0.396057	0.716089	0.103027
BP_GO:0006998_nuclear+envelope+organization	0.41229	0.49378	0.358848
BP_GO:0048596_embryonic+camera-type+eye+morphogenesis	0.815479	0.815479	#N/A
BP_GO:0006020_inositol+metabolic+process	0.523353	0.710553	0.136104
BP_GO:0001763_morphogenesis+of+a+branching+structure	0.934397	0.977329	0.776308
BP_GO:0019227_action+potential+propagation	0.384153	0.274954	0.572055
BP_GO:0009266_response+to+temperature+stimulus	0.687692	0.742474	0.398776
BP_GO:0035019_somatic+stem+cell+maintenance	0.48239	0.772311	0.209418
CC_GO:0001939_female+pronucleus	0.060986	0.303558	0.0254388
BP_GO:0009103_lipopolysaccharide+biosynthetic+process	0.592962	0.506387	0.624467
BP_GO:0040008_regulation+of+growth	0.833254	0.781613	0.722795
BP_GO:0006378_mRNA+polyadenylation	0.926076	0.877263	0.806686
MF_GO:0004415_hyalurononglucosaminidase+activity	0.331395	0.385126	0.372815
BP_GO:0001783_B+cell+apoptosis	0.636678	0.961752	0.205169
CC_GO:0014704_intercalated+disc	0.449798	0.301095	0.804569
BP_GO:0044255_cellular+lipid+metabolic+process	0.0325902	0.0476416	0.201742
CC_GO:0009898_internal+side+of+plasma+membrane	0.561142	0.725941	0.406885
BP_GO:0042490_mechanoreceptor+differentiation	0.387445	0.702709	0.105999
BP_GO:0021798_forebrain+dorsal/ventral+pattern+formation	0.146827	0.12687	0.315826
BP_GO:0006583_melanin+biosynthetic+process+from+tyrosine	0.463784	0.900675	0.0787529
MF_GO:0043236_laminin+binding	0.942916	0.762374	0.93595
BP_GO:0048305_immunoglobulin+secretion	0.912253	0.95458	0.589523
MF_GO:0016799_hydrolase+activity,+hydrolyzing+N-glycosyl+compounds	0.247713	0.758773	0.0915726
MF_GO:0031683_G-protein+beta/gamma-subunit+binding	0.101235	0.200353	0.153967
BP_GO:0016447_somatic+recombination+of+immunoglobulin+gene+segme	0.293632	0.646836	0.0703293
CC_GO:0005833_hemoglobin+complex	0.764053	0.504258	0.95916
MF_GO:0051219_phosphoprotein+binding	0.962687	0.980494	0.402231
CC_GO:0000502_proteasome+complex	0.854971	0.819761	0.705606

MF_GO:0051378_serotonin+binding	0.0257378	#N/A	0.0257378
MF_GO:0031490_chromatin+DNA+binding	0.253345	0.815919	0.0329672
MF_GO:0051087_chaperone+binding	0.0160275	0.0352043	0.0889885
CC_GO:0005858_axonemal+dynein+complex	0.856522	0.800025	0.747092
BP_GO:0046888_negative+regulation+of+hormone+secretion	0.555429	0.533362	0.549241
MF_GO:0008641_small+protein+activating+enzyme+activity	0.322639	0.371862	0.368911
BP_GO:0021984_adenohypophysis+development	0.976509	0.99726	0.313535
BP_GO:0050966_detection+of+mechanical+stimulus+involved+in+sensory+p	0.812732	0.829101	0.577222
MF_GO:0042043_neurexin+binding	0.656035	0.507561	0.748565
CC_GO:0016471_vacuolar+proton-transporting+V-type+ATPase+complex	0.550713	0.924383	0.0352217
CC_GO:0010369_chromocenter	0.183212	#N/A	0.183212
BP_GO:0007128_meiotic+prophase+I	0.766011	0.799165	0.528705
MF_GO:0004972_N-methyl-D-aspartate+selective+glutamate+receptor+acti	0.625403	0.644096	0.533239
BP_GO:0006614_SRP-dependent+cotranslational+protein+targeting+to+mer	0.191561	#N/A	0.191561
BP_GO:0007341_penetration+of+zona+pellucida	0.680958	0.618194	0.642112
BP_GO:0021799_cerebral+cortex+radially+oriented+cell+migration	0.888722	0.920206	0.625452
CC_GO:0005732_small+nucleolar+ribonucleoprotein+complex	0.580308	0.916728	0.136514
BP_GO:0032438_melanosome+organization	0.289535	0.430714	0.237626
BP_GO:0042596_fear+response	0.743279	0.990363	0.0785192
CC_GO:0001518_voltage-gated+sodium+channel+complex	0.514787	0.187533	0.826389
BP_GO:0006325_establishment+or+maintenance+of+chromatin+architectur	0.992634	0.990788	0.906972
BP_GO:0043039_tRNA+aminoacylation	0.760236	0.786686	0.540371
MF_GO:0016876_ligase+activity,+forming+aminoacyl-tRNA+and+related+co	0.760236	0.786686	0.540371
BP_GO:0045104_intermediate+filament+cytoskeleton+organization	0.484508	0.445934	0.519508
BP_GO:0021675_nerve+development	0.466496	0.984164	0.0524938
BP_GO:0060013_righting+reflex	0.65439	0.780087	0.416836
CC_GO:0022627_cytosolic+small+ribosomal+subunit	0.015357	0.0841948	0.0362624
CC_GO:0005883_neurofilament	0.426644	0.732299	0.189184
MF_GO:0000175_3'-5'-exoribonuclease+activity	0.255434	0.173263	0.504634
CC_GO:0005579_membrane+attack+complex	0.835713	0.959805	0.0946881
BP_GO:0019433_triacylglycerol+catabolic+process	0.905155	0.691698	0.928144
BP_GO:0006572_tyrosine+catabolic+process	0.0560617	0.0560617	#N/A
CC_GO:0031430_M+band	0.647506	0.575975	0.64237
BP_GO:0045577_regulation+of+B+cell+differentiation	0.26233	0.224238	0.537417
BP_GO:0032024_positive+regulation+of+insulin+secretion	0.61723	0.428959	0.755431
BP_GO:0030212_hyaluronan+metabolic+process	0.919024	0.919024	#N/A
MF_GO:0004013_adenosylhomocysteinase+activity	0.221008	0.135685	0.552406
MF_GO:0004033_aldo-keto+reductase+activity	0.629673	0.599954	0.584723
BP_GO:0031103_axon+regeneration	0.0197082	0.101481	0.0447048
BP_GO:0046835_carbohydrate+phosphorylation	0.886171	0.734605	0.873134
BP_GO:0019530_taurine+metabolic+process	0.748523	0.635312	0.737775
MF_GO:0016597_amino+acid+binding	0.0462998	0.0780319	0.178117
MF_GO:0004032_aldehyde+reductase+activity	0.0344546	0.0366206	0.247376
BP_GO:0030239_myofibril+assembly	0.943009	0.430819	0.968185
CC_GO:0043034_costamere	0.94992	0.6887	0.944406
CC_GO:0030055_cell-substrate+junction	0.436644	0.449938	0.454164
BP_GO:0018106_peptidyl-histidine+phosphorylation	0.916802	0.883555	0.761243

MF_GO:0004673_protein+histidine+kinase+activity	0.916802	0.883555	0.761243
MF_GO:0004768_stearoyl-CoA+9-desaturase+activity	0.00806864	0.000886956	0.509405
MF_GO:0050291_sphingosine+N-acyltransferase+activity	0.101814	0.117229	0.289737
BP_GO:0002028_regulation+of+sodium+ion+transport	0.79447	0.79447	#N/A
MF_GO:0019864_IgG+binding	0.223291	0.102355	0.636865
BP_GO:0015937_coenzyme+A+biosynthetic+process	0.930859	0.881769	0.828584
CC_GO:0005916_fascia+adherens	0.729227	0.482966	0.84583
BP_GO:0050729_positive+regulation+of+inflammatory+response	0.131933	0.320118	0.116133
BP_GO:0006107_oxaloacetate+metabolic+process	0.54992	0.540778	0.530501
BP_GO:0042168_heme+metabolic+process	0.759348	0.582815	0.876649
BP_GO:0043086_negative+regulation+of+catalytic+activity	0.767142	0.590474	0.809047
BP_GO:0051272_positive+regulation+of+cell+motion	0.0681521	0.954247	0.000481973
BP_GO:0007029_endoplasmic+reticulum+organization	0.4284	0.50171	0.340231
BP_GO:0009168_purine+ribonucleoside+monophosphate+biosynthetic+pro	0.343762	0.577421	0.0984196
MF_GO:0019239_deaminase+activity	0.343762	0.577421	0.0984196
BP_GO:0008631_induction+of+apoptosis+by+oxidative+stress	0.827064	0.476336	0.897286
BP_GO:0043552_positive+regulation+of+phosphoinositide+3-kinase+activity	0.213093	0.293513	0.279676
BP_GO:0042759_long-chain+fatty+acid+biosynthetic+process	0.00107047	0.00403188	0.0536263
BP_GO:0006004_fucose+metabolic+process	0.729246	0.729246	#N/A
BP_GO:0019395_fatty+acid+oxidation	0.897622	0.977948	0.322993
MF_GO:0016776_phosphotransferase+activity,+phosphate+group+as+accep	0.171125	0.172099	0.365728
MF_GO:0019201_nucleotide+kinase+activity	0.171125	0.172099	0.365728
MF_GO:0001968_fibronectin+binding	0.289825	0.649191	0.0892939
BP_GO:0045588_positive+regulation+of+gamma-delta+T+cell+differentiatio	0.614539	0.513114	0.6631
BP_GO:0030177_positive+regulation+of+Wnt+receptor+signaling+pathway	0.980732	0.937483	0.918465
BP_GO:0051091_positive+regulation+of+transcription+factor+activity	0.23519	0.441446	0.0933721
BP_GO:0016202_regulation+of+striated+muscle+development	0.774459	0.754976	0.635802
MF_GO:0000146_microfilament+motor+activity	0.854816	0.64101	0.949954
MF_GO:0005522_profilin+binding	0.833663	0.833663	#N/A
MF_GO:0004559_alpha-mannosidase+activity	0.988445	0.988445	#N/A
BP_GO:0008088_axon+cargo+transport	0.376836	0.433759	0.356504
MF_GO:0047760_butyrate-CoA+ligase+activity	0.344207	0.511645	0.16969
MF_GO:0004499_flavin-containing+monooxygenase+activity	0.0183371	0.0139202	0.270791
BP_GO:0006012_galactose+metabolic+process	0.0790669	0.000855125	0.769736
BP_GO:0030278_regulation+of+ossification	0.474434	0.835996	0.0969425
BP_GO:0043408_regulation+of+MAPKKK+cascade	0.667397	0.655575	0.591908
CC_GO:0000145_exocyst	0.00715616	0.00515928	0.364158
MF_GO:0003857_3-hydroxyacyl-CoA+dehydrogenase+activity	0.214578	0.00906332	0.616649
BP_GO:0048011_nerve+growth+factor+receptor+signaling+pathway	0.567746	0.562755	0.526482
MF_GO:0004679_AMP-activated+protein+kinase+activity	0.496539	0.765238	0.355443
MF_GO:0003847_1-alkyl-2-acetylglycerophosphocholine+esterase+activity	0.308273	0.55631	0.0802024
BP_GO:0000904_cell+morphogenesis+involved+in+differentiation	0.808229	0.793764	0.621951
MF_GO:0016504_protease+activator+activity	0.511065	0.364455	0.680475
BP_GO:0050873_brown+fat+cell+differentiation	0.367707	0.256658	0.605162
BP_GO:0006884_cell+volume+homeostasis	0.894206	0.95515	0.459378
BP_GO:0000070_mitotic+sister+chromatid+segregation	0.235761	0.217079	0.33941
MF_GO:0004565_beta-galactosidase+activity	0.065186	0.00549183	0.955977

MF_GO:0004720_protein-lysine+6-oxidase+activity	0.934822	0.427627	0.995568
BP_GO:0032060_bleb+formation	0.798235	0.991143	0.195829
BP_GO:0045931_positive+regulation+of+mitotic+cell+cycle	0.795521	0.644267	0.803391
BP_GO:0030595_leukocyte+chemotaxis	0.768018	0.722822	0.67853
MF_GO:0008131_amine+oxidase+activity	0.392543	0.557623	0.27145
BP_GO:0007088_regulation+of+mitosis	0.12865	0.306193	0.147119
BP_GO:0008211_glucocorticoid+metabolic+process	0.38501	0.511871	0.309096
BP_GO:0051098_regulation+of+binding	0.944117	0.983155	0.466059
CC_GO:0000276_mitochondrial+proton-transporting+ATP+synthase+comple	0.891464	0.782406	0.840722
BP_GO:0008635_caspase+activation+via+cytochrome+c	0.891551	#N/A	0.891551
BP_GO:0045351_type+I+interferon+biosynthetic+process	0.360501	0.263345	0.583604
BP_GO:0045727_positive+regulation+of+translation	0.520745	0.43645	0.609821
BP_GO:0055074_calcium+ion+homeostasis	0.909445	0.943983	0.666538
BP_GO:0050771_negative+regulation+of+axonogenesis	0.0596727	0.216818	0.0850579
MF_GO:0005066_transmembrane+receptor+protein+tyrosine+kinase+signal	0.501294	0.332283	0.704335
CC_GO:0005795_Golgi+stack	0.522011	0.444616	0.656539
MF_GO:0008093_cytoskeletal+adaptor+activity	0.962041	0.954157	0.77108
CC_GO:0005744_mitochondrial+inner+membrane+presequence+translocase	0.332888	0.632364	0.136312
BP_GO:0045792_negative+regulation+of+cell+size	0.113094	0.134828	0.309019
BP_GO:0048008_platelet-derived+growth+factor+receptor+signaling+pathw	0.265066	0.251957	0.395603
MF_GO:0008266_poly(U)+binding	0.84653	0.496862	0.948084
MF_GO:0019899_enzyme+binding	0.445201	0.81791	0.0919078
BP_GO:0051289_protein+homotetramerization	0.795592	0.766209	0.712621
BP_GO:0045080_positive+regulation+of+chemokine+biosynthetic+process	0.605256	0.519272	0.641973
CC_GO:0005606_laminin-1+complex	0.13799	0.0364233	0.523058
BP_GO:0046579_positive+regulation+of+Ras+protein+signal+transduction	0.694871	0.527423	0.765062
BP_GO:0001832_blastocyst+growth	0.262868	0.37772	0.267058
BP_GO:0006308_DNA+catabolic+process	0.486933	0.412346	0.587105
MF_GO:0030276_clathrin+binding	0.851611	0.890358	0.558173
MF_GO:0008499_UDP-galactose:beta-N-acetylglucosamine+beta-1,3-galact	0.730202	0.769875	0.526476
MF_GO:0030295_protein+kinase+activator+activity	0.0921822	#N/A	0.0921822
MF_GO:0004563_beta-N-acetylhexosaminidase+activity	0.479925	0.698379	0.237056
BP_GO:0014047_glutamate+secretion	0.839412	0.839412	#N/A
BP_GO:0045773_positive+regulation+of+axon+extension	0.231421	0.737224	0.0262722
BP_GO:0048384_retinoic+acid+receptor+signaling+pathway	0.333263	0.075776	0.858958
BP_GO:0030511_positive+regulation+of+transforming+growth+factor+beta+	0.817063	0.822778	0.567896
MF_GO:0005498_sterol+carrier+activity	0.765471	0.769798	0.630396
BP_GO:0042756_drinking+behavior	0.304165	0.564718	0.213497
MF_GO:0042153_RPTP-like+protein+binding	0.23305	0.257603	0.361181
BP_GO:0022409_positive+regulation+of+cell-cell+adhesion	0.810935	0.948693	0.272669
BP_GO:0030433_ER-associated+protein+catabolic+process	0.141086	0.481092	0.0502116
MF_GO:0004445_inositol-polyphosphate+5-phosphatase+activity	0.207673	0.207673	#N/A
BP_GO:0010552_positive+regulation+of+specific+transcription+from+RNA+	0.87962	0.892699	0.692308
BP_GO:0007600_sensory+perception	0.76711	0.76711	#N/A
MF_GO:0003828_alpha-N-acetylneuraminate+alpha-2,8-sialyltransferase+a	0.665569	0.532473	0.786675
BP_GO:0046548_retinal+rod+cell+development	0.37421	0.633849	0.17725
MF_GO:0004861_cyclin-dependent+protein+kinase+inhibitor+activity	0.456158	0.251082	0.657646

MF_GO:0035257_nuclear+hormone+receptor+binding	0.253035	0.340795	0.252554
BP_GO:0019430_removal+of+superoxide+radicals	0.348038	0.662323	0.198813
BP_GO:0002089_lens+morphogenesis+in+camera-type+eye	0.136695	0.175774	0.27888
BP_GO:0051384_response+to+glucocorticoid+stimulus	0.311527	0.234822	0.425405
MF_GO:0008467_[heparan+sulfate]-glucosamine+3-sulfotransferase+1+acti	0.158878	0.569607	0.0759387
MF_GO:0001515_opioid+peptide+activity	0.544576	0.700728	0.211092
BP_GO:0042088_T-helper+1+type+immune+response	0.0179988	0.0434476	0.111296
CC_GO:0001931_uropod	0.85344	0.731823	0.867816
BP_GO:0002467_germinal+center+formation	0.196241	0.913014	0.0134267
CC_GO:0005851_eukaryotic+translation+initiation+factor+2B+complex	0.749392	0.873636	0.474254
BP_GO:0055015_ventricular+cardiac+muscle+cell+development	0.621949	0.640926	0.489394
BP_GO:0015701_bicarbonate+transport	0.279304	0.234125	0.435324
BP_GO:0048644_muscle+morphogenesis	0.484883	0.569715	0.423924
BP_GO:0047496_vesicle+transport+along+microtubule	0.266198	0.425195	0.154029
BP_GO:0007127_meiosis+I	0.991994	0.9833	0.871573
BP_GO:0040020_regulation+of+meiosis	0.670266	0.595895	0.644823
MF_GO:0005173_stem+cell+factor+receptor+binding	0.792231	0.788321	0.587256
BP_GO:0009306_protein+secretion	0.268894	0.569875	0.218697
BP_GO:0060271_cilium+morphogenesis	0.107749	0.0439452	0.4184
BP_GO:0048339_paraxial+mesoderm+development	0.719082	0.719082	#N/A
BP_GO:0021631_optic+nerve+morphogenesis	0.0726709	0.0331723	0.478506
CC_GO:0030659_cytoplasmic+vesicle+membrane	0.805071	0.805071	#N/A
MF_GO:0008517_folic+acid+transporter+activity	0.431782	0.90921	0.0947717
BP_GO:0001779_natural+killer+cell+differentiation	0.750528	0.750528	#N/A
BP_GO:0009101_glycoprotein+biosynthetic+process	0.0544025	0.0639966	0.20383
BP_GO:0048562_embryonic+organ+morphogenesis	0.264046	0.33687	0.314965
CC_GO:0044431_Golgi+apparatus+part	0.388874	0.308811	0.565762
BP_GO:0045787_positive+regulation+of+cell+cycle	0.966886	0.977608	0.673043
MF_GO:0004726_non-membrane+spanning+protein+tyrosine+phosphatase-	0.933382	0.87807	0.848185
BP_GO:0000245_spliceosome+assembly	0.175426	0.781699	0.0761445
CC_GO:0030008_TRAPP+complex	0.238328	0.607898	0.126693
BP_GO:0033014_tetrapyrrole+biosynthetic+process	0.207929	0.0923641	0.512919
BP_GO:0048812_neurite+morphogenesis	0.990018	0.928758	0.988506
BP_GO:0043666_regulation+of+phosphoprotein+phosphatase+activity	0.0446857	0.478737	0.000109934
BP_GO:0006261_DNA-dependent+DNA+replication	0.671304	0.275215	0.786566
BP_GO:0009650_UV+protection	0.480053	0.480053	#N/A
BP_GO:0009620_response+to+fungus	0.475265	0.814498	0.208998
MF_GO:0008190_eukaryotic+initiation+factor+4E+binding	0.720142	0.956855	0.258987
BP_GO:0006590_thyroid+hormone+generation	0.754891	0.899032	0.440333
CC_GO:0005662_DNA+replication+factor+A+complex	0.48846	0.315545	0.638731
CC_GO:0005869_dynactin+complex	0.0564887	0.375585	0.036925
BP_GO:0048762_mesenchymal+cell+differentiation	0.507641	0.823048	0.134618
BP_GO:0006346_methylation-dependent+chromatin+silencing	0.662637	0.655224	0.585645
BP_GO:0048699_generation+of+neurons	0.089045	0.792095	0.0279391
CC_GO:0019031_viral+envelope	0.406612	0.667731	0.25506
BP_GO:0009083_branched+chain+family+amino+acid+catabolic+process	0.408614	0.179633	0.673766
MF_GO:0003704_specific+RNA+polymerase+II+transcription+factor+activity	0.94765	0.948661	0.642626

CC_GO:0019005_SCF+ubiquitin+ligase+complex	0.382342	0.166456	0.559347
BP_GO:0008366_axon+ensheathment	0.545076	0.747809	0.345216
BP_GO:0050727_regulation+of+inflammatory+response	0.417131	0.70901	0.236035
BP_GO:0051225_spindle+assembly	0.574242	0.547672	0.559603
BP_GO:0000154_rRNA+modification	0.81096	0.85755	0.533873
BP_GO:0050892_intestinal+absorption	0.635477	0.714372	0.360402
BP_GO:0048489_synaptic+vesicle+transport	0.205162	0.604713	0.0518333
MF_GO:0017040_ceramidase+activity	0.210808	0.356104	0.206419
BP_GO:0007379_segment+specification	0.131138	0.137579	0.288941
MF_GO:0004620_phospholipase+activity	0.60754	0.796275	0.147617
BP_GO:0016574_histone+ubiquitination	0.129855	0.231964	0.195659
BP_GO:0050908_detection+of+light+stimulus+involved+in+visual+perceptio	0.133741	0.331128	0.0540194
BP_GO:0048515_spermatid+differentiation	0.569449	0.513787	0.59291
BP_GO:0007030_Golgi+organization	0.0821592	0.214033	0.109582
MF_GO:0042169_SH2+domain+binding	0.256347	0.479056	0.0869839
MF_GO:0017016_Ras+GTPase+binding	0.189714	0.129829	0.337457
BP_GO:0045198_establishment+of+epithelial+cell+apical/basal+polarity	0.203073	0.765508	0.0984754
BP_GO:0006415_translational+termination	0.54797	0.407839	0.769473
MF_GO:0003747_translation+release+factor+activity	0.54797	0.407839	0.769473
MF_GO:0002039_p53+binding	0.999682	0.999682	#N/A
BP_GO:0009792_embryonic+development+ending+in+birth+or+egg+hatchir	0.455924	0.495245	0.447183
BP_GO:0001957_intramembranous+ossification	0.466215	0.764002	0.155128
BP_GO:0002070_epithelial+cell+maturation	0.442167	0.704747	0.265442
MF_GO:0004960_thromboxane+receptor+activity	0.196784	0.228943	0.330039
CC_GO:0005637_nuclear+inner+membrane	0.549342	0.580565	0.448753
MF_GO:0031419_cobalamin+binding	0.0785059	0.0785059	#N/A
BP_GO:0021513_spinal+cord+dorsal/ventral+patterning	0.204778	0.233768	0.347901
MF_GO:0005487_nucleocytoplasmic+transporter+activity	0.997024	0.999244	0.423286
MF_GO:0035014_phosphoinositide+3-kinase+regulator+activity	0.465324	0.620411	0.304087
BP_GO:0045879_negative+regulation+of+smoothened+signaling+pathway	0.0887747	0.413724	0.03105
MF_GO:0004708_MAP+kinase+kinase+activity	0.475276	0.880606	0.0616947
BP_GO:0015012_heparan+sulfate+proteoglycan+biosynthetic+process	0.668152	0.303275	0.906481
MF_GO:0019863_IgE+binding	0.296265	0.609484	0.117681
BP_GO:0001101_response+to+acid	0.829767	0.945042	0.470752
BP_GO:0021516_dorsal+spinal+cord+development	0.529728	0.663591	0.402098
BP_GO:0046621_negative+regulation+of+organ+growth	0.154071	0.123793	0.422572
BP_GO:0048541_Peyer's+patch+development	0.655761	0.655761	#N/A
MF_GO:0031405_lipoic+acid+binding	0.273499	0.289923	0.392021
MF_GO:0017091_AU-rich+element+binding	0.918815	0.813989	0.910049
BP_GO:0006265_DNA+topological+change	0.257061	0.365062	0.271249
MF_GO:0003916_DNA+topoisomerase+activity	0.257061	0.365062	0.271249
MF_GO:0050220_prostaglandin-E+synthase+activity	0.690486	#N/A	0.690486
BP_GO:0002328_pro-B+cell+differentiation	0.819375	0.879355	0.58824
BP_GO:0009725_response+to+hormone+stimulus	0.604429	0.740268	0.2432
BP_GO:0000288_nuclear-transcribed+mRNA+catabolic+process,+deadenylat	0.879147	0.919573	0.553677
BP_GO:0043576_regulation+of+respiratory+gaseous+exchange	0.851498	0.702798	0.840904
BP_GO:0030071_regulation+of+mitotic+metaphase/anaphase+transition	0.999903	0.999903	#N/A

BP_GO:0048341_paraxial+mesoderm+formation	0.0838887	0.184856	0.139443
BP_GO:0019217_regulation+of+fatty+acid+metabolic+process	0.393193	0.266356	0.631601
BP_GO:0021680_cerebellar+Purkinje+cell+layer+development	0.242648	0.508908	0.179064
BP_GO:0018101_peptidyl-citrulline+biosynthetic+process+from+peptidyl-arg	0.939209	0.931819	0.838283
MF_GO:0004668_protein-arginine+deiminase+activity	0.939209	0.931819	0.838283
BP_GO:0009950_dorsal/ventral+axis+specification	0.906205	0.962896	0.26646
BP_GO:0046580_negative+regulation+of+Ras+protein+signal+transduction	0.103815	0.17799	0.19428
BP_GO:0030851_granulocyte+differentiation	0.51147	0.500682	0.517533
MF_GO:0042054_histone+methyltransferase+activity	0.156439	0.17369	0.328197
BP_GO:0043193_positive+regulation+of+gene-specific+transcription	0.639572	0.553731	0.655557
BP_GO:0050769_positive+regulation+of+neurogenesis	0.902865	0.917507	0.706569
MF_GO:0043021_ribonucleoprotein+binding	0.103965	0.0886415	0.367344
BP_GO:0007638_mechanosensory+behavior	0.952274	0.872717	0.892999
MF_GO:0016922_ligand-dependent+nuclear+receptor+binding	0.6786	0.716773	0.512765
BP_GO:0048485_sympathetic+nervous+system+development	0.654287	0.256072	0.85271
BP_GO:0046627_negative+regulation+of+insulin+receptor+signaling+pathwa	0.272623	0.232184	0.476043
BP_GO:0016226_iron-sulfur+cluster+assembly	0.158472	0.181415	0.253305
BP_GO:0060042_retina+morphogenesis+in+camera-type+eye	0.0659237	#N/A	0.0659237
BP_GO:0032094_response+to+food	0.959615	0.88177	0.905394
MF_GO:0004947_bradykinin+receptor+activity	0.0193619	0.153158	0.0333261
BP_GO:0007004_telomere+maintenance+via+telomerase	0.619461	0.619461	#N/A
MF_GO:0016624_oxidoreductase+activity,+acting+on+the+aldehyde+or+oxo	0.81154	0.81154	#N/A
BP_GO:0045078_positive+regulation+of+interferon-gamma+biosynthetic+pr	0.589486	0.669653	0.428858
BP_GO:0042136_neurotransmitter+biosynthetic+process	0.0976558	0.317775	0.0710963
MF_GO:0034450_ubiquitin-ubiquitin+ligase+activity	0.399814	0.399814	#N/A
BP_GO:0060040_retinal+bipolar+neuron+differentiation	0.499341	0.883742	0.164474
BP_GO:0030307_positive+regulation+of+cell+growth	0.217721	0.417165	0.201575
BP_GO:0032331_negative+regulation+of+chondrocyte+differentiation	0.804371	0.985058	0.0962109
MF_GO:0005000_vasopressin+receptor+activity	0.92129	0.895562	0.829825
MF_GO:0017176_phosphatidylinositol+N-acetylglucosaminyltransferase+act	0.0109307	#N/A	0.0109307
CC_GO:0001940_male+pronucleus	0.0285881	0.19435	0.0254388
BP_GO:0006777_Mo-molybdopterin+cofactor+biosynthetic+process	0.870975	0.978857	0.242644
BP_GO:0030858_positive+regulation+of+epithelial+cell+differentiation	0.580281	0.704846	0.26646
MF_GO:0004981_muscarinic+acetylcholine+receptor+activity	0.456528	0.645141	0.161362
BP_GO:0006516_glycoprotein+catabolic+process	0.510913	0.451918	0.576073
BP_GO:0035095_behavioral+response+to+nicotine	0.774897	0.706328	0.702841
BP_GO:0007006_mitochondrial+membrane+organization	0.499701	0.642355	0.232332
BP_GO:0046686_response+to+cadmium+ion	0.495473	0.742854	0.207152
CC_GO:0042589_zymogen+granule+membrane	0.778042	0.974292	0.120072
BP_GO:0042053_regulation+of+dopamine+metabolic+process	0.916811	0.97667	0.402437
CC_GO:0046540_U4/U6+x+U5+tri-snRNP+complex	0.395173	0.719043	0.278706
MF_GO:0004982_N-formyl+peptide+receptor+activity	0.658306	0.583581	0.638657
BP_GO:0032314_regulation+of+Rac+GTPase+activity	0.162055	0.0615383	0.810637
BP_GO:0048665_neuron+fate+specification	0.689879	0.689433	0.571037
BP_GO:0042428_serotonin+metabolic+process	0.607244	0.515184	0.649689
CC_GO:0012505_endomembrane+system	0.320018	0.344897	0.401167
BP_GO:0010224_response+to+UV-B	0.14406	0.263918	0.182307

CC_GO:0000784_nuclear+chromosome,+telomeric+region	0.936838	0.936838	#N/A
BP_GO:0042423_catecholamine+biosynthetic+process	0.486261	0.686184	0.258439
BP_GO:0000239_pachytene	0.549337	0.884863	0.0168659
BP_GO:0007431_salivary+gland+development	0.372497	0.486697	0.318055
BP_GO:0014044_Schwann+cell+development	0.736933	0.538457	0.811975
BP_GO:0006685_sphingomyelin+catabolic+process	0.481961	0.481961	#N/A
BP_GO:0048715_negative+regulation+of+oligodendrocyte+differentiation	0.798543	0.714035	0.732132
BP_GO:0033151_V(D)J+recombination	0.425889	0.266347	0.680562
CC_GO:0030137_COPI-coated+vesicle	0.987687	0.926507	0.957172
BP_GO:0045638_negative+regulation+of+myeloid+cell+differentiation	0.558742	0.655089	0.399533
BP_GO:0035094_response+to+nicotine	0.723153	0.463628	0.760583
BP_GO:0048484_enteric+nervous+system+development	0.442697	0.902443	0.106887
BP_GO:0021527_spinal+cord+association+neuron+differentiation	0.35573	0.947182	0.110927
BP_GO:0050678_regulation+of+epithelial+cell+proliferation	0.266998	0.388433	0.283878
BP_GO:0048709_oligodendrocyte+differentiation	0.228912	0.410228	0.18513
CC_GO:0044464_cell+part	0.444093	0.701173	0.192577
BP_GO:0060045_positive+regulation+of+cardiac+muscle+cell+proliferation	0.494387	0.808238	0.23299
BP_GO:0060135_maternal+process+involved+in+pregnancy	0.520961	0.553007	0.468305
BP_GO:0000012_single+strand+break+repair	0.814005	0.976023	0.322385
MF_GO:0004952_dopamine+receptor+activity	0.792569	0.88109	0.438178
MF_GO:0004826_phenylalanine-tRNA+ligase+activity	0.824791	0.944279	0.462973
BP_GO:0048703_embryonic+viscerocranium+morphogenesis	0.531324	0.97903	0.176726
BP_GO:0033138_positive+regulation+of+peptidyl-serine+phosphorylation	0.501227	0.763634	0.191
BP_GO:0045776_negative+regulation+of+blood+pressure	0.591935	0.841846	0.3025
MF_GO:0004994_somatostatin+receptor+activity	0.933171	0.995285	0.427886
MF_GO:0001601_peptide+YY+receptor+activity	0.454635	0.913887	0.209251
MF_GO:0003831_beta-N-acetylglucosaminylglycopeptide+beta-1,4-galactos	0.235538	0.261675	0.368778
CC_GO:0017146_N-methyl-D-aspartate+selective+glutamate+receptor+com	0.629234	0.875732	0.303098
BP_GO:0006354_RNA+elongation	0.925033	0.63893	0.941739
MF_GO:0004652_polynucleotide+adenylyltransferase+activity	0.769894	0.740009	0.648307
BP_GO:0009253_peptidoglycan+catabolic+process	0.0474377	0.0653213	0.133027
BP_GO:0030819_positive+regulation+of+cAMP+biosynthetic+process	0.938391	0.985575	0.405849
BP_GO:0060038_cardiac+muscle+cell+proliferation	0.467051	0.600864	0.2432
BP_GO:0051023_regulation+of+immunoglobulin+secretion	0.635324	0.635324	#N/A
MF_GO:0030235_nitric-oxide+synthase+regulator+activity	0.87605	0.537651	0.921539
BP_GO:0045861_negative+regulation+of+proteolysis	0.981991	0.979661	0.791107
BP_GO:0046697_decidualization	0.926794	0.848506	0.849716
BP_GO:0042035_regulation+of+cytokine+biosynthetic+process	0.751302	0.931866	0.226327
BP_GO:0031575_G1/S+transition+checkpoint	0.28608	0.440352	0.288992
CC_GO:0001740_Barr+body	0.63896	0.659678	0.489196
CC_GO:0005865_striated+muscle+thin+filament	0.175264	0.112496	0.457402
BP_GO:0030300_regulation+of+cholesterol+absorption	0.00527114	0.00613183	0.218272
BP_GO:0006526_arginine+biosynthetic+process	0.327243	0.0580554	0.966247
MF_GO:0001758_retinal+dehydrogenase+activity	0.303332	0.0950762	0.892672
BP_GO:0006597_spermine+biosynthetic+process	0.957649	0.777483	0.953135
CC_GO:0002102_podosome	0.155978	0.161334	0.275317
BP_GO:0006547_histidine+metabolic+process	0.390976	0.390976	#N/A

MF_GO:0004645_phosphorylase+activity	0.535527	0.530293	0.518905
BP_GO:0008295_spermidine+biosynthetic+process	0.596776	0.536343	0.591228
MF_GO:0004022_alcohol+dehydrogenase+activity	0.442023	0.526193	0.418407
MF_GO:0004470_malic+enzyme+activity	0.000431713	0.000431713	#N/A
BP_GO:0002639_positive+regulation+of+immunoglobulin+production	0.65027	0.65027	#N/A
BP_GO:0014823_response+to+activity	0.233359	0.492173	0.156335
CC_GO:0005826_contractile+ring	0.287116	0.287116	#N/A
BP_GO:0060047_heart+contraction	0.882487	0.786161	0.819703
MF_GO:0003846_2-acylglycerol+O-acyltransferase+activity	0.743982	0.923642	0.307898
MF_GO:0004111_creatine+kinase+activity	0.930442	0.868816	0.845851
BP_GO:0033089_positive+regulation+of+T+cell+differentiation+in+the+thym	0.258362	0.352797	0.260264
BP_GO:0043534_blood+vessel+endothelial+cell+migration	0.780144	0.574739	0.88891
CC_GO:0009341_beta-galactosidase+complex	0.00549183	0.00549183	#N/A
BP_GO:0042590_antigen+processing+and+presentation+of+exogenous+pep	0.299475	0.137051	0.636865
BP_GO:0001798_positive+regulation+of+type+IIa+hypersensitivity	0.423697	0.267017	0.636865
BP_GO:0043149_stress+fiber+formation	0.543068	0.981905	0.139344
CC_GO:0010008_endosome+membrane	0.133911	0.153474	0.245111
MF_GO:0008967_phosphoglycolate+phosphatase+activity	0.0470489	0.0102043	0.480287
CC_GO:0031672_A+band	0.713231	0.471061	0.894933
BP_GO:0020027_hemoglobin+metabolic+process	0.424499	0.357791	0.599116
BP_GO:0006707_cholesterol+catabolic+process	0.000266722	0.0660626	2.50507E-05
MF_GO:0004028_3-chloroallyl+aldehyde+dehydrogenase+activity	0.673952	0.36701	0.836009
BP_GO:0006699_bile+acid+biosynthetic+process	0.00286687	0.00332206	0.204641
MF_GO:0051010_microtubule+plus-end+binding	0.23689	0.16251	0.607404
BP_GO:0006081_cellular+aldehyde+metabolic+process	0.729371	0.827699	0.339224
BP_GO:0050832_defense+response+to+fungus	0.330215	0.286383	0.539333
MF_GO:0016717_oxidoreductase+activity,+acting+on+paired+donors,+with-	4.21394E-05	0.000042057	0.145869
BP_GO:0046638_positive+regulation+of+alpha-beta+T+cell+differentiation	0.236748	0.212394	0.479725
MF_GO:0004740_pyruvate+dehydrogenase+(acetyl-transferring)+kinase+act	0.95101	0.933404	0.761243
BP_GO:0002526_acute+inflammatory+response	0.678143	0.706157	0.587735
MF_GO:0004749_ribose+phosphate+diphosphokinase+activity	0.22271	0.254879	0.350389
BP_GO:0006101_citrate+metabolic+process	0.30751	0.504826	0.234797
MF_GO:0004062_aryl+sulfotransferase+activity	0.0906877	0.131787	0.230548
BP_GO:0021940_positive+regulation+of+granule+cell+precursor+proliferatic	0.80576	0.726875	0.731468
CC_GO:0016010_dystrophin-associated+glycoprotein+complex	0.55189	0.201766	0.84623
BP_GO:0042159_lipoprotein+catabolic+process	0.651739	0.651739	#N/A
CC_GO:0030140_trans-Golgi+network+transport+vesicle	0.941592	0.941592	#N/A
BP_GO:0009404_toxin+metabolic+process	0.616212	0.464058	0.653118
BP_GO:0007167_enzyme+linked+receptor+protein+signaling+pathway	0.808707	0.85039	0.479725
BP_GO:0007009_plasma+membrane+organization	0.448586	0.701445	0.120156
BP_GO:0051346_negative+regulation+of+hydrolase+activity	0.0739695	0.0739695	#N/A
MF_GO:0004030_aldehyde+dehydrogenase+[NAD(P)+]+activity	0.813205	0.827699	0.557107
BP_GO:0022027_interkinetic+nuclear+migration	0.867589	0.969539	0.154931
BP_GO:0032886_regulation+of+microtubule-based+process	0.867589	0.969539	0.154931
BP_GO:0006361_transcription+initiation+from+RNA+polymerase+I+promote	0.996808	0.996808	#N/A
BP_GO:0048771_tissue+remodeling	0.90417	0.90417	#N/A
BP_GO:0006742_NADP+catabolic+process	0.876116	0.876116	#N/A

MF_GO:0015175_neutral+amino+acid+transmembrane+transporter+activity	0.377433	#N/A	0.377433
BP_GO:0001523_retinoid+metabolic+process	0.849009	0.526193	0.875864
BP_GO:0051138_positive+regulation+of+NK+T+cell+differentiation	0.954258	0.976184	0.478428
BP_GO:0006497_protein+amino+acid+lipidation	0.0392635	0.100678	0.0956909
BP_GO:0045123_cellular+extravasation	0.803177	0.826903	0.530079
BP_GO:0006596_polyamine+biosynthetic+process	0.235656	0.364785	0.250482
BP_GO:0033344_cholesterol+efflux	0.538438	0.538438	#N/A
BP_GO:0050715_positive+regulation+of+cytokine+secretion	0.266149	0.559478	0.0656662
BP_GO:0045647_negative+regulation+of+erythrocyte+differentiation	0.696455	0.696455	#N/A
BP_GO:0032963_collagen+metabolic+process	0.999578	0.997962	0.955829
BP_GO:0002318_myeloid+progenitor+cell+differentiation	0.419123	0.366334	0.521241
MF_GO:0003997_acyl-CoA+oxidase+activity	0.878117	0.878117	#N/A
BP_GO:0007176_regulation+of+epidermal+growth+factor+receptor+activity	0.220615	0.530061	0.122108
BP_GO:0006105_succinate+metabolic+process	0.612958	0.61322	0.530501
MF_GO:0004957_prostaglandin+E+receptor+activity	0.392237	0.543998	0.309612
BP_GO:0070059_apoptosis+in+response+to+endoplasmic+reticulum+stress	0.394598	#N/A	0.394598
BP_GO:0050718_positive+regulation+of+interleukin-1+beta+secretion	0.86312	0.983615	0.0656662
BP_GO:0042541_hemoglobin+biosynthetic+process	0.962629	0.993081	0.242438
CC_GO:0005664_nuclear+origin+of+replication+recognition+complex	0.670006	0.168032	0.943508
BP_GO:0002024_diet+induced+thermogenesis	0.194746	0.373877	0.185282
BP_GO:0016556_mRNA+modification	0.0682034	0.310018	0.0169487
BP_GO:0000103_sulfate+assimilation	0.355014	0.371561	0.430293
BP_GO:0007288_sperm+axoneme+assembly	0.0419216	0.0349682	0.170888
BP_GO:0045636_positive+regulation+of+melanocyte+differentiation	0.186871	0.190815	0.35083
BP_GO:0001778_plasma+membrane+repair	0.35075	0.291219	0.573921
BP_GO:0042307_positive+regulation+of+protein+import+into+nucleus	0.403338	0.206596	0.681739
BP_GO:0035235_ionotropic+glutamate+receptor+signaling+pathway	0.491779	0.733885	0.350567
BP_GO:0002925_positive+regulation+of+humoral+immune+response+media	0.536691	0.536691	#N/A
BP_GO:0014037_Schwann+cell+differentiation	0.595441	0.558987	0.589781
BP_GO:0046326_positive+regulation+of+glucose+import	0.553609	0.711686	0.434047
BP_GO:0046836_glycolipid+transport	0.639497	0.639497	#N/A
MF_GO:0017089_glycolipid+transporter+activity	0.639497	0.639497	#N/A
MF_GO:0051861_glycolipid+binding	0.639497	0.639497	#N/A
MF_GO:0015923_mannosidase+activity	0.976336	0.976336	#N/A
MF_GO:0004614_phosphoglucomutase+activity	0.75107	0.363749	0.90457
MF_GO:0004551_nucleotide+diphosphatase+activity	0.239214	0.351996	0.266826
MF_GO:0001530_lipopolysaccharide+binding	0.935117	0.946509	0.702686
CC_GO:0001527_microfibril	0.746408	0.87183	0.422225
MF_GO:0015101_organic+cation+transmembrane+transporter+activity	0.504167	0.521129	0.492765
BP_GO:0045651_positive+regulation+of+macrophage+differentiation	0.315347	0.152954	0.63464
MF_GO:0004046_aminoacylase+activity	0.741418	0.87092	0.415349
MF_GO:0030976_thiamin+pyrophosphate+binding	0.598728	0.525087	0.652522
BP_GO:0042116_macrophage+activation	0.529467	0.690302	0.238139
BP_GO:0000281_cytokinesis+after+mitosis	0.508225	0.50332	0.510967
BP_GO:0015816_glycine+transport	0.937337	0.986815	0.21765
MF_GO:0015187_glycine+transmembrane+transporter+activity	0.937337	0.986815	0.21765
BP_GO:0007091_mitotic+metaphase/anaphase+transition	0.199406	0.472528	0.175069

BP_GO:0032020_ISG15-protein+conjugation	0.00329468	0.00756894	0.109757
BP_GO:0043277_apoptotic+cell+clearance	0.809405	0.905567	0.470332
CC_GO:0043227_membrane-bounded+organelle	0.687246	0.896496	0.113239
BP_GO:0001667_ameboidal+cell+migration	0.553713	0.581122	0.494744
BP_GO:0042535_positive+regulation+of+tumor+necrosis+factor+biosyntheti	0.92616	0.695025	0.938067
BP_GO:0021955_central+nervous+system+neuron+axonogenesis	0.511454	0.459782	0.592181
MF_GO:0043023_ribosomal+large+subunit+binding	0.000576075	0.00276756	0.0447723
MF_GO:0004718_Janus+kinase+activity	0.821349	0.821349	#N/A
BP_GO:0009166_nucleotide+catabolic+process	0.019884	0.0955381	0.054663
BP_GO:0030501_positive+regulation+of+bone+mineralization	0.581955	0.736732	0.366906
BP_GO:0001838_embryonic+epithelial+tube+formation	0.623015	0.649318	0.524106
BP_GO:0007216_metabotropic+glutamate+receptor+signaling+pathway	0.81752	0.440049	0.981036
BP_GO:0001991_regulation+of+systemic+arterial+blood+pressure+by+circul	0.107955	0.135729	0.213497
BP_GO:0002829_negative+regulation+of+T-helper+2+type+immune+respon	0.0029444	0.317762	0.00182623
BP_GO:0050919_negative+chemotaxis	0.204249	0.116258	0.661093
MF_GO:0030898_actin-dependent+ATPase+activity	0.320283	0.320283	#N/A
BP_GO:0050793_regulation+of+developmental+process	0.718251	0.340323	0.969285
BP_GO:0050774_negative+regulation+of+dendrite+morphogenesis	0.230305	0.947626	0.0369109
BP_GO:0009437_carnitine+metabolic+process	0.949047	0.873156	0.879668
BP_GO:0001835_blastocyst+hatching	0.137274	0.139387	0.378533
BP_GO:0000255_allantoin+metabolic+process	0.360296	0.323887	0.530501
BP_GO:0019441_tryptophan+catabolic+process+to+kynurenine	0.701939	0.701939	#N/A
BP_GO:0043403_skeletal+muscle+regeneration	0.790779	0.784894	0.684293
BP_GO:0046651_lymphocyte+proliferation	0.918944	#N/A	0.918944
CC_GO:0008290_F-actin+capping+protein+complex	0.398399	0.52841	0.261623
BP_GO:0007094_mitotic+cell+cycle+spindle+assembly+checkpoint	0.721114	0.276881	0.922482
BP_GO:0045332_phospholipid+translocation	0.985757	0.973234	0.850672
BP_GO:0045213_neurotransmitter+receptor+metabolic+process	0.047026	0.0145925	0.425621
BP_GO:0035196_gene+silencing+by+miRNA,+production+of+miRNAs	0.0212507	0.0212507	#N/A
BP_GO:0043542_endothelial+cell+migration	0.618165	0.618165	#N/A
MF_GO:0004104_cholinesterase+activity	0.246701	0.145123	0.535479
BP_GO:0015908_fatty+acid+transport	0.848613	0.240776	0.984703
MF_GO:0046934_phosphatidylinositol-4,5-bisphosphate+3-kinase+activity	0.590771	0.205152	0.970435
BP_GO:0001660_fever	0.0213803	0.00521807	0.380183
CC_GO:0005793_ER-Golgi+intermediate+compartment	0.595556	0.756703	0.235421
MF_GO:0004126_cytidine+deaminase+activity	0.538549	0.255206	0.786879
MF_GO:0004769_steroid+delta-isomerase+activity	0.230922	0.314318	0.263037
MF_GO:0045499_chemorepellent+activity	0.260557	0.260557	#N/A
BP_GO:0043009_chordate+embryonic+development	0.976314	0.921647	0.935164
MF_GO:0004185_serine-type+carboxypeptidase+activity	0.56063	0.45985	0.684666
CC_GO:0031982_vesicle	0.230237	0.218518	0.448404
BP_GO:0051271_negative+regulation+of+cell+motion	0.077432	0.083186	0.326988
BP_GO:0015833_peptide+transport	0.960253	0.960253	#N/A
MF_GO:0015197_peptide+transporter+activity	0.960253	0.960253	#N/A
BP_GO:0046849_bone+remodeling	0.336543	0.835738	0.0577987
BP_GO:0008053_mitochondrial+fusion	0.987414	0.999633	0.417328
BP_GO:0032733_positive+regulation+of+interleukin-10+production	0.521108	0.617922	0.411138

BP_GO:0015871_choline+transport	0.0812091	0.451637	0.0318348
MF_GO:0016462_pyrophosphatase+activity	0.917514	0.737359	0.892055
BP_GO:0042473_outer+ear+morphogenesis	0.129111	0.112838	0.435418
CC_GO:0030132_clathrin+coat+of+coated+pit	0.483705	0.561087	0.416435
BP_GO:0002035_brain+renin-angiotensin+system	0.0112615	0.153158	0.0137486
BP_GO:0048843_negative+regulation+of+axon+extension+involved+in+axor	0.0813726	0.116258	0.234443
BP_GO:0051262_protein+tetramerization	0.435566	0.459779	0.440759
MF_GO:0005332_gamma-aminobutyric+acid:sodium+symporter+activity	0.941367	0.941367	#N/A
BP_GO:0042420_dopamine+catabolic+process	0.771939	0.771939	#N/A
BP_GO:0050768_negative+regulation+of+neurogenesis	0.931716	0.974636	0.342468
MF_GO:0008113_peptide-methionine-(S)-S-oxide+reductase+activity	0.241327	0.158228	0.503591
BP_GO:0012501_programmed+cell+death	0.740375	0.965459	0.182603
BP_GO:0050803_regulation+of+synapse+structure+and+activity	0.849384	0.988946	0.204596
MF_GO:0015355_secondary+active+monocarboxylate+transmembrane+trar	0.110916	0.588965	0.0617438
MF_GO:0004300_enoyl-CoA+hydratase+activity	0.0550704	0.00185936	0.739333
BP_GO:0050778_positive+regulation+of+immune+response	0.322065	0.595763	0.185274
BP_GO:0030384_phosphoinositide+metabolic+process	0.807961	0.920512	0.242478
CC_GO:0031252_cell+leading+edge	0.7849	0.730666	0.696404
CC_GO:0016469_proton-transporting+two-sector+ATPase+complex	0.953868	0.729859	0.989539
CC_GO:0031253_cell+projection+membrane	0.614083	0.570646	0.607404
MF_GO:0004427_inorganic+diphosphatase+activity	0.58908	0.31361	0.705615
BP_GO:0006351_transcription,+DNA-dependent	0.473785	0.364244	0.600489
BP_GO:0030146_diuresis	0.948251	0.980037	0.598536
BP_GO:0045061_thymic+T+cell+selection	0.44655	0.162542	0.786532
BP_GO:0045930_negative+regulation+of+mitotic+cell+cycle	0.111454	0.484102	0.0460818
BP_GO:0048255_mRNA+stabilization	0.135094	0.172477	0.284457
BP_GO:0010172_embryonic+body+morphogenesis	0.782224	0.949051	0.297358
BP_GO:0030261_chromosome+condensation	0.0658426	0.191807	0.103636
MF_GO:0009374_biotin+binding	0.140503	0.1423	0.381048
BP_GO:0001938_positive+regulation+of+endothelial+cell+proliferation	0.557528	0.302123	0.76533
BP_GO:0006927_transformed+cell+apoptosis	0.335848	0.397986	0.366953
CC_GO:0019815_B+cell+receptor+complex	0.39983	0.560198	0.351927
BP_GO:0000303_response+to+superoxide	0.0874414	0.232039	0.117646
MF_GO:0046965_retinoid+X+receptor+binding	0.788495	0.503538	0.943985
CC_GO:0005658_alpha+DNA+polymerase:primase+complex	0.47504	0.152989	0.950496
CC_GO:0005868_cytoplasmic+dynein+complex	0.106816	#N/A	0.106816
BP_GO:0050435_beta-amyloid+metabolic+process	0.131237	0.302446	0.159798
BP_GO:0030818_negative+regulation+of+cAMP+biosynthetic+process	0.67001	0.385358	0.819747
BP_GO:0000089_mitotic+metaphase	0.431575	0.431575	#N/A
MF_GO:0004656_procollagen-proline+4-dioxygenase+activity	0.635423	0.635423	#N/A
BP_GO:0021545_cranial+nerve+development	0.318719	0.357294	0.378694
BP_GO:0002051_osteoblast+fate+commitment	0.00820403	0.0447001	0.0449289
MF_GO:0004308_exo-alpha-sialidase+activity	0.845753	0.918287	0.645232
BP_GO:0032816_positive+regulation+of+natural+killer+cell+activation	0.629842	0.585348	0.614253
BP_GO:0033299_secretion+of+lysosomal+enzymes	0.629842	0.585348	0.614253
BP_GO:0045736_negative+regulation+of+cyclin-dependent+protein+kinase+	0.163317	0.251082	0.236919
BP_GO:0001736_establishment+of+planar+polarity	0.857764	0.83589	0.673133

MF_GO:0050431_transforming+growth+factor+beta+binding	0.779378	0.688873	0.754368
MF_GO:0004536_deoxyribonuclease+activity	0.430328	0.319909	0.587105
BP_GO:0045333_cellular+respiration	0.934666	0.936754	0.729379
MF_GO:0042801_polo+kinase+kinase+activity	0.311385	0.130208	0.666372
BP_GO:0009967_positive+regulation+of+signal+transduction	0.492658	#N/A	0.492658
BP_GO:0045779_negative+regulation+of+bone+resorption	0.950062	0.950062	#N/A
MF_GO:0030332_cyclin+binding	0.535703	0.546739	0.503941
BP_GO:0051881_regulation+of+mitochondrial+membrane+potential	0.0695184	0.192649	0.113655
BP_GO:0048617_embryonic+foregut+morphogenesis	0.862553	0.842475	0.752298
BP_GO:0006906_vesicle+fusion	0.0674841	0.0249089	0.276151
BP_GO:0019885_antigen+processing+and+presentation+of+endogenous+pe	0.46869	0.375571	0.581797
BP_GO:0002326_B+cell+lineage+commitment	0.46522	0.60085	0.352516
CC_GO:0005652_nuclear+lamina	0.413909	0.429948	0.448753
BP_GO:0006269_DNA+replication,+synthesis+of+RNA+primer	0.882543	0.497245	0.954291
MF_GO:0004095_carnitine+O-palmitoyltransferase+activity	0.444613	0.401225	0.521384
BP_GO:0002904_positive+regulation+of+B+cell+apoptosis	0.650541	0.925294	0.185698
CC_GO:0005721_centromeric+heterochromatin	0.956651	0.68587	0.955387
BP_GO:0060296_regulation+of+cilium+beat+frequency+involved+in+ciliary+	0.139177	0.0349682	0.4184
BP_GO:0015074_DNA+integration	0.852205	0.948937	0.438698
MF_GO:0000179_rRNA+(adenine-N6,N6-)-dimethyltransferase+activity	0.85882	0.85755	0.617569
MF_GO:0008649_rRNA+methyltransferase+activity	0.85882	0.85755	0.617569
CC_GO:0008091_spectrin	0.275173	0.275173	#N/A
BP_GO:0006610_ribosomal+protein+import+into+nucleus	0.80899	0.791265	0.635028
BP_GO:0046632_alpha-beta+T+cell+differentiation	0.337223	0.279325	0.441433
BP_GO:0048013_ephrin+receptor+signaling+pathway	0.0995013	0.0819395	0.335573
BP_GO:0048558_embryonic+gut+morphogenesis	0.29366	0.0197821	0.712623
MF_GO:0005313_L-glutamate+transmembrane+transporter+activity	0.143338	0.507177	0.0636398
BP_GO:0007386_compartment+specification	0.609546	0.609546	#N/A
MF_GO:0005005_transmembrane-ephrin+receptor+activity	0.214474	0.213555	0.372789
BP_GO:0007028_cytoplasm+organization	0.817527	0.817527	#N/A
MF_GO:0043015_gamma-tubulin+binding	0.630425	0.400907	0.765046
BP_GO:0001817_regulation+of+cytokine+production	0.22676	0.0551736	0.704239
BP_GO:0009308_cellular+amine+metabolic+process	0.633827	0.836247	0.432682
MF_GO:0015631_tubulin+binding	0.165374	0.66212	0.0364198
CC_GO:0005853_eukaryotic+translation+elongation+factor+1+complex	0.640681	0.37549	0.796051
MF_GO:0048365_Rac+GTPase+binding	0.626692	0.626692	#N/A
MF_GO:0016614_oxidoreductase+activity,+acting+on+CH-OH+group+of+do	0.143306	0.110291	0.388914
BP_GO:0050965_detection+of+temperature+stimulus+involved+in+sensory+	0.544732	0.568561	0.494725
BP_GO:0051146_striated+muscle+cell+differentiation	0.966518	0.971141	0.647395
BP_GO:0055072_iron+ion+homeostasis	0.0604723	0.0541114	0.278456
BP_GO:0001895_retina+homeostasis	0.146237	#N/A	0.146237
BP_GO:0048149_behavioral+response+to+ethanol	0.888299	0.819333	0.803921
BP_GO:0051898_negative+regulation+of+protein+kinase+B+signaling+casca	0.67751	0.626775	0.641531
BP_GO:0060158_dopamine+receptor,+phospholipase+C+activating+pathwa	0.99741	0.99741	#N/A
MF_GO:0005337_nucleoside+transmembrane+transporter+activity	0.827036	0.4841	0.915264
BP_GO:0007599_hemostasis	0.467412	0.467412	#N/A
BP_GO:0032713_negative+regulation+of+interleukin-4+production	0.110552	0.208743	0.178811

BP_GO:0048608_reproductive+structure+development	0.598859	0.537986	0.63179
BP_GO:0031119_tRNA+pseudouridine+synthesis	0.601599	0.535642	0.608481
BP_GO:0050810_regulation+of+steroid+biosynthetic+process	0.210932	#N/A	0.210932
BP_GO:0001825_blastocyst+formation	0.765794	0.898725	0.402087
MF_GO:0004439_phosphatidylinositol-4,5-bisphosphate+5-phosphatase+act	0.873241	0.748989	0.869242
BP_GO:0034109_homotypic+cell-cell+adhesion	0.994581	0.994581	#N/A
MF_GO:0042562_hormone+binding	0.809631	0.933594	0.197657
BP_GO:0045822_negative+regulation+of+heart+contraction	0.615039	0.199456	0.979733
MF_GO:0004500_dopamine+beta-monooxygenase+activity	0.140828	0.200994	0.246784
BP_GO:0030166_proteoglycan+biosynthetic+process	0.987396	0.901473	0.969757
BP_GO:0043129_surfactant+homeostasis	0.757156	0.757156	#N/A
BP_GO:0043069_negative+regulation+of+programmed+cell+death	0.110442	0.114314	0.358222
BP_GO:0045745_positive+regulation+of+G-protein+coupled+receptor+prote	0.17246	0.199511	0.272967
BP_GO:0060124_positive+regulation+of+growth+hormone+secretion	0.865328	0.960693	0.422514
BP_GO:0042787_protein+ubiquitination+during+ubiquitin-dependent+prote	0.376133	0.622966	0.292903
MF_GO:0017147_Wnt-protein+binding	0.713567	0.953054	0.190256
BP_GO:0019228_regulation+of+action+potential+in+neuron	0.209898	0.209898	#N/A
CC_GO:0030673_axolemma	0.529343	0.401225	0.719596
MF_GO:0005381_iron+ion+transmembrane+transporter+activity	0.0709927	0.186126	0.118039
BP_GO:0030010_establishment+of+cell+polarity	0.530094	0.772542	0.261118
MF_GO:0001730_2'-5'-oligoadenylate+synthetase+activity	0.664768	0.209751	0.830856
BP_GO:0045580_regulation+of+T+cell+differentiation	0.237282	0.189519	0.537417
BP_GO:0042403_thyroid+hormone+metabolic+process	0.441367	0.693666	0.12057
MF_GO:0042623_ATPase+activity,+coupled	0.319424	0.319424	#N/A
BP_GO:0033152_immunoglobulin+V(D)J+recombination	0.72357	0.776721	0.448026
BP_GO:0001514_selenocysteine+incorporation	0.915266	0.974335	0.265422
BP_GO:0006091_generation+of+precursor+metabolites+and+energy	0.452105	0.429099	0.527672
BP_GO:0030949_positive+regulation+of+vascular+endothelial+growth+facto	0.91419	0.991309	0.0829484
BP_GO:0031122_cytoplasmic+microtubule+organization	0.47205	0.278113	0.810637
MF_GO:0016742_hydroxymethyl-,+formyl-+and+related+transferase+activit	0.561989	0.403194	0.769668
CC_GO:0005663_DNA+replication+factor+C+complex	0.339769	0.339769	#N/A
MF_GO:0003689_DNA+clamp+loader+activity	0.339769	0.339769	#N/A
BP_GO:0048675_axon+extension	0.642	0.327334	0.83211
MF_GO:0005088_Ras+guanyl-nucleotide+exchange+factor+activity	0.442844	0.33497	0.674234
BP_GO:0048565_gut+development	0.684858	0.684858	#N/A
BP_GO:0015780_nucleotide-sugar+transport	0.787606	0.732028	0.695257
MF_GO:0005338_nucleotide-sugar+transmembrane+transporter+activity	0.787606	0.732028	0.695257
BP_GO:0021854_hypothalamus+development	0.725204	0.913823	0.302383
BP_GO:0046642_negative+regulation+of+alpha-beta+T+cell+proliferation	0.0447307	0.02975	0.447269
BP_GO:0021795_cerebral+cortex+cell+migration	0.476992	0.736151	0.113239
BP_GO:0021696_cerebellar+cortex+morphogenesis	0.243444	0.226893	0.355538
CC_GO:0070044_synaptobrevin+2-SNAP-25-syntaxin-1a+complex	0.32431	0.795157	0.0709637
CC_GO:0005638_lamin+filament	0.469696	0.583259	0.302938
BP_GO:0015879_carnitine+transport	0.514949	0.572003	0.475662
MF_GO:0015226_carnitine+transporter+activity	0.514949	0.572003	0.475662
BP_GO:0021884_forebrain+neuron+development	0.948381	0.949231	0.664263
BP_GO:0006875_cellular+metal+ion+homeostasis	0.0372264	0.668351	0.0104172

BP_GO:0042476_odontogenesis	0.0466811	0.0690563	0.186561
BP_GO:0006595_polyamine+metabolic+process	0.194948	0.199706	0.354458
BP_GO:0006287_base-excision+repair,+gap-filling	0.15832	0.15832	#N/A
CC_GO:0005930_axoneme	0.175274	0.175274	#N/A
BP_GO:0031532_actin+cytoskeleton+reorganization	0.29777	0.313257	0.369972
BP_GO:0001945_lymph+vessel+development	0.333562	0.789897	0.0786699
BP_GO:0060011_Sertoli+cell+proliferation	0.708393	0.743375	0.548967
MF_GO:0003840_gamma-glutamyltransferase+activity	0.527538	0.638071	0.399341
BP_GO:0035021_negative+regulation+of+Rac+protein+signal+transduction	0.355672	0.174931	0.810637
MF_GO:0003923_GPI-anchor+transamidase+activity	0.568958	0.798928	0.388836
BP_GO:0030033_microvillus+assembly	0.950719	0.923065	0.798338
BP_GO:0045806_negative+regulation+of+endocytosis	0.423203	0.534223	0.359632
MF_GO:0004060_arylamine+N-acetyltransferase+activity	0.738966	0.148747	0.974297
BP_GO:0043303_mast+cell+degranulation	0.67188	0.921286	0.381622
BP_GO:0050882_voluntary+musculoskeletal+movement	0.803898	0.638229	0.783617
MF_GO:0004784_superoxide+dismutase+activity	0.281051	#N/A	0.281051
BP_GO:0046887_positive+regulation+of+hormone+secretion	0.936054	0.983231	0.511563
BP_GO:0008406_gonad+development	0.335617	0.197997	0.499946
BP_GO:0043200_response+to+amino+acid+stimulus	0.379418	0.447667	0.349783
MF_GO:0004735_pyrroline-5-carboxylate+reductase+activity	0.859767	0.731565	0.813415
BP_GO:0021612_facial+nerve+structural+organization	0.960895	0.96439	0.655113
BP_GO:0007143_female+meiosis	0.350854	0.244795	0.666968
MF_GO:0004974_leukotriene+receptor+activity	0.559271	0.42383	0.736347
BP_GO:0042416_dopamine+biosynthetic+process	0.950432	0.99001	0.232332
CC_GO:0043204_perikaryon	0.866982	0.866982	#N/A
BP_GO:0055088_lipid+homeostasis	0.555373	0.555373	#N/A
BP_GO:0060173_limb+development	0.149381	0.279665	0.142883
CC_GO:0008250_oligosaccharyltransferase+complex	0.225957	0.768411	0.0982403
CC_GO:0005958_DNA-dependent+protein+kinase+complex	0.876874	0.879355	0.680562
MF_GO:0005499_vitamin+D+binding	0.734238	0.656472	0.710508
BP_GO:0015844_monoamine+transport	0.940624	0.960664	0.530142
MF_GO:0000030_mannosyltransferase+activity	0.997571	0.992807	0.918648
MF_GO:0005243_gap+junction+channel+activity	0.0572474	0.0726112	0.163034
BP_GO:0032495_response+to+muramyl+dipeptide	0.890829	0.890829	#N/A
MF_GO:0046703_natural+killer+cell+lectin-like+receptor+binding	0.413722	0.148026	0.637388
CC_GO:0031513_nonmotile+primary+cilium	0.996644	0.996578	0.770281
BP_GO:0002669_positive+regulation+of+T+cell+anergy	0.174447	0.471448	0.105067
MF_GO:0008353_RNA+polymerase+subunit+kinase+activity	0.501468	0.599988	0.402223
BP_GO:0006510_ATP-dependent+proteolysis	0.726046	0.726046	#N/A
MF_GO:0004176_ATP-dependent+peptidase+activity	0.726046	0.726046	#N/A
BP_GO:0016322_neuron+remodeling	0.701575	0.701575	#N/A
CC_GO:0070032_synaptobrevin+2-SNAP-25-syntaxin-1a-complexin+I+compl	0.358473	0.830519	0.0709637
MF_GO:0005152_interleukin-1+receptor+antagonist+activity	0.908181	0.654879	0.97567
CC_GO:0032281_alpha-amino-3-hydroxy-5-methyl-4-isoxazolepropionic+aci	0.684007	0.760313	0.394889
CC_GO:0035098_ESC/E(Z)+complex	0.137659	0.136092	0.328197
BP_GO:0051798_positive+regulation+of+hair+follicle+development	0.680816	0.680816	#N/A
CC_GO:0005798_Golgi-associated+vesicle	0.316574	0.61516	0.166624

BP_GO:0021938_smoothened+signaling+pathway+involved+in+regulation+c	0.519279	0.542606	0.484808
BP_GO:0048025_negative+regulation+of+nuclear+mRNA+splicing,+via+splic	0.944208	0.981886	0.328384
MF_GO:0016594_glycine+binding	0.683346	0.624515	0.639882
BP_GO:0048814_regulation+of+dendrite+morphogenesis	0.547733	0.539016	0.528798
BP_GO:0032720_negative+regulation+of+tumor+necrosis+factor+productio	0.858483	0.403462	0.961117
MF_GO:0004999_vasoactive+intestinal+polypeptide+receptor+activity	0.746041	0.337874	0.912376
BP_GO:0007066_female+meiosis+sister+chromatid+cohesion	0.543602	0.572053	0.508805
BP_GO:0045672_positive+regulation+of+osteoclast+differentiation	0.359015	0.693705	0.154634
BP_GO:0045987_positive+regulation+of+smooth+muscle+contraction	0.462103	0.224871	0.732948
BP_GO:0060235_lens+induction+in+camera-type+eye	0.192834	0.304441	0.237243
BP_GO:0050828_regulation+of+liquid+surface+tension	0.226434	0.0915328	0.60636
BP_GO:0007292_female+gamete+generation	0.61835	0.576525	0.592345
BP_GO:0030259_lipid+glycosylation	0.296124	0.296124	#N/A
CC_GO:0005956_protein+kinase+CK2+complex	0.916525	0.979741	0.216739
BP_GO:0021853_cerebral+cortex+GABAergic+interneuron+migration	0.89823	0.89823	#N/A
MF_GO:0008301_DNA+bending+activity	0.472314	0.73426	0.234685
MF_GO:0008853_exodeoxyribonuclease+III+activity	0.837568	0.936527	0.250154
BP_GO:0021707_cerebellar+granule+cell+differentiation	0.210155	0.210155	#N/A
BP_GO:0032689_negative+regulation+of+interferon-gamma+production	0.750516	0.677716	0.710379
CC_GO:0045179_apical+cortex	0.767528	0.30847	0.990107
BP_GO:0021569_rhombomere+3+development	0.899319	0.96439	0.28479
MF_GO:0004985_opioid+receptor+activity	0.699337	0.75253	0.522845
BP_GO:0000289_nuclear-transcribed+mRNA+poly(A)+tail+shortening	0.892944	0.892944	#N/A
MF_GO:0004791_thioredoxin-disulfide+reductase+activity	0.830801	0.65804	0.828405
BP_GO:0006824_cobalt+ion+transport	0.516378	0.493783	0.529574
BP_GO:0021772_olfactory+bulb+development	0.555757	0.608462	0.469547
MF_GO:0035254_glutamate+receptor+binding	0.125001	0.242815	0.17725
BP_GO:0050953_sensory+perception+of+light+stimulus	0.540987	0.660597	0.30465
CC_GO:0001917_photoreceptor+inner+segment	0.0223373	0.0351198	0.189291
MF_GO:0016208_AMP+binding	0.0295182	0.00356644	0.507963
BP_GO:0048505_regulation+of+timing+of+cell+differentiation	0.0509946	0.0421343	0.278691
MF_GO:0015467_G-protein+activated+inward+rectifier+potassium+channel	0.659186	0.810654	0.240728
BP_GO:0001823_mesonephros+development	0.540388	0.480396	0.57658
BP_GO:0032332_positive+regulation+of+chondrocyte+differentiation	0.76441	0.955319	0.441357
BP_GO:0014051_gamma-aminobutyric+acid+secretion	0.420028	0.609023	0.188593
MF_GO:0030519_snoRNP+binding	0.330676	0.223691	0.473217
CC_GO:0043186_P+granule	0.401111	0.443158	0.416483
BP_GO:0051967_negative+regulation+of+synaptic+transmission,+glutamate	0.406897	0.370863	0.539981
BP_GO:0006432_phenylalanyl-tRNA+aminoacylation	0.772897	0.951537	0.462973
BP_GO:0008156_negative+regulation+of+DNA+replication	0.687904	0.767887	0.557218
CC_GO:0043159_acrosomal+matrix	0.772698	0.897568	0.552715
MF_GO:0017150_tRNA+dihydrouridine+synthase+activity	0.103286	0.533299	0.065962
BP_GO:0001661_conditioned+taste+aversion	0.87941	0.735284	0.839196
BP_GO:0033327_Leydig+cell+differentiation	0.0770277	0.113997	0.208796
BP_GO:0042177_negative+regulation+of+protein+catabolic+process	0.903229	0.903229	#N/A
MF_GO:0004052_arachidonate+12-lipoxygenase+activity	0.751695	0.810043	0.436288
BP_GO:0045862_positive+regulation+of+proteolysis	0.813678	0.705312	0.764901

BP_GO:0021879_forebrain+neuron+differentiation	0.494567	0.687134	0.383327
BP_GO:0050868_negative+regulation+of+T+cell+activation	0.528858	0.499239	0.533904
MF_GO:0004950_chemokine+receptor+activity	0.231331	#N/A	0.231331
MF_GO:0046920_alpha(1,3)-fucosyltransferase+activity	0.705425	0.815271	0.447152
CC_GO:0032045_guanyl-nucleotide+exchange+factor+complex	0.122025	0.0957224	0.366416
BP_GO:0000079_regulation+of+cyclin-dependent+protein+kinase+activity	0.7039	0.267918	0.835516
BP_GO:0014003_oligodendrocyte+development	0.654392	0.899289	0.237302
BP_GO:0043623_cellular+protein+complex+assembly	0.975782	0.937077	0.918718
CC_GO:0000801_central+element	0.659004	0.749997	0.462461
MF_GO:0045182_translation+regulator+activity	0.197362	0.197362	#N/A
BP_GO:0019216_regulation+of+lipid+metabolic+process	0.370064	0.208208	0.534328
MF_GO:0003945_N-acetyllactosamine+synthase+activity	0.251313	0.280783	0.368778
MF_GO:0042826_histone+deacetylase+binding	0.162487	0.160627	0.401104
BP_GO:0051930_regulation+of+sensory+perception+of+pain	0.533925	0.230952	0.925951
BP_GO:0022011_myelination+in+the+peripheral+nervous+system	0.839006	0.697267	0.811975
CC_GO:0035085_cilium+axoneme	0.8043	0.960161	0.0931954
CC_GO:0000120_RNA+polymerase+I+transcription+factor+complex	0.488811	0.242449	0.644657
BP_GO:0014832_urinary+bladder+smooth+muscle+contraction	0.764056	0.876645	0.285342
MF_GO:0001642_group+III+metabotropic+glutamate+receptor+activity	0.731877	0.631241	0.705559
MF_GO:0015016_[heparan+sulfate]-glucosamine+N-sulfotransferase+activit	0.519429	0.885754	0.128228
MF_GO:0008504_monoamine+transmembrane+transporter+activity	0.891548	0.960664	0.282501
CC_GO:0048500_signal+recognition+particle	0.252468	#N/A	0.252468
MF_GO:0008312_7S+RNA+binding	0.252468	#N/A	0.252468
BP_GO:0021797_forebrain+anterior/posterior+pattern+formation	0.463072	0.599891	0.26646
CC_GO:0032983_kainate+selective+glutamate+receptor+complex	0.529984	0.0771446	0.937177
MF_GO:0019104_DNA+N-glycosylase+activity	0.820785	0.869159	0.457609
MF_GO:0015279_store-operated+calcium+channel+activity	0.30572	0.302503	0.386693
CC_GO:0005736_DNA-directed+RNA+polymerase+I+complex	0.761277	0.6379	0.743056
CC_GO:0001652_granular+component	0.761977	0.896558	0.400006
CC_GO:0005773_vacuole	0.0983518	0.493214	0.0352217
BP_GO:0016973_poly(A)++mRNA+export+from+nucleus	0.996114	0.989947	0.903235
MF_GO:0004969_histamine+receptor+activity	0.493393	0.559893	0.431083
CC_GO:0033391_chromatoid+body	0.571811	0.679923	0.416483
BP_GO:0021979_hypothalamus+cell+differentiation	0.239386	0.290949	0.321747
BP_GO:0030252_growth+hormone+secretion	0.858283	0.894996	0.490067
BP_GO:0055012_ventricular+cardiac+muscle+cell+differentiation	0.579494	0.579494	#N/A
BP_GO:0050913_sensory+perception+of+bitter+taste	0.395609	0.483413	0.369775
BP_GO:0006465_signal+peptide+processing	0.0045417	#N/A	0.0045417
MF_GO:0016934_extracellular-glycine-gated+chloride+channel+activity	0.567226	0.43173	0.738151
BP_GO:0021542_dentate+gyrus+development	0.252148	0.735284	0.128472
BP_GO:0009303_rRNA+transcription	0.276072	0.570782	0.154089
MF_GO:0015057_thrombin+receptor+activity	0.740062	0.515295	0.889222
BP_GO:0006400_tRNA+modification	0.658167	0.63005	0.596784
BP_GO:0045604_regulation+of+epidermal+cell+differentiation	0.788975	0.679012	0.788711
MF_GO:0050327_testosterone+17-beta-dehydrogenase+activity	0.01416	#N/A	0.01416
MF_GO:0047961_glycine+N-acyltransferase+activity	0.371135	0.610919	0.166528
BP_GO:0019510_S-adenosylhomocysteine+catabolic+process	0.135685	0.135685	#N/A

BP_GO:0001960_negative+regulation+of+cytokine+mediated+signaling+path	0.423302	0.27826	0.690492
MF_GO:0004082_bisphosphoglycerate+mutase+activity	0.626225	0.904999	0.297516
MF_GO:0004083_bisphosphoglycerate+2-phosphatase+activity	0.626225	0.904999	0.297516
MF_GO:0004619_phosphoglycerate+mutase+activity	0.626225	0.904999	0.297516
BP_GO:0043056_forward+locomotion	0.775	0.515998	0.894933
BP_GO:0055002_striated+muscle+cell+development	0.775	0.515998	0.894933
MF_GO:0008034_lipoprotein+binding	0.227636	0.205371	0.448391
MF_GO:0005504_fatty+acid+binding	0.495744	0.495744	#N/A
BP_GO:0016125_sterol+metabolic+process	0.519069	0.519069	#N/A
CC_GO:0005945_6-phosphofructokinase+complex	0.83825	0.85375	0.58804
MF_GO:0003872_6-phosphofructokinase+activity	0.83825	0.85375	0.58804
BP_GO:0030193_regulation+of+blood+coagulation	0.921812	0.954194	0.706941
BP_GO:0015909_long-chain+fatty+acid+transport	0.842899	0.729212	0.810946
MF_GO:0004345_glucose-6-phosphate+dehydrogenase+activity	0.608311	0.608311	#N/A
MF_GO:0004090_carbonyl+reductase+(NADPH)+activity	0.499652	0.499652	#N/A
BP_GO:0001937_negative+regulation+of+endothelial+cell+proliferation	0.630983	0.523408	0.690492
BP_GO:0043496_regulation+of+protein+homodimerization+activity	0.308174	0.395077	0.335311
BP_GO:0006144_purine+base+metabolic+process	0.283	0.126869	0.732558
BP_GO:0001915_negative+regulation+of+T+cell+mediated+cytotoxicity	0.186451	0.186451	#N/A
MF_GO:0004332_fructose-bisphosphate+aldolase+activity	0.526438	0.526438	#N/A
BP_GO:0035025_positive+regulation+of+Rho+protein+signal+transduction	0.161514	0.275894	0.214864
BP_GO:0045777_positive+regulation+of+blood+pressure	0.916343	0.135729	0.993232
BP_GO:0005980_glycogen+catabolic+process	0.516246	0.50671	0.518905
MF_GO:0004944_C5a+anaphylatoxin+receptor+activity	0.60897	0.672493	0.509382
MF_GO:0003876_AMP+deaminase+activity	0.638666	0.911071	0.0984196
MF_GO:0004144_diacylglycerol+O-acyltransferase+activity	0.615321	0.888544	0.307898
CC_GO:0016234_inclusion+body	0.595504	0.344715	0.837687
BP_GO:0008154_actin+polymerization+or+depolymerization	0.688876	0.688876	#N/A
MF_GO:0004505_phenylalanine+4-monooxygenase+activity	0.0969035	0.0998195	0.246699
CC_GO:0032154_cleavage+furrow	0.794165	0.808851	0.651234
MF_GO:0004064_arylesterase+activity	0.707402	0.590997	0.732723
BP_GO:0050717_positive+regulation+of+interleukin-1+alpha+secretion	0.460411	0.827697	0.0656662
BP_GO:0006509_membrane+protein+ectodomain+proteolysis	0.914836	0.914836	#N/A
MF_GO:0008449_N-acetylglucosamine-6-sulfatase+activity	0.736613	0.736209	0.580783
MF_GO:0004473_malate+dehydrogenase+(oxaloacetate-decarboxylating)+(I	3.54325E-06	3.54325E-06	#N/A
BP_GO:0050732_negative+regulation+of+peptidyl-tyrosine+phosphorylation	0.494487	0.494487	#N/A
BP_GO:0030857_negative+regulation+of+epithelial+cell+differentiation	0.206409	0.0878228	0.690492
BP_GO:0002025_vasodilation+by+norepinephrine-epinephrine+involved+in+	0.2526	0.373877	0.242175
BP_GO:0031649_heat+generation	0.2526	0.373877	0.242175
MF_GO:0004065_arylsulfatase+activity	0.294942	0.167376	0.508721
BP_GO:0045600_positive+regulation+of+fat+cell+differentiation	0.948506	0.930256	0.829836
BP_GO:0043691_reverse+cholesterol+transport	0.0781064	0.0781064	#N/A
MF_GO:0016212_kynurenine-oxoglutarate+transaminase+activity	0.834765	0.917618	0.390442
BP_GO:0060325_face+morphogenesis	0.180974	#N/A	0.180974
BP_GO:0048251_elastic+fiber+assembly	0.995966	0.979392	0.955829
BP_GO:0006689_ganglioside+catabolic+process	0.62908	0.598335	0.586761
BP_GO:0014070_response+to+organic+cyclic+substance	0.53367	0.736735	0.226933

BP_GO:0018894_dibenzo-p-dioxin+metabolic+process	0.642022	0.464058	0.694957
MF_GO:0004594_pantothenate+kinase+activity	0.671152	0.671152	#N/A
MF_GO:0003985_acetyl-CoA+C-acetyltransferase+activity	0.042283	0.0270456	0.395967
MF_GO:0042578_phosphoric+ester+hydrolase+activity	0.82855	0.82855	#N/A
BP_GO:0006642_triacylglycerol+mobilization	0.975221	0.912924	0.930778
BP_GO:0006536_glutamate+metabolic+process	0.92175	0.6715	0.966247
BP_GO:0042359_vitamin+D+metabolic+process	0.116789	0.0208523	0.793093
BP_GO:0030029_actin+filament-based+process	0.701797	0.539373	0.781904
BP_GO:0045621_positive+regulation+of+lymphocyte+differentiation	0.308511	0.308511	#N/A
BP_GO:0006564_L-serine+biosynthetic+process	0.415042	0.415042	#N/A
BP_GO:0045103_intermediate+filament-based+process	0.453693	0.40475	0.555683
BP_GO:0015760_glucose-6-phosphate+transport	0.760144	0.670606	0.72603
CC_GO:0031362_anchored+to+external+side+of+plasma+membrane	0.44237	0.44237	#N/A
BP_GO:0001805_positive+regulation+of+type+III+hypersensitivity	0.367476	0.139731	0.636865
MF_GO:0008469_histone-arginine+N-methyltransferase+activity	0.652765	0.746925	0.397729
BP_GO:0009060_aerobic+respiration	0.208321	0.322279	0.225256
BP_GO:0046541_saliva+secretion	0.796514	0.523358	0.835191
BP_GO:0015697_quaternary+ammonium+group+transport	0.633783	0.572003	0.614472
MF_GO:0030247_polysaccharide+binding	0.419533	0.666419	0.168562
MF_GO:0003716_RNA+polymerase+I+transcription+termination+factor+acti	0.994441	0.994441	#N/A
BP_GO:0006103_2-oxoglutarate+metabolic+process	0.521406	0.504826	0.530501
BP_GO:0006549_isoleucine+metabolic+process	0.521406	0.504826	0.530501
BP_GO:0006600_creatine+metabolic+process	0.521406	0.504826	0.530501
BP_GO:0046449_creatinine+metabolic+process	0.521406	0.504826	0.530501
CC_GO:0043259_laminin-10+complex	0.405875	0.497735	0.343039
MF_GO:0004775_succinate-CoA+ligase+(ADP-forming)+activity	0.0839989	0.0241317	0.384914
BP_GO:0006868_glutamine+transport	0.859523	0.889329	0.555306
MF_GO:0015186_L-glutamine+transmembrane+transporter+activity	0.859523	0.889329	0.555306
BP_GO:0045907_positive+regulation+of+vasoconstriction	0.687014	0.597189	0.690492
BP_GO:0008228_opsonization	0.912991	0.912991	#N/A
MF_GO:0030676_Rac+guanyl-nucleotide+exchange+factor+activity	0.137236	0.215741	0.217528
CC_GO:0060053_neurofilament+cytoskeleton	0.483883	0.777355	0.125198
MF_GO:0047238_glucuronosyl-N-acetylgalactosaminyl-proteoglycan+4-beta	0.0383817	0.0303869	0.199794
MF_GO:0050510_N-acetylgalactosaminyl-proteoglycan+3-beta-glucuronosyl	0.0383817	0.0303869	0.199794
MF_GO:0004792_thiosulfate+sulfurtransferase+activity	0.281068	0.281068	#N/A
BP_GO:0045663_positive+regulation+of+myoblast+differentiation	0.159588	0.159588	#N/A
MF_GO:0004165_dodecenoyl-CoA+delta-isomerase+activity	0.0288983	0.0670808	0.121289
BP_GO:0008215_spermine+metabolic+process	0.439411	0.777483	0.0894403
BP_GO:0043392_negative+regulation+of+DNA+binding	0.495727	0.494731	0.498658
BP_GO:0031397_negative+regulation+of+protein+ubiquitination	0.504368	0.504368	#N/A
MF_GO:0004859_phospholipase+inhibitor+activity	0.331235	0.471987	0.313961
BP_GO:0060157_urinary+bladder+development	0.424043	0.424043	#N/A
BP_GO:0043374_CD8-positive,+alpha-beta+T+cell+differentiation	0.0463256	0.0133832	0.3106
BP_GO:0043393_regulation+of+protein+binding	0.0850364	0.126261	0.191715
BP_GO:0060068_vagina+development	0.460574	0.549481	0.417328
MF_GO:0008475_procollagen-lysine+5-dioxygenase+activity	0.739432	0.739432	#N/A
MF_GO:0016884_carbon-nitrogen+ligase+activity,+with+glutamine+as+amic	0.799711	0.676664	0.79075

MF_GO:0004955_prostaglandin+receptor+activity	0.577992	0.85193	0.309612
BP_GO:0002281_macrophage+activation+during+immune+response	0.569737	0.446484	0.689882
BP_GO:0002238_response+to+molecule+of+fungal+origin	0.427172	#N/A	0.427172
CC_GO:0005640_nuclear+outer+membrane	0.0680967	#N/A	0.0680967
MF_GO:0004117_calmodulin-dependent+cyclic-nucleotide+phosphodiestera	0.829973	0.837834	0.602487
MF_GO:0048101_calcium-+and+calmodulin-regulated+3',5'-cyclic-GMP+pho	0.829973	0.837834	0.602487
BP_GO:0030953_spindle+astral+microtubule+organization	0.969539	0.969539	#N/A
BP_GO:0050820_positive+regulation+of+coagulation	0.0588007	0.302446	0.0604468
BP_GO:0001955_blood+vessel+maturation	0.163412	0.242449	0.239739
BP_GO:0060087_relaxation+of+vascular+smooth+muscle	0.487171	0.523358	0.467965
BP_GO:0042133_neurotransmitter+metabolic+process	0.619745	0.84251	0.185993
BP_GO:0042246_tissue+regeneration	0.837129	0.909248	0.42603
BP_GO:0008347_glial+cell+migration	0.406046	0.656822	0.162858
BP_GO:0003016_respiratory+system+process	0.650516	0.48926	0.689065
BP_GO:0045926_negative+regulation+of+growth	0.950263	0.992422	0.280921
BP_GO:0003014_renal+system+process	0.117816	0.0809794	0.321314
BP_GO:0006725_cellular+aromatic+compound+metabolic+process	0.312985	0.401613	0.337035
BP_GO:0007182_common-partner+SMAD+protein+phosphorylation	0.235382	0.235382	#N/A
MF_GO:0001786_phosphatidylserine+binding	0.458357	0.432026	0.524556
MF_GO:0033829_O-fucosylpeptide+3-beta-N-acetylglucosaminyltransferase	0.849577	0.68393	0.867914
BP_GO:0050819_negative+regulation+of+coagulation	0.0940424	0.0459047	0.337061
BP_GO:0006651_diacylglycerol+biosynthetic+process	0.598226	0.789572	0.395985
MF_GO:0000036_acyl+carrier+activity	0.217281	0.0211634	0.683721
BP_GO:0050665_hydrogen+peroxide+biosynthetic+process	0.528493	#N/A	0.528493
MF_GO:0004937_alpha1-adrenergic+receptor+activity	0.064305	0.064305	#N/A
BP_GO:0048007_antigen+processing+and+presentation,+exogenous+lipid+a	0.976184	0.976184	#N/A
CC_GO:0000015_phosphopyruvate+hydratase+complex	0.812422	0.897822	0.398201
MF_GO:0004634_phosphopyruvate+hydratase+activity	0.812422	0.897822	0.398201
MF_GO:0008559_xenobiotic-transporting+ATPase+activity	0.79477	0.778483	0.633802
BP_GO:0035051_cardiac+cell+differentiation	0.145365	0.145365	#N/A
MF_GO:0030169_low-density+lipoprotein+binding	0.874504	0.874504	#N/A
BP_GO:0043267_negative+regulation+of+potassium+ion+transport	0.761678	0.632716	0.774721
BP_GO:0045986_negative+regulation+of+smooth+muscle+contraction	0.424417	0.499714	0.371168
BP_GO:0019941_modification-dependent+protein+catabolic+process	0.0640665	0.159694	0.109757
BP_GO:0002377_immunoglobulin+production	0.534796	0.351997	0.754753
BP_GO:0007097_nuclear+migration	0.202134	0.202134	#N/A
BP_GO:0051707_response+to+other+organism	0.624814	0.624814	#N/A
BP_GO:0002481_antigen+processing+and+presentation+of+exogenous+prot	0.311189	0.279405	0.489677
MF_GO:0016763_transferase+activity,+transferring+pentosyl+groups	0.458164	0.458164	#N/A
MF_GO:0001872_zymosan+binding	0.542228	0.790673	0.168562
BP_GO:0019432_triacylglycerol+biosynthetic+process	0.377114	0.789572	0.170541
BP_GO:0009410_response+to+xenobiotic+stimulus	0.584266	0.416765	0.658984
BP_GO:0002682_regulation+of+immune+system+process	0.00262136	0.149109	0.00363311
BP_GO:0006926_virus-infected+cell+apoptosis	0.919553	0.825287	0.865834
BP_GO:0016458_gene+silencing	0.883617	0.883617	#N/A
BP_GO:0001820_serotonin+secretion	0.956894	0.975253	0.76237
BP_GO:0009605_response+to+external+stimulus	0.166175	0.333078	0.188829

MF_GO:0017160_Ral+GTPase+binding	0.0440947	0.0440947	#N/A
MF_GO:0005375_copper+ion+transmembrane+transporter+activity	0.624096	0.660226	0.485766
MF_GO:0004311_farnesyltranstransferase+activity	0.821824	0.821824	#N/A
MF_GO:0030506_ankyrin+binding	0.829648	0.423651	0.972853
MF_GO:0003987_acetate-CoA+ligase+activity	0.00362266	0.000535771	0.489683
BP_GO:0006519_cellular+amino+acid+and+derivative+metabolic+process	0.183731	0.352456	0.152514
MF_GO:0046875_ephrin+receptor+binding	0.230537	0.249437	0.335573
BP_GO:0019987_negative+regulation+of+anti-apoptosis	0.149902	0.556595	0.0852807
CC_GO:0005955_calcineurin+complex	0.407167	0.746571	0.0892257
BP_GO:0048935_peripheral+nervous+system+neuron+development	0.779662	0.747272	0.653527
MF_GO:0004517_nitric-oxide+synthase+activity	0.458816	0.458816	#N/A
BP_GO:0045404_positive+regulation+of+interleukin-4+biosynthetic+process	0.288673	0.578872	0.205277
MF_GO:0042301_phosphate+binding	0.359519	0.492686	0.334569
BP_GO:0002573_myeloid+leukocyte+differentiation	0.13377	0.307476	0.113239
BP_GO:0010043_response+to+zinc+ion	0.802839	0.802839	#N/A
BP_GO:0030104_water+homeostasis	0.109869	0.0443888	0.382034
BP_GO:0032436_positive+regulation+of+proteasomal+ubiquitin-dependent+	0.665444	0.665444	#N/A
CC_GO:0005774_vacuolar+membrane	0.450909	0.450909	#N/A
BP_GO:0006085_acetyl-CoA+biosynthetic+process	0.00061245	4.04089E-05	0.489683
BP_GO:0007184_SMAD+protein+nuclear+translocation	0.082964	0.082964	#N/A
MF_GO:0016309_1-phosphatidylinositol-5-phosphate+4-kinase+activity	0.637885	0.933238	0.265071
MF_GO:0004862_cAMP-dependent+protein+kinase+inhibitor+activity	0.975616	0.818275	0.983431
BP_GO:0050869_negative+regulation+of+B+cell+activation	0.349145	0.188539	0.559559
BP_GO:0006582_melanin+metabolic+process	0.199199	0.342549	0.227376
MF_GO:0016972_thiol+oxidase+activity	0.645057	0.748461	0.381303
BP_GO:0019642_anaerobic+glycolysis	0.00395934	0.00907415	0.103928
MF_GO:0004459_L-lactate+dehydrogenase+activity	0.00395934	0.00907415	0.103928
CC_GO:0005790_smooth+endoplasmic+reticulum	0.055609	0.134081	0.116354
MF_GO:0004667_prostaglandin-D+synthase+activity	0.673715	0.269726	0.837849
BP_GO:0014068_positive+regulation+of+phosphoinositide+3-kinase+cascad	0.736131	0.363479	0.943985
MF_GO:0042166_acetylcholine+binding	0.339638	0.903234	0.0770714
BP_GO:0006527_arginine+catabolic+process	0.287791	0.41706	0.25032
BP_GO:0030323_respiratory+tube+development	0.420665	0.195831	0.806639
MF_GO:0004528_phosphodiesterase+I+activity	0.26379	0.351996	0.288841
BP_GO:0046685_response+to+arsenic	0.0407197	0.0407197	#N/A
CC_GO:0005779_integral+to+peroxisomal+membrane	0.153721	0.08248	0.394175
BP_GO:0035088_establishment+or+maintenance+of+apical/basal+cell+polar	0.286578	0.25657	0.47978
BP_GO:0030321_transepithelial+chloride+transport	0.98431	0.995228	0.52545
BP_GO:0046339_diacylglycerol+metabolic+process	0.0553722	0.114844	0.134706
MF_GO:0004370_glycerol+kinase+activity	0.0461947	0.0864715	0.161521
BP_GO:0060044_negative+regulation+of+cardiac+muscle+cell+proliferation	0.221218	0.160073	0.530237
BP_GO:0009263_deoxyribonucleotide+biosynthetic+process	0.463221	0.0888931	0.959702
MF_GO:0004748_ribonucleoside-diphosphate+reductase+activity	0.463221	0.0888931	0.959702
BP_GO:0051450_myoblast+proliferation	0.515605	0.362098	0.617053
MF_GO:0008384_IkappaB+kinase+activity	0.915837	0.975021	0.350049
BP_GO:0010273_detoxification+of+copper+ion	0.00371266	0.904917	4.24092E-05
MF_GO:0016671_oxidoreductase+activity,+acting+on+sulfur+group+of+don	0.511696	0.520432	0.491593

BP_GO:0050872_white+fat+cell+differentiation	0.996996	0.995402	0.85931
BP_GO:0048340_paraxial+mesoderm+morphogenesis	0.95107	0.95107	#N/A
MF_GO:0003988_acetyl-CoA+C-acyltransferase+activity	0.80312	0.80312	#N/A
BP_GO:0006620_posttranslational+protein+targeting+to+membrane	0.208715	0.262251	0.306968
CC_GO:0005782_peroxisomal+matrix	0.393941	0.393941	#N/A
MF_GO:0003865_3-oxo-5-alpha-steroid+4-dehydrogenase+activity	0.952301	0.952301	#N/A
MF_GO:0047184_1-acylglycerophosphocholine+O-acyltransferase+activity	0.654569	0.636238	0.578062
BP_GO:0002862_negative+regulation+of+inflammatory+response+to+antige	0.33676	0.0673196	0.705977
MF_GO:0016986_transcription+initiation+factor+activity	0.872819	0.697224	0.893537
BP_GO:0032400_melanosome+localization	0.170661	0.0802588	0.631859
CC_GO:0000940_outer+kinetochore+of+condensed+chromosome	0.876916	0.895451	0.592343
BP_GO:0019724_B+cell+mediated+immunity	0.891879	0.507021	0.933565
BP_GO:0051891_positive+regulation+of+cardioblast+differentiation	0.695681	0.64407	0.642626
BP_GO:0046325_negative+regulation+of+glucose+import	0.622096	0.221438	0.947952
BP_GO:0045662_negative+regulation+of+myoblast+differentiation	0.374305	0.615022	0.274631
MF_GO:0015238_drug+transporter+activity	0.545731	0.545731	#N/A
BP_GO:0048545_response+to+steroid+hormone+stimulus	0.341445	0.673992	0.0892257
MF_GO:0019797_procollagen-proline+3-dioxygenase+activity	0.127071	0.127071	#N/A
BP_GO:0006560_proline+metabolic+process	0.918965	0.918965	#N/A
MF_GO:0004700_atypical+protein+kinase+C+activity	0.273231	0.273231	#N/A
MF_GO:0004346_glucose-6-phosphatase+activity	0.592978	0.592978	#N/A
BP_GO:0045475_locomotor+rhythm	0.109993	0.291635	0.0886392
BP_GO:0051496_positive+regulation+of+stress+fiber+formation	0.0949619	0.175711	0.171872
BP_GO:0031558_induction+of+apoptosis+in+response+to+chemical+stimulu	0.503055	0.503055	#N/A
BP_GO:0021860_pyramidal+neuron+development	0.382584	0.711548	0.0956909
BP_GO:0006972_hyperosmotic+response	0.415897	0.576254	0.261198
BP_GO:0018401_peptidyl-proline+hydroxylation+to+4-hydroxy-L-proline	0.528375	0.528375	#N/A
MF_GO:0004663_Rab-protein+geranylgeranyltransferase+activity	0.0637073	0.0635165	0.31467
MF_GO:0004069_aspartate+transaminase+activity	0.656916	0.553017	0.695803
MF_GO:0008898_homocysteine+S-methyltransferase+activity	0.154028	0.154028	#N/A
MF_GO:0016866_intramolecular+transferase+activity	0.371861	0.359787	0.476839
MF_GO:0016286_small+conductance+calcium-activated+potassium+channe	0.0250756	0.0250756	#N/A
CC_GO:0005747_mitochondrial+respiratory+chain+complex+I	0.753747	0.626567	0.730091
MF_GO:0019976_interleukin-2+binding	0.957323	0.957323	#N/A
BP_GO:0009100_glycoprotein+metabolic+process	0.551213	0.469079	0.630618
BP_GO:0010575_positive+regulation+vascular+endothelial+growth+factor+p	0.0300536	0.00521807	0.642626
BP_GO:0006686_sphingomyelin+biosynthetic+process	0.713679	0.875015	0.258098
BP_GO:0043306_positive+regulation+of+mast+cell+degranulation	0.781412	0.975253	0.331386
BP_GO:0030049_muscle+filament+sliding	0.711434	#N/A	0.711434
CC_GO:0016461_unconventional+myosin+complex	0.711434	#N/A	0.711434
BP_GO:0007195_dopamine+receptor,+adenylate+cyclase+inhibiting+pathwa	0.558163	0.722788	0.280224
BP_GO:0001996_positive+regulation+of+heart+rate+by+epinephrine-norepi	0.162106	0.237916	0.242175
BP_GO:0001997_positive+regulation+of+the+force+of+heart+contraction+b	0.162106	0.237916	0.242175
MF_GO:0016155_formyltetrahydrofolate+dehydrogenase+activity	0.536839	0.536839	#N/A
BP_GO:0016559_peroxisome+fission	0.567889	0.29232	0.857775
BP_GO:0000320_re-entry+into+mitotic+cell+cycle	0.910003	0.910003	#N/A
MF_GO:0045502_dynein+binding	0.215406	0.155394	0.401948

BP_GO:0007213_acetylcholine+receptor+signaling,+muscarinic+pathway	0.337738	0.337738	#N/A
BP_GO:0016117_carotenoid+biosynthetic+process	0.0313919	0.0313919	#N/A
MF_GO:0019209_kinase+activator+activity	0.828054	0.47339	0.88631
BP_GO:0045216_cell-cell+junction+organization	0.195169	0.321128	0.202974
BP_GO:0001844_protein+insertion+into+mitochondrial+membrane+during+	0.353009	0.262486	0.495061
BP_GO:0007095_mitotic+cell+cycle+G2/M+transition+DNA+damage+checkp	0.709236	0.83216	0.34229
BP_GO:0043068_positive+regulation+of+programmed+cell+death	0.085879	0.085879	#N/A
BP_GO:0045617_negative+regulation+of+keratinocyte+differentiation	0.608281	0.608281	#N/A
MF_GO:0017076_purine+nucleotide+binding	0.179345	0.179345	#N/A
BP_GO:0019884_antigen+processing+and+presentation+of+exogenous+anti	0.463849	0.0540389	0.982863
BP_GO:0035234_germ+cell+programmed+cell+death	0.748899	0.720796	0.631452
BP_GO:0007016_cytoskeletal+anchoring+at+plasma+membrane	0.477463	0.173671	0.724208
BP_GO:0045022_early+endosome+to+late+endosome+transport	0.141894	0.145055	0.359243
BP_GO:0032469_endoplasmic+reticulum+calcium+ion+homeostasis	0.101927	0.638229	0.0354418
MF_GO:0051020_GTPase+binding	0.273404	0.252657	0.45966
BP_GO:0048268_clathrin+coat+assembly	0.897041	0.97648	0.558173
MF_GO:0008502_melatonin+receptor+activity	0.44694	0.520833	0.420771
MF_GO:0004712_protein+serine/threonine/tyrosine+kinase+activity	0.583134	0.409474	0.754368
BP_GO:0001553_luteinization	0.176052	0.176052	#N/A
MF_GO:0019208_phosphatase+regulator+activity	0.877977	0.753841	0.852747
BP_GO:0007090_regulation+of+S+phase+of+mitotic+cell+cycle	0.878037	0.768387	0.818443
MF_GO:0030371_translation+repressor+activity	0.4439	0.161271	0.700822
BP_GO:0006929_substrate-bound+cell+migration	0.932116	0.999169	0.346471
MF_GO:0032947_protein+complex+scaffold	0.300188	0.300188	#N/A
MF_GO:0016151_nickel+ion+binding	0.128707	#N/A	0.128707
CC_GO:0042598_vesicular+fraction	0.608116	0.979103	0.135205
BP_GO:0007034_vacuolar+transport	0.0994034	0.0994034	#N/A
CC_GO:0030904_retromer+complex	0.0994034	0.0994034	#N/A
MF_GO:0003896_DNA+primase+activity	0.82787	0.497245	0.950496
BP_GO:0001812_positive+regulation+of+type+I+hypersensitivity	0.922009	0.975253	0.636865
CC_GO:0030118_clathrin+coat	0.896525	0.976592	0.266095
MF_GO:0000293_ferric-chelate+reductase+activity	0.66493	0.269617	0.830513
CC_GO:0001891_phagocytic+cup	0.30631	0.320384	0.414172
BP_GO:0048488_synaptic+vesicle+endocytosis	0.679174	0.79108	0.367296
BP_GO:0009448_gamma-aminobutyric+acid+metabolic+process	0.215043	0.27074	0.307422
MF_GO:0016817_hydrolase+activity,+acting+on+acid+anhydrides	0.683615	0.810957	0.337876
BP_GO:0001675_acrosome+formation	0.662417	0.820648	0.283784
BP_GO:0042989_sequestering+of+actin+monomers	0.0125674	0.143074	0.0233487
BP_GO:0009181_purine+ribonucleoside+diphosphate+catabolic+process	0.699625	0.848835	0.290584
MF_GO:0008454_alpha-1,3-mannosylglycoprotein+4-beta-N-acetylglucosam	0.917181	0.165914	0.991453
BP_GO:0030502_negative+regulation+of+bone+mineralization	0.209557	#N/A	0.209557
MF_GO:0000309_nicotinamide-nucleotide+adenylyltransferase+activity	0.715898	0.633506	0.693958
MF_GO:0019871_sodium+channel+inhibitor+activity	0.881346	0.881346	#N/A
MF_GO:0004597_peptide-aspartate+beta-dioxygenase+activity	0.587387	0.64531	0.442774
BP_GO:0030510_regulation+of+BMP+signaling+pathway	0.797465	0.814961	0.650585
BP_GO:0002320_lymphoid+progenitor+cell+differentiation	0.116705	0.423166	0.093015
BP_GO:0008090_retrograde+axon+cargo+transport	0.836957	0.836957	#N/A

BP_GO:0033209_tumor+necrosis+factor-mediated+signaling+pathway	0.394426	0.652321	0.154427
BP_GO:0051090_regulation+of+transcription+factor+activity	0.758297	0.491481	0.893537
MF_GO:0005094_Rho+GDP-dissociation+inhibitor+activity	0.53479	#N/A	0.53479
BP_GO:0060009_Sertoli+cell+development	0.267949	0.17744	0.458571
BP_GO:0030518_steroid+hormone+receptor+signaling+pathway	0.529036	0.529036	#N/A
MF_GO:0004849_uridine+kinase+activity	0.437822	0.313215	0.560699
MF_GO:0004558_alpha-glucosidase+activity	0.936151	0.936151	#N/A
MF_GO:0001882_nucleoside+binding	0.221294	0.221294	#N/A
BP_GO:0033143_regulation+of+steroid+hormone+receptor+signaling+pathv	0.630419	#N/A	0.630419
CC_GO:0030136_clathrin-coated+vesicle	0.717229	0.700981	0.629959
BP_GO:0045055_regulated+secretory+pathway	0.730883	0.857848	0.327269
MF_GO:0043560_insulin+receptor+substrate+binding	0.504162	0.668686	0.274719
BP_GO:0030091_protein+repair	0.295116	0.172199	0.503591
MF_GO:0000318_protein-methionine-R-oxide+reductase+activity	0.295116	0.172199	0.503591
BP_GO:0001953_negative+regulation+of+cell-matrix+adhesion	0.0290854	0.488703	0.0107145
BP_GO:0030224_monocyte+differentiation	0.748715	0.748715	#N/A
BP_GO:0006670_sphingosine+metabolic+process	0.452751	#N/A	0.452751
MF_GO:0050508_glucuronosyl-N-acetylglucosaminyl-proteoglycan+4-alpha-	0.501741	0.501741	#N/A
MF_GO:0042799_histone+lysine+N-methyltransferase+activity+(H4-K20+spe	0.505599	0.314463	0.760724
MF_GO:0004385_guanylate+kinase+activity	0.174415	0.301792	0.187113
BP_GO:0040012_regulation+of+locomotion	0.164751	#N/A	0.164751
BP_GO:0002636_positive+regulation+of+germinal+center+formation	0.581508	0.354653	0.811832
BP_GO:0001842_neural+fold+formation	0.252053	#N/A	0.252053
MF_GO:0015027_coreceptor,+soluble+ligand+activity	0.141618	0.182244	0.250403
BP_GO:0032147_activation+of+protein+kinase+activity	0.764382	0.730951	0.672881
MF_GO:0016505_apoptotic+protease+activator+activity	0.511424	0.464315	0.570232
MF_GO:0003726_double-stranded+RNA+adenosine+deaminase+activity	0.746308	0.546765	0.837292
BP_GO:0018125_peptidyl-cysteine+methylation	0.863884	0.741106	0.838564
MF_GO:0005548_phospholipid+transporter+activity	0.302326	0.514607	0.254721
BP_GO:0006855_multidrug+transport	0.480875	0.501552	0.464927
MF_GO:0047631_ADP-ribose+diphosphatase+activity	0.181543	0.180833	0.387743
MF_GO:0004322_ferroxidase+activity	0.430796	0.0972662	0.759203
BP_GO:0060056_mammary+gland+involution	0.298329	0.25239	0.429959
MF_GO:0008409_5'-3'+exonuclease+activity	0.197208	#N/A	0.197208
BP_GO:0048820_hair+follicle+maturation	0.637237	0.515009	0.710854
BP_GO:0044249_cellular+biosynthetic+process	0.400897	0.485839	0.350389
BP_GO:0006542_glutamine+biosynthetic+process	0.423014	0.476086	0.400858
MF_GO:0004356_glutamate-ammonia+ligase+activity	0.423014	0.476086	0.400858
MF_GO:0004430_1-phosphatidylinositol+4-kinase+activity	0.613977	0.613977	#N/A
BP_GO:0007089_traversing+start+control+point+of+mitotic+cell+cycle	0.924535	0.924535	#N/A
BP_GO:0007252_I-kappaB+phosphorylation	0.535211	0.908304	0.202815
BP_GO:0016584_nucleosome+positioning	0.22657	0.358445	0.253884
MF_GO:0005031_tumor+necrosis+factor+receptor+activity	0.345046	0.641937	0.228116
CC_GO:0031512_motile+primary+cilium	0.817087	0.94534	0.242745
MF_GO:0035197_siRNA+binding	0.948981	0.948981	#N/A
BP_GO:0007144_female+meiosis+I	0.0378049	0.249674	0.0168659
MF_GO:0016149_translation+release+factor+activity,+codon+specific	0.256321	0.256321	#N/A

BP_GO:0006808_regulation+of+nitrogen+utilization	0.22596	#N/A	0.22596
BP_GO:0006290_pyrimidine+dimer+repair	0.461329	0.724019	0.156424
BP_GO:0044265_cellular+macromolecule+catabolic+process	0.669751	0.790585	0.351253
BP_GO:0030513_positive+regulation+of+BMP+signaling+pathway	0.991286	0.991286	#N/A
MF_GO:0008173_RNA+methyltransferase+activity	0.357548	#N/A	0.357548
BP_GO:0006891_intra-Golgi+vesicle-mediated+transport	0.948186	0.957206	0.651439
MF_GO:0005528_FK506+binding	0.778198	#N/A	0.778198
BP_GO:0048302_regulation+of+isotype+switching+to+IgG+isotypes	0.0796024	0.339907	0.0317619
BP_GO:0001757_somite+specification	0.475171	0.475171	#N/A
BP_GO:0006166_purine+ribonucleoside+salvage	0.365852	0.300413	0.558273
MF_GO:0003886_DNA+(cytosine-5-)-methyltransferase+activity	0.867291	0.923889	0.462252
MF_GO:0005229_intracellular+calcium+activated+chloride+channel+activity	0.997277	0.675755	0.998976
BP_GO:0040037_negative+regulation+of+fibroblast+growth+factor+recepto	0.467417	0.649318	0.247085
BP_GO:0006000_fructose+metabolic+process	0.167634	0.266304	0.215753
BP_GO:0006003_fructose+2,6-bisphosphate+metabolic+process	0.167634	0.266304	0.215753
MF_GO:0003873_6-phosphofructo-2-kinase+activity	0.167634	0.266304	0.215753
MF_GO:0004331_fructose-2,6-bisphosphate+2-phosphatase+activity	0.167634	0.266304	0.215753
BP_GO:0046549_retinal+cone+cell+development	0.470966	0.470966	#N/A
MF_GO:0004965_GABA-B+receptor+activity	0.0786119	0.0524572	0.437649
BP_GO:0051155_positive+regulation+of+striated+muscle+cell+differentiatio	0.808256	0.883959	0.428721
MF_GO:0048038_quinone+binding	0.527072	0.836247	0.27145
MF_GO:0008568_microtubule-severing+ATPase+activity	0.203937	0.116879	0.599004
BP_GO:0031000_response+to+caffeine	0.930429	0.953098	0.57664
BP_GO:0001994_norepinephrine-epinephrine+vasoconstriction+involved+in	0.076082	0.076082	#N/A
BP_GO:0043367_CD4-positive,+alpha+beta+T+cell+differentiation	0.559178	0.83092	0.3106
MF_GO:0003918_DNA+topoisomerase+(ATP-hydrolyzing)+activity	0.620449	0.570334	0.610791
BP_GO:0034101_erythrocyte+homeostasis	0.23318	0.161271	0.423578
BP_GO:0021522_spinal+cord+motor+neuron+differentiation	0.396367	0.798592	0.0506576
BP_GO:0050806_positive+regulation+of+synaptic+transmission	0.75477	0.913401	0.232332
BP_GO:0048664_neuron+fate+determination	0.474327	0.474327	#N/A
MF_GO:0005220_inositol+1,4,5-triphosphate-sensitive+calcium-release+cha	0.33714	0.278854	0.540457
MF_GO:0008095_inositol-1,4,5-triphosphate+receptor+activity	0.33714	0.278854	0.540457
BP_GO:0045446_endothelial+cell+differentiation	0.120367	0.648885	0.0439128
MF_GO:0016891_endoribonuclease+activity,+producing+5'-phosphomonoes	0.845506	0.845506	#N/A
CC_GO:0008537_proteasome+activator+complex	0.701423	0.116734	0.931886
MF_GO:0008538_proteasome+activator+activity	0.701423	0.116734	0.931886
MF_GO:0004616_phosphogluconate+dehydrogenase+(decarboxylating)+act	0.495538	0.467614	0.538318
BP_GO:0030643_cellular+phosphate+ion+homeostasis	0.157127	#N/A	0.157127
BP_GO:0007603_phototransduction,+visible+light	0.325253	0.728211	0.162578
BP_GO:0006491_N-glycan+processing	0.999041	0.999041	#N/A
BP_GO:0048294_negative+regulation+of+isotype+switching+to+IgE+isotype:	0.00383372	0.456422	0.000714557
BP_GO:0042698_ovulation+cycle	0.92014	0.736253	0.899077
MF_GO:0004802_transketolase+activity	0.264857	0.181414	0.578801
BP_GO:0032402_melanosome+transport	0.499024	0.404929	0.631859
BP_GO:0009607_response+to+biotic+stimulus	0.50011	0.0697139	0.852067
MF_GO:0008073_ornithine+decarboxylase+inhibitor+activity	0.1331	0.199706	0.231377
BP_GO:0006435_threonyl-tRNA+aminoacylation	0.725346	0.640391	0.701889

MF_GO:0004829_threonine-tRNA+ligase+activity	0.725346	0.640391	0.701889
BP_GO:0040023_establishment+of+nucleus+localization	0.730506	0.682935	0.652498
BP_GO:0046903_secretion	0.252637	0.153817	0.462288
BP_GO:0007220_Notch+receptor+processing	0.153684	#N/A	0.153684
MF_GO:0003953_NAD++nucleosidase+activity	0.457858	#N/A	0.457858
BP_GO:0009612_response+to+mechanical+stimulus	0.991595	0.989409	0.811587
BP_GO:0031116_positive+regulation+of+microtubule+polymerization	0.111754	0.111754	#N/A
CC_GO:0042405_nuclear+inclusion+body	0.94616	0.94616	#N/A
BP_GO:0007412_axon+target+recognition	0.957272	0.957272	#N/A
CC_GO:0017133_mitochondrial+electron+transfer+flavoprotein+complex	0.655313	0.695787	0.487453
MF_GO:0004800_thyroxine+5'-deiodinase+activity	0.148096	#N/A	0.148096
MF_GO:0004758_serine+C-palmitoyltransferase+activity	0.0874446	0.405496	0.0220822
MF_GO:0004566_beta-glucuronidase+activity	0.522394	0.323154	0.653051
BP_GO:0021895_cerebral+cortex+neuron+differentiation	0.902875	0.931503	0.558273
BP_GO:0007215_glutamate+signaling+pathway	0.98028	0.993624	0.518559
BP_GO:0045747_positive+regulation+of+Notch+signaling+pathway	0.152517	0.585326	0.0794965
BP_GO:0045629_negative+regulation+of+T-helper+2+cell+differentiation	0.0272519	#N/A	0.0272519
BP_GO:0015695_organic+cation+transport	0.492765	#N/A	0.492765
MF_GO:0003724_RNA+helicase+activity	0.971022	0.900827	0.928824
BP_GO:0045217_cell-cell+junction+maintenance	0.303449	0.293666	0.450919
BP_GO:0007000_nucleolus+organization	0.663748	0.663748	#N/A
CC_GO:0000109_nucleotide-excision+repair+complex	0.174558	0.198005	0.336729
BP_GO:0030644_cellular+chloride+ion+homeostasis	0.805546	0.910978	0.340342
CC_GO:0032421_stereocilium+bundle	0.623361	0.912996	0.282527
CC_GO:0000802_transverse+filament	0.545758	0.651539	0.446688
BP_GO:0050779_RNA+destabilization	0.907849	0.891159	0.711456
BP_GO:0045884_regulation+of+survival+gene+product+expression	0.294198	0.174697	0.650464
BP_GO:0048676_axon+extension+involved+in+development	0.497391	0.321103	0.741062
BP_GO:0031623_receptor+internalization	0.345924	0.345924	#N/A
CC_GO:0030285_integral+to+synaptic+vesicle+membrane	0.244669	0.826041	0.0655268
BP_GO:0035284_brain+segmentation	0.698609	0.931105	0.340548
CC_GO:0001741_XY+body	0.383055	0.383055	#N/A
BP_GO:0007199_G-protein+signaling,+coupled+to+cGMP+nucleotide+secon	0.0583999	0.0583999	#N/A
BP_GO:0010025_wax+biosynthetic+process	0.98859	0.978995	0.85754
BP_GO:0048678_response+to+axon+injury	0.219034	#N/A	0.219034
BP_GO:0030521_androgen+receptor+signaling+pathway	0.364476	#N/A	0.364476
MF_GO:0004705_JUN+kinase+activity	0.628206	0.738938	0.367665
BP_GO:0051493_regulation+of+cytoskeleton+organization	0.36589	0.148792	0.810637
BP_GO:0021575_hindbrain+morphogenesis	0.274533	0.274533	#N/A
BP_GO:0000087_M+phase+of+mitotic+cell+cycle	0.463654	0.463654	#N/A
MF_GO:0005104_fibroblast+growth+factor+receptor+binding	0.701146	0.935084	0.109741
BP_GO:0042326_negative+regulation+of+phosphorylation	0.448029	0.448029	#N/A
BP_GO:0048513_organ+development	0.389118	0.613502	0.291689
MF_GO:0008081_phosphoric+diester+hydrolase+activity	0.933489	0.948759	0.615672
BP_GO:0007256_activation+of+JNKK+activity	0.798747	0.631638	0.835458
MF_GO:0050811_GABA+receptor+binding	0.586461	0.586461	#N/A
BP_GO:0006467_protein+thiol-disulfide+exchange	0.907085	0.949091	0.491593

CC_GO:0031965_nuclear+membrane	0.519178	0.843835	0.0892257
MF_GO:0004687_myosin+light+chain+kinase+activity	0.356115	0.352007	0.459682
BP_GO:0021571_rhombomere+5+development	0.376789	0.176212	0.607606
BP_GO:0006420_arginyl-tRNA+aminoacylation	0.444822	#N/A	0.444822
MF_GO:0004814_arginine-tRNA+ligase+activity	0.444822	#N/A	0.444822
BP_GO:0031529_ruffle+organization	0.117406	0.157949	0.261198
BP_GO:0051298_centrosome+duplication	0.417533	0.894918	0.127073
BP_GO:0006264_mitochondrial+DNA+replication	0.224459	0.0791443	0.753095
MF_GO:0005172_vascular+endothelial+growth+factor+receptor+binding	0.824344	0.413141	0.972987
BP_GO:0045947_negative+regulation+of+translational+initiation	0.927483	0.956855	0.539678
BP_GO:0000380_alternative+nuclear+mRNA+splicing,+via+spliceosome	0.70125	0.467305	0.759671
BP_GO:0021819_layer+formation+in+the+cerebral+cortex	0.767649	0.767649	#N/A
BP_GO:0051457_maintenance+of+protein+location+in+nucleus	0.212746	0.212746	#N/A
BP_GO:0048557_embryonic+digestive+tract+morphogenesis	0.711939	0.781085	0.554459
MF_GO:0042162_telomeric+DNA+binding	0.691071	0.691071	#N/A
BP_GO:0045197_establishment+or+maintenance+of+epithelial+cell+apical/ł	0.856802	0.976032	0.463522
BP_GO:0017158_regulation+of+calcium+ion-dependent+exocytosis	0.496983	0.521742	0.46428
BP_GO:0031077_post-embryonic+camera-type+eye+development	0.33874	0.452248	0.335679
MF_GO:0005078_MAP-kinase+scaffold+activity	0.121073	0.121073	#N/A
BP_GO:0030072_peptide+hormone+secretion	0.283118	0.283118	#N/A
MF_GO:0004887_thyroid+hormone+receptor+activity	0.976527	0.473674	0.99345
BP_GO:0006120_mitochondrial+electron+transport,+NADH+to+ubiquinone	0.903667	0.869652	0.747694
MF_GO:0003708_retinoic+acid+receptor+activity	0.64358	0.64358	#N/A
MF_GO:0003910_DNA+ligase+(ATP)+activity	0.168466	0.139271	0.448026
BP_GO:0030219_megakaryocyte+differentiation	0.734733	0.734733	#N/A
BP_GO:0031398_positive+regulation+of+protein+ubiquitination	0.633145	0.821988	0.237117
BP_GO:0060218_hemopoietic+stem+cell+differentiation	0.918835	0.918835	#N/A
BP_GO:0042633_hair+cycle	0.213581	0.213581	#N/A
BP_GO:0051567_histone+H3-K9+methylation	0.102897	0.18713	0.174828
MF_GO:0008413_8-oxo-7,8-dihydroguanine+triphosphatase+activity	0.168328	0.0592753	0.706671
BP_GO:0008595_determination+of+anterior/posterior+axis,+embryo	0.603943	0.676405	0.424616
BP_GO:0048645_organ+formation	0.291218	#N/A	0.291218
MF_GO:0004739_pyruvate+dehydrogenase+(acetyl-transferring)+activity	0.197002	0.263956	0.279687
BP_GO:0050856_regulation+of+T+cell+receptor+signaling+pathway	0.561945	0.348218	0.679806
CC_GO:0031932_TORC2+complex	0.0842683	0.0842683	#N/A
MF_GO:0016160_amylase+activity	0.965697	0.749725	0.986232
MF_GO:0005275_amine+transmembrane+transporter+activity	0.604066	0.891079	0.292099
MF_GO:0004946_bombesin+receptor+activity	0.39874	0.509833	0.315949
BP_GO:0021800_cerebral+cortex+tangential+migration	0.675991	0.675991	#N/A
MF_GO:0019237_centromeric+DNA+binding	0.249126	0.249126	#N/A
BP_GO:0006997_nucleus+organization	0.0539677	0.130835	0.11553
CC_GO:0031931_TORC1+complex	0.0894789	0.0894789	#N/A
BP_GO:0031167_rRNA+methylation	0.792926	0.509653	0.916482
BP_GO:0006323_DNA+packaging	0.649211	0.593506	0.629323
BP_GO:0001831_trophectodermal+cellular+morphogenesis	0.634257	0.708964	0.51269
BP_GO:0002455_humoral+immune+response+mediated+by+circulating+imr	0.00868832	#N/A	0.00868832
MF_GO:0001537_N-acetylgalactosamine+4-O-sulfotransferase+activity	0.962111	0.961008	0.720646

MF_GO:0051019_mitogen-activated+protein+kinase+binding	0.484579	0.484579	#N/A
BP_GO:0048012_hepatocyte+growth+factor+receptor+signaling+pathway	0.376671	0.571487	0.212155
CC_GO:0009331_glycerol-3-phosphate+dehydrogenase+complex	0.398022	0.4307	0.42059
BP_GO:0032609_interferon-gamma+production	0.626583	0.440295	0.780142
BP_GO:0015809_arginine+transport	0.863266	0.906018	0.659624
MF_GO:0015181_arginine+transmembrane+transporter+activity	0.863266	0.906018	0.659624
BP_GO:0000086_G2/M+transition+of+mitotic+cell+cycle	0.504466	0.618388	0.34229
BP_GO:0048661_positive+regulation+of+smooth+muscle+cell+proliferation	0.729347	#N/A	0.729347
BP_GO:0050767_regulation+of+neurogenesis	0.198582	0.275548	0.26646
BP_GO:0030538_embryonic+genitalia+morphogenesis	0.747001	0.688448	0.676538
BP_GO:0048853_forebrain+morphogenesis	0.18944	0.488703	0.145125
BP_GO:0006704_glucocorticoid+biosynthetic+process	0.00011645	8.07888E-07	0.767927
BP_GO:0046470_phosphatidylcholine+metabolic+process	0.93258	0.93258	#N/A
BP_GO:0033153_T+cell+receptor+V(D)J+recombination	0.612618	0.84668	0.355105
BP_GO:0042523_positive+regulation+of+tyrosine+phosphorylation+of+Stat5	0.470652	0.470652	#N/A
BP_GO:0060078_regulation+of+postsynaptic+membrane+potential	0.0863906	0.199705	0.120967
MF_GO:0008107_galactoside+2-alpha-L-fucosyltransferase+activity	0.496435	0.20686	0.714703
BP_GO:0060084_synaptic+transmission+involved+in+micturition	0.703625	0.805005	0.519171
BP_GO:0051963_regulation+of+synaptogenesis	0.818334	0.929759	0.528798
BP_GO:0032228_regulation+of+synaptic+transmission,+GABAergic	0.892403	0.892403	#N/A
BP_GO:0008594_photoreceptor+cell+morphogenesis	0.13083	0.376518	0.0668318
MF_GO:0017069_snRNA+binding	0.849784	0.781798	0.763901
CC_GO:0005672_transcription+factor+TFIIA+complex	0.302687	0.302687	#N/A
BP_GO:0060083_smooth+muscle+contraction+involved+in+micturition	0.311158	0.419868	0.285342
BP_GO:0042637_catagen	0.585268	0.396142	0.673795
BP_GO:0051101_regulation+of+DNA+binding	0.315891	#N/A	0.315891
BP_GO:0000212_meiotic+spindle+organization	0.951753	0.978215	0.51039
CC_GO:0044445_cytosolic+part	0.61323	0.54604	0.631452
BP_GO:0045807_positive+regulation+of+endocytosis	0.553837	0.649178	0.458377
BP_GO:0001574_ganglioside+biosynthetic+process	0.114325	0.371247	0.0524915
BP_GO:0030903_notochord+development	0.372654	0.842475	0.134014
BP_GO:0006623_protein+targeting+to+vacuole	0.784236	0.615325	0.828714
MF_GO:0004938_alpha2-adrenergic+receptor+activity	0.946808	0.974806	0.512321
BP_GO:0048563_post-embryonic+organ+morphogenesis	0.502267	0.502267	#N/A
MF_GO:0005098_Ran+GTPase+activator+activity	0.221718	0.212338	0.421416
BP_GO:0050871_positive+regulation+of+B+cell+activation	0.39551	0.276517	0.537873
MF_GO:0008656_caspase+activator+activity	0.853567	0.942399	0.569718
BP_GO:0048712_negative+regulation+of+astrocyte+differentiation	0.882935	0.919896	0.530237
BP_GO:0006426_glycyl-tRNA+aminoacylation	0.982465	0.802825	0.976165
MF_GO:0004820_glycine-tRNA+ligase+activity	0.982465	0.802825	0.976165
BP_GO:0000002_mitochondrial+genome+maintenance	0.337336	#N/A	0.337336
BP_GO:0017000_antibiotic+biosynthetic+process	0.32238	0.0403701	0.748552
BP_GO:0042471_ear+morphogenesis	0.203551	#N/A	0.203551
BP_GO:0007132_meiotic+metaphase+I	0.942373	0.979812	0.432607
MF_GO:0008020_G-protein+coupled+photoreceptor+activity	0.728069	0.638002	0.709887
BP_GO:0007217_tachykinin+signaling+pathway	0.405546	0.276848	0.55021
BP_GO:0001706_endoderm+formation	0.575835	0.575835	#N/A

BP_GO:0060164_regulation+of+timing+of+neuron+differentiation	0.0231114	0.0231114	#N/A
MF_GO:0005227_calcium+activated+cation+channel+activity	0.729427	0.883314	0.26555
BP_GO:0010553_negative+regulation+of+specific+transcription+from+RNA+	0.931152	0.984461	0.316684
CC_GO:0034706_sodium+channel+complex	0.247957	0.247957	#N/A
BP_GO:0050672_negative+regulation+of+lymphocyte+proliferation	0.751716	0.751716	#N/A
MF_GO:0015321_sodium-dependent+phosphate+transmembrane+transpor	0.782877	0.987466	0.265954
BP_GO:0006207_'de+novo'+pyrimidine+base+biosynthetic+process	0.205059	0.67944	0.0904313
MF_GO:0015267_channel+activity	0.816965	0.816965	#N/A
CC_GO:0008023_transcription+elongation+factor+complex	0.999337	0.999337	#N/A
BP_GO:0051965_positive+regulation+of+synaptogenesis	0.730607	0.730607	#N/A
MF_GO:0031403_lithium+ion+binding	0.591805	0.591805	#N/A
BP_GO:0010825_positive+regulation+of+centrosome+duplication	0.919727	0.919727	#N/A
BP_GO:0006388_tRNA+splicing,+via+endonucleolytic+cleavage+and+ligatior	0.238183	0.240215	0.354462
MF_GO:0008241_peptidyl-dipeptidase+activity	0.810134	0.810134	#N/A
BP_GO:0007442_hindgut+morphogenesis	0.185693	0.206596	0.347901
BP_GO:0030718_germ-line+stem+cell+maintenance	0.626673	0.626673	#N/A
BP_GO:0042421_norepinephrine+biosynthetic+process	0.877924	0.835714	0.737941
MF_GO:0050780_dopamine+receptor+binding	0.268651	0.144462	0.497694
MF_GO:0005519_cytoskeletal+regulatory+protein+binding	0.0796156	0.228581	0.0826748
BP_GO:0021913_regulation+of+transcription+from+RNA+polymerase+II+prc	0.46062	0.46062	#N/A
BP_GO:0001710_mesodermal+cell+fate+commitment	0.091777	0.0789934	0.379462
BP_GO:0001976_regulation+of+systemic+arterial+blood+pressure+by+neurc	0.881158	0.953457	0.371168
MF_GO:0004305_ethanolamine+kinase+activity	0.34281	0.34281	#N/A
BP_GO:0042832_defense+response+to+protozoan	0.457604	0.840762	0.0553116
BP_GO:0021543_pallium+development	0.394104	0.671477	0.260278
BP_GO:0031642_negative+regulation+of+myelination	0.964175	0.964175	#N/A
BP_GO:0031503_protein+complex+localization	0.820403	0.883108	0.462252
MF_GO:0001602_pancreatic+polypeptide+receptor+activity	0.614626	0.913887	0.271786
BP_GO:0042448_progesterone+metabolic+process	0.473341	0.46873	0.498284
MF_GO:0004461_lactose+synthase+activity	0.0365447	0.143649	0.0744877
MF_GO:0015087_cobalt+ion+transmembrane+transporter+activity	0.613307	0.493783	0.699077
MF_GO:0032137_guanine/thymine+mispair+binding	0.845974	0.845974	#N/A
BP_GO:0045089_positive+regulation+of+innate+immune+response	0.933311	0.930483	0.694616
BP_GO:0043586_tongue+development	0.373814	0.529545	0.253987
BP_GO:0032651_regulation+of+interleukin-1+beta+production	0.89916	0.642319	0.904364
BP_GO:0002666_positive+regulation+of+T+cell+tolerance+induction	0.50825	0.525513	0.478428
BP_GO:0016080_synaptic+vesicle+targeting	0.0965519	0.0870856	0.263212
CC_GO:0032040_small-subunit+processome	0.735539	0.712839	0.616862
BP_GO:0006883_cellular+sodium+ion+homeostasis	0.923442	0.798049	0.877105
MF_GO:0001733_galactosylceramide+sulfotransferase+activity	0.596018	0.758625	0.283936
BP_GO:0042711_maternal+behavior	0.922253	0.922253	#N/A
BP_GO:0001894_tissue+homeostasis	0.724637	0.893333	0.233947
MF_GO:0004035_alkaline+phosphatase+activity	0.619354	0.709011	0.493415
MF_GO:0051879_Hsp90+protein+binding	0.916614	0.962112	0.454115
BP_GO:0035067_negative+regulation+of+histone+acetylation	0.559507	0.559507	#N/A
BP_GO:0021570_rhombomere+4+development	0.880632	0.877304	0.655113
BP_GO:0006405_RNA+export+from+nucleus	0.977936	0.989113	0.800877

BP_GO:0016242_negative+regulation+of+macroautophagy	0.385595	0.608396	0.185993
BP_GO:0046649_lymphocyte+activation	0.365346	0.769708	0.0505015
MF_GO:0003917_DNA+topoisomerase+type+I+activity	0.249699	0.492229	0.126609
MF_GO:0017025_TATA-binding+protein+binding	0.509917	0.261075	0.828751
CC_GO:0042765_GPI-anchor+transamidase+complex	0.60924	0.798928	0.400534
BP_GO:0048755_branching+morphogenesis+of+a+nerve	0.87482	0.785247	0.809264
CC_GO:0044442_microtubule-based+flagellum+part	0.845323	0.932115	0.363936
MF_GO:0016615_malate+dehydrogenase+activity	0.386169	0.362398	0.498897
MF_GO:0004719_protein-L-isoaspartate+(D-aspartate)+O-methyltransferase	0.836608	0.452723	0.969143
BP_GO:0009247_glycolipid+biosynthetic+process	0.456397	0.685194	0.317534
BP_GO:0006024_glycosaminoglycan+biosynthetic+process	0.899236	0.485502	0.988268
BP_GO:0045859_regulation+of+protein+kinase+activity	0.373818	0.373818	#N/A
BP_GO:0006177_GMP+biosynthetic+process	0.887166	#N/A	0.887166
BP_GO:0006112_energy+reserve+metabolic+process	0.37451	0.720378	0.210625
MF_GO:0008934_inositol-1(or+4)-monophosphatase+activity	0.441717	0.441717	#N/A
BP_GO:0021529_spinal+cord+oligodendrocyte+cell+differentiation	0.291984	#N/A	0.291984
BP_GO:0021530_spinal+cord+oligodendrocyte+cell+fate+specification	0.291984	#N/A	0.291984
CC_GO:0001520_outer+dense+fiber	0.26279	0.602671	0.168235
BP_GO:0015872_dopamine+transport	0.904392	0.904392	#N/A
BP_GO:0006360_transcription+from+RNA+polymerase+I+promoter	0.644657	#N/A	0.644657
BP_GO:0032715_negative+regulation+of+interleukin-6+production	0.615565	0.403462	0.803921
BP_GO:0042274_ribosomal+small+subunit+biogenesis	0.0950099	#N/A	0.0950099
MF_GO:0048487_beta-tubulin+binding	0.785332	0.194807	0.942566
BP_GO:0051603_proteolysis+involved+in+cellular+protein+catabolic+proces	0.718306	0.948231	0.0966008
BP_GO:0060039_pericardium+development	0.677633	0.677633	#N/A
MF_GO:0008900_hydrogen:potassium-exchanging+ATPase+activity	0.902213	0.911705	0.630057
MF_GO:0004966_galanin+receptor+activity	0.420399	0.567279	0.278494
BP_GO:0060291_long-term+synaptic+potentiation	0.683617	0.8649	0.232332
BP_GO:0045749_negative+regulation+of+S+phase+of+mitotic+cell+cycle	0.143298	0.256856	0.178151
BP_GO:0009048_dosage+compensation,+by+inactivation+of+X+chromosom	0.373446	0.894918	0.100159
MF_GO:0005549_odorant+binding	0.35121	0.254581	0.499585
MF_GO:0015026_coreceptor+activity	0.0988759	0.0429872	0.357977
CC_GO:0001674_female+germ+cell+nucleus	0.735635	0.735635	#N/A
CC_GO:0005655_nucleolar+ribonuclease+P+complex	0.233575	0.0323974	0.660875
BP_GO:0006266_DNA+ligation	0.357609	0.737642	0.185091
MF_GO:0004995_tachykinin+receptor+activity	0.206279	0.338751	0.202914
CC_GO:0048786_presynaptic+active+zone	0.946804	0.915327	0.803662
BP_GO:0021555_midbrain-hindbrain+boundary+morphogenesis	0.445426	0.445426	#N/A
BP_GO:0045578_negative+regulation+of+B+cell+differentiation	0.304673	#N/A	0.304673
BP_GO:0042147_retrograde+transport,+endosome+to+Golgi	0.151177	0.151177	#N/A
MF_GO:0003964_RNA-directed+DNA+polymerase+activity	0.657881	0.613461	0.615941
BP_GO:0031133_regulation+of+axon+diameter	0.808008	0.907731	0.356504
MF_GO:0016527_brain-specific+angiogenesis+inhibitor+activity	0.634139	0.848942	0.378692
BP_GO:0016539_intein-mediated+protein+splicing	0.78214	0.848988	0.456728
BP_GO:0006481_C-terminal+protein+amino+acid+methylation	0.852965	0.972566	0.47163
BP_GO:0010824_regulation+of+centrosome+duplication	0.929848	0.896558	0.779592
MF_GO:0004865_type+1+serine/threonine+specific+protein+phosphatase+i	0.882079	0.508853	0.924639

BP_GO:0008589_regulation+of+smoothened+signaling+pathway	0.277886	0.738424	0.0271827
MF_GO:0015277_kainate+selective+glutamate+receptor+activity	0.35175	0.0771446	0.912518
BP_GO:0007262_STAT+protein+nuclear+translocation	0.708195	0.708195	#N/A
BP_GO:0009743_response+to+carbohydrate+stimulus	0.876645	0.876645	#N/A
BP_GO:0045110_intermediate+filament+bundle+assembly	0.589735	0.704821	0.356504
MF_GO:0004406_H3/H4+histone+acetyltransferase+activity	0.116488	0.255175	0.128112
MF_GO:0050501_hyaluronan+synthase+activity	0.424135	0.74141	0.244546
BP_GO:0045085_negative+regulation+of+interleukin-2+biosynthetic+proces	0.353069	0.456422	0.350442
BP_GO:0050710_negative+regulation+of+cytokine+secretion	0.547418	0.397212	0.717614
BP_GO:0042668_auditory+receptor+cell+fate+determination	0.16939	0.0678173	0.453529
BP_GO:0021985_neurohypophysis+development	0.3131	0.393898	0.321747
BP_GO:0006283_transcription-coupled+nucleotide-excision+repair	0.396989	0.462767	0.399963
BP_GO:0045953_negative+regulation+of+natural+killer+cell+mediated+cyto	0.775801	0.775801	#N/A
BP_GO:0050955_thermoception	0.62116	0.912996	0.280133
MF_GO:0008327_methyl-CpG+binding	0.956176	0.956176	#N/A
BP_GO:0001992_regulation+of+systemic+arterial+blood+pressure+by+vasop	0.203262	0.489399	0.159229
BP_GO:0050968_detection+of+chemical+stimulus+involved+in+sensory+per	0.889237	0.971206	0.285342
MF_GO:0035035_histone+acetyltransferase+binding	0.119593	0.214896	0.178151
MF_GO:0048029_monosaccharide+binding	0.63665	0.366682	0.861788
BP_GO:0048747_muscle+fiber+development	0.898908	0.877138	0.715253
MF_GO:0008745_N-acetylmuramoyl-L-alanine+amidase+activity	0.0929275	#N/A	0.0929275
BP_GO:0002309_T+cell+proliferation+during+immune+response	0.478052	#N/A	0.478052
CC_GO:0030127_COPII+vesicle+coat	0.490886	0.560367	0.399779
BP_GO:0050917_sensory+perception+of+umami+taste	0.116742	0.0554532	0.369775
BP_GO:0021615_glossopharyngeal+nerve+morphogenesis	0.806611	0.615506	0.8032
MF_GO:0015018_galactosylgalactosylxylosylprotein+3-beta-glucuronosyltra	0.940326	0.875743	0.862776
BP_GO:0014002_astrocyte+development	0.70314	0.836717	0.321747
MF_GO:0008061_chitin+binding	0.0918052	0.248237	0.1255
BP_GO:0045348_positive+regulation+of+MHC+class+II+biosynthetic+proces	0.967151	0.967151	#N/A
BP_GO:0007041_lysosomal+transport	0.895081	0.901409	0.636658
BP_GO:0045900_negative+regulation+of+translational+elongation	0.653109	#N/A	0.653109
BP_GO:0006505_GPI+anchor+metabolic+process	0.552136	0.922837	0.198837
MF_GO:0016654_oxidoreductase+activity,+acting+on+NADH+or+NADPH,+di	0.75024	0.65804	0.724071
BP_GO:0006422_aspartyl-tRNA+aminoacylation	0.846709	0.782387	0.748177
MF_GO:0004815_aspartate-tRNA+ligase+activity	0.846709	0.782387	0.748177
BP_GO:0043631_RNA+polyadenylation	0.541969	0.75742	0.210604
BP_GO:0021869_forebrain+ventricular+zone+progenitor+cell+division	0.870163	0.776278	0.80076
BP_GO:0043584_nose+development	0.506285	0.82083	0.264516
BP_GO:0051205_protein+insertion+into+membrane	0.431968	0.699211	0.281447
BP_GO:0016050_vesicle+organization	0.603831	0.603831	#N/A
BP_GO:0043270_positive+regulation+of+ion+transport	0.903179	0.953826	0.44918
CC_GO:0033269_internode+region+of+axon	0.95962	0.971358	0.632164
MF_GO:0032549_ribonucleoside+binding	0.783809	0.868193	0.412855
BP_GO:0030101_natural+killer+cell+activation	0.232891	0.341428	0.246505
MF_GO:0030304_trypsin+inhibitor+activity	0.97847	0.850197	0.979309
CC_GO:0005577_fibrinogen+complex	0.714361	0.807954	0.472567
BP_GO:0006603_phosphocreatine+metabolic+process	0.807508	0.583504	0.845851

MF_GO:0042132_fructose+1,6-bisphosphate+1-phosphatase+activity	0.685727	0.685727	#N/A
BP_GO:0043627_response+to+estrogen+stimulus	0.784939	0.732227	0.690492
CC_GO:0005862_muscle+thin+filament+tropomyosin	0.116077	#N/A	0.116077
MF_GO:0017159_pantetheine+hydrolase+activity	0.11847	0.151445	0.260264
MF_GO:0047131_saccharopine+dehydrogenase+(NAD+,+L-glutamate-formir	0.272964	0.272964	#N/A
MF_GO:0003960_NADPH:quinone+reductase+activity	0.351711	0.450838	0.339224
BP_GO:0001998_angiotensin+mediated+vasoconstriction+involved+in+regu	0.364397	0.50174	0.310505
MF_GO:0008184_glycogen+phosphorylase+activity	0.904488	0.964314	0.518905
BP_GO:0019322_pentose+biosynthetic+process	0.396631	0.396631	#N/A
MF_GO:0042134_rRNA+primary+transcript+binding	0.99525	0.99525	#N/A
MF_GO:0030296_protein+tyrosine+kinase+activator+activity	0.788401	0.732227	0.696404
MF_GO:0017067_tyrosine-ester+sulfotransferase+activity	0.244413	0.244413	#N/A
MF_GO:0043395_heparan+sulfate+proteoglycan+binding	0.532511	0.516141	0.530079
MF_GO:0046592_polyamine+oxidase+activity	6.60885E-05	9.96579E-05	0.0184743
BP_GO:0048807_female+genitalia+morphogenesis	0.291983	0.291983	#N/A
MF_GO:0004942_anaphylatoxin+receptor+activity	0.710154	0.693307	0.609824
MF_GO:0004607_phosphatidylcholine-sterol+O-acyltransferase+activity	0.882415	0.882415	#N/A
CC_GO:0005924_cell-substrate+adherens+junction	0.967099	0.967099	#N/A
BP_GO:0001973_adenosine+receptor+signaling+pathway	0.144493	0.144493	#N/A
MF_GO:0015645_fatty-acid+ligase+activity	0.626435	0.921038	0.16969
BP_GO:0048193_Golgi+vesicle+transport	0.0950998	0.189562	0.165174
MF_GO:0003986_acetyl-CoA+hydrolase+activity	0.097068	#N/A	0.097068
BP_GO:0032613_interleukin-10+production	0.631113	0.631113	#N/A
BP_GO:0032615_interleukin-12+production	0.631113	0.631113	#N/A
MF_GO:0004743_pyruvate+kinase+activity	0.930643	0.930643	#N/A
BP_GO:0042730_fibrinolysis	0.942708	0.93742	0.757901
MF_GO:0000247_C-8+sterol+isomerase+activity	0.556286	0.556286	#N/A
BP_GO:0002072_optic+cup+morphogenesis+involved+in+camera-type+eye+	0.836009	#N/A	0.836009
BP_GO:0046208_spermine+catabolic+process	0.346743	#N/A	0.346743
BP_GO:0043490_malate-aspartate+shuttle	0.124281	0.124281	#N/A
MF_GO:0004063_aryldialkylphosphatase+activity	0.590997	0.590997	#N/A
BP_GO:0045639_positive+regulation+of+myeloid+cell+differentiation	0.34534	0.585326	0.218272
MF_GO:0047750_cholestenol+delta-isomerase+activity	0.747963	0.747963	#N/A
BP_GO:0042308_negative+regulation+of+protein+import+into+nucleus	0.994877	0.933535	0.983431
MF_GO:0004373_glycogen+(starch)+synthase+activity	0.674413	#N/A	0.674413
MF_GO:0016768_spermine+synthase+activity	0.777483	0.777483	#N/A
MF_GO:0004772_sterol+O-acyltransferase+activity	0.0741065	0.0808557	0.259191
MF_GO:0034736_cholesterol+O-acyltransferase+activity	0.0741065	0.0808557	0.259191
MF_GO:0034737_ergosterol+O-acyltransferase+activity	0.0741065	0.0808557	0.259191
MF_GO:0034738_lanosterol+O-acyltransferase+activity	0.0741065	0.0808557	0.259191
BP_GO:0019509_methionine+salvage	0.564893	#N/A	0.564893
BP_GO:0001935_endothelial+cell+proliferation	0.0886337	0.0886337	#N/A
MF_GO:0019955_cytokine+binding	0.200894	0.200894	#N/A
BP_GO:0006544_glycine+metabolic+process	0.62444	0.62444	#N/A
MF_GO:0019003_GDP+binding	0.107015	0.393787	0.0683669
MF_GO:0008170_N-methyltransferase+activity	0.588147	0.588147	#N/A
BP_GO:0006907_pinocytosis	0.479751	#N/A	0.479751

BP_GO:0006529_asparagine+biosynthetic+process	0.647854	0.647854	#N/A
MF_GO:0004066_asparagine+synthase+(glutamine-hydrolyzing)+activity	0.647854	0.647854	#N/A
BP_GO:0016554_cytidine+to+uridine+editing	0.38439	0.38439	#N/A
MF_GO:0004014_adenosylmethionine+decarboxylase+activity	0.953135	#N/A	0.953135
BP_GO:0006778_porphyrin+metabolic+process	0.350166	#N/A	0.350166
MF_GO:0043185_vascular+endothelial+growth+factor+receptor+3+binding	0.0183494	0.241526	0.0121029
BP_GO:0030497_fatty+acid+elongation	0.38156	0.29094	0.549635
MF_GO:0003920_GMP+reductase+activity	0.493872	0.493872	#N/A
MF_GO:0003858_3-hydroxybutyrate+dehydrogenase+activity	0.137388	0.137388	#N/A
MF_GO:0001846_opsonin+binding	0.26584	#N/A	0.26584
MF_GO:0004478_methionine+adenosyltransferase+activity	0.369983	0.369983	#N/A
BP_GO:0045019_negative+regulation+of+nitric+oxide+biosynthetic+process	0.0522965	#N/A	0.0522965
BP_GO:0007527_adult+somatic+muscle+development	0.725856	0.412002	0.858204
MF_GO:0008453_alanine-glyoxylate+transaminase+activity	0.00208044	0.00208044	#N/A
BP_GO:0014909_smooth+muscle+cell+migration	0.725334	0.368471	0.88171
MF_GO:0004074_biliverdin+reductase+activity	0.356993	#N/A	0.356993
BP_GO:0006569_tryptophan+catabolic+process	0.697645	0.697645	#N/A
BP_GO:0043462_regulation+of+ATPase+activity	0.899209	#N/A	0.899209
BP_GO:0019452_L-cysteine+catabolic+process+to+taurine	0.840024	0.720096	0.795183
MF_GO:0004359_glutaminase+activity	0.0515364	0.0390746	0.293098
BP_GO:0048554_positive+regulation+of+metalloenzyme+activity	0.899206	0.904917	0.690492
MF_GO:0015114_phosphate+transmembrane+transporter+activity	0.0728266	0.0728266	#N/A
BP_GO:0001561_fatty+acid+alpha-oxidation	0.866239	0.629166	0.892445
BP_GO:0019853_L-ascorbic+acid+biosynthetic+process	0.388887	#N/A	0.388887
BP_GO:0002074_extraocular+skeletal+muscle+development	0.490983	#N/A	0.490983
MF_GO:0005547_phosphatidylinositol-3,4,5-triphosphate+binding	0.560896	0.560896	#N/A
BP_GO:0019344_cysteine+biosynthetic+process	0.289973	0.289973	#N/A
CC_GO:0005927_muscle+tendon+junction	0.677556	#N/A	0.677556
BP_GO:0048260_positive+regulation+of+receptor-mediated+endocytosis	0.964479	0.9575	0.79736
MF_GO:0018685_alkane+1-monooxygenase+activity	0.237249	0.898615	0.0111547
MF_GO:0047756_chondroitin+4-sulfotransferase+activity	0.829923	#N/A	0.829923
MF_GO:0004465_lipoprotein+lipase+activity	0.0499613	0.0499613	#N/A
BP_GO:0002329_pre-B+cell+differentiation	0.578742	0.578742	#N/A
MF_GO:0005099_Ras+GTPase+activator+activity	0.411986	0.411986	#N/A
MF_GO:0004421_hydroxymethylglutaryl-CoA+synthase+activity	0.172521	0.0919985	0.497242
MF_GO:0004476_mannose-6-phosphate+isomerase+activity	0.847465	0.92148	0.514566
BP_GO:0018119_peptidyl-cysteine+S-nitrosylation	0.732802	0.473691	0.827815
BP_GO:0032959_inositol+trisphosphate+biosynthetic+process	0.454392	0.234163	0.713492
BP_GO:0045730_respiratory+burst	0.805714	0.732227	0.72603
BP_GO:0015808_L-alanine+transport	0.950903	0.950903	#N/A
BP_GO:0015824_proline+transport	0.950903	0.950903	#N/A
MF_GO:0005280_hydrogen:amino+acid+symporter+activity	0.950903	0.950903	#N/A
MF_GO:0005302_L-tyrosine+transmembrane+transporter+activity	0.950903	0.950903	#N/A
MF_GO:0015180_L-alanine+transmembrane+transporter+activity	0.950903	0.950903	#N/A
MF_GO:0015193_L-proline+transmembrane+transporter+activity	0.950903	0.950903	#N/A
BP_GO:0001543_ovarian+follicle+rupture	0.156195	0.175054	0.310505
BP_GO:0006097_glyoxylate+cycle	0.722606	0.722606	#N/A

BP_GO:0006102_isocitrate+metabolic+process	0.722606	0.722606	#N/A
MF_GO:0004450_isocitrate+dehydrogenase+(NADP+)+activity	0.722606	0.722606	#N/A
CC_GO:0005584_collagen+type+I	0.310284	#N/A	0.310284
BP_GO:0043507_positive+regulation+of+JNK+activity	0.304582	0.276549	0.448391
BP_GO:0051451_myoblast+migration	0.819894	0.734029	0.748572
MF_GO:0004776_succinate-CoA+ligase+(GDP-forming)+activity	0.754118	#N/A	0.754118
MF_GO:0003951_NAD++kinase+activity	0.756307	0.519837	0.824679
BP_GO:0006555_methionine+metabolic+process	0.173357	0.173357	#N/A
BP_GO:0002018_renin-angiotensin+regulation+of+aldosterone+production	0.129855	0.135729	0.310505
BP_GO:0002019_regulation+of+renal+output+by+angiotensin	0.129855	0.135729	0.310505
MF_GO:0004531_deoxyribonuclease+II+activity	0.284085	0.284085	#N/A
MF_GO:0004449_isocitrate+dehydrogenase+(NAD+)+activity	0.79693	0.906733	0.442331
BP_GO:0060346_bone+trabecula+formation	0.155128	#N/A	0.155128
BP_GO:0001885_endothelial+cell+development	0.803118	0.49456	0.88891
BP_GO:0019439_aromatic+compound+catabolic+process	0.210959	0.349465	0.226933
MF_GO:0004301_epoxide+hydrolase+activity	0.210959	0.349465	0.226933
BP_GO:0006570_tyrosine+metabolic+process	0.48065	0.48065	#N/A
MF_GO:0047749_cholestanetriol+26-monooxygenase+activity	0.000831137	0.000831137	#N/A
MF_GO:0003963_RNA-3'-phosphate+cyclase+activity	0.292193	0.0527143	0.801137
MF_GO:0004067_asparaginase+activity	0.0452065	0.0452065	#N/A
BP_GO:0030206_chondroitin+sulfate+biosynthetic+process	0.0517091	0.0303869	0.334166
MF_GO:0018738_S-formylglutathione+hydrolase+activity	0.279002	0.279002	#N/A
MF_GO:0003973_(S)-2-hydroxy-acid+oxidase+activity	0.881687	0.881687	#N/A
BP_GO:0021520_spinal+cord+motor+neuron+cell+fate+specification	0.682643	#N/A	0.682643
BP_GO:0009081_branched+chain+family+amino+acid+metabolic+process	0.404782	#N/A	0.404782
BP_GO:0009082_branched+chain+family+amino+acid+biosynthetic+process	0.404782	#N/A	0.404782
MF_GO:0004084_branched-chain-amino-acid+transaminase+activity	0.404782	#N/A	0.404782
BP_GO:0001788_antibody-dependent+cellular+cytotoxicity	0.494204	0.139731	0.855765
MF_GO:0019770_IgG+receptor+activity	0.494204	0.139731	0.855765
BP_GO:0051900_regulation+of+mitochondrial+depolarization	0.464497	0.464497	#N/A
CC_GO:0017109_glutamate-cysteine+ligase+complex	0.464497	0.464497	#N/A
BP_GO:0046626_regulation+of+insulin+receptor+signaling+pathway	0.125066	0.205316	0.21075
MF_GO:0050051_leukotriene-B4+20-monooxygenase+activity	0.815212	0.865923	0.564618
BP_GO:0051926_negative+regulation+of+calcium+ion+transport	0.632716	0.632716	#N/A
BP_GO:0022407_regulation+of+cell-cell+adhesion	0.863988	0.863988	#N/A
BP_GO:0006563_L-serine+metabolic+process	0.608042	0.608042	#N/A
BP_GO:0001768_establishment+of+T+cell+polarity	0.664514	0.664514	#N/A
MF_GO:0004019_adenylosuccinate+synthase+activity	0.420174	#N/A	0.420174
BP_GO:0050777_negative+regulation+of+immune+response	0.0462788	0.0462788	#N/A
MF_GO:0005161_platelet-derived+growth+factor+receptor+binding	0.540098	0.571178	0.485477
BP_GO:0046879_hormone+secretion	0.415359	0.415359	#N/A
MF_GO:0030215_semaphorin+receptor+binding	0.273997	0.273997	#N/A
MF_GO:0004998_transferrin+receptor+activity	0.215846	0.861179	0.0139884
BP_GO:0042531_positive+regulation+of+tyrosine+phosphorylation+of+STAT	0.738552	0.738552	#N/A
BP_GO:0043506_regulation+of+JNK+activity	0.847318	0.399952	0.955829
MF_GO:0004586_ornithine+decarboxylase+activity	0.109565	0.281018	0.123372
BP_GO:0033627_cell+adhesion+mediated+by+integrin	0.360013	0.698312	0.15241

BP_GO:0006518_peptide+metabolic+process	0.936491	0.936491	#N/A
MF_GO:0003878_ATP+citrate+synthase+activity	0.122894	0.0241317	0.630593
BP_GO:0042732_D-xylose+metabolic+process	0.687386	0.813241	0.421405
MF_GO:0004471_malate+dehydrogenase+(decarboxylating)+activity	3.36033E-10	3.36033E-10	#N/A
BP_GO:0055013_cardiac+muscle+cell+development	0.375501	#N/A	0.375501
BP_GO:0032099_negative+regulation+of+appetite	0.662368	0.914857	0.218272
MF_GO:0003883_CTP+synthase+activity	0.181676	0.252941	0.267525
BP_GO:0016558_protein+import+into+peroxisome+matrix	0.0844129	0.043902	0.405473
BP_GO:0050901_leukocyte+tethering+or+rolling	0.0228897	0.0378905	0.146898
BP_GO:0009313_oligosaccharide+catabolic+process	0.598335	0.598335	#N/A
BP_GO:0006991_response+to+sterol+depletion	0.000787852	0.000787852	#N/A
BP_GO:0032796_uropod+organization	0.993425	0.94999	0.968846
MF_GO:0004372_glycine+hydroxymethyltransferase+activity	0.522685	0.522685	#N/A
BP_GO:0000083_regulation+of+transcription+of+G1/S-phase+of+mitotic+ce	0.307965	#N/A	0.307965
BP_GO:0051938_L-glutamate+import	0.304899	0.304899	#N/A
BP_GO:0043312_neutrophil+degranulation	0.0976785	0.0976785	#N/A
BP_GO:0048002_antigen+processing+and+presentation+of+peptide+antiger	0.751555	#N/A	0.751555
MF_GO:0008429_phosphatidylethanolamine+binding	0.134465	0.134465	#N/A
CC_GO:0031088_platelet+dense+granule+membrane	0.637828	#N/A	0.637828
BP_GO:0050714_positive+regulation+of+protein+secretion	0.982178	0.973427	0.850672
MF_GO:0008238_exopeptidase+activity	0.87851	0.859947	0.716465
BP_GO:0042373_vitamin+K+metabolic+process	0.271924	0.550204	0.162439
CC_GO:0008247_1-alkyl-2-acetylglycerophosphocholine+esterase+complex	0.438131	0.438131	#N/A
BP_GO:0046329_negative+regulation+of+JNK+cascade	0.871359	0.871359	#N/A
MF_GO:0019166_trans-2-enoyl-CoA+reductase+(NADPH)+activity	0.725695	#N/A	0.725695
MF_GO:0016403_dimethylargininase+activity	0.489921	0.738343	0.25032
MF_GO:0004335_galactokinase+activity	0.873134	#N/A	0.873134
MF_GO:0030188_chaperone+regulator+activity	0.329285	0.411271	0.344424
CC_GO:0005964_phosphorylase+kinase+complex	0.429185	#N/A	0.429185
MF_GO:0004689_phosphorylase+kinase+activity	0.429185	#N/A	0.429185
BP_GO:0042994_cytoplasmic+sequestering+of+transcription+factor	0.827215	0.827215	#N/A
MF_GO:0050262_ribosylnicotinamide+kinase+activity	0.0712279	0.615022	0.00896425
BP_GO:0006739_NADP+metabolic+process	0.304343	0.299611	0.421405
BP_GO:0006111_regulation+of+gluconeogenesis	0.494763	0.776367	0.218272
MF_GO:0016620_oxidoreductase+activity,+acting+on+the+aldehyde+or+oxo	0.899063	0.685164	0.907249
BP_GO:0030865_cortical+cytoskeleton+organization	0.427438	0.488965	0.408813
BP_GO:0008210_estrogen+metabolic+process	0.0190866	#N/A	0.0190866
MF_GO:0030375_thyroid+hormone+receptor+coactivator+activity	0.446854	0.641865	0.290482
BP_GO:0006525_arginine+metabolic+process	0.378497	0.0491333	0.888014
MF_GO:0004053_arginase+activity	0.378497	0.0491333	0.888014
BP_GO:0006275_regulation+of+DNA+replication	0.12516	0.12516	#N/A
CC_GO:0031528_microvillus+membrane	0.606403	0.606403	#N/A
BP_GO:0045619_regulation+of+lymphocyte+differentiation	0.204837	#N/A	0.204837
BP_GO:0030573_bile+acid+catabolic+process	0.00500574	0.00500574	#N/A
MF_GO:0017130_poly(rC)+binding	0.846183	0.496585	0.926819
BP_GO:0043366_beta+selection	0.330065	0.284068	0.479725
MF_GO:0019902_phosphatase+binding	0.463784	0.463784	#N/A

BP_GO:0002537_production+of+nitric+oxide+during+acute+inflammatory+r	0.62342	0.62342	#N/A
CC_GO:0019867_outer+membrane	0.124073	0.124073	#N/A
BP_GO:0006532_aspartate+biosynthetic+process	0.553017	0.553017	#N/A
BP_GO:0019550_glutamate+catabolic+process+to+aspartate	0.553017	0.553017	#N/A
BP_GO:0019551_glutamate+catabolic+process+to+2-oxoglutarate	0.553017	0.553017	#N/A
BP_GO:0051725_protein+amino+acid+de-ADP-ribosylation	0.333614	0.333614	#N/A
MF_GO:0003875_ADP-ribosylarginine+hydrolase+activity	0.333614	0.333614	#N/A
BP_GO:0051583_dopamine+uptake	0.101741	#N/A	0.101741
BP_GO:0001845_phagolysosome+formation	0.548708	0.548708	#N/A
BP_GO:0001771_formation+of+immunological+synapse	0.381128	0.381128	#N/A
MF_GO:0030023_extracellular+matrix+constituent+conferring+elasticity	0.383465	0.72931	0.151607
BP_GO:0006198_cAMP+catabolic+process	0.00663605	0.00663605	#N/A
BP_GO:0014916_regulation+of+lung+blood+pressure	0.609998	0.609998	#N/A
MF_GO:0004962_endothelin+receptor+activity	0.859263	0.672796	0.859188
BP_GO:0051602_response+to+electrical+stimulus	0.883297	0.973427	0.402358
MF_GO:0047804_cysteine-S-conjugate+beta-lyase+activity	0.378509	0.436806	0.390442
BP_GO:0030836_positive+regulation+of+actin+filament+depolymerization	0.160238	0.182341	0.309019
BP_GO:0009264_deoxyribonucleotide+catabolic+process	0.430532	0.625113	0.28568
BP_GO:0046459_short-chain+fatty+acid+metabolic+process	0.799938	0.799938	#N/A
MF_GO:0003785_actin+monomer+binding	0.0974241	0.50415	0.0325873
MF_GO:0000900_translation+repressor+activity,+nucleic+acid+binding	0.907729	0.907729	#N/A
MF_GO:0042056_chemoattractant+activity	0.985608	0.9575	0.914909
BP_GO:0045723_positive+regulation+of+fatty+acid+biosynthetic+process	0.455115	#N/A	0.455115
BP_GO:0051024_positive+regulation+of+immunoglobulin+secretion	0.66521	0.66521	#N/A
MF_GO:0005159_insulin-like+growth+factor+receptor+binding	0.808378	0.948809	0.344858
BP_GO:0008582_regulation+of+synaptic+growth+at+neuromuscular+junctic	0.0802274	0.0802274	#N/A
MF_GO:0046912_transferase+activity,+transferring+acyl+groups,+acyl+grou	0.00488373	0.00488373	#N/A
BP_GO:0009156_ribonucleoside+monophosphate+biosynthetic+process	0.646637	0.820464	0.350389
BP_GO:0051291_protein+heterooligomerization	0.246699	#N/A	0.246699
MF_GO:0008124_4-alpha-hydroxytetrahydrobiopterin+dehydratase+activity	0.246699	#N/A	0.246699
BP_GO:0018346_protein+amino+acid+prenylation	0.00776309	0.00776309	#N/A
MF_GO:0008318_protein+prenyltransferase+activity	0.00776309	0.00776309	#N/A
BP_GO:0006068_ethanol+catabolic+process	0.280701	0.526193	0.187572
BP_GO:0042987_amyloid+precursor+protein+catabolic+process	0.0715554	0.194405	0.113239
BP_GO:0045212_neurotransmitter+receptor+biosynthetic+process	0.267348	0.267348	#N/A
BP_GO:0050650_chondroitin+sulfate+proteoglycan+biosynthetic+process	0.278451	0.333089	0.344858
BP_GO:0043280_positive+regulation+of+caspase+activity	0.180722	0.0523786	0.629871
BP_GO:0019218_regulation+of+steroid+metabolic+process	0.504826	0.504826	#N/A
BP_GO:0046543_development+of+secondary+female+sexual+characteristics	0.504826	0.504826	#N/A
BP_GO:0046544_development+of+secondary+male+sexual+characteristics	0.504826	0.504826	#N/A
BP_GO:0000085_G2+phase+of+mitotic+cell+cycle	0.635467	0.733885	0.446688
MF_GO:0047237_glucuronylgalactosylproteoglycan+4-beta-N-acetylgalactos	0.370396	0.370396	#N/A
BP_GO:0002755_MyD88-dependent+toll-like+receptor+signaling+pathway	0.675067	#N/A	0.675067
MF_GO:0004447_iodide+peroxidase+activity	0.059773	0.059773	#N/A
BP_GO:0043619_regulation+of+transcription+from+RNA+polymerase+II+prc	0.930401	0.930401	#N/A
MF_GO:0004161_dimethylallyltranstransferase+activity	0.0262747	0.0262747	#N/A
MF_GO:0004337_geranyltranstransferase+activity	0.0262747	0.0262747	#N/A

MF_GO:0019103_pyrimidine+nucleotide+binding	0.995228	0.995228	#N/A
BP_GO:0001834_trophectodermal+cell+proliferation	0.366697	#N/A	0.366697
BP_GO:0001919_regulation+of+receptor+recycling	0.150238	0.488844	0.0754335
MF_GO:0005068_transmembrane+receptor+protein+tyrosine+kinase+adapt	0.996892	0.987665	0.947952
BP_GO:0046426_negative+regulation+of+JAK-STAT+cascade	0.636083	#N/A	0.636083
BP_GO:0006086_acetyl-CoA+biosynthetic+process+from+pyruvate	0.135318	0.135318	#N/A
CC_GO:0030314_junctional+membrane+complex	0.981081	0.981081	#N/A
BP_GO:0042511_positive+regulation+of+tyrosine+phosphorylation+of+Stat1	0.940772	0.940772	#N/A
BP_GO:0007172_signal+complex+assembly	0.676258	0.676258	#N/A
MF_GO:0015272_ATP-activated+inward+rectifier+potassium+channel+activi	0.654397	#N/A	0.654397
BP_GO:0031098_stress-activated+protein+kinase+signaling+pathway	0.741782	0.968836	0.172222
MF_GO:0005372_water+transporter+activity	0.346861	0.646581	0.175469
BP_GO:0035166_post-embryonic+hemopoiesis	0.585988	0.384769	0.72603
BP_GO:0032743_positive+regulation+of+interleukin-2+production	0.617922	0.617922	#N/A
BP_GO:0043372_positive+regulation+of+CD4-positive,+alpha+beta+T+cell+d	0.617922	0.617922	#N/A
BP_GO:0002033_vasodilation+by+angiotensin+involved+in+regulation+of+sy	0.404143	0.404143	#N/A
BP_GO:0043415_positive+regulation+of+skeletal+muscle+regeneration	0.954704	0.954704	#N/A
BP_GO:0006089_lactate+metabolic+process	0.96591	0.976153	0.72603
BP_GO:0035313_wound+healing,+spreading+of+epidermal+cells	0.519008	0.543817	0.483208
BP_GO:0002752_cell+surface+pattern+recognition+receptor+signaling+path	0.405805	#N/A	0.405805
BP_GO:0001879_detection+of+yeast	0.321782	0.620081	0.168562
BP_GO:0008064_regulation+of+actin+polymerization+or+depolymerization	0.736256	#N/A	0.736256
MF_GO:0003994_aconitate+hydratase+activity	0.341704	0.699396	0.135852
MF_GO:0009384_N-acylmannosamine+kinase+activity	0.599319	0.599319	#N/A
MF_GO:0004020_adenylylsulfate+kinase+activity	0.183524	0.183524	#N/A
MF_GO:0004781_sulfate+adenylyltransferase+(ATP)+activity	0.183524	0.183524	#N/A
BP_GO:0042094_interleukin-2+biosynthetic+process	0.0789233	0.137053	0.182973
MF_GO:0004560_alpha-L-fucosidase+activity	0.370318	0.370318	#N/A
BP_GO:0001738_morphogenesis+of+a+polarized+epithelium	0.178483	0.184431	0.343039
BP_GO:0008615_pyridoxine+biosynthetic+process	0.828911	0.922744	0.468384
BP_GO:0002347_response+to+tumor+cell	0.475022	0.184431	0.791167
BP_GO:0033194_response+to+hydroperoxide	0.583991	0.178753	0.88891
MF_GO:0004419_hydroxymethylglutaryl-CoA+lyase+activity	0.039912	0.174754	0.0614944
BP_GO:0030262_apoptotic+nuclear+changes	0.693221	0.732227	0.538022
BP_GO:0006537_glutamate+biosynthetic+process	0.856943	0.856943	#N/A
BP_GO:0006562_proline+catabolic+process	0.856943	0.856943	#N/A
MF_GO:0004657_proline+dehydrogenase+activity	0.856943	0.856943	#N/A
MF_GO:0004943_C3a+anaphylatoxin+receptor+activity	0.819911	0.693307	0.785204
BP_GO:0002863_positive+regulation+of+inflammatory+response+to+antiger	0.509893	0.732227	0.279623
BP_GO:0046855_inositol+phosphate+dephosphorylation	0.502941	0.502941	#N/A
BP_GO:0032092_positive+regulation+of+protein+binding	0.978927	0.751085	0.986014
BP_GO:0031953_negative+regulation+of+protein+amino+acid+autophospho	0.126081	0.126081	#N/A
BP_GO:0051563_smooth+endoplasmic+reticulum+calcium+ion+homeostasis	0.33953	0.733885	0.113239
MF_GO:0015464_acetylcholine+receptor+activity	0.158549	0.306546	0.181597
BP_GO:0045359_positive+regulation+of+interferon-beta+biosynthetic+proc	0.308499	0.114569	0.689882
MF_GO:0004777_succinate-semialdehyde+dehydrogenase+activity	0.786009	0.685164	0.738851
MF_GO:0016361_activin+receptor+activity,+type+I	0.771457	0.771457	#N/A

BP_GO:0048739_cardiac+muscle+fiber+development	0.531961	0.0629565	0.949954
BP_GO:0006048_UDP-N-acetylglucosamine+biosynthetic+process	0.0199971	0.0199971	#N/A
MF_GO:0008476_protein-tyrosine+sulfotransferase+activity	0.983032	0.872184	0.968867
MF_GO:0004342_glucosamine-6-phosphate+deaminase+activity	0.0623911	0.0623911	#N/A
CC_GO:0031616_spindle+pole+centrosome	0.396088	0.741192	0.154029
MF_GO:0004008_copper-exporting+ATPase+activity	0.977082	0.977082	#N/A
MF_GO:0005007_fibroblast+growth+factor+receptor+activity	0.690259	0.690259	#N/A
BP_GO:0001878_response+to+yeast	0.465844	0.799291	0.168562
BP_GO:0006163_purine+nucleotide+metabolic+process	0.327766	0.143919	0.667216
BP_GO:0009258_10-formyltetrahydrofolate+catabolic+process	0.289311	0.289311	#N/A
BP_GO:0002268_follicular+dendritic+cell+differentiation	0.201987	#N/A	0.201987
BP_GO:0001302_replicative+cell+aging	0.30427	0.30427	#N/A
CC_GO:0031304_intrinsic+to+mitochondrial+inner+membrane	0.280362	0.325679	0.355422
MF_GO:0008396_oxysterol+7-alpha-hydroxylase+activity	0.00503884	0.00243921	0.204641
BP_GO:0009191_ribonucleoside+diphosphate+catabolic+process	0.692478	0.737359	0.530608
MF_GO:0030159_receptor+signaling+complex+scaffold+activity	0.556941	0.677233	0.398655
BP_GO:0031987_locomotion+during+locomotory+behavior	0.432802	0.432802	#N/A
BP_GO:0046070_dGTP+metabolic+process	0.849188	0.849188	#N/A
MF_GO:0033188_sphingomyelin+synthase+activity	0.297464	0.459158	0.258098
MF_GO:0047493_ceramide+cholinephosphotransferase+activity	0.297464	0.459158	0.258098
MF_GO:0019870_potassium+channel+inhibitor+activity	0.796876	#N/A	0.796876
CC_GO:0000939_inner+kinetochore+of+condensed+chromosome	0.842501	0.6517	0.849017
BP_GO:0043589_skin+morphogenesis	0.419235	0.419235	#N/A
BP_GO:0042089_cytokine+biosynthetic+process	0.753891	0.559565	0.79456
BP_GO:0050851_antigen+receptor-mediated+signaling+pathway	0.973796	0.868574	0.947952
MF_GO:0015651_quaternary+ammonium+group+transmembrane+transpor	0.614472	#N/A	0.614472
BP_GO:0001505_regulation+of+neurotransmitter+levels	0.0393522	0.0393522	#N/A
MF_GO:0008273_calcium,+potassium:sodium+antiporter+activity	0.0581254	#N/A	0.0581254
BP_GO:0001826_inner+cell+mass+cell+differentiation	0.131586	#N/A	0.131586
MF_GO:0008670_2,4-dienoyl-CoA+reductase+(NADPH)+activity	0.0413488	0.00812817	0.479468
BP_GO:0010035_response+to+inorganic+substance	0.857391	0.857391	#N/A
MF_GO:0004686_elongation+factor-2+kinase+activity	0.64422	#N/A	0.64422
MF_GO:0004618_phosphoglycerate+kinase+activity	0.66962	0.976373	0.0865089
BP_GO:0060166_olfactory+pit+development	0.20072	#N/A	0.20072
BP_GO:0045682_regulation+of+epidermis+development	0.555142	0.487922	0.589781
BP_GO:0009268_response+to+pH	0.139815	0.167998	0.285342
MF_GO:0030507_spectrin+binding	0.74786	0.74786	#N/A
BP_GO:0031579_membrane+raft+organization	0.346378	0.730951	0.120156
BP_GO:0002244_hemopoietic+progenitor+cell+differentiation	0.111064	0.302446	0.113239
BP_GO:0002286_T+cell+activation+during+immune+response	0.111064	0.302446	0.113239
BP_GO:0051491_positive+regulation+of+filopodium+formation	0.408613	0.560268	0.316279
BP_GO:0009957_epidermal+cell+fate+specification	0.390182	0.171083	0.710854
BP_GO:0048733_sebaceous+gland+development	0.390182	0.171083	0.710854
BP_GO:0042770_DNA+damage+response,+signal+transduction	0.188146	0.188146	#N/A
BP_GO:0009651_response+to+salt+stress	0.621765	0.824773	0.310505
CC_GO:0033267_axon+part	0.323162	0.323162	#N/A
BP_GO:0032703_negative+regulation+of+interleukin-2+production	0.0329206	0.0329206	#N/A

BP_GO:0002763_positive+regulation+of+myeloid+leukocyte+differentiation	0.685369	0.790552	0.450347
BP_GO:0051124_synaptic+growth+at+neuromuscular+junction	0.541624	0.541624	#N/A
BP_GO:0006002_fructose+6-phosphate+metabolic+process	0.785602	0.785602	#N/A
MF_GO:0030544_Hsp70+protein+binding	0.836098	0.836098	#N/A
CC_GO:0016460_myosin+II+complex	0.192942	0.11557	0.488479
MF_GO:0043531_ADP+binding	0.850586	0.850586	#N/A
MF_GO:0004692_cGMP-dependent+protein+kinase+activity	0.0990499	0.0990499	#N/A
MF_GO:0003688_DNA+replication+origin+binding	0.784751	0.6517	0.766074
CC_GO:0045335_phagocytic+vesicle	0.137351	0.137351	#N/A
MF_GO:0004797_thymidine+kinase+activity	0.854125	#N/A	0.854125
MF_GO:0005093_Rab+GDP-dissociation+inhibitor+activity	0.498821	0.498821	#N/A
MF_GO:0004315_3-oxoacyl-[acyl-carrier-protein]+synthase+activity	0.198195	0.198195	#N/A
BP_GO:0006654_phosphatidic+acid+biosynthetic+process	0.647714	0.647714	#N/A
BP_GO:0051290_protein+heterotetramerization	0.514913	0.514913	#N/A
MF_GO:0000010_trans-hexaprenyltranstransferase+activity	0.514913	0.514913	#N/A
MF_GO:0050347_trans-octaprenyltranstransferase+activity	0.514913	0.514913	#N/A
BP_GO:0015804_neutral+amino+acid+transport	0.386234	#N/A	0.386234
MF_GO:0033857_diphosphoinositol-pentakisphosphate+kinase+activity	0.650431	0.782779	0.407382
MF_GO:0019834_phospholipase+A2+inhibitor+activity	0.278537	#N/A	0.278537
MF_GO:0004609_phosphatidylserine+decarboxylase+activity	0.59074	0.59074	#N/A
BP_GO:0045921_positive+regulation+of+exocytosis	0.244013	0.244013	#N/A
CC_GO:0005753_mitochondrial+proton-transporting+ATP+synthase+comple	0.656311	0.656311	#N/A
BP_GO:0045634_regulation+of+melanocyte+differentiation	0.993624	0.993624	#N/A
BP_GO:0034613_cellular+protein+localization	0.247058	0.247058	#N/A
MF_GO:0043130_ubiquitin+binding	0.651128	0.651128	#N/A
MF_GO:0015108_chloride+transmembrane+transporter+activity	0.132178	0.132178	#N/A
MF_GO:0046922_peptide-O-fucosyltransferase+activity	0.725168	0.725168	#N/A
BP_GO:0007263_nitric+oxide+mediated+signal+transduction	4.24092E-05	#N/A	4.24092E-05
BP_GO:0060055_angiogenesis+involved+in+wound+healing	0.734215	#N/A	0.734215
BP_GO:0051925_regulation+of+calcium+ion+transport+via+voltage-gated+c	0.00710038	#N/A	0.00710038
BP_GO:0051135_positive+regulation+of+NK+T+cell+activation	0.462047	0.578872	0.369454
MF_GO:0030548_acetylcholine+receptor+regulator+activity	0.0145925	0.0145925	#N/A
MF_GO:0030158_protein+xylosyltransferase+activity	0.957385	0.566739	0.988322
MF_GO:0045569_TRAIL+binding	0.136443	0.0371082	0.592698
BP_GO:0035022_positive+regulation+of+Rac+protein+signal+transduction	0.413035	0.413035	#N/A
MF_GO:0003868_4-hydroxyphenylpyruvate+dioxygenase+activity	0.371112	0.371112	#N/A
BP_GO:0009186_deoxyribonucleoside+diphosphate+metabolic+process	0.597201	0.0809794	0.959702
BP_GO:0015893_drug+transport	0.748656	0.748656	#N/A
BP_GO:0048087_positive+regulation+of+pigmentation+during+developmen	0.237581	#N/A	0.237581
BP_GO:0044257_cellular+protein+catabolic+process	0.663038	0.835909	0.351253
MF_GO:0004021_alanine+transaminase+activity	0.823704	0.823704	#N/A
BP_GO:0030050_vesicle+transport+along+actin+filament	0.624865	0.342549	0.804099
MF_GO:0002020_protease+binding	0.311017	0.167468	0.605364
MF_GO:0005137_interleukin-5+receptor+binding	0.156738	0.297898	0.185356
MF_GO:0004967_glucagon+receptor+activity	0.222622	0.539777	0.119117
MF_GO:0047150_betaine-homocysteine+S-methyltransferase+activity	0.353788	0.353788	#N/A
MF_GO:0004850_uridine+phosphorylase+activity	0.0376257	0.282716	0.026092

BP_GO:0014850_response+to+muscle+activity	0.984317	0.984317	#N/A
CC_GO:0043205_fibril	0.758667	0.758667	#N/A
MF_GO:0008109_N-acetyllactosaminide+beta-1,6-N-acetylglucosaminyltran	0.0996198	0.0996198	#N/A
BP_GO:0006649_phospholipid+transfer+to+membrane	0.82358	0.973427	0.268058
MF_GO:0016316_phosphatidylinositol-3,4-bisphosphate+4-phosphatase+act	0.989468	0.976518	0.899328
MF_GO:0034597_phosphatidyl-inositol-4,5-bisphosphate+4-phosphatase+ac	0.989468	0.976518	0.899328
CC_GO:0042175_nuclear+envelope-endoplasmic+reticulum+network	0.88173	0.88173	#N/A
BP_GO:0046022_positive+regulation+of+transcription+from+RNA+polymera	0.0400205	0.0400205	#N/A
BP_GO:0051014_actin+filament+severing	0.345289	0.345289	#N/A
MF_GO:0005069_transmembrane+receptor+protein+tyrosine+kinase+docki	0.869282	0.870495	0.67748
MF_GO:0017112_Rab+guanyl-nucleotide+exchange+factor+activity	0.363515	0.631001	0.203868
MF_GO:0004771_sterol+esterase+activity	0.438528	0.445354	0.467738
BP_GO:0007194_negative+regulation+of+adenylate+cyclase+activity	0.470573	0.470573	#N/A
CC_GO:0005583_fibrillar+collagen	0.87765	#N/A	0.87765
BP_GO:0046827_positive+regulation+of+protein+export+from+nucleus	0.999232	0.999232	#N/A
BP_GO:0014902_myotube+differentiation	0.956737	#N/A	0.956737
BP_GO:0042488_positive+regulation+of+odontogenesis+of+dentine-contain	0.348421	0.552903	0.247085
BP_GO:0010766_negative+regulation+of+sodium+ion+transport	0.967389	0.967389	#N/A
BP_GO:0030916_otic+vesicle+formation	0.359596	0.758865	0.112979
MF_GO:0004925_prolactin+receptor+activity	0.661223	#N/A	0.661223
BP_GO:0001893_maternal+placenta+development	0.607691	#N/A	0.607691
MF_GO:0004516_nicotinate+phosphoribosyltransferase+activity	0.104235	0.104235	#N/A
BP_GO:0000084_S+phase+of+mitotic+cell+cycle	0.0274029	0.0300079	0.201604
MF_GO:0003867_4-aminobutyrate+transaminase+activity	0.0956035	0.0892238	0.307422
MF_GO:0047298_(S)-3-amino-2-methylpropionate+transaminase+activity	0.0956035	0.0892238	0.307422
MF_GO:0047696_beta-adrenergic+receptor+kinase+activity	0.833325	0.833325	#N/A
MF_GO:0001729_ceramide+kinase+activity	0.212908	0.212908	#N/A
BP_GO:0008333_endosome+to+lysosome+transport	0.00815218	0.0249089	0.0754335
MF_GO:0004660_protein+farnesyltransferase+activity	0.32953	0.32953	#N/A
BP_GO:0032230_positive+regulation+of+synaptic+transmission,+GABAergic	0.0299415	0.0299415	#N/A
BP_GO:0060081_membrane+hyperpolarization	0.120997	0.423486	0.071884
MF_GO:0030742_GTP-dependent+protein+binding	0.340098	0.340098	#N/A
BP_GO:0007051_spindle+organization	0.401548	0.114636	0.802328
BP_GO:0031401_positive+regulation+of+protein+modification+process	0.0639066	0.19836	0.0956909
BP_GO:0045542_positive+regulation+of+cholesterol+biosynthetic+process	0.101119	0.101119	#N/A
CC_GO:0016528_sarcoplasm	0.376231	#N/A	0.376231
CC_GO:0044428_nuclear+part	0.878968	0.878968	#N/A
BP_GO:0035024_negative+regulation+of+Rho+protein+signal+transduction	0.00608271	0.186349	0.00395928
MF_GO:0004591_oxoglutarate+dehydrogenase+(succinyl-transferring)+activ	0.846256	0.846256	#N/A
MF_GO:0004127_cytidylate+kinase+activity	0.618709	0.869999	0.242446
BP_GO:0016199_axon+midline+choice+point+recognition	0.838836	0.838836	#N/A
CC_GO:0042587_glycogen+granule	0.0962054	0.0211634	0.574003
BP_GO:0031648_protein+destabilization	0.91304	0.91304	#N/A
MF_GO:0004605_phosphatidate+cytidylyltransferase+activity	0.943489	0.938837	0.757292
MF_GO:0016857_racemase+and+epimerase+activity,+acting+on+carbohydra	0.206641	0.000309874	0.988268
BP_GO:0006702_androgen+biosynthetic+process	0.956387	0.956387	#N/A
MF_GO:0016705_oxidoreductase+activity,+acting+on+paired+donors,+with-	0.536531	0.536531	#N/A

MF_GO:0004109_coproporphyrinogen+oxidase+activity	0.956833	0.956833	#N/A
MF_GO:0004348_glucosylceramidase+activity	0.910817	0.875992	0.773206
BP_GO:0033700_phospholipid+efflux	0.422095	0.514607	0.376692
BP_GO:0006772_thiamin+metabolic+process	0.626995	0.6063	0.574993
BP_GO:0008065_establishment+of+blood-nerve+barrier	0.722658	0.908367	0.310505
MF_GO:0004142_diacylglycerol+cholinephosphotransferase+activity	0.214063	#N/A	0.214063
BP_GO:0016482_cytoplasmic+transport	0.838796	0.967475	0.328384
BP_GO:0051781_positive+regulation+of+cell+division	0.115919	0.115919	#N/A
MF_GO:0010302_2-oxoglutarate-dependent+dioxygenase+activity	0.360721	0.765032	0.110003
MF_GO:0051747_DNA+demethylase+activity	0.360721	0.765032	0.110003
BP_GO:0021831_embryonic+olfactory+bulb+interneuron+precursor+migrati	0.204403	0.298578	0.261198
MF_GO:0003938_IMP+dehydrogenase+activity	0.918564	#N/A	0.918564
MF_GO:0047276_N-acetyllactosaminide+3-alpha-galactosyltransferase+activ	0.741938	0.741938	#N/A
BP_GO:0033364_mast+cell+secretory+granule+organization	0.906277	0.901433	0.717614
BP_GO:0051084_'de+novo'+posttranslational+protein+folding	0.254671	0.262486	0.383082
BP_GO:0008608_attachment+of+spindle+microtubules+to+kinetochore	0.807006	0.807006	#N/A
MF_GO:0008474_palmitoyl-(protein)+hydrolase+activity	0.217544	#N/A	0.217544
MF_GO:0004958_prostaglandin+F+receptor+activity	0.570753	#N/A	0.570753
BP_GO:0002924_negative+regulation+of+humoral+immune+response+medi	0.236848	0.295909	0.316684
CC_GO:0030125_clathrin+vesicle+coat	0.268379	0.268379	#N/A
BP_GO:0007171_activation+of+transmembrane+receptor+protein+tyrosine-	0.768525	0.768525	#N/A
BP_GO:0060152_microtubule-based+peroxisome+localization	0.470902	0.470902	#N/A
MF_GO:0005025_transforming+growth+factor+beta+receptor+activity,+type	0.854223	0.706157	0.829094
BP_GO:0045409_negative+regulation+of+interleukin-6+biosynthetic+proces	0.168076	0.188539	0.316684
BP_GO:0043206_fibril+organization	0.9643	0.801549	0.955829
BP_GO:0007185_transmembrane+receptor+protein+tyrosine+phosphatase+	0.774617	0.93262	0.334297
BP_GO:0014065_phosphoinositide+3-kinase+cascade	0.273616	0.273616	#N/A
BP_GO:0050982_detection+of+mechanical+stimulus	0.747963	0.949836	0.242745
BP_GO:0048016_inositol+phosphate-mediated+signaling	0.622344	#N/A	0.622344
BP_GO:0016045_detection+of+bacterium	0.948	0.948	#N/A
MF_GO:0030144_alpha-1,6-mannosyl-glycoprotein+6-beta-N-acetylglucosar	0.231342	0.546593	0.12391
BP_GO:0002352_B+cell+negative+selection	0.417328	#N/A	0.417328
BP_GO:0010524_positive+regulation+of+calcium+ion+transport+into+cytosc	0.417328	#N/A	0.417328
BP_GO:0032471_reduction+of+endoplasmic+reticulum+calcium+ion+concer	0.417328	#N/A	0.417328
BP_GO:0033137_negative+regulation+of+peptidyl-serine+phosphorylation	0.417328	#N/A	0.417328
BP_GO:0033081_regulation+of+T+cell+differentiation+in+the+thymus	0.455388	0.455388	#N/A
MF_GO:0004833_tryptophan+2,3-dioxygenase+activity	0.34916	0.34916	#N/A
MF_GO:0004691_cAMP-dependent+protein+kinase+activity	0.959185	0.959185	#N/A
BP_GO:0009609_response+to+symbiotic+bacterium	0.705977	#N/A	0.705977
BP_GO:0051702_interaction+with+symbiont	0.705977	#N/A	0.705977
BP_GO:0043266_regulation+of+potassium+ion+transport	0.741969	0.741969	#N/A
BP_GO:0051642_centrosome+localization	0.0723988	0.0723988	#N/A
MF_GO:0004477_methenyltetrahydrofolate+cyclohydrolase+activity	0.686847	0.828101	0.398497
MF_GO:0004488_methylenetetrahydrofolate+dehydrogenase+(NADP+)+act	0.686847	0.828101	0.398497
MF_GO:0004727_prenylated+protein+tyrosine+phosphatase+activity	0.693021	#N/A	0.693021
BP_GO:0045401_positive+regulation+of+interleukin-3+biosynthetic+process	0.911821	0.975253	0.479725
BP_GO:0045425_positive+regulation+of+granulocyte+macrophage+colony-s	0.911821	0.975253	0.479725

BP_GO:0002260_lymphocyte+homeostasis	0.385118	0.824544	0.0892257
BP_GO:0015884_folic+acid+transport	0.105607	#N/A	0.105607
BP_GO:0032226_positive+regulation+of+synaptic+transmission,+dopaminer	0.776514	0.800472	0.592181
BP_GO:0033603_positive+regulation+of+dopamine+secretion	0.776514	0.800472	0.592181
BP_GO:0030828_positive+regulation+of+cGMP+biosynthetic+process	0.829282	0.805854	0.685731
MF_GO:0004704_NF-kappaB-inducing+kinase+activity	0.803728	#N/A	0.803728
MF_GO:0003692_left-handed+Z-DNA+binding	0.877833	0.746759	0.837292
BP_GO:0031396_regulation+of+protein+ubiquitination	0.104627	#N/A	0.104627
MF_GO:0001614_purinergic+nucleotide+receptor+activity	0.526554	0.526554	#N/A
BP_GO:0045010_actin+nucleation	0.579989	0.735093	0.366112
MF_GO:0042978_ornithine+decarboxylase+activator+activity	0.395846	0.281018	0.58186
BP_GO:0031296_B+cell+costimulation	0.820338	0.660115	0.811832
BP_GO:0035054_embryonic+heart+tube+anterior/posterior+pattern+forma	0.363683	#N/A	0.363683
MF_GO:0005128_erythropoietin+receptor+binding	0.946993	0.946993	#N/A
MF_GO:0004145_diamine+N-acetyltransferase+activity	0.551872	#N/A	0.551872
BP_GO:0060024_rhythmic+synaptic+transmission	0.707995	0.581761	0.715253
MF_GO:0004611_phosphoenolpyruvate+carboxykinase+activity	0.222065	0.222065	#N/A
MF_GO:0004613_phosphoenolpyruvate+carboxykinase+(GTP)+activity	0.222065	0.222065	#N/A
BP_GO:0001954_positive+regulation+of+cell-matrix+adhesion	0.48376	0.552903	0.424616
MF_GO:0019807_aspartoacylase+activity	0.415349	#N/A	0.415349
BP_GO:0009590_detection+of+gravity	0.806914	0.806914	#N/A
BP_GO:0002227_innate+immune+response+in+mucosa	0.013503	0.013503	#N/A
BP_GO:0048318_axial+mesoderm+development	0.0509966	0.0728472	0.195381
BP_GO:0043547_positive+regulation+of+GTPase+activity	0.126888	0.126888	#N/A
CC_GO:0031091_platelet+alpha+granule	0.868678	0.868678	#N/A
BP_GO:0051128_regulation+of+cellular+component+organization	0.18074	0.0824901	0.538999
BP_GO:0021591_ventricular+system+development	0.719148	0.719148	#N/A
MF_GO:0001888_glucuronyl-galactosyl-proteoglycan+4-alpha-N-acetylgluco	0.898912	0.976577	0.427515
BP_GO:0050794_regulation+of+cellular+process	0.99585	0.99585	#N/A
CC_GO:0005968_Rab-protein+geranylgeranyltransferase+complex	0.130318	0.133849	0.31467
BP_GO:0007033_vacuole+organization	0.768927	0.959003	0.242478
BP_GO:0006925_inflammatory+cell+apoptosis	0.984524	0.990955	0.754753
CC_GO:0000805_X+chromosome	0.267149	0.267149	#N/A
CC_GO:0000806_Y+chromosome	0.267149	0.267149	#N/A
MF_GO:0035005_phosphatidylinositol-4-phosphate+3-kinase+activity	0.0920896	0.698268	0.00824627
BP_GO:0051875_pigment+granule+localization	0.157132	0.0391994	0.631859
BP_GO:0051904_pigment+granule+transport	0.157132	0.0391994	0.631859
BP_GO:0001780_neutrophil+homeostasis	0.547513	0.333078	0.72603
BP_GO:0006828_manganese+ion+transport	0.301148	0.178753	0.572743
MF_GO:0004765_shikimate+kinase+activity	0.502586	0.481939	0.52192
MF_GO:0050509_N-acetylglucosaminyl-proteoglycan+4-beta-glucuronosyltr	0.345528	0.345528	#N/A
BP_GO:0048478_replication+fork+protection	0.733134	#N/A	0.733134
MF_GO:0004576_oligosaccharyl+transferase+activity	0.351502	#N/A	0.351502
MF_GO:0050839_cell+adhesion+molecule+binding	0.815742	0.968355	0.279504
MF_GO:0004637_phosphoribosylamine-glycine+ligase+activity	0.912562	0.881454	0.769668
BP_GO:0007098_centrosome+cycle	0.691435	0.894918	0.292777
BP_GO:0014054_positive+regulation+of+gamma-aminobutyric+acid+secreti	0.54592	0.54592	#N/A

MF_GO:0003829_beta-1,3-galactosyl-O-glycosyl-glycoprotein+beta-1,6-N-ac	0.308166	0.308166	#N/A
BP_GO:0006788_heme+oxidation	0.541578	0.541578	#N/A
MF_GO:0004392_heme+oxygenase+(decyclizing)+activity	0.541578	0.541578	#N/A
BP_GO:0006534_cysteine+metabolic+process	0.93954	0.93954	#N/A
BP_GO:0050883_musculoskeletal+movement,+spinal+reflex+action	0.656072	0.581761	0.641531
BP_GO:0002513_tolerance+induction+to+self+antigen	0.398913	0.456422	0.400341
BP_GO:0045066_regulatory+T+cell+differentiation	0.398913	0.456422	0.400341
BP_GO:0006029_proteoglycan+metabolic+process	0.0844794	#N/A	0.0844794
BP_GO:0051799_negative+regulation+of+hair+follicle+development	0.147967	#N/A	0.147967
MF_GO:0005026_transforming+growth+factor+beta+receptor+activity,+type	0.588504	0.644723	0.478428
MF_GO:0050321_tau-protein+kinase+activity	0.989775	0.998936	0.582585
BP_GO:0015860_purine+nucleoside+transport	0.370462	0.370462	#N/A
MF_GO:0005415_nucleoside:sodium+symporter+activity	0.370462	0.370462	#N/A
BP_GO:0045739_positive+regulation+of+DNA+repair	0.544776	0.544776	#N/A
BP_GO:0022010_myelination+in+the+central+nervous+system	0.402764	0.402764	#N/A
BP_GO:0033092_positive+regulation+of+immature+T+cell+proliferation+in+	0.19636	0.456422	0.135818
MF_GO:0003870_5-aminolevulinate+synthase+activity	0.229658	#N/A	0.229658
BP_GO:0048730_epidermis+morphogenesis	0.543101	0.543101	#N/A
MF_GO:0050544_arachidonic+acid+binding	0.700994	0.930256	0.232332
MF_GO:0030306_ADP-ribosylation+factor+binding	0.479918	0.252103	0.724796
BP_GO:0019230_proprioception	0.54966	0.824242	0.225256
MF_GO:0031386_protein+tag	0.541429	0.915516	0.109757
MF_GO:0004963_follicle-stimulating+hormone+receptor+activity	0.696052	#N/A	0.696052
BP_GO:0032831_positive+regulation+of+CD4-positive,+CD25-positive,+alpha	0.828991	0.828991	#N/A
BP_GO:0006625_protein+targeting+to+peroxisome	0.126265	0.126265	#N/A
MF_GO:0003827_alpha-1,3-mannosylglycoprotein+2-beta-N-acetylglucosam	0.666349	0.666349	#N/A
BP_GO:0019348_dolichol+metabolic+process	0.262451	#N/A	0.262451
CC_GO:0033185_dolichol-phosphate-mannose+synthase+complex	0.262451	#N/A	0.262451
MF_GO:0004582_dolichyl-phosphate+beta-D-mannosyltransferase+activity	0.262451	#N/A	0.262451
BP_GO:0008612_peptidyl-lysine+modification+to+hypusine	0.409032	0.409032	#N/A
BP_GO:0048199_vesicle+targeting,+to,+from+or+within+Golgi	0.560574	0.746539	0.327269
BP_GO:0008209_androgen+metabolic+process	0.550241	0.816212	0.235191
BP_GO:0006621_protein+retention+in+ER+lumen	0.932903	0.909993	0.781987
MF_GO:0046923_ER+retention+sequence+binding	0.932903	0.909993	0.781987
CC_GO:0005665_DNA-directed+RNA+polymerase+II,+core+complex	0.157463	0.174338	0.314135
BP_GO:0035089_establishment+of+apical/basal+cell+polarity	0.6893	0.880253	0.316684
BP_GO:0001946_lymphangiogenesis	0.124745	#N/A	0.124745
BP_GO:0016926_protein+desumoylation	0.776839	0.776839	#N/A
BP_GO:0009059_macromolecule+biosynthetic+process	0.112179	0.151585	0.245441
BP_GO:0014056_regulation+of+acetylcholine+secretion	0.774305	0.774305	#N/A
MF_GO:0000268_peroxisome+targeting+sequence+binding	0.616126	0.616126	#N/A
MF_GO:0004911_interleukin-2+receptor+activity	0.859964	0.859964	#N/A
BP_GO:0043044_ATP-dependent+chromatin+remodeling	0.648902	0.674173	0.535867
MF_GO:0031491_nucleosome+binding	0.648902	0.674173	0.535867
BP_GO:0046668_regulation+of+retinal+cell+programmed+cell+death	0.941213	0.941213	#N/A
BP_GO:0002224_toll-like+receptor+signaling+pathway	0.84363	0.84363	#N/A
MF_GO:0019215_intermediate+filament+binding	0.134778	0.329405	0.131348

BP_GO:0019307_mannose+biosynthetic+process	0.902901	0.83677	0.804026
MF_GO:0004615_phosphomannomutase+activity	0.902901	0.83677	0.804026
BP_GO:0014031_mesenchymal+cell+development	0.0987309	#N/A	0.0987309
CC_GO:0030892_mitotic+cohesin+complex	0.66148	0.479692	0.739153
MF_GO:0003989_acetyl-CoA+carboxylase+activity	0.203804	0.203804	#N/A
BP_GO:0042036_negative+regulation+of+cytokine+biosynthetic+process	0.963959	0.963959	#N/A
MF_GO:0015056_corticotrophin-releasing+factor+receptor+activity	0.521326	#N/A	0.521326
MF_GO:0045309_protein+phosphorylated+amino+acid+binding	0.525774	#N/A	0.525774
MF_GO:0000774_adenyl-nucleotide+exchange+factor+activity	0.0580525	0.00977008	0.544736
BP_GO:0002790_peptide+secretion	0.353734	0.514607	0.285342
BP_GO:0035282_segmentation	0.197718	0.503054	0.113239
BP_GO:0045667_regulation+of+osteoblast+differentiation	0.414239	0.414239	#N/A
BP_GO:0031017_exocrine+pancreas+development	0.793527	#N/A	0.793527
BP_GO:0043433_negative+regulation+of+transcription+factor+activity	0.176552	0.176552	#N/A
BP_GO:0048537_mucosal-associated+lymphoid+tissue+development	0.603818	0.603818	#N/A
MF_GO:0003884_D-amino-acid+oxidase+activity	0.708891	0.708891	#N/A
BP_GO:0032435_negative+regulation+of+proteasomal+ubiquitin-dependent	0.694434	0.694434	#N/A
MF_GO:0005353_fructose+transmembrane+transporter+activity	0.373354	#N/A	0.373354
BP_GO:0006587_serotonin+biosynthetic+process+from+tryptophan	0.178117	#N/A	0.178117
BP_GO:0042427_serotonin+biosynthetic+process	0.178117	#N/A	0.178117
MF_GO:0004510_tryptophan+5-monooxygenase+activity	0.178117	#N/A	0.178117
BP_GO:0046883_regulation+of+hormone+secretion	0.0415749	0.144574	0.0821129
MF_GO:0004452_isopentenyl-diphosphate+delta-isomerase+activity	0.00191384	0.00191384	#N/A
BP_GO:0034644_cellular+response+to+UV	0.790956	#N/A	0.790956
MF_GO:0019911_structural+constituent+of+myelin+sheath	0.907663	0.907663	#N/A
BP_GO:0043508_negative+regulation+of+JNK+activity	0.855778	0.855778	#N/A
MF_GO:0030272_5-formyltetrahydrofolate+cyclo-ligase+activity	0.764079	0.258372	0.952228
BP_GO:0007258_JUN+phosphorylation	0.400567	0.400567	#N/A
BP_GO:0030328_prenylcysteine+catabolic+process	0.911272	0.859232	0.797224
MF_GO:0016670_oxidoreductase+activity,+acting+on+sulfur+group+of+don	0.911272	0.859232	0.797224
BP_GO:0048619_embryonic+hindgut+morphogenesis	0.808326	0.781085	0.676538
BP_GO:0001504_neurotransmitter+uptake	0.171251	0.364102	0.15987
BP_GO:0006488_dolichol-linked+oligosaccharide+biosynthetic+process	0.66261	#N/A	0.66261
BP_GO:0016577_histone+demethylation	0.509854	0.509854	#N/A
BP_GO:0048711_positive+regulation+of+astrocyte+differentiation	0.0101491	0.0101491	#N/A
BP_GO:0034612_response+to+tumor+necrosis+factor	0.0267218	0.0308118	0.194063
BP_GO:0045143_homologous+chromosome+segregation	0.543122	0.88709	0.145153
MF_GO:0004475_mannose-1-phosphate+guanylyltransferase+activity	0.941573	0.987466	0.491544
BP_GO:0042095_interferon-gamma+biosynthetic+process	0.0386997	0.0137055	0.384965
MF_GO:0043790_dimethyladenosine+transferase+activity	0.509653	0.509653	#N/A
MF_GO:0015036_disulfide+oxidoreductase+activity	0.479984	0.475742	0.496146
MF_GO:0004314_[acyl-carrier-protein]+S-malonyltransferase+activity	0.0474686	0.0211634	0.369995
CC_GO:0002080_acrosomal+membrane	0.365505	#N/A	0.365505
MF_GO:0004320_oleoyl-[acyl-carrier-protein]+hydrolase+activity	0.213946	0.213946	#N/A
CC_GO:0000137_Golgi+cis+cisterna	0.900463	0.402402	0.980519
CC_GO:0005915_zonula+adherens	0.674703	0.889838	0.279504
MF_GO:0030350_iron-responsive+element+binding	0.370648	0.370648	#N/A

MF_GO:0016888_endodeoxyribonuclease+activity,+producing+5'-phosphom	0.975346	0.999478	0.309743
MF_GO:0070052_collagen+V+binding	0.421406	0.256205	0.646186
BP_GO:0045063_T-helper+1+cell+differentiation	0.217321	0.20828	0.384965
MF_GO:0001566_non-kinase+phorbol+ester+receptor+activity	0.69803	#N/A	0.69803
CC_GO:0042588_zymogen+granule	0.975682	0.975682	#N/A
CC_GO:0031233_intrinsic+to+external+side+of+plasma+membrane	0.710816	#N/A	0.710816
BP_GO:0032319_regulation+of+Rho+GTPase+activity	0.0753487	0.73329	0.00395928
MF_GO:0001965_G-protein+alpha-subunit+binding	0.787119	0.813595	0.593349
MF_GO:0042813_Wnt+receptor+activity	0.915458	0.95439	0.601469
MF_GO:0019210_kinase+inhibitor+activity	0.232943	#N/A	0.232943
BP_GO:0006613_cotranslational+protein+targeting+to+membrane	0.906394	#N/A	0.906394
CC_GO:0005784_translocon+complex	0.906394	#N/A	0.906394
BP_GO:0009798_axis+specification	0.261359	0.472528	0.20189
MF_GO:0004360_glutamine-fructose-6-phosphate+transaminase+(isomerizi	0.412737	0.434134	0.442112
BP_GO:0055009_atrial+cardiac+muscle+morphogenesis	0.37008	0.329201	0.489394
MF_GO:0004422_hypoxanthine+phosphoribosyltransferase+activity	0.546049	0.507021	0.558273
BP_GO:0015746_citrate+transport	0.975624	0.959035	0.852965
MF_GO:0015137_citrate+transmembrane+transporter+activity	0.975624	0.959035	0.852965
BP_GO:0000076_DNA+replication+checkpoint	0.540684	0.645503	0.409763
BP_GO:0040009_regulation+of+growth+rate	0.649555	0.649555	#N/A
BP_GO:0009088_threonine+biosynthetic+process	0.116029	0.116029	#N/A
MF_GO:0004795_threonine+synthase+activity	0.116029	0.116029	#N/A
BP_GO:0048547_gut+morphogenesis	0.211276	0.583688	0.0892257
BP_GO:0010592_positive+regulation+of+lamellipodium+assembly	0.133268	0.175711	0.261198
BP_GO:0045136_development+of+secondary+sexual+characteristics	0.500027	#N/A	0.500027
BP_GO:0060058_positive+regulation+of+apoptosis+involved+in+mammary+	0.500027	#N/A	0.500027
BP_GO:0043067_regulation+of+programmed+cell+death	0.113506	#N/A	0.113506
BP_GO:0018193_peptidyl-amino+acid+modification	0.881506	0.965436	0.442774
BP_GO:0043043_peptide+biosynthetic+process	0.58595	0.58595	#N/A
BP_GO:0006796_phosphate+metabolic+process	0.710351	#N/A	0.710351
BP_GO:0046950_cellular+ketone+body+metabolic+process	0.439936	0.439936	#N/A
MF_GO:0008260_3-oxoacid+CoA-transferase+activity	0.439936	0.439936	#N/A
BP_GO:0006119_oxidative+phosphorylation	0.3011	0.507021	0.225256
BP_GO:0042249_establishment+of+polarity+of+embryonic+epithelium	0.0450954	0.0131941	0.43001
BP_GO:0002031_G-protein+coupled+receptor+internalization	0.616184	0.807541	0.326245
BP_GO:0046671_negative+regulation+of+retinal+cell+programmed+cell+dea	0.0900993	#N/A	0.0900993
CC_GO:0016533_cyclin-dependent+protein+kinase+5+activator+complex	0.413863	#N/A	0.413863
MF_GO:0016534_cyclin-dependent+protein+kinase+5+activator+activity	0.413863	#N/A	0.413863
MF_GO:0034211_GTP-dependent+protein+kinase+activity	0.517673	#N/A	0.517673
MF_GO:0004534_5'-3'+exoribonuclease+activity	0.536944	0.531097	0.521412
BP_GO:0045627_positive+regulation+of+T-helper+1+cell+differentiation	0.316233	#N/A	0.316233
CC_GO:0030056_hemidesmosome	0.894096	0.894096	#N/A
MF_GO:0008177_succinate+dehydrogenase+(ubiquinone)+activity	0.977598	0.830728	0.970106
BP_GO:0021587_cerebellum+morphogenesis	0.41877	0.283879	0.610939
BP_GO:0007354_zygotic+determination+of+anterior/posterior+axis,+embry	0.850325	0.850325	#N/A
BP_GO:0001705_ectoderm+formation	0.697354	0.697354	#N/A
BP_GO:0015696_ammonium+transport	0.438269	#N/A	0.438269

MF_GO:0005143_interleukin-12+receptor+binding	0.735767	0.494731	0.817419
BP_GO:0045581_negative+regulation+of+T+cell+differentiation	0.95491	#N/A	0.95491
BP_GO:0000720_pyrimidine+dimer+repair+via+nucleotide-excision+repair	0.435388	0.384769	0.52525
MF_GO:0004909_interleukin-1,+Type+I,+activating+receptor+activity	0.0840952	0.251567	0.100335
BP_GO:0010216_maintenance+of+DNA+methylation	0.618849	#N/A	0.618849
BP_GO:0030431_sleep	0.0225498	0.0225498	#N/A
BP_GO:0022612_gland+morphogenesis	0.391886	0.83092	0.0892257
BP_GO:0045916_negative+regulation+of+complement+activation	0.23957	0.23957	#N/A
BP_GO:0006200_ATP+catabolic+process	0.848835	0.848835	#N/A
MF_GO:0016492_neurotensin+receptor+activity,+G-protein+coupled	0.533724	0.263101	0.774597
MF_GO:0005123_death+receptor+binding	0.0704747	0.103886	0.205309
BP_GO:0015888_thiamin+transport	0.466531	0.7634	0.201688
MF_GO:0015234_thiamin+transmembrane+transporter+activity	0.466531	0.7634	0.201688
BP_GO:0007052_mitotic+spindle+organization	0.0682752	#N/A	0.0682752
MF_GO:0004043_L-aminoadipate-semialdehyde+dehydrogenase+activity	0.637412	0.637412	#N/A
BP_GO:0006893_Golgi+to+plasma+membrane+transport	0.777823	0.938066	0.324173
MF_GO:0015270_dihydropyridine-sensitive+calcium+channel+activity	0.304659	0.304659	#N/A
BP_GO:0045821_positive+regulation+of+glycolysis	0.325413	#N/A	0.325413
MF_GO:0005176_ErbB-2+class+receptor+binding	0.299729	0.286749	0.428721
BP_GO:0006944_membrane+fusion	0.256847	#N/A	0.256847
MF_GO:0008432_JUN+kinase+binding	0.997518	0.997518	#N/A
MF_GO:0042809_vitamin+D+receptor+binding	0.447333	0.759565	0.186271
MF_GO:0045523_interleukin-27+receptor+binding	0.587281	0.587281	#N/A
MF_GO:0050062_long-chain-fatty-acyl-CoA+reductase+activity	0.98695	0.981183	0.85754
BP_GO:0060060_post-embryonic+retina+morphogenesis+in+camera-type+e	0.364137	0.364137	#N/A
MF_GO:0070064_proline-rich+region+binding	0.825034	0.908557	0.496444
BP_GO:0006370_mRNA+capping	0.989266	0.936239	0.958205
BP_GO:0008089_anterograde+axon+cargo+transport	0.921235	0.937827	0.678505
BP_GO:0048295_positive+regulation+of+isotype+switching+to+IgE+isotypes	0.214344	0.276517	0.299432
MF_GO:0005134_interleukin-2+receptor+binding	0.970011	0.684357	0.98544
MF_GO:0015501_glutamate:sodium+symporter+activity	0.747279	0.711686	0.649666
BP_GO:0031848_protection+from+non-homologous+end+joining+at+telome	0.495196	0.495196	#N/A
BP_GO:0015889_cobalamin+transport	0.493783	0.493783	#N/A
BP_GO:0042048_olfactory+behavior	0.225531	0.667865	0.0668318
CC_GO:0017177_alpha-glucosidase+II+complex	0.994943	0.994943	#N/A
BP_GO:0031630_regulation+of+synaptic+vesicle+fusion+to+presynaptic+me	0.78336	#N/A	0.78336
MF_GO:0004307_ethanolaminephosphotransferase+activity	0.575015	#N/A	0.575015
BP_GO:0051295_establishment+of+meiotic+spindle+localization	0.979812	0.979812	#N/A
BP_GO:0032330_regulation+of+chondrocyte+differentiation	0.848132	0.95723	0.395974
BP_GO:0006398_histone+mRNA+3'-end+processing	0.438606	0.158117	0.78352
BP_GO:0032753_positive+regulation+of+interleukin-4+production	0.931006	0.928156	0.737941
BP_GO:0047484_regulation+of+response+to+osmotic+stress	0.868503	#N/A	0.868503
MF_GO:0042800_histone+lysine+N-methyltransferase+activity+(H3-K4+spec	0.460642	0.460642	#N/A
BP_GO:0042594_response+to+starvation	0.535757	0.924213	0.0956909
BP_GO:0015858_nucleoside+transport	0.761685	0.59103	0.781513
MF_GO:0003823_antigen+binding	0.272871	#N/A	0.272871
BP_GO:0042267_natural+killer+cell+mediated+cytotoxicity	0.601328	0.559681	0.584596

BP_GO:0006659_phosphatidylserine+biosynthetic+process	0.967346	0.542018	0.993839
BP_GO:0006429_leucyl-tRNA+aminoacylation	0.504577	0.504577	#N/A
MF_GO:0004823_leucine-tRNA+ligase+activity	0.504577	0.504577	#N/A
BP_GO:0045742_positive+regulation+of+epidermal+growth+factor+recepto	0.137363	#N/A	0.137363
MF_GO:0017057_6-phosphogluconolactonase+activity	0.610515	0.610515	#N/A
BP_GO:0030147_natriuresis	0.87993	0.925754	0.58612
MF_GO:0017077_oxidative+phosphorylation+uncoupler+activity	0.126132	0.126132	#N/A
MF_GO:0008746_NAD(P)+transhydrogenase+activity	0.384636	0.22991	0.627274
MF_GO:0008750_NAD(P)++transhydrogenase+(AB-specific)+activity	0.384636	0.22991	0.627274
BP_GO:0051302_regulation+of+cell+division	0.205597	0.205597	#N/A
BP_GO:0007042_lysosomal+lumen+acidification	0.130036	0.566148	0.0392464
MF_GO:0008339_MP+kinase+activity	0.720055	0.895273	0.333711
BP_GO:0006301_postreplication+repair	0.749628	0.749628	#N/A
MF_GO:0051864_histone+demethylase+activity+(H3-K36+specific)	0.753143	0.937985	0.284604
BP_GO:0016540_protein+autoprocessing	0.792205	0.796181	0.627138
MF_GO:0004310_farnesyl-diphosphate+farnesyltransferase+activity	0.129333	0.129333	#N/A
MF_GO:0004329_formate-tetrahydrofolate+ligase+activity	0.79809	0.79809	#N/A
MF_GO:0031489_myosin+V+binding	0.189786	0.0571134	0.631859
BP_GO:0001839_neural+plate+morphogenesis	0.079777	#N/A	0.079777
MF_GO:0042289_MHC+class+II+protein+binding	0.931874	#N/A	0.931874
BP_GO:0016048_detection+of+temperature+stimulus	0.164914	0.243681	0.247085
BP_GO:0050906_detection+of+stimulus+involved+in+sensory+perception	0.0382666	#N/A	0.0382666
BP_GO:0007589_body+fluid+secretion	0.307371	#N/A	0.307371
BP_GO:0042773_ATP+synthesis+coupled+electron+transport	0.476333	0.476333	#N/A
BP_GO:0035117_embryonic+arm+morphogenesis	0.936382	0.936382	#N/A
BP_GO:0009296_flagellum+assembly	0.192675	#N/A	0.192675
CC_GO:0031514_motile+secondary+cilium	0.192675	#N/A	0.192675
BP_GO:0030910_olfactory+placode+formation	0.280131	0.43582	0.253987
BP_GO:0043537_negative+regulation+of+blood+vessel+endothelial+cell+mi	0.954836	0.893767	0.874926
BP_GO:0030330_DNA+damage+response,+signal+transduction+by+p53+cla	0.885525	#N/A	0.885525
BP_GO:0019478_D-amino+acid+catabolic+process	0.347807	#N/A	0.347807
BP_GO:0032225_regulation+of+synaptic+transmission,+dopaminergic	0.779124	#N/A	0.779124
CC_GO:0000164_protein+phosphatase+type+1+complex	0.135365	0.135365	#N/A
BP_GO:0002358_B+cell+homeostatic+proliferation	0.409151	0.254556	0.631452
BP_GO:0046013_regulation+of+T+cell+homeostatic+proliferation	0.818775	0.818775	#N/A
BP_GO:0021510_spinal+cord+development	0.754585	0.754585	#N/A
BP_GO:0007320_insemination	0.702533	0.702533	#N/A
MF_GO:0008171_O-methyltransferase+activity	0.941697	0.95675	0.693755
MF_GO:0004535_poly(A)-specific+ribonuclease+activity	0.258101	0.31372	0.332619
MF_GO:0019767_IgE+receptor+activity	0.838473	0.975253	0.285818
BP_GO:0006083_acetate+metabolic+process	0.815152	0.685164	0.784507
BP_GO:0042129_regulation+of+T+cell+proliferation	0.878494	0.823582	0.765232
BP_GO:0048863_stem+cell+differentiation	0.288758	0.315369	0.379462
CC_GO:0045298_tubulin+complex	0.52738	0.52738	#N/A
BP_GO:0050862_positive+regulation+of+T+cell+receptor+signaling+pathwa	0.514737	0.514737	#N/A
BP_GO:0005989_lactose+biosynthetic+process	0.0744877	#N/A	0.0744877
BP_GO:0030516_regulation+of+axon+extension	0.549974	0.549974	#N/A

BP_GO:0032924_activin+receptor+signaling+pathway	0.868565	0.814961	0.754368
MF_GO:0017002_activin+receptor+activity	0.868565	0.814961	0.754368
MF_GO:0030515_snoRNA+binding	0.22099	#N/A	0.22099
BP_GO:0045124_regulation+of+bone+resorption	0.764191	0.764191	#N/A
MF_GO:0003835_beta-galactoside+alpha-2,6-sialyltransferase+activity	0.357667	0.725882	0.132234
MF_GO:0019207_kinase+regulator+activity	0.967669	0.909529	0.899149
BP_GO:0043569_negative+regulation+of+insulin-like+growth+factor+recept	0.410372	0.410372	#N/A
BP_GO:0045076_regulation+of+interleukin-2+biosynthetic+process	0.953668	0.951176	0.765232
CC_GO:0030893_meiotic+cohesin+complex	0.372721	0.600478	0.237796
BP_GO:0043297_apical+junction+assembly	0.835581	0.835581	#N/A
CC_GO:0005838_proteasome+regulatory+particle	0.976432	0.955967	0.865075
CC_GO:0032998_Fc-epsilon+receptor+I+complex	0.0884896	0.0894655	0.285818
MF_GO:0004918_interleukin-8+receptor+activity	0.71985	0.845122	0.424178
BP_GO:0042487_regulation+of+odontogenesis+of+dentine-containing+tootl	0.388178	0.724943	0.158899
CC_GO:0000783_nuclear+telomere+cap+complex	0.822115	0.822115	#N/A
CC_GO:0005712_chiasma	0.69625	0.69625	#N/A
CC_GO:0031201_SNARE+complex	0.193376	0.193376	#N/A
BP_GO:0032788_saturated+monocarboxylic+acid+metabolic+process	0.143569	0.581876	0.0434315
BP_GO:0032789_unsaturated+monocarboxylic+acid+metabolic+process	0.143569	0.581876	0.0434315
BP_GO:0009063_amino+acid+catabolic+process	0.884893	0.884893	#N/A
BP_GO:0046666_retinal+cell+programmed+cell+death	0.82185	#N/A	0.82185
MF_GO:0004525_ribonuclease+III+activity	0.605143	0.605143	#N/A
MF_GO:0033842_N-acetyl-beta-glucosaminyl-glycoprotein+4-beta-N-acetylg	0.952273	0.952273	#N/A
BP_GO:0021912_regulation+of+transcription+from+RNA+polymerase+II+prc	0.334694	0.334694	#N/A
BP_GO:0045686_negative+regulation+of+glial+cell+differentiation	0.334694	0.334694	#N/A
BP_GO:0045687_positive+regulation+of+glial+cell+differentiation	0.334694	0.334694	#N/A
BP_GO:0006703_estrogen+biosynthetic+process	0.929821	0.872034	0.829094
BP_GO:0035129_post-embryonic+hindlimb+morphogenesis	0.854346	0.955319	0.418697
BP_GO:0050878_regulation+of+body+fluid+levels	0.628759	0.726633	0.445407
MF_GO:0003720_telomerase+activity	0.605399	0.605399	#N/A
BP_GO:0021524_visceral+motor+neuron+differentiation	0.239608	#N/A	0.239608
BP_GO:0050801_ion+homeostasis	0.920547	#N/A	0.920547
CC_GO:0005785_signal+recognition+particle+receptor+complex	0.569806	0.883843	0.172314
CC_GO:0016327_apicolateral+plasma+membrane	0.733299	0.731192	0.604607
BP_GO:0009912_auditory+receptor+cell+fate+commitment	0.902696	0.789608	0.848638
BP_GO:0060023_soft+palate+development	0.55211	0.636247	0.435418
MF_GO:0016299_regulator+of+G-protein+signaling+activity	0.301304	0.15659	0.607359
BP_GO:0046068_cGMP+metabolic+process	0.384491	0.384491	#N/A
CC_GO:0005742_mitochondrial+outer+membrane+translocase+complex	0.455811	0.706231	0.242297
MF_GO:0004569_glycoprotein+endo-alpha-1,2-mannosidase+activity	0.769425	0.751681	0.641849
BP_GO:0006977_DNA+damage+response,+signal+transduction+by+p53+clas	0.19773	0.19773	#N/A
MF_GO:0043398_HLH+domain+binding	0.557665	0.557665	#N/A
MF_GO:0004523_ribonuclease+H+activity	0.616476	0.792883	0.34575
BP_GO:0006168_adenine+salvage	0.483527	0.418975	0.558273
BP_GO:0046083_adenine+metabolic+process	0.483527	0.418975	0.558273
BP_GO:0007622_rhythmic+behavior	0.438836	0.438836	#N/A
BP_GO:0021978_telencephalon+regionalization	0.651556	0.816212	0.363398

BP_GO:0051482_elevation+of+cytosolic+calcium+ion+concentration+during	0.714642	0.720777	0.58612
BP_GO:0016198_axon+choice+point+recognition	0.394447	#N/A	0.394447
CC_GO:0030688_preribosome,+small+subunit+precursor	0.088358	0.224509	0.12429
BP_GO:0045793_positive+regulation+of+cell+size	0.908763	0.908763	#N/A
BP_GO:0021506_anterior+neuropore+closure	0.276276	0.530203	0.179937
BP_GO:0007468_regulation+of+rhodopsin+gene+expression	0.127046	0.715044	0.0145821
MF_GO:0008481_sphinganine+kinase+activity	0.894851	#N/A	0.894851
CC_GO:0005852_eukaryotic+translation+initiation+factor+3+complex	0.687139	0.54761	0.715997
BP_GO:0045872_positive+regulation+of+rhodopsin+gene+expression	0.459581	0.979234	0.0145821
BP_GO:0048671_negative+regulation+of+collateral+sprouting	0.327334	0.327334	#N/A
BP_GO:0045950_negative+regulation+of+mitotic+recombination	0.768346	0.817458	0.55269
BP_GO:0021670_lateral+ventricle+development	0.700437	0.700437	#N/A
BP_GO:0048659_smooth+muscle+cell+proliferation	0.572813	0.77476	0.310505
BP_GO:0033088_negative+regulation+of+immature+T+cell+proliferation+in-	0.44194	0.546524	0.373343
BP_GO:0051932_synaptic+transmission,+GABAergic	0.775255	0.775255	#N/A
BP_GO:0040036_regulation+of+fibroblast+growth+factor+receptor+signaling	0.391564	0.729095	0.158899
MF_GO:0005166_neurotrophin+p75+receptor+binding	0.101164	0.511788	0.0334087
BP_GO:0030520_estrogen+receptor+signaling+pathway	0.703463	0.703463	#N/A
BP_GO:0048732_gland+development	0.780938	0.283344	0.952587
BP_GO:0021754_facial+nucleus+development	0.353652	0.176212	0.655113
BP_GO:0016071_mRNA+metabolic+process	0.406196	0.559681	0.313679
MF_GO:0015204_urea+transmembrane+transporter+activity	0.805318	0.805318	#N/A
BP_GO:0019276_UDP-N-acetylgalactosamine+metabolic+process	0.783697	0.84009	0.546165
MF_GO:0008376_acetylgalactosaminyltransferase+activity	0.783697	0.84009	0.546165
BP_GO:0045079_negative+regulation+of+chemokine+biosynthetic+process	0.297969	0.297969	#N/A
MF_GO:0030060_L-malate+dehydrogenase+activity	0.362398	0.362398	#N/A
MF_GO:0005185_neurohypophyseal+hormone+activity	0.997521	0.997521	#N/A
BP_GO:0031571_G1+DNA+damage+checkpoint	0.687347	0.452626	0.791167
BP_GO:0032922_circadian+regulation+of+gene+expression	0.899218	0.925754	0.641531
BP_GO:0060160_negative+regulation+of+dopamine+receptor+signaling+pat	0.899218	0.925754	0.641531
BP_GO:0021775_smoothened+signaling+pathway+involved+in+ventral+spin	0.0416836	0.0197821	0.347901
BP_GO:0021776_smoothened+signaling+pathway+involved+in+spinal+cord+	0.0416836	0.0197821	0.347901
BP_GO:0035295_tube+development	0.0416836	0.0197821	0.347901
BP_GO:0042270_protection+from+natural+killer+cell+mediated+cytotoxicity	0.0520086	0.0520086	#N/A
MF_GO:0008518_reduced+folate+carrier+activity	0.0834644	0.131485	0.201688
MF_GO:0047485_protein+N-terminus+binding	0.198228	0.50463	0.112979
MF_GO:0045294_alpha-catenin+binding	0.74716	0.406289	0.880863
MF_GO:0004666_prostaglandin-endoperoxide+synthase+activity	0.280055	0.591845	0.145442
BP_GO:0032494_response+to+peptidoglycan	0.930483	0.930483	#N/A
BP_GO:0032874_positive+regulation+of+stress-activated+MAPK+cascade	0.930483	0.930483	#N/A
BP_GO:0022038_corpus+callosum+development	0.599089	0.599089	#N/A
MF_GO:0005113_patched+binding	0.734381	0.734381	#N/A
BP_GO:0006104_succinyl-CoA+metabolic+process	0.604743	0.604743	#N/A
BP_GO:0006431_methionyl-tRNA+aminoacylation	0.747547	0.605944	0.750212
MF_GO:0004825_methionine-tRNA+ligase+activity	0.747547	0.605944	0.750212
BP_GO:0006601_creatine+biosynthetic+process	0.114976	0.21839	0.178696
CC_GO:0016580_Sin3+complex	0.213961	#N/A	0.213961

MF_GO:0046404_ATP-dependent+polydeoxyribonucleotide+5'-hydroxyl-kin:	0.738325	0.738325	#N/A
MF_GO:0019153_protein-disulfide+reductase+(glutathione)+activity	0.266617	0.400108	0.265009
BP_GO:0001881_receptor+recycling	0.250527	0.692695	0.0728421
CC_GO:0005682_snRNP+U5	0.965642	0.965642	#N/A
BP_GO:0032635_interleukin-6+production	0.227624	0.227624	#N/A
BP_GO:0007020_microtubule+nucleation	0.24122	#N/A	0.24122
MF_GO:0001607_neuromedin+U+receptor+activity	0.241816	0.272293	0.350245
MF_GO:0047522_15-oxoprostaglandin+13-oxidase+activity	0.583943	0.715281	0.394184
MF_GO:0004921_interleukin-11+receptor+activity	0.819693	0.819693	#N/A
MF_GO:0019970_interleukin-11+binding	0.819693	0.819693	#N/A
BP_GO:0055014_atrial+cardiac+muscle+cell+development	0.529545	0.529545	#N/A
MF_GO:0032405_MutLalpha+complex+binding	0.193252	0.193252	#N/A
MF_GO:0032407_MutSalpha+complex+binding	0.193252	0.193252	#N/A
MF_GO:0004103_choline+kinase+activity	0.148014	0.148014	#N/A
BP_GO:0045910_negative+regulation+of+DNA+recombination	0.728739	0.728739	#N/A
CC_GO:0032301_MutSalpha+complex	0.728739	0.728739	#N/A
CC_GO:0005673_transcription+factor+TFIIE+complex	0.573142	#N/A	0.573142
BP_GO:0016255_attachment+of+GPI+anchor+to+protein	0.400534	#N/A	0.400534
BP_GO:0008037_cell+recognition	0.524988	0.524988	#N/A
BP_GO:0051606_detection+of+stimulus	0.524988	0.524988	#N/A
MF_GO:0033754_indoleamine+2,3-dioxygenase+activity	0.921334	0.921334	#N/A
CC_GO:0019028_viral+capsid	0.332179	0.48481	0.282541
MF_GO:0004649_poly(ADP-ribose)+glycohydrolase+activity	0.930685	0.930685	#N/A
MF_GO:0019888_protein+phosphatase+regulator+activity	0.954424	0.954424	#N/A
MF_GO:0005095_GTPase+inhibitor+activity	0.911698	#N/A	0.911698
BP_GO:0007021_tubulin+complex+assembly	0.886165	0.886165	#N/A
BP_GO:0006433_prolyl-tRNA+aminoacylation	0.926625	0.657759	0.950248
MF_GO:0004827_proline-tRNA+ligase+activity	0.926625	0.657759	0.950248
MF_GO:0019212_phosphatase+inhibitor+activity	0.767131	0.767131	#N/A
MF_GO:0001785_prostaglandin+J+receptor+activity	0.680657	0.565742	0.691213
MF_GO:0004956_prostaglandin+D+receptor+activity	0.680657	0.565742	0.691213
MF_GO:0043566_structure-specific+DNA+binding	0.186457	0.817458	0.0151603
BP_GO:0010454_negative+regulation+of+cell+fate+commitment	0.343303	0.396108	0.379462
CC_GO:0055037_recycling+endosome	0.59765	0.397986	0.728714
MF_GO:0047196_long-chain-alcohol+O-fatty-acyltransferase+activity	0.887187	0.887187	#N/A
BP_GO:0008634_negative+regulation+of+survival+gene+product+expression	0.0266255	#N/A	0.0266255
MF_GO:0035240_dopamine+binding	0.979762	0.979762	#N/A
BP_GO:0015819_lysine+transport	0.651264	0.906018	0.221806
MF_GO:0015189_L-lysine+transmembrane+transporter+activity	0.651264	0.906018	0.221806
BP_GO:0006047_UDP-N-acetylglucosamine+metabolic+process	0.873575	0.873575	#N/A
MF_GO:0004174_electron-transferring-flavoprotein+dehydrogenase+activit	0.713603	0.514533	0.776962
BP_GO:0008105_asymmetric+protein+localization	0.51014	0.51014	#N/A
BP_GO:0050974_detection+of+mechanical+stimulus+involved+in+sensory+p	0.940928	0.907848	0.811587
MF_GO:0004816_asparagine-tRNA+ligase+activity	0.378331	#N/A	0.378331
BP_GO:0022405_hair+cycle+process	0.713606	0.842226	0.418697
BP_GO:0048496_maintenance+of+organ+identity	0.0139724	0.00341559	0.342942
MF_GO:0003913_DNA+photolyase+activity	0.665344	0.755097	0.465801

MF_GO:0003836_beta-galactoside+alpha-2,3-sialyltransferase+activity	0.670536	0.694369	0.54647
BP_GO:0045737_positive+regulation+of+cyclin-dependent+protein+kinase+a	0.124329	0.479697	0.0569656
BP_GO:0021937_Purkinje+cell-granule+cell+precursor+cell+signaling+involve	0.705027	0.642467	0.654661
MF_GO:0003863_3-methyl-2-oxobutanoate+dehydrogenase+(2-methylprop	0.893042	0.893042	#N/A
MF_GO:0033691_sialic+acid+binding	0.563293	0.898981	0.146898
BP_GO:0000244_assembly+of+spliceosomal+tri-snRNP	0.385484	#N/A	0.385484
MF_GO:0015220_choline+transmembrane+transporter+activity	0.451637	0.451637	#N/A
MF_GO:0005146_leukemia+inhibitory+factor+receptor+binding	0.554627	0.534145	0.543463
BP_GO:0030917_midbrain-hindbrain+boundary+development	0.894033	0.658572	0.912777
MF_GO:0008332_low+voltage-gated+calcium+channel+activity	0.234153	#N/A	0.234153
BP_GO:0021526_medial+motor+column+neuron+differentiation	0.982025	0.982025	#N/A
BP_GO:0010155_regulation+of+proton+transport	0.911705	0.911705	#N/A
BP_GO:0045851_pH+reduction	0.911705	0.911705	#N/A
MF_GO:0008120_ceramide+glucosyltransferase+activity	0.385397	0.385397	#N/A
MF_GO:0004045_aminoacyl-tRNA+hydrolase+activity	0.824913	0.830192	0.643346
BP_GO:0032320_positive+regulation+of+Ras+GTPase+activity	0.569123	0.488703	0.608426
BP_GO:0048570_notochord+morphogenesis	0.777374	0.842475	0.530237
BP_GO:0051348_negative+regulation+of+transferase+activity	0.821532	0.835909	0.627833
BP_GO:0051051_negative+regulation+of+transport	0.77345	0.77345	#N/A
BP_GO:0006903_vesicle+targeting	0.45359	#N/A	0.45359
BP_GO:0043001_Golgi+to+plasma+membrane+protein+transport	0.290729	0.711535	0.0905615
BP_GO:0032100_positive+regulation+of+appetite	0.759624	0.759624	#N/A
CC_GO:0044459_plasma+membrane+part	0.283225	#N/A	0.283225
BP_GO:0035110_leg+morphogenesis	0.726388	0.726388	#N/A
BP_GO:0006054_N-acetylneuraminate+metabolic+process	0.806393	0.806393	#N/A
MF_GO:0016176_superoxide-generating+NADPH+oxidase+activator+activity	0.326442	0.721927	0.1104
MF_GO:0016520_growth+hormone-releasing+hormone+receptor+activity	0.847044	0.929759	0.490067
BP_GO:0043517_positive+regulation+of+DNA+damage+response,+signal+tra	0.37083	#N/A	0.37083
BP_GO:0042346_positive+regulation+of+NF-kappaB+import+into+nucleus	0.456372	0.543878	0.395608
BP_GO:0050954_sensory+perception+of+mechanical+stimulus	0.186192	0.243681	0.285342
CC_GO:0030849_autosome	0.476934	0.574103	0.394286
BP_GO:0006499_N-terminal+protein+myristoylation	0.835037	0.835037	#N/A
BP_GO:0018008_N-terminal+peptidyl-glycine+N-myristoylation	0.835037	0.835037	#N/A
MF_GO:0004379_glycylpeptide+N-tetradecanoyltransferase+activity	0.835037	0.835037	#N/A
BP_GO:0009217_purine+deoxyribonucleoside+triphosphate+catabolic+proc	0.0592753	0.0592753	#N/A
MF_GO:0004169_dolichyl-phosphate-mannose-protein+mannosyltransferas	0.989154	0.989154	#N/A
BP_GO:0006477_protein+amino+acid+sulfation	0.817004	0.817004	#N/A
BP_GO:0032324_molybdopterin+cofactor+biosynthetic+process	0.914856	0.914856	#N/A
MF_GO:0008442_3-hydroxyisobutyrate+dehydrogenase+activity	0.599293	0.602636	0.538318
MF_GO:0004321_fatty-acyl-CoA+synthase+activity	0.791804	0.374336	0.92932
BP_GO:0015840_urea+transport	0.718642	0.718642	#N/A
BP_GO:0002456_T+cell+mediated+immunity	0.682119	0.682119	#N/A
CC_GO:0017101_aminoacyl-tRNA+synthetase+multienzyme+complex	0.899851	0.974732	0.443188
BP_GO:0050973_detection+of+mechanical+stimulus+involved+in+equilibrio	0.0387591	#N/A	0.0387591
BP_GO:0043030_regulation+of+macrophage+activation	0.3533	0.578913	0.232332
BP_GO:0006424_glutamyl-tRNA+aminoacylation	0.787339	0.787339	#N/A
MF_GO:0004818_glutamate-tRNA+ligase+activity	0.787339	0.787339	#N/A

BP_GO:0006890_retrograde+vesicle-mediated+transport,+Golgi+to+ER	0.857676	0.857676	#N/A
MF_GO:0004949_cannabinoid+receptor+activity	0.230408	0.230408	#N/A
MF_GO:0003880_C-terminal+protein+carboxyl+methyltransferase+activity	0.803242	0.972566	0.238176
BP_GO:0033631_cell-cell+adhesion+mediated+by+integrin	0.792694	0.792694	#N/A
BP_GO:0045077_negative+regulation+of+interferon-gamma+biosynthetic+p	0.432318	0.456422	0.447797
BP_GO:0021882_regulation+of+transcription+from+RNA+polymerase+II+prc	0.692457	#N/A	0.692457
BP_GO:0021893_cerebral+cortex+GABAergic+interneuron+fate+commitmer	0.692457	#N/A	0.692457
MF_GO:0004844_uracil+DNA+N-glycosylase+activity	0.00122679	#N/A	0.00122679
CC_GO:0000172_ribonuclease+MRP+complex	0.660875	#N/A	0.660875
MF_GO:0030971_receptor+tyrosine+kinase+binding	0.31589	0.119033	0.692278
BP_GO:0010225_response+to+UV-C	0.867675	#N/A	0.867675
BP_GO:0045814_negative+regulation+of+gene+expression,+epigenetic	0.843835	0.843835	#N/A
BP_GO:0008078_mesodermal+cell+migration	0.64641	0.64641	#N/A
BP_GO:0021533_cell+differentiation+in+hindbrain	0.700743	0.700743	#N/A
BP_GO:0006437_tyrosyl-tRNA+aminoacylation	0.490902	#N/A	0.490902
MF_GO:0004831_tyrosine-tRNA+ligase+activity	0.490902	#N/A	0.490902
BP_GO:0060163_subpallium+neuron+fate+commitment	0.16188	#N/A	0.16188
BP_GO:0006438_valyl-tRNA+aminoacylation	0.274375	0.340757	0.330881
MF_GO:0004832_valine-tRNA+ligase+activity	0.274375	0.340757	0.330881
MF_GO:0004105_choline-phosphate+cytidylyltransferase+activity	0.611167	0.584507	0.574003
BP_GO:0035315_hair+cell+differentiation	0.656804	0.842226	0.332914
MF_GO:0001641_group+II+metabotropic+glutamate+receptor+activity	0.363791	0.276681	0.539981
CC_GO:0005666_DNA-directed+RNA+polymerase+III+complex	0.22301	0.442812	0.175189
BP_GO:0045132_meiotic+chromosome+segregation	0.72701	0.72701	#N/A
BP_GO:0043320_natural+killer+cell+degranulation	0.654202	0.589006	0.631859
BP_GO:0002016_regulation+of+blood+volume+by+renin-angiotensin	0.88424	0.908367	0.641531
MF_GO:0008459_chondroitin+6-sulfotransferase+activity	0.880467	#N/A	0.880467
MF_GO:0034482_chondroitin+2-O-sulfotransferase+activity	0.880467	#N/A	0.880467
MF_GO:0016499_orexin+receptor+activity	0.855356	0.855356	#N/A
BP_GO:0001560_regulation+of+cell+growth+by+extracellular+stimulus	0.989192	0.99298	0.78648
MF_GO:0002046_opsin+binding	0.98533	0.98533	#N/A
MF_GO:0008159_positive+transcription+elongation+factor+activity	0.649944	0.841592	0.324356
BP_GO:0009107_lipoate+biosynthetic+process	0.303493	0.684589	0.113557
BP_GO:0048172_regulation+of+short-term+neuronal+synaptic+plasticity	0.0771446	0.0771446	#N/A
CC_GO:0008328_ionotropic+glutamate+receptor+complex	0.0771446	0.0771446	#N/A
MF_GO:0017056_structural+constituent+of+nuclear+pore	0.410552	0.410552	#N/A
BP_GO:0051546_keratinocyte+migration	0.950741	0.773018	0.943985
BP_GO:0042516_regulation+of+tyrosine+phosphorylation+of+Stat3+protein	0.504025	0.76887	0.235657
BP_GO:0008298_intracellular+mRNA+localization	0.784102	0.784102	#N/A
MF_GO:0004658_propionyl-CoA+carboxylase+activity	0.629336	0.779433	0.381048
MF_GO:0001665_alpha-N-acetylgalactosaminide+alpha-2,6-sialyltransferase	0.103335	0.434487	0.0524915
MF_GO:0004351_glutamate+decarboxylase+activity	0.271288	0.421202	0.253921
BP_GO:0002204_somatic+recombination+of+immunoglobulin+genes+during	0.742873	0.742873	#N/A
BP_GO:0021562_vestibulocochlear+nerve+development	0.48181	0.383165	0.592181
BP_GO:0001306_age-dependent+response+to+oxidative+stress	0.470586	0.835909	0.139698
CC_GO:0031527_filopodium+membrane	0.864665	0.937055	0.511364
CC_GO:0032433_filopodium+tip	0.864665	0.937055	0.511364

BP_GO:0007343_egg+activation	0.0686523	0.277188	0.0654447
BP_GO:0021902_commitment+of+a+neuronal+cell+to+a+specific+type+of+r	0.449362	0.671477	0.26646
BP_GO:0021905_forebrain-midbrain+boundary+formation	0.449362	0.671477	0.26646
BP_GO:0042327_positive+regulation+of+phosphorylation	0.387304	0.180461	0.694616
MF_GO:0004485_methylcrotonoyl-CoA+carboxylase+activity	0.550879	0.550879	#N/A
BP_GO:0021557_oculomotor+nerve+development	0.324029	0.324029	#N/A
BP_GO:0021558_trochlear+nerve+development	0.324029	0.324029	#N/A
BP_GO:0006379_mRNA+cleavage	0.489932	0.340346	0.646661
MF_GO:0004809_tRNA+(guanine-N2-)-methyltransferase+activity	0.937513	0.937513	#N/A
BP_GO:0045116_protein+neddylation	0.216871	0.745117	0.038678
BP_GO:0030046_parallel+actin+filament+bundle+formation	0.0535154	0.0133832	0.474138
BP_GO:0009583_detection+of+light+stimulus	0.832649	0.832649	#N/A
BP_GO:0022402_cell+cycle+process	0.208796	#N/A	0.208796
MF_GO:0004152_dihydroorotate+dehydrogenase+activity	0.144861	0.67944	0.0247901
MF_GO:0004158_dihydroorotate+oxidase+activity	0.144861	0.67944	0.0247901
BP_GO:0048808_male+genitalia+morphogenesis	0.614619	0.614619	#N/A
BP_GO:0030162_regulation+of+proteolysis	0.796164	0.796164	#N/A
BP_GO:0002768_immune+response-regulating+cell+surface+receptor+signa	0.611412	0.611412	#N/A
MF_GO:0008599_protein+phosphatase+type+1+regulator+activity	0.386357	0.250206	0.604768
BP_GO:0009624_response+to+nematode	0.360931	0.611638	0.215723
BP_GO:0031282_regulation+of+guanylate+cyclase+activity	0.702627	0.702627	#N/A
MF_GO:0008048_calcium+sensitive+guanylate+cyclase+activator+activity	0.702627	0.702627	#N/A
MF_GO:0030249_guanylate+cyclase+regulator+activity	0.702627	0.702627	#N/A
BP_GO:0042503_tyrosine+phosphorylation+of+Stat3+protein	0.763944	0.818245	0.543463
BP_GO:0045835_negative+regulation+of+meiosis	0.763944	0.818245	0.543463
BP_GO:0006428_isoleucyl-tRNA+aminoacylation	0.888229	0.888229	#N/A
MF_GO:0004822_isoleucine-tRNA+ligase+activity	0.888229	0.888229	#N/A
BP_GO:0010551_regulation+of+specific+transcription+from+RNA+polymera	0.702625	0.702625	#N/A
BP_GO:0043179_rhythmic+excitation	0.147074	0.0444404	0.58612
BP_GO:0050881_musculoskeletal+movement	0.668648	0.668648	#N/A
MF_GO:0003912_DNA+nucleotidylexotransferase+activity	0.0168281	#N/A	0.0168281
BP_GO:0060012_synaptic+transmission,+glycinergic	0.600493	0.390933	0.738151
BP_GO:0000578_embryonic+axis+specification	0.291671	0.483657	0.231302
CC_GO:0043514_interleukin-12+complex	0.831754	0.675367	0.817419
MF_GO:0033883_pyridoxal+phosphatase+activity	0.657051	0.423616	0.777975
BP_GO:0002223_stimulatory+C-type+lectin+receptor+signaling+pathway	0.341428	0.341428	#N/A
BP_GO:0031573_intra-S+DNA+damage+checkpoint	0.239809	0.239809	#N/A
BP_GO:0032735_positive+regulation+of+interleukin-12+production	0.872712	0.872712	#N/A
BP_GO:0006427_histidyl-tRNA+aminoacylation	0.356021	0.333889	0.463139
MF_GO:0004821_histidine-tRNA+ligase+activity	0.356021	0.333889	0.463139
BP_GO:0001711_endodermal+cell+fate+commitment	0.152019	0.152019	#N/A
BP_GO:0032714_negative+regulation+of+interleukin-5+production	0.62331	0.456422	0.710379
BP_GO:0006558_L-phenylalanine+metabolic+process	0.649689	#N/A	0.649689
BP_GO:0019889_pteridine+metabolic+process	0.649689	#N/A	0.649689
BP_GO:0046146_tetrahydrobiopterin+metabolic+process	0.649689	#N/A	0.649689
MF_GO:0004757_sepiapterin+reductase+activity	0.649689	#N/A	0.649689
BP_GO:0046168_glycerol-3-phosphate+catabolic+process	0.288495	0.27817	0.42059

MF_GO:0004367_glycerol-3-phosphate+dehydrogenase+(NAD+)+activity	0.288495	0.27817	0.42059
BP_GO:0006436_tryptophanyl-tRNA+aminoacylation	0.92595	0.990098	0.388487
MF_GO:0004830_tryptophan-tRNA+ligase+activity	0.92595	0.990098	0.388487
MF_GO:0005326_neurotransmitter+transporter+activity	0.567859	0.567859	#N/A
MF_GO:0005118_sevenless+binding	0.789652	0.789652	#N/A
BP_GO:0010720_positive+regulation+of+cell+development	0.700475	#N/A	0.700475
BP_GO:0046514_ceramide+catabolic+process	0.205068	#N/A	0.205068
BP_GO:0001544_initiation+of+primordial+ovarian+follicle+growth	0.53158	0.53158	#N/A
BP_GO:0001547_antral+ovarian+follicle+growth	0.53158	0.53158	#N/A
BP_GO:0007403_glial+cell+fate+determination	0.13514	0.607825	0.0334087
MF_GO:0047783_corticosterone+18-monooxygenase+activity	0.95983	#N/A	0.95983
MF_GO:0017134_fibroblast+growth+factor+binding	0.167419	0.157752	0.359332
BP_GO:0042255_ribosome+assembly	0.545326	#N/A	0.545326
MF_GO:0004596_peptide+alpha-N-acetyltransferase+activity	0.206977	0.467339	0.141547
BP_GO:0000272_polysaccharide+catabolic+process	0.224168	0.248237	0.347468
BP_GO:0006030_chitin+metabolic+process	0.224168	0.248237	0.347468
BP_GO:0045630_positive+regulation+of+T-helper+2+cell+differentiation	0.690437	0.690437	#N/A
BP_GO:0033058_directional+locomotion	0.875732	0.875732	#N/A
MF_GO:0001632_leukotriene+B4+receptor+activity	0.763774	0.763774	#N/A
BP_GO:0006423_cysteinyl-tRNA+aminoacylation	0.193614	0.118792	0.48333
MF_GO:0004817_cysteine-tRNA+ligase+activity	0.193614	0.118792	0.48333
BP_GO:0055075_potassium+ion+homeostasis	0.708031	0.67124	0.630057
BP_GO:0046960_sensitization	0.664704	0.664704	#N/A
BP_GO:0060292_long+term+synaptic+depression	0.664704	0.664704	#N/A
MF_GO:0001588_dopamine+receptor+coupled+via+Gs	0.664704	0.664704	#N/A
BP_GO:0001573_ganglioside+metabolic+process	0.294172	0.566148	0.175712
BP_GO:0021536_diencephalon+development	0.498657	0.498657	#N/A
BP_GO:0006638_neutral+lipid+metabolic+process	0.654585	0.902241	0.232332
BP_GO:0045741_positive+regulation+of+epidermal+growth+factor+receptor	0.266983	#N/A	0.266983
BP_GO:0002507_tolerance+induction	0.514116	0.514116	#N/A
CC_GO:0005914_spot+adherens+junction	0.558404	0.558404	#N/A
BP_GO:0043567_regulation+of+insulin-like+growth+factor+receptor+signalin	0.598669	#N/A	0.598669
BP_GO:0060133_somatotropin+secreting+cell+development	0.598669	#N/A	0.598669
BP_GO:0042404_thyroid+hormone+catabolic+process	0.609333	#N/A	0.609333
MF_GO:0001639_PLC+activating+metabotropic+glutamate+receptor+activit	0.668907	0.668907	#N/A
MF_GO:0005315_inorganic+phosphate+transmembrane+transporter+activit	0.799516	0.799516	#N/A
BP_GO:0050708_regulation+of+protein+secretion	0.697429	0.43112	0.817411
BP_GO:0046425_regulation+of+JAK-STAT+cascade	0.887181	0.887181	#N/A
BP_GO:0043558_regulation+of+translational+initiation+in+response+to+stre	0.578842	0.376394	0.724725
BP_GO:0032695_negative+regulation+of+interleukin-12+production	0.90537	0.90537	#N/A
MF_GO:0015276_ligand-gated+ion+channel+activity	0.380597	#N/A	0.380597
CC_GO:0032389_MutLalpha+complex	0.808807	0.817458	0.629557
MF_GO:0005131_growth+hormone+receptor+binding	0.822791	#N/A	0.822791
BP_GO:0045608_negative+regulation+of+auditory+receptor+cell+differentia	0.313463	0.313463	#N/A
MF_GO:0001716_L-amino-acid+oxidase+activity	0.859637	0.859637	#N/A
BP_GO:0021568_rhombomere+2+development	0.454809	0.658572	0.28479
CC_GO:0033270_paranode+region+of+axon	0.617448	0.534132	0.632164

BP_GO:0046427_positive+regulation+of+JAK-STAT+cascade	0.740774	0.740774	#N/A
MF_GO:0032183_SUMO+binding	0.027204	#N/A	0.027204
BP_GO:0050847_progesterone+receptor+signaling+pathway	0.220203	#N/A	0.220203
BP_GO:0017145_stem+cell+division	0.767479	#N/A	0.767479
BP_GO:0007567_parturition	0.24051	0.441779	0.197657
MF_GO:0008743_L-threonine+3-dehydrogenase+activity	0.530664	0.530664	#N/A
MF_GO:0005432_calcium:sodium+antiporter+activity	0.287942	0.321549	0.37161
MF_GO:0004948_calcitonin+receptor+activity	0.87677	0.852522	0.72341
MF_GO:0016428_tRNA+(cytosine-5-)-methyltransferase+activity	0.656182	0.843946	0.329451
BP_GO:0045408_regulation+of+interleukin-6+biosynthetic+process	0.987248	0.987248	#N/A
CC_GO:0000214_tRNA-intron+endonuclease+complex	0.726374	0.240215	0.940348
MF_GO:0000213_tRNA-intron+endonuclease+activity	0.726374	0.240215	0.940348
CC_GO:0030880_RNA+polymerase+complex	0.85172	#N/A	0.85172
BP_GO:0043516_regulation+of+DNA+damage+response,+signal+transductio	0.896558	0.896558	#N/A
MF_GO:0004647_phosphoserine+phosphatase+activity	0.261363	0.261363	#N/A
MF_GO:0004366_glycerol-3-phosphate+O-acyltransferase+activity	0.029344	0.029344	#N/A
MF_GO:0005427_proton-dependent+oligopeptide+secondary+active+transr	0.602903	0.602903	#N/A
BP_GO:0021965_spinal+cord+ventral+commissure+morphogenesis	0.345068	#N/A	0.345068
MF_GO:0008486_diphosphoinositol-polyphosphate+diphosphatase+activity	0.15172	#N/A	0.15172
BP_GO:0051297_centrosome+organization	0.261622	0.194807	0.483113
CC_GO:0000930_gamma-tubulin+complex	0.261622	0.194807	0.483113
BP_GO:0006189_'de+novo'+IMP+biosynthetic+process	0.345506	0.0968655	0.769668
BP_GO:0007418_ventral+midline+development	0.210069	0.226893	0.347901
BP_GO:0060032_notochord+regression	0.210069	0.226893	0.347901
BP_GO:0060259_regulation+of+feeding+behavior	0.479314	0.208208	0.770281
MF_GO:0001594_trace-amine+receptor+activity	0.884203	0.952893	0.50777
BP_GO:0050432_catecholamine+secretion	0.84101	0.84101	#N/A
BP_GO:0060126_somatotropin+secreting+cell+differentiation	0.445341	0.283344	0.647652
BP_GO:0006434_seryl-tRNA+aminoacylation	0.889182	#N/A	0.889182
MF_GO:0004828_serine-tRNA+ligase+activity	0.889182	#N/A	0.889182
BP_GO:0050916_sensory+perception+of+sweet+taste	0.369775	#N/A	0.369775
MF_GO:0003730_mRNA+3'-UTR+binding	0.423307	0.807088	0.127062
BP_GO:0048343_paraxial+mesodermal+cell+fate+commitment	0.296486	0.476187	0.2432
MF_GO:0004446_multiple+inositol-polyphosphate+phosphatase+activity	0.206744	0.381763	0.196093
MF_GO:0000702_oxidized+base+lesion+DNA+N-glycosylase+activity	0.0882921	0.483614	0.0307172
MF_GO:0005148_prolactin+receptor+binding	0.459936	0.818345	0.146655
BP_GO:0002443_leukocyte+mediated+immunity	0.199593	0.199593	#N/A
MF_GO:0001540_beta-amyloid+binding	0.204562	#N/A	0.204562
BP_GO:0022605_oogenesis+stage	0.261137	#N/A	0.261137
BP_GO:0045744_negative+regulation+of+G-protein+coupled+receptor+prot	0.551253	#N/A	0.551253
BP_GO:0001781_neutrophil+apoptosis	0.960516	0.960516	#N/A
MF_GO:0008494_translation+activator+activity	0.0459248	#N/A	0.0459248
CC_GO:0000779_condensed+chromosome,+centromeric+region	0.34328	#N/A	0.34328
CC_GO:0031618_nuclear+centromeric+heterochromatin	0.34328	#N/A	0.34328
BP_GO:0007549_dosage+compensation	0.484397	0.48893	0.489196
BP_GO:0007529_establishment+of+synaptic+specificity+at+neuromuscular+	0.434771	0.434771	#N/A
MF_GO:0004910_interleukin-1,+Type+II,+blocking+receptor+activity	0.584129	#N/A	0.584129

MF_GO:0001635_calcitonin+gene-related+polypeptide+receptor+activity	0.799202	#N/A	0.799202
BP_GO:0030327_prenylated+protein+catabolic+process	0.358848	#N/A	0.358848
BP_GO:0045746_negative+regulation+of+Notch+signaling+pathway	0.302012	0.188661	0.559452
MF_GO:0001671_ATPase+activator+activity	0.036943	#N/A	0.036943
MF_GO:0030189_chaperone+activator+activity	0.036943	#N/A	0.036943
BP_GO:0032011_ARF+protein+signal+transduction	0.796241	0.690106	0.750557
MF_GO:0047066_phospholipid-hydroperoxide+glutathione+peroxidase+acti	0.989361	#N/A	0.989361
CC_GO:0005787_signal+peptidase+complex	0.156665	#N/A	0.156665
BP_GO:0000432_positive+regulation+of+transcription+from+RNA+polymera	0.421557	0.421557	#N/A
MF_GO:0004813_alanine-tRNA+ligase+activity	0.401298	0.513052	0.349783
MF_GO:0030569_chymotrypsin+inhibitor+activity	0.969203	#N/A	0.969203
BP_GO:0010519_negative+regulation+of+phospholipase+activity	0.385166	#N/A	0.385166
MF_GO:0004454_ketohexokinase+activity	0.989966	0.989966	#N/A
CC_GO:0033018_sarcoplasmic+reticulum+lumen	0.742897	#N/A	0.742897
BP_GO:0010389_regulation+of+G2/M+transition+of+mitotic+cell+cycle	0.983431	#N/A	0.983431
MF_GO:0017174_glycine+N-methyltransferase+activity	0.644855	0.644855	#N/A
BP_GO:0008292_acetylcholine+biosynthetic+process	0.0787067	#N/A	0.0787067
MF_GO:0005307_choline:sodium+symporter+activity	0.0787067	#N/A	0.0787067
BP_GO:0046952_ketone+body+catabolic+process	0.547404	0.547404	#N/A
MF_GO:0008410_CoA-transferase+activity	0.547404	0.547404	#N/A
MF_GO:0008127_quercetin+2,3-dioxygenase+activity	0.155756	0.155756	#N/A
BP_GO:0046314_phosphocreatine+biosynthetic+process	0.583504	0.583504	#N/A
MF_GO:0003845_11-beta-hydroxysteroid+dehydrogenase+activity	0.280549	0.280549	#N/A
MF_GO:0000334_3-hydroxyanthranilate+3,4-dioxygenase+activity	0.188881	0.188881	#N/A
MF_GO:0004939_beta-adrenergic+receptor+activity	0.719949	0.719949	#N/A
MF_GO:0031699_beta-3+adrenergic+receptor+binding	0.719949	0.719949	#N/A
MF_GO:0004846_urate+oxidase+activity	0.00979482	0.00979482	#N/A
BP_GO:0006741_NADP+biosynthetic+process	0.50174	0.50174	#N/A
MF_GO:0051370_ZASP+binding	0.290482	#N/A	0.290482
BP_GO:0034370_triglyceride-rich+lipoprotein+particle+remodeling	0.00186829	0.00186829	#N/A
BP_GO:0060332_positive+regulation+of+response+to+interferon-gamma	0.438691	0.438691	#N/A
BP_GO:0060335_positive+regulation+of+interferon-gamma-mediated+signa	0.438691	0.438691	#N/A
MF_GO:0030429_kynureninase+activity	0.822022	0.822022	#N/A
BP_GO:0030832_regulation+of+actin+filament+length	0.288749	0.288749	#N/A
BP_GO:0015939_pantothenate+metabolic+process	0.151445	0.151445	#N/A
BP_GO:0006084_acetyl-CoA+metabolic+process	0.190971	#N/A	0.190971
MF_GO:0004996_thyroid-stimulating+hormone+receptor+activity	0.51237	#N/A	0.51237
BP_GO:0045721_negative+regulation+of+gluconeogenesis	0.448183	#N/A	0.448183
BP_GO:0045923_positive+regulation+of+fatty+acid+metabolic+process	0.448183	#N/A	0.448183
MF_GO:0004055_argininosuccinate+synthase+activity	0.111854	0.111854	#N/A
BP_GO:0042360_vitamin+E+metabolic+process	0.124004	#N/A	0.124004
MF_GO:0008431_vitamin+E+binding	0.124004	#N/A	0.124004
MF_GO:0003983_UTP:glucose-1-phosphate+uridylyltransferase+activity	0.587423	0.587423	#N/A
BP_GO:0032570_response+to+progesterone+stimulus	0.690492	#N/A	0.690492
BP_GO:0033484_nitric+oxide+homeostasis	0.690492	#N/A	0.690492
BP_GO:0042524_negative+regulation+of+tyrosine+phosphorylation+of+Stat	0.690492	#N/A	0.690492
BP_GO:0052547_regulation+of+peptidase+activity	0.690492	#N/A	0.690492

BP_GO:0051918_negative+regulation+of+fibrinolysis	0.722608	0.722608	#N/A
MF_GO:0051213_dioxygenase+activity	0.357713	#N/A	0.357713
MF_GO:0047130_saccharopine+dehydrogenase+(NADP+,+L-lysine-forming)+	0.25901	0.25901	#N/A
BP_GO:0042694_muscle+cell+fate+specification	0.831975	#N/A	0.831975
BP_GO:0001775_cell+activation	0.732227	0.732227	#N/A
BP_GO:0001959_regulation+of+cytokine+mediated+signaling+pathway	0.732227	0.732227	#N/A
BP_GO:0002523_leukocyte+migration+during+inflammatory+response	0.732227	0.732227	#N/A
BP_GO:0002842_positive+regulation+of+T+cell+mediated+immune+respons	0.732227	0.732227	#N/A
BP_GO:0032600_chemokine+receptor+transport+out+of+membrane+raft	0.732227	0.732227	#N/A
BP_GO:0032913_negative+regulation+of+transforming+growth+factor-beta3	0.732227	0.732227	#N/A
BP_GO:0033625_positive+regulation+of+integrin+activation	0.732227	0.732227	#N/A
BP_GO:0033630_positive+regulation+of+cell+adhesion+mediated+by+integr	0.732227	0.732227	#N/A
BP_GO:0033632_regulation+of+cell-cell+adhesion+mediated+by+integrin	0.732227	0.732227	#N/A
BP_GO:0033634_positive+regulation+of+cell-cell+adhesion+mediated+by+ir	0.732227	0.732227	#N/A
BP_GO:0034107_negative+regulation+of+erythrocyte+clearance	0.732227	0.732227	#N/A
BP_GO:0034119_negative+regulation+of+erythrocyte+aggregation	0.732227	0.732227	#N/A
BP_GO:0042103_positive+regulation+of+T+cell+homeostatic+proliferation	0.732227	0.732227	#N/A
BP_GO:0046014_negative+regulation+of+T+cell+homeostatic+proliferation	0.732227	0.732227	#N/A
CC_GO:0060170_cilium+membrane	0.732227	0.732227	#N/A
MF_GO:0016410_N-acyltransferase+activity	0.493863	0.493863	#N/A
MF_GO:0047963_glycine+N-choloyltransferase+activity	0.493863	0.493863	#N/A
BP_GO:0006067_ethanol+metabolic+process	0.339224	#N/A	0.339224
BP_GO:0042375_quinone+cofactor+metabolic+process	0.339224	#N/A	0.339224
BP_GO:0046164_alcohol+catabolic+process	0.339224	#N/A	0.339224
MF_GO:0004024_alcohol+dehydrogenase+activity,+zinc-dependent	0.339224	#N/A	0.339224
BP_GO:0051347_positive+regulation+of+transferase+activity	0.0506129	0.0506129	#N/A
BP_GO:0007284_spermatogonial+cell+division	0.549481	0.549481	#N/A
BP_GO:0034633_retinol+transport	0.549481	0.549481	#N/A
BP_GO:0060059_embryonic+retina+morphogenesis+in+camera-type+eye	0.549481	0.549481	#N/A
BP_GO:0060065_uterus+development	0.549481	0.549481	#N/A
MF_GO:0034632_retinol+transporter+activity	0.549481	0.549481	#N/A
MF_GO:0008747_N-acetylneuraminate+lyase+activity	0.48284	#N/A	0.48284
BP_GO:0006116_NADH+oxidation	0.519684	0.519684	#N/A
MF_GO:0030395_lactose+binding	0.872818	0.872818	#N/A
MF_GO:0008443_phosphofructokinase+activity	0.522076	0.522076	#N/A
BP_GO:0043501_skeletal+muscle+adaptation	0.340231	#N/A	0.340231
BP_GO:0055001_muscle+cell+development	0.340231	#N/A	0.340231
CC_GO:0009348_ornithine+carbamoyltransferase+complex	0.093173	0.093173	#N/A
MF_GO:0004585_ornithine+carbamoyltransferase+activity	0.093173	0.093173	#N/A
MF_GO:0016743_carboxyl-+or+carbamoyltransferase+activity	0.093173	0.093173	#N/A
MF_GO:0017129_triglyceride+binding	0.0877314	0.0877314	#N/A
BP_GO:0030241_muscle+thick+filament+assembly	0.442035	#N/A	0.442035
MF_GO:0047568_3-oxo-5-beta-steroid+4-dehydrogenase+activity	0.203726	0.203726	#N/A
MF_GO:0047787_delta4-3-oxosteroid+5beta-reductase+activity	0.203726	0.203726	#N/A
MF_GO:0033778_7alpha-hydroxycholest-4-en-3-one+12alpha-hydroxylase+a	0.048375	#N/A	0.048375
BP_GO:0046439_L-cysteine+metabolic+process	0.720096	0.720096	#N/A
MF_GO:0017172_cysteine+dioxygenase+activity	0.720096	0.720096	#N/A

CC_GO:0016014_dystrobrevin+complex	0.593449	#N/A	0.593449
MF_GO:0017166_vinculin+binding	0.720316	#N/A	0.720316
MF_GO:0051371_muscle+alpha-actinin+binding	0.720316	#N/A	0.720316
MF_GO:0047305_(R)-3-amino-2-methylpropionate-pyruvate+transaminase+	0.0143777	0.0143777	#N/A
MF_GO:0008458_carnitine+O-octanoyltransferase+activity	0.525804	0.525804	#N/A
MF_GO:0008119_thiopurine+S-methyltransferase+activity	0.234182	0.234182	#N/A
BP_GO:0032972_regulation+of+muscle+filament+sliding+speed	0.564723	#N/A	0.564723
MF_GO:0008320_protein+transmembrane+transporter+activity	0.0305771	0.0305771	#N/A
MF_GO:0048244_phytanoyl-CoA+dioxygenase+activity	0.892445	#N/A	0.892445
MF_GO:0001512_dihydronicotinamide+riboside+quinone+reductase+activit	0.220944	0.220944	#N/A
MF_GO:0043184_vascular+endothelial+growth+factor+receptor+2+binding	0.0121029	#N/A	0.0121029
BP_GO:0003009_skeletal+muscle+contraction	0.981036	#N/A	0.981036
BP_GO:0032236_positive+regulation+of+calcium+ion+transport+via+store-o	0.981036	#N/A	0.981036
BP_GO:0002544_chronic+inflammatory+response	0.260264	#N/A	0.260264
MF_GO:0034235_GPI+anchor+binding	0.260264	#N/A	0.260264
MF_GO:0008466_glycogenin+glucosyltransferase+activity	0.705178	0.705178	#N/A
MF_GO:0004648_phosphoserine+transaminase+activity	0.621757	0.621757	#N/A
BP_GO:0050482_arachidonic+acid+secretion	0.0830618	#N/A	0.0830618
MF_GO:0004608_phosphatidylethanolamine+N-methyltransferase+activity	0.969989	0.969989	#N/A
MF_GO:0051183_vitamin+transporter+activity	0.778747	0.778747	#N/A
MF_GO:0030339_fatty-acyl-ethyl-ester+synthase+activity	0.991185	#N/A	0.991185
BP_GO:0051651_maintenance+of+location+in+cell	0.682825	0.682825	#N/A
BP_GO:0042941_D-alanine+transport	0.209763	#N/A	0.209763
BP_GO:0042942_D-serine+transport	0.209763	#N/A	0.209763
BP_GO:0031585_regulation+of+inositol-1,4,5-triphosphate+receptor+activit	0.342549	0.342549	#N/A
BP_GO:0032252_secretory+granule+localization	0.342549	0.342549	#N/A
BP_GO:0051643_ER+localization	0.342549	0.342549	#N/A
CC_GO:0042641_actomyosin	0.342549	0.342549	#N/A
MF_GO:0047105_4-trimethylammoniobutyraldehyde+dehydrogenase+activi	0.907249	#N/A	0.907249
MF_GO:0005360_insulin-responsive+hydrogen:glucose+symporter+activity	0.876687	0.876687	#N/A
MF_GO:0004411_homogentisate+1,2-dioxygenase+activity	0.0840664	0.0840664	#N/A
CC_GO:0045098_type+III+intermediate+filament	0.482992	0.482992	#N/A
BP_GO:0018931_naphthalene+metabolic+process	0.997955	0.997955	#N/A
MF_GO:0016206_catechol+O-methyltransferase+activity	0.95675	0.95675	#N/A
MF_GO:0050124_N-acylneuraminate-9-phosphatase+activity	0.00749285	0.00749285	#N/A
MF_GO:0004086_carbamoyl-phosphate+synthase+activity	0.32246	0.32246	#N/A
MF_GO:0004087_carbamoyl-phosphate+synthase+(ammonia)+activity	0.32246	0.32246	#N/A
BP_GO:0034213_quinolinate+catabolic+process	0.107486	0.107486	#N/A
MF_GO:0004514_nicotinate-nucleotide+diphosphorylase+(carboxylating)+ac	0.107486	0.107486	#N/A
BP_GO:0015759_beta-glucoside+transport	0.806398	0.806398	#N/A
BP_GO:0060192_negative+regulation+of+lipase+activity	0.806398	0.806398	#N/A
BP_GO:0030070_insulin+processing	0.0513313	#N/A	0.0513313
BP_GO:0007525_somatic+muscle+development	0.0107821	0.0107821	#N/A
BP_GO:0060004_reflex	0.250328	#N/A	0.250328
BP_GO:0014829_vascular+smooth+muscle+contraction	0.349508	#N/A	0.349508
BP_GO:0032836_glomerular+basement+membrane+development	0.44221	0.44221	#N/A
MF_GO:0004502_kynurenine+3-monooxygenase+activity	0.201762	#N/A	0.201762

MF_GO:0047747_cholate-CoA+ligase+activity	0.793012	0.793012	#N/A
BP_GO:0003065_positive+regulation+of+heart+rate+by+epinephrine	0.105732	#N/A	0.105732
BP_GO:0001766_membrane+raft+polarization	0.148428	0.148428	#N/A
BP_GO:0046631_alpha-beta+T+cell+activation	0.148428	0.148428	#N/A
BP_GO:0046633_alpha-beta+T+cell+proliferation	0.148428	0.148428	#N/A
MF_GO:0047865_dimethylglycine+dehydrogenase+activity	0.924791	0.924791	#N/A
BP_GO:0045540_regulation+of+cholesterol+biosynthetic+process	0.387903	0.387903	#N/A
BP_GO:0032098_regulation+of+appetite	0.530142	#N/A	0.530142
BP_GO:0051608_histamine+transport	0.530142	#N/A	0.530142
BP_GO:0051615_histamine+uptake	0.530142	#N/A	0.530142
BP_GO:0001993_regulation+of+systemic+arterial+blood+pressure+by+norep	0.149822	0.149822	#N/A
MF_GO:0004941_beta2-adrenergic+receptor+activity	0.149822	0.149822	#N/A
MF_GO:0008179_adenylate+cyclase+binding	0.149822	0.149822	#N/A
MF_GO:0030171_voltage-gated+proton+channel+activity	0.167998	0.167998	#N/A
BP_GO:0032148_activation+of+protein+kinase+B+activity	0.0701159	0.0701159	#N/A
MF_GO:0043422_protein+kinase+B+binding	0.0701159	0.0701159	#N/A
MF_GO:0047757_chondroitin-glucuronate+5-epimerase+activity	0.0252502	0.0252502	#N/A
BP_GO:0002446_neutrophil+mediated+immunity	0.5517	0.5517	#N/A
BP_GO:0032956_regulation+of+actin+cytoskeleton+organization	0.5517	0.5517	#N/A
BP_GO:0045088_regulation+of+innate+immune+response	0.5517	0.5517	#N/A
BP_GO:0019318_hexose+metabolic+process	0.751944	#N/A	0.751944
MF_GO:0004034_aldose+1-epimerase+activity	0.751944	#N/A	0.751944
MF_GO:0033971_hydroxyisourate+hydrolase+activity	0.732558	#N/A	0.732558
MF_GO:0003837_beta-ureidopropionase+activity	0.172205	0.172205	#N/A
BP_GO:0006060_sorbitol+metabolic+process	0.547611	#N/A	0.547611
MF_GO:0003939_L-iditol+2-dehydrogenase+activity	0.547611	#N/A	0.547611
BP_GO:0033574_response+to+testosterone+stimulus	0.526193	0.526193	#N/A
MF_GO:0004716_receptor+signaling+protein+tyrosine+kinase+activity	0.479725	#N/A	0.479725
MF_GO:0004617_phosphoglycerate+dehydrogenase+activity	0.721685	0.721685	#N/A
MF_GO:0016619_malate+dehydrogenase+(oxaloacetate-decarboxylating)+a	0.937973	0.937973	#N/A
MF_GO:0016404_15-hydroxyprostaglandin+dehydrogenase+(NAD+)+activity	0.644207	0.644207	#N/A
BP_GO:0043648_dicarboxylic+acid+metabolic+process	0.76077	0.76077	#N/A
MF_GO:0030343_vitamin+D3+25-hydroxylase+activity	0.000131768	0.000131768	#N/A
CC_GO:0005586_collagen+type+III	0.304889	0.304889	#N/A
BP_GO:0046340_diacylglycerol+catabolic+process	0.993939	0.993939	#N/A
MF_GO:0033878_hormone-sensitive+lipase+activity	0.993939	0.993939	#N/A
MF_GO:0004741_[pyruvate+dehydrogenase+(lipoamide)]+phosphatase+act	0.62841	0.62841	#N/A
MF_GO:0015368_calcium:cation+antiporter+activity	0.239776	#N/A	0.239776
BP_GO:0002920_regulation+of+humoral+immune+response	0.42254	0.42254	#N/A
MF_GO:0003941_L-serine+ammonia-lyase+activity	0.915567	0.915567	#N/A
MF_GO:0003885_D-arabinono-1,4-lactone+oxidase+activity	0.560461	#N/A	0.560461
MF_GO:0016899_oxidoreductase+activity,+acting+on+the+CH-OH+group+of	0.560461	#N/A	0.560461
MF_GO:0050105_L-gulonolactone+oxidase+activity	0.560461	#N/A	0.560461
BP_GO:0032224_positive+regulation+of+synaptic+transmission,+cholinergic	0.612304	#N/A	0.612304
BP_GO:0009446_putrescine+biosynthetic+process	0.536343	0.536343	#N/A
MF_GO:0008783_agmatinase+activity	0.536343	0.536343	#N/A
MF_GO:0004924_oncostatin-M+receptor+activity	0.308787	0.308787	#N/A

MF_GO:0003991_acetylglutamate+kinase+activity	0.966247	#N/A	0.966247
MF_GO:0004042_amino-acid+N-acetyltransferase+activity	0.966247	#N/A	0.966247
BP_GO:0030814_regulation+of+cAMP+metabolic+process	0.941933	#N/A	0.941933
MF_GO:0004334_fumarylacetoacetase+activity	0.641542	0.641542	#N/A
BP_GO:0015810_aspartate+transport	0.11408	0.11408	#N/A
MF_GO:0003996_acyl-CoA+ligase+activity	0.921038	0.921038	#N/A
MF_GO:0050998_nitric-oxide+synthase+binding	0.466056	#N/A	0.466056
BP_GO:0017144_drug+metabolic+process	0.561375	#N/A	0.561375
BP_GO:0060262_negative+regulation+of+N-terminal+protein+palmitoylatio	0.766656	0.766656	#N/A
BP_GO:0045834_positive+regulation+of+lipid+metabolic+process	0.670032	0.670032	#N/A
BP_GO:0051005_negative+regulation+of+lipoprotein+lipase+activity	0.670032	0.670032	#N/A
MF_GO:0004464_leukotriene-C4+synthase+activity	0.618653	0.618653	#N/A
MF_GO:0001875_lipopolysaccharide+receptor+activity	0.713492	#N/A	0.713492
MF_GO:0004992_platelet+activating+factor+receptor+activity	0.713492	#N/A	0.713492
BP_GO:0048170_positive+regulation+of+long-term+neuronal+synaptic+plas	0.0618794	0.0618794	#N/A
MF_GO:0050046_lathosterol+oxidase+activity	0.0901475	0.0901475	#N/A
BP_GO:0021768_nucleus+accumbens+development	0.557107	#N/A	0.557107
MF_GO:0004504_peptidylglycine+monooxygenase+activity	0.87162	0.87162	#N/A
MF_GO:0004598_peptidylamidoglycolate+lyase+activity	0.87162	0.87162	#N/A
MF_GO:0004796_thromboxane-A+synthase+activity	0.9759	0.9759	#N/A
MF_GO:0004096_catalase+activity	0.315504	0.315504	#N/A
MF_GO:0016684_oxidoreductase+activity,+acting+on+peroxide+as+accepto	0.315504	0.315504	#N/A
BP_GO:0006942_regulation+of+striated+muscle+contraction	0.364102	0.364102	#N/A
BP_GO:0045988_negative+regulation+of+striated+muscle+contraction	0.364102	0.364102	#N/A
BP_GO:0051481_reduction+of+cytosolic+calcium+ion+concentration	0.364102	0.364102	#N/A
MF_GO:0004920_interleukin-10+receptor+activity	0.0206287	0.0206287	#N/A
BP_GO:0002711_positive+regulation+of+T+cell+mediated+immunity	0.516141	0.516141	#N/A
BP_GO:0002923_regulation+of+humoral+immune+response+mediated+by+	0.516141	0.516141	#N/A
BP_GO:0034113_heterotypic+cell-cell+adhesion	0.516141	0.516141	#N/A
BP_GO:0050855_regulation+of+B+cell+receptor+signaling+pathway	0.516141	0.516141	#N/A
BP_GO:0050857_positive+regulation+of+antigen+receptor-mediated+signali	0.516141	0.516141	#N/A
BP_GO:0050677_positive+regulation+of+urothelial+cell+proliferation	0.9575	0.9575	#N/A
MF_GO:0030883_endogenous+lipid+antigen+binding	0.578872	0.578872	#N/A
MF_GO:0016034_maleylacetoacetate+isomerase+activity	0.0965501	0.0965501	#N/A
BP_GO:0032288_myelin+formation	0.0454798	0.0454798	#N/A
BP_GO:0001999_renal+response+to+blood+flow+during+renin-angiotensin+	0.310505	#N/A	0.310505
BP_GO:0048143_astrocyte+activation	0.310505	#N/A	0.310505
MF_GO:0031702_type+1+angiotensin+receptor+binding	0.310505	#N/A	0.310505
MF_GO:0031703_type+2+angiotensin+receptor+binding	0.310505	#N/A	0.310505
MF_GO:0030492_hemoglobin+binding	0.394768	#N/A	0.394768
BP_GO:0006127_glycerophosphate+shuttle	0.307208	0.307208	#N/A
BP_GO:0015794_glycerol-3-phosphate+transport	0.307208	0.307208	#N/A
MF_GO:0015169_glycerol-3-phosphate+transmembrane+transporter+activit	0.307208	0.307208	#N/A
MF_GO:0004361_glutaryl-CoA+dehydrogenase+activity	0.576409	#N/A	0.576409
BP_GO:0006069_ethanol+oxidation	0.827815	#N/A	0.827815
BP_GO:0046294_formaldehyde+catabolic+process	0.827815	#N/A	0.827815
BP_GO:0051409_response+to+nitrosative+stress	0.827815	#N/A	0.827815

MF_GO:0051903_S-(hydroxymethyl)glutathione+dehydrogenase+activity	0.827815	#N/A	0.827815
BP_GO:0002246_healing+during+inflammatory+response	0.116265	0.116265	#N/A
CC_GO:0030690_Noc1p-Noc2p+complex	0.731295	#N/A	0.731295
CC_GO:0005588_collagen+type+V	0.574743	0.574743	#N/A
MF_GO:0015291_secondary+active+transmembrane+transporter+activity	0.40854	#N/A	0.40854
MF_GO:0047536_2-aminoadipate+transaminase+activity	0.983212	0.983212	#N/A
BP_GO:0045329_carnitine+biosynthetic+process	0.0996144	0.0996144	#N/A
MF_GO:0008336_gamma-butyrobetaine+dioxygenase+activity	0.0996144	0.0996144	#N/A
MF_GO:0004990_oxytocin+receptor+activity	0.994594	#N/A	0.994594
BP_GO:0008300_isoprenoid+catabolic+process	0.504437	0.504437	#N/A
MF_GO:0008111_alpha-methylacyl-CoA+racemase+activity	0.504437	0.504437	#N/A
BP_GO:0032344_regulation+of+aldosterone+metabolic+process	0.523358	0.523358	#N/A
BP_GO:0045794_negative+regulation+of+cell+volume	0.523358	0.523358	#N/A
BP_GO:0060073_micturition	0.523358	0.523358	#N/A
BP_GO:0060082_eye+blink+reflex	0.523358	0.523358	#N/A
MF_GO:0060072_large+conductance+calcium-activated+potassium+channe	0.523358	0.523358	#N/A
MF_GO:0004520_endodeoxyribonuclease+activity	0.138796	0.138796	#N/A
MF_GO:0030228_lipoprotein+receptor+activity	0.437209	0.437209	#N/A
BP_GO:0016480_negative+regulation+of+transcription+from+RNA+polymer	0.817787	0.817787	#N/A
BP_GO:0030195_negative+regulation+of+blood+coagulation	0.563669	0.563669	#N/A
BP_GO:0032367_intracellular+cholesterol+transport	0.261748	0.261748	#N/A
MF_GO:0004123_cystathionine+gamma-lyase+activity	0.298374	0.298374	#N/A
BP_GO:0031639_plasminogen+activation	0.194405	0.194405	#N/A
MF_GO:0016628_oxidoreductase+activity,+acting+on+the+CH-CH+group+of	0.194405	0.194405	#N/A
BP_GO:0000147_actin+cortical+patch+assembly	0.616638	0.616638	#N/A
BP_GO:0007499_ectoderm+and+mesoderm+interaction	0.184431	0.184431	#N/A
BP_GO:0030859_polarized+epithelial+cell+differentiation	0.184431	0.184431	#N/A
BP_GO:0060197_cloacal+septation	0.184431	0.184431	#N/A
BP_GO:0006696_ergosterol+biosynthetic+process	0.160884	0.160884	#N/A
MF_GO:0030409_glutamate+formimidoyltransferase+activity	0.313436	0.313436	#N/A
MF_GO:0030412_formimidoyltetrahydrofolate+cyclodeaminase+activity	0.313436	0.313436	#N/A
BP_GO:0015761_mannose+transport	0.52795	0.52795	#N/A
CC_GO:0000323_lytic+vacuole	0.52795	0.52795	#N/A
MF_GO:0015578_mannose+transmembrane+transporter+activity	0.52795	0.52795	#N/A
BP_GO:0017014_protein+amino+acid+nitrosylation	0.738343	0.738343	#N/A
MF_GO:0004397_histidine+ammonia-lyase+activity	0.294842	0.294842	#N/A
MF_GO:0016211_ammonia+ligase+activity	0.294842	0.294842	#N/A
MF_GO:0033981_D-dopachrome+decarboxylase+activity	0.102386	0.102386	#N/A
MF_GO:0047323_[3-methyl-2-oxobutanoate+dehydrogenase+(acetyl-transfe	0.415242	0.415242	#N/A
BP_GO:0048677_axon+extension+involved+in+regeneration	0.127351	0.127351	#N/A
BP_GO:0009152_purine+ribonucleotide+biosynthetic+process	0.504085	0.504085	#N/A
MF_GO:0004018_adenylosuccinate+lyase+activity	0.504085	0.504085	#N/A
MF_GO:0031008_filamin-C+binding	0.804569	#N/A	0.804569
MF_GO:0004750_ribulose-phosphate+3-epimerase+activity	0.263049	0.263049	#N/A
BP_GO:0006106_fumarate+metabolic+process	0.787875	#N/A	0.787875
CC_GO:0045239_tricarboxylic+acid+cycle+enzyme+complex	0.787875	#N/A	0.787875
MF_GO:0004333_fumarate+hydratase+activity	0.787875	#N/A	0.787875

BP_GO:0009855_determination+of+bilateral+symmetry	0.119686	0.119686	#N/A
BP_GO:0048388_endosomal+lumen+acidification	0.249742	#N/A	0.249742
MF_GO:0033791_3alpha,7alpha,12alpha-trihydroxy-5beta-cholestanoyl-CoA	0.788242	0.788242	#N/A
BP_GO:0006543_glutamine+catabolic+process	0.293098	#N/A	0.293098
BP_GO:0050861_positive+regulation+of+B+cell+receptor+signaling+pathway	0.050569	0.050569	#N/A
BP_GO:0045908_negative+regulation+of+vasodilation	0.608708	0.608708	#N/A
MF_GO:0017020_myosin+phosphatase+regulator+activity	0.608708	0.608708	#N/A
CC_GO:0016942_insulin-like+growth+factor+binding+protein+complex	0.59381	#N/A	0.59381
MF_GO:0033783_25-hydroxycholesterol+7alpha-hydroxylase+activity	0.204641	#N/A	0.204641
MF_GO:0033867_Fas-activated+serine/threonine+kinase+activity	0.25109	#N/A	0.25109
MF_GO:0001851_complement+component+C3b+binding	0.960017	0.960017	#N/A
MF_GO:0004371_glycerone+kinase+activity	0.263333	#N/A	0.263333
MF_GO:0042171_lysophosphatidic+acid+acyltransferase+activity	0.262486	0.262486	#N/A
BP_GO:0019556_histidine+catabolic+process+to+glutamate+and+formamide	0.749305	0.749305	#N/A
MF_GO:0016812_hydrolase+activity,+acting+on+carbon-nitrogen+(but+not+	0.749305	0.749305	#N/A
MF_GO:0050480_imidazolonepropionase+activity	0.749305	0.749305	#N/A
MF_GO:0008420_CTD+phosphatase+activity	0.891841	0.891841	#N/A
BP_GO:0006208_pyrimidine+base+catabolic+process	0.67944	0.67944	#N/A
MF_GO:0017113_dihydropyrimidine+dehydrogenase+(NADP+)+activity	0.67944	0.67944	#N/A
MF_GO:0033961_cis-stilbene-oxide+hydrolase+activity	0.226933	#N/A	0.226933
BP_GO:0015976_carbon+utilization	0.0515584	0.0515584	#N/A
MF_GO:0008480_sarcosine+dehydrogenase+activity	0.4753	#N/A	0.4753
MF_GO:0016153_urocanate+hydratase+activity	0.421063	0.421063	#N/A
MF_GO:0030504_inorganic+diphosphate+transmembrane+transporter+activ	0.394092	0.394092	#N/A
MF_GO:0033735_aspartate+dehydrogenase+activity	0.680694	0.680694	#N/A
MF_GO:0004051_arachidonate+5-lipoxygenase+activity	0.625427	0.625427	#N/A
MF_GO:0004133_glycogen+debranching+enzyme+activity	0.619159	0.619159	#N/A
MF_GO:0031593_polyubiquitin+binding	0.619159	0.619159	#N/A
MF_GO:0042602_flavin+reductase+activity	0.769872	#N/A	0.769872
BP_GO:0019919_peptidyl-arginine+methylation,+to+asymmetrical-dimethyl	0.717106	0.717106	#N/A
BP_GO:0035247_peptidyl-arginine+omega-N-methylation	0.717106	0.717106	#N/A
MF_GO:0016275_[cytochrome+c]-arginine+N-methyltransferase+activity	0.717106	0.717106	#N/A
MF_GO:0035241_protein-arginine+omega-N+monomethyltransferase+activi	0.717106	0.717106	#N/A
MF_GO:0035242_protein-arginine+omega-N+asymmetric+methyltransferase	0.717106	0.717106	#N/A
MF_GO:0050121_N-acylglucosamine+2-epimerase+activity	0.514566	#N/A	0.514566
MF_GO:0008114_phosphogluconate+2-dehydrogenase+activity	0.353962	0.353962	#N/A
BP_GO:0009074_aromatic+amino+acid+family+catabolic+process	0.411138	0.411138	#N/A
MF_GO:0004838_tyrosine+transaminase+activity	0.411138	0.411138	#N/A
BP_GO:0031223_auditory+behavior	0.558277	0.558277	#N/A
MF_GO:0005314_high-affinity+glutamate+transmembrane+transporter+acti	0.558277	0.558277	#N/A
MF_GO:0016595_glutamate+binding	0.558277	0.558277	#N/A
BP_GO:0046654_tetrahydrofolate+biosynthetic+process	0.631964	0.631964	#N/A
MF_GO:0003934_GTP+cyclohydrolase+I+activity	0.631964	0.631964	#N/A
BP_GO:0006148_inosine+catabolic+process	0.503785	0.503785	#N/A
BP_GO:0006149_deoxyinosine+catabolic+process	0.503785	0.503785	#N/A
BP_GO:0006161_deoxyguanosine+catabolic+process	0.503785	0.503785	#N/A
BP_GO:0034418_urate+biosynthetic+process	0.503785	0.503785	#N/A

BP_GO:0042278_purine+nucleoside+metabolic+process	0.503785	0.503785	#N/A
BP_GO:0046115_guanosine+catabolic+process	0.503785	0.503785	#N/A
MF_GO:0004731_purine-nucleoside+phosphorylase+activity	0.503785	0.503785	#N/A
BP_GO:0042167_heme+catabolic+process	0.104474	#N/A	0.104474
CC_GO:0005715_late+recombination+nodule	0.110456	#N/A	0.110456
BP_GO:0032196_transposition	0.184874	0.184874	#N/A
BP_GO:0030853_negative+regulation+of+granulocyte+differentiation	0.188539	0.188539	#N/A
BP_GO:0045656_negative+regulation+of+monocyte+differentiation	0.188539	0.188539	#N/A
BP_GO:0045659_negative+regulation+of+neutrophil+differentiation	0.188539	0.188539	#N/A
BP_GO:0045844_positive+regulation+of+striated+muscle+development	0.75546	#N/A	0.75546
BP_GO:0000705_achiasmate+meiosis+I	0.69996	#N/A	0.69996
BP_GO:0051309_female+meiosis+chromosome+separation	0.69996	#N/A	0.69996
BP_GO:0051353_positive+regulation+of+oxidoreductase+activity	0.0703353	#N/A	0.0703353
BP_GO:0060265_positive+regulation+of+respiratory+burst+during+acute+in	0.63585	0.63585	#N/A
MF_GO:0051637_Gram-positive+bacterial+cell+surface+binding	0.63585	0.63585	#N/A
MF_GO:0030267_glyoxylate+reductase+(NADP)+activity	0.767132	0.767132	#N/A
MF_GO:0047273_galactosylgalactosylglucosylceramide+beta-D-acetylgalact	0.733448	0.733448	#N/A
BP_GO:0032964_collagen+biosynthetic+process	0.0662449	#N/A	0.0662449
BP_GO:0051127_positive+regulation+of+actin+nucleation	0.0159029	0.0159029	#N/A
MF_GO:0004978_adrenocorticotropin+receptor+activity	0.963919	0.963919	#N/A
BP_GO:0042347_negative+regulation+of+NF-kappaB+import+into+nucleus	0.629871	#N/A	0.629871
CC_GO:0043293_apoptosome	0.629871	#N/A	0.629871
MF_GO:0003976_UDP-N-acetylglucosamine-lysosomal-enzyme+N-acetylgluc	0.569875	0.569875	#N/A
BP_GO:0043244_regulation+of+protein+complex+disassembly	0.289389	#N/A	0.289389
BP_GO:0019464_glycine+decarboxylation+via+glycine+cleavage+system	0.78441	0.78441	#N/A
MF_GO:0004375_glycine+dehydrogenase+(decarboxylating)+activity	0.78441	0.78441	#N/A
BP_GO:0010107_potassium+ion+import	0.311574	#N/A	0.311574
BP_GO:0006646_phosphatidylethanolamine+biosynthetic+process	0.257631	#N/A	0.257631
CC_GO:0000808_origin+recognition+complex	0.6517	0.6517	#N/A
BP_GO:0006760_folic+acid+and+derivative+metabolic+process	0.538564	#N/A	0.538564
MF_GO:0004905_type+I+interferon+receptor+activity	0.811542	0.811542	#N/A
BP_GO:0014853_regulation+of+excitatory+postsynaptic+membrane+potent	0.8301	0.8301	#N/A
MF_GO:0004884_ecdysteroid+hormone+receptor+activity	0.168649	0.168649	#N/A
BP_GO:0001922_B-1+B+cell+homeostasis	0.947952	#N/A	0.947952
BP_GO:0010812_negative+regulation+of+cell-substrate+adhesion	0.416971	#N/A	0.416971
BP_GO:0033345_asparagine+catabolic+process+via+L-aspartate	0.0780622	0.0780622	#N/A
MF_GO:0008123_cholesterol+7-alpha-monooxygenase+activity	2.50507E-05	#N/A	2.50507E-05
BP_GO:0002084_protein+depalmitoylation	0.351253	#N/A	0.351253
BP_GO:0032429_regulation+of+phospholipase+A2+activity	0.351253	#N/A	0.351253
BP_GO:0042371_vitamin+K+biosynthetic+process	0.162439	#N/A	0.162439
MF_GO:0047057_vitamin-K-epoxide+reductase+(warfarin-sensitive)+activity	0.162439	#N/A	0.162439
MF_GO:0050682_AF-2+domain+binding	0.859739	0.859739	#N/A
BP_GO:0046415_urate+metabolic+process	0.478816	0.478816	#N/A
BP_GO:0051156_glucose+6-phosphate+metabolic+process	0.478816	0.478816	#N/A
CC_GO:0030870_Mre11+complex	0.310263	0.310263	#N/A
MF_GO:0033613_transcription+activator+binding	0.557079	0.557079	#N/A
MF_GO:0004140_dephospho-CoA+kinase+activity	0.94548	0.94548	#N/A

MF_GO:0004595_pantetheine-phosphate+adenylyltransferase+activity	0.94548	0.94548	#N/A
BP_GO:0051823_regulation+of+synapse+structural+plasticity	0.897168	#N/A	0.897168
BP_GO:0001887_selenium+metabolic+process	0.104921	0.104921	#N/A
BP_GO:0030240_muscle+thin+filament+assembly	0.553497	#N/A	0.553497
BP_GO:0055008_cardiac+muscle+morphogenesis	0.553497	#N/A	0.553497
BP_GO:0019852_L-ascorbic+acid+metabolic+process	0.416765	0.416765	#N/A
MF_GO:0004357_glutamate-cysteine+ligase+activity	0.416765	0.416765	#N/A
BP_GO:0033993_response+to+lipid	0.930256	0.930256	#N/A
BP_GO:0048669_collateral+sprouting+in+the+absence+of+injury	0.733885	0.733885	#N/A
CC_GO:0051233_spindle+midzone	0.733885	0.733885	#N/A
MF_GO:0047499_calcium-independent+phospholipase+A2+activity	0.393863	0.393863	#N/A
BP_GO:0001923_B-1+B+cell+differentiation	0.348218	0.348218	#N/A
BP_GO:0006627_mitochondrial+protein+processing+during+import	0.0725837	#N/A	0.0725837
CC_GO:0042720_mitochondrial+inner+membrane+peptidase+complex	0.0725837	#N/A	0.0725837
MF_GO:0047134_protein-disulfide+reductase+activity	0.772361	#N/A	0.772361
BP_GO:0034332_adherens+junction+organization	0.232748	0.232748	#N/A
BP_GO:0051647_nucleus+localization	0.232748	0.232748	#N/A
CC_GO:0042585_germinal+vesicle	0.348671	0.348671	#N/A
MF_GO:0004770_sterol+carrier+protein+X-related+thiolase+activity	0.609845	0.609845	#N/A
MF_GO:0033814_propanoyl-CoA+C-acyltransferase+activity	0.609845	0.609845	#N/A
CC_GO:0030121_AP-1+adaptor+complex	0.02676	0.02676	#N/A
BP_GO:0014740_negative+regulation+of+muscle+hyperplasia	0.707543	0.707543	#N/A
BP_GO:0014806_smooth+muscle+hyperplasia	0.707543	0.707543	#N/A
BP_GO:0051549_positive+regulation+of+keratinocyte+migration	0.483208	#N/A	0.483208
BP_GO:0015817_histidine+transport	0.910833	0.910833	#N/A
MF_GO:0005290_L-histidine+transmembrane+transporter+activity	0.910833	0.910833	#N/A
MF_GO:0003978_UDP-glucose+4-epimerase+activity	0.000309874	0.000309874	#N/A
MF_GO:0004058_aromatic-L-amino-acid+decarboxylase+activity	0.152514	#N/A	0.152514
MF_GO:0030748_amine+N-methyltransferase+activity	0.20819	0.20819	#N/A
BP_GO:0033578_protein+amino+acid+glycosylation+in+Golgi	0.0169702	0.0169702	#N/A
BP_GO:0043112_receptor+metabolic+process	0.0169702	0.0169702	#N/A
CC_GO:0032580_Golgi+cisterna+membrane	0.0169702	0.0169702	#N/A
MF_GO:0008424_glycoprotein+6-alpha-L-fucosyltransferase+activity	0.0169702	0.0169702	#N/A
MF_GO:0046921_alpha(1,6)-fucosyltransferase+activity	0.0169702	0.0169702	#N/A
BP_GO:0060318_definitive+erythrocyte+differentiation	0.25758	0.25758	#N/A
BP_GO:0015798_myo-inositol+transport	0.434469	0.434469	#N/A
MF_GO:0050512_lactosylceramide+4-alpha-galactosyltransferase+activity	0.380399	0.380399	#N/A
BP_GO:0031129_inductive+cell-cell+signaling	0.27759	0.27759	#N/A
BP_GO:0006545_glycine+biosynthetic+process	0.0870977	0.0870977	#N/A
MF_GO:0004146_dihydrofolate+reductase+activity	0.0870977	0.0870977	#N/A
BP_GO:0042447_hormone+catabolic+process	0.91857	0.91857	#N/A
BP_GO:0045362_positive+regulation+of+interleukin-1+biosynthetic+process	0.191829	0.191829	#N/A
BP_GO:0045368_positive+regulation+of+interleukin-13+biosynthetic+proces	0.191829	0.191829	#N/A
CC_GO:0000221_vacuolar+proton-transporting+V-type+ATPase,+V1+domair	0.269061	#N/A	0.269061
MF_GO:0050613_delta14-sterol+reductase+activity	0.00865438	0.00865438	#N/A
MF_GO:0005157_macrophage+colony+stimulating+factor+receptor+binding	0.599626	0.599626	#N/A
BP_GO:0006636_unsaturated+fatty+acid+biosynthetic+process	6.69512E-05	6.69512E-05	#N/A

MF_GO:0016213_linoleoyl-CoA+desaturase+activity	6.69512E-05	6.69512E-05	#N/A
BP_GO:0006598_polyamine+catabolic+process	9.96579E-05	9.96579E-05	#N/A
CC_GO:0032311_angiogenin-PRI+complex	0.586599	0.586599	#N/A
BP_GO:0018158_protein+amino+acid+oxidation	0.88891	#N/A	0.88891
BP_GO:0051897_positive+regulation+of+protein+kinase+B+signaling+cascac	0.88891	#N/A	0.88891
MF_GO:0005534_galactose+binding	0.0971705	#N/A	0.0971705
MF_GO:0016936_galactoside+binding	0.0971705	#N/A	0.0971705
CC_GO:0045160_myosin+I+complex	0.77924	0.77924	#N/A
MF_GO:0004903_growth+hormone+receptor+activity	0.530501	#N/A	0.530501
MF_GO:0017046_peptide+hormone+binding	0.530501	#N/A	0.530501
BP_GO:0007019_microtubule+depolymerization	0.0165072	#N/A	0.0165072
BP_GO:0031115_negative+regulation+of+microtubule+polymerization	0.0165072	#N/A	0.0165072
MF_GO:0043890_N-acetylgalactosamine-6-sulfatase+activity	0.218496	#N/A	0.218496
BP_GO:0009132_nucleoside+diphosphate+metabolic+process	0.167959	#N/A	0.167959
BP_GO:0015938_coenzyme+A+catabolic+process	0.167959	#N/A	0.167959
BP_GO:0046356_acetyl-CoA+catabolic+process	0.167959	#N/A	0.167959
BP_GO:0006581_acetylcholine+catabolic+process	0.488844	0.488844	#N/A
MF_GO:0003990_acetylcholinesterase+activity	0.488844	0.488844	#N/A
MF_GO:0004304_estrone+sulfotransferase+activity	0.0135317	#N/A	0.0135317
MF_GO:0004508_steroid+17-alpha-monooxygenase+activity	2.55414E-07	2.55414E-07	#N/A
BP_GO:0021785_branchiomotor+neuron+axon+guidance	0.219548	0.219548	#N/A
BP_GO:0006535_cysteine+biosynthetic+process+from+serine	0.399952	0.399952	#N/A
BP_GO:0019343_cysteine+biosynthetic+process+via+cystathionine	0.399952	0.399952	#N/A
MF_GO:0004122_cystathionine+beta-synthase+activity	0.399952	0.399952	#N/A
MF_GO:0004423_iduronate-2-sulfatase+activity	0.0734417	0.0734417	#N/A
CC_GO:0032127_dense+core+granule+membrane	0.488965	0.488965	#N/A
BP_GO:0048017_inositol+lipid-mediated+signaling	0.40804	0.40804	#N/A
BP_GO:0050663_cytokine+secretion	0.358133	0.358133	#N/A
BP_GO:0007096_regulation+of+exit+from+mitosis	0.22114	0.22114	#N/A
BP_GO:0006653_lecithin+metabolic+process	0.195083	#N/A	0.195083
BP_GO:0006776_vitamin+A+metabolic+process	0.195083	#N/A	0.195083
MF_GO:0008374_O-acyltransferase+activity	0.195083	#N/A	0.195083
MF_GO:0047173_phosphatidylcholine-retinol+O-acyltransferase+activity	0.195083	#N/A	0.195083
MF_GO:0004856_xylulokinase+activity	0.813241	0.813241	#N/A
BP_GO:0001985_negative+regulation+of+heart+rate+in+baroreceptor+respo	0.203336	0.203336	#N/A
MF_GO:0051786_all-trans-retinol+13,14-reductase+activity	0.438108	#N/A	0.438108
BP_GO:0030204_chondroitin+sulfate+metabolic+process	0.334166	#N/A	0.334166
BP_GO:0033037_polysaccharide+localization	0.334166	#N/A	0.334166
BP_GO:0045637_regulation+of+myeloid+cell+differentiation	0.754753	#N/A	0.754753
MF_GO:0004001_adenosine+kinase+activity	0.296243	0.296243	#N/A
BP_GO:0002066_columnar/cuboidal+epithelial+cell+development	0.00959661	0.00959661	#N/A
BP_GO:0032943_mononuclear+cell+proliferation	0.400341	#N/A	0.400341
BP_GO:0042306_regulation+of+protein+import+into+nucleus	0.400341	#N/A	0.400341
BP_GO:0048298_positive+regulation+of+isotype+switching+to+IgA+isotypes	0.400341	#N/A	0.400341
BP_GO:0046587_positive+regulation+of+calcium-dependent+cell-cell+adhes	0.782654	0.782654	#N/A
CC_GO:0016939_kinesin+II+complex	0.782654	0.782654	#N/A
BP_GO:0046038_GMP+catabolic+process	0.718697	0.718697	#N/A

MF_GO:0042296_ISG15+ligase+activity	0.396528	0.396528	#N/A
BP_GO:0006949_syncytium+formation	0.384769	0.384769	#N/A
BP_GO:0046327_glycerol+biosynthetic+process+from+pyruvate	0.196409	0.196409	#N/A
MF_GO:0004155_6,7-dihydropteridine+reductase+activity	0.0621059	0.0621059	#N/A
BP_GO:0002651_positive+regulation+of+tolerance+induction+to+self+antige	0.478428	#N/A	0.478428
BP_GO:0002663_positive+regulation+of+B+cell+tolerance+induction	0.478428	#N/A	0.478428
CC_GO:0043235_receptor+complex	0.478428	#N/A	0.478428
BP_GO:0043124_negative+regulation+of+I-kappaB+kinase/NF-kappaB+casca	0.502544	0.502544	#N/A
MF_GO:0002113_interleukin-33+binding	0.502544	0.502544	#N/A
MF_GO:0002114_interleukin-33+receptor+activity	0.502544	0.502544	#N/A
BP_GO:0051001_negative+regulation+of+nitric-oxide+synthase+activity	0.120156	#N/A	0.120156
MF_GO:0033130_acetylcholine+receptor+binding	0.429453	0.429453	#N/A
MF_GO:0003958_NADPH-hemoprotein+reductase+activity	0.090362	#N/A	0.090362
BP_GO:0009124_nucleoside+monophosphate+biosynthetic+process	0.370572	0.370572	#N/A
BP_GO:0009133_nucleoside+diphosphate+biosynthetic+process	0.370572	0.370572	#N/A
MF_GO:0004343_glucosamine+6-phosphate+N-acetyltransferase+activity	0.148961	0.148961	#N/A
BP_GO:0007497_posterior+midgut+development	0.672796	0.672796	#N/A
MF_GO:0001600_endothelin-B+receptor+activity	0.672796	0.672796	#N/A
BP_GO:0002248_connective+tissue+replacement+during+inflammatory+res	0.976153	0.976153	#N/A
BP_GO:0010634_positive+regulation+of+epithelial+cell+migration	0.976153	0.976153	#N/A
BP_GO:0051541_elastin+metabolic+process	0.976153	0.976153	#N/A
BP_GO:0021903_rostrocaudal+neural+tube+patterning	0.135818	#N/A	0.135818
MF_GO:0046539_histamine+N-methyltransferase+activity	0.0588009	0.0588009	#N/A
MF_GO:0004766_spermidine+synthase+activity	0.0243693	#N/A	0.0243693
BP_GO:0034976_response+to+endoplasmic+reticulum+stress	0.204991	0.204991	#N/A
BP_GO:0006591_ornithine+metabolic+process	0.281018	0.281018	#N/A
MF_GO:0008792_arginine+decarboxylase+activity	0.281018	0.281018	#N/A
MF_GO:0005055_laminin+receptor+activity	0.438385	0.438385	#N/A
BP_GO:0033275_actin-myosin+filament+sliding	0.0195496	0.0195496	#N/A
CC_GO:0000138_Golgi+trans+cisterna	0.258098	#N/A	0.258098
BP_GO:0001909_leukocyte+mediated+cytotoxicity	0.850062	0.850062	#N/A
MF_GO:0008281_sulfonylurea+receptor+activity	0.289342	0.289342	#N/A
MF_GO:0046316_gluconokinase+activity	0.52192	#N/A	0.52192
BP_GO:0008611_ether+lipid+biosynthetic+process	0.445757	0.445757	#N/A
MF_GO:0016287_glycerone-phosphate+O-acyltransferase+activity	0.445757	0.445757	#N/A
MF_GO:0004312_fatty-acid+synthase+activity	0.0211634	0.0211634	#N/A
MF_GO:0004313_[acyl-carrier-protein]+S-acetyltransferase+activity	0.0211634	0.0211634	#N/A
MF_GO:0004316_3-oxoacyl-[acyl-carrier-protein]+reductase+activity	0.0211634	0.0211634	#N/A
MF_GO:0004317_3-hydroxypalmitoyl-[acyl-carrier-protein]+dehydratase+ac	0.0211634	0.0211634	#N/A
MF_GO:0004319_enoyl-[acyl-carrier-protein]+reductase+(NADPH,+B-specifi	0.0211634	0.0211634	#N/A
BP_GO:0015886_heme+transport	0.51395	#N/A	0.51395
MF_GO:0015232_heme+transporter+activity	0.51395	#N/A	0.51395
BP_GO:0006900_membrane+budding	0.973427	0.973427	#N/A
BP_GO:0014049_positive+regulation+of+glutamate+secretion	0.973427	0.973427	#N/A
BP_GO:0032308_positive+regulation+of+prostaglandin+secretion	0.973427	0.973427	#N/A
BP_GO:0032731_positive+regulation+of+interleukin-1+beta+production	0.973427	0.973427	#N/A
BP_GO:0034405_response+to+fluid+shear+stress	0.973427	0.973427	#N/A

BP_GO:0043132_NAD+transport	0.973427	0.973427	#N/A
BP_GO:0044254_multicellular+organismal+protein+catabolic+process	0.973427	0.973427	#N/A
BP_GO:0046931_pore+complex+biogenesis	0.973427	0.973427	#N/A
BP_GO:0051901_positive+regulation+of+mitochondrial+depolarization	0.973427	0.973427	#N/A
BP_GO:0009231_riboflavin+biosynthetic+process	0.148749	0.148749	#N/A
MF_GO:0008531_riboflavin+kinase+activity	0.148749	0.148749	#N/A
MF_GO:0045127_N-acetylglucosamine+kinase+activity	0.394722	0.394722	#N/A
BP_GO:0042276_error-prone+postreplication+DNA+repair	0.000334826	0.000334826	#N/A
MF_GO:0017125_deoxycytidyl+transferase+activity	0.000334826	0.000334826	#N/A
BP_GO:0042738_exogenous+drug+catabolic+process	0.473691	0.473691	#N/A
BP_GO:0035229_positive+regulation+of+glutamate-cysteine+ligase+activity	0.533715	0.533715	#N/A
MF_GO:0035226_glutamate-cysteine+ligase+catalytic+subunit+binding	0.533715	0.533715	#N/A
BP_GO:0006683_galactosylceramide+catabolic+process	0.320924	0.320924	#N/A
MF_GO:0004336_galactosylceramidase+activity	0.320924	0.320924	#N/A
MF_GO:0019782_ISG15+activating+enzyme+activity	0.125912	0.125912	#N/A
BP_GO:0042840_D-glucuronate+catabolic+process	0.290843	#N/A	0.290843
BP_GO:0046185_aldehyde+catabolic+process	0.290843	#N/A	0.290843
MF_GO:0008106_alcohol+dehydrogenase+(NADP+)+activity	0.290843	#N/A	0.290843
MF_GO:0047939_L-glucuronate+reductase+activity	0.290843	#N/A	0.290843
MF_GO:0045029_UDP-activated+nucleotide+receptor+activity	0.980304	0.980304	#N/A
BP_GO:0006684_sphingomyelin+metabolic+process	0.402402	0.402402	#N/A
CC_GO:0030288_outer+membrane-bounded+periplasmic+space	0.39688	#N/A	0.39688
BP_GO:0005997_xylulose+metabolic+process	0.421405	#N/A	0.421405
MF_GO:0050038_L-xylulose+reductase+activity	0.421405	#N/A	0.421405
CC_GO:0005895_interleukin-5+receptor+complex	0.297898	0.297898	#N/A
BP_GO:0055070_copper+ion+homeostasis	0.0336489	#N/A	0.0336489
BP_GO:0030389_fructosamine+metabolic+process	0.409927	#N/A	0.409927
MF_GO:0030387_fructosamine-3-kinase+activity	0.409927	#N/A	0.409927
MF_GO:0004736_pyruvate+carboxylase+activity	0.33923	0.33923	#N/A
MF_GO:0003979_UDP-glucose+6-dehydrogenase+activity	0.591554	#N/A	0.591554
MF_GO:0004075_biotin+carboxylase+activity	0.0261039	0.0261039	#N/A
BP_GO:0045075_regulation+of+interleukin-12+biosynthetic+process	0.475321	0.475321	#N/A
BP_GO:0015881_creatine+transport	0.271602	0.271602	#N/A
MF_GO:0005308_creatine+transporter+activity	0.271602	0.271602	#N/A
MF_GO:0005309_creatine:sodium+symporter+activity	0.271602	0.271602	#N/A
MF_GO:0033858_N-acetylgalactosamine+kinase+activity	0.266355	#N/A	0.266355
BP_GO:0016024_CDP-diacylglycerol+biosynthetic+process	0.757292	#N/A	0.757292
BP_GO:0002316_follicular+B+cell+differentiation	0.234163	0.234163	#N/A
BP_GO:0032237_activation+of+store-operated+calcium+channel+activity	0.234163	0.234163	#N/A
BP_GO:0030728_ovulation	0.175054	0.175054	#N/A
BP_GO:0019676_ammonia+assimilation+cycle	0.426917	0.426917	#N/A
BP_GO:0042450_arginine+biosynthetic+process+via+ornithine	0.426917	0.426917	#N/A
MF_GO:0004056_argininosuccinate+lyase+activity	0.426917	0.426917	#N/A
MF_GO:0004306_ethanolamine-phosphate+cytidylyltransferase+activity	0.197506	0.197506	#N/A
MF_GO:0032440_2-alkenal+reductase+activity	0.715281	0.715281	#N/A
BP_GO:0008653_lipopolysaccharide+metabolic+process	0.0270558	0.0270558	#N/A
MF_GO:0050528_acyloxyacyl+hydrolase+activity	0.0270558	0.0270558	#N/A

BP_GO:0046462_monoacylglycerol+metabolic+process	0.789572	0.789572	#N/A
MF_GO:0017120_polyphosphoinositide+phosphatase+activity	0.795953	0.795953	#N/A
BP_GO:0031638_zymogen+activation	0.903341	0.903341	#N/A
MF_GO:0047277_globoside+alpha-N-acetylgalactosaminyltransferase+activi	0.0397997	0.0397997	#N/A
MF_GO:0033884_phosphoethanolamine/phosphocholine+phosphatase+acti	0.492485	#N/A	0.492485
BP_GO:0010464_regulation+of+mesenchymal+cell+proliferation	0.214213	0.214213	#N/A
BP_GO:0051150_regulation+of+smooth+muscle+cell+differentiation	0.214213	0.214213	#N/A
MF_GO:0004168_dolichol+kinase+activity	0.354952	#N/A	0.354952
BP_GO:0006114_glycerol+biosynthetic+process	0.276059	0.276059	#N/A
BP_GO:0055089_fatty+acid+homeostasis	0.276059	0.276059	#N/A
BP_GO:0051170_nuclear+import	0.0229985	0.0229985	#N/A
BP_GO:0042489_negative+regulation+of+odontogenesis+of+dentine-contai	0.554115	#N/A	0.554115
MF_GO:0047015_3-hydroxy-2-methylbutyryl-CoA+dehydrogenase+activity	0.60719	#N/A	0.60719
MF_GO:0000210_NAD++diphosphatase+activity	0.276655	0.276655	#N/A
MF_GO:0008478_pyridoxal+kinase+activity	0.468384	#N/A	0.468384
MF_GO:0008460_dTDP-glucose+4,6-dehydratase+activity	0.269704	0.269704	#N/A
BP_GO:0046653_tetrahydrofolate+metabolic+process	0.928696	0.928696	#N/A
MF_GO:0008115_sarcosine+oxidase+activity	0.928696	0.928696	#N/A
MF_GO:0050031_L-pipecolate+oxidase+activity	0.928696	0.928696	#N/A
BP_GO:0050863_regulation+of+T+cell+activation	0.528275	0.528275	#N/A
MF_GO:0000250_lanosterol+synthase+activity	0.307929	0.307929	#N/A
MF_GO:0051538_3+iron,+4+sulfur+cluster+binding	0.970106	#N/A	0.970106
MF_GO:0004092_carnitine+O-acetyltransferase+activity	0.327537	0.327537	#N/A
BP_GO:0006507_GPI+anchor+release	0.644026	0.644026	#N/A
MF_GO:0004621_glycosylphosphatidylinositol+phospholipase+D+activity	0.644026	0.644026	#N/A
BP_GO:0035246_peptidyl-arginine+N-methylation	0.397729	#N/A	0.397729
BP_GO:0019402_galactitol+metabolic+process	0.987429	#N/A	0.987429
BP_GO:0002502_peptide+antigen+assembly+with+MHC+class+I+protein+co	0.67861	#N/A	0.67861
BP_GO:0010149_senescence	0.67861	#N/A	0.67861
CC_GO:0042824_MHC+class+I+peptide+loading+complex	0.67861	#N/A	0.67861
MF_GO:0004353_glutamate+dehydrogenase+[NAD(P)+]+activity	0.457675	0.457675	#N/A
MF_GO:0001571_non-tyrosine+kinase+fibroblast+growth+factor+receptor+	0.605289	0.605289	#N/A
MF_GO:0047012_sterol-4-alpha-carboxylate+3-dehydrogenase+(decarboxyl	0.00943018	0.00943018	#N/A
BP_GO:0060043_regulation+of+cardiac+muscle+cell+proliferation	0.642626	#N/A	0.642626
BP_GO:0060314_regulation+of+ryanodine-sensitive+calcium-release+chann	0.57664	#N/A	0.57664
MF_GO:0008144_drug+binding	0.57664	#N/A	0.57664
BP_GO:0007100_mitotic+centrosome+separation	0.154458	0.154458	#N/A
BP_GO:0030490_maturation+of+SSU-rRNA	0.0860281	#N/A	0.0860281
BP_GO:0048752_semicircular+canal+morphogenesis	0.910451	0.910451	#N/A
BP_GO:0060005_vestibular+reflex	0.910451	0.910451	#N/A
MF_GO:0043874_acireductone+synthase+activity	0.724499	#N/A	0.724499
MF_GO:0004340_glucokinase+activity	0.299611	0.299611	#N/A
BP_GO:0002001_renin+secretion+into+blood+stream	0.135729	0.135729	#N/A
MF_GO:0001596_angiotensin+type+I+receptor+activity	0.135729	0.135729	#N/A
BP_GO:0032957_inositol+trisphosphate+metabolic+process	0.719868	0.719868	#N/A
MF_GO:0035300_inositol-1,3,4-trisphosphate+5/6-kinase+activity	0.719868	0.719868	#N/A
MF_GO:0047325_inositol+tetrakisphosphate+1-kinase+activity	0.719868	0.719868	#N/A

MF_GO:0003955_NAD(P)H+dehydrogenase+(quinone)+activity	0.446182	0.446182	#N/A
MF_GO:0047391_alkylglycerophosphoethanolamine+phosphodiesterase+ac	0.288841	#N/A	0.288841
MF_GO:0016641_oxidoreductase+activity,+acting+on+the+CH-NH2+group+o	0.950728	#N/A	0.950728
CC_GO:0031092_platelet+alpha+granule+membrane	0.146898	#N/A	0.146898
BP_GO:0043681_protein+import+into+mitochondrion	0.0364747	0.0364747	#N/A
BP_GO:0001811_negative+regulation+of+type+I+hypersensitivity	0.0673196	0.0673196	#N/A
MF_GO:0015211_purine+nucleoside+transmembrane+transporter+activity	0.0348154	0.0348154	#N/A
MF_GO:0000014_single-stranded+DNA+specific+endodeoxyribonuclease+ac	0.737989	#N/A	0.737989
MF_GO:0004157_dihydropyrimidinase+activity	0.0413731	0.0413731	#N/A
BP_GO:0045909_positive+regulation+of+vasodilation	0.230669	0.230669	#N/A
BP_GO:0002036_regulation+of+L-glutamate+transport	0.237476	0.237476	#N/A
BP_GO:0002520_immune+system+development	0.0892257	#N/A	0.0892257
BP_GO:0010523_negative+regulation+of+calcium+ion+transport+into+cytos	0.0892257	#N/A	0.0892257
BP_GO:0010559_regulation+of+glycoprotein+biosynthetic+process	0.0892257	#N/A	0.0892257
BP_GO:0014042_positive+regulation+of+neuron+maturation	0.0892257	#N/A	0.0892257
BP_GO:0014911_positive+regulation+of+smooth+muscle+cell+migration	0.0892257	#N/A	0.0892257
BP_GO:0021747_cochlear+nucleus+development	0.0892257	#N/A	0.0892257
BP_GO:0032835_glomerulus+development	0.0892257	#N/A	0.0892257
BP_GO:0033033_negative+regulation+of+myeloid+cell+apoptosis	0.0892257	#N/A	0.0892257
BP_GO:0033689_negative+regulation+of+osteoblast+proliferation	0.0892257	#N/A	0.0892257
BP_GO:0043375_CD8-positive,+alpha-beta+T+cell+lineage+commitment	0.0892257	#N/A	0.0892257
BP_GO:0045069_regulation+of+viral+genome+replication	0.0892257	#N/A	0.0892257
BP_GO:0048743_positive+regulation+of+skeletal+muscle+fiber+developmer	0.0892257	#N/A	0.0892257
BP_GO:0048753_pigment+granule+organization	0.0892257	#N/A	0.0892257
BP_GO:0042264_peptidyl-aspartic+acid+hydroxylation	0.442774	#N/A	0.442774
MF_GO:0004496_mevalonate+kinase+activity	0.270779	0.270779	#N/A
BP_GO:0032640_tumor+necrosis+factor+production	0.114569	0.114569	#N/A
BP_GO:0007424_open+tracheal+system+development	0.989898	#N/A	0.989898
BP_GO:0009052_pentose-phosphate+shunt,+non-oxidative+branch	0.354171	#N/A	0.354171
MF_GO:0004751_ribose-5-phosphate+isomerase+activity	0.354171	#N/A	0.354171
BP_GO:0007632_visual+behavior	0.711686	0.711686	#N/A
BP_GO:0034341_response+to+interferon-gamma	0.896663	0.896663	#N/A
CC_GO:0031314_extrinsic+to+mitochondrial+inner+membrane	0.325679	0.325679	#N/A
MF_GO:0042624_ATPase+activity,+uncoupled	0.0732233	0.0732233	#N/A
MF_GO:0008321_Ral+guanyl-nucleotide+exchange+factor+activity	0.271594	#N/A	0.271594
BP_GO:0018344_protein+geranylgeranylation	0.14685	0.14685	#N/A
MF_GO:0008859_exoribonuclease+II+activity	0.182155	0.182155	#N/A
MF_GO:0047560_3-dehydrosphinganine+reductase+activity	0.304247	0.304247	#N/A
MF_GO:0008200_ion+channel+inhibitor+activity	0.535017	#N/A	0.535017
MF_GO:0030550_acetylcholine+receptor+inhibitor+activity	0.535017	#N/A	0.535017
MF_GO:0004662_CAAX-protein+geranylgeranyltransferase+activity	0.00881736	0.00881736	#N/A
BP_GO:0046477_glycosylceramide+catabolic+process	0.264332	#N/A	0.264332
MF_GO:0004557_alpha-galactosidase+activity	0.264332	#N/A	0.264332
BP_GO:0009203_ribonucleoside+triphosphate+catabolic+process	0.530608	#N/A	0.530608
MF_GO:0000215_tRNA+2'-phosphotransferase+activity	0.0185187	#N/A	0.0185187
MF_GO:0008665_2'-phosphotransferase+activity	0.0185187	#N/A	0.0185187
BP_GO:0015680_intracellular+copper+ion+transport	0.935207	0.935207	#N/A

MF_GO:0043682_copper-transporting+ATPase+activity	0.935207	0.935207	#N/A
MF_GO:0008117_sphinganine-1-phosphate+aldolase+activity	0.408461	0.408461	#N/A
BP_GO:0046487_glyoxylate+metabolic+process	0.0309367	0.0309367	#N/A
BP_GO:0046724_oxalic+acid+secretion	0.0309367	0.0309367	#N/A
MF_GO:0004760_serine-pyruvate+transaminase+activity	0.0309367	0.0309367	#N/A
BP_GO:0046381_CMP-N-acetylneuraminate+metabolic+process	0.734677	0.734677	#N/A
MF_GO:0030338_CMP-N-acetylneuraminate+monooxygenase+activity	0.734677	0.734677	#N/A
BP_GO:0051754_meiotic+sister+chromatid+cohesion,+centromeric	0.739153	#N/A	0.739153
MF_GO:0001874_zymosan+receptor+activity	0.168562	#N/A	0.168562
BP_GO:0006892_post-Golgi+vesicle-mediated+transport	0.319684	0.319684	#N/A
MF_GO:0004515_nicotinate-nucleotide+adenylyltransferase+activity	0.111785	0.111785	#N/A
BP_GO:0044236_multicellular+organismal+metabolic+process	0.456172	0.456172	#N/A
BP_GO:0051006_positive+regulation+of+lipoprotein+lipase+activity	0.136194	#N/A	0.136194
BP_GO:0043122_regulation+of+I-kappaB+kinase/NF-kappaB+cascade	0.121462	#N/A	0.121462
BP_GO:0045357_regulation+of+interferon-beta+biosynthetic+process	0.121462	#N/A	0.121462
CC_GO:0045323_interleukin-1+receptor+complex	0.121462	#N/A	0.121462
MF_GO:0005199_structural+constituent+of+cell+wall	0.691321	0.691321	#N/A
BP_GO:0016119_carotene+metabolic+process	0.136969	0.136969	#N/A
BP_GO:0006687_glycosphingolipid+metabolic+process	0.718528	0.718528	#N/A
BP_GO:0008049_male+courtship+behavior	0.718528	0.718528	#N/A
BP_GO:0031323_regulation+of+cellular+metabolic+process	0.718528	0.718528	#N/A
BP_GO:0016572_histone+phosphorylation	0.281021	0.281021	#N/A
BP_GO:0009229_thiamin+diphosphate+biosynthetic+process	0.574993	#N/A	0.574993
MF_GO:0004788_thiamin+diphosphokinase+activity	0.574993	#N/A	0.574993
BP_GO:0040019_positive+regulation+of+embryonic+development	0.900267	0.900267	#N/A
MF_GO:0009000_selenocysteine+lyase+activity	0.105999	0.105999	#N/A
BP_GO:0015722_canalicular+bile+acid+transport	0.277473	#N/A	0.277473
MF_GO:0042030_ATPase+inhibitor+activity	0.246539	0.246539	#N/A
MF_GO:0004368_glycerol-3-phosphate+dehydrogenase+activity	0.633741	0.633741	#N/A
BP_GO:0042495_detection+of+triacylated+bacterial+lipoprotein	0.661514	0.661514	#N/A
MF_GO:0042497_triacylated+lipoprotein+binding	0.661514	0.661514	#N/A
MF_GO:0048040_UDP-glucuronate+decarboxylase+activity	0.404543	0.404543	#N/A
BP_GO:0006401_RNA+catabolic+process	0.714983	#N/A	0.714983
BP_GO:0000096_sulfur+amino+acid+metabolic+process	0.581761	0.581761	#N/A
BP_GO:0021590_cerebellum+maturation	0.581761	0.581761	#N/A
BP_GO:0021679_cerebellar+molecular+layer+development	0.581761	0.581761	#N/A
BP_GO:0021750_vestibular+nucleus+development	0.581761	0.581761	#N/A
BP_GO:0032353_negative+regulation+of+hormone+biosynthetic+process	0.581761	0.581761	#N/A
BP_GO:0048791_calcium+ion-dependent+exocytosis+of+neurotransmitter	0.581761	0.581761	#N/A
BP_GO:0002726_positive+regulation+of+T+cell+cytokine+production	0.928156	0.928156	#N/A
BP_GO:0002821_positive+regulation+of+adaptive+immune+response	0.928156	0.928156	#N/A
BP_GO:0051251_positive+regulation+of+lymphocyte+activation	0.928156	0.928156	#N/A
MF_GO:0008456_alpha-N-acetylgalactosaminidase+activity	0.40713	0.40713	#N/A
BP_GO:0002761_regulation+of+myeloid+leukocyte+differentiation	0.0317619	#N/A	0.0317619
MF_GO:0045130_keratan+sulfotransferase+activity	0.679647	#N/A	0.679647
CC_GO:0030478_actin+cap	0.38646	0.38646	#N/A
BP_GO:0007522_visceral+muscle+development	0.949954	#N/A	0.949954

MF_GO:0008112_nicotinamide+N-methyltransferase+activity	0.18267	#N/A	0.18267
MF_GO:0047223_beta-1,3-galactosyl-O-glycosyl-glycoprotein+beta-1,3-N-ac	0.605826	0.605826	#N/A
BP_GO:0006167_AMP+biosynthetic+process	0.236766	#N/A	0.236766
BP_GO:0021660_rhombomere+3+formation	0.931105	0.931105	#N/A
BP_GO:0021666_rhombomere+5+formation	0.931105	0.931105	#N/A
CC_GO:0030485_smooth+muscle+contractile+fiber	0.0401366	0.0401366	#N/A
CC_GO:0016600_flotillin+complex	0.207637	0.207637	#N/A
BP_GO:0030913_paranodal+junction+assembly	0.0515264	0.0515264	#N/A
BP_GO:0006021_inositol+biosynthetic+process	0.722142	0.722142	#N/A
MF_GO:0004512_inositol-3-phosphate+synthase+activity	0.722142	0.722142	#N/A
BP_GO:0048790_maintenance+of+presynaptic+active+zone+structure	0.402358	#N/A	0.402358
BP_GO:0050975_sensory+perception+of+touch	0.402358	#N/A	0.402358
MF_GO:0050659_N-acetylgalactosamine+4-sulfate+6-O-sulfotransferase+ac	0.519407	#N/A	0.519407
BP_GO:0009890_negative+regulation+of+biosynthetic+process	0.30472	0.30472	#N/A
MF_GO:0016971_flavin-linked+sulfhydryl+oxidase+activity	0.835759	0.835759	#N/A
BP_GO:0006066_cellular+alcohol+metabolic+process	0.126236	0.126236	#N/A
BP_GO:0019285_glycine+betaine+biosynthetic+process+from+choline	0.126236	0.126236	#N/A
MF_GO:0008812_choline+dehydrogenase+activity	0.126236	0.126236	#N/A
BP_GO:0001880_Mullerian+duct+regression	0.973698	0.973698	#N/A
BP_GO:0048382_mesendoderm+development	0.973698	0.973698	#N/A
MF_GO:0030729_acetoacetate-CoA+ligase+activity	0.0855459	0.0855459	#N/A
BP_GO:0019673_GDP-mannose+metabolic+process	0.260893	#N/A	0.260893
MF_GO:0008446_GDP-mannose+4,6-dehydratase+activity	0.260893	#N/A	0.260893
BP_GO:0032287_myelin+maintenance+in+the+peripheral+nervous+system	0.275251	#N/A	0.275251
MF_GO:0055100_adiponectin+binding	0.902047	0.902047	#N/A
BP_GO:0043457_regulation+of+cellular+respiration	0.365929	0.365929	#N/A
BP_GO:0030201_heparan+sulfate+proteoglycan+metabolic+process	0.635712	#N/A	0.635712
MF_GO:0016250_N-sulfoglucosamine+sulfohydrolase+activity	0.635712	#N/A	0.635712
MF_GO:0004782_sulfinoalanine+decarboxylase+activity	0.795183	#N/A	0.795183
MF_GO:0033989_3alpha,7alpha,12alpha-trihydroxy-5beta-cholest-24-enoyl-	0.437245	#N/A	0.437245
BP_GO:0000266_mitochondrial+fission	0.431759	0.431759	#N/A
BP_GO:0006784_heme+a+biosynthetic+process	0.431759	0.431759	#N/A
BP_GO:0017004_cytochrome+complex+assembly	0.431759	0.431759	#N/A
BP_GO:0018347_protein+amino+acid+farnesylation	0.431759	0.431759	#N/A
MF_GO:0030161_calpain+inhibitor+activity	0.640704	#N/A	0.640704
BP_GO:0006178_guanine+salvage	0.558273	#N/A	0.558273
BP_GO:0045964_positive+regulation+of+dopamine+metabolic+process	0.558273	#N/A	0.558273
BP_GO:0046100_hypoxanthine+metabolic+process	0.558273	#N/A	0.558273
MF_GO:0004698_calcium-dependent+protein+kinase+C+activity	0.204232	0.204232	#N/A
BP_GO:0007208_serotonin+receptor,+phospholipase+C+activating+pathway	0.036213	#N/A	0.036213
BP_GO:0016560_protein+import+into+peroxisome+matrix,+docking	0.629166	0.629166	#N/A
MF_GO:0004031_aldehyde+oxidase+activity	0.801893	#N/A	0.801893
MF_GO:0004631_phosphomevalonate+kinase+activity	0.0010834	0.0010834	#N/A
BP_GO:0002479_antigen+processing+and+presentation+of+exogenous+pep	0.650384	#N/A	0.650384
BP_GO:0050823_peptide+antigen+stabilization	0.650384	#N/A	0.650384
BP_GO:0016444_somatic+cell+DNA+recombination	0.285939	0.285939	#N/A
MF_GO:0016844_strictosidine+synthase+activity	0.98127	0.98127	#N/A

BP_GO:0000075_cell+cycle+checkpoint	0.9276	0.9276	#N/A
BP_GO:0042892_chloramphenicol+transport	0.733406	#N/A	0.733406
MF_GO:0004139_deoxyribose-phosphate+aldolase+activity	0.625113	0.625113	#N/A
MF_GO:0008329_pattern+recognition+receptor+activity	0.957371	0.957371	#N/A
BP_GO:0006206_pyrimidine+base+metabolic+process	0.110969	0.110969	#N/A
BP_GO:0006213_pyrimidine+nucleoside+metabolic+process	0.110969	0.110969	#N/A
MF_GO:0009032_thymidine+phosphorylase+activity	0.110969	0.110969	#N/A
BP_GO:0001987_vasoconstriction+of+artery+involved+in+baroreceptor+res	0.429147	0.429147	#N/A
BP_GO:0045818_negative+regulation+of+glycogen+catabolic+process	0.429147	0.429147	#N/A
BP_GO:0045819_positive+regulation+of+glycogen+catabolic+process	0.429147	0.429147	#N/A
CC_GO:0005639_integral+to+nuclear+inner+membrane	0.312263	0.312263	#N/A
MF_GO:0004487_methylenetetrahydrofolate+dehydrogenase+(NAD+)+activ	0.398497	#N/A	0.398497
MF_GO:0004108_citrate+(Si)-synthase+activity	0.0464441	0.0464441	#N/A
BP_GO:0040001_establishment+of+mitotic+spindle+localization	0.510967	#N/A	0.510967
BP_GO:0001886_endothelial+cell+morphogenesis	0.601898	0.601898	#N/A
MF_GO:0030160_GKAP/Homer+scaffold+activity	0.166315	0.166315	#N/A
MF_GO:0004326_tetrahydrofolylpolyglutamate+synthase+activity	0.247058	0.247058	#N/A
BP_GO:0051694_pointed-end+actin+filament+capping	0.326988	#N/A	0.326988
MF_GO:0051011_microtubule+minus-end+binding	0.326988	#N/A	0.326988
MF_GO:0050104_L-gulonate+3-dehydrogenase+activity	0.137798	0.137798	#N/A
MF_GO:0008887_glycerate+kinase+activity	0.803452	0.803452	#N/A
BP_GO:0007225_patched+ligand+processing	0.590383	0.590383	#N/A
BP_GO:0045657_positive+regulation+of+monocyte+differentiation	0.552903	0.552903	#N/A
BP_GO:0019067_viral+assembly,+maturation,+egress,+and+release	0.559565	0.559565	#N/A
MF_GO:0015093_ferrous+iron+transmembrane+transporter+activity	0.405319	#N/A	0.405319
BP_GO:0048389_intermediate+mesoderm+development	0.432127	#N/A	0.432127
MF_GO:0004854_xanthine+dehydrogenase+activity	0.639042	0.639042	#N/A
MF_GO:0004855_xanthine+oxidase+activity	0.639042	0.639042	#N/A
MF_GO:0004801_transaldolase+activity	0.724656	0.724656	#N/A
MF_GO:0030731_guanidinoacetate+N-methyltransferase+activity	0.178696	#N/A	0.178696
CC_GO:0005796_Golgi+lumen	0.745382	0.745382	#N/A
BP_GO:0031110_regulation+of+microtubule+polymerization+or+depolymer	0.756231	0.756231	#N/A
BP_GO:0048549_positive+regulation+of+pinocytosis	0.754587	0.754587	#N/A
CC_GO:0031143_pseudopodium	0.754587	0.754587	#N/A
MF_GO:0004339_glucan+1,4-alpha-glucosidase+activity	0.58169	0.58169	#N/A
BP_GO:0002431_Fc+receptor+mediated+stimulatory+signaling+pathway	0.285818	#N/A	0.285818
BP_GO:0032765_positive+regulation+of+mast+cell+cytokine+production	0.285818	#N/A	0.285818
BP_GO:0033026_negative+regulation+of+mast+cell+apoptosis	0.285818	#N/A	0.285818
BP_GO:0009262_deoxyribonucleotide+metabolic+process	0.959702	#N/A	0.959702
BP_GO:0032872_regulation+of+stress-activated+MAPK+cascade	0.584596	#N/A	0.584596
BP_GO:0042345_regulation+of+NF-kappaB+import+into+nucleus	0.584596	#N/A	0.584596
CC_GO:0030891_VCB+complex	0.527426	#N/A	0.527426
BP_GO:0045841_negative+regulation+of+mitotic+metaphase/anaphase+tra	0.982441	#N/A	0.982441
CC_GO:0005816_spindle+pole+body	0.982441	#N/A	0.982441
MF_GO:0004458_D-lactate+dehydrogenase+(cytochrome)+activity	0.635416	0.635416	#N/A
MF_GO:0008720_D-lactate+dehydrogenase+activity	0.635416	0.635416	#N/A
BP_GO:0015822_ornithine+transport	0.906018	0.906018	#N/A

MF_GO:0000064_L-ornithine+transmembrane+transporter+activity	0.906018	0.906018	#N/A
MF_GO:0005289_high+affinity+arginine+transmembrane+transporter+activi	0.906018	0.906018	#N/A
MF_GO:0005020_stem+cell+factor+receptor+activity	0.423166	0.423166	#N/A
BP_GO:0005991_trehalose+metabolic+process	0.126296	0.126296	#N/A
MF_GO:0004555_alpha,alpha-trehalase+activity	0.126296	0.126296	#N/A
BP_GO:0032446_protein+modification+by+small+protein+conjugation	0.0956909	#N/A	0.0956909
MF_GO:0004839_ubiquitin+activating+enzyme+activity	0.0956909	#N/A	0.0956909
MF_GO:0004774_succinate-CoA+ligase+activity	0.227563	#N/A	0.227563
BP_GO:0045090_retroviral+genome+replication	0.0980494	0.0980494	#N/A
BP_GO:0000052_citrulline+metabolic+process	0.192649	0.192649	#N/A
BP_GO:0019244_lactate+biosynthetic+process+from+pyruvate	0.192649	0.192649	#N/A
BP_GO:0019805_quinolinate+biosynthetic+process	0.192649	0.192649	#N/A
BP_GO:0021988_olfactory+lobe+development	0.192649	0.192649	#N/A
BP_GO:0021990_neural+plate+formation	0.192649	0.192649	#N/A
MF_GO:0050809_diazepam+binding	0.192649	0.192649	#N/A
MF_GO:0008907_integrase+activity	0.940034	0.940034	#N/A
BP_GO:0050428_3'-phosphoadenosine+5'-phosphosulfate+biosynthetic+prc	0.509841	0.509841	#N/A
CC_GO:0009336_sulfate+adenylyltransferase+complex+(ATP)	0.509841	0.509841	#N/A
MF_GO:0004349_glutamate+5-kinase+activity	0.100912	0.100912	#N/A
MF_GO:0004350_glutamate-5-semialdehyde+dehydrogenase+activity	0.100912	0.100912	#N/A
CC_GO:0032593_insulin-responsive+compartment	0.936009	0.936009	#N/A
BP_GO:0015698_inorganic+anion+transport	0.679761	#N/A	0.679761
BP_GO:0043271_negative+regulation+of+ion+transport	0.679761	#N/A	0.679761
BP_GO:0015721_bile+acid+and+bile+salt+transport	0.144574	0.144574	#N/A
BP_GO:0019896_axon+transport+of+mitochondrion	0.67497	0.67497	#N/A
MF_GO:0047291_lactosylceramide+alpha-2,3-sialyltransferase+activity	0.229671	0.229671	#N/A
CC_GO:0005818_aster	0.745518	0.745518	#N/A
BP_GO:0031076_embryonic+camera-type+eye+development	0.648162	#N/A	0.648162
MF_GO:0045030_UTP-activated+nucleotide+receptor+activity	0.94604	0.94604	#N/A
BP_GO:0030382_sperm+mitochondrion+organization	0.618567	0.618567	#N/A
BP_GO:0006668_sphinganine-1-phosphate+metabolic+process	0.059323	#N/A	0.059323
MF_GO:0042392_sphingosine-1-phosphate+phosphatase+activity	0.059323	#N/A	0.059323
BP_GO:0048539_bone+marrow+development	0.370799	0.370799	#N/A
MF_GO:0047280_nicotinamide+phosphoribosyltransferase+activity	0.651894	#N/A	0.651894
BP_GO:0051300_spindle+pole+body+organization	0.071656	0.071656	#N/A
MF_GO:0000225_N-acetylglucosaminylphosphatidylinositol+deacetylase+ac	0.418707	0.418707	#N/A
BP_GO:0007402_ganglion+mother+cell+fate+determination	0.511788	0.511788	#N/A
BP_GO:0008052_sensory+organ+boundary+specification	0.511788	0.511788	#N/A
MF_GO:0031750_D3+dopamine+receptor+binding	0.304068	0.304068	#N/A
MF_GO:0004655_porphobilinogen+synthase+activity	0.865135	#N/A	0.865135
BP_GO:0006474_N-terminal+protein+amino+acid+acetylation	0.275722	#N/A	0.275722
BP_GO:0060323_head+morphogenesis	0.63707	0.63707	#N/A
MF_GO:0047933_glucose-1,6-bisphosphate+synthase+activity	0.44655	0.44655	#N/A
BP_GO:0030488_tRNA+methylation	0.0324252	#N/A	0.0324252
MF_GO:0016429_tRNA+(adenine-N1-)-methyltransferase+activity	0.0324252	#N/A	0.0324252
MF_GO:0008526_phosphatidylinositol+transporter+activity	0.188993	#N/A	0.188993
MF_GO:0004904_interferon+receptor+activity	0.881454	0.881454	#N/A

BP_GO:0050746_regulation+of+lipoprotein+metabolic+process	0.16543	0.16543	#N/A
MF_GO:0008970_phospholipase+A1+activity	0.16543	0.16543	#N/A
MF_GO:0001848_complement+binding	0.358971	#N/A	0.358971
BP_GO:0009311_oligosaccharide+metabolic+process	0.764363	0.764363	#N/A
MF_GO:0004573_mannosyl-oligosaccharide+glucosidase+activity	0.764363	0.764363	#N/A
BP_GO:0043461_proton-transporting+ATP+synthase+complex+assembly	0.208799	0.208799	#N/A
CC_GO:0005971_ribonucleoside-diphosphate+reductase+complex	0.305856	0.305856	#N/A
BP_GO:0019532_oxalate+transport	0.372282	0.372282	#N/A
MF_GO:0015116_sulfate+transmembrane+transporter+activity	0.372282	0.372282	#N/A
MF_GO:0019531_oxalate+transmembrane+transporter+activity	0.372282	0.372282	#N/A
BP_GO:0032474_otolith+morphogenesis	0.654082	#N/A	0.654082
BP_GO:0006203_dGTP+catabolic+process	0.706671	#N/A	0.706671
BP_GO:0006930_substrate-bound+cell+migration,+cell+extension	0.430819	0.430819	#N/A
BP_GO:0021592_fourth+ventricle+development	0.430819	0.430819	#N/A
BP_GO:0021678_third+ventricle+development	0.430819	0.430819	#N/A
BP_GO:0009069_serine+family+amino+acid+metabolic+process	0.766209	0.766209	#N/A
BP_GO:0070179_D-serine+biosynthetic+process	0.766209	0.766209	#N/A
MF_GO:0018114_threonine+racemase+activity	0.766209	0.766209	#N/A
MF_GO:0030378_serine+racemase+activity	0.766209	0.766209	#N/A
BP_GO:0009972_cytidine+deamination	0.718886	#N/A	0.718886
MF_GO:0030226_apolipoprotein+receptor+activity	0.723604	#N/A	0.723604
MF_GO:0019007_N-acetylneuraminic+acid+phosphate+synthase+activity	0.306272	0.306272	#N/A
BP_GO:0030174_regulation+of+DNA+replication+initiation	0.409763	#N/A	0.409763
MF_GO:0004408_holocytochrome-c+synthase+activity	0.874777	0.874777	#N/A
BP_GO:0009913_epidermal+cell+differentiation	0.452248	0.452248	#N/A
MF_GO:0030305_heparanase+activity	0.308389	0.308389	#N/A
CC_GO:0030014_CCR4-NOT+complex	0.344142	0.344142	#N/A
MF_GO:0004561_alpha-N-acetylglucosaminidase+activity	0.375622	0.375622	#N/A
MF_GO:0050277_sedoheptulokinase+activity	0.476561	0.476561	#N/A
BP_GO:0050983_spermidine+catabolic+process+to+deoxyhypusine,+using+c	0.223213	0.223213	#N/A
MF_GO:0034038_deoxyhypusine+synthase+activity	0.223213	0.223213	#N/A
BP_GO:0046874_quinolinate+metabolic+process	0.165367	0.165367	#N/A
MF_GO:0001760_aminocarboxymuconate-semialdehyde+decarboxylase+act	0.165367	0.165367	#N/A
BP_GO:0008054_cyclin+catabolic+process	0.708964	0.708964	#N/A
BP_GO:0002756_MyD88-independent+toll-like+receptor+signaling+pathway	0.689882	#N/A	0.689882
BP_GO:0032481_positive+regulation+of+type+I+interferon+production	0.689882	#N/A	0.689882
BP_GO:0006235_dTTP+biosynthetic+process	0.283784	#N/A	0.283784
BP_GO:0002769_natural+killer+cell+inhibitory+signaling+pathway	0.681854	0.681854	#N/A
BP_GO:0046886_positive+regulation+of+hormone+biosynthetic+process	0.173571	0.173571	#N/A
MF_GO:0043758_acetate-CoA+ligase+(ADP-forming)+activity	0.877043	#N/A	0.877043
BP_GO:0007231_osmosensory+signaling+pathway	0.916002	#N/A	0.916002
BP_GO:0030103_vasopressin+secretion	0.916002	#N/A	0.916002
BP_GO:0042538_hyperosmotic+salinity+response	0.916002	#N/A	0.916002
MF_GO:0005034_osmosensor+activity	0.916002	#N/A	0.916002
MF_GO:0004463_leukotriene-A4+hydrolase+activity	0.639035	0.639035	#N/A
BP_GO:0015918_sterol+transport	0.242707	0.242707	#N/A
BP_GO:0055092_sterol+homeostasis	0.242707	0.242707	#N/A

BP_GO:0046825_regulation+of+protein+export+from+nucleus	0.526482	#N/A	0.526482
BP_GO:0048609_reproductive+process+in+a+multicellular+organism	0.526482	#N/A	0.526482
BP_GO:0051463_negative+regulation+of+cortisol+secretion	0.526482	#N/A	0.526482
BP_GO:0060125_negative+regulation+of+growth+hormone+secretion	0.526482	#N/A	0.526482
MF_GO:0051428_peptide+hormone+receptor+binding	0.526482	#N/A	0.526482
BP_GO:0006842_tricarboxylic+acid+transport	0.852965	#N/A	0.852965
BP_GO:0015744_succinate+transport	0.852965	#N/A	0.852965
MF_GO:0015141_succinate+transmembrane+transporter+activity	0.852965	#N/A	0.852965
MF_GO:0015142_tricarboxylic+acid+transmembrane+transporter+activity	0.852965	#N/A	0.852965
BP_GO:0033605_positive+regulation+of+catecholamine+secretion	0.800472	0.800472	#N/A
BP_GO:0046007_negative+regulation+of+activated+T+cell+proliferation	0.437082	0.437082	#N/A
MF_GO:0019900_kinase+binding	0.129829	0.129829	#N/A
BP_GO:0002082_regulation+of+oxidative+phosphorylation	0.904917	0.904917	#N/A
BP_GO:0006568_tryptophan+metabolic+process	0.904917	0.904917	#N/A
BP_GO:0015677_copper+ion+import	0.904917	0.904917	#N/A
BP_GO:0018205_peptidyl-lysine+modification	0.904917	0.904917	#N/A
BP_GO:0042093_T-helper+cell+differentiation	0.904917	0.904917	#N/A
BP_GO:0042414_epinephrine+metabolic+process	0.904917	0.904917	#N/A
BP_GO:0048553_negative+regulation+of+metalloenzyme+activity	0.904917	0.904917	#N/A
BP_GO:0051542_elastin+biosynthetic+process	0.904917	0.904917	#N/A
BP_GO:0060003_copper+ion+export	0.904917	0.904917	#N/A
MF_GO:0016532_superoxide+dismutase+copper+chaperone+activity	0.904917	0.904917	#N/A
MF_GO:0047988_hydroxyacid-oxoacid+transhydrogenase+activity	0.879372	#N/A	0.879372
BP_GO:0031508_centromeric+heterochromatin+formation	0.751361	#N/A	0.751361
MF_GO:0008482_sulfite+oxidase+activity	0.79211	0.79211	#N/A
BP_GO:0002040_sprouting+angiogenesis	0.0990649	0.0990649	#N/A
BP_GO:0007440_foregut+morphogenesis	0.0990649	0.0990649	#N/A
BP_GO:0045618_positive+regulation+of+keratinocyte+differentiation	0.0990649	0.0990649	#N/A
BP_GO:0046533_negative+regulation+of+photoreceptor+cell+differentiatior	0.0990649	0.0990649	#N/A
BP_GO:0033025_regulation+of+mast+cell+apoptosis	0.955348	0.955348	#N/A
BP_GO:0048870_cell+motility	0.955348	0.955348	#N/A
BP_GO:0051088_PMA-inducible+membrane+protein+ectodomain+proteoly:	0.955348	0.955348	#N/A
MF_GO:0047598_7-dehydrocholesterol+reductase+activity	0.0779934	0.0779934	#N/A
MF_GO:0004733_pyridoxamine-phosphate+oxidase+activity	0.922744	0.922744	#N/A
MF_GO:0008609_alkylglycerone-phosphate+synthase+activity	0.335943	0.335943	#N/A
MF_GO:0008444_CDP-diacylglycerol-glycerol-3-phosphate+3-phosphatidyltr	0.241896	0.241896	#N/A
MF_GO:0004085_butyryl-CoA+dehydrogenase+activity	0.00916758	0.00916758	#N/A
BP_GO:0006027_glycosaminoglycan+catabolic+process	0.22323	0.22323	#N/A
MF_GO:0004363_glutathione+synthase+activity	0.277042	#N/A	0.277042
MF_GO:0003834_beta-carotene+15,15'-monooxygenase+activity	0.226399	0.226399	#N/A
MF_GO:0000254_C-4+methylsterol+oxidase+activity	0.321859	#N/A	0.321859
MF_GO:0016531_copper+chaperone+activity	0.751193	#N/A	0.751193
MF_GO:0050080_malonyl-CoA+decarboxylase+activity	0.970257	0.970257	#N/A
BP_GO:0030208_dermatan+sulfate+biosynthetic+process	0.720646	#N/A	0.720646
MF_GO:0004506_squalene+monooxygenase+activity	0.202737	0.202737	#N/A
MF_GO:0003943_N-acetylgalactosamine-4-sulfatase+activity	0.431541	#N/A	0.431541
MF_GO:0008116_prostaglandin-I+synthase+activity	0.583998	#N/A	0.583998

MF_GO:0008545_JUN+kinase+kinase+activity	0.509896	0.509896	#N/A
BP_GO:0043171_peptide+catabolic+process	0.921018	0.921018	#N/A
MF_GO:0008240_tripeptidyl-peptidase+activity	0.921018	0.921018	#N/A
MF_GO:0047708_biotinidase+activity	0.459181	0.459181	#N/A
CC_GO:0030665_clathrin+coated+vesicle+membrane	0.700981	0.700981	#N/A
CC_GO:0009288_flagellin-based+flagellum	0.68045	0.68045	#N/A
BP_GO:0021695_cerebellar+cortex+development	0.286749	0.286749	#N/A
BP_GO:0021697_cerebellar+cortex+formation	0.286749	0.286749	#N/A
BP_GO:0031914_negative+regulation+of+synaptic+plasticity	0.286749	0.286749	#N/A
BP_GO:0032801_receptor+catabolic+process	0.286749	0.286749	#N/A
BP_GO:0033136_serine+phosphorylation+of+STAT3+protein	0.286749	0.286749	#N/A
BP_GO:0045956_positive+regulation+of+calcium+ion-dependent+exocytosis	0.286749	0.286749	#N/A
BP_GO:0046826_negative+regulation+of+protein+export+from+nucleus	0.286749	0.286749	#N/A
MF_GO:0030549_acetylcholine+receptor+activator+activity	0.286749	0.286749	#N/A
MF_GO:0043125_ErbB-3+class+receptor+binding	0.286749	0.286749	#N/A
BP_GO:0051403_stress-activated+MAPK+cascade	0.812307	0.812307	#N/A
MF_GO:0042806_fucose+binding	0.866749	0.866749	#N/A
MF_GO:0033925_mannosyl-glycoprotein+endo-beta-N-acetylglucosaminida	0.0215584	0.0215584	#N/A
MF_GO:0034602_oxoglutarate+dehydrogenase+(NAD+)+activity	0.941136	0.941136	#N/A
BP_GO:0001971_negative+regulation+of+activation+of+membrane+attack+	0.352974	0.352974	#N/A
BP_GO:0045918_negative+regulation+of+cytolysis	0.352974	0.352974	#N/A
MF_GO:0050815_phosphoserine+binding	0.908557	0.908557	#N/A
MF_GO:0050816_phosphothreonine+binding	0.908557	0.908557	#N/A
MF_GO:0008761_UDP-N-acetylglucosamine+2-epimerase+activity	0.733522	0.733522	#N/A
MF_GO:0004382_guanosine-diphosphatase+activity	0.737359	0.737359	#N/A
MF_GO:0045134_uridine-diphosphatase+activity	0.737359	0.737359	#N/A
BP_GO:0006531_aspartate+metabolic+process	0.628248	0.628248	#N/A
MF_GO:0008445_D-aspartate+oxidase+activity	0.628248	0.628248	#N/A
CC_GO:0000407_pre-autophagosomal+structure	0.59264	0.59264	#N/A
BP_GO:0048242_epinephrine+secretion	0.287419	0.287419	#N/A
MF_GO:0004729_protoporphyrinogen+oxidase+activity	0.355422	#N/A	0.355422
MF_GO:0004061_arylformamidase+activity	0.424575	0.424575	#N/A
BP_GO:0030322_stabilization+of+membrane+potential	0.722239	0.722239	#N/A
BP_GO:0042592_homeostatic+process	0.21755	0.21755	#N/A
BP_GO:0032682_negative+regulation+of+chemokine+production	0.72603	#N/A	0.72603
BP_GO:0014067_negative+regulation+of+phosphoinositide+3-kinase+cascac	0.205316	0.205316	#N/A
BP_GO:0050918_positive+chemotaxis	0.205316	0.205316	#N/A
BP_GO:0045724_positive+regulation+of+flagellum+assembly	0.808332	#N/A	0.808332
MF_GO:0008890_glycine+C-acetyltransferase+activity	0.107963	0.107963	#N/A
BP_GO:0010046_response+to+mycotoxin	0.264179	#N/A	0.264179
MF_GO:0004572_mannosyl-oligosaccharide+1,3-1,6-alpha-mannosidase+act	0.959003	0.959003	#N/A
BP_GO:0045626_negative+regulation+of+T-helper+1+cell+differentiation	0.719588	0.719588	#N/A
BP_GO:0007260_tyrosine+phosphorylation+of+STAT+protein	0.494731	0.494731	#N/A
BP_GO:0042517_positive+regulation+of+tyrosine+phosphorylation+of+Stat3	0.494731	0.494731	#N/A
MF_GO:0004696_glycogen+synthase+kinase+3+activity	0.998936	0.998936	#N/A
BP_GO:0051685_maintenance+of+ER+location	0.652987	#N/A	0.652987
BP_GO:0042231_interleukin-13+biosynthetic+process	0.0137055	0.0137055	#N/A

BP_GO:0045906_negative+regulation+of+vasoconstriction	0.52338	0.52338	#N/A
BP_GO:0015724_formate+transport	0.105127	0.105127	#N/A
MF_GO:0015499_formate+transmembrane+transporter+activity	0.105127	0.105127	#N/A
CC_GO:0008352_katanin+complex	0.9608	#N/A	0.9608
BP_GO:0019682_glyceraldehyde-3-phosphate+metabolic+process	0.435639	0.435639	#N/A
MF_GO:0004807_triose-phosphate+isomerase+activity	0.435639	0.435639	#N/A
BP_GO:0010614_negative+regulation+of+cardiac+muscle+hypertrophy	0.489394	#N/A	0.489394
MF_GO:0004137_deoxycytidine+kinase+activity	0.130484	0.130484	#N/A
MF_GO:0016784_3-mercaptopyruvate+sulfurtransferase+activity	0.419837	0.419837	#N/A
MF_GO:0015362_high+affinity+sodium:dicarboxylate+symporter+activity	0.598815	0.598815	#N/A
BP_GO:0002029_desensitization+of+G-protein+coupled+receptor+protein+s	0.626767	0.626767	#N/A
BP_GO:0046856_phosphoinositide+dephosphorylation	0.475851	0.475851	#N/A
BP_GO:0051895_negative+regulation+of+focal+adhesion+formation	0.475851	0.475851	#N/A
MF_GO:0016314_phosphatidylinositol-3,4,5-trisphosphate+3-phosphatase+a	0.475851	0.475851	#N/A
MF_GO:0051800_phosphatidylinositol-3,4-bisphosphate+3-phosphatase+act	0.475851	0.475851	#N/A
MF_GO:0004738_pyruvate+dehydrogenase+activity	0.733236	0.733236	#N/A
MF_GO:0017106_activin+inhibitor+activity	0.652026	#N/A	0.652026
MF_GO:0016603_glutaminyl-peptide+cyclotransferase+activity	0.501166	0.501166	#N/A
BP_GO:0007296_vitellogenesis	0.956367	#N/A	0.956367
MF_GO:0016262_protein+N-acetylglucosaminyltransferase+activity	0.919293	0.919293	#N/A
MF_GO:0033872_[heparan+sulfate]-glucosamine+3-sulfotransferase+3+activ	0.225256	0.225256	#N/A
BP_GO:0002827_positive+regulation+of+T-helper+1+type+immune+respons	0.317762	0.317762	#N/A
MF_GO:0045509_interleukin-27+receptor+activity	0.317762	0.317762	#N/A
BP_GO:0021572_rhombomere+6+development	0.495102	#N/A	0.495102
BP_GO:0000055_ribosomal+large+subunit+export+from+nucleus	0.0447723	#N/A	0.0447723
MF_GO:0005139_interleukin-7+receptor+binding	0.383951	0.383951	#N/A
MF_GO:0042805_actinin+binding	0.829095	0.829095	#N/A
BP_GO:0001986_negative+regulation+of+the+force+of+heart+contraction+c	0.0521351	0.0521351	#N/A
CC_GO:0042599_lamellar+body	0.750056	0.750056	#N/A
BP_GO:0002457_T+cell+antigen+processing+and+presentation	0.698312	0.698312	#N/A
BP_GO:0009401_phosphoenolpyruvate-dependent+sugar+phosphotransfera	0.715541	0.715541	#N/A
BP_GO:0000046_autophagic+vacuole+fusion	0.185993	#N/A	0.185993
BP_GO:0016236_macroautophagy	0.185993	#N/A	0.185993
BP_GO:0051480_cytosolic+calcium+ion+homeostasis	0.185993	#N/A	0.185993
CC_GO:0044433_cytoplasmic+vesicle+part	0.185993	#N/A	0.185993
BP_GO:0043089_positive+regulation+of+Cdc42+GTPase+activity	0.575789	0.575789	#N/A
BP_GO:0045899_positive+regulation+of+transcriptional+preinitiation+comp	0.480748	0.480748	#N/A
CC_GO:0000506_glycosylphosphatidylinositol-N-acetylglucosaminyltransfera	0.168073	0.168073	#N/A
BP_GO:0046069_cGMP+catabolic+process	0.718451	#N/A	0.718451
MF_GO:0004112_cyclic-nucleotide+phosphodiesterase+activity	0.718451	#N/A	0.718451
MF_GO:0015278_calcium-release+channel+activity	0.118469	0.118469	#N/A
MF_GO:0004347_glucose-6-phosphate+isomerase+activity	0.947592	0.947592	#N/A
BP_GO:0018120_peptidyl-arginine+ADP-ribosylation	0.000431851	0.000431851	#N/A
CC_GO:0000275_mitochondrial+proton-transporting+ATP+synthase+comple	0.622541	0.622541	#N/A
MF_GO:0017047_adrenocorticotropin-releasing+hormone+binding	0.175091	#N/A	0.175091
BP_GO:0031062_positive+regulation+of+histone+methylation	0.0523786	0.0523786	#N/A
BP_GO:0051154_negative+regulation+of+striated+muscle+cell+differentiatio	0.187936	#N/A	0.187936

MF_GO:0001618_viral+receptor+activity	0.120217	0.120217	#N/A
MF_GO:0005175_CD27+receptor+binding	0.120217	0.120217	#N/A
BP_GO:0001550_ovarian+cumulus+expansion	0.829094	#N/A	0.829094
BP_GO:0018208_peptidyl-proline+modification	0.586015	#N/A	0.586015
BP_GO:0031274_positive+regulation+of+pseudopodium+formation	0.472528	0.472528	#N/A
BP_GO:0042483_negative+regulation+of+odontogenesis	0.472528	0.472528	#N/A
BP_GO:0051171_regulation+of+nitrogen+compound+metabolic+process	0.472528	0.472528	#N/A
BP_GO:0051988_regulation+of+attachment+of+spindle+microtubules+to+ki	0.472528	0.472528	#N/A
MF_GO:0042577_lipid+phosphatase+activity	0.781327	#N/A	0.781327
MF_GO:0001691_pseudophosphatase+activity	0.145245	0.145245	#N/A
BP_GO:0019255_glucose+1-phosphate+metabolic+process	0.0311313	0.0311313	#N/A
MF_GO:0004610_phosphoacetylglucosamine+mutase+activity	0.0311313	0.0311313	#N/A
MF_GO:0008520_L-ascorbate:sodium+symporter+activity	0.942368	0.942368	#N/A
BP_GO:0045185_maintenance+of+protein+location	0.461538	0.461538	#N/A
MF_GO:0050827_toxin+receptor+binding	0.484677	#N/A	0.484677
BP_GO:0046853_inositol+and+derivative+phosphorylation	0.728923	0.728923	#N/A
MF_GO:0000824_inositol+tetrakisphosphate+3-kinase+activity	0.728923	0.728923	#N/A
MF_GO:0004163_diphosphomevalonate+decarboxylase+activity	0.0179275	0.0179275	#N/A
MF_GO:0004156_dihydropteroate+synthase+activity	0.108336	0.108336	#N/A
MF_GO:0008705_methionine+synthase+activity	0.108336	0.108336	#N/A
MF_GO:0004633_phosphopantothenoylcysteine+decarboxylase+activity	0.828584	#N/A	0.828584
BP_GO:0016082_synaptic+vesicle+priming	0.876993	#N/A	0.876993
BP_GO:0015014_heparan+sulfate+proteoglycan+biosynthetic+process,+poly	0.165995	0.165995	#N/A
BP_GO:0030202_heparin+metabolic+process	0.165995	0.165995	#N/A
MF_GO:0004394_heparan+sulfate+2-O-sulfotransferase+activity	0.165995	0.165995	#N/A
BP_GO:0046087_cytidine+metabolic+process	0.707704	#N/A	0.707704
BP_GO:0033108_mitochondrial+respiratory+chain+complex+assembly	0.363797	0.363797	#N/A
BP_GO:0009200_deoxyribonucleoside+triphosphate+metabolic+process	0.0809794	0.0809794	#N/A
MF_GO:0005384_manganese+ion+transmembrane+transporter+activity	0.178753	0.178753	#N/A
MF_GO:0008398_sterol+14-demethylase+activity	0.290401	0.290401	#N/A
BP_GO:0042986_positive+regulation+of+amyloid+precursor+protein+biosyn	0.182973	#N/A	0.182973
CC_GO:0005675_holo+TFIIH+complex	0.0926365	#N/A	0.0926365
BP_GO:0032229_negative+regulation+of+synaptic+transmission,+GABAergic	0.605203	0.605203	#N/A
BP_GO:0015670_carbon+dioxide+transport	0.153817	0.153817	#N/A
BP_GO:0043308_eosinophil+degranulation	0.0249089	0.0249089	#N/A
CC_GO:0031902_late+endosome+membrane	0.0249089	0.0249089	#N/A
MF_GO:0005283_sodium:amino+acid+symporter+activity	0.21765	#N/A	0.21765
MF_GO:0004578_chitobiosyldiphosphodolichol+beta-mannosyltransferase+	0.741006	0.741006	#N/A
MF_GO:0004395_hexaprenyldihydroxybenzoate+methyltransferase+activity	0.599188	#N/A	0.599188
MF_GO:0008425_2-polyprenyl-6-methoxy-1,4-benzoquinone+methyltransfe	0.599188	#N/A	0.599188
BP_GO:0043071_positive+regulation+of+non-apoptotic+programmed+cell+c	0.245142	#N/A	0.245142
BP_GO:0006766_vitamin+metabolic+process	0.540309	#N/A	0.540309
BP_GO:0016074_snoRNA+metabolic+process	0.28411	#N/A	0.28411
MF_GO:0003826_alpha-ketoacid+dehydrogenase+activity	0.62952	0.62952	#N/A
MF_GO:0004677_DNA-dependent+protein+kinase+activity	0.84668	0.84668	#N/A
MF_GO:0003947_(N-acetylneuraminyl)-galactosylglucosylceramide+N-acety	0.382326	0.382326	#N/A
BP_GO:0006041_glucosamine+metabolic+process	0.376578	0.376578	#N/A

BP_GO:0046370_fructose+biosynthetic+process	0.376578	0.376578	#N/A
BP_GO:0045026_plasma+membrane+fusion	0.57318	#N/A	0.57318
BP_GO:0006556_S-adenosylmethionine+biosynthetic+process	0.320674	0.320674	#N/A
BP_GO:0035083_cilium+axoneme+assembly	0.38725	0.38725	#N/A
BP_GO:0050658_RNA+transport	0.936648	0.936648	#N/A
BP_GO:0016553_base+conversion+or+substitution+editing	0.746759	0.746759	#N/A
MF_GO:0043843_ADP-specific+glucokinase+activity	0.98015	0.98015	#N/A
MF_GO:0030586_[methionine+synthase]+reductase+activity	0.210685	0.210685	#N/A
MF_GO:0047138_aquacobalamin+reductase+activity	0.210685	0.210685	#N/A
BP_GO:0055069_zinc+ion+homeostasis	0.818468	#N/A	0.818468
MF_GO:0004077_biotin-[acetyl-CoA-carboxylase]+ligase+activity	0.345496	0.345496	#N/A
MF_GO:0004078_biotin-[methylcrotonoyl-CoA-carboxylase]+ligase+activity	0.345496	0.345496	#N/A
MF_GO:0004079_biotin-[methylmalonyl-CoA-carboxytransferase]+ligase+ac	0.345496	0.345496	#N/A
MF_GO:0004080_biotin-[propionyl-CoA-carboxylase+(ATP-hydrolyzing)]+liga	0.345496	0.345496	#N/A
BP_GO:0045602_negative+regulation+of+endothelial+cell+differentiation	0.969285	#N/A	0.969285
BP_GO:0006931_substrate-bound+cell+migration,+cell+attachment+to+subs	0.0352734	#N/A	0.0352734
BP_GO:0045601_regulation+of+endothelial+cell+differentiation	0.573578	#N/A	0.573578
BP_GO:0051569_regulation+of+histone+H3-K4+methylation	0.46178	0.46178	#N/A
BP_GO:0000381_regulation+of+alternative+nuclear+mRNA+splicing,+via+sp	0.951217	0.951217	#N/A
BP_GO:0007518_myoblast+cell+fate+determination	0.951217	0.951217	#N/A
MF_GO:0003944_N-acetylglucosamine-1-phosphodiester+alpha-N-acetylglu	0.255707	0.255707	#N/A
MF_GO:0000104_succinate+dehydrogenase+activity	0.0841972	#N/A	0.0841972
BP_GO:0006220_pyrimidine+nucleotide+metabolic+process	0.026092	#N/A	0.026092
BP_GO:0046108_uridine+metabolic+process	0.026092	#N/A	0.026092
BP_GO:0021935_granule+cell+precursor+tangential+migration	0.0732981	0.0732981	#N/A
MF_GO:0004132_dCMP+deaminase+activity	0.678796	0.678796	#N/A
BP_GO:0007093_mitotic+cell+cycle+checkpoint	0.388934	0.388934	#N/A
MF_GO:0008455_alpha-1,6-mannosylglycoprotein+2-beta-N-acetylglucosam	0.656539	#N/A	0.656539
BP_GO:0051964_negative+regulation+of+synaptogenesis	0.667091	0.667091	#N/A
MF_GO:0004324_ferredoxin-NADP++reductase+activity	0.0739788	0.0739788	#N/A
MF_GO:0004493_methylmalonyl-CoA+epimerase+activity	0.561738	#N/A	0.561738
MF_GO:0008262_importin-alpha+export+receptor+activity	0.913295	#N/A	0.913295
BP_GO:0045083_negative+regulation+of+interleukin-12+biosynthetic+proce	0.386273	0.386273	#N/A
BP_GO:0007521_muscle+cell+fate+determination	0.12182	#N/A	0.12182
MF_GO:0004466_long-chain-acyl-CoA+dehydrogenase+activity	0.012398	0.012398	#N/A
MF_GO:0001735_prenylcysteine+oxidase+activity	0.859232	0.859232	#N/A
MF_GO:0003882_CDP-diacylglycerol-serine+O-phosphatidyltransferase+acti	0.993839	#N/A	0.993839
MF_GO:0030791_arsenite+methyltransferase+activity	0.000589013	0.000589013	#N/A
MF_GO:0033882_choloyl-CoA+hydrolase+activity	0.0536681	#N/A	0.0536681
MF_GO:0004567_beta-mannosidase+activity	0.387155	0.387155	#N/A
MF_GO:0046935_1-phosphatidylinositol-3-kinase+regulator+activity	0.919471	0.919471	#N/A
BP_GO:0002315_marginal+zone+B+cell+differentiation	0.0553116	#N/A	0.0553116
BP_GO:0019730_antimicrobial+humoral+response	0.0553116	#N/A	0.0553116
BP_GO:0042536_negative+regulation+of+tumor+necrosis+factor+biosynthet	0.0553116	#N/A	0.0553116
BP_GO:0045064_T-helper+2+cell+differentiation	0.0553116	#N/A	0.0553116
BP_GO:0045082_positive+regulation+of+interleukin-10+biosynthetic+proces	0.0553116	#N/A	0.0553116
MF_GO:0019002_GMP+binding	0.000638167	0.000638167	#N/A

BP_GO:0021759_globus+pallidus+development	0.298578	0.298578	#N/A
MF_GO:0008108_UDP-glucose:hexose-1-phosphate+uridylyltransferase+act	0.155237	0.155237	#N/A
BP_GO:0006296_nucleotide-excision+repair,+DNA+incision,+5'-to+lesion	0.457609	#N/A	0.457609
MF_GO:0004098_cerebroside-sulfatase+activity	0.737736	0.737736	#N/A
MF_GO:0005006_epidermal+growth+factor+receptor+activity	0.11289	#N/A	0.11289
BP_GO:0001866_NK+T+cell+proliferation	0.572699	0.572699	#N/A
BP_GO:0045062_extrathymic+T+cell+selection	0.572699	0.572699	#N/A
BP_GO:0050691_regulation+of+defense+response+to+virus+by+host	0.572699	0.572699	#N/A
CC_GO:0016011_dystroglycan+complex	0.686964	0.686964	#N/A
BP_GO:0042026_protein+refolding	0.404364	#N/A	0.404364
BP_GO:0045585_positive+regulation+of+cytotoxic+T+cell+differentiation	0.404364	#N/A	0.404364
CC_GO:0005719_nuclear+euchromatin	0.914909	#N/A	0.914909
MF_GO:0047710_bis(5'-adenosyl)-triphosphatase+activity	0.118625	#N/A	0.118625
BP_GO:0051865_protein+autoubiquitination	0.42196	0.42196	#N/A
BP_GO:0006430_lysyl-tRNA+aminoacylation	0.942315	0.942315	#N/A
MF_GO:0004824_lysine-tRNA+ligase+activity	0.942315	0.942315	#N/A
MF_GO:0004588_orotate+phosphoribosyltransferase+activity	0.528247	#N/A	0.528247
MF_GO:0004590_orotidine-5'-phosphate+decarboxylase+activity	0.528247	#N/A	0.528247
BP_GO:0019428_allantoin+biosynthetic+process	0.764186	0.764186	#N/A
MF_GO:0042328_heparan+sulfate+N-acetylglucosaminyltransferase+activity	0.288796	0.288796	#N/A
MF_GO:0030331_estrogen+receptor+binding	0.470278	0.470278	#N/A
MF_GO:0046966_thyroid+hormone+receptor+binding	0.470278	0.470278	#N/A
MF_GO:0046975_histone+lysine+N-methyltransferase+activity+(H3-K36+spe	0.470278	0.470278	#N/A
MF_GO:0008924_malate+dehydrogenase+(acceptor)+activity	0.507016	0.507016	#N/A
BP_GO:0002314_germinal+center+B+cell+differentiation	0.173685	0.173685	#N/A
BP_GO:0002906_negative+regulation+of+mature+B+cell+apoptosis	0.173685	0.173685	#N/A
BP_GO:0006154_adenosine+catabolic+process	0.173685	0.173685	#N/A
BP_GO:0006157_deoxyadenosine+catabolic+process	0.173685	0.173685	#N/A
BP_GO:0010460_positive+regulation+of+heart+rate	0.173685	0.173685	#N/A
BP_GO:0046061_dATP+catabolic+process	0.173685	0.173685	#N/A
BP_GO:0046101_hypoxanthine+biosynthetic+process	0.173685	0.173685	#N/A
BP_GO:0046103_inosine+biosynthetic+process	0.173685	0.173685	#N/A
BP_GO:0046111_xanthine+biosynthetic+process	0.173685	0.173685	#N/A
BP_GO:0021870_Cajal-Retzius+cell+differentiation	0.113239	#N/A	0.113239
BP_GO:0051966_regulation+of+synaptic+transmission,+glutamatergic	0.113239	#N/A	0.113239
BP_GO:0060075_regulation+of+resting+membrane+potential	0.113239	#N/A	0.113239
MF_GO:0004676_3-phosphoinositide-dependent+protein+kinase+activity	0.392195	0.392195	#N/A
MF_GO:0004556_alpha-amylase+activity	0.986232	#N/A	0.986232
BP_GO:0019046_reactivation+of+latent+virus	0.696398	0.696398	#N/A
BP_GO:0007635_chemosensory+behavior	0.279148	0.279148	#N/A
MF_GO:0004699_calcium-independent+protein+kinase+C+activity	0.279148	0.279148	#N/A
BP_GO:0060319_primitive+erythrocyte+differentiation	0.240564	0.240564	#N/A
BP_GO:0048592_eye+morphogenesis	0.283879	0.283879	#N/A
MF_GO:0004494_methylmalonyl-CoA+mutase+activity	0.497715	0.497715	#N/A
MF_GO:0005521_lamin+binding	0.388377	0.388377	#N/A
MF_GO:0047545_2-hydroxyglutarate+dehydrogenase+activity	0.0677675	#N/A	0.0677675
BP_GO:0021960_anterior+commissure+morphogenesis	0.162858	#N/A	0.162858

BP_GO:0045226_extracellular+polysaccharide+biosynthetic+process	0.700517	#N/A	0.700517
MF_GO:0008831_dTDP-4-dehydrorhamnose+reductase+activity	0.700517	#N/A	0.700517
CC_GO:0043626_PCNA+complex	0.439975	0.439975	#N/A
MF_GO:0030337_DNA+polymerase+processivity+factor+activity	0.439975	0.439975	#N/A
MF_GO:0005035_death+receptor+activity	0.247085	#N/A	0.247085
MF_GO:0048406_nerve+growth+factor+binding	0.247085	#N/A	0.247085
BP_GO:0015755_fructose+transport	0.648864	#N/A	0.648864
BP_GO:0015757_galactose+transport	0.648864	#N/A	0.648864
MF_GO:0005354_galactose+transmembrane+transporter+activity	0.648864	#N/A	0.648864
MF_GO:0005536_glucose+binding	0.648864	#N/A	0.648864
MF_GO:0015284_fructose+uniporter+activity	0.648864	#N/A	0.648864
BP_GO:0048878_chemical+homeostasis	0.879853	#N/A	0.879853
MF_GO:0003940_L-iduronidase+activity	0.879853	#N/A	0.879853
BP_GO:0052565_response+to+defense-related+host+nitric+oxide+productic	0.685731	#N/A	0.685731
MF_GO:0015446_arsenite+transmembrane-transporting+ATPase+activity	0.668351	0.668351	#N/A
BP_GO:0000042_protein+targeting+to+Golgi	0.908367	#N/A	0.908367
MF_GO:0047936_glucose+1-dehydrogenase+activity	0.953327	0.953327	#N/A
BP_GO:0006662_glycerol+ether+metabolic+process	0.314623	#N/A	0.314623
BP_GO:0001830_trophectodermal+cell+fate+commitment	0.428358	#N/A	0.428358
MF_GO:0019785_ISG15-specific+protease+activity	0.00254819	0.00254819	#N/A
MF_GO:0004694_eukaryotic+translation+initiation+factor+2alpha+kinase+a	0.376394	0.376394	#N/A
MF_GO:0016229_steroid+dehydrogenase+activity	0.804614	#N/A	0.804614
MF_GO:0046899_nucleoside+triphosphate+adenylate+kinase+activity	0.584495	#N/A	0.584495
BP_GO:0042226_interleukin-6+biosynthetic+process	0.84712	#N/A	0.84712
BP_GO:0021897_forebrain+astrocyte+development	0.488703	0.488703	#N/A
BP_GO:0042992_negative+regulation+of+transcription+factor+import+into+	0.488703	0.488703	#N/A
BP_GO:0045685_regulation+of+glial+cell+differentiation	0.488703	0.488703	#N/A
BP_GO:0045762_positive+regulation+of+adenylate+cyclase+activity	0.488703	0.488703	#N/A
BP_GO:0046929_negative+regulation+of+neurotransmitter+secretion	0.488703	0.488703	#N/A
CC_GO:0005845_mRNA+cap+binding+complex	0.958205	#N/A	0.958205
MF_GO:0004482_mRNA+(guanine-N7-)-methyltransferase+activity	0.958205	#N/A	0.958205
MF_GO:0047800_cysteamine+dioxygenase+activity	0.199397	0.199397	#N/A
BP_GO:0015732_prostaglandin+transport	0.908407	0.908407	#N/A
MF_GO:0015132_prostaglandin+transmembrane+transporter+activity	0.908407	0.908407	#N/A
BP_GO:0007035_vacuolar+acidification	0.0392464	#N/A	0.0392464
BP_GO:0032799_low-density+lipoprotein+receptor+metabolic+process	0.796181	0.796181	#N/A
BP_GO:0032803_regulation+of+low-density+lipoprotein+receptor+catabolic	0.796181	0.796181	#N/A
BP_GO:0032764_negative+regulation+of+mast+cell+cytokine+production	0.00395928	#N/A	0.00395928
BP_GO:0042092_T-helper+2+type+immune+response	0.00395928	#N/A	0.00395928
BP_GO:0043380_regulation+of+memory+T+cell+differentiation	0.00395928	#N/A	0.00395928
MF_GO:0003874_6-pyruvoyltetrahydropterin+synthase+activity	0.522564	0.522564	#N/A
BP_GO:0034474_U2+snRNA+3'-end+processing	0.226192	0.226192	#N/A
BP_GO:0033371_T+cell+secretory+granule+organization	0.717614	#N/A	0.717614
BP_GO:0033373_maintenance+of+protease+location+in+mast+cell+secretor	0.717614	#N/A	0.717614
BP_GO:0033382_maintenance+of+granzyme+B+location+in+T+cell+secretor	0.717614	#N/A	0.717614
CC_GO:0042629_mast+cell+granule	0.717614	#N/A	0.717614
BP_GO:0001777_T+cell+homeostatic+proliferation	0.631452	#N/A	0.631452

BP_GO:0030264_nuclear+fragmentation+during+apoptosis	0.631452	#N/A	0.631452
BP_GO:0033599_regulation+of+mammary+gland+epithelial+cell+proliferatic	0.631452	#N/A	0.631452
MF_GO:0051434_BH3+domain+binding	0.631452	#N/A	0.631452
BP_GO:0030578_PML+body+organization	0.139463	0.139463	#N/A
BP_GO:0045343_regulation+of+MHC+class+I+biosynthetic+process	0.139463	0.139463	#N/A
MF_GO:0004991_parathyroid+hormone+receptor+activity	0.226623	0.226623	#N/A
BP_GO:0040032_post-embryonic+body+morphogenesis	0.720378	0.720378	#N/A
BP_GO:0002086_diaphragm+contraction	0.530862	0.530862	#N/A
BP_GO:0042461_photoreceptor+cell+development	0.654276	0.654276	#N/A
MF_GO:0004490_methylglutaconyl-CoA+hydratase+activity	0.869095	#N/A	0.869095
MF_GO:0001681_sialate+O-acetylesterase+activity	0.733324	0.733324	#N/A
BP_GO:0006650_glycerophospholipid+metabolic+process	0.685164	0.685164	#N/A
BP_GO:0006678_glucosylceramide+metabolic+process	0.685164	0.685164	#N/A
BP_GO:0006681_galactosylceramide+metabolic+process	0.685164	0.685164	#N/A
BP_GO:0009450_gamma-aminobutyric+acid+catabolic+process	0.685164	0.685164	#N/A
MF_GO:0004724_magnesium-dependent+protein+serine/threonine+phosph	0.884422	0.884422	#N/A
BP_GO:0006090_pyruvate+metabolic+process	0.179911	0.179911	#N/A
CC_GO:0005967_mitochondrial+pyruvate+dehydrogenase+complex	0.179911	0.179911	#N/A
CC_GO:0045254_pyruvate+dehydrogenase+complex	0.179911	0.179911	#N/A
MF_GO:0004742_dihydrolipoyllysine-residue+acetyltransferase+activity	0.179911	0.179911	#N/A
BP_GO:0006188_IMP+biosynthetic+process	0.683733	0.683733	#N/A
MF_GO:0003937_IMP+cyclohydrolase+activity	0.683733	0.683733	#N/A
MF_GO:0004643_phosphoribosylaminoimidazolecarboxamide+formyltransf	0.683733	0.683733	#N/A
BP_GO:0002369_T+cell+cytokine+production	0.730951	0.730951	#N/A
BP_GO:0042982_amyloid+precursor+protein+metabolic+process	0.730951	0.730951	#N/A
BP_GO:0048639_positive+regulation+of+developmental+growth	0.730951	0.730951	#N/A
BP_GO:0048729_tissue+morphogenesis	0.730951	0.730951	#N/A
BP_GO:0060022_hard+palate+development	0.730951	0.730951	#N/A
BP_GO:0006663_platelet+activating+factor+biosynthetic+process	0.13169	0.13169	#N/A
MF_GO:0047192_1-alkylglycerophosphocholine+O-acetyltransferase+activit	0.13169	0.13169	#N/A
BP_GO:0042492_gamma-delta+T+cell+differentiation	0.848638	#N/A	0.848638
BP_GO:0021512_spinal+cord+anterior/posterior+patterning	0.63978	0.63978	#N/A
BP_GO:0030704_vitelline+membrane+formation	0.148606	#N/A	0.148606
MF_GO:0008316_structural+constituent+of+vitelline+membrane	0.148606	#N/A	0.148606
MF_GO:0047316_glutamine-phenylpyruvate+transaminase+activity	0.436806	0.436806	#N/A
BP_GO:0010159_specification+of+organ+position	0.372438	#N/A	0.372438
MF_GO:0005171_hepatocyte+growth+factor+receptor+binding	0.6028	#N/A	0.6028
MF_GO:0008510_sodium:bicarbonate+symporter+activity	0.129142	#N/A	0.129142
BP_GO:0006680_glucosylceramide+catabolic+process	0.773206	#N/A	0.773206
MF_GO:0033781_cholesterol+24-hydroxylase+activity	0.226908	#N/A	0.226908
MF_GO:0045437_uridine+nucleosidase+activity	0.00787354	#N/A	0.00787354
BP_GO:0045833_negative+regulation+of+lipid+metabolic+process	0.140889	0.140889	#N/A
BP_GO:0050996_positive+regulation+of+lipid+catabolic+process	0.140889	0.140889	#N/A
MF_GO:0008448_N-acetylglucosamine-6-phosphate+deacetylase+activity	0.800349	0.800349	#N/A
BP_GO:0006622_protein+targeting+to+lysosome	0.95844	0.95844	#N/A
BP_GO:0048278_vesicle+docking	0.803549	0.803549	#N/A
BP_GO:0030950_establishment+or+maintenance+of+actin+cytoskeleton+pc	0.842475	0.842475	#N/A

BP_GO:0045175_basal+protein+localization	0.842475	0.842475	#N/A
BP_GO:0035308_negative+regulation+of+protein+amino+acid+dephosphory	0.940674	#N/A	0.940674
BP_GO:0046604_positive+regulation+of+mitotic+centrosome+separation	0.802328	#N/A	0.802328
BP_GO:0016598_protein+arginylation	0.978186	0.978186	#N/A
MF_GO:0004057_arginyltransferase+activity	0.978186	0.978186	#N/A
MF_GO:0046525_xylosylprotein+4-beta-galactosyltransferase+activity	0.185191	0.185191	#N/A
MF_GO:0003952_NAD++synthase+(glutamine-hydrolyzing)+activity	0.109871	0.109871	#N/A
MF_GO:0008795_NAD++synthase+activity	0.109871	0.109871	#N/A
BP_GO:0006978_DNA+damage+response,+signal+transduction+by+p53+clas	0.935164	#N/A	0.935164
BP_GO:0033205_cytokinesis+during+cell+cycle	0.935164	#N/A	0.935164
MF_GO:0017061_S-methyl-5-thioadenosine+phosphorylase+activity	0.84871	0.84871	#N/A
BP_GO:0030422_RNA+interference,+production+of+siRNA	0.772824	0.772824	#N/A
BP_GO:0032507_maintenance+of+protein+location+in+cell	0.209507	0.209507	#N/A
CC_GO:0000932_cytoplasmic+mRNA+processing+body	0.209507	0.209507	#N/A
BP_GO:0007207_muscarinic+acetylcholine+receptor,+phospholipase+C+acti	0.306546	0.306546	#N/A
MF_GO:0016997_alpha-sialidase+activity	0.536612	#N/A	0.536612
MF_GO:0030274_LIM+domain+binding	0.461422	0.461422	#N/A
MF_GO:0004489_methylenetetrahydrofolate+reductase+(NADPH)+activity	0.0443493	0.0443493	#N/A
BP_GO:0009120_deoxyribonucleoside+metabolic+process	0.580698	#N/A	0.580698
BP_GO:0019047_provirus+integration	0.352772	#N/A	0.352772
MF_GO:0047874_dolichyldiphosphatase+activity	0.648973	0.648973	#N/A
BP_GO:0030220_platelet+formation	0.319706	0.319706	#N/A
BP_GO:0060155_platelet+dense+granule+organization	0.215184	0.215184	#N/A
BP_GO:0018191_peptidyl-serine+octanoylation	0.847255	0.847255	#N/A
MF_GO:0016412_serine+O-acyltransferase+activity	0.847255	0.847255	#N/A
BP_GO:0001754_eye+photoreceptor+cell+differentiation	0.76904	0.76904	#N/A
BP_GO:0002287_alpha-beta+T+cell+activation+during+immune+response	0.170868	0.170868	#N/A
BP_GO:0002476_antigen+processing+and+presentation+of+endogenous+pe	0.170868	0.170868	#N/A
BP_GO:0002477_antigen+processing+and+presentation+of+exogenous+pep	0.170868	0.170868	#N/A
CC_GO:0032398_MHC+class+Ib+protein+complex	0.170868	0.170868	#N/A
BP_GO:0021544_subpallium+development	0.387839	#N/A	0.387839
BP_GO:0014707_branchiomeric+skeletal+muscle+development	0.412333	#N/A	0.412333
BP_GO:0048625_myoblast+cell+fate+commitment	0.412333	#N/A	0.412333
BP_GO:0031943_regulation+of+glucocorticoid+metabolic+process	0.921755	0.921755	#N/A
CC_GO:0031371_ubiquitin+conjugating+enzyme+complex	0.921755	0.921755	#N/A
MF_GO:0030191_Hsp70/Hsc70+protein+inhibitor+activity	0.921755	0.921755	#N/A
MF_GO:0030911_TPR+domain+binding	0.921755	0.921755	#N/A
BP_GO:0018144_RNA-protein+covalent+cross-linking	0.791167	#N/A	0.791167
BP_GO:0042149_cellular+response+to+glucose+starvation	0.791167	#N/A	0.791167
BP_GO:0043153_entrainment+of+circadian+clock+by+photoperiod	0.621935	#N/A	0.621935
MF_GO:0005459_UDP-galactose+transmembrane+transporter+activity	0.836176	#N/A	0.836176
BP_GO:0031583_G-protein+signaling,+phospholipase+D+activating+pathwa	0.58612	#N/A	0.58612
MF_GO:0031707_endothelin+A+receptor+binding	0.58612	#N/A	0.58612
BP_GO:0005513_detection+of+calcium+ion	0.151824	0.151824	#N/A
BP_GO:0032781_positive+regulation+of+ATPase+activity	0.151824	0.151824	#N/A
MF_GO:0008242_omega+peptidase+activity	0.927564	#N/A	0.927564
MF_GO:0034722_gamma-glutamyl-peptidase+activity	0.927564	#N/A	0.927564

BP_GO:0042713_sperm+ejaculation	0.814961	0.814961	#N/A
BP_GO:0043084_penile+erection	0.814961	0.814961	#N/A
BP_GO:0050999_regulation+of+nitric-oxide+synthase+activity	0.814961	0.814961	#N/A
MF_GO:0048186_inhibin+beta-A+binding	0.814961	0.814961	#N/A
BP_GO:0021511_spinal+cord+patterning	0.62515	0.62515	#N/A
BP_GO:0051057_positive+regulation+of+small+GTPase+mediated+signal+tra	0.62515	0.62515	#N/A
BP_GO:0032388_positive+regulation+of+intracellular+transport	0.797927	#N/A	0.797927
MF_GO:0005047_signal+recognition+particle+binding	0.172314	#N/A	0.172314
BP_GO:0015739_sialic+acid+transport	0.297559	0.297559	#N/A
MF_GO:0015136_sialic+acid+transmembrane+transporter+activity	0.297559	0.297559	#N/A
BP_GO:0009056_catabolic+process	0.287344	0.287344	#N/A
BP_GO:0007057_spindle+assembly+involved+in+female+meiosis+I	0.298813	0.298813	#N/A
BP_GO:0046785_microtubule+polymerization	0.298813	0.298813	#N/A
BP_GO:0051442_negative+regulation+of+ubiquitin-protein+ligase+activity+c	0.298813	0.298813	#N/A
BP_GO:0009062_fatty+acid+catabolic+process	0.520642	0.520642	#N/A
MF_GO:0016402_pristanoyl-CoA+oxidase+activity	0.226992	0.226992	#N/A
BP_GO:0021771_lateral+geniculate+nucleus+development	0.592181	#N/A	0.592181
BP_GO:0042320_regulation+of+circadian+sleep/wake+cycle,+REM+sleep	0.592181	#N/A	0.592181
BP_GO:0045188_regulation+of+circadian+sleep/wake+cycle,+non-REM+slee	0.592181	#N/A	0.592181
BP_GO:0045759_negative+regulation+of+action+potential	0.592181	#N/A	0.592181
BP_GO:0006421_asparaginyl-tRNA+aminoacylation	0.748177	#N/A	0.748177
BP_GO:0009589_detection+of+UV	0.0748351	0.0748351	#N/A
MF_GO:0004325_ferrochelatase+activity	0.0748351	0.0748351	#N/A
CC_GO:0000176_nuclear+exosome+(RNase+complex)	0.699921	0.699921	#N/A
BP_GO:0048319_axial+mesoderm+morphogenesis	0.315369	0.315369	#N/A
CC_GO:0019908_nuclear+cyclin-dependent+protein+kinase+holoenzyme+cc	0.899149	#N/A	0.899149
BP_GO:0019310_inositol+catabolic+process	0.396313	0.396313	#N/A
MF_GO:0050113_inositol+oxygenase+activity	0.396313	0.396313	#N/A
BP_GO:0046850_regulation+of+bone+remodeling	0.914857	0.914857	#N/A
BP_GO:0046831_regulation+of+RNA+export+from+nucleus	0.704551	0.704551	#N/A
BP_GO:0015936_coenzyme+A+metabolic+process	0.0749757	0.0749757	#N/A
MF_GO:0004420_hydroxymethylglutaryl-CoA+reductase+(NADPH)+activity	0.0749757	0.0749757	#N/A
BP_GO:0021920_regulation+of+transcription+from+RNA+polymerase+II+prc	0.524607	0.524607	#N/A
MF_GO:0030229_very-low-density+lipoprotein+receptor+activity	0.665647	0.665647	#N/A
MF_GO:0005008_hepatocyte+growth+factor+receptor+activity	0.212155	#N/A	0.212155
MF_GO:0004138_deoxyguanosine+kinase+activity	0.92682	0.92682	#N/A
BP_GO:0000917_barrier+septum+formation	0.724328	0.724328	#N/A
BP_GO:0001762_beta-alanine+transport	0.705822	0.705822	#N/A
BP_GO:0015734_taurine+transport	0.705822	0.705822	#N/A
MF_GO:0001761_beta-alanine+transmembrane+transporter+activity	0.705822	0.705822	#N/A
MF_GO:0005368_taurine+transmembrane+transporter+activity	0.705822	0.705822	#N/A
MF_GO:0005369_taurine:sodium+symporter+activity	0.705822	0.705822	#N/A
BP_GO:0042534_regulation+of+tumor+necrosis+factor+biosynthetic+proces	0.951176	0.951176	#N/A
CC_GO:0031234_extrinsic+to+internal+side+of+plasma+membrane	0.594688	0.594688	#N/A
MF_GO:0005127_ciliary+neurotrophic+factor+receptor+binding	0.534145	0.534145	#N/A
BP_GO:0042745_circadian+sleep/wake+cycle	0.202062	0.202062	#N/A
MF_GO:0004853_uroporphyrinogen+decarboxylase+activity	0.874585	0.874585	#N/A

BP_GO:0032968_positive+regulation+of+RNA+elongation+from+RNA+polym	0.637462	#N/A	0.637462
CC_GO:0045252_oxoglutarate+dehydrogenase+complex	0.365289	#N/A	0.365289
MF_GO:0004149_dihydrolipoyllysine-residue+succinyltransferase+activity	0.365289	#N/A	0.365289
MF_GO:0051435_BH4+domain+binding	0.677233	0.677233	#N/A
BP_GO:0042990_regulation+of+transcription+factor+import+into+nucleus	0.97702	0.97702	#N/A
BP_GO:0048822_enucleate+erythrocyte+development	0.114763	0.114763	#N/A
MF_GO:0004587_ornithine-oxo-acid+transaminase+activity	0.543233	0.543233	#N/A
MF_GO:0005219_ryanodine-sensitive+calcium-release+channel+activity	0.936796	0.936796	#N/A
MF_GO:0050333_thiamin-triphosphatase+activity	0.6063	0.6063	#N/A
CC_GO:0030482_actin+cable	0.678844	0.678844	#N/A
BP_GO:0035026_leading+edge+cell+differentiation	0.0484452	0.0484452	#N/A
BP_GO:0051223_regulation+of+protein+transport	0.513814	#N/A	0.513814
MF_GO:0032794_GTPase+activating+protein+binding	0.513814	#N/A	0.513814
BP_GO:0000018_regulation+of+DNA+recombination	0.372463	0.372463	#N/A
MF_GO:0030629_U6+snRNA+3'-end+binding	0.238782	#N/A	0.238782
MF_GO:0008387_steroid+7-alpha-hydroxylase+activity	0.00243921	0.00243921	#N/A
MF_GO:0033782_24-hydroxycholesterol+7alpha-hydroxylase+activity	0.00243921	0.00243921	#N/A
MF_GO:0004988_mu-opioid+receptor+activity	0.482435	#N/A	0.482435
MF_GO:0016941_natriuretic+peptide+receptor+activity	0.450894	0.450894	#N/A
BP_GO:0016246_RNA+interference	0.969285	0.969285	#N/A
CC_GO:0016442_RNA-induced+silencing+complex	0.969285	0.969285	#N/A
MF_GO:0032556_pyrimidine+deoxyribonucleotide+binding	0.990612	#N/A	0.990612
BP_GO:0018916_nitrobenzene+metabolic+process	0.738264	0.738264	#N/A
BP_GO:0042178_xenobiotic+catabolic+process	0.738264	0.738264	#N/A
BP_GO:0046483_heterocycle+metabolic+process	0.714834	#N/A	0.714834
MF_GO:0004070_aspartate+carbamoyltransferase+activity	0.961357	#N/A	0.961357
MF_GO:0003842_1-pyrroline-5-carboxylate+dehydrogenase+activity	0.819848	0.819848	#N/A
MF_GO:0004418_hydroxymethylbilane+synthase+activity	0.0338878	0.0338878	#N/A
BP_GO:0045768_positive+regulation+of+anti-apoptosis	0.101999	#N/A	0.101999
BP_GO:0046898_response+to+cycloheximide	0.101999	#N/A	0.101999
CC_GO:0044429_mitochondrial+part	0.101999	#N/A	0.101999
MF_GO:0050290_sphingomyelin+phosphodiesterase+D+activity	0.321408	0.321408	#N/A
BP_GO:0001949_sebaceous+gland+cell+differentiation	0.396142	0.396142	#N/A
MF_GO:0043140_ATP-dependent+3'-5'+DNA+helicase+activity	0.55269	#N/A	0.55269
BP_GO:0019725_cellular+homeostasis	0.856728	0.856728	#N/A
BP_GO:0048871_multicellular+organismal+homeostasis	0.856728	0.856728	#N/A
MF_GO:0008330_protein+tyrosine/threonine+phosphatase+activity	0.544921	0.544921	#N/A
MF_GO:0008579_JUN+kinase+phosphatase+activity	0.544921	0.544921	#N/A
MF_GO:0004491_methylmalonate-semialdehyde+dehydrogenase+(acylating	0.315515	0.315515	#N/A
MF_GO:0018478_malonate-semialdehyde+dehydrogenase+(acetylating)+ac	0.315515	0.315515	#N/A
MF_GO:0017049_GTP-Rho+binding	0.76107	#N/A	0.76107
BP_GO:0006933_negative+regulation+of+cell+adhesion+involved+in+substra	0.567724	0.567724	#N/A
BP_GO:0051649_establishment+of+localization+in+cell	0.023924	#N/A	0.023924
CC_GO:0031226_intrinsic+to+plasma+membrane	0.023924	#N/A	0.023924
MF_GO:0043495_protein+anchor	0.023924	#N/A	0.023924
MF_GO:0015559_multidrug+efflux+pump+activity	0.464927	#N/A	0.464927
BP_GO:0006383_transcription+from+RNA+polymerase+III+promoter	0.442812	0.442812	#N/A

CC_GO:0005969_serine-pyruvate+aminotransferase+complex	0.340161	0.340161	#N/A
BP_GO:0032066_nucleolus+to+nucleoplasm+transport	0.191954	0.191954	#N/A
BP_GO:0019102_male+somatic+sex+determination	0.247322	#N/A	0.247322
MF_GO:0004882_androgen+receptor+activity	0.247322	#N/A	0.247322
BP_GO:0006682_galactosylceramide+biosynthetic+process	0.283936	#N/A	0.283936
BP_GO:0032275_luteinizing+hormone+secretion	0.683935	#N/A	0.683935
BP_GO:0032870_cellular+response+to+hormone+stimulus	0.683935	#N/A	0.683935
BP_GO:0046884_follicle-stimulating+hormone+secretion	0.683935	#N/A	0.683935
MF_GO:0030562_rRNA+2'-O-ribose+methylation+guide+activity	0.742832	#N/A	0.742832
MF_GO:0003860_3-hydroxyisobutyryl-CoA+hydrolase+activity	0.19999	0.19999	#N/A
BP_GO:0045927_positive+regulation+of+growth	0.723489	0.723489	#N/A
MF_GO:0016565_general+transcriptional+repressor+activity	0.723489	0.723489	#N/A
BP_GO:0006231_dTMP+biosynthetic+process	0.769397	#N/A	0.769397
MF_GO:0004799_thymidylate+synthase+activity	0.769397	#N/A	0.769397
BP_GO:0042789_mRNA+transcription+from+RNA+polymerase+II+promoter	0.601358	#N/A	0.601358
BP_GO:0006496_protein+amino+acid+terminal+N-glycosylation	0.368778	#N/A	0.368778
BP_GO:0051270_regulation+of+cell+motion	0.368778	#N/A	0.368778
BP_GO:0060046_regulation+of+acrosome+reaction	0.368778	#N/A	0.368778
BP_GO:0060054_positive+regulation+of+epithelial+cell+proliferation+involv	0.368778	#N/A	0.368778
MF_GO:0016433_rRNA+(adenine)+methyltransferase+activity	0.965613	0.965613	#N/A
BP_GO:0042273_ribosomal+large+subunit+biogenesis	0.590363	0.590363	#N/A
MF_GO:0047395_glycerophosphoinositol+glycerophosphodiesterase+activit	0.490045	#N/A	0.490045
MF_GO:0047115_trans-1,2-dihydrobenzene-1,2-diol+dehydrogenase+activit	0.478247	0.478247	#N/A
MF_GO:0047837_D-xylose+1-dehydrogenase+(NADP+)+activity	0.478247	0.478247	#N/A
MF_GO:0016519_gastric+inhibitory+peptide+receptor+activity	0.686401	#N/A	0.686401
BP_GO:0000105_histidine+biosynthetic+process	0.828101	0.828101	#N/A
CC_GO:0005847_mRNA+cleavage+and+polyadenylation+specificity+factor+c	0.882633	0.882633	#N/A
BP_GO:0006669_sphinganine-1-phosphate+biosynthetic+process	0.889814	#N/A	0.889814
MF_GO:0017050_D-erythro-sphingosine+kinase+activity	0.889814	#N/A	0.889814
BP_GO:0017085_response+to+insecticide	0.914018	0.914018	#N/A
BP_GO:0050684_regulation+of+mRNA+processing	0.417783	#N/A	0.417783
BP_GO:0006450_regulation+of+translational+fidelity	0.488643	#N/A	0.488643
BP_GO:0002071_glandular+epithelial+cell+maturation	0.127158	#N/A	0.127158
MF_GO:0008780_acyl-[acyl-carrier-protein]-UDP-N-acetylglucosamine+O-ac	0.127158	#N/A	0.127158
BP_GO:0007500_mesodermal+cell+fate+determination	0.363398	#N/A	0.363398
CC_GO:0005623_cell	0.895273	0.895273	#N/A
BP_GO:0019543_propionate+catabolic+process	0.0444058	0.0444058	#N/A
MF_GO:0047547_2-methylcitrate+dehydratase+activity	0.0444058	0.0444058	#N/A
BP_GO:0048219_inter-Golgi+cisterna+vesicle-mediated+transport	0.218171	0.218171	#N/A
BP_GO:0034504_protein+localization+in+nucleus	0.779592	#N/A	0.779592
BP_GO:0019695_choline+metabolic+process	0.561175	0.561175	#N/A
BP_GO:0017187_peptidyl-glutamic+acid+carboxylation	0.735407	#N/A	0.735407
MF_GO:0008488_gamma-glutamyl+carboxylase+activity	0.735407	#N/A	0.735407
BP_GO:0002017_regulation+of+blood+volume+by+renal+aldosterone	0.959302	#N/A	0.959302
BP_GO:0032342_aldosterone+biosynthetic+process	0.959302	#N/A	0.959302
MF_GO:0004507_steroid+11-beta-monooxygenase+activity	0.959302	#N/A	0.959302
CC_GO:0030122_AP-2+adaptor+complex	0.310297	#N/A	0.310297

BP_GO:0009236_cobalamin+biosynthetic+process	0.259576	0.259576	#N/A
MF_GO:0008817_cob(I)yrinic+acid+a,c-diamide+adenosyltransferase+activit	0.259576	0.259576	#N/A
MF_GO:0005129_granulocyte+macrophage+colony-stimulating+factor+rece	0.321858	#N/A	0.321858
BP_GO:0046471_phosphatidylglycerol+metabolic+process	0.131727	0.131727	#N/A
MF_GO:0017171_serine+hydrolase+activity	0.248054	#N/A	0.248054
BP_GO:0035090_maintenance+of+apical/basal+cell+polarity	0.885447	0.885447	#N/A
BP_GO:0009794_regulation+of+mitotic+cell+cycle,+embryonic	0.193002	0.193002	#N/A
MF_GO:0017080_sodium+channel+regulator+activity	0.821642	#N/A	0.821642
MF_GO:0004438_phosphatidylinositol-3-phosphatase+activity	0.71702	0.71702	#N/A
BP_GO:0034505_tooth+mineralization	0.445506	#N/A	0.445506
MF_GO:0043498_cell+surface+binding	0.445506	#N/A	0.445506
MF_GO:0046848_hydroxyapatite+binding	0.445506	#N/A	0.445506
BP_GO:0009113_purine+base+biosynthetic+process	0.769668	#N/A	0.769668
MF_GO:0004641_phosphoribosylformylglycinamidine+cyclo-ligase+activity	0.769668	#N/A	0.769668
MF_GO:0004644_phosphoribosylglycinamide+formyltransferase+activity	0.769668	#N/A	0.769668
BP_GO:0031946_regulation+of+glucocorticoid+biosynthetic+process	0.776367	0.776367	#N/A
BP_GO:0042921_glucocorticoid+receptor+signaling+pathway	0.776367	0.776367	#N/A
MF_GO:0004883_glucocorticoid+receptor+activity	0.776367	0.776367	#N/A
BP_GO:0019100_male+germ-line+sex+determination	0.0675511	#N/A	0.0675511
BP_GO:0060350_endochondral+bone+morphogenesis	0.0675511	#N/A	0.0675511
CC_GO:0042564_NLS-dependent+protein+nuclear+import+complex	0.86114	0.86114	#N/A
BP_GO:0032206_positive+regulation+of+telomere+maintenance	0.490354	0.490354	#N/A
BP_GO:0002121_inter-male+aggressive+behavior	0.517785	#N/A	0.517785
BP_GO:0002124_territorial+aggressive+behavior	0.517785	#N/A	0.517785
BP_GO:0008050_female+courtship+behavior	0.517785	#N/A	0.517785
BP_GO:0048047_mating+behavior,+sex+discrimination	0.517785	#N/A	0.517785
CC_GO:0032590_dendrite+membrane	0.517785	#N/A	0.517785
BP_GO:0009257_10-formyltetrahydrofolate+biosynthetic+process	0.592883	0.592883	#N/A
BP_GO:0030948_negative+regulation+of+vascular+endothelial+growth+fact	0.608788	0.608788	#N/A
MF_GO:0004798_thymidylate+kinase+activity	0.64567	0.64567	#N/A
BP_GO:0030320_cellular+monovalent+inorganic+anion+homeostasis	0.180909	#N/A	0.180909
BP_GO:0051044_positive+regulation+of+membrane+protein+ectodomain+p	0.611997	#N/A	0.611997
MF_GO:0003975_UDP-N-acetylglucosamine-dolichyl-phosphate+N-acetylglu	0.21132	#N/A	0.21132
MF_GO:0008963_phospho-N-acetylmuramoyl-pentapeptide-transferase+act	0.21132	#N/A	0.21132
BP_GO:0030035_microspike+assembly	0.0811855	0.0811855	#N/A
BP_GO:0045623_negative+regulation+of+T-helper+cell+differentiation	0.778369	0.778369	#N/A
BP_GO:0051896_regulation+of+protein+kinase+B+signaling+cascade	0.559105	0.559105	#N/A
BP_GO:0031664_regulation+of+lipopolysaccharide-mediated+signaling+path	0.500031	0.500031	#N/A
BP_GO:0001941_postsynaptic+membrane+organization	0.479492	#N/A	0.479492
MF_GO:0004148_dihydrolipoyl+dehydrogenase+activity	0.897568	0.897568	#N/A
BP_GO:0006348_chromatin+silencing+at+telomere	0.914107	#N/A	0.914107
BP_GO:0042073_intraflagellar+transport	0.515743	#N/A	0.515743
MF_GO:0008808_cardiolipin+synthase+activity	0.515743	#N/A	0.515743
MF_GO:0005412_glucose:sodium+symporter+activity	0.360402	#N/A	0.360402
BP_GO:0046822_regulation+of+nucleocytoplasmic+transport	0.373343	#N/A	0.373343
BP_GO:0051444_negative+regulation+of+ubiquitin-protein+ligase+activity	0.373343	#N/A	0.373343
MF_GO:0055105_ubiquitin-protein+ligase+inhibitor+activity	0.373343	#N/A	0.373343

MF_GO:0015019_heparan-alpha-glucosaminide+N-acetyltransferase+activit	0.946801	#N/A	0.946801
MF_GO:0043425_bHLH+transcription+factor+binding	0.890703	#N/A	0.890703
CC_GO:0001650_fibrillar+center	0.221066	#N/A	0.221066
MF_GO:0015051_X-opioid+receptor+activity	0.759032	0.759032	#N/A
BP_GO:0001555_oocyte+growth	0.0215832	#N/A	0.0215832
BP_GO:0032212_positive+regulation+of+telomere+maintenance+via+telom	0.536023	0.536023	#N/A
CC_GO:0005697_telomerase+holoenzyme+complex	0.536023	0.536023	#N/A
MF_GO:0000332_template+for+synthesis+of+G-rich+strand+of+telomere+D	0.536023	0.536023	#N/A
BP_GO:0048845_venous+blood+vessel+morphogenesis	0.832392	0.832392	#N/A
BP_GO:0000379_tRNA-type+intron+splice+site+recognition+and+cleavage	0.240215	0.240215	#N/A
BP_GO:0014813_satellite+cell+commitment	0.140096	#N/A	0.140096
BP_GO:0007197_muscarinic+acetylcholine+receptor,+adenylate+cyclase+inl	0.822404	0.822404	#N/A
BP_GO:0048627_myoblast+development	0.784894	0.784894	#N/A
MF_GO:0030942_endoplasmic+reticulum+signal+peptide+binding	0.997148	#N/A	0.997148
BP_GO:0008593_regulation+of+Notch+signaling+pathway	0.647986	0.647986	#N/A
BP_GO:0007158_neuron+adhesion	0.252296	#N/A	0.252296
CC_GO:0045180_basal+cortex	0.929027	#N/A	0.929027
BP_GO:0032808_lacrimal+gland+development	0.645983	0.645983	#N/A
MF_GO:0032810_sterol+response+element+binding	0.380541	0.380541	#N/A
MF_GO:0008418_protein+N-terminal+asparagine+amidohydrolase+activity	0.409768	0.409768	#N/A
MF_GO:0015054_gastrin+receptor+activity	0.315853	0.315853	#N/A
MF_GO:0005142_interleukin-11+receptor+binding	0.585033	0.585033	#N/A
MF_GO:0004951_cholecystokinin+receptor+activity	0.466841	0.466841	#N/A
CC_GO:0002111_BRCA2-BRAF35+complex	0.554232	0.554232	#N/A
MF_GO:0000400_four-way+junction+DNA+binding	0.554232	0.554232	#N/A
BP_GO:0046619_optic+placode+formation+involved+in+camera-type+eye	0.870495	0.870495	#N/A
BP_GO:0003097_renal+water+transport	0.728714	#N/A	0.728714
BP_GO:0042631_cellular+response+to+water+deprivation	0.728714	#N/A	0.728714
CC_GO:0031303_integral+to+endosome+membrane	0.728714	#N/A	0.728714
CC_GO:0055038_recycling+endosome+membrane	0.728714	#N/A	0.728714
MF_GO:0033871_[heparan+sulfate]-glucosamine+3-sulfotransferase+2+acti	0.666772	#N/A	0.666772
MF_GO:0019135_deoxyhypusine+monooxygenase+activity	0.668739	0.668739	#N/A
BP_GO:0002903_negative+regulation+of+B+cell+apoptosis	0.684357	0.684357	#N/A
BP_GO:0051935_glutamate+uptake+during+transmission+of+nerve+impulse	0.835909	0.835909	#N/A
BP_GO:0006798_polyphosphate+catabolic+process	0.976518	0.976518	#N/A
MF_GO:0015926_glucosidase+activity	0.900029	0.900029	#N/A
MF_GO:0033919_glucan+1,3-alpha-glucosidase+activity	0.900029	0.900029	#N/A
MF_GO:0005087_Ran+guanyl-nucleotide+exchange+factor+activity	0.482028	#N/A	0.482028
MF_GO:0004819_glutamine-tRNA+ligase+activity	0.974732	0.974732	#N/A
BP_GO:0015855_pyrimidine+transport	0.434172	0.434172	#N/A
MF_GO:0005350_pyrimidine+transmembrane+transporter+activity	0.434172	0.434172	#N/A
MF_GO:0016455_RNA+polymerase+II+transcription+mediator+activity	0.296511	#N/A	0.296511
BP_GO:0000291_nuclear-transcribed+mRNA+catabolic+process,+exonucleol	0.31372	0.31372	#N/A
BP_GO:0050773_regulation+of+dendrite+development	0.235599	0.235599	#N/A
MF_GO:0030116_glial+cell+line-derived+neurotrophic+factor+receptor+binc	0.262619	#N/A	0.262619
BP_GO:0043654_recognition+of+apoptotic+cell	0.238139	#N/A	0.238139
MF_GO:0043014_alpha-tubulin+binding	0.556443	#N/A	0.556443

BP_GO:0007024_alpha-tubulin+folding	0.553824	0.553824	#N/A
BP_GO:0014889_muscle+atrophy	0.553824	0.553824	#N/A
BP_GO:0048936_peripheral+nervous+system+neuron+axonogenesis	0.553824	0.553824	#N/A
BP_GO:0048137_spermatocyte+division	0.531277	#N/A	0.531277
BP_GO:0045046_protein+import+into+peroxisome+membrane	0.444289	#N/A	0.444289
MF_GO:0004530_deoxyribonuclease+I+activity	0.690574	#N/A	0.690574
BP_GO:0009628_response+to+abiotic+stimulus	0.574003	#N/A	0.574003
MF_GO:0047290_(alpha-N-acetylneuraminyl-2,3-beta-galactosyl-1,3)-N-acet	0.568961	0.568961	#N/A
MF_GO:0047677_arachidonate+8-lipoxygenase+activity	0.493161	#N/A	0.493161
MF_GO:0050473_arachidonate+15-lipoxygenase+activity	0.493161	#N/A	0.493161
BP_GO:0018343_protein+farnesylation	0.189291	#N/A	0.189291
BP_GO:0030823_regulation+of+cGMP+metabolic+process	0.189291	#N/A	0.189291
MF_GO:0001918_farnesylated+protein+binding	0.189291	#N/A	0.189291
BP_GO:0048246_macrophage+chemotaxis	0.303998	#N/A	0.303998
MF_GO:0031727_CCR2+chemokine+receptor+binding	0.303998	#N/A	0.303998
BP_GO:0042706_eye+photoreceptor+cell+fate+commitment	0.661785	#N/A	0.661785
MF_GO:0004852_uroporphyrinogen-III+synthase+activity	0.480237	0.480237	#N/A
MF_GO:0004728_receptor+signaling+protein+tyrosine+phosphatase+activity	0.685249	0.685249	#N/A
BP_GO:0006463_steroid+hormone+receptor+complex+assembly	0.840347	0.840347	#N/A
BP_GO:0046661_male+sex+differentiation	0.840347	0.840347	#N/A
MF_GO:0035259_glucocorticoid+receptor+binding	0.840347	0.840347	#N/A
BP_GO:0006551_leucine+metabolic+process	0.674248	0.674248	#N/A
BP_GO:0035148_lumen+formation	0.824544	0.824544	#N/A
MF_GO:0015106_bicarbonate+transmembrane+transporter+activity	0.589845	0.589845	#N/A
MF_GO:0015301_anion:anion+antiporter+activity	0.589845	0.589845	#N/A
MF_GO:0008139_nuclear+localization+sequence+binding	0.8593	0.8593	#N/A
MF_GO:0004980_melanocyte+stimulating+hormone+receptor+activity	0.437611	#N/A	0.437611
BP_GO:0043316_cytotoxic+T+cell+degranulation	0.631859	#N/A	0.631859
MF_GO:0000156_two-component+response+regulator+activity	0.423193	0.423193	#N/A
BP_GO:0009996_negative+regulation+of+cell+fate+specification	0.52413	0.52413	#N/A
BP_GO:0042660_positive+regulation+of+cell+fate+specification	0.52413	0.52413	#N/A
BP_GO:0021873_forebrain+neuroblast+division	0.84852	#N/A	0.84852
BP_GO:0045769_negative+regulation+of+asymmetric+cell+division	0.84852	#N/A	0.84852
BP_GO:0051661_maintenance+of+centrosome+location	0.84852	#N/A	0.84852
MF_GO:0016501_prostacyclin+receptor+activity	0.943985	#N/A	0.943985
MF_GO:0016709_oxidoreductase+activity,+acting+on+paired+donors,+with-	0.0225884	0.0225884	#N/A
BP_GO:0045780_positive+regulation+of+bone+resorption	0.962808	0.962808	#N/A
CC_GO:0030532_small+nuclear+ribonucleoprotein+complex	0.482697	0.482697	#N/A
BP_GO:0032525_somite+rostral/caudal+axis+specification	0.20189	#N/A	0.20189
BP_GO:0006576_biogenic+amine+metabolic+process	0.824242	0.824242	#N/A
BP_GO:0009405_pathogenesis	0.824242	0.824242	#N/A
BP_GO:0031061_negative+regulation+of+histone+methylation	0.824242	0.824242	#N/A
BP_GO:0032048_cardiolipin+metabolic+process	0.824242	0.824242	#N/A
BP_GO:0040029_regulation+of+gene+expression,+epigenetic	0.824242	0.824242	#N/A
MF_GO:0010385_double-stranded+methylated+DNA+binding	0.824242	0.824242	#N/A
BP_GO:0009214_cyclic+nucleotide+catabolic+process	0.198615	0.198615	#N/A
MF_GO:0004113_2',3'-cyclic-nucleotide+3'-phosphodiesterase+activity	0.198615	0.198615	#N/A

CC_GO:0045259_proton-transporting+ATP+synthase+complex	0.8339	0.8339	#N/A
CC_GO:0030868_smooth+endoplasmic+reticulum+membrane	0.122819	0.122819	#N/A
MF_GO:0005042_netrin+receptor+activity	0.289438	0.289438	#N/A
BP_GO:0051584_regulation+of+dopamine+uptake	0.925754	0.925754	#N/A
BP_GO:0051586_positive+regulation+of+dopamine+uptake	0.925754	0.925754	#N/A
MF_GO:0001591_dopamine+receptor+coupled+via+Gi/Go	0.925754	0.925754	#N/A
MF_GO:0001670_dopamine+D2+receptor+activity	0.925754	0.925754	#N/A
BP_GO:0006278_RNA-dependent+DNA+replication	0.613461	0.613461	#N/A
MF_GO:0003721_telomeric+template+RNA+reverse+transcriptase+activity	0.613461	0.613461	#N/A
BP_GO:0016340_calcium-dependent+cell-matrix+adhesion	0.903356	0.903356	#N/A
BP_GO:0008216_spermidine+metabolic+process	0.0894403	#N/A	0.0894403
BP_GO:0042628_mating+plug+formation	0.861753	0.861753	#N/A
BP_GO:0043480_pigment+accumulation+in+tissues	0.418697	#N/A	0.418697
BP_GO:0045113_regulation+of+integrin+biosynthetic+process	0.179283	#N/A	0.179283
CC_GO:0005674_transcription+factor+TFIIF+complex	0.847895	#N/A	0.847895
BP_GO:0047497_mitochondrion+transport+along+microtubule	0.937827	0.937827	#N/A
MF_GO:0019784_NEDD8-specific+protease+activity	0.271821	0.271821	#N/A
MF_GO:0017162_aryl+hydrocarbon+receptor+binding	0.260896	#N/A	0.260896
BP_GO:0016239_positive+regulation+of+macroautophagy	0.563277	0.563277	#N/A
MF_GO:0032403_protein+complex+binding	0.434695	#N/A	0.434695
BP_GO:0060349_bone+morphogenesis	0.489957	#N/A	0.489957
BP_GO:0006873_cellular+ion+homeostasis	0.340342	#N/A	0.340342
BP_GO:0030007_cellular+potassium+ion+homeostasis	0.340342	#N/A	0.340342
BP_GO:0006307_DNA+dealkylation	0.806378	#N/A	0.806378
MF_GO:0003908_methylated-DNA-[protein]-cysteine+S-methyltransferase+	0.806378	#N/A	0.806378
BP_GO:0016264_gap+junction+assembly	0.887133	#N/A	0.887133
BP_GO:0007400_neuroblast+fate+determination	0.0917164	#N/A	0.0917164
BP_GO:0021779_oligodendrocyte+cell+fate+commitment	0.0917164	#N/A	0.0917164
BP_GO:0060165_regulation+of+timing+of+subpallium+neuron+differentiatic	0.0917164	#N/A	0.0917164
MF_GO:0004484_mRNA+guanylyltransferase+activity	0.936239	0.936239	#N/A
MF_GO:0004651_polynucleotide+5'-phosphatase+activity	0.936239	0.936239	#N/A
MF_GO:0003844_1,4-alpha-glucan+branching+enzyme+activity	0.916798	0.916798	#N/A
BP_GO:0007495_visceral+mesoderm-endoderm+interaction+involved+in+m	0.953041	#N/A	0.953041
BP_GO:0021794_thalamus+development	0.225513	#N/A	0.225513
BP_GO:0009992_cellular+water+homeostasis	0.824773	0.824773	#N/A
BP_GO:0019858_cytosine+metabolic+process	0.307353	0.307353	#N/A
MF_GO:0016908_MAP+kinase+2+activity	0.307353	0.307353	#N/A
BP_GO:0000185_activation+of+MAPKKK+activity	0.800637	#N/A	0.800637
BP_GO:0070207_protein+homotrimerization	0.398776	#N/A	0.398776
MF_GO:0004081_bis(5'-nucleosyl)-tetraphosphatase+(asymmetrical)+activit	0.246046	0.246046	#N/A
MF_GO:0008796_bis(5'-nucleosyl)-tetraphosphatase+activity	0.246046	0.246046	#N/A
BP_GO:0010667_negative+regulation+of+cardiac+muscle+cell+apoptosis	0.417684	0.417684	#N/A
BP_GO:0010735_positive+regulation+of+transcription+via+serum+response	0.417684	0.417684	#N/A
BP_GO:0051929_positive+regulation+of+calcium+ion+transport+via+voltage	0.417684	0.417684	#N/A
BP_GO:0055117_regulation+of+cardiac+muscle+contraction	0.417684	0.417684	#N/A
BP_GO:0060261_positive+regulation+of+transcription+initiation+from+RNA	0.417684	0.417684	#N/A
MF_GO:0010736_serum+response+element+binding	0.417684	0.417684	#N/A

BP_GO:0046878_positive+regulation+of+saliva+secretion	0.488722	#N/A	0.488722
BP_GO:0043568_positive+regulation+of+insulin-like+growth+factor+recepto	0.929759	0.929759	#N/A
BP_GO:0021692_cerebellar+Purkinje+cell+layer+morphogenesis	0.269558	0.269558	#N/A
BP_GO:0045299_otolith+mineralization	0.269558	0.269558	#N/A
BP_GO:0051928_positive+regulation+of+calcium+ion+transport	0.269558	0.269558	#N/A
MF_GO:0015085_calcium+ion+transmembrane+transporter+activity	0.269558	0.269558	#N/A
MF_GO:0030899_calcium-dependent+ATPase+activity	0.269558	0.269558	#N/A
CC_GO:0016342_catenin+complex	0.954318	0.954318	#N/A
BP_GO:0002335_mature+B+cell+differentiation	0.504075	#N/A	0.504075
BP_GO:0002380_immunoglobulin+secretion+during+immune+response	0.504075	#N/A	0.504075
BP_GO:0060122_inner+ear+receptor+stereocilium+organization	0.653385	0.653385	#N/A
MF_GO:0034617_tetrahydrobiopterin+binding	0.255151	0.255151	#N/A
MF_GO:0034618_arginine+binding	0.255151	0.255151	#N/A
CC_GO:0005683_snRNP+U7	0.78352	#N/A	0.78352
MF_GO:0005471_ATP:ADP+antiporter+activity	0.616967	#N/A	0.616967
BP_GO:0045065_cytotoxic+T+cell+differentiation	0.78233	0.78233	#N/A
MF_GO:0001593_dopamine+D4+receptor+activity	0.280224	#N/A	0.280224
BP_GO:0002355_detection+of+tumor+cell	0.140695	0.140695	#N/A
BP_GO:0002860_positive+regulation+of+natural+killer+cell+mediated+cytot	0.140695	0.140695	#N/A
BP_GO:0002098_tRNA+wobble+uridine+modification	0.883501	0.883501	#N/A
MF_GO:0004730_pseudouridylate+synthase+activity	0.429239	#N/A	0.429239
BP_GO:0060161_positive+regulation+of+dopamine+receptor+signaling+path	0.641531	#N/A	0.641531
MF_GO:0001592_dopamine+D3+receptor+activity	0.641531	#N/A	0.641531
CC_GO:0032444_activin+responsive+factor+complex	0.195381	#N/A	0.195381
BP_GO:0043587_tongue+morphogenesis	0.896447	0.896447	#N/A
BP_GO:0042752_regulation+of+circadian+rhythm	0.490018	0.490018	#N/A
BP_GO:0051151_negative+regulation+of+smooth+muscle+cell+differentiatio	0.984572	0.984572	#N/A
MF_GO:0003881_CDP-diacylglycerol-inositol+3-phosphatidyltransferase+act	0.517294	0.517294	#N/A
CC_GO:0033268_node+of+Ranvier	0.0199048	0.0199048	#N/A
CC_GO:0043194_initial+segment	0.0199048	0.0199048	#N/A
BP_GO:0001988_positive+regulation+of+heart+rate+in+baroreceptor+respo	0.803921	#N/A	0.803921
BP_GO:0014061_regulation+of+norepinephrine+secretion	0.803921	#N/A	0.803921
BP_GO:0032691_negative+regulation+of+interleukin-1+beta+production	0.803921	#N/A	0.803921
BP_GO:0060112_generation+of+ovulation+cycle+rhythm	0.803921	#N/A	0.803921
CC_GO:0043203_axon+hillock	0.638198	0.638198	#N/A
BP_GO:0030299_cholesterol+absorption	0.0454619	#N/A	0.0454619
BP_GO:0051977_lysophospholipid+transport	0.528568	#N/A	0.528568
BP_GO:0042059_negative+regulation+of+epidermal+growth+factor+recepto	0.258044	0.258044	#N/A
MF_GO:0051425_PTB+domain+binding	0.258044	0.258044	#N/A
BP_GO:0007621_negative+regulation+of+female+receptivity	0.765841	#N/A	0.765841
BP_GO:0030157_pancreatic+juice+secretion	0.840973	0.840973	#N/A
MF_GO:0004380_glycoprotein-fucosylgalactoside+alpha-N-acetylgalactosam	0.98638	0.98638	#N/A
MF_GO:0004381_fucosylgalactoside+3-alpha-galactosyltransferase+activity	0.98638	0.98638	#N/A
CC_GO:0005861_troponin+complex	0.329201	0.329201	#N/A
MF_GO:0048256_flap+endonuclease+activity	0.341694	0.341694	#N/A
MF_GO:0050178_phenylpyruvate+tautomerase+activity	0.813857	#N/A	0.813857
BP_GO:0006473_protein+amino+acid+acetylation	0.156497	0.156497	#N/A

MF_GO:0005221_intracellular+cyclic+nucleotide+activated+cation+channel+	0.291241	0.291241	#N/A
BP_GO:0042271_susceptibility+to+natural+killer+cell+mediated+cytotoxicity	0.878273	0.878273	#N/A
BP_GO:0045058_T+cell+selection	0.240334	#N/A	0.240334
MF_GO:0008437_thyrotropin-releasing+hormone+activity	0.909658	#N/A	0.909658
MF_GO:0004498_calcidiol+1-monooxygenase+activity	0.611434	#N/A	0.611434
BP_GO:0010455_positive+regulation+of+cell+fate+commitment	0.396108	0.396108	#N/A
BP_GO:0045187_regulation+of+circadian+sleep/wake+cycle,+sleep	0.784991	#N/A	0.784991
BP_GO:0048387_negative+regulation+of+retinoic+acid+receptor+signaling+	0.997104	0.997104	#N/A
BP_GO:0008057_eye+pigment+granule+organization	0.180233	0.180233	#N/A
BP_GO:0030835_negative+regulation+of+actin+filament+depolymerization	0.180233	0.180233	#N/A
BP_GO:0032401_establishment+of+melanosome+localization	0.180233	0.180233	#N/A
BP_GO:0043482_cellular+pigment+accumulation	0.180233	0.180233	#N/A
BP_GO:0042664_negative+regulation+of+endodermal+cell+fate+specificatic	0.379462	#N/A	0.379462
BP_GO:0031536_positive+regulation+of+exit+from+mitosis	0.313832	#N/A	0.313832
BP_GO:0015811_L-cystine+transport	0.731407	0.731407	#N/A
MF_GO:0015184_L-cystine+transmembrane+transporter+activity	0.731407	0.731407	#N/A
BP_GO:0001510_RNA+methylation	0.293253	0.293253	#N/A
MF_GO:0001734_mRNA+(N6-adenosine)-methyltransferase+activity	0.293253	0.293253	#N/A
MF_GO:0016422_mRNA+(2'-O-methyladenosine-N6-)-methyltransferase+ac	0.293253	0.293253	#N/A
BP_GO:0030210_heparin+biosynthetic+process	0.988268	#N/A	0.988268
MF_GO:0050379_UDP-glucuronate+5'-epimerase+activity	0.988268	#N/A	0.988268
MF_GO:0008403_25-hydroxycholecalciferol-24-hydroxylase+activity	0.793093	#N/A	0.793093
MF_GO:0045545_syndecan+binding	0.238852	0.238852	#N/A
BP_GO:0050907_detection+of+chemical+stimulus+involved+in+sensory+per	0.907848	0.907848	#N/A
BP_GO:0050961_detection+of+temperature+stimulus+involved+in+sensory+	0.907848	0.907848	#N/A
BP_GO:0015747_urate+transport	0.450512	#N/A	0.450512
MF_GO:0015143_urate+transmembrane+transporter+activity	0.450512	#N/A	0.450512
BP_GO:0002023_reduction+of+food+intake+in+response+to+dietary+excess	0.350245	#N/A	0.350245
BP_GO:0008633_activation+of+pro-apoptotic+gene+products	0.0548139	#N/A	0.0548139
BP_GO:0043279_response+to+alkaloid	0.0548139	#N/A	0.0548139
MF_GO:0008768_UDP-sugar+diphosphatase+activity	0.600366	#N/A	0.600366
BP_GO:0030223_neutrophil+differentiation	0.788385	0.788385	#N/A
BP_GO:0032790_ribosome+disassembly	0.245429	#N/A	0.245429
MF_GO:0043024_ribosomal+small+subunit+binding	0.245429	#N/A	0.245429
BP_GO:0060272_embryonic+skeletal+joint+morphogenesis	0.395974	#N/A	0.395974
BP_GO:0060348_bone+development	0.233947	#N/A	0.233947
CC_GO:0005585_collagen+type+II	0.233947	#N/A	0.233947
BP_GO:0045407_positive+regulation+of+interleukin-5+biosynthetic+process	0.24184	#N/A	0.24184
BP_GO:0042506_tyrosine+phosphorylation+of+Stat5+protein	0.818245	0.818245	#N/A
BP_GO:0042508_tyrosine+phosphorylation+of+Stat1+protein	0.818245	0.818245	#N/A
BP_GO:0045875_negative+regulation+of+sister+chromatid+cohesion	0.999212	0.999212	#N/A
BP_GO:0015898_amiloride+transport	0.836247	0.836247	#N/A
BP_GO:0042518_negative+regulation+of+tyrosine+phosphorylation+of+Stat	0.498263	#N/A	0.498263
BP_GO:0046655_folic+acid+metabolic+process	0.306968	#N/A	0.306968
BP_GO:0007228_positive+regulation+of+hh+target+transcription+factor+act	0.816212	0.816212	#N/A
BP_GO:0046639_negative+regulation+of+alpha-beta+T+cell+differentiation	0.816212	0.816212	#N/A
BP_GO:0060020_Bergmann+glial+cell+differentiation	0.816212	0.816212	#N/A

MF_GO:0043237_laminin-1+binding	0.816212	0.816212	#N/A
MF_GO:0003977_UDP-N-acetylglucosamine+diphosphorylase+activity	0.669762	#N/A	0.669762
CC_GO:0005760_gamma+DNA+polymerase+complex	0.275215	0.275215	#N/A
BP_GO:0010039_response+to+iron+ion	0.77091	#N/A	0.77091
CC_GO:0005960_glycine+cleavage+complex	0.4829	#N/A	0.4829
BP_GO:0046834_lipid+phosphorylation	0.144287	0.144287	#N/A
MF_GO:0001727_lipid+kinase+activity	0.144287	0.144287	#N/A
MF_GO:0047620_acylglycerol+kinase+activity	0.144287	0.144287	#N/A
BP_GO:0045184_establishment+of+protein+localization	0.606691	0.606691	#N/A
BP_GO:0008355_olfactory+learning	0.448116	#N/A	0.448116
BP_GO:0010646_regulation+of+cell+communication	0.448116	#N/A	0.448116
BP_GO:0021586_pons+maturation	0.448116	#N/A	0.448116
BP_GO:0060179_male+mating+behavior	0.448116	#N/A	0.448116
MF_GO:0008176_tRNA+(guanine-N7-)-methyltransferase+activity	0.768252	#N/A	0.768252
BP_GO:0035036_sperm-egg+recognition	0.499392	#N/A	0.499392
MF_GO:0030273_melanin-concentrating+hormone+receptor+activity	0.770281	#N/A	0.770281
BP_GO:0015864_pyrimidine+nucleoside+transport	0.911014	0.911014	#N/A
MF_GO:0015389_pyrimidine-+and+adenine-specific:sodium+symporter+acti	0.911014	0.911014	#N/A
MF_GO:0015390_purine-specific+nucleoside:sodium+symporter+activity	0.911014	0.911014	#N/A
BP_GO:0051102_DNA+ligation+during+DNA+recombination	0.448026	#N/A	0.448026
BP_GO:0051103_DNA+ligation+during+DNA+repair	0.448026	#N/A	0.448026
CC_GO:0060077_inhibitory+synapse	0.934485	0.934485	#N/A
MF_GO:0015495_gamma-aminobutyric+acid:hydrogen+symporter+activity	0.934485	0.934485	#N/A
CC_GO:0000153_cytoplasmic+ubiquitin+ligase+complex	0.170792	#N/A	0.170792
BP_GO:0043248_proteasome+assembly	0.23128	#N/A	0.23128
BP_GO:0007065_male+meiosis+sister+chromatid+cohesion	0.237796	#N/A	0.237796
BP_GO:0034230_enkephalin+processing	0.627138	#N/A	0.627138
BP_GO:0045725_positive+regulation+of+glycogen+biosynthetic+process	0.160906	#N/A	0.160906
BP_GO:0046321_positive+regulation+of+fatty+acid+oxidation	0.160906	#N/A	0.160906
BP_GO:0035038_female+pronucleus+formation	0.277188	0.277188	#N/A
BP_GO:0035039_male+pronucleus+formation	0.277188	0.277188	#N/A
CC_GO:0033011_perinuclear+theca	0.277188	0.277188	#N/A
MF_GO:0004986_delta-opioid+receptor+activity	0.549984	#N/A	0.549984
MF_GO:0004744_retinal+isomerase+activity	0.715044	0.715044	#N/A
BP_GO:0007321_sperm+displacement	0.472396	#N/A	0.472396
MF_GO:0004723_calcium-dependent+protein+serine/threonine+phosphata:	0.472396	#N/A	0.472396
MF_GO:0004059_aralkylamine+N-acetyltransferase+activity	0.719497	#N/A	0.719497
MF_GO:0004167_dopachrome+isomerase+activity	0.0451637	#N/A	0.0451637
MF_GO:0035299_inositol+pentakisphosphate+2-kinase+activity	0.396734	#N/A	0.396734
CC_GO:0012510_trans-Golgi+network+transport+vesicle+membrane	0.781393	0.781393	#N/A
MF_GO:0033819_lipoyl(octanoyl)+transferase+activity	0.113557	#N/A	0.113557
MF_GO:0008897_holo-[acyl-carrier-protein]+synthase+activity	0.151585	0.151585	#N/A
BP_GO:0042297_vocal+learning	0.798017	#N/A	0.798017
MF_GO:0008507_sodium:iodide+symporter+activity	0.191077	#N/A	0.191077
BP_GO:0018146_keratan+sulfate+biosynthetic+process	0.607657	0.607657	#N/A
MF_GO:0001517_N-acetylglucosamine+6-O-sulfotransferase+activity	0.607657	0.607657	#N/A
BP_GO:0009629_response+to+gravity	0.744564	0.744564	#N/A

BP_GO:0048840_otolith+development	0.744564	0.744564	#N/A
CC_GO:0043020_NADPH+oxidase+complex	0.744564	0.744564	#N/A
CC_GO:0005775_vacuolar+lumen	0.509152	0.509152	#N/A
MF_GO:0015105_arsenite+transmembrane+transporter+activity	0.959035	0.959035	#N/A
MF_GO:0004891_glycine-inhibited+chloride+channel+activity	0.572667	0.572667	#N/A
MF_GO:0050694_galactose+3-O-sulfotransferase+activity	0.279888	#N/A	0.279888
MF_GO:0005132_interferon-alpha/beta+receptor+binding	0.774119	0.774119	#N/A
MF_GO:0015125_bile+acid+transmembrane+transporter+activity	0.314979	0.314979	#N/A
MF_GO:0004808_tRNA+(5-methylaminomethyl-2-thiouridylate)-methyltrans	0.289589	0.289589	#N/A
CC_GO:0032437_cuticular+plate	0.463227	#N/A	0.463227
CC_GO:0060091_kinocilium	0.463227	#N/A	0.463227
BP_GO:0042350_GDP-L-fucose+biosynthetic+process	0.637462	#N/A	0.637462
MF_GO:0047341_fucose-1-phosphate+guanylyltransferase+activity	0.637462	#N/A	0.637462
BP_GO:0009593_detection+of+chemical+stimulus	0.243681	0.243681	#N/A
BP_GO:0021559_trigeminal+nerve+development	0.243681	0.243681	#N/A
MF_GO:0005329_dopamine+transmembrane+transporter+activity	0.927091	0.927091	#N/A
MF_GO:0005330_dopamine:sodium+symporter+activity	0.927091	0.927091	#N/A
MF_GO:0000253_3-keto+sterol+reductase+activity	0.562664	0.562664	#N/A
MF_GO:0050576_3-keto-steroid+reductase+activity	0.562664	0.562664	#N/A
BP_GO:0042107_cytokine+metabolic+process	0.761085	0.761085	#N/A
BP_GO:0060008_Sertoli+cell+differentiation	0.217701	0.217701	#N/A
CC_GO:0030677_ribonuclease+P+complex	0.646661	#N/A	0.646661
BP_GO:0006368_RNA+elongation+from+RNA+polymerase+II+promoter	0.841592	0.841592	#N/A
BP_GO:0048133_male+germ-line+stem+cell+division	0.757503	0.757503	#N/A
MF_GO:0000217_DNA+secondary+structure+binding	0.645503	0.645503	#N/A
BP_GO:0042790_transcription+of+nuclear+rRNA+large+RNA+polymerase+I+	0.708048	0.708048	#N/A
BP_GO:0021891_olfactory+bulb+interneuron+development	0.309195	#N/A	0.309195
BP_GO:0035106_operant+conditioning	0.735284	0.735284	#N/A
BP_GO:0046323_glucose+import	0.735284	0.735284	#N/A
BP_GO:0051968_positive+regulation+of+synaptic+transmission,+glutamater	0.735284	0.735284	#N/A
MF_GO:0001590_dopamine+D1+receptor+activity	0.735284	0.735284	#N/A
BP_GO:0021521_ventral+spinal+cord+interneuron+specification	0.367384	#N/A	0.367384
BP_GO:0030954_spindle+astral+microtubule+nucleation	0.502017	0.502017	#N/A
MF_GO:0042163_interleukin-12+beta+subunit+binding	0.817419	#N/A	0.817419
BP_GO:0000712_resolution+of+meiotic+joint+molecules+as+recombinants	0.817458	0.817458	#N/A
BP_GO:0007060_male+meiosis+chromosome+segregation	0.817458	0.817458	#N/A
BP_GO:0043060_meiotic+metaphase+I+plate+congression	0.817458	0.817458	#N/A
BP_GO:0051257_spindle+midzone+assembly+involved+in+meiosis	0.817458	0.817458	#N/A
MF_GO:0045295_gamma-catenin+binding	0.880863	#N/A	0.880863
MF_GO:0051393_alpha-actinin+binding	0.880863	#N/A	0.880863
MF_GO:0070097_delta-catenin+binding	0.880863	#N/A	0.880863
BP_GO:0042137_sequestering+of+neurotransmitter	0.422471	#N/A	0.422471
CC_GO:0060076_excitatory+synapse	0.422471	#N/A	0.422471
MF_GO:0015319_sodium:inorganic+phosphate+symporter+activity	0.422471	#N/A	0.422471
CC_GO:0042571_immunoglobulin+complex,+circulating	0.359243	#N/A	0.359243
BP_GO:0032289_myelin+formation+in+the+central+nervous+system	0.842226	0.842226	#N/A
BP_GO:0031053_primary+microRNA+processing	0.924846	0.924846	#N/A

MF_GO:0000171_ribonuclease+MRP+activity	0.583612	#N/A	0.583612
BP_GO:0051639_actin+filament+network+formation	0.474138	#N/A	0.474138
BP_GO:0006844_acyl+carnitine+transport	0.404636	#N/A	0.404636
MF_GO:0015227_acyl+carnitine+transporter+activity	0.404636	#N/A	0.404636
BP_GO:0002729_positive+regulation+of+natural+killer+cell+cytokine+produ	0.59807	#N/A	0.59807
BP_GO:0032825_positive+regulation+of+natural+killer+cell+differentiation	0.59807	#N/A	0.59807
BP_GO:0046148_pigment+biosynthetic+process	0.253987	#N/A	0.253987
BP_GO:0048852_diencephalon+morphogenesis	0.253987	#N/A	0.253987
BP_GO:0033577_protein+amino+acid+glycosylation+in+endoplasmic+reticul	0.945028	0.945028	#N/A
BP_GO:0006895_Golgi+to+endosome+transport	0.566304	#N/A	0.566304
MF_GO:0030292_protein+tyrosine+kinase+inhibitor+activity	0.757621	#N/A	0.757621
BP_GO:0045994_positive+regulation+of+translational+initiation+by+iron	0.397986	0.397986	#N/A
BP_GO:0045004_DNA+replication+proofreading	0.165343	0.165343	#N/A
BP_GO:0046032_ADP+catabolic+process	0.387743	#N/A	0.387743
BP_GO:0046709_IDP+catabolic+process	0.387743	#N/A	0.387743
BP_GO:0001984_vasodilation+of+artery+during+baroreceptor+response+to-	0.371168	#N/A	0.371168
BP_GO:0031645_negative+regulation+of+neurological+system+process	0.371168	#N/A	0.371168
MF_GO:0031716_calcitonin+receptor+binding	0.371168	#N/A	0.371168
BP_GO:0060334_regulation+of+interferon-gamma-mediated+signaling+path	0.454115	#N/A	0.454115
BP_GO:0060338_regulation+of+type+I+interferon-mediated+signaling+path	0.454115	#N/A	0.454115
MF_GO:0004940_beta1-adrenergic+receptor+activity	0.242175	#N/A	0.242175
BP_GO:0006688_glycosphingolipid+biosynthetic+process	0.794604	0.794604	#N/A
MF_GO:0001962_alpha-1,3-galactosyltransferase+activity	0.794604	0.794604	#N/A
CC_GO:0035086_flagellar+axoneme	0.657999	0.657999	#N/A
BP_GO:0040013_negative+regulation+of+locomotion	0.317081	0.317081	#N/A
MF_GO:0016608_growth+hormone-releasing+hormone+activity	0.317081	0.317081	#N/A
BP_GO:0001819_positive+regulation+of+cytokine+production	0.694616	#N/A	0.694616
BP_GO:0045740_positive+regulation+of+DNA+replication	0.694616	#N/A	0.694616
BP_GO:0030222_eosinophil+differentiation	0.215723	#N/A	0.215723
MF_GO:0030380_interleukin-17E+receptor+binding	0.215723	#N/A	0.215723
BP_GO:0006782_protoporphyrinogen+IX+biosynthetic+process	0.161271	0.161271	#N/A
BP_GO:0001562_response+to+protozoan	0.867816	#N/A	0.867816
BP_GO:0001808_negative+regulation+of+type+IV+hypersensitivity	0.867816	#N/A	0.867816
BP_GO:0050688_regulation+of+defense+response+to+virus	0.867816	#N/A	0.867816
MF_GO:0008367_bacterial+binding	0.867816	#N/A	0.867816
BP_GO:0006408_snRNA+export+from+nucleus	0.382076	#N/A	0.382076
BP_GO:0021930_granule+cell+precursor+proliferation	0.658572	0.658572	#N/A
BP_GO:0048483_autonomic+nervous+system+development	0.658572	0.658572	#N/A
MF_GO:0000170_sphingosine+hydroxylase+activity	0.464182	0.464182	#N/A
BP_GO:0006007_glucose+catabolic+process	0.78674	0.78674	#N/A
BP_GO:0030449_regulation+of+complement+activation	0.266493	0.266493	#N/A
BP_GO:0018283_iron+incorporation+into+metallo-sulfur+cluster	0.982581	0.982581	#N/A
MF_GO:0031071_cysteine+desulfurase+activity	0.982581	0.982581	#N/A
MF_GO:0008569_minus-end-directed+microtubule+motor+activity	0.528223	0.528223	#N/A
MF_GO:0004581_dolichyl-phosphate+beta-glucosyltransferase+activity	0.418471	#N/A	0.418471
BP_GO:0033141_positive+regulation+of+peptidyl-serine+phosphorylation+o	0.990955	0.990955	#N/A
MF_GO:0005133_interferon-gamma+receptor+binding	0.990955	0.990955	#N/A

BP_GO:0006851_mitochondrial+calcium+ion+transport	0.220885	0.220885	#N/A
BP_GO:0048312_intracellular+distribution+of+mitochondria	0.220885	0.220885	#N/A
BP_GO:0002572_pro-T+cell+differentiation	0.299803	#N/A	0.299803
MF_GO:0043548_phosphoinositide+3-kinase+binding	0.299803	#N/A	0.299803
CC_GO:0042581_specific+granule	0.839962	0.839962	#N/A
BP_GO:0051573_negative+regulation+of+histone+H3-K9+methylation	0.33513	0.33513	#N/A
MF_GO:0004398_histidine+decarboxylase+activity	0.378027	0.378027	#N/A
BP_GO:0048284_organelle+fusion	0.910876	#N/A	0.910876
BP_GO:0010507_negative+regulation+of+autophagy	0.450347	#N/A	0.450347
BP_GO:0033028_myeloid+cell+apoptosis	0.450347	#N/A	0.450347
MF_GO:0005135_interleukin-3+receptor+binding	0.450347	#N/A	0.450347
BP_GO:0046986_negative+regulation+of+hemoglobin+biosynthetic+process	0.312134	0.312134	#N/A
MF_GO:0031770_growth+hormone-releasing+hormone+receptor+binding	0.851771	0.851771	#N/A
BP_GO:0021858_GABAergic+neuron+differentiation+in+the+basal+ganglia	0.131365	#N/A	0.131365
BP_GO:0001920_negative+regulation+of+receptor+recycling	0.839522	0.839522	#N/A
CC_GO:0009346_citrate+lyase+complex	0.401613	0.401613	#N/A
MF_GO:0008815_citrate+(pro-3S)-lyase+activity	0.401613	0.401613	#N/A
MF_GO:0016830_carbon-carbon+lyase+activity	0.401613	0.401613	#N/A
BP_GO:0021933_radial+glia+guided+migration+of+granule+cell	0.187408	0.187408	#N/A
BP_GO:0051386_regulation+of+nerve+growth+factor+receptor+signaling+pa	0.187408	0.187408	#N/A
CC_GO:0016507_fatty+acid+beta-oxidation+multienzyme+complex	0.415164	#N/A	0.415164
MF_GO:0016509_long-chain-3-hydroxyacyl-CoA+dehydrogenase+activity	0.415164	#N/A	0.415164
MF_GO:0004170_dUTP+diphosphatase+activity	0.664554	0.664554	#N/A
BP_GO:0000305_response+to+oxygen+radical	0.81616	0.81616	#N/A
MF_GO:0019534_toxin+transporter+activity	0.149505	0.149505	#N/A
BP_GO:0046324_regulation+of+glucose+import	0.831687	0.831687	#N/A
CC_GO:0031315_extrinsic+to+mitochondrial+outer+membrane	0.519036	#N/A	0.519036
MF_GO:0050254_rhodopsin+kinase+activity	0.180461	0.180461	#N/A
BP_GO:0043624_cellular+protein+complex+disassembly	0.428721	#N/A	0.428721
BP_GO:0009691_cytokinin+biosynthetic+process	0.112823	0.112823	#N/A
BP_GO:0045980_negative+regulation+of+nucleotide+metabolic+process	0.755818	#N/A	0.755818
BP_GO:0006304_DNA+modification	0.0441558	#N/A	0.0441558
CC_GO:0005726_perichromatin+fibrils	0.994421	0.994421	#N/A
BP_GO:0033326_cerebrospinal+fluid+secretion	0.925422	0.925422	#N/A
BP_GO:0048546_digestive+tract+morphogenesis	0.925422	0.925422	#N/A
BP_GO:0002508_central+tolerance+induction	0.316684	#N/A	0.316684
BP_GO:0002635_negative+regulation+of+germinal+center+formation	0.316684	#N/A	0.316684
BP_GO:0002897_positive+regulation+of+central+B+cell+tolerance+inductior	0.316684	#N/A	0.316684
BP_GO:0032862_activation+of+Rho+GTPase+activity	0.316684	#N/A	0.316684
BP_GO:0033085_negative+regulation+of+T+cell+differentiation+in+the+thyr	0.316684	#N/A	0.316684
MF_GO:0004603_phenylethanolamine+N-methyltransferase+activity	0.543465	#N/A	0.543465
BP_GO:0000072_M+phase+specific+microtubule+process	0.720166	0.720166	#N/A
CC_GO:0045111_intermediate+filament+cytoskeleton	0.90774	#N/A	0.90774
CC_GO:0032302_MutSbeta+complex	0.507021	0.507021	#N/A
BP_GO:0009105_lipoic+acid+biosynthetic+process	0.684589	0.684589	#N/A
MF_GO:0016992_lipoate+synthase+activity	0.684589	0.684589	#N/A
MF_GO:0017140_lipoic+acid+synthase+activity	0.684589	0.684589	#N/A

BP_GO:0002361_CD4-positive,+CD25-positive,+alpha-beta+regulatory+T+cel	0.456422	0.456422	#N/A
BP_GO:0002637_regulation+of+immunoglobulin+production	0.456422	0.456422	#N/A
BP_GO:0002677_negative+regulation+of+chronic+inflammatory+response	0.456422	0.456422	#N/A
BP_GO:0002851_positive+regulation+of+peripheral+T+cell+tolerance+induc	0.456422	0.456422	#N/A
BP_GO:0032693_negative+regulation+of+interleukin-10+production	0.456422	0.456422	#N/A
BP_GO:0032914_positive+regulation+of+transforming+growth+factor-beta1	0.456422	0.456422	#N/A
BP_GO:0035066_positive+regulation+of+histone+acetylation	0.456422	0.456422	#N/A
BP_GO:0045591_positive+regulation+of+regulatory+T+cell+differentiation	0.456422	0.456422	#N/A
BP_GO:0010569_regulation+of+double-strand+break+repair+via+homologou	0.532734	#N/A	0.532734
BP_GO:0034502_protein+localization+to+chromosome	0.532734	#N/A	0.532734
BP_GO:0045650_negative+regulation+of+macrophage+differentiation	0.497247	0.497247	#N/A
BP_GO:0007210_serotonin+receptor+signaling+pathway	0.610112	0.610112	#N/A
MF_GO:0005232_serotonin-activated+cation-selective+channel+activity	0.610112	0.610112	#N/A
BP_GO:0002253_activation+of+immune+response	0.669044	0.669044	#N/A
BP_GO:0002381_immunoglobulin+production+during+immune+response	0.669044	0.669044	#N/A
BP_GO:0006965_positive+regulation+of+biosynthetic+process+of+antibacte	0.669044	0.669044	#N/A
BP_GO:0032701_negative+regulation+of+interleukin-18+production	0.669044	0.669044	#N/A
BP_GO:0034136_negative+regulation+of+toll-like+receptor+2+signaling+pat	0.669044	0.669044	#N/A
BP_GO:0008616_queuosine+biosynthetic+process	0.904599	0.904599	#N/A
MF_GO:0008479_queuine+tRNA-ribosyltransferase+activity	0.904599	0.904599	#N/A
BP_GO:0046949_acyl-CoA+biosynthetic+process	0.123033	0.123033	#N/A
MF_GO:0043754_dihydrolipoyllysine-residue+(2-methylpropanoyl)transfera	0.123033	0.123033	#N/A
MF_GO:0008605_protein+kinase+CK2+regulator+activity	0.216739	#N/A	0.216739
BP_GO:0048855_adenohypophysis+morphogenesis	0.12057	#N/A	0.12057
MF_GO:0001972_retinoic+acid+binding	0.759392	#N/A	0.759392
BP_GO:0009649_entrainment+of+circadian+clock	0.156116	#N/A	0.156116
MF_GO:0008532_N-acetyllactosaminide+beta-1,3-N-acetylglucosaminyltran	0.595544	0.595544	#N/A
MF_GO:0031957_very-long-chain-fatty-acid-CoA+ligase+activity	0.436434	0.436434	#N/A
BP_GO:0032317_regulation+of+Rap+GTPase+activity	0.885441	#N/A	0.885441
BP_GO:0031034_myosin+filament+assembly	0.427793	0.427793	#N/A
MF_GO:0032036_myosin+heavy+chain+binding	0.427793	0.427793	#N/A
BP_GO:0042985_negative+regulation+of+amyloid+precursor+protein+biosy	0.529923	#N/A	0.529923
MF_GO:0016019_peptidoglycan+receptor+activity	0.211877	#N/A	0.211877
MF_GO:0008097_5S+rRNA+binding	0.456115	0.456115	#N/A
BP_GO:0042309_homoiothermy	0.528987	0.528987	#N/A
BP_GO:0048714_positive+regulation+of+oligodendrocyte+differentiation	0.81779	0.81779	#N/A
MF_GO:0004997_thyrotropin-releasing+hormone+receptor+activity	0.820539	#N/A	0.820539
MF_GO:0047685_amine+sulfotransferase+activity	0.130835	0.130835	#N/A
BP_GO:0048857_neural+nucleus+development	0.994972	0.994972	#N/A
BP_GO:0007423_sensory+organ+development	0.120967	#N/A	0.120967
BP_GO:0010038_response+to+metal+ion	0.158422	#N/A	0.158422
BP_GO:0021589_cerebellum+structural+organization	0.750916	0.750916	#N/A
BP_GO:0021813_cell-cell+adhesion+involved+in+neuronal-glial+interactions	0.750916	0.750916	#N/A
BP_GO:0021942_radial+glia+guided+migration+of+Purkinje+cell	0.750916	0.750916	#N/A
BP_GO:0060018_astrocyte+fate+commitment	0.148085	0.148085	#N/A
MF_GO:0047045_testosterone+17-beta-dehydrogenase+(NADP+)+activity	0.718072	0.718072	#N/A
BP_GO:0032696_negative+regulation+of+interleukin-13+production	0.710379	#N/A	0.710379

BP_GO:0001682_tRNA+5'-leader+removal	0.0323974	0.0323974	#N/A
CC_GO:0030133_transport+vesicle	0.54676	#N/A	0.54676
CC_GO:0001651_dense+fibrillar+component	0.302708	#N/A	0.302708
BP_GO:0009134_nucleoside+diphosphate+catabolic+process	0.245898	0.245898	#N/A
BP_GO:0009143_nucleoside+triphosphate+catabolic+process	0.245898	0.245898	#N/A
BP_GO:0003029_detection+of+hypoxic+conditions+in+blood+by+carotid+bc	0.821328	0.821328	#N/A
BP_GO:0060064_Spemann+organizer+formation+at+the+anterior+end+of+t	0.483819	#N/A	0.483819
MF_GO:0003851_2-hydroxyacylsphingosine+1-beta-galactosyltransferase+a	0.685194	0.685194	#N/A
MF_GO:0050577_GDP-L-fucose+synthase+activity	0.968006	0.968006	#N/A
BP_GO:0046373_L-arabinose+metabolic+process	0.399321	0.399321	#N/A
MF_GO:0046556_alpha-N-arabinofuranosidase+activity	0.399321	0.399321	#N/A
BP_GO:0007168_receptor+guanylyl+cyclase+signaling+pathway	0.374088	#N/A	0.374088
BP_GO:0016344_meiotic+chromosome+movement+towards+spindle+pole	0.895448	0.895448	#N/A
BP_GO:0040038_polar+body+extrusion+after+meiotic+divisions	0.895448	0.895448	#N/A
BP_GO:0007506_gonadal+mesoderm+development	0.197997	0.197997	#N/A
BP_GO:0014808_release+of+sequestered+calcium+ion+into+cytosol+by+sar	0.820799	#N/A	0.820799
MF_GO:0001567_cholesterol+25-hydroxylase+activity	0.833553	#N/A	0.833553
MF_GO:0008395_steroid+hydroxylase+activity	0.833553	#N/A	0.833553
CC_GO:0009317_acetyl-CoA+carboxylase+complex	0.779433	0.779433	#N/A
CC_GO:0016235_aggresome	0.514797	#N/A	0.514797
BP_GO:0000256_allantoin+catabolic+process	0.209435	0.209435	#N/A
MF_GO:0004037_allantoicase+activity	0.209435	0.209435	#N/A
BP_GO:0030540_female+genitalia+development	0.62579	#N/A	0.62579
MF_GO:0001631_cysteinyl+leukotriene+receptor+activity	0.0886784	0.0886784	#N/A
BP_GO:0002003_angiotensin+maturation	0.908367	0.908367	#N/A
MF_GO:0042922_neuromedin+U+receptor+binding	0.499563	0.499563	#N/A
MF_GO:0016005_phospholipase+A2+activator+activity	0.483906	#N/A	0.483906
BP_GO:0002085_repression+of+premature+neural+plate+formation	0.828234	#N/A	0.828234
MF_GO:0003948_N4-(beta-N-acetylglucosaminyl)-L-asparaginase+activity	0.250365	#N/A	0.250365
BP_GO:0048681_negative+regulation+of+axon+regeneration	0.532534	0.532534	#N/A
BP_GO:0050807_regulation+of+synapse+organization	0.532534	0.532534	#N/A
BP_GO:0021889_olfactory+bulb+interneuron+differentiation	0.473948	#N/A	0.473948
BP_GO:0051026_chiasma+formation	0.432607	#N/A	0.432607
MF_GO:0047915_ganglioside+galactosyltransferase+activity	0.561713	#N/A	0.561713
MF_GO:0050681_androgen+receptor+binding	0.907022	0.907022	#N/A
MF_GO:0001604_urotensin+II+receptor+activity	0.915433	0.915433	#N/A
BP_GO:0021874_Wnt+receptor+signaling+pathway+in+forebrain+neuroblas	0.476187	0.476187	#N/A
BP_GO:0045839_negative+regulation+of+mitosis	0.41158	0.41158	#N/A
MF_GO:0004987_kappa-opioid+receptor+activity	0.603693	0.603693	#N/A
BP_GO:0045661_regulation+of+myoblast+differentiation	0.99298	0.99298	#N/A
MF_GO:0046527_glucosyltransferase+activity	0.205645	0.205645	#N/A
BP_GO:0018009_N-terminal+peptidyl-L-cysteine+N-palmitoylation	0.180773	#N/A	0.180773
MF_GO:0008757_S-adenosylmethionine-dependent+methyltransferase+acti	0.415689	#N/A	0.415689
MF_GO:0046403_polynucleotide+3'-phosphatase+activity	0.563221	0.563221	#N/A
CC_GO:0031231_intrinsic+to+peroxisomal+membrane	0.427861	#N/A	0.427861
MF_GO:0004811_tRNA+isopentenyltransferase+activity	0.669419	0.669419	#N/A
BP_GO:0042667_auditory+receptor+cell+fate+specification	0.565681	#N/A	0.565681

BP_GO:0045609_positive+regulation+of+auditory+receptor+cell+differentiat	0.565681	#N/A	0.565681
BP_GO:0021535_cell+migration+in+hindbrain	0.267497	#N/A	0.267497
BP_GO:0048934_peripheral+nervous+system+neuron+differentiation	0.267497	#N/A	0.267497
BP_GO:0051355_proprioception+during+equilibrioception	0.267497	#N/A	0.267497
BP_GO:0035260_internal+genitalia+morphogenesis	0.223585	0.223585	#N/A
BP_GO:0034339_regulation+of+transcription+from+RNA+polymerase+II+prc	0.98638	0.98638	#N/A
BP_GO:0002882_positive+regulation+of+chronic+inflammatory+response+t	0.76422	0.76422	#N/A
BP_GO:0008038_neuron+recognition	0.988139	0.988139	#N/A
BP_GO:0055078_sodium+ion+homeostasis	0.67124	0.67124	#N/A
BP_GO:0043486_histone+exchange	0.863448	0.863448	#N/A
MF_GO:0030354_melanin-concentrating+hormone+activity	0.715125	0.715125	#N/A
BP_GO:0022004_midbrain-hindbrain+boundary+maturation+during+brain+c	0.711441	0.711441	#N/A
MF_GO:0042164_interleukin-12+alpha+subunit+binding	0.675367	0.675367	#N/A
BP_GO:0060231_mesenchymal+to+epithelial+transition	0.358222	#N/A	0.358222
BP_GO:0002432_granuloma+formation	0.589006	0.589006	#N/A
MF_GO:0000224_peptide-N4-(N-acetyl-beta-glucosaminyl)asparagine+amid	0.770793	#N/A	0.770793
MF_GO:0051267_CP2+mannose-ethanolamine+phosphotransferase+activity	0.567824	0.567824	#N/A
BP_GO:0001980_regulation+of+systemic+arterial+blood+pressure+by+ische	0.500187	#N/A	0.500187
CC_GO:0048476_Holliday+junction+resolvase+complex	0.590369	#N/A	0.590369
MF_GO:0008821_crossover+junction+endodeoxyribonuclease+activity	0.590369	#N/A	0.590369
MF_GO:0004521_endoribonuclease+activity	0.582721	0.582721	#N/A
MF_GO:0032542_sulfiredoxin+activity	0.230823	0.230823	#N/A
MF_GO:0019706_protein-cysteine+S-palmitoleyltransferase+activity	0.176479	#N/A	0.176479
BP_GO:0007614_short-term+memory	0.94699	#N/A	0.94699
BP_GO:0033555_multicellular+organismal+response+to+stress	0.94699	#N/A	0.94699
CC_GO:0048787_presynaptic+active+zone+membrane	0.94699	#N/A	0.94699
BP_GO:0001774_microglial+cell+activation	0.232332	#N/A	0.232332
BP_GO:0001956_positive+regulation+of+neurotransmitter+secretion	0.232332	#N/A	0.232332
BP_GO:0014048_regulation+of+glutamate+secretion	0.232332	#N/A	0.232332
BP_GO:0034599_cellular+response+to+oxidative+stress	0.232332	#N/A	0.232332
BP_GO:0042775_mitochondrial+ATP+synthesis+coupled+electron+transport	0.232332	#N/A	0.232332
BP_GO:0050812_regulation+of+acyl-CoA+biosynthetic+process	0.232332	#N/A	0.232332
MF_GO:0019855_calcium+channel+inhibitor+activity	0.731043	0.731043	#N/A
BP_GO:0006747_FAD+biosynthetic+process	0.971874	0.971874	#N/A
MF_GO:0003919_FMN+adenylyltransferase+activity	0.971874	0.971874	#N/A
MF_GO:0033699_DNA+5'-adenosine+monophosphate+hydrolase+activity	0.127825	#N/A	0.127825
BP_GO:0006515_misfolded+or+incompletely+synthesized+protein+catabolic	0.35138	#N/A	0.35138
BP_GO:0019932_second-messenger-mediated+signaling	0.286639	0.286639	#N/A
BP_GO:0002554_serotonin+secretion+by+platelet	0.664789	0.664789	#N/A
BP_GO:0003056_regulation+of+vascular+smooth+muscle+contraction	0.664789	0.664789	#N/A
CC_GO:0031240_external+side+of+cell+outer+membrane	0.664789	0.664789	#N/A
MF_GO:0008118_N-acetyllactosaminide+alpha-2,3-sialyltransferase+activity	0.248695	0.248695	#N/A
MF_GO:0004875_complement+receptor+activity	0.513204	#N/A	0.513204
BP_GO:0007062_sister+chromatid+cohesion	0.347171	0.347171	#N/A
BP_GO:0010042_response+to+manganese+ion	0.693539	0.693539	#N/A
BP_GO:0032025_response+to+cobalt+ion	0.693539	0.693539	#N/A
BP_GO:0032026_response+to+magnesium+ion	0.693539	0.693539	#N/A

MF_GO:0051990_(R)-2-hydroxyglutarate+dehydrogenase+activity	0.693539	0.693539	#N/A
MF_GO:0003830_beta-1,4-mannosylglycoprotein+4-beta-N-acetylglucosami	0.54036	#N/A	0.54036
BP_GO:0048050_post-embryonic+eye+morphogenesis	0.291142	#N/A	0.291142
BP_GO:0043438_acetoacetic+acid+metabolic+process	0.613491	#N/A	0.613491
CC_GO:0033162_melanosome+membrane	0.613491	#N/A	0.613491
MF_GO:0016716_oxidoreductase+activity,+acting+on+paired+donors,+with-	0.613491	#N/A	0.613491
BP_GO:0048250_mitochondrial+iron+ion+transport	0.868248	0.868248	#N/A
BP_GO:0006082_organic+acid+metabolic+process	0.924213	0.924213	#N/A
BP_GO:0021999_neural+plate+anterioposterior+pattern+formation	0.291866	0.291866	#N/A
BP_GO:0032583_regulation+of+gene-specific+transcription	0.291866	0.291866	#N/A
MF_GO:0032394_MHC+class+Ib+receptor+activity	0.432173	0.432173	#N/A
MF_GO:0042288_MHC+class+I+protein+binding	0.432173	0.432173	#N/A
BP_GO:0002536_respiratory+burst+during+acute+inflammatory+response	0.451324	#N/A	0.451324
BP_GO:0010310_regulation+of+hydrogen+peroxide+metabolic+process	0.451324	#N/A	0.451324
BP_GO:0010671_negative+regulation+of+oxygen+and+reactive+oxygen+spe	0.451324	#N/A	0.451324
BP_GO:0031665_negative+regulation+of+lipopolysaccharide-mediated+sign	0.451324	#N/A	0.451324
MF_GO:0008379_thioredoxin+peroxidase+activity	0.451324	#N/A	0.451324
BP_GO:0009249_protein+lipoylation	0.473221	0.473221	#N/A
BP_GO:0021761_limbic+system+development	0.291673	#N/A	0.291673
MF_GO:0005144_interleukin-13+receptor+binding	0.646553	0.646553	#N/A
MF_GO:0001621_platelet+ADP+receptor+activity	0.44918	#N/A	0.44918
BP_GO:0006337_nucleosome+disassembly	0.559681	0.559681	#N/A
MF_GO:0004509_steroid+21-monooxygenase+activity	0.186363	#N/A	0.186363
MF_GO:0005092_GDP-dissociation+inhibitor+activity	0.599434	#N/A	0.599434
BP_GO:0009187_cyclic+nucleotide+metabolic+process	0.0970532	#N/A	0.0970532
MF_GO:0005223_intracellular+cGMP+activated+cation+channel+activity	0.0970532	#N/A	0.0970532
MF_GO:0005502_11-cis+retinal+binding	0.0605071	0.0605071	#N/A
MF_GO:0003980_UDP-glucose:glycoprotein+glucosyltransferase+activity	0.276097	#N/A	0.276097
CC_GO:0032592_integral+to+mitochondrial+membrane	0.640659	#N/A	0.640659
MF_GO:0016846_carbon-sulfur+lyase+activity	0.437713	#N/A	0.437713
BP_GO:0002317_plasma+cell+differentiation	0.83092	0.83092	#N/A
BP_GO:0016333_morphogenesis+of+follicular+epithelium	0.83092	0.83092	#N/A
BP_GO:0048621_post-embryonic+gut+morphogenesis	0.83092	0.83092	#N/A
BP_GO:0010629_negative+regulation+of+gene+expression	0.394286	#N/A	0.394286
BP_GO:0034984_cellular+response+to+DNA+damage+stimulus	0.394286	#N/A	0.394286
BP_GO:0043046_DNA+methylation+during+gametogenesis	0.394286	#N/A	0.394286
MF_GO:0050429_calcium-dependent+phospholipase+C+activity	0.471557	#N/A	0.471557
MF_GO:0033798_thyroxine+5-deiodinase+activity	0.842224	#N/A	0.842224
BP_GO:0008626_induction+of+apoptosis+by+granzyme	0.383134	0.383134	#N/A
BP_GO:0048525_negative+regulation+of+viral+reproduction	0.778372	#N/A	0.778372
MF_GO:0005136_interleukin-4+receptor+binding	0.276517	0.276517	#N/A
BP_GO:0019101_female+somatic+sex+determination	0.773475	0.773475	#N/A
BP_GO:0060014_granulosa+cell+differentiation	0.773475	0.773475	#N/A
BP_GO:0003032_detection+of+oxygen	0.139698	#N/A	0.139698
BP_GO:0003069_vasodilation+by+acetylcholine+involved+in+regulation+of+	0.139698	#N/A	0.139698
BP_GO:0010260_organ+senescence	0.139698	#N/A	0.139698
BP_GO:0048773_erythrophore+differentiation	0.139698	#N/A	0.139698

BP_GO:0055093_response+to+hyperoxia	0.139698	#N/A	0.139698
MF_GO:0004027_alcohol+sulfotransferase+activity	0.430293	#N/A	0.430293
MF_GO:0004964_lutropin-choriogonadotropic+hormone+receptor+activity	0.192198	#N/A	0.192198
MF_GO:0004004_ATP-dependent+RNA+helicase+activity	0.838489	0.838489	#N/A
BP_GO:0051100_negative+regulation+of+binding	0.135166	0.135166	#N/A
MF_GO:0008252_nucleotidase+activity	0.280588	0.280588	#N/A
BP_GO:0006356_regulation+of+transcription+from+RNA+polymerase+I+pro	0.978087	0.978087	#N/A
CC_GO:0030685_nucleolar+preribosome	0.695806	#N/A	0.695806
BP_GO:0007113_endomitotic+cell+cycle	0.745117	0.745117	#N/A
MF_GO:0019781_NEDD8+activating+enzyme+activity	0.745117	0.745117	#N/A
MF_GO:0015379_potassium:chloride+symporter+activity	0.904258	0.904258	#N/A
MF_GO:0032558_adenyl+deoxyribonucleotide+binding	0.467934	0.467934	#N/A
BP_GO:0046578_regulation+of+Ras+protein+signal+transduction	0.729729	#N/A	0.729729
MF_GO:0004671_protein-S-isoprenylcysteine+O-methyltransferase+activity	0.729729	#N/A	0.729729
MF_GO:0008140_cAMP+response+element+binding+protein+binding	0.729729	#N/A	0.729729
BP_GO:0048691_positive+regulation+of+axon+extension+involved+in+reger	0.526928	#N/A	0.526928
BP_GO:0006585_dopamine+biosynthetic+process+from+tyrosine	0.859886	0.859886	#N/A
MF_GO:0004511_tyrosine+3-monooxygenase+activity	0.859886	0.859886	#N/A
BP_GO:0032962_positive+regulation+of+inositol+trisphosphate+biosyntheti	0.647652	#N/A	0.647652
BP_GO:0035092_sperm+chromatin+condensation	0.695507	#N/A	0.695507
BP_GO:0000027_ribosomal+large+subunit+assembly+and+maintenance	0.440352	0.440352	#N/A
BP_GO:0010458_exit+from+mitosis	0.440352	0.440352	#N/A
BP_GO:0021554_optic+nerve+development	0.440352	0.440352	#N/A
MF_GO:0005155_epidermal+growth+factor+receptor+activating+ligand+act	0.0825002	#N/A	0.0825002
MF_GO:0005335_serotonin:sodium+symporter+activity	0.865149	0.865149	#N/A
BP_GO:0001696_gastric+acid+secretion	0.76217	#N/A	0.76217
MF_GO:0005253_anion+channel+activity	0.76217	#N/A	0.76217
BP_GO:0000080_G1+phase+of+mitotic+cell+cycle	0.103413	#N/A	0.103413
BP_GO:0045924_regulation+of+female+receptivity	0.489399	0.489399	#N/A
MF_GO:0001589_dopamine+D5+receptor+activity	0.489399	0.489399	#N/A
BP_GO:0031947_negative+regulation+of+glucocorticoid+biosynthetic+proce	0.334292	0.334292	#N/A
BP_GO:0045823_positive+regulation+of+heart+contraction	0.334292	0.334292	#N/A
BP_GO:0045989_positive+regulation+of+striated+muscle+contraction	0.334292	0.334292	#N/A
MF_GO:0003869_4-nitrophenylphosphatase+activity	0.334292	0.334292	#N/A
MF_GO:0004362_glutathione-disulfide+reductase+activity	0.9721	0.9721	#N/A
MF_GO:0004503_monophenol+monooxygenase+activity	0.962482	0.962482	#N/A
BP_GO:0032863_activation+of+Rac+GTPase+activity	0.608426	#N/A	0.608426
BP_GO:0035020_regulation+of+Rac+protein+signal+transduction	0.608426	#N/A	0.608426
BP_GO:0021508_floor+plate+formation	0.347901	#N/A	0.347901
BP_GO:0021517_ventral+spinal+cord+development	0.347901	#N/A	0.347901
BP_GO:0030886_negative+regulation+of+myeloid+dendritic+cell+activation	0.922286	0.922286	#N/A
MF_GO:0005183_gonadotropin+hormone-releasing+hormone+activity	0.71663	0.71663	#N/A
BP_GO:0030655_beta-lactam+antibiotic+catabolic+process	0.228388	0.228388	#N/A
MF_GO:0008800_beta-lactamase+activity	0.228388	0.228388	#N/A
BP_GO:0016267_O-glycan+processing,+core+1	0.94911	0.94911	#N/A
MF_GO:0016263_glycoprotein-N-acetylgalactosamine+3-beta-galactosyltrar	0.94911	0.94911	#N/A
BP_GO:0043487_regulation+of+RNA+stability	0.990107	#N/A	0.990107

BP_GO:0035262_gonad+morphogenesis	0.921978	0.921978	#N/A
BP_GO:0006624_vacuolar+protein+processing	0.191143	0.191143	#N/A
BP_GO:0043313_regulation+of+neutrophil+degranulation	0.185495	0.185495	#N/A
BP_GO:0048850_hypophysis+morphogenesis	0.283344	0.283344	#N/A
BP_GO:0051453_regulation+of+intracellular+pH	0.612836	0.612836	#N/A
MF_GO:0015386_potassium:hydrogen+antiporter+activity	0.612836	0.612836	#N/A
BP_GO:0021523_somatic+motor+neuron+differentiation	0.93861	#N/A	0.93861
BP_GO:0021703_locus+ceruleus+development	0.93861	#N/A	0.93861
BP_GO:0048486_parasympathetic+nervous+system+development	0.93861	#N/A	0.93861
BP_GO:0006984_ER-nuclear+signaling+pathway	0.979733	#N/A	0.979733
MF_GO:0003701_RNA+polymerase+I+transcription+factor+activity	0.442979	#N/A	0.442979
MF_GO:0004968_gonadotropin-releasing+hormone+receptor+activity	0.822213	0.822213	#N/A
BP_GO:0048597_post-embryonic+camera-type+eye+morphogenesis	0.52525	#N/A	0.52525
MF_GO:0008386_cholesterol+monooxygenase+(side-chain-cleaving)+activit	0.196256	0.196256	#N/A
MF_GO:0017065_single-strand+selective+uracil+DNA+N-glycosylase+activity	0.0307172	#N/A	0.0307172
BP_GO:0045347_negative+regulation+of+MHC+class+II+biosynthetic+proces	0.19155	0.19155	#N/A
MF_GO:0001883_purine+nucleoside+binding	0.52178	#N/A	0.52178
MF_GO:0004923_leukemia+inhibitory+factor+receptor+activity	0.179078	#N/A	0.179078
CC_GO:0005751_mitochondrial+respiratory+chain+complex+IV	0.372228	0.372228	#N/A
MF_GO:0050692_DBD+domain+binding	0.671715	0.671715	#N/A
BP_GO:0014012_axon+regeneration+in+the+peripheral+nervous+system	0.897457	0.897457	#N/A
MF_GO:0016536_cyclin-dependent+protein+kinase+5+activator+regulator+a	0.923412	0.923412	#N/A
MF_GO:0016746_transferase+activity,+transferring+acyl+groups	0.343511	#N/A	0.343511
BP_GO:0021658_rhombomere+3+morphogenesis	0.28479	#N/A	0.28479
BP_GO:0045210_FasL+biosynthetic+process	0.0701477	#N/A	0.0701477
MF_GO:0046625_sphingolipid+binding	0.772913	0.772913	#N/A
MF_GO:0008534_oxidized+purine+base+lesion+DNA+N-glycosylase+activity	0.483614	0.483614	#N/A
MF_GO:0034039_8-oxo-7,8-dihydroguanine+DNA+N-glycosylase+activity	0.483614	0.483614	#N/A
MF_GO:0015334_high+affinity+oligopeptide+transporter+activity	0.503107	#N/A	0.503107
BP_GO:0007382_specification+of+segmental+identity,+maxillary+segment	0.82083	0.82083	#N/A
BP_GO:0021532_neural+tube+patterning	0.82083	0.82083	#N/A
BP_GO:0042305_specification+of+segmental+identity,+mandibular+segmen	0.82083	0.82083	#N/A
BP_GO:0042527_negative+regulation+of+tyrosine+phosphorylation+of+Stat	0.342523	#N/A	0.342523
MF_GO:0004577_N-acetylglucosaminyldiphosphodolichol+N-acetylglucosam	0.494888	0.494888	#N/A
BP_GO:0033554_cellular+response+to+stress	0.43112	0.43112	#N/A
BP_GO:0001712_ectodermal+cell+fate+commitment	0.111881	0.111881	#N/A
BP_GO:0045955_negative+regulation+of+calcium+ion-dependent+exocytosi	0.111881	0.111881	#N/A
BP_GO:0006709_progesterone+catabolic+process	0.441779	0.441779	#N/A
MF_GO:0047006_20-alpha-hydroxysteroid+dehydrogenase+activity	0.441779	0.441779	#N/A
MF_GO:0005165_neurotrophin+receptor+binding	0.0334087	#N/A	0.0334087
MF_GO:0004584_dolichyl-phosphate-mannose-glycolipid+alpha-mannosyltr	0.171336	#N/A	0.171336
BP_GO:0051383_kinetochore+organization	0.76992	#N/A	0.76992
MF_GO:0043515_kinetochore+binding	0.76992	#N/A	0.76992
BP_GO:0010032_meiotic+chromosome+condensation	0.572053	0.572053	#N/A
BP_GO:0033315_meiotic+cell+cycle+DNA+replication+checkpoint	0.572053	0.572053	#N/A
BP_GO:0042138_meiotic+DNA+double-strand+break+formation	0.572053	0.572053	#N/A
MF_GO:0030280_structural+constituent+of+epidermis	0.417784	#N/A	0.417784

BP_GO:0050890_cognition	0.503348	#N/A	0.503348
BP_GO:0042256_mature+ribosome+assembly	0.302938	#N/A	0.302938
BP_GO:0000290_deadenylation-dependent+decapping+of+nuclear-transcrib	0.441359	0.441359	#N/A
MF_GO:0002055_adenine+binding	0.418975	0.418975	#N/A
MF_GO:0003999_adenine+phosphoribosyltransferase+activity	0.418975	0.418975	#N/A
MF_GO:0045517_interleukin-20+receptor+binding	0.522048	0.522048	#N/A
MF_GO:0050265_RNA+uridylyltransferase+activity	0.820564	0.820564	#N/A
BP_GO:0045836_positive+regulation+of+meiosis	0.127062	#N/A	0.127062
BP_GO:0045948_positive+regulation+of+translational+initiation	0.127062	#N/A	0.127062
MF_GO:0042156_zinc-mediated+transcriptional+activator+activity	0.589161	0.589161	#N/A
BP_GO:0022408_negative+regulation+of+cell-cell+adhesion	0.84009	0.84009	#N/A
MF_GO:0004102_choline+O-acetyltransferase+activity	0.0444404	0.0444404	#N/A
BP_GO:0031344_regulation+of+cell+projection+organization	0.632164	#N/A	0.632164
MF_GO:0004479_methionyl-tRNA+formyltransferase+activity	0.641174	0.641174	#N/A
CC_GO:0030061_mitochondrial+crista	0.249265	0.249265	#N/A
BP_GO:0032621_interleukin-18+production	0.727395	0.727395	#N/A
BP_GO:0050701_interleukin-1+secretion	0.727395	0.727395	#N/A
BP_GO:0030194_positive+regulation+of+blood+coagulation	0.642319	0.642319	#N/A
MF_GO:0015068_glycine+amidinotransferase+activity	0.21839	0.21839	#N/A
CC_GO:0032426_stereocilium+bundle+tip	0.799039	0.799039	#N/A
BP_GO:0046058_cAMP+metabolic+process	0.806238	0.806238	#N/A
MF_GO:0003905_alkylbase+DNA+N-glycosylase+activity	0.427432	#N/A	0.427432
BP_GO:0032727_positive+regulation+of+interferon-alpha+production	0.575267	#N/A	0.575267
BP_GO:0048280_vesicle+fusion+with+Golgi+apparatus	0.544814	#N/A	0.544814
MF_GO:0008251_tRNA-specific+adenosine+deaminase+activity	0.382676	#N/A	0.382676
BP_GO:0000271_polysaccharide+biosynthetic+process	0.97903	0.97903	#N/A
BP_GO:0048702_embryonic+neurocranium+morphogenesis	0.97903	0.97903	#N/A
CC_GO:0005797_Golgi+medial+cisterna	0.609817	0.609817	#N/A
MF_GO:0047220_galactosylxylosylprotein+3-beta-galactosyltransferase+acti	0.609817	0.609817	#N/A
BP_GO:0006896_Golgi+to+vacuole+transport	0.642448	0.642448	#N/A
BP_GO:0042268_regulation+of+cytolysis	0.0392237	#N/A	0.0392237
CC_GO:0043189_H4/H2A+histone+acetyltransferase+complex	0.332597	#N/A	0.332597
BP_GO:0000238_zygotene	0.648179	0.648179	#N/A
CC_GO:0044451_nucleoplasm+part	0.584242	#N/A	0.584242
MF_GO:0015326_cationic+amino+acid+transmembrane+transporter+activit	0.221806	#N/A	0.221806
CC_GO:0005831_steroid+hormone+aporeceptor+complex	0.689675	0.689675	#N/A
MF_GO:0047429_nucleoside-triphosphate+diphosphatase+activity	0.0347364	#N/A	0.0347364
MF_GO:0030021_extracellular+matrix+structural+constituent+conferring+cc	0.596398	0.596398	#N/A
MF_GO:0030345_structural+constituent+of+tooth+enamel	0.596398	0.596398	#N/A
BP_GO:0032637_interleukin-8+production	0.55829	0.55829	#N/A
BP_GO:0017183_peptidyl-diphthamide+biosynthetic+process+from+peptidy	0.436188	#N/A	0.436188
MF_GO:0004164_diphthine+synthase+activity	0.436188	#N/A	0.436188
MF_GO:0009019_tRNA+(guanine-N1-)-methyltransferase+activity	0.718133	#N/A	0.718133
MF_GO:0042019_interleukin-23+binding	0.102929	0.102929	#N/A
MF_GO:0042020_interleukin-23+receptor+activity	0.102929	0.102929	#N/A
BP_GO:0002678_positive+regulation+of+chronic+inflammatory+response	0.579459	0.579459	#N/A
BP_GO:0002830_positive+regulation+of+T-helper+2+type+immune+respons	0.579459	0.579459	#N/A

BP_GO:0045743_positive+regulation+of+fibroblast+growth+factor+receptor	0.0829484	#N/A	0.0829484
MF_GO:0005222_intracellular+cAMP+activated+cation+channel+activity	0.56713	#N/A	0.56713
BP_GO:0008535_respiratory+chain+complex+IV+assembly	0.45149	#N/A	0.45149
BP_GO:0043467_regulation+of+generation+of+precursor+metabolites+and+	0.729379	0.729379	#N/A
MF_GO:0004810_tRNA+adenylyltransferase+activity	0.835027	#N/A	0.835027
MF_GO:0009022_tRNA+nucleotidyltransferase+activity	0.835027	#N/A	0.835027
MF_GO:0008419_RNA+lariat+debranching+enzyme+activity	0.422733	#N/A	0.422733
CC_GO:0000439_core+TFIIH+complex	0.0663048	#N/A	0.0663048
BP_GO:0019731_antibacterial+humoral+response	0.247596	0.247596	#N/A
CC_GO:0002095_caveolar+macromolecular+signaling+complex	0.365349	0.365349	#N/A
BP_GO:0009956_radial+pattern+formation	0.5262	0.5262	#N/A
MF_GO:0008917_lipopolysaccharide+N-acetylglucosaminyltransferase+activ	0.326085	0.326085	#N/A
CC_GO:0009320_phosphoribosylaminoimidazole+carboxylase+complex	0.0968655	0.0968655	#N/A
MF_GO:0004638_phosphoribosylaminoimidazole+carboxylase+activity	0.0968655	0.0968655	#N/A
MF_GO:0004639_phosphoribosylaminoimidazolesuccinocarboxamide+synth	0.0968655	0.0968655	#N/A
CC_GO:0005850_eukaryotic+translation+initiation+factor+2+complex	0.724725	#N/A	0.724725
CC_GO:0017117_single-stranded+DNA-dependent+ATP-dependent+DNA+he	0.186902	#N/A	0.186902
MF_GO:0017116_single-stranded+DNA-dependent+ATP-dependent+DNA+h	0.186902	#N/A	0.186902
MF_GO:0005138_interleukin-6+receptor+binding	0.548631	0.548631	#N/A
BP_GO:0006055_CMP-N-acetylneuraminate+biosynthetic+process	0.20125	#N/A	0.20125
MF_GO:0008781_N-acylneuraminate+cytidylyltransferase+activity	0.20125	#N/A	0.20125
MF_GO:0008187_poly-pyrimidine+tract+binding	0.746124	0.746124	#N/A
CC_GO:0033588_Elongator+holoenzyme+complex	0.206384	0.206384	#N/A
MF_GO:0004574_oligo-1,6-glucosidase+activity	0.619366	#N/A	0.619366
MF_GO:0016937_short-branched-chain-acyl-CoA+dehydrogenase+activity	0.0656258	0.0656258	#N/A
MF_GO:0003921_GMP+synthase+activity	0.55023	#N/A	0.55023
MF_GO:0003922_GMP+synthase+(glutamine-hydrolyzing)+activity	0.55023	#N/A	0.55023
BP_GO:0006419_alanyl-tRNA+aminoacylation	0.349783	#N/A	0.349783
BP_GO:0034620_cellular+response+to+unfolded+protein	0.349783	#N/A	0.349783
MF_GO:0008470_isovaleryl-CoA+dehydrogenase+activity	0.152229	0.152229	#N/A
BP_GO:0021885_forebrain+cell+migration	0.314419	#N/A	0.314419
BP_GO:0050760_negative+regulation+of+thymidylate+synthase+biosyntheti	0.178151	#N/A	0.178151
BP_GO:0006222_UMP+biosynthetic+process	0.0247901	#N/A	0.0247901

Liver

Genome-scale model(GSM)

		Reporter P-value	
**#Feature**	**No-direction**	**Down regulated**	**Up regulated**
ATP	0.79568	0.795099	0.60653
NADH	0.886683	0.411895	0.982921
NADPH	0.050385	0.0443511	0.322635
Pyrophosphate	0.547332	0.486285	0.591528
ADP	0.748214	0.689696	0.678596
AMP	0.811108	0.777829	0.674495
Oxygen	0.232216	0.511498	0.138818

UDP	0.653506	0.751719	0.434787
NADP+	0.129047	0.209138	0.215033
Orthophosphate	0.317792	0.098859	0.807257
CoA	0.0952951	0.0334656	0.608848
Ubiquinone	0.980005	0.638722	0.986987
Ubiquinol	0.978682	0.638722	0.986117
NAD+	0.782924	0.347532	0.973398
Acetyl-CoA	0.0543946	0.005892	0.766236
Benzo[a]pyrene-4,5-oxide	0.0152579	0.0333638	0.116941
Benzo[a]pyrene-7,8-diol	0.0152579	0.0333638	0.116941
GTP	0.206645	0.67336	0.0486162
CO2	0.47832	0.459296	0.524229
(5Z,8Z,11Z,14Z)-Icosatetraenoic_acid	0.216575	0.420202	0.195505
H2O	0.993856	0.99042	0.842674
UTP	0.149749	0.645891	0.0286595
CTP	0.174355	0.666155	0.0372382
3',5'-Cyclic_GMP	0.137595	0.0902877	0.501745
(1R,2S)-Naphthalene_1,2-oxide	0.0294155	0.124667	0.0636556
(1S,2R)-Naphthalene_1,2-oxide	0.0294155	0.124667	0.0636556
1-Nitronaphthalene-5,6-oxide	0.0250819	0.124667	0.0511602
GDP	0.744594	0.553439	0.800857
UDP-D-galactose	0.389218	0.679522	0.110304
2-Amino-4-hydroxy-6-(erythro-1,2,3-trihydroxypropyl)dihydropteridine_tripł	0.776499	0.566448	0.840519
1-Nitronaphthalene-7,8-oxide	0.0300754	0.124667	0.0632989
3',5'-Cyclic_AMP	0.11809	0.116948	0.353678
2-Amino-4-hydroxy-6-(D-erythro-1,2,3-trihydroxypropyl)-7,8-dihydropteridin	0.756875	0.528504	0.840519
UDP-N-acetyl-D-glucosamine	0.274123	0.423539	0.256028
Trichloroethene	0.0171544	0.124667	0.027161
Bromobenzene-3,4-oxide	0.0027965	0.0280007	0.0218518
Bromobenzene-2,3-oxide	0.0027965	0.0280007	0.0218518
Isomaltose	0.739929	0.46806	0.865099
H+	0.443008	0.127045	0.907122
UDP-N-acetyl-D-galactosamine	0.662463	0.617423	0.616136
Dihydroneopterin_phosphate	0.719058	0.46806	0.849887
Linoleate	0.126171	0.0245314	0.585527
CMP	0.224661	0.401532	0.165355
9(10)-EpOME	0.255399	0.0934751	0.585527
12(13)-EpOME	0.255399	0.0934751	0.585527
11,12-EET	0.0069552	0.0179824	0.0709358
2,2-Dichloroacetaldehyde	0.172391	0.278007	0.213427
1,1-DichloroethylenE_epoxide	0.172391	0.278007	0.213427
Chloroacetyl_chloride	0.172391	0.278007	0.213427
1,2-Dibromoethane	0.0591697	0.0875465	0.213427
2-Bromoacetaldehyde	0.0591697	0.0875465	0.213427
IMP	0.767709	0.74328	0.643683
Palmitoyl-CoA	0.387435	0.18288	0.764883
Ubiquitin	0.738264	0.754607	0.548557

Benzo[a]pyrene-7,8-oxide	0.0873153	0.107861	0.235014
RX	0.0362952	0.0875465	0.117
HX	0.0362952	0.0875465	0.117
7,8-Dihydro-7-hydroxy-8-S-glutathionyl-benzo[a]pyrene	0.0362952	0.0875465	0.117
2-(S-Glutathionyl)acetyl_chloride	0.0362952	0.0875465	0.117
S-(2-Chloroacetyl)glutathione	0.0362952	0.0875465	0.117
2-(S-Glutathionyl)acetyl_glutathione	0.0362952	0.0875465	0.117
S-(1,2-Dichlorovinyl)glutathione	0.0362952	0.0875465	0.117
GlutathionE_episulfonium_ion	0.0362952	0.0875465	0.117
S-(Formylmethyl)glutathione	0.0362952	0.0875465	0.117
Phosphatidylethanolamine	0.914219	0.933468	0.675712
14,15-EET	0.00986319	0.00366293	0.206612
8,9-EET	0.00986319	0.00366293	0.206612
5,6-EET	0.00986319	0.00366293	0.206612
Benzpyrene	0.104603	0.107861	0.283555
Benzo[a]pyrene-9,10-oxide	0.104603	0.107861	0.283555
Benzo[a]pyrene-7,8-dihydrodiol-9,10-oxide	0.104603	0.107861	0.283555
9-Hydroxybenzo[a]pyrene	0.104603	0.107861	0.283555
9-Hydroxybenzo[a]pyrene-4,5-oxide	0.104603	0.107861	0.283555
Pyruvate	0.0609857	0.0843032	0.237539
S-Adenosyl-L-homocysteine	0.907705	0.98454	0.313239
Ferricytochrome_c	0.506135	0.692384	0.324337
1-Organyl-2-lyso-sn-glycero-3-phosphocholine	0.842771	0.948971	0.415602
Methylglyoxal	0.284627	0.267024	0.479317
2-Oxoglutarate	0.899551	0.929986	0.442971
2-Methoxyestradiol-17beta	0.68343	0.859391	0.327289
D-myo-Inositol_1,4,5-trisphosphate	0.130859	0.449063	0.0187538
Phosphatidylcholine	0.93747	0.984001	0.533879
dGTP	0.144979	0.151171	0.350594
3-(2-Aminoethyl)-1H-indol-5-ol	0.723782	0.915854	0.19346
Acetate	0.564187	0.429417	0.689085
S-Adenosyl-L-methionine	0.927574	0.991729	0.237645
1-Phosphatidyl-D-myo-inositol_4,5-bisphosphate	0.375503	0.728332	0.0230097
1-Acyl-sn-glycero-3-phosphocholine	0.914721	0.956026	0.625979
dATP	0.245299	0.151171	0.616759
GMP	0.514927	0.454216	0.583
dTTP	0.106754	0.0445558	0.616759
Trichloroethanol	0.267831	0.653151	0.149889
Testosterone	0.808782	0.835411	0.552643
3'-Phosphoadenylyl_sulfate	0.121808	0.255256	0.166752
3-Hydroxyanthranilate	0.660831	0.817306	0.28721
H2O2	0.876064	0.658164	0.907719
UMP	0.37997	0.40073	0.430619
1-(1-Alkenyl)-sn-glycero-3-phosphoethanolamine	0.791336	0.888417	0.520626
O-1-Alk-1-enyl-2-acyl-sn-glycero-3-phosphoethanolamine	0.860767	0.946108	0.533879
NH3	0.679193	0.75137	0.333355
IDP	0.669569	0.617752	0.629995

Tetrahydrofolate	0.498398	0.424083	0.676438
Succinate	0.814224	0.8008	0.631496
L-Glutamine	0.474658	0.32065	0.67035
CMP-N-acetylneuraminate	0.173367	0.538017	0.0281232
N-Acylsphingosine	0.340265	0.532738	0.184285
1-Acyl-sn-glycero-3-phosphoethanolamine	0.839075	0.889819	0.625979
dCTP	0.0942169	0.0313617	0.616759
L-Histidine	0.648846	0.827773	0.305764
Acetaldehyde	0.829235	0.808693	0.675906
Fatty_acid	0.679933	0.920844	0.151396
L-Glutamate	0.703731	0.578086	0.774401
Naphthyl-2-oxomethyl-succinyl-CoA	0.738509	0.638364	0.787218
FADH2	0.507501	0.390067	0.708261
Lysophospholipid	0.821544	0.889819	0.581087
1-Radyl-2-acyl-sn-glycero-3-phosphocholine	0.821544	0.889819	0.581087
Glutathione	0.957441	0.849835	0.939227
16(R)-HETE	0.0512051	0.0166079	0.368005
(S)-Methylmalonate_semialdehyde	0.857603	0.562017	0.929387
Acceptor	0.00102547	0.000799903	0.311258
(S)-3-Hydroxybutanoyl-CoA	0.273796	0.0627946	0.869572
N-Acetyl-D-mannosamine	0.804313	0.82076	0.573555
Adenosine_3',5'-bisphosphate	0.104324	0.257063	0.140597
ITP	0.405665	0.554831	0.297179
Succinyl-CoA	0.80422	0.891545	0.446632
2,6-Dimethyl-5-methylene-3-oxo-heptanoyl-CoA	0.920063	0.881518	0.787218
N(pi)-Methyl-L-histidine	0.690469	0.869805	0.28721
N-Methyltyramine	0.690469	0.869805	0.28721
Hordenine	0.690469	0.869805	0.28721
2-Hydroxyestradiol-17beta	0.690469	0.869805	0.28721
CH3-R	0.690469	0.869805	0.28721
2-Hydroxypropylphosphonate	0.690469	0.869805	0.28721
Methylcobalamin	0.690469	0.869805	0.28721
1-Phenanthrol	0.690469	0.869805	0.28721
1-Methoxyphenanthrene	0.690469	0.869805	0.28721
Reduced_acceptor	0.000169082	6.97712E-05	0.37314
Octadecanoic_acid	0.67827	0.366248	0.888568
Tetradecanoyl-CoA	0.841336	0.692786	0.862318
Hexadecanoic_acid	0.173519	0.102422	0.606484
Hexanoyl-CoA	0.468084	0.298182	0.825011
beta-D-Fructose	0.819795	0.807155	0.657482
Phosphatidate	0.882705	0.98387	0.279851
sn-Glycerol_3-phosphate	0.248002	0.13118	0.782426
L-Arginine	0.490967	0.123811	0.932054
Glutaryl-CoA	0.831731	0.753311	0.764452
Androst-4-ene-3,17-dione	0.929024	0.841538	0.880224
Dolichyl_phosphate	0.482027	0.8106	0.193666
Estradiol-17beta	0.49548	0.737397	0.257435

Estrone	0.49548	0.737397	0.257435
Indole-3-acetaldehyde	0.88554	0.877262	0.663366
1,2-Dihydronaphthalene-1,2-diol	0.302506	0.616533	0.208368
Naphthalene-1,2-diol	0.302506	0.616533	0.208368
3-Keto-beta-D-galactose	0.491867	0.420087	0.639618
beta-D-Fructose_6-phosphate	0.299107	0.321392	0.40126
beta-Alanine	0.604417	0.575013	0.577038
Adenosine	0.460098	0.204282	0.803339
1,2-Diacyl-sn-glycerol	0.880722	0.904121	0.640756
Decanoyl-CoA	0.807626	0.692786	0.825011
alpha-D-Glucose	0.456048	0.466165	0.469399
Tetradecanoic_acid	0.07545	0.029167	0.606484
FAD	0.755847	0.704519	0.682237
Xanthosine_5'-phosphate	0.660324	0.296344	0.800782
trans-Hex-2-enoyl-CoA	0.0240379	0.00717202	0.683468
Progesterone	0.104172	0.0261493	0.747841
3-Isopropylbut-3-enoyl-CoA	0.661558	0.406443	0.91976
Aldehyde	0.909002	0.886354	0.742483
3alpha,7alpha-Dihydroxy-5beta-cholestan-26-al	0.909002	0.886354	0.742483
3-Chloroallyl_aldehyde	0.909002	0.886354	0.742483
cis-3-Chloroallyl_aldehyde	0.909002	0.886354	0.742483
Ecgonine_methyl_ester	0.33644	0.231408	0.620669
3,4-Dihydroxymandelaldehyde	0.431244	0.374652	0.5389
Parathion	0.577184	0.754315	0.316581
Paraoxon	0.577184	0.754315	0.316581
20-HETE	0.721225	0.979165	0.150197
Naphthalene	0.287419	0.629062	0.208368
1,2-Dihydroxy-3,4-epoxy-1,2,3,4-tetrahydronaphthalene	0.287419	0.629062	0.208368
beta-Naphthol	0.287419	0.629062	0.208368
Trichloroacetate	0.165003	0.515105	0.0901174
UDP-D-galacturonate	0.410478	0.327158	0.639618
dTDP-glucose	0.410478	0.327158	0.639618
dTDP-D-glucuronate	0.410478	0.327158	0.639618
2-Dehydro-3-deoxy-D-xylonate	0.410478	0.327158	0.639618
(4S)-5-Hydroxy-2,4-dioxopentanoate	0.410478	0.327158	0.639618
13(S)-HODE	0.410478	0.327158	0.639618
13-OxoODE	0.410478	0.327158	0.639618
beta-D-Galactose	0.398408	0.314515	0.639618
Ethylene_oxide	0.407894	0.324442	0.639618
4-Aminobutanoate	0.710189	0.625061	0.718327
Acyl-CoA	0.369294	0.734568	0.071717
Octanoyl-CoA	0.854392	0.756927	0.825011
Lauroyl-CoA	0.854392	0.756927	0.825011
Uridine	0.343034	0.260382	0.544606
N-Acetyl-D-mannosamine_6-phosphate	0.735702	0.740175	0.571607
trans-Hexadec-2-enoyl-CoA	0.238074	0.143858	0.683468
Cortisol	0.709717	0.58088	0.747841

L-Proline	0.943495	0.789505	0.968114
17alpha-Hydroxyprogesterone	0.135698	0.0344612	0.747841
5-Hydroxyindoleacetaldehyde	0.935389	0.799301	0.942808
D-Glyceraldehyde	0.672688	0.488627	0.909623
1-Nitronaphthalene	0.251977	0.629062	0.168086
Formaldehyde	0.810778	0.859362	0.518656
Glycine	0.924219	0.93544	0.51616
Nicotinate_d-ribonucleotide	0.172483	0.199766	0.325034
4-Aminobutanal	0.935273	0.944134	0.663366
Imidazole-4-acetaldehyde	0.935273	0.944134	0.663366
beta-Aminopropion_aldehyde	0.935273	0.944134	0.663366
Hydroxyacetone	0.438945	0.350535	0.639618
Sorbitol_6-phosphate	0.438945	0.350535	0.639618
Sorbose_1-phosphate	0.438945	0.350535	0.639618
Butanal	0.438945	0.350535	0.639618
1-Butanol	0.438945	0.350535	0.639618
3alpha,7alpha,24-Trihydroxy-5beta-cholestanoyl-CoA	0.438945	0.350535	0.639618
3alpha,7alpha-Dihydroxy-5beta-24-oxocholestanoyl-CoA	0.438945	0.350535	0.639618
3alpha,7alpha,12alpha,24-Tetrahydroxy-5beta-cholestanoyl-CoA	0.438945	0.350535	0.639618
3alpha,7alpha,12alpha-Trihydroxy-5beta-24-oxocholestanoyl-CoA	0.438945	0.350535	0.639618
1-(4'-Hydroxyphenyl)ethanol	0.438945	0.350535	0.639618
4'-Hydroxyacetophenone	0.438945	0.350535	0.639618
L-Cysteine	0.388204	0.415018	0.422391
Deoxyadenosine	0.321049	0.13971	0.827242
4-Hydroxyphenylacetaldehyde	0.46697	0.525329	0.414051
Phenylacetaldehyde	0.46697	0.525329	0.414051
3,4-Dihydroxyphenylacetaldehyde	0.46697	0.525329	0.414051
Galactosylceramide	0.942349	0.845576	0.885442
Protein_lysine	0.884899	0.942587	0.218487
beta-D-Fructose_2-phosphate	0.78856	0.808818	0.571607
Thiamin	0.78856	0.808818	0.571607
Thiamin_monophosphate	0.78856	0.808818	0.571607
N1-(5-Phospho-alpha-D-ribosyl)-5,6-dimethylbenzimidazole	0.78856	0.808818	0.571607
alpha-Ribazole	0.78856	0.808818	0.571607
5-Amino-6-(5'-phosphoribitylamino)uracil	0.78856	0.808818	0.571607
4-(1-D-Ribitylamino)-5-amino-2,6-dihydroxypyrimidine	0.78856	0.808818	0.571607
2-Acyl-sn-glycerol_3-phosphate	0.359212	0.201368	0.88341
2-Naphthoyl-CoA	0.359212	0.201368	0.88341
3-Hydroxy-5-oxohexanoyl-CoA	0.359212	0.201368	0.88341
2-Naphthoic_acid	0.359212	0.201368	0.88341
trans-Oct-2-enoyl-CoA	0.159544	0.076931	0.683468
2-trans-Dodecenoyl-CoA	0.159544	0.076931	0.683468
trans-Tetradec-2-enoyl-CoA	0.159544	0.076931	0.683468
trans-Dec-2-enoyl-CoA	0.159544	0.076931	0.683468
L-Tyrosine	0.782265	0.797285	0.547099
Benzoyl_acetyl-CoA	0.770032	0.556939	0.890355
S-Benzoate_coenzyme_a	0.770032	0.556939	0.890355

4-(2-Aminoethyl)-1,2-benzenediol	0.637461	0.865077	0.197669
6-Oxo-2-hydroxycyclohexane-1-carboxyl-CoA	0.863042	0.833095	0.696119
Propanoyl-CoA	0.919071	0.928962	0.600261
UDP-D-glucuronate	0.488557	0.343003	0.642003
4-Nitrophenyl_phosphate	0.386341	0.381114	0.4678
4-Nitrophenol	0.386341	0.381114	0.4678
3-Methylcrotonyl-CoA	0.211738	0.134665	0.622719
Pregnenolone	0.0219852	0.0182971	0.342072
17alpha-Hydroxypregnenolone	0.0219852	0.0182971	0.342072
GDP-L-fucose	0.835422	0.769905	0.749025
Glutathione_disulfide	0.984638	0.869874	0.973076
N4-Acetylaminobutanal	0.900372	0.799301	0.909623
Chloral_hydrate	0.494737	0.447179	0.5389
3-Oxotetradecanoyl-CoA	0.244931	0.0543653	0.683113
dADP	0.639824	0.53811	0.697756
dGDP	0.639824	0.53811	0.697756
Nicotinate_d-ribonucleoside	0.271892	0.341025	0.325034
D-Galactose	0.165994	0.198581	0.31154
Deoxyguanosine	0.420393	0.201958	0.827242
Triacylglycerol	0.702929	0.795431	0.44455
Inosine	0.459769	0.236838	0.827242
Octadecanoyl-[acyl-carrier_protein]	0.179979	0.0759349	0.709944
5,10-Methylenetetrahydrofolate	0.492309	0.51493	0.443397
Mercaptopyruvate	0.0965352	0.117663	0.288659
Tyramine	0.622811	0.874939	0.197669
Cytidine	0.640373	0.30937	0.869333
Deoxycytidine	0.640373	0.30937	0.869333
Formate	0.58643	0.656558	0.447524
Sphingosine_1-phosphate	0.692319	0.897271	0.509956
Sphingosine	0.692319	0.897271	0.509956
dTMP	0.553218	0.330419	0.827242
L-Alanine	0.100678	0.101622	0.39633
L-Tryptophan	0.833528	0.969731	0.245504
4-Hydroxyphenylacetyl-CoA	0.720368	0.556939	0.88341
4-Hydroxyphenylacetylglycine	0.720368	0.556939	0.88341
3-Oxopimeloyl-CoA	0.720368	0.556939	0.88341
Phenylacetyl-CoA	0.720368	0.556939	0.88341
Phenylacetylglycine	0.720368	0.556939	0.88341
trans-4-Hydroxy-L-proline	0.95238	0.91948	0.836204
1-Acyl-sn-glycerol_3-phosphate	0.37868	0.705978	0.0759224
11-Deoxycortisol	0.722102	0.579494	0.747841
11-Deoxycorticosterone	0.722102	0.579494	0.747841
17alpha,21-Dihydroxypregnenolone	0.722102	0.579494	0.747841
Dolichyl_phosphate_d-mannose	0.694781	0.896088	0.338191
UDP-glucuronate	0.590569	0.571329	0.560904
Perillic_acid	0.974444	0.937857	0.909623
3-Phospho-D-glycerate	0.46248	0.642083	0.309568

3alpha,7alpha-Dihydroxy-5beta-cholestanate	0.97364	0.936047	0.909623
4-Trimethylammoniobutanoate	0.871359	0.739373	0.909623
D-Glycerate	0.971526	0.931324	0.909623
Methylmalonate	0.974368	0.897875	0.942808
5-Hydroxyindoleacetate	0.974368	0.897875	0.942808
TCE_epoxide	0.12957	0.629062	0.0532069
Chloral	0.12957	0.629062	0.0532069
Ferricytochrome_b5	0.11202	0.0643952	0.662068
(S)-3-Hydroxyhexadecanoyl-CoA	0.282594	0.0476071	0.683113
(S)-3-Hydroxytetradecanoyl-CoA	0.282594	0.0476071	0.683113
Phosphoenolpyruvate	0.267767	0.269448	0.443113
Starch	0.978536	0.907345	0.948558
L-Phenylalanine	0.295878	0.336965	0.369484
Poly-beta-hydroxybutyrate	0.577175	0.465091	0.677525
((R)-3-Hydroxybutanoyl)(n-2)	0.577175	0.465091	0.677525
Ecgonine	0.577175	0.465091	0.677525
Guanosine	0.583354	0.341025	0.827242
N-Ribosylnicotinamide	0.583354	0.341025	0.827242
Xanthosine	0.583354	0.341025	0.827242
Tryptamine	0.350481	0.620691	0.197669
Phenethylamine	0.350481	0.620691	0.197669
Lauric	0.298393	0.100225	0.814379
acid	0.298393	0.100225	0.814379
Thymidine	0.397111	0.161894	0.827242
1-Acylglycerol	0.546914	0.586139	0.458773
Nicotinamide_d-ribonucleotide	0.639212	0.296344	0.850524
CDP	0.533456	0.779353	0.172859
5,10-Methenyltetrahydrofolate	0.744117	0.68419	0.676438
Cholesterol	0.00348994	0.090579	0.00273266
dCMP	0.649751	0.422135	0.827242
L-Methionine	0.698944	0.596308	0.767551
Sphinganine	0.354707	0.734378	0.184285
L-Cysteate	0.140727	0.151353	0.342014
L-Aspartate	0.529043	0.529043	#N/A
2-Aminoacrylate	0.161073	0.177709	0.342014
10-Formyltetrahydrofolate	0.836349	0.809344	0.676438
UDP-glucose	0.2467	0.17127	0.562153
Oxaloacetate	0.743284	0.590301	0.861825
dGMP	0.312683	0.296344	0.438387
1D-myo-Inositol_1,4-bisphosphate	0.372525	0.372525	#N/A
1D-myo-Inositol_1,3,4-trisphosphate	0.372525	0.372525	#N/A
p-Benzoquinone	0.981138	0.989427	0.473708
5(S)-HETE	0.853107	0.582255	0.899065
(15S)-15-Hydroxy-5,8,11-cis-13-trans-eicosatetraenoate	0.853107	0.582255	0.899065
Tetrahydrobiopterin	0.319283	0.220353	0.542691
3beta-Hydroxyandrost-5-en-17-one	0.440813	0.406991	0.564081
16alpha-Hydroxydehydroepiandrosterone	0.440813	0.406991	0.564081

1D-myo-Inositol_1,3,4,5-tetrakisphosphate	0.233946	0.315508	0.241099
Dolichyl_diphosphate	0.564911	0.810283	0.336616
11beta,17alpha,21-Trihydroxypregnenolone	0.825694	0.579494	0.909626
Maleic_acid	0.711091	0.711091	#N/A
Maleamate	0.711091	0.711091	#N/A
N-Cyclohexylformamide	0.711091	0.711091	#N/A
Cyclohexylamine	0.711091	0.711091	#N/A
21-Hydroxypregnenolone	0.471577	0.579494	0.342072
Estrone_glucuronide	0.543653	0.571329	0.497899
Androsterone	0.543653	0.571329	0.497899
Androsterone_glucuronide	0.543653	0.571329	0.497899
Testosterone_glucuronide	0.543653	0.571329	0.497899
Estradiol-17beta_3-glucuronide	0.543653	0.571329	0.497899
3alpha-Hydroxy-5beta-androstan-17-one	0.543653	0.571329	0.497899
Etiocholan-3alpha-ol-17-one_3-glucuronide	0.543653	0.571329	0.497899
2-Methoxyestrone	0.543653	0.571329	0.497899
2-Methoxyestrone_3-glucuronide	0.543653	0.571329	0.497899
2-Methoxy-estradiol-17beta_3-glucuronide	0.543653	0.571329	0.497899
Trichloroethanol_glucuronide	0.543653	0.571329	0.497899
Ethanol	0.447378	0.563585	0.409843
Indole-3-acetate	0.958637	0.897875	0.909623
2-Propyn-1-al	0.958637	0.897875	0.909623
Propynoate	0.958637	0.897875	0.909623
D-Glucuronolactone	0.958637	0.897875	0.909623
D-Glucarate	0.958637	0.897875	0.909623
4-Trimethylammoniobutanal	0.958637	0.897875	0.909623
Imidazole-4-acetate	0.958637	0.897875	0.909623
4-Acetamidobutanoate	0.958637	0.897875	0.909623
3-Butyn-1-al	0.958637	0.897875	0.909623
3-Butynoate	0.958637	0.897875	0.909623
trans-3-Chloroacrylic_acid	0.958637	0.897875	0.909623
cis-3-Chloroacrylic_acid	0.958637	0.897875	0.909623
Perillyl_aldehyde	0.958637	0.897875	0.909623
3alpha,7alpha,26-Trihydroxy-5beta-cholestane	0.406408	0.364208	0.491273
Glycerol	0.0462841	0.0462841	#N/A
Leukotriene_b4	0.933155	0.945765	0.657005
20-OH-Leukotriene_b4	0.933155	0.945765	0.657005
Icosanoic_acid	0.97288	0.964628	0.796377
Docosanoic_acid	0.97288	0.964628	0.796377
Tetracosanoic_acid	0.97288	0.964628	0.796377
Hexacosanoic_acid	0.97288	0.964628	0.796377
19(S)-HETE	0.880419	0.408851	0.923814
D-Sorbitol	0.0976106	0.0656491	0.421201
Glycerone_phosphate	0.628711	0.659759	0.464493
Uracil	0.125555	0.336544	0.0427394
Bromobenzene	0.00649288	0.0101236	0.0532069
4-Bromophenol	0.00649288	0.0101236	0.0532069

4-Bromophenol-2,3-epoxide	0.00649288	0.0101236	0.0532069
Dihydroceramide	0.136612	0.29469	0.0856105
3-Methoxy-4-hydroxyphenylacetaldehyde	0.387105	0.330953	0.592139
3-Methoxy-4-hydroxyphenylglycolaldehyde	0.387105	0.330953	0.592139
Methylimidazole_acetaldehyde	0.387105	0.330953	0.592139
Estriol	0.450035	0.749561	0.153296
16alpha-Hydroxyestrone	0.450035	0.749561	0.153296
Stearoyl-CoA	0.241714	0.0875313	0.709944
4-Sulfolactone	0.584098	0.45929	0.677525
HSO3-	0.584098	0.45929	0.677525
Crotonoyl-CoA	0.00622651	0.000439062	0.549773
(S)-Malate	0.205216	0.205216	#N/A
Butanoic_acid	0.438423	0.589161	0.212968
Hexanoic_acid	0.438423	0.589161	0.212968
Octanoic_acid	0.438423	0.589161	0.212968
Decanoic_acid	0.438423	0.589161	0.212968
(S)-3-Hydroxyisobutyrate	0.373818	0.104931	0.728761
3-Oxopalmitoyl-CoA	0.301274	0.0172412	0.683113
(S)-3-Hydroxydodecanoyl-CoA	0.301274	0.0172412	0.683113
3-Oxododecanoyl-CoA	0.301274	0.0172412	0.683113
(S)-Hydroxydecanoyl-CoA	0.301274	0.0172412	0.683113
3-Oxodecanoyl-CoA	0.301274	0.0172412	0.683113
(S)-Hydroxyoctanoyl-CoA	0.301274	0.0172412	0.683113
3-Oxooctanoyl-CoA	0.301274	0.0172412	0.683113
(S)-Hydroxyhexanoyl-CoA	0.301274	0.0172412	0.683113
3-Oxohexanoyl-CoA	0.301274	0.0172412	0.683113
Putrescine	0.429669	0.801947	0.178334
Guanine	0.24431	0.275646	0.357737
dAMP	0.572396	0.296344	0.827242
Corticosterone	0.573794	0.369042	0.759102
Aminoacetone	0.486233	0.620691	0.333355
Melatonin	0.907372	0.885892	0.731639
Oleoyl-[acyl-carrier_protein]	0.0759349	0.0759349	#N/A
Dihydrobiopterin	0.145364	0.220353	0.234178
Malonyl-CoA	0.00160798	0.000718473	0.415956
(S)-1-Phenylethanol	0.931242	0.954786	0.473708
e-	0.931242	0.954786	0.473708
2,6-Dihydroxycyclohexane-1-carboxyl-CoA	0.931242	0.954786	0.473708
2-Hydroxy-4-isopropenylcyclohexane-1-carboxyl-CoA	0.931242	0.954786	0.473708
4-Isopropenyl-2-oxy-cyclohexanecarboxyl-CoA	0.931242	0.954786	0.473708
Myrtenol	0.931242	0.954786	0.473708
Myrtenal	0.931242	0.954786	0.473708
Pinocarvone	0.931242	0.954786	0.473708
3-Hydroxy-2,6-dimethyl-5-methylene-heptanoyl-CoA	0.931242	0.954786	0.473708
4,4'-Dihydroxy-alpha-methylstilbene	0.931242	0.954786	0.473708
9,10-Dihydroxyanthracene	0.931242	0.954786	0.473708
2-Acetyl-1-alkyl-sn-glycero-3-phosphocholine	0.663596	0.860116	0.11199

Sphinganine_1-phosphate	0.406172	0.897271	0.184285
Ceramide_1-phosphate	0.406172	0.897271	0.184285
dUDP	0.293404	0.130446	0.697756
dCDP	0.293404	0.130446	0.697756
Adenine	0.547865	0.50656	0.593212
G12336	0.790786	0.678066	0.747494
Fe3+	0.562128	0.325949	0.787985
Lauric_acid	0.207738	0.304459	0.212968
2-Methylprop-2-enoyl-CoA	0.0239385	0.0153718	0.459325
2-Methylbut-2-enoyl-CoA	0.0239385	0.0153718	0.459325
1H-Imidazole-4-ethanamine	0.385846	0.71982	0.197669
FMN	0.236141	0.259285	0.376257
Riboflavin	0.236141	0.259285	0.376257
4-Methyl-2-oxopentanoate	0.628889	0.689671	0.465653
3-Methyl-2-oxobutanoic_acid	0.628889	0.689671	0.465653
Protein_N6-methyl-L-lysine	0.820685	0.921511	0.218487
2-Oxobutanoate	0.0724587	0.018988	0.426188
Protein_asparagine	0.489823	0.783937	0.336616
Androst-5-ene-3beta,17beta-diol	0.599347	0.579494	0.564081
16alpha-Hydroxyandrost-4-ene-3,17-dione	0.599347	0.579494	0.564081
Sphingomyelin	0.512788	0.512788	#N/A
L-Serine	0.317176	0.0480941	0.900687
Oxidized_thioredoxin	0.782643	0.354448	0.95461
Thioredoxin	0.782643	0.354448	0.95461
Butanoyl-CoA	0.285839	0.285839	#N/A
Amylose	0.840481	0.857186	0.556827
Retinal	0.526542	0.25734	0.89627
Phenylpyruvate	0.384403	0.384403	#N/A
3-(4-Hydroxyphenyl)pyruvate	0.384403	0.384403	#N/A
L-Kynurenine	0.876976	0.958569	0.247068
3-Hydroxy-L-kynurenine	0.876976	0.958569	0.247068
Primary_alcohol	0.524371	0.563585	0.491273
3,4-Dihydroxyphenylethyleneglycol	0.524371	0.563585	0.491273
1-Hydroxymethylnaphthalene	0.524371	0.563585	0.491273
1-Naphthaldehyde	0.524371	0.563585	0.491273
(2-Naphthyl)methanol	0.524371	0.563585	0.491273
2-Naphthaldehyde	0.524371	0.563585	0.491273
Acetoacetate	0.158981	0.158981	#N/A
Isocitrate	0.872936	0.909567	0.485119
Cyromazine	0.381632	0.640627	0.110335
N-Cyclopropylammeline	0.381632	0.640627	0.110335
N-Cyclopropylammelide	0.381632	0.640627	0.110335
Cyanuric_acid	0.381632	0.640627	0.110335
Glycolate	0.823052	0.823052	#N/A
3-Oxostearoyl-[acp]	0.213422	0.213422	#N/A
Xanthine	0.504765	0.648576	0.357737
L-Citrulline	0.323926	0.323926	#N/A

5-Methyl-3-oxo-4-hexenoyl-CoA	0.78812	#N/A	0.78812
Prostaglandin_F2alpha	0.493073	0.493073	#N/A
Prostaglandin_E2	0.493073	0.493073	#N/A
D-Glucono-1,5-lactone_6-phosphate	0.428901	0.428901	#N/A
Acetoacetyl-CoA	0.29774	0.0172412	0.728761
(2S,3S)-3-Hydroxy-2-methylbutanoyl-CoA	0.29774	0.0172412	0.728761
2-Methylacetoacetyl-CoA	0.29774	0.0172412	0.728761
(3S)-3-Hydroxyadipyl-CoA	0.29774	0.0172412	0.728761
3-Oxoadipyl-CoA	0.29774	0.0172412	0.728761
Hypoxanthine	0.446146	0.38494	0.593212
Xylitol	0.0883721	0.0656491	0.465269
GM2	0.744583	0.720237	0.619372
G06780	0.744583	0.720237	0.619372
Glyoxylate	0.937742	0.937742	#N/A
myo-Inositol_hexakisphosphate	0.612645	0.615693	0.543964
1D-myo-Inositol_1,3,4,5,6-pentakisphosphate	0.612645	0.615693	0.543964
Biliverdin	0.509496	0.593903	0.419228
Glucosylceramide	0.972266	0.814781	0.988579
15H-11,12-EETA	0.929525	0.408851	0.966589
Ferrocytochrome_b5	0.202846	0.202846	#N/A
Nicotinamide	0.680485	0.542772	0.684196
p-Benzenediol	0.929999	0.905013	0.751797
L-erythro-4-Hydroxyglutamate	0.732787	0.732787	#N/A
G00011	0.798865	0.568772	0.843903
G00012	0.798865	0.568772	0.843903
1-Phosphatidyl-1D-myo-inositol_4-phosphate	0.77857	0.77857	#N/A
Selenomethionine	0.662676	0.524848	0.767551
3-Phospho-D-glyceroyl_phosphate	0.0745416	0.112636	0.204271
Deoxyuridine	0.421442	0.280431	0.729234
Deamino-NAD+	0.181985	0.181985	#N/A
Methanol	0.360506	0.383951	0.419733
Dodecanoic_acid	0.188216	0.287432	0.212968
(S)-3-Methyl-2-oxopentanoic_acid	0.805706	0.90403	0.465653
G13043	0.619711	0.678066	0.487421
Hexadecanoyl-[acp]	0.421889	0.421889	#N/A
Acyl-carrier_protein	0.421889	0.421889	#N/A
cis-2-Methyl-5-isopropylhexa-2,5-dienoyl-CoA	0.127602	0.00494079	0.834449
trans-2-Methyl-5-isopropylhexa-2,5-dienoyl-CoA	0.127602	0.00494079	0.834449
L-Isoleucine	0.800982	0.899862	0.465653
D-Sedoheptulose_7-phosphate	0.486752	0.419633	0.612076
D-Erythrose_4-phosphate	0.486752	0.419633	0.612076
GM4	0.875687	0.919788	0.698723
(S)-Lactate	0.0312609	0.018988	0.288659
2-Hydroxybutanoic_acid	0.0312609	0.018988	0.288659
3-Mercaptolactate	0.0312609	0.018988	0.288659
L-Ornithine	0.37392	0.138713	0.892017
Octanoyl-[acp]	0.240077	0.413599	0.150903

L-1-Pyrroline-3-hydroxy-5-carboxylate	0.896683	0.813853	0.836204
Cys-Gly	0.728316	0.681155	0.651026
Peptide	0.641466	0.463021	0.811818
5-Hydroxy-L-tryptophan	0.699749	0.928592	0.234178
1D-myo-Inositol_3-phosphate	0.930707	0.831823	0.902358
myo-Inositol	0.319939	0.398966	0.31154
dTDP	0.239509	0.406055	0.158017
dUMP	0.543188	0.766874	0.282577
dUTP	0.108583	0.539195	0.0326968
Diacylglycerol	0.251092	0.464975	0.11736
Cocaine	0.157299	0.125781	0.452431
3-Oxo-(7Z,10Z,13Z,16Z)-Docosatetraenoyl-CoA	7.45852E-07	7.45852E-07	#N/A
3-Oxo-(8Z,11Z,14Z)-Icosatrienoyl-CoA	7.45852E-07	7.45852E-07	#N/A
3-Oxo-(7Z,10Z,13Z,16Z,19Z)-Docosapentaenoyl-CoA	7.45852E-07	7.45852E-07	#N/A
Retinoate	0.380155	0.102875	0.89627
2,3-Bisphospho-D-glycerate	0.681986	0.907557	0.359947
D-Tagatose_6-phosphate	0.863777	0.870032	0.620544
D-Tagatose_1,6-bisphosphate	0.863777	0.870032	0.620544
11H-14,15-EETA	0.870977	0.408851	0.939175
3-Oxoicosanoyl-CoA	0.856455	0.856455	#N/A
3-Oxodocosanoyl-CoA	0.856455	0.856455	#N/A
3-Oxotetracosanoyl-CoA	0.856455	0.856455	#N/A
3-Oxostearoyl-CoA	0.0772442	0.0772442	#N/A
Sarcosine	0.956268	0.980476	0.51616
S-Adenosylmethioninamine	0.465507	0.727321	0.296744
omega-Hydroxy_fatty_acid	0.215329	0.901705	0.0301611
Fumarate	0.69463	0.509611	0.80146
11-Dehydrocorticosterone	0.369042	0.369042	#N/A
(2E)-Octadecenoyl-CoA	0.313766	#N/A	0.313766
(2E)-Octadecenoyl-[acp]	0.313766	#N/A	0.313766
Diethylthiophosphoric_acid	0.752798	0.638869	0.751797
Diethylphosphoric_acid	0.752798	0.638869	0.751797
4-Hydroxyphenyl_acetate	0.752798	0.638869	0.751797
Cholesta-5,7-dien-3beta-ol	0.0368431	0.0368431	#N/A
Leukotriene_a4	0.757972	0.757972	#N/A
5-Hydroxykynurenamine	0.218368	0.366101	0.194338
14-Demethyllanosterol	0.0333241	0.0222876	0.368967
Reduced_flavoprotein	0.695904	0.505605	0.731639
6-Hydroxymelatonin	0.695904	0.505605	0.731639
Oxidized_flavoprotein	0.695904	0.505605	0.731639
OPC6-CoA	0.767644	0.695053	0.706805
OPC4-CoA	0.767644	0.695053	0.706805
Choline	0.317778	0.261933	0.532237
N-Acetyl-D-galactosamine	0.68271	0.643192	0.619372
Phenol	0.56904	0.615693	0.45433
Phenolic_phosphate	0.56904	0.615693	0.45433
7alpha,12alpha-Dihydroxycholest-4-en-3-one	0.849013	0.849013	#N/A

5'-Methylthioadenosine	0.48188	0.881119	0.0403061
Procollagen_5-hydroxy-L-lysine	0.78029	0.78029	#N/A
G13032	0.440312	0.603148	0.264553
(S)-3-Hydroxy-3-methylglutaryl-CoA	0.0915674	0.0450534	0.536676
Galactitol	0.0656491	0.0656491	#N/A
D-Xylose	0.0656491	0.0656491	#N/A
L-Arabitol	0.0656491	0.0656491	#N/A
L-Arabinose	0.0656491	0.0656491	#N/A
Lactaldehyde	0.0656491	0.0656491	#N/A
Propane-1,2-diol	0.0656491	0.0656491	#N/A
beta-D-Galactosyl-1,4-beta-D-glucosylceramide	0.936852	0.603148	0.988579
Octadecanoyl-CoA	0.937343	0.930007	0.714518
Tetracosanoyl-CoA	0.937343	0.930007	0.714518
Se-Adenosylselenohomocysteine	0.488658	0.336828	0.60381
Deoxyinosine	0.160184	0.160184	#N/A
3-beta-D-Galactosyl-sn-glycerol	0.4719	0.603148	0.31154
L-Leucine	0.346399	0.287165	0.465653
Oleoyl-CoA	0.0875313	0.0875313	#N/A
Phenylacetic_acid	0.4639	0.391366	0.592139
4-Hydroxyphenylacetate	0.4639	0.391366	0.592139
3,4-Dihydroxyphenylacetate	0.4639	0.391366	0.592139
3,4-Dihydroxymandelate	0.4639	0.391366	0.592139
Homovanillate	0.4639	0.391366	0.592139
3-Methoxy-4-hydroxymandelate	0.4639	0.391366	0.592139
Methylimidazoleacetic_acid	0.4639	0.391366	0.592139
L-Asparagine	0.0858659	0.156342	0.174165
3-Hydroxy-5-methylhex-4-enoyl-CoA	0.728761	#N/A	0.728761
N-(omega)-Hydroxyarginine	0.529623	0.529623	#N/A
Nitric_oxide	0.529623	0.529623	#N/A
UDP-D-xylose	0.912787	0.536118	0.98686
L-Valine	0.406863	0.387518	0.465653
3-Dehydrosphinganine	0.386168	0.386168	#N/A
11beta-Hydroxyandrost-4-ene-3,17-dione	0.85475	0.527834	0.958228
3-Methyl-2-oxopentanoate	0.260671	0.16164	0.465653
beta-D-Fructose_2,6-bisphosphate	0.513567	0.689063	0.261699
D-Glucosamine_6-phosphate	0.291777	0.291777	#N/A
(2R)-2-Hydroxy-3-(phosphonooxy)-propanal	0.324674	0.50794	0.207594
Propenoyl-CoA	0.0151428	0.00494079	0.459325
5-Carboxy-2-pentenoyl-CoA	0.0151428	0.00494079	0.459325
L-Noradrenaline	0.4273	0.4273	#N/A
Sulfate	0.625701	0.625701	#N/A
2-Acyl-1-alkyl-sn-glycero-3-phosphate	0.909281	0.897271	0.77045
1-Alkyl-2-acylglycerol	0.909281	0.897271	0.77045
2-Oxoadipate	0.983091	0.983091	#N/A
Ethanolamine	0.709343	0.88429	0.431926
trans,trans-Farnesyl_diphosphate	0.483558	0.483558	#N/A
Pantetheine_4'-phosphate	0.653007	0.414314	0.838145

DWA-2	0.651073	0.678066	0.507369
sn-glycero-3-Phosphocholine	0.848642	0.838944	0.649542
Citrate	0.0122185	0.0112256	0.252249
Selenide	0.0717898	0.113014	0.206198
Fe2+	0.503106	0.132137	0.787985
L-Glutamate_5-semialdehyde	0.513484	0.513484	#N/A
Alcohol	0.429559	0.432272	0.473714
(2E,6Z,9Z,12Z,15Z,18Z,21Z)-Tetracosahepta-2,6,9,12,15,18,21-enoyl-CoA	0.639703	0.695053	0.459325
(2E,6Z,9Z,12Z,15Z,18Z)-Tetracosahexa-2,6,9,12,15,18-enoyl-CoA	0.639703	0.695053	0.459325
1,3-Diaminopropane	0.592909	0.845064	0.333355
N-Methylputrescine	0.592909	0.845064	0.333355
1-Methylpyrrolinium	0.592909	0.845064	0.333355
Cadaverine	0.592909	0.845064	0.333355
5-Aminopentanal	0.592909	0.845064	0.333355
G00144	0.516535	0.597958	0.39021
2-Oxoarginine	0.623153	0.217678	0.950073
D-Ribose_5-phosphate	0.33791	0.23781	0.612076
Aminofructose_6-phosphate	0.33791	0.23781	0.612076
Iminoerythrose_4-phosphate	0.33791	0.23781	0.612076
Dodecanoyl-[acyl-carrier_protein]	0.505435	0.505435	#N/A
Tetradecanoyl-[acp]	0.505435	0.505435	#N/A
11beta-Hydroxyprogesterone	0.013711	2.43045E-06	0.759102
21-Deoxycortisol	0.013711	2.43045E-06	0.759102
(S)-1-Pyrroline-5-carboxylate	0.817511	0.661493	0.836204
D-myo-Inositol_3,4-bisphosphate	0.979359	0.943196	0.902358
Pyridoxal	0.965209	0.92391	0.8866
3-Isopropylbut-3-enoic_acid	0.447809	0.235592	0.785666
5-Methylhex-4-enoyl-CoA	0.366852	0.211451	0.706805
Taurine	0.560006	0.465075	0.597522
Hypotaurine	0.560006	0.465075	0.597522
(5-L-Glutamyl)-peptide	0.814823	0.681155	0.811818
Leukotriene_c4	0.814823	0.681155	0.811818
Leukotriene_d4	0.814823	0.681155	0.811818
R-S-Glutathione	0.814823	0.681155	0.811818
R-S-Alanylglycine	0.814823	0.681155	0.811818
3-Cyano-L-alanine	0.814823	0.681155	0.811818
gamma-Glutamyl-beta-cyanoalanine	0.814823	0.681155	0.811818
gamma-Glutamyl-beta-aminopropiononitrile	0.814823	0.681155	0.811818
2-Methylpropanoyl-CoA	0.169154	0.169154	#N/A
(S)-2-Methylbutanoyl-CoA	0.169154	0.169154	#N/A
Dolichyl_d-glucosyl_phosphate	0.129737	0.251142	0.182512
Protein_N6-(lipoyl)lysine	0.397736	0.660302	0.150903
myo-Inositol_4-phosphate	0.495841	0.495841	#N/A
N6-D-Biotinyl-L-lysine	0.255753	0.501017	0.210268
Dextrin	0.97334	0.787691	0.964143
3-Methylbutanoyl-CoA	0.406436	0.406436	#N/A
N6,N6,N6-Trimethyl-L-lysine	0.329186	0.645586	0.210268

Protein_N6,N6,N6-trimethyl-L-lysine	0.329186	0.645586	0.210268
beta-D-Fructose_1,6-bisphosphate	0.723489	0.723489	#N/A
beta-D-Glucose_6-phosphate	0.255918	0.255918	#N/A
Homogentisate	0.0743135	0.0743135	#N/A
3-Oxo-(8Z,11Z,14Z,17Z)-Icosatetraenoyl-CoA	0.00109241	0.00109241	#N/A
3-Oxo-(11Z,14Z,17Z)-Icosatrienoyl-CoA	0.00109241	0.00109241	#N/A
(5Z,8Z,11Z,14Z)-Icosatetraenoyl-CoA	3.17117E-09	3.17117E-09	#N/A
(5Z,8Z,11Z,14Z,17Z)-Icosapentaenoyl-CoA	3.17117E-09	3.17117E-09	#N/A
3-Phosphonooxypyruvate	0.768633	0.768633	#N/A
1,1-Dichloroethylene	0.995459	0.997343	0.815988
Nucleoside_triphosphate	0.93671	0.93671	#N/A
NDP	0.93671	0.93671	#N/A
(9Z,12Z,15Z)-Octadecatrienoyl-CoA	9.94312E-07	9.94312E-07	#N/A
3-Oxohexadecanoyl-CoA	0.320018	0.320018	#N/A
3-Hydroxyoctadecanoyl-CoA	0.0738016	0.0738016	#N/A
3-Hydroxyoctadecanoyl-[acp]	0.0738016	0.0738016	#N/A
5alpha-Cholest-7-en-3beta-ol	0.0935189	0.0935189	#N/A
Urate	0.120726	0.120726	#N/A
Urocanate	0.363198	0.363198	#N/A
(R)-3-Hydroxybutanoate	0.188318	0.188318	#N/A
S-Aminomethyldihydrolipoylprotein	0.725436	0.725436	#N/A
Bilirubin	0.419228	#N/A	0.419228
Formylanthranilate	0.73401	0.73401	#N/A
L-Formylkynurenine	0.73401	0.73401	#N/A
D-Glutamine	0.0824961	0.060439	0.340447
D-Glutamate	0.0824961	0.060439	0.340447
Agmatine	0.426865	0.426865	#N/A
L-Cystathionine	0.352293	0.352293	#N/A
Selenocystathionine	0.352293	0.352293	#N/A
Oxalosuccinate	0.755966	0.755966	#N/A
Oxooctadecanoyl-CoA	0.504931	0.504931	#N/A
L-Lysine	0.774675	0.774675	#N/A
Selenocysteine	0.149598	0.149598	#N/A
5,6-Dihydrouracil	0.241545	0.241545	#N/A
5,6-Dihydrothymine	0.241545	0.241545	#N/A
1,7-Dimethylxanthine	0.683509	0.667067	0.596068
Vitamin_K	0.273391	0.488759	0.205136
Icosanoyl-CoA	0.930007	0.930007	#N/A
Dcosanoyl-CoA	0.930007	0.930007	#N/A
3-Oxohexacosanoyl-CoA	0.930007	0.930007	#N/A
Hydroxypyruvate	0.269445	0.269445	#N/A
Thymine	0.359032	0.359032	#N/A
Phytoceramide	0.161468	0.161468	#N/A
L-Adrenaline	0.366101	0.366101	#N/A
Methylamine	0.366101	0.366101	#N/A
N-Acetylputrescine	0.366101	0.366101	#N/A
N-Methylhistamine	0.366101	0.366101	#N/A

3-Methoxytyramine	0.366101	0.366101	#N/A
L-Normetanephrine	0.366101	0.366101	#N/A
L-Metanephrine	0.366101	0.366101	#N/A
3-Hydroxykynurenamine	0.366101	0.366101	#N/A
4,8-Dihydroxyquinoline	0.366101	0.366101	#N/A
4,6-Dihydroxyquinoline	0.366101	0.366101	#N/A
Chitobiose	0.747908	0.739	0.619372
GM3	0.747908	0.739	0.619372
G13033	0.747908	0.739	0.619372
G08421	0.747908	0.739	0.619372
(7Z,10Z,13Z,16Z)-Docosatetraenoyl-CoA	0.00339566	1.81796E-05	0.714518
(7Z,10Z,13Z,16Z,19Z)-Docosapentaenoyl-CoA	0.00339566	1.81796E-05	0.714518
Carbamoyl_phosphate	0.667984	0.127354	0.960197
3-Sulfopyruvate	0.604368	0.604368	#N/A
3-Sulfino-L-alanine	0.604368	0.604368	#N/A
3-Sulfinylpyruvate	0.604368	0.604368	#N/A
D-4-Hydroxy-2-oxoglutarate	0.604368	0.604368	#N/A
Cortisone	0.527834	0.527834	#N/A
Adrenosterone	0.527834	0.527834	#N/A
Tetrahydrocorticosterone	0.527834	0.527834	#N/A
3alpha,21-Dihydroxy-5beta-pregnane-11,20-dione	0.527834	0.527834	#N/A
Lanosterol	0.212576	0.212576	#N/A
Digalactosylceramide	0.89663	#N/A	0.89663
Ethanolamine_phosphate	0.48943	0.452929	0.532237
Dimethylallyl_diphosphate	0.0467073	0.0467073	#N/A
Geranyl_diphosphate	0.0467073	0.0467073	#N/A
Pseudoecgonine	0.95335	0.814924	0.930009
Pseudoecgonyl-CoA	0.95335	0.814924	0.930009
4-(2-Aminophenyl)-2,4-dioxobutanoate	0.925358	0.925358	#N/A
4-(2-Amino-3-hydroxyphenyl)-2,4-dioxobutanoate	0.925358	0.925358	#N/A
cis-Aconitate	0.404159	0.721734	0.17598
alpha-L-Fucoside	0.432272	0.432272	#N/A
4,4-Dimethyl-5alpha-cholesta-8,14,24-trien-3beta-ol	0.0356526	0.0356526	#N/A
4alpha-Methyl-5alpha-ergosta-8,14,24(28)-trien-3beta-ol	0.0356526	0.0356526	#N/A
4alpha-Methylzymosterol-4-carboxylate	0.0424427	0.0178389	0.368967
Galactan	0.603148	0.603148	#N/A
Lactose	0.603148	0.603148	#N/A
Lactose_6-phosphate	0.603148	0.603148	#N/A
Galactose	0.603148	0.603148	#N/A
3-Ketolactose	0.603148	0.603148	#N/A
GM1	0.603148	0.603148	#N/A
N-Acetylneuraminate	0.58678	0.58678	#N/A
Protoporphyrinogen_IX	0.516895	0.621185	0.401821
D-Glucuronate	0.367351	0.367351	#N/A
O-Phospho-L-serine	0.310002	0.310002	#N/A
Acetyl_phosphate	0.114786	0.0893435	0.361965
Benzoyl_phosphate	0.114786	0.0893435	0.361965

Benzoate	0.114786	0.0893435	0.361965
G13035	0.482553	0.103844	0.88458
G13042	0.482553	0.103844	0.88458
CDP-choline	0.359895	0.500995	0.30522
Dihydrolipoylprotein	0.820569	0.820569	#N/A
trans-2,3-didehydroacyl-CoA	0.068506	0.0157016	0.520066
trans,trans-2,3,4,5-tetradehydroacyl-CoA	0.068506	0.0157016	0.520066
G00013	0.908602	0.908602	#N/A
G00014	0.908602	0.908602	#N/A
Acetyl-[acyl-carrier_protein]	0.256126	0.256126	#N/A
Acetoacetyl-[acp]	0.256126	0.256126	#N/A
Butyryl-[acp]	0.256126	0.256126	#N/A
3-Oxodecanoyl-[acp]	0.256126	0.256126	#N/A
3-Oxooctanoyl-[acp]	0.256126	0.256126	#N/A
3-Oxohexadecanoyl-[acp]	0.256126	0.256126	#N/A
3-Oxotetradecanoyl-[acp]	0.256126	0.256126	#N/A
3-Oxohexanoyl-[acp]	0.256126	0.256126	#N/A
Hexanoyl-[acp]	0.256126	0.256126	#N/A
Decanoyl-[acp]	0.256126	0.256126	#N/A
3-Oxododecanoyl-[acp]	0.256126	0.256126	#N/A
N,N-Dimethylglycine	0.960443	0.960443	#N/A
OPC8-CoA	0.695053	0.695053	#N/A
trans-2-Enoyl-OPC8-CoA	0.695053	0.695053	#N/A
trans-2-Enoyl-OPC6-CoA	0.695053	0.695053	#N/A
trans-2-Enoyl-OPC4-CoA	0.695053	0.695053	#N/A
(6Z,9Z,12Z,15Z,18Z,21Z)-Tetracosahexaenoyl-CoA	0.695053	0.695053	#N/A
(6Z,9Z,12Z,15Z,18Z)-Tetracosapentaenoyl-CoA	0.695053	0.695053	#N/A
Dihydrofolate	0.392526	0.120198	0.784825
Succinate_semialdehyde	0.332625	0.332625	#N/A
Holo-[carboxylase]	0.00167435	0.00167435	#N/A
Melibiose	0.410299	0.56791	0.31154
Cholesterol_ester	0.497882	0.487976	0.509063
L-2-Aminoadipate_6-semialdehyde	0.377829	0.377829	#N/A
Coproporphyrinogen_III	0.852881	0.852881	#N/A
(R)-S-Lactoylglutathione	0.549185	0.549185	#N/A
Acetyl_adenylate	0.0117801	0.000529408	0.52962
Propinol_adenylate	0.0117801	0.000529408	0.52962
3-Oxopropanoate	0.142221	0.142221	#N/A
Dephospho-CoA	0.414314	0.414314	#N/A
5alpha-Androstane-3,17-dione	0.959331	0.959331	#N/A
17beta-Hydroxyandrostan-3-one	0.959331	0.959331	#N/A
7alpha,12alpha-Dihydroxy-5alpha-cholestan-3-one	0.959331	0.959331	#N/A
Porphobilinogen	0.367098	0.0534875	0.871207
L-4-Hydroxyglutamate_semialdehyde	0.730222	0.730222	#N/A
N-Acetyl-L-aspartate	0.475792	#N/A	0.475792
N-Formyl-L-aspartate	0.475792	#N/A	0.475792
Protoporphyrin	0.144379	0.105532	0.401821

Hemoglobin	0.593903	0.593903	#N/A
Globin	0.593903	0.593903	#N/A
CO	0.593903	0.593903	#N/A
3-Keto-4-methylzymosterol	0.0947502	0.0947502	#N/A
5-Methyltetrahydrofolate	0.035371	0.035371	#N/A
5-Aminolevulinate	0.289796	#N/A	0.289796
Squalene	0.0571958	0.0571958	#N/A
ADP-ribose	0.384096	0.40209	0.433073
L-2-Aminoadipate	0.936395	0.936395	#N/A
Heparan_sulfate	0.922075	0.922075	#N/A
2-Acyl-1-(1-alkenyl)-sn-glycero-3-phosphate	0.731713	0.88429	0.373285
GDP-D-mannose	0.796669	0.75926	0.680444
2-Hexaprenyl-6-methoxy-1,4-benzoquinone	0.132535	0.132535	#N/A
2-Hexaprenyl-3-methyl-6-methoxy-1,4-benzoquinone	0.253384	0.580097	0.127067
2-Hexaprenyl-3-methyl-5-hydroxy-6-methoxy-1,4-benzoquinone	0.284097	#N/A	0.284097
GDP-4-dehydro-6-deoxy-D-mannose	0.82696	0.96661	0.308056
20alpha-Hydroxycholesterol	4.80119E-05	4.80119E-05	#N/A
17alpha,20alpha-Dihydroxycholesterol	4.80119E-05	4.80119E-05	#N/A
5-Phosphoribosylamine	0.721439	#N/A	0.721439
Selenophosphate	0.15072	0.260847	0.206198
S-Glutaryldihydrolipoamide	0.868758	0.868758	#N/A
3-Carboxy-1-hydroxypropyl-ThPP	0.868758	0.868758	#N/A
[Dihydrolipoyllysine-residue_succinyltransferase]_S-succinyldihydrolipoyllysi	0.868758	0.868758	#N/A
CMP-2-aminoethylphosphonate	0.96909	0.578224	0.992703
Ceramide_2-aminoethylphosphonate	0.96909	0.578224	0.992703
Phosphatidylserine	0.96909	0.578224	0.992703
5'-Phosphoribosyl-N-formylglycinamide	0.635284	0.381901	0.785069
Prostaglandin_H2	0.342179	0.624027	0.186583
Pyridoxal_phosphate	0.944069	0.92391	0.792547
Pyridoxine	0.944069	0.92391	0.792547
Pyridoxine_phosphate	0.944069	0.92391	0.792547
Pyridoxamine	0.944069	0.92391	0.792547
Pyridoxamine_phosphate	0.944069	0.92391	0.792547
2-Methyl-1-hydroxypropyl-ThPP	0.90403	0.90403	#N/A
[Dihydrolipoyllysine-residue_(2-methylpropanoyl)transferase]_S-(2-methylpr	0.90403	0.90403	#N/A
3-Methyl-1-hydroxybutyl-ThPP	0.90403	0.90403	#N/A
[Dihydrolipoyllysine-residue_(2-methylpropanoyl)transferase]_S-(3-methylbu	0.90403	0.90403	#N/A
2-Methyl-1-hydroxybutyl-ThPP	0.90403	0.90403	#N/A
[Dihydrolipoyllysine-residue_(2-methylpropanoyl)transferase]_S-(2-methylbu	0.90403	0.90403	#N/A
tRNA(Glu)	0.812483	0.812483	#N/A
L-Glutamyl-tRNA(Glu)	0.812483	0.812483	#N/A
Protein_N6-(octanoyl)lysine	0.365989	0.708353	0.150903
Lipoyl-[acp]	0.365989	0.708353	0.150903
GDP-mannose	0.346879	#N/A	0.346879
Chitin	0.28398	0.29518	0.394073
N-Acetyl-D-glucosamine	0.28398	0.29518	0.394073
N-Acetyl-D-glucosaminide	0.28398	0.29518	0.394073

6-Pyruvoyltetrahydropterin	0.832388	0.560221	0.886963
1L-myo-Inositol_1-phosphate	0.41726	0.41726	#N/A
L-Threonine	0.94746	0.94746	#N/A
XTP	0.0644278	0.292941	0.0546347
Sucrose	0.652467	0.56791	0.64916
3,4-Dihydroxy-L-phenylalanine	0.979045	0.979045	#N/A
3alpha,7alpha-Dihydroxy-5beta-cholestanoyl-CoA	0.211451	0.211451	#N/A
3alpha,7alpha-Dihydroxy-5beta-cholest-24-enoyl-CoA	0.211451	0.211451	#N/A
3alpha,7alpha,12alpha-Trihydroxy-5beta-cholestanoyl-CoA	0.211451	0.211451	#N/A
3alpha,7alpha,12alpha-Trihydroxy-5beta-cholest-24-enoyl-CoA	0.211451	0.211451	#N/A
Naphthyl-2-methylene-succinyl-CoA	0.211451	0.211451	#N/A
Citronellyl-CoA	0.211451	0.211451	#N/A
Geranoyl-CoA	0.211451	0.211451	#N/A
(2E)-5-Methylhexa-2,4-dienoyl-CoA	0.211451	0.211451	#N/A
Orotate	0.132488	#N/A	0.132488
(S)-Dihydroorotate	0.503552	#N/A	0.503552
tRNA(Asp)	0.808178	0.808178	#N/A
L-Aspartyl-tRNA(Asp)	0.808178	0.808178	#N/A
2-Hydroxyphenylacetate	0.197876	0.197876	#N/A
5-Hydroxyisourate	0.0184298	0.0184298	#N/A
1,3,7-Trimethyluric_acid	0.0184298	0.0184298	#N/A
3,6,8-Trimethylallantoin	0.0184298	0.0184298	#N/A
Anthranilate	0.832226	0.832226	#N/A
3-Oxooctadecanoyl-CoA	0.403712	0.403712	#N/A
(S)-3-Hydroxyoctadecanoyl-CoA	0.403712	0.403712	#N/A
(S)-3-Hydroxytetracosanoyl-CoA	0.403712	0.403712	#N/A
3-Oxohexacosanoyl	0.403712	0.403712	#N/A
(S)-3-Hydroxyhexacosanoyl-CoA	0.403712	0.403712	#N/A
(S)-3-Hydroxy(11Z,14Z,17Z)-Icosatrienoyl-CoA	0.403712	0.403712	#N/A
(S)-3-Hydroxy(7Z,10Z,13Z,16Z)-Docosatetraenoyl-CoA	0.403712	0.403712	#N/A
(S)-3-Hydroxy(7Z,10Z,13Z,16Z,19Z)-Docosapentaenoyl-CoA	0.403712	0.403712	#N/A
(S)-3-Hydroxyicosanoyl-CoA	0.403712	0.403712	#N/A
(S)-3-Hydroxydocosanoyl-CoA	0.403712	0.403712	#N/A
(S)-3-Hydroxy(8Z,11Z,14Z,17Z)-Icosatetraenoyl-CoA	0.403712	0.403712	#N/A
(S)-3-Hydroxy(8Z,11Z,14Z)-Icosatrienoyl-CoA	0.403712	0.403712	#N/A
N6-(L-1,3-Dicarboxypropyl)-L-lysine	0.306146	0.306146	#N/A
Perillyl-CoA	0.814924	0.814924	#N/A
(+)-7-Isojasmonic_acid_CoA	0.706805	#N/A	0.706805
7-Methyl-3-oxo-6-octenoyl-CoA	0.706805	#N/A	0.706805
5beta-Androstane-3,17-dione	0.24913	0.24913	#N/A
5beta-Pregnane-3,20-dione	0.24913	0.24913	#N/A
5beta-Dihydrotestosterone	0.24913	0.24913	#N/A
11beta,21-Dihydroxy-3,20-oxo-5beta-pregnan-18-al	0.24913	0.24913	#N/A
Aldosterone	0.24913	0.24913	#N/A
11beta,21-Dihydroxy-5beta-pregnane-3,20-dione	0.24913	0.24913	#N/A
7alpha-Hydroxy-5beta-cholestan-3-one	0.24913	0.24913	#N/A
7alpha-Hydroxycholest-4-en-3-one	0.24913	0.24913	#N/A

7alpha,12alpha-Dihydroxy-5beta-cholestan-3-one	0.24913	0.24913	#N/A
21-Hydroxy-5beta-pregnane-3,11,20-trione	0.24913	0.24913	#N/A
Pantetheine	0.307418	#N/A	0.307418
Pantothenate	0.307418	#N/A	0.307418
O-Phospho-4-hydroxy-L-threonine	0.651339	0.651339	#N/A
2-Oxo-3-hydroxy-4-phosphobutanoate	0.651339	0.651339	#N/A
(6Z,9Z,12Z,15Z)-Octadecateraenoyl-CoA	7.13154E-05	7.13154E-05	#N/A
(6Z,9Z,12Z)-Octadecatrienoyl-CoA	7.13154E-05	7.13154E-05	#N/A
Pyridine-2,3-dicarboxylate	0.143957	0.143957	#N/A
6Z,9Z,12Z)-Octadecatrienoyl-CoA	1.81796E-05	1.81796E-05	#N/A
3-Oxo-(9Z,12Z,15Z,18Z)-Tetracosatetraenoyl-CoA	1.81796E-05	1.81796E-05	#N/A
(9Z,12Z,15Z,18Z,21Z)-Tetracosapentaenoyl-CoA	1.81796E-05	1.81796E-05	#N/A
3alpha,7alpha,12alpha-Trihydroxy-5beta-cholanate	0.806085	0.806085	#N/A
Chenodeoxycholate	0.806085	0.806085	#N/A
3alpha,7alpha,12alpha-Trihydroxy-5beta-cholestanoate	0.806085	0.806085	#N/A
3-Ureidopropionate	0.215672	0.215672	#N/A
3-Ureidoisobutyrate	0.215672	0.215672	#N/A
3-Aminoisobutanoate	0.215672	0.215672	#N/A
L-Gulono-1,4-lactone	0.595226	#N/A	0.595226
L-xylo-Hexulonolactone	0.595226	#N/A	0.595226
4-Fumarylacetoacetate	0.669339	0.669339	#N/A
Butanoic	0.922331	0.922331	#N/A
Dodecanoic	0.922331	0.922331	#N/A
Tetradecanoic	0.922331	0.922331	#N/A
Caffeine	0.596068	#N/A	0.596068
Cinnavalininate	0.362695	0.362695	#N/A
Glutaconyl-1-CoA	0.609882	#N/A	0.609882
S-(Hydroxymethyl)glutathione	0.837452	#N/A	0.837452
S-Formylglutathione	0.837452	#N/A	0.837452
Carnitine	0.134868	0.134868	#N/A
Hydrogen_sulfide	0.345706	0.345706	#N/A
L-Cystine	0.345706	0.345706	#N/A
Desmosterol	0.239318	0.239318	#N/A
24,25-Dihydrolanosterol	0.239318	0.239318	#N/A
5alpha-Cholesta-7,24-dien-3beta-ol	0.239318	0.239318	#N/A
Isofucosterol	0.239318	0.239318	#N/A
Sitosterol	0.239318	0.239318	#N/A
24-Methylenecholesterol	0.239318	0.239318	#N/A
Campesterol	0.239318	0.239318	#N/A
Zymosterol	0.239318	0.239318	#N/A
5alpha-Cholest-8-en-3beta-ol	0.239318	0.239318	#N/A
4,4-Dimethyl-5alpha-cholesta-8-en-3beta-ol	0.239318	0.239318	#N/A
7-Dehydrodesmosterol	0.239318	0.239318	#N/A
5-Formiminotetrahydrofolate	0.360651	0.360651	#N/A
5-Formyltetrahydrofolate	0.360651	0.360651	#N/A
(25R)-3-alpha,7-alpha,12-alpha-trihydroxy-5-beta-cholestan-26-oyl-CoA	0.801791	0.801791	#N/A
4-Imidazolone-5-propanoate	0.766734	0.766734	#N/A

N-Formimino-L-glutamate	0.766734	0.766734	#N/A
6-Phospho-D-gluconate	0.4004	0.4004	#N/A
D-Ribulose_5-phosphate	0.4004	0.4004	#N/A
4-Methylthio-2-oxobutanoic_acid	0.455485	0.455485	#N/A
Bilirubin_beta-diglucuronide	0.370243	0.370243	#N/A
D-Urobilinogen	0.370243	0.370243	#N/A
G13040	0.370243	0.370243	#N/A
G09660	0.370243	0.370243	#N/A
Formamidopyrimidine_nucleoside_triphosphate	0.660631	0.660631	#N/A
2,5-Diaminopyrimidine_nucleoside_triphosphate	0.660631	0.660631	#N/A
Nicotinate	0.542772	0.542772	#N/A
Linoleoyl-CoA	0.00153796	0.00153796	#N/A
gamma-Linolenoyl-CoA	0.00153796	0.00153796	#N/A
(6Z,9Z,12Z)-Octadecatrienoic_acid	0.00153796	0.00153796	#N/A
(9Z,12Z,15Z)-Octadecatrienoic_acid	0.00153796	0.00153796	#N/A
Stearidonic_acid	0.00153796	0.00153796	#N/A
(6Z,9Z,12Z,15Z)-Octadecatetraenoyl-CoA	0.00153796	0.00153796	#N/A
(R)-Lactaldehyde	0.782786	0.782786	#N/A
7alpha-Hydroxycholesterol	3.91874E-05	#N/A	3.91874E-05
Oxidized_dithiothreitol	0.205136	#N/A	0.205136
Vitamin_K_epoxide	0.205136	#N/A	0.205136
alpha-D-Glucose_6-phosphate	0.519455	0.519455	#N/A
Folate	0.120198	0.120198	#N/A
4alpha-Methylfecosterol	0.0165709	0.0165709	#N/A
G13031	0.264553	#N/A	0.264553
Acetylcholine	0.528836	0.528836	#N/A
G13034	0.103844	0.103844	#N/A
G13041	0.103844	0.103844	#N/A
(4Z,7Z,10Z,13Z,16Z,19Z)-Docosahexaenoyl-CoA	0.184249	0.184249	#N/A
(4Z,7Z,10Z,13Z,16Z)-Docosapentaenoyl-CoA	0.184249	0.184249	#N/A
But-2-enoyl-[acyl-carrier_protein]	0.0357105	0.0357105	#N/A
(3R)-3-Hydroxybutanoyl-[acyl-carrier_protein]	0.0357105	0.0357105	#N/A
(3R)-3-Hydroxydecanoyl-[acyl-carrier_protein]	0.0357105	0.0357105	#N/A
trans-Dec-2-enoyl-[acp]	0.0357105	0.0357105	#N/A
(3R)-3-Hydroxyoctanoyl-[acyl-carrier_protein]	0.0357105	0.0357105	#N/A
trans-Oct-2-enoyl-[acp]	0.0357105	0.0357105	#N/A
(3R)-3-Hydroxypalmitoyl-[acyl-carrier_protein]	0.0357105	0.0357105	#N/A
trans-Hexadec-2-enoyl-[acp]	0.0357105	0.0357105	#N/A
(3R)-3-Hydroxytetradecanoyl-[acyl-carrier_protein]	0.0357105	0.0357105	#N/A
trans-Tetradec-2-enoyl-[acp]	0.0357105	0.0357105	#N/A
trans-Dodec-2-enoyl-[acp]	0.0357105	0.0357105	#N/A
(R)-3-Hydroxyhexanoyl-[acp]	0.0357105	0.0357105	#N/A
trans-Hex-2-enoyl-[acp]	0.0357105	0.0357105	#N/A
(R)-3-Hydroxydodecanoyl-[acp]	0.0357105	0.0357105	#N/A
6-Hydroxyhexanoic_acid	0.338196	#N/A	0.338196
Adipate_semialdehyde	0.338196	#N/A	0.338196
L-Xylulose	0.465269	#N/A	0.465269

Choline_phosphate	0.532237	#N/A	0.532237
dTDP-4-dehydro-6-deoxy-alpha-D-Glucose	0.31697	0.31697	#N/A
4,6-Dideoxy-4-oxo-dTDP-D-glucose	0.31697	0.31697	#N/A
L-Pipecolate	0.92943	0.92943	#N/A
2,3,4,5-Tetrahydropyridine-2-carboxylate	0.92943	0.92943	#N/A
6-(alpha-D-glucosaminyl)-1D-myo-inositol	0.671595	0.671595	#N/A
2-Phosphoglycolate	0.243503	0.243503	#N/A
Reduced_Vitamin_K	0.488759	0.488759	#N/A
1-(1-Alkenyl)-sn-glycero-3-phosphate	0.336194	#N/A	0.336194
R-S-Alanine	0.368428	#N/A	0.368428
Phosphatidylglycerol	0.730511	#N/A	0.730511
Cardiolipin	0.730511	#N/A	0.730511
Raffinose	0.31154	#N/A	0.31154
1-alpha-D-Galactosyl-myo-inositol	0.31154	#N/A	0.31154
Epimelibiose	0.31154	#N/A	0.31154
D-Mannose	0.31154	#N/A	0.31154
Melibiitol	0.31154	#N/A	0.31154
Stachyose	0.31154	#N/A	0.31154
Digalactosyl-diacylglycerol	0.31154	#N/A	0.31154
1,2-Diacyl-3-beta-D-galactosyl-sn-glycerol	0.31154	#N/A	0.31154
alpha-D-Galactose	0.31154	#N/A	0.31154
CMP-N-glycoloylneuraminate	0.753558	0.753558	#N/A
N-Glycoloyl-neuraminate	0.753558	0.753558	#N/A
Guanosine_3'-diphosphate_5'-triphosphate	0.595625	#N/A	0.595625
Guanosine_3',5'-bis(diphosphate)	0.595625	#N/A	0.595625
2,5-Dihydroxypyridine	0.177221	0.177221	#N/A
4-Hydroxybenzoate	0.177221	0.177221	#N/A
4-Hydroxybenzaldehyde	0.177221	0.177221	#N/A
Glutaminyl-tRNA	0.668754	0.668754	#N/A
L-Glutamyl-tRNA(Gln)	0.668754	0.668754	#N/A
L-Asparaginyl-tRNA(Asn)	0.668754	0.668754	#N/A
L-Aspartyl-tRNA(Asn)	0.668754	0.668754	#N/A
Betaine_aldehyde	0.165242	0.165242	#N/A
4-Pyridoxate	0.814084	#N/A	0.814084
2,5-Dihydroxybenzoate	0.814084	#N/A	0.814084
Gentisate_aldehyde	0.814084	#N/A	0.814084
1-Methylnicotinamide	0.814084	#N/A	0.814084
N1-Methyl-4-pyridone-5-carboxamide	0.814084	#N/A	0.814084
N1-Methyl-2-pyridone-5-carboxamide	0.814084	#N/A	0.814084
Dihydropteroate	0.293976	0.293976	#N/A
Tetrahydrofolyl-[Glu](n)	0.293976	0.293976	#N/A
1-Methylxanthine	0.667067	0.667067	#N/A
1-Methyluric_acid	0.667067	0.667067	#N/A
1,7-Dimethyluric_acid	0.667067	0.667067	#N/A
Theobromine	0.667067	0.667067	#N/A
3,7-Dimethyluric_acid	0.667067	0.667067	#N/A
7-Methylxanthine	0.667067	0.667067	#N/A

7-Methyluric_acid	0.667067	0.667067	#N/A
3alpha,7alpha-Dihydroxy-5beta-cholestane	0.257604	0.257604	#N/A
3alpha,7alpha,12alpha-Trihydroxy-5beta-cholestane	0.257604	0.257604	#N/A
3alpha,7alpha,12alpha,26-Tetrahydroxy-5beta-cholestane	0.257604	0.257604	#N/A
(R)-Lactate	0.663771	0.663771	#N/A
alpha,alpha-Trehalose	0.16531	0.16531	#N/A
Itaconate	0.273948	#N/A	0.273948
L-Glutamyl_5-phosphate	0.136373	0.136373	#N/A
G00143	0.462701	0.462701	#N/A
2-Deoxy-D-ribose_5-phosphate	0.463349	0.463349	#N/A
Deoxyribose	0.463349	0.463349	#N/A
Insulin	0.44494	0.44494	#N/A
Insulin_dithiol	0.44494	0.44494	#N/A
Protein_dithiol	0.44494	0.44494	#N/A
Protein_disulfide	0.44494	0.44494	#N/A
Retinol	0.745694	0.745694	#N/A
11-cis-Retinol	0.745694	0.745694	#N/A
11-cis-Retinal	0.745694	0.745694	#N/A
Apocytochrome_c	0.879963	0.879963	#N/A
N-Acetylneuraminate_9-phosphate	0.353555	0.353555	#N/A
Undecaprenyl-diphospho-N-acetylmuramoyl-(N-acetylglucosamine)-L-alanyl-	0.575786	0.575786	#N/A
Undecaprenyl-diphospho-N-acetylmuramoyl-(N-acetylglucosamine)-L-alanyl-	0.575786	0.575786	#N/A
Undecaprenyl-diphospho-N-acetylmuramoyl-(N-acetylglucosamine)-L-alanyl-	0.575786	0.575786	#N/A
D-4'-Phosphopantothenate	0.0505518	#N/A	0.0505518
Sulfite	0.805273	0.805273	#N/A
Acylglycerone_phosphate	0.382806	0.382806	#N/A
O-Alkylglycerone_phosphate	0.382806	0.382806	#N/A
gamma-L-Glutamyl-L-cysteine	0.324362	#N/A	0.324362
beta-Carotene	0.272744	0.272744	#N/A
(S)-2,3-Epoxysqualene	0.248092	0.248092	#N/A
Biotin	0.501017	0.501017	#N/A
D-Aspartate	0.65725	0.65725	#N/A
5-Hydroxy-N-formylkynurenine	0.468284	0.468284	#N/A
5-Hydroxykynurenine	0.468284	0.468284	#N/A
H2S2O3	0.463777	0.463777	#N/A
Phosphatidylinositol-3,4,5-trisphosphate	0.516676	0.516676	#N/A
N-Acetylornithine	0.979673	0.979673	#N/A
Isopentenyl_diphosphate	0.0309697	0.0309697	#N/A
Obtusifoliol	0.337754	0.337754	#N/A
Biotinyl-5'-AMP	0.392149	0.392149	#N/A
Adenylyl_sulfate	0.558405	#N/A	0.558405
UDP-N-acetyl-D-mannosamine	0.631421	0.631421	#N/A
alpha-D-Glucose_1-phosphate	0.19731	0.19731	#N/A
Sulfatide	0.756313	0.756313	#N/A
P1,P3-Bis(5'-adenosyl)_triphosphate	0.15666	#N/A	0.15666
Orotidine_5'-phosphate	0.565489	#N/A	0.565489
2-Hydroxyglutarate	0.0969243	#N/A	0.0969243

trans-Tetradeca-2-enoyl-CoA	0.714518	#N/A	0.714518
trans-Hexadeca-2-enoyl-CoA	0.714518	#N/A	0.714518
trans-Octadeca-2-enoyl	0.714518	#N/A	0.714518
trans-Icosa-2-enoyl-CoA	0.714518	#N/A	0.714518
Icosnoyl-CoA	0.714518	#N/A	0.714518
trans-Docosa-2-enoyl-CoA	0.714518	#N/A	0.714518
Docosanoyl-CoA	0.714518	#N/A	0.714518
trans-Tetracosa-2-enoyl-CoA	0.714518	#N/A	0.714518
trans-Hexacosa-2-enoyl-CoA	0.714518	#N/A	0.714518
Hexacosanoyl-CoA	0.714518	#N/A	0.714518
trans-(8Z,11Z,14Z,17Z)-Icosatetraene-2-enoyl-CoA	0.714518	#N/A	0.714518
(8Z,11Z,14Z,17Z)-Icosatetraenoyl-CoA	0.714518	#N/A	0.714518
trans-(8Z,11Z,14Z)-Icosatriene-2-enoyl-CoA	0.714518	#N/A	0.714518
(8Z,11Z,14Z)-Icosatrienoyl-CoA	0.714518	#N/A	0.714518
trans-(11Z,14Z,17Z)-Icosatriene-2-enoyl-CoA	0.714518	#N/A	0.714518
(11Z,14Z,17Z)-Icosatrienoyl-CoA	0.714518	#N/A	0.714518
trans-(7Z,10Z,13Z,16Z)-Docosatetraene-2-enoyl-CoA	0.714518	#N/A	0.714518
trans-(7Z,10Z,13Z,16Z,19Z)-Docosapentaene-2-enoyl-CoA	0.714518	#N/A	0.714518
G13036	0.88458	#N/A	0.88458
beta-D-Glucose	0.952536	0.952536	#N/A
D-Glucono-1,5-lactone	0.952536	0.952536	#N/A
Cysteamine	0.244581	0.244581	#N/A
trans,trans,cis-Geranylgeranyl_diphosphate	0.913127	0.913127	#N/A
Dehydrodolichol_diphosphate	0.913127	0.913127	#N/A
Maltose	0.56791	0.56791	#N/A
3-Methylglutaconyl-CoA	0.8748	#N/A	0.8748
all-trans-Hexaprenyl_diphosphate	0.776427	#N/A	0.776427
3-Hexaprenyl-4-hydroxybenzoate	0.776427	#N/A	0.776427
5-Phospho-alpha-D-ribose_1-diphosphate	0.516141	#N/A	0.516141
2-Acetolactate	0.679309	#N/A	0.679309
(S)-2-Acetolactate	0.679309	#N/A	0.679309
(S)-2-Aceto-2-hydroxybutanoate	0.679309	#N/A	0.679309
N-Acetyl-D-glucosamine_6-phosphate	0.812694	0.812694	#N/A
Poly-L-glutamate	0.928378	#N/A	0.928378
Heme	0.105532	0.105532	#N/A
(R)-Mevalonate	0.105702	0.105702	#N/A
Coproporphyrinogen_I	0.879788	0.879788	#N/A
Thiamin_triphosphate	0.637241	0.637241	#N/A
Thiamin_diphosphate	0.637241	0.637241	#N/A
HCO3-	0.960197	#N/A	0.960197
Hydroxymethylbilane	0.0534875	0.0534875	#N/A
3-Hydroxypropionyl-CoA	0.245204	0.245204	#N/A
3-Hydroxypropanoate	0.245204	0.245204	#N/A
(S)-3-Hydroxyisobutyryl-CoA	0.245204	0.245204	#N/A
1-(1-Alkenyl)-sn-glycerol	0.595883	0.595883	#N/A
GlA_protein	0.754215	#N/A	0.754215
GlA_protein_precursor	0.754215	#N/A	0.754215

Reduced_ferredoxin	0.958228	#N/A	0.958228
Oxidized_ferredoxin	0.958228	#N/A	0.958228
Reduced_adrenal_ferredoxin	0.958228	#N/A	0.958228
Oxidized_adrenal_ferredoxin	0.958228	#N/A	0.958228
18-Hydroxycorticosterone	0.958228	#N/A	0.958228
Cob(I)yrinate_a,c_diamide	0.30672	0.30672	#N/A
Adenosyl_cobyrinate_a,c_diamide	0.30672	0.30672	#N/A
Triphosphate	0.30672	0.30672	#N/A
5'-Phosphoribosylglycinamide	0.785069	#N/A	0.785069
Presqualene_diphosphate	0.0604779	0.0604779	#N/A
3,5-Diiodo-L-tyrosine	0.211836	0.211836	#N/A
O-(4-Hydroxy-3,5-diidophenyl)-3,5-diiodo-L-tyrosine	0.211836	0.211836	#N/A
HI	0.211836	0.211836	#N/A
Iodine	0.211836	0.211836	#N/A
3-Iodo-L-tyrosine	0.211836	0.211836	#N/A
Triiodothyronine	0.211836	0.211836	#N/A
Lipoylprotein	0.900747	0.900747	#N/A
D-Glucose	0.903	0.903	#N/A
Uroporphyrinogen_III	0.520785	0.520785	#N/A
D-Ornithine	0.698997	0.698997	#N/A
5-Amino-2-oxopentanoic_acid	0.698997	0.698997	#N/A
D-Proline	0.698997	0.698997	#N/A
1-Pyrroline-2-carboxylate	0.698997	0.698997	#N/A
D-Arginine	0.698997	0.698997	#N/A
5-Guanidino-2-oxopentanoate	0.698997	0.698997	#N/A
cis-4-Hydroxy-D-proline	0.698997	0.698997	#N/A
1-Pyrroline-4-hydroxy-2-carboxylate	0.698997	0.698997	#N/A
2-Hexaprenyl-6-methoxyphenol	0.0377646	0.0377646	#N/A
P1,P4-Bis(5'-adenosyl)_tetraphosphate	0.292941	0.292941	#N/A
P1,P4-Bis(5'-uridyl)_tetraphosphate	0.292941	0.292941	#N/A
P1,P4-Bis(5'-guanosyl)_tetraphosphate	0.292941	0.292941	#N/A
P1,P4-Bis(5'-xanthosyl)_tetraphosphate	0.292941	0.292941	#N/A
19-Hydroxyandrost-4-ene-3,17-dione	0.557056	#N/A	0.557056
19-Hydroxytestosterone	0.557056	#N/A	0.557056
(3R)-3-Isopropenyl-6-oxoheptanoate	0.930009	#N/A	0.930009
(3R)-3-Isopropenyl-6-oxoheptanoyl-CoA	0.930009	#N/A	0.930009
cis-2-Methyl-5-isopropylhexa-2,5-dienoic_acid	0.930009	#N/A	0.930009
trans-2-Methyl-5-isopropylhexa-2,5-dienoic_acid	0.930009	#N/A	0.930009
CDP-diacylglycerol	0.555332	0.555332	#N/A
2-(Formamido)-N1-(5'-phosphoribosyl)acetamidine	0.381901	0.381901	#N/A
D-Glucosamine	0.186562	0.186562	#N/A
D-Glucosaminide	0.186562	0.186562	#N/A
Pentosans	0.186562	0.186562	#N/A
Calcidiol	0.641927	#N/A	0.641927
Calcitriol	0.641927	#N/A	0.641927
5-(2'-Carboxyethyl)-4,6-dihydroxypicolinate	0.861825	#N/A	0.861825
3,4-Dihydroxybenzoate	0.861825	#N/A	0.861825

Benzene-1,2,4-triol	0.861825	#N/A	0.861825
1,1-Dichloro-2-(dihydroxy-4'-chlorophenyl)-2-(4'-chlorophenyl)ethylene	0.861825	#N/A	0.861825
2,3-Dihydroxy_1,1,1-Trichloro-2,2-bis(4'-chlorophenyl)ethane	0.861825	#N/A	0.861825
4-Sulfocatechol	0.861825	#N/A	0.861825
3,4-Dihydroxyfluorene	0.861825	#N/A	0.861825
2,2',3-Trihydroxybiphenyl	0.861825	#N/A	0.861825
1,2-Dihydroxyfluorene	0.861825	#N/A	0.861825
2,2',3-Trihydroxydiphenylether	0.861825	#N/A	0.861825
Carbazole	0.861825	#N/A	0.861825
2'-Aminobiphenyl-2,3-diol	0.861825	#N/A	0.861825
Styrene	0.861825	#N/A	0.861825
Styrene_cis-glycol	0.861825	#N/A	0.861825
Phenanthrene-3,4-diol	0.861825	#N/A	0.861825
Phenanthracene	0.861825	#N/A	0.861825
(+)-cis-3,4-Dihydrophenanthrene-3,4-diol	0.861825	#N/A	0.861825
CDP-ethanolamine	0.578224	0.578224	#N/A
4alpha-Methylzymosterol	0.597253	0.597253	#N/A
Carnosine	0.315471	#N/A	0.315471
beta-Alanyl-N(pi)-methyl-L-histidine	0.315471	#N/A	0.315471
Betaine	0.850014	0.850014	#N/A
C15811	0.980994	0.980994	#N/A
C15812	0.980994	0.980994	#N/A
(6Z,9Z,12Z,15Z,18Z,21Z)-3-Hydroxytetracosahexa-6,9,12,15,18,21-enoyl-CoA	0.459325	#N/A	0.459325
(6Z,9Z,12Z,15Z,18Z,21Z)-3-Oxotetracosahexa-6,9,12,15,18,21-enoyl-CoA	0.459325	#N/A	0.459325
(6Z,9Z,12Z,15Z,18Z)-3-Hydroxytetracosapenta-6,9,12,15,18-enoyl-CoA	0.459325	#N/A	0.459325
(6Z,9Z,12Z,15Z,18Z)-3-Oxotetracosapenta-6,9,12,15,18-enoyl-CoA	0.459325	#N/A	0.459325
G00005	0.868537	#N/A	0.868537
G10526	0.868537	#N/A	0.868537
G00006	0.868537	#N/A	0.868537
Sulfur	0.708353	0.708353	#N/A
Ascorbate	0.566174	0.566174	#N/A
Dehydroascorbate	0.566174	0.566174	#N/A
Allantoate	0.255109	0.255109	#N/A
(-)-Ureidoglycolate	0.255109	0.255109	#N/A
5-Oxoproline	0.786089	0.786089	#N/A
2-Amino-3-oxoadipate	0.410088	#N/A	0.410088
Lipoyl-AMP	0.514211	0.514211	#N/A
3-D-Glucosyl-1,2-diacylglycerol	0.323412	#N/A	0.323412
Diglucosyl-diacylglycerol	0.323412	#N/A	0.323412
Alkyl_sulfate	0.473714	#N/A	0.473714
2-Acyl-sn-glycero-3-phosphocholine	0.939803	0.939803	#N/A
2-Acyl-sn-glycero-3-phosphoethanolamine	0.939803	0.939803	#N/A
Dopaquinone	0.961277	0.961277	#N/A
4-Coumarate	0.961277	0.961277	#N/A
3,4-Dihydroxy-trans-cinnamate	0.961277	0.961277	#N/A
5,6-Dihydroxyindole	0.961277	0.961277	#N/A
(S)-N-Methylcoclaurine	0.961277	0.961277	#N/A

3'-Hydroxy-N-methyl-(S)-coclaurine	0.961277	0.961277	#N/A
Indole-5,6-quinone	0.961277	0.961277	#N/A
22beta-Hydroxycholesterol	0.241271	0.241271	#N/A
20alpha,22beta-Dihydroxycholesterol	0.241271	0.241271	#N/A
Ferredoxin	0.241271	0.241271	#N/A
4-Methylpentanal	0.241271	0.241271	#N/A
L-2-Aminoadipate_adenylate	0.526734	0.526734	#N/A
alpha-Aminoadipoyl-S-acyl_enzyme	0.526734	0.526734	#N/A
Holo-Lys2	0.526734	0.526734	#N/A
G00145	0.39021	#N/A	0.39021
20alpha-Hydroxy-4-pregnen-3-one	0.484596	0.484596	#N/A
17alpha,20alpha-Dihydroxypregn-4-en-3-one	0.484596	0.484596	#N/A
1-(5'-Phosphoribosyl)-5-amino-4-imidazolecarboxamide	0.462956	0.462956	#N/A
5-Amino-4-imidazolecarboxyamide	0.462956	0.462956	#N/A
L-2-Amino-3-oxobutanoic_acid	0.826047	0.826047	#N/A
L-Methionyl-tRNA	0.669005	0.669005	#N/A
dITP	0.0546347	#N/A	0.0546347
2'-Deoxyinosine_5'-phosphate	0.0546347	#N/A	0.0546347
RNA	0.752916	0.752916	#N/A
6-Lactoyl-5,6,7,8-tetrahydropterin	0.886963	#N/A	0.886963
Lipid_hydroperoxide	0.949826	#N/A	0.949826
Lipid	0.949826	#N/A	0.949826
Aminoimidazole_ribotide	0.13167	0.13167	#N/A
1-(5-Phospho-D-ribosyl)-5-amino-4-imidazolecarboxylate	0.13167	0.13167	#N/A

Liver

Reactome

		Reporter P-value	
#Feature	**No-direction**	**Down regulated**	**Up regulated**
Olfactory_Receptor_-_G_Protein_olfactory_trimer_complex_formation	0.774466	0.999905	0.00407533
Cleavage_at_the_3-Splice_Site_and_Exon_Ligation	0.901625	0.999504	0.0551545
Formation_of_Exon_Junction_Complex	0.901625	0.999504	0.0551545
Formation_of_an_intermediate_Spliceosomal_C_complex	0.954321	0.99984	0.0855156
Lariat_Formation_and_5-Splice_Site_Cleavage	0.936379	0.999632	0.0855156
Formation_of_the_active_Spliceosomal_C_complex	0.931372	0.999583	0.0855156
Recruitment_of_additional_gamma_tubulin/_gamma_TuRC_to_the_centros	0.440636	0.438048	0.486872
Formation_of_the_Spliceosomal_B_Complex	0.8899	0.999875	0.0233878
Dissociation_of_Phospho-Nlp_from_the_centrosome	0.330625	0.443105	0.272004
Loss_of_C-Nap-1_from_centrosomes	0.330625	0.443105	0.272004
Plk1-mediated_phosphorylation_of_Nlp	0.330625	0.443105	0.272004
Recruitment_of_CDK11p58_to_the_centrosomes	0.330625	0.443105	0.272004
Recruitment_of_Plk1_to_centrosomes	0.330625	0.443105	0.272004
GPCRs_that_act_as_GEFs_for_G_alpha-i	0.912517	0.991668	0.21075
Kinetochore_capture_of_astral_microtubules	0.954376	0.426273	0.998055
Translocation_of_ribosome_by_3_bases_in_the_3_direction	0.50346	0.0962867	0.933955

Aminoacyl-tRNA__binds_to_the_ribosome_at_the_A-site	0.487693	0.0853217	0.933955
Hydrolysis_of_eEF1A:GTP	0.487693	0.0853217	0.933955
Release_of_40S_and_60S_subunits_from_the_80S_ribosome_	0.516158	0.0872385	0.940697
GTP_bound_eRF3:eRF1_complex_binds_the_peptidyl_tRNA:mRNA:80S_Ribo	0.418495	0.0714092	0.917414
GTP_Hydrolysis_by_eRF3_bound_to_the_eRF1:mRNA:polypeptide:80S_Ribo	0.418495	0.0714092	0.917414
Polypeptide_release_from_the_eRF3-GDP:eRF1:mRNA:80S_Ribosome_comp	0.418495	0.0714092	0.917414
eIF5B:GTP_is_hydrolyzed_and_released	0.520463	0.12728	0.917414
The_60S_subunit_joins_the_translation_initiation_complex_	0.520463	0.12728	0.917414
Peptide_transfer_from_P-site_tRNA_to_the_A-site_tRNA	0.459002	0.0872385	0.917414
GAPs_inactivate_Rho_GTPase:GTP_by_hydrolysis	0.0916079	0.0234752	0.663017
Association_of_phospho-L13a_with_GAIT_element_of_Ceruloplasmin_mRN	0.939999	0.842425	0.884201
Formation_of_translation_initiation_complexes_yielding_circularized_Cerulo	0.933332	0.823133	0.884201
eIF2:GTP_is_hydrolyzed,_eIFs_are_released_	0.938714	0.823439	0.896699
Start_codon_recognition	0.938714	0.823439	0.896699
Formation_of_translation_initiation_complexes_containing_mRNA_that_do	0.938888	0.823133	0.896699
Ribosomal_scanning_	0.938888	0.823133	0.896699
GEFs_activate_Rho_GTPase:GDP	0.168366	0.120879	0.475857
Formation_of_the_43S_pre-initiation_complex	0.913072	0.789043	0.870642
ATP_Hydrolysis_By_Myosin	0.439894	0.346635	0.555406
Calcium_Binds_Troponin-C	0.439894	0.346635	0.555406
Myosin_Binds_ATP	0.439894	0.346635	0.555406
Release_Of_ADP_From_Myosin	0.439894	0.346635	0.555406
Dissociation_of_L13a_from_the_60s_ribosomal_subunit	0.349989	0.121404	0.791306
GPCRs_that_act_as_GEFs_for_G_alpha-q/11	0.164355	0.786452	0.00536115
GPCRs_that_act_as_GEFs_for_G_alpha-s	0.794772	0.957119	0.263319
eIF3_and_eIF1A_bind_to_the_40S_subunit	0.840298	0.648702	0.849021
Addition_of_nucleotides_leads_to_transcript_elongation	0.718281	0.919924	0.294672
Recruitment_of_elongation_factors_to_form_elongation_complex	0.718281	0.919924	0.294672
ATAC_spliceosome_mediated_3_splice_site_cleavage,_exon_ligation	0.401462	0.929587	0.0441608
ATAC_spliceosome_mediated_Lariat_formation,5_splice_site_cleavage_	0.401462	0.929587	0.0441608
Formation_of_AT-AC_B_Complex	0.401462	0.929587	0.0441608
Formation_of_AT-AC_C_complex	0.401462	0.929587	0.0441608
Formation_of_pre-mRNPs	0.267327	0.852638	0.0436104
Abortive_initiation_after_formation_of_the_first_phosphodiester_bond	0.182259	0.636233	0.0569671
Abortive_Initiation_Before_Second_Transition	0.182259	0.636233	0.0569671
Fall_Back_to_Closed_Pre-initiation_Complex	0.182259	0.636233	0.0569671
Formation_of_the_closed_pre-initiation_complex	0.182259	0.636233	0.0569671
Newly_Formed_Phosphodiester_Bond_Stabilized_and_PPi_Released	0.182259	0.636233	0.0569671
NTP_Binds_Active_Site_of_RNA_Polymerase_II	0.182259	0.636233	0.0569671
Nucleophillic_Attack_by_3-hydroxyl_Oxygen_of_nascent_transcript_on_the	0.182259	0.636233	0.0569671
RNA_Polymerase_II_Promoter_Opening:_First_Transition	0.182259	0.636233	0.0569671
Cleavage_of_mRNA_at_the_3-end	0.856938	0.959355	0.343989
Addition_of_Nucleotides_5_through_9_on_the_growing_Transcript	0.258351	0.636233	0.106673
NADH_enters_the_respiratory_chain_at_Complex_I	0.924444	0.60583	0.934751
Formation_of_AT-AC_A_complex	0.127722	0.580378	0.0521192
Actin/tubulin:prefoldin_complex_associates_with_CCT/TriC	0.675285	0.534703	0.718806
2-4_nt.backtracking_of_Pol_II_complex_on_the_template_leading_to_elong	0.547632	0.894791	0.147566

7-14_nt._Backtracking_of_Pol_II_complex_on_the_template_leading_to_elo	0.547632	0.894791	0.147566
Abortive_termination_of_elongation_after_arrest	0.547632	0.894791	0.147566
Elongating_transcript_encounters_a_lesion_in_the_template	0.547632	0.894791	0.147566
Pol_II_elongation_complex_moves_on_the_template_as_transcript_elongat	0.547632	0.894791	0.147566
Resumption_of_elongation_after_recovery_from_pausing	0.547632	0.894791	0.147566
Separation_of_elongating_transcript_from_template	0.547632	0.894791	0.147566
Formation_of_NR-NCOR_Complex	0.43782	0.548854	0.355457
Binding_of_TFIIE_to_the_growing_preinitiation_complex	0.055637	0.522072	0.0116753
mRNA_polyadenylation	0.761659	0.985499	0.0676645
Crk_binding_to_p130cas	0.516114	0.552165	0.456524
Ubiquitination_of_Cyclin_B_by_phospho-APC/C:Cdc20_complex	0.218514	0.764747	0.0116495
Assembly_of_repair_proteins_at_the_site_of_Pol_II_blockage	0.321668	0.446746	0.301024
Displacement_of_stalled_Pol_II__from_the_lesion_site_	0.321668	0.446746	0.301024
Phosphorylation_of_p130Cas_by_Src-FAK_complex	0.542316	0.552165	0.499068
Recruitment_of_p130Cas_to_FAK	0.542316	0.552165	0.499068
Interaction_of_SOS_with_Grb2_bound_to_FADK1	0.811295	0.771955	0.681353
Ubiquitination_of_cell_cycle_proteins_targeted_by_the_APC/C:Cdh1comple	0.627474	0.920786	0.137869
Translocation_of_Preproinsulin_to_Endoplasmic_Reticulum	0.596369	0.977356	0.246514
Recruitment_of_RNA_Polymerase_II_Holoenzyme_by_TFIIF_to_the_pol_II_p	0.0446355	0.522072	0.00610424
Recruitment_of_Grb2_to_pFADK1	0.75493	0.696412	0.681353
Ubiquitination_of_Cyclin_A_by_APC/C:Cdc20_complex	0.343689	0.844792	0.0456677
Phosphorylation_of_PLC-gamma1	0.770882	0.534147	0.840853
Association_of_Cyclin_B:Cdc2_with_Cdc20:APC/C_complex	0.253215	0.792962	0.0121756
Auto-phosphorylation_of_FADK1_at_Y397	0.64511	0.550525	0.681353
Phosphorylation_of_pFADK1_by_SRC	0.64511	0.550525	0.681353
Translocation_of_FADK1_to_Focal_complexes	0.64511	0.550525	0.681353
Orc1_is_phosphorylated_by_cyclin_A/CDK2	0.183708	0.0866338	0.546229
DNA_Replication_Factor_A_(RPA)_associates_with_the_pre-replicative_com	0.252012	0.144487	0.641985
Association_of_cell_cycle_proteins_with_the_APC/C:Cdh1_complex	0.685396	0.938411	0.155735
Free_APC/C_phosphorylated_by_Cyclin_B:Cdc2	0.358436	0.792962	0.0335047
The_high_affinity_receptor_complex_binds_to_G-protein	0.401021	0.575836	0.235001
The_receptor:G-protein_complex__binds_GTP	0.401021	0.575836	0.235001
The_receptor:G-protein_complex_dissociates	0.401021	0.575836	0.235001
The_receptor:G-protein_complex_releases_GDP	0.401021	0.575836	0.235001
Hyperphosphorylation_(Ser2)_of_RNA_Pol_II_CTD_by_P-TEFb_complex	0.357476	0.839733	0.0719445
Hypophosphorylation_of_RNA_Pol_II_CTD_by_FCP1P_protein	0.216752	0.490399	0.160635
Capping_complex_formation	0.629658	0.662233	0.52802
Dissociation_of__transcript_with_5-GMP_from_GT	0.629658	0.662233	0.52802
Formation_of_the_CE:GMP_intermediate_complex	0.629658	0.662233	0.52802
Hydrolysis_of_the_5-end_of_the_nascent_transcript_by_the_capping_enzy	0.629658	0.662233	0.52802
Methylation_of_GMP-cap_by_RNA_Methyltransferase	0.629658	0.662233	0.52802
SPT5_subunit_of_Pol_II_binds_the_RNA_triphosphatase_(RTP)	0.629658	0.662233	0.52802
Transfer_of_GMP_from_the_capping_enzyme_GT_site_to_5-end_of_mRNA	0.629658	0.662233	0.52802
alphaIIbbeta3:c-Src_clustering	0.605066	0.496859	0.681353
Autophosphorylation_of_c-src	0.605066	0.496859	0.681353
Interaction_of_alphaIIbbeta3_with_Fibrinogen	0.605066	0.496859	0.681353
Release_of_Csk_from_Src	0.605066	0.496859	0.681353

Dephosphorylation_of_inactive_c-src_by_PTPB1	0.592983	0.481597	0.681353
Assembly_in_clathrin-coated_vesicles_(CCVs)	0.516546	0.621597	0.356861
Association_of_Cyclin_A_with_the_APC/C	0.39323	0.87232	0.050797
Activation_of_claspin	0.224166	0.117743	0.641985
Recruitment_of_TAP_to_the_EJC	0.730576	0.989871	0.0529393
Abortive_Initiation_After_Second_Transition	0.375173	0.490399	0.345746
Dissociation_of_Rho_GTP:GDP_from_GDI_complex	0.160222	0.0429589	0.509576
GDIs_block_activation_of_Rho_GTPase:GDP	0.160222	0.0429589	0.509576
unfolded_actin/tubulin_associates_with_prefoldin	0.76514	0.587098	0.810506
Vamp2_associated_trans-Golgi_Network_Derived_Vesicle_Uncoating	0.463065	0.610623	0.308784
Loading_of_claspin_onto_DNA_during_replication_origin_firing	0.271805	0.154266	0.641985
Multiubiquitination_of_Nek2A	0.409598	0.909565	0.0419327
Activation_of_GT	0.471097	0.662233	0.307878
RNA_Polymerase_II_CTD_(phosphorylated)_binds_to_CE	0.471097	0.662233	0.307878
Vamp7_associated_trans-Golgi_Network_Derived_Vesicle_Uncoating	0.283641	0.264919	0.452978
Vamp8_associated_trans-Golgi_Network_Derived_Vesicle_Uncoating	0.396499	0.448256	0.399507
Cdc45_associates_with_the_pre-replicative_complex_at_the_origin	0.242672	0.125039	0.641985
G-protein_beta-gamma_subunits_rebind_the_alpha-GDP_subunit	0.522483	0.645824	0.307284
Abortive_termination_of_early_transcription_elongation_by_DSIF:NELF	0.345211	0.874129	0.0563465
Formation_of_DSIF:NELF:early_elongation_complex	0.345211	0.874129	0.0563465
Addition_of_nucleotides_10_and_11_on_the_growing_transcript:_Third_Tra	0.349571	0.490399	0.307878
Addition_of_nucleotides_between_position_+11_and_+30	0.349571	0.490399	0.307878
Extrusion_of_5-end_of_30_nt_long_transcript_through_the_pore_in_Pol_II_	0.349571	0.490399	0.307878
Phosphorylation_(Ser5)_of_RNA_pol_II_CTD	0.349571	0.490399	0.307878
PDGF_binds_to_extracellular_matrix_proteins	0.172065	0.241192	0.262977
Sprouty_sequesters_Cbl_away_from_active_EGFR	0.125373	0.141945	0.310303
CDK_and_DDK_associate_with_the_Mcm10:pre-replicative_complex	0.25587	0.131396	0.641985
Association_of_Nek2A_with_MCC:APC/C	0.467687	0.931852	0.0464206
Association_of_Cyclin_A:Cdk2_with_Cdh1	0.499405	0.87232	0.0731392
Phosphorylation_of_Cdh1_by_Cyclin_A:Cdk2	0.499405	0.87232	0.0731392
Cbl-mediated_ubiquitination_of_CIN85	0.135013	0.202993	0.233331
Phosphorylation_of_SLP-76	0.831225	0.632002	0.849605
G-protein_Gq_activation_by_PAR	0.965137	0.978959	0.570397
G12/13_activation_by_PAR	0.852851	0.907994	0.47463
Cleavage_of_Signal_Peptide_(Preproinsulin)	0.37203	0.988552	0.0710678
3-_incision_of_DNA_by_XPG_in_GG-NER	0.313089	0.197405	0.61592
5-incision_of_DNA_by_ERCC1-XPF_in_GG-NER	0.313089	0.197405	0.61592
Binding_of_ERCC1-XPF_to_preincision_complex_	0.313089	0.197405	0.61592
Vamp2_associated_trans-Golgi_Network_Vesicle_Scission	0.321899	0.553032	0.185442
Activation_of_APC/C:Cdc20_by_dissociation_of_Cdc20:phospho-APC/C_fror	0.533675	0.964013	0.0464206
Binding_of_the_MCC_complex_to_the_APC/C_complex	0.533675	0.964013	0.0464206
Ubiquitination_of_Securin_by_phospho-APC/C:Cdc20_complex	0.329505	0.947751	0.00708584
Dissociation_of_PTRF:Polymerase_I/Nascent_Pre_rRNA_Complex:TTF-I:Sal_I	0.982211	0.975795	0.840506
PTRF_Binds_the_Polymerase_I_Transcription_Complex/Nascent_Pre_rRNA_	0.982211	0.975795	0.840506
Interaction_of_integrin_alpha_3_beta_1_with_Laminin-5	0.152922	0.0982707	0.565227
Interaction_of_integrin_alpha_6_beta_1_with_Laminin-1	0.152922	0.0982707	0.565227
Interaction_of_integrin_alpha_7_beta_1_with_Laminin-1	0.152922	0.0982707	0.565227

Interaction_of_integrin_alpha_7_beta_1_with_Laminin-2	0.152922	0.0982707	0.565227
alphaIIbbeta3_activation	0.594272	0.458463	0.701398
EGFR_non-clathrin_mediated_endocytosis	0.160281	0.218705	0.26765
Vamp7_associated_trans-Golgi_Network_Vesicle_Scission	0.161599	0.198043	0.30267
Vamp8_associated_trans-Golgi_Network_Vesicle_Scission	0.256315	0.374849	0.262611
Free_APC/C_phosphorylated_by_Plk1	0.606982	0.8854	0.129714
Phosphorylation_of_TBSMs_in_LAT	0.837903	0.633209	0.849605
Multiubiquitination_of_APC/C-associated_Cdh1	0.588368	0.947751	0.0593694
Formation_of_open_bubble_structure_in_DNA_by_helicases	0.401936	0.281488	0.61592
Recruitment_of_repair_factors_to_form_preincision_complex	0.401936	0.281488	0.61592
Recruitment_of_RNA_Polymerase_III_to_TFIIIB:SNAPc:Type_3_Promoter_Co	0.883396	0.916483	0.534279
RNA_Polymerase_III_Abortive_Initiation_At_Type_3_Open_Promoters	0.883396	0.916483	0.534279
RNA_Polymerase_III_Promoter_Opening_at_Type_3_Promoters	0.883396	0.916483	0.534279
RNA_Polymerase_III_Simple_Start_Sequence_Initiation_At_Type_3_Promote	0.883396	0.916483	0.534279
Loss_of_Rrn3_from_RNA_Polymerase_I_promoter_escape_complex	0.975709	0.965096	0.840506
Recruitment_of_Active_RNA_Polymerase_I_to_SL1:phos.UBF-1:rDNA_Prom	0.975709	0.965096	0.840506
Polymerase_I_Transcription_Complex/Nascent_Pre_rRNA_Complex_pauses_	0.971287	0.956212	0.840506
Interaction_of_alpha_2_beta_1_with_Lamini-5	0.434209	0.377901	0.573833
Interaction_of_integrin_alpha_1_beta_1_with_Laminin-1	0.434209	0.377901	0.573833
Vamp2_associated_trans-Golgi_Network_AP-1_Binding_And_Cargo_Capture	0.0916586	0.385459	0.0558503
Association_of_Securin_with_Cdc20:APC/C_complex	0.384248	0.964013	0.00707329
DSIF_complex_binds_to_RNA_Pol_II_(hypophosphorylated)	0.0534396	0.291331	0.0563465
Recruitment_of__RNA_polymerase_III_to_TFIIIB:TFIIIC:TFIIIA:Type_1_Promo	0.740516	0.750784	0.552057
RNA_Polymerase_III_Abortive_Initiation_At_Type_1_Open_Promoters	0.740516	0.750784	0.552057
RNA_Polymerase_III_Promoter_Opening_at_Type_1_Promoters	0.740516	0.750784	0.552057
RNA_Polymerase_III_Simple_Start_Sequence_Initiation_At_Type_1_Promote	0.740516	0.750784	0.552057
Elongation_of_pre-rRNA_transcript	0.962955	0.940969	0.840506
Akt_binds_eNOS_complex_via_HSP90	0.256269	0.111653	0.631764
Akt_phosphorylates_eNOS	0.256269	0.111653	0.631764
NO_biosynthesis	0.256269	0.111653	0.631764
Change_of_PKC_theta_conformation	0.857271	0.7251	0.821547
Vamp7_associated_trans-Golgi_Network_AP-1_Binding_And_Cargo_Capture	0.0279391	0.0771008	0.101057
Vamp8_associated_trans-Golgi_Network_AP-1_Binding_And_Cargo_Capture	0.0594043	0.198751	0.0891641
Activation_of_ZAP-70	0.787084	0.600644	0.803131
Phosphorylation_of_ZAP-70_by_Lck	0.787084	0.600644	0.803131
Recruitment_of_ZAP-70_to_phosphorylated_ITAMs	0.787084	0.600644	0.803131
Association_of_Cdh1_with_the_APC/C	0.659769	0.964013	0.0655866
Dissociation_of_phospho-Cdh1_from_the_APC/C_complex	0.659769	0.964013	0.0655866
Dissociation_of_Cdc20_from_APC/C_complex	0.477283	0.964013	0.0125555
Inactivation_of_Lck_by_Csk	0.830764	0.672068	0.821547
3_incision_of_the_lesioned_strand_of_DNA_in_TC-NER	0.648843	0.380335	0.81034
5_incision_leading_to_excision_of_DNA_fragment_with_lesion_in_TC-NER	0.648843	0.380335	0.81034
Cleavage_of_Intronless_Pre-mRNA_at_3-end	0.817998	0.736945	0.743924
Recruitment_of_RNA_Polymerase_III_to_the_TFIIIB:TFIIIC:_Type_2_Promote	0.699014	0.703862	0.552057
RNA_Polymerase_III_Abortive_Initiation_At_Type_2_Open_Promoters	0.699014	0.703862	0.552057
RNA_Polymerase_III_Promoter_Opening_at_Type_2_Promoters	0.699014	0.703862	0.552057
RNA_Polymerase_III_Simple_Start_Sequence_Initiation_At_Type_2_Promote	0.699014	0.703862	0.552057

Adhesion_via_alpha_2_beta_1_glycoprotein	0.380209	0.623776	0.182572
Interaction_of_integrin_alpha_1_beta_1_with_Collagen_IV	0.380209	0.623776	0.182572
Interaction_of_integrin_alpha_10_beta_1_with_Collagen-II	0.380209	0.623776	0.182572
Interaction_of_integrin_alpha_11_beta_1_with_Collagen-I	0.380209	0.623776	0.182572
Adenylate_cyclase_increases_the_GTPase_activity_of_G_alpha-olf	0.242054	0.552874	0.0736961
Adenylate_cyclase_increases_the_GTPase_activity_of_Gi_alpha	0.242054	0.552874	0.0736961
Dissociation_of_the_Gi_alpha:G_olf_complex	0.242054	0.552874	0.0736961
G_alpha-olf:GTP_binds_to_Gi_alpha1:GTP:adenylate_cyclase_complex	0.242054	0.552874	0.0736961
Sustained_activation_of_SRC_kinase_by_SHP2	0.578485	0.658214	0.377987
Cdt1_is_displaced_from_the_pre-replicative_complex.	0.261864	0.109411	0.628372
Mcm10_associates_with_the_pre-replicative_complex,_stabilizing_Mcm2-7	0.261864	0.109411	0.628372
Dephosphorylation_of_Lck-pY505_by_CD45	0.836304	0.678114	0.821547
Association_of_Cyclin_B/Cdk1_with_replicative_origin_inhibits_pre-RC_forn	0.0556919	0.0534253	0.347814
Internal_Methylation_of_mRNA	0.0332716	0.240236	0.0340837
Interaction_of_integrin_alpha_V_beta_3_with_Fibrillin	0.960489	0.801681	0.967191
Beta-tubulin:GTP:Cofactor_D:alpha-tubulin:GTP:Cofactor_E+_Cofactor_C->_	0.474057	0.530339	0.354599
Beta-tubulin:GTP:Cofactor_D:alpha-tubulin:GTP:Cofactor_E:Cofactor_C->_Be	0.474057	0.530339	0.354599
Mcm2-7_associates_with_the_Cdt1:CDC6:ORC:origin_complex,_forming_the	0.32461	0.109411	0.741552
Activation_of_Lck	0.841597	0.684068	0.821547
Phosphorylation_of_ITAM_motifs_in_CD3_complexes	0.841597	0.684068	0.821547
Autocatalytic_phosphorylation_of_FGFR4	0.586407	0.886038	0.0921756
FGFR4_binds_to_FGF	0.586407	0.886038	0.0921756
Autocatalytic_phosphorylation_of_FGFR2c	0.532028	0.887176	0.0873199
Recognition_and_binding_of_the_mRNA_cap_by_the_cap-binding_complex	0.0391702	0.291331	0.0340837
Formation_of_the_Cleavage_and_Polyadenylation_Complex	0.931618	0.736945	0.95337
Interaction_of_integrin_alpha_3_beta_1_with_Thrombospondin-1	0.654347	0.545214	0.695265
Interaction_of_integrin_alpha_IIb_beta_3_with_Thrombospondin	0.9164	0.829218	0.843749
Gi_alpha1:GTP_binds_to_adenylate_cyclase	0.132451	0.378074	0.0736961
Interaction_of_integrin_alpha_6_beta_4_with_Laminin-5	0.0492427	0.0468975	0.329385
Binding_of_SRC_tyrosine_kinase	0.0398623	0.22722	0.0418303
Src-mediated_phoshorylation_of_FcR1_gamma	0.0398623	0.22722	0.0418303
Electron_transfer_from_reduced_cytochrome_c_to_molecular_oxygen	0.289538	0.401972	0.301777
Beta-tubulin:GTP:Cofactor_D+alpha-tubulin:GTP:Cofactor_E->_Beta-tubulin:	0.40661	0.458899	0.354599
PI3K_catalytic_subunit_binds_to_Gab1:Grb2:PI3K:EGF:EGFR	0.669066	0.542049	0.742075
PI3K_converts_Phosphatidylinositol-4,5-bisphosphate_to_Phosphatidylinosit	0.669066	0.542049	0.742075
Cyclin_D:Cdk4/6_mediated_phosphorylation_of_Rb_and_dissociation_of_Rl	0.0320598	0.0485205	0.155839
Association_of_MRN_with_sites_of_DSB_	0.996292	0.383155	0.999251
BRCA1_associates_with_53BP1at_the_site_of_DNA_double-strand_break	0.999474	0.677503	0.99983
Sos-mediated_nucleotide_exchange_of_Ras_(EGF:EGFR-Sos:Grb2:Shc)	0.49388	0.69555	0.238003
Autocatalytic_phosphorylation_of_FGFR1c	0.489903	0.873188	0.0873199
FGFR1c_binds_to_FGF	0.489903	0.873188	0.0873199
Autocatalytic_phosphorylation_of_FGFR3c	0.458586	0.850256	0.0873199
FGFR3c_binds_to_FGF	0.458586	0.850256	0.0873199
GAP_Recruitment_to_the_Coatomer:Arf1-GTP_Complex	0.862289	0.9811	0.231601
methionine_+_tRNA(Met)_+_ATP_=>_Met-tRNA(Met)_+_AMP_+_pyrophos	0.991592	0.987086	0.843243
Exchange_of_ADP_for_ATP_in_CCT/TriC:actin_complex	0.769412	0.71241	0.697251
Hydrolysis_of_ATP_and_release_of_folded_actin_from_CCT/TriC	0.769412	0.71241	0.697251

Multi-ubiquitination_of_phospho-beta-catenin_by_SCF-beta-TrCP1	0.997423	0.998896	0.716385
leucine_+_tRNA(Leu)_+_ATP_=>_Leu-tRNA(Leu)_+_AMP_+_pyrophosphate	0.979153	0.971349	0.78877
Binding_of_TFIIA_and_TFIIB_to_the_pol_II_promoter:TFIID_complex	0.140635	0.708307	0.0113319
proline_+_tRNA(Pro)_+_ATP_=>_Pro-tRNA(Pro)_+_AMP_+_pyrophosphate	0.995856	0.987086	0.922921
glutamate_+_tRNA(Glu)_+_ATP_=>_Glu-tRNA(Glu)_+_AMP_+_pyrophosphat	0.991864	0.990207	0.78877
aspartate_+_tRNA(Asp)_+_ATP_=>_Asp-tRNA(Asp)_+_AMP_+_pyrophosphat	0.993971	0.993085	0.78877
snRNP_nuclear_import_and_release	0.352588	0.860811	0.128376
snRNP:Snurportin_complex_formation	0.352588	0.860811	0.128376
snRNA_Cap_hypermethylation	0.216055	0.741303	0.0717486
Syk-mediated_phosphorylation_of_Phospholipase_C_gamma_2	0.0279839	0.109286	0.071425
ADP_and_Pi_bind_to_ATPase	0.971977	0.842721	0.964579
ATP_is_synthesized_from_ADP_and_Pi_by_ATPase	0.971977	0.842721	0.964579
Enzyme-bound_ATP_is_released	0.971977	0.842721	0.964579
NEXT1_is_cleaved_to_produce_NICD1	0.804976	0.890071	0.433608
NEXT2_is_cleaved_to_produce_NICD2	0.804976	0.890071	0.433608
NEXT3_is_cleaved_to_produce_NICD3	0.804976	0.890071	0.433608
NEXT4_is_cleaved_to_produce_NICD4	0.804976	0.890071	0.433608
GP_Ib-IX-V_binds_to_[vWF:Collagen]_complex	0.0553154	0.11524	0.146179
Formation_of_CSL-NICD_coactivator_complex	0.947078	0.847375	0.898174
(ARMS)Rap1-GTP_binds_and_activates_B-Raf	0.0455277	0.0964746	0.140562
Activated_RAF_complex_binds_MEK	0.367197	0.436709	0.354466
RAF_phosphorylates_MEK1	0.367197	0.436709	0.354466
RAF_phosphorylates_MEK2	0.367197	0.436709	0.354466
phospho-ATM_(Serine_1981)_associates_with_DNA_at_the_site_of_double	0.998889	0.429865	0.99983
53BP1_associates_with_gamma_H2AX_at_nuclear_foci	0.998914	0.435336	0.99983
IKKbeta_phosphorylates_IkB_causing_NF-kB_to_dissociate	0.28512	0.430102	0.223747
Recruitment_of_Dna2_endonuclease	0.102271	0.021015	0.937171
Removal_of_RNA_primer_and_dissociation_of_RPA_and_Dna2	0.102271	0.021015	0.937171
Repair_synthesis_for_gap-filling__by_DNA_pol_delta__in_TC-NER	0.0377431	0.0217586	0.719874
Repair_synthesis_of_patch_~27-30_bases_long__by_DNA_Pol_Delta	#N/A	#N/A	#N/A
Coat_Complex_Formation	0.69842	0.91723	0.231601
arginine_+_tRNA(Arg)_+_ATP_=>_Arg-tRNA(Arg)_+_AMP_+_pyrophosphate	0.989294	0.987086	0.78877
glutamine_+_tRNA(Gln)_+_ATP_=>_Gln-tRNA(Gln)_+_AMP_+_pyrophosphat	0.989294	0.987086	0.78877
isoleucine_+_tRNA(Ile)_+_ATP_=>_Ile-tRNA(Ile)_+_AMP_+_pyrophosphate	0.989294	0.987086	0.78877
lysine_+_tRNA(Lys)_+_ATP_=>_Lys-tRNA(Lys)_+_AMP_+_pyrophosphate	0.989294	0.987086	0.78877
snRNP_complex_assembly	0.269194	0.860811	0.0717486
Release_from_the_NPC_and_Disassembly_of_the_mRNP	0.472398	0.907376	0.0369124
Aflatoxin_B1_oxidized_to_Aflatoxin-8,9-oxide	0.647623	0.94524	0.143328
CYP3A4_can_N-demethylate_loperaminde	0.647623	0.94524	0.143328
CYP3A43_catalyzes_the_6beta-hydroxylation_of_testosterone	0.647623	0.94524	0.143328
CYP3A5_oxidises_aflatoxin_B1_to_aflatoxin-8,9-oxide	0.647623	0.94524	0.143328
CYP3A7_can_6beta-hydroxylate_testosterone	0.647623	0.94524	0.143328
CYP4A11_omega-hydroxylates_laurate	0.647623	0.94524	0.143328
CYP4B1_can_12-hydroxylate_arachidonic_acid	0.647623	0.94524	0.143328
CYP4F11_omega-hydroxylates_3-hydroxypalmitate	0.647623	0.94524	0.143328
CYP4F12_hydroxylates_arachidonic_acid	0.647623	0.94524	0.143328
CYP4F2_omega-hydroxylate_leukotriene_B4,_thus_inactivating_it	0.647623	0.94524	0.143328

CYP4F8_hydroxylates_prostaglandin_H2	0.647623	0.94524	0.143328
Dehydrogenation_of_Testosterone_to_form_6-Dehydrotestosterone	0.647623	0.94524	0.143328
Thromboxane_synthase_(CYP5A1)_mediates_the_isomerization_of_prostag	0.647623	0.94524	0.143328
Adhesion_of_alpha_IIb_beta_3_integrin_to_fibrin_network	0.845329	0.758694	0.778233
Glutathione_conjugation_of_cytosolic_substrates	0.0639341	0.268385	0.038939
Binding_of_Syk_tyrosine_kinase	0.0362131	0.14708	0.071425
Type_II_receptor_phosphorylates_type_I_receptor	0.55478	0.425386	0.632552
Assembly_of_EGFR_complex_in_clathrin-coated_vesicles	0.197644	0.430068	0.152385
Activated_Adenylate_cyclase_catalyses_cAMP_synthesis	0.417902	0.763227	0.0849009
Adenylate_cyclase_converts_ATP_into_cyclic_AMP	0.417902	0.763227	0.0849009
Adenylaye_cyclase_increases_the_GTPase_activity_of_G_alpha-olf	0.417902	0.763227	0.0849009
G_alpha_(s)_activates_adenylate_cyclase	0.417902	0.763227	0.0849009
Galpha-olf:GTP_binds_to_adenylate_cyclase_and_activates_it	0.417902	0.763227	0.0849009
The_G_alpha-olf:GDP:Adenylate_cyclase_complex_dissociates	0.417902	0.763227	0.0849009
Cbl_escapes_Cdc42-mediated_inhibition_by_down-regulating_the_adaptor_	0.16806	0.218088	0.286016
Interaction_of_integrin_alpha_8_beta_1_with_Tenascin-c	0.842401	0.734453	0.821731
Interaction_of_integrin_alpha_V_beta_3_with_Tenascin	0.842401	0.734453	0.821731
TORC2_(mTOR)_phosphorylates_AKT_at_S473	0.436553	0.158772	0.920723
ABCA1_tetramer_binds_apoA-I	0.192124	0.203729	0.37985
ABCA1-mediated_transport_of_intracellular_cholesterol_to_the_cell_surfac	0.192124	0.203729	0.37985
ABCA1-mediated_transport_of_intracellular_phospholipid_to_the_cell_surfa	0.192124	0.203729	0.37985
ABCA7:Apo1A-mediated_phospholipid_efflux	0.192124	0.203729	0.37985
CaMK_IV_phosphorylates_CREB	0.149226	0.354605	0.121073
Phosphorylation_of_histone_H2AX_at_Serine-139_by_ATM_at_the_site_of_	0.999044	0.340005	0.99983
Sos-mediated_nucleotide_exchange_of_Ras_(EGF:EGFR-Sos:Grb2)	0.452787	0.599413	0.286982
MDC1/NFBD1associates_with_gamma_H2AX_at_nuclear_foci	0.999149	0.371344	0.99983
Association_of_gamma-H2AX_with_NBS1_	0.997644	0.229308	0.999251
Association_of_beta-catenin_with__the_SCF-beta-TrCP1_ubiquitin_ligase_co	0.999382	0.999602	0.802004
RPA_binds_to_the_Flap	0.188407	0.0490602	0.937171
Ubiquitination_of_phospho-p27/p21	0.380743	0.162831	0.70988
Ca2+_influx_through_voltage_gated_Ca2+_channels	0.868079	0.930011	0.373455
CAK-mediated_phosphorylation_of_Cyclin_B1:Cdc2_complexes	0.330119	0.195176	0.674541
Phosphorylation_of_the_Emi1_DSGxxS_degron_by_Cyclin_B:Cdc2	0.0796853	0.15848	0.153393
Wee1-_mediated_phosphorylation_of_Cyclin_B1:phospho-Cdc2_complexes	0.337972	0.323721	0.482169
The_signal_peptide_is_excised_from_pre-pro-NGF	0.883716	0.992846	0.081182
Recruitment_of_the_Rad9-Hus1-Rad1_complex_to_DNA	0.160667	0.279452	0.13093
Activation_of_IKK_complex	0.518937	0.727539	0.259957
Transport_of_the_export-competent_complex_through_the_NPC	0.521374	0.943019	0.0369124
Interaction_of_integrin_alpha_D_beta_2_with_fibrin	0.975077	0.968116	0.76616
Interaction_of_integrin_alpha_X_beta_2_with_fibrin	0.975077	0.968116	0.76616
glycogen_phosphorylase,_liver_form,_tetramer_b_+_ATP_=>_glycogen_pho	0.908393	0.978891	0.400264
Interaction_of_integrin_alpha_4_beta-1_with_Thrombospondin	0.931561	0.855433	0.856018
betaPIX_pushes_CIN85_away_from_Cbl	0.138125	0.236756	0.1956
Electron_transfer_from_ubiquinol_to_cytochrome_c_of_complex_III	0.566038	0.261338	0.755252
PI3-kinase_binds_to_the_active_receptor	0.520723	0.301761	0.827945
PI3K_catalyses_the_phosphorylation_of_PIP2_to_PIP3	0.520723	0.301761	0.827945
TCR_complex_interacts_with_peptide_antigen-presenting_MHC_Class_I	0.664294	0.695724	0.50993

AKT_phosphorylates_IKKalpha	0.557058	0.272563	0.850167
Notch_1-ligand_complex_is_cleaved_to_produce_NEXT1	0.955254	0.902144	0.864841
Notch_2-ligand_complex_is_cleaved_to_produce_NEXT2	0.955254	0.902144	0.864841
Notch_3-ligand_complex_is_cleaved_to_produce_NEXT3	0.955254	0.902144	0.864841
Notch_4-ligand_complex_is_cleaved_to_produce_NEXT4	0.955254	0.902144	0.864841
AKT_phosphorylates_p21Cip1_and_p27Kip1	0.288237	0.0846572	0.813537
Interaction_of_integrin_alpha_E_beta_7_with_E-cadherin	0.595881	0.713701	0.323684
Stabilisation_of_RAS:RAF_by_14-3-3	0.244462	0.279054	0.354466
Transient_dissociation_of_14-3-3_upon_Ras_binding	0.244462	0.279054	0.354466
Active_IRS_recruits_PI3K_to_the_plasma_membrane_and_activates_it	0.516692	0.402389	0.653706
Sos-mediated_nucleotide_exchange_of_Ras_(PDGF_receptor:Grb2:Sos)	0.504193	0.613865	0.325216
Endocytosis_(internalization)_of_clathrin-coated_vesicle	0.476606	0.632583	0.292521
Activation_of_S6K1	0.479816	0.177784	0.920723
AKT_can_phosphorylate_forkhead_box_transcription_factors	0.562146	0.328032	0.801585
Calmodulin_binds_CaMK_IV	0.048985	0.120464	0.121073
CaMK_IV_autophosphorylation	0.048985	0.120464	0.121073
CamKIV_enters_the_nucleus	0.048985	0.120464	0.121073
Activated_type_I_receptor_phosphorylates_R-Smad1/5/8_directly	0.421924	0.417285	0.479605
An_anchoring_protein,_Endofin,_recruits_R-Smad1/5/8	0.421924	0.417285	0.479605
Phospho-R-Smad1/5/8_dissociates_from_the_receptor_complex	0.421924	0.417285	0.479605
Cbl_binds_and_ubiquitinates_Phospho-Sprouty	0.110785	0.0982137	0.345286
Cbl_ubiquitinates_Sprouty	0.110785	0.0982137	0.345286
Dephosphorylation_of_cyclin_B2:phospho-Cdc2_(Thr_14)_by_Cdc25	0.0977444	0.124054	0.278935
Dephosphorylation_of_cytoplasmic_Cyclin_B1:phospho-Cdc2_(Thr_14,_Tyr_	0.0977444	0.124054	0.278935
Dephosphorylation_of_nuclear_Cyclin_B1:phospho-Cdc2_(Thr_14,_Tyr15)_c	0.0977444	0.124054	0.278935
Phosphorylation_of_the_SA2_Cohesion_Complex	0.787483	0.745916	0.67201
Phosphorylation_of_the_Scc1:Cohesion_Complex	0.787483	0.745916	0.67201
Repair_synthesis_for_gap-filling_by_DNA_pol_epsilon_in_TC-NER	0.148419	0.109469	0.664955
Repair_synthesis_of_~27-30_bases_long_patch__by_DNA_Pol_Epsilon	#N/A	#N/A	#N/A
Recognition_and_Binding_of_Core_Promoter_Elements_by_TFIID	0.19511	0.671568	0.039099
Activation_of_NF-kB_complex	0.382566	0.415481	0.411309
Interaction_between_SRP_and_SRP_Receptor	0.400574	0.853586	0.219104
Formation_of_active_Pol_II_complex_with_lesioned_DNA_template	0.112332	0.572889	0.0349967
Resumption_of_transcription_after_TC-NER	0.112332	0.572889	0.0349967
RNA_Pol_II_is_blocked_by_the_lesion_leading_to_reduced_transcription	0.112332	0.572889	0.0349967
Binding_of_GP_VI:Fc_Epsilon_R1_gamma_receptor_complex_with_collagen	0.0375455	0.184171	0.0590063
STAT_binds_to_the_active_receptor	0.263507	0.214998	0.519899
Interaction_of_integrin_alpha_V_beta_3_with_vitronectin	0.849615	0.736323	0.821731
Interaction_of_integrin_alpha_V_beta_5_with_vitronectin	0.849615	0.736323	0.821731
Basigin_binds_to_integrins	0.376697	0.327864	0.574587
Heterophilic_interaction_of_PECAM-1_and_Integrin_alpha-v_beta-3	0.884269	0.795432	0.821731
Interaction_of_integrin_alpha_5_beta_1_with_Osteopontin	0.7954	0.648668	0.821731
Interaction_of_integrin_alpha_8_beta_1_with_Osteopontin	0.7954	0.648668	0.821731
Integrin_alpha_5_beta_1_binds_fibronectin	0.881402	0.790454	0.821731
Interaction_of_integrin_alpha_8_beta_1_with_Fibronectin	0.881402	0.790454	0.821731
Interaction_of_integrin_alpha_IIb_beta_3_with_Fibronectin	0.881402	0.790454	0.821731
Interaction_of_integrin_alpha_V_beta_1_with_Fibronectin	0.881402	0.790454	0.821731

Interaction_of_integrin_alpha_V_beta_3_with_Fibronectin	0.881402	0.790454	0.821731
Interaction_of_integrin_alpha_V_beta_6_with_Fibronectin	0.881402	0.790454	0.821731
Interaction_of_integrin_alpha_IIb_beta_3_with_van_Willebrand_factor	0.747781	0.576593	0.821731
Interaction_of_integrin_alpha_V_beta_3_with_von_Willbrand_Factor	0.747781	0.576593	0.821731
The_p75NTR:NgR:MDGI_complex_reduces_RHOA-GDI_activity,_displacing_F	0.0100707	0.3626	0.00771257
Adenylate_cyclase_produces_cAMP	0.254288	0.605393	0.0849009
Dephosphorylation_of_PAG_by_SHP2	0.478229	0.454867	0.528919
AKT_phosphorylates_caspase-9	0.345201	0.0807454	0.920723
Activation_of_cytosolic_AMPK_by_phosphorylation	0.641085	0.896169	0.165367
Phosphorylation_of_complexed_TSC2_by_PKB	0.382078	0.0998108	0.920723
AKT_phosphorylates__TSC2,_inhibiting_it	0.349526	0.0828609	0.920723
AKT_phosphorylates_GSK3	0.786841	0.510574	0.920723
Interaction_of_Tie2_and_p85_of_PI3K	0.598762	0.406314	0.793133
Interaction_of_integrin_alpha_V_beta_3_with_Bone_sialoprotein_2	0.851742	0.720996	0.82422
Notch_1_heterodimer_binds_with_a_Notch_ligand_in_the_extracellular_sp	0.985849	0.902144	0.978556
Notch_2_heterodimer_binds_with_a_Notch_ligand_in_the_extracellular_sp	0.985849	0.902144	0.978556
Notch_3_heterodimer_binds_with_a_Notch_ligand_in_the_extracellular_sp	0.985849	0.902144	0.978556
Notch_4_heterodimer_binds_with_a_Notch_ligand_in_the_extracellular_sp	0.985849	0.902144	0.978556
AKT_phosphorylates_MDM2	0.623718	0.29206	0.920723
Phosphorylation_of_proteins_involved_in_the_G1/S_transition_by_Cyclin_A	0.192625	0.260944	0.278352
AKT_phosphorylates_PRAS40	0.345519	0.0809001	0.920723
Phosphorylation_and_activation_of_eIF4G_by_activated_S6K1	0.643147	0.313594	0.920723
ICAMs_1-4_bind_to_Integrin_LFA-1	0.795256	0.808496	0.539122
Binding_of_phospho-p27/p21:Cdk2:Cyclin_E/A_to_the_SCF(Skp2):Cks1_com	0.458827	0.173652	0.786082
CTMP_and/or_TRB3_inhibit_AKT_phosphorylation	0.407618	0.114422	0.920723
PKB_attachment_to_plasma_membrane	0.407618	0.114422	0.920723
Caveolin-1_dissociates_from_eNOS:CaM:HSP90_complex	0.258423	0.302184	0.339388
HSP90_binds_eNOS:Caveolin-1:CaM_complex	0.258423	0.302184	0.339388
AKT_phosphorylates_BAD	0.541618	0.212082	0.920723
PI3K_activation	0.788536	0.62542	0.834701
Gab1:Grb2:PI3K_binds_to_EGF:Phospho-EGFR	0.646356	0.7395	0.383717
Notch_1_precursor_cleaved_to_form_a_heterodimer	0.949042	0.879718	0.883255
Notch_2_precursor_cleaved_to_form_a_heterodimer	0.949042	0.879718	0.883255
Notch_3_precursor_cleaved_to_form_a_heterodimer	0.949042	0.879718	0.883255
Notch_4_precursor_cleaved_to_form_a_heterodimer	0.949042	0.879718	0.883255
Axonal_transport_of_NGF:Trk_complexes	0.478933	0.646936	0.292521
Sos-mediated_nucleotide_exchange_of_Ras_(Tie2_receptor:Grb2:Sos)	0.581864	0.734829	0.280318
Phospho-IKK_Complex_phosphorylates_IkB_within_the_IkB:NFkB_Complex	0.521286	0.519336	0.508513
Removal_of_remaining_Flap	0.142348	0.0248343	0.937171
Multiple_proteins_are_localized_at_replication_fork	0.852397	0.386087	0.961512
Loading_of_PCNA_-_Sliding_Clamp_Formation	0.119854	0.0385711	0.943137
RFC_dissociates_after_sliding_clamp_formation	0.119854	0.0385711	0.943137
Ubiquitination_of_stimulated_EGFR_(Cbl:Grb2)	0.321707	0.445643	0.286016
Cargo,_Sec31p:Sec13p,_and_v-SNARE_recruitment	0.134367	0.230116	0.199222
Vesicle_Budding	0.134367	0.230116	0.199222
Vesicle_Uncoating	0.134367	0.230116	0.199222
Cyclin_B/Cdk1_mediate_phosphorylation_of_NuMA	0.220511	0.284828	0.278935

Phosphorylation_of_Cdh1_by_Cyclin_B1:Cdc2	0.18837	0.18975	0.392873
I-Smad_binds_to_type_I_receptor,_preventing_Smad1/5/8_from_being_act	0.779107	0.581134	0.832453
Autocatalytic_phosphorylation_of_FGFR3b	0.80283	0.869816	0.434346
FGFR3b_binds_to_FGF	0.80283	0.869816	0.434346
Initiation_of_RNA_Polymerase_III_Productive_Transcription	0.760591	0.86334	0.351475
Resumption_of_RNA_Polymerase_III_Productive_Transcription	0.760591	0.86334	0.351475
RNA_Polymerase_III_Productive_Transcription	0.760591	0.86334	0.351475
RNA_Polymerase_III_Retractive_RNase_Activity_at_U-tract_Pause_Sites	0.760591	0.86334	0.351475
RNA_Polymerase_III_Termination_and_release_of_transcribed_mRNA	0.760591	0.86334	0.351475
RNA_Polymerase_III_Transcriptional_Pause_at_Terminator_Sequence	0.760591	0.86334	0.351475
chylomicron_=>_TG-depleted_chylomicron_+_50_long-chain_fatty_acids_+_	0.0556001	0.00924819	0.847708
LILRs_interact_with_MHC_Class_I	0.253944	0.103165	0.830998
Platelet_glycoprotein_IV_[plasma_membrane]_GP_IV_:_Collagen_IV_compl	0.0963426	0.269467	0.102571
MAC1_binds_JAM-C	0.957366	0.972281	0.606467
vWF_binds_to_collagen	0.0398351	0.11164	0.102571
Integrin_alphaX_beta2_binds_JAM-C	0.947311	0.909087	0.825121
alpha-tubulin:GTP:Cofactor_B__+Cofactor_E_->__alpha-tubulin:GTP:_Cofact	0.103215	0.12705	0.292266
Association_of_Rb_with_Cyclin_E:Cdk2_complexes	0.448102	0.367917	0.550172
Cyclin_E:Cdk2-mediated_phosphorylation_of_Rb	0.448102	0.367917	0.550172
AKT_can_phosphorylate_NUR77	0.249769	0.0340151	0.920723
Transport_(efflux)_of_bile_salts_by_ABCC3_(MRP3)	0.445856	0.541883	0.240063
Surface_deployment_of_alpha_IIb_beta_3_integrin	0.844135	0.720996	0.821731
Phosphorylation_and_inactivation_of_eEF2K_by_activated_S6K1	0.469059	0.1209	0.883226
Guanine_nucleotide_exchange_on_Ral	0.768631	0.741297	0.64673
Activation_of_SHP2_through_the_binding_to_phospho-Gab1	0.477105	0.451342	0.528919
Dephosphorylation_of_Gab1_by_SHP2	0.477105	0.451342	0.528919
SHP2_dephosphorylates_Tyr_992_on_EGFR	0.477105	0.451342	0.528919
Phosphorylation_of_TSC2_by_PKB	0.37432	0.0822052	0.920723
PDK1_phosphorylates_AKT_at_T308	0.442462	0.119947	0.920723
Phosphorylation_of_PKB_by_PDK1	0.442462	0.119947	0.920723
PIP3_recruits_PDK1_and_AKT_to_the_membrane	0.442462	0.119947	0.920723
AKT_phosphorylates_CREB	0.716041	0.382573	0.920723
Autocatalytic_phosphorylation_of_FGFR2b	0.972139	0.972139	#N/A
p130Cas_and_C3G_bind_PDGFR_bound_Crk	0.174765	0.266277	0.230282
Phosphorylation_and_activation_of_eIF4B_by_activated_S6K1	0.537507	0.1209	0.926307
Akt1_phosphorylates_BAD_protein	0.578168	0.224656	0.920723
CAK-mediated_phosphorylation_of_Cyclin_E:Cdk2	0.657765	0.198416	0.90358
Inactivation_of_PDC_by_phosphorylation_of_PDC_E1_alpha_component	0.897472	0.917701	0.534594
Phosphorylation_of_Ribosomal_protein_S6_by_activated_S6K1	0.45664	0.1209	0.873769
Interaction_of_integrin_alpha_9_beta_1_with_tenascin-c	0.860287	0.759545	0.894054
PI3K_produces_PIP3_and_other_phosphatidyl_inositides	0.713364	0.500312	0.834701
Phosphorylation_of_Cyclin_E:Cdk2_complexes	0.66687	0.387785	0.832603
Formation_of_Okazaki_fragments	0.159231	0.0255425	0.937171
Formation_of_Processive_Complex	0.159231	0.0255425	0.937171
Formation_of_the_Flap_Intermediate	0.159231	0.0255425	0.937171
Phosphorylation_of_EGFR_by_SRC_kinase	0.196728	0.22208	0.345932
RFC_binding_displaces_Pol_Alpha	0.116749	0.0340867	0.943137

RFC_binding_displaces_Pol_Alpha_on_the_C-strand_of_the_telomere	0.116749	0.0340867	0.943137
Ubiquitination_of_stimulated_EGFR_(Cbl)	0.149118	0.186631	0.286016
Free_CDT1_associates_with_CDC6:ORC:origin_complexes	0.139104	0.0724189	0.501325
Formation_of_Cyclin_B:Cdc2_complexes	0.146586	0.18975	0.278935
Myt-1_mediated_phosphorylation_of_Cyclin_B:Cdc2_complexes	0.146586	0.18975	0.278935
Phosphorylation_of_Cyclin_B1_in_the_CRS_domain	0.146586	0.18975	0.278935
Phosphorylation_of_M_phase_proteins_by_active_Cyclin_B1:Cdc2_complex	0.146586	0.18975	0.278935
Translocation_of_CRS_phosphorylated_Cyclin_B1:Cdc2_complexes	0.146586	0.18975	0.278935
Translocation_of_Cyclin_B1:phospho-Cdc2_complexes_to_the_nucleus	0.146586	0.18975	0.278935
Translocation_of_Cyclin_B1:phospho-Cdc2_to_the_cytoplasm	0.146586	0.18975	0.278935
Sos:Grb2_binds_to_Phospho-Shc:EGF:Phospho-EGFR	0.68482	0.774992	0.406799
Basigin_binds_Mannose-carrying_cell_recognition_molecules	0.366955	0.650939	0.127648
Recruitment_of_Dna2_endonuclease_to_the_C_strand	0.0685227	0.0372488	0.719874
Removal_of_RNA_primer_and_dissociation_of_RPA_and_Dna2_from_the_C	0.0685227	0.0372488	0.719874
SOS_mediated_nucleotide_exchange_of_RAS_(SHC)	0.70009	0.906211	0.169634
SOS_mediated_nucleotide_exchange_of_RAS_(IRS)	0.697566	0.824973	0.334936
Phoshorylation_of_PECAM-1_by_Fyn_or_Lyn_or_c-Src	0.560462	0.586974	0.464388
Sculpting_and_pinching-off_of_Golgi_vessicle	0.996299	0.997398	0.674323
Melanocortin_receptors_bind_melanocortins	0.948856	0.949555	0.602429
eIF2_activation	0.923595	0.956028	0.592962
Formation_of_eIF2:GDP:eIF2B_intermediate	0.923595	0.956028	0.592962
Activation_of_TAK1-TAB2_complex	0.335923	0.464113	0.296241
Association_of_NuMA_with_microtubules	0.847942	0.637236	0.8744
Ubiquitination_of_Emi1_by_SCF-beta-TrCP	0.95602	0.995907	0.468988
Association_of_CCT/TriC_with_sphingosine_kinase_1	0.531729	0.393995	0.612975
KIR2DS1_interacting_with_HLA-C_group_2_(Cw3)	0.66437	0.33092	0.945669
NKG2A-CD94_heterdimer_interacts_with_HLA-E	0.149354	0.311553	0.10991
alpha-tubulin:GTP_+_Cofactor_B_->_alpha-tubulin:GTP:_Cofactor_B	0.0828096	0.099344	0.292266
alpha-tubulin:GTP_+_Cofactor_E_->__alpha-tubulin:GTP:Cofactor_E	0.0846828	0.12705	0.192319
PKA_phosphorylates_PDE4B	0.51608	0.479396	0.598459
Activated_type_I_receptor_phosphorylates_R-SMAD_directly	0.704215	0.823952	0.431402
An_anchoring_protein,_SARA,_recruits_R-SMAD	0.704215	0.823952	0.431402
Phospho-R-SMAD_dissociates_from_the_receptor_complex	0.704215	0.823952	0.431402
Activation_of_BAD_by_calcineurin	0.911407	0.864726	0.782167
LFA1_binds_JAM-A	0.987429	0.972624	0.894054
DARPP-32_is_dephosphorylated_on_Thr34_by_PP2B	0.731838	0.600724	0.731767
gamma-secretase_cleaves_the_p75NTR_transmembrane_domain	0.320132	0.866985	0.0380599
Autocatalytic_phosphorylation_of_Klotho-bound_FGFR1c	0.822758	0.947741	0.279341
FGFR1c_binds_to_Klotho-bound_FGF23	0.822758	0.947741	0.279341
Localization_of_Cbl:Grb2_to_the_membrane	0.396888	0.494834	0.340612
Phosphorylation_of_Cbl_(EGFR:Cbl:Grb2)	0.396888	0.494834	0.340612
Calcium_Influx_through_Voltage-gated_Calcium_Channels	0.358638	0.428381	0.35984
AKT_can_phosphorylate_SRK	0.475614	0.1209	0.920723
AKT_translocates_to_the_nucleus	0.475614	0.1209	0.920723
PHLPP_dephosphorylates_S473_in_AKT	0.475614	0.1209	0.920723
Dephosphorylation_of_Cyclin_E:Cdk2_complexes_by_Cdc25A	0.291705	0.11109	0.751622
Mcm2-7_is_phosphorylated_by_DDK	0.813579	0.641746	0.83766

Activation_of_Src	0.227384	0.235208	0.4031
SH2_domain_of_Src_binds_to_the_active_receptor	0.227384	0.235208	0.4031
PI3K_phosphorylates_PIP2_to_PIP3	0.79315	0.669206	0.809165
Recruitment_of_PI3K_to_plasmamembrane	0.79315	0.669206	0.809165
(Frs2)Rap1-GTP_binds_to_and_activates_B-Raf	0.129265	0.183229	0.248475
Phosphorylation_of_Cyclin_D:Cdk4/6_complexes	0.363652	0.126075	0.74261
Dissociation_of_beta-catenin_from_Axin_and_association_of_beta_catenin_	0.899836	0.916373	0.566558
Phosphoryation_of_phospho-_(Ser45,_Thr41)_beta-catenin__at_Ser37_by_(	0.899836	0.916373	0.566558
Phosphorylation_of_APC_component_of_the_destruction_complex	0.899836	0.916373	0.566558
Phosphorylation_of_beta-catenin_at_Ser45_by_CK1_alpha	0.899836	0.916373	0.566558
Phosphorylation_of_phospho-(Ser45_)_at_Thr_41_by_GSK-3	0.899836	0.916373	0.566558
Phosphorylation_of_phospho-(Ser45,Thr41,Ser37)_at_Ser33_by_GSK-3	0.899836	0.916373	0.566558
3,5,3-triiodothyronine_+_PAPS_=>_3,5,3-triiodothyronine_4-sulfate_+_PAP	0.00187041	0.00354144	0.079818
Phosphorylation_of_Cyclin_E2:Cdk2_complexes_by_Myt1	0.610791	0.387785	0.795683
Phosphorylation_of_Cyclin_E1:Cdk2_complexes_by_Myt1	0.569767	0.387785	0.743429
L-Glutamate_loading_of_synaptic_vesicle	0.139292	0.289581	0.164075
Recruitment_of_ITK_to_SLP-76	0.659494	0.547165	0.697454
CDC6_association_with_ORC:origin_complexes_mediated_by_MCM8	0.141604	0.0724189	0.551491
Association_of_Cyclin_A:phospho-Cdk2(Thr_160)_with_E2F1/E2F3	0.0448636	0.0984682	0.133682
Phosphorylation_of_E2F1/E2F3_by_Cyclin_A:phosph-Cdk2(Thr_160)	0.0448636	0.0984682	0.133682
RPA_binds_to_the_Flap_on_the_C-strand	0.148644	0.0909449	0.719874
Proteolysis_of_the_IGF:IGFBP-3:ALS_Complex_by_Matrix_Metalloproteinase	0.27143	0.72717	0.107791
Recruitment_of_Rad17-RFC_complex_to_DNA	0.021376	0.0391461	0.141902
Binding_of_IRS_to_insulin_receptor	0.955772	0.989669	0.417957
Dissociation_of_IRS-P_from_insulin_receptor	0.955772	0.989669	0.417957
Phosphorylation_of_IRS	0.955772	0.989669	0.417957
Neuropeptide_Y_receptors_can_bind_neuropeptide_Y-related_peptides	0.53874	0.766028	0.379192
Docking_of_the_Mature_intronless_derived_transcript_derived_mRNA,_TAI	0.867381	0.990957	0.108713
Trace_amine-associated_receptors_can_bind_trace_amines	0.771785	0.840104	0.567582
nascent_chylomicron_+_spherical_HDL:apoC-II:apoC-III:apoE_=>spherical__I	0.0923074	0.0220134	0.922277
TG-depleted_chylomicron_+_spherical_HDL_=>_chylomicron_remnant_+_sp	0.0923074	0.0220134	0.922277
Creation_of_the_Membrane_Attack_Complex_(MAC)	0.890583	0.967234	0.104248
Release_of_Tubulin_alpha-4A_chain	0.064427	0.099344	0.192319
Glutathione_conjugation_of_luminal_substrates	0.628042	0.363076	0.877635
MHC_Class_I_interacts_with_CD160	0.547301	0.414979	0.712466
KIR2DL1_interacting_with_HLA-C_group_2_(Cw3)	0.339501	0.33092	0.490238
KIR2DL2/3_interacting_with_HLA-C_group_1_(Cw4)	0.339501	0.33092	0.490238
KIR2DL4_interacting_with_HLA-G	0.339501	0.33092	0.490238
KIR3DL1_interacting_with_HLA_Bw4	0.339501	0.33092	0.490238
KIR3DL2_interacting_with_HLA-A3	0.339501	0.33092	0.490238
NGF_binding_to_p75NTR_inactivates_RHOA	0.0714558	#N/A	0.0714558
eNOS:Caveolin-1:NOSTRIN:dynamin-2_complex_binds_N-WASP	0.511094	0.486086	0.542279
Interaction_of_integrin_alpha_4_beta-1_with_Osteopontin	0.815375	0.676921	0.894054
Interaction_of_integrin_alpha_9_beta_1_with_Osteopontin	0.815375	0.676921	0.894054
Interaction_of_integrin_alpha_4_beta-1_with_Fibronectin	0.907274	0.821081	0.894054
eNOS:Caveolin-1_complex_binds_to_CaM	0.362689	0.302184	0.524333
VLA-4_binds_JAM-B	0.936969	0.873192	0.894054

Interaction_of_integrin_alpha_9_beta_1_with_VCAM-1	0.976804	0.949558	0.894054
VCAM-1interacts_with_VLA-4	0.976804	0.949558	0.894054
LTC4_is_exported_from_the_cell	0.319543	0.41357	0.240063
VEGF-A,C,D,E_bind_to_VEGFR2_leading_to_receptor_dimerization	0.0880506	0.235739	0.0819458
ERKs_are_inactivated_by_dual-specific_phosphatases_(DUSPs)	0.644838	0.573801	0.700286
binding_of_Cbl_to_EGFR	0.182584	0.199768	0.340612
Phosphorylation_of_Cbl_(EGFR:Cbl)	0.182584	0.199768	0.340612
Ral-GDS_binds_to_Ras-GTP	0.509115	0.335103	0.700458
Association_of_DFF_with_alpha:beta_importin	0.293581	0.389687	0.226486
Translocation_of_DFF_to_the_nucleus	0.293581	0.389687	0.226486
MadCAM_interacts_with_Integrin_alpha-4_beta-7	0.90971	0.825234	0.894054
factor_XI:platelet_glycoprotein_(GP)_Ib:IX:V_complex_->_factor_XIa:platele	0.319658	0.46324	0.294405
Caspase-mediated_cleavage_of_gelsolin	0.370992	0.172244	0.582442
factor_IX_->_factor_IXa_+_factor_IX_activation_peptide_(factor_XIa_catalys	0.0507637	0.0302724	0.294405
I-SMAD_competes_with_R-SMAD_for_type_I_receptor	0.237019	0.185562	0.431402
Activation_of_Rap1	0.164544	0.214376	0.282678
cAMP_induces_dissociation_of_inactive_PKA_tetramers	0.810675	0.810675	#N/A
Gab1_binds_phosphatidylinositol-3,4,5-trisphosphate	0.541503	0.538057	0.517116
Gab1_phosphorylation_by_EGFR_kinase	0.541503	0.538057	0.517116
Gab1:Grb2_binds_to_EGF:Phospho-EGFR	0.541503	0.538057	0.517116
factor_XI:platelet_glycoprotein_(GP)_Ib:IX:V_complex_->_factor_XIa:platele	0.513563	0.750215	0.294405
RIT/RIN-GTP_binds_B-Raf	0.141935	0.101069	0.504496
CAK-mediated_phosphorylation_of_Cyclin_A:Cdc2_complexes	0.488626	0.198416	0.824807
CAK-mediated_phosphorylation_of_Cyclin_A:Cdk2	0.488626	0.198416	0.824807
Shc_binds_to_the_phospho-receptor:ligand_complex	0.314286	0.331346	0.406799
Shc_phosphorylation_by_phospho-EGFR:EGF	0.314286	0.331346	0.406799
BMP2_binds_to_the_receptor_complex	0.781486	0.658076	0.7629
3,3-diiodothyronine_+_PAPS_=>_3,3-diiodothyronine_4-sulfate_+_PAP	0.00107766	0.00354144	0.0575292
p75NTR_ICD_signals_to_NF-kB	0.290646	0.295142	0.428156
Basigin_binds_CD98_complex	0.257527	0.864679	0.0347023
Association_of_MCM8_with_ORC:origin_complex	0.112226	0.0724189	0.512835
C3G_stimulates_nucleotide_exchange_on_Rap1	0.0225969	0.110362	0.049997
The_ligand_trap_binds_the_ligand_BMP2,_blocking_BMP_signalling	0.125225	0.0565593	0.360328
(Frs2)C3G_stimulates_nucleotide_exchange_on_Rap1	0.0526286	0.236329	0.049997
Sos:Grb2_complex_binds_to_EGF:EGFR_complex	0.659842	0.677124	0.517116
TRAF6:Phosho-TAK1:Tab1:PhosphoTAB2_mediated_phosphorylation_of_MA	0.854163	0.889414	0.514741
Myt-1_mediated_phosphorylation_of_Cyclin_A:Cdc2	0.499894	0.387785	0.673317
Phosphorylation_of_Cyclin_A:Cdk2_at_Tyr_15	0.499894	0.387785	0.673317
Wee1-mediated_phosphorylation_of_Cyclin_A:phospho-Cdc2_complexes	0.499894	0.387785	0.673317
SOS_phosphorylation_and_dissociation_(SHC)	0.972394	0.988156	0.319284
p62_recruits_an_atypical_PKC	0.320495	0.556165	0.136559
SOS_phosphorylation_and_dissociation_(IRS)	0.971869	0.969869	0.672364
Coat_Assembly	0.308263	0.582957	0.199222
Loss_of_Sar1p_GTPase	0.308263	0.582957	0.199222
IKK-beta_is_recruited	0.447856	0.783869	0.134013
IKKbeta_is_activated	0.447856	0.783869	0.134013
Phosphorylated_Emi1_binds_the_beta-TrCP_in_the_SCF_complex	0.986385	0.99938	0.548643

Nrage_sequesters_Che1_in_the_cytoplasm	0.395246	0.927933	0.0529219
Caspase-mediated_cleavage_of_Desmoglein_1	0.365342	0.487393	0.202313
Caspase-mediated_cleavage_of_Desmoglein_2	0.365342	0.487393	0.202313
Caspase-mediated_cleavage_of_Desmoglein_3	0.365342	0.487393	0.202313
Phosphorylation_of_JNK_by_the_Activated_TRAF6:Phospho-TAK1:TAB1:Pho	0.693944	0.789655	0.437896
Myelin_components_can_interact_with_p75NTR:NgR:LINGO1	0.0152398	0.568462	0.00129941
TRAF6:Phosho-TAK1:Tab1:PhosphoTAB2_mediated_phosphorylation_of_the	0.800042	0.898042	0.427629
SOS_phosphorylation_and_dissociation_(IRS,_Crk)	0.87596	0.906131	0.530529
Binding_of_SNAPc,_Oct-1,_and_Staf_to_Type_3_Promoter	0.586126	0.441194	0.69223
Beta-tubulin:GTP:_Cofactor_A+_Cofactor_D_->__Beta-tubulin:GTP:Cofactor_	0.955901	0.955571	0.633605
Granzyme-B_activates_BID_by_cleavage	0.433684	0.218976	0.738814
Binding_of_SHC_to_insulin_receptor	0.934512	0.995785	0.230741
Dissociation_of_SHC-P_from_insulin_receptor	0.934512	0.995785	0.230741
Phosphorylation_of_SHC	0.934512	0.995785	0.230741
Signal_Recognition_(Preproinsulin)	0.559786	0.940811	0.316974
Binding_of_Rrn3_to_RNA_Polymerase_I	0.919701	0.935153	0.652423
Acetylcholine_clearance_from_synaptic_cleft	0.840272	0.451393	0.936836
Exocytosis_of_alpha_1_antitrypsin	0.869623	0.731971	0.834032
Conversion_of_C4_into_C4a_and_C4b	0.880568	0.857457	0.688987
kallikrein_+_alpha2-macroglobulin_->_kallikrein:alpha2-macrogloulin	0.660318	0.854717	0.317979
CYP1B1_4-hydroxylates_estradiol-17beta	0.125545	0.605071	0.0554156
CYP2D6_4-hydroxylates_debrisoquine	0.125545	0.605071	0.0554156
CYP2W1_can_oxidize_indole	0.125545	0.605071	0.0554156
Dehydrogenation_of_Sparteine_to_form_2-Dehydrosparteine	0.125545	0.605071	0.0554156
Activation_of_C5	0.971474	0.976207	0.590813
Cleavage_of_C3_by_C3_convertase	0.971474	0.976207	0.590813
prekallikrein:kininogen:C1q_binding_protein_tetramer_->_kallikrein:kininog	0.0741714	0.105237	0.231915
4-cholesten-7alpha,_12alpha-diol-3-one_is_reduced_to_5beta-cholesten-7a	0.065522	0.0297711	0.519512
4-cholesten-7alpha,12alpha,24(S)-triol-3-one_is_reduced_to_5beta-cholesta	0.065522	0.0297711	0.519512
4-cholesten-7alpha,12alpha,27-triol-3-one_is_reduced_to_5beta-cholestan-7	0.065522	0.0297711	0.519512
4-cholesten-7alpha,24(S)-diol-3-one_is_reduced_to_5beta-cholestan-7alpha	0.065522	0.0297711	0.519512
4-cholesten-7alpha,27-diol-3-one_is_reduced_to_5beta-cholestan-7alpha,27	0.065522	0.0297711	0.519512
4-cholesten-7alpha-ol-3-one_is_reduced_to_5beta-cholestan-7alpha-ol-3-or	0.065522	0.0297711	0.519512
Reduction_of_isocaproaldehyde_to_4-methylpentan-1-ol	0.065522	0.0297711	0.519512
Release_of_Kappa-actin	0.934153	0.943456	0.826755
SHP2_binds_to_the_active_receptor	0.252193	0.146705	0.628842
p75NTR_and_RHOA-GDI_interact	0.111811	#N/A	0.111811
p75NTR_reduces_RHO-GDI_activity	0.111811	#N/A	0.111811
Homodimerization_of_VEGF_proteins	0.0465601	0.191403	0.0153815
Replication_initiation_regulation_by_Rb1/E2F1	0.183805	0.139037	0.424755
ERK1/2/5_activate_RSK1/2/3	0.657807	0.657807	#N/A
Inactive_catalytic_PP2B_is_activated_by_the_binding_of_calmodulin	0.473536	0.342399	0.622216
Nck_binds_to_the_active_PDGF_receptor	0.789972	0.75918	0.655421
pAMPK_inactivates_ACC2_inhibiting_malonyl-CoA_synthesis	0.101963	0.204686	0.165367
Activation_of_PLC_beta-1/4	0.640599	0.860631	0.181712
Inactivation_of_PLC_beta	0.640599	0.860631	0.181712
PIP2_hydrolysis	0.640599	0.860631	0.181712

Formation_of_clathrin-coated_vesicle	0.70509	0.789948	0.417809
NICD1_trafficks_to_the_nucleus	0.937741	0.732821	0.962425
NICD2_trafficks_to_the_nucleus	0.937741	0.732821	0.962425
NICD3_trafficks_to_the_nucleus	0.937741	0.732821	0.962425
NICD4_trafficks_to_the_nucleus	0.937741	0.732821	0.962425
Notch_1_heterodimer_trafficks_to_the_plasma_membrane	0.937741	0.732821	0.962425
Notch_1_precursor_transport_to_golgi	0.937741	0.732821	0.962425
Notch_2_heterodimer_trafficks_to_the_plasma_membrane	0.937741	0.732821	0.962425
Notch_3_heterodimer_trafficks_to_the_plasma_membrane	0.937741	0.732821	0.962425
Notch_4_heterodimer_trafficks_to_the_plasma_membrane	0.937741	0.732821	0.962425
Proteolysis_of_the_IGF:IGFBP-3:ALS_Complex_by_Cathepsin_L	0.176624	0.356134	0.138658
Formation_of_Cyclin_E:Cdk2_complexes	0.433257	0.179311	0.751622
Translocation_of_Cyclin_E:Cdk2_complex__to_the_nucleus	0.433257	0.179311	0.751622
Phosphorylation_of_ChREBP_at_Serine_568_by_AMPK	0.352529	0.756039	0.23406
Cleavage_of_Procaspase-7_by_the_apoptosome	0.298881	0.105952	0.692064
Cleavage_of_ Procaspase-3_by_the_apoptosome	0.298881	0.105952	0.692064
Association_of_INK4A_with_Cdk4/6	0.0259431	0.29547	0.0227039
PEX-19_docks_ABCD1/ABCD3_to_peroximal_membrane	0.241518	0.241518	#N/A
Caspase_mediated_cleavage_of_alpha-II-Fodrin	0.100502	0.100502	#N/A
Creatine_transport_across_the_plasma_membrane	0.886017	0.886017	#N/A
SLC6A12_(BGT-1)-mediated_uptake_of_GABA_and_betaine	0.886017	0.886017	#N/A
SLC6A6-mediated_uptake_of_taurine_and_beta-alanine	0.886017	0.886017	#N/A
phospho-RAF_binds_14-3-3	0.37299	0.268754	0.62172
Active_PLC-gamma1_dissociates_from_EGFR	0.173803	0.118791	0.517116
EGFR_activates_PLC-gamma1_by_phosphorylation	0.173803	0.118791	0.517116
Phospholipase_C-gamma1_binds_to_the_activated_EGF_receptor	0.173803	0.118791	0.517116
14-3-3epsilon_attentuates_NADE-related_apoptosis	0.20738	0.265193	0.28931
MCM2-7_mediated_fork_unwinding	0.72844	0.449854	0.83766
Mcm4,6,7_trimer_forms_and_associates_with_the_replication_fork	0.72844	0.449854	0.83766
Fc_gamma_receptors_interact_with_antigen-bound_IgG	0.710897	0.400101	0.850435
p75NTR:NADE_promotes_caspase2/3_activation	0.0159088	0.0318756	0.136559
G_Protein_trimer_formation_(olfactory)	0.456958	0.456958	#N/A
Activation_of_Rac1	0.576461	0.49245	0.614385
Grb2/Sos1_complex_binds_to_the_active_receptor	0.734653	0.693989	0.655421
Association_of_p21/p27_with_Cyclin_E/Cdk2_complexes	0.101107	0.120253	0.291475
Cyclin_E/A:Cdk2-mediated__phosphorylation_of_p27/p21	0.101107	0.120253	0.291475
Inactivation_of_Cyclin_A:Cdk2_complexes_by_p27/p21	0.101107	0.120253	0.291475
Inactivation_of_Cyclin_E:Cdk2_complexes_by_p27/p21	0.101107	0.120253	0.291475
LKB1_phosphorylates_the_alpha_subunit_of_AMPK_heterotrimer	0.376265	0.796243	0.165367
Crk_binds_to_the_active_PDGF_receptor	0.431847	0.404473	0.517443
Cdc25A_mediated_dephosphorylation_of_Cyclin_A:phospho-Cdk2	0.122718	0.11109	0.453813
Dephosphorylation_of_nuclear_Cyclin_A:phosph-Cdc2_complexes	0.122718	0.11109	0.453813
Caspase-mediated_cleavage_of_Etk	0.300129	0.231544	0.551451
DNA_polymerase_alpha:primase_binds_at_the_origin	0.516054	0.169433	0.92231
The_primase_component_of_DNA_polymerase:primase_synthesizes_a_6-10	0.516054	0.169433	0.92231
The_primase_component_of_DNA_polymerase:primase_synthesizes_a_6-10	0.516054	0.169433	0.92231
Orc6_associates_with_Orc1:Orc4:Orc5:Orc3:Orc2:origin_complexes,_formin	0.196603	0.142566	0.512835

GTP-bound_RAC_contributes_to_JNK_activation	0.708651	0.626902	0.674468
Assembly_of_the_destruction_complex	0.93586	0.946721	0.545865
Association_of_beta-catenin_with_the_destruction_complex	0.93586	0.946721	0.545865
NRP-1_forms_a_ternary_complex_with_VEGF165_and_VEGFR1	0.310218	0.260511	0.476443
NRAGE_activates_JNK	0.392082	0.756017	0.139735
Caspase-mediated_cleavage_of_E-Cadherin	0.129963	0.35991	0.0743934
POL_delta_associates_with_AP_site_displacing_POL_Beta	0.180333	0.0900967	0.622235
p-nitrophenol_+_PAPS_=>_p-nitrophenol_sulfate_+_PAP	0.00584774	0.00354144	0.288386
Phosphorylation_of_4E-BP1_by_activated_mTORC1	0.752874	0.759707	0.538172
Vamp7_associated_Lysosomal_vesicle_docking_and_fusion	0.314925	0.0471733	0.936963
Inactivation_of_MEK1_by_p34cdc2	0.39781	0.408177	0.453813
VEGF-A,B,PLGF_bind_to_VEGFR1_leading_to_receptor_dimerization	0.244243	0.19908	0.575906
factor_XI_+_platelet_glycoprotein_(GP)_Ib:IX:V_complex_->_factor_XI:plate	0.26608	0.375214	0.294405
cytidine_+_ATP_=>_cytidine_5-monophosphate_(CMP)_+_ADP	0.496804	0.578724	0.383782
Ligation_of_DNA_at_sites_of_patch_replacement	0.0880091	0.03021	0.622235
Phosphorylation_of_the_Emi1_DSGxxS_degron_by_Plk1	0.221631	0.120548	0.534923
Activation_and_Release_of_the_TRAF6:Phospho-Tak1:Tab1:Phospho-Tab2_(	0.518955	0.405593	0.619935
Formation_of_the_Viral_dsRNA:TLR3:TRIF:TRAF6:TAB1:TAB2_Complex	0.518955	0.405593	0.619935
2-(alpha-hydroxyethyl)-TPP_+_lipoamide_=>_S-acetyldihydrolipoamide_+_Tl	0.500002	0.598661	0.287996
dihydrolipoamide_+_FAD_=>_lipoamide_+_FADH2_[pyruvate_dehydrogena:	0.500002	0.598661	0.287996
FADH2_+_NAD+_=>_FAD_+_NADH_+_H+_[pyruvate_dehydrogenase]	0.500002	0.598661	0.287996
pyruvate_+_TPP_=>_2-(alpha-hydroxyethyl)-TPP_+_CO2	0.500002	0.598661	0.287996
S-acetyldihydrolipoamide_+_CoA_=>_acetyl-CoA_+_dihydrolipoamide	0.500002	0.598661	0.287996
G-protein_alpha_subunit_is_inactivated	0.154817	0.186164	0.310611
Removal_of_remaining_Flap_from_the_C-strand	0.0909967	0.0424377	0.719874
A_ligand:p75NTR_complex_binds_to_Nrage	0.375654	0.927933	0.0281087
Loading_of_PCNA_-_Sliding_Clamp_Formation_on_the_C-strand_of_the_tel	0.0706051	0.0706051	#N/A
RFC_dissociates_after_sliding_clamp_formation_on_the_C-strand_of_the_te	0.0706051	0.0706051	#N/A
Phosphorylation_of_DLC1_by_MAPK_8	0.451525	0.911714	0.0169145
Arf1_Activation_by_GBF1	0.107261	0.33476	0.11151
trans-Golgi_Network_Coat_Activation	0.107261	0.33476	0.11151
Cleavage_of__flap_structures	0.0910863	0.0318392	0.622235
Opioid_receptors_bind_opioid_peptides	0.380665	0.487085	0.345402
Inactivation_of_Myt1_kinase	0.736193	0.387274	0.932848
Inactivation_of_Wee1_kinase	0.736193	0.387274	0.932848
Activation_of_SRC_by_Ral-GTP	0.68988	0.855232	0.26094
Phosphorylation_of_DLC2_by_MAPK-8	0.296183	0.851488	0.035892
Binding_of_TFIIIC_to_TFIIIA:Type_I_Promoter_complex_	0.570183	0.397394	0.749487
Beta-tubulin:GTP_+_Cofactor_D_->__Beta-tubulin:GTP:_Cofactor_D	0.889713	0.882678	0.633605
Docking_of_Mature_Replication_Dependent_Histone_mRNA_with_the_NPC	0.55006	0.920801	0.108713
Inhibitory_effect_of_Grb10_on_insulin_signalling	0.954982	0.994869	0.432025
phosphatidylinositol_+_UDP-N-acetyl-D-glucosamine_->_N-acetylglucosamir	0.0472035	#N/A	0.0472035
Beta-tubulin:GTP_+_Cofactor_A_->__Beta-tubulin:GTP:_Cofactor_A	0.955571	0.955571	#N/A
Somatostatin_receptors_bind_somatostatin	0.945173	0.992513	0.4316
Assembly_of_RNA_Polymerase_I_Holoenzyme_(mouse)	0.286886	0.839068	0.0822383
cholesterol_ester_+_H2O_->_cholesterol_+_fatty_acid	0.86403	0.844468	0.689661
diacylglycerol_+_H2O_->_2-acylglycerol_+_fatty_acid	0.86403	0.844468	0.689661

phosphorylated_HSL_dimer_+_FABP4_->_phosphorylated_HSL_dimer:FABP4	0.86403	0.844468	0.689661
triacylglycerol_+_H2O_->_diacylglycerol_+_fatty_acid	0.86403	0.844468	0.689661
Factor_D_cleaves_C3(H2O)-bound_Factor_B	0.569229	0.814217	0.206298
Factor_D_cleaves_C3b-bound_Factor_B	0.569229	0.814217	0.206298
CYP2C18_initiates_bioactivation_of_phenytoin_by_4-hydroxylation	0.0519631	0.099618	0.158971
CYP2C19_5-hydroxylates_omeprazole	0.0519631	0.099618	0.158971
CYP2C8_inactivates_paclitaxel_by_6alpha-hydroxylation	0.0519631	0.099618	0.158971
CYP2C9_inactivates_tolbutamide_by_4methyl-hydroxylation	0.0519631	0.099618	0.158971
Formation_of_alternate_C5_convertase	0.782216	0.775234	0.590813
Properdin_stabilizes_C3b:Bb_bound_to_cell_surfaces	0.782216	0.775234	0.590813
ApoB-48_+_40_triacylglycerol_+_60_phospholipid_=>_ApoB-48:TG:PL_comp	0.0581153	0.0684285	0.254096
factor_XII_->_factor_XIIa	0.624719	0.843238	0.231915
cytosolic_GK:GKRP_complex_<=>_glucokinase_(GK)_+_glucokinase_regulato	0.221574	0.221574	#N/A
GK:GKRP_[cytosol]_=>_GK:GKRP_[nucleoplasm]	0.221574	0.221574	#N/A
glucokinase_(GK)_+_glucokinase_regulatory_protein_(GKRP)_<=>_GK:GKRP_	0.221574	0.221574	#N/A
nucleoplasmic_GK:GKRP_complex_=>_glucokinase_(GK)_+_glucokinase_regu	0.221574	0.221574	#N/A
hormone-sensitive_lipase_(HSL)_+_2_ATP_->_phosphorylated_HSL_+_2_AD	0.982383	0.975367	0.780397
Calmodulin_activates_Cam-PDE_1	0.263814	0.202879	0.598459
cAMP_hydrolysis_by_Cam-PDE_1	0.263814	0.202879	0.598459
cAMP_hydrolysis_by_PDE_4	0.263814	0.202879	0.598459
Type_II_receptor_recruits_type_I_receptor	0.239786	0.108405	0.431402
PKC_phosphorylates_GRK2	0.497454	0.508864	0.476207
Grb7_binds_to_the_active_PDGF_receptor	0.158947	0.0938839	0.655421
chylomicron_remnant:apoE_complex_+_LDLR_=>_chylomicron_remnant:ap	0.629216	0.629216	#N/A
chylomicron_remnant:apoE:LDLR_complex_=>_chylomicron_remnant:apoE_	0.629216	0.629216	#N/A
chylomicron_remnant:apoE:LDLR_complex_[coated_vesicle_membrane]_=>	0.629216	0.629216	#N/A
chylomicron_remnant:apoE:LDLR_complex_[plasma_membrane]_=>_chylon	0.629216	0.629216	#N/A
eNOS:Caveolin-1_complex_binds_to_Nostrin	0.503061	0.408808	0.68331
eNOS:Caveolin-1:NOSTRIN_complex_binds_dynamin-2	0.503061	0.408808	0.68331
Exocytosis_of_thrombospondin	0.829518	0.812096	0.693218
PLC-gamma_binds_to_the_active_receptor	0.203417	0.129674	0.655421
PLC-gamma_hydrolyses_PIP2	0.203417	0.129674	0.655421
GAP_binds_to_PDGF-beta_receptors_only	0.181049	0.11133	0.655421
MAP_kinase_activates_MAPKAPK2,_MAPKAPK3_and_MSK1	0.726479	0.908091	0.249325
Cleavage_of_PAK-2_at_212	0.532193	0.308178	0.881133
Proteolysis_of_the_IGF:IGFBP-3:ALS_Complex_by_Thrombin	0.357801	0.661409	0.138658
Interaction_of_Tie2_and_Shp2	0.324682	0.233538	0.56795
p38MAPK_phosphorylates_MSK1	0.444221	0.52552	0.381741
FMO1_N-oxidizes_the_anti-cancer_drug_tamoxifen	0.022568	0.0174677	0.279522
FMO2_S-oxidizes_the_antithyroid_drug_methimazole	0.022568	0.0174677	0.279522
FMO3_N-oxidizes_the_tertiary_amine_trimethylamine	0.022568	0.0174677	0.279522
Nicotine_N-oxidised_to_Nicotine-N-oxide	0.022568	0.0174677	0.279522
LCAT_+_discoidal_HDL_<=>_LCAT:discoidal_HDL_complex	0.880822	0.790231	0.846116
LCAT_+_spherical_HDL_<=>_LCAT:spherical_HDL_complex	0.880822	0.790231	0.846116
LCAT:discoidal_HDL_complex_<=>_LCAT_+_discoidal_HDL	0.880822	0.790231	0.846116
LCAT:spherical_HDL_complex_<=>_LCAT_+_spherical_HDL	0.880822	0.790231	0.846116
Phosphorylation_of_Platelet_Sec-1	0.646714	0.673717	0.476207

Proteolysis_of_the_IGF:IGFBP-3:ALS_Complex_by_Plasmin	0.565305	0.864061	0.138658
ERK5_activates_the_transcription_factor_MEF2	0.839122	0.839122	#N/A
Proteolysis_of_the_IGF:IGFBP-5:ALS_Complex_by_PAPP-A2	0.54261	0.941333	0.253167
5,6-dihydrothymine_+_H2O_=>_beta-ureidoisobutyrate	0.966965	0.966965	#N/A
5,6-dihydrouracil_+_H2O_=>_beta-ureidopropionate	0.966965	0.966965	#N/A
ERK1/2_phosphorylates_MSK1	0.530279	0.536543	0.494174
TBK1_is_Recruited_to_the_Viral_dsRNA:TLR3:TRIF_Complex	0.730678	0.67328	0.68272
VEGF-C,D_bind_to_VEGFR3_leading_to_receptor_dimerization	0.0843611	0.261066	0.0819458
MERTK_receptor_binds_ligands_(Gas6_or_Protein_S)	0.739072	0.838355	0.293212
AMP_binds_to_gamma_subunit_of_AMP_kinase_heterotrimer	0.288039	0.756039	0.165367
adenosine_5-diphosphate_+_thioredoxin_(reduced)_=>_2-deoxyadenosine_	0.352381	0.0982637	0.713983
cytidine_5-diphosphate_+_thioredoxin_(reduced)_=>_2-deoxycytidine_5-dip	0.352381	0.0982637	0.713983
guanosine_5-diphosphate_+_thioredoxin_(reduced)_=>_2-deoxyguanosine_	0.352381	0.0982637	0.713983
uridine_5-diphosphate_+_thioredoxin_(reduced)_=>_2-deoxyuridine_5-diph	0.352381	0.0982637	0.713983
Activation_of_Talin	0.305055	0.116486	0.623734
Phosphorylation_and_Release_of_IRF3	0.699281	0.596133	0.700767
Viral_dsRNA:TLR3:TRIF:TBK1_complex_recruits_IRF3	0.699281	0.596133	0.700767
Autocatalytic_phosphorylation_of_FGFR1b	0.827379	0.827379	#N/A
FGFR1b_binds_to_FGF	0.827379	0.827379	#N/A
Receptors_CCR3,_4_and_5_bind_CCL5_ligand	0.304275	0.297015	0.437407
Activation_of_PDC_by_dephosphorylation_of_phospho-E1_alpha_compone	0.220605	0.280421	0.287996
pro-prothrombin_->_prothrombin_+_prothrombin_propeptide	0.775995	0.858813	0.324814
eIF4F_binds_to_mRNP	0.833938	0.697451	0.815575
Formation_of_the_cap-binding_eIF4F_complex	0.833938	0.697451	0.815575
Caspase-mediated_cleavage_of_MASK	0.743081	0.743081	#N/A
STAT3_activation	0.200114	0.248654	0.308844
TNF_Mediated_Activation_of_Pro-caspase_8	0.522831	0.408076	0.722912
TRADD:TRAF2:RIP1:FADD_complex_binds_Pro-Caspase_8__	0.522831	0.408076	0.722912
EGFR_autophosphorylation	0.232635	0.164003	0.517116
EGFR_binds_EGF_ligand	0.232635	0.164003	0.517116
EGFR_dimerization	0.232635	0.164003	0.517116
pro-factor_IX_->_factor_IX_+_factor_IX_propeptide	0.266323	0.318924	0.324814
NF-kB_migrates_to_the_nucleus_and_turns_on_transcription	0.351052	0.265203	0.65479
Formation_of_Cyclin_D:Cdk4/6_complexes_	0.120987	0.103499	0.380258
Translocation_of_Cyclin_D:Cdk4/6_complexes_from_the_cytoplasm_to_the	0.120987	0.103499	0.380258
pro-factor_X_->_factor_X_+_factor_X_propeptide	0.866198	0.92869	0.324814
SKI_complexes_with_the_Smad_complex,_suppressing_BMP2_signalling	0.23694	0.448443	0.0898088
uPAR_precursor_+_acyl-GPI_->_uPAR-acyl-GPI_+_uPAR_propeptide	0.170993	0.478552	0.118372
Basigin_binds_MCT1,_MCT2,_MCT3_or_MCT4	0.114704	0.463161	0.0699279
lactate_+_H+_[cytosol]_<=>_lactate_+_H+_[extracellular]	0.114704	0.463161	0.0699279
lactate_+_H+_[extracellular]_<=>_lactate_+_H+_[cytosol]	0.114704	0.463161	0.0699279
depalmitoylation_of_eNOS	0.765244	0.745887	0.615093
Regeneration_of_eEF1A:GTP_by_eEF1B_activity	0.750647	0.380911	0.868635
Binding_of_PI3K_regulatory_alpha_subunit_to_Gab1:Grb2	0.896209	0.954987	0.283165
pro-protein_Z_->_protein_Z_+_protein_Z_propeptide	0.7655	0.849966	0.324814
N-hydroxy-2-acetylaminofluorene_+_PAPS_=>_2-acetylaminofluorene-N-sul	0.00321898	0.00354144	0.240041
Interaction_of_Tie2_and_Shc1	0.461615	0.517344	0.418243

Crks_SH3_domain_engages_C3G	0.0976013	0.168514	0.191519
pro-protein_C_->_protein_C_+_protein_C_propeptide	0.757501	0.843135	0.324814
CDK5_phosphorylates_DARPP-32_on_Thr75	0.325991	0.179311	0.659267
Formation_of_a_heteromeric_BMP_receptor_complex	0.974235	0.945613	0.867003
adenosine_5-diphosphate_+_glutaredoxin_(reduced)_=>_2-deoxyadenosine	0.267341	0.0982637	0.599776
cytidine_5-diphosphate_+_glutaredoxin_(reduced)_=>_2-deoxycytidine_5-di	0.267341	0.0982637	0.599776
guanosine_5-diphosphate_+_glutaredoxin_(reduced)_=>_2-deoxyguanosine	0.267341	0.0982637	0.599776
uridine_5-diphosphate_+_glutaredoxin_(reduced)_=>_2-deoxyuridine_5-dip	0.267341	0.0982637	0.599776
Interaction_of_integrin_alpha_V_beta_8_with_vitronectin	0.424872	0.534097	0.376349
Phospho-Frs2:CrkL_engages_C3G	0.199809	0.353694	0.191519
Endothelin_receptors_bind_endothelin	0.891718	0.666197	0.878191
NRP-2_associates_with_VEGFR1_forming_complexes_on__cell_surface	0.404868	0.378666	0.476443
Latent_TGF-beta1_is_cleaved_by_furin	0.69888	0.858491	0.311443
Phosphorylation_of_PF2K-Pase_by_PKA_catalytic_subunit	0.619625	0.619625	#N/A
Antizyme_inhibitor_binds_to_OAZ_and_stablizes_ODC_complex	0.108897	0.20981	0.175638
Antizyme_OAZ_binds_to_Ornithine_decarboxylase	0.108897	0.20981	0.175638
pro-factor_VII_->_factor_VII_+_factor_VII_propeptide	0.937034	0.973662	0.324814
Exocytosis_of_TGF_beta	0.48616	0.450455	0.538546
MEK1_binds_ERK-1	0.914901	0.914901	#N/A
MEK1_phosphorylates_ERK-1	0.914901	0.914901	#N/A
MEK2_binds_ERK-2	0.914901	0.914901	#N/A
MEK2_phosphorylates_ERK-2	0.914901	0.914901	#N/A
TNF:TNF-R1_binds_TRADD,_TRAF2_and_RIP_Complex	0.197068	0.42843	0.0612713
TRADD:TRAF2:RIP1_complex_dissociates_from_the_TNF-alpha:TNF-R1_com	0.197068	0.42843	0.0612713
pro-GAS6_->_GAS6_+_GAS6_propeptide	0.540518	0.63328	0.324814
Orc1_associates_with_Orc4:Orc5:Orc3:Orc2:origin_complexes	0.0562857	0.142566	0.0793108
Translocation_of_TRAF6_to_CBM_complex	0.415401	0.367918	0.529726
pro-protein_S_->_protein_S_+_protein_S_propeptide	0.949122	0.980115	0.324814
ARMS_is_phosphorylated_by_active_TrkA_receptor	0.371264	0.613228	0.191519
Disassociation_of_Processive_Complex_and_Completed_Telomere_End	0.0989631	0.0417047	0.719874
Formation_of_C-strand_Okazaki_fragments	0.0989631	0.0417047	0.719874
Formation_of_Processive_Complex_on_the_C-strand_of_the_telomere	0.0989631	0.0417047	0.719874
Formation_of_the_Flap_Intermediate_on_the_C-strand	0.0989631	0.0417047	0.719874
IRAK_interacts_with_TRAF6	0.216766	0.437722	0.188501
MYD88_dissociates	0.216766	0.437722	0.188501
Interaction_of_SOS-1_to_Tie2_bound__Grb2	0.832304	0.838352	0.570312
Cdc6_protein_is_phosphorylated_by_CDK	0.247358	0.179311	0.517599
Megalin_internalizes_the_cubilin-DBP:Calcidiol_complex	0.701774	0.6099	0.733757
Luteinizing_hormone_receptor_can_bind_LH	0.295361	#N/A	0.295361
Thyroid-stimulating_hormone_receptor_can_bind_TSH	0.335449	#N/A	0.335449
c-src_associates_with_Cx43_in_gap_junctions	0.453187	0.458455	0.476487
Closure_of_gap_junction	0.453187	0.458455	0.476487
Phosphorylation_of_Cx43_by_c-src	0.453187	0.458455	0.476487
Receptor_CXCR2_binds_ligands_CXCL1_to_7	0.386923	0.323995	0.54145
Part_of_pro-beta-NGF_is_processed_to_mature_beta-NGF	0.590465	0.858491	0.170067
DNA_strand_displacement_synthesis	0.0990676	0.0276931	0.622235
Activation_of_the_Anaphase_Promoting_Complex_(APC)_by_Plk1	0.482635	0.140442	0.794968

Disassociation_of_PLC-gamma1_from_SLP-76	0.578046	0.445771	0.761558
Recruitment_of_PLC-gamma1_to_SLP-76	0.578046	0.445771	0.761558
Association_of__the_XRCC4:DNA_ligase_IV_complex_with_the_DNA-PK:DN/	0.941716	0.951129	0.674216
Removal_of_repair_proteins_and_ligation_of_the_processed_ends_of_the_	0.941716	0.951129	0.674216
Follicle-stimulating_hormone_receptor_can_bind_FSH	0.392234	0.511296	0.374325
Release_of_the_Mature_intronless_transcript_derived_Histone_mRNA:SLBI	0.801279	0.842337	0.45334
JNK_phosphorylates_BIM,_BAD_and_other_targets	0.838329	0.897157	0.373082
p62_is_recruited_and_forms_a_complex_with_TRAF6	0.287097	0.567163	0.136559
I-Smad_competes_with_Co-Smad_for_R-Smad1/5/8	0.0264372	0.0676984	0.0898088
Binding_of_pro-NGF_to_p75NTR:sortilin	0.618074	0.92231	0.353593
Binding_of_TFIIIC_to_Type_2_promoter	0.472902	0.261749	0.749487
Docking_of_Mature_Histone_mRNA_complex:TAP_at_the_NPC	0.714724	0.991931	0.108713
5-HT1_and_5A_receptors_can_bind_serotonin	0.541733	0.864133	0.222717
Proteinase-activated_receptors_can_bind_thrombin	0.753371	0.570981	0.879419
Autophosphorylation_of_insulin_receptor	0.948308	0.994869	0.284315
Dissociation_of_insulin_from_insulin_receptor	0.948308	0.994869	0.284315
Insulin_binding	0.948308	0.994869	0.284315
Internalisation_of_the_insulin_receptor	0.948308	0.994869	0.284315
thioredoxin,_oxidized_+_NADPH_+_H+_=>_thioredoxin,_reduced_+_NADP+	0.8753	0.971854	0.470884
fibrinogen_->_fibrin_monomer_+_2_fibrinopeptide_A_+_2_fibrinopeptide_B	0.650722	0.685971	0.473835
Spherical_HDL_binds_C_and_E_apolipoproteins	0.37284	0.115906	0.922277
glycogen-glycogenin-1_+_n_orthophosphate_=>_limit_dextrin-glycogenin-1_	0.824602	0.853187	0.518256
glycogen-glycogenin-1_+_n_orthophosphate_=>_limit_dextrin-glycogenin-1_	0.824602	0.853187	0.518256
glycogen-glycogenin-2_+_n_orthophosphate_=>_limit_dextrin-glycogenin-2_	0.824602	0.853187	0.518256
glycogen-glycogenin-2_+_n_orthophosphate_=>_limit_dextrin-glycogenin-2_	0.824602	0.853187	0.518256
poly{(1,4)-alpha-glucosyl}_glycogenin-1_+_n_orthophosphate_=>_glycogeni	0.824602	0.853187	0.518256
poly{(1,4)-alpha-glucosyl}_glycogenin-1_+_n_orthophosphate_=>_glycogeni	0.824602	0.853187	0.518256
poly{(1,4)-alpha-glucosyl}_glycogenin-2_+_n_orthophosphate_=>_glycogeni	0.824602	0.853187	0.518256
poly{(1,4)-alpha-glucosyl}_glycogenin-2_+_n_orthophosphate_=>_glycogeni	0.824602	0.853187	0.518256
ApoB-48:TG:PL_complex_+_100_triacylglycerols_+_ApoA-I_+_ApoA-IV_=>_n	0.0133342	0.0133342	#N/A
nascent_chylomicron_[endoplasmic_reticulum_lumen]_=>_nascent_chylom	0.0133342	0.0133342	#N/A
plasminogen:histidine-rich_glycoprotein_->_plasmin_+_histidine-rich_glycop	0.662427	0.662427	#N/A
plasminogen:histidine-rich_glycoprotein_->_plasmin_+_histidine-rich_glycop	0.662427	0.662427	#N/A
C3(H2O):Factor_Bb-mediated_C3_cleavage_leads_to_C3b_deposition_on_a_	0.813096	0.814217	0.590813
Factor_B_binds_to_Complement_factor_3(H2O)_(C3(H2O))	0.813096	0.814217	0.590813
Factor_B_binds_to_surface-associated_C3b	0.813096	0.814217	0.590813
Exocytosis_of_alpha_2_macroglobulin	0.847005	0.854717	0.585239
C5a_receptor_binds_C5a_anaphylatoxin	0.818161	0.814217	0.605512
Exocytosis_of_Alpha_Actinins	0.307982	0.391985	0.298482
5-hydroxyindole_acetaldehyde_to_5-hydroxyindole_acetic_acid	0.575748	0.251248	0.826349
Acetaldehyde_+_NAD+_<=>_Acetate_+_NADH_+_H+_[cytosolic]	0.575748	0.251248	0.826349
Acetaldehyde_+_NAD+_<=>_Acetate_+_NADH_+_H+_[mitochondrial]	0.575748	0.251248	0.826349
Conversion_of_C2_into_C2a_and_C2b	0.983246	0.98496	0.688987
Formation_of_C5b:C6_complex	0.889152	0.889152	#N/A
Extracellular_processing_of_novel_PDGFs	0.91526	0.87748	0.781981
Formation_of_C3_convertase_(C4b:C2a_complex)	0.987204	0.987204	#N/A
Formation_of_classic_C5_convertase	0.987204	0.987204	#N/A

kallikrein:kininogen:C1q_binding_protein_tetramer_->_kallikrein_+_activate	0.324736	0.535574	0.231915
prekallikrein_+_kininogen:C1q_binding_protein_tetramer_->_prekallikrein:k	0.324736	0.535574	0.231915
adenosine_5-diphosphate_(ADP)_+_ADP_<=>_adenosine_5-monophosphate	0.255559	0.354731	0.25169
adenosine_5-monophosphate_(AMP)_+_ATP_<=>_adenosine_5-diphosphate	0.255559	0.354731	0.25169
TFPI_+_TF:F7a_+_factor_Xa_->_TFPI:TF:F7a:factor_Xa	0.555399	0.555399	#N/A
DAG_stimulates_protein_kinase_C-delta	0.384218	0.384218	#N/A
ATP_+_alpha-D-Glucose_=>_ADP_+_alpha-D-glucose_6-phosphate_[glucokir	0.223963	0.223963	#N/A
ATP_+_alpha-D-Glucose_=>_ADP_+_alpha-D-glucose_6-phosphate_[hexokin	0.223963	0.223963	#N/A
glucokinase_[nucleoplasm]_=>_glucokinase_[cytosol]	0.223963	0.223963	#N/A
PRDM4_inhibits_cyclin_E_transcription	0.0942171	0.187003	0.165768
benzoate_+_Coenzyme_A_+_ATP_=>_benzoyl-CoA_+_AMP_+_pyrophospha	0.392148	0.584069	0.179973
phenylacetate_+_Coenzyme_A_+_ATP_=>_phenylacetyl-CoA_+_AMP_+_pyr	0.392148	0.584069	0.179973
salicylic_acid_+_Coenzyme_A_+_ATP_=>_salicylate-CoA_+_AMP_+_pyropho	0.392148	0.584069	0.179973
Formation_of_the_IGF:IGFBP-5:ALS_Complex	0.551774	0.941333	0.225104
eNOS_associates_with_Caveolin-1	0.561614	0.461323	0.68331
Nucleotide_exchange_on_RHOA	0.111322	0.418037	0.0987046
Exocytosis_of_VEGF	0.0049258	0.0416043	0.0153815
Autophosphorylation_of_PDGF_alpha_receptors	0.276433	0.17962	0.655421
Autophosphorylation_of_PDGF_alpha/beta_receptors	0.276433	0.17962	0.655421
Autophosphorylation_of_PDGF_beta_receptors	0.276433	0.17962	0.655421
Caspase_3-mediated_cleavage_of_PKC_delta	0.342441	0.342441	#N/A
Caspase-mediated_cleavage_of_PKC_theta	0.342441	0.342441	#N/A
Translocation_of_PDGF_from_ER_to_Golgi	0.474802	0.363478	0.602203
Mouse_pro-EGF_is_cleaved_by_ADAM_sheddases	0.593254	0.948345	0.251649
phosphorylated_perilipin_+_H2O_->_perilipin_+_orthophosphate	0.631454	0.144712	0.841115
Caspase-mediated_cleavage_of_plectin-1	0.763182	0.68616	0.722912
Transport_(efflux)_of_bile_salts_by_ABCB11_(bile_salt_export_pump)	0.650539	0.601186	0.628584
NQO1_interaction_with_ODC	0.187198	0.270207	0.259699
Phosphorylation_of_CARMA1	0.570424	0.560447	0.536004
RSK1/2/3_phosphorylates_CREB_at_Serine_133	0.67075	0.67075	#N/A
Transport_(influx)_of_bile_salts_and_acids_by_OATP-A	0.00315487	0.00315487	#N/A
thymidine_5-monophosphate_(TMP)_+_H2O_=>_thymidine_+_orthophosph	0.24006	0.225347	0.403345
uridine_5-monophosphate_(UMP)_+_H2O_=>_uridine_+_orthophosphate	0.24006	0.225347	0.403345
LDL_+_LDLR_=>_LDL:LDLR_complex	0.555606	0.555606	#N/A
LDL:LDLR_complex_[plasma_membrane]_=>_LDL:LDLR_complex_[clathrin-c	0.555606	0.555606	#N/A
LDLR:LDL_complex_=>_LDLR_+_LDL	0.555606	0.555606	#N/A
LDLR:LDL_complex_[coated_vesicle_membrane]_=>_LDLR:LDL_complex_[er	0.555606	0.555606	#N/A
Conversion_of_pro-apoA-I_to_apoA-I	0.1746	0.467617	0.106957
Interaction_of_Tie2_and_Dok-2	0.475041	0.298833	0.669482
Interaction_of_Tie2_and_Grb7	0.209741	0.150342	0.570312
P-selectin_binds_P-selectin_ligand	0.0992396	0.183013	0.157208
SLC7A10-mediated_uptake_of_small_neutral_amino_acids	0.426824	0.88131	0.185087
SLC7A5-mediated_uptake_of_neutral_amino_acids	0.426824	0.88131	0.185087
SLC7A6_(y+LAT2)-mediated_exchange_of_extracellular_leucine_for_cytosoli	0.426824	0.88131	0.185087
SLC7A8-mediated_uptake_of_neutral_amino_acids	0.426824	0.88131	0.185087
Caspase-mediated_cleavage_of_FADK_1	0.360591	0.360591	#N/A
PLC_beta_1/4-mediated_hydrolysis	0.8491	0.911372	0.392444

guanosine_5-monophosphate_(GMP)_+_H2O_=>_guanosine_+_orthophosp	0.190966	0.16058	0.403345
inosine_5-monophosphate_(IMP)_+_H2O_=>_inosine_+_orthophosphate	0.190966	0.16058	0.403345
Formation_of_the_MCC_complex	0.315152	#N/A	0.315152
2-deoxyadenosine_+_ATP_=>_2-deoxyadenosine_5-monophosphate_+_ADP	0.601417	0.455019	0.760595
SKI_complexes_with_the_SMAD_complex,_suppressing_TGF-beta_signaling	0.98225	0.98225	#N/A
ABCD1/ABCD3_mediates_long_chain_fatty_acid_transport_in_to_peroxisom	0.566034	0.566034	#N/A
Peroxisomal_uptake_of_very_long-chain_fatty_acyl_CoA	0.566034	0.566034	#N/A
mannose_(a1-2)_mannose_(a1-6)_(ethanolamineP)_mannose_(a1-4)_glucos	0.709466	0.527614	0.836917
FAS_Mediated_Activation_of_Pro-caspase_8	0.820605	0.362539	0.896539
FASL:FAS_Receptor_Trimer:FADD_complex_binds_pro-Caspase-8	0.820605	0.362539	0.896539
Cleavage_of_Procaspase-9_to_Caspase-9	0.508793	0.20459	0.692064
Cytochrome_C:Apaf-1_binds_Procaspase-9	0.508793	0.20459	0.692064
TrkA_phosphorylates_PLCG1	0.285159	0.444807	0.185404
17-Hydroxypregnenolone_is_dehydrogenated_to_form_pregn-5-ene-3,20-d	0.240808	0.322013	0.271791
DHA_isomerizes_to_4-Androstene3,17-dione	0.240808	0.322013	0.271791
Pregn-5-ene-3,20-dione_isomerizes_to_progesterone	0.240808	0.322013	0.271791
Pregn-5-ene-3,20-dione-17-ol_isomerizes_to_17-hydroxyprogesterone	0.240808	0.322013	0.271791
Pregnenolone_is_dehydrogenated_to_form_pregn-5-ene-3,20-dione	0.240808	0.322013	0.271791
Caspase-mediated_cleavage_of_Rock-1	0.627439	0.627439	#N/A
Caspase-mediated_cleavage_of_Lamin_A	0.268745	0.268745	#N/A
Caspase-mediated_cleavage_of_Lamin_B1	0.268745	0.268745	#N/A
Regulation_of_MKLP-1_by_phosphorylation	0.231883	0.0509742	0.914
Association_of_Cks1_with_SCF(Skp2)_complex	0.918833	0.596767	0.929361
Angiotensin_II_binds_to_angiotensin_II_receptor_(types_1_and_2)	0.0143408	0.163526	0.017279
Proteolysis_of_the_IGF:IGFBP-4_Complex_by_PAPP-A	0.597418	0.941333	0.267452
Interaction_of_Tie2_and_Grb2	0.706685	0.700146	0.570312
PKA_phosphorylates_CREB	0.955903	0.955903	#N/A
Formation_of_Cyclin_A:Cdc2_complexes	0.196907	0.179311	0.453813
Formation_of_Cyclin_A:Cdk2_complexes	0.196907	0.179311	0.453813
Translocation_of_Cyclin_A:Cdk2_complexes_to_the_nucleus	0.196907	0.179311	0.453813
Translocation_of_Cyclin_A:phospho-Cdc2_(Thr_14)_to_the_nucleus	0.196907	0.179311	0.453813
palmitoylation_of_eNOS	0.363574	0.363574	#N/A
eNOS_binds_NOSIP	0.406538	0.461323	0.380022
Phosphorylation_of_UBF-1:rDNA_Promoter	0.941331	0.941331	#N/A
Regulation_of_MKLP-2_by_phosphorylation	0.40208	0.140986	0.914
Receptor_CXCR3_binds_ligands_CXCL9_to_11	0.915866	0.970983	0.689718
Basigin_binds_CyPA	0.553698	0.553698	#N/A
Formation_of_the_IGF:IGFBP-3:ALS_Complex	0.290328	0.619688	0.138658
Phosphorylated_MAPK1_phosphorylates_ATF-2	0.774251	0.875315	0.312
cholesterol_+_phosphatidylcholine_(lecithin)_=>_cholesterol_ester_+_2-lyso	0.982293	0.967029	0.846116
Caspase_3-mediated_cleavage_of_DFF45_(117)	0.0163978	0.0211088	0.226486
Cleavage_of_DFF45_(224)_by_caspase-3	0.0163978	0.0211088	0.226486
Interaction_and_oligomerization_of_MALT1_to_Bcl10	0.395755	0.326698	0.529726
Phospho-Shc_dissociates_from_the_TrkA_receptor	0.274463	0.546469	0.167319
Shc_binds_to_the_activated_TrkA_receptor	0.274463	0.546469	0.167319
Shc,_complexed_with_TrkA,_is_tyrosine-phosphorylated	0.274463	0.546469	0.167319
Sequestration_of_BAD_protein_by_14-3-3	0.776876	0.778152	0.62172

DARPP-32_phosphorylated_on_T34_binds_to_PP1,_inhibiting_its_function	0.68051	0.144712	0.875561
DARPP-32_phosphorylated_on_Thr75_binds_to_PKA,_inhibiting_its_functio	0.866684	0.810601	0.75663
PKA_phosphorylates_DARPP-32_on_Thr34	0.866684	0.810601	0.75663
Detection_of_damage_during_initiation_of__DNA_synthesis_in_S-phase	0.477162	0.16343	0.943137
The_polymerase_component_of_DNA_polymerase_alpha:primase_synthesi:	0.477162	0.16343	0.943137
The_polymerase_component_of_DNA_polymerase_alpha:primase_synthesi:	0.477162	0.16343	0.943137
Caspase_mediated_cleavage_of_beta-catenin	0.235507	0.183359	0.548136
Dissociation_of_Caspase-3_from_SMAC:XIAP:Caspase-3	0.381196	0.172244	0.697478
Dissociation_of_Caspase-7_from_SMAC:XIAP:Caspase-7	0.381196	0.172244	0.697478
SMAC_binds_XIAP:Caspase-3	0.381196	0.172244	0.697478
SMAC_binds_XIAP:Caspase-7	0.381196	0.172244	0.697478
Phosphorylation_of_Bcl10	0.421254	0.396687	0.468668
Association_of_DFF40_with_chromatin	0.0521275	0.0146032	0.268853
Cleavage_of_DNA_by_DFF40	0.0521275	0.0146032	0.268853
Orc4_associates_with_Orc5:Orc3:Orc2:origin_complexes	0.142566	0.142566	#N/A
IRAK_is_activated	0.183801	0.392704	0.188501
p75NTR_interacts_with_IRAK:MYD88	0.183801	0.392704	0.188501
TrkA_recruits_RIT_and_RIN	0.260765	0.179265	0.504496
Vamp2_associated_secretory_vesicle_to_plasma_membrane_transport	0.832529	0.935325	0.593439
Phospho-R-Smad1/5/8_forms_a_complex_with_Co-Smad	0.128055	0.295846	0.0898088
The_phospho-R-Smad1/5/8:Co-Smad_transfers_to_the_nucleus	0.128055	0.295846	0.0898088
Succinate_<=>_Fumarate_(with_FAD_redox_reaction_on_enzyme)	0.74717	0.724652	0.634364
Transfer_of_electrons_through_the_succinate_dehydrogenase_complex	0.74717	0.724652	0.634364
Down_Regulation_of_Emi1_through_Phosphorylation_of_Emi1	0.370162	0.120548	0.914
Caspase_mediated_cleavage_of_C-IAP1	0.168263	0.248031	0.228664
NRIF_and_TRAF6_may_activate_JNK	0.210391	0.482878	0.136559
ARMS:Crk_complex_binds_to_active_TrkA_receptor	0.181586	0.339462	0.191519
ERK5_is_activated	0.885368	0.885368	#N/A
TRADD:TRAF2:RIP1_complex_binds_FADD	0.408076	0.408076	#N/A
RIP1_phosphorylates_IKKs	0.830129	0.9057	0.356104
Phospho-Frs2_binds_CrkL	0.343513	0.618671	0.191519
Regulation_of_NudC_by_phosphorylation	0.810352	0.58917	0.914
Dehydration_of_5-HpETE_to_leukotriene_A4	0.814567	0.711566	0.759984
Oxidation_of_arachidonic_acid_to_5-HpETE	0.814567	0.711566	0.759984
propionyl-CoA_+_CO2_+_ATP_<=>_D-methylmalonyl-CoA_+_ADP_+_orthop	0.605804	0.683235	0.385965
Association_of_RAD52_with_the_RPA_complex	0.87356	0.483939	0.990704
Formation_of_RAD52_heptameric_ring_structure_complexes_on_ssDNA	0.87356	0.483939	0.990704
beta-methylcrotonyl-CoA_+_ATP_+_CO2_<=>_beta-methylglutaconyl-CoA_+	0.399347	0.449254	0.385965
Interaction_of_Tie2_and_Grb14	0.652191	0.636371	0.570312
Recruitment_of_SLP-76_to_Gads	0.710177	0.590165	0.761558
Bombesin-like_receptors_bind_bombesin_homologues	0.287935	0.287935	#N/A
Release_of_the_Mature_intronless_derived_mRNA,_TAP,_and_Aly/Ref_fror	0.923069	0.956691	0.45334
Release_of_the_SLBP_independent_Histone_mRNA_from_the_NPC	0.923069	0.956691	0.45334
Oxidation_of_Cysteine_to_Cystine_in_Proinsulin	0.756551	0.929837	0.311174
SLC6A14-mediated_uptake_of_basic_and_neutral_amino_acids_and_of_bet	0.171104	#N/A	0.171104
Binding_of_ATR-ATRIP_to_the_RPA-ssDNA_complex	0.331665	0.331665	#N/A
pro-NGF_dimerizes	0.410523	0.939983	0.0303844

Formation_of_NELF_complex_	0.993489	0.993489	#N/A
p75NTR:NgR_complex_interacts_with_the_axonal_inhibitor_LINGO1	0.172486	0.574268	0.0638742
Association_of_RAD51_with_the_RPA_complex	0.472687	0.483939	0.472819
2-methyl-1-hydroxybutyl-TPP_+_lipoamide_=>_S-(2-methylbutanoyl)-dihydr	0.815268	0.815268	#N/A
2-methyl-1-hydroxypropyl-TPP_+_lipoamide_=>_S-(isobutyryl)-dihydrolipoar	0.815268	0.815268	#N/A
3-methyl-1-hydroxybutyl-TPP_+_lipoamide_=>_S-isovaleryldihydrolipoamide	0.815268	0.815268	#N/A
alpha-keto-beta-methylvalerate_+_TPP_=>_2-methyl-1-hydroxybutyl-TPP_+_	0.815268	0.815268	#N/A
alpha-ketoisocaproate_+_TPP_=>_3-methyl-1-hydroxybutyl-TPP_+_CO2	0.815268	0.815268	#N/A
alpha-ketoisovalerate_+_TPP_=>_2-methyl-1-hydroxypropyl-TPP_+_CO2	0.815268	0.815268	#N/A
dihydrolipoamide_+_FAD_=>_lipoamide_+_FADH2_[branched-chain_ketoac	0.815268	0.815268	#N/A
FADH2_+_NAD+_=>_FAD_+_NADH_+_H+_[branched-chain_ketoacid_dehydr	0.815268	0.815268	#N/A
S-(2-methylbutanoyl)-dihydrolipoamide_+_CoA_=>_alpha-methylbutyryl-Co/	0.815268	0.815268	#N/A
S-(isobutyryl)-dihydrolipoamide_+_CoA_=>_isobutyryl-CoA_+_dihydrolipoan	0.815268	0.815268	#N/A
S-isovaleryldihydrolipoamide_+_CoA_=>_isovaleryl-CoA_+_dihydrolipoamide	0.815268	0.815268	#N/A
GRB2:SOS_binds_to_SHC-P	0.937846	0.979583	0.319284
GRB2:SOS_binds_IRS-P	0.936505	0.933629	0.672364
Surface_deployment_of_GP_Ib-IX-V_complex	0.402315	0.375214	0.487086
Transport_of_the_Mature_IntronlessTranscript_Derived_Histone_mRNA:SLE	0.877695	0.920801	0.45334
Phosphorylation_of_Wee1_kinase_by_Chk1	0.685324	0.549062	0.773405
Receptor_CCR7_binds_CCL19_and_21_ligands	0.789579	0.762079	0.645601
Galanin_receptors_can_bind_galanin	0.183803	0.565111	0.075167
2-deoxyadenosine_5-diphosphate_(dADP)_+_ATP_<=>_dATP_+_adenosine_	0.723842	0.59559	0.77871
2-deoxycytidine_5-diphosphate_(dCDP)_+_ATP_<=>_dCTP_+_adenosine_5-c	0.723842	0.59559	0.77871
2-deoxyguanosine_5-diphosphate_(dGDP)_+_ATP_<=>_dGTP_+_adenosine_	0.723842	0.59559	0.77871
2-deoxyuridine_5-diphosphate_(dUDP)_+_ATP_<=>_deoxyuridine_5-triphos	0.723842	0.59559	0.77871
2-deoxyuridine_5-diphosphate_(dUDP)_+_ATP_<=>_dUTP_+_adenosine_5-d	0.723842	0.59559	0.77871
2-deoxyuridine_5-triphosphate_(dUTP)_+_ADP_<=>_2-deoxyuridine_5-diph	0.723842	0.59559	0.77871
adenosine_5-diphosphate_(ADP)_+_CTP_<=>_ATP_+_cytidine_5-diphosphat	0.723842	0.59559	0.77871
adenosine_5-diphosphate_(ADP)_+_dATP_<=>_ATP_+_2-deoxyadenosine_5	0.723842	0.59559	0.77871
adenosine_5-diphosphate_(ADP)_+_dCTP_<=>_ATP_+_2-deoxycytidine_5-di	0.723842	0.59559	0.77871
adenosine_5-diphosphate_(ADP)_+_dGTP_<=>_ATP_+_2-deoxyguanosine_5	0.723842	0.59559	0.77871
adenosine_5-diphosphate_(ADP)_+_dUTP_<=>_ATP_+_2-deoxyuridine_5-dip	0.723842	0.59559	0.77871
adenosine_5-diphosphate_(ADP)_+_GTP_<=>_ATP_+_guanosine_5-diphospl	0.723842	0.59559	0.77871
adenosine_5-diphosphate_(ADP)_+_TTP_<=>_ATP_+_thymidine_5-diphosph	0.723842	0.59559	0.77871
adenosine_5-diphosphate_(ADP)_+_UTP_<=>_ATP_+_uridine_5-diphosphate	0.723842	0.59559	0.77871
cytidine_5-diphosphate_(CDP)_+_ATP_<=>_CTP_+_adenosine_5-diphosphat	0.723842	0.59559	0.77871
guanosine_5-diphosphate_(GDP)_+_ATP_<=>_GTP_+_adenosine_5-diphospl	0.723842	0.59559	0.77871
thymidine_5-diphosphate_(TDP)_+_ATP_<=>_thymidine_5-triphosphate_(TT	0.723842	0.59559	0.77871
thymidine_5-diphosphate_(TDP)_+_ATP_<=>_TTP_+_adenosine_5-diphosph	0.723842	0.59559	0.77871
uridine_5-diphosphate_(UDP)_+_ATP_<=>_uridine_5-triphosphate_(UTP)_+_	0.723842	0.59559	0.77871
uridine_5-diphosphate_(UDP)_+_ATP_<=>_UTP_+_adenosine_5-diphosphate	0.723842	0.59559	0.77871
The_geminin_component_of_geminin:Cdt1_complexes_is_ubiquitinated,_re	0.315055	0.379595	0.352182
De_novo_formation_of_eIF2:GTP	0.921712	0.903923	0.716521
Met-tRNAi_binds_to_eIF2:GTP_to_form_the_ternary_complex	0.921712	0.903923	0.716521
Polyubiquitinated_NRIF_binds_to_p62_(Sequestosome)	0.295266	0.352111	0.337568
Polyubiquitinated_NRIF_migrates_to_the_nucleus	0.295266	0.352111	0.337568
factor_XIII_->_factor_XIII_cleaved_tetramer_+_2_factor_XIII_A_activation_p	0.723363	0.723363	#N/A

Exocytosis_of_fibrinogen	0.610097	0.651033	0.473835
n_fibrin_monomers_->_fibrin_multimer	0.610097	0.651033	0.473835
prothrombin_->_activated_thrombin_(factor_IIa)_+_thrombin_activation_pe	0.850654	0.850654	#N/A
protein_C_->_activated_protein_C_+_protein_C_heavy_chain_activation_pe	0.527946	0.623208	0.373229
2-Phospho-D-glycerate_<=>_3-Phospho-D-glycerate	0.622147	0.895551	0.305515
3-Phospho-D-glycerate_<=>_2-Phospho-D-glycerate	0.622147	0.895551	0.305515
Activation_of_MBL	0.779557	0.723847	0.688987
MBL_binds_to_repetitive_carbohydrate_structures_on_the_surfaces_of_vir	0.779557	0.723847	0.688987
ATP_+_D-fructose_6-phosphate_=>_ADP_+_D-fructose_1,6-bisphosphate	0.828904	0.844058	0.584579
factor_X_->_factor_Xa_+_factor_X_activation_peptide_(VIIIa:IXa_catalyst)	0.212721	0.212721	#N/A
urokinase_plasminogen_activator_(one-chain):uPAR_->_urokinase_plasminc	0.561992	0.561992	#N/A
alpha-D-glucose_6-phosphate_+_NADP+_=>_D-glucono-1,5-lactone_6-phosp	0.604901	0.604901	#N/A
alpha-D-glucose_6-phosphate_+_NADP+_=>_D-glucono-1,5-lactone_6-phosp	0.604901	0.604901	#N/A
Spontaneous_hydrolysis_of_C3_thioester	0.814217	0.814217	#N/A
3,7,24THCA_is_conjugated_with_Coenzyme_A_(SLC27A2_VLCS)	0.837768	0.932856	0.340112
DHCA_is_conjugated_with_Coenzyme_A_(SLC27A2_VLCS)	0.837768	0.932856	0.340112
phytanate_+_CoA-SH_+_ATP_=>_phytanoyl-CoA_+_AMP_+_pyrophosphate	0.837768	0.932856	0.340112
pristanate_+_CoA-SH_+_ATP_=>_pristanoyl-CoA_+_AMP_+_pyrophosphate	0.837768	0.932856	0.340112
TetraHCA_is_conjugated_with_Coenzyme_A_(SLC27A2_VLCS)	0.837768	0.932856	0.340112
THCA_is_conjugated_with_Coenzyme_A_(SLC27A2_VLCS)	0.837768	0.932856	0.340112
Caspase_mediated_cleavage_of_BAP31	0.662746	0.812758	0.454361
2-Phospho-D-glycerate_<=>_Phosphoenolpyruvate_+_H2O	0.357897	0.392915	0.402462
Phosphoenolpyruvate_+_H2O_<=>_2-Phospho-D-glycerate	0.357897	0.392915	0.402462
factor_IX_->_factor_IXa_+_factor_IX_activation_peptide_(TF:F7a_catalyst)	0.334056	0.334056	#N/A
D-fructose_1,6-bisphosphate_<=>_dihydroxyacetone_phosphate_+_D-glycer	0.526241	0.526241	#N/A
dihydroxyacetone_phosphate_+_D-glyceraldehyde_3-phosphate_<=>_D-fru	0.526241	0.526241	#N/A
Conversion_of_palmitic_acid_to_palmitoyl-CoA	0.152228	0.0204224	0.867057
factor_X_->_factor_Xa_+_factor_X_activation_peptide_(TF:F7_catalyst)	0.964644	0.964644	#N/A
factor_X_->_factor_Xa_+_factor_X_activation_peptide_(TF:F7a_catalyst)	0.964644	0.964644	#N/A
urokinase_plasminogen_activator_(two-chain):uPAR_+_plasminogen_activa	0.347155	0.347155	#N/A
ABCG1-mediated_transport_of_intracellular_cholesterol_to_the_cell_surfac	0.021537	0.00186921	0.724473
factor_VIII:von_Willibrand_factor_multimer_->_factor_VIIIa_+_factor_VIIIa_	0.434564	0.434564	#N/A
Beta_adrenoceptors_bind_catecholamines	0.261923	0.379356	0.251425
Formation_of_the_IGF:IGFBP-6_Complex	0.842137	0.932974	0.351096
Formation_of_the_IGF:IGFBP-2_Complex	0.177941	0.194724	0.351096
kallikrein_+_C1Inh_->_kallikrein:C1Inh	0.212553	#N/A	0.212553
Cdc42_lures_Cbl_away_from_the_receptor	0.199222	0.312768	0.219873
PIP3_binds_to_RhoA_and_activates_it	0.0987046	#N/A	0.0987046
Transfer_of_LPS_onto_TLR4_from_CD14	0.0215514	0.257872	0.00489277
ADP_+_Orthophosphate_+_Succinyl-CoA_<=>_ATP_+_Succinate_+_CoA	0.550487	#N/A	0.550487
GDP_+_Orthophosphate_+_Succinyl-CoA_<=>_GTP_+_Succinate_+_CoA	0.550487	#N/A	0.550487
2-deoxyadenosine_5-diphosphate_(dADP)_+_ADP_<=>_2-deoxyadenosine_5	0.439079	0.612188	0.25169
2-deoxyadenosine_5-monophosphate_(dAMP)_+_ATP_<=>_2-deoxyadenosi	0.439079	0.612188	0.25169
cytidine_5-diphosphate_(CDP)_+_ADP_<=>_cytidine_5-monophosphate_(CM	0.439079	0.612188	0.25169
cytidine_5-monophosphate_(CMP)_+_ATP_<=>_cytidine_5-diphosphate_(CD	0.439079	0.612188	0.25169
Reduction_of_ferric_cytochrome_B5A_to_ferrous_cytochrome_B5A	0.083709	0.051845	0.464051
SAM_is_sythesized_from_methionines_reaction_with_ATP	0.513168	0.375603	0.693022

Phosphorylation_of_PKC_theta	0.560447	0.560447	#N/A
Autophosphorylation_of_PAK-2p34_in_the_activation_loop	0.809379	0.5934	0.881133
Partial_autophosphorylation_of_PAK-2_at__Ser-19,_Ser-20,_Ser-55,_Ser-19:	0.809379	0.5934	0.881133
Proteolytic_PAK-2p34_fragment_translocates_to_the_nucleus	0.809379	0.5934	0.881133
Interaction_of_PECAM-1_and_SHP-1	0.56855	0.565734	0.525519
Interaction_of_PECAM-1_and_SHP-2	0.56855	0.565734	0.525519
2_acetyl-CoA_<=>_acetoacetyl-CoA+CoA	0.411417	0.4617	0.400313
acetoacetyl-CoA+CoA_<=>_2_acetyl-CoA	0.411417	0.4617	0.400313
alpha-methyl-acetoacetyl-CoA_+_CoA_<=>_propionyl-CoA_+_acetyl-CoA	0.411417	0.4617	0.400313
Caspase-mediated_cleavage_of_vimentin_at_DSVD_(85)	0.213321	0.213321	#N/A
perilipin_+_3_ATP_->_phosphorylated_perilipin_+_3_ADP	0.946713	0.952735	0.665998
Formation_of_the_IGF:IGFBP-4_Complex	0.616747	0.941333	0.228175
Release_of_Filamin-A	0.627424	0.44794	0.772382
Transport_(influx)_of_glycocholate_and_taurocholate_by_OATP-8	0.979299	0.907786	0.950838
Transport_(influx)_of_glycocholate_and_taurocholate_by_OATP-C	0.979299	0.907786	0.950838
2-deoxyuridine_5-monophosphate_(dUMP)_+_H2O_=>_2-deoxyuridine_+_c	0.456137	0.561318	0.403345
Caspase-mediated_cleavage_of_GAS2	0.0189422	0.0189422	#N/A
Ligand_bound_to_TLR6:TLR2	0.693399	0.655301	0.631415
L-Glutamine_transport_into_neurons	0.367691	0.367691	#N/A
Pp2a_mediated_localization_of_Rb_protein_in_chromatin	0.504346	0.819592	0.263129
Disassembly_of_SR-BI-bound_spherical_HDL	0.138779	0.138779	#N/A
spherical_HDL_and_SR-BI_receptor_form_a_complex_at_the_cell_surface	0.138779	0.138779	#N/A
Alpha-1_adrenoceptors_bind_catecholamines	0.0726319	0.0726319	#N/A
lactose_+_H2O_=>_D-glucose_+_D-galactose	0.856768	0.953575	0.546053
Viral_dsRNA:TLR3:TRIF_Complex_Recruits_RIP1	0.453921	0.316104	0.68272
Viral_dsRNA:TLR3:TRIF_Complex_Releases_Activated_RIP1	0.453921	0.316104	0.68272
Formation_of_the_IGF:IGFBP-1_Complex	0.351149	0.421458	0.351096
By_PACAP_type_1_receptor	0.452191	0.255857	0.624167
Active_PLCG1_dissociates_from_TrkA_receptor	0.100077	0.174585	0.185404
Binding_of_PLCG1_to_active_TrkA_receptor	0.100077	0.174585	0.185404
MAPKAPK2_phosphorylates_CREB_at_Serine_133	0.839398	0.945205	0.292632
Cyclophosphamide_is_4-hydroxylated_by_CYP2B6	0.00336096	0.0066243	0.0585514
CYP2J2_epoxygenates_arachidonic_acid	0.00336096	0.0066243	0.0585514
Cbl_binds_to_Grb2	0.696684	0.880276	0.219873
CPT1_converts_palmitoyl-CoA_to_palmitoyl_carnitine	0.615588	0.405689	0.801001
Further_cleavage_of_LTD4_forms_LTE4	0.791749	0.727702	0.708494
2-deoxyguanosine_5-monophosphate_(dGMP)_+_H2O_=>_2-deoxyguanosir	0.380413	0.42784	0.403345
2-deoxyinosine_5-monophosphate_(dIMP)_+_H2O_=>_2-deoxyinosine_+_oi	0.380413	0.42784	0.403345
TRAIL_Mediated_Activation_of_Pro-caspase_8	0.510794	0.34977	0.722912
TRAIL:TRAIL-Receptor2_Trimer:FADD_complex_binds_Caspase-8	0.510794	0.34977	0.722912
Phosphorylation_of_Syntaxin-4	0.256439	0.214176	0.404544
Caspase-mediated_cleavage_of_Tau	0.288401	0.288401	#N/A
depalmitoylated_eNOS_translocates_from_plasma_membrane	0.461323	0.461323	#N/A
N-myristoylation_of_eNOS	0.461323	0.461323	#N/A
Caspase-mediated_cleavage_of_farnesyltransferase/geranyl-_geranyltransfe	0.0185082	0.0185082	#N/A
(S)-Lactate_+_NAD+_<=>_Pyruvate_+_NADH_+_H+	0.00543578	0.0117481	0.113723
Pyruvate_+_NADH_+_H+_<=>_(S)-Lactate_+_NAD+	0.00543578	0.0117481	0.113723

2-deoxyuridine_+_orthophosphate_<=>_uracil_+_2-deoxy-D-ribose_1-phosp	0.0193013	0.111813	0.0313842
uracil_+_2-deoxy-D-ribose_1-phosphate_<=>_2-deoxyuridine_+_orthophosp	0.0193013	0.111813	0.0313842
FAD_can_be_hydrolyzed_back_to_FMN	0.168354	0.205668	0.275656
Receptors_CXCR4_and_7_bind__CXCL12_ligand	0.34066	0.289635	0.528724
Dimerization_of_Tie2/Ang1_complex	0.371403	0.298833	0.570312
Interaction_of_Tie2_with_Ang1	0.371403	0.298833	0.570312
Caspase_mediated_cleavage_of_HIP-55	0.113336	0.113336	#N/A
MSK1_activates_CREB	0.51346	0.520341	0.494174
Dimerisation_of_phospho-ERK-1	0.875315	0.875315	#N/A
Dimerisation_of_phospho-ERK-2	0.875315	0.875315	#N/A
ERK5_translocates_to_the_nucleus	0.875315	0.875315	#N/A
ERKs_are_inactivated_by_protein_phosphatase_2A	0.875315	0.875315	#N/A
Nuclear_translocation_of_phospho-ERK-1_dimer	0.875315	0.875315	#N/A
Nuclear_translocation_of_phospho-ERK-2_dimer	0.875315	0.875315	#N/A
Phospho-R-SMAD_forms_a_complex_with_CO-SMAD	0.97936	0.97936	#N/A
The_phospho-R-SMAD:CO-SMAD_transfers_to_the_nucleus	0.97936	0.97936	#N/A
alpha-D-glucose_(extracellular)_<=>_alpha-D-glucose_(cytosol)_[GLUT1]	0.527618	0.527618	#N/A
alpha-D-glucose_(extracellular)_<=>_alpha-D-glucose_(cytosol)_[GLUT3]	0.527618	0.527618	#N/A
alpha-D-glucose_(extracellular)_<=>_alpha-D-glucose_(cytosol)_[GLUT4]	0.527618	0.527618	#N/A
Dehydroascorbate_transport_across_the_plasma_membrane	0.527618	0.527618	#N/A
PKA_catalytic_subunit_translocates_to_the_nucleus	0.810601	0.810601	#N/A
Dissociation_of_phosphorylated_4EBP1_from_eIF4E	0.832353	0.866788	0.538172
Release_of_eIF4E_from_the_inactive_eIF4E:4E-BP_complex_	0.832353	0.866788	0.538172
Co-transport_(influx)_of_bile_salts_and_acids_and_sodium_ions_by_ASBT	0.0275833	0.165291	0.0259073
Cytochrome_C_Binds_to_Apaf-1	0.692064	#N/A	0.692064
Frs2_binds_to_active_TrkA_receptor	0.393624	0.618671	0.185404
Frs2_is_phosphorylated_by_active_TrkA_receptor	0.393624	0.618671	0.185404
urokinase_plasminogen_activator_(two-chain):uPAR_+_plasminogen_activa	0.359942	0.197757	0.718865
PRDM4_(SC1)_binds_to_p75NTR	0.0543212	0.109601	0.136559
p75NTR_interacts_with_RIP2	0.15044	#N/A	0.15044
Caspase_mediated_cleavage_of_APC	0.209114	0.209114	#N/A
EIF5A_+_spermidine_<=>_EIF5A(Dhp)_+_1,3-diaminopropane	0.653523	0.232847	0.841074
EIF5A(Dhp)_+_1,3-diaminopropane_<=>_EIF5A_+_spermidine	0.653523	0.232847	0.841074
FA_anion_diffuses_laterally_to_UCP	0.0186251	0.136319	0.0199071
FA_anion_flip-flops_to_the_opposite_surface	0.0186251	0.136319	0.0199071
Protons_are_translocated_from_the_intermembrane_space_to_the_matrix	0.0186251	0.136319	0.0199071
The_FA_anion_diffuses_away_laterally_from_UCP	0.0186251	0.136319	0.0199071
2-deoxycytidine_+_ATP_=>_2-deoxycytidine_5-monophosphate_(dCMP)_+_	0.723202	0.588508	0.760595
2-deoxycytidine_+_ATP_=>_2-deoxycytidine_5-monophosphate_+_ADP	0.723202	0.588508	0.760595
2-deoxyguanosine_+_ATP_=>_2-deoxyguanosine_5-monophosphate_+_ADP	0.723202	0.588508	0.760595
2-deoxyinosine_+_ATP_=>_2-deoxyinosine_5-monophosphate_+_ADP	0.723202	0.588508	0.760595
2-deoxyuridine_+_ATP_=>_2-deoxyuridine_5-monophosphate_+_ADP	0.723202	0.588508	0.760595
thymidine_+_ATP_=>_thymidine_5-monophosphate_+_ADP	0.723202	0.588508	0.760595
Relocalization_of_nuclearly_localized_phospho-(T286):cyclin_D1:Cdk4_to_cy	0.244543	0.15628	0.590676
Orc5_associates_with_Orc3:Orc2:origin_complexes	0.0956828	0.0956828	#N/A
Phosphorylation_of_Cyclin_D1_on_Thr-286_by_GSK-3_beta	0.809757	0.809757	#N/A
LDLR_[endosome_membrane]_=>_LDLR_[plasma_membrane]	0.624911	0.624911	#N/A

BAD_displaces_tBID_from_BCL-2_sequestration	0.0974418	0.249616	0.0986206
Release_of_Bromodomain_and_PHD_finger-containing_protein_3	0.528179	0.353902	0.742573
ERK1/2_activates_ELK1	0.787513	0.658149	0.789338
Phosphorylation_of_Cdc25A_at_Ser-123_by_Chk2	0.135892	0.135892	#N/A
Association_of_RAD50:MRE11_complex_with_NBS1_via_MRE11_interactior	0.369798	0.563449	0.211269
Bradykinin_receptors_B1_and_B2_bind_to_bradykinin	0.45371	0.628251	0.354012
Nuclear_translocation_of_catalytic_domain_of_Mst3	0.946731	0.946731	#N/A
cis-Aconitate_+_H2O_<=>_Isocitrate	0.193373	0.376244	0.146108
Citrate_<=>_cis-Aconitate_+_H2O	0.193373	0.376244	0.146108
G_alpha_13_activates_Rho_guanine_nucleotide_exchange_factor_1_(p115-	0.425454	0.577685	0.273161
Caspase-mediated_cleavage_of_Acinus	0.328278	0.328278	#N/A
Succinyl_CoA_and_glycine_condense_to_form_5-aminolevulinate_(ALA)	0.103253	0.117845	0.239301
Dicer_cleaves_pre-miRNA_to_mature_miRNA	0.28791	0.28791	#N/A
MSK1_activates_ATF1	0.305649	0.27007	0.494174
Release_of_Serotransferrin	0.424665	0.46997	0.428595
Ligands_bind_L-selectin	0.557302	0.717413	0.286204
Calcium_binds_calmodulin	0.236943	0.192615	0.396152
Release_of_Calmodulin	0.236943	0.192615	0.396152
TRAF6_is_Recruited_to_the_Viral_dsRNA:TLR3:TRIF_Complex	0.358912	0.217885	0.68272
Activation_of_Cdc25C	0.463712	0.140442	0.914
Dissociation_of_Caspase-9_from_SMAC:XIAP:Caspase-9	0.478493	0.20459	0.697478
SMAC_binds_XIAP:Caspase-9	0.478493	0.20459	0.697478
p75NTR_binds_to_NADE	0.055701	0.113681	0.136559
Translocation_of_RIAM_to_plasma_membrane	0.311302	0.215822	0.481263
NKG2D_homodimer_interacting_with_ligands	0.629642	0.506385	0.70858
Association_of_DNA-PKcs_with_Ku-bound_ends_of_DNA_double-strand_br	0.959417	0.944684	0.777119
Removal_of_3-phosphoglycolate_(PG)__moiety_from_DSB_ends	0.959417	0.944684	0.777119
Synapsis,_or_interaction_between_two_DNA-PK:DNA_complexes_at_oppos	0.959417	0.944684	0.777119
Sar1p_Activation_And_Membrane_Binding	0.477201	0.561082	0.428816
Interaction_of_Tie2_with_Ang2	0.846472	0.398209	0.924005
Cholecystokinin_receptors_bind_cholecystokinin	0.394317	0.351814	0.529037
FasL:Fas_binds_FADD	0.763364	0.362539	0.869975
Caspase-mediated_cleavage_of_claspin	0.286154	0.286154	#N/A
Binding_of_IP3_to_IP3_receptor	0.344117	0.287374	0.538919
Entry_of_Ca++_from_platelet_dense_tubular_system	0.344117	0.287374	0.538919
IP3_binds_with_the_IP3_receptor,_opening_the_Ca2+_channel	0.344117	0.287374	0.538919
Release_of_calcium_from_intracellular_stores_by_IP3_receptor_activation	0.344117	0.287374	0.538919
3-carboxy-1-hydroxypropyl-TPP_+_lipoamide_=>_S-succinyldihydrolipoamid	0.916097	0.972665	0.370793
4-carboxy-1-hydroxybutyl-TPP_+_lipoamide_=>_S-glutaryldihydrolipoamide_	0.916097	0.972665	0.370793
alpha-ketoadipate_+_TPP_=>_4-carboxy-1-hydroxybutyl-TPP_+_CO2	0.916097	0.972665	0.370793
alpha-ketoglutarate_+_TPP_=>_3-carboxy-1-hydroxypropyl-TPP_+_CO2	0.916097	0.972665	0.370793
dihydrolipoamide_+_FAD_=>_lipoamide_+_FADH2_[alpha-ketoglutarate_de	0.916097	0.972665	0.370793
FADH2_+_NAD+_=>_FAD_+_NADH_+_H+_[alpha-ketoglutarate_dehydrogen	0.916097	0.972665	0.370793
S-glutaryldihydrolipoamide_+_CoA_=>_glutaryl-CoA_+_dihydrolipoamide	0.916097	0.972665	0.370793
S-succinyldihydrolipoamide_+_CoA_=>_succinyl-CoA_+_dihydrolipoamide	0.916097	0.972665	0.370793
Pyruvate_+_CO2_+_ATP_=>_ADP_+_Orthophosphate_+_Oxaloacetate	0.453249	0.524154	0.385965
Transfer_of_electrons_from_ETF_to_ubiquinone_by_ETF-QO	0.65019	0.688954	0.488106

Thyroxine_is_deiodinated_to_triiodothyronine	0.158521	#N/A	0.158521
Vamp7_associated_Lysosome_to_Plasma_membrane_transport	0.429517	0.0300598	0.866875
Formation_of_P-TEFb_complex	0.509271	0.447979	0.588692
Interaction_of_Bcl10_to_CARMA1	0.464186	0.396687	0.529726
Oligomerization_of_Bcl10	0.464186	0.396687	0.529726
Vamp8_associated_secretory_vesicle_to_plasma_membrane_transport	0.745346	#N/A	0.745346
Active_JNK_moves_to_the_nucleus_and_phosphorylates_different_transcri	0.624054	0.730872	0.373082
Formation_of_elongin_complex	0.433347	0.743381	0.251852
Myristoylation_of_tBID_by_NMT1	0.40989	0.40989	#N/A
Nuclear_export_of_snRNA_transcripts	0.0914898	#N/A	0.0914898
Rab5-mediated_recruitment_of_class_III_PI3K_to_TLR9	0.762959	0.762959	#N/A
Interaction_of_Tie2_and_Ang4	0.526076	0.481704	0.570312
DARC__is_a_non-specific_receptor_for_many_chemokines	0.478898	0.371333	0.644916
Phosphorylation_of_Cdc25A_at_Ser-123_by_Chk1	0.139123	0.139123	#N/A
Active_NTRK1_binds_IRS1/2	0.372215	0.453257	0.375726
Recruitment_of_Acetylated_SL1_to_phosUBF-1:rDNA_Promoter	0.862806	0.871756	0.703673
Association_of_Mal_to_activated_TLR4	0.00676703	0.11568	0.00489277
Sphingomyelinase_is_activated_by_the_NGF:p75NTR_complex	0.250353	0.649045	0.136559
IRAK1-P(n):TRAF6_binds_MEKK1	0.454793	0.454793	#N/A
Association_of_TFAM:mt_promoter_complex_with_POLRMT:TFB2M	0.410399	0.410399	#N/A
SLC7A1_(CAT-1)-mediated_uptake_of_cationic_amino_acids	0.853907	0.896598	0.653984
SLC7A2,_isoform_A_(CAT-2A)-mediated_uptake_of_cationic_amino_acids	0.853907	0.896598	0.653984
SLC7A3_(CAT-3)-mediated_uptake_of_cationic_amino_acids	0.853907	0.896598	0.653984
Acetylation_of_SL1	0.771195	#N/A	0.771195
Receptor_CXCR1_binds_CXCL6_and_CXCL8_ligands	0.484114	0.377406	0.644916
uridine_+_ATP_=>_uridine_5-monophosphate_(UMP)_+_ADP	0.272762	0.522451	0.129911
Neurotensin_receptors_can_bind_neurotensins	0.865097	0.799263	0.76521
5-HT2_receptor_can_bind_serotonin	0.0345114	#N/A	0.0345114
M1,_M3_and_M5_receptors_bind_acetylcholine	0.234326	0.413848	0.171675
Re-integration_of_insulin_receptor_into_plasma_membrane	0.944065	0.979207	0.448488
dehydroepiandrosterone_(DHEA)_+_PAPS_=>_DHEA_sulfate_+_PAP	0.102944	#N/A	0.102944
Disassociation_of_PLC-gamma1_from_LAT	0.57859	0.397033	0.761558
Recruitment_of_PLC-gamma1_to_LAT	0.57859	0.397033	0.761558
Association_of__RPA_complexes_with_ssDNA	0.483939	0.483939	#N/A
Stalling_of_DNA_replication_fork_and_RPA_binding	0.483939	0.483939	#N/A
Formation_of_eEF1B_complex	0.694457	0.403213	0.786799
Opioid_binds_MOR	0.190401	0.239547	0.283288
Opioid_dissociates_from_MOR	0.190401	0.239547	0.283288
phenylalanine_+_tRNA(Phe)_+_ATP_=>_Phe-tRNA(Phe)_+_AMP_+_pyropho	0.465315	#N/A	0.465315
DIT_and_MIT_combine_to_form_triiodothyronine	0.697906	0.178927	0.900521
Iodide_is_organified	0.697906	0.178927	0.900521
Two_DITs_combine_to_form_thyroxine	0.697906	0.178927	0.900521
Tyrosine_is_diiodinated	0.697906	0.178927	0.900521
Tyrosine_is_monoiodinated	0.697906	0.178927	0.900521
Receptors_CCR1,_2_and_8_bind_CCL16_ligand	0.177006	0.299671	0.222472
Dissociation_of_LIG3:XRCC1_complex_from_site_of_BER	0.305658	0.305658	#N/A
DNA_ligation_via_the_single-nucleotide_replacement_pathway	0.305658	0.305658	#N/A

Recruitment__of__LIG3:XRRC1_complex_to_the_site_of_repair_by_POL_Be	0.305658	0.305658	#N/A
Resynthesis_of_excised_residue	0.305658	0.305658	#N/A
Vasopressin_receptor_type_1_bind_vasopressin	0.511987	0.919571	0.1696
Gonadotropin-releasing_hormone_receptor_can_bind_GnRH	0.674851	0.812161	0.471217
5-HT4,_6_and_7_receptors_can_bind_serotonin	0.508026	0.567448	0.418307
XPC_binds_to_HR23B_forming_a_heterodimeric_complex	0.0981449	0.0541157	0.513153
XPC:HR23B_complex_binds_to_damaged_DNA_site_with_lesion	0.0981449	0.0541157	0.513153
Recruitment_of_Gads_to_LAT	0.799547	0.70011	0.761558
Transport_of_the_Mature_Intronless_Transcript_Derived_Histone_mRNA:T/	0.971124	0.991931	0.45334
Transport_of_the_Mature_intronless_transcript_derived_mRNA:TAP:Aly/Re	0.971124	0.991931	0.45334
Basigin_binds_Matrix_metalloproteinase-1	0.506624	0.575112	0.4052
Association_of_HMGB1/HMGB2_with_chromatin	0.410399	0.425714	0.448987
Digestion_of_diacylglycerols_by_extracellular_PTL:colipase	0.583894	0.666531	0.481794
Digestion_of_triacylglycerols_by_extracellular_PTL:colipase	0.583894	0.666531	0.481794
Reuptake_of_dopamine_from_the_synaptic_cleft_into_neurons	0.954492	0.954492	#N/A
Receptor_CCR10_binds_CCL27_and_28_ligands	0.588968	0.817909	0.356741
IkB_is_ubiquitinated_and_degraded	0.18221	0.20762	0.337568
glutathione_(oxidized)_+_NADPH_+_H+_=>_2_glutathione_(reduced)_+_NA	0.97056	0.971854	0.715885
Phosphorylated_Orc1_is_ubiquitinated_while_still_associated_with_chroma	0.108845	0.379595	0.0979772
Ubiquitinated_Orc1_enters_the_cytosol	0.108845	0.379595	0.0979772
activated_thrombin_(factor_IIa)_+_antithrombin_III:heparin_->_thrombin:a	0.476622	0.476622	#N/A
thrombin:antithrombin_III:heparin_->_thrombin:cleaved_antithrombin_III:h	0.476622	0.476622	#N/A
thrombin:cleaved_antithrombin_III:heparin_->_thrombin:cleaved_antithrom	0.476622	0.476622	#N/A
2_phosphorylated_HSL_monomers_->_phosphorylated_HSL_dimer	0.987744	0.991913	0.780397
phosphorylated_HSL_+_H2O_->_HSL_+_orthophosphate	0.987744	0.991913	0.780397
Phosphorylated_HSL_dimer_translocates_from_the_cytosol_to_the_lipid_p	0.987744	0.991913	0.780397
D-fructose_1,6-bisphosphate_+_H2O_=>_D-fructose_6-phosphate_+_orthop	0.679213	0.679213	#N/A
prothrombin_->_activated_thrombin_(factor_IIa)_+_thrombin_activation_p	0.832056	0.832056	#N/A
histidine-rich_glycoprotein_+_plasminogen_<->_histidine-rich_glycoprotein:	0.925254	0.925254	#N/A
histidine-rich_glycoprotein:plasminogen_<->_histidine-rich_glycoprotein_+_	0.925254	0.925254	#N/A
plasminogen_+_histidine-rich_glycoprotein_->_plasminogen:histidine-rich_g	0.925254	0.925254	#N/A
Coumarin_is_7-hydroxylated_by_CYP2A13	0.0128387	#N/A	0.0128387
Coumarin_is_7-hydroxylated_by_CYP2A6	0.0128387	#N/A	0.0128387
creatine_+_ATP_=>_phosphocreatine_+_ADP_[CKB,CKM]	0.784784	0.784784	#N/A
ADP-ATP_translocase_maintains_a_high_ADP:ATP_ratio_in_the_matrix	0.0174073	0.0324281	0.127558
m_UDP-glucose_+_{(1,4)-alpha-D-glucosyl}n_glycogenin-1_=>_m_UDP_+_{(1	0.849586	0.69754	0.827547
m_UDP-glucose_+_{(1,4)-alpha-D-glucosyl}n_glycogenin-1_=>_m_UDP_+_{(1	0.849586	0.69754	0.827547
m_UDP-glucose_+_{(1,4)-alpha-D-glucosyl}n_glycogenin-2_=>_m_UDP_+_{(1	0.849586	0.69754	0.827547
m_UDP-glucose_+_{(1,4)-alpha-D-glucosyl}n_glycogenin-2_=>_m_UDP_+_{(1	0.849586	0.69754	0.827547
activated_thrombin_(factor_IIa)_+_thrombomodulin_->_activated_thrombi	0.490541	0.613716	0.373229
factor_V_->_factor_Va_+_factor_V_activation_peptide	0.696562	0.696562	#N/A
chylomicron_remnant_+_apoE_=>_chylomicron_remnant:apoE_complex	0.551882	0.551882	#N/A
factor_XIII_cleaved_tetramer_+_2_Ca++_->_factor_XIIIa_+_2_factor_XIII_B_	0.698761	0.698761	#N/A
alpha-2-antiplasmin_+_plasmin_->_alpha-2-antiplasmin:plasmin	0.880134	0.880134	#N/A
Caspase_mediated_cleavage_of_vimentin_at_IDVD_(259)	0.651844	0.48383	0.722912
ADP_+_Phosphoenolpyruvate_=>_ATP_+_Pyruvate_(pyruvate_kinase_M2)	0.922464	0.922464	#N/A
2-deoxycytidine_5-diphosphate_(dCDP)_+_ADP_<=>_deoxycytidine_5-monc	0.293442	0.46029	0.25169

2-deoxycytidine_5-monophosphate_(dCMP)_+_ATP_<=>_deoxycytidine_5-d	0.293442	0.46029	0.25169
uridine_5-diphosphate_(UDP)_+_ADP_<=>_uridine_5-monophosphate_(UM	0.293442	0.46029	0.25169
uridine_5-monophosphate_(UMP)_+_ATP_<=>_uridine_5-diphosphate_(UDI	0.293442	0.46029	0.25169
Co-transport_(influx)_of_bile_salts_and_sodium_ions_by_NTCP	0.906081	0.880736	0.752476
limit_dextrin-glycogenin-1_=>_{(1,6)-alpha-glucosyl}poly{(1,4)-alpha-glucosy	0.716473	0.716473	#N/A
limit_dextrin-glycogenin-2_=>_{(1,6)-alpha-glucosyl}poly{(1,4)-alpha-glucosy	0.716473	0.716473	#N/A
{(1,6)-alpha-glucosyl}poly{(1,4)-alpha-glucosyl}glycogenin-1_=>_poly{(1,4)-al	0.716473	0.716473	#N/A
{(1,6)-alpha-glucosyl}poly{(1,4)-alpha-glucosyl}glycogenin-2_=>_poly{(1,4)-al	0.716473	0.716473	#N/A
aspartate_[mitochondrial_matrix]_+_glutamate_[cytosol]_=>_aspartate_[cy	0.134445	0.134445	#N/A
crosslinked_fibrin_multimer:tissue_plasminogen_activator_(one-chain)_+_p	0.967366	0.929255	0.871785
crosslinked_fibrin_multimer:tissue_plasminogen_activator_(one-chain)_->_(	0.967366	0.929255	0.871785
crosslinked_fibrin_multimer:tissue_plasminogen_activator_(one-chain):plasi	0.967366	0.929255	0.871785
crosslinked_fibrin_multimer:tissue_plasminogen_activator_(two-chain)_+_p	0.967366	0.929255	0.871785
crosslinked_fibrin_multimer:tissue_plasminogen_activator_(two-chain):plasi	0.967366	0.929255	0.871785
3-ketohexacosanoyl-CoA_+_CoASH_=>_tetracosanoyl-CoA_+_acetyl-CoA	0.344991	0.153621	0.675942
Connexin_oligomerization_in__ER-Golgi-Intermediate_Compartment	0.0225848	#N/A	0.0225848
factor_Va_+_factor_Xa_->_Va:Xa_complex_(prothrombinase)	0.857107	0.857107	#N/A
lysophosphatic_acid_+_fatty_acyl_CoA__=>_phosphatidic_acid_+_CoA_(1)_	0.420493	0.815245	0.118645
By_adenosine_A2a_receptor	0.142746	0.142746	#N/A
S-Adenosyl_methionine_<=>_Decarboxylated-Adenosyl_methionine_+_CO2	0.927314	#N/A	0.927314
Ligand_binds_to_TLR7_or_TLR8	0.838408	0.639704	0.850395
Binding_of_APOBEC-1_to_form_editosome	0.389485	0.389485	#N/A
C4_deamination_of_cytidine	0.389485	0.389485	#N/A
perilipin:CGI-58_complex_->_perilipin_+_CGI-58	0.329941	#N/A	0.329941
factor_VIIIa_+_factor_IXa_->_factor_VIIIa:factor_IXa	0.0414707	0.0414707	#N/A
factor_VII_->_factor_VIIa	0.985433	0.985433	#N/A
factor_Va_->_factor_Vi	0.662395	0.662395	#N/A
Translocation_of_PKC_theta_to_plasma_membrane	0.644641	0.644641	#N/A
acetoacetyl-CoA+acetyl-CoA_=>_HMG-CoA	0.182856	0.10148	0.49749
Condensation_of_acetyl_CoA_with_acetoacetyl_CoA_to_form_HMG-CoA	0.182856	0.10148	0.49749
(2S)-pristanoyl-CoA_+_O2_=>_trans-2,3-dehydropristanoyl-CoA_+_H2O2_(A	0.923783	0.923783	#N/A
25(S)_DHCA-CoA_is_dehydrogenated_to_25(S)_3alpha,7alpha-dihydroxy-5b	0.923783	0.923783	#N/A
25(S)_THCA-CoA_is_dehydrogenated_to_25(S)_3alpha,7alpha,12alpha-trihy	0.923783	0.923783	#N/A
hexacosanoyl-CoA_+_O2_=>_trans-2,3-dehydrohexacosanoyl-CoA_+_H2O2	0.923783	0.923783	#N/A
urokinase_plasminogen_activator_+_urokinase_plasminogen_activator_rece	0.197757	0.197757	#N/A
isocitrate_+_NADP+_=>_2-oxoglutarate_+_CO2_+_NADPH_+_H+	0.714979	0.714979	#N/A
eEF1A_complexes_with_GTP	0.672052	0.426931	0.791981
eEF1A:GTP:aminoacyl_tRNA_ternary_complex_formation.	0.672052	0.426931	0.791981
Caspase-mediated_cleavage_of_vimentin_at_TNLD_(429)	0.125025	0.125025	#N/A
fibrin_multimer,_crosslinked:tissue_plasminogen_activator_(one-chain)_+_p	0.879231	0.698443	0.871785
fibrin_multimer,_crosslinked:tissue_plasminogen_activator_(two-chain)_+_p	0.879231	0.698443	0.871785
ADP_is_exchanged_for_ATP_in_the__ADP:CCT/TriC:tubulin_complex	0.340827	0.340827	#N/A
Hydrolysis_of_ATP_and_release_of_tubulin_folding_intermediate_from_CC	0.340827	0.340827	#N/A
Isocitrate_+_NAD+_=>_alpha-ketoglutarate_+_CO2_+_NADH_+_H+	0.787664	0.897333	0.444837
Caspase-8_activates_BID_by_cleavage	0.212816	0.0428514	0.722912
CYP2S1_can_deactivate_all-trans-retinoic_acid	0.631418	#N/A	0.631418
GPI-bound_CD14_binds_LPS	0.569999	0.569999	#N/A

Secreted_CD14_binds_LPS	0.569999	0.569999	#N/A
glycolate_+_O2_=>_glyoxylate_+_H2O2	0.872184	0.872184	#N/A
glyoxylate_+_O2_=>_oxalate_+_H2O2	0.872184	0.872184	#N/A
isoleucine_+_alpha-ketoglutarate_<=>_alpha-keto-beta-methylvalerate_+_g	0.409111	#N/A	0.409111
isoleucine_+_alpha-ketoglutarate_<=>_alpha-keto-beta-methylvalerate_+_g	0.409111	#N/A	0.409111
L-Glutamate_[cytosolic]_from_leucine_catabolism	0.409111	#N/A	0.409111
L-Glutamate_[mitochondrial]_from_leucine_catabolism	0.409111	#N/A	0.409111
valine_+_alpha-ketoglutarate_<=>_alpha-ketoisovalerate_+_glutamate_[cyt	0.409111	#N/A	0.409111
valine_+_alpha-ketoglutarate_<=>_alpha-ketoisovalerate_+_glutamate_[mit	0.409111	#N/A	0.409111
Orc3_associates_with_Orc2_constitutively_bound_at_origins_of_replication	0.178385	0.178385	#N/A
CD47_binds_SIRP	0.863757	0.869216	0.665597
Glutamate_and_cysteine_combine	0.46648	0.46648	#N/A
inosine_5-monophosphate_+_L-aspartate_+_GTP_=>_adenylosuccinate_+_g	0.423912	#N/A	0.423912
kininogen_+_C1q_binding_protein_tetramer_->_kininogen:C1q_binding_prc	0.535574	0.535574	#N/A
Release_of_novel_PDGFs_as_latent_factors	0.539019	0.568389	0.486212
pro-prothrombin,_uncarboxylated_+_10_CO2_+_10_O2_+_10_vitamin_K_hy	0.73628	0.613716	0.726915
Dimeric_TGF-beta1_binds_to_the_receptor	0.418062	#N/A	0.418062
Reduction_of_semidehydroascorbate_to_ascorbate	0.356841	0.333879	0.464051
Conversion_of_Serotonin_to_5-hydroxyindole_acetaldehyde	0.311425	0.311425	#N/A
Dietary_tyramine_is_oxidatively_deaminated_to_an_aldehyde_by_MAOB	0.311425	0.311425	#N/A
Metabolism_of_Noradrenaline	0.311425	0.311425	#N/A
Oxidation_of_3-Methoxytyramine_to_homovanillic_acid	0.311425	0.311425	#N/A
oxidation_of_dopamine_to_3,4dihydroxyphenylacetic_acid_(DOPAC)	0.311425	0.311425	#N/A
Oxidative_deamination_of_5-Hydroxytryptamine_by_MAOA	0.311425	0.311425	#N/A
Oxidative_deamination_of_Phenyethylamine_by_MAOB	0.311425	0.311425	#N/A
uridine_5-triphosphate_+_glutamine_+_ATP_+_H2O_=>_cytidine_5-triphosp	0.191956	0.261944	0.276163
CD200_binds_to_CD200R	0.932453	#N/A	0.932453
Caveolin-1_binds_Basigin	0.536735	0.364146	0.68331
Thrombin-mediated_activation_of_PAR3	0.745066	0.745066	#N/A
factor_XIIa_+_C1Inh_->_factor_XIIa:C1Inh	0.815938	0.947104	0.364705
tissue_factor_(TF)_+_activated_factor_VII_(F7a)_->_TF:F7a_complex	0.927167	0.927167	#N/A
tissue_factor_(TF)_+_factor_VII_(F7)_->_TF:F7_complex	0.927167	0.927167	#N/A
vitamin_K_epoxide_->_vitamin_K_hydroquinone	0.280625	0.548267	0.172749
OLR1_binds_to_oxidized_LDL	0.819585	0.819585	#N/A
pro-factor_IX,_uncarboxylated_+_12_CO2_+_12_O2_+_12_vitamin_K_hydrc	0.0601938	0.00255619	0.726915
ATP_+_D-galactose_=>_ADP_+_D-galactose_1-phosphate	0.863538	#N/A	0.863538
Release_of_Talin-1	0.419163	0.183931	0.729493
arginine_=>_ornithine_+_urea	0.383808	0.0565427	0.878189
Activation_of_PKC	0.273609	0.214176	0.476207
aspartate_+_alpha-ketoglutarate_<=>_oxaloacetate_+_glutamate_[cytosol]	0.551418	0.551418	#N/A
aspartate_+_alpha-ketoglutarate_<=>_oxaloacetate_+_glutamate_[mitochoı	0.551418	0.551418	#N/A
oxaloacetate_+_glutamate_<=>_aspartate_+_alpha-ketoglutarate_[cytosolic]	0.551418	0.551418	#N/A
oxaloacetate_+_glutamate_<=>_aspartate_+_alpha-ketoglutarate_[mitochoı	0.551418	0.551418	#N/A
cytidine_5-monophosphate_(CMP)_+_H2O_=>_cytidine_+_orthophosphate	0.225347	0.225347	#N/A
Exocytosis_of_protease_nexin_II_APP	0.550853	0.725434	0.337553
Binding_of_HREV1_to_lesioned_DNA_template	0.00289463	0.00289463	#N/A
Elongation_by_HREV1_protein	0.00289463	0.00289463	#N/A

Misinsertion_of__bases_opposite_to_the_lesion_by_HREV1	0.00289463	0.00289463	#N/A
Thrombin-mediated_activation_of_PAR4	0.849462	0.613716	0.879419
Sequestration_of_tBID_by_BCL-2	0.0166985	0.0428514	0.0986206
D-ribose_5-phosphate_+_2-deoxyadenosine_5-triphosphate_(dATP)_=>_5-P	0.641454	0.810426	0.356431
D-ribose_5-phosphate_+_ATP_=>_5-phospho-alpha-D-ribose_1-diphosphate	0.641454	0.810426	0.356431
ABCG5:ABCG8-mediated_export_of_cholesterol_and_phytosterols	0.317365	0.317365	#N/A
Ligand_binds_to_TLR10	0.577517	0.655301	0.45065
1-palmitoylglycerone_phosphate_+_hexadecanol_=>_O-hexadecylglycerone	0.352465	0.352465	#N/A
palmitoyl-CoA_+_DHAP_=>_1-palmitoylglycerone_phosphate_+_CoASH	0.352465	0.352465	#N/A
pro-factor_X,_uncarboxylated_+_11_CO2_+_11_O2_+_11_vitamin_K_hydro	0.88195	0.85782	0.726915
(S)-3-Hydroxyhexadecanoyl-CoA+NAD<=>3-Oxopalmitoyl-CoA+NADH+H	0.183111	0.183111	#N/A
(S)-3-Hydroxytetradecanoyl-CoA+NAD<=>3-Oxotetradecanoyl-CoA+NADH+H	0.183111	0.183111	#N/A
3-Oxodecanoyl-CoA+CoA-SH<=>Octanoyl-CoA	0.183111	0.183111	#N/A
3-Oxododecanoyl-CoA+CoA-SH<=>Decanoyl-CoA	0.183111	0.183111	#N/A
3-Oxohexanoyl-CoA+CoA-SH<=>Butanoyl-CoA	0.183111	0.183111	#N/A
3-Oxooctanoyl-CoA+CoA-SH<=>Hexanoyl-CoA	0.183111	0.183111	#N/A
3-Oxopalmitoyl-CoA+CoA-SH<=>myristoyl-CoA	0.183111	0.183111	#N/A
3-Oxotetradecanoyl-CoA+CoA-SH<=>Lauroyl-CoA	0.183111	0.183111	#N/A
trans-Hexadec-2-enoyl-CoA+H2O<=>(S)-3-Hydroxyhexadecanoyl-CoA	0.183111	0.183111	#N/A
trans-Tetradec-2-enoyl-CoA+H2O<=>(S)-3-Hydroxytetradecanoyl-CoA	0.183111	0.183111	#N/A
beta-NGF_dimer_binds_to_TrkA_receptor	0.136382	0.255857	0.185404
The_bound_receptor_dimerizes	0.136382	0.255857	0.185404
TrkA_receptor_autophosphorylates	0.136382	0.255857	0.185404
Ethanol_+_NAD+_<=>_Acetaldehyde_+_NADH_+_H+_[class_IV,_mu_or_sign	0.516279	#N/A	0.516279
adenylyl_sulfate_(APS)_+_ATP_=>_PAPS_+_ADP	0.19379	0.19379	#N/A
ATP_+_sulfate_=>_adenylyl_sulfate_(APS)_+_pyrophosphate	0.19379	0.19379	#N/A
Trans-phosphorylation_of_Tie2	0.4867	0.4867	#N/A
poly{(1,4)-alpha-D-glucosyl}_glycogenin-1_=>_glycogen-glycogenin-1	0.904074	0.904074	#N/A
poly{(1,4)-alpha-D-glucosyl}_glycogenin-2_=>_glycogen-glycogenin-2	0.904074	0.904074	#N/A
Phosphorylation_of_PDE3B_by_AKT-1	0.555837	0.555837	#N/A
Viral_dsRNA_bound_TRL3_Recruits_TRIF	0.316172	0.124574	0.68272
Noradrenaline_clearance_from_the_synaptic_cleft	0.556086	0.520388	0.558629
adenosine_5-monophosphate_(AMP)_+_H2O_=>_inosine_5-monophosphat	0.711798	0.978604	0.108081
Hypoxanthine_+_H2O_+_O2_=>_Xanthine_+_H2O2	0.79322	0.633632	0.792058
Xanthine_+_H2O_+_O2_=>_Urate_+_H2O2	0.79322	0.633632	0.792058
Hydrolysis_of_cAMP_to_5_AMP_by_Phosphorylated_PDE3B	0.627845	0.73991	0.427483
Interaction_of_PECAM-1_and_PLC_gamma1	0.522449	0.522449	#N/A
pro-protein_Z,_uncarboxylated_+_13_CO2_+_13_O2_+_13_vitamin_K_hydr	0.718095	0.58381	0.726915
tBID_activates_BAK_protein	0.0502504	0.0428514	0.272904
tBID_binds_to_inactive_BAK_protein	0.0502504	0.0428514	0.272904
Formation_of_RAD50:MRE11_complex	0.563449	0.563449	#N/A
Exocytosis_of_PDGF	0.425404	0.252495	0.655421
pro-protein_C,_uncarboxylated_+_8_CO2_+_8_O2_+_8_vitamin_K_hydroqu	0.704155	0.561184	0.726915
Interaction_of_Csk_with_PAG	0.519074	0.519074	#N/A
Reduction_of_androstenedione_to_testosterone	0.557455	0.557455	#N/A
apoA-I_binds_to_CUBN:AMN	0.332296	0.0581266	0.830715
NAT1_acetylation	0.159105	0.159105	#N/A

NAT2_acetylation	0.159105	0.159105	#N/A
The_acetyl_group_from_acetyl-CoA_is_transferred_to_the_NAT1	0.159105	0.159105	#N/A
ACTH_specifically_binds_with_melanocortin_receptor_2	0.764047	0.764047	#N/A
Nicotinate_D-ribonucleotide_+_ATP_=>_deamino-NAD+_+_pyrophosphate_	0.628797	0.628797	#N/A
Nicotinate_D-ribonucleotide_+_ATP_=>_deamino-NAD+_+_pyrophosphate_	0.628797	0.628797	#N/A
Ascorbate_transport_across_the_plasma_membrane	0.547212	0.547212	#N/A
Reduction_of_2-trans-4-cis-decadienoyl-CoA_to_form_3-trans-decenoyl-CoA	0.0481154	0.0106337	0.480452
ADP_+_3-Phospho-D-glyceroyl_phosphate_<=>_ATP_+_3-Phospho-D-glycera	0.663638	0.971423	0.095814
ATP_+_3-Phospho-D-glycerate_<=>_ADP_+_1,3-bisphospho-D-glycerate	0.663638	0.971423	0.095814
factor_VIII_+_von_Willebrand_factor_multimer_->_factor_VIII:von_Willibrar	0.342064	0.342064	#N/A
Glutamine_transport_from_astrocytes	0.662365	0.662365	#N/A
SLC38A1_(ATA1)-mediated_uptake_of_neutral_amino_acids	0.662365	0.662365	#N/A
2-deoxyuridine_3-monophosphate_+_H2O_=>_2-deoxyuridine_+_orthophos	0.403345	#N/A	0.403345
uridine_2-monophosphate_+_H2O_=>_uridine_+_orthophosphate	0.403345	#N/A	0.403345
uridine_2-monophosphate_+_H2O_=>_uridine_+_orthophosphate_	0.403345	#N/A	0.403345
uridine_3-monophosphate_+_H2O_=>_uridine_+_orthophosphate	0.403345	#N/A	0.403345
Formation_of_ERCC1-XPF_heterodimeric_complex	0.208129	0.208129	#N/A
Pantothenate_is_phosphorylated_[PANK1;3;4]	0.613826	0.613826	#N/A
Pantothenate_is_phosphorylated_[PANK2]	0.613826	0.613826	#N/A
tBID_activates_BAX_protein	0.161759	0.0428514	0.626322
tBID_binds_to_inactive_BAX_protein	0.161759	0.0428514	0.626322
alanine_+_alpha-ketoglutarate_<=>_pyruvate_+_glutamate	0.814124	0.814124	#N/A
pyruvate_+_glutamate_<=>_alanine_+_alpha-ketoglutarate	0.814124	0.814124	#N/A
Formation_of_mChREBP:mMlx_complex	0.774638	0.771426	0.626217
uracil_+_D-ribose_1-phosphate_<=>_uridine_+_orthophosphate	0.0440675	0.29094	0.0313842
uridine_+_orthophosphate_<=>_uracil_+_D-ribose_1-phosphate	0.0440675	0.29094	0.0313842
phenylalanine_+_tetrahydrobiopterin_+_O2_=>_tyrosine_+_4a-hydroxytetra	0.445723	0.445723	#N/A
Tyrosine_is_hydroxylated_to_dopa	0.445723	0.445723	#N/A
Thrombin-mediated_activation_of_PAR1	0.674549	0.674549	#N/A
Phosphorylation_and_activation_of_CHK2_by_ATM	0.334144	0.334144	#N/A
thioredoxin,_oxidized_(nucleus)_<=>_thioredoxin,_oxidized_(cytosol)	0.310736	#N/A	0.310736
thioredoxin,_reduced_(cytosol)_<=>_thioredoxin,_reduced_(nucleus)	0.310736	#N/A	0.310736
Translocation_of_caspase-3_to_the_nucleus	0.172244	0.172244	#N/A
pro-factor_VII,_uncarboxylated_+_10_CO2_+_10_O2_+_10_vitamin_K_hydr	0.967934	0.977928	0.726915
creatine_+_ATP_=>_phosphocreatine_+_ADP_[CK_octamer]	0.88924	0.773511	0.835696
ATP_+_Acetate_+_CoA_<=>_AMP_+_Pyrophosphate_+_Acetyl-CoA	0.00697004	0.000278058	0.490243
Interaction_of_tBID_with_BCL-xl	0.0189367	0.0428514	0.11175
Excision_of__the_abasic_sugar_phosphate_(dRP)_residue_at_the_strand_b	0.636825	0.737962	0.443105
POL_Beta_mediated_incorporation_of_the_first_replacement_nucleotide	0.636825	0.737962	0.443105
Recruitment_of_POL_Beta_to_the_AP_site	0.636825	0.737962	0.443105
alpha-secretase_cleaves_the_p75NTR_extracellular_domain	0.75476	0.948345	0.256225
inosine_5-monophosphate_(IMP)_+_NAD+_+_H2O_=>_xanthosine_5-mono	0.909907	#N/A	0.909907
Formation_of_TSC1:TSC2_complex	0.291494	0.291494	#N/A
1,3-bisphospho-D-glycerate_+_NADH_+_H+_<=>_D-glyceraldehyde_3-phosp	0.158937	0.316245	0.175013
D-glyceraldehyde_3-phosphate_+_orthophosphate_+_NAD+_<=>_1,3-bisph	0.158937	0.316245	0.175013
JAM-B_binds_JAM-C	0.608666	0.893312	0.196228
pro-GAS6,_uncarboxylated_+_11_CO2_+_11_O2_+_11_vitamin_K_hydroqui	0.342253	0.119264	0.726915

Phosphorylation_of_mpChREBP_(Thr_666)_at_Ser(196)_by_mPKA	0.881997	0.911904	0.626217
Phosphorylation_of_mChREBP_at_Thr_(666)_residue_by_mPKA	0.881997	0.911904	0.626217
EIF5A(Dhp)_+_O2_=>_EIF5A(Hyp)	0.841074	#N/A	0.841074
10-formylTHF_polyglutamate_<=>_5,10-methenylTHF_polyglutamate_+_H2(	0.680297	0.818007	0.402746
5,10-methenylTHF_polyglutamate_+_H2O_<=>_10-formylTHF_polyglutamat	0.680297	0.818007	0.402746
5,10-methenylTHF_polyglutamate_+_NADPH_+_H+_<=>_5,10-methyleneTH	0.680297	0.818007	0.402746
5,10-methyleneTHF_polyglutamate_+_NADP+_<=>_5,10-methenylTHF_poly|	0.680297	0.818007	0.402746
THF_polyglutamate_+_formate_+_ATP_=>_10-formylTHF_polyglutamate_+_	0.680297	0.818007	0.402746
Interaction_of_BIM_with_BCL2_	0.390186	0.814474	0.0986206
Cholesterol_translocates_to_the_inner_mitochondrial_membrane	0.527968	0.417977	0.62
DNA_polymerase_epsilon_binds_at_the_origin	0.559516	0.414837	0.664955
Formation_of_DSIF_complex_	0.516544	#N/A	0.516544
Oligomerization_of_CARMA1	0.451919	0.396687	0.536004
Translocation_of_CARMA1_to_Plasma_membrane	0.451919	0.396687	0.536004
ornithine_=>_putrescine_+_CO2	0.259699	#N/A	0.259699
Phosphopantetheine_conjugation_of_the_ACP_domain_of_FAS	0.0190448	0.0190448	#N/A
Interaction_of_PUMA_and_Bcl-2	0.095992	#N/A	0.095992
Spermidine__=>_N-acetylated_spermidine	0.550319	#N/A	0.550319
Spermine__=>_N-acetylated_spermine	0.550319	#N/A	0.550319
oxaloacetate_+_GTP_=>_phosphoenolpyruvate_+_GDP_+_CO2_[cytosol]	0.231843	0.231843	#N/A
oxaloacetate_+_GTP_=>_phosphoenolpyruvate_+_GDP_+_CO2_[mitochondi	0.231843	0.231843	#N/A
Interaction_of_NOXA_and_Bcl-2	0.124962	#N/A	0.124962
Digestion_of_branched_starch_(amylopectin)_by_extracellular_amylase	0.999242	#N/A	0.999242
Digestion_of_linear_starch_(amylose)_by_extracellular_amylase	0.999242	#N/A	0.999242
Inactivation_of_APC/C_via_CDC20_sequestration	0.624364	#N/A	0.624364
TRAIL:TRAIL_receptor-2_Trimer_Binds_FADD	0.34977	0.34977	#N/A
FASL_binds_FAS_Receptor	0.869975	#N/A	0.869975
Trimerization_of_the_FASL:FAS_receptor_complex	0.869975	#N/A	0.869975
SLC7A9-mediated_exchange_of_extracellular_arginine,_lysine,_or_cystine_f	0.542418	0.542418	#N/A
pro-protein_S,_uncarboxylated_+_11_CO2_+_11_O2_+_11_vitamin_K_hydr	0.978249	0.987764	0.726915
MAD2_associates_with_the_Mad1_kinetochore_complex	0.75063	0.143547	0.97831
MAD2_converted__to_an_inhibitory_state_via_interaction_with_Mad1	0.75063	0.143547	0.97831
Release_of_activated_MAD2_from_kinetochores	0.75063	0.143547	0.97831
Binding_of_Grb2_to_Gab1	0.91114	0.91114	#N/A
Interaction_of_APE1_with_DNA_ligase_I	0.340931	0.330999	0.443105
Binding_of_Pol_zeta_to_lesioned_DNA_template	0.788906	0.180342	0.979716
Elongation_by_Pol_zeta_complex	0.788906	0.180342	0.979716
Formation_of_Pol_zeta_complex	0.788906	0.180342	0.979716
Phosphorylation_of_MDM2_at_serine-395_by_ATM_kinase	0.86908	0.86908	#N/A
Association_of_DFF40_with_DFF45	0.0190887	0.0146032	0.226486
Cleaved_fragments_of_DFF45_dissociate_from__DFF40	0.0190887	0.0146032	0.226486
Pannexin_1/Pannexin2_mediated_neuronal_gap_junction_communication	0.318479	0.781735	0.0740866
SLC7A11-mediated_exchange_of_extracellular_cysteine_and_cytosolic_glut	0.0206663	#N/A	0.0206663
Formation_of_cap_binding_complex_(CBC)	0.076653	#N/A	0.076653
NRIF_binds_to_p75NTR	0.136559	#N/A	0.136559
NGF_homodimer_binds_to_p75NTR	0.136559	#N/A	0.136559
Interaction_of_BAD_with_Bcl-2	0.354879	0.777134	0.0986206

Release_of_Cytochrome_c_from_mitochondria	0.68915	#N/A	0.68915
Phosphorylation_of_Cdc25A_at_Ser-123_in_response_to_DNA_damage	0.210138	0.210138	#N/A
Translocation_of_Cdc25_to_the_nucleus	0.210138	0.210138	#N/A
Translocation_of_Cdc25B_to_the_cytoplasm	0.210138	0.210138	#N/A
glycine_+_O2_=>_glyoxylate_+_H2O2_+_NH4+	0.701659	0.701659	#N/A
TNF_Binds_TNF-R1	0.102596	0.402254	0.0612713
Association_of_Ku_heterodimer_with_ends_of_DNA_double-strand_break	0.925251	0.898705	0.777119
Akt_phosphorylates_Foxo1_in_the_presence_of_IRS2	0.199384	0.328037	0.227142
Tryptophan_is_hydroxylated	0.188421	#N/A	0.188421
pregnenolone_+_PAPS_=>_pregnenolone_sulfate_+_PAP	0.0529338	#N/A	0.0529338
Phosphorylation_of__BRCA1_at_multiple_sites_by_ATM	0.83192	0.83192	#N/A
Reducing_equivalents_from_beta-oxidation_of_fatty_acids_transfer_to_ETF	0.672508	0.745605	0.488106
Phosphorylation_of_p53_at_ser-15_by_ATM_kinase	0.745897	0.562496	0.781489
Microprocessor_complex_cleaves_pri-miRNA_to_pre-miRNA	0.692776	0.692776	#N/A
CXADR_binds_to_AMICA1	0.0316408	0.0316408	#N/A
Nectin_2_binds_CD226	0.595134	0.815768	0.287818
pyrophosphate_+_H2O_=>_2_orthophosphate	0.703076	#N/A	0.703076
estrone_+_PAPS_=>_estrone_3-sulfate_+_PAP	0.0762643	#N/A	0.0762643
Phosphorylation_of_MDC1/NFBD1_by_ATM_(within__2_c-term_BRCT_dom	0.620673	0.620673	#N/A
2-amino-3-carboxymuconate_semialdehyde_=>_2-aminomuconate_semiald	0.447979	0.447979	#N/A
Loading_of_dopamine_into_synaptic_veiscles	0.303445	0.573976	0.180195
loading_of_Serotonin_in_synaptic_vesicles	0.303445	0.573976	0.180195
CD48_binds_CD244	0.822982	#N/A	0.822982
Displacement_of_hNTH1_glycosylase_by_APE1_at_the_AP_site	0.43147	#N/A	0.43147
Formation_of_FACT_complex	0.702207	0.702207	#N/A
Epithelial_cadherin_binds_to_KLRG1_in_mice	0.824028	0.824028	#N/A
adenosine_5-monophosphate_(AMP)_+_H2O_=>_adenosine_+_orthophosp	0.385002	0.11098	0.79024
Connexin_45/Connexin_36_mediated_neuronal_gap_junction_communicati	0.985106	0.985106	#N/A
Receptor_CXCR6_binds_CXCL16_ligand	0.808448	0.808448	#N/A
palmitoyl-CoA_+_2_NADPH_+_2_H+_=>_hexadecanol_+_2_NADP+_[FAR1]	0.98365	0.976871	0.847403
p75NTR_interacts_with_the_NOGO_receptor	0.20097	0.297848	0.256225
Association_of_Emi1_with_Cdh1	0.458345	0.306565	0.639441
Phosphorylation_of__NBS1_by_ATM	0.324364	0.562496	0.211269
Association_of_Emi1_with_Cdc20	0.070826	0.306565	0.0578904
Interaction_between_FEN1_and_PCNA	0.352736	0.352736	#N/A
Receptor_CCR6_binds_CCL20_ligand	0.451043	0.621658	0.314006
Dimerisation_of_Activated_Protein_1_(AP-1)_heterodimer	0.0705539	0.055805	0.312
Formation_of_Elongin_BC_complex	0.251852	#N/A	0.251852
Displacement_of_MBD4_glycosylase_by_APE1_at_the_AP_site	0.151087	#N/A	0.151087
Association_of_RAD51_with_RAD52:DNA_double-strand_break_ends	0.947089	#N/A	0.947089
Interaction_of_BIM_with_BCL-xl	0.409909	0.814474	0.11175
CRTAM_binds_to_NECL2	0.130161	0.150981	0.287818
Interaction_of_PUMA_and_Bcl-XL	0.10498	#N/A	0.10498
Transport_of_palmitoyl_carnitine_into_mitochondria	0.579915	0.696846	0.408646
Formation_of_SL1	0.703673	#N/A	0.703673
Interaction_of_NOXA_and_Bcl-XL	0.13578	#N/A	0.13578
Human_chorionic_gonadotropin_(hCG)_is_a_heterodimer	0.471217	#N/A	0.471217

Luteinizing_hormone_is_a_heterodimer	0.471217	#N/A	0.471217
Cdt1_associates_with_geminin	0.432992	#N/A	0.432992
Thyrotropin_is_a_heterodimer	0.542273	#N/A	0.542273
CyP60_chaperones_Basigin	0.139999	0.139999	#N/A
Isopentenyl_pyrophosphate_rearranges_to_dimethylallyl_pyrophosphate	0.0874438	0.0243357	0.521077
L-glutamate_gamma-semialdehyde_+_NAD+_=>_glutamate_+_NADH_+_H+	0.913819	0.913819	#N/A
Phosphorylation_and_activation_of_Chk1_by_ATM_kinase	0.340717	0.340717	#N/A
ATP_+_D-fructose_6-phosphate_=>_ADP_+_D-fructose_2,6-bisphosphate_[l	0.275148	0.275148	#N/A
D-fructose_2,6-bisphosphate_+_H2O_=>_D-fructose_6-phosphate_+_orthop	0.275148	0.275148	#N/A
Dephosphorylation_of_PF2K-Pase_by_PP2A_complex	0.275148	0.275148	#N/A
Fructose_2,6-bisphosphate_is_hydrolyzed_to_form_fructose-6-phosphate_a	0.275148	0.275148	#N/A
glutaredoxin_(oxidized)_+_glutathione_(reduced)_=>_glutaredoxin_(reduce	0.190474	#N/A	0.190474
Association_of_RAD51_with_BRCA2	0.836487	#N/A	0.836487
The_orphan_QRFP_receptor_can_bind_neuropeptide_QRFP	0.218056	0.218056	#N/A
Interaction__of_BAD_with_BCL-xl___	0.374058	0.777134	0.11175
D2-like_dopamine_receptors_bind_to_dopamine	0.890017	0.917026	0.636029
Arachidonic_acid_oxidised_to_PGG2	0.288543	0.588234	0.155748
Peroxidative_reduction_of_PGG2_to_PGH2	0.288543	0.588234	0.155748
Basigin_interacts_with_CD43	0.695629	0.364146	0.857739
Interaction_of_APE1_with_FEN1	0.353087	0.348042	0.443105
Corticotropin_cleavage_from_POMC	0.34722	0.34722	#N/A
Tachykinin_receptor_2_(NK2)_binds_to_nuerokinin_A	0.447267	0.349284	0.578898
asparagine_+_tRNA(Asn)_+_ATP_=>_Asn-tRNA(Asn)_+_AMP_+_pyrophosph	0.383648	#N/A	0.383648
lysophosphatidic_acid_+_fatty_acyl_CoA__=>_phosphatidic_acid_+_CoA_(3)	0.685631	0.685631	#N/A
Prolactin-releasing_hormone_receptor_binds_PRH	0.556519	#N/A	0.556519
Recruitment_and_activation_of_Chk1	0.138178	0.138178	#N/A
glucosaminyl-acyl-PI_+_dolichol_phosphate_D-mannose_->_mannose(al1-4)	0.814938	0.814938	#N/A
Tachykinin_receptor_1_(NK1)_binds_to_substance_P	0.336493	#N/A	0.336493
threonine_+_tRNA(Thr)_+_ATP_=>_Thr-tRNA(Thr)_+_AMP_+_pyrophosphate	0.635432	0.635432	#N/A
Tachykinin_receptor_3_(NK3)_binds_to_neurokinin_B	0.299785	0.299785	#N/A
Orexin_2_receptor_can_bind_orexin-B_neuropeptide	0.44712	0.809816	0.143228
IRAK1-P(n)_combines_with_TRAF6	0.437722	0.437722	#N/A
Alpha-2_adrenoceptors_bind_catecholamines	0.969849	0.969849	#N/A
Exportin_complex_translocates_pre-miRNA_to_cytosol	0.608319	#N/A	0.608319
Exportin-5_recognizes_3_overhang_of_pre-miRNA_	0.608319	#N/A	0.608319
Nociceptin_receptor_binds_to_nociceptin	0.549165	0.549165	#N/A
D-galactose_1-phosphate_+_UDP-glucose_<=>_D-glucose_1-phosphate_+_L	0.752317	0.752317	#N/A
Binding_of_ADAR2_homodimer_to_dsRNA_duplex	0.337172	0.337172	#N/A
Deamination_at_C6_position_of_adenosine_in_Editosome_(ADAR2)	0.337172	0.337172	#N/A
Formation_of_ADAR2__homodimer	0.337172	0.337172	#N/A
Displacement_of_MYH_glycosylase_by_APE1_at_the_AP_site	0.96016	0.995601	0.443105
Follitropin_is_a_heterodimer	0.635118	0.511296	0.676965
Two_NPFF_receptors_can_bind_neuropeptide_FF	0.170612	0.170612	#N/A
SLC38A5-mediated_uptake_of_glutamine,_histidine,_asparagine,_and_serin	0.207237	0.786562	0.025653
Displacement_of_TDG_glycosylase_by_APE1_at_the_AP_site	0.208315	#N/A	0.208315
SLC6A15-mediated_amino_acid_uptake	0.139273	0.251196	0.194387
histidine_+_tRNA(His)_+_ATP_=>_His-tRNA(His)_+_AMP_+_pyrophosphate	0.362136	0.340507	0.464795

Orexin_1_receptor_can_bind_orexin-A_neuropeptide	0.360712	0.71264	0.143228
tryptophan_+_tRNA(Trp)_+_ATP_=>_Trp-tRNA(Trp)_+_AMP_+_pyrophospha	0.917571	0.987253	0.393121
Vasopressin_receptor_type_2_bind_vasopressin	0.93532	0.919571	0.770651
Displacement_of_hSMUG1_glycosylase_by_APE1_at_the_AP_site	0.0855455	#N/A	0.0855455
Grb2_binds_Sos	0.972775	0.972775	#N/A
SLC6A18-mediated_glycine_uptake	0.659506	0.578445	0.648945
SLC6A19-mediated_uptake_of_neutral_amino_acids	0.659506	0.578445	0.648945
Displacement_of_hOGG1_glycosylase_by_APE1_at_the_AP_site	0.448955	0.484426	0.443105
NPC1L1_inactivation_by_ezetimibe	0.951067	0.897459	0.858137
NPC1L1-mediated_cholesterol_uptake	0.951067	0.897459	0.858137
D1-like_dopamine_receptors_bind_to_dopamine	0.658889	0.658889	#N/A
alpha-methylbutyryl-CoA_+_FAD_=>_tiglyl-CoA_+_FADH2	0.00437343	0.00437343	#N/A
Butanoyl-CoA+FAD<=>Crotonoyl-CoA+FADH2	0.00437343	0.00437343	#N/A
Hexanoyl-CoA+FAD<=>trans-Hex-2-enoyl-CoA+FADH2	0.00437343	0.00437343	#N/A
Displacement_of__MPG_glycosylase_by_APE1_at_the_AP_site	0.411194	#N/A	0.411194
Digestion_of_triacylglycerols_by_extracellular_pancreatic_lipase-related_pr	0.581175	0.666531	0.443747
mannose_(a1-2)_mannose_(a1-6)_(ethanolamineP)_mannose_(a1-4)_glucos	0.0909979	0.284128	0.0939466
Receptor_CXC1_binds_to_the_ligands_lymphotactin_and_cytokine_SCM-1_	0.818768	#N/A	0.818768
H3_and_H4_receptors_binds_histamine	0.675057	0.630421	0.620933
CD40_interacting_with_CD40L	0.83137	0.83137	#N/A
serine_+_tRNA(Ser)_+_ATP_=>_Ser-tRNA(Ser)_+_AMP_+_pyrophosphate	0.879789	#N/A	0.879789
Conversion_of_Ran-GDP_to_Ran-GTP	0.23684	#N/A	0.23684
alanine_+_tRNA(Ala)_+_ATP_=>_Ala-tRNA(Ala)_+_AMP_+_pyrophosphate	0.405759	0.512645	0.355847
5Beta-cholestan-7alpha,12alpha,24(S)-triol-3-one_is_reduced_to_5beta-cho	0.635891	0.635891	#N/A
5Beta-cholestan-7alpha,12alpha,27-triol-3-one_is_reduced_to_5beta-choles	0.635891	0.635891	#N/A
5beta-cholestan-7alpha,24(S)-diol-3-one_is_reduced_to_5beta-cholestan-3a	0.635891	0.635891	#N/A
5beta-cholestan-7alpha,27-diol-3-one_is_reduced_to_5beta-cholestan-3alpl	0.635891	0.635891	#N/A
5beta-cholestan-7alpha-ol-3-one_is_reduced_to_5beta-cholestan-3alpha,_7	0.635891	0.635891	#N/A
5Beta-cholesten-7alpha,_12alpha-diol-3-one_is_reduced_to_5beta-cholesta	0.635891	0.635891	#N/A
ATP_+_beta-D-fructose_=>_ADP_+_D-fructose_1-phosphate	0.987096	0.987096	#N/A
Transport_of_Choline	0.0877234	#N/A	0.0877234
acetoacetate+succinyl-CoA_<=>_acetoacetyl-CoA+succinate	0.545581	0.545581	#N/A
Spermine_is_oxidized_to_spermidine	0.0227744	#N/A	0.0227744
Pro-prothrombin_is_transported_from_the_endoplasmic_reticulum_to_the_	0.613716	0.613716	#N/A
benzoyl-CoA_+_glycine_=>_benzoyl_glycine_(hippuric_acid)_+_Coenzyme_A	0.176824	#N/A	0.176824
salicylate-CoA_+_glycine_=>_salicyluric_acid_+_Coenzyme_A	0.176824	#N/A	0.176824
p-hydroxyphenylpyruvate_+_O2_=>_homogentisate_+_CO2	0.166076	0.166076	#N/A
tryptophan_+_O2_=>_N-formylkynurenine_[TDO]	0.236677	0.236677	#N/A
Oxidation_of_cortisol_to_yield_cortisone	0.288834	0.288834	#N/A
Exocytosis_of_kininogen	0.628251	0.628251	#N/A
3-hydroxyanthranilate_+_O2_=>_2-amino-3-carboxymuconate_semialdehyd	0.199015	0.199015	#N/A
Release_of_Titin	0.32334	0.32334	#N/A
3-hydroxykynurenine_+_H2O_=>_3-hydroxyanthranilate_+_alanine	0.811972	0.811972	#N/A
antithrombin_III_+_heparin_->_antithrombin_III:heparin	0.354693	0.354693	#N/A
4a-hydroxytetrahydrobiopterin_=>_q-dihydrobiopterin_+_H2O	0.146641	#N/A	0.146641
UTP_+_D-glucose_1-phosphate_<=>_pyrophosphate_+_UDP-glucose_[liver]	0.583986	0.583986	#N/A
UTP_+_D-glucose_1-phosphate_<=>_pyrophosphate_+_UDP-glucose_[musc	0.583986	0.583986	#N/A

Surface_deployment_of_LAMP2	0.713451	#N/A	0.713451
Exocytosis_of_histidine_rich_glycoprotein	0.714463	0.714463	#N/A
Pro-factor_IX_is_transported_from_the_endoplasmic_reticulum_to_the_Go	0.00255619	0.00255619	#N/A
lysine_+_alpha-ketoglutarate_+NADPH_+_H+_=>_saccharopine_+_NADP+_+	0.267865	0.267865	#N/A
saccharopine_+_NAD+_+_H2O_=>_alpha-aminoadipic_semialdehyde_+_glut	0.267865	0.267865	#N/A
Chenodeoxycholoyl_CoA_reacts_with_glycine_or_taurine_to_form_glycoche	0.49425	0.49425	#N/A
Choloyl_CoA_reacts_with_glycine_or_taurine_to_form_glycocholate_or_tau	0.49425	0.49425	#N/A
Cytosolic_chenodeoxycholoyl-CoA_or_choloyl-CoA_are_conjugated_with_gl	0.49425	0.49425	#N/A
Exocytosis_of_osteonectin	0.924166	#N/A	0.924166
Ethanol_+_NAD+_<=>_Acetaldehyde_+_NADH_+_H+_[class_II,_pi/pi]	0.345658	#N/A	0.345658
Apolipoprotein_A-I_binds_membrane-associated_cholesterol_and_phospho	0.0581266	0.0581266	#N/A
Discoidal_HDL_binds_membrane-associated_free_cholesterol	0.0581266	0.0581266	#N/A
pre-beta_HDL_binds_membrane-associated_cholesterol_and_phospholipid_	0.0581266	0.0581266	#N/A
Release_of_Apolipoprotein_A-I_	0.0581266	0.0581266	#N/A
Spherical_HDL_binds_membrane-associated_free_cholesterol_and_phospho	0.0581266	0.0581266	#N/A
7-hydroxycoumarin_can_form_an_O-glucuronide	0.663927	#N/A	0.663927
Formation_of_N-glucuronides	0.663927	#N/A	0.663927
Formation_of_O-glucuronides	0.663927	#N/A	0.663927
carbamoyl_phosphate_+_ornithine_=>_citrulline_+_orthophosphate	0.102689	0.102689	#N/A
4-Cholesten-7alpha,24(S)-diol-3-one_is_12alpha-hydroxylated_to_4-cholest	0.0557297	#N/A	0.0557297
4-Cholesten-7alpha,27-diol-3-one_is_12alpha-hydroxylated_to_4-Cholesten-	0.0557297	#N/A	0.0557297
4-Cholesten-7alpha-ol-3-one_is_12alpha-hydroxylated_to_7-alpha,12-alpha-	0.0557297	#N/A	0.0557297
HMG_CoA_=>_acetoacetic_acid+_acetyl_CoA	0.185008	0.185008	#N/A
4,8-dimethylnonanoyl-CoA_+_carnitine_=>_4,8-dimethylnonanoylcarnitine_	0.524869	0.524869	#N/A
6-mercaptopurine_can_be_S-methylated	0.243602	0.243602	#N/A
phytanoyl-CoA_+_2-oxoglutarate_+_O2_=>_2-hydroxyphytanoyl-CoA_+_suc	0.882702	#N/A	0.882702
Connexin_oligomerization_in_endoplasmic_reticulum_membrane	0.16292	#N/A	0.16292
Exocytosis_of_Fructose-bisphosphate_aldolase	0.675942	0.675942	#N/A
Exocytosis_of_Coagulation_factor_XIIIA	0.715568	0.715568	#N/A
Diiodinated_tyrosine_can_be_deiodinated	0.116907	0.116907	#N/A
Monoiodinated_tyrosine_can_be_deiodinated	0.116907	0.116907	#N/A
n_UDP-glucose_+_glycogenin-1_=>_n_UDP_+_{(1,4)-alpha-D-glucosyl}n_glyc	0.69754	0.69754	#N/A
n_UDP-glucose_+_glycogenin-2_=>_n_UDP_+_{(1,4)-alpha-D-glucosyl}n_glyc	0.69754	0.69754	#N/A
Exocytosis_of_plasminogen	0.929255	0.929255	#N/A
Calcidiol_binds_to_DBP_in_the_bloodstream_and_is_delivered_to_the_kidr	0.769281	0.769281	#N/A
DBP:Calcidiol_is_sequestered_by_cubilin_on_the_cell_surface	0.769281	0.769281	#N/A
DBP:Calcidiol_translocates_into_lysosomes	0.769281	0.769281	#N/A
Vitamin_D3_translocates_into_the_cytosol	0.769281	0.769281	#N/A
Vitamin_D3_translocates_to_the_blood_serum_and_is_transported_to_the_	0.769281	0.769281	#N/A
gamma-butyrobetaine_semialdehyde_+_NAD+_=>_gamma-butyrobetaine_+	0.897865	#N/A	0.897865
Exocytosis_of_Insulin-like_growth_factor	0.351096	#N/A	0.351096
S-adenoylhomocysteine_is_hydrolyzed	0.297504	0.297504	#N/A
homogentisate_+_O2_=>_maleylacetoacetate	0.0932862	0.0932862	#N/A
Zymosterol_is_isomerized_to_cholesta-7,24-dien-3beta-ol	0.873396	0.873396	#N/A
CYP2F1_dehydrogenates_3-methylindole	0.997043	0.997043	#N/A
3,4-dihydroxybenzoate_can_be_O-methylated	0.949856	0.949856	#N/A
Methylation_of_3,4-dihydroxypheylacetic_acid_to_homovanillic_acid	0.949856	0.949856	#N/A

methylation_of_Dopamine_to_form_3-Methoxytyramine	0.949856	0.949856	#N/A
2_ATP_+_NH4+_+_HCO3-_=>_2_ADP_+_orthophosphate_+_carbamoyl_pho	0.329461	0.329461	#N/A
carbamoyl_phosphate_+_L-aspartate_<=>_N-carbamoyl_L-aspartate_+_orth	0.329461	0.329461	#N/A
L-glutamine_+_2_ATP_+_HCO3-_+_H2O_=>_carbamoyl_phosphate_+_L-glut	0.329461	0.329461	#N/A
N-carbamoyl_L-aspartate_+_H+_<=>_(S)-dihydroorotate_+_H2O	0.329461	0.329461	#N/A
A_phospho-ribosyl_group_is_added_to_quinolinate	0.117358	0.117358	#N/A
Pro-factor_X_is_transported_from_the_endoplasmic_reticulum_to_the_Gol	0.85782	0.85782	#N/A
Surface_deployment_of_GpIV	0.664248	0.664248	#N/A
kynurenine_+_O2_+_NADPH_+_H+_=>_3-hydroxykynurenine_+_NADP+_+_I	0.211741	#N/A	0.211741
3,7,24THCA_is_conjugated_with_Coenzyme_A_(SLC27A5_BACS)	0.783304	0.783304	#N/A
Cytosolic_cholate_and_chenodeoxycholate_are_conjugated_with_Coenzym	0.783304	0.783304	#N/A
DHCA_is_conjugated_with_Coenzyme_A_(SLC27A5_BACS)	0.783304	0.783304	#N/A
TetraHCA_is_conjugated_with_Coenzyme_A_(SLC27A5_BACS)	0.783304	0.783304	#N/A
THCA_is_conjugated_with_Coenzyme_A_(SLC27A5_BACS)	0.783304	0.783304	#N/A
SLC38A4_(ATA3)-mediated_uptake_of_arginine_and_lysine	0.352255	0.352255	#N/A
Exocytosis_of_complement_factor_D	0.08247	#N/A	0.08247
phosphoenolpyruvate_[mitochondrial_matrix]_+_citrate_[cytosol]_=>_phos	0.264656	0.264656	#N/A
Acetaminophen_oxidised_to_N-acetylbenzoquinoneimine_(NAPQI)	0.794144	#N/A	0.794144
Benzene_is_hydroxylated_to_phenol	0.794144	#N/A	0.794144
Dehalogenation_of_carbon_tetrachloride_to_form_a_free_radical	0.794144	#N/A	0.794144
Dehalogenation_of_the_poly-halogenated_hydrocarbon_Halothane_to_forr	0.794144	#N/A	0.794144
MEOS_oxidizes_ethanol_to_acetaldehyde	0.794144	#N/A	0.794144
Vinyl_chloride_is_oxidized_to_2-Chloroethylene_oxide	0.794144	#N/A	0.794144
beta-ureidoisobutyrate_+_H2O_=>_beta-aminoisobutyrate_+_NH4+_+_CO2	0.182474	0.182474	#N/A
beta-ureidopropionate_+_H2O_=>_beta-alanine_+_NH4+_+_CO2	0.182474	0.182474	#N/A
Ethanol_+_NAD+_<=>_Acetaldehyde_+_NADH_+_H+_[class_I,_alpha/alpha]	0.525242	0.525242	#N/A
aspartate_+_glutamine_+_ATP_<=>_asparagine_+_glutamate_+_AMP_+_py	0.38116	0.38116	#N/A
5,10-methyleneTHF_polyglutamate_+_glycine_<=>_tetrahydrofolate_polyglu	0.373125	0.373125	#N/A
beta-hydroxy-trimethyllysine_=>_gamma-butyrobetaine_aldehyde_+_glycine	0.373125	0.373125	#N/A
Tetrahydrofolate_polyglutamate_(THF_polyglutamate)_+_serine_<=>_5,10-i	0.373125	0.373125	#N/A
25-Hydroxylation_of_vitamin_D3_in_liver	0.000235072	0.000235072	#N/A
D-beta_hydroxybutyrate+NAD+_<=>_acetoacetate+NADH+H+	0.295177	0.295177	#N/A
Reduction_of_Acetoacetate_to_beta-Hydroxybutyrate_	0.295177	0.295177	#N/A
glutamate_+_acetyl_CoA_=>_N-acetyl_glutamate_+_CoA	0.960188	#N/A	0.960188
Exocytosis_of_alpha_2_antiplasmin	0.575699	0.575699	#N/A
fumarylacetoacetate_=>_fumarate_+_acetoacetate	0.63604	0.63604	#N/A
Conversion_of_Diacylglycerol_to_Triacylglycerol	0.878728	0.878728	#N/A
FMN_can_be_hydrolyzed_back_to_riboflavin	0.336043	#N/A	0.336043
Cholesta-7,24-dien-3beta-ol_is_desaturated_to_form_cholesta-5,7,24-trien-	0.0995716	0.0995716	#N/A
2_H2O2_=>_O2_+_2_H2O	0.322732	0.322732	#N/A
2-hydroxyphytanoyl-CoA_=>_pristanal_+_formyl-CoA	0.100521	0.100521	#N/A
Binding_of_ACF_to_stem-looped_RNA	0.626853	0.626853	#N/A
Pro-protein_Z_is_transported_from_the_endoplasmic_reticulum_to_the_Go	0.58381	0.58381	#N/A
dc-Adenosyl_methionine_+_Spermidine_=>_Spermine_+_5-methylthioaden	0.697934	0.697934	#N/A
glutaryl-CoA_+_FAD_=>_crotonyl-CoA_+_FADH2_+_CO2	0.573411	#N/A	0.573411
Exocytosis_of_low-affinity_platelet_factor_IV_	0.410886	#N/A	0.410886
Exocytosis_of_Platelet_factor_4	0.410886	#N/A	0.410886

Formation_of_Caspase-8_dimer	0.722912	#N/A	0.722912
Translocation_of_active_caspase-8_to_the_mitochondrial_membrane	0.722912	#N/A	0.722912
2-acylglycerol_+_H2O_->_glycerol_+_fatty_acid	0.470465	0.470465	#N/A
alpha-aminoadipate_+_alpha-ketoglutarate_=>_alpha-ketoadipate_+_glutan	0.979195	0.979195	#N/A
malate_[mitochondrial_matrix]_+_orthophosphate_[cytosol]_<=>_malate_[	0.412397	#N/A	0.412397
Isomerization_of_(2R)-pristanoyl-CoA_to_(2S)-pristanoyl-CoA	0.504387	0.504387	#N/A
Isomerization_of_25(R)_DHCA-CoA_to_25(S)_DHCA-CoA	0.504387	0.504387	#N/A
Isomerization_of_25(R)_TetraHCA-CoA_to_(24R,_25R)_3alpha,7alpha,12alpl	0.504387	0.504387	#N/A
Isomerization_of_25(R)_THCA-CoA_to_25(S)_THCA-CoA	0.504387	0.504387	#N/A
Isomerization_of_3,7,24THCA-CoA_to_(24R,_25R)_3alpha,7alpha,24-trihydr	0.504387	0.504387	#N/A
Pro-protein_C_is_transported_from_the_endoplasmic_reticulum_to_the_G	0.561184	0.561184	#N/A
Reduction_of_desmosterol_to_cholesterol	0.204478	0.204478	#N/A
N-formiminoglutamate_+_tetrahydrofolate_=>_glutamate_+_5-formiminote	0.320731	0.320731	#N/A
histidine_=>_urocanate_+_NH4+	0.302713	0.302713	#N/A
5-phosphoribosyl-5-aminoimidazole-4-N-succinocarboxamide_(SAICAR)_<=>	0.504049	0.504049	#N/A
adenylosuccinate_=>_adenosine_5-monophosphate_+_fumarate	0.504049	0.504049	#N/A
D-ribulose_5-phosphate_<=>_xylulose_5-phosphate	0.271803	0.271803	#N/A
xylulose_5-phosphate_<=>_D-ribulose_5-phosphate	0.271803	0.271803	#N/A
Fumarate_+_H2O_<=>_(S)-Malate	0.77825	#N/A	0.77825
CD84_homodimerises	0.206616	0.206616	#N/A
alpha-D-glucose_(extracellular)_<=>_alpha-D-glucose_(cytosol)_[GLUT2]	0.637656	#N/A	0.637656
Transport_(efflux)_of_fructose,_galactose,_and_glucose_by_GLUT2	0.637656	#N/A	0.637656
25-hydroxycholesterol_is_7alpha-hydroxylated_by_CYP7B1	0.214579	#N/A	0.214579
27-hydroxycholesterol_is_7alpha-hydroxylated	0.214579	#N/A	0.214579
Guanine_+_H2O_=>_Xanthine_+_NH3	0.19238	#N/A	0.19238
PMP34-mediated_exchange_of_cytosolic_ATP_for_peroxisomal_AMP	0.95938	0.95938	#N/A
thymine_+_NADPH_+_H+_=>_5,6-dihydrothymine_+_NADP+	0.672618	0.672618	#N/A
uracil_+_NADPH_+_H+_=>_5,6-dihydrouracil_+_NADP+	0.672618	0.672618	#N/A
PS-GAP_interacts_ with_caspase-activated_PAK-2p34	0.0685033	0.0685033	#N/A
Interaction_of_PAK-2p34_with_PS-GAP_results_in_accumulation_of_PAK-2p	0.0685033	0.0685033	#N/A
7alpha-hydroxycholesterol_is_oxidized_and_isomerized_to_4-cholesten-7al	0.902847	0.902847	#N/A
Cholest-5-ene-3beta,7alpha,24(S)-triol_is_oxidized_and_isomerized_to_4-ch	0.902847	0.902847	#N/A
Cholest-5-ene-3beta,7alpha,27-triol_is_oxidized_and_isomerized_to_4-chol	0.902847	0.902847	#N/A
urocanate_+_H2O_=>_4-imidazolone-5-propionate	0.424425	0.424425	#N/A
Conversion_of_Glycerol_to_Glycerol-3-phosphate	0.082408	0.082408	#N/A
2-deoxycytidine_5-monophosphate_(dCMP)_+_H2O_=>_2-deoxycytidine_+_	0.561318	0.561318	#N/A
6-phospho-D-gluconate_+_NADP+_=>_D-ribulose_5-phosphate_+_CO2_+_N	0.359877	0.359877	#N/A
tyrosine_+_alpha-ketoglutarate_=>_p-hydroxyphenylpyruvate_+_glutamate	0.414893	0.414893	#N/A
2-deoxyguanosine_+_orthophosphate_<=>_guanine_+_2-deoxy-D-ribose_1-	0.503761	0.503761	#N/A
2-deoxyinosine_+_orthophosphate_<=>_hypoxanthine_+_2-deoxy-D-ribose_	0.503761	0.503761	#N/A
guanine_+_2-deoxy-D-ribose_1-phosphate_<=>_2-deoxyguanosine_+_ortho	0.503761	0.503761	#N/A
guanine_+_D-ribose_1-phosphate_<=>_guanosine_+_orthophosphate	0.503761	0.503761	#N/A
guanosine_+_orthophosphate_<=>_guanine_+_D-ribose_1-phosphate	0.503761	0.503761	#N/A
hypoxanthine_+_2-deoxy-D-ribose_1-phosphate_<=>_2-deoxyinosine_+_ort	0.503761	0.503761	#N/A
hypoxanthine_+_D-ribose_1-phosphate_<=>_inosine_+_orthophosphate	0.503761	0.503761	#N/A
inosine_+_orthophosphate_<=>_hypoxanthine_+_D-ribose_1-phosphate	0.503761	0.503761	#N/A
Association_of_Cx43__with_ZO-1	0.746476	#N/A	0.746476

Association_of_Golgi__transport_vesicles_with_microtubules_and_transpor	0.746476	#N/A	0.746476
Budding_of_connexon-containing_transport_vesicles_from_the_Golgi	0.746476	#N/A	0.746476
Connexin_oligomerization_in_Trans-Golgi_Network_(TGN)	0.746476	#N/A	0.746476
Docking_of_connexons_into_junctional,_double-membrane_spanning_chan	0.746476	#N/A	0.746476
Insertion_of_connexons_into_the_plasma_membrane_resulting_in_the_for	0.746476	#N/A	0.746476
Transport_of_connexins_to_the_Trans-Golgi_Network_(TGN)	0.746476	#N/A	0.746476
Association_of_LBP_with_LPS	0.630557	0.630557	#N/A
glyoxylate_+_NADPH_+_H+_=>_glycolate_+_NADP+	0.757895	0.757895	#N/A
Cholesterol_is_hydroxylated_to_7alpha-hydroxycholesterol_by_CYP7A1	5.57915E-05	#N/A	5.57915E-05
NAD+_is_phosphorylated_to_NADP+	0.519149	0.519149	#N/A
Adenylation_of_phosphopantetheine	0.937786	0.937786	#N/A
Phosphorylation_of_dephospho-CoA_to_produce_CoA	0.937786	0.937786	#N/A
glutamine_+_H2O_=>_glutamate_+_NH4+_[kidney]	0.045686	0.045686	#N/A
L-Glutamine_conversion_to_L-Glutamate	0.045686	0.045686	#N/A
crosslinked_fibrin_multimer_+_tissue_plasminogen_activator_(one-chain)_-	0.871785	#N/A	0.871785
Exocytosis_of_EGF	0.888895	#N/A	0.888895
Exocytosis_of_factor_V	0.669153	0.669153	#N/A
Acetaminophen_can_form_an_O-_sulfate_conjugate	0.052903	0.052903	#N/A
Dopamine_can_form_an_O-sulfate_conjugate	0.052903	0.052903	#N/A
N-hydroxy-4-aminobiphenyl_can_form_a_sulfate_conjugate	0.052903	0.052903	#N/A
Phenol_can_form_a_sulfate_conjugate	0.052903	0.052903	#N/A
A_phosphoribosyl_group_is_added_to_nicotinate_to_form_nicotinate_mon	0.682968	0.682968	#N/A
3-ketopristanoyl-CoA_+_CoASH_=>_4,8,12-trimethyltridecanoyl-CoA_+_prop	0.605533	0.605533	#N/A
Thiolysis_of_3alpha,7alpha,12alpha-trihydroxy-5beta-cholan-24-one-CoA_yi	0.605533	0.605533	#N/A
Thiolysis_of_3alpha,7alpha-dihydroxy-5beta-cholan-24-one-CoA_yields_che	0.605533	0.605533	#N/A
SLC38A3-mediated_uptake_of_glutamine,_histidine,_asparagine,_and_alani	0.901556	0.901556	#N/A
UDP-galactose_<=>_UDP-glucose	0.000517818	0.000517818	#N/A
Decarboxylation_of_5-hydroxytryptophan_forms_serotonin	0.162834	#N/A	0.162834
Dopa_is_decarboxylated_to_dopamine	0.162834	#N/A	0.162834
Addition_of_isopentenyl_pyrophosphate_to_DMAPP	0.50863	0.50863	#N/A
Another_isopentenyl_pyrophosphate_is_added_to_geranyl_pyrophosphate	0.50863	0.50863	#N/A
DHF_is_reduced_to_tetrahydrofolate_(THF)	0.0964226	0.0964226	#N/A
Folate_is_reduced_to_dihydrofolate_(DHF)	0.0964226	0.0964226	#N/A
Exocytosis_of_Metalloproteinase_inhibitor_1	0.0522023	#N/A	0.0522023
ornithine_(cytosolic)_+_citrulline_(mitochondrial)_=>_ornithine_(mitochond	0.218103	0.218103	#N/A
Microtubule-independent_trafficking_of_connexons_to_the_plasma_memb	0.0321673	#N/A	0.0321673
N-acetylspermidine_is_oxidized_to_putrescine	0.000181641	0.000181641	#N/A
N-acetylspermine_is_oxidised_to_spermidine	0.000181641	0.000181641	#N/A
Exocytosis_of_plasminogen_activator_inhibitor	0.698443	0.698443	#N/A
2_glutathione,_reduced_+_H2O2_=>_glutathione,_oxidized_+_2_H2O	0.879101	#N/A	0.879101
Release_of_Cofilin-1	0.316454	#N/A	0.316454
beta-estradiol_+_PAPS_=>_beta-estradiol_3-sulfate_+_PAP	0.0170591	#N/A	0.0170591
CYP2U1_can_omega-hydroxylate_arachidonate	8.13263E-07	8.13263E-07	#N/A
Ethylene_is_oxidized_to_Ethylene_oxide_by_CYP1A1	8.13263E-07	8.13263E-07	#N/A
Hydroxylation_of_pregnenolone_to_form_17alpha-hydroxypregnenolone	8.13263E-07	8.13263E-07	#N/A
Hydroxylation_of_progesterone_to_form_17alpha-hydroxyprogesterone	8.13263E-07	8.13263E-07	#N/A
N-atom_dealkylation_of_caffeine	8.13263E-07	8.13263E-07	#N/A

N-hydroxylation_of_4-aminobiphenyl	8.13263E-07	8.13263E-07	#N/A
S-atom_dealkylation_of_6-methylmercaptopurine	8.13263E-07	8.13263E-07	#N/A
Side_chain_cleavage_of_17alpha-hydroxyprenenolone_to_yield_DHA	8.13263E-07	8.13263E-07	#N/A
Side_chain_cleavage_of_17alpha-hydroxyprogesterone_to_form_4-Androst	8.13263E-07	8.13263E-07	#N/A
Exocytosis_of_albumin	0.880736	0.880736	#N/A
Serum_albumin_binds_2-lysophosphatidylcholine	0.880736	0.880736	#N/A
adenosine_+_ATP_=>_adenosine_5-monophosphate_(AMP)_+_ADP	0.304072	0.304072	#N/A
Secretion_and_activation_of_the_latent_large_complex_of_TGF-beta1	0.404519	#N/A	0.404519
q-dihydrobiopterin_+_NADH_+_H+_=>_tetrahydrobiopterin_+_NAD+	0.0703352	0.0703352	#N/A
2-oxoglutarate_[mitochondrial_matrix]_+_2-oxoadipate_[cytosol]_<=>_2-ox	0.00177623	0.00177623	#N/A
Putrescine_+_dc-Adenosyl_methionine__=>_Spermidine_+_5-methylthioad	0.0294544	#N/A	0.0294544
Arginine<=>Agmatine+CO2	0.289289	0.289289	#N/A
Acetyl-ACP(intermediate)__=>_Acetyl-synthase_	0.0258374	0.0258374	#N/A
Condensation_of_Malonyl-ACP_and_Acetate_on_different_monomers_of_F	0.0258374	0.0258374	#N/A
Conversion_of_Malonyl-CoA_to_Malonyl-ACP	0.0258374	0.0258374	#N/A
Dehydration_of_beta-Hydroxybutyryl-ACP_to_Crotonoyl-ACP_	0.0258374	0.0258374	#N/A
Formation_of_Acetyl-ACP(intermediate)	0.0258374	0.0258374	#N/A
Formation_of_fatty_acid_synthase_(FAS)_dimer	0.0258374	0.0258374	#N/A
Hydrolysis_of_Palmitoyl-ACP_to_Palmitic_Acid	0.0258374	0.0258374	#N/A
Reduction_of_Acetoacetyl-ACP_to_beta-Hydroxybutyryl-ACP	0.0258374	0.0258374	#N/A
Reduction_of_Crotonoyl-ACP_to_Butyrl-ACP	0.0258374	0.0258374	#N/A
Cytosolic_folate_export_across_the_plasma_membrane	0.513506	#N/A	0.513506
Extracellular_folate_import_across_the_plasma_membrane	0.513506	#N/A	0.513506
Riboflavin_is_phosphorylated_to_FMN	0.159064	0.159064	#N/A
uPAR-acyl-GPI_+_H2O_->_uPAR_+_long-chain_fatty_acid	0.189367	0.189367	#N/A
Exocytosis_of_multimerin	0.875542	#N/A	0.875542
Pro-factor_VII_is_transported_from_the_endoplasmic_reticulum_to_the_G	0.977928	0.977928	#N/A
malate_[mitochondrial_matrix]_+_alpha-ketoglutarate_[cytosol]_<=>_malat	0.610606	#N/A	0.610606
PRDM4_translocates_to_the_nucleus	0.109601	0.109601	#N/A
UDP-glucose_is_oxidised_to_UDP-glucuronate	0.587954	#N/A	0.587954
Formation_of_Malonyl-CoA_from_Acetyl-CoA_(liver)	0.0313975	0.0313975	#N/A
Generation_of_Cytoplasmic_Acetyl_CoA_from_Citrate	0.0291875	0.0291875	#N/A
D-glyceraldehyde_3-phosphate_+_D-fructose_6-phosphate___<=>_xylulose_	0.666281	0.666281	#N/A
D-glyceraldehyde_3-phosphate_+_sedoheptulose_7-phosphate<=>_xylulose	0.666281	0.666281	#N/A
ribose_5-phosphate_+_xylulose_5-phosphate_<=>_sedoheptulose_7-phosp	0.666281	0.666281	#N/A
xylulose_5-phosphate_+_D-erythrose_4-phosphate_<=>_D-glyceraldehyde_	0.666281	0.666281	#N/A
argininosuccinate_<=>_fumarate_+_arginine	0.430045	0.430045	#N/A
alpha-methyl-beta-hydroxybutyryl-CoA_+_NAD+_<=>_alpha-methylacetoac	0.60298	#N/A	0.60298
sequestered_tissue_factor_->_tissue_factor	0.517288	0.517288	#N/A
Squalene_2,3-epoxide_cyclizes,_forming_lanosterol	0.315398	0.315398	#N/A
acetyl-CoA_+_carnitine_=>_acetylcarnitine_+_CoASH	0.33437	0.33437	#N/A
propionyl-CoA_+_carnitine_=>_propionylcarnitine_+_CoASH	0.33437	0.33437	#N/A
alpha-ketoglutarate_+_NH4+_+_NADPH_+_H+_=>_glutamate_+_NADP+	0.459555	0.459555	#N/A
glutamate_+_NAD+_=>_alpha-ketoglutarate_+_NH4+_+_NADH_+_H+	0.459555	0.459555	#N/A
Release_of_Adenylyl_cyclase-associated_protein_1	0.464632	#N/A	0.464632
4-carboxycholesta-8(9),24-dien-3beta-ol_is_decarboxylated_and_oxidized_t	0.012204	0.012204	#N/A
4-methyl,4-carboxycholesta-8(9),24-dien-3beta-ol_is_decarboxylated_and_c	0.012204	0.012204	#N/A

Surface_deployment_of_P-selectin	0.157208	#N/A	0.157208
Conversion_of_coproporphyrinogen_III_to_protoporphyrinogen_IX	0.585251	0.585251	#N/A
Association_of_BID_with_mitochondrial_membrane	0.0428514	0.0428514	#N/A
Translocation_of_tBID_to_mitochondria	0.0428514	0.0428514	#N/A
Mevalonate_is_phosphorylated_to_mevalonate-5-phosphate	0.279331	0.279331	#N/A
Viral_dsRNA_binds_the_Toll-Like_Receptor_3_(TLR3)	0.124574	0.124574	#N/A
D-ribulose_5-phosphate_<=>_ribose_5-phosphate	0.360078	#N/A	0.360078
ribose_5-phosphate_<=>_D-ribulose_5-phosphate	0.360078	#N/A	0.360078
Phosphorylation_of_Cdc25C_at_Ser_216_by_Chk2(Cds1)	0.222489	0.222489	#N/A
Hydrolysis_of_phosphatidylcholine	0.157929	#N/A	0.157929
Phospho-PLA2_translocates_to_membranes_when_intracellular_calcium_le	0.157929	#N/A	0.157929
Release_of_WD_repeat-containing_protein_1	0.435826	0.435826	#N/A
Surface_deployment_of_PECAM-1	0.772427	0.772427	#N/A
Trans-homophilic_interaction_of_PECAM-1	0.772427	0.772427	#N/A
glyoxylate_+_alanine_=>_glycine_+_pyruvate	0.0367672	0.0367672	#N/A
Pro-GAS6_is_transported_from_the_endoplasmic_reticulum_to_the_Golgi_	0.119264	0.119264	#N/A
Thiamin_is_pyrophosphorylated	0.572051	#N/A	0.572051
Gly-3-P+FAD->DHAP+FADH2_(catalyzed_by_mitochondrial_Gly-Phos_dehydı	0.628526	0.628526	#N/A
Surface_deployment_of_CD63	0.0269769	#N/A	0.0269769
Pyridine_can_be_N-methylated	0.192863	#N/A	0.192863
Surface_deployment_of_CD9	0.0591031	0.0591031	#N/A
Release_of_Superoxide_dismutase_[Cu-Zn]	0.283683	#N/A	0.283683
(24R,_25R)_3alpha,7alpha,12alpha,24-tetrahydroxy-5beta-cholestanoyl-CoA	0.439957	#N/A	0.439957
(24R,_25R)_3alpha,7alpha,24-trihydroxy-5beta-cholestanoyl-CoA_is_oxidize	0.439957	#N/A	0.439957
25(S)_3alpha,7alpha,12alpha-trihydroxy-5beta-cholest-24-enoyl-CoA_is_hyd	0.439957	#N/A	0.439957
25(S)_3alpha,7alpha-dihydroxy-5beta-cholest-24-enoyl-CoA_is_hydrated_to_	0.439957	#N/A	0.439957
3-hydroxyhexacosanoyl-CoA_+_NAD+_=>_3-ketohexacosanoyl-CoA_+_NADH	0.439957	#N/A	0.439957
3-hydroxypristanoyl-CoA_+_NAD+_=>_3-ketoxypristanoyl-CoA_+_NADH_+_I	0.439957	#N/A	0.439957
trans-2,3-dehydrohexacosanoyl-CoA_+_H2O_=>_3-hydroxyhexacosanoyl-Co	0.439957	#N/A	0.439957
trans-2,3-dehydropristanoyl-CoA_+_H2O_=>_3-hydroxypristanoyl-CoA	0.439957	#N/A	0.439957
Guanine_+_PRPP_=>_GMP_+_PPi	0.556007	#N/A	0.556007
Hypoxanthine_+_PRPP_=>_IMP_+_PPi	0.556007	#N/A	0.556007
Mevalonate-5-phosphate_is_further_phosphorylated.	0.00164651	0.00164651	#N/A
thymidine_+_orthophosphate_<=>_thymine_+_2-deoxy-D-ribose_1-phospha	0.12091	0.12091	#N/A
thymine_+_2-deoxy-D-ribose_1-phosphate_<=>_thymidine_+_orthophospha	0.12091	0.12091	#N/A
alpha-D-Glucose_6-phosphate_<=>_D-Glucose_1-phosphate	0.905814	0.905814	#N/A
D-Glucose_1-phosphate_<=>_alpha-D-Glucose_6-phosphate	0.905814	0.905814	#N/A
Acetyl-CoA_+_H2O_+_Oxaloacetate_=>_Citrate_+_CoA	0.0536558	0.0536558	#N/A
Conversion_of_cytosolic_5-methyltetrahydrofolate_(5-methylTHF)_to_5-me	0.256199	0.256199	#N/A
Conversion_of_cytosolic_THF_to_THF-polyglutamate	0.256199	0.256199	#N/A
Conversion_of_mitochondrial_THF_to_THF-polyglutamate	0.256199	0.256199	#N/A
Ligation_of_newly_synthesized_repair_patch_to_incised_DNA_in_GG-NER	0.330999	0.330999	#N/A
Ligation_of_newly_synthesized_repair_patch_to_incised_DNA_in_TC-NER	0.330999	0.330999	#N/A
Exocytosis_of_Clusterin	0.743647	0.743647	#N/A
D-fructose_6-phosphate_+_D-erythrose_4-phosphate__<=>__sedoheptulos	0.716454	0.716454	#N/A
sedoheptulose_7-phosphate_+_D-glyceraldehyde_3-phosphate_<=>_D-eryt	0.716454	0.716454	#N/A
guanidinoacetate_+_S-adenosylmethionine_=>_creatine_+_S-adenosylhomc	0.188922	#N/A	0.188922

Heterdimerization_of_CEACAMs	0.392139	0.392139	#N/A
JAM-A_homodimerises	0.995724	0.995724	#N/A
CYP26A1_breaks_down_all-trans-retinoic_acid_by_4-hydroxylation	0.970894	#N/A	0.970894
3alpha,_7alpha-dihydroxy-5beta-cholestan-26-al_is_oxidized_to_3alpha,_7a	0.221653	0.221653	#N/A
3alpha,7alpha,12alpha,24(S)-tetrahydroxy-5beta-cholestan-27-al_is_oxidizec	0.221653	0.221653	#N/A
3alpha,7alpha,12alpha-trihydroxy-5beta-cholestan-27-al_is_oxidized_to_3al	0.221653	0.221653	#N/A
3alpha,7alpha,24(S)-trihydroxy-5beta-cholestan-27-al_is_oxidized_to_3alpha	0.221653	0.221653	#N/A
5beta-cholestan-3alpha,_7alpha,_12alpha-triol_is_hydroxylated_to_5beta-c	0.221653	0.221653	#N/A
5beta-cholestan-3alpha,_7alpha,_26-triol_is_oxidized_to_3alpha,_7alpha-di	0.221653	0.221653	#N/A
5beta-cholestan-3alpha,_7alpha-diol_is_hydroxylated_to_5beta-cholestan-3	0.221653	0.221653	#N/A
5beta-cholestan-3alpha,7alpha,12alpha,24(S),27-pentol_is_oxidized_to_3alp	0.221653	0.221653	#N/A
5beta-cholestan-3alpha,7alpha,12alpha,24(S)-tetrol_is_hydroxylated_to_5be	0.221653	0.221653	#N/A
5beta-cholestan-3alpha,7alpha,12alpha,27-tetrol_is_oxidized_to_3alpha,7al	0.221653	0.221653	#N/A
5beta-cholestan-3alpha,7alpha,24(S),27-tetrol_is_oxidized_to_3alpha,7alpha	0.221653	0.221653	#N/A
5beta-cholestan-3alpha,7alpha,24(S)-triol_is_hydroxylated_to_5beta-choles	0.221653	0.221653	#N/A
Cholesterol_is_hydroxylated_to_27-hydroxycholesterol_by_CYP27	0.221653	0.221653	#N/A
Release_of_Vinculin	0.681577	0.681577	#N/A
trehalose_+_H2O_=>_2_D-glucose	0.136466	0.136466	#N/A
Exocytosis_of_factor_VIII	0.636095	0.636095	#N/A
Exocytosis_of_fibronectin	0.758297	0.758297	#N/A
Cleavage_of_the_gamma-glutamyl_bond_of_LTC4_forms_LTD4	0.789577	#N/A	0.789577
Binding_of_TFIIIA_To_type_1_Promoter	0.72023	0.72023	#N/A
Phosphatidic_acid_+_H2O_<=>_Diacylglycerol_+__phosphate_(3)	0.884052	0.884052	#N/A
Exocytosis_of_Proactivator_polypeptide	0.230863	#N/A	0.230863
Condensation_of_nicotinamide_to_nicotinamide_D-ribonucleotide_(NMN)	0.64602	#N/A	0.64602
N-acetylglucosaminyl-PI_+_H2O_->_glucosaminyl-PI_+_acetate	0.422162	0.422162	#N/A
ALAD_octamer_associates_with_Pb++,_forming_a_catalytically_inactive_cor	0.855038	#N/A	0.855038
Two_molecules_of_ALA_condense_to_form_porphobilinogen_(PBG)	0.855038	#N/A	0.855038
Exocytosis_of_C1_inhibitor	0.364705	#N/A	0.364705
proline_=>_L-1-pyrroline-5-carboxylate	0.424398	0.424398	#N/A
glutamate_+_NH4+_+_ATP_=>_glutamine_+_ADP_+_orthophosphate	0.537885	0.537885	#N/A
Glutamate_conversion_to_glutamine	0.537885	0.537885	#N/A
LTA4_is_hydrolyzed_to_LTB4	0.633625	0.633625	#N/A
Active_PLCG1_hydrolyses_PIP2	0.252051	0.252051	#N/A
PLC_gamma_2-mediated_hydrolysis	0.252051	0.252051	#N/A
PLC-gamma1_hydrolyses_PIP2	0.252051	0.252051	#N/A
Translocation_of_PLC-gamma1_to_PIP2	0.252051	0.252051	#N/A
Condensation_of_phosphopantothenate_with_cysteine	0.037643	#N/A	0.037643
Exocytosis_of_Protein_S	0.987764	0.987764	#N/A
Pro-protein_S_is_transported_from_the_endoplasmic_reticulum_to_the_Go	0.987764	0.987764	#N/A
Release_of_Calumenin_	0.206829	#N/A	0.206829
JAM-B_homodimerises	0.893312	0.893312	#N/A
Cholesta-5,7,24-trien-3beta-ol_is_reduced_to_desmosterol	0.0869813	0.0869813	#N/A
Release_of_Peptidyl-prolyl_cis-trans_isomerase_A	0.291199	0.291199	#N/A
Release_of_SMAC_from_mitochondria	0.731473	#N/A	0.731473
gamma-glutamylcysteine_combines_with_glycine_to_form_glutathione	0.285424	#N/A	0.285424
4,4-dimethylcholesta-8(9),24-dien-3beta-ol_is_oxidized_to_4-methyl,4-carbo	0.32888	#N/A	0.32888

4-methylcholesta-8(9),24-dien-3beta-ol_is_oxidized_to_4-carboxycholesta-8	0.32888	#N/A	0.32888
(S)-Malate_+_NAD+_<=>_Oxaloacetate_+_NADH_+_H+	0.608273	0.608273	#N/A
Oxaloacetate_+_NADH_+_H+_<=>_(S)-Malate_+_NAD+	0.608273	0.608273	#N/A
Oxidative_demethylation_of_1-EtA_damaged_DNA_By_ABH2	0.119926	#N/A	0.119926
Oxidative_demethylation_of_1-MeA_damaged_DNA_By_ABH2	0.119926	#N/A	0.119926
Squalene_is_oxidized_to_its_epoxide	0.212702	0.212702	#N/A
Prostacyclin_synthase_(CYP8A1)_mediates_the_isomerization_of_prostaglan	0.580697	#N/A	0.580697
Extracellular_5-methyltetrahydrofolate_import_across_the_plasma_membra	0.141706	0.141706	#N/A
TRAIL_Binds_TRAIL-Receptor2	0.422824	0.422824	#N/A
Trimerization_of_TRAIL:_TRAIL_receptor-2_complex	0.422824	0.422824	#N/A
glycine_+_tRNA(Gly)_+_ATP_=>_Gly-tRNA(Gly)_+_AMP_+_pyrophosphate	0.792978	0.792978	#N/A
Oxidation_of_protoporphyrinogen_IX_to_protoporphyrin_IX	0.361285	#N/A	0.361285
N-formylkynurenine_+_H2O_=>_kynurenine_+_formate_[mouse]	0.427797	0.427797	#N/A
Exocytosis_of_hepatocyte_growth_factor	0.367719	0.367719	#N/A
alpha-D-glucose_6-phosphate_[cytosol]_=>_alpha-D-glucose_6-phosphate_[	0.71779	#N/A	0.71779
Oligomerization__of_BAK_at_the_mitochondrial_membrane	0.272904	#N/A	0.272904
2-deoxyadenosine_5-monophosphate_(dAMP)_+_H2O_=>_deoxyadenosine_	0.11098	0.11098	#N/A
D-glyceraldehyde_3-phosphate_<=>_dihydroxyacetone_phosphate	0.438416	0.438416	#N/A
dihydroxyacetone_phosphate_<=>_D-glyceraldehyde_3-phosphate	0.438416	0.438416	#N/A
Exocytosis_of_vWF	0.17776	0.17776	#N/A
Inhibition_of_GRK2_by_calmodulin	0.621812	0.621812	#N/A
Hydrolysis_of_PIP3_to_PIP2	0.476984	0.476984	#N/A
PTEN_dephosphorylates_PIP3	0.476984	0.476984	#N/A
Engulfed_CpG_DNA_binds_to_endosomal_TLR9	0.254155	0.254155	#N/A
alpha-D-glucose_6-phosphate_<=>_D-fructose_6-phosphate	0.940036	0.940036	#N/A
D-fructose_6-phosphate_<=>_alpha-D-Glucose_6-phosphate	0.940036	0.940036	#N/A
Association_of_RAD52_with_ssDNA_at_resected_ends_of_double-strand_b	0.990704	#N/A	0.990704
Phosphatidic_acid_+_H2O_<=>_Diacylglycerol_+__phosphate_(2)	0.771815	#N/A	0.771815
Binding_of_Pol_eta_to_lesioned_DNA_template	0.815176	0.815176	#N/A
Elongation_by_Pol_eta_	0.815176	0.815176	#N/A
Insertion_of_correct_bases_opposite_to_the_lesion_by_Pol_eta	0.815176	0.815176	#N/A
thymidine_+_ATP_=>_thymidine_5-monophosphate_(dTMP)_+_ADP	0.765099	#N/A	0.765099
Mevalonate-5-pyrophosphate_is_decarboxylated	0.022148	0.022148	#N/A
Phosphopantothenoylcysteine_is_decarboxylated	0.818487	#N/A	0.818487
Relocalization_of_nuclearly_localized_Cyclin_D1_to_the_cytoplasm	0.140782	0.140782	#N/A
SLC36A1-mediated_uptake_of_glycine,_proline,_and_alanine	0.574848	0.574848	#N/A
SLC36A2-mediated_uptake_of_glycine_and_proline	0.574848	0.574848	#N/A
2-deoxycytidine_+_H2O_=>_2-deoxyuridine_+_NH4+	0.69999	#N/A	0.69999
cytidine_+_H2O_=>_uridine_+_NH4+	0.69999	#N/A	0.69999
TFAM_binds_to_mitochondrial_promoters	0.369356	0.369356	#N/A
Lanosterol_is_oxidatively_demethylated_to_4,4-dimethylcholesta-8(9),14,24	0.298403	0.298403	#N/A
(S)-3-Hydroxydodecanoyl-CoA+NAD<=>3-Oxododecanoyl-CoA+NADH+H	0.747744	#N/A	0.747744
(S)-Hydroxybutanoyl-CoA+NAD<=>Acetoacetyl-CoA+NADH+H	0.747744	#N/A	0.747744
(S)-Hydroxydecanoyl-CoA+NAD<=>3-Oxodecanoyl-CoA+NADH+H	0.747744	#N/A	0.747744
(S)-Hydroxyhexanoyl-CoA+NAD<=>3-Oxohexanoyl-CoA+NADH+H	0.747744	#N/A	0.747744
(S)-Hydroxyoctanoyl-CoA+NAD<=>3-Oxooctanoyl-CoA+NADH+H	0.747744	#N/A	0.747744
Translocation_of_p21_to_the_nucleus	0.254326	#N/A	0.254326

Release_of_Secretogranin-3	0.742563	0.742563	#N/A
Autophosphorylation_of_DNA-PKcs	0.836524	0.836524	#N/A
Release_of_78_kDa_glucose-regulated_protein	0.576457	#N/A	0.576457
Binding_of_ADAR1_homodimer_to_dsRNA_duplex	0.737981	0.737981	#N/A
Deamination_at_C6_position_of_adenosine_in_Editosome_(ADAR1)	0.737981	0.737981	#N/A
Formation_of_ADAR1_homodimer	0.737981	0.737981	#N/A
Adenosine_3,5-bisphosphate_(PAP)_+_H2O_=>_AMP_+_orthophosphate	0.519886	#N/A	0.519886
Phosphatidic_acid_+_H2O_<=>_Diacylglycerol_+__phosphate_(1)	0.549829	#N/A	0.549829
Isomerization_of_3-trans-decenoyl-CoA_to_form_trans-dec-2-enoyl-CoA	0.131396	#N/A	0.131396
Isomerization_of_cis,cis-3,6-Dodecadienoyl-CoA_to_form_trans,cis-Lauro-2,(	0.131396	#N/A	0.131396
palmitoyl-CoA+FAD<=>trans-Hexadec-2-enoyl-CoA+FADH2	0.724124	0.724124	#N/A
2-trans-Dodecenoyl-CoA+H2O<=>(S)-3-Hydroxydodecanoyl-CoA	0.047599	0.047599	#N/A
Crotonoyl-CoA+H2O<=>(S)-3-Hydroxybutanoyl-CoA	0.047599	0.047599	#N/A
trans-Dec-2-enoyl-CoA+H2O<=>(S)-Hydroxydecanoyl-CoA	0.047599	0.047599	#N/A
trans-Hex-2-enoyl-CoA+H2O<=>(S)-Hydroxyhexanoyl-CoA	0.047599	0.047599	#N/A
trans-Oct-2-enoyl-CoA+H2O<=>(S)-Hydroxyoctanoyl-CoA	0.047599	0.047599	#N/A
2-deoxycytidine_5-monophosphate_(dCMP)_+_H2O_=>_2-deoxyuridine_5-r	0.671996	0.671996	#N/A
mannose(a1-4)glucosaminyl-acyl-PI_+_phosphatidylethanolamine_->_(ethar	0.479834	0.479834	#N/A
D-methylmalonyl-CoA_<=>_L-methylmalonyl-CoA	0.559331	#N/A	0.559331
lysophosphatidic_acid_+_fatty_acyl_CoA__=>_phosphatidic_acid_+_CoA_(5)	0.0669198	#N/A	0.0669198
lauroyl-CoA+FAD<=>2-trans-Dodecenoyl-CoA+FADH2	0.0157291	0.0157291	#N/A
myristoyl-CoA+FAD<=>trans-Tetradec-2-enoyl-CoA+FADH2	0.0157291	0.0157291	#N/A
UDP-glucuronate_transport_from_the_cytosol_to_ER_lumen	0.436833	#N/A	0.436833
acetyl-CoA_+_H2O_=>_acetate_+_CoASH	0.061388	#N/A	0.061388
Hydrolysis_of_choloyl-CoA_to_cholate_and_CoASH	0.061388	#N/A	0.061388
Cleavage_of_cytosine_glycol_by_hNTH1_glycosylase	0.459492	#N/A	0.459492
Cleavage_of_dihydrouracil_by_hNTH1_glycosylase	0.459492	#N/A	0.459492
Cleavage_of_formamidopyrimidine__by_hNTH1_glycosylase_	0.459492	#N/A	0.459492
Cleavage_of_thymine_glycol__by_hNTH1_glycosylase__	0.459492	#N/A	0.459492
hNTH1_glycosylase_mediated_recognition_and_binding_of_cytosine_glycol	0.459492	#N/A	0.459492
hNTH1_glycosylase_mediated_recognition_and_binding_of_dihydrouracil_	0.459492	#N/A	0.459492
hNTH1_glycosylase_mediated_recognition_and_binding_of_formamidopyrir	0.459492	#N/A	0.459492
hNTH1_glycosylase_mediated_recognition_and_binding_of_thymine_glycol	0.459492	#N/A	0.459492
orotate_+_5-phospho-alpha-D-ribose_1-diphosphate_(PRPP)_<=>_orotidine_	0.527212	#N/A	0.527212
orotidine_5-monophosphate_=>_uridine_5-monophosphate_+_CO2	0.527212	#N/A	0.527212
Dephosphorylation_of_pChREBP_(Ser_196)_by_PP2A	0.626217	#N/A	0.626217
Dephosphorylation_of_pChREBP_(Ser_568)_by_PP2A	0.626217	#N/A	0.626217
Dephosphorylation_of_pChREBP_(Thr_666)_by_PP2A	0.626217	#N/A	0.626217
Nuclear_transport_of_pChREBP_(Thr_666)_protein	0.626217	#N/A	0.626217
PhosphoChREBP_(Thr_666)__is_exported_to_cytosol	0.626217	#N/A	0.626217
2-Deoxyadenosine_+_H2O_=>_2-Deoxyinosine_+_NH3	0.183945	0.183945	#N/A
Adenosine_+_H2O_=>_Inosine_+_NH3	0.183945	0.183945	#N/A
PDK1_attachment_to_plasma_membrane	0.396687	0.396687	#N/A
Translocation_of_PDK1_to_Plasma_membrane	0.396687	0.396687	#N/A
L-methylmalonyl-CoA_<=>_succinyl-CoA	0.497943	0.497943	#N/A
Oxidative_demethylation_of_1-EtA_damaged_DNA_By_ABH3	0.755838	0.755838	#N/A
Oxidative_demethylation_of_1-MeA_damaged_DNA_By_ABH3	0.755838	0.755838	#N/A

Oxidative_demethylation_of_3-MeC_damaged_DNA_By_ABH3	0.755838	0.755838	#N/A
Dab2_interacts_with_clathrin	0.3737	0.3737	#N/A
Dab2_is_recruited_to_the_junctional_plaques	0.3737	0.3737	#N/A
Dynamin_is_recruited_to_the_gap_junction_plaque	0.3737	0.3737	#N/A
Internalization_of_gap_junction_plaques	0.3737	0.3737	#N/A
Part_of_the_ICD_migrates_to_the_nucleus	0.256225	#N/A	0.256225
The_p75NTR_C-terminal_fragment_enters_endosomes	0.256225	#N/A	0.256225
L-1-pyrroline-5-carboxylate_+_NADPH_+_H+_=>_proline_+_NADP+	0.723175	0.723175	#N/A
Translocation_of_p27_to_the_nucleoplasm	0.224372	0.224372	#N/A
Dimerization_of_Phospho-IRF3	0.60578	#N/A	0.60578
Dimerized_Phospho-IRF3_is_Transported_To_The_Nucleus	0.60578	#N/A	0.60578
Exocytosis_of_SRGN	0.709611	#N/A	0.709611
JAM-C_homodimerises	0.196228	#N/A	0.196228
Oligomerization_of_BAX_at_the_mitochondrial_membrane	0.626322	#N/A	0.626322
Translocation_of_activated_BAX_to_the_mitochondria	0.626322	#N/A	0.626322
Biotin_transport_across_the_plasma_membrane	0.0565694	#N/A	0.0565694
Pantothenate_transport_across_the_plasma_membrane	0.0565694	#N/A	0.0565694
Dimerisation_of_CREB	0.970137	0.970137	#N/A
Digestion_of_1-6_linkages_of_limit_dextrins_to_yield_maltose,_maltotriose	0.529719	0.529719	#N/A
maltose_+_H2O_=>_2_D-glucose_(maltase-glucoamylase)	0.529719	0.529719	#N/A
maltose_+_H2O_=>_2_D-glucose_(sucrase-isomaltase)	0.529719	0.529719	#N/A
maltotriose_+_H2O_=>_maltose_+_D-glucose_(maltase-glucoamylase)	0.529719	0.529719	#N/A
maltotriose_+_H2O_=>_maltose_+_D-glucose_(sucrase-isomaltase)	0.529719	0.529719	#N/A
sucrose_+_H2O_=>_glucose_+_fructose	0.529719	0.529719	#N/A
beta-methylglutaconyl-CoA_+_H2O_<=>_beta-hydroxy-beta-methylglutaryl-	0.859028	#N/A	0.859028
5-phosphoribosyl-5-aminoimidazole-4-carboxamide_(AICAR)_+_10-formylte	0.676769	0.676769	#N/A
5-phosphoribosyl-5-formaminoimidazole-4-carboxamide_(FAICAR)_<=>_inos	0.676769	0.676769	#N/A
Dissociation_of_phosphoribosyl_pyrophosphate_amidotransferase_tetrame	0.476437	#N/A	0.476437
Formation_of_phosphoribosyl_pyrophosphate_amidotransferase_tetramer	0.476437	#N/A	0.476437
5-phospho-alpha-D-ribose_1-diphosphate_(PRPP)_+_H2O_+_L-glutamine_<	0.476437	#N/A	0.476437
GTP_loading_by_Rheb	0.757128	0.757128	#N/A
Cholesterol_is_hydroxylated_to_24-hydroxycholesterol_by_CYP46A1	0.236472	#N/A	0.236472
Pro-beta-NGF_and_mature_beta-NGF_are_secreted	0.185404	#N/A	0.185404
pro-beta-NGF_homodimer_transits_to_the_golgi_apparatus	0.185404	#N/A	0.185404
Dissociation_of_dimeric_phospho-ATM_complexes	0.562496	0.562496	#N/A
Intermolecular_autophosphorylation_of_ATM_within_dimeric_ATM_comple	0.562496	0.562496	#N/A
Amidation_of_deamino-NAD+_to_NAD+	0.119792	0.119792	#N/A
Mitochondrial_recruitment_of_Drp1	0.558135	0.558135	#N/A
5,10-methyleneTHF_polyglutamate_+_NADPH_+_H+_=>_5-methylTHF_poly	0.0513994	0.0513994	#N/A
Release_of_Profilin-1	0.176417	#N/A	0.176417
(2S)-pristanoyl-CoA_+_O2_=>_trans-2,3-dehydropristanoyl-CoA_+_H2O2_(A	0.236555	0.236555	#N/A
Ferrous_iron_is_inserted_into_protoporphyrin_IX_to_form_heme	0.0836906	0.0836906	#N/A
Reduction_of_HMG-CoA_produces_mevalonate	0.0838372	0.0838372	#N/A
Cleavage_of__thymine_by_MBD4_glycosylase	0.0940472	#N/A	0.0940472
Cleavage_of_uracil_by_MBD4_glycosylase	0.0940472	#N/A	0.0940472
MBD4_glycosylase_mediated_recognition_and_binding_of_a_thymine_opp	0.0940472	#N/A	0.0940472
MBD4_glycosylase_mediated_recognition_and_binding_of_an_uracil_oppo	0.0940472	#N/A	0.0940472

Uroporphyrinogen_I_is_decarboxylated_to_form_coproporphyrinogen_I	0.864571	0.864571	#N/A
Uroporphyrinogen_III_is_decarboxylated_to_form_coproporphyrinogen_III	0.864571	0.864571	#N/A
glutamate_+_L-glutamate_gamma-semialdehyde_<=>_ornithine_+_alpha-ke	0.541581	0.541581	#N/A
ornithine_+_alpha-ketoglutarate_<=>_glutamate_+_L-glutamate_gamma-se	0.541581	0.541581	#N/A
24-hydroxycholesterol_is_7alpha-hydroxylated_to_yield_cholest-5-ene-3bet	0.00348805	0.00348805	#N/A
UBF-1_Binds_rDNA_Promoter	0.871756	0.871756	#N/A
Four_PBGs_combine_through_deamination_to_form_hydroxymethylbilane_	0.0400177	0.0400177	#N/A
2-deoxyguanosine_5-diphosphate_(dGDP)_+_ADP_<=>_2-deoxyguanosine_5	0.15422	0.15422	#N/A
2-deoxyguanosine_5-monophosphate_(dGMP)_+_ATP_<=>_2-deoxyguanosi	0.15422	0.15422	#N/A
guanosine_5-diphosphate_(GDP)_+_ADP_<=>_guanosine_5-monophosphate	0.15422	0.15422	#N/A
guanosine_5-monophosphate_(GMP)_+_ATP_<=>_guanosine_5-diphosphate	0.15422	0.15422	#N/A
beta-hydroxyisobutyryl-CoA_+_H2O_=>_beta-hydroxyisobutyrate_+_CoA	0.209992	0.209992	#N/A
Basigin_homodimerises	0.364146	0.364146	#N/A
2-deoxyuridine_5-monophosphate_(dUMP)_+_N5,N10-methylene_tetrahyd	0.760113	#N/A	0.760113
MCM8_mediated_fork_unwinding	0.1309	0.1309	#N/A
Exocytosis_of_Thymosin_beta-4	0.153372	0.153372	#N/A
Reduction_of_presqualene_diphosphate_to_form_squalene	0.562421	0.562421	#N/A
Two_FPP_molecules_dimerize_to_form_presqualene_diphosphate	0.562421	0.562421	#N/A
2-deoxycytidine_5-diphosphate_(dCDP)_+_ADP_<=>_2-deoxycytidine_5-mo	0.692113	0.692113	#N/A
2-deoxycytidine_5-monophosphate_(dCMP)_+_ATP_<=>_2-deoxycytidine_5	0.692113	0.692113	#N/A
11-deoxycorticosterone_is_oxidised_to_corticosterone_by_CYP11B2	0.952617	#N/A	0.952617
11-deoxycortisol_is_oxidised_to_cortisol_by_CYP11B1	0.952617	#N/A	0.952617
Conversion_of_18-hydroxycorticosterone_to_aldosterone	0.952617	#N/A	0.952617
Hydroxylation_of_corticosterone_to_form_18-hydroxycorticosterone	0.952617	#N/A	0.952617
4,4-dimethylcholesta-8(9),14,24-trien-3beta-ol_is_reduced_to_4,4-dimethyl	0.369316	0.369316	#N/A
4,4-dimethylcholesta-8(9),14,24-trien-3beta-ol_is_reduced_to_4,4-dimethyl	0.369316	0.369316	#N/A
5-phosphoribosylformylglycinamidine_(FGAM)_+_ATP_=>_5-phosphoribosyl	0.760379	#N/A	0.760379
5-phosphoribosylamine_+_glycine_+_ATP_<=>_5-phosphoribosylglycinamide	0.760379	#N/A	0.760379
5-phosphoribosylglycinamide_(GAR)_+_10-formyl-tetrahydrofolate_=>_5-ph	0.760379	#N/A	0.760379
2-deoxyuridine_5-diphosphate_(dUDP)_+_ADP_<=>_2deoxyuridine_5-mono	0.640019	0.640019	#N/A
2-deoxyuridine_5-monophosphate_(dUMP)_+_ATP_<=>_2-deoxyuridine_5-c	0.640019	0.640019	#N/A
thymidine_5-diphosphate_(TDP)_+_ADP_<=>_thymidine_5-monophosphate	0.640019	0.640019	#N/A
thymidine_5-monophosphate_(TMP)_+_ATP_<=>_thymidine_5-diphosphate	0.640019	0.640019	#N/A
SLC38A2_(ATA2)-mediated_uptake_of_neutral_amino_acids	0.942175	0.942175	#N/A
Dephosphorylation_of_phospho-Cdh1	0.639441	#N/A	0.639441
Receptor_CCR9__binds_CCL25_ligand	0.358941	#N/A	0.358941
Association_of_mTERF_with_the_termination_sequence	0.726209	#N/A	0.726209
M2_and_M4_receptors_bind_acetylcholine	0.555338	0.555338	#N/A
glutamine_+_tRNA(Glu)_+_ATP_=>_Glu-tRNA(Glu)_+_AMP_+_pyrophosphat	0.969584	0.969584	#N/A
SLC43A1_(LAT3)-mediated_uptake_of_large_neutral_amino_acids	0.558572	#N/A	0.558572
tyrosine_+_tRNA(Tyr)_+_ATP_=>Tyr-tRNA(Tyr)_+_AMP_+_pyrophosphate	0.633546	#N/A	0.633546
Conversion_of_HMB_to_uroporphyrinogen_III	0.481188	0.481188	#N/A
Conversion_of_Dihydroxyacetone_Phosphate_to_Glycerol_-3-_phosphate	0.42397	#N/A	0.42397
Nicotinate_D-ribonucleotide_+_ATP_=>_deamino-NAD+_+_pyrophosphate_	0.686667	#N/A	0.686667
Translocation_of_BIM_to_mitochondria	0.814474	0.814474	#N/A
Alignment_Of_The_RNA_Template_On_The_Telomeric_Chromosome_End	0.609009	0.609009	#N/A
Disassociation_of_Telomerase_RNP_and_the_Chromosome_End	0.609009	0.609009	#N/A

Elongation_of_Extended_Telomeric_Chromosome_End	0.609009	0.609009	#N/A
Elongation_Of_The_Telomeric_Chromosome_End	0.609009	0.609009	#N/A
Recruitment_of_Telomerase_RNP_to_the_Telomeric_Chromosome_End	0.609009	0.609009	#N/A
Translocation_Of_Telomerase_RNP_And_Alignment_Of_RNA_Template_(TE	0.609009	0.609009	#N/A
APE1_mediated_endonucleolytic_cleavage_at_the_5_side_of_the_base-free	0.443105	#N/A	0.443105
Displacement_of_UNG2_glycosylase_by__APE1_at_the_AP_site	0.443105	#N/A	0.443105
MGMT/hAGT_mediated_DNA_Damage_Reversal	0.796486	#N/A	0.796486
Translocation_of_PUMA_protein_to_mitochondria	0.289196	#N/A	0.289196
Homodimerization__of_DFF40	0.0146032	0.0146032	#N/A
methylmalonate_semialdehyde_+_NAD+_+_CoA_=>_propionyl-CoA_+_CO2_	0.742151	#N/A	0.742151
Phosphorylated_Foxo1_is_excluded_from_the_nucleus	0.227142	#N/A	0.227142
Phosphorylated_FOXO1A_is_excluded_from_the_nucleus	0.227142	#N/A	0.227142
Adrostenedione_is_converted_to_estrone_by_Aromatase_(CYP19A1)	0.518492	#N/A	0.518492
Testosterone_is_converted_to_estradiol	0.518492	#N/A	0.518492
cysteine_+_tRNA(Cys)_+_ATP_=>_Cys-tRNA(Cys)_+_AMP_+_pyrophosphate	0.484153	#N/A	0.484153
DARPP-32_is_dephosphorylated_on_Thr75_by_PP2A	0.75663	#N/A	0.75663
Further_hydroxylation_of_calcidiol_in_kidney_to_form_calcitriol	0.60706	#N/A	0.60706
Oxytocin_receptor_bind_oxytocin	0.992016	0.992016	#N/A
1,25-dihydroxyvitamin_D3_is_deactivated	0.783384	#N/A	0.783384
CYP24A1_catalyzes_tjhe_initial_step_in_the_deactivation_of_the_hormonal	0.783384	#N/A	0.783384
D-glucono-1,5-lactone_6-phosphate_+_H2O_=>_6-phospho-D-gluconate	0.109909	0.109909	#N/A
Connexin_62_mediated_neuronal_gap_junction_communication	0.0617622	#N/A	0.0617622
valine_+_tRNA(Val)_+_ATP_=>_Val-tRNA(Val)_+_AMP_+_pyrophosphate	0.347138	0.347138	#N/A
Receptor_CX3C1_binds_fractalkine	0.150128	#N/A	0.150128
CoA_transport_across_the_inner_mitochondrial_membrane	0.314298	#N/A	0.314298
N-acetylation_of_serotonin	0.711439	#N/A	0.711439
Iodide_is_taken_up_by_thyroid_epithelial_cells	0.201187	#N/A	0.201187
CYP26B1_also_deactivates_all-trans-retinoic_acid_by_4-hydroxylation	0.414655	0.414655	#N/A
CYP26C1_deactivates_9-cis-retinoic_acid_by_4-hydroxylation	0.414655	0.414655	#N/A
Protaglandin_E_synthase_isomerizes_PGH2_to_PGE2	0.653344	#N/A	0.653344
4-methylcholesta-8(9),24-dien-3-one_is_reduced_to_4-methylcholesta-8(9),	0.560219	0.560219	#N/A
Zymosterone_(cholesta-8(9),24-dien-3-one)_is_reduced_to_zymosterol_(cho	0.560219	0.560219	#N/A
GPCR_neuropeptide_receptor_binds_neuropeptides_B_and_W	0.614678	0.614678	#N/A
H2_receptor_binds_histamine	0.293829	#N/A	0.293829
GTP-binding_activates_eEF2	0.63753	0.63753	#N/A
LTA4_conjugates_with_glutathione_to_form_LTC4	0.681148	0.681148	#N/A
Translocation_of_activated_BAD_protein_to_mitochondria	0.777134	0.777134	#N/A
beta-hydroxyisobutyrate_+_NAD+_<=>_methylmalonyl_semialdehyde_+_NA	0.598602	0.598602	#N/A
hydrolysis_of__2-deoxyuridine_5-triphosphate_to_form__2-deoxyuridine_5	0.658236	0.658236	#N/A
Platelet-derived_TREM-1_ligand_binds_to_TREM-1	0.663162	0.663162	#N/A
H1_receptor_binds_histamine	0.449302	0.449302	#N/A
Noradrenaline_is_converted_to_adrenaline	0.541803	#N/A	0.541803
Cholesterol_is_released_into_the_inner_mitochondrial_membrane	0.62	#N/A	0.62
Phosphorylation_of_Cdc25C_at_Ser_216_by_Chk1	0.230141	0.230141	#N/A
TTF-I_binds_to_the_Sal_Box	0.758634	0.758634	#N/A
Association_of_RAD51_with_the_resected_ends_of_the_DNA_double-stran	0.472819	#N/A	0.472819
Dopamine_is_oxidised_to_noradrenaline	0.527922	0.527922	#N/A

Synthesis_of_noradrenaline	0.527922	0.527922	#N/A
Translocation_of_BMF_to_mitochondria	0.45322	#N/A	0.45322
Cholesterol_is_hydroxylated_to_25-hydroxycholesterol	0.823429	#N/A	0.823429
G_protein-coupled_estrogen_receptor_1_binds_estrogen_non-classically	0.918924	0.918924	#N/A
Digestion_of_cholesterol_esters_by_extracellular_CEL_(bile_salt-dependent	0.469205	#N/A	0.469205
Digestion_of_monoacylglycerols_by_extracellular_CEL_(bile_salt-dependent	0.469205	#N/A	0.469205
Digestion_of_triacylglycerols_by_extracellular_CEL_(bile_salt-dependent_lip	0.469205	#N/A	0.469205
Production_of_ceramide_which_can_activate_JNK_and_other_targets	0.649045	0.649045	#N/A
tyrosine_+_tRNA(Tyr)_+_ATP_=>_Tyr-tRNA(Tyr)_+_AMP_+_pyrophosphate	0.355307	#N/A	0.355307
SLC6A20-mediated_uptake_of_proline	0.304344	#N/A	0.304344
FMN_is_futher_phosphorylated_to_FAD	0.966399	0.966399	#N/A
Entry_of_Ca++_from_plasma	0.658462	0.658462	#N/A
Decanoyl-CoA+FAD<=>trans-Dec-2-enoyl-CoA+FADH2	0.915421	0.915421	#N/A
dehydrogenation_of_4-cis-decenoyl-CoA_to_form_2-trans-4-cis-decadienoy	0.915421	0.915421	#N/A
Octanoyl-CoA+FAD<=>trans-Oct-2-enoyl-CoA+FADH2	0.915421	0.915421	#N/A
Reduction_of_dehydroascorbate_to_ascorbate	0.901008	0.901008	#N/A
21-hydroxylation_of_progesterone_to_form_11-deoxycorticosterone	0.196522	#N/A	0.196522
Hydroxylation_of_17-hydroxyprogesterone_to_form_11-deoxycortisol	0.196522	#N/A	0.196522
Exocytosis_of_beta-thromboglobulin	0.363286	0.363286	#N/A
Exocytosis_of_neutrophil-activating_peptide_2	0.363286	0.363286	#N/A
Cleavage_8-oxo_guanine__by_MYH_glycosylase	0.995601	0.995601	#N/A
MYH_glycosylase_mediated_recognition_and_binding_of_an_adenine_oppo	0.995601	0.995601	#N/A
(ethanolamineP)_mannose_(a1-2)_mannose_(a1-6)_(ethanolamineP)_mann	0.0939466	#N/A	0.0939466
mannose_(a1-6)_(ethanolamineP)_mannose_(a1-4)_glucosaminyl-acyl-PI_+_	0.271385	#N/A	0.271385
27-hydroxycholesterol_+_PAPS_=>_27-hydroxycholesterol_3-sulfate_+_PAP	0.433285	#N/A	0.433285
cholesterol_+_PAPS_=>_cholesterol_sulfate_+_PAP	0.433285	#N/A	0.433285
Cleavage_of__thymine_by_TDG_glycosylase	0.157221	#N/A	0.157221
Cleavage_of_ethenocytosine_by_TDG_glycosylase_	0.157221	#N/A	0.157221
Cleavage_of_uracil_by_TDG_glycosylase	0.157221	#N/A	0.157221
TDG_glycosylase_mediated_recognition_and_binding_of_an_ethenocytosin	0.157221	#N/A	0.157221
TDG_glycosylase_mediated_recognition_and_binding_of_an_thymine_oppo	0.157221	#N/A	0.157221
TDG_glycosylase_mediated_recognition_and_binding_of_an_uracil_opposit	0.157221	#N/A	0.157221
Connexin_36_mediated_neuronal_gap_junction_communication	0.879742	0.879742	#N/A
alpha-D-Glucose_6-phosphate_+_H2O_=>_alpha-D-Glucose_+_Orthophosph	0.417932	0.417932	#N/A
lithocholate_+_PAPS_=>_lithocholate_sulfate_+_PAP	0.538031	#N/A	0.538031
taurolithocholate_+_PAPS_=>_taurolithocholate_sulfate_+_PAP	0.538031	#N/A	0.538031
20alpha,22beta-hydroxycholesterol_is_cleaved_by_CYP11A1_to_yield_pregr	0.206306	0.206306	#N/A
Oxidation_of_22beta-hydroxycholesterol_to_20alpha,22beta-hydroxycholes	0.206306	0.206306	#N/A
Oxidation_of_cholesterol_to_22beta-hydroxycholesterol	0.206306	0.206306	#N/A
Cleavage_of_uracil_by_hSMUG1_glycosylase	0.0365246	#N/A	0.0365246
hSMUG1_glycosylase_mediated_recognition_and_binding_of_uracil_within_	0.0365246	#N/A	0.0365246
Thiamin_transport_across_the_plasma_membrane	0.211667	#N/A	0.211667
malate_+_NAD+_<=>_oxaloacetate_+_NADH_+_H+	0.226288	0.226288	#N/A
glucosaminyl-PI_+_fatty_acyl-CoA_->_glucosaminyl-acyl-PI_+_CoA-SH	0.349796	#N/A	0.349796
Cleavage_of_8-oxo_guanine_by_hOGG1_glycosylase	0.484426	0.484426	#N/A
Cleavage_of_formamidopyrimidine_by_hOGG1_glycosylase	0.484426	0.484426	#N/A
hOGG1_glycosylase_mediated_recognition_and_binding_of_a_formamidopy	0.484426	0.484426	#N/A

hOGG1_glycosylase_mediated_recognition_and_binding_of_an_8-oxo_guan	0.484426	0.484426	#N/A
Conversion_of_glycerol-3-phosphate_to_lysophosphatidic_acid	0.0443969	0.0443969	#N/A
Adenine_+_PRPP_=>_AMP_+_PPi	0.422419	0.422419	#N/A
Synthesis_of_o-acetylcholine	0.0514977	0.0514977	#N/A
CPT2_converts_palmitoyl_carnitine_to_palmitoyl-CoA	0.220262	#N/A	0.220262
arginine_+_glycine_=>_ornithine_+_guanidoacetate	0.228109	0.228109	#N/A
Cleavage_of_3-methyladenine_by_MPG_glycosylase_	0.430539	#N/A	0.430539
Cleavage_of_ethenoadenine_by_MPG_glycosylase	0.430539	#N/A	0.430539
Cleavage_of_hypoxanthine_by_MPG_glycosylase	0.430539	#N/A	0.430539
MPG_glycosylase_mediated_recognition_and_binding_of_3-methyladenine_	0.430539	#N/A	0.430539
MPG_glycosylase_mediated_recognition_and_binding_of_ethenoadenine_	0.430539	#N/A	0.430539
MPG_glycosylase_mediated_recognition_and_binding_of_hypoxanthine	0.430539	#N/A	0.430539
Phosphorylated_Cdc6_is_exported_from_the_nucleus	0.570497	#N/A	0.570497
Cytosolic_tetrahydrofolate_import_across_the_inner_mitochondrial_memb	0.40765	#N/A	0.40765
Mitochondrial_tetrahydrofolate_export_across_the_inner_mitochondrial_m	0.40765	#N/A	0.40765
SLC16A10-mediated_uptake_of_aromatic_amino_acids	0.486555	#N/A	0.486555
HCO3-_transport_through_ion_channel	0.556023	0.556023	#N/A
tryptophan_+_O2_=>_N-formylkynurenine_[IDO]	0.576339	0.576339	#N/A
5-phosphoribosyl-5-aminoimidazole_(AIR)_+_CO2_<=>_5-phosphoribosyl-5-	0.106486	0.106486	#N/A
5-phosphoribosyl-5-aminoimidazole-4-carboxylate_(CAIR)_+_L-aspartate_+_	0.106486	0.106486	#N/A
isobutyryl-CoA_+_FAD_=>_methacrylyl-CoA_+_FADH2	0.331516	0.331516	#N/A
(ethanolamineP)_mannose_(a1-4)_glucosaminyl-acyl-PI_+_dolichol_phospha	0.746462	0.746462	#N/A
xanthosine_5-monophosphate_(XMP)_+_L-glutamine_+_ATP_+_H2O_=>_gu	0.548292	#N/A	0.548292
isovaleryl-CoA_+_FAD_=>_beta-methylcrotonyl-CoA_+_FADH2	0.162549	0.162549	#N/A
(S)-dihydroorotate_+_ubiquinone_=>_orotate_+_ubiquinol	0.0299266	#N/A	0.0299266

Muscle

Gene ontology(GO)

		Reporter P-value	
**#Feature**	**No-direction**	**Down regulated**	**Up regulated**
CC_GO:0016020_membrane	0.987993	0.0970771	0.999988
CC_GO:0016021_integral+to+membrane	0.741861	0.995926	0.0752493
CC_GO:0005634_nucleus	0.178335	3.53952E-06	0.999793
MF_GO:0005515_protein+binding	0.992254	0.00022393	1
CC_GO:0005737_cytoplasm	0.539865	2.47775E-08	1
MF_GO:0004872_receptor+activity	0.254799	1	4.79791E-08
MF_GO:0046872_metal+ion+binding	0.780252	0.0280655	0.998639
CC_GO:0005886_plasma+membrane	0.998698	0.994339	0.959492
MF_GO:0008270_zinc+ion+binding	0.879298	0.0934126	0.99837
BP_GO:0006355_regulation+of+transcription,+DNA-dependent	0.957028	0.0153314	0.999995
BP_GO:0007186_G-protein+coupled+receptor+protein+signaling+pathway	0.0660732	1	1.23205E-09
MF_GO:0003677_DNA+binding	0.334657	0.000187611	0.997696
MF_GO:0000166_nucleotide+binding	0.967153	0.0669997	0.99997
BP_GO:0006810_transport	0.998627	0.0342896	1

BP_GO:0006350_transcription	0.678444	0.00443485	0.999593
BP_GO:0008150_biological_process	0.10499	0.0118539	0.710467
CC_GO:0005576_extracellular+region	0.99995	0.998304	0.998529
CC_GO:0005575_cellular_component	0.0492073	0.00975371	0.526139
MF_GO:0003674_molecular_function	0.217423	0.0180174	0.853407
MF_GO:0016787_hydrolase+activity	0.933598	0.0465851	0.999951
MF_GO:0016740_transferase+activity	0.612521	0.0125749	0.99768
CC_GO:0005622_intracellular	0.597837	0.000222361	0.999973
MF_GO:0005524_ATP+binding	0.946592	0.207986	0.999218
BP_GO:0007608_sensory+perception+of+smell	0.00161329	1	1.66658E-11
MF_GO:0004984_olfactory+receptor+activity	0.00112239	1	7.29727E-12
BP_GO:0007165_signal+transduction	0.991151	0.999906	0.504297
CC_GO:0005739_mitochondrion	2.76748E-06	0	0.999978
BP_GO:0007275_multicellular+organismal+development	0.988181	0.732443	0.994849
MF_GO:0005509_calcium+ion+binding	0.983431	0.804309	0.984189
CC_GO:0005783_endoplasmic+reticulum	0.8174	0.0731132	0.997938
MF_GO:0003676_nucleic+acid+binding	0.842621	0.00981557	0.999832
MF_GO:0016301_kinase+activity	0.673825	0.0182028	0.997141
MF_GO:0003700_transcription+factor+activity	0.850684	0.0301801	0.999624
MF_GO:0004871_signal+transducer+activity	0.978763	0.99995	0.252438
MF_GO:0003824_catalytic+activity	0.0215925	0.0205879	0.244261
MF_GO:0005488_binding	0.323283	0.037293	0.884325
BP_GO:0008152_metabolic+process	0.00870239	0.000241937	0.689502
CC_GO:0005794_Golgi+apparatus	0.589943	0.0296493	0.992758
BP_GO:0006468_protein+amino+acid+phosphorylation	0.92696	0.302754	0.99306
MF_GO:0016491_oxidoreductase+activity	0.151917	0.0118951	0.840928
BP_GO:0006811_ion+transport	0.998832	0.866331	0.999209
BP_GO:0055114_oxidation+reduction	0.0851312	0.00610745	0.77884
MF_GO:0004672_protein+kinase+activity	0.848579	0.102982	0.995245
MF_GO:0003723_RNA+binding	0.268696	0.000684868	0.992558
MF_GO:0004930_G-protein+coupled+receptor+activity	0.858711	0.99998	0.0342226
BP_GO:0030154_cell+differentiation	0.974217	0.956642	0.85203
BP_GO:0015031_protein+transport	0.3464	0.00322239	0.996358
BP_GO:0006511_ubiquitin-dependent+protein+catabolic+process	0.221061	7.75828E-05	0.999752
BP_GO:0045449_regulation+of+transcription	0.632452	0.0562461	0.982847
MF_GO:0043565_sequence-specific+DNA+binding	0.555066	0.00390949	0.998326
MF_GO:0008233_peptidase+activity	0.949388	0.508319	0.988451
BP_GO:0007049_cell+cycle	0.290977	0.43766	0.270422
BP_GO:0007155_cell+adhesion	0.999465	0.998472	0.957124
CC_GO:0005856_cytoskeleton	0.192855	0.00103213	0.965125
MF_GO:0004674_protein+serine/threonine+kinase+activity	0.710962	0.0690132	0.986772
BP_GO:0006508_proteolysis	0.99268	0.961457	0.953943
BP_GO:0006915_apoptosis	0.0759732	0.0595637	0.340399
CC_GO:0030054_cell+junction	0.997902	0.974873	0.981655
MF_GO:0000287_magnesium+ion+binding	0.625819	0.191607	0.927603
CC_GO:0005615_extracellular+space	0.98439	0.833449	0.976734
CC_GO:0005887_integral+to+plasma+membrane	0.798049	0.978193	0.270516

CC_GO:0030529_ribonucleoprotein+complex	1.42727E-09	5.83888E-12	0.273664
BP_GO:0006412_translation	9.34273E-08	1.53839E-11	0.595204
MF_GO:0005525_GTP+binding	0.257956	0.0270524	0.912432
BP_GO:0045944_positive+regulation+of+transcription+from+RNA+polymera	0.824399	0.0294214	0.99931
MF_GO:0005216_ion+channel+activity	0.994041	0.954499	0.968711
CC_GO:0005829_cytosol	0.0649726	0.0109035	0.727857
CC_GO:0005743_mitochondrial+inner+membrane	0.000281501	2.88622E-08	0.997979
BP_GO:0006955_immune+response	0.715708	0.52542	0.753912
MF_GO:0005506_iron+ion+binding	0.45992	0.188314	0.775513
MF_GO:0016874_ligase+activity	0.886177	0.188599	0.998232
MF_GO:0003779_actin+binding	0.409941	0.0177308	0.970031
CC_GO:0005578_proteinaceous+extracellular+matrix	0.999939	0.985918	0.999814
BP_GO:0006397_mRNA+processing	0.22388	0.0361563	0.79282
MF_GO:0005215_transporter+activity	0.998751	0.619231	0.999908
BP_GO:0007242_intracellular+signaling+cascade	0.481684	0.0375667	0.966625
CC_GO:0005840_ribosome	1.20481E-10	7.51665E-12	0.112702
CC_GO:0045202_synapse	0.836722	0.414761	0.95094
BP_GO:0006629_lipid+metabolic+process	0.973566	0.757324	0.983325
CC_GO:0005624_membrane+fraction	0.939612	0.682552	0.964408
BP_GO:0051301_cell+division	0.734472	0.910374	0.367592
MF_GO:0030528_transcription+regulator+activity	0.279542	0.192525	0.539695
BP_GO:0050896_response+to+stimulus	0.350995	0.993102	0.0113042
BP_GO:0007264_small+GTPase+mediated+signal+transduction	0.643868	0.217884	0.951419
CC_GO:0005874_microtubule	0.588814	0.149348	0.930045
CC_GO:0031410_cytoplasmic+vesicle	0.965961	0.191914	0.999491
BP_GO:0008380_RNA+splicing	0.212308	0.0233219	0.830662
MF_GO:0016757_transferase+activity,+transferring+glycosyl+groups	0.676512	0.621586	0.632606
BP_GO:0007283_spermatogenesis	0.819615	0.814271	0.668599
CC_GO:0005667_transcription+factor+complex	0.0971803	0.0214852	0.637619
BP_GO:0006974_response+to+DNA+damage+stimulus	0.0153589	0.0419224	0.0909278
BP_GO:0000122_negative+regulation+of+transcription+from+RNA+polymer	0.423198	0.0372138	0.954606
MF_GO:0003735_structural+constituent+of+ribosome	1.72647E-10	1.81298E-10	0.032905
BP_GO:0006281_DNA+repair	0.00290395	0.0685938	0.0084564
MF_GO:0009055_electron+carrier+activity	0.48924	0.0737557	0.91961
BP_GO:0007399_nervous+system+development	0.895344	0.811446	0.814542
CC_GO:0005768_endosome	0.914957	0.251168	0.998282
CC_GO:0042995_cell+projection	0.0523606	0.158402	0.0998585
MF_GO:0005198_structural+molecule+activity	0.974102	0.973613	0.821095
BP_GO:0006886_intracellular+protein+transport	0.814073	0.450297	0.935844
MF_GO:0005125_cytokine+activity	0.960465	0.882099	0.902499
CC_GO:0005694_chromosome	0.0559771	0.0406231	0.319825
MF_GO:0005096_GTPase+activator+activity	0.998333	0.990027	0.967029
BP_GO:0005975_carbohydrate+metabolic+process	0.307311	0.201208	0.597842
MF_GO:0008415_acyltransferase+activity	0.907772	0.827454	0.82524
MF_GO:0005529_sugar+binding	0.838854	0.99046	0.230167
MF_GO:0008236_serine-type+peptidase+activity	0.98867	0.734299	0.995814
BP_GO:0006813_potassium+ion+transport	0.863474	0.473484	0.943914

MF_GO:0004713_protein+tyrosine+kinase+activity	0.775054	0.349275	0.910018
MF_GO:0008237_metallopeptidase+activity	0.954331	0.926368	0.841516
BP_GO:0007067_mitosis	0.652779	0.860534	0.343037
CC_GO:0005792_microsome	0.99126	0.501297	0.999258
CC_GO:0005764_lysosome	0.768117	0.466172	0.902277
BP_GO:0016192_vesicle-mediated+transport	0.527551	0.0518937	0.972719
MF_GO:0016563_transcription+activator+activity	0.373278	0.00774708	0.97696
BP_GO:0016568_chromatin+modification	0.914453	0.587247	0.958683
BP_GO:0001701_in+utero+embryonic+development	0.0169607	0.00500383	0.301942
MF_GO:0004252_serine-type+endopeptidase+activity	0.962716	0.839297	0.938622
CC_GO:0009897_external+side+of+plasma+membrane	0.980277	0.997949	0.562901
MF_GO:0008083_growth+factor+activity	0.305439	0.368172	0.35162
MF_GO:0030145_manganese+ion+binding	0.422165	0.208233	0.770931
BP_GO:0007507_heart+development	0.96649	0.929078	0.864413
MF_GO:0005244_voltage-gated+ion+channel+activity	0.987407	0.953051	0.931526
BP_GO:0008284_positive+regulation+of+cell+proliferation	0.606326	0.283043	0.839155
BP_GO:0008283_cell+proliferation	0.272984	0.120329	0.619022
BP_GO:0006954_inflammatory+response	0.8699	0.711911	0.846684
CC_GO:0005681_spliceosome	0.10911	0.0245172	0.61039
CC_GO:0031225_anchored+to+membrane	0.731479	0.256577	0.931118
CC_GO:0045211_postsynaptic+membrane	0.501677	0.514397	0.487313
CC_GO:0005882_intermediate+filament	0.0506305	0.711403	0.012693
MF_GO:0004721_phosphoprotein+phosphatase+activity	0.496262	0.227526	0.799051
MF_GO:0042803_protein+homodimerization+activity	0.25836	0.266638	0.387247
MF_GO:0017111_nucleoside-triphosphatase+activity	0.836028	0.716505	0.795642
BP_GO:0008285_negative+regulation+of+cell+proliferation	0.967462	0.653669	0.990098
MF_GO:0003774_motor+activity	0.9648	0.72868	0.986258
BP_GO:0016055_Wnt+receptor+signaling+pathway	0.220047	0.0327024	0.829551
MF_GO:0005085_guanyl-nucleotide+exchange+factor+activity	0.826873	0.569794	0.88903
BP_GO:0006464_protein+modification+process	0.503672	0.419475	0.61851
MF_GO:0016829_lyase+activity	0.0797536	0.266819	0.0799471
BP_GO:0006814_sodium+ion+transport	0.981056	0.913168	0.941439
MF_GO:0008168_methyltransferase+activity	0.347577	0.192328	0.650205
MF_GO:0030955_potassium+ion+binding	0.546747	0.317627	0.732839
MF_GO:0008289_lipid+binding	0.938806	0.654772	0.960465
MF_GO:0003924_GTPase+activity	0.577116	0.513214	0.605494
MF_GO:0005516_calmodulin+binding	0.708604	0.204919	0.952326
MF_GO:0003682_chromatin+binding	0.6913	0.484421	0.752797
MF_GO:0016853_isomerase+activity	0.0557298	0.00394474	0.673262
MF_GO:0016791_phosphatase+activity	0.0678078	0.0130412	0.517284
MF_GO:0016564_transcription+repressor+activity	0.61478	0.356247	0.795277
MF_GO:0015293_symporter+activity	0.993092	0.987391	0.8904
BP_GO:0006260_DNA+replication	0.00299036	0.00879709	0.0617253
BP_GO:0006470_protein+amino+acid+dephosphorylation	0.311677	0.0810285	0.784124
BP_GO:0042981_regulation+of+apoptosis	0.526179	0.206176	0.867819
MF_GO:0005102_receptor+binding	0.603311	0.512535	0.626691
MF_GO:0008234_cysteine-type+peptidase+activity	0.17473	0.116041	0.457406

BP_GO:0006816_calcium+ion+transport	0.999541	0.95115	0.998853
BP_GO:0006457_protein+folding	0.41106	0.174643	0.807618
BP_GO:0006897_endocytosis	0.990441	0.73689	0.996084
CC_GO:0005730_nucleolus	0.241573	0.00966373	0.893339
MF_GO:0020037_heme+binding	0.975567	0.804616	0.970221
MF_GO:0004866_endopeptidase+inhibitor+activity	0.878336	0.999026	0.173752
BP_GO:0009887_organ+morphogenesis	0.883288	0.935183	0.589752
BP_GO:0006357_regulation+of+transcription+from+RNA+polymerase+II+prc	0.79973	0.297325	0.965123
BP_GO:0019236_response+to+pheromone	0.608714	0.993742	0.105876
BP_GO:0043066_negative+regulation+of+apoptosis	0.815875	0.858692	0.559219
MF_GO:0005179_hormone+activity	0.218048	0.55747	0.151201
MF_GO:0031402_sodium+ion+binding	0.918471	0.873392	0.804689
MF_GO:0004222_metalloendopeptidase+activity	0.929763	0.835477	0.865663
BP_GO:0008610_lipid+biosynthetic+process	0.512216	0.572647	0.442538
MF_GO:0016503_pheromone+receptor+activity	0.622125	0.994853	0.106563
CC_GO:0005777_peroxisome	0.00503901	0.000209036	0.619102
BP_GO:0009952_anterior/posterior+pattern+formation	0.924962	0.381817	0.98246
MF_GO:0004386_helicase+activity	0.774694	0.42863	0.867798
BP_GO:0022900_electron+transport+chain	0.00222771	0.000500468	0.533894
MF_GO:0004725_protein+tyrosine+phosphatase+activity	0.343616	0.13473	0.668488
BP_GO:0001525_angiogenesis	0.915011	0.659226	0.941498
BP_GO:0016311_dephosphorylation	0.113379	0.0345532	0.499514
MF_GO:0004842_ubiquitin-protein+ligase+activity	0.223819	0.0036421	0.960333
CC_GO:0043234_protein+complex	0.404314	0.0708502	0.962621
MF_GO:0005550_pheromone+binding	0.630852	0.961945	0.225326
MF_GO:0004867_serine-type+endopeptidase+inhibitor+activity	0.908804	0.997844	0.31782
BP_GO:0007601_visual+perception	0.91767	0.989599	0.398016
MF_GO:0004518_nuclease+activity	0.615513	0.0963254	0.958685
MF_GO:0004497_monooxygenase+activity	0.955433	0.891883	0.886234
BP_GO:0045941_positive+regulation+of+transcription	0.312269	0.138663	0.67335
BP_GO:0016042_lipid+catabolic+process	0.629082	0.738326	0.437279
BP_GO:0006952_defense+response	0.914102	0.992071	0.408303
MF_GO:0008201_heparin+binding	0.651523	0.403545	0.823389
BP_GO:0042127_regulation+of+cell+proliferation	0.799327	0.490693	0.88116
BP_GO:0045087_innate+immune+response	0.962478	0.955318	0.791696
BP_GO:0006950_response+to+stress	0.607701	0.120223	0.972214
BP_GO:0007154_cell+communication	0.498671	0.326289	0.664176
BP_GO:0006334_nucleosome+assembly	0.483034	0.168921	0.904262
BP_GO:0045786_negative+regulation+of+cell+cycle	0.49729	0.324862	0.686684
BP_GO:0006935_chemotaxis	0.607542	0.301803	0.780382
BP_GO:0006917_induction+of+apoptosis	0.560005	0.419056	0.688317
MF_GO:0005249_voltage-gated+potassium+channel+activity	0.880462	0.761642	0.828243
CC_GO:0000786_nucleosome	0.0330862	0.0145943	0.429831
CC_GO:0005929_cilium	0.015584	0.0830011	0.0495314
BP_GO:0042742_defense+response+to+bacterium	0.984981	0.945186	0.9322
CC_GO:0009986_cell+surface	0.652691	0.47663	0.703595
CC_GO:0005923_tight+junction	0.981601	0.994164	0.661198

BP_GO:0007169_transmembrane+receptor+protein+tyrosine+kinase+signali	0.902084	0.782175	0.852518
BP_GO:0007218_neuropeptide+signaling+pathway	0.959809	0.990786	0.6511
BP_GO:0006631_fatty+acid+metabolic+process	0.303884	0.131746	0.824156
BP_GO:0030198_extracellular+matrix+organization	0.676133	0.471369	0.754163
CC_GO:0030424_axon	0.956096	0.797201	0.94093
BP_GO:0007229_integrin-mediated+signaling+pathway	0.830304	0.708837	0.783251
MF_GO:0046982_protein+heterodimerization+activity	0.889687	0.585708	0.946707
BP_GO:0007411_axon+guidance	0.774149	0.433144	0.874051
MF_GO:0046983_protein+dimerization+activity	0.0126491	0.00274132	0.387512
MF_GO:0016887_ATPase+activity	0.945317	0.850403	0.891237
CC_GO:0043025_cell+soma	0.752656	0.231203	0.937194
MF_GO:0042802_identical+protein+binding	0.399747	0.0836555	0.892607
BP_GO:0001558_regulation+of+cell+growth	0.286289	0.138873	0.684466
MF_GO:0005267_potassium+channel+activity	0.826535	0.782544	0.717492
MF_GO:0017124_SH3+domain+binding	0.600372	0.544507	0.595582
MF_GO:0016798_hydrolase+activity,+acting+on+glycosyl+bonds	0.679561	0.639495	0.617971
BP_GO:0030036_actin+cytoskeleton+organization	0.262641	0.14288	0.592822
BP_GO:0030324_lung+development	0.876082	0.532321	0.93847
CC_GO:0005741_mitochondrial+outer+membrane	0.00822514	0.00128974	0.505915
BP_GO:0007166_cell+surface+receptor+linked+signal+transduction	0.993	0.991942	0.863575
BP_GO:0007018_microtubule-based+movement	0.977335	0.856016	0.968186
BP_GO:0007626_locomotory+behavior	0.979219	0.901915	0.942367
BP_GO:0007605_sensory+perception+of+sound	0.922331	0.483427	0.984178
MF_GO:0016779_nucleotidyltransferase+activity	0.000518853	0.00259421	0.0385224
MF_GO:0031404_chloride+ion+binding	0.607442	0.617821	0.5417
CC_GO:0031012_extracellular+matrix	0.935206	0.78301	0.911143
CC_GO:0016324_apical+plasma+membrane	0.999677	0.99196	0.992141
BP_GO:0007420_brain+development	0.176756	0.0336194	0.679258
MF_GO:0003743_translation+initiation+factor+activity	0.085628	0.00817464	0.719253
CC_GO:0005604_basement+membrane	0.903755	0.894289	0.730214
MF_GO:0008134_transcription+factor+binding	0.637112	0.553193	0.660327
BP_GO:0016481_negative+regulation+of+transcription	0.570517	0.451919	0.664537
BP_GO:0006364_rRNA+processing	0.00941503	0.00381079	0.25738
BP_GO:0043065_positive+regulation+of+apoptosis	0.155103	0.2476	0.216248
BP_GO:0001501_skeletal+system+development	0.598573	0.382442	0.740449
MF_GO:0003713_transcription+coactivator+activity	0.702606	0.280138	0.916429
BP_GO:0006916_anti-apoptosis	0.0358762	0.00698482	0.488074
BP_GO:0045893_positive+regulation+of+transcription,+DNA-dependent	0.93332	0.190229	0.997832
BP_GO:0065002_intracellular+protein+transmembrane+transport	0.46103	0.173077	0.817713
BP_GO:0030900_forebrain+development	0.715221	0.568823	0.734718
MF_GO:0051082_unfolded+protein+binding	0.528248	0.283899	0.832267
BP_GO:0007268_synaptic+transmission	0.92767	0.796864	0.894316
BP_GO:0007156_homophilic+cell+adhesion	0.698464	0.907905	0.311923
CC_GO:0005625_soluble+fraction	0.247592	0.103584	0.688436
MF_GO:0016702_oxidoreductase+activity,+acting+on+single+donors+with+i	0.11633	0.16017	0.247015
MF_GO:0003714_transcription+corepressor+activity	0.636813	0.295733	0.879343
BP_GO:0001764_neuron+migration	0.512862	0.030471	0.954422

MF_GO:0050660_FAD+binding	0.694068	0.26824	0.932414
MF_GO:0005089_Rho+guanyl-nucleotide+exchange+factor+activity	0.74264	0.581268	0.778235
CC_GO:0019717_synaptosome	0.830357	0.747125	0.753973
CC_GO:0008076_voltage-gated+potassium+channel+complex	0.741077	0.328321	0.898665
MF_GO:0005262_calcium+channel+activity	0.998491	0.984874	0.978596
MF_GO:0005254_chloride+channel+activity	0.768831	0.588846	0.78004
MF_GO:0008565_protein+transporter+activity	0.820611	0.176137	0.989564
BP_GO:0001503_ossification	0.96714	0.995301	0.577654
MF_GO:0019992_diacylglycerol+binding	0.740981	0.667542	0.684411
BP_GO:0035023_regulation+of+Rho+protein+signal+transduction	0.718887	0.489943	0.823969
CC_GO:0015629_actin+cytoskeleton	0.277709	0.244701	0.441659
MF_GO:0003777_microtubule+motor+activity	0.906816	0.776106	0.871942
MF_GO:0008026_ATP-dependent+helicase+activity	0.675273	0.145681	0.927384
MF_GO:0004888_transmembrane+receptor+activity	0.907169	0.924247	0.675282
MF_GO:0004714_transmembrane+receptor+protein+tyrosine+kinase+activi	0.862154	0.864862	0.695518
MF_GO:0005507_copper+ion+binding	0.864561	0.696792	0.852273
BP_GO:0009790_embryonic+development	0.866743	0.54743	0.93183
BP_GO:0030326_embryonic+limb+morphogenesis	0.848313	0.753094	0.78046
BP_GO:0009791_post-embryonic+development	0.934953	0.535873	0.978938
MF_GO:0004519_endonuclease+activity	0.874672	0.473396	0.943906
BP_GO:0007409_axonogenesis	0.837734	0.589823	0.88723
BP_GO:0042254_ribosome+biogenesis	0.0530627	0.13061	0.120612
BP_GO:0008033_tRNA+processing	0.0221304	0.0170906	0.27926
CC_GO:0005746_mitochondrial+respiratory+chain	0.00209464	0.000431411	0.797565
BP_GO:0007219_Notch+signaling+pathway	0.72388	0.924723	0.2371
BP_GO:0042472_inner+ear+morphogenesis	0.953991	0.881027	0.8854
BP_GO:0045892_negative+regulation+of+transcription,+DNA-dependent	0.350487	0.508678	0.269936
BP_GO:0006694_steroid+biosynthetic+process	0.979896	0.880851	0.953902
BP_GO:0009058_biosynthetic+process	0.496603	0.153087	0.85785
BP_GO:0007389_pattern+specification+process	0.893643	0.92833	0.613007
BP_GO:0045454_cell+redox+homeostasis	0.00142071	0.0099307	0.0310953
MF_GO:0004221_ubiquitin+thiolesterase+activity	0.322331	0.15436	0.573724
BP_GO:0030182_neuron+differentiation	0.994208	0.973118	0.948768
CC_GO:0030027_lamellipodium	0.287824	0.4256	0.273134
BP_GO:0051726_regulation+of+cell+cycle	0.67452	0.279474	0.928979
CC_GO:0008021_synaptic+vesicle	0.553391	0.374729	0.718821
MF_GO:0030170_pyridoxal+phosphate+binding	0.648833	0.670606	0.524133
BP_GO:0006633_fatty+acid+biosynthetic+process	0.317584	0.342388	0.396245
BP_GO:0016337_cell-cell+adhesion	0.933895	0.65021	0.953645
BP_GO:0008104_protein+localization	0.977486	0.700636	0.992779
BP_GO:0010468_regulation+of+gene+expression	0.979886	0.99786	0.591262
BP_GO:0006417_regulation+of+translation	0.302769	0.400019	0.322436
BP_GO:0006486_protein+amino+acid+glycosylation	0.909228	0.727588	0.89501
BP_GO:0008202_steroid+metabolic+process	0.981618	0.889834	0.957033
CC_GO:0016459_myosin+complex	0.865276	0.625607	0.936474
BP_GO:0007519_skeletal+muscle+development	0.852904	0.344057	0.986559
MF_GO:0004197_cysteine-type+endopeptidase+activity	0.120311	0.383786	0.067857

MF_GO:0035091_phosphoinositide+binding	0.134675	0.0306321	0.621933
BP_GO:0051246_regulation+of+protein+metabolic+process	0.0347366	0.00402353	0.512106
BP_GO:0006812_cation+transport	0.568727	0.78886	0.306778
BP_GO:0006821_chloride+transport	0.81742	0.25593	0.963383
BP_GO:0015992_proton+transport	0.822494	0.553763	0.941723
MF_GO:0019787_small+conjugating+protein+ligase+activity	0.0992059	0.0101457	0.702735
CC_GO:0005643_nuclear+pore	0.812146	0.560179	0.890953
BP_GO:0043687_post-translational+protein+modification	0.0549384	0.00443753	0.616452
MF_GO:0003707_steroid+hormone+receptor+activity	0.0569786	0.0417508	0.354647
BP_GO:0006869_lipid+transport	0.953061	0.528584	0.984139
BP_GO:0007596_blood+coagulation	0.982294	0.987559	0.772317
BP_GO:0008203_cholesterol+metabolic+process	0.97926	0.772819	0.986654
BP_GO:0008360_regulation+of+cell+shape	0.459678	0.0628007	0.882043
BP_GO:0007050_cell+cycle+arrest	0.245935	0.312979	0.314209
MF_GO:0004879_ligand-dependent+nuclear+receptor+activity	0.0771442	0.0417508	0.458597
CC_GO:0005875_microtubule+associated+complex	0.717342	0.297477	0.945564
BP_GO:0045165_cell+fate+commitment	0.9256	0.995415	0.274731
CC_GO:0005789_endoplasmic+reticulum+membrane	0.825493	0.708452	0.787386
CC_GO:0005654_nucleoplasm	0.123066	0.162108	0.253232
BP_GO:0007126_meiosis	0.74398	0.961065	0.305994
BP_GO:0050909_sensory+perception+of+taste	0.34773	0.934451	0.0792478
BP_GO:0006754_ATP+biosynthetic+process	0.760892	0.312408	0.957244
BP_GO:0006979_response+to+oxidative+stress	0.558631	0.254684	0.851107
MF_GO:0015078_hydrogen+ion+transmembrane+transporter+activity	0.828215	0.489189	0.969775
CC_GO:0048471_perinuclear+region+of+cytoplasm	0.835504	0.357961	0.961441
BP_GO:0030097_hemopoiesis	0.738662	0.51187	0.822582
CC_GO:0000785_chromatin	0.690697	0.318065	0.876764
BP_GO:0007267_cell-cell+signaling	0.610313	0.674845	0.479466
BP_GO:0051028_mRNA+transport	0.82492	0.748238	0.743701
MF_GO:0008017_microtubule+binding	0.452424	0.209293	0.658494
CC_GO:0005635_nuclear+envelope	0.976642	0.826458	0.972483
CC_GO:0016023_cytoplasmic+membrane-bounded+vesicle	0.722807	0.574937	0.732189
BP_GO:0048704_embryonic+skeletal+system+morphogenesis	0.595823	0.168712	0.894757
BP_GO:0006887_exocytosis	0.984823	0.875791	0.974175
CC_GO:0000775_chromosome,+centromeric+region	0.914434	0.995801	0.289822
BP_GO:0009953_dorsal/ventral+pattern+formation	0.965459	0.922576	0.875735
MF_GO:0004812_aminoacyl-tRNA+ligase+activity	0.512295	0.156449	0.937152
BP_GO:0006096_glycolysis	0.342989	0.0400401	0.972742
BP_GO:0006006_glucose+metabolic+process	0.62729	0.312512	0.859398
BP_GO:0046777_protein+amino+acid+autophosphorylation	0.966208	0.900521	0.905048
CC_GO:0045121_membrane+raft	0.415423	0.195395	0.694498
MF_GO:0051536_iron-sulfur+cluster+binding	0.049669	0.0842658	0.174006
CC_GO:0000139_Golgi+membrane	0.11005	0.0365481	0.605306
CC_GO:0030425_dendrite	0.685006	0.070714	0.959104
MF_GO:0004527_exonuclease+activity	0.29471	0.296346	0.422269
BP_GO:0016477_cell+migration	0.886014	0.65115	0.922639
CC_GO:0005938_cell+cortex	0.841645	0.863441	0.572998

BP_GO:0001568_blood+vessel+development	0.479751	0.593939	0.3925
BP_GO:0006874_cellular+calcium+ion+homeostasis	0.886215	0.182998	0.991317
BP_GO:0001822_kidney+development	0.766707	0.451911	0.874959
BP_GO:0043524_negative+regulation+of+neuron+apoptosis	0.83975	0.708747	0.798894
CC_GO:0005813_centrosome	0.970848	0.750546	0.970995
BP_GO:0009615_response+to+virus	0.45566	0.828472	0.187986
BP_GO:0006836_neurotransmitter+transport	0.995235	0.971363	0.961937
BP_GO:0007010_cytoskeleton+organization	0.647659	0.171524	0.949861
BP_GO:0006396_RNA+processing	0.795272	0.547691	0.885947
CC_GO:0030018_Z+disc	0.208492	0.146115	0.550758
BP_GO:0007243_protein+kinase+cascade	0.145603	0.0386708	0.733548
CC_GO:0045177_apical+part+of+cell	0.917416	0.953508	0.674884
BP_GO:0051216_cartilage+development	0.609367	0.247345	0.820241
MF_GO:0031072_heat+shock+protein+binding	0.251725	0.2022	0.521004
BP_GO:0019221_cytokine-mediated+signaling+pathway	0.579991	0.913494	0.161957
BP_GO:0009968_negative+regulation+of+signal+transduction	0.862588	0.422562	0.943475
CC_GO:0005802_trans-Golgi+network	0.947234	0.832976	0.917258
CC_GO:0016323_basolateral+plasma+membrane	0.907456	0.924191	0.674486
MF_GO:0005097_Rab+GTPase+activator+activity	0.935077	0.614888	0.975052
BP_GO:0043010_camera-type+eye+development	0.465179	0.604516	0.357845
MF_GO:0003702_RNA+polymerase+II+transcription+factor+activity	0.0138782	0.00192058	0.454212
BP_GO:0007179_transforming+growth+factor+beta+receptor+signaling+pat	0.607204	0.424271	0.739926
BP_GO:0006139_nucleobase,+nucleoside,+nucleotide+and+nucleic+acid+me	0.0705813	0.124105	0.180816
MF_GO:0008092_cytoskeletal+protein+binding	0.700905	0.338372	0.844745
MF_GO:0004715_non-membrane+spanning+protein+tyrosine+kinase+activi	0.868259	0.695888	0.848955
MF_GO:0008009_chemokine+activity	0.564479	0.10091	0.868888
MF_GO:0004175_endopeptidase+activity	0.391302	0.0176547	0.984878
BP_GO:0006310_DNA+recombination	0.760615	0.757513	0.635384
MF_GO:0005230_extracellular+ligand-gated+ion+channel+activity	0.499151	0.287912	0.711145
MF_GO:0030594_neurotransmitter+receptor+activity	0.499151	0.287912	0.711145
BP_GO:0007160_cell-matrix+adhesion	0.912836	0.393725	0.986919
MF_GO:0016773_phosphotransferase+activity,+alcohol+group+as+acceptor	0.912719	0.740958	0.89437
BP_GO:0042593_glucose+homeostasis	0.863537	0.388645	0.960074
BP_GO:0008344_adult+locomotory+behavior	0.0739574	0.231346	0.0936496
BP_GO:0001570_vasculogenesis	0.120126	0.00685397	0.850549
BP_GO:0042475_odontogenesis+of+dentine-containing+tooth	0.455437	0.29001	0.65849
BP_GO:0006418_tRNA+aminoacylation+for+protein+translation	0.384612	0.0585667	0.937152
BP_GO:0051056_regulation+of+small+GTPase+mediated+signal+transductio	0.66106	0.62252	0.6089
MF_GO:0008138_protein+tyrosine/serine/threonine+phosphatase+activity	0.00246133	0.00467751	0.111152
BP_GO:0015986_ATP+synthesis+coupled+proton+transport	0.696651	0.340034	0.959287
BP_GO:0008643_carbohydrate+transport	0.142644	0.0445758	0.575275
BP_GO:0001756_somitogenesis	0.544471	0.436689	0.610701
MF_GO:0005351_sugar:hydrogen+symporter+activity	0.0918948	0.0445758	0.429118
BP_GO:0008654_phospholipid+biosynthetic+process	0.412837	0.221155	0.759621
MF_GO:0019904_protein+domain+specific+binding	0.606899	0.650745	0.462949
BP_GO:0006865_amino+acid+transport	0.938894	0.997268	0.245462
BP_GO:0001889_liver+development	0.307456	0.46169	0.26939

BP_GO:0007368_determination+of+left/right+symmetry	0.736608	0.735493	0.604632
BP_GO:0007417_central+nervous+system+development	0.983286	0.692775	0.983899
MF_GO:0003755_peptidyl-prolyl+cis-trans+isomerase+activity	0.372234	0.222534	0.642557
BP_GO:0042391_regulation+of+membrane+potential	0.9187	0.480743	0.981337
CC_GO:0001726_ruffle	0.238476	0.271806	0.350389
BP_GO:0000902_cell+morphogenesis	0.958715	0.832347	0.926965
BP_GO:0032313_regulation+of+Rab+GTPase+activity	0.944184	0.610364	0.979644
CC_GO:0005834_heterotrimeric+G-protein+complex	0.306319	0.235633	0.532809
BP_GO:0007286_spermatid+development	0.435334	0.730625	0.248955
MF_GO:0003899_DNA-directed+RNA+polymerase+activity	0.0050891	0.00152821	0.332359
MF_GO:0004091_carboxylesterase+activity	0.9629	0.845673	0.938341
BP_GO:0006730_one-carbon+compound+metabolic+process	0.368909	0.756218	0.121586
MF_GO:0004180_carboxypeptidase+activity	0.779614	0.976561	0.255176
MF_GO:0004896_cytokine+receptor+activity	0.704634	0.918199	0.397545
BP_GO:0000187_activation+of+MAPK+activity	0.729201	0.451262	0.822582
BP_GO:0000226_microtubule+cytoskeleton+organization	0.738401	0.838674	0.361382
MF_GO:0008146_sulfotransferase+activity	0.810592	0.906968	0.452261
BP_GO:0019882_antigen+processing+and+presentation	0.769877	0.50955	0.829567
BP_GO:0007517_muscle+development	0.239193	0.320374	0.294783
BP_GO:0018108_peptidyl-tyrosine+phosphorylation	0.493743	0.416902	0.567723
CC_GO:0030117_membrane+coat	0.375927	0.325396	0.483758
BP_GO:0007017_microtubule-based+process	0.697336	0.275808	0.958726
BP_GO:0016567_protein+ubiquitination	0.201636	0.0634452	0.787257
BP_GO:0030216_keratinocyte+differentiation	0.0434132	0.439362	0.0126654
MF_GO:0050381_unspecific+monooxygenase+activity	0.820487	0.974017	0.418811
MF_GO:0016820_hydrolase+activity,+acting+on+acid+anhydrides,+catalyzin	0.86787	0.492024	0.945002
BP_GO:0001843_neural+tube+closure	0.532798	0.783219	0.252537
BP_GO:0042493_response+to+drug	0.966809	0.938522	0.865533
BP_GO:0030163_protein+catabolic+process	0.383845	0.120224	0.81185
MF_GO:0051287_NAD+binding	0.448804	0.22257	0.810508
BP_GO:0030334_regulation+of+cell+migration	0.842431	0.49878	0.912904
BP_GO:0000165_MAPKKK+cascade	0.00114745	0.00160506	0.136608
BP_GO:0050885_neuromuscular+process+controlling+balance	0.902624	0.83908	0.79959
CC_GO:0030173_integral+to+Golgi+membrane	0.906097	0.558893	0.963732
MF_GO:0004693_cyclin-dependent+protein+kinase+activity	0.621585	0.902004	0.283051
BP_GO:0030879_mammary+gland+development	0.0104694	0.0140916	0.171634
CC_GO:0005925_focal+adhesion	0.611102	0.519643	0.655878
MF_GO:0043169_cation+binding	0.323695	0.415806	0.32201
CC_GO:0005905_coated+pit	0.750479	0.474299	0.864386
BP_GO:0003007_heart+morphogenesis	0.275866	0.38806	0.291742
BP_GO:0048706_embryonic+skeletal+system+development	0.440603	0.28316	0.59833
MF_GO:0008080_N-acetyltransferase+activity	0.953826	0.745972	0.953976
BP_GO:0043473_pigmentation	0.811372	0.520621	0.857682
CC_GO:0019898_extrinsic+to+membrane	0.967076	0.708372	0.965361
MF_GO:0003887_DNA-directed+DNA+polymerase+activity	0.00046331	0.00333326	0.028946
CC_GO:0030141_secretory+granule	0.818318	0.821424	0.635256
CC_GO:0019861_flagellum	0.263812	0.294675	0.369593

BP_GO:0006506_GPI+anchor+biosynthetic+process	0.695803	0.383467	0.836152
BP_GO:0008544_epidermis+development	0.000302333	0.0822822	0.000500273
BP_GO:0031424_keratinization	0.00441089	0.225641	0.00419847
MF_GO:0000155_two-component+sensor+activity	0.869676	0.976784	0.328909
BP_GO:0009987_cellular+process	0.116011	0.00779426	0.846436
BP_GO:0030183_B+cell+differentiation	0.844975	0.81685	0.709457
MF_GO:0019901_protein+kinase+binding	0.791972	0.546632	0.852878
BP_GO:0035264_multicellular+organism+growth	0.542772	0.840938	0.0977699
BP_GO:0006413_translational+initiation	0.559094	0.462849	0.621961
BP_GO:0006366_transcription+from+RNA+polymerase+II+promoter	0.62563	0.0766117	0.973766
MF_GO:0003746_translation+elongation+factor+activity	0.181816	0.0238884	0.881295
MF_GO:0005319_lipid+transporter+activity	0.998802	0.950481	0.995149
MF_GO:0016712_oxidoreductase+activity,+acting+on+paired+donors,+with-	0.686167	0.841294	0.460301
BP_GO:0001666_response+to+hypoxia	0.419932	0.570832	0.303534
MF_GO:0004722_protein+serine/threonine+phosphatase+activity	0.356383	0.347607	0.464201
MF_GO:0015662_ATPase+activity,+coupled+to+transmembrane+movement	0.581063	0.382478	0.732861
MF_GO:0042626_ATPase+activity,+coupled+to+transmembrane+movement	0.772591	0.419714	0.905601
CC_GO:0043005_neuron+projection	0.28273	0.689521	0.0738101
BP_GO:0040007_growth	0.879788	0.768301	0.83163
BP_GO:0045596_negative+regulation+of+cell+differentiation	0.390075	0.201825	0.654169
MF_GO:0004177_aminopeptidase+activity	0.193814	0.0301008	0.68642
MF_GO:0005044_scavenger+receptor+activity	0.920047	0.867756	0.805967
CC_GO:0000151_ubiquitin+ligase+complex	0.546432	0.134083	0.898389
BP_GO:0016358_dendrite+development	0.616351	0.757409	0.427868
MF_GO:0003684_damaged+DNA+binding	0.0716783	0.89516	0.000772314
MF_GO:0008430_selenium+binding	0.44707	0.196932	0.910567
CC_GO:0042383_sarcolemma	0.0747221	0.00539203	0.793177
MF_GO:0005200_structural+constituent+of+cytoskeleton	0.278782	0.0921244	0.727531
BP_GO:0001702_gastrulation+with+mouth+forming+second	0.606142	0.239445	0.825664
BP_GO:0006605_protein+targeting	0.623342	0.324511	0.851072
BP_GO:0019538_protein+metabolic+process	0.293198	0.176475	0.60581
BP_GO:0048705_skeletal+system+morphogenesis	0.984778	0.836243	0.986385
MF_GO:0005496_steroid+binding	0.186963	0.0243169	0.808264
BP_GO:0048754_branching+morphogenesis+of+a+tube	0.736468	0.406871	0.855245
MF_GO:0016881_acid-amino+acid+ligase+activity	0.963727	0.854982	0.934402
MF_GO:0003705_RNA+polymerase+II+transcription+factor+activity,+enhanc	0.347939	0.057392	0.901428
BP_GO:0007281_germ+cell+development	0.49649	0.261696	0.742075
BP_GO:0040018_positive+regulation+of+multicellular+organism+growth	0.94215	0.945736	0.725988
BP_GO:0008542_visual+learning	0.950297	0.919941	0.80687
BP_GO:0045665_negative+regulation+of+neuron+differentiation	0.355094	0.139821	0.764849
BP_GO:0006333_chromatin+assembly+or+disassembly	0.94576	0.637775	0.956918
BP_GO:0001890_placenta+development	0.946364	0.943039	0.731009
CC_GO:0030286_dynein+complex	0.550213	0.379529	0.728155
CC_GO:0016529_sarcoplasmic+reticulum	0.0861026	0.0335812	0.612413
BP_GO:0006958_complement+activation,+classical+pathway	0.978205	0.973714	0.798907
MF_GO:0051015_actin+filament+binding	0.598482	0.13948	0.980892
CC_GO:0008305_integrin+complex	0.959882	0.976326	0.707729

BP_GO:0032496_response+to+lipopolysaccharide	0.600146	0.208206	0.909691
BP_GO:0006606_protein+import+into+nucleus	0.693398	0.557422	0.736358
MF_GO:0045028_purinergic+nucleotide+receptor+activity,+G-protein+coupl	0.833575	0.781069	0.72911
BP_GO:0048469_cell+maturation	0.850722	0.816112	0.70417
BP_GO:0006338_chromatin+remodeling	0.887152	0.616836	0.915484
BP_GO:0007628_adult+walking+behavior	0.593303	0.40012	0.710915
CC_GO:0005921_gap+junction	0.962006	0.870146	0.918251
MF_GO:0003725_double-stranded+RNA+binding	0.600112	0.164281	0.894417
MF_GO:0003729_mRNA+binding	0.865312	0.823893	0.724182
BP_GO:0030317_sperm+motility	0.782984	0.711264	0.708477
BP_GO:0005977_glycogen+metabolic+process	0.445931	0.401272	0.557378
BP_GO:0010811_positive+regulation+of+cell-substrate+adhesion	0.335639	0.20083	0.509384
BP_GO:0016126_sterol+biosynthetic+process	0.937178	0.818364	0.892489
BP_GO:0006826_iron+ion+transport	0.557851	0.254251	0.815489
CC_GO:0005911_cell-cell+junction	0.941422	0.564835	0.963084
BP_GO:0001658_ureteric+bud+branching	0.971794	0.951228	0.873065
MF_GO:0016758_transferase+activity,+transferring+hexosyl+groups	0.653648	0.744299	0.466953
BP_GO:0001656_metanephros+development	0.648958	0.720719	0.497021
BP_GO:0006914_autophagy	0.0125105	0.0231339	0.135527
BP_GO:0030030_cell+projection+organization	0.596298	0.955279	0.0607976
CC_GO:0001669_acrosome	0.638109	0.877359	0.311123
MF_GO:0004364_glutathione+transferase+activity	0.577418	0.016383	0.997658
MF_GO:0008483_transaminase+activity	0.69061	0.785501	0.377924
CC_GO:0030863_cortical+cytoskeleton	0.0374227	0.0278205	0.204144
BP_GO:0006953_acute-phase+response	0.999319	0.981887	0.992381
BP_GO:0001649_osteoblast+differentiation	0.187123	0.0777665	0.51514
BP_GO:0008217_regulation+of+blood+pressure	0.0935634	0.197165	0.150482
CC_GO:0005769_early+endosome	0.874163	0.222133	0.998913
BP_GO:0045444_fat+cell+differentiation	0.412756	0.52962	0.349865
BP_GO:0030073_insulin+secretion	0.871678	0.871736	0.670577
BP_GO:0009954_proximal/distal+pattern+formation	0.294983	0.13466	0.590473
MF_GO:0004601_peroxidase+activity	0.486081	0.395631	0.669805
MF_GO:0015297_antiporter+activity	0.384584	0.360452	0.457648
BP_GO:0000160_two-component+signal+transduction+system+(phosphorel	0.922816	0.996826	0.328909
BP_GO:0050731_positive+regulation+of+peptidyl-tyrosine+phosphorylation	0.961291	0.944863	0.823632
BP_GO:0048663_neuron+fate+commitment	0.86058	0.799904	0.753156
BP_GO:0007266_Rho+protein+signal+transduction	0.522901	0.353407	0.697105
BP_GO:0006695_cholesterol+biosynthetic+process	0.846546	0.629665	0.871012
BP_GO:0007015_actin+filament+organization	0.999227	0.938941	0.997835
CC_GO:0005770_late+endosome	0.602588	0.346102	0.837116
BP_GO:0001755_neural+crest+cell+migration	0.950933	0.858977	0.897432
BP_GO:0019835_cytolysis	0.507609	0.215014	0.782961
BP_GO:0009636_response+to+toxin	0.40652	0.284176	0.549444
BP_GO:0051258_protein+polymerization	0.800412	0.519356	0.91344
BP_GO:0009117_nucleotide+metabolic+process	0.0470612	0.00578432	0.540411
CC_GO:0031224_intrinsic+to+membrane	0.671507	0.887178	0.318374
MF_GO:0016788_hydrolase+activity,+acting+on+ester+bonds	0.948105	0.510432	0.976969

BP_GO:0040014_regulation+of+multicellular+organism+growth	0.720259	0.725374	0.580081
BP_GO:0001569_patterning+of+blood+vessels	0.282135	0.153073	0.552285
MF_GO:0004553_hydrolase+activity,+hydrolyzing+O-glycosyl+compounds	0.227039	0.417142	0.194022
CC_GO:0030131_clathrin+adaptor+complex	0.286918	0.453713	0.244754
BP_GO:0030178_negative+regulation+of+Wnt+receptor+signaling+pathway	0.429227	0.0335332	0.934074
BP_GO:0007189_G-protein+signaling,+adenylate+cyclase+activating+pathwa	0.639927	0.489266	0.678445
BP_GO:0007610_behavior	0.184209	0.360072	0.197266
BP_GO:0007059_chromosome+segregation	0.950743	0.959445	0.779606
BP_GO:0048666_neuron+development	0.72564	0.221687	0.91672
BP_GO:0009566_fertilization	0.384486	0.144437	0.705436
CC_GO:0045095_keratin+filament	0.0345065	0.640079	0.00996666
BP_GO:0042632_cholesterol+homeostasis	0.984148	0.555108	0.99729
MF_GO:0004857_enzyme+inhibitor+activity	0.131195	0.135422	0.344268
CC_GO:0030426_growth+cone	0.00518989	0.0126106	0.064382
MF_GO:0016772_transferase+activity,+transferring+phosphorus-containing-	0.405316	0.340224	0.570802
MF_GO:0004114_3',5'-cyclic-nucleotide+phosphodiesterase+activity	0.884159	0.84962	0.770635
BP_GO:0015758_glucose+transport	0.215443	0.00443135	0.907214
BP_GO:0006879_cellular+iron+ion+homeostasis	0.180647	0.388972	0.176051
MF_GO:0016831_carboxy-lyase+activity	0.142051	0.468919	0.092129
BP_GO:0008286_insulin+receptor+signaling+pathway	0.852101	0.0520213	0.99813
BP_GO:0006829_zinc+ion+transport	0.197645	0.19409	0.367542
BP_GO:0042552_myelination	0.333193	0.417217	0.323643
MF_GO:0005083_small+GTPase+regulator+activity	0.412337	0.599126	0.273203
BP_GO:0000082_G1/S+transition+of+mitotic+cell+cycle	0.78669	0.577278	0.823557
MF_GO:0018024_histone-lysine+N-methyltransferase+activity	0.596332	0.220929	0.775587
BP_GO:0006986_response+to+unfolded+protein	0.976066	0.952135	0.888719
BP_GO:0007369_gastrulation	0.988852	0.894274	0.972509
BP_GO:0006919_caspase+activation	0.817352	0.728709	0.769908
BP_GO:0006259_DNA+metabolic+process	0.988306	0.715288	0.992043
BP_GO:0008584_male+gonad+development	0.869003	0.508952	0.932911
BP_GO:0001892_embryonic+placenta+development	0.475778	0.403364	0.588275
CC_GO:0005839_proteasome+core+complex	0.102298	0.00710034	0.942364
MF_GO:0004298_threonine-type+endopeptidase+activity	0.102298	0.00710034	0.942364
MF_GO:0005272_sodium+channel+activity	0.588343	0.43983	0.6534
MF_GO:0004890_GABA-A+receptor+activity	0.117411	0.194985	0.203505
BP_GO:0008652_amino+acid+biosynthetic+process	0.0363032	0.124725	0.075112
MF_GO:0016616_oxidoreductase+activity,+acting+on+the+CH-OH+group+of	0.00287403	0.00350143	0.202191
MF_GO:0005201_extracellular+matrix+structural+constituent	0.989668	0.971759	0.911733
BP_GO:0030335_positive+regulation+of+cell+migration	0.556214	0.245677	0.764197
BP_GO:0007265_Ras+protein+signal+transduction	0.429868	0.454366	0.440835
MF_GO:0004181_metallocarboxypeptidase+activity	0.602762	0.911737	0.204398
BP_GO:0007005_mitochondrion+organization	0.152093	0.0711842	0.549877
BP_GO:0006909_phagocytosis	0.385066	0.741285	0.135273
CC_GO:0005913_cell-cell+adherens+junction	0.74043	0.703863	0.645167
BP_GO:0021987_cerebral+cortex+development	0.821921	0.768625	0.717127
BP_GO:0001657_ureteric+bud+development	0.962678	0.951528	0.779725
BP_GO:0030855_epithelial+cell+differentiation	0.745662	0.757425	0.58055

MF_GO:0008373_sialyltransferase+activity	0.826957	0.48285	0.910276
BP_GO:0043588_skin+development	0.761039	0.556978	0.792312
MF_GO:0016627_oxidoreductase+activity,+acting+on+the+CH-CH+group+of	0.644528	0.301883	0.896454
BP_GO:0001707_mesoderm+formation	0.81373	0.356735	0.953215
CC_GO:0005740_mitochondrial+envelope	0.456194	0.0553269	0.9897
BP_GO:0048468_cell+development	0.322121	0.234804	0.535779
BP_GO:0006913_nucleocytoplasmic+transport	0.466532	0.554091	0.367943
CC_GO:0005819_spindle	0.766418	0.523113	0.828717
BP_GO:0031175_neurite+development	0.995437	0.997241	0.753388
MF_GO:0003697_single-stranded+DNA+binding	0.0556336	0.0981224	0.150254
BP_GO:0048511_rhythmic+process	0.775448	0.897846	0.277373
BP_GO:0001541_ovarian+follicle+development	0.263516	0.00114092	0.96458
CC_GO:0000119_mediator+complex	0.690804	0.312861	0.907212
BP_GO:0007223_Wnt+receptor+signaling+pathway,+calcium+modulating+p	0.449479	0.738666	0.235334
CC_GO:0009434_microtubule-based+flagellum	0.786691	0.826116	0.607744
BP_GO:0007606_sensory+perception+of+chemical+stimulus	0.349501	0.927138	0.235099
BP_GO:0034097_response+to+cytokine+stimulus	0.569225	0.462929	0.618402
BP_GO:0006941_striated+muscle+contraction	0.689071	0.169923	0.983555
MF_GO:0005520_insulin-like+growth+factor+binding	0.327311	0.46126	0.25231
BP_GO:0006099_tricarboxylic+acid+cycle	0.100521	0.0270522	0.839584
BP_GO:0051016_barbed-end+actin+filament+capping	0.481431	0.123082	0.731231
BP_GO:0030199_collagen+fibril+organization	0.998782	0.945868	0.995764
MF_GO:0004129_cytochrome-c+oxidase+activity	0.367592	0.252542	0.762016
MF_GO:0050662_coenzyme+binding	0.0324988	0.0720588	0.12331
BP_GO:0051260_protein+homooligomerization	0.198989	0.219622	0.354304
BP_GO:0032312_regulation+of+ARF+GTPase+activity	0.872387	0.490312	0.974707
MF_GO:0008060_ARF+GTPase+activator+activity	0.872387	0.490312	0.974707
MF_GO:0005178_integrin+binding	0.44661	0.344293	0.60983
BP_GO:0048536_spleen+development	0.649864	0.313799	0.781703
BP_GO:0048538_thymus+development	0.826647	0.171997	0.976565
BP_GO:0042102_positive+regulation+of+T+cell+proliferation	0.361515	0.185412	0.653293
MF_GO:0005245_voltage-gated+calcium+channel+activity	0.921171	0.511851	0.980891
BP_GO:0019233_sensory+perception+of+pain	0.523448	0.65685	0.387484
BP_GO:0007492_endoderm+development	0.749022	0.672186	0.700845
BP_GO:0007612_learning	0.905214	0.990894	0.206663
BP_GO:0042733_embryonic+digit+morphogenesis	0.983023	0.321989	0.999078
CC_GO:0001750_photoreceptor+outer+segment	0.84697	0.72236	0.801251
MF_GO:0004869_cysteine+protease+inhibitor+activity	0.870157	0.885141	0.637518
CC_GO:0005922_connexon+complex	0.995867	0.994451	0.883698
BP_GO:0007602_phototransduction	0.810944	0.936597	0.35407
BP_GO:0007613_memory	0.650702	0.702615	0.495672
BP_GO:0007623_circadian+rhythm	0.994136	0.999323	0.384386
BP_GO:0044267_cellular+protein+metabolic+process	0.745434	0.72168	0.618668
CC_GO:0001533_cornified+envelope	0.00206534	0.0401394	0.0115465
BP_GO:0006641_triacylglycerol+metabolic+process	0.671847	0.417859	0.806497
CC_GO:0001725_stress+fiber	0.823979	0.0755219	0.999677
BP_GO:0006094_gluconeogenesis	0.419965	0.362248	0.577802

MF_GO:0019838_growth+factor+binding	0.172496	0.0743399	0.560063
BP_GO:0030001_metal+ion+transport	0.542056	0.405727	0.657282
BP_GO:0006469_negative+regulation+of+protein+kinase+activity	0.0382347	0.00493921	0.558475
MF_GO:0005540_hyaluronic+acid+binding	0.630101	0.846622	0.276019
BP_GO:0007157_heterophilic+cell+adhesion	0.132663	0.602809	0.0148307
BP_GO:0030218_erythrocyte+differentiation	0.897585	0.941859	0.573797
BP_GO:0030155_regulation+of+cell+adhesion	0.837755	0.893519	0.395783
BP_GO:0009408_response+to+heat	0.30971	0.106288	0.868063
MF_GO:0004089_carbonate+dehydratase+activity	0.430103	0.832847	0.080336
BP_GO:0030217_T+cell+differentiation	0.947732	0.78474	0.957889
BP_GO:0007569_cell+aging	0.714795	0.885943	0.295038
BP_GO:0006820_anion+transport	0.297987	0.285196	0.439976
MF_GO:0016849_phosphorus-oxygen+lyase+activity	0.695191	0.74862	0.551898
BP_GO:0007585_respiratory+gaseous+exchange	0.982919	0.917532	0.94915
CC_GO:0000118_histone+deacetylase+complex	0.112261	0.0381692	0.541633
BP_GO:0043410_positive+regulation+of+MAPKKK+cascade	0.343497	0.304374	0.481907
MF_GO:0005544_calcium-dependent+phospholipid+binding	0.450586	0.192978	0.717902
CC_GO:0016605_PML+body	0.0913411	0.172549	0.166394
BP_GO:0009411_response+to+UV	0.446967	0.389579	0.532915
BP_GO:0048589_developmental+growth	0.976816	0.334244	0.995008
BP_GO:0030509_BMP+signaling+pathway	0.0601975	0.155937	0.118986
BP_GO:0050679_positive+regulation+of+epithelial+cell+proliferation	0.938808	0.434598	0.976105
MF_GO:0008378_galactosyltransferase+activity	0.445449	0.854338	0.117044
BP_GO:0045597_positive+regulation+of+cell+differentiation	0.683681	0.676339	0.58403
MF_GO:0005328_neurotransmitter:sodium+symporter+activity	0.955774	0.838398	0.92866
BP_GO:0006289_nucleotide-excision+repair	0.56786	0.370103	0.725065
BP_GO:0035116_embryonic+hindlimb+morphogenesis	0.706596	0.70441	0.594598
MF_GO:0030414_protease+inhibitor+activity	0.977852	0.771838	0.972863
MF_GO:0019843_rRNA+binding	0.0229559	0.000229442	0.945794
MF_GO:0005126_hematopoietin/interferon-class+(D200-domain)+cytokine+	0.140727	0.398599	0.135542
BP_GO:0006936_muscle+contraction	0.307814	0.0216337	0.934086
BP_GO:0006749_glutathione+metabolic+process	0.026598	0.000792111	0.665068
BP_GO:0016049_cell+growth	0.691182	0.269556	0.924376
BP_GO:0018105_peptidyl-serine+phosphorylation	0.858318	0.174022	0.976012
BP_GO:0042110_T+cell+activation	0.901406	0.611986	0.938046
MF_GO:0016769_transferase+activity,+transferring+nitrogenous+groups	0.625974	0.428219	0.791573
BP_GO:0001932_regulation+of+protein+amino+acid+phosphorylation	0.595693	0.181722	0.894612
MF_GO:0031418_L-ascorbic+acid+binding	0.335077	0.0533616	0.938612
BP_GO:0048535_lymph+node+development	0.983224	0.829177	0.983142
MF_GO:0051539_4+iron,+4+sulfur+cluster+binding	0.106704	0.128516	0.274932
MF_GO:0008324_cation+transmembrane+transporter+activity	0.30308	0.443907	0.286049
BP_GO:0002053_positive+regulation+of+mesenchymal+cell+proliferation	0.115838	0.0544291	0.465337
BP_GO:0008016_regulation+of+heart+contraction	0.232852	0.0933634	0.727101
BP_GO:0030890_positive+regulation+of+B+cell+proliferation	0.864579	0.79393	0.771326
BP_GO:0006644_phospholipid+metabolic+process	0.555564	0.712685	0.376586
CC_GO:0015630_microtubule+cytoskeleton	0.780251	0.0511236	0.989067
BP_GO:0009190_cyclic+nucleotide+biosynthetic+process	0.614729	0.74862	0.43581

BP_GO:0007528_neuromuscular+junction+development	0.0547467	0.0313057	0.445232
BP_GO:0015711_organic+anion+transport	0.686549	0.732569	0.574119
BP_GO:0007566_embryo+implantation	0.895747	0.5728	0.962923
BP_GO:0032526_response+to+retinoic+acid	0.347584	0.0503156	0.790827
BP_GO:0046677_response+to+antibiotic	0.664434	0.742132	0.464238
BP_GO:0006885_regulation+of+pH	0.622068	0.572152	0.600446
CC_GO:0030176_integral+to+endoplasmic+reticulum+membrane	0.249836	0.157738	0.599074
BP_GO:0033077_T+cell+differentiation+in+the+thymus	0.297946	0.176046	0.548348
MF_GO:0004402_histone+acetyltransferase+activity	0.558855	0.812455	0.302141
BP_GO:0001947_heart+looping	0.531298	0.350647	0.689639
BP_GO:0021766_hippocampus+development	0.684027	0.526737	0.739995
MF_GO:0004623_phospholipase+A2+activity	0.379644	0.729644	0.189492
BP_GO:0030901_midbrain+development	0.36895	0.303666	0.52912
BP_GO:0007276_gamete+generation	0.272123	0.266536	0.372081
BP_GO:0060079_regulation+of+excitatory+postsynaptic+membrane+potent	0.810516	0.973374	0.200032
MF_GO:0005261_cation+channel+activity	0.324448	0.65626	0.128827
BP_GO:0048646_anatomical+structure+formation	0.90304	0.956018	0.551798
BP_GO:0007565_female+pregnancy	0.413919	0.492001	0.387018
MF_GO:0050840_extracellular+matrix+binding	0.810471	0.813439	0.632022
BP_GO:0001816_cytokine+production	0.262685	0.0653437	0.709833
MF_GO:0008137_NADH+dehydrogenase+(ubiquinone)+activity	0.0046705	0.00256843	0.44388
BP_GO:0006928_cell+motion	0.653499	0.569337	0.641232
BP_GO:0008299_isoprenoid+biosynthetic+process	0.738714	0.232923	0.955921
BP_GO:0051092_positive+regulation+of+NF-kappaB+transcription+factor+ac	0.946655	0.604117	0.95988
MF_GO:0016706_oxidoreductase+activity,+acting+on+paired+donors,+with-	0.152034	0.0865728	0.585443
MF_GO:0004709_MAP+kinase+kinase+kinase+activity	0.835953	0.834885	0.640526
MF_GO:0019001_guanyl+nucleotide+binding	0.766695	0.64915	0.760015
BP_GO:0007611_learning+or+memory	0.839638	0.961473	0.334249
BP_GO:0009653_anatomical+structure+morphogenesis	0.716043	0.563539	0.746237
MF_GO:0004629_phospholipase+C+activity	0.759289	0.282644	0.919093
BP_GO:0051276_chromosome+organization	0.326922	0.486287	0.289294
BP_GO:0060021_palate+development	0.750136	0.384258	0.919696
CC_GO:0000793_condensed+chromosome	0.773774	0.35012	0.903482
BP_GO:0031069_hair+follicle+morphogenesis	0.743004	0.606518	0.724511
BP_GO:0007595_lactation	0.646177	0.114802	0.950342
MF_GO:0008013_beta-catenin+binding	0.191219	0.0482948	0.599661
BP_GO:0042113_B+cell+activation	0.865436	0.68429	0.8506
BP_GO:0001942_hair+follicle+development	0.659737	0.865876	0.316317
MF_GO:0015171_amino+acid+transmembrane+transporter+activity	0.687629	0.983665	0.165958
MF_GO:0016493_C-C+chemokine+receptor+activity	0.408097	0.849021	0.0981151
BP_GO:0007254_JNK+cascade	0.5941	0.584103	0.55055
CC_GO:0005669_transcription+factor+TFIID+complex	0.00982976	0.157246	0.0155549
BP_GO:0006888_ER+to+Golgi+vesicle-mediated+transport	0.552213	0.156128	0.964581
MF_GO:0003712_transcription+cofactor+activity	0.520633	0.516039	0.513115
BP_GO:0000184_nuclear-transcribed+mRNA+catabolic+process,+nonsense-r	0.972597	0.976739	0.704082
CC_GO:0000795_synaptonemal+complex	0.715093	0.733854	0.586398
CC_GO:0005762_mitochondrial+large+ribosomal+subunit	0.0517007	0.0337984	0.528802

BP_GO:0007269_neurotransmitter+secretion	0.755069	0.83437	0.408356
CC_GO:0001673_male+germ+cell+nucleus	0.363175	0.257975	0.534951
MF_GO:0017137_Rab+GTPase+binding	0.9897	0.933368	0.965002
MF_GO:0004970_ionotropic+glutamate+receptor+activity	0.684561	0.959372	0.125329
MF_GO:0005164_tumor+necrosis+factor+receptor+binding	0.774097	0.593893	0.770971
BP_GO:0007339_binding+of+sperm+to+zona+pellucida	0.318601	0.811512	0.107731
BP_GO:0001580_detection+of+chemical+stimulus+involved+in+sensory+per	0.497769	0.888009	0.284485
CC_GO:0042612_MHC+class+I+protein+complex	0.0526241	0.173249	0.0920335
BP_GO:0001974_blood+vessel+remodeling	0.642075	0.727062	0.512627
CC_GO:0031594_neuromuscular+junction	0.883736	0.573513	0.933507
BP_GO:0032880_regulation+of+protein+localization	0.751044	0.303203	0.877982
BP_GO:0030032_lamellipodium+assembly	0.982833	0.72463	0.989111
BP_GO:0050776_regulation+of+immune+response	0.52994	0.27685	0.710249
BP_GO:0010332_response+to+gamma+radiation	0.414567	0.461072	0.420277
BP_GO:0030336_negative+regulation+of+cell+migration	0.697602	0.357532	0.90589
MF_GO:0004653_polypeptide+N-acetylgalactosaminyltransferase+activity	0.715138	0.688629	0.612949
BP_GO:0042130_negative+regulation+of+T+cell+proliferation	0.576839	0.533544	0.575352
BP_GO:0007224_smoothened+signaling+pathway	0.87041	0.202725	0.980849
BP_GO:0030318_melanocyte+differentiation	0.925408	0.321529	0.990134
BP_GO:0000723_telomere+maintenance	0.446079	0.16166	0.889154
BP_GO:0007249_I-kappaB+kinase/NF-kappaB+cascade	0.450291	0.656515	0.259356
MF_GO:0005543_phospholipid+binding	0.246934	0.176972	0.441727
BP_GO:0001502_cartilage+condensation	0.624447	0.68705	0.470833
BP_GO:0035108_limb+morphogenesis	0.245815	0.39842	0.237493
BP_GO:0001708_cell+fate+specification	0.796486	0.441818	0.922042
BP_GO:0042221_response+to+chemical+stimulus	0.735499	0.380017	0.866212
BP_GO:0030574_collagen+catabolic+process	0.961594	0.679443	0.965561
BP_GO:0000059_protein+import+into+nucleus,+docking	0.810512	0.811608	0.616121
CC_GO:0016363_nuclear+matrix	0.454355	0.2706	0.69836
BP_GO:0030308_negative+regulation+of+cell+growth	0.619737	0.518926	0.631439
MF_GO:0004889_nicotinic+acetylcholine-activated+cation-selective+channe	0.847005	0.388698	0.958175
BP_GO:0008306_associative+learning	0.899664	0.924082	0.717797
CC_GO:0005932_microtubule+basal+body	0.414564	0.83565	0.0333778
MF_GO:0005003_ephrin+receptor+activity	0.560417	0.605841	0.493774
BP_GO:0030534_adult+behavior	0.225201	0.0661711	0.669438
MF_GO:0005234_extracellular-glutamate-gated+ion+channel+activity	0.624351	0.944864	0.125329
CC_GO:0015935_small+ribosomal+subunit	5.67891E-05	2.47725E-06	0.519744
CC_GO:0005863_striated+muscle+thick+filament	0.543499	0.193047	0.921385
BP_GO:0006520_amino+acid+metabolic+process	0.516465	0.59902	0.388671
MF_GO:0004697_protein+kinase+C+activity	0.277857	0.167018	0.538842
BP_GO:0007512_adult+heart+development	0.121722	0.360209	0.103586
MF_GO:0046914_transition+metal+ion+binding	0.286452	0.112301	0.770682
CC_GO:0005581_collagen	0.970511	0.910523	0.90782
BP_GO:0006637_acyl-CoA+metabolic+process	0.446735	0.237723	0.746156
MF_GO:0051537_2+iron,+2+sulfur+cluster+binding	0.0545935	0.0582263	0.252955
BP_GO:0030301_cholesterol+transport	0.908781	0.819377	0.856957
BP_GO:0001934_positive+regulation+of+protein+amino+acid+phosphorylati	0.977483	0.992847	0.805647

MF_GO:0005539_glycosaminoglycan+binding	0.0646974	0.414651	0.0374791
MF_GO:0046873_metal+ion+transmembrane+transporter+activity	0.4845	0.288738	0.657282
BP_GO:0048286_alveolus+development	0.670174	0.654742	0.601689
BP_GO:0008219_cell+death	0.741013	0.635933	0.724454
BP_GO:0007040_lysosome+organization	0.862469	0.794947	0.785904
BP_GO:0044237_cellular+metabolic+process	0.00436837	0.00370248	0.262777
BP_GO:0016525_negative+regulation+of+angiogenesis	0.276986	0.501362	0.151798
BP_GO:0048066_pigmentation+during+development	0.90544	0.842154	0.804802
BP_GO:0019370_leukotriene+biosynthetic+process	0.585072	0.171778	0.933125
BP_GO:0042445_hormone+metabolic+process	0.904617	0.477048	0.958684
MF_GO:0050661_NADP+binding	0.965535	0.814749	0.955811
BP_GO:0051789_response+to+protein+stimulus	0.846265	0.988001	0.237438
CC_GO:0005912_adherens+junction	0.988941	0.971623	0.905163
BP_GO:0048873_homeostasis+of+number+of+cells+within+a+tissue	0.745233	0.592764	0.791044
CC_GO:0005903_brush+border	0.658102	0.91511	0.275756
MF_GO:0046961_proton-transporting+ATPase+activity,+rotational+mechani	0.600377	0.37335	0.847913
MF_GO:0004860_protein+kinase+inhibitor+activity	0.769042	0.554028	0.799177
BP_GO:0031016_pancreas+development	0.155517	0.0758581	0.520122
MF_GO:0005484_SNAP+receptor+activity	0.741777	0.363323	0.947093
BP_GO:0016043_cellular+component+organization	0.604783	0.780012	0.299674
BP_GO:0060041_retina+development+in+camera-type+eye	0.374469	0.130978	0.807321
MF_GO:0005057_receptor+signaling+protein+activity	0.915634	0.935663	0.678152
MF_GO:0016818_hydrolase+activity,+acting+on+acid+anhydrides,+in+phosp	0.466696	0.235777	0.712046
BP_GO:0042060_wound+healing	0.87525	0.770749	0.819156
BP_GO:0035115_embryonic+forelimb+morphogenesis	0.344563	0.296363	0.481302
BP_GO:0048593_camera-type+eye+morphogenesis	0.593409	0.229946	0.872015
CC_GO:0000776_kinetochore	0.346876	0.690207	0.180516
BP_GO:0006302_double-strand+break+repair	0.771938	0.944808	0.414178
BP_GO:0016998_cell+wall+catabolic+process	0.885659	0.91741	0.609429
MF_GO:0003995_acyl-CoA+dehydrogenase+activity	0.377664	0.171853	0.787961
BP_GO:0031290_retinal+ganglion+cell+axon+guidance	0.736509	0.453426	0.835354
BP_GO:0042384_cilium+assembly	0.37	0.374045	0.447944
MF_GO:0016566_specific+transcriptional+repressor+activity	0.768675	0.649603	0.80867
CC_GO:0000781_chromosome,+telomeric+region	0.162745	0.360455	0.155942
BP_GO:0030902_hindbrain+development	0.0411734	0.389136	0.0172568
BP_GO:0001824_blastocyst+development	0.391309	0.310961	0.566745
BP_GO:0007422_peripheral+nervous+system+development	0.96807	0.937729	0.864257
MF_GO:0004864_protein+phosphatase+inhibitor+activity	0.181363	0.102183	0.546222
BP_GO:0050680_negative+regulation+of+epithelial+cell+proliferation	0.330018	0.13968	0.735567
BP_GO:0048167_regulation+of+synaptic+plasticity	0.948636	0.692877	0.96097
BP_GO:0000398_nuclear+mRNA+splicing,+via+spliceosome	0.984343	0.854143	0.982626
MF_GO:0004190_aspartic-type+endopeptidase+activity	0.60877	0.698166	0.440103
BP_GO:0021983_pituitary+gland+development	0.496282	0.640894	0.379601
MF_GO:0005212_structural+constituent+of+eye+lens	0.995228	0.974237	0.96036
BP_GO:0048839_inner+ear+development	0.808444	0.262319	0.974787
BP_GO:0001829_trophectodermal+cell+differentiation	0.752891	0.82011	0.455779
BP_GO:0007214_gamma-aminobutyric+acid+signaling+pathway	0.384109	0.0665384	0.801424

CC_GO:0042734_presynaptic+membrane	0.939213	0.962225	0.566602
MF_GO:0008227_amine+receptor+activity	0.904662	0.961201	0.555772
CC_GO:0022626_cytosolic+ribosome	0.0379895	0.0395533	0.351371
BP_GO:0042157_lipoprotein+metabolic+process	0.962617	0.821578	0.940423
BP_GO:0002474_antigen+processing+and+presentation+of+peptide+antigen	0.0245296	0.145479	0.0457582
CC_GO:0030017_sarcomere	0.260627	0.091727	0.761667
BP_GO:0019886_antigen+processing+and+presentation+of+exogenous+pep	0.99848	0.949385	0.993307
BP_GO:0006839_mitochondrial+transport	0.936867	0.810541	0.896142
BP_GO:0006071_glycerol+metabolic+process	0.830802	0.56403	0.858504
BP_GO:0007178_transmembrane+receptor+protein+serine/threonine+kinas	0.508947	0.679448	0.332069
BP_GO:0006635_fatty+acid+beta-oxidation	0.085499	0.0905016	0.328207
CC_GO:0005778_peroxisomal+membrane	0.226981	0.0902888	0.763111
BP_GO:0048514_blood+vessel+morphogenesis	0.566431	0.354487	0.729051
BP_GO:0001759_induction+of+an+organ	0.737532	0.286435	0.928225
BP_GO:0042098_T+cell+proliferation	0.273236	0.501856	0.155225
CC_GO:0008287_protein+serine/threonine+phosphatase+complex	0.638719	0.601171	0.608705
BP_GO:0016485_protein+processing	0.650018	0.510086	0.712042
BP_GO:0001958_endochondral+ossification	0.334316	0.356383	0.397842
BP_GO:0006471_protein+amino+acid+ADP-ribosylation	0.0367689	0.543921	0.00212611
MF_GO:0004707_MAP+kinase+activity	0.630208	0.423719	0.746167
MF_GO:0004437_inositol+or+phosphatidylinositol+phosphatase+activity	0.232146	0.130979	0.53492
MF_GO:0016747_transferase+activity,+transferring+acyl+groups+other+thar	0.827764	0.750214	0.745801
CC_GO:0005614_interstitial+matrix	0.121799	0.0610447	0.344339
MF_GO:0004435_phosphoinositide+phospholipase+C+activity	0.641277	0.123287	0.905818
MF_GO:0005247_voltage-gated+chloride+channel+activity	0.968215	0.783586	0.966979
BP_GO:0051402_neuron+apoptosis	0.698364	0.468777	0.774966
MF_GO:0004198_calcium-dependent+cysteine-type+endopeptidase+activity	0.429969	0.342988	0.597724
MF_GO:0017048_Rho+GTPase+binding	0.619611	0.915884	0.214547
MF_GO:0008514_organic+anion+transmembrane+transporter+activity	0.862342	0.95496	0.586243
BP_GO:0006665_sphingolipid+metabolic+process	0.950824	0.98074	0.60538
MF_GO:0004835_tubulin-tyrosine+ligase+activity	0.47107	0.636218	0.363148
BP_GO:0006700_C21-steroid+hormone+biosynthetic+process	0.873303	0.604453	0.890035
BP_GO:0009409_response+to+cold	0.205013	0.236183	0.343088
MF_GO:0005242_inward+rectifier+potassium+channel+activity	0.179946	0.327195	0.205349
MF_GO:0005070_SH3/SH2+adaptor+activity	0.431229	0.202088	0.840021
BP_GO:0001782_B+cell+homeostasis	0.914597	0.621866	0.980764
BP_GO:0008624_induction+of+apoptosis+by+extracellular+signals	0.225727	0.725894	0.0648129
BP_GO:0009966_regulation+of+signal+transduction	0.821071	0.456071	0.905175
BP_GO:0001662_behavioral+fear+response	0.234749	0.224382	0.382066
MF_GO:0008553_hydrogen-exporting+ATPase+activity,+phosphorylative+m	0.779966	0.780002	0.574976
MF_GO:0008022_protein+C-terminus+binding	0.0489506	0.0991857	0.133391
BP_GO:0030514_negative+regulation+of+BMP+signaling+pathway	0.966946	0.668411	0.979962
CC_GO:0019897_extrinsic+to+plasma+membrane	0.482797	0.343196	0.655618
BP_GO:0032012_regulation+of+ARF+protein+signal+transduction	0.957535	0.922169	0.872752
MF_GO:0005086_ARF+guanyl-nucleotide+exchange+factor+activity	0.957535	0.922169	0.872752
BP_GO:0001975_response+to+amphetamine	0.900879	0.979528	0.590335
BP_GO:0006284_base-excision+repair	0.62503	0.783633	0.337649

CC_GO:0014069_postsynaptic+density	0.265396	0.770927	0.0910182
BP_GO:0042491_auditory+receptor+cell+differentiation	0.865769	0.707759	0.868047
BP_GO:0035176_social+behavior	0.657402	0.674221	0.538936
BP_GO:0007338_single+fertilization	0.0759924	0.258504	0.0843071
BP_GO:0006414_translational+elongation	0.0494331	0.00056162	0.946249
MF_GO:0015380_anion+exchanger+activity	0.114936	0.205082	0.191048
BP_GO:0031018_endocrine+pancreas+development	0.29049	0.349191	0.344942
BP_GO:0048169_regulation+of+long-term+neuronal+synaptic+plasticity	0.118299	0.231327	0.176559
CC_GO:0030057_desmosome	0.0845893	0.132259	0.166691
BP_GO:0002009_morphogenesis+of+an+epithelium	0.228749	0.405008	0.195892
BP_GO:0045214_sarcomere+organization	0.00377667	0.000124362	0.628659
BP_GO:0050766_positive+regulation+of+phagocytosis	0.825706	0.882541	0.538382
MF_GO:0005537_mannose+binding	0.957859	0.877415	0.900565
BP_GO:0016044_membrane+organization	0.143961	0.365918	0.11644
MF_GO:0003950_NAD++ADP-ribosyltransferase+activity	0.32624	0.0588899	0.634702
BP_GO:0030168_platelet+activation	0.917633	0.70798	0.92686
BP_GO:0031668_cellular+response+to+extracellular+stimulus	0.0803315	0.129736	0.185228
MF_GO:0016805_dipeptidase+activity	0.735675	0.667037	0.676779
CC_GO:0005626_insoluble+fraction	0.507731	0.575919	0.378799
BP_GO:0006898_receptor-mediated+endocytosis	0.168548	0.197472	0.29108
CC_GO:0030175_filopodium	0.548119	0.507254	0.555635
MF_GO:0004467_long-chain-fatty-acid-CoA+ligase+activity	0.311656	0.229956	0.556973
MF_GO:0005518_collagen+binding	0.329134	0.0768227	0.813399
BP_GO:0030099_myeloid+cell+differentiation	0.995554	0.980145	0.948202
MF_GO:0030165_PDZ+domain+binding	0.901168	0.86712	0.765631
MF_GO:0008047_enzyme+activator+activity	0.862449	0.878444	0.680052
BP_GO:0030593_neutrophil+chemotaxis	0.868897	0.490605	0.913564
BP_GO:0007026_negative+regulation+of+microtubule+depolymerization	0.574141	0.0122101	0.957827
MF_GO:0004407_histone+deacetylase+activity	0.221317	0.0230648	0.847212
BP_GO:0006833_water+transport	0.716038	0.625186	0.682818
BP_GO:0006171_cAMP+biosynthetic+process	0.348553	0.567617	0.199826
BP_GO:0007274_neuromuscular+synaptic+transmission	0.71553	0.384328	0.876135
CC_GO:0030315_T-tubule	0.554475	0.269291	0.840999
BP_GO:0002062_chondrocyte+differentiation	0.601724	0.496368	0.640517
BP_GO:0009611_response+to+wounding	0.626045	0.871263	0.180432
BP_GO:0007076_mitotic+chromosome+condensation	0.471986	0.195294	0.61421
MF_GO:0004143_diacylglycerol+kinase+activity	0.0512327	0.266505	0.0561784
BP_GO:0050900_leukocyte+migration	0.86292	0.709294	0.844595
BP_GO:0007031_peroxisome+organization	0.0153338	0.001778	0.683409
CC_GO:0005891_voltage-gated+calcium+channel+complex	0.924453	0.670504	0.960417
BP_GO:0001836_release+of+cytochrome+c+from+mitochondria	0.60084	0.0746749	0.953844
BP_GO:0050905_neuromuscular+process	0.391672	0.176273	0.686694
BP_GO:0008045_motor+axon+guidance	0.808606	0.861021	0.544779
BP_GO:0050880_regulation+of+blood+vessel+size	0.664136	0.397896	0.734059
BP_GO:0007405_neuroblast+proliferation	0.65981	0.553953	0.688819
BP_GO:0048856_anatomical+structure+development	0.991902	0.843226	0.990417
BP_GO:0050808_synapse+organization	0.559067	0.355818	0.79449

MF_GO:0003993_acid+phosphatase+activity	0.973952	0.993178	0.641372
BP_GO:0044419_interspecies+interaction+between+organisms	0.793044	0.629847	0.776602
BP_GO:0008272_sulfate+transport	0.554466	0.109632	0.837501
BP_GO:0030282_bone+mineralization	0.618192	0.68995	0.463073
BP_GO:0015671_oxygen+transport	0.755268	0.870623	0.458586
MF_GO:0005344_oxygen+transporter+activity	0.755268	0.870623	0.458586
MF_GO:0030552_cAMP+binding	0.803364	0.329463	0.887879
BP_GO:0050852_T+cell+receptor+signaling+pathway	0.961019	0.82254	0.944552
BP_GO:0030878_thyroid+gland+development	0.392454	0.450662	0.388697
CC_GO:0000794_condensed+nuclear+chromosome	0.142615	0.0891982	0.45285
BP_GO:0006367_transcription+initiation+from+RNA+polymerase+II+promot	0.00187504	0.118003	0.00355292
BP_GO:0048477_oogenesis	0.326709	0.497218	0.256954
BP_GO:0019827_stem+cell+maintenance	0.321739	0.131923	0.657028
BP_GO:0001654_eye+development	0.898268	0.486069	0.961206
BP_GO:0042462_eye+photoreceptor+cell+development	0.0381402	0.291434	0.0389197
BP_GO:0001964_startle+response	0.809303	0.968396	0.425703
MF_GO:0005184_neuropeptide+hormone+activity	0.921037	0.24324	0.990613
BP_GO:0001709_cell+fate+determination	0.520028	0.164637	0.87029
CC_GO:0016607_nuclear+speck	0.970174	0.439364	0.994146
BP_GO:0035249_synaptic+transmission,+glutamatergic	0.711747	0.695427	0.60629
BP_GO:0045666_positive+regulation+of+neuron+differentiation	0.303925	0.00958153	0.88469
BP_GO:0043434_response+to+peptide+hormone+stimulus	0.10597	0.337797	0.108965
BP_GO:0019915_sequestering+of+lipid	0.471308	0.35905	0.684827
CC_GO:0030016_myofibril	0.0435039	0.0128425	0.670753
CC_GO:0005811_lipid+particle	0.0556584	0.0679211	0.286451
BP_GO:0043029_T+cell+homeostasis	0.812028	0.725425	0.746143
BP_GO:0009749_response+to+glucose+stimulus	0.623406	0.718722	0.392036
MF_GO:0004806_triacylglycerol+lipase+activity	0.0460582	0.220305	0.0340281
CC_GO:0005605_basal+lamina	0.783548	0.786687	0.607957
BP_GO:0018107_peptidyl-threonine+phosphorylation	0.796653	0.375508	0.892619
BP_GO:0048844_artery+morphogenesis	0.686068	0.737366	0.538882
BP_GO:0016064_immunoglobulin+mediated+immune+response	0.666383	0.348083	0.840973
BP_GO:0043123_positive+regulation+of+I-kappaB+kinase/NF-kappaB+casca	0.490373	0.82336	0.12816
BP_GO:0050853_B+cell+receptor+signaling+pathway	0.981963	0.975204	0.821929
BP_GO:0006541_glutamine+metabolic+process	0.629446	0.622181	0.573206
MF_GO:0004012_phospholipid-translocating+ATPase+activity	0.19874	0.397123	0.17491
CC_GO:0030139_endocytic+vesicle	0.320868	0.453722	0.260896
BP_GO:0043113_receptor+clustering	0.516632	0.0743572	0.961891
BP_GO:0006479_protein+amino+acid+methylation	0.528754	0.12096	0.932699
BP_GO:0055010_ventricular+cardiac+muscle+morphogenesis	0.192579	0.0983054	0.626478
BP_GO:0007204_elevation+of+cytosolic+calcium+ion+concentration	0.251131	0.424221	0.258119
BP_GO:0051085_chaperone+cofactor-dependent+protein+folding	0.805147	0.0369496	0.999716
BP_GO:0035265_organ+growth	0.306179	0.152743	0.621249
BP_GO:0016331_morphogenesis+of+embryonic+epithelium	0.0472811	0.0207802	0.320958
CC_GO:0000792_heterochromatin	0.468756	0.445183	0.51673
MF_GO:0005158_insulin+receptor+binding	0.283108	0.0947961	0.80558
BP_GO:0042474_middle+ear+morphogenesis	0.847893	0.79394	0.732611

BP_GO:0016571_histone+methylation	0.678042	0.578425	0.685033
BP_GO:0017148_negative+regulation+of+translation	0.468951	0.6692	0.180663
BP_GO:0006904_vesicle+docking+during+exocytosis	0.563428	0.373302	0.685221
CC_GO:0017053_transcriptional+repressor+complex	0.412325	0.224322	0.754209
CC_GO:0000159_protein+phosphatase+type+2A+complex	0.742608	0.827487	0.456899
BP_GO:0007498_mesoderm+development	0.363242	0.132797	0.833598
BP_GO:0045669_positive+regulation+of+osteoblast+differentiation	0.213134	0.29068	0.283354
BP_GO:0007259_JAK-STAT+cascade	0.512366	0.754448	0.221768
CC_GO:0005871_kinesin+complex	0.882863	0.79443	0.815141
BP_GO:0006493_protein+amino+acid+O-linked+glycosylation	0.679378	0.728499	0.45167
BP_GO:0010001_glial+cell+differentiation	0.567914	0.757844	0.323713
BP_GO:0035050_embryonic+heart+tube+development	0.0854288	0.507734	0.0657148
BP_GO:0006164_purine+nucleotide+biosynthetic+process	0.645503	0.745599	0.419371
BP_GO:0046330_positive+regulation+of+JNK+cascade	0.857808	0.667403	0.860073
BP_GO:0043161_proteasomal+ubiquitin-dependent+protein+catabolic+proc	0.279273	0.219394	0.504168
BP_GO:0000209_protein+polyubiquitination	0.745622	0.421793	0.895561
MF_GO:0015450_P-P-bond-hydrolysis-driven+protein+transmembrane+tran	0.697283	0.409272	0.831439
BP_GO:0030968_endoplasmic+reticulum+unfolded+protein+response	0.904357	0.889725	0.717097
MF_GO:0005149_interleukin-1+receptor+binding	0.205455	0.338055	0.234902
BP_GO:0050850_positive+regulation+of+calcium-mediated+signaling	0.94986	0.826517	0.923952
BP_GO:0007205_activation+of+protein+kinase+C+activity	0.089512	0.328907	0.0914795
CC_GO:0045120_pronucleus	0.19745	0.246828	0.302306
BP_GO:0045860_positive+regulation+of+protein+kinase+activity	0.996988	0.582538	0.999685
CC_GO:0005788_endoplasmic+reticulum+lumen	0.512091	0.141248	0.787467
MF_GO:0003690_double-stranded+DNA+binding	0.324524	0.156125	0.739252
BP_GO:0045190_isotype+switching	0.0375143	0.425415	0.0258027
BP_GO:0006298_mismatch+repair	0.241024	0.861201	0.0330531
MF_GO:0000049_tRNA+binding	0.554062	0.394357	0.677372
BP_GO:0045595_regulation+of+cell+differentiation	0.37112	0.250979	0.581911
CC_GO:0044424_intracellular+part	0.25069	0.653179	0.0659601
MF_GO:0004993_serotonin+receptor+activity	0.922179	0.90167	0.795378
CC_GO:0000178_exosome+(RNase+complex)	0.152564	0.0138106	0.774382
CC_GO:0000300_peripheral+to+membrane+of+membrane+fraction	0.212409	0.174462	0.555665
MF_GO:0008527_taste+receptor+activity	0.689878	0.888009	0.448112
BP_GO:0030833_regulation+of+actin+filament+polymerization	0.0122923	0.0437318	0.0721309
BP_GO:0006911_phagocytosis,+engulfment	0.405747	0.321252	0.520891
BP_GO:0006807_nitrogen+compound+metabolic+process	0.960964	0.960644	0.724096
MF_GO:0030674_protein+binding,+bridging	0.112965	0.144188	0.279714
CC_GO:0031941_filamentous+actin	0.282626	0.122971	0.56239
MF_GO:0008484_sulfuric+ester+hydrolase+activity	0.160359	0.118398	0.363369
BP_GO:0048738_cardiac+muscle+development	0.842944	0.761066	0.762718
MF_GO:0010181_FMN+binding	0.534222	0.1549	0.931307
BP_GO:0050830_defense+response+to+Gram-positive+bacterium	0.889847	0.0899102	0.976597
MF_GO:0048037_cofactor+binding	0.785647	0.808693	0.535679
BP_GO:0015914_phospholipid+transport	0.321083	0.1651	0.602702
BP_GO:0046716_muscle+maintenance	0.302579	0.132435	0.577509
BP_GO:0042573_retinoic+acid+metabolic+process	0.918286	0.998355	0.433593

BP_GO:0030048_actin+filament-based+movement	0.216417	0.0426023	0.765114
BP_GO:0007162_negative+regulation+of+cell+adhesion	0.920892	0.55134	0.924938
CC_GO:0000299_integral+to+membrane+of+membrane+fraction	0.500549	0.139356	0.882729
BP_GO:0051017_actin+filament+bundle+formation	0.637579	0.530081	0.746925
MF_GO:0015250_water+channel+activity	0.823865	0.625186	0.833424
BP_GO:0031663_lipopolysaccharide-mediated+signaling+pathway	0.212302	0.2516	0.326325
MF_GO:0004675_transmembrane+receptor+protein+serine/threonine+kina	0.330749	0.4497	0.304913
MF_GO:0005024_transforming+growth+factor+beta+receptor+activity	0.330749	0.4497	0.304913
MF_GO:0004016_adenylate+cyclase+activity	0.534698	0.693478	0.300084
BP_GO:0043085_positive+regulation+of+catalytic+activity	0.683971	0.211008	0.886374
MF_GO:0017110_nucleoside-diphosphatase+activity	0.834542	0.499719	0.925389
MF_GO:0050897_cobalt+ion+binding	0.741742	0.196222	0.900566
BP_GO:0048598_embryonic+morphogenesis	0.830272	0.413673	0.895024
BP_GO:0007200_G-protein+signaling,+coupled+to+IP3+second+messenger+	0.0109508	0.570451	0.000549445
MF_GO:0008603_cAMP-dependent+protein+kinase+regulator+activity	0.742024	0.527932	0.812311
CC_GO:0033177_proton-transporting+two-sector+ATPase+complex,+proton	0.942414	0.897325	0.828272
BP_GO:0046847_filopodium+formation	0.990392	0.903504	0.974397
BP_GO:0042771_DNA+damage+response,+signal+transduction+by+p53+clas	0.745833	0.714103	0.65597
BP_GO:0051145_smooth+muscle+cell+differentiation	0.670859	0.714993	0.567724
MF_GO:0016790_thiolester+hydrolase+activity	0.602929	0.41951	0.705007
BP_GO:0032869_cellular+response+to+insulin+stimulus	0.756269	0.668893	0.709198
CC_GO:0031105_septin+complex	0.798759	0.587497	0.824179
BP_GO:0048015_phosphoinositide-mediated+signaling	0.687453	0.83826	0.487223
MF_GO:0004683_calmodulin-dependent+protein+kinase+activity	0.815488	0.568995	0.873165
MF_GO:0008235_metalloexopeptidase+activity	0.422593	0.372431	0.526803
BP_GO:0048701_embryonic+cranial+skeleton+morphogenesis	0.533855	0.454379	0.599374
CC_GO:0005942_phosphoinositide+3-kinase+complex	0.999712	0.874328	0.999418
MF_GO:0004926_non-G-protein+coupled+7TM+receptor+activity	0.770541	0.652153	0.761249
BP_GO:0007190_activation+of+adenylate+cyclase+activity	0.550306	0.718937	0.327348
CC_GO:0005884_actin+filament	0.401734	0.283648	0.634696
BP_GO:0050728_negative+regulation+of+inflammatory+response	0.864759	0.754393	0.810507
BP_GO:0048821_erythrocyte+development	0.0356432	0.23358	0.0475901
BP_GO:0001837_epithelial+to+mesenchymal+transition	0.321473	0.457423	0.23822
MF_GO:0046933_hydrogen+ion+transporting+ATP+synthase+activity,+rotat	0.430655	0.244005	0.855552
MF_GO:0004428_inositol+or+phosphatidylinositol+kinase+activity	0.769912	0.73501	0.673924
BP_GO:0009888_tissue+development	0.982864	0.960838	0.881014
CC_GO:0005902_microvillus	0.614052	0.729168	0.48645
BP_GO:0016197_endosome+transport	0.827535	0.764836	0.728921
BP_GO:0006809_nitric+oxide+biosynthetic+process	0.735651	0.643951	0.70192
BP_GO:0040015_negative+regulation+of+multicellular+organism+growth	0.360167	0.134567	0.601675
CC_GO:0031227_intrinsic+to+endoplasmic+reticulum+membrane	0.985863	0.984847	0.866682
MF_GO:0015269_calcium-activated+potassium+channel+activity	0.44373	0.0297686	0.768207
BP_GO:0030539_male+genitalia+development	0.317296	0.396571	0.344047
CC_GO:0005720_nuclear+heterochromatin	0.604972	0.748095	0.401376
BP_GO:0055007_cardiac+muscle+cell+differentiation	0.436462	0.390194	0.585694
MF_GO:0001619_lysosphingolipid+and+lysophosphatidic+acid+receptor+act	0.815258	0.84147	0.592788
BP_GO:0007340_acrosome+reaction	0.825419	0.478	0.887845

BP_GO:0010165_response+to+X-ray	0.816932	0.976224	0.357303
BP_GO:0008543_fibroblast+growth+factor+receptor+signaling+pathway	0.0222407	0.0256968	0.199125
BP_GO:0042417_dopamine+metabolic+process	0.950277	0.911644	0.851563
CC_GO:0043197_dendritic+spine	0.531731	0.41106	0.698284
BP_GO:0018298_protein-chromophore+linkage	0.593018	0.965443	0.0899899
BP_GO:0016070_RNA+metabolic+process	0.499671	0.143714	0.789204
BP_GO:0016573_histone+acetylation	0.240417	0.697056	0.10144
BP_GO:0006342_chromatin+silencing	0.931001	0.864848	0.850594
CC_GO:0030672_synaptic+vesicle+membrane	0.941798	0.880381	0.850502
BP_GO:0006968_cellular+defense+response	0.147091	0.554678	0.0440676
CC_GO:0005814_centriole	0.0680133	0.304283	0.0219104
BP_GO:0007530_sex+determination	0.926632	0.973387	0.578992
BP_GO:0007548_sex+differentiation	0.824138	0.647829	0.851208
BP_GO:0002088_lens+development+in+camera-type+eye	0.320589	0.160907	0.653412
MF_GO:0019905_syntaxin+binding	0.512857	0.457141	0.548553
BP_GO:0007416_synaptogenesis	0.970644	0.743226	0.975126
CC_GO:0005758_mitochondrial+intermembrane+space	0.104878	0.089667	0.348935
BP_GO:0001522_pseudouridine+synthesis	0.0381449	0.0428946	0.15267
MF_GO:0009982_pseudouridine+synthase+activity	0.0381449	0.0428946	0.15267
BP_GO:0048265_response+to+pain	0.98769	0.897948	0.970282
BP_GO:0031589_cell-substrate+adhesion	0.462576	0.193784	0.946422
BP_GO:0006957_complement+activation,+alternative+pathway	0.104595	0.276159	0.104501
MF_GO:0008307_structural+constituent+of+muscle	0.323941	0.827954	0.030072
BP_GO:0019363_pyridine+nucleotide+biosynthetic+process	0.346137	0.285152	0.52786
BP_GO:0048741_skeletal+muscle+fiber+development	0.409141	0.718377	0.142203
BP_GO:0006937_regulation+of+muscle+contraction	0.155606	0.0734999	0.737622
BP_GO:0006825_copper+ion+transport	0.348522	0.20991	0.562263
BP_GO:0007159_leukocyte+adhesion	0.423555	0.814185	0.121867
MF_GO:0016290_palmitoyl-CoA+hydrolase+activity	0.561479	0.273569	0.75541
BP_GO:0032760_positive+regulation+of+tumor+necrosis+factor+production	0.97169	0.633636	0.985419
BP_GO:0043087_regulation+of+GTPase+activity	0.897371	0.930772	0.664526
CC_GO:0045263_proton-transporting+ATP+synthase+complex,+coupling+fa	0.220238	0.0593248	0.987418
BP_GO:0009072_aromatic+amino+acid+family+metabolic+process	0.525651	0.848675	0.173717
MF_GO:0003954_NADH+dehydrogenase+activity	0.0274501	0.0121591	0.58412
BP_GO:0055085_transmembrane+transport	0.00414234	0.00159038	0.217119
MF_GO:0022891_substrate-specific+transmembrane+transporter+activity	0.00414234	0.00159038	0.217119
BP_GO:0001676_long-chain+fatty+acid+metabolic+process	0.341789	0.285885	0.547759
BP_GO:0042744_hydrogen+peroxide+catabolic+process	0.226468	0.326752	0.217986
BP_GO:0048813_dendrite+morphogenesis	0.661983	0.300695	0.975997
MF_GO:0005355_glucose+transmembrane+transporter+activity	0.00913411	0.00350968	0.334309
MF_GO:0004935_adrenoceptor+activity	0.353759	0.52188	0.27929
MF_GO:0016209_antioxidant+activity	0.12938	0.0497758	0.778979
BP_GO:0022008_neurogenesis	0.905709	0.974419	0.689278
BP_GO:0046676_negative+regulation+of+insulin+secretion	0.603436	0.0603444	0.972749
CC_GO:0001772_immunological+synapse	0.927893	0.589499	0.989791
BP_GO:0009116_nucleoside+metabolic+process	0.768533	0.471983	0.925983
BP_GO:0042640_anagen	0.596254	0.657143	0.431323

MF_GO:0008143_poly(A)+binding	0.793178	0.247076	0.967078
CC_GO:0012506_vesicle+membrane	0.450186	0.0927714	0.963506
BP_GO:0006691_leukotriene+metabolic+process	0.562607	0.347628	0.766963
BP_GO:0042755_eating+behavior	0.276645	0.137642	0.599966
BP_GO:0006309_DNA+fragmentation+during+apoptosis	0.639767	0.201894	0.910026
BP_GO:0006783_heme+biosynthetic+process	0.242012	0.00640176	0.933221
MF_GO:0016291_acyl-CoA+thioesterase+activity	0.851732	0.436634	0.949003
MF_GO:0015075_ion+transmembrane+transporter+activity	0.721503	0.798844	0.560941
MF_GO:0019903_protein+phosphatase+binding	0.711332	0.286976	0.911522
BP_GO:0043330_response+to+exogenous+dsRNA	0.723777	0.320071	0.815478
BP_GO:0002026_regulation+of+the+force+of+heart+contraction	0.0955197	0.0218777	0.755989
MF_GO:0005385_zinc+ion+transmembrane+transporter+activity	0.112444	0.287445	0.124183
BP_GO:0051899_membrane+depolarization	0.982481	0.731322	0.990934
BP_GO:0030100_regulation+of+endocytosis	0.600053	0.340811	0.917139
BP_GO:0048010_vascular+endothelial+growth+factor+receptor+signaling+p	0.0532572	0.173191	0.0948238
CC_GO:0005761_mitochondrial+ribosome	0.00458336	0.0408479	0.0260676
BP_GO:0001516_prostaglandin+biosynthetic+process	0.44456	0.246514	0.611279
BP_GO:0046488_phosphatidylinositol+metabolic+process	0.430694	0.354965	0.638136
BP_GO:0019722_calcium-mediated+signaling	0.982324	0.939653	0.922595
BP_GO:0000045_autophagic+vacuole+formation	0.0545762	0.0361798	0.320139
BP_GO:0008630_DNA+damage+response,+signal+transduction+resulting+in	0.862422	0.846509	0.717059
BP_GO:0050821_protein+stabilization	0.316407	0.240662	0.580728
BP_GO:0010467_gene+expression	0.754068	0.979444	0.217435
BP_GO:0015672_monovalent+inorganic+cation+transport	0.138969	0.149247	0.347654
BP_GO:0006801_superoxide+metabolic+process	0.389759	0.498835	0.347333
BP_GO:0046928_regulation+of+neurotransmitter+secretion	0.454285	0.902736	0.218419
BP_GO:0006857_oligopeptide+transport	0.177863	0.111718	0.512619
BP_GO:0009880_embryonic+pattern+specification	0.823183	0.610279	0.848797
CC_GO:0005876_spindle+microtubule	0.811573	0.370619	0.875604
BP_GO:0001833_inner+cell+mass+cell+proliferation	0.314136	0.141129	0.709455
BP_GO:0006352_transcription+initiation	0.146043	0.0660974	0.570989
BP_GO:0001556_oocyte+maturation	0.936023	0.757829	0.939333
CC_GO:0016604_nuclear+body	0.379239	0.1359	0.691801
BP_GO:0002052_positive+regulation+of+neuroblast+proliferation	0.396822	0.423116	0.430319
MF_GO:0004602_glutathione+peroxidase+activity	0.402268	0.257695	0.878451
MF_GO:0031625_ubiquitin+protein+ligase+binding	0.120712	0.100296	0.353579
CC_GO:0032420_stereocilium	0.469329	0.578723	0.379362
BP_GO:0006446_regulation+of+translational+initiation	0.284422	0.104048	0.739335
CC_GO:0005657_replication+fork	0.582272	0.797969	0.244111
CC_GO:0005892_nicotinic+acetylcholine-gated+receptor-channel+complex	0.798462	0.487792	0.88738
BP_GO:0045494_photoreceptor+cell+maintenance	0.437764	0.431318	0.480801
MF_GO:0008509_anion+transmembrane+transporter+activity	0.51972	0.566629	0.470898
MF_GO:0001664_G-protein-coupled+receptor+binding	0.496037	0.201124	0.749055
BP_GO:0006183_GTP+biosynthetic+process	0.00115986	0.000826857	0.123265
MF_GO:0015035_protein+disulfide+oxidoreductase+activity	0.00714039	0.00406723	0.26408
BP_GO:0006182_cGMP+biosynthetic+process	0.502963	0.19232	0.671337
BP_GO:0007257_activation+of+JNK+activity	0.824191	0.578691	0.868278

BP_GO:0048566_embryonic+gut+development	0.698858	0.482796	0.760513
BP_GO:0019226_transmission+of+nerve+impulse	0.790169	0.883728	0.619714
BP_GO:0007631_feeding+behavior	0.87432	0.772647	0.831349
BP_GO:0010212_response+to+ionizing+radiation	0.260922	0.167218	0.484839
MF_GO:0043022_ribosome+binding	0.0800705	0.375287	0.033636
BP_GO:0006402_mRNA+catabolic+process	0.857709	0.696907	0.855395
BP_GO:0060052_neurofilament+cytoskeleton+organization	0.933782	0.969729	0.348573
BP_GO:0048266_behavioral+response+to+pain	0.977226	0.933547	0.92
MF_GO:0004843_ubiquitin-specific+protease+activity	0.148956	0.566732	0.106099
MF_GO:0030983_mismatched+DNA+binding	0.183287	0.876702	0.0330531
BP_GO:0021549_cerebellum+development	0.87968	0.831401	0.751504
BP_GO:0043523_regulation+of+neuron+apoptosis	0.843051	0.562098	0.86459
MF_GO:0008271_secondary+active+sulfate+transmembrane+transporter+a	0.946746	0.641356	0.94773
MF_GO:0004983_neuropeptide+Y+receptor+activity	0.301381	0.560967	0.212812
BP_GO:0016265_death	0.495494	0.44982	0.55442
BP_GO:0030238_male+sex+determination	0.770278	0.949962	0.199243
CC_GO:0015934_large+ribosomal+subunit	0.0742851	0.0889822	0.29962
BP_GO:0000050_urea+cycle	0.975084	0.953285	0.856629
BP_GO:0005978_glycogen+biosynthetic+process	0.320158	0.214902	0.796877
BP_GO:0016310_phosphorylation	0.231652	0.231652	#N/A
MF_GO:0048407_platelet-derived+growth+factor+binding	0.798261	0.738695	0.706235
BP_GO:0001916_positive+regulation+of+T+cell+mediated+cytotoxicity	0.0853205	0.175431	0.154494
BP_GO:0050730_regulation+of+peptidyl-tyrosine+phosphorylation	0.310613	0.0823619	0.647345
BP_GO:0000910_cytokinesis	0.0872413	0.522974	0.0442669
MF_GO:0016811_hydrolase+activity,+acting+on+carbon-nitrogen+(but+not+	0.134198	0.439798	0.0887528
BP_GO:0042135_neurotransmitter+catabolic+process	0.607915	0.447772	0.795614
BP_GO:0006487_protein+amino+acid+N-linked+glycosylation	0.0976837	0.151979	0.214261
BP_GO:0060048_cardiac+muscle+contraction	0.849044	0.307562	0.982564
MF_GO:0004017_adenylate+kinase+activity	0.457623	0.386215	0.588964
MF_GO:0019205_nucleobase,+nucleoside,+nucleotide+kinase+activity	0.444001	0.396955	0.575412
MF_GO:0017127_cholesterol+transporter+activity	0.658575	0.272453	0.862087
BP_GO:0045766_positive+regulation+of+angiogenesis	0.458609	0.163902	0.825958
MF_GO:0016651_oxidoreductase+activity,+acting+on+NADH+or+NADPH	0.021871	0.0101797	0.695464
BP_GO:0035137_hindlimb+morphogenesis	0.496754	0.548033	0.452777
MF_GO:0005542_folic+acid+binding	0.996136	0.895239	0.992853
BP_GO:0046622_positive+regulation+of+organ+growth	0.92566	0.676223	0.968092
MF_GO:0016538_cyclin-dependent+protein+kinase+regulator+activity	0.153979	0.141369	0.37172
BP_GO:0030512_negative+regulation+of+transforming+growth+factor+beta	0.563242	0.15909	0.865785
MF_GO:0003854_3-beta-hydroxy-delta5-steroid+dehydrogenase+activity	0.268815	0.64958	0.18295
BP_GO:0008632_apoptotic+program	0.679731	0.388937	0.810239
BP_GO:0043407_negative+regulation+of+MAP+kinase+activity	0.146577	0.151658	0.380856
BP_GO:0046641_positive+regulation+of+alpha-beta+T+cell+proliferation	0.0336934	0.794759	0.00238952
BP_GO:0045582_positive+regulation+of+T+cell+differentiation	0.859493	0.621454	0.898061
BP_GO:0035162_embryonic+hemopoiesis	0.507244	0.31378	0.575809
MF_GO:0005391_sodium:potassium-exchanging+ATPase+activity	0.581142	0.490905	0.683288
BP_GO:0006270_DNA+replication+initiation	0.706513	0.626736	0.671146
MF_GO:0046332_SMAD+binding	0.861815	0.683324	0.921496

MF_GO:0008191_metalloendopeptidase+inhibitor+activity	0.830486	0.768758	0.731163
CC_GO:0005759_mitochondrial+matrix	0.101696	0.0208276	0.75118
MF_GO:0019841_retinol+binding	0.808614	0.550211	0.908635
BP_GO:0006878_cellular+copper+ion+homeostasis	0.762298	0.674307	0.724676
MF_GO:0008253_5'-nucleotidase+activity	0.315935	0.0860465	0.718805
BP_GO:0006548_histidine+catabolic+process	0.760183	0.890989	0.375614
BP_GO:0045060_negative+thymic+T+cell+selection	0.269412	0.737718	0.0512996
BP_GO:0006800_oxygen+and+reactive+oxygen+species+metabolic+process	0.0878716	0.00613838	0.66443
CC_GO:0005771_multivesicular+body	0.671579	0.302649	0.893244
BP_GO:0009395_phospholipid+catabolic+process	0.219068	0.631744	0.117584
MF_GO:0004702_receptor+signaling+protein+serine/threonine+kinase+activ	0.933139	0.787229	0.898402
BP_GO:0045453_bone+resorption	0.94111	0.588059	0.971201
BP_GO:0030889_negative+regulation+of+B+cell+proliferation	0.565104	0.81194	0.386853
BP_GO:0006744_ubiquinone+biosynthetic+process	0.205247	0.0543717	0.895102
MF_GO:0016303_1-phosphatidylinositol-3-kinase+activity	0.974178	0.874328	0.95133
BP_GO:0019752_carboxylic+acid+metabolic+process	0.485436	0.927001	0.141626
MF_GO:0008375_acetylglucosaminyltransferase+activity	0.735841	0.916241	0.419093
BP_GO:0046854_phosphoinositide+phosphorylation	0.889924	0.840652	0.786978
BP_GO:0051607_defense+response+to+virus	0.975466	0.982127	0.776664
MF_GO:0008195_phosphatidate+phosphatase+activity	0.247106	0.226922	0.379463
CC_GO:0035253_ciliary+rootlet	0.854284	0.926144	0.412871
BP_GO:0021954_central+nervous+system+neuron+development	0.98281	0.996024	0.316484
MF_GO:0015198_oligopeptide+transporter+activity	0.140197	0.0700199	0.512619
BP_GO:0017156_calcium+ion-dependent+exocytosis	0.981416	0.46077	0.99803
BP_GO:0021952_central+nervous+system+projection+neuron+axonogenesis	0.749741	0.777003	0.562863
CC_GO:0008180_signalosome	0.192262	0.14521	0.55318
MF_GO:0008276_protein+methyltransferase+activity	0.769081	0.486651	0.873009
MF_GO:0015485_cholesterol+binding	0.396992	0.286001	0.56181
CC_GO:0005776_autophagic+vacuole	0.308549	0.197278	0.580231
BP_GO:0006376_mRNA+splice+site+selection	0.96124	0.996349	0.230631
BP_GO:0042542_response+to+hydrogen+peroxide	0.758691	0.206863	0.924796
BP_GO:0008629_induction+of+apoptosis+by+intracellular+signals	0.0342931	0.00394169	0.260166
BP_GO:0016051_carbohydrate+biosynthetic+process	0.970772	0.862068	0.94098
MF_GO:0003841_1-acylglycerol-3-phosphate+O-acyltransferase+activity	0.442757	0.218679	0.742995
BP_GO:0000186_activation+of+MAPKK+activity	0.309024	0.0136176	0.957387
BP_GO:0007032_endosome+organization	0.391143	0.529212	0.150504
BP_GO:0000188_inactivation+of+MAPK+activity	0.83005	0.073383	0.93651
MF_GO:0030234_enzyme+regulator+activity	0.958271	0.753529	0.956198
CC_GO:0042470_melanosome	0.673802	0.52116	0.711174
BP_GO:0030325_adrenal+gland+development	0.237488	0.40119	0.231494
BP_GO:0051209_release+of+sequestered+calcium+ion+into+cytosol	0.953053	0.6641	0.960072
BP_GO:0050673_epithelial+cell+proliferation	0.91244	0.623517	0.924741
CC_GO:0005641_nuclear+envelope+lumen	0.62023	0.593599	0.577064
BP_GO:0031295_T+cell+costimulation	0.00049256	0.473174	0.000259901
BP_GO:0030316_osteoclast+differentiation	0.979905	0.591451	0.990581
BP_GO:0033198_response+to+ATP	0.595325	0.595799	0.529921
BP_GO:0001967_suckling+behavior	0.982862	0.963541	0.907119

BP_GO:0045671_negative+regulation+of+osteoclast+differentiation	0.421632	0.250251	0.592798
BP_GO:0006306_DNA+methylation	0.942532	0.759482	0.931081
BP_GO:0045668_negative+regulation+of+osteoblast+differentiation	0.693174	0.270434	0.853363
CC_GO:0033178_proton-transporting+two-sector+ATPase+complex,+catalyt	0.68287	0.490064	0.804779
BP_GO:0043525_positive+regulation+of+neuron+apoptosis	0.442386	0.128778	0.67062
BP_GO:0048148_behavioral+response+to+cocaine	0.936968	0.952452	0.711792
BP_GO:0048872_homeostasis+of+number+of+cells	0.527935	0.198357	0.906497
BP_GO:0031047_gene+silencing+by+RNA	0.958028	0.630772	0.956809
MF_GO:0004003_ATP-dependent+DNA+helicase+activity	0.1746	0.285323	0.228205
BP_GO:0043616_keratinocyte+proliferation	0.19279	0.67302	0.0588678
MF_GO:0016780_phosphotransferase+activity,+for+other+substituted+phos	0.592484	0.289314	0.883011
CC_GO:0000922_spindle+pole	0.866115	0.762839	0.90303
BP_GO:0050804_regulation+of+synaptic+transmission	0.752898	0.80851	0.519883
MF_GO:0019825_oxygen+binding	0.909962	0.875997	0.763766
BP_GO:0002087_regulation+of+respiratory+gaseous+exchange+by+neurolo	0.440823	0.383496	0.564133
MF_GO:0003796_lysozyme+activity	0.832515	0.895655	0.56693
BP_GO:0014032_neural+crest+cell+development	0.695716	0.893654	0.54061
BP_GO:0016601_Rac+protein+signal+transduction	0.610801	0.418274	0.743037
BP_GO:0000077_DNA+damage+checkpoint	0.357914	0.624388	0.220186
BP_GO:0001508_regulation+of+action+potential	0.965869	0.982028	0.575659
CC_GO:0043231_intracellular+membrane-bounded+organelle	0.807214	0.679163	0.772626
MF_GO:0015299_solute:hydrogen+antiporter+activity	0.382486	0.485659	0.356243
CC_GO:0043198_dendritic+shaft	0.903375	0.260229	0.989826
CC_GO:0031461_cullin-RING+ubiquitin+ligase+complex	0.205519	0.100296	0.578714
CC_GO:0030126_COPI+vesicle+coat	0.260119	0.0931007	0.558896
BP_GO:0048146_positive+regulation+of+fibroblast+proliferation	0.127257	0.420974	0.118025
MF_GO:0030553_cGMP+binding	0.880031	0.786667	0.809657
CC_GO:0005680_anaphase-promoting+complex	0.632142	0.159906	0.947204
CC_GO:0016272_prefoldin+complex	0.25973	0.229848	0.563139
MF_GO:0004383_guanylate+cyclase+activity	0.653657	0.384829	0.6995
CC_GO:0005844_polysome	0.765653	0.6024	0.787085
BP_GO:0021915_neural+tube+development	0.611315	0.758401	0.440512
MF_GO:0005452_inorganic+anion+exchanger+activity	0.452541	0.548781	0.393835
BP_GO:0007586_digestion	0.510752	0.699152	0.437969
BP_GO:0006959_humoral+immune+response	0.477658	0.406747	0.525065
BP_GO:0050877_neurological+system+process	0.77486	0.521264	0.858226
BP_GO:0016486_peptide+hormone+processing	0.94637	0.807507	0.912703
BP_GO:0016339_calcium-dependent+cell-cell+adhesion	0.793685	0.867657	0.492655
BP_GO:0050910_detection+of+mechanical+stimulus+involved+in+sensory+p	0.962106	0.538669	0.975076
BP_GO:0000724_double-strand+break+repair+via+homologous+recombinat	0.026554	0.350081	0.0122594
MF_GO:0016494_C-X-C+chemokine+receptor+activity	0.498338	0.915671	0.0612057
MF_GO:0009881_photoreceptor+activity	0.869899	0.965443	0.365508
BP_GO:0006228_UTP+biosynthetic+process	0.0017394	0.000826857	0.194095
BP_GO:0006241_CTP+biosynthetic+process	0.0017394	0.000826857	0.194095
MF_GO:0004550_nucleoside+diphosphate+kinase+activity	0.0017394	0.000826857	0.194095
MF_GO:0004526_ribonuclease+P+activity	0.0567602	0.0291527	0.334226
MF_GO:0008417_fucosyltransferase+activity	0.608389	0.676847	0.483514

CC_GO:0016585_chromatin+remodeling+complex	0.730243	0.420154	0.842138
BP_GO:0002076_osteoblast+development	0.457362	0.304276	0.659915
BP_GO:0001542_ovulation+from+ovarian+follicle	0.882543	0.452006	0.987005
BP_GO:0006476_protein+amino+acid+deacetylation	0.964806	0.791177	0.989813
BP_GO:0007141_male+meiosis+I	0.440016	0.763341	0.289583
CC_GO:0005832_chaperonin-containing+T-complex	0.167705	0.154733	0.408971
CC_GO:0042788_polysomal+ribosome	0.0358249	0.111623	0.025277
CC_GO:0031674_I+band	0.0145374	0.00210377	0.411981
CC_GO:0042627_chylomicron	0.943913	0.736105	0.935689
MF_GO:0004303_estradiol+17-beta-dehydrogenase+activity	0.286416	0.518808	0.199348
CC_GO:0043292_contractile+fiber	0.323747	0.323747	#N/A
CC_GO:0005901_caveola	0.0279076	0.029241	0.292463
MF_GO:0015020_glucuronosyltransferase+activity	0.742594	0.746094	0.602059
MF_GO:0016298_lipase+activity	0.591129	0.591504	0.53767
MF_GO:0005080_protein+kinase+C+binding	0.0118205	0.0015391	0.726253
BP_GO:0045059_positive+thymic+T+cell+selection	0.640131	0.389701	0.885302
BP_GO:0009435_NAD+biosynthetic+process	0.715821	0.807317	0.425025
BP_GO:0042572_retinol+metabolic+process	0.787898	0.823404	0.57981
BP_GO:0050798_activated+T+cell+proliferation	0.309145	0.699689	0.232496
BP_GO:0043409_negative+regulation+of+MAPKKK+cascade	0.127442	0.0344165	0.583333
BP_GO:0045576_mast+cell+activation	0.88609	0.846416	0.74227
CC_GO:0030864_cortical+actin+cytoskeleton	0.991774	0.614209	0.999034
BP_GO:0030031_cell+projection+assembly	0.575059	0.15176	0.850095
BP_GO:0045579_positive+regulation+of+B+cell+differentiation	0.436476	0.651112	0.223091
MF_GO:0043621_protein+self-association	0.284506	0.0518812	0.879089
BP_GO:0009165_nucleotide+biosynthetic+process	0.551381	0.583027	0.476269
CC_GO:0005765_lysosomal+membrane	0.980472	0.924029	0.931145
BP_GO:0000060_protein+import+into+nucleus,+translocation	0.733128	0.723376	0.628414
BP_GO:0050796_regulation+of+insulin+secretion	0.693826	0.672391	0.599055
MF_GO:0004745_retinol+dehydrogenase+activity	0.951138	0.955488	0.739034
MF_GO:0008121_ubiquinol-cytochrome-c+reductase+activity	0.0466677	0.00595322	0.694105
BP_GO:0045086_positive+regulation+of+interleukin-2+biosynthetic+process	0.715077	0.814611	0.410044
BP_GO:0002504_antigen+processing+and+presentation+of+peptide+or+poly	0.99095	0.994005	0.851295
CC_GO:0042613_MHC+class+II+protein+complex	0.99095	0.994005	0.851295
BP_GO:0043011_myeloid+dendritic+cell+differentiation	0.981685	0.962557	0.866911
BP_GO:0018345_protein+palmitoylation	0.793154	0.696682	0.771066
MF_GO:0016409_palmitoyltransferase+activity	0.793154	0.696682	0.771066
BP_GO:0042104_positive+regulation+of+activated+T+cell+proliferation	0.779139	0.686488	0.726311
CC_GO:0046658_anchored+to+plasma+membrane	0.752515	0.0986586	0.968708
MF_GO:0005021_vascular+endothelial+growth+factor+receptor+activity	0.717658	0.852651	0.340075
CC_GO:0005952_cAMP-dependent+protein+kinase+complex	0.897515	0.351912	0.971121
BP_GO:0007173_epidermal+growth+factor+receptor+signaling+pathway	0.301285	0.377801	0.3356
MF_GO:0030675_Rac+GTPase+activator+activity	0.842917	0.905888	0.395123
BP_GO:0045765_regulation+of+angiogenesis	0.343692	0.296561	0.548166
MF_GO:0051018_protein+kinase+A+binding	0.913414	0.820519	0.871614
CC_GO:0005881_cytoplasmic+microtubule	0.404291	0.230124	0.508253
BP_GO:0006835_dicarboxylic+acid+transport	0.976868	0.978173	0.741813

BP_GO:0030500_regulation+of+bone+mineralization	0.938365	0.992575	0.127911
BP_GO:0001933_negative+regulation+of+protein+amino+acid+phosphorylat	0.440714	0.0589056	0.84698
BP_GO:0050870_positive+regulation+of+T+cell+activation	0.970885	0.768771	0.983925
MF_GO:0017017_MAP+kinase+tyrosine/serine/threonine+phosphatase+acti	0.384608	0.149636	0.805392
CC_GO:0043296_apical+junction+complex	0.688416	0.654296	0.623216
BP_GO:0001952_regulation+of+cell-matrix+adhesion	0.28466	0.0741687	0.739347
MF_GO:0050750_low-density+lipoprotein+receptor+binding	0.825914	0.455936	0.924636
BP_GO:0001678_cellular+glucose+homeostasis	0.858736	0.586897	0.856733
MF_GO:0008199_ferric+iron+binding	0.354435	0.110441	0.83418
BP_GO:0006996_organelle+organization	0.513652	0.150461	0.968482
MF_GO:0016307_phosphatidylinositol+phosphate+kinase+activity	0.384233	0.293247	0.638136
BP_GO:0006940_regulation+of+smooth+muscle+contraction	0.0636941	0.0488779	0.259677
BP_GO:0008637_apoptotic+mitochondrial+changes	0.0397631	0.0734738	0.160397
BP_GO:0016079_synaptic+vesicle+exocytosis	0.418369	0.182326	0.730892
BP_GO:0009267_cellular+response+to+starvation	0.84752	0.682108	0.835632
BP_GO:0050884_neuromuscular+process+controlling+posture	0.380847	0.374486	0.47935
MF_GO:0042277_peptide+binding	0.328419	0.516407	0.276442
BP_GO:0007188_G-protein+signaling,+coupled+to+cAMP+nucleotide+secon	0.166403	0.390586	0.137663
BP_GO:0006779_porphyrin+biosynthetic+process	0.0508387	0.0119855	0.738005
BP_GO:0006939_smooth+muscle+contraction	0.999692	0.993896	0.990208
BP_GO:0042100_B+cell+proliferation	0.908057	0.551274	0.943023
MF_GO:0016918_retinal+binding	0.839892	0.62954	0.884076
BP_GO:0044262_cellular+carbohydrate+metabolic+process	0.0406407	0.0358703	0.434336
BP_GO:0021702_cerebellar+Purkinje+cell+differentiation	0.998388	0.871168	0.997798
BP_GO:0042554_superoxide+release	0.232484	0.448608	0.1526
MF_GO:0015377_cation:chloride+symporter+activity	0.812324	0.328714	0.955128
MF_GO:0008536_Ran+GTPase+binding	0.660768	0.992374	0.000375208
CC_GO:0016581_NuRD+complex	0.896726	0.205338	0.995451
BP_GO:0051605_protein+maturation+via+proteolysis	0.670055	0.472522	0.708168
BP_GO:0060216_definitive+hemopoiesis	0.917379	0.919245	0.748568
BP_GO:0000302_response+to+reactive+oxygen+species	0.259669	0.50247	0.0971794
BP_GO:0001841_neural+tube+formation	0.272362	0.370887	0.304914
CC_GO:0031966_mitochondrial+membrane	0.0140938	0.00238922	0.89561
BP_GO:0050770_regulation+of+axonogenesis	0.754662	0.430175	0.843285
MF_GO:0048306_calcium-dependent+protein+binding	0.387339	0.660852	0.158141
BP_GO:0035136_forelimb+morphogenesis	0.733526	0.821516	0.25065
BP_GO:0019800_peptide+cross-linking+via+chondroitin+4-sulfate+glycosam	0.0904094	0.370048	0.059428
MF_GO:0008889_glycerophosphodiester+phosphodiesterase+activity	0.725822	0.590224	0.712701
BP_GO:0046907_intracellular+transport	0.545241	0.0158936	0.964671
BP_GO:0007193_G-protein+signaling,+adenylate+cyclase+inhibiting+pathwa	0.934488	0.728658	0.924772
BP_GO:0007625_grooming+behavior	0.674293	0.685558	0.545311
BP_GO:0007130_synaptonemal+complex+assembly	0.34217	0.334265	0.455977
MF_GO:0019894_kinesin+binding	0.820584	0.942094	0.297416
BP_GO:0021953_central+nervous+system+neuron+differentiation	0.373024	0.0575884	0.791443
BP_GO:0006626_protein+targeting+to+mitochondrion	0.547804	0.0682211	0.951462
BP_GO:0009755_hormone-mediated+signaling	0.876866	0.25943	0.975362
CC_GO:0005815_microtubule+organizing+center	0.82966	0.722064	0.786692

CC_GO:0031519_PcG+protein+complex	0.0655391	0.00610803	0.513088
CC_GO:0009925_basal+plasma+membrane	0.909213	0.846907	0.806559
BP_GO:0021537_telencephalon+development	0.514789	0.627627	0.359658
BP_GO:0007191_dopamine+receptor,+adenylate+cyclase+activating+pathwa	0.466641	0.77638	0.189942
BP_GO:0042325_regulation+of+phosphorylation	0.703593	0.188139	0.913402
BP_GO:0001659_temperature+homeostasis	0.817988	0.45086	0.868527
CC_GO:0005801_cis-Golgi+network	0.302765	0.1265	0.736526
BP_GO:0032729_positive+regulation+of+interferon-gamma+production	0.667808	0.637635	0.603093
BP_GO:0006983_ER+overload+response	0.988579	0.621605	0.992834
BP_GO:0008340_determination+of+adult+life+span	0.487045	0.202788	0.610349
BP_GO:0030850_prostate+gland+development	0.0709038	0.076785	0.226147
BP_GO:0002027_regulation+of+heart+rate	0.674554	0.528889	0.714778
BP_GO:0060113_inner+ear+receptor+cell+differentiation	0.62755	0.296127	0.94263
MF_GO:0047555_3',5'-cyclic-GMP+phosphodiesterase+activity	0.599466	0.399494	0.669382
BP_GO:0021904_dorsoventral+neural+tube+patterning	0.882332	0.567205	0.914597
MF_GO:0004945_angiotensin+type+II+receptor+activity	0.595936	0.178949	0.791538
BP_GO:0008361_regulation+of+cell+size	0.953905	0.845316	0.910168
MF_GO:0005246_calcium+channel+regulator+activity	0.648128	0.801094	0.319047
BP_GO:0045176_apical+protein+localization	0.0511037	0.0685189	0.152141
CC_GO:0005763_mitochondrial+small+ribosomal+subunit	0.0195721	0.0441915	0.120646
BP_GO:0001655_urogenital+system+development	0.276239	0.384458	0.292658
MF_GO:0008601_protein+phosphatase+type+2A+regulator+activity	0.594039	0.861381	0.226638
MF_GO:0042393_histone+binding	0.939689	0.940372	0.657361
BP_GO:0030432_peristalsis	0.976235	0.985642	0.730308
BP_GO:0009617_response+to+bacterium	0.905217	0.83871	0.806579
MF_GO:0008094_DNA-dependent+ATPase+activity	0.599763	0.437702	0.648973
BP_GO:0007212_dopamine+receptor+signaling+pathway	0.718184	0.290365	0.914466
CC_GO:0000228_nuclear+chromosome	0.0444674	0.368439	0.0193132
BP_GO:0045471_response+to+ethanol	0.836058	0.991786	0.267029
BP_GO:0007129_synapsis	0.960314	0.899424	0.900927
BP_GO:0048168_regulation+of+neuronal+synaptic+plasticity	0.339737	0.716408	0.123845
MF_GO:0008158_hedgehog+receptor+activity	0.946172	0.885415	0.864919
CC_GO:0000790_nuclear+chromatin	0.758576	0.579862	0.785406
BP_GO:0042220_response+to+cocaine	0.936688	0.945664	0.754485
CC_GO:0005677_chromatin+silencing+complex	0.972055	0.894582	0.981862
CC_GO:0043209_myelin+sheath	0.543834	0.372229	0.685034
BP_GO:0048240_sperm+capacitation	0.151102	0.339885	0.162436
BP_GO:0006032_chitin+catabolic+process	0.140597	0.393919	0.124274
MF_GO:0004568_chitinase+activity	0.140597	0.393919	0.124274
BP_GO:0006611_protein+export+from+nucleus	0.968449	0.863578	0.965291
BP_GO:0042074_cell+migration+involved+in+gastrulation	0.79492	0.591546	0.852094
BP_GO:0007435_salivary+gland+morphogenesis	0.1142	0.287885	0.138032
BP_GO:0042438_melanin+biosynthetic+process	0.473539	0.246471	0.574838
BP_GO:0016579_protein+deubiquitination	0.0572474	0.378455	0.0273588
BP_GO:0015991_ATP+hydrolysis+coupled+proton+transport	0.531598	0.626309	0.265436
BP_GO:0016446_somatic+hypermutation+of+immunoglobulin+genes	0.112481	0.605519	0.059933
MF_GO:0050699_WW+domain+binding	0.569539	0.491777	0.605843

BP_GO:0001539_ciliary+or+flagellar+motility	0.867829	0.768766	0.800164
MF_GO:0004522_pancreatic+ribonuclease+activity	0.995711	0.997226	0.538298
BP_GO:0007140_male+meiosis	0.768741	0.6971	0.701707
BP_GO:0045109_intermediate+filament+organization	0.139355	0.0808258	0.32937
BP_GO:0007131_reciprocal+meiotic+recombination	0.56808	0.457735	0.602369
MF_GO:0005248_voltage-gated+sodium+channel+activity	0.946399	0.88499	0.859237
CC_GO:0022625_cytosolic+large+ribosomal+subunit	0.136783	0.11206	0.547743
CC_GO:0005885_Arp2/3+protein+complex	0.0653852	0.113722	0.180602
MF_GO:0016810_hydrolase+activity,+acting+on+carbon-nitrogen+(but+not+	0.781551	0.744683	0.665532
BP_GO:0042311_vasodilation	0.34991	0.204158	0.526874
BP_GO:0006910_phagocytosis,+recognition	0.348093	0.395874	0.385529
BP_GO:0051259_protein+oligomerization	0.73628	0.252625	0.921164
BP_GO:0042692_muscle+cell+differentiation	0.767851	0.469292	0.849577
CC_GO:0046581_intercellular+canaliculus	0.965042	0.988256	0.761271
MF_GO:0004029_aldehyde+dehydrogenase+(NAD)+activity	0.263707	0.15821	0.576177
BP_GO:0006013_mannose+metabolic+process	0.791911	0.650883	0.786364
BP_GO:0008206_bile+acid+metabolic+process	0.632526	0.698788	0.528294
MF_GO:0008508_bile+acid:sodium+symporter+activity	0.513313	0.565637	0.460647
BP_GO:0006656_phosphatidylcholine+biosynthetic+process	0.751691	0.492867	0.819814
BP_GO:0007520_myoblast+fusion	0.7741	0.731028	0.684905
MF_GO:0004365_glyceraldehyde-3-phosphate+dehydrogenase+(phosphoryl	0.985427	0.771166	0.979942
MF_GO:0008943_glyceraldehyde-3-phosphate+dehydrogenase+activity	0.985427	0.771166	0.979942
BP_GO:0045785_positive+regulation+of+cell+adhesion	0.431709	0.262295	0.626999
BP_GO:0030866_cortical+actin+cytoskeleton+organization	0.739649	0.74829	0.606384
BP_GO:0007044_cell-substrate+junction+assembly	0.606987	0.446964	0.763737
BP_GO:0010259_multicellular+organismal+aging	0.912155	0.988568	0.143571
BP_GO:0008277_regulation+of+G-protein+coupled+receptor+protein+signal	0.0281327	0.0781487	0.0576129
BP_GO:0009086_methionine+biosynthetic+process	0.338529	0.56889	0.241706
BP_GO:0046034_ATP+metabolic+process	0.564097	0.48775	0.63699
BP_GO:0030148_sphingolipid+biosynthetic+process	0.330327	0.762061	0.115488
MF_GO:0005100_Rho+GTPase+activator+activity	0.97125	0.921681	0.900601
MF_GO:0004630_phospholipase+D+activity	0.388059	0.644	0.243342
BP_GO:0006108_malate+metabolic+process	0.00459556	0.11029	0.00520357
BP_GO:0043353_enucleate+erythrocyte+differentiation	0.845002	0.986774	0.155707
BP_GO:0006044_N-acetylglucosamine+metabolic+process	0.563821	0.329804	0.680466
BP_GO:0006805_xenobiotic+metabolic+process	0.0765045	0.0100421	0.692406
BP_GO:0030225_macrophage+differentiation	0.20002	0.284321	0.265556
BP_GO:0006672_ceramide+metabolic+process	0.0113471	0.117447	0.01757
BP_GO:0042310_vasoconstriction	0.590974	0.337648	0.704516
BP_GO:0007250_activation+of+NF-kappaB-inducing+kinase+activity	0.680157	0.710563	0.499322
BP_GO:0006729_tetrahydrobiopterin+biosynthetic+process	0.47962	0.337279	0.81393
BP_GO:0050772_positive+regulation+of+axonogenesis	0.508155	0.180126	0.931105
BP_GO:0016575_histone+deacetylation	0.782651	0.929431	0.306398
MF_GO:0051920_peroxiredoxin+activity	0.459307	0.0761071	0.980847
BP_GO:0007270_nerve-nerve+synaptic+transmission	0.94245	0.420462	0.976766
BP_GO:0019222_regulation+of+metabolic+process	0.879432	0.528094	0.956205
MF_GO:0005545_phosphatidylinositol+binding	0.810922	0.771554	0.716282

MF_GO:0017153_sodium:dicarboxylate+symporter+activity	0.96375	0.978173	0.567292
BP_GO:0006750_glutathione+biosynthetic+process	0.736256	0.608324	0.724933
BP_GO:0002011_morphogenesis+of+an+epithelial+sheet	0.981145	0.959649	0.869588
BP_GO:0006546_glycine+catabolic+process	0.489724	0.213253	0.886587
MF_GO:0045296_cadherin+binding	0.780949	0.904402	0.457543
MF_GO:0005501_retinoid+binding	0.940196	0.940622	0.61643
BP_GO:0030838_positive+regulation+of+actin+filament+polymerization	0.822072	0.763626	0.725465
BP_GO:0045843_negative+regulation+of+striated+muscle+development	0.730003	0.765758	0.539372
MF_GO:0003956_NAD(P)+-protein-arginine+ADP-ribosyltransferase+activity	0.2714	0.357671	0.287414
CC_GO:0045261_proton-transporting+ATP+synthase+complex,+catalytic+co	0.409653	0.216743	0.905371
BP_GO:0048147_negative+regulation+of+fibroblast+proliferation	0.376364	0.149523	0.685518
BP_GO:0017157_regulation+of+exocytosis	0.103231	0.147667	0.235704
BP_GO:0046328_regulation+of+JNK+cascade	0.0983324	0.0983324	#N/A
BP_GO:0032755_positive+regulation+of+interleukin-6+production	0.683259	0.0916959	0.920172
BP_GO:0021756_striatum+development	0.543211	0.423801	0.650916
BP_GO:0048854_brain+morphogenesis	0.145857	0.199598	0.262383
BP_GO:0006584_catecholamine+metabolic+process	0.539796	0.742412	0.274746
MF_GO:0031177_phosphopantetheine+binding	0.845818	0.997829	0.0411508
MF_GO:0008528_peptide+receptor+activity,+G-protein+coupled	0.617926	0.848402	0.232591
BP_GO:0001963_synaptic+transmission,+dopaminergic	0.709734	0.828245	0.521678
MF_GO:0017075_syntaxin-1+binding	0.545064	0.782255	0.422148
BP_GO:0002064_epithelial+cell+development	0.0568627	0.218023	0.0783066
BP_GO:0007413_axonal+fasciculation	0.811789	0.678928	0.792186
BP_GO:0006098_pentose-phosphate+shunt	0.69122	0.69122	#N/A
BP_GO:0007271_synaptic+transmission,+cholinergic	0.553112	0.199111	0.755786
MF_GO:0004931_ATP-gated+cation+channel+activity	0.184643	0.147502	0.435912
MF_GO:0015385_sodium:hydrogen+antiporter+activity	0.790076	0.788395	0.620636
BP_GO:0048070_regulation+of+pigmentation+during+development	0.830665	0.230139	0.971611
BP_GO:0045410_positive+regulation+of+interleukin-6+biosynthetic+process	0.547356	0.902682	0.0941865
BP_GO:0031032_actomyosin+structure+organization	0.211843	0.0631452	0.706777
BP_GO:0045084_positive+regulation+of+interleukin-12+biosynthetic+proces	0.878492	0.933315	0.666869
BP_GO:0009416_response+to+light+stimulus	0.629288	0.58955	0.59501
MF_GO:0004622_lysophospholipase+activity	0.0348267	0.104397	0.0951098
BP_GO:0060088_auditory+receptor+cell+stereocilium+organization	0.982937	0.942792	0.924245
BP_GO:0008625_induction+of+apoptosis+via+death+domain+receptors	0.444884	0.888127	0.0145888
BP_GO:0002063_chondrocyte+development	0.419068	0.318339	0.522923
BP_GO:0043281_regulation+of+caspase+activity	0.108848	0.229881	0.128057
BP_GO:0009314_response+to+radiation	0.825862	0.630312	0.82953
BP_GO:0042551_neuron+maturation	0.588839	0.510255	0.647802
CC_GO:0000780_condensed+nuclear+chromosome,+centromeric+region	0.992677	0.995311	0.766508
BP_GO:0030279_negative+regulation+of+ossification	0.765806	0.781279	0.642993
MF_GO:0008440_inositol+trisphosphate+3-kinase+activity	0.495805	0.617067	0.22486
BP_GO:0035239_tube+morphogenesis	0.493701	0.435891	0.564537
BP_GO:0006817_phosphate+transport	0.614803	0.493223	0.679183
BP_GO:0002360_T+cell+lineage+commitment	0.982679	0.513157	0.988341
MF_GO:0005160_transforming+growth+factor+beta+receptor+binding	0.887195	0.927975	0.564955
BP_GO:0006924_activated+T+cell+apoptosis	0.131083	0.335156	0.132703

MF_GO:0015077_monovalent+inorganic+cation+transmembrane+transport	0.616797	0.537417	0.658049
MF_GO:0001609_adenosine+receptor+activity,+G-protein+coupled	0.754065	0.515882	0.809107
BP_GO:0045880_positive+regulation+of+smoothened+signaling+pathway	0.7038	0.470451	0.94476
BP_GO:0040011_locomotion	0.899357	0.571331	0.982343
MF_GO:0005041_low-density+lipoprotein+receptor+activity	0.896957	0.838186	0.793091
CC_GO:0016328_lateral+plasma+membrane	0.419135	0.41023	0.480336
BP_GO:0010033_response+to+organic+substance	0.89541	0.932506	0.576033
BP_GO:0043249_erythrocyte+maturation	0.599028	0.601526	0.534318
BP_GO:0014059_regulation+of+dopamine+secretion	0.511909	0.935255	0.0441491
MF_GO:0003727_single-stranded+RNA+binding	0.5805	0.232774	0.69773
MF_GO:0003678_DNA+helicase+activity	0.813647	0.826737	0.641904
BP_GO:0006268_DNA+unwinding+during+replication	0.861827	0.742501	0.809432
BP_GO:0043491_protein+kinase+B+signaling+cascade	0.86869	0.699676	0.865641
BP_GO:0060080_regulation+of+inhibitory+postsynaptic+membrane+potenti	0.582109	0.795278	0.329902
BP_GO:0060037_pharyngeal+system+development	0.954606	0.903262	0.860117
MF_GO:0016165_lipoxygenase+activity	0.372913	0.228953	0.714749
BP_GO:0043488_regulation+of+mRNA+stability	0.718474	0.619324	0.702801
CC_GO:0000800_lateral+element	0.458844	0.565849	0.41021
MF_GO:0016500_protein-hormone+receptor+activity	0.628023	0.536431	0.637923
MF_GO:0000149_SNARE+binding	0.203862	0.0712698	0.56973
MF_GO:0004579_dolichyl-diphosphooligosaccharide-protein+glycotransfera	0.492351	0.368759	0.689348
BP_GO:0006349_genetic+imprinting	0.314001	0.550692	0.187674
BP_GO:0008585_female+gonad+development	0.700475	0.228163	0.86293
MF_GO:0004767_sphingomyelin+phosphodiesterase+activity	0.535668	0.149553	0.845671
CC_GO:0015030_Cajal+body	0.578594	0.339822	0.846942
MF_GO:0015280_amiloride-sensitive+sodium+channel+activity	0.678167	0.849886	0.456895
BP_GO:0006303_double-strand+break+repair+via+nonhomologous+end+joi	0.194163	0.207638	0.306892
CC_GO:0001739_sex+chromatin	0.568483	0.529137	0.565537
BP_GO:0048304_positive+regulation+of+isotype+switching+to+IgG+isotypes	0.780899	#N/A	0.780899
CC_GO:0042105_alpha-beta+T+cell+receptor+complex	0.951831	0.913919	0.829222
MF_GO:0003906_DNA-(apurinic+or+apyrimidinic+site)+lyase+activity	0.53992	0.794106	0.213526
CC_GO:0000242_pericentriolar+material	0.51091	0.435947	0.569883
MF_GO:0017136_NAD-dependent+histone+deacetylase+activity	0.95374	0.831726	0.981862
BP_GO:0060134_prepulse+inhibition	0.912668	0.946812	0.78988
BP_GO:0007616_long-term+memory	0.529845	0.514851	0.528534
MF_GO:0008188_neuropeptide+receptor+activity	0.401872	0.991735	0.00809299
MF_GO:0003711_transcription+elongation+regulator+activity	0.990648	0.747895	0.997589
BP_GO:0009948_anterior/posterior+axis+specification	0.530465	0.267481	0.738506
MF_GO:0050733_RS+domain+binding	0.999747	0.821681	0.999928
CC_GO:0032391_photoreceptor+connecting+cilium	0.8594	0.880287	0.613019
BP_GO:0019229_regulation+of+vasoconstriction	0.66883	0.337145	0.826828
CC_GO:0031526_brush+border+membrane	0.510524	0.917714	0.121242
MF_GO:0004000_adenosine+deaminase+activity	0.888555	0.785724	0.828181
BP_GO:0007342_fusion+of+sperm+to+egg+plasma+membrane	0.241892	0.332306	0.289881
BP_GO:0042176_regulation+of+protein+catabolic+process	0.634057	0.48359	0.68755
BP_GO:0060136_embryonic+process+involved+in+female+pregnancy	0.561164	0.638141	0.393367
CC_GO:0005786_signal+recognition+particle,+endoplasmic+reticulum+targe	0.0855171	0.0220276	0.592953

BP_GO:0032259_methylation	0.0627046	0.260512	0.0707454
MF_GO:0016251_general+RNA+polymerase+II+transcription+factor+activity	0.0395663	0.00533213	0.45639
BP_GO:0032355_response+to+estradiol+stimulus	0.00366691	0.145885	0.00613417
BP_GO:0046620_regulation+of+organ+growth	0.646012	0.515852	0.700621
BP_GO:0045954_positive+regulation+of+natural+killer+cell+mediated+cytot	0.477111	0.424958	0.520751
BP_GO:0006559_L-phenylalanine+catabolic+process	0.444794	0.74264	0.198363
CC_GO:0005859_muscle+myosin+complex	0.45639	0.129851	0.74649
BP_GO:0042158_lipoprotein+biosynthetic+process	0.766187	0.297065	0.940594
CC_GO:0016012_sarcoglycan+complex	0.018823	0.102211	0.0475714
MF_GO:0008035_high-density+lipoprotein+binding	0.352151	0.352151	#N/A
BP_GO:0055003_cardiac+myofibril+assembly	0.0393977	0.0383405	0.237624
BP_GO:0006956_complement+activation	0.86718	0.96117	0.285278
BP_GO:0042574_retinal+metabolic+process	0.828543	0.817627	0.61643
MF_GO:0016868_intramolecular+transferase+activity,+phosphotransferases	0.614437	0.614437	#N/A
MF_GO:0005523_tropomyosin+binding	0.244374	0.713059	0.106515
BP_GO:0042953_lipoprotein+transport	0.976129	0.694979	0.980454
BP_GO:0002237_response+to+molecule+of+bacterial+origin	0.120149	0.35748	0.0977789
BP_GO:0006693_prostaglandin+metabolic+process	0.816075	0.992244	0.461288
BP_GO:0046513_ceramide+biosynthetic+process	0.584433	0.789583	0.307374
MF_GO:0043499_eukaryotic+cell+surface+binding	0.294442	0.834839	0.0884732
MF_GO:0030151_molybdenum+ion+binding	0.527592	0.476842	0.580109
BP_GO:0002021_response+to+dietary+excess	0.689006	0.159209	0.904855
BP_GO:0043406_positive+regulation+of+MAP+kinase+activity	0.646151	0.474131	0.693287
BP_GO:0060347_heart+trabecula+formation	0.786327	0.704497	0.730517
BP_GO:0030041_actin+filament+polymerization	0.767826	0.602828	0.780481
BP_GO:0010765_positive+regulation+of+sodium+ion+transport	0.364258	0.227157	0.794563
BP_GO:0032868_response+to+insulin+stimulus	0.482674	0.287821	0.69097
MF_GO:0003756_protein+disulfide+isomerase+activity	0.0874369	0.0743641	0.317968
MF_GO:0004115_3',5'-cyclic-AMP+phosphodiesterase+activity	0.83724	0.873506	0.534274
MF_GO:0017114_wide-spectrum+protease+inhibitor+activity	0.61061	0.607609	0.5736
BP_GO:0008015_blood+circulation	0.345117	0.313977	0.442393
BP_GO:0017015_regulation+of+transforming+growth+factor+beta+receptor	0.285408	0.50629	0.15793
BP_GO:0051604_protein+maturation	0.8091	0.529905	0.877263
MF_GO:0008308_voltage-gated+anion+channel+activity	0.0536387	0.0223993	0.706072
MF_GO:0016813_hydrolase+activity,+acting+on+carbon-nitrogen+(but+not+	0.223916	0.127154	0.526836
BP_GO:0048041_focal+adhesion+formation	0.81361	0.325497	0.956591
BP_GO:0050860_negative+regulation+of+T+cell+receptor+signaling+pathwa	0.793299	0.657852	0.762371
BP_GO:0030856_regulation+of+epithelial+cell+differentiation	0.611015	0.881404	0.216744
BP_GO:0015813_L-glutamate+transport	0.936738	0.974283	0.456749
MF_GO:0017154_semaphorin+receptor+activity	0.461312	0.487694	0.433149
MF_GO:0030246_carbohydrate+binding	0.452043	0.291461	0.714953
MF_GO:0048185_activin+binding	0.528266	0.281488	0.69014
BP_GO:0009312_oligosaccharide+biosynthetic+process	0.557661	0.711786	0.463702
BP_GO:0048009_insulin-like+growth+factor+receptor+signaling+pathway	0.180235	0.531624	0.0450876
BP_GO:0045445_myoblast+differentiation	0.634437	0.519655	0.700314
MF_GO:0016814_hydrolase+activity,+acting+on+carbon-nitrogen+(but+not+	0.89363	0.615883	0.906142
BP_GO:0007588_excretion	0.727036	0.92221	0.285598

MF_GO:0042605_peptide+antigen+binding	0.358026	0.748294	0.1184
BP_GO:0006882_cellular+zinc+ion+homeostasis	0.115954	0.145041	0.237301
CC_GO:0033017_sarcoplasmic+reticulum+membrane	0.75389	0.647999	0.797245
BP_GO:0050790_regulation+of+catalytic+activity	0.617228	0.402011	0.705704
CC_GO:0030130_clathrin+coat+of+trans-Golgi+network+vesicle	0.414232	0.119857	0.714541
BP_GO:0030098_lymphocyte+differentiation	0.660045	0.63584	0.602418
MF_GO:0001784_phosphotyrosine+binding	0.805032	0.598031	0.872284
MF_GO:0004047_aminomethyltransferase+activity	0.315737	0.213253	0.726725
BP_GO:0046902_regulation+of+mitochondrial+membrane+permeability	0.939488	0.410634	0.980224
MF_GO:0004571_mannosyl-oligosaccharide+1,2-alpha-mannosidase+activity	0.592892	0.531812	0.599701
CC_GO:0030496_midbody	0.746147	0.933756	0.400112
MF_GO:0005388_calcium-transporting+ATPase+activity	0.831872	0.46346	0.92663
BP_GO:0045885_positive+regulation+of+survival+gene+product+expression	0.745009	0.87245	0.418196
CC_GO:0000307_cyclin-dependent+protein+kinase+holoenzyme+complex	0.324163	0.517078	0.112951
MF_GO:0004396_hexokinase+activity	0.393605	0.47123	0.308967
MF_GO:0005154_epidermal+growth+factor+receptor+binding	0.932913	0.983778	0.33331
BP_GO:0006122_mitochondrial+electron+transport,+ubiquinol+to+cytochro	0.0493979	0.0228505	0.664557
CC_GO:0005954_calcium-+and+calmodulin-dependent+protein+kinase+com	0.89685	0.776649	0.866909
BP_GO:0045599_negative+regulation+of+fat+cell+differentiation	0.664983	0.965081	0.113056
BP_GO:0048048_embryonic+eye+morphogenesis	0.356505	0.620513	0.14232
BP_GO:0006790_sulfur+metabolic+process	0.427948	0.704765	0.273296
BP_GO:0045664_regulation+of+neuron+differentiation	0.650257	0.743323	0.50415
BP_GO:0016338_calcium-independent+cell-cell+adhesion	0.874793	0.448858	0.906009
BP_GO:0048103_somatic+stem+cell+division	0.134652	0.409769	0.0912603
BP_GO:0006353_transcription+termination	0.214792	0.189971	0.449983
BP_GO:0006970_response+to+osmotic+stress	0.0669182	0.0474189	0.525425
BP_GO:0031647_regulation+of+protein+stability	0.308424	0.179829	0.582803
CC_GO:0044444_cytoplasmic+part	0.428842	0.154548	0.777535
BP_GO:0030517_negative+regulation+of+axon+extension	0.828369	0.371391	0.918153
BP_GO:0022904_respiratory+electron+transport+chain	0.0657218	0.0259137	0.555021
MF_GO:0016847_1-aminocyclopropane-1-carboxylate+synthase+activity	0.596154	0.596154	#N/A
BP_GO:0009396_folic+acid+and+derivative+biosynthetic+process	0.087287	0.0722215	0.475568
MF_GO:0008331_high+voltage-gated+calcium+channel+activity	0.988791	0.870245	0.97714
BP_GO:0019953_sexual+reproduction	0.645414	0.660534	0.524194
BP_GO:0043388_positive+regulation+of+DNA+binding	0.976176	0.426938	0.994696
BP_GO:0006573_valine+metabolic+process	0.805256	0.805256	#N/A
MF_GO:0004540_ribonuclease+activity	0.438738	0.00425356	0.84362
BP_GO:0006072_glycerol-3-phosphate+metabolic+process	0.832309	0.47649	0.960009
BP_GO:0050930_induction+of+positive+chemotaxis	0.746336	0.7008	0.659347
BP_GO:0018149_peptide+cross-linking	0.561711	0.506442	0.564751
MF_GO:0003810_protein-glutamine+gamma-glutamyltransferase+activity	0.561711	0.506442	0.564751
BP_GO:0042743_hydrogen+peroxide+metabolic+process	0.279093	0.066531	0.866538
CC_GO:0005587_collagen+type+IV	0.323192	0.576992	0.142713
BP_GO:0008354_germ+cell+migration	0.43777	0.699304	0.228578
BP_GO:0048745_smooth+muscle+development	0.617408	0.891036	0.306514
BP_GO:0001578_microtubule+bundle+formation	0.263445	0.0843069	0.685284
BP_GO:0035121_tail+morphogenesis	0.217838	0.501724	0.196113

CC_GO:0033179_proton-transporting+V-type+ATPase,+V0+domain	0.888929	0.888929	#N/A
BP_GO:0045732_positive+regulation+of+protein+catabolic+process	0.741659	0.347168	0.904871
BP_GO:0051592_response+to+calcium+ion	0.729994	0.393314	0.826903
MF_GO:0016175_superoxide-generating+NADPH+oxidase+activity	0.269681	0.522633	0.126693
BP_GO:0051924_regulation+of+calcium+ion+transport	0.92611	0.908425	0.762698
MF_GO:0000062_acyl-CoA+binding	0.27251	0.286804	0.384953
BP_GO:0006461_protein+complex+assembly	0.676448	0.411047	0.808442
MF_GO:0008239_dipeptidyl-peptidase+activity	0.626879	0.675615	0.466734
MF_GO:0004128_cytochrome-b5+reductase+activity	0.536776	0.32887	0.716937
BP_GO:0043526_neuroprotection	0.290693	0.146384	0.607565
BP_GO:0045648_positive+regulation+of+erythrocyte+differentiation	0.953722	0.27498	0.982579
MF_GO:0004703_G-protein+coupled+receptor+kinase+activity	0.981052	0.620682	0.989952
MF_GO:0016407_acetyltransferase+activity	0.673157	#N/A	0.673157
BP_GO:0045995_regulation+of+embryonic+development	0.830315	0.930292	0.167674
MF_GO:0008046_axon+guidance+receptor+activity	0.537135	0.863751	0.253986
BP_GO:0043154_negative+regulation+of+caspase+activity	0.200066	0.200066	#N/A
BP_GO:0042415_norepinephrine+metabolic+process	0.543547	0.57151	0.474021
BP_GO:0007163_establishment+or+maintenance+of+cell+polarity	0.992313	0.997508	0.589094
BP_GO:0016925_protein+sumoylation	0.118394	0.293464	0.100415
MF_GO:0019789_SUMO+ligase+activity	0.118394	0.293464	0.100415
BP_GO:0000038_very-long-chain+fatty+acid+metabolic+process	0.68669	0.595514	0.742299
MF_GO:0017022_myosin+binding	0.54382	0.217377	0.825595
MF_GO:0001948_glycoprotein+binding	0.641473	0.598463	0.60377
MF_GO:0004659_prenyltransferase+activity	0.0691708	0.0911963	0.258852
BP_GO:0007406_negative+regulation+of+neuroblast+proliferation	0.852284	0.681972	0.828039
BP_GO:0042446_hormone+biosynthetic+process	0.510496	0.48159	0.525944
BP_GO:0002262_myeloid+cell+homeostasis	0.166638	0.161564	0.436528
BP_GO:0001776_leukocyte+homeostasis	0.21794	0.0396522	0.871208
BP_GO:0030203_glycosaminoglycan+metabolic+process	0.53159	0.640687	0.35502
BP_GO:0045039_protein+import+into+mitochondrial+inner+membrane	0.366546	0.0682211	0.935117
CC_GO:0042719_mitochondrial+intermembrane+space+protein+transporter	0.366546	0.0682211	0.935117
BP_GO:0050829_defense+response+to+Gram-negative+bacterium	0.142883	0.111676	0.266375
MF_GO:0016308_1-phosphatidylinositol-4-phosphate+5-kinase+activity	0.295255	0.181752	0.638136
BP_GO:0006221_pyrimidine+nucleotide+biosynthetic+process	0.290977	0.290977	#N/A
BP_GO:0007568_aging	0.127957	0.269734	0.135986
BP_GO:0060351_cartilage+development+involved+in+endochondral+bone+	0.740802	0.307355	0.966333
BP_GO:0007398_ectoderm+development	0.298468	0.263745	0.491306
MF_GO:0005112_Notch+binding	0.163916	0.270492	0.220206
BP_GO:0016188_synaptic+vesicle+maturation	0.231387	0.266403	0.34869
BP_GO:0045778_positive+regulation+of+ossification	0.576778	0.933987	0.20376
BP_GO:0006612_protein+targeting+to+membrane	0.231132	0.0952975	0.606415
MF_GO:0030374_ligand-dependent+nuclear+receptor+transcription+coactiv	0.475006	0.169643	0.807086
BP_GO:0048568_embryonic+organ+development	0.543708	0.436043	0.576317
BP_GO:0045598_regulation+of+fat+cell+differentiation	0.94544	0.674491	0.965046
BP_GO:0008343_adult+feeding+behavior	0.538646	0.241395	0.730894
BP_GO:0045670_regulation+of+osteoclast+differentiation	0.661418	0.406415	0.854397
BP_GO:0043583_ear+development	0.185304	0.0613784	0.89561

BP_GO:0045840_positive+regulation+of+mitosis	0.0358546	0.13633	0.0692682
MF_GO:0004977_melanocortin+receptor+activity	0.703107	#N/A	0.703107
BP_GO:0043278_response+to+morphine	0.790258	0.661351	0.76584
BP_GO:0006561_proline+biosynthetic+process	0.00124219	0.000504707	0.278302
BP_GO:0021846_cell+proliferation+in+forebrain	0.694683	0.572252	0.68962
BP_GO:0019933_cAMP-mediated+signaling	0.0807737	0.195479	0.13418
CC_GO:0000123_histone+acetyltransferase+complex	0.503066	0.402607	0.573025
BP_GO:0018279_protein+amino+acid+N-linked+glycosylation+via+asparagin	0.80818	0.76528	0.694374
BP_GO:0006406_mRNA+export+from+nucleus	0.446581	0.763497	0.182155
BP_GO:0060070_Wnt+receptor+signaling+pathway+through+beta-catenin	0.350946	0.419006	0.315823
CC_GO:0035097_histone+methyltransferase+complex	0.539068	0.297425	0.690246
CC_GO:0043195_terminal+button	0.872158	0.922542	0.573187
BP_GO:0015904_tetracycline+transport	0.887126	0.962993	0.334146
MF_GO:0015520_tetracycline:hydrogen+antiporter+activity	0.887126	0.962993	0.334146
MF_GO:0031202_RNA+splicing+factor+activity,+transesterification+mechan	0.871895	0.880937	0.664731
CC_GO:0033180_proton-transporting+V-type+ATPase,+V1+domain	0.408172	0.29314	0.643419
BP_GO:0015807_L-amino+acid+transport	0.505512	0.524301	0.483578
MF_GO:0015179_L-amino+acid+transmembrane+transporter+activity	0.505512	0.524301	0.483578
BP_GO:0007346_regulation+of+mitotic+cell+cycle	0.883691	0.895047	0.667713
MF_GO:0008408_3'-5'+exonuclease+activity	0.4171	0.130753	0.889738
BP_GO:0045429_positive+regulation+of+nitric+oxide+biosynthetic+process	0.663653	0.566433	0.666429
MF_GO:0004908_interleukin-1+receptor+activity	0.737286	0.949568	0.350837
BP_GO:0048667_cell+morphogenesis+involved+in+neuron+differentiation	0.814933	0.829709	0.580917
BP_GO:0035058_sensory+cilium+assembly	0.278712	0.250928	0.437454
BP_GO:0007617_mating+behavior	0.837867	0.9757	0.425396
CC_GO:0042101_T+cell+receptor+complex	0.758707	0.758707	#N/A
BP_GO:0060017_parathyroid+gland+development	0.935231	0.838939	0.875516
BP_GO:0040016_embryonic+cleavage	0.4247	0.209386	0.561275
BP_GO:0030111_regulation+of+Wnt+receptor+signaling+pathway	0.957134	0.703924	0.957745
BP_GO:0000278_mitotic+cell+cycle	0.450392	#N/A	0.450392
BP_GO:0033365_protein+localization+in+organelle	0.136463	0.0353471	0.474718
CC_GO:0016514_SWI/SNF+complex	0.942157	0.593355	0.96073
BP_GO:0032088_negative+regulation+of+NF-kappaB+transcription+factor+a	0.592656	0.287807	0.813627
BP_GO:0048599_oocyte+development	0.751694	0.02496	0.98678
BP_GO:0006998_nuclear+envelope+organization	0.0382214	0.0148934	0.698222
BP_GO:0048596_embryonic+camera-type+eye+morphogenesis	0.746677	0.961435	0.0886825
BP_GO:0006020_inositol+metabolic+process	0.174877	0.0845004	0.627851
BP_GO:0001763_morphogenesis+of+a+branching+structure	0.652141	0.402788	0.743186
BP_GO:0019227_action+potential+propagation	0.579027	0.709518	0.441896
BP_GO:0009266_response+to+temperature+stimulus	0.895544	0.688032	0.900793
BP_GO:0035019_somatic+stem+cell+maintenance	0.157058	0.5751	0.117575
CC_GO:0001939_female+pronucleus	0.375915	0.837323	0.215761
BP_GO:0009103_lipopolysaccharide+biosynthetic+process	0.869656	0.327368	0.95484
BP_GO:0040008_regulation+of+growth	0.513671	0.48844	0.530957
BP_GO:0006378_mRNA+polyadenylation	0.902586	0.85139	0.779493
MF_GO:0004415_hyalurononglucosaminidase+activity	0.144423	0.309961	0.15793
BP_GO:0001783_B+cell+apoptosis	0.336825	0.0711217	0.808521

CC_GO:0014704_intercalated+disc	0.971268	0.684005	0.986329
BP_GO:0044255_cellular+lipid+metabolic+process	0.0365429	0.0284059	0.448302
CC_GO:0009898_internal+side+of+plasma+membrane	0.0995738	0.171194	0.189493
BP_GO:0042490_mechanoreceptor+differentiation	0.509366	0.478722	0.534583
BP_GO:0021798_forebrain+dorsal/ventral+pattern+formation	0.229483	0.139765	0.443754
BP_GO:0006583_melanin+biosynthetic+process+from+tyrosine	0.804022	0.614158	0.790419
MF_GO:0043236_laminin+binding	0.0365346	0.00201121	0.832489
BP_GO:0048305_immunoglobulin+secretion	0.801421	0.833722	0.689232
MF_GO:0016799_hydrolase+activity,+hydrolyzing+N-glycosyl+compounds	0.0264023	0.810846	0.00137623
MF_GO:0031683_G-protein+beta/gamma-subunit+binding	0.639652	0.797572	0.288298
BP_GO:0016447_somatic+recombination+of+immunoglobulin+gene+segme	0.30862	0.605519	0.211549
CC_GO:0005833_hemoglobin+complex	0.725391	0.746865	0.572273
MF_GO:0051219_phosphoprotein+binding	0.722409	0.827661	0.455908
CC_GO:0000502_proteasome+complex	0.578982	0.623461	0.460381
MF_GO:0051378_serotonin+binding	0.813946	0.886492	0.594178
MF_GO:0031490_chromatin+DNA+binding	0.495954	0.515306	0.471496
MF_GO:0051087_chaperone+binding	0.370224	0.0647583	0.852688
CC_GO:0005858_axonemal+dynein+complex	0.767745	0.758295	0.630542
BP_GO:0046888_negative+regulation+of+hormone+secretion	0.804904	0.444099	0.912353
MF_GO:0008641_small+protein+activating+enzyme+activity	0.574629	0.899984	0.0687224
BP_GO:0021984_adenohypophysis+development	0.830225	#N/A	0.830225
BP_GO:0050966_detection+of+mechanical+stimulus+involved+in+sensory+p	0.973895	0.999112	0.351581
MF_GO:0042043_neurexin+binding	0.452581	0.325228	0.569423
CC_GO:0016471_vacuolar+proton-transporting+V-type+ATPase+complex	0.676132	0.713134	0.498346
CC_GO:0010369_chromocenter	0.987932	0.990698	0.717804
BP_GO:0007128_meiotic+prophase+I	0.43556	0.746812	0.111284
MF_GO:0004972_N-methyl-D-aspartate+selective+glutamate+receptor+acti	0.773376	0.996279	0.164785
BP_GO:0006614_SRP-dependent+cotranslational+protein+targeting+to+me	0.0707826	0.0103698	0.592953
BP_GO:0007341_penetration+of+zona+pellucida	0.346302	0.710764	0.190269
BP_GO:0021799_cerebral+cortex+radially+oriented+cell+migration	0.387361	0.0997748	0.810174
CC_GO:0005732_small+nucleolar+ribonucleoprotein+complex	0.960205	0.715746	0.971799
BP_GO:0032438_melanosome+organization	0.580543	0.400506	0.760632
BP_GO:0042596_fear+response	0.491686	0.603003	0.416812
CC_GO:0001518_voltage-gated+sodium+channel+complex	0.959466	0.863424	0.914697
BP_GO:0006325_establishment+or+maintenance+of+chromatin+architectur	0.532562	0.393689	0.699491
BP_GO:0043039_tRNA+aminoacylation	0.524789	0.335757	0.864231
MF_GO:0016876_ligase+activity,+forming+aminoacyl-tRNA+and+related+co	0.524789	0.335757	0.864231
BP_GO:0045104_intermediate+filament+cytoskeleton+organization	0.682686	0.950202	0.164818
BP_GO:0021675_nerve+development	0.632629	0.464191	0.715338
BP_GO:0060013_righting+reflex	0.869809	0.72062	0.842892
CC_GO:0022627_cytosolic+small+ribosomal+subunit	0.704017	0.704017	#N/A
CC_GO:0005883_neurofilament	0.655862	0.880436	0.166198
MF_GO:0000175_3'-5'-exoribonuclease+activity	0.0118685	0.00610776	0.355209
CC_GO:0005579_membrane+attack+complex	0.386488	0.528726	0.293295
BP_GO:0019433_triacylglycerol+catabolic+process	0.534877	0.378171	0.792677
BP_GO:0006572_tyrosine+catabolic+process	0.588386	0.74264	0.328363
CC_GO:0031430_M+band	0.142624	0.0500753	0.485651

BP_GO:0045577_regulation+of+B+cell+differentiation	0.749003	0.861116	0.394418
BP_GO:0032024_positive+regulation+of+insulin+secretion	0.50255	0.50255	#N/A
BP_GO:0030212_hyaluronan+metabolic+process	0.982751	0.999813	0.429464
MF_GO:0004013_adenosylhomocysteinase+activity	0.213816	0.0595719	0.743743
MF_GO:0004033_aldo-keto+reductase+activity	0.813496	0.0338702	0.999866
BP_GO:0031103_axon+regeneration	0.641645	0.11763	0.97866
BP_GO:0046835_carbohydrate+phosphorylation	0.514409	0.519105	0.494132
BP_GO:0019530_taurine+metabolic+process	0.749829	0.88638	0.339702
MF_GO:0016597_amino+acid+binding	0.332827	0.863318	0.0732439
MF_GO:0004032_aldehyde+reductase+activity	0.324992	0.228537	0.681769
BP_GO:0030239_myofibril+assembly	0.000500808	0.00235224	0.0445389
CC_GO:0043034_costamere	0.955042	0.641784	0.970757
CC_GO:0030055_cell-substrate+junction	0.375763	0.329394	0.56963
BP_GO:0018106_peptidyl-histidine+phosphorylation	0.340548	0.340548	#N/A
MF_GO:0004673_protein+histidine+kinase+activity	0.340548	0.340548	#N/A
MF_GO:0004768_stearoyl-CoA+9-desaturase+activity	0.48806	0.555697	0.36453
MF_GO:0050291_sphingosine+N-acyltransferase+activity	0.392783	0.746328	0.186096
BP_GO:0002028_regulation+of+sodium+ion+transport	0.643842	0.288345	0.824336
MF_GO:0019864_IgG+binding	0.811703	0.767747	0.70673
BP_GO:0015937_coenzyme+A+biosynthetic+process	0.179209	0.146609	0.519628
CC_GO:0005916_fascia+adherens	0.672324	0.241789	0.941003
BP_GO:0050729_positive+regulation+of+inflammatory+response	0.979346	0.959059	0.863018
BP_GO:0006107_oxaloacetate+metabolic+process	0.737721	0.737721	#N/A
BP_GO:0042168_heme+metabolic+process	0.698759	0.559935	0.73845
BP_GO:0043086_negative+regulation+of+catalytic+activity	0.216277	0.27411	0.300766
BP_GO:0051272_positive+regulation+of+cell+motion	0.418153	0.339445	0.528577
BP_GO:0007029_endoplasmic+reticulum+organization	0.529508	0.305774	0.770065
BP_GO:0009168_purine+ribonucleoside+monophosphate+biosynthetic+pro	0.928525	0.929488	0.754406
MF_GO:0019239_deaminase+activity	0.928525	0.929488	0.754406
BP_GO:0008631_induction+of+apoptosis+by+oxidative+stress	0.273448	0.407919	0.189489
BP_GO:0043552_positive+regulation+of+phosphoinositide+3-kinase+activity	0.828682	0.938311	0.614009
BP_GO:0042759_long-chain+fatty+acid+biosynthetic+process	0.516733	0.562956	0.449288
BP_GO:0006004_fucose+metabolic+process	0.720634	0.769483	0.433872
BP_GO:0019395_fatty+acid+oxidation	0.0381125	0.276136	0.0190642
MF_GO:0016776_phosphotransferase+activity,+phosphate+group+as+accep	0.399288	0.374364	0.528043
MF_GO:0019201_nucleotide+kinase+activity	0.399288	0.374364	0.528043
MF_GO:0001968_fibronectin+binding	0.19669	0.405762	0.182346
BP_GO:0045588_positive+regulation+of+gamma-delta+T+cell+differentiatio	0.929444	0.755986	0.9301
BP_GO:0030177_positive+regulation+of+Wnt+receptor+signaling+pathway	0.423174	0.505361	0.397113
BP_GO:0051091_positive+regulation+of+transcription+factor+activity	0.7579	0.86923	0.494414
BP_GO:0016202_regulation+of+striated+muscle+development	0.650338	0.403434	0.757512
MF_GO:0000146_microfilament+motor+activity	0.0340965	0.0477187	0.229218
MF_GO:0005522_profilin+binding	0.288382	0.320188	0.367505
MF_GO:0004559_alpha-mannosidase+activity	0.728112	0.530067	0.807076
BP_GO:0008088_axon+cargo+transport	0.901384	0.82415	0.846023
MF_GO:0047760_butyrate-CoA+ligase+activity	0.928675	0.707337	0.950381
MF_GO:0004499_flavin-containing+monooxygenase+activity	0.881566	0.700576	0.855224

BP_GO:0006012_galactose+metabolic+process	0.254215	0.256671	0.432253
BP_GO:0030278_regulation+of+ossification	0.380225	0.618347	0.261348
BP_GO:0043408_regulation+of+MAPKKK+cascade	0.449336	0.293035	0.610507
CC_GO:0000145_exocyst	0.369429	0.578849	0.276765
MF_GO:0003857_3-hydroxyacyl-CoA+dehydrogenase+activity	0.192411	0.192411	#N/A
BP_GO:0048011_nerve+growth+factor+receptor+signaling+pathway	0.988706	0.785852	0.989163
MF_GO:0004679_AMP-activated+protein+kinase+activity	0.521072	0.36626	0.635915
MF_GO:0003847_1-alkyl-2-acetylglycerophosphocholine+esterase+activity	0.71697	0.824524	0.569379
BP_GO:0000904_cell+morphogenesis+involved+in+differentiation	0.733006	0.496148	0.840075
MF_GO:0016504_protease+activator+activity	0.53074	0.207522	0.868656
BP_GO:0050873_brown+fat+cell+differentiation	0.196482	0.290762	0.210015
BP_GO:0006884_cell+volume+homeostasis	0.332721	0.170167	0.58744
BP_GO:0000070_mitotic+sister+chromatid+segregation	0.226217	0.78381	0.053627
MF_GO:0004565_beta-galactosidase+activity	0.825253	0.126943	0.9838
MF_GO:0004720_protein-lysine+6-oxidase+activity	0.677441	0.633718	0.621559
BP_GO:0032060_bleb+formation	0.16901	0.317409	0.198144
BP_GO:0045931_positive+regulation+of+mitotic+cell+cycle	0.96502	0.968856	0.626639
BP_GO:0030595_leukocyte+chemotaxis	0.979373	0.923158	0.929068
MF_GO:0008131_amine+oxidase+activity	0.770785	0.914117	0.308045
BP_GO:0007088_regulation+of+mitosis	0.163326	0.114272	0.469997
BP_GO:0008211_glucocorticoid+metabolic+process	0.560874	0.536113	0.552242
BP_GO:0051098_regulation+of+binding	0.833624	0.403434	0.92645
CC_GO:0000276_mitochondrial+proton-transporting+ATP+synthase+comple	0.139696	0.139696	#N/A
BP_GO:0008635_caspase+activation+via+cytochrome+c	0.060813	0.060813	#N/A
BP_GO:0045351_type+I+interferon+biosynthetic+process	0.572686	0.199386	0.734774
BP_GO:0045727_positive+regulation+of+translation	0.898415	0.756441	0.858792
BP_GO:0055074_calcium+ion+homeostasis	0.137685	0.0330225	0.537385
BP_GO:0050771_negative+regulation+of+axonogenesis	0.332174	0.293035	0.454224
MF_GO:0005066_transmembrane+receptor+protein+tyrosine+kinase+signal	0.616899	0.209358	0.988697
CC_GO:0005795_Golgi+stack	0.204013	0.13432	0.355305
MF_GO:0008093_cytoskeletal+adaptor+activity	0.886128	0.303922	0.945791
CC_GO:0005744_mitochondrial+inner+membrane+presequence+translocase	0.456922	0.101162	0.989443
BP_GO:0045792_negative+regulation+of+cell+size	0.917278	0.917278	#N/A
BP_GO:0048008_platelet-derived+growth+factor+receptor+signaling+pathw	0.147522	0.178272	0.309855
MF_GO:0008266_poly(U)+binding	0.591999	0.0777017	0.927973
MF_GO:0019899_enzyme+binding	0.515504	0.580747	0.453798
BP_GO:0051289_protein+homotetramerization	0.114887	0.198004	0.195429
BP_GO:0045080_positive+regulation+of+chemokine+biosynthetic+process	0.185653	0.181285	0.293388
CC_GO:0005606_laminin-1+complex	0.727143	0.876519	0.167674
BP_GO:0046579_positive+regulation+of+Ras+protein+signal+transduction	0.167711	0.213804	0.285685
BP_GO:0001832_blastocyst+growth	0.0206587	0.133733	0.0420733
BP_GO:0006308_DNA+catabolic+process	0.325975	0.25384	0.483725
MF_GO:0030276_clathrin+binding	0.385769	0.639856	0.252402
MF_GO:0008499_UDP-galactose:beta-N-acetylglucosamine+beta-1,3-galacto	0.714229	0.711786	0.637963
MF_GO:0030295_protein+kinase+activator+activity	0.0327293	0.104144	0.0853832
MF_GO:0004563_beta-N-acetylhexosaminidase+activity	0.460051	0.694284	0.107729
BP_GO:0014047_glutamate+secretion	0.827307	0.302181	0.98323

BP_GO:0045773_positive+regulation+of+axon+extension	0.0500847	0.00985446	0.601666
BP_GO:0048384_retinoic+acid+receptor+signaling+pathway	0.688562	0.802711	0.475889
BP_GO:0030511_positive+regulation+of+transforming+growth+factor+beta+	0.369784	0.114898	0.59081
MF_GO:0005498_sterol+carrier+activity	0.589455	0.294966	0.943189
BP_GO:0042756_drinking+behavior	0.987383	0.804886	0.98085
MF_GO:0042153_RPTP-like+protein+binding	0.878439	0.673859	0.872542
BP_GO:0022409_positive+regulation+of+cell-cell+adhesion	0.713162	0.813175	0.420742
BP_GO:0030433_ER-associated+protein+catabolic+process	0.150464	0.150464	#N/A
MF_GO:0004445_inositol-polyphosphate+5-phosphatase+activity	0.765538	0.527057	0.855765
BP_GO:0010552_positive+regulation+of+specific+transcription+from+RNA+p	0.222405	0.164893	0.595172
BP_GO:0007600_sensory+perception	0.491451	0.483293	0.50706
MF_GO:0003828_alpha-N-acetylneuraminate+alpha-2,8-sialyltransferase+ac	0.977092	0.954311	0.861882
BP_GO:0046548_retinal+rod+cell+development	0.848981	0.772647	0.782208
MF_GO:0004861_cyclin-dependent+protein+kinase+inhibitor+activity	0.557616	0.70986	0.327154
MF_GO:0035257_nuclear+hormone+receptor+binding	0.840843	0.927501	0.538914
BP_GO:0019430_removal+of+superoxide+radicals	0.428089	0.374568	0.541981
BP_GO:0002089_lens+morphogenesis+in+camera-type+eye	0.677018	0.701253	0.564267
BP_GO:0051384_response+to+glucocorticoid+stimulus	0.302976	0.45765	0.281419
MF_GO:0008467_[heparan+sulfate]-glucosamine+3-sulfotransferase+1+activ	0.160424	0.671992	0.0172737
MF_GO:0001515_opioid+peptide+activity	0.101306	0.19035	0.136995
BP_GO:0042088_T-helper+1+type+immune+response	0.943792	0.906823	0.813415
CC_GO:0001931_uropod	0.702278	0.715642	0.518923
BP_GO:0002467_germinal+center+formation	0.925847	0.805635	0.877325
CC_GO:0005851_eukaryotic+translation+initiation+factor+2B+complex	0.070602	0.070602	#N/A
BP_GO:0055015_ventricular+cardiac+muscle+cell+development	0.782542	0.94662	0.229393
BP_GO:0015701_bicarbonate+transport	0.284206	0.0525026	0.569039
BP_GO:0048644_muscle+morphogenesis	0.312741	0.312741	#N/A
BP_GO:0047496_vesicle+transport+along+microtubule	0.744845	0.803388	0.498067
BP_GO:0007127_meiosis+I	0.414406	0.593891	0.318161
BP_GO:0040020_regulation+of+meiosis	0.977349	0.977349	#N/A
MF_GO:0005173_stem+cell+factor+receptor+binding	0.875172	0.222143	0.95253
BP_GO:0009306_protein+secretion	0.772164	0.748454	0.640237
BP_GO:0060271_cilium+morphogenesis	0.305222	0.372038	0.313767
BP_GO:0048339_paraxial+mesoderm+development	0.226613	0.442859	0.156519
BP_GO:0021631_optic+nerve+morphogenesis	0.910715	0.903177	0.750003
CC_GO:0030659_cytoplasmic+vesicle+membrane	0.332598	0.169554	0.588041
MF_GO:0008517_folic+acid+transporter+activity	0.984781	0.892585	0.962478
BP_GO:0001779_natural+killer+cell+differentiation	0.807734	0.807734	#N/A
BP_GO:0009101_glycoprotein+biosynthetic+process	0.201067	0.0223686	0.527195
BP_GO:0048562_embryonic+organ+morphogenesis	0.0944882	0.399641	0.0899625
CC_GO:0044431_Golgi+apparatus+part	0.557381	0.466042	0.630306
BP_GO:0045787_positive+regulation+of+cell+cycle	0.905794	0.987385	0.448216
MF_GO:0004726_non-membrane+spanning+protein+tyrosine+phosphatase+	0.936031	0.957876	0.614707
BP_GO:0000245_spliceosome+assembly	0.330965	0.490714	0.316693
CC_GO:0030008_TRAPP+complex	0.294623	0.294623	#N/A
BP_GO:0033014_tetrapyrrole+biosynthetic+process	0.52656	0.0384488	0.937116
BP_GO:0048812_neurite+morphogenesis	0.225474	0.159335	0.621354

BP_GO:0043666_regulation+of+phosphoprotein+phosphatase+activity	0.593311	0.4883	0.721113
BP_GO:0006261_DNA-dependent+DNA+replication	0.360543	0.228286	0.755044
BP_GO:0009650_UV+protection	0.890268	0.540605	0.933402
BP_GO:0009620_response+to+fungus	0.802859	0.300211	0.976572
MF_GO:0008190_eukaryotic+initiation+factor+4E+binding	0.248127	0.0637751	0.9364
BP_GO:0006590_thyroid+hormone+generation	0.167402	0.219199	0.282682
CC_GO:0005662_DNA+replication+factor+A+complex	0.00913858	0.0057627	0.411817
CC_GO:0005869_dynactin+complex	0.125249	0.223643	0.147363
BP_GO:0048762_mesenchymal+cell+differentiation	0.117149	0.0476143	0.431738
BP_GO:0006346_methylation-dependent+chromatin+silencing	0.289534	0.311337	0.392125
BP_GO:0048699_generation+of+neurons	0.0968973	0.505282	0.0457525
CC_GO:0019031_viral+envelope	0.700476	0.438729	0.846088
BP_GO:0009083_branched+chain+family+amino+acid+catabolic+process	0.444045	0.404708	0.529184
MF_GO:0003704_specific+RNA+polymerase+II+transcription+factor+activity	0.224454	0.224454	#N/A
CC_GO:0019005_SCF+ubiquitin+ligase+complex	0.149844	0.00667186	0.752649
BP_GO:0008366_axon+ensheathment	0.725919	0.725041	0.585927
BP_GO:0050727_regulation+of+inflammatory+response	0.532345	0.431159	0.633393
BP_GO:0051225_spindle+assembly	0.962311	0.908584	0.880775
BP_GO:0000154_rRNA+modification	0.45847	0.820405	0.0990216
BP_GO:0050892_intestinal+absorption	0.109047	0.344547	0.119729
BP_GO:0048489_synaptic+vesicle+transport	0.057444	0.157298	0.103912
MF_GO:0017040_ceramidase+activity	0.119482	0.726791	0.0221072
BP_GO:0007379_segment+specification	0.218036	0.406252	0.208641
MF_GO:0004620_phospholipase+activity	0.101021	0.436929	0.0647004
BP_GO:0016574_histone+ubiquitination	0.509505	0.544451	0.475989
BP_GO:0050908_detection+of+light+stimulus+involved+in+visual+perceptio	0.775274	0.688408	0.71734
BP_GO:0048515_spermatid+differentiation	0.218243	0.223565	0.382681
BP_GO:0007030_Golgi+organization	0.641014	0.740074	0.476345
MF_GO:0042169_SH2+domain+binding	0.722118	0.657852	0.675523
MF_GO:0017016_Ras+GTPase+binding	0.589898	0.394901	0.753569
BP_GO:0045198_establishment+of+epithelial+cell+apical/basal+polarity	0.960709	0.934486	0.81905
BP_GO:0006415_translational+termination	0.49139	0.13609	0.807631
MF_GO:0003747_translation+release+factor+activity	0.49139	0.13609	0.807631
MF_GO:0002039_p53+binding	0.992742	0.997514	0.80522
BP_GO:0009792_embryonic+development+ending+in+birth+or+egg+hatchir	0.852924	#N/A	0.852924
BP_GO:0001957_intramembranous+ossification	0.702614	0.308807	0.863116
BP_GO:0002070_epithelial+cell+maturation	0.0663462	0.0196344	0.558997
MF_GO:0004960_thromboxane+receptor+activity	0.713114	0.168422	0.934548
CC_GO:0005637_nuclear+inner+membrane	0.556152	0.697937	0.340389
MF_GO:0031419_cobalamin+binding	0.632718	0.447089	0.672114
BP_GO:0021513_spinal+cord+dorsal/ventral+patterning	0.893222	0.458768	0.925407
MF_GO:0005487_nucleocytoplasmic+transporter+activity	0.757492	0.531736	0.798501
MF_GO:0035014_phosphoinositide+3-kinase+regulator+activity	0.999933	0.589179	0.999948
BP_GO:0045879_negative+regulation+of+smoothened+signaling+pathway	0.857458	0.788417	0.765945
MF_GO:0004708_MAP+kinase+kinase+activity	0.14288	0.229217	0.218109
BP_GO:0015012_heparan+sulfate+proteoglycan+biosynthetic+process	0.160164	0.177289	0.355843
MF_GO:0019863_IgE+binding	0.85943	0.824812	0.769594

BP_GO:0001101_response+to+acid	0.741212	0.475158	0.864113
BP_GO:0021516_dorsal+spinal+cord+development	0.382218	0.42763	0.401345
BP_GO:0046621_negative+regulation+of+organ+growth	0.0341529	0.167062	0.060065
BP_GO:0048541_Peyer's+patch+development	0.814735	0.779221	0.681872
MF_GO:0031405_lipoic+acid+binding	0.737503	0.248799	0.966755
MF_GO:0017091_AU-rich+element+binding	0.164853	0.694276	0.089526
BP_GO:0006265_DNA+topological+change	0.86991	#N/A	0.86991
MF_GO:0003916_DNA+topoisomerase+activity	0.86991	#N/A	0.86991
MF_GO:0050220_prostaglandin-E+synthase+activity	0.359399	0.0590372	0.648003
BP_GO:0002328_pro-B+cell+differentiation	0.0724275	0.437468	0.0604722
BP_GO:0009725_response+to+hormone+stimulus	0.76163	0.847208	0.323673
BP_GO:0000288_nuclear-transcribed+mRNA+catabolic+process,+deadenylat	0.239071	0.275477	0.348151
BP_GO:0043576_regulation+of+respiratory+gaseous+exchange	0.479223	0.183651	0.652899
BP_GO:0030071_regulation+of+mitotic+metaphase/anaphase+transition	0.640996	0.261817	0.83821
BP_GO:0048341_paraxial+mesoderm+formation	0.636106	0.410042	0.692548
BP_GO:0019217_regulation+of+fatty+acid+metabolic+process	0.188401	0.292802	0.188286
BP_GO:0021680_cerebellar+Purkinje+cell+layer+development	0.830701	0.654618	0.847365
BP_GO:0018101_peptidyl-citrulline+biosynthetic+process+from+peptidyl-arg	0.646385	0.646385	#N/A
MF_GO:0004668_protein-arginine+deiminase+activity	0.646385	0.646385	#N/A
BP_GO:0009950_dorsal/ventral+axis+specification	0.126745	0.173304	0.257316
BP_GO:0046580_negative+regulation+of+Ras+protein+signal+transduction	0.211393	0.195102	0.369774
BP_GO:0030851_granulocyte+differentiation	0.97816	0.97816	#N/A
MF_GO:0042054_histone+methyltransferase+activity	0.584076	0.789647	0.350709
BP_GO:0043193_positive+regulation+of+gene-specific+transcription	0.247218	0.445503	0.220763
BP_GO:0050769_positive+regulation+of+neurogenesis	0.208518	#N/A	0.208518
MF_GO:0043021_ribonucleoprotein+binding	0.661747	0.0629457	0.994352
BP_GO:0007638_mechanosensory+behavior	0.119813	#N/A	0.119813
MF_GO:0016922_ligand-dependent+nuclear+receptor+binding	0.279528	0.257573	0.449872
BP_GO:0048485_sympathetic+nervous+system+development	0.129192	0.183651	0.208393
BP_GO:0046627_negative+regulation+of+insulin+receptor+signaling+pathwa	0.372041	0.15581	0.657386
BP_GO:0016226_iron-sulfur+cluster+assembly	0.0167256	0.0669354	0.0636853
BP_GO:0060042_retina+morphogenesis+in+camera-type+eye	0.487876	0.692703	0.387499
BP_GO:0032094_response+to+food	0.900188	0.915778	0.702154
MF_GO:0004947_bradykinin+receptor+activity	0.395774	0.192326	0.555609
BP_GO:0007004_telomere+maintenance+via+telomerase	0.460474	0.450183	0.498706
MF_GO:0016624_oxidoreductase+activity,+acting+on+the+aldehyde+or+oxo	0.494151	0.264611	0.889646
BP_GO:0045078_positive+regulation+of+interferon-gamma+biosynthetic+pr	0.659164	0.556173	0.660627
BP_GO:0042136_neurotransmitter+biosynthetic+process	0.630807	0.975292	0.120265
MF_GO:0034450_ubiquitin-ubiquitin+ligase+activity	0.121755	0.16013	0.268096
BP_GO:0060040_retinal+bipolar+neuron+differentiation	0.391783	0.421293	0.42385
BP_GO:0030307_positive+regulation+of+cell+growth	0.708039	0.474605	0.827312
BP_GO:0032331_negative+regulation+of+chondrocyte+differentiation	0.897361	0.911165	0.554131
MF_GO:0005000_vasopressin+receptor+activity	0.823641	0.671924	0.798388
MF_GO:0017176_phosphatidylinositol+N-acetylglucosaminyltransferase+act	0.875979	0.271522	0.976575
CC_GO:0001940_male+pronucleus	0.151814	0.837323	0.0502926
BP_GO:0006777_Mo-molybdopterin+cofactor+biosynthetic+process	0.275151	0.307045	0.372118
BP_GO:0030858_positive+regulation+of+epithelial+cell+differentiation	0.0505371	0.0308081	0.277854

MF_GO:0004981_muscarinic+acetylcholine+receptor+activity	0.863135	0.862501	0.698853
BP_GO:0006516_glycoprotein+catabolic+process	0.794904	0.593655	0.914061
BP_GO:0035095_behavioral+response+to+nicotine	0.860182	0.854617	0.702839
BP_GO:0007006_mitochondrial+membrane+organization	0.00364992	0.0511926	0.0125823
BP_GO:0046686_response+to+cadmium+ion	0.530482	0.743235	0.404638
CC_GO:0042589_zymogen+granule+membrane	0.798009	0.508327	0.855458
BP_GO:0042053_regulation+of+dopamine+metabolic+process	0.654962	0.854831	0.46679
CC_GO:0046540_U4/U6+x+U5+tri-snRNP+complex	0.785356	0.517003	0.953561
MF_GO:0004982_N-formyl+peptide+receptor+activity	0.319781	0.533687	0.250415
BP_GO:0032314_regulation+of+Rac+GTPase+activity	0.810124	0.859799	0.599527
BP_GO:0048665_neuron+fate+specification	0.85906	#N/A	0.85906
BP_GO:0042428_serotonin+metabolic+process	0.975073	0.866478	0.959083
CC_GO:0012505_endomembrane+system	0.449918	0.449918	#N/A
BP_GO:0010224_response+to+UV-B	0.554581	0.156399	0.841764
CC_GO:0000784_nuclear+chromosome,+telomeric+region	0.154996	0.0331648	0.575251
BP_GO:0042423_catecholamine+biosynthetic+process	0.914411	0.880269	0.76491
BP_GO:0000239_pachytene	0.272525	0.16313	0.597147
BP_GO:0007431_salivary+gland+development	0.449684	0.315009	0.751838
BP_GO:0014044_Schwann+cell+development	0.466961	0.504006	0.443134
BP_GO:0006685_sphingomyelin+catabolic+process	0.688424	0.399136	0.800415
BP_GO:0048715_negative+regulation+of+oligodendrocyte+differentiation	0.881821	0.748947	0.853025
BP_GO:0033151_V(D)J+recombination	0.171788	0.513157	0.141168
CC_GO:0030137_COPI-coated+vesicle	0.166848	0.579021	0.038257
BP_GO:0045638_negative+regulation+of+myeloid+cell+differentiation	0.335393	0.812793	0.0392982
BP_GO:0035094_response+to+nicotine	0.913313	0.854617	0.814422
BP_GO:0048484_enteric+nervous+system+development	0.61682	0.283329	0.879262
BP_GO:0021527_spinal+cord+association+neuron+differentiation	0.679875	0.909327	0.312652
BP_GO:0050678_regulation+of+epithelial+cell+proliferation	0.0252668	0.0252668	#N/A
BP_GO:0048709_oligodendrocyte+differentiation	0.983603	0.868144	0.977497
CC_GO:0044464_cell+part	0.652162	0.464233	0.718482
BP_GO:0060045_positive+regulation+of+cardiac+muscle+cell+proliferation	0.744253	0.845997	0.284286
BP_GO:0060135_maternal+process+involved+in+pregnancy	0.30875	0.383435	0.300597
BP_GO:0000012_single+strand+break+repair	0.762519	0.779675	0.522055
MF_GO:0004952_dopamine+receptor+activity	0.702706	0.814936	0.558233
MF_GO:0004826_phenylalanine-tRNA+ligase+activity	0.384526	0.561881	0.256446
BP_GO:0048703_embryonic+viscerocranium+morphogenesis	0.798059	0.794873	0.622079
BP_GO:0033138_positive+regulation+of+peptidyl-serine+phosphorylation	0.889278	0.681614	0.883453
BP_GO:0045776_negative+regulation+of+blood+pressure	0.843331	0.814936	0.751316
MF_GO:0004994_somatostatin+receptor+activity	0.878539	0.757284	0.839206
MF_GO:0001601_peptide+YY+receptor+activity	0.14455	0.570532	0.065141
MF_GO:0003831_beta-N-acetylglucosaminylglycopeptide+beta-1,4-galactos	0.361342	0.0598375	0.791956
CC_GO:0017146_N-methyl-D-aspartate+selective+glutamate+receptor+com	0.622689	0.986064	0.22628
BP_GO:0006354_RNA+elongation	0.60015	0.102035	0.974778
MF_GO:0004652_polynucleotide+adenylyltransferase+activity	0.181314	0.789949	0.0333933
BP_GO:0009253_peptidoglycan+catabolic+process	0.964075	0.72282	0.98302
BP_GO:0030819_positive+regulation+of+cAMP+biosynthetic+process	0.763973	0.699176	0.706352
BP_GO:0060038_cardiac+muscle+cell+proliferation	0.0497904	0.0443198	0.302489

BP_GO:0051023_regulation+of+immunoglobulin+secretion	0.496421	0.222742	0.821278
MF_GO:0030235_nitric-oxide+synthase+regulator+activity	0.676367	0.693423	0.541506
BP_GO:0045861_negative+regulation+of+proteolysis	0.91595	0.900698	0.766998
BP_GO:0046697_decidualization	0.204396	0.191347	0.406688
BP_GO:0042035_regulation+of+cytokine+biosynthetic+process	0.165715	0.00424853	0.814936
BP_GO:0031575_G1/S+transition+checkpoint	0.0581682	0.0227851	0.347037
CC_GO:0001740_Barr+body	0.536034	0.536034	#N/A
CC_GO:0005865_striated+muscle+thin+filament	0.505615	0.253366	0.88049
BP_GO:0030300_regulation+of+cholesterol+absorption	0.51411	0.330079	0.797276
BP_GO:0006526_arginine+biosynthetic+process	0.99662	0.994927	0.832082
MF_GO:0001758_retinal+dehydrogenase+activity	0.196891	0.435816	0.148347
BP_GO:0006597_spermine+biosynthetic+process	0.392676	#N/A	0.392676
CC_GO:0002102_podosome	0.0140795	0.00909277	0.229468
BP_GO:0006547_histidine+metabolic+process	0.770244	0.706206	0.692655
MF_GO:0004645_phosphorylase+activity	0.977201	0.977201	#N/A
BP_GO:0008295_spermidine+biosynthetic+process	0.563248	0.579265	0.510081
MF_GO:0004022_alcohol+dehydrogenase+activity	0.703905	0.53428	0.715276
MF_GO:0004470_malic+enzyme+activity	0.00939294	0.262428	0.0036244
BP_GO:0002639_positive+regulation+of+immunoglobulin+production	0.486654	0.486654	#N/A
BP_GO:0014823_response+to+activity	0.783291	0.599982	0.870132
CC_GO:0005826_contractile+ring	0.632452	0.294724	0.758851
BP_GO:0060047_heart+contraction	0.0342652	0.0478401	0.181877
MF_GO:0003846_2-acylglycerol+O-acyltransferase+activity	0.761792	0.825752	0.42094
MF_GO:0004111_creatine+kinase+activity	0.424558	0.146681	0.924882
BP_GO:0033089_positive+regulation+of+T+cell+differentiation+in+the+thym	0.802835	0.802835	#N/A
BP_GO:0043534_blood+vessel+endothelial+cell+migration	0.154936	0.154936	#N/A
CC_GO:0009341_beta-galactosidase+complex	0.521209	0.126943	0.888081
BP_GO:0042590_antigen+processing+and+presentation+of+exogenous+pep	0.514349	0.192418	0.70673
BP_GO:0001798_positive+regulation+of+type+IIa+hypersensitivity	0.589404	#N/A	0.589404
BP_GO:0043149_stress+fiber+formation	0.930777	0.813722	0.885465
CC_GO:0010008_endosome+membrane	0.5961	#N/A	0.5961
MF_GO:0008967_phosphoglycolate+phosphatase+activity	0.129959	0.387295	0.0396378
CC_GO:0031672_A+band	0.147614	0.0566156	0.384752
BP_GO:0020027_hemoglobin+metabolic+process	0.619384	0.672662	0.493008
BP_GO:0006707_cholesterol+catabolic+process	0.82289	0.343815	0.903547
MF_GO:0004028_3-chloroallyl+aldehyde+dehydrogenase+activity	0.163286	#N/A	0.163286
BP_GO:0006699_bile+acid+biosynthetic+process	0.537086	#N/A	0.537086
MF_GO:0051010_microtubule+plus-end+binding	0.228253	0.499373	0.195299
BP_GO:0006081_cellular+aldehyde+metabolic+process	0.821466	0.70105	0.780707
BP_GO:0050832_defense+response+to+fungus	0.647524	0.455357	0.741374
MF_GO:0016717_oxidoreductase+activity,+acting+on+paired+donors,+with-	0.464625	0.455687	0.506199
BP_GO:0046638_positive+regulation+of+alpha-beta+T+cell+differentiation	0.260593	0.404646	0.19387
MF_GO:0004740_pyruvate+dehydrogenase+(acetyl-transferring)+kinase+act	0.68493	0.68493	#N/A
BP_GO:0002526_acute+inflammatory+response	0.487694	0.438752	0.544102
MF_GO:0004749_ribose+phosphate+diphosphokinase+activity	0.750122	0.750122	#N/A
BP_GO:0006101_citrate+metabolic+process	0.935008	0.935008	#N/A
MF_GO:0004062_aryl+sulfotransferase+activity	0.599375	0.730211	0.288283

BP_GO:0021940_positive+regulation+of+granule+cell+precursor+proliferatic	0.158863	0.0861649	0.357673
CC_GO:0016010_dystrophin-associated+glycoprotein+complex	0.78652	0.879302	0.480703
BP_GO:0042159_lipoprotein+catabolic+process	0.996737	0.97651	0.976844
CC_GO:0030140_trans-Golgi+network+transport+vesicle	0.2593	0.2593	#N/A
BP_GO:0009404_toxin+metabolic+process	0.578395	0.829633	0.37373
BP_GO:0007167_enzyme+linked+receptor+protein+signaling+pathway	0.66989	0.74673	0.483004
BP_GO:0007009_plasma+membrane+organization	0.144038	0.0835522	0.452324
BP_GO:0051346_negative+regulation+of+hydrolase+activity	0.853033	0.105346	0.996885
MF_GO:0004030_aldehyde+dehydrogenase+[NAD(P)+]+activity	0.551869	0.70105	0.365844
BP_GO:0022027_interkinetic+nuclear+migration	0.512357	0.711442	0.387341
BP_GO:0032886_regulation+of+microtubule-based+process	0.512357	0.711442	0.387341
BP_GO:0006361_transcription+initiation+from+RNA+polymerase+I+promote	0.650112	0.650112	#N/A
BP_GO:0048771_tissue+remodeling	0.962834	0.976266	0.553866
BP_GO:0006742_NADP+catabolic+process	0.475685	0.680488	0.175331
MF_GO:0015175_neutral+amino+acid+transmembrane+transporter+activity	0.951408	0.951408	#N/A
BP_GO:0001523_retinoid+metabolic+process	0.566685	0.471187	0.621686
BP_GO:0051138_positive+regulation+of+NK+T+cell+differentiation	0.770159	0.660959	0.735732
BP_GO:0006497_protein+amino+acid+lipidation	0.560259	0.34263	0.842468
BP_GO:0045123_cellular+extravasation	0.412564	0.785672	0.134892
BP_GO:0006596_polyamine+biosynthetic+process	0.49212	0.598541	0.390796
BP_GO:0033344_cholesterol+efflux	0.713648	0.133662	0.971726
BP_GO:0050715_positive+regulation+of+cytokine+secretion	0.33474	0.417924	0.346087
BP_GO:0045647_negative+regulation+of+erythrocyte+differentiation	0.73255	0.73255	#N/A
BP_GO:0032963_collagen+metabolic+process	0.631351	0.630044	0.556618
BP_GO:0002318_myeloid+progenitor+cell+differentiation	0.391848	0.307801	0.545513
MF_GO:0003997_acyl-CoA+oxidase+activity	0.566731	0.502314	0.591727
BP_GO:0007176_regulation+of+epidermal+growth+factor+receptor+activity	0.937283	0.892114	0.823384
BP_GO:0006105_succinate+metabolic+process	0.763307	0.763307	#N/A
MF_GO:0004957_prostaglandin+E+receptor+activity	0.0994314	0.369552	0.0690711
BP_GO:0070059_apoptosis+in+response+to+endoplasmic+reticulum+stress	0.349613	0.0946968	0.778312
BP_GO:0050718_positive+regulation+of+interleukin-1+beta+secretion	0.369708	0.417924	0.396389
BP_GO:0042541_hemoglobin+biosynthetic+process	0.438583	0.708896	0.221128
CC_GO:0005664_nuclear+origin+of+replication+recognition+complex	0.0233864	0.749056	0.00364498
BP_GO:0002024_diet+induced+thermogenesis	0.257259	0.250998	0.401397
BP_GO:0016556_mRNA+modification	0.734507	0.713617	0.608712
BP_GO:0000103_sulfate+assimilation	0.212751	#N/A	0.212751
BP_GO:0007288_sperm+axoneme+assembly	0.000994668	0.000994668	#N/A
BP_GO:0045636_positive+regulation+of+melanocyte+differentiation	0.639038	0.18931	0.916779
BP_GO:0001778_plasma+membrane+repair	0.846354	0.478833	0.932722
BP_GO:0042307_positive+regulation+of+protein+import+into+nucleus	0.891116	0.515034	0.955869
BP_GO:0035235_ionotropic+glutamate+receptor+signaling+pathway	0.966225	0.989693	0.361716
BP_GO:0002925_positive+regulation+of+humoral+immune+response+medi	0.846664	0.638915	0.862051
BP_GO:0014037_Schwann+cell+differentiation	0.365862	0.506094	0.238517
BP_GO:0046326_positive+regulation+of+glucose+import	0.555587	0.891677	0.0315437
BP_GO:0046836_glycolipid+transport	0.666464	0.239411	0.817533
MF_GO:0017089_glycolipid+transporter+activity	0.666464	0.239411	0.817533
MF_GO:0051861_glycolipid+binding	0.666464	0.239411	0.817533

MF_GO:0015923_mannosidase+activity	0.697838	0.446513	0.807076
MF_GO:0004614_phosphoglucomutase+activity	0.793842	0.793842	#N/A
MF_GO:0004551_nucleotide+diphosphatase+activity	0.80618	#N/A	0.80618
MF_GO:0001530_lipopolysaccharide+binding	0.936124	0.689252	0.95147
CC_GO:0001527_microfibril	0.700758	0.456647	0.748892
MF_GO:0015101_organic+cation+transmembrane+transporter+activity	0.779446	0.973118	0.41099
BP_GO:0045651_positive+regulation+of+macrophage+differentiation	0.699743	0.898716	0.123281
MF_GO:0004046_aminoacylase+activity	0.986015	0.920219	0.974823
MF_GO:0030976_thiamin+pyrophosphate+binding	0.901544	0.575732	0.948787
BP_GO:0042116_macrophage+activation	0.602939	0.680002	0.386776
BP_GO:0000281_cytokinesis+after+mitosis	0.654962	0.515638	0.700091
BP_GO:0015816_glycine+transport	0.330766	0.415965	0.342245
MF_GO:0015187_glycine+transmembrane+transporter+activity	0.330766	0.415965	0.342245
BP_GO:0007091_mitotic+metaphase/anaphase+transition	0.0328891	#N/A	0.0328891
BP_GO:0032020_ISG15-protein+conjugation	0.33092	0.33092	#N/A
BP_GO:0043277_apoptotic+cell+clearance	0.78517	0.612393	0.86082
CC_GO:0043227_membrane-bounded+organelle	0.757702	0.465885	0.85854
BP_GO:0001667_ameboidal+cell+migration	0.400843	0.210408	0.569608
BP_GO:0042535_positive+regulation+of+tumor+necrosis+factor+biosyntheti	0.465582	0.715214	0.334273
BP_GO:0021955_central+nervous+system+neuron+axonogenesis	0.876204	0.954237	0.270958
MF_GO:0043023_ribosomal+large+subunit+binding	0.169194	0.133194	0.504402
MF_GO:0004718_Janus+kinase+activity	0.521751	0.547744	0.483004
BP_GO:0009166_nucleotide+catabolic+process	0.903605	#N/A	0.903605
BP_GO:0030501_positive+regulation+of+bone+mineralization	0.677973	0.979292	0.259525
BP_GO:0001838_embryonic+epithelial+tube+formation	0.0705054	0.0228943	0.697049
BP_GO:0007216_metabotropic+glutamate+receptor+signaling+pathway	0.118005	0.0881479	0.373538
BP_GO:0001991_regulation+of+systemic+arterial+blood+pressure+by+circul	0.996785	0.804886	0.99597
BP_GO:0002829_negative+regulation+of+T-helper+2+type+immune+respon	0.63743	0.314349	0.938174
BP_GO:0050919_negative+chemotaxis	0.511424	0.612995	0.402684
MF_GO:0030898_actin-dependent+ATPase+activity	0.527324	0.313589	0.835918
BP_GO:0050793_regulation+of+developmental+process	0.564576	0.564576	#N/A
BP_GO:0050774_negative+regulation+of+dendrite+morphogenesis	0.010208	0.0834467	0.0125479
BP_GO:0009437_carnitine+metabolic+process	0.399052	0.648488	0.228873
BP_GO:0001835_blastocyst+hatching	0.971069	0.410766	0.989836
BP_GO:0000255_allantoin+metabolic+process	0.916401	0.88638	0.748646
BP_GO:0019441_tryptophan+catabolic+process+to+kynurenine	0.75537	0.888417	0.537675
BP_GO:0043403_skeletal+muscle+regeneration	0.108827	0.211603	0.173249
BP_GO:0046651_lymphocyte+proliferation	0.126411	0.717288	0.0142296
CC_GO:0008290_F-actin+capping+protein+complex	0.52569	#N/A	0.52569
BP_GO:0007094_mitotic+cell+cycle+spindle+assembly+checkpoint	0.102468	0.500517	0.0716011
BP_GO:0045332_phospholipid+translocation	0.830736	0.833311	0.650146
BP_GO:0045213_neurotransmitter+receptor+metabolic+process	0.290689	0.230341	0.56963
BP_GO:0035196_gene+silencing+by+miRNA,+production+of+miRNAs	0.333768	0.133194	0.856692
BP_GO:0043542_endothelial+cell+migration	0.195624	0.114898	0.383733
MF_GO:0004104_cholinesterase+activity	0.838463	#N/A	0.838463
BP_GO:0015908_fatty+acid+transport	0.575944	0.423402	0.76341
MF_GO:0046934_phosphatidylinositol-4,5-bisphosphate+3-kinase+activity	0.799086	0.874328	0.619893

BP_GO:0001660_fever	0.102417	0.181285	0.174265
CC_GO:0005793_ER-Golgi+intermediate+compartment	0.446965	0.0985211	0.864728
MF_GO:0004126_cytidine+deaminase+activity	0.371964	0.0216893	0.940451
MF_GO:0004769_steroid+delta-isomerase+activity	0.602628	0.941018	0.273303
MF_GO:0045499_chemorepellent+activity	0.573725	0.539069	0.565478
BP_GO:0043009_chordate+embryonic+development	0.803102	0.924899	0.407793
MF_GO:0004185_serine-type+carboxypeptidase+activity	0.968707	0.983982	0.687347
CC_GO:0031982_vesicle	0.376221	0.199982	0.795813
BP_GO:0051271_negative+regulation+of+cell+motion	0.421488	0.25168	0.651534
BP_GO:0015833_peptide+transport	0.652103	0.415122	0.875446
MF_GO:0015197_peptide+transporter+activity	0.652103	0.415122	0.875446
BP_GO:0046849_bone+remodeling	0.567079	0.575868	0.519025
BP_GO:0008053_mitochondrial+fusion	0.21217	0.118235	0.674707
BP_GO:0032733_positive+regulation+of+interleukin-10+production	0.609005	0.185612	0.900704
BP_GO:0015871_choline+transport	0.940896	#N/A	0.940896
MF_GO:0016462_pyrophosphatase+activity	0.035037	0.035037	#N/A
BP_GO:0042473_outer+ear+morphogenesis	0.976353	0.91434	0.94476
CC_GO:0030132_clathrin+coat+of+coated+pit	0.637078	0.509572	0.6815
BP_GO:0002035_brain+renin-angiotensin+system	0.809713	0.804886	0.697063
BP_GO:0048843_negative+regulation+of+axon+extension+involved+in+axor	0.667767	0.727328	0.428702
BP_GO:0051262_protein+tetramerization	0.775782	#N/A	0.775782
MF_GO:0005332_gamma-aminobutyric+acid:sodium+symporter+activity	0.782094	0.712036	0.72215
BP_GO:0042420_dopamine+catabolic+process	0.588687	0.281407	0.814812
BP_GO:0050768_negative+regulation+of+neurogenesis	0.592778	0.86607	0.356021
MF_GO:0008113_peptide-methionine-(S)-S-oxide+reductase+activity	0.458226	0.458226	#N/A
BP_GO:0012501_programmed+cell+death	0.360978	0.12216	0.745861
BP_GO:0050803_regulation+of+synapse+structure+and+activity	0.465583	0.465583	#N/A
MF_GO:0015355_secondary+active+monocarboxylate+transmembrane+trar	0.314635	0.222562	0.532459
MF_GO:0004300_enoyl-CoA+hydratase+activity	0.0542105	0.0542105	#N/A
BP_GO:0050778_positive+regulation+of+immune+response	0.670204	0.0181809	0.957102
BP_GO:0030384_phosphoinositide+metabolic+process	0.11627	0.146925	0.261897
CC_GO:0031252_cell+leading+edge	0.37513	0.751371	0.129561
CC_GO:0016469_proton-transporting+two-sector+ATPase+complex	0.437525	0.386239	0.573982
CC_GO:0031253_cell+projection+membrane	0.205879	0.146793	0.366736
MF_GO:0004427_inorganic+diphosphatase+activity	0.130737	0.130737	#N/A
BP_GO:0006351_transcription,+DNA-dependent	0.0137771	0.00154335	0.202152
BP_GO:0030146_diuresis	0.287602	#N/A	0.287602
BP_GO:0045061_thymic+T+cell+selection	0.0779018	0.0779018	#N/A
BP_GO:0045930_negative+regulation+of+mitotic+cell+cycle	0.863598	#N/A	0.863598
BP_GO:0048255_mRNA+stabilization	0.591274	0.273893	0.82319
BP_GO:0010172_embryonic+body+morphogenesis	0.418921	0.456877	0.428293
BP_GO:0030261_chromosome+condensation	0.575178	0.509231	0.581471
MF_GO:0009374_biotin+binding	0.525661	0.637517	0.315622
BP_GO:0001938_positive+regulation+of+endothelial+cell+proliferation	0.0112265	0.0071321	0.374784
BP_GO:0006927_transformed+cell+apoptosis	0.223227	0.470171	0.158402
CC_GO:0019815_B+cell+receptor+complex	0.914285	0.944559	0.489499
BP_GO:0000303_response+to+superoxide	0.290598	0.243292	0.466636

MF_GO:0046965_retinoid+X+receptor+binding	0.600589	0.714255	0.418646
CC_GO:0005658_alpha+DNA+polymerase:primase+complex	0.198981	0.623607	0.0655875
CC_GO:0005868_cytoplasmic+dynein+complex	0.443011	0.349075	0.649916
BP_GO:0050435_beta-amyloid+metabolic+process	0.6212	0.452295	0.795135
BP_GO:0030818_negative+regulation+of+cAMP+biosynthetic+process	0.45952	0.466203	0.476633
BP_GO:0000089_mitotic+metaphase	0.136003	0.196292	0.237387
MF_GO:0004656_procollagen-proline+4-dioxygenase+activity	0.561218	0.261666	0.921115
BP_GO:0021545_cranial+nerve+development	0.350071	0.64404	0.180516
BP_GO:0002051_osteoblast+fate+commitment	0.244101	0.193657	0.454208
MF_GO:0004308_exo-alpha-sialidase+activity	0.78758	0.656224	0.815601
BP_GO:0032816_positive+regulation+of+natural+killer+cell+activation	0.741155	0.384565	0.886505
BP_GO:0033299_secretion+of+lysosomal+enzymes	0.741155	0.384565	0.886505
BP_GO:0045736_negative+regulation+of+cyclin-dependent+protein+kinase+	0.413617	0.70986	0.0818516
BP_GO:0001736_establishment+of+planar+polarity	0.155349	0.920723	0.0235797
MF_GO:0050431_transforming+growth+factor+beta+binding	0.64615	0.596314	0.628341
MF_GO:0004536_deoxyribonuclease+activity	0.493354	0.515013	0.483725
BP_GO:0045333_cellular+respiration	0.325086	0.614001	0.244837
MF_GO:0042801_polo+kinase+kinase+activity	0.883423	0.925947	0.594539
BP_GO:0009967_positive+regulation+of+signal+transduction	0.506281	#N/A	0.506281
BP_GO:0045779_negative+regulation+of+bone+resorption	0.755828	#N/A	0.755828
MF_GO:0030332_cyclin+binding	0.727372	0.790273	0.591716
BP_GO:0051881_regulation+of+mitochondrial+membrane+potential	0.560113	0.081309	0.946341
BP_GO:0048617_embryonic+foregut+morphogenesis	0.581157	0.393248	0.712487
BP_GO:0006906_vesicle+fusion	0.985509	#N/A	0.985509
BP_GO:0019885_antigen+processing+and+presentation+of+endogenous+pe	0.142829	0.35145	0.155761
BP_GO:0002326_B+cell+lineage+commitment	0.97519	0.513157	0.987698
CC_GO:0005652_nuclear+lamina	0.144851	0.138982	0.340389
BP_GO:0006269_DNA+replication,+synthesis+of+RNA+primer	0.0857192	0.387375	0.0787853
MF_GO:0004095_carnitine+O-palmitoyltransferase+activity	0.544791	0.544791	#N/A
BP_GO:0002904_positive+regulation+of+B+cell+apoptosis	0.484197	0.0579951	0.805844
CC_GO:0005721_centromeric+heterochromatin	0.397001	0.837323	0.192267
BP_GO:0060296_regulation+of+cilium+beat+frequency+involved+in+ciliary+	0.204762	0.250928	0.313767
BP_GO:0015074_DNA+integration	0.206406	0.352131	0.233939
MF_GO:0000179_rRNA+(adenine-N6,N6-)-dimethyltransferase+activity	0.703158	0.820405	0.301317
MF_GO:0008649_rRNA+methyltransferase+activity	0.703158	0.820405	0.301317
CC_GO:0008091_spectrin	0.649311	0.205882	0.820465
BP_GO:0006610_ribosomal+protein+import+into+nucleus	0.507484	0.550436	0.460172
BP_GO:0046632_alpha-beta+T+cell+differentiation	0.77971	0.496553	0.86411
BP_GO:0048013_ephrin+receptor+signaling+pathway	0.705835	0.711729	0.545872
BP_GO:0048558_embryonic+gut+morphogenesis	0.973698	0.873548	0.970882
MF_GO:0005313_L-glutamate+transmembrane+transporter+activity	0.917775	0.992665	0.317371
BP_GO:0007386_compartment+specification	0.218491	0.13158	0.508179
MF_GO:0005005_transmembrane-ephrin+receptor+activity	0.660054	0.863751	0.303672
BP_GO:0007028_cytoplasm+organization	0.550338	0.550338	#N/A
MF_GO:0043015_gamma-tubulin+binding	0.416205	0.1149	0.673567
BP_GO:0001817_regulation+of+cytokine+production	0.302742	0.366417	0.348823
BP_GO:0009308_cellular+amine+metabolic+process	0.921272	0.962618	0.397785

MF_GO:0015631_tubulin+binding	0.433166	0.18406	0.74612
CC_GO:0005853_eukaryotic+translation+elongation+factor+1+complex	0.510362	0.107443	0.899235
MF_GO:0048365_Rac+GTPase+binding	0.251848	0.475571	0.188442
MF_GO:0016614_oxidoreductase+activity,+acting+on+CH-OH+group+of+do	0.840877	0.246991	0.981914
BP_GO:0050965_detection+of+temperature+stimulus+involved+in+sensory+	0.340802	0.397619	0.355591
BP_GO:0051146_striated+muscle+cell+differentiation	0.448637	0.6075	0.324536
BP_GO:0055072_iron+ion+homeostasis	0.727953	0.343993	0.824475
BP_GO:0001895_retina+homeostasis	0.723883	0.598387	0.706235
BP_GO:0048149_behavioral+response+to+ethanol	0.79267	#N/A	0.79267
BP_GO:0051898_negative+regulation+of+protein+kinase+B+signaling+casca	0.901524	0.822353	0.815849
BP_GO:0060158_dopamine+receptor,+phospholipase+C+activating+pathwa	0.880435	0.846076	0.740338
MF_GO:0005337_nucleoside+transmembrane+transporter+activity	0.568546	0.104749	0.933097
BP_GO:0007599_hemostasis	0.734914	0.351001	0.827917
BP_GO:0032713_negative+regulation+of+interleukin-4+production	0.220988	0.176625	0.528194
BP_GO:0048608_reproductive+structure+development	0.954941	0.933571	0.81403
BP_GO:0031119_tRNA+pseudouridine+synthesis	0.0522144	#N/A	0.0522144
BP_GO:0050810_regulation+of+steroid+biosynthetic+process	0.614907	0.537866	0.624799
BP_GO:0001825_blastocyst+formation	0.211729	0.162566	0.541405
MF_GO:0004439_phosphatidylinositol-4,5-bisphosphate+5-phosphatase+ac	0.323594	0.461285	0.227873
BP_GO:0034109_homotypic+cell-cell+adhesion	0.888161	0.823743	0.785365
MF_GO:0042562_hormone+binding	0.312216	0.562591	0.105563
BP_GO:0045822_negative+regulation+of+heart+contraction	0.169434	0.169434	#N/A
MF_GO:0004500_dopamine+beta-monooxygenase+activity	0.434487	0.763721	0.17074
BP_GO:0030166_proteoglycan+biosynthetic+process	0.975804	0.952293	0.855019
BP_GO:0043129_surfactant+homeostasis	0.540875	0.385717	0.668517
BP_GO:0043069_negative+regulation+of+programmed+cell+death	0.260255	0.53756	0.212984
BP_GO:0045745_positive+regulation+of+G-protein+coupled+receptor+prote	0.806484	0.94913	0.339598
BP_GO:0060124_positive+regulation+of+growth+hormone+secretion	0.865246	0.696073	0.836065
BP_GO:0042787_protein+ubiquitination+during+ubiquitin-dependent+prote	0.881875	0.957732	0.268096
MF_GO:0017147_Wnt-protein+binding	0.995134	0.977996	0.949539
BP_GO:0019228_regulation+of+action+potential+in+neuron	0.34512	0.323776	0.422407
CC_GO:0030673_axolemma	0.698333	0.875166	0.33854
MF_GO:0005381_iron+ion+transmembrane+transporter+activity	0.297246	0.459824	0.257419
BP_GO:0030010_establishment+of+cell+polarity	0.997763	0.995434	0.878564
MF_GO:0001730_2'-5'-oligoadenylate+synthetase+activity	0.537568	0.555035	0.498067
BP_GO:0045580_regulation+of+T+cell+differentiation	0.371621	0.280922	0.636757
BP_GO:0042403_thyroid+hormone+metabolic+process	0.981282	#N/A	0.981282
MF_GO:0042623_ATPase+activity,+coupled	0.757839	0.262803	0.879868
BP_GO:0033152_immunoglobulin+V(D)J+recombination	0.188777	0.932752	0.0298295
BP_GO:0001514_selenocysteine+incorporation	0.168636	0.0896205	0.49482
BP_GO:0006091_generation+of+precursor+metabolites+and+energy	0.205232	0.102719	0.54104
BP_GO:0030949_positive+regulation+of+vascular+endothelial+growth+facto	0.332376	0.457714	0.33042
BP_GO:0031122_cytoplasmic+microtubule+organization	0.902117	0.850501	0.785319
MF_GO:0016742_hydroxymethyl-,+formyl-+and+related+transferase+activit	0.278371	#N/A	0.278371
CC_GO:0005663_DNA+replication+factor+C+complex	0.781788	0.696629	0.726145
MF_GO:0003689_DNA+clamp+loader+activity	0.781788	0.696629	0.726145
BP_GO:0048675_axon+extension	0.593396	0.593396	#N/A

MF_GO:0005088_Ras+guanyl-nucleotide+exchange+factor+activity	0.59982	#N/A	0.59982
BP_GO:0048565_gut+development	0.903666	0.938754	0.616853
BP_GO:0015780_nucleotide-sugar+transport	0.573979	0.469323	0.693625
MF_GO:0005338_nucleotide-sugar+transmembrane+transporter+activity	0.573979	0.469323	0.693625
BP_GO:0021854_hypothalamus+development	0.349245	0.0579951	0.677686
BP_GO:0046642_negative+regulation+of+alpha-beta+T+cell+proliferation	0.795564	0.650357	0.767429
BP_GO:0021795_cerebral+cortex+cell+migration	0.695697	0.115576	0.900292
BP_GO:0021696_cerebellar+cortex+morphogenesis	0.300179	0.290974	0.424767
CC_GO:0070044_synaptobrevin+2-SNAP-25-syntaxin-1a+complex	0.224956	0.0287452	0.589222
CC_GO:0005638_lamin+filament	0.466532	0.224981	0.872845
BP_GO:0015879_carnitine+transport	0.90301	0.98183	0.398628
MF_GO:0015226_carnitine+transporter+activity	0.90301	0.98183	0.398628
BP_GO:0021884_forebrain+neuron+development	0.669644	0.875405	0.297482
BP_GO:0006875_cellular+metal+ion+homeostasis	0.5309	#N/A	0.5309
BP_GO:0042476_odontogenesis	0.196424	0.33437	0.217801
BP_GO:0006595_polyamine+metabolic+process	0.0153582	0.00167475	0.452167
BP_GO:0006287_base-excision+repair,+gap-filling	0.125947	0.0463796	0.732494
CC_GO:0005930_axoneme	0.184281	0.0603352	0.443895
BP_GO:0031532_actin+cytoskeleton+reorganization	0.754041	0.603201	0.761158
BP_GO:0001945_lymph+vessel+development	0.864586	0.912839	0.686679
BP_GO:0060011_Sertoli+cell+proliferation	0.140113	0.237314	0.178859
MF_GO:0003840_gamma-glutamyltransferase+activity	0.757021	0.740233	0.633458
BP_GO:0035021_negative+regulation+of+Rac+protein+signal+transduction	0.720121	0.737027	0.575494
MF_GO:0003923_GPI-anchor+transamidase+activity	0.37357	0.0276373	0.928024
BP_GO:0030033_microvillus+assembly	0.635277	0.866067	0.404867
BP_GO:0045806_negative+regulation+of+endocytosis	0.427621	0.499707	0.358337
MF_GO:0004060_arylamine+N-acetyltransferase+activity	0.927014	#N/A	0.927014
BP_GO:0043303_mast+cell+degranulation	0.67749	0.597188	0.65167
BP_GO:0050882_voluntary+musculoskeletal+movement	0.562732	0.494221	0.633389
MF_GO:0004784_superoxide+dismutase+activity	0.159064	0.283644	0.200793
BP_GO:0046887_positive+regulation+of+hormone+secretion	0.925866	#N/A	0.925866
BP_GO:0008406_gonad+development	0.295856	#N/A	0.295856
BP_GO:0043200_response+to+amino+acid+stimulus	0.776165	0.925304	0.16412
MF_GO:0004735_pyrroline-5-carboxylate+reductase+activity	0.0050365	0.00134222	0.521497
BP_GO:0021612_facial+nerve+structural+organization	0.238021	0.386082	0.256146
BP_GO:0007143_female+meiosis	0.123296	0.0177204	0.679317
MF_GO:0004974_leukotriene+receptor+activity	0.349977	0.105613	0.609369
BP_GO:0042416_dopamine+biosynthetic+process	0.362025	#N/A	0.362025
CC_GO:0043204_perikaryon	0.888579	0.697077	0.866348
BP_GO:0055088_lipid+homeostasis	0.747286	0.436342	0.946104
BP_GO:0060173_limb+development	0.932677	0.0502015	0.996281
CC_GO:0008250_oligosaccharyltransferase+complex	0.951299	0.967784	0.689348
CC_GO:0005958_DNA-dependent+protein+kinase+complex	0.24108	#N/A	0.24108
MF_GO:0005499_vitamin+D+binding	0.603824	0.770165	0.356796
BP_GO:0015844_monoamine+transport	0.73922	0.349242	0.832464
MF_GO:0000030_mannosyltransferase+activity	0.329932	0.49835	0.191654
MF_GO:0005243_gap+junction+channel+activity	0.329969	0.694287	0.211468

BP_GO:0032495_response+to+muramyl+dipeptide	0.857778	0.518081	0.928931
MF_GO:0046703_natural+killer+cell+lectin-like+receptor+binding	0.856791	0.372534	0.966538
CC_GO:0031513_nonmotile+primary+cilium	0.989588	0.889811	0.993735
BP_GO:0002669_positive+regulation+of+T+cell+anergy	0.891561	0.793748	0.852966
MF_GO:0008353_RNA+polymerase+subunit+kinase+activity	0.735736	0.415042	0.80283
BP_GO:0006510_ATP-dependent+proteolysis	0.364078	0.276405	0.630467
MF_GO:0004176_ATP-dependent+peptidase+activity	0.364078	0.276405	0.630467
BP_GO:0016322_neuron+remodeling	0.998623	0.994459	0.943629
CC_GO:0070032_synaptobrevin+2-SNAP-25-syntaxin-1a-complexin+I+compl	0.720908	0.782255	0.589222
MF_GO:0005152_interleukin-1+receptor+antagonist+activity	0.229622	0.569146	0.169819
CC_GO:0032281_alpha-amino-3-hydroxy-5-methyl-4-isoxazolepropionic+aci	0.477612	0.55494	0.442564
CC_GO:0035098_ESC/E(Z)+complex	0.496234	0.789647	0.206595
BP_GO:0051798_positive+regulation+of+hair+follicle+development	0.458942	0.501632	0.44055
CC_GO:0005798_Golgi-associated+vesicle	0.544226	0.545953	0.516679
BP_GO:0021938_smoothened+signaling+pathway+involved+in+regulation+c	0.405021	0.233528	0.556824
BP_GO:0048025_negative+regulation+of+nuclear+mRNA+splicing,+via+splic	0.968943	0.870679	0.933331
MF_GO:0016594_glycine+binding	0.268156	0.548342	0.216542
BP_GO:0048814_regulation+of+dendrite+morphogenesis	0.662921	0.854831	0.450094
BP_GO:0032720_negative+regulation+of+tumor+necrosis+factor+productio	0.583623	0.502514	0.659575
MF_GO:0004999_vasoactive+intestinal+polypeptide+receptor+activity	0.321447	0.655818	0.145439
BP_GO:0007066_female+meiosis+sister+chromatid+cohesion	0.849152	#N/A	0.849152
BP_GO:0045672_positive+regulation+of+osteoclast+differentiation	0.671703	0.26574	0.975669
BP_GO:0045987_positive+regulation+of+smooth+muscle+contraction	0.639454	#N/A	0.639454
BP_GO:0060235_lens+induction+in+camera-type+eye	0.675324	0.716744	0.527785
BP_GO:0050828_regulation+of+liquid+surface+tension	0.997398	0.994183	0.96147
BP_GO:0007292_female+gamete+generation	0.995107	0.927299	0.983869
BP_GO:0030259_lipid+glycosylation	0.939533	0.910851	0.779164
CC_GO:0005956_protein+kinase+CK2+complex	0.0538581	0.0981261	0.1643
BP_GO:0021853_cerebral+cortex+GABAergic+interneuron+migration	0.88253	0.589792	0.892545
MF_GO:0008301_DNA+bending+activity	0.551313	0.69217	0.37469
MF_GO:0008853_exodeoxyribonuclease+III+activity	0.758516	0.758516	#N/A
BP_GO:0021707_cerebellar+granule+cell+differentiation	0.977086	0.899748	0.938561
BP_GO:0032689_negative+regulation+of+interferon-gamma+production	0.939321	0.875509	0.850141
CC_GO:0045179_apical+cortex	0.246724	0.683486	0.0741982
BP_GO:0021569_rhombomere+3+development	0.465047	0.68807	0.269562
MF_GO:0004985_opioid+receptor+activity	0.198486	0.424513	0.157048
BP_GO:0000289_nuclear-transcribed+mRNA+poly(A)+tail+shortening	0.580974	0.55806	0.556889
MF_GO:0004791_thioredoxin-disulfide+reductase+activity	0.266118	0.112767	0.802288
BP_GO:0006824_cobalt+ion+transport	0.788634	0.792913	0.62432
BP_GO:0021772_olfactory+bulb+development	0.276479	0.232774	0.396225
MF_GO:0035254_glutamate+receptor+binding	0.97265	0.97265	#N/A
BP_GO:0050953_sensory+perception+of+light+stimulus	0.676557	0.732428	0.437329
CC_GO:0001917_photoreceptor+inner+segment	0.765515	0.468422	0.943429
MF_GO:0016208_AMP+binding	0.0630357	0.0630357	#N/A
BP_GO:0048505_regulation+of+timing+of+cell+differentiation	0.732424	0.902789	0.157129
MF_GO:0015467_G-protein+activated+inward+rectifier+potassium+channel	0.26473	0.54672	0.157215
BP_GO:0001823_mesonephros+development	0.86829	0.628007	0.895154

BP_GO:0032332_positive+regulation+of+chondrocyte+differentiation	0.475718	0.513258	0.452607
BP_GO:0014051_gamma-aminobutyric+acid+secretion	0.815852	0.328928	0.902289
MF_GO:0030519_snoRNP+binding	0.612317	0.78223	0.353404
CC_GO:0043186_P+granule	0.858462	0.811198	0.731704
BP_GO:0051967_negative+regulation+of+synaptic+transmission,+glutamate	0.639733	0.323776	0.750905
BP_GO:0006432_phenylalanyl-tRNA+aminoacylation	0.452997	0.687106	0.256446
BP_GO:0008156_negative+regulation+of+DNA+replication	0.833382	0.650471	0.836791
CC_GO:0043159_acrosomal+matrix	0.77632	0.869708	0.479752
MF_GO:0017150_tRNA+dihydrouridine+synthase+activity	0.312246	0.551955	0.114357
BP_GO:0001661_conditioned+taste+aversion	0.555167	0.854831	0.326119
BP_GO:0033327_Leydig+cell+differentiation	0.122332	0.251123	0.165216
BP_GO:0042177_negative+regulation+of+protein+catabolic+process	0.872256	0.986064	0.516839
MF_GO:0004052_arachidonate+12-lipoxygenase+activity	0.533324	0.334235	0.818199
BP_GO:0045862_positive+regulation+of+proteolysis	0.377001	0.272581	0.564479
BP_GO:0021879_forebrain+neuron+differentiation	0.302925	0.307697	0.380094
BP_GO:0050868_negative+regulation+of+T+cell+activation	0.987862	#N/A	0.987862
MF_GO:0004950_chemokine+receptor+activity	0.109772	#N/A	0.109772
MF_GO:0046920_alpha(1,3)-fucosyltransferase+activity	0.49083	0.489743	0.499553
CC_GO:0032045_guanyl-nucleotide+exchange+factor+complex	0.683975	0.483195	0.71814
BP_GO:0000079_regulation+of+cyclin-dependent+protein+kinase+activity	0.302754	0.622033	0.149023
BP_GO:0014003_oligodendrocyte+development	0.96608	0.987914	0.789772
BP_GO:0043623_cellular+protein+complex+assembly	0.656586	0.389758	0.802354
CC_GO:0000801_central+element	0.306234	0.369893	0.35063
MF_GO:0045182_translation+regulator+activity	0.881194	0.449985	0.924525
BP_GO:0019216_regulation+of+lipid+metabolic+process	0.243109	0.814936	0.0931541
MF_GO:0003945_N-acetyllactosamine+synthase+activity	0.100526	0.0842165	0.432253
MF_GO:0042826_histone+deacetylase+binding	0.202672	0.164456	0.345935
BP_GO:0051930_regulation+of+sensory+perception+of+pain	0.940548	0.948491	0.615712
BP_GO:0022011_myelination+in+the+peripheral+nervous+system	0.306945	0.284385	0.443134
CC_GO:0035085_cilium+axoneme	0.0911281	#N/A	0.0911281
CC_GO:0000120_RNA+polymerase+I+transcription+factor+complex	0.209352	0.493995	0.129715
BP_GO:0014832_urinary+bladder+smooth+muscle+contraction	0.783325	0.828664	0.563072
MF_GO:0001642_group+III+metabotropic+glutamate+receptor+activity	0.137534	0.676205	0.0637548
MF_GO:0015016_[heparan+sulfate]-glucosamine+N-sulfotransferase+activit	0.76125	0.763813	0.569784
MF_GO:0008504_monoamine+transmembrane+transporter+activity	0.921154	0.736884	0.913645
CC_GO:0048500_signal+recognition+particle	0.0857998	0.0103698	0.898203
MF_GO:0008312_7S+RNA+binding	0.0857998	0.0103698	0.898203
BP_GO:0021797_forebrain+anterior/posterior+pattern+formation	0.0484692	0.0741867	0.207615
CC_GO:0032983_kainate+selective+glutamate+receptor+complex	0.340502	0.304017	0.526541
MF_GO:0019104_DNA+N-glycosylase+activity	0.684433	0.772453	0.472872
MF_GO:0015279_store-operated+calcium+channel+activity	0.350651	0.863509	0.140951
CC_GO:0005736_DNA-directed+RNA+polymerase+I+complex	0.321709	0.321709	#N/A
CC_GO:0001652_granular+component	0.932757	0.0399844	0.996925
CC_GO:0005773_vacuole	0.450788	0.450788	#N/A
BP_GO:0016973_poly(A)++mRNA+export+from+nucleus	0.655643	0.451149	0.754665
MF_GO:0004969_histamine+receptor+activity	0.435101	0.430076	0.478253
CC_GO:0033391_chromatoid+body	0.555833	0.636096	0.440648

BP_GO:0021979_hypothalamus+cell+differentiation	0.328872	0.281278	0.546683
BP_GO:0030252_growth+hormone+secretion	0.72057	0.754034	0.60945
BP_GO:0055012_ventricular+cardiac+muscle+cell+differentiation	0.37247	0.37247	#N/A
BP_GO:0050913_sensory+perception+of+bitter+taste	0.677369	#N/A	0.677369
BP_GO:0006465_signal+peptide+processing	0.970997	0.125394	0.997829
MF_GO:0016934_extracellular-glycine-gated+chloride+channel+activity	0.19887	0.548342	0.147713
BP_GO:0021542_dentate+gyrus+development	0.419442	0.489774	0.396851
BP_GO:0009303_rRNA+transcription	0.424663	0.0332187	0.997423
MF_GO:0015057_thrombin+receptor+activity	0.442199	0.133205	0.68243
BP_GO:0006400_tRNA+modification	0.443153	0.443153	#N/A
BP_GO:0045604_regulation+of+epidermal+cell+differentiation	0.422566	0.183564	0.879167
MF_GO:0050327_testosterone+17-beta-dehydrogenase+activity	0.130251	0.141154	0.268676
MF_GO:0047961_glycine+N-acyltransferase+activity	0.233958	0.145752	0.443433
BP_GO:0019510_S-adenosylhomocysteine+catabolic+process	0.0595719	0.0595719	#N/A
BP_GO:0001960_negative+regulation+of+cytokine+mediated+signaling+path	0.388161	0.326267	0.557645
MF_GO:0004082_bisphosphoglycerate+mutase+activity	0.152448	0.152448	#N/A
MF_GO:0004083_bisphosphoglycerate+2-phosphatase+activity	0.152448	0.152448	#N/A
MF_GO:0004619_phosphoglycerate+mutase+activity	0.152448	0.152448	#N/A
BP_GO:0043056_forward+locomotion	0.0414226	0.0566156	0.157971
BP_GO:0055002_striated+muscle+cell+development	0.0414226	0.0566156	0.157971
MF_GO:0008034_lipoprotein+binding	0.739967	0.363173	0.946104
MF_GO:0005504_fatty+acid+binding	0.749595	0.323537	0.874593
BP_GO:0016125_sterol+metabolic+process	0.566626	0.483704	0.636238
CC_GO:0005945_6-phosphofructokinase+complex	0.558323	0.558323	#N/A
MF_GO:0003872_6-phosphofructokinase+activity	0.558323	0.558323	#N/A
BP_GO:0030193_regulation+of+blood+coagulation	0.702528	0.702528	#N/A
BP_GO:0015909_long-chain+fatty+acid+transport	0.360262	0.360262	#N/A
MF_GO:0004345_glucose-6-phosphate+dehydrogenase+activity	0.0265306	0.30743	0.0220952
MF_GO:0004090_carbonyl+reductase+(NADPH)+activity	0.027983	0.027983	#N/A
BP_GO:0001937_negative+regulation+of+endothelial+cell+proliferation	0.313631	0.356251	0.374784
BP_GO:0043496_regulation+of+protein+homodimerization+activity	0.137765	0.735681	0.0374857
BP_GO:0006144_purine+base+metabolic+process	0.687843	0.0673314	0.951361
BP_GO:0001915_negative+regulation+of+T+cell+mediated+cytotoxicity	0.827108	0.853589	0.557645
MF_GO:0004332_fructose-bisphosphate+aldolase+activity	0.130597	0.130597	#N/A
BP_GO:0035025_positive+regulation+of+Rho+protein+signal+transduction	0.555087	0.678489	0.437192
BP_GO:0045777_positive+regulation+of+blood+pressure	0.990444	0.53428	0.997511
BP_GO:0005980_glycogen+catabolic+process	0.945781	0.945781	#N/A
MF_GO:0004944_C5a+anaphylatoxin+receptor+activity	0.647556	0.69419	0.475287
MF_GO:0003876_AMP+deaminase+activity	0.754406	#N/A	0.754406
MF_GO:0004144_diacylglycerol+O-acyltransferase+activity	0.825752	0.825752	#N/A
CC_GO:0016234_inclusion+body	0.791024	0.606661	0.846023
BP_GO:0008154_actin+polymerization+or+depolymerization	0.408709	0.148964	0.675064
MF_GO:0004505_phenylalanine+4-monooxygenase+activity	0.404073	0.616346	0.200964
CC_GO:0032154_cleavage+furrow	0.00048192	0.00399038	0.0248534
MF_GO:0004064_arylesterase+activity	0.477333	0.477333	#N/A
BP_GO:0050717_positive+regulation+of+interleukin-1+alpha+secretion	0.462713	0.417924	0.552183
BP_GO:0006509_membrane+protein+ectodomain+proteolysis	0.837545	#N/A	0.837545

MF_GO:0008449_N-acetylglucosamine-6-sulfatase+activity	0.274116	#N/A	0.274116
MF_GO:0004473_malate+dehydrogenase+(oxaloacetate-decarboxylating)+(I	0.000211855	0.010599	0.0036244
BP_GO:0050732_negative+regulation+of+peptidyl-tyrosine+phosphorylation	0.915848	0.943449	0.557645
BP_GO:0030857_negative+regulation+of+epithelial+cell+differentiation	0.111474	0.279602	0.0996829
BP_GO:0002025_vasodilation+by+norepinephrine-epinephrine+involved+in+	0.155188	0.250998	0.210015
BP_GO:0031649_heat+generation	0.155188	0.250998	0.210015
MF_GO:0004065_arylsulfatase+activity	0.26771	#N/A	0.26771
BP_GO:0045600_positive+regulation+of+fat+cell+differentiation	0.483089	0.483089	#N/A
BP_GO:0043691_reverse+cholesterol+transport	0.517902	0.268519	0.6886
MF_GO:0016212_kynurenine-oxoglutarate+transaminase+activity	0.307221	0.241174	0.548356
BP_GO:0060325_face+morphogenesis	0.396811	0.0454836	0.809418
BP_GO:0048251_elastic+fiber+assembly	0.417392	0.351658	0.505696
BP_GO:0006689_ganglioside+catabolic+process	0.625287	0.625287	#N/A
BP_GO:0014070_response+to+organic+cyclic+substance	0.335454	0.147555	0.587329
BP_GO:0018894_dibenzo-p-dioxin+metabolic+process	0.146287	0.829633	0.0248544
MF_GO:0004594_pantothenate+kinase+activity	0.394713	0.358827	0.519628
MF_GO:0003985_acetyl-CoA+C-acetyltransferase+activity	0.118569	0.515462	0.0178469
MF_GO:0042578_phosphoric+ester+hydrolase+activity	0.73883	0.440773	0.906257
BP_GO:0006642_triacylglycerol+mobilization	0.997939	0.998334	0.792677
BP_GO:0006536_glutamate+metabolic+process	0.593268	0.836649	0.16412
BP_GO:0042359_vitamin+D+metabolic+process	0.250011	0.47375	0.217991
BP_GO:0030029_actin+filament-based+process	0.0856632	0.0393797	0.333239
BP_GO:0045621_positive+regulation+of+lymphocyte+differentiation	0.494252	0.662105	0.269018
BP_GO:0006564_L-serine+biosynthetic+process	0.667997	0.204972	0.972364
BP_GO:0045103_intermediate+filament-based+process	0.500812	#N/A	0.500812
BP_GO:0015760_glucose-6-phosphate+transport	0.532498	0.532498	#N/A
CC_GO:0031362_anchored+to+external+side+of+plasma+membrane	0.643409	#N/A	0.643409
BP_GO:0001805_positive+regulation+of+type+III+hypersensitivity	0.70673	#N/A	0.70673
MF_GO:0008469_histone-arginine+N-methyltransferase+activity	0.223461	0.223461	#N/A
BP_GO:0009060_aerobic+respiration	0.176212	0.505099	0.0518608
BP_GO:0046541_saliva+secretion	0.765639	0.863509	0.544502
BP_GO:0015697_quaternary+ammonium+group+transport	0.564469	0.848508	0.298122
MF_GO:0030247_polysaccharide+binding	0.781404	0.781404	#N/A
MF_GO:0003716_RNA+polymerase+I+transcription+termination+factor+acti	0.33643	0.33643	#N/A
BP_GO:0006103_2-oxoglutarate+metabolic+process	0.88638	0.88638	#N/A
BP_GO:0006549_isoleucine+metabolic+process	0.88638	0.88638	#N/A
BP_GO:0006600_creatine+metabolic+process	0.88638	0.88638	#N/A
BP_GO:0046449_creatinine+metabolic+process	0.88638	0.88638	#N/A
CC_GO:0043259_laminin-10+complex	0.597632	0.165513	0.839059
MF_GO:0004775_succinate-CoA+ligase+(ADP-forming)+activity	0.210617	0.192818	0.434336
BP_GO:0006868_glutamine+transport	0.876828	0.898549	0.581888
MF_GO:0015186_L-glutamine+transmembrane+transporter+activity	0.876828	0.898549	0.581888
BP_GO:0045907_positive+regulation+of+vasoconstriction	0.166407	0.060549	0.696862
BP_GO:0008228_opsonization	0.993386	0.928813	0.9866
MF_GO:0030676_Rac+guanyl-nucleotide+exchange+factor+activity	0.626178	0.230884	0.819851
CC_GO:0060053_neurofilament+cytoskeleton	0.449011	0.297783	0.701507
MF_GO:0047238_glucuronosyl-N-acetylgalactosaminyl-proteoglycan+4-beta	0.86342	0.40208	0.935406

MF_GO:0050510_N-acetylgalactosaminyl-proteoglycan+3-beta-glucuronosyl	0.86342	0.40208	0.935406
MF_GO:0004792_thiosulfate+sulfurtransferase+activity	0.00391691	0.0179405	0.0509382
BP_GO:0045663_positive+regulation+of+myoblast+differentiation	0.829358	#N/A	0.829358
MF_GO:0004165_dodecenoyl-CoA+delta-isomerase+activity	0.135243	0.135243	#N/A
BP_GO:0008215_spermine+metabolic+process	0.518188	#N/A	0.518188
BP_GO:0043392_negative+regulation+of+DNA+binding	0.914973	0.233528	0.985904
BP_GO:0031397_negative+regulation+of+protein+ubiquitination	0.629082	0.794789	0.429039
MF_GO:0004859_phospholipase+inhibitor+activity	0.555067	#N/A	0.555067
BP_GO:0060157_urinary+bladder+development	0.031953	#N/A	0.031953
BP_GO:0043374_CD8-positive,+alpha-beta+T+cell+differentiation	0.81942	#N/A	0.81942
BP_GO:0043393_regulation+of+protein+binding	0.457946	#N/A	0.457946
BP_GO:0060068_vagina+development	0.183811	0.0946968	0.61643
MF_GO:0008475_procollagen-lysine+5-dioxygenase+activity	0.274903	0.185687	0.590488
MF_GO:0016884_carbon-nitrogen+ligase+activity,+with+glutamine+as+amic	0.859376	0.492741	0.970673
MF_GO:0004955_prostaglandin+receptor+activity	0.235683	0.802231	0.0690711
BP_GO:0002281_macrophage+activation+during+immune+response	0.596329	0.141929	0.854738
BP_GO:0002238_response+to+molecule+of+fungal+origin	0.415528	0.366754	0.544707
CC_GO:0005640_nuclear+outer+membrane	0.277373	0.436219	0.271132
MF_GO:0004117_calmodulin-dependent+cyclic-nucleotide+phosphodiestera	0.19252	#N/A	0.19252
MF_GO:0048101_calcium-+and+calmodulin-regulated+3',5'-cyclic-GMP+pho	0.19252	#N/A	0.19252
BP_GO:0030953_spindle+astral+microtubule+organization	0.48369	0.711442	0.328392
BP_GO:0050820_positive+regulation+of+coagulation	0.495999	0.3009	0.639489
BP_GO:0001955_blood+vessel+maturation	0.773718	0.387904	0.955694
BP_GO:0060087_relaxation+of+vascular+smooth+muscle	0.459602	#N/A	0.459602
BP_GO:0042133_neurotransmitter+metabolic+process	0.990754	0.937855	0.963828
BP_GO:0042246_tissue+regeneration	0.919018	0.961581	0.467974
BP_GO:0008347_glial+cell+migration	0.936007	0.876614	0.851848
BP_GO:0003016_respiratory+system+process	0.149151	0.149151	#N/A
BP_GO:0045926_negative+regulation+of+growth	0.827033	#N/A	0.827033
BP_GO:0003014_renal+system+process	0.3903	0.709162	0.23251
BP_GO:0006725_cellular+aromatic+compound+metabolic+process	0.0902838	#N/A	0.0902838
BP_GO:0007182_common-partner+SMAD+protein+phosphorylation	0.920347	0.758059	0.890296
MF_GO:0001786_phosphatidylserine+binding	0.456752	0.147278	0.902148
MF_GO:0033829_O-fucosylpeptide+3-beta-N-acetylglucosaminyltransferase	0.320564	0.238268	0.579197
BP_GO:0050819_negative+regulation+of+coagulation	0.547951	0.547951	#N/A
BP_GO:0006651_diacylglycerol+biosynthetic+process	0.341174	0.359517	0.42094
MF_GO:0000036_acyl+carrier+activity	0.993398	0.993398	#N/A
BP_GO:0050665_hydrogen+peroxide+biosynthetic+process	0.39495	0.829633	0.158645
MF_GO:0004937_alpha1-adrenergic+receptor+activity	0.514231	0.943985	0.139985
BP_GO:0048007_antigen+processing+and+presentation,+exogenous+lipid+a	0.741429	0.660959	0.703536
CC_GO:0000015_phosphopyruvate+hydratase+complex	0.9486	0.81923	0.911835
MF_GO:0004634_phosphopyruvate+hydratase+activity	0.9486	0.81923	0.911835
MF_GO:0008559_xenobiotic-transporting+ATPase+activity	0.893164	0.652181	0.893612
BP_GO:0035051_cardiac+cell+differentiation	0.776738	0.86449	0.560995
MF_GO:0030169_low-density+lipoprotein+binding	0.976563	0.976563	#N/A
BP_GO:0043267_negative+regulation+of+potassium+ion+transport	0.911389	0.355404	0.97223
BP_GO:0045986_negative+regulation+of+smooth+muscle+contraction	0.391358	0.758005	0.20253

BP_GO:0019941_modification-dependent+protein+catabolic+process	0.474072	0.474072	#N/A
BP_GO:0002377_immunoglobulin+production	0.271119	0.857587	0.0664658
BP_GO:0007097_nuclear+migration	0.26001	0.543249	0.193711
BP_GO:0051707_response+to+other+organism	0.728083	0.684296	0.645316
BP_GO:0002481_antigen+processing+and+presentation+of+exogenous+prot	0.0574202	0.155721	0.11232
MF_GO:0016763_transferase+activity,+transferring+pentosyl+groups	0.9634	0.997483	0.19387
MF_GO:0001872_zymosan+binding	0.620699	0.620699	#N/A
BP_GO:0019432_triacylglycerol+biosynthetic+process	0.536216	0.599726	0.42094
BP_GO:0009410_response+to+xenobiotic+stimulus	0.516122	0.221567	0.723165
BP_GO:0002682_regulation+of+immune+system+process	0.948637	0.733568	0.940252
BP_GO:0006926_virus-infected+cell+apoptosis	0.794205	#N/A	0.794205
BP_GO:0016458_gene+silencing	0.547456	0.711348	0.402027
BP_GO:0001820_serotonin+secretion	0.753915	0.728818	0.659152
BP_GO:0009605_response+to+external+stimulus	0.230741	0.00137781	0.888119
MF_GO:0017160_Ral+GTPase+binding	0.430833	0.584461	0.357904
MF_GO:0005375_copper+ion+transmembrane+transporter+activity	0.429077	0.143468	0.703588
MF_GO:0004311_farnesyltranstransferase+activity	0.0903972	0.332808	0.0441015
MF_GO:0030506_ankyrin+binding	0.0429391	0.10076	0.121645
MF_GO:0003987_acetate-CoA+ligase+activity	0.119844	0.220987	0.171629
BP_GO:0006519_cellular+amino+acid+and+derivative+metabolic+process	0.978718	0.983225	0.693446
MF_GO:0046875_ephrin+receptor+binding	0.475694	0.43803	0.545872
BP_GO:0019987_negative+regulation+of+anti-apoptosis	0.950162	0.0579951	0.99912
CC_GO:0005955_calcineurin+complex	0.930922	0.822861	0.89561
BP_GO:0048935_peripheral+nervous+system+neuron+development	0.130739	0.184351	0.25065
MF_GO:0004517_nitric-oxide+synthase+activity	0.737394	0.349559	0.950131
BP_GO:0045404_positive+regulation+of+interleukin-4+biosynthetic+process	0.623694	0.605352	0.578112
MF_GO:0042301_phosphate+binding	0.781803	0.781803	#N/A
BP_GO:0002573_myeloid+leukocyte+differentiation	0.569423	0.3009	0.720229
BP_GO:0010043_response+to+zinc+ion	0.781183	0.804483	0.552183
BP_GO:0030104_water+homeostasis	0.22266	#N/A	0.22266
BP_GO:0032436_positive+regulation+of+proteasomal+ubiquitin-dependent-	0.284306	0.0943925	0.808186
CC_GO:0005774_vacuolar+membrane	0.390195	0.769287	0.194282
BP_GO:0006085_acetyl-CoA+biosynthetic+process	0.234633	0.220987	0.434336
BP_GO:0007184_SMAD+protein+nuclear+translocation	0.980393	0.758059	0.978796
MF_GO:0016309_1-phosphatidylinositol-5-phosphate+4-kinase+activity	0.15349	0.15349	#N/A
MF_GO:0004862_cAMP-dependent+protein+kinase+inhibitor+activity	0.239749	0.24046	0.35684
BP_GO:0050869_negative+regulation+of+B+cell+activation	0.0459292	#N/A	0.0459292
BP_GO:0006582_melanin+metabolic+process	0.803036	#N/A	0.803036
MF_GO:0016972_thiol+oxidase+activity	0.0164219	0.0690567	0.0551495
BP_GO:0019642_anaerobic+glycolysis	0.0133693	0.0133693	#N/A
MF_GO:0004459_L-lactate+dehydrogenase+activity	0.0133693	0.0133693	#N/A
CC_GO:0005790_smooth+endoplasmic+reticulum	0.262429	0.487826	0.145249
MF_GO:0004667_prostaglandin-D+synthase+activity	0.897077	#N/A	0.897077
BP_GO:0014068_positive+regulation+of+phosphoinositide+3-kinase+cascad	0.834536	0.908781	0.420079
MF_GO:0042166_acetylcholine+binding	0.468904	0.0597074	0.980603
BP_GO:0006527_arginine+catabolic+process	0.230987	0.230987	#N/A
BP_GO:0030323_respiratory+tube+development	0.0977629	0.0325086	0.390033

MF_GO:0004528_phosphodiesterase+I+activity	0.733826	#N/A	0.733826
BP_GO:0046685_response+to+arsenic	0.755354	0.402202	0.846606
CC_GO:0005779_integral+to+peroxisomal+membrane	0.079757	0.0984393	0.209219
BP_GO:0035088_establishment+or+maintenance+of+apical/basal+cell+polai	0.489079	0.360084	0.587112
BP_GO:0030321_transepithelial+chloride+transport	0.15914	0.588599	0.0837695
BP_GO:0046339_diacylglycerol+metabolic+process	0.342561	0.46838	0.277829
MF_GO:0004370_glycerol+kinase+activity	0.877263	0.315092	0.961035
BP_GO:0060044_negative+regulation+of+cardiac+muscle+cell+proliferation	0.52217	0.0305007	0.9183
BP_GO:0009263_deoxyribonucleotide+biosynthetic+process	0.857156	0.709162	0.820585
MF_GO:0004748_ribonucleoside-diphosphate+reductase+activity	0.857156	0.709162	0.820585
BP_GO:0051450_myoblast+proliferation	0.340401	0.221567	0.515597
MF_GO:0008384_IkappaB+kinase+activity	0.991843	#N/A	0.991843
BP_GO:0010273_detoxification+of+copper+ion	0.323216	0.455555	0.314737
MF_GO:0016671_oxidoreductase+activity,+acting+on+sulfur+group+of+don	0.766195	0.736232	0.642206
BP_GO:0050872_white+fat+cell+differentiation	0.885789	0.885789	#N/A
BP_GO:0048340_paraxial+mesoderm+morphogenesis	0.200374	0.140784	0.39481
MF_GO:0003988_acetyl-CoA+C-acyltransferase+activity	0.59612	0.59612	#N/A
BP_GO:0006620_posttranslational+protein+targeting+to+membrane	0.899039	0.892585	0.675606
CC_GO:0005782_peroxisomal+matrix	0.293508	0.293508	#N/A
MF_GO:0003865_3-oxo-5-alpha-steroid+4-dehydrogenase+activity	0.571849	0.61745	0.456736
MF_GO:0047184_1-acylglycerophosphocholine+O-acyltransferase+activity	0.0630877	0.094379	0.215123
BP_GO:0002862_negative+regulation+of+inflammatory+response+to+antige	0.67031	0.67031	#N/A
MF_GO:0016986_transcription+initiation+factor+activity	0.115177	0.115177	#N/A
BP_GO:0032400_melanosome+localization	0.403231	0.499373	0.382604
CC_GO:0000940_outer+kinetochore+of+condensed+chromosome	0.308238	#N/A	0.308238
BP_GO:0019724_B+cell+mediated+immunity	0.16569	0.568808	0.0948086
BP_GO:0051891_positive+regulation+of+cardioblast+differentiation	0.207467	0.207467	#N/A
BP_GO:0046325_negative+regulation+of+glucose+import	0.704955	0.539607	0.785825
BP_GO:0045662_negative+regulation+of+myoblast+differentiation	0.307159	0.307159	#N/A
MF_GO:0015238_drug+transporter+activity	0.415727	0.369115	0.541545
BP_GO:0048545_response+to+steroid+hormone+stimulus	0.427325	#N/A	0.427325
MF_GO:0019797_procollagen-proline+3-dioxygenase+activity	0.569601	0.569601	#N/A
BP_GO:0006560_proline+metabolic+process	0.236339	0.236339	#N/A
MF_GO:0004700_atypical+protein+kinase+C+activity	0.707235	0.893842	0.415079
MF_GO:0004346_glucose-6-phosphatase+activity	0.91354	0.91354	#N/A
BP_GO:0045475_locomotor+rhythm	0.183876	0.441589	0.158989
BP_GO:0051496_positive+regulation+of+stress+fiber+formation	0.676651	0.822212	0.304225
BP_GO:0031558_induction+of+apoptosis+in+response+to+chemical+stimulu	0.308198	0.743235	0.141099
BP_GO:0021860_pyramidal+neuron+development	0.612463	0.552278	0.621354
BP_GO:0006972_hyperosmotic+response	0.623272	0.110851	0.894224
BP_GO:0018401_peptidyl-proline+hydroxylation+to+4-hydroxy-L-proline	0.261666	0.261666	#N/A
MF_GO:0004663_Rab-protein+geranylgeranyltransferase+activity	0.192407	0.230371	0.29391
MF_GO:0004069_aspartate+transaminase+activity	0.45127	0.45127	#N/A
MF_GO:0008898_homocysteine+S-methyltransferase+activity	0.714759	#N/A	0.714759
MF_GO:0016866_intramolecular+transferase+activity	0.901509	0.760517	0.859708
MF_GO:0016286_small+conductance+calcium-activated+potassium+channe	0.042484	0.0297686	0.375421
CC_GO:0005747_mitochondrial+respiratory+chain+complex+I	0.316713	0.316713	#N/A

MF_GO:0019976_interleukin-2+binding	0.56544	0.638464	0.414718
BP_GO:0009100_glycoprotein+metabolic+process	0.601439	0.500067	0.671821
BP_GO:0010575_positive+regulation+vascular+endothelial+growth+factor+p	0.175217	0.169653	0.395488
BP_GO:0006686_sphingomyelin+biosynthetic+process	0.93158	0.817732	0.902025
BP_GO:0043306_positive+regulation+of+mast+cell+degranulation	0.973356	0.728818	0.973527
BP_GO:0030049_muscle+filament+sliding	0.973082	0.973082	#N/A
CC_GO:0016461_unconventional+myosin+complex	0.973082	0.973082	#N/A
BP_GO:0007195_dopamine+receptor,+adenylate+cyclase+inhibiting+pathwa	0.380699	#N/A	0.380699
BP_GO:0001996_positive+regulation+of+heart+rate+by+epinephrine-norepi	0.0899559	0.213767	0.139985
BP_GO:0001997_positive+regulation+of+the+force+of+heart+contraction+b	0.0899559	0.213767	0.139985
MF_GO:0016155_formyltetrahydrofolate+dehydrogenase+activity	0.240293	0.544922	0.172776
BP_GO:0016559_peroxisome+fission	0.122028	0.191728	0.216575
BP_GO:0000320_re-entry+into+mitotic+cell+cycle	0.837113	0.734274	0.776556
MF_GO:0045502_dynein+binding	0.808195	0.48668	0.940064
BP_GO:0007213_acetylcholine+receptor+signaling,+muscarinic+pathway	0.911169	0.533129	0.944288
BP_GO:0016117_carotenoid+biosynthetic+process	0.957765	0.711736	0.985982
MF_GO:0019209_kinase+activator+activity	0.239759	0.239759	#N/A
BP_GO:0045216_cell-cell+junction+organization	0.882412	0.606146	0.952947
BP_GO:0001844_protein+insertion+into+mitochondrial+membrane+during+	0.114382	0.0579951	0.358917
BP_GO:0007095_mitotic+cell+cycle+G2/M+transition+DNA+damage+checkp	0.261587	0.0329778	0.698241
BP_GO:0043068_positive+regulation+of+programmed+cell+death	0.71019	0.71019	#N/A
BP_GO:0045617_negative+regulation+of+keratinocyte+differentiation	0.124305	#N/A	0.124305
MF_GO:0017076_purine+nucleotide+binding	0.569763	0.61256	0.505215
BP_GO:0019884_antigen+processing+and+presentation+of+exogenous+anti	0.425431	0.468626	0.415342
BP_GO:0035234_germ+cell+programmed+cell+death	0.231729	0.0579951	0.584948
BP_GO:0007016_cytoskeletal+anchoring+at+plasma+membrane	0.229049	0.229049	#N/A
BP_GO:0045022_early+endosome+to+late+endosome+transport	0.649295	0.224249	0.842758
BP_GO:0032469_endoplasmic+reticulum+calcium+ion+homeostasis	0.911275	0.599953	0.929529
MF_GO:0051020_GTPase+binding	0.135659	#N/A	0.135659
BP_GO:0048268_clathrin+coat+assembly	0.658727	0.905199	0.334662
MF_GO:0008502_melatonin+receptor+activity	0.932458	0.907448	0.762168
MF_GO:0004712_protein+serine/threonine/tyrosine+kinase+activity	0.929096	0.483126	0.995373
BP_GO:0001553_luteinization	0.741249	0.741249	#N/A
MF_GO:0019208_phosphatase+regulator+activity	0.110894	#N/A	0.110894
BP_GO:0007090_regulation+of+S+phase+of+mitotic+cell+cycle	0.744506	0.744506	#N/A
MF_GO:0030371_translation+repressor+activity	0.125818	0.292411	0.112869
BP_GO:0006929_substrate-bound+cell+migration	0.3711	0.190358	0.748583
MF_GO:0032947_protein+complex+scaffold	0.634728	#N/A	0.634728
MF_GO:0016151_nickel+ion+binding	0.25558	0.96662	0.0178094
CC_GO:0042598_vesicular+fraction	0.884257	#N/A	0.884257
BP_GO:0007034_vacuolar+transport	0.190218	0.441316	0.166213
CC_GO:0030904_retromer+complex	0.190218	0.441316	0.166213
MF_GO:0003896_DNA+primase+activity	0.0787853	#N/A	0.0787853
BP_GO:0001812_positive+regulation+of+type+I+hypersensitivity	0.901741	#N/A	0.901741
CC_GO:0030118_clathrin+coat	0.217706	#N/A	0.217706
MF_GO:0000293_ferric-chelate+reductase+activity	0.536439	0.963823	0.123215
CC_GO:0001891_phagocytic+cup	0.526993	0.526993	#N/A

BP_GO:0048488_synaptic+vesicle+endocytosis	0.782352	0.943956	0.433022
BP_GO:0009448_gamma-aminobutyric+acid+metabolic+process	0.710201	0.710201	#N/A
MF_GO:0016817_hydrolase+activity,+acting+on+acid+anhydrides	0.187229	#N/A	0.187229
BP_GO:0001675_acrosome+formation	0.494235	0.8393	0.236066
BP_GO:0042989_sequestering+of+actin+monomers	0.519792	0.519792	#N/A
BP_GO:0009181_purine+ribonucleoside+diphosphate+catabolic+process	0.318815	0.160922	0.549495
MF_GO:0008454_alpha-1,3-mannosylglycoprotein+4-beta-N-acetylglucosam	0.163821	0.269409	0.204674
BP_GO:0030502_negative+regulation+of+bone+mineralization	0.988453	0.954105	0.939291
MF_GO:0000309_nicotinamide-nucleotide+adenylyltransferase+activity	0.49019	0.293198	0.766311
MF_GO:0019871_sodium+channel+inhibitor+activity	0.976988	0.8929	0.955198
MF_GO:0004597_peptide-aspartate+beta-dioxygenase+activity	0.284556	0.156878	0.506298
BP_GO:0030510_regulation+of+BMP+signaling+pathway	0.15142	0.13633	0.313743
BP_GO:0002320_lymphoid+progenitor+cell+differentiation	0.79375	#N/A	0.79375
BP_GO:0008090_retrograde+axon+cargo+transport	0.795337	0.73772	0.701507
BP_GO:0033209_tumor+necrosis+factor-mediated+signaling+pathway	0.836682	0.836682	#N/A
BP_GO:0051090_regulation+of+transcription+factor+activity	0.00933402	0.0166581	0.144105
MF_GO:0005094_Rho+GDP-dissociation+inhibitor+activity	0.528541	0.519905	0.521393
BP_GO:0060009_Sertoli+cell+development	0.137504	0.141752	0.354599
BP_GO:0030518_steroid+hormone+receptor+signaling+pathway	0.586251	0.254933	0.904725
MF_GO:0004849_uridine+kinase+activity	0.0614697	0.0517448	0.35647
MF_GO:0004558_alpha-glucosidase+activity	0.483695	0.128717	0.937024
MF_GO:0001882_nucleoside+binding	0.233743	0.536102	0.0829919
BP_GO:0033143_regulation+of+steroid+hormone+receptor+signaling+pathv	0.45817	0.988244	0.0417389
CC_GO:0030136_clathrin-coated+vesicle	0.880665	0.905199	0.696675
BP_GO:0045055_regulated+secretory+pathway	0.570246	0.274867	0.875358
MF_GO:0043560_insulin+receptor+substrate+binding	0.417745	#N/A	0.417745
BP_GO:0030091_protein+repair	0.217506	0.217506	#N/A
MF_GO:0000318_protein-methionine-R-oxide+reductase+activity	0.217506	0.217506	#N/A
BP_GO:0001953_negative+regulation+of+cell-matrix+adhesion	0.402195	0.578197	0.329023
BP_GO:0030224_monocyte+differentiation	0.829092	0.911478	0.396397
BP_GO:0006670_sphingosine+metabolic+process	0.102893	0.26512	0.0964228
MF_GO:0050508_glucuronosyl-N-acetylglucosaminyl-proteoglycan+4-alpha-	0.082502	0.082502	#N/A
MF_GO:0042799_histone+lysine+N-methyltransferase+activity+(H4-K20+spe	0.958887	0.679918	0.963858
MF_GO:0004385_guanylate+kinase+activity	0.0611326	0.191437	0.101242
BP_GO:0040012_regulation+of+locomotion	0.274257	#N/A	0.274257
BP_GO:0002636_positive+regulation+of+germinal+center+formation	0.639628	0.683118	0.478264
BP_GO:0001842_neural+fold+formation	0.996643	0.996643	#N/A
MF_GO:0015027_coreceptor,+soluble+ligand+activity	0.163671	0.163671	#N/A
BP_GO:0032147_activation+of+protein+kinase+activity	0.710096	0.522843	0.738121
MF_GO:0016505_apoptotic+protease+activator+activity	0.899978	0.899978	#N/A
MF_GO:0003726_double-stranded+RNA+adenosine+deaminase+activity	0.49591	0.12697	0.944653
BP_GO:0018125_peptidyl-cysteine+methylation	0.834059	0.561263	0.859745
MF_GO:0005548_phospholipid+transporter+activity	0.117763	0.381015	0.0521211
BP_GO:0006855_multidrug+transport	0.25254	0.199046	0.413707
MF_GO:0047631_ADP-ribose+diphosphatase+activity	0.83573	0.945531	0.524882
MF_GO:0004322_ferroxidase+activity	0.436863	0.353431	0.601339
BP_GO:0060056_mammary+gland+involution	0.178026	0.113777	0.543275

MF_GO:0008409_5'-3'+exonuclease+activity	0.120506	0.551978	0.0134323
BP_GO:0048820_hair+follicle+maturation	0.56047	0.815529	0.157129
BP_GO:0044249_cellular+biosynthetic+process	0.305401	0.305401	#N/A
BP_GO:0006542_glutamine+biosynthetic+process	0.972873	0.972873	#N/A
MF_GO:0004356_glutamate-ammonia+ligase+activity	0.972873	0.972873	#N/A
MF_GO:0004430_1-phosphatidylinositol+4-kinase+activity	0.835582	0.735998	0.787617
BP_GO:0007089_traversing+start+control+point+of+mitotic+cell+cycle	0.282084	0.598554	0.188771
BP_GO:0007252_I-kappaB+phosphorylation	0.974006	#N/A	0.974006
BP_GO:0016584_nucleosome+positioning	0.0538882	0.0839363	0.160293
MF_GO:0005031_tumor+necrosis+factor+receptor+activity	0.980771	0.992344	0.561591
CC_GO:0031512_motile+primary+cilium	0.807366	0.807366	#N/A
MF_GO:0035197_siRNA+binding	0.460763	0.495511	0.438688
BP_GO:0007144_female+meiosis+I	0.377725	0.631475	0.155452
MF_GO:0016149_translation+release+factor+activity,+codon+specific	0.807631	#N/A	0.807631
BP_GO:0006808_regulation+of+nitrogen+utilization	0.650723	0.339116	0.89561
BP_GO:0006290_pyrimidine+dimer+repair	0.537785	0.534607	0.51662
BP_GO:0044265_cellular+macromolecule+catabolic+process	0.560335	0.755189	0.23775
BP_GO:0030513_positive+regulation+of+BMP+signaling+pathway	0.913762	#N/A	0.913762
MF_GO:0008173_RNA+methyltransferase+activity	0.533408	0.0815483	0.862054
BP_GO:0006891_intra-Golgi+vesicle-mediated+transport	0.459803	0.574478	0.330034
MF_GO:0005528_FK506+binding	0.675699	0.835699	0.276948
BP_GO:0048302_regulation+of+isotype+switching+to+IgG+isotypes	0.237276	0.237276	#N/A
BP_GO:0001757_somite+specification	0.814721	#N/A	0.814721
BP_GO:0006166_purine+ribonucleoside+salvage	0.289611	0.289611	#N/A
MF_GO:0003886_DNA+(cytosine-5-)-methyltransferase+activity	0.725195	0.725195	#N/A
MF_GO:0005229_intracellular+calcium+activated+chloride+channel+activity	0.686597	0.901407	0.375681
BP_GO:0040037_negative+regulation+of+fibroblast+growth+factor+recepto	0.284291	0.0277487	0.744387
BP_GO:0006000_fructose+metabolic+process	0.620599	0.625552	0.531576
BP_GO:0006003_fructose+2,6-bisphosphate+metabolic+process	0.620599	0.625552	0.531576
MF_GO:0003873_6-phosphofructo-2-kinase+activity	0.620599	0.625552	0.531576
MF_GO:0004331_fructose-2,6-bisphosphate+2-phosphatase+activity	0.620599	0.625552	0.531576
BP_GO:0046549_retinal+cone+cell+development	0.34677	0.310968	0.446937
MF_GO:0004965_GABA-B+receptor+activity	0.488095	0.672278	0.36241
BP_GO:0051155_positive+regulation+of+striated+muscle+cell+differentiatio	0.768059	0.768059	#N/A
MF_GO:0048038_quinone+binding	0.860306	0.934209	0.397785
MF_GO:0008568_microtubule-severing+ATPase+activity	0.41496	0.621757	0.209035
BP_GO:0031000_response+to+caffeine	0.655915	0.655915	#N/A
BP_GO:0001994_norepinephrine-epinephrine+vasoconstriction+involved+in	0.37289	0.943985	0.0641272
BP_GO:0043367_CD4-positive,+alpha+beta+T+cell+differentiation	0.779135	#N/A	0.779135
MF_GO:0003918_DNA+topoisomerase+(ATP-hydrolyzing)+activity	0.539991	#N/A	0.539991
BP_GO:0034101_erythrocyte+homeostasis	0.13925	0.319321	0.112869
BP_GO:0021522_spinal+cord+motor+neuron+differentiation	0.985173	0.767746	0.984037
BP_GO:0050806_positive+regulation+of+synaptic+transmission	0.0439004	0.17312	0.0772869
BP_GO:0048664_neuron+fate+determination	0.155889	0.157991	0.369617
MF_GO:0005220_inositol+1,4,5-triphosphate-sensitive+calcium-release+cha	0.900584	0.599953	0.918375
MF_GO:0008095_inositol-1,4,5-triphosphate+receptor+activity	0.900584	0.599953	0.918375
BP_GO:0045446_endothelial+cell+differentiation	0.65598	0.0872244	0.926832

MF_GO:0016891_endoribonuclease+activity,+producing+5'-phosphomonoes	0.71099	0.884488	0.434051
CC_GO:0008537_proteasome+activator+complex	0.687722	0.687722	#N/A
MF_GO:0008538_proteasome+activator+activity	0.687722	0.687722	#N/A
MF_GO:0004616_phosphogluconate+dehydrogenase+(decarboxylating)+act	0.956421	0.956421	#N/A
BP_GO:0030643_cellular+phosphate+ion+homeostasis	0.839052	0.881736	0.646386
BP_GO:0007603_phototransduction,+visible+light	0.864343	0.683601	0.84352
BP_GO:0006491_N-glycan+processing	0.580654	0.575201	0.533687
BP_GO:0048294_negative+regulation+of+isotype+switching+to+IgE+isotype:	0.417095	0.417095	#N/A
BP_GO:0042698_ovulation+cycle	0.395563	0.634637	0.285168
MF_GO:0004802_transketolase+activity	0.601036	0.756707	0.429146
BP_GO:0032402_melanosome+transport	0.261	0.246471	0.382604
BP_GO:0009607_response+to+biotic+stimulus	0.568888	0.142894	0.83344
MF_GO:0008073_ornithine+decarboxylase+inhibitor+activity	0.000693123	0.00167475	0.0827742
BP_GO:0006435_threonyl-tRNA+aminoacylation	0.444378	0.171323	0.864231
MF_GO:0004829_threonine-tRNA+ligase+activity	0.444378	0.171323	0.864231
BP_GO:0040023_establishment+of+nucleus+localization	0.316312	0.134659	0.577856
BP_GO:0046903_secretion	0.0931958	0.0931958	#N/A
BP_GO:0007220_Notch+receptor+processing	0.203131	#N/A	0.203131
MF_GO:0003953_NAD++nucleosidase+activity	0.959265	0.926038	0.866429
BP_GO:0009612_response+to+mechanical+stimulus	0.821667	0.849886	0.552183
BP_GO:0031116_positive+regulation+of+microtubule+polymerization	0.275666	0.43507	0.269748
CC_GO:0042405_nuclear+inclusion+body	0.602251	0.289259	0.761325
BP_GO:0007412_axon+target+recognition	0.14897	0.0232381	0.553464
CC_GO:0017133_mitochondrial+electron+transfer+flavoprotein+complex	0.137958	0.137958	#N/A
MF_GO:0004800_thyroxine+5'-deiodinase+activity	0.739479	0.725943	0.64096
MF_GO:0004758_serine+C-palmitoyltransferase+activity	0.773123	0.826662	0.599359
MF_GO:0004566_beta-glucuronidase+activity	0.283795	0.388363	0.278235
BP_GO:0021895_cerebral+cortex+neuron+differentiation	0.362434	0.362434	#N/A
BP_GO:0007215_glutamate+signaling+pathway	0.819039	0.819039	#N/A
BP_GO:0045747_positive+regulation+of+Notch+signaling+pathway	0.955403	0.972765	0.588437
BP_GO:0045629_negative+regulation+of+T-helper+2+cell+differentiation	0.869527	0.869527	#N/A
BP_GO:0015695_organic+cation+transport	0.773189	0.973118	0.32751
MF_GO:0003724_RNA+helicase+activity	0.963923	0.87352	0.932713
BP_GO:0045217_cell-cell+junction+maintenance	0.220807	0.220807	#N/A
BP_GO:0007000_nucleolus+organization	0.486718	0.486718	#N/A
CC_GO:0000109_nucleotide-excision+repair+complex	0.996133	0.829683	0.995161
BP_GO:0030644_cellular+chloride+ion+homeostasis	0.969175	0.78526	0.958126
CC_GO:0032421_stereocilium+bundle	0.606634	0.630096	0.53844
CC_GO:0000802_transverse+filament	0.475658	0.29041	0.62404
BP_GO:0050779_RNA+destabilization	0.68165	0.694276	0.58671
BP_GO:0045884_regulation+of+survival+gene+product+expression	0.34814	0.34814	#N/A
BP_GO:0048676_axon+extension+involved+in+development	0.699512	0.083328	0.947277
BP_GO:0031623_receptor+internalization	0.916518	0.726911	0.938099
CC_GO:0030285_integral+to+synaptic+vesicle+membrane	0.619648	#N/A	0.619648
BP_GO:0035284_brain+segmentation	0.355685	0.68807	0.0912299
CC_GO:0001741_XY+body	0.3435	0.369561	0.410476
BP_GO:0007199_G-protein+signaling,+coupled+to+cGMP+nucleotide+secon	0.778657	0.778657	#N/A

BP_GO:0010025_wax+biosynthetic+process	0.0596502	0.716977	0.0103605
BP_GO:0048678_response+to+axon+injury	0.108801	0.081309	0.436528
BP_GO:0030521_androgen+receptor+signaling+pathway	0.723187	0.90462	0.204996
MF_GO:0004705_JUN+kinase+activity	0.506922	0.743235	0.329685
BP_GO:0051493_regulation+of+cytoskeleton+organization	0.955257	0.860857	0.90529
BP_GO:0021575_hindbrain+morphogenesis	0.333747	0.333747	#N/A
BP_GO:0000087_M+phase+of+mitotic+cell+cycle	0.523126	0.356043	0.630174
MF_GO:0005104_fibroblast+growth+factor+receptor+binding	0.581049	0.881736	0.278681
BP_GO:0042326_negative+regulation+of+phosphorylation	0.407863	0.289259	0.542965
BP_GO:0048513_organ+development	0.336572	0.336572	#N/A
MF_GO:0008081_phosphoric+diester+hydrolase+activity	0.985259	0.985259	#N/A
BP_GO:0007256_activation+of+JNKK+activity	0.145923	#N/A	0.145923
MF_GO:0050811_GABA+receptor+binding	0.739815	0.832452	0.541914
BP_GO:0006467_protein+thiol-disulfide+exchange	0.227099	0.401885	0.229537
CC_GO:0031965_nuclear+membrane	0.890638	0.731331	0.89561
MF_GO:0004687_myosin+light+chain+kinase+activity	0.268835	0.268835	#N/A
BP_GO:0021571_rhombomere+5+development	0.0681629	#N/A	0.0681629
BP_GO:0006420_arginyl-tRNA+aminoacylation	0.0351488	0.0346156	0.176063
MF_GO:0004814_arginine-tRNA+ligase+activity	0.0351488	0.0346156	0.176063
BP_GO:0031529_ruffle+organization	0.904438	0.670682	0.901164
BP_GO:0051298_centrosome+duplication	0.461848	0.71851	0.299345
BP_GO:0006264_mitochondrial+DNA+replication	0.216628	0.216628	#N/A
MF_GO:0005172_vascular+endothelial+growth+factor+receptor+binding	0.488454	0.316759	0.733722
BP_GO:0045947_negative+regulation+of+translational+initiation	0.281354	0.281354	#N/A
BP_GO:0000380_alternative+nuclear+mRNA+splicing,+via+spliceosome	0.76351	0.705683	0.68361
BP_GO:0021819_layer+formation+in+the+cerebral+cortex	0.863151	0.900378	0.531922
BP_GO:0051457_maintenance+of+protein+location+in+nucleus	0.738078	0.807717	0.450024
BP_GO:0048557_embryonic+digestive+tract+morphogenesis	0.0211594	0.0240571	0.138409
MF_GO:0042162_telomeric+DNA+binding	0.575251	#N/A	0.575251
BP_GO:0045197_establishment+or+maintenance+of+epithelial+cell+apical/l	0.547282	#N/A	0.547282
BP_GO:0017158_regulation+of+calcium+ion-dependent+exocytosis	0.950048	0.60096	0.993542
BP_GO:0031077_post-embryonic+camera-type+eye+development	0.417519	0.155264	0.678209
MF_GO:0005078_MAP-kinase+scaffold+activity	0.547331	0.547331	#N/A
BP_GO:0030072_peptide+hormone+secretion	0.69453	#N/A	0.69453
MF_GO:0004887_thyroid+hormone+receptor+activity	0.274493	0.242396	0.405442
BP_GO:0006120_mitochondrial+electron+transport,+NADH+to+ubiquinone	0.279781	0.279781	#N/A
MF_GO:0003708_retinoic+acid+receptor+activity	0.916826	0.916826	#N/A
MF_GO:0003910_DNA+ligase+(ATP)+activity	0.156765	0.264289	0.215367
BP_GO:0030219_megakaryocyte+differentiation	0.771064	0.911478	0.266627
BP_GO:0031398_positive+regulation+of+protein+ubiquitination	0.0416118	0.00946933	0.625298
BP_GO:0060218_hemopoietic+stem+cell+differentiation	0.353087	0.255387	0.60926
BP_GO:0042633_hair+cycle	0.359738	0.517206	0.247536
BP_GO:0051567_histone+H3-K9+methylation	0.310778	0.337259	0.397267
MF_GO:0008413_8-oxo-7,8-dihydroguanine+triphosphatase+activity	0.950697	0.950697	#N/A
BP_GO:0008595_determination+of+anterior/posterior+axis,+embryo	0.432064	0.315264	0.552101
BP_GO:0048645_organ+formation	0.653482	0.700186	0.476653
MF_GO:0004739_pyruvate+dehydrogenase+(acetyl-transferring)+activity	0.747835	0.480578	0.889646

BP_GO:0050856_regulation+of+T+cell+receptor+signaling+pathway	0.776971	0.329627	0.973949
CC_GO:0031932_TORC2+complex	0.566443	0.670682	0.4572
MF_GO:0016160_amylase+activity	0.571127	#N/A	0.571127
MF_GO:0005275_amine+transmembrane+transporter+activity	0.564862	0.973118	0.122135
MF_GO:0004946_bombesin+receptor+activity	0.139814	#N/A	0.139814
BP_GO:0021800_cerebral+cortex+tangential+migration	0.990581	0.589792	0.996689
MF_GO:0019237_centromeric+DNA+binding	0.587895	0.800636	0.372643
BP_GO:0006997_nucleus+organization	0.233213	0.0824356	0.536286
CC_GO:0031931_TORC1+complex	0.78769	0.670682	0.747044
BP_GO:0031167_rRNA+methylation	0.95378	0.95378	#N/A
BP_GO:0006323_DNA+packaging	0.997088	0.830271	0.99654
BP_GO:0001831_trophectodermal+cellular+morphogenesis	0.547497	0.32766	0.798919
BP_GO:0002455_humoral+immune+response+mediated+by+circulating+imr	0.0638179	#N/A	0.0638179
MF_GO:0001537_N-acetylgalactosamine+4-O-sulfotransferase+activity	0.801307	0.559461	0.894978
MF_GO:0051019_mitogen-activated+protein+kinase+binding	0.867715	0.724396	0.861855
BP_GO:0048012_hepatocyte+growth+factor+receptor+signaling+pathway	0.577663	#N/A	0.577663
CC_GO:0009331_glycerol-3-phosphate+dehydrogenase+complex	0.583288	0.583288	#N/A
BP_GO:0032609_interferon-gamma+production	0.858763	0.858763	#N/A
BP_GO:0015809_arginine+transport	0.706046	0.932471	0.120136
MF_GO:0015181_arginine+transmembrane+transporter+activity	0.706046	0.932471	0.120136
BP_GO:0000086_G2/M+transition+of+mitotic+cell+cycle	0.936023	0.816228	0.889988
BP_GO:0048661_positive+regulation+of+smooth+muscle+cell+proliferation	0.191161	0.492752	0.0686472
BP_GO:0050767_regulation+of+neurogenesis	0.0910126	0.14517	0.207615
BP_GO:0030538_embryonic+genitalia+morphogenesis	0.295983	#N/A	0.295983
BP_GO:0048853_forebrain+morphogenesis	0.0572416	#N/A	0.0572416
BP_GO:0006704_glucocorticoid+biosynthetic+process	0.811753	0.811753	#N/A
BP_GO:0046470_phosphatidylcholine+metabolic+process	0.306467	0.306467	#N/A
BP_GO:0033153_T+cell+receptor+V(D)J+recombination	0.476503	#N/A	0.476503
BP_GO:0042523_positive+regulation+of+tyrosine+phosphorylation+of+Stat5	0.73024	#N/A	0.73024
BP_GO:0060078_regulation+of+postsynaptic+membrane+potential	0.926848	0.983741	0.305857
MF_GO:0008107_galactoside+2-alpha-L-fucosyltransferase+activity	0.285543	#N/A	0.285543
BP_GO:0060084_synaptic+transmission+involved+in+micturition	0.817507	0.854831	0.64106
BP_GO:0051963_regulation+of+synaptogenesis	0.560707	0.854831	0.287456
BP_GO:0032228_regulation+of+synaptic+transmission,+GABAergic	0.79963	0.841401	0.626045
BP_GO:0008594_photoreceptor+cell+morphogenesis	0.292807	0.598387	0.199442
MF_GO:0017069_snRNA+binding	0.554783	0.546679	0.529065
CC_GO:0005672_transcription+factor+TFIIA+complex	0.366426	0.373495	0.446532
BP_GO:0060083_smooth+muscle+contraction+involved+in+micturition	0.695318	0.754034	0.555523
BP_GO:0042637_catagen	0.59447	0.444684	0.652216
BP_GO:0051101_regulation+of+DNA+binding	0.252507	0.401716	0.211405
BP_GO:0000212_meiotic+spindle+organization	0.458617	0.257595	0.770441
CC_GO:0044445_cytosolic+part	0.0751911	0.0751911	#N/A
BP_GO:0045807_positive+regulation+of+endocytosis	0.197525	0.480383	0.157132
BP_GO:0001574_ganglioside+biosynthetic+process	0.0963033	0.110486	0.299957
BP_GO:0030903_notochord+development	0.875864	#N/A	0.875864
BP_GO:0006623_protein+targeting+to+vacuole	0.0520454	0.0680954	0.240004
MF_GO:0004938_alpha2-adrenergic+receptor+activity	0.529949	0.357278	0.74128

BP_GO:0048563_post-embryonic+organ+morphogenesis	0.475	0.475	#N/A
MF_GO:0005098_Ran+GTPase+activator+activity	0.322649	0.431457	0.290383
BP_GO:0050871_positive+regulation+of+B+cell+activation	0.227341	#N/A	0.227341
MF_GO:0008656_caspase+activator+activity	0.271416	0.271416	#N/A
BP_GO:0048712_negative+regulation+of+astrocyte+differentiation	0.0842622	0.0721623	0.374784
BP_GO:0006426_glycyl-tRNA+aminoacylation	0.187331	0.187331	#N/A
MF_GO:0004820_glycine-tRNA+ligase+activity	0.187331	0.187331	#N/A
BP_GO:0000002_mitochondrial+genome+maintenance	0.0841731	0.1737	0.145589
BP_GO:0017000_antibiotic+biosynthetic+process	0.43043	0.684598	0.162961
BP_GO:0042471_ear+morphogenesis	0.521641	0.594038	0.404345
BP_GO:0007132_meiotic+metaphase+I	0.200622	0.200622	#N/A
MF_GO:0008020_G-protein+coupled+photoreceptor+activity	0.711959	0.785931	0.439549
BP_GO:0007217_tachykinin+signaling+pathway	0.367671	0.440075	0.379423
BP_GO:0001706_endoderm+formation	0.880005	0.880005	#N/A
BP_GO:0060164_regulation+of+timing+of+neuron+differentiation	0.299833	0.299833	#N/A
MF_GO:0005227_calcium+activated+cation+channel+activity	0.625427	0.19146	0.843514
BP_GO:0010553_negative+regulation+of+specific+transcription+from+RNA+	0.576242	#N/A	0.576242
CC_GO:0034706_sodium+channel+complex	0.634299	#N/A	0.634299
BP_GO:0050672_negative+regulation+of+lymphocyte+proliferation	0.853726	0.503097	0.96492
MF_GO:0015321_sodium-dependent+phosphate+transmembrane+transpor	0.727655	0.59004	0.71931
BP_GO:0006207_'de+novo'+pyrimidine+base+biosynthetic+process	0.170652	0.111224	0.531064
MF_GO:0015267_channel+activity	0.462676	#N/A	0.462676
CC_GO:0008023_transcription+elongation+factor+complex	0.680545	0.0771753	0.943219
BP_GO:0051965_positive+regulation+of+synaptogenesis	0.878856	0.878856	#N/A
MF_GO:0031403_lithium+ion+binding	0.249638	0.0701433	0.82004
BP_GO:0010825_positive+regulation+of+centrosome+duplication	0.944562	#N/A	0.944562
BP_GO:0006388_tRNA+splicing,+via+endonucleolytic+cleavage+and+ligatior	0.0870741	0.0270651	0.644475
MF_GO:0008241_peptidyl-dipeptidase+activity	0.294946	0.689679	0.156276
BP_GO:0007442_hindgut+morphogenesis	0.862218	#N/A	0.862218
BP_GO:0030718_germ-line+stem+cell+maintenance	0.387428	0.200878	0.596078
BP_GO:0042421_norepinephrine+biosynthetic+process	0.909911	#N/A	0.909911
MF_GO:0050780_dopamine+receptor+binding	0.727222	0.567798	0.789574
MF_GO:0005519_cytoskeletal+regulatory+protein+binding	0.864565	#N/A	0.864565
BP_GO:0021913_regulation+of+transcription+from+RNA+polymerase+II+prc	0.975404	0.904397	0.931063
BP_GO:0001710_mesodermal+cell+fate+commitment	0.327786	0.219348	0.626639
BP_GO:0001976_regulation+of+systemic+arterial+blood+pressure+by+neurc	0.54844	0.423758	0.612282
MF_GO:0004305_ethanolamine+kinase+activity	0.480246	0.349768	0.677272
BP_GO:0042832_defense+response+to+protozoan	0.268298	#N/A	0.268298
BP_GO:0021543_pallium+development	0.0299915	0.0557691	0.119614
BP_GO:0031642_negative+regulation+of+myelination	0.313317	0.357756	0.367844
BP_GO:0031503_protein+complex+localization	0.635035	0.380036	0.84835
MF_GO:0001602_pancreatic+polypeptide+receptor+activity	0.0966852	0.311292	0.106733
BP_GO:0042448_progesterone+metabolic+process	0.540813	0.567289	0.502305
MF_GO:0004461_lactose+synthase+activity	0.244777	0.172464	0.429492
MF_GO:0015087_cobalt+ion+transmembrane+transporter+activity	0.631203	0.55261	0.62432
MF_GO:0032137_guanine/thymine+mispair+binding	0.333255	0.381584	0.376665
BP_GO:0045089_positive+regulation+of+innate+immune+response	0.633108	#N/A	0.633108

BP_GO:0043586_tongue+development	0.671265	0.679948	0.584069
BP_GO:0032651_regulation+of+interleukin-1+beta+production	0.0658025	0.135602	0.145964
BP_GO:0002666_positive+regulation+of+T+cell+tolerance+induction	0.785826	0.763538	0.639259
BP_GO:0016080_synaptic+vesicle+targeting	0.179197	#N/A	0.179197
CC_GO:0032040_small-subunit+processome	0.732823	#N/A	0.732823
BP_GO:0006883_cellular+sodium+ion+homeostasis	0.624139	#N/A	0.624139
MF_GO:0001733_galactosylceramide+sulfotransferase+activity	0.890728	0.761312	0.84231
BP_GO:0042711_maternal+behavior	0.412779	0.570101	0.346564
BP_GO:0001894_tissue+homeostasis	0.944952	0.839284	0.895256
MF_GO:0004035_alkaline+phosphatase+activity	0.269107	0.319234	0.344274
MF_GO:0051879_Hsp90+protein+binding	0.0743809	0.0667853	0.352666
BP_GO:0035067_negative+regulation+of+histone+acetylation	0.54252	0.54252	#N/A
BP_GO:0021570_rhombomere+4+development	0.256146	#N/A	0.256146
BP_GO:0006405_RNA+export+from+nucleus	0.772149	0.624378	0.754665
BP_GO:0016242_negative+regulation+of+macroautophagy	0.961749	0.919212	0.861538
BP_GO:0046649_lymphocyte+activation	0.893752	0.922309	0.559486
MF_GO:0003917_DNA+topoisomerase+type+I+activity	0.935041	#N/A	0.935041
MF_GO:0017025_TATA-binding+protein+binding	0.0377862	0.0377862	#N/A
CC_GO:0042765_GPI-anchor+transamidase+complex	0.540276	0.0293713	0.928024
BP_GO:0048755_branching+morphogenesis+of+a+nerve	0.60401	0.232774	0.799376
CC_GO:0044442_microtubule-based+flagellum+part	0.294467	0.464588	0.274739
MF_GO:0016615_malate+dehydrogenase+activity	0.103202	0.136678	0.261704
MF_GO:0004719_protein-L-isoaspartate+(D-aspartate)+O-methyltransferase	0.808544	0.713481	0.748506
BP_GO:0009247_glycolipid+biosynthetic+process	0.733949	0.522724	0.765733
BP_GO:0006024_glycosaminoglycan+biosynthetic+process	0.329935	0.299078	0.434174
BP_GO:0045859_regulation+of+protein+kinase+activity	0.883305	0.883305	#N/A
BP_GO:0006177_GMP+biosynthetic+process	0.188738	0.513615	0.134683
BP_GO:0006112_energy+reserve+metabolic+process	0.127212	0.127212	#N/A
MF_GO:0008934_inositol-1(or+4)-monophosphatase+activity	0.0706753	0.0706753	#N/A
BP_GO:0021529_spinal+cord+oligodendrocyte+cell+differentiation	0.677851	0.671459	0.568202
BP_GO:0021530_spinal+cord+oligodendrocyte+cell+fate+specification	0.677851	0.671459	0.568202
CC_GO:0001520_outer+dense+fiber	0.451765	0.465105	0.46564
BP_GO:0015872_dopamine+transport	0.66751	#N/A	0.66751
BP_GO:0006360_transcription+from+RNA+polymerase+I+promoter	0.364248	0.493995	0.281234
BP_GO:0032715_negative+regulation+of+interleukin-6+production	0.643118	0.562898	0.659575
BP_GO:0042274_ribosomal+small+subunit+biogenesis	0.484036	0.370094	0.573698
MF_GO:0048487_beta-tubulin+binding	0.0317331	0.0317331	#N/A
BP_GO:0051603_proteolysis+involved+in+cellular+protein+catabolic+proces	0.76959	0.802174	0.530285
BP_GO:0060039_pericardium+development	0.257355	#N/A	0.257355
MF_GO:0008900_hydrogen:potassium-exchanging+ATPase+activity	0.922058	0.938249	0.609887
MF_GO:0004966_galanin+receptor+activity	0.962413	0.670961	0.968962
BP_GO:0060291_long-term+synaptic+potentiation	0.26445	0.423758	0.262804
BP_GO:0045749_negative+regulation+of+S+phase+of+mitotic+cell+cycle	0.660663	0.267058	0.94476
BP_GO:0009048_dosage+compensation,+by+inactivation+of+X+chromosom	0.470721	0.78978	0.254716
MF_GO:0005549_odorant+binding	0.262564	#N/A	0.262564
MF_GO:0015026_coreceptor+activity	0.250695	0.190124	0.419888
CC_GO:0001674_female+germ+cell+nucleus	0.854153	0.973132	0.470701

CC_GO:0005655_nucleolar+ribonuclease+P+complex	0.0402854	0.00910863	0.623122
BP_GO:0006266_DNA+ligation	0.0115598	0.356113	0.0058689
MF_GO:0004995_tachykinin+receptor+activity	0.238725	#N/A	0.238725
CC_GO:0048786_presynaptic+active+zone	0.0714464	0.0714464	#N/A
BP_GO:0021555_midbrain-hindbrain+boundary+morphogenesis	0.328342	0.328342	#N/A
BP_GO:0045578_negative+regulation+of+B+cell+differentiation	0.0895397	#N/A	0.0895397
BP_GO:0042147_retrograde+transport,+endosome+to+Golgi	0.760533	0.934605	0.18137
MF_GO:0003964_RNA-directed+DNA+polymerase+activity	0.60698	0.646409	0.475723
BP_GO:0031133_regulation+of+axon+diameter	0.717778	0.717778	#N/A
MF_GO:0016527_brain-specific+angiogenesis+inhibitor+activity	0.472597	0.472597	#N/A
BP_GO:0016539_intein-mediated+protein+splicing	0.781208	#N/A	0.781208
BP_GO:0006481_C-terminal+protein+amino+acid+methylation	0.0271004	0.0271004	#N/A
BP_GO:0010824_regulation+of+centrosome+duplication	0.978072	0.655893	0.985518
MF_GO:0004865_type+1+serine/threonine+specific+protein+phosphatase+i	0.0870192	0.143937	0.180824
BP_GO:0008589_regulation+of+smoothened+signaling+pathway	0.0829627	#N/A	0.0829627
MF_GO:0015277_kainate+selective+glutamate+receptor+activity	0.196084	0.13685	0.526541
BP_GO:0007262_STAT+protein+nuclear+translocation	0.907662	0.641248	0.9144
BP_GO:0009743_response+to+carbohydrate+stimulus	0.739738	0.828664	0.409445
BP_GO:0045110_intermediate+filament+bundle+assembly	0.689534	0.689534	#N/A
MF_GO:0004406_H3/H4+histone+acetyltransferase+activity	0.957357	0.999033	0.465627
MF_GO:0050501_hyaluronan+synthase+activity	0.23676	0.0800308	0.546382
BP_GO:0045085_negative+regulation+of+interleukin-2+biosynthetic+proces	0.644812	0.419406	0.725342
BP_GO:0050710_negative+regulation+of+cytokine+secretion	0.321415	0.843177	0.100194
BP_GO:0042668_auditory+receptor+cell+fate+determination	0.767927	0.740273	0.639313
BP_GO:0021985_neurohypophysis+development	0.374749	0.31785	0.546683
BP_GO:0006283_transcription-coupled+nucleotide-excision+repair	0.420834	0.0578872	0.807388
BP_GO:0045953_negative+regulation+of+natural+killer+cell+mediated+cyto	0.941811	0.941811	#N/A
BP_GO:0050955_thermoception	0.469133	#N/A	0.469133
MF_GO:0008327_methyl-CpG+binding	0.617251	0.495511	0.702768
BP_GO:0001992_regulation+of+systemic+arterial+blood+pressure+by+vaso[	0.491403	0.825681	0.245331
BP_GO:0050968_detection+of+chemical+stimulus+involved+in+sensory+per	0.81569	0.948047	0.480253
MF_GO:0035035_histone+acetyltransferase+binding	0.536463	0.267058	0.850247
MF_GO:0048029_monosaccharide+binding	0.715059	0.586578	0.7058
BP_GO:0048747_muscle+fiber+development	0.764362	0.742857	0.627481
MF_GO:0008745_N-acetylmuramoyl-L-alanine+amidase+activity	0.637585	0.418967	0.815564
BP_GO:0002309_T+cell+proliferation+during+immune+response	0.932954	#N/A	0.932954
CC_GO:0030127_COPII+vesicle+coat	0.802369	0.914615	0.321117
BP_GO:0050917_sensory+perception+of+umami+taste	0.881767	0.882554	0.728842
BP_GO:0021615_glossopharyngeal+nerve+morphogenesis	0.464198	0.182131	0.870166
MF_GO:0015018_galactosylgalactosylxylosylprotein+3-beta-glucuronosyltra	0.406824	0.254293	0.571134
BP_GO:0014002_astrocyte+development	0.483786	0.372801	0.571409
MF_GO:0008061_chitin+binding	0.293745	0.554856	0.223156
BP_GO:0045348_positive+regulation+of+MHC+class+II+biosynthetic+proces	0.489935	#N/A	0.489935
BP_GO:0007041_lysosomal+transport	0.547043	0.266218	0.861538
BP_GO:0045900_negative+regulation+of+translational+elongation	0.191386	0.0245397	0.898203
BP_GO:0006505_GPI+anchor+metabolic+process	0.921706	0.748503	0.896317
MF_GO:0016654_oxidoreductase+activity,+acting+on+NADH+or+NADPH,+di	0.518951	0.293618	0.802288

BP_GO:0006422_aspartyl-tRNA+aminoacylation	0.845542	#N/A	0.845542
MF_GO:0004815_aspartate-tRNA+ligase+activity	0.845542	#N/A	0.845542
BP_GO:0043631_RNA+polyadenylation	0.0983769	0.599325	0.0393496
BP_GO:0021869_forebrain+ventricular+zone+progenitor+cell+division	0.089113	0.0417198	0.546683
BP_GO:0043584_nose+development	0.0223972	#N/A	0.0223972
BP_GO:0051205_protein+insertion+into+membrane	0.732079	0.651508	0.698931
BP_GO:0016050_vesicle+organization	0.450282	0.450282	#N/A
BP_GO:0043270_positive+regulation+of+ion+transport	0.636872	0.636872	#N/A
CC_GO:0033269_internode+region+of+axon	0.314837	0.109495	0.816809
MF_GO:0032549_ribonucleoside+binding	0.783756	#N/A	0.783756
BP_GO:0030101_natural+killer+cell+activation	0.919207	0.663948	0.921403
MF_GO:0030304_trypsin+inhibitor+activity	0.522349	0.610379	0.448488
CC_GO:0005577_fibrinogen+complex	0.975186	0.975186	#N/A
BP_GO:0006603_phosphocreatine+metabolic+process	0.0984776	0.0984776	#N/A
MF_GO:0042132_fructose+1,6-bisphosphate+1-phosphatase+activity	0.884885	0.647297	0.906257
BP_GO:0043627_response+to+estrogen+stimulus	0.0812084	0.073383	0.300378
CC_GO:0005862_muscle+thin+filament+tropomyosin	0.763716	0.763716	#N/A
MF_GO:0017159_pantetheine+hydrolase+activity	0.342476	0.346658	0.429023
MF_GO:0047131_saccharopine+dehydrogenase+(NAD+,+L-glutamate-formir	0.55113	0.710575	0.354513
MF_GO:0003960_NADPH:quinone+reductase+activity	0.937771	0.955355	0.681769
BP_GO:0001998_angiotensin+mediated+vasoconstriction+involved+in+regul	0.160053	0.804886	0.0117782
MF_GO:0008184_glycogen+phosphorylase+activity	0.88007	0.88007	#N/A
BP_GO:0019322_pentose+biosynthetic+process	0.581695	0.994706	0.0117782
MF_GO:0042134_rRNA+primary+transcript+binding	0.119521	0.119521	#N/A
MF_GO:0030296_protein+tyrosine+kinase+activator+activity	0.107242	#N/A	0.107242
MF_GO:0017067_tyrosine-ester+sulfotransferase+activity	0.602831	0.823068	0.288283
MF_GO:0043395_heparan+sulfate+proteoglycan+binding	0.278655	#N/A	0.278655
MF_GO:0046592_polyamine+oxidase+activity	0.25302	0.25302	#N/A
BP_GO:0048807_female+genitalia+morphogenesis	0.0503647	#N/A	0.0503647
MF_GO:0004942_anaphylatoxin+receptor+activity	0.795278	#N/A	0.795278
MF_GO:0004607_phosphatidylcholine-sterol+O-acyltransferase+activity	0.274691	0.274691	#N/A
CC_GO:0005924_cell-substrate+adherens+junction	0.916871	#N/A	0.916871
BP_GO:0001973_adenosine+receptor+signaling+pathway	0.302822	#N/A	0.302822
MF_GO:0015645_fatty-acid+ligase+activity	0.950381	#N/A	0.950381
BP_GO:0048193_Golgi+vesicle+transport	0.226172	0.341545	0.256637
MF_GO:0003986_acetyl-CoA+hydrolase+activity	0.96535	0.96535	#N/A
BP_GO:0032613_interleukin-10+production	0.0282387	0.333077	0.0117782
BP_GO:0032615_interleukin-12+production	0.0282387	0.333077	0.0117782
MF_GO:0004743_pyruvate+kinase+activity	0.225213	0.0750448	0.645316
BP_GO:0042730_fibrinolysis	0.984225	0.860587	0.974789
MF_GO:0000247_C-8+sterol+isomerase+activity	0.39711	0.518399	0.339226
BP_GO:0002072_optic+cup+morphogenesis+involved+in+camera-type+eye+	0.14232	#N/A	0.14232
BP_GO:0046208_spermine+catabolic+process	0.435438	0.402372	0.507063
BP_GO:0043490_malate-aspartate+shuttle	0.66117	0.805086	0.392749
MF_GO:0004063_aryldialkylphosphatase+activity	0.358281	0.358281	#N/A
BP_GO:0045639_positive+regulation+of+myeloid+cell+differentiation	0.544425	0.544425	#N/A
MF_GO:0047750_cholestenol+delta-isomerase+activity	0.483704	0.483704	#N/A

BP_GO:0042308_negative+regulation+of+protein+import+into+nucleus	0.103831	0.24046	0.140979
MF_GO:0004373_glycogen+(starch)+synthase+activity	0.360376	0.360376	#N/A
MF_GO:0016768_spermine+synthase+activity	0.340907	#N/A	0.340907
MF_GO:0004772_sterol+O-acyltransferase+activity	0.824343	0.824343	#N/A
MF_GO:0034736_cholesterol+O-acyltransferase+activity	0.824343	0.824343	#N/A
MF_GO:0034737_ergosterol+O-acyltransferase+activity	0.824343	0.824343	#N/A
MF_GO:0034738_lanosterol+O-acyltransferase+activity	0.824343	0.824343	#N/A
BP_GO:0019509_methionine+salvage	0.0278884	0.373845	0.00861585
BP_GO:0001935_endothelial+cell+proliferation	0.101365	0.101365	#N/A
MF_GO:0019955_cytokine+binding	0.0247217	#N/A	0.0247217
BP_GO:0006544_glycine+metabolic+process	0.719656	0.672939	0.646017
MF_GO:0019003_GDP+binding	0.244417	0.321756	0.303166
MF_GO:0008170_N-methyltransferase+activity	0.949103	0.949103	#N/A
BP_GO:0006907_pinocytosis	0.0883032	#N/A	0.0883032
BP_GO:0006529_asparagine+biosynthetic+process	0.0605963	#N/A	0.0605963
MF_GO:0004066_asparagine+synthase+(glutamine-hydrolyzing)+activity	0.0605963	#N/A	0.0605963
BP_GO:0016554_cytidine+to+uridine+editing	0.867313	#N/A	0.867313
MF_GO:0004014_adenosylmethionine+decarboxylase+activity	0.510081	#N/A	0.510081
BP_GO:0006778_porphyrin+metabolic+process	0.0440985	#N/A	0.0440985
MF_GO:0043185_vascular+endothelial+growth+factor+receptor+3+binding	0.0251224	#N/A	0.0251224
BP_GO:0030497_fatty+acid+elongation	0.00595594	0.00900735	0.117227
MF_GO:0003920_GMP+reductase+activity	0.0406696	0.0138783	0.396397
MF_GO:0003858_3-hydroxybutyrate+dehydrogenase+activity	0.85199	0.843984	0.679524
MF_GO:0001846_opsonin+binding	0.182736	0.380301	0.164968
MF_GO:0004478_methionine+adenosyltransferase+activity	0.7168	#N/A	0.7168
BP_GO:0045019_negative+regulation+of+nitric+oxide+biosynthetic+process	0.0314866	0.0314866	#N/A
BP_GO:0007527_adult+somatic+muscle+development	0.722548	0.859569	0.403777
MF_GO:0008453_alanine-glyoxylate+transaminase+activity	0.563002	0.563002	#N/A
BP_GO:0014909_smooth+muscle+cell+migration	0.465845	0.465845	#N/A
MF_GO:0004074_biliverdin+reductase+activity	0.540433	0.0359424	0.974028
BP_GO:0006569_tryptophan+catabolic+process	0.794488	#N/A	0.794488
BP_GO:0043462_regulation+of+ATPase+activity	4.65524E-05	4.65524E-05	#N/A
BP_GO:0019452_L-cysteine+catabolic+process+to+taurine	0.339702	#N/A	0.339702
MF_GO:0004359_glutaminase+activity	0.772113	0.709548	0.692301
BP_GO:0048554_positive+regulation+of+metalloenzyme+activity	0.209511	0.073383	0.621354
MF_GO:0015114_phosphate+transmembrane+transporter+activity	0.581728	0.620603	0.493938
BP_GO:0001561_fatty+acid+alpha-oxidation	0.709829	0.493548	0.787537
BP_GO:0019853_L-ascorbic+acid+biosynthetic+process	0.800354	0.621603	0.811098
BP_GO:0002074_extraocular+skeletal+muscle+development	0.505844	0.505844	#N/A
MF_GO:0005547_phosphatidylinositol-3,4,5-triphosphate+binding	0.805855	0.725508	0.732501
BP_GO:0019344_cysteine+biosynthetic+process	0.19577	0.19577	#N/A
CC_GO:0005927_muscle+tendon+junction	0.139739	0.139739	#N/A
BP_GO:0048260_positive+regulation+of+receptor-mediated+endocytosis	0.836218	0.905199	0.52883
MF_GO:0018685_alkane+1-monooxygenase+activity	0.84275	#N/A	0.84275
MF_GO:0047756_chondroitin+4-sulfotransferase+activity	0.730314	#N/A	0.730314
MF_GO:0004465_lipoprotein+lipase+activity	0.0674107	0.0674107	#N/A
BP_GO:0002329_pre-B+cell+differentiation	0.754403	0.932752	0.300378

MF_GO:0005099_Ras+GTPase+activator+activity	0.138451	#N/A	0.138451
MF_GO:0004421_hydroxymethylglutaryl-CoA+synthase+activity	0.993957	0.989558	0.89204
MF_GO:0004476_mannose-6-phosphate+isomerase+activity	0.736219	0.771788	0.559001
BP_GO:0018119_peptidyl-cysteine+S-nitrosylation	0.889767	0.53428	0.950131
BP_GO:0032959_inositol+trisphosphate+biosynthetic+process	0.578242	0.316436	0.77546
BP_GO:0045730_respiratory+burst	0.139345	0.156821	0.300378
BP_GO:0015808_L-alanine+transport	0.105609	0.177945	0.199421
BP_GO:0015824_proline+transport	0.105609	0.177945	0.199421
MF_GO:0005280_hydrogen:amino+acid+symporter+activity	0.105609	0.177945	0.199421
MF_GO:0005302_L-tyrosine+transmembrane+transporter+activity	0.105609	0.177945	0.199421
MF_GO:0015180_L-alanine+transmembrane+transporter+activity	0.105609	0.177945	0.199421
MF_GO:0015193_L-proline+transmembrane+transporter+activity	0.105609	0.177945	0.199421
BP_GO:0001543_ovarian+follicle+rupture	0.52131	0.804886	0.216691
BP_GO:0006097_glyoxylate+cycle	0.306041	0.105832	0.702799
BP_GO:0006102_isocitrate+metabolic+process	0.306041	0.105832	0.702799
MF_GO:0004450_isocitrate+dehydrogenase+(NADP+)+activity	0.306041	0.105832	0.702799
CC_GO:0005584_collagen+type+I	0.952625	#N/A	0.952625
BP_GO:0043507_positive+regulation+of+JNK+activity	0.741269	0.816928	0.504554
BP_GO:0051451_myoblast+migration	0.803457	0.233082	0.973574
MF_GO:0004776_succinate-CoA+ligase+(GDP-forming)+activity	0.1652	0.1652	#N/A
MF_GO:0003951_NAD++kinase+activity	0.692039	0.504169	0.757677
BP_GO:0006555_methionine+metabolic+process	0.965942	0.965942	#N/A
BP_GO:0002018_renin-angiotensin+regulation+of+aldosterone+production	0.967816	0.804886	0.960408
BP_GO:0002019_regulation+of+renal+output+by+angiotensin	0.967816	0.804886	0.960408
MF_GO:0004531_deoxyribonuclease+II+activity	0.684483	0.165088	0.95082
MF_GO:0004449_isocitrate+dehydrogenase+(NAD+)+activity	0.920237	0.518322	0.973967
BP_GO:0060346_bone+trabecula+formation	0.545998	0.0454836	0.968123
BP_GO:0001885_endothelial+cell+development	0.187051	0.221567	0.312364
BP_GO:0019439_aromatic+compound+catabolic+process	0.343547	0.226599	0.571812
MF_GO:0004301_epoxide+hydrolase+activity	0.343547	0.226599	0.571812
BP_GO:0006570_tyrosine+metabolic+process	0.460286	0.32645	0.621354
MF_GO:0047749_cholestanetriol+26-monooxygenase+activity	0.42941	0.668324	0.246209
MF_GO:0003963_RNA-3'-phosphate+cyclase+activity	0.683779	#N/A	0.683779
MF_GO:0004067_asparaginase+activity	0.241411	0.0534964	0.732427
BP_GO:0030206_chondroitin+sulfate+biosynthetic+process	0.868626	#N/A	0.868626
MF_GO:0018738_S-formylglutathione+hydrolase+activity	0.938059	#N/A	0.938059
MF_GO:0003973_(S)-2-hydroxy-acid+oxidase+activity	0.339119	#N/A	0.339119
BP_GO:0021520_spinal+cord+motor+neuron+cell+fate+specification	0.716344	#N/A	0.716344
BP_GO:0009081_branched+chain+family+amino+acid+metabolic+process	0.0569993	0.20848	0.0775121
BP_GO:0009082_branched+chain+family+amino+acid+biosynthetic+process	0.0569993	0.20848	0.0775121
MF_GO:0004084_branched-chain-amino-acid+transaminase+activity	0.0569993	0.20848	0.0775121
BP_GO:0001788_antibody-dependent+cellular+cytotoxicity	0.306678	#N/A	0.306678
MF_GO:0019770_IgG+receptor+activity	0.306678	#N/A	0.306678
BP_GO:0051900_regulation+of+mitochondrial+depolarization	0.621334	0.332057	0.808186
CC_GO:0017109_glutamate-cysteine+ligase+complex	0.621334	0.332057	0.808186
BP_GO:0046626_regulation+of+insulin+receptor+signaling+pathway	0.367706	0.65931	0.187271
MF_GO:0050051_leukotriene-B4+20-monooxygenase+activity	0.988015	#N/A	0.988015

BP_GO:0051926_negative+regulation+of+calcium+ion+transport	0.8165	0.355404	0.950131
BP_GO:0022407_regulation+of+cell-cell+adhesion	0.499985	0.699689	0.300378
BP_GO:0006563_L-serine+metabolic+process	0.309956	0.672939	0.12531
BP_GO:0001768_establishment+of+T+cell+polarity	0.596397	0.596397	#N/A
MF_GO:0004019_adenylosuccinate+synthase+activity	0.225592	0.212666	0.394533
BP_GO:0050777_negative+regulation+of+immune+response	0.589993	0.825784	0.269018
MF_GO:0005161_platelet-derived+growth+factor+receptor+binding	0.596635	#N/A	0.596635
BP_GO:0046879_hormone+secretion	0.190536	0.179365	0.374461
MF_GO:0030215_semaphorin+receptor+binding	0.99022	0.959415	0.940185
MF_GO:0004998_transferrin+receptor+activity	0.308095	0.661844	0.130131
BP_GO:0042531_positive+regulation+of+tyrosine+phosphorylation+of+STAT	0.699793	0.699793	#N/A
BP_GO:0043506_regulation+of+JNK+activity	0.320074	0.320074	#N/A
MF_GO:0004586_ornithine+decarboxylase+activity	0.174389	0.611994	0.0538467
BP_GO:0033627_cell+adhesion+mediated+by+integrin	0.563344	0.563344	#N/A
BP_GO:0006518_peptide+metabolic+process	0.273669	0.0131991	0.914654
MF_GO:0003878_ATP+citrate+synthase+activity	0.164781	0.112653	0.434336
BP_GO:0042732_D-xylose+metabolic+process	0.903845	#N/A	0.903845
MF_GO:0004471_malate+dehydrogenase+(decarboxylating)+activity	0.0036244	#N/A	0.0036244
BP_GO:0055013_cardiac+muscle+cell+development	0.341883	0.341883	#N/A
BP_GO:0032099_negative+regulation+of+appetite	0.699871	0.379754	0.852467
MF_GO:0003883_CTP+synthase+activity	0.851091	0.851091	#N/A
BP_GO:0016558_protein+import+into+peroxisome+matrix	0.0229279	0.0229279	#N/A
BP_GO:0050901_leukocyte+tethering+or+rolling	0.24078	#N/A	0.24078
BP_GO:0009313_oligosaccharide+catabolic+process	0.525137	0.525137	#N/A
BP_GO:0006991_response+to+sterol+depletion	0.477776	0.296292	0.676006
BP_GO:0032796_uropod+organization	0.950565	0.428115	0.994034
MF_GO:0004372_glycine+hydroxymethyltransferase+activity	0.32515	0.32515	#N/A
BP_GO:0000083_regulation+of+transcription+of+G1/S-phase+of+mitotic+ce	0.425831	0.48357	0.411817
BP_GO:0051938_L-glutamate+import	0.511744	#N/A	0.511744
BP_GO:0043312_neutrophil+degranulation	0.999951	#N/A	0.999951
BP_GO:0048002_antigen+processing+and+presentation+of+peptide+antiger	0.924254	0.924254	#N/A
MF_GO:0008429_phosphatidylethanolamine+binding	0.0226217	0.0226217	#N/A
CC_GO:0031088_platelet+dense+granule+membrane	0.74258	0.908753	0.340079
BP_GO:0050714_positive+regulation+of+protein+secretion	0.650146	#N/A	0.650146
MF_GO:0008238_exopeptidase+activity	0.850134	0.920575	0.522757
BP_GO:0042373_vitamin+K+metabolic+process	0.0836392	#N/A	0.0836392
CC_GO:0008247_1-alkyl-2-acetylglycerophosphocholine+esterase+complex	0.555817	0.824524	0.231449
BP_GO:0046329_negative+regulation+of+JNK+cascade	0.0411	0.254634	0.036209
MF_GO:0019166_trans-2-enoyl-CoA+reductase+(NADPH)+activity	0.0198916	0.0198916	#N/A
MF_GO:0016403_dimethylargininase+activity	0.196701	0.13634	0.456525
MF_GO:0004335_galactokinase+activity	0.643073	0.643073	#N/A
MF_GO:0030188_chaperone+regulator+activity	0.276412	#N/A	0.276412
CC_GO:0005964_phosphorylase+kinase+complex	0.876711	0.876711	#N/A
MF_GO:0004689_phosphorylase+kinase+activity	0.876711	0.876711	#N/A
BP_GO:0042994_cytoplasmic+sequestering+of+transcription+factor	0.45911	0.458768	0.483509
MF_GO:0050262_ribosylnicotinamide+kinase+activity	0.170528	0.0349453	0.679795
BP_GO:0006739_NADP+metabolic+process	0.861737	0.586897	0.906374

BP_GO:0006111_regulation+of+gluconeogenesis	0.502668	0.379754	0.623932
MF_GO:0016620_oxidoreductase+activity,+acting+on+the+aldehyde+or+oxo	0.194671	0.255609	0.287893
BP_GO:0030865_cortical+cytoskeleton+organization	0.190365	0.190365	#N/A
BP_GO:0008210_estrogen+metabolic+process	0.27442	0.141154	0.590184
MF_GO:0030375_thyroid+hormone+receptor+coactivator+activity	0.694704	0.277955	0.904725
BP_GO:0006525_arginine+metabolic+process	0.189021	0.172621	0.381369
MF_GO:0004053_arginase+activity	0.189021	0.172621	0.381369
BP_GO:0006275_regulation+of+DNA+replication	0.0912691	0.163519	0.183053
CC_GO:0031528_microvillus+membrane	0.132755	0.146793	0.300378
BP_GO:0045619_regulation+of+lymphocyte+differentiation	0.0306423	0.361067	0.0110289
BP_GO:0030573_bile+acid+catabolic+process	0.326423	#N/A	0.326423
MF_GO:0017130_poly(rC)+binding	0.0777017	0.0777017	#N/A
BP_GO:0043366_beta+selection	0.553121	0.553121	#N/A
MF_GO:0019902_phosphatase+binding	0.0677759	#N/A	0.0677759
BP_GO:0002537_production+of+nitric+oxide+during+acute+inflammatory+r	0.916724	0.916724	#N/A
CC_GO:0019867_outer+membrane	0.0880549	0.0880549	#N/A
BP_GO:0006532_aspartate+biosynthetic+process	0.318208	0.318208	#N/A
BP_GO:0019550_glutamate+catabolic+process+to+aspartate	0.318208	0.318208	#N/A
BP_GO:0019551_glutamate+catabolic+process+to+2-oxoglutarate	0.318208	0.318208	#N/A
BP_GO:0051725_protein+amino+acid+de-ADP-ribosylation	0.00690544	0.00690544	#N/A
MF_GO:0003875_ADP-ribosylarginine+hydrolase+activity	0.00690544	0.00690544	#N/A
BP_GO:0051583_dopamine+uptake	0.817522	#N/A	0.817522
BP_GO:0001845_phagolysosome+formation	0.621646	0.620566	0.552183
BP_GO:0001771_formation+of+immunological+synapse	0.775698	0.700678	0.707104
MF_GO:0030023_extracellular+matrix+constituent+conferring+elasticity	0.693546	#N/A	0.693546
BP_GO:0006198_cAMP+catabolic+process	0.759171	#N/A	0.759171
BP_GO:0014916_regulation+of+lung+blood+pressure	0.102137	0.131558	0.249821
MF_GO:0004962_endothelin+receptor+activity	0.345912	#N/A	0.345912
BP_GO:0051602_response+to+electrical+stimulus	0.106684	0.0293923	0.552183
MF_GO:0047804_cysteine-S-conjugate+beta-lyase+activity	0.241174	0.241174	#N/A
BP_GO:0030836_positive+regulation+of+actin+filament+depolymerization	0.674556	0.674556	#N/A
BP_GO:0009264_deoxyribonucleotide+catabolic+process	0.176741	0.176741	#N/A
BP_GO:0046459_short-chain+fatty+acid+metabolic+process	0.127008	0.255609	0.169766
MF_GO:0003785_actin+monomer+binding	0.917642	0.602477	0.955841
MF_GO:0000900_translation+repressor+activity,+nucleic+acid+binding	0.77292	0.77292	#N/A
MF_GO:0042056_chemoattractant+activity	0.819042	0.888123	0.52883
BP_GO:0045723_positive+regulation+of+fatty+acid+biosynthetic+process	0.717146	0.717146	#N/A
BP_GO:0051024_positive+regulation+of+immunoglobulin+secretion	0.93195	#N/A	0.93195
MF_GO:0005159_insulin-like+growth+factor+receptor+binding	0.00713192	0.148398	0.00775572
BP_GO:0008582_regulation+of+synaptic+growth+at+neuromuscular+junctic	0.777491	0.534918	0.839436
MF_GO:0046912_transferase+activity,+transferring+acyl+groups,+acyl+grou	0.37198	0.383588	0.434336
BP_GO:0009156_ribonucleoside+monophosphate+biosynthetic+process	0.723755	0.723755	#N/A
BP_GO:0051291_protein+heterooligomerization	0.616346	0.616346	#N/A
MF_GO:0008124_4-alpha-hydroxytetrahydrobiopterin+dehydratase+activity	0.616346	0.616346	#N/A
BP_GO:0018346_protein+amino+acid+prenylation	0.203248	0.203248	#N/A
MF_GO:0008318_protein+prenyltransferase+activity	0.203248	0.203248	#N/A
BP_GO:0006068_ethanol+catabolic+process	0.641441	#N/A	0.641441

BP_GO:0042987_amyloid+precursor+protein+catabolic+process	0.796603	#N/A	0.796603
BP_GO:0045212_neurotransmitter+receptor+biosynthetic+process	0.620208	0.13432	0.938099
BP_GO:0050650_chondroitin+sulfate+proteoglycan+biosynthetic+process	0.0242724	#N/A	0.0242724
BP_GO:0043280_positive+regulation+of+caspase+activity	0.447854	0.447854	#N/A
BP_GO:0019218_regulation+of+steroid+metabolic+process	0.662105	0.662105	#N/A
BP_GO:0046543_development+of+secondary+female+sexual+characteristics	0.662105	0.662105	#N/A
BP_GO:0046544_development+of+secondary+male+sexual+characteristics	0.662105	0.662105	#N/A
BP_GO:0000085_G2+phase+of+mitotic+cell+cycle	0.992175	0.988449	0.873863
MF_GO:0047237_glucuronylgalactosylproteoglycan+4-beta-N-acetylgalactos	0.157349	#N/A	0.157349
BP_GO:0002755_MyD88-dependent+toll-like+receptor+signaling+pathway	0.481873	0.43003	0.544707
MF_GO:0004447_iodide+peroxidase+activity	0.547966	#N/A	0.547966
BP_GO:0043619_regulation+of+transcription+from+RNA+polymerase+II+prc	0.730584	0.787737	0.528041
MF_GO:0004161_dimethylallyltranstransferase+activity	0.0880903	0.418131	0.0441015
MF_GO:0004337_geranyltranstransferase+activity	0.0880903	0.418131	0.0441015
MF_GO:0019103_pyrimidine+nucleotide+binding	0.527207	0.588599	0.44938
BP_GO:0001834_trophectodermal+cell+proliferation	0.285158	0.149897	0.592839
BP_GO:0001919_regulation+of+receptor+recycling	0.677174	0.187047	0.938099
MF_GO:0005068_transmembrane+receptor+protein+tyrosine+kinase+adapt	0.0494953	0.0494953	#N/A
BP_GO:0046426_negative+regulation+of+JAK-STAT+cascade	0.0816181	0.0816181	#N/A
BP_GO:0006086_acetyl-CoA+biosynthetic+process+from+pyruvate	0.260836	0.260836	#N/A
CC_GO:0030314_junctional+membrane+complex	0.551847	0.2587	0.797245
BP_GO:0042511_positive+regulation+of+tyrosine+phosphorylation+of+Stat1	0.254478	0.333219	0.307742
BP_GO:0007172_signal+complex+assembly	0.660567	#N/A	0.660567
MF_GO:0015272_ATP-activated+inward+rectifier+potassium+channel+activi	0.4975	0.0589449	0.940036
BP_GO:0031098_stress-activated+protein+kinase+signaling+pathway	0.917563	0.741611	0.905754
MF_GO:0005372_water+transporter+activity	0.180241	#N/A	0.180241
BP_GO:0035166_post-embryonic+hemopoiesis	0.484497	0.484497	#N/A
BP_GO:0032743_positive+regulation+of+interleukin-2+production	0.185612	0.185612	#N/A
BP_GO:0043372_positive+regulation+of+CD4-positive,+alpha+beta+T+cell+d	0.185612	0.185612	#N/A
BP_GO:0002033_vasodilation+by+angiotensin+involved+in+regulation+of+sy	0.00277971	#N/A	0.00277971
BP_GO:0043415_positive+regulation+of+skeletal+muscle+regeneration	0.729442	0.729442	#N/A
BP_GO:0006089_lactate+metabolic+process	0.25358	0.156821	0.528041
BP_GO:0035313_wound+healing,+spreading+of+epidermal+cells	0.381329	0.381329	#N/A
BP_GO:0002752_cell+surface+pattern+recognition+receptor+signaling+path	0.366754	0.366754	#N/A
BP_GO:0001879_detection+of+yeast	0.57401	0.57401	#N/A
BP_GO:0008064_regulation+of+actin+polymerization+or+depolymerization	0.961674	0.495445	0.994034
MF_GO:0003994_aconitate+hydratase+activity	0.77818	0.8253	0.558552
MF_GO:0009384_N-acylmannosamine+kinase+activity	0.955257	#N/A	0.955257
MF_GO:0004020_adenylylsulfate+kinase+activity	0.358557	#N/A	0.358557
MF_GO:0004781_sulfate+adenylyltransferase+(ATP)+activity	0.358557	#N/A	0.358557
BP_GO:0042094_interleukin-2+biosynthetic+process	0.894533	0.907408	0.671313
MF_GO:0004560_alpha-L-fucosidase+activity	0.723028	0.723028	#N/A
BP_GO:0001738_morphogenesis+of+a+polarized+epithelium	0.00556602	0.165513	0.00445032
BP_GO:0008615_pyridoxine+biosynthetic+process	0.701228	0.701228	#N/A
BP_GO:0002347_response+to+tumor+cell	0.419913	#N/A	0.419913
BP_GO:0033194_response+to+hydroperoxide	0.416696	0.221567	0.680762
MF_GO:0004419_hydroxymethylglutaryl-CoA+lyase+activity	0.8319	0.8319	#N/A

BP_GO:0030262_apoptotic+nuclear+changes	0.0682936	#N/A	0.0682936
BP_GO:0006537_glutamate+biosynthetic+process	0.536985	0.536985	#N/A
BP_GO:0006562_proline+catabolic+process	0.536985	0.536985	#N/A
MF_GO:0004657_proline+dehydrogenase+activity	0.536985	0.536985	#N/A
MF_GO:0004943_C3a+anaphylatoxin+receptor+activity	0.723821	#N/A	0.723821
BP_GO:0002863_positive+regulation+of+inflammatory+response+to+antigen	0.116314	#N/A	0.116314
BP_GO:0046855_inositol+phosphate+dephosphorylation	0.587495	0.426467	0.69081
BP_GO:0032092_positive+regulation+of+protein+binding	0.687383	#N/A	0.687383
BP_GO:0031953_negative+regulation+of+protein+amino+acid+autophospho	0.37654	0.277794	0.557645
BP_GO:0051563_smooth+endoplasmic+reticulum+calcium+ion+homeostasis	0.985747	0.988449	0.795135
MF_GO:0015464_acetylcholine+receptor+activity	0.174758	0.0225334	0.752359
BP_GO:0045359_positive+regulation+of+interferon-beta+biosynthetic+proce	0.311384	#N/A	0.311384
MF_GO:0004777_succinate-semialdehyde+dehydrogenase+activity	0.280879	0.280879	#N/A
MF_GO:0016361_activin+receptor+activity,+type+I	0.200142	0.0647454	0.628341
BP_GO:0048739_cardiac+muscle+fiber+development	0.281739	0.00637966	0.952947
BP_GO:0006048_UDP-N-acetylglucosamine+biosynthetic+process	0.225404	0.225404	#N/A
MF_GO:0008476_protein-tyrosine+sulfotransferase+activity	0.192722	0.192722	#N/A
MF_GO:0004342_glucosamine-6-phosphate+deaminase+activity	0.805807	0.466423	0.903832
CC_GO:0031616_spindle+pole+centrosome	0.570344	0.71851	0.371593
MF_GO:0004008_copper-exporting+ATPase+activity	0.650105	#N/A	0.650105
MF_GO:0005007_fibroblast+growth+factor+receptor+activity	0.054733	0.054733	#N/A
BP_GO:0001878_response+to+yeast	0.489686	0.489686	#N/A
BP_GO:0006163_purine+nucleotide+metabolic+process	0.118696	0.118696	#N/A
BP_GO:0009258_10-formyltetrahydrofolate+catabolic+process	0.172776	#N/A	0.172776
BP_GO:0002268_follicular+dendritic+cell+differentiation	0.82032	#N/A	0.82032
BP_GO:0001302_replicative+cell+aging	0.92791	0.829683	0.866839
CC_GO:0031304_intrinsic+to+mitochondrial+inner+membrane	0.106946	0.0123678	0.687543
MF_GO:0008396_oxysterol+7-alpha-hydroxylase+activity	0.437338	#N/A	0.437338
BP_GO:0009191_ribonucleoside+diphosphate+catabolic+process	0.782302	0.166028	0.980901
MF_GO:0030159_receptor+signaling+complex+scaffold+activity	0.794047	0.794047	#N/A
BP_GO:0031987_locomotion+during+locomotory+behavior	0.362753	0.596921	0.229218
BP_GO:0046070_dGTP+metabolic+process	0.0752514	0.12135	0.19387
MF_GO:0033188_sphingomyelin+synthase+activity	0.842532	0.550725	0.902025
MF_GO:0047493_ceramide+cholinephosphotransferase+activity	0.842532	0.550725	0.902025
MF_GO:0019870_potassium+channel+inhibitor+activity	0.685674	0.353249	0.855519
CC_GO:0000939_inner+kinetochore+of+condensed+chromosome	0.200872	0.842592	0.0142572
BP_GO:0043589_skin+morphogenesis	0.14086	0.86314	0.00445032
BP_GO:0042089_cytokine+biosynthetic+process	0.376088	0.296486	0.535239
BP_GO:0050851_antigen+receptor-mediated+signaling+pathway	0.481089	0.414319	0.559486
MF_GO:0015651_quaternary+ammonium+group+transmembrane+transpor	0.707279	0.848508	0.397884
BP_GO:0001505_regulation+of+neurotransmitter+levels	0.20719	0.20719	#N/A
MF_GO:0008273_calcium,+potassium:sodium+antiporter+activity	0.367219	#N/A	0.367219
BP_GO:0001826_inner+cell+mass+cell+differentiation	0.335436	0.335436	#N/A
MF_GO:0008670_2,4-dienoyl-CoA+reductase+(NADPH)+activity	0.548339	0.548339	#N/A
BP_GO:0010035_response+to+inorganic+substance	0.197665	#N/A	0.197665
MF_GO:0004686_elongation+factor-2+kinase+activity	0.480192	0.221085	0.757589
MF_GO:0004618_phosphoglycerate+kinase+activity	0.200739	#N/A	0.200739

BP_GO:0060166_olfactory+pit+development	0.791509	0.987914	0.134178
BP_GO:0045682_regulation+of+epidermis+development	0.46144	0.717183	0.238517
BP_GO:0009268_response+to+pH	0.778355	0.735738	0.67495
MF_GO:0030507_spectrin+binding	0.688912	#N/A	0.688912
BP_GO:0031579_membrane+raft+organization	0.14502	0.020665	0.707104
BP_GO:0002244_hemopoietic+progenitor+cell+differentiation	0.584679	0.3009	0.795135
BP_GO:0002286_T+cell+activation+during+immune+response	0.584679	0.3009	0.795135
BP_GO:0051491_positive+regulation+of+filopodium+formation	0.700428	0.700428	#N/A
BP_GO:0009957_epidermal+cell+fate+specification	0.775935	0.981179	0.157129
BP_GO:0048733_sebaceous+gland+development	0.775935	0.981179	0.157129
BP_GO:0042770_DNA+damage+response,+signal+transduction	0.640683	0.640683	#N/A
BP_GO:0009651_response+to+salt+stress	0.883488	0.804886	0.795813
CC_GO:0033267_axon+part	0.381937	0.521951	0.315823
BP_GO:0032703_negative+regulation+of+interleukin-2+production	0.3002	0.3002	#N/A
BP_GO:0002763_positive+regulation+of+myeloid+leukocyte+differentiation	0.667856	0.667856	#N/A
BP_GO:0051124_synaptic+growth+at+neuromuscular+junction	0.996469	0.988449	0.937765
BP_GO:0006002_fructose+6-phosphate+metabolic+process	0.749168	0.361753	0.903832
MF_GO:0030544_Hsp70+protein+binding	0.0813831	0.0618762	0.332323
CC_GO:0016460_myosin+II+complex	0.740445	0.740445	#N/A
MF_GO:0043531_ADP+binding	0.479463	0.479463	#N/A
MF_GO:0004692_cGMP-dependent+protein+kinase+activity	0.507297	0.295897	0.713057
MF_GO:0003688_DNA+replication+origin+binding	0.141286	0.749056	0.0142572
CC_GO:0045335_phagocytic+vesicle	0.90993	#N/A	0.90993
MF_GO:0004797_thymidine+kinase+activity	0.43736	#N/A	0.43736
MF_GO:0005093_Rab+GDP-dissociation+inhibitor+activity	0.89893	0.971541	0.45984
MF_GO:0004315_3-oxoacyl-[acyl-carrier-protein]+synthase+activity	0.710035	0.710035	#N/A
BP_GO:0006654_phosphatidic+acid+biosynthetic+process	0.703886	#N/A	0.703886
BP_GO:0051290_protein+heterotetramerization	0.752637	0.172969	0.971797
MF_GO:0000010_trans-hexaprenyltranstransferase+activity	0.752637	0.172969	0.971797
MF_GO:0050347_trans-octaprenyltranstransferase+activity	0.752637	0.172969	0.971797
BP_GO:0015804_neutral+amino+acid+transport	0.947075	0.947075	#N/A
MF_GO:0033857_diphosphoinositol-pentakisphosphate+kinase+activity	0.444885	0.597743	0.328823
MF_GO:0019834_phospholipase+A2+inhibitor+activity	0.169707	#N/A	0.169707
MF_GO:0004609_phosphatidylserine+decarboxylase+activity	0.30024	0.30024	#N/A
BP_GO:0045921_positive+regulation+of+exocytosis	0.846766	0.987771	0.210667
CC_GO:0005753_mitochondrial+proton-transporting+ATP+synthase+comple	0.758972	0.758972	#N/A
BP_GO:0045634_regulation+of+melanocyte+differentiation	0.846076	0.846076	#N/A
BP_GO:0034613_cellular+protein+localization	0.163858	0.146793	0.369617
MF_GO:0043130_ubiquitin+binding	0.206592	0.0232381	0.798039
MF_GO:0015108_chloride+transmembrane+transporter+activity	0.53819	0.0525026	0.960526
MF_GO:0046922_peptide-O-fucosyltransferase+activity	0.67376	0.67376	#N/A
BP_GO:0007263_nitric+oxide+mediated+signal+transduction	0.21767	0.455555	0.160905
BP_GO:0060055_angiogenesis+involved+in+wound+healing	0.253514	0.221567	0.432253
BP_GO:0051925_regulation+of+calcium+ion+transport+via+voltage-gated+c	0.236388	0.020665	0.847464
BP_GO:0051135_positive+regulation+of+NK+T+cell+activation	0.523557	0.605352	0.427215
MF_GO:0030548_acetylcholine+receptor+regulator+activity	0.402756	0.402756	#N/A
MF_GO:0030158_protein+xylosyltransferase+activity	0.90087	0.944466	0.589128

MF_GO:0045569_TRAIL+binding	0.322395	0.322395	#N/A
BP_GO:0035022_positive+regulation+of+Rac+protein+signal+transduction	0.859896	0.859896	#N/A
MF_GO:0003868_4-hydroxyphenylpyruvate+dioxygenase+activity	0.244608	#N/A	0.244608
BP_GO:0009186_deoxyribonucleoside+diphosphate+metabolic+process	0.840324	0.709162	0.804231
BP_GO:0015893_drug+transport	0.530671	0.645389	0.395937
BP_GO:0048087_positive+regulation+of+pigmentation+during+developmen	0.411783	0.0579951	0.89561
BP_GO:0044257_cellular+protein+catabolic+process	0.504978	0.767746	0.23775
MF_GO:0004021_alanine+transaminase+activity	0.254583	0.254583	#N/A
BP_GO:0030050_vesicle+transport+along+actin+filament	0.377516	#N/A	0.377516
MF_GO:0002020_protease+binding	0.567143	0.567143	#N/A
MF_GO:0005137_interleukin-5+receptor+binding	0.512199	0.350412	0.665559
MF_GO:0004967_glucagon+receptor+activity	0.667726	0.667726	#N/A
MF_GO:0047150_betaine-homocysteine+S-methyltransferase+activity	0.769801	#N/A	0.769801
MF_GO:0004850_uridine+phosphorylase+activity	0.88838	#N/A	0.88838
BP_GO:0014850_response+to+muscle+activity	0.742534	0.802428	0.528041
CC_GO:0043205_fibril	0.264062	0.534342	0.16412
MF_GO:0008109_N-acetyllactosaminide+beta-1,6-N-acetylglucosaminyltran	0.0832338	#N/A	0.0832338
BP_GO:0006649_phospholipid+transfer+to+membrane	0.145312	#N/A	0.145312
MF_GO:0016316_phosphatidylinositol-3,4-bisphosphate+4-phosphatase+ac	0.919388	0.515703	0.973877
MF_GO:0034597_phosphatidyl-inositol-4,5-bisphosphate+4-phosphatase+ac	0.919388	0.515703	0.973877
CC_GO:0042175_nuclear+envelope-endoplasmic+reticulum+network	0.470294	0.372506	0.587112
BP_GO:0046022_positive+regulation+of+transcription+from+RNA+polymera	0.383482	0.358944	0.477133
BP_GO:0051014_actin+filament+severing	0.85413	0.85413	#N/A
MF_GO:0005069_transmembrane+receptor+protein+tyrosine+kinase+docki	0.321627	0.321627	#N/A
MF_GO:0017112_Rab+guanyl-nucleotide+exchange+factor+activity	0.47932	0.67546	0.298716
MF_GO:0004771_sterol+esterase+activity	0.705976	0.882901	0.335879
BP_GO:0007194_negative+regulation+of+adenylate+cyclase+activity	0.585211	0.452378	0.664269
CC_GO:0005583_fibrillar+collagen	0.443614	0.351658	0.571677
BP_GO:0046827_positive+regulation+of+protein+export+from+nucleus	0.796738	0.684325	0.756046
BP_GO:0014902_myotube+differentiation	0.318395	0.221567	0.53972
BP_GO:0042488_positive+regulation+of+odontogenesis+of+dentine-contain	0.787589	0.762833	0.660149
BP_GO:0010766_negative+regulation+of+sodium+ion+transport	0.895644	0.531744	0.955198
BP_GO:0030916_otic+vesicle+formation	0.0553268	0.244657	0.0590008
MF_GO:0004925_prolactin+receptor+activity	0.660252	0.927137	0.191994
BP_GO:0001893_maternal+placenta+development	0.942052	0.851184	0.881205
MF_GO:0004516_nicotinate+phosphoribosyltransferase+activity	0.416823	0.368974	0.515135
BP_GO:0000084_S+phase+of+mitotic+cell+cycle	0.830649	0.929801	0.45155
MF_GO:0003867_4-aminobutyrate+transaminase+activity	0.87353	0.87353	#N/A
MF_GO:0047298_(S)-3-amino-2-methylpropionate+transaminase+activity	0.87353	0.87353	#N/A
MF_GO:0047696_beta-adrenergic+receptor+kinase+activity	0.982753	0.399242	0.999411
MF_GO:0001729_ceramide+kinase+activity	0.40009	0.40009	#N/A
BP_GO:0008333_endosome+to+lysosome+transport	0.81992	0.187047	0.985463
MF_GO:0004660_protein+farnesyltransferase+activity	0.499691	0.499691	#N/A
BP_GO:0032230_positive+regulation+of+synaptic+transmission,+GABAergic	0.445146	#N/A	0.445146
BP_GO:0060081_membrane+hyperpolarization	0.953723	0.756819	0.953687
MF_GO:0030742_GTP-dependent+protein+binding	0.609417	0.766012	0.369617
BP_GO:0007051_spindle+organization	0.914588	0.698514	0.921605

BP_GO:0031401_positive+regulation+of+protein+modification+process	0.353408	0.353408	#N/A
BP_GO:0045542_positive+regulation+of+cholesterol+biosynthetic+process	0.770944	0.268519	0.952172
CC_GO:0016528_sarcoplasm	0.731994	0.120523	0.979688
CC_GO:0044428_nuclear+part	0.517846	0.162768	0.852367
BP_GO:0035024_negative+regulation+of+Rho+protein+signal+transduction	0.502692	0.502692	#N/A
MF_GO:0004591_oxoglutarate+dehydrogenase+(succinyl-transferring)+activ	0.575732	0.575732	#N/A
MF_GO:0004127_cytidylate+kinase+activity	0.21967	0.21967	#N/A
BP_GO:0016199_axon+midline+choice+point+recognition	0.99845	0.99845	#N/A
CC_GO:0042587_glycogen+granule	0.809078	0.525559	0.879447
BP_GO:0031648_protein+destabilization	0.46858	0.339416	0.619003
MF_GO:0004605_phosphatidate+cytidylyltransferase+activity	0.458311	0.719056	0.233377
MF_GO:0016857_racemase+and+epimerase+activity,+acting+on+carbohydra	0.370531	0.461268	0.355843
BP_GO:0006702_androgen+biosynthetic+process	0.614828	0.699003	0.456736
MF_GO:0016705_oxidoreductase+activity,+acting+on+paired+donors,+with-	0.843449	#N/A	0.843449
MF_GO:0004109_coproporphyrinogen+oxidase+activity	0.505954	0.514792	0.493719
MF_GO:0004348_glucosylceramidase+activity	0.984004	0.898195	0.960805
BP_GO:0033700_phospholipid+efflux	0.381015	0.381015	#N/A
BP_GO:0006772_thiamin+metabolic+process	0.643634	0.643634	#N/A
BP_GO:0008065_establishment+of+blood-nerve+barrier	0.614873	0.804886	0.32774
MF_GO:0004142_diacylglycerol+cholinephosphotransferase+activity	0.761756	0.150272	0.979417
BP_GO:0016482_cytoplasmic+transport	0.674757	0.870679	0.312443
BP_GO:0051781_positive+regulation+of+cell+division	0.153235	0.167062	0.315823
MF_GO:0010302_2-oxoglutarate-dependent+dioxygenase+activity	0.0798854	#N/A	0.0798854
MF_GO:0051747_DNA+demethylase+activity	0.0798854	#N/A	0.0798854
BP_GO:0021831_embryonic+olfactory+bulb+interneuron+precursor+migrati	0.925538	#N/A	0.925538
MF_GO:0003938_IMP+dehydrogenase+activity	0.0683642	0.513615	0.0162885
MF_GO:0047276_N-acetyllactosaminide+3-alpha-galactosyltransferase+activ	0.489837	0.486031	0.499703
BP_GO:0033364_mast+cell+secretory+granule+organization	0.11438	#N/A	0.11438
BP_GO:0051084_'de+novo'+posttranslational+protein+folding	0.991012	#N/A	0.991012
BP_GO:0008608_attachment+of+spindle+microtubules+to+kinetochore	0.742278	0.662206	0.691834
MF_GO:0008474_palmitoyl-(protein)+hydrolase+activity	0.273825	0.445872	0.23775
MF_GO:0004958_prostaglandin+F+receptor+activity	0.798599	0.712704	0.732862
BP_GO:0002924_negative+regulation+of+humoral+immune+response+medi	0.609996	0.657852	0.495385
CC_GO:0030125_clathrin+vesicle+coat	0.772257	0.73744	0.662629
BP_GO:0007171_activation+of+transmembrane+receptor+protein+tyrosine-	0.391506	#N/A	0.391506
BP_GO:0060152_microtubule-based+peroxisome+localization	0.938228	0.916081	0.787537
MF_GO:0005025_transforming+growth+factor+beta+receptor+activity,+type	0.682601	0.634637	0.628341
BP_GO:0045409_negative+regulation+of+interleukin-6+biosynthetic+proces	0.328517	#N/A	0.328517
BP_GO:0043206_fibril+organization	0.417366	0.417366	#N/A
BP_GO:0007185_transmembrane+receptor+protein+tyrosine+phosphatase+	0.948407	#N/A	0.948407
BP_GO:0014065_phosphoinositide+3-kinase+cascade	0.525702	0.65931	0.374784
BP_GO:0050982_detection+of+mechanical+stimulus	0.641218	0.641218	#N/A
BP_GO:0048016_inositol+phosphate-mediated+signaling	0.744082	0.744082	#N/A
BP_GO:0016045_detection+of+bacterium	0.558574	0.558574	#N/A
MF_GO:0030144_alpha-1,6-mannosyl-glycoprotein+6-beta-N-acetylglucosar	0.454039	0.643484	0.297797
BP_GO:0002352_B+cell+negative+selection	0.0946968	0.0946968	#N/A
BP_GO:0010524_positive+regulation+of+calcium+ion+transport+into+cytosc	0.0946968	0.0946968	#N/A

BP_GO:0032471_reduction+of+endoplasmic+reticulum+calcium+ion+concer	0.0946968	0.0946968	#N/A
BP_GO:0033137_negative+regulation+of+peptidyl-serine+phosphorylation	0.0946968	0.0946968	#N/A
BP_GO:0033081_regulation+of+T+cell+differentiation+in+the+thymus	0.0602048	0.18099	0.0996829
MF_GO:0004833_tryptophan+2,3-dioxygenase+activity	0.685523	0.888417	0.296354
MF_GO:0004691_cAMP-dependent+protein+kinase+activity	0.821496	0.684325	0.794559
BP_GO:0009609_response+to+symbiotic+bacterium	0.45086	0.45086	#N/A
BP_GO:0051702_interaction+with+symbiont	0.45086	0.45086	#N/A
BP_GO:0043266_regulation+of+potassium+ion+transport	0.758838	0.586897	0.780458
BP_GO:0051642_centrosome+localization	0.960023	0.960023	#N/A
MF_GO:0004477_methenyltetrahydrofolate+cyclohydrolase+activity	0.633135	0.633135	#N/A
MF_GO:0004488_methylenetetrahydrofolate+dehydrogenase+(NADP+)+act	0.633135	0.633135	#N/A
MF_GO:0004727_prenylated+protein+tyrosine+phosphatase+activity	0.578927	#N/A	0.578927
BP_GO:0045401_positive+regulation+of+interleukin-3+biosynthetic+process	0.883959	0.728818	0.859957
BP_GO:0045425_positive+regulation+of+granulocyte+macrophage+colony-s	0.883959	0.728818	0.859957
BP_GO:0002260_lymphocyte+homeostasis	0.657225	0.246873	0.89561
BP_GO:0015884_folic+acid+transport	0.762154	#N/A	0.762154
BP_GO:0032226_positive+regulation+of+synaptic+transmission,+dopaminer	0.208472	0.208472	#N/A
BP_GO:0033603_positive+regulation+of+dopamine+secretion	0.208472	0.208472	#N/A
BP_GO:0030828_positive+regulation+of+cGMP+biosynthetic+process	0.329444	#N/A	0.329444
MF_GO:0004704_NF-kappaB-inducing+kinase+activity	0.261273	0.261273	#N/A
MF_GO:0003692_left-handed+Z-DNA+binding	0.768488	0.288587	0.944653
BP_GO:0031396_regulation+of+protein+ubiquitination	0.495416	0.495416	#N/A
MF_GO:0001614_purinergic+nucleotide+receptor+activity	0.371828	0.675906	0.17924
BP_GO:0045010_actin+nucleation	0.593892	#N/A	0.593892
MF_GO:0042978_ornithine+decarboxylase+activator+activity	0.855911	0.611994	0.8882
BP_GO:0031296_B+cell+costimulation	0.465595	0.473174	0.478264
BP_GO:0035054_embryonic+heart+tube+anterior/posterior+pattern+forma	0.5167	0.5167	#N/A
MF_GO:0005128_erythropoietin+receptor+binding	0.708896	0.708896	#N/A
MF_GO:0004145_diamine+N-acetyltransferase+activity	0.683811	#N/A	0.683811
BP_GO:0060024_rhythmic+synaptic+transmission	0.975656	0.618129	0.993542
MF_GO:0004611_phosphoenolpyruvate+carboxykinase+activity	0.461692	0.61256	0.336626
MF_GO:0004613_phosphoenolpyruvate+carboxykinase+(GTP)+activity	0.461692	0.61256	0.336626
BP_GO:0001954_positive+regulation+of+cell-matrix+adhesion	0.751021	0.762833	0.595889
MF_GO:0019807_aspartoacylase+activity	0.975983	0.798941	0.974823
BP_GO:0009590_detection+of+gravity	0.0205329	#N/A	0.0205329
BP_GO:0002227_innate+immune+response+in+mucosa	0.942438	#N/A	0.942438
BP_GO:0048318_axial+mesoderm+development	0.85607	0.85607	#N/A
BP_GO:0043547_positive+regulation+of+GTPase+activity	0.473179	0.65931	0.306627
CC_GO:0031091_platelet+alpha+granule	0.844664	#N/A	0.844664
BP_GO:0051128_regulation+of+cellular+component+organization	0.113185	0.25313	0.147999
BP_GO:0021591_ventricular+system+development	0.607489	0.423758	0.718403
MF_GO:0001888_glucuronyl-galactosyl-proteoglycan+4-alpha-N-acetylgluco	0.51	0.51	#N/A
BP_GO:0050794_regulation+of+cellular+process	0.945946	0.78526	0.930733
CC_GO:0005968_Rab-protein+geranylgeranyltransferase+complex	0.29391	#N/A	0.29391
BP_GO:0007033_vacuole+organization	0.480057	#N/A	0.480057
BP_GO:0006925_inflammatory+cell+apoptosis	0.0241233	#N/A	0.0241233
CC_GO:0000805_X+chromosome	0.550108	0.550108	#N/A

CC_GO:0000806_Y+chromosome	0.550108	0.550108	#N/A
MF_GO:0035005_phosphatidylinositol-4-phosphate+3-kinase+activity	0.403854	#N/A	0.403854
BP_GO:0051875_pigment+granule+localization	0.671141	0.620566	0.625341
BP_GO:0051904_pigment+granule+transport	0.671141	0.620566	0.625341
BP_GO:0001780_neutrophil+homeostasis	0.349925	0.156821	0.678292
BP_GO:0006828_manganese+ion+transport	0.772225	0.720773	0.680762
MF_GO:0004765_shikimate+kinase+activity	0.313639	0.498318	0.247662
MF_GO:0050509_N-acetylglucosaminyl-proteoglycan+4-beta-glucuronosyltr	0.265644	0.265644	#N/A
BP_GO:0048478_replication+fork+protection	0.29009	0.805738	0.0501012
MF_GO:0004576_oligosaccharyl+transferase+activity	0.749133	0.757582	0.599178
MF_GO:0050839_cell+adhesion+molecule+binding	0.5232	0.127858	0.88859
MF_GO:0004637_phosphoribosylamine-glycine+ligase+activity	0.891988	#N/A	0.891988
BP_GO:0007098_centrosome+cycle	0.204559	0.3265	0.236717
BP_GO:0014054_positive+regulation+of+gamma-aminobutyric+acid+secreti	0.555565	#N/A	0.555565
MF_GO:0003829_beta-1,3-galactosyl-O-glycosyl-glycoprotein+beta-1,6-N-ac	0.708321	#N/A	0.708321
BP_GO:0006788_heme+oxidation	0.763421	0.763421	#N/A
MF_GO:0004392_heme+oxygenase+(decyclizing)+activity	0.763421	0.763421	#N/A
BP_GO:0006534_cysteine+metabolic+process	0.227235	0.227235	#N/A
BP_GO:0050883_musculoskeletal+movement,+spinal+reflex+action	0.991638	0.814936	0.993542
BP_GO:0002513_tolerance+induction+to+self+antigen	0.645587	0.645587	#N/A
BP_GO:0045066_regulatory+T+cell+differentiation	0.645587	0.645587	#N/A
BP_GO:0006029_proteoglycan+metabolic+process	0.921458	0.839284	0.843446
BP_GO:0051799_negative+regulation+of+hair+follicle+development	0.657196	0.563395	0.660149
MF_GO:0005026_transforming+growth+factor+beta+receptor+activity,+type	0.773279	0.759107	0.639259
MF_GO:0050321_tau-protein+kinase+activity	0.409848	0.154915	0.756046
BP_GO:0015860_purine+nucleoside+transport	0.101228	0.338659	0.0829919
MF_GO:0005415_nucleoside:sodium+symporter+activity	0.101228	0.338659	0.0829919
BP_GO:0045739_positive+regulation+of+DNA+repair	0.277705	#N/A	0.277705
BP_GO:0022010_myelination+in+the+central+nervous+system	0.955354	0.8544	0.910921
BP_GO:0033092_positive+regulation+of+immature+T+cell+proliferation+in+	0.257645	0.419406	0.237003
MF_GO:0003870_5-aminolevulinate+synthase+activity	0.889939	#N/A	0.889939
BP_GO:0048730_epidermis+morphogenesis	0.377299	0.155264	0.716519
MF_GO:0050544_arachidonic+acid+binding	0.250785	0.533717	0.150508
MF_GO:0030306_ADP-ribosylation+factor+binding	0.60543	0.60543	#N/A
BP_GO:0019230_proprioception	0.0989897	0.423758	0.0518608
MF_GO:0031386_protein+tag	0.249373	0.114445	0.597663
MF_GO:0004963_follicle-stimulating+hormone+receptor+activity	0.538743	0.46037	0.593762
BP_GO:0032831_positive+regulation+of+CD4-positive,+CD25-positive,+alpha	0.523364	0.523364	#N/A
BP_GO:0006625_protein+targeting+to+peroxisome	0.0267507	0.0267507	#N/A
MF_GO:0003827_alpha-1,3-mannosylglycoprotein+2-beta-N-acetylglucosam	0.0423113	0.0423113	#N/A
BP_GO:0019348_dolichol+metabolic+process	0.109902	0.401994	0.0686439
CC_GO:0033185_dolichol-phosphate-mannose+synthase+complex	0.109902	0.401994	0.0686439
MF_GO:0004582_dolichyl-phosphate+beta-D-mannosyltransferase+activity	0.109902	0.401994	0.0686439
BP_GO:0008612_peptidyl-lysine+modification+to+hypusine	0.0292012	0.0292012	#N/A
BP_GO:0048199_vesicle+targeting,+to,+from+or+within+Golgi	0.654942	0.278167	0.875358
BP_GO:0008209_androgen+metabolic+process	0.346084	0.141154	0.696996
BP_GO:0006621_protein+retention+in+ER+lumen	0.0524518	0.0524518	#N/A

MF_GO:0046923_ER+retention+sequence+binding	0.0524518	0.0524518	#N/A
CC_GO:0005665_DNA-directed+RNA+polymerase+II,+core+complex	0.958898	0.60786	0.985488
BP_GO:0035089_establishment+of+apical/basal+cell+polarity	0.470062	#N/A	0.470062
BP_GO:0001946_lymphangiogenesis	0.126952	#N/A	0.126952
BP_GO:0016926_protein+desumoylation	0.307915	0.569911	0.188041
BP_GO:0009059_macromolecule+biosynthetic+process	0.215302	0.215302	#N/A
BP_GO:0014056_regulation+of+acetylcholine+secretion	0.966714	#N/A	0.966714
MF_GO:0000268_peroxisome+targeting+sequence+binding	0.388664	0.115576	0.787537
MF_GO:0004911_interleukin-2+receptor+activity	0.499707	0.584973	0.414718
BP_GO:0043044_ATP-dependent+chromatin+remodeling	0.520069	#N/A	0.520069
MF_GO:0031491_nucleosome+binding	0.520069	#N/A	0.520069
BP_GO:0046668_regulation+of+retinal+cell+programmed+cell+death	0.882339	#N/A	0.882339
BP_GO:0002224_toll-like+receptor+signaling+pathway	0.977346	#N/A	0.977346
MF_GO:0019215_intermediate+filament+binding	0.799758	0.889071	0.486849
BP_GO:0019307_mannose+biosynthetic+process	0.165556	0.0250071	0.721184
MF_GO:0004615_phosphomannomutase+activity	0.165556	0.0250071	0.721184
BP_GO:0014031_mesenchymal+cell+development	0.787446	0.448558	0.89561
CC_GO:0030892_mitotic+cohesin+complex	0.995247	0.995247	#N/A
MF_GO:0003989_acetyl-CoA+carboxylase+activity	0.372719	0.508507	0.315622
BP_GO:0042036_negative+regulation+of+cytokine+biosynthetic+process	0.0538291	0.283321	0.0444941
MF_GO:0015056_corticotrophin-releasing+factor+receptor+activity	0.491589	0.177532	0.814674
MF_GO:0045309_protein+phosphorylated+amino+acid+binding	0.543334	0.477343	0.58352
MF_GO:0000774_adenyl-nucleotide+exchange+factor+activity	0.43026	0.11728	0.826539
BP_GO:0002790_peptide+secretion	0.579597	0.432715	0.67495
BP_GO:0035282_segmentation	0.637721	0.372193	0.795135
BP_GO:0045667_regulation+of+osteoblast+differentiation	0.139537	0.146793	0.315823
BP_GO:0031017_exocrine+pancreas+development	0.24583	0.24583	#N/A
BP_GO:0043433_negative+regulation+of+transcription+factor+activity	0.450259	0.418031	0.512159
BP_GO:0048537_mucosal-associated+lymphoid+tissue+development	0.793762	0.792494	0.634406
MF_GO:0003884_D-amino-acid+oxidase+activity	0.363601	0.363601	#N/A
BP_GO:0032435_negative+regulation+of+proteasomal+ubiquitin-dependent	0.313884	0.114898	0.696996
MF_GO:0005353_fructose+transmembrane+transporter+activity	0.83356	0.917939	0.49107
BP_GO:0006587_serotonin+biosynthetic+process+from+tryptophan	0.427235	0.669144	0.243039
BP_GO:0042427_serotonin+biosynthetic+process	0.427235	0.669144	0.243039
MF_GO:0004510_tryptophan+5-monooxygenase+activity	0.427235	0.669144	0.243039
BP_GO:0046883_regulation+of+hormone+secretion	0.559564	#N/A	0.559564
MF_GO:0004452_isopentenyl-diphosphate+delta-isomerase+activity	0.986599	0.82471	0.985982
BP_GO:0034644_cellular+response+to+UV	0.704194	0.0579951	0.990108
MF_GO:0019911_structural+constituent+of+myelin+sheath	0.227798	0.227798	#N/A
BP_GO:0043508_negative+regulation+of+JNK+activity	0.966864	#N/A	0.966864
MF_GO:0030272_5-formyltetrahydrofolate+cyclo-ligase+activity	0.023462	0.023462	#N/A
BP_GO:0007258_JUN+phosphorylation	0.203866	0.535276	0.104106
BP_GO:0030328_prenylcysteine+catabolic+process	0.611691	0.626396	0.53147
MF_GO:0016670_oxidoreductase+activity,+acting+on+sulfur+group+of+don	0.611691	0.626396	0.53147
BP_GO:0048619_embryonic+hindgut+morphogenesis	0.138409	#N/A	0.138409
BP_GO:0001504_neurotransmitter+uptake	0.0541681	0.0307204	0.344916
BP_GO:0006488_dolichol-linked+oligosaccharide+biosynthetic+process	0.798613	0.693326	0.750977

BP_GO:0016577_histone+demethylation	0.333571	0.333571	#N/A
BP_GO:0048711_positive+regulation+of+astrocyte+differentiation	0.0365741	0.0126494	0.383708
BP_GO:0034612_response+to+tumor+necrosis+factor	0.256229	0.00914161	0.924247
BP_GO:0045143_homologous+chromosome+segregation	0.337714	0.0914479	0.770441
MF_GO:0004475_mannose-1-phosphate+guanylyltransferase+activity	0.490394	0.490394	#N/A
BP_GO:0042095_interferon-gamma+biosynthetic+process	0.408724	0.408724	#N/A
MF_GO:0043790_dimethyladenosine+transferase+activity	0.942541	0.942541	#N/A
MF_GO:0015036_disulfide+oxidoreductase+activity	0.792243	#N/A	0.792243
MF_GO:0004314_[acyl-carrier-protein]+S-malonyltransferase+activity	0.26864	0.26864	#N/A
CC_GO:0002080_acrosomal+membrane	0.646699	0.291765	0.86005
MF_GO:0004320_oleoyl-[acyl-carrier-protein]+hydrolase+activity	0.638336	0.525559	0.668772
CC_GO:0000137_Golgi+cis+cisterna	0.386578	#N/A	0.386578
CC_GO:0005915_zonula+adherens	0.705179	0.705179	#N/A
MF_GO:0030350_iron-responsive+element+binding	0.225723	#N/A	0.225723
MF_GO:0016888_endodeoxyribonuclease+activity,+producing+5'-phosphom	0.0364893	0.0128649	0.380641
MF_GO:0070052_collagen+V+binding	0.987446	#N/A	0.987446
BP_GO:0045063_T-helper+1+cell+differentiation	0.794757	0.311476	0.951064
MF_GO:0001566_non-kinase+phorbol+ester+receptor+activity	0.971906	#N/A	0.971906
CC_GO:0042588_zymogen+granule	0.951499	#N/A	0.951499
CC_GO:0031233_intrinsic+to+external+side+of+plasma+membrane	0.46338	0.435938	0.512601
BP_GO:0032319_regulation+of+Rho+GTPase+activity	0.799804	0.799804	#N/A
MF_GO:0001965_G-protein+alpha-subunit+binding	0.236659	0.236659	#N/A
MF_GO:0042813_Wnt+receptor+activity	0.977996	0.977996	#N/A
MF_GO:0019210_kinase+inhibitor+activity	0.870943	0.870943	#N/A
BP_GO:0006613_cotranslational+protein+targeting+to+membrane	0.591215	0.388453	0.728953
CC_GO:0005784_translocon+complex	0.591215	0.388453	0.728953
BP_GO:0009798_axis+specification	0.536408	#N/A	0.536408
MF_GO:0004360_glutamine-fructose-6-phosphate+transaminase+(isomerizi	0.833881	#N/A	0.833881
BP_GO:0055009_atrial+cardiac+muscle+morphogenesis	0.731929	0.731929	#N/A
MF_GO:0004422_hypoxanthine+phosphoribosyltransferase+activity	0.804773	0.781887	0.668417
BP_GO:0015746_citrate+transport	0.817501	#N/A	0.817501
MF_GO:0015137_citrate+transmembrane+transporter+activity	0.817501	#N/A	0.817501
BP_GO:0000076_DNA+replication+checkpoint	0.358317	0.510436	0.29496
BP_GO:0040009_regulation+of+growth+rate	0.713507	0.37653	0.866839
BP_GO:0009088_threonine+biosynthetic+process	0.796209	0.498318	0.880011
MF_GO:0004795_threonine+synthase+activity	0.796209	0.498318	0.880011
BP_GO:0048547_gut+morphogenesis	0.973887	#N/A	0.973887
BP_GO:0010592_positive+regulation+of+lamellipodium+assembly	0.811018	0.670682	0.789486
BP_GO:0045136_development+of+secondary+sexual+characteristics	0.108801	0.0579951	0.432253
BP_GO:0060058_positive+regulation+of+apoptosis+involved+in+mammary+	0.108801	0.0579951	0.432253
BP_GO:0043067_regulation+of+programmed+cell+death	0.830408	0.53756	0.89561
BP_GO:0018193_peptidyl-amino+acid+modification	0.506298	#N/A	0.506298
BP_GO:0043043_peptide+biosynthetic+process	0.838509	#N/A	0.838509
BP_GO:0006796_phosphate+metabolic+process	0.231681	0.231681	#N/A
BP_GO:0046950_cellular+ketone+body+metabolic+process	0.18716	0.18716	#N/A
MF_GO:0008260_3-oxoacid+CoA-transferase+activity	0.18716	0.18716	#N/A
BP_GO:0006119_oxidative+phosphorylation	0.199572	#N/A	0.199572

BP_GO:0042249_establishment+of+polarity+of+embryonic+epithelium	0.431456	0.299735	0.610777
BP_GO:0002031_G-protein+coupled+receptor+internalization	0.346476	0.364235	0.416558
BP_GO:0046671_negative+regulation+of+retinal+cell+programmed+cell+dea	0.7637	0.404606	0.89561
CC_GO:0016533_cyclin-dependent+protein+kinase+5+activator+complex	0.5303	#N/A	0.5303
MF_GO:0016534_cyclin-dependent+protein+kinase+5+activator+activity	0.5303	#N/A	0.5303
MF_GO:0034211_GTP-dependent+protein+kinase+activity	0.578074	#N/A	0.578074
MF_GO:0004534_5'-3'+exoribonuclease+activity	0.803963	0.549021	0.861436
BP_GO:0045627_positive+regulation+of+T-helper+1+cell+differentiation	0.891959	0.891959	#N/A
CC_GO:0030056_hemidesmosome	0.966904	0.980691	0.701507
MF_GO:0008177_succinate+dehydrogenase+(ubiquinone)+activity	0.0643698	0.0643698	#N/A
BP_GO:0021587_cerebellum+morphogenesis	0.936298	0.711895	0.94476
BP_GO:0007354_zygotic+determination+of+anterior/posterior+axis,+embry	0.396727	#N/A	0.396727
BP_GO:0001705_ectoderm+formation	0.937191	0.935633	0.74087
BP_GO:0015696_ammonium+transport	0.920722	0.920722	#N/A
MF_GO:0005143_interleukin-12+receptor+binding	0.300322	0.295295	0.420079
BP_GO:0045581_negative+regulation+of+T+cell+differentiation	0.0870539	0.0870539	#N/A
BP_GO:0000720_pyrimidine+dimer+repair+via+nucleotide-excision+repair	0.330439	0.330439	#N/A
MF_GO:0004909_interleukin-1,+Type+I,+activating+receptor+activity	0.584394	#N/A	0.584394
BP_GO:0010216_maintenance+of+DNA+methylation	0.202932	#N/A	0.202932
BP_GO:0030431_sleep	0.952819	0.952819	#N/A
BP_GO:0022612_gland+morphogenesis	0.871208	#N/A	0.871208
BP_GO:0045916_negative+regulation+of+complement+activation	0.909926	0.917202	0.694354
BP_GO:0006200_ATP+catabolic+process	0.549495	#N/A	0.549495
MF_GO:0016492_neurotensin+receptor+activity,+G-protein+coupled	0.483184	0.483184	#N/A
MF_GO:0005123_death+receptor+binding	0.936559	0.936559	#N/A
BP_GO:0015888_thiamin+transport	0.992064	0.893616	0.984743
MF_GO:0015234_thiamin+transmembrane+transporter+activity	0.992064	0.893616	0.984743
BP_GO:0007052_mitotic+spindle+organization	0.450922	0.230124	0.713745
MF_GO:0004043_L-aminoadipate-semialdehyde+dehydrogenase+activity	0.932225	0.932225	#N/A
BP_GO:0006893_Golgi+to+plasma+membrane+transport	0.624527	0.675645	0.497334
MF_GO:0015270_dihydropyridine-sensitive+calcium+channel+activity	0.645753	0.645753	#N/A
BP_GO:0045821_positive+regulation+of+glycolysis	0.688625	0.688625	#N/A
MF_GO:0005176_ErbB-2+class+receptor+binding	0.470251	0.470251	#N/A
BP_GO:0006944_membrane+fusion	0.737029	#N/A	0.737029
MF_GO:0008432_JUN+kinase+binding	0.952368	0.986986	0.552706
MF_GO:0042809_vitamin+D+receptor+binding	0.719175	0.535791	0.767543
MF_GO:0045523_interleukin-27+receptor+binding	0.512308	#N/A	0.512308
MF_GO:0050062_long-chain-fatty-acyl-CoA+reductase+activity	0.0103605	#N/A	0.0103605
BP_GO:0060060_post-embryonic+retina+morphogenesis+in+camera-type+e	0.272685	0.307692	0.362383
MF_GO:0070064_proline-rich+region+binding	0.886242	0.503397	0.955198
BP_GO:0006370_mRNA+capping	0.0154447	#N/A	0.0154447
BP_GO:0008089_anterograde+axon+cargo+transport	0.94737	#N/A	0.94737
BP_GO:0048295_positive+regulation+of+isotype+switching+to+IgE+isotypes	0.75872	#N/A	0.75872
MF_GO:0005134_interleukin-2+receptor+binding	0.651278	0.103635	0.96492
MF_GO:0015501_glutamate:sodium+symporter+activity	0.864051	0.864051	#N/A
BP_GO:0031848_protection+from+non-homologous+end+joining+at+telome	0.539133	0.372193	0.679095
BP_GO:0015889_cobalamin+transport	0.62432	#N/A	0.62432

BP_GO:0042048_olfactory+behavior	0.967999	0.598387	0.991083
CC_GO:0017177_alpha-glucosidase+II+complex	0.575201	0.575201	#N/A
BP_GO:0031630_regulation+of+synaptic+vesicle+fusion+to+presynaptic+me	0.0175095	0.0175095	#N/A
MF_GO:0004307_ethanolaminephosphotransferase+activity	0.951708	0.620612	0.979417
BP_GO:0051295_establishment+of+meiotic+spindle+localization	0.156121	0.156121	#N/A
BP_GO:0032330_regulation+of+chondrocyte+differentiation	0.00399824	0.00399824	#N/A
BP_GO:0006398_histone+mRNA+3'-end+processing	0.370735	0.370735	#N/A
BP_GO:0032753_positive+regulation+of+interleukin-4+production	0.817148	0.414229	0.932606
BP_GO:0047484_regulation+of+response+to+osmotic+stress	0.194706	0.194706	#N/A
MF_GO:0042800_histone+lysine+N-methyltransferase+activity+(H3-K4+spec	0.822731	0.60343	0.852367
BP_GO:0042594_response+to+starvation	0.316021	0.316021	#N/A
BP_GO:0015858_nucleoside+transport	0.868802	0.41105	0.964793
MF_GO:0003823_antigen+binding	0.417945	#N/A	0.417945
BP_GO:0042267_natural+killer+cell+mediated+cytotoxicity	0.320757	0.245624	0.512159
BP_GO:0006659_phosphatidylserine+biosynthetic+process	0.876318	0.876318	#N/A
BP_GO:0006429_leucyl-tRNA+aminoacylation	0.426851	0.426851	#N/A
MF_GO:0004823_leucine-tRNA+ligase+activity	0.426851	0.426851	#N/A
BP_GO:0045742_positive+regulation+of+epidermal+growth+factor+recepto	0.946813	0.960118	0.70213
MF_GO:0017057_6-phosphogluconolactonase+activity	0.486445	0.486445	#N/A
BP_GO:0030147_natriuresis	0.622782	#N/A	0.622782
MF_GO:0017077_oxidative+phosphorylation+uncoupler+activity	0.615066	0.615066	#N/A
MF_GO:0008746_NAD(P)+transhydrogenase+activity	0.721576	0.721576	#N/A
MF_GO:0008750_NAD(P)++transhydrogenase+(AB-specific)+activity	0.721576	0.721576	#N/A
BP_GO:0051302_regulation+of+cell+division	0.0849891	0.0849891	#N/A
BP_GO:0007042_lysosomal+lumen+acidification	0.889755	0.889755	#N/A
MF_GO:0008339_MP+kinase+activity	0.495526	0.495526	#N/A
BP_GO:0006301_postreplication+repair	0.458616	0.280219	0.668417
MF_GO:0051864_histone+demethylase+activity+(H3-K36+specific)	0.602034	0.602034	#N/A
BP_GO:0016540_protein+autoprocessing	0.4801	0.52871	0.443414
MF_GO:0004310_farnesyl-diphosphate+farnesyltransferase+activity	0.801565	#N/A	0.801565
MF_GO:0004329_formate-tetrahydrofolate+ligase+activity	0.243436	0.544922	0.136676
MF_GO:0031489_myosin+V+binding	0.588925	0.499373	0.625341
BP_GO:0001839_neural+plate+morphogenesis	0.824154	#N/A	0.824154
MF_GO:0042289_MHC+class+II+protein+binding	0.70576	0.300897	0.90092
BP_GO:0016048_detection+of+temperature+stimulus	0.702594	#N/A	0.702594
BP_GO:0050906_detection+of+stimulus+involved+in+sensory+perception	0.709345	#N/A	0.709345
BP_GO:0007589_body+fluid+secretion	0.613765	#N/A	0.613765
BP_GO:0042773_ATP+synthesis+coupled+electron+transport	0.145945	0.0226997	0.695464
BP_GO:0035117_embryonic+arm+morphogenesis	0.337715	0.337715	#N/A
BP_GO:0009296_flagellum+assembly	0.44409	0.44409	#N/A
CC_GO:0031514_motile+secondary+cilium	0.44409	0.44409	#N/A
BP_GO:0030910_olfactory+placode+formation	0.527785	#N/A	0.527785
BP_GO:0043537_negative+regulation+of+blood+vessel+endothelial+cell+mi	0.307383	0.737422	0.089063
BP_GO:0030330_DNA+damage+response,+signal+transduction+by+p53+clas	0.892263	0.281174	0.990108
BP_GO:0019478_D-amino+acid+catabolic+process	0.246678	0.246678	#N/A
BP_GO:0032225_regulation+of+synaptic+transmission,+dopaminergic	0.850571	0.854831	0.659575
CC_GO:0000164_protein+phosphatase+type+1+complex	0.814329	#N/A	0.814329

BP_GO:0002358_B+cell+homeostatic+proliferation	0.138106	0.138106	#N/A
BP_GO:0046013_regulation+of+T+cell+homeostatic+proliferation	0.924046	0.584973	0.96492
BP_GO:0021510_spinal+cord+development	0.212424	0.209386	0.374784
BP_GO:0007320_insemination	0.739744	0.739744	#N/A
MF_GO:0008171_O-methyltransferase+activity	0.643566	0.146494	0.942025
MF_GO:0004535_poly(A)-specific+ribonuclease+activity	0.769984	0.511663	0.845013
MF_GO:0019767_IgE+receptor+activity	0.973527	#N/A	0.973527
BP_GO:0006083_acetate+metabolic+process	0.550851	0.550851	#N/A
BP_GO:0042129_regulation+of+T+cell+proliferation	0.821453	0.399587	0.940188
BP_GO:0048863_stem+cell+differentiation	0.574461	#N/A	0.574461
CC_GO:0045298_tubulin+complex	0.189977	0.189977	#N/A
BP_GO:0050862_positive+regulation+of+T+cell+receptor+signaling+pathway	0.530092	0.530092	#N/A
BP_GO:0005989_lactose+biosynthetic+process	0.429492	#N/A	0.429492
BP_GO:0030516_regulation+of+axon+extension	0.769477	0.390406	0.906651
BP_GO:0032924_activin+receptor+signaling+pathway	0.270824	0.522843	0.178859
MF_GO:0017002_activin+receptor+activity	0.270824	0.522843	0.178859
MF_GO:0030515_snoRNA+binding	0.23208	0.23208	#N/A
BP_GO:0045124_regulation+of+bone+resorption	0.575892	#N/A	0.575892
MF_GO:0003835_beta-galactoside+alpha-2,6-sialyltransferase+activity	0.612298	#N/A	0.612298
MF_GO:0019207_kinase+regulator+activity	0.357608	#N/A	0.357608
BP_GO:0043569_negative+regulation+of+insulin-like+growth+factor+recept	0.518829	0.289259	0.733204
BP_GO:0045076_regulation+of+interleukin-2+biosynthetic+process	0.618182	0.399587	0.751655
CC_GO:0030893_meiotic+cohesin+complex	0.268833	#N/A	0.268833
BP_GO:0043297_apical+junction+assembly	0.954046	0.97123	0.685578
CC_GO:0005838_proteasome+regulatory+particle	0.290689	0.131373	0.633379
CC_GO:0032998_Fc-epsilon+receptor+I+complex	0.480064	#N/A	0.480064
MF_GO:0004918_interleukin-8+receptor+activity	0.0411683	#N/A	0.0411683
BP_GO:0042487_regulation+of+odontogenesis+of+dentine-containing+tootl	0.370531	#N/A	0.370531
CC_GO:0000783_nuclear+telomere+cap+complex	0.286713	0.372193	0.319353
CC_GO:0005712_chiasma	0.649393	0.649393	#N/A
CC_GO:0031201_SNARE+complex	0.981941	0.782255	0.985463
BP_GO:0032788_saturated+monocarboxylic+acid+metabolic+process	0.625944	0.42219	0.742299
BP_GO:0032789_unsaturated+monocarboxylic+acid+metabolic+process	0.625944	0.42219	0.742299
BP_GO:0009063_amino+acid+catabolic+process	0.587126	0.587126	#N/A
BP_GO:0046666_retinal+cell+programmed+cell+death	0.39272	0.39272	#N/A
MF_GO:0004525_ribonuclease+III+activity	0.254921	0.0229165	0.856692
MF_GO:0033842_N-acetyl-beta-glucosaminyl-glycoprotein+4-beta-N-acetylg	0.461499	0.34411	0.604455
BP_GO:0021912_regulation+of+transcription+from+RNA+polymerase+II+prc	0.969747	0.904397	0.910921
BP_GO:0045686_negative+regulation+of+glial+cell+differentiation	0.969747	0.904397	0.910921
BP_GO:0045687_positive+regulation+of+glial+cell+differentiation	0.969747	0.904397	0.910921
BP_GO:0006703_estrogen+biosynthetic+process	0.779502	0.779502	#N/A
BP_GO:0035129_post-embryonic+hindlimb+morphogenesis	0.376059	0.474336	0.351296
BP_GO:0050878_regulation+of+body+fluid+levels	0.957063	#N/A	0.957063
MF_GO:0003720_telomerase+activity	0.448805	0.451944	0.475723
BP_GO:0021524_visceral+motor+neuron+differentiation	0.834542	0.796163	0.70765
BP_GO:0050801_ion+homeostasis	0.938119	#N/A	0.938119
CC_GO:0005785_signal+recognition+particle+receptor+complex	0.88279	#N/A	0.88279

CC_GO:0016327_apicolateral+plasma+membrane	0.813527	0.920723	0.440248
BP_GO:0009912_auditory+receptor+cell+fate+commitment	0.154346	0.0155045	0.763722
BP_GO:0060023_soft+palate+development	0.4787	0.4787	#N/A
MF_GO:0016299_regulator+of+G-protein+signaling+activity	0.165751	#N/A	0.165751
BP_GO:0046068_cGMP+metabolic+process	0.993511	#N/A	0.993511
CC_GO:0005742_mitochondrial+outer+membrane+translocase+complex	0.790645	0.900853	0.443083
MF_GO:0004569_glycoprotein+endo-alpha-1,2-mannosidase+activity	0.913404	#N/A	0.913404
BP_GO:0006977_DNA+damage+response,+signal+transduction+by+p53+clas	0.591065	0.758059	0.354082
MF_GO:0043398_HLH+domain+binding	0.952074	#N/A	0.952074
MF_GO:0004523_ribonuclease+H+activity	0.937271	#N/A	0.937271
BP_GO:0006168_adenine+salvage	0.276585	0.276585	#N/A
BP_GO:0046083_adenine+metabolic+process	0.276585	0.276585	#N/A
BP_GO:0007622_rhythmic+behavior	0.918504	0.918504	#N/A
BP_GO:0021978_telencephalon+regionalization	0.964659	0.979292	0.696996
BP_GO:0051482_elevation+of+cytosolic+calcium+ion+concentration+during	0.17375	#N/A	0.17375
BP_GO:0016198_axon+choice+point+recognition	0.786604	0.786604	#N/A
CC_GO:0030688_preribosome,+small+subunit+precursor	0.391165	0.391165	#N/A
BP_GO:0045793_positive+regulation+of+cell+size	0.977103	0.960758	0.856294
BP_GO:0021506_anterior+neuropore+closure	0.448964	0.48093	0.446997
BP_GO:0007468_regulation+of+rhodopsin+gene+expression	0.874861	0.955655	0.469285
MF_GO:0008481_sphinganine+kinase+activity	0.267931	0.571067	0.145964
CC_GO:0005852_eukaryotic+translation+initiation+factor+3+complex	0.217279	0.217279	#N/A
BP_GO:0045872_positive+regulation+of+rhodopsin+gene+expression	0.479504	#N/A	0.479504
BP_GO:0048671_negative+regulation+of+collateral+sprouting	0.835219	0.835219	#N/A
BP_GO:0045950_negative+regulation+of+mitotic+recombination	0.560721	0.381584	0.697611
BP_GO:0021670_lateral+ventricle+development	0.135119	0.543249	0.0477817
BP_GO:0048659_smooth+muscle+cell+proliferation	0.942389	0.942389	#N/A
BP_GO:0033088_negative+regulation+of+immature+T+cell+proliferation+in-	0.216066	0.611265	0.0818516
BP_GO:0051932_synaptic+transmission,+GABAergic	0.976635	#N/A	0.976635
BP_GO:0040036_regulation+of+fibroblast+growth+factor+receptor+signalin	0.404929	#N/A	0.404929
MF_GO:0005166_neurotrophin+p75+receptor+binding	0.127128	0.037553	0.566943
BP_GO:0030520_estrogen+receptor+signaling+pathway	0.522787	0.698882	0.32991
BP_GO:0048732_gland+development	0.121086	0.369855	0.0933032
BP_GO:0021754_facial+nucleus+development	0.189317	#N/A	0.189317
BP_GO:0016071_mRNA+metabolic+process	0.365376	0.3027	0.512159
MF_GO:0015204_urea+transmembrane+transporter+activity	0.672793	0.779442	0.445381
BP_GO:0019276_UDP-N-acetylgalactosamine+metabolic+process	0.91337	0.876108	0.779164
MF_GO:0008376_acetylgalactosaminyltransferase+activity	0.91337	0.876108	0.779164
BP_GO:0045079_negative+regulation+of+chemokine+biosynthetic+process	0.528451	0.792431	0.237691
MF_GO:0030060_L-malate+dehydrogenase+activity	0.136678	0.136678	#N/A
MF_GO:0005185_neurohypophyseal+hormone+activity	0.512412	#N/A	0.512412
BP_GO:0031571_G1+DNA+damage+checkpoint	0.916776	#N/A	0.916776
BP_GO:0032922_circadian+regulation+of+gene+expression	0.881257	0.814936	0.780458
BP_GO:0060160_negative+regulation+of+dopamine+receptor+signaling+pat	0.881257	0.814936	0.780458
BP_GO:0021775_smoothened+signaling+pathway+involved+in+ventral+spin	0.834046	#N/A	0.834046
BP_GO:0021776_smoothened+signaling+pathway+involved+in+spinal+cord+	0.834046	#N/A	0.834046
BP_GO:0035295_tube+development	0.834046	#N/A	0.834046

BP_GO:0042270_protection+from+natural+killer+cell+mediated+cytotoxicity	0.517112	0.418814	0.604811
MF_GO:0008518_reduced+folate+carrier+activity	0.996935	#N/A	0.996935
MF_GO:0047485_protein+N-terminus+binding	0.250759	0.730215	0.0590008
MF_GO:0045294_alpha-catenin+binding	0.564427	0.146793	0.899693
MF_GO:0004666_prostaglandin-endoperoxide+synthase+activity	0.639596	0.798419	0.370474
BP_GO:0032494_response+to+peptidoglycan	0.928931	#N/A	0.928931
BP_GO:0032874_positive+regulation+of+stress-activated+MAPK+cascade	0.928931	#N/A	0.928931
BP_GO:0022038_corpus+callosum+development	0.275252	0.794789	0.0477817
MF_GO:0005113_patched+binding	0.510546	#N/A	0.510546
BP_GO:0006104_succinyl-CoA+metabolic+process	0.268573	0.533087	0.169766
BP_GO:0006431_methionyl-tRNA+aminoacylation	0.30554	0.30554	#N/A
MF_GO:0004825_methionine-tRNA+ligase+activity	0.30554	0.30554	#N/A
BP_GO:0006601_creatine+biosynthetic+process	0.471724	0.264958	0.701289
CC_GO:0016580_Sin3+complex	0.436973	0.164456	0.774068
MF_GO:0046404_ATP-dependent+polydeoxyribonucleotide+5'-hydroxyl-kina	0.0678252	0.168297	0.125483
MF_GO:0019153_protein-disulfide+reductase+(glutathione)+activity	0.437192	#N/A	0.437192
BP_GO:0001881_receptor+recycling	0.24726	0.24726	#N/A
CC_GO:0005682_snRNP+U5	0.283814	0.283814	#N/A
BP_GO:0032635_interleukin-6+production	0.641135	0.686565	0.510001
BP_GO:0007020_microtubule+nucleation	0.454863	0.454863	#N/A
MF_GO:0001607_neuromedin+U+receptor+activity	0.992888	0.992888	#N/A
MF_GO:0047522_15-oxoprostaglandin+13-oxidase+activity	0.396857	0.398445	0.455416
MF_GO:0004921_interleukin-11+receptor+activity	0.328061	0.288946	0.471034
MF_GO:0019970_interleukin-11+binding	0.328061	0.288946	0.471034
BP_GO:0055014_atrial+cardiac+muscle+cell+development	0.649866	0.679948	0.530617
MF_GO:0032405_MutLalpha+complex+binding	0.0798226	0.215438	0.115112
MF_GO:0032407_MutSalpha+complex+binding	0.0798226	0.215438	0.115112
MF_GO:0004103_choline+kinase+activity	0.385132	0.191437	0.677272
BP_GO:0045910_negative+regulation+of+DNA+recombination	0.376665	#N/A	0.376665
CC_GO:0032301_MutSalpha+complex	0.376665	#N/A	0.376665
CC_GO:0005673_transcription+factor+TFIIE+complex	0.372757	0.596696	0.24093
BP_GO:0016255_attachment+of+GPI+anchor+to+protein	0.928024	#N/A	0.928024
BP_GO:0008037_cell+recognition	0.371782	#N/A	0.371782
BP_GO:0051606_detection+of+stimulus	0.371782	#N/A	0.371782
MF_GO:0033754_indoleamine+2,3-dioxygenase+activity	0.799748	0.888417	0.488205
CC_GO:0019028_viral+capsid	0.535514	0.69157	0.354178
MF_GO:0004649_poly(ADP-ribose)+glycohydrolase+activity	0.133975	0.133975	#N/A
MF_GO:0019888_protein+phosphatase+regulator+activity	0.0963781	0.0963781	#N/A
MF_GO:0005095_GTPase+inhibitor+activity	0.527128	#N/A	0.527128
BP_GO:0007021_tubulin+complex+assembly	0.300217	0.44899	0.270307
BP_GO:0006433_prolyl-tRNA+aminoacylation	0.475684	0.178156	0.798551
MF_GO:0004827_proline-tRNA+ligase+activity	0.475684	0.178156	0.798551
MF_GO:0019212_phosphatase+inhibitor+activity	0.472115	0.280002	0.685879
MF_GO:0001785_prostaglandin+J+receptor+activity	0.644547	0.644547	#N/A
MF_GO:0004956_prostaglandin+D+receptor+activity	0.644547	0.644547	#N/A
MF_GO:0043566_structure-specific+DNA+binding	0.0375922	0.381584	0.0134323
BP_GO:0010454_negative+regulation+of+cell+fate+commitment	0.881178	#N/A	0.881178

CC_GO:0055037_recycling+endosome	0.936943	0.63955	0.964467
MF_GO:0047196_long-chain-alcohol+O-fatty-acyltransferase+activity	0.853326	0.716977	0.819022
BP_GO:0008634_negative+regulation+of+survival+gene+product+expression	0.78043	#N/A	0.78043
MF_GO:0035240_dopamine+binding	0.859604	#N/A	0.859604
BP_GO:0015819_lysine+transport	0.335672	0.71729	0.120136
MF_GO:0015189_L-lysine+transmembrane+transporter+activity	0.335672	0.71729	0.120136
BP_GO:0006047_UDP-N-acetylglucosamine+metabolic+process	0.985191	#N/A	0.985191
MF_GO:0004174_electron-transferring-flavoprotein+dehydrogenase+activity	0.504337	0.504337	#N/A
BP_GO:0008105_asymmetric+protein+localization	0.818051	0.678424	0.793987
BP_GO:0050974_detection+of+mechanical+stimulus+involved+in+sensory+p	0.849886	0.849886	#N/A
MF_GO:0004816_asparagine-tRNA+ligase+activity	0.704386	0.758498	0.52308
BP_GO:0022405_hair+cycle+process	0.1998	0.209386	0.351296
BP_GO:0048496_maintenance+of+organ+identity	0.569758	0.569758	#N/A
MF_GO:0003913_DNA+photolyase+activity	0.844017	0.844017	#N/A
MF_GO:0003836_beta-galactoside+alpha-2,3-sialyltransferase+activity	0.721313	0.426627	0.844843
BP_GO:0045737_positive+regulation+of+cyclin-dependent+protein+kinase+a	0.564109	0.820672	0.245237
BP_GO:0021937_Purkinje+cell-granule+cell+precursor+cell+signaling+involv	0.916331	0.916331	#N/A
MF_GO:0003863_3-methyl-2-oxobutanoate+dehydrogenase+(2-methylprop	0.0225379	0.0225379	#N/A
MF_GO:0033691_sialic+acid+binding	0.246021	0.666803	0.0804572
BP_GO:0000244_assembly+of+spliceosomal+tri-snRNP	0.139953	0.139953	#N/A
MF_GO:0015220_choline+transmembrane+transporter+activity	0.973637	#N/A	0.973637
MF_GO:0005146_leukemia+inhibitory+factor+receptor+binding	0.802134	0.802134	#N/A
BP_GO:0030917_midbrain-hindbrain+boundary+development	0.33996	0.33996	#N/A
MF_GO:0008332_low+voltage-gated+calcium+channel+activity	0.794836	0.247191	0.967651
BP_GO:0021526_medial+motor+column+neuron+differentiation	0.737122	#N/A	0.737122
BP_GO:0010155_regulation+of+proton+transport	0.938249	0.938249	#N/A
BP_GO:0045851_pH+reduction	0.938249	0.938249	#N/A
MF_GO:0008120_ceramide+glucosyltransferase+activity	0.865938	#N/A	0.865938
MF_GO:0004045_aminoacyl-tRNA+hydrolase+activity	0.911103	0.250817	0.995016
BP_GO:0032320_positive+regulation+of+Ras+GTPase+activity	0.632286	0.7873	0.374784
BP_GO:0048570_notochord+morphogenesis	0.0323887	0.0305007	0.230664
BP_GO:0051348_negative+regulation+of+transferase+activity	0.590126	0.590126	#N/A
BP_GO:0051051_negative+regulation+of+transport	0.505689	0.505689	#N/A
BP_GO:0006903_vesicle+targeting	0.086207	0.086207	#N/A
BP_GO:0043001_Golgi+to+plasma+membrane+protein+transport	0.190609	0.195353	0.352295
BP_GO:0032100_positive+regulation+of+appetite	0.685532	0.869042	0.330475
CC_GO:0044459_plasma+membrane+part	0.919144	0.880164	0.788829
BP_GO:0035110_leg+morphogenesis	0.579554	0.643403	0.466701
BP_GO:0006054_N-acetylneuraminate+metabolic+process	0.983328	#N/A	0.983328
MF_GO:0016176_superoxide-generating+NADPH+oxidase+activator+activity	0.810392	#N/A	0.810392
MF_GO:0016520_growth+hormone-releasing+hormone+receptor+activity	0.371111	#N/A	0.371111
BP_GO:0043517_positive+regulation+of+DNA+damage+response,+signal+tra	0.397978	0.8479	0.0818516
BP_GO:0042346_positive+regulation+of+NF-kappaB+import+into+nucleus	0.764936	0.633399	0.751838
BP_GO:0050954_sensory+perception+of+mechanical+stimulus	0.712502	#N/A	0.712502
CC_GO:0030849_autosome	0.529877	0.529877	#N/A
BP_GO:0006499_N-terminal+protein+myristoylation	0.976843	0.865946	0.956292
BP_GO:0018008_N-terminal+peptidyl-glycine+N-myristoylation	0.976843	0.865946	0.956292

MF_GO:0004379_glycylpeptide+N-tetradecanoyltransferase+activity	0.976843	0.865946	0.956292
BP_GO:0009217_purine+deoxyribonucleoside+triphosphate+catabolic+proc	0.986152	0.986152	#N/A
MF_GO:0004169_dolichyl-phosphate-mannose-protein+mannosyltransferas	0.358546	0.358546	#N/A
BP_GO:0006477_protein+amino+acid+sulfation	0.535442	0.535442	#N/A
BP_GO:0032324_molybdopterin+cofactor+biosynthetic+process	0.646383	0.57215	0.636552
MF_GO:0008442_3-hydroxyisobutyrate+dehydrogenase+activity	0.612658	0.612658	#N/A
MF_GO:0004321_fatty-acyl-CoA+synthase+activity	0.100413	0.100413	#N/A
BP_GO:0015840_urea+transport	0.234421	#N/A	0.234421
BP_GO:0002456_T+cell+mediated+immunity	0.653517	0.419406	0.776894
CC_GO:0017101_aminoacyl-tRNA+synthetase+multienzyme+complex	0.703501	0.703501	#N/A
BP_GO:0050973_detection+of+mechanical+stimulus+involved+in+equilibrio	0.621793	#N/A	0.621793
BP_GO:0043030_regulation+of+macrophage+activation	0.591335	#N/A	0.591335
BP_GO:0006424_glutamyl-tRNA+aminoacylation	0.804056	0.645799	0.798551
MF_GO:0004818_glutamate-tRNA+ligase+activity	0.804056	0.645799	0.798551
BP_GO:0006890_retrograde+vesicle-mediated+transport,+Golgi+to+ER	0.0701018	0.0701018	#N/A
MF_GO:0004949_cannabinoid+receptor+activity	0.339337	#N/A	0.339337
MF_GO:0003880_C-terminal+protein+carboxyl+methyltransferase+activity	0.0365774	0.0365774	#N/A
BP_GO:0033631_cell-cell+adhesion+mediated+by+integrin	0.877953	0.877953	#N/A
BP_GO:0045077_negative+regulation+of+interferon-gamma+biosynthetic+p	0.324369	0.419406	0.329897
BP_GO:0021882_regulation+of+transcription+from+RNA+polymerase+II+prc	0.776089	0.232774	0.964297
BP_GO:0021893_cerebral+cortex+GABAergic+interneuron+fate+commitmer	0.776089	0.232774	0.964297
MF_GO:0004844_uracil+DNA+N-glycosylase+activity	0.586998	0.586998	#N/A
CC_GO:0000172_ribonuclease+MRP+complex	0.0505241	0.00425356	0.623122
MF_GO:0030971_receptor+tyrosine+kinase+binding	0.739781	0.739781	#N/A
BP_GO:0010225_response+to+UV-C	0.307549	0.307549	#N/A
BP_GO:0045814_negative+regulation+of+gene+expression,+epigenetic	0.731331	0.731331	#N/A
BP_GO:0008078_mesodermal+cell+migration	0.526652	0.797128	0.230664
BP_GO:0021533_cell+differentiation+in+hindbrain	0.262527	0.0612756	0.74087
BP_GO:0006437_tyrosyl-tRNA+aminoacylation	0.701551	0.701551	#N/A
MF_GO:0004831_tyrosine-tRNA+ligase+activity	0.701551	0.701551	#N/A
BP_GO:0060163_subpallium+neuron+fate+commitment	0.891068	0.987914	0.304183
BP_GO:0006438_valyl-tRNA+aminoacylation	0.711343	0.577676	0.723088
MF_GO:0004832_valine-tRNA+ligase+activity	0.711343	0.577676	0.723088
MF_GO:0004105_choline-phosphate+cytidylyltransferase+activity	0.636488	0.24871	0.879447
BP_GO:0035315_hair+cell+differentiation	0.147178	0.209386	0.25035
MF_GO:0001641_group+II+metabotropic+glutamate+receptor+activity	0.328176	0.168246	0.630218
CC_GO:0005666_DNA-directed+RNA+polymerase+III+complex	0.0345481	0.0345481	#N/A
BP_GO:0045132_meiotic+chromosome+segregation	0.915153	0.915153	#N/A
BP_GO:0043320_natural+killer+cell+degranulation	0.828541	0.846436	0.625341
BP_GO:0002016_regulation+of+blood+volume+by+renin-angiotensin	0.624849	0.814936	0.32774
MF_GO:0008459_chondroitin+6-sulfotransferase+activity	0.477929	0.303862	0.66834
MF_GO:0034482_chondroitin+2-O-sulfotransferase+activity	0.477929	0.303862	0.66834
MF_GO:0016499_orexin+receptor+activity	0.0441466	#N/A	0.0441466
BP_GO:0001560_regulation+of+cell+growth+by+extracellular+stimulus	0.869382	0.784643	0.788225
MF_GO:0002046_opsin+binding	0.50706	#N/A	0.50706
MF_GO:0008159_positive+transcription+elongation+factor+activity	0.131368	0.233866	0.195834
BP_GO:0009107_lipoate+biosynthetic+process	0.344675	0.0690964	0.820634

BP_GO:0048172_regulation+of+short-term+neuronal+synaptic+plasticity	0.39108	0.323776	0.526541
CC_GO:0008328_ionotropic+glutamate+receptor+complex	0.39108	0.323776	0.526541
MF_GO:0017056_structural+constituent+of+nuclear+pore	0.140023	0.140023	#N/A
BP_GO:0051546_keratinocyte+migration	0.570448	0.794265	0.284287
BP_GO:0042516_regulation+of+tyrosine+phosphorylation+of+Stat3+protein	0.169211	0.0788751	0.523852
BP_GO:0008298_intracellular+mRNA+localization	0.211452	0.383875	0.201244
MF_GO:0004658_propionyl-CoA+carboxylase+activity	0.368316	0.368316	#N/A
MF_GO:0001665_alpha-N-acetylgalactosaminide+alpha-2,6-sialyltransferase	0.0454754	0.0311207	0.299957
MF_GO:0004351_glutamate+decarboxylase+activity	0.130261	#N/A	0.130261
BP_GO:0002204_somatic+recombination+of+immunoglobulin+genes+during	0.537758	0.381584	0.668417
BP_GO:0021562_vestibulocochlear+nerve+development	0.408828	0.408828	#N/A
BP_GO:0001306_age-dependent+response+to+oxidative+stress	0.592193	0.592193	#N/A
CC_GO:0031527_filopodium+membrane	0.946422	#N/A	0.946422
CC_GO:0032433_filopodium+tip	0.946422	#N/A	0.946422
BP_GO:0007343_egg+activation	0.713642	0.713642	#N/A
BP_GO:0021902_commitment+of+a+neuronal+cell+to+a+specific+type+of+r	0.044341	0.0557691	0.207615
BP_GO:0021905_forebrain-midbrain+boundary+formation	0.044341	0.0557691	0.207615
BP_GO:0042327_positive+regulation+of+phosphorylation	0.0967073	#N/A	0.0967073
MF_GO:0004485_methylcrotonoyl-CoA+carboxylase+activity	0.854757	0.854757	#N/A
BP_GO:0021557_oculomotor+nerve+development	0.354433	0.354433	#N/A
BP_GO:0021558_trochlear+nerve+development	0.354433	0.354433	#N/A
BP_GO:0006379_mRNA+cleavage	0.362242	0.208494	0.623122
MF_GO:0004809_tRNA+(guanine-N2-)-methyltransferase+activity	0.235128	0.0658049	0.687017
BP_GO:0045116_protein+neddylation	0.2565	0.249765	0.40161
BP_GO:0030046_parallel+actin+filament+bundle+formation	0.137256	0.237932	0.202893
BP_GO:0009583_detection+of+light+stimulus	0.527585	0.527585	#N/A
BP_GO:0022402_cell+cycle+process	0.764809	0.670298	0.718988
MF_GO:0004152_dihydroorotate+dehydrogenase+activity	0.250426	0.151698	0.531064
MF_GO:0004158_dihydroorotate+oxidase+activity	0.250426	0.151698	0.531064
BP_GO:0048808_male+genitalia+morphogenesis	0.843645	0.975414	0.294643
BP_GO:0030162_regulation+of+proteolysis	0.785885	#N/A	0.785885
BP_GO:0002768_immune+response-regulating+cell+surface+receptor+signa	0.644406	#N/A	0.644406
MF_GO:0008599_protein+phosphatase+type+1+regulator+activity	0.647204	0.647204	#N/A
BP_GO:0009624_response+to+nematode	0.996325	0.951455	0.983412
BP_GO:0031282_regulation+of+guanylate+cyclase+activity	0.768079	0.768079	#N/A
MF_GO:0008048_calcium+sensitive+guanylate+cyclase+activator+activity	0.768079	0.768079	#N/A
MF_GO:0030249_guanylate+cyclase+regulator+activity	0.768079	0.768079	#N/A
BP_GO:0042503_tyrosine+phosphorylation+of+Stat3+protein	0.908047	0.639648	0.935869
BP_GO:0045835_negative+regulation+of+meiosis	0.908047	0.639648	0.935869
BP_GO:0006428_isoleucyl-tRNA+aminoacylation	0.9919	#N/A	0.9919
MF_GO:0004822_isoleucine-tRNA+ligase+activity	0.9919	#N/A	0.9919
BP_GO:0010551_regulation+of+specific+transcription+from+RNA+polymera	0.524796	0.62342	0.410476
BP_GO:0043179_rhythmic+excitation	0.91395	0.988142	0.370184
BP_GO:0050881_musculoskeletal+movement	0.778842	0.778842	#N/A
MF_GO:0003912_DNA+nucleotidylexotransferase+activity	0.627521	0.673681	0.503965
BP_GO:0060012_synaptic+transmission,+glycinergic	0.575138	0.548342	0.558269
BP_GO:0000578_embryonic+axis+specification	0.491479	#N/A	0.491479

CC_GO:0043514_interleukin-12+complex	0.146491	0.295295	0.171518
MF_GO:0033883_pyridoxal+phosphatase+activity	0.147314	0.513376	0.064931
BP_GO:0002223_stimulatory+C-type+lectin+receptor+signaling+pathway	0.701628	0.663948	0.627348
BP_GO:0031573_intra-S+DNA+damage+checkpoint	0.659633	0.558154	0.668417
BP_GO:0032735_positive+regulation+of+interleukin-12+production	0.964423	#N/A	0.964423
BP_GO:0006427_histidyl-tRNA+aminoacylation	0.606278	0.598222	0.552751
MF_GO:0004821_histidine-tRNA+ligase+activity	0.606278	0.598222	0.552751
BP_GO:0001711_endodermal+cell+fate+commitment	0.090703	0.090703	#N/A
BP_GO:0032714_negative+regulation+of+interleukin-5+production	0.462537	0.419406	0.528194
BP_GO:0006558_L-phenylalanine+metabolic+process	0.422037	0.422037	#N/A
BP_GO:0019889_pteridine+metabolic+process	0.422037	0.422037	#N/A
BP_GO:0046146_tetrahydrobiopterin+metabolic+process	0.422037	0.422037	#N/A
MF_GO:0004757_sepiapterin+reductase+activity	0.422037	0.422037	#N/A
BP_GO:0046168_glycerol-3-phosphate+catabolic+process	0.246991	0.246991	#N/A
MF_GO:0004367_glycerol-3-phosphate+dehydrogenase+(NAD+)+activity	0.246991	0.246991	#N/A
BP_GO:0006436_tryptophanyl-tRNA+aminoacylation	0.128969	0.641343	0.0249488
MF_GO:0004830_tryptophan-tRNA+ligase+activity	0.128969	0.641343	0.0249488
MF_GO:0005326_neurotransmitter+transporter+activity	0.699838	0.782255	0.484438
MF_GO:0005118_sevenless+binding	0.299975	0.476547	0.247536
BP_GO:0010720_positive+regulation+of+cell+development	0.724682	0.54443	0.767981
BP_GO:0046514_ceramide+catabolic+process	0.17135	#N/A	0.17135
BP_GO:0001544_initiation+of+primordial+ovarian+follicle+growth	0.999161	#N/A	0.999161
BP_GO:0001547_antral+ovarian+follicle+growth	0.999161	#N/A	0.999161
BP_GO:0007403_glial+cell+fate+determination	0.699915	#N/A	0.699915
MF_GO:0047783_corticosterone+18-monooxygenase+activity	0.876001	0.34284	0.979217
MF_GO:0017134_fibroblast+growth+factor+binding	0.0294536	0.0768194	0.106962
BP_GO:0042255_ribosome+assembly	0.751751	#N/A	0.751751
MF_GO:0004596_peptide+alpha-N-acetyltransferase+activity	0.0587352	0.0277565	0.382973
BP_GO:0000272_polysaccharide+catabolic+process	0.584086	0.554856	0.564531
BP_GO:0006030_chitin+metabolic+process	0.584086	0.554856	0.564531
BP_GO:0045630_positive+regulation+of+T-helper+2+cell+differentiation	0.430143	0.679109	0.237691
BP_GO:0033058_directional+locomotion	0.768878	0.986064	0.123081
MF_GO:0001632_leukotriene+B4+receptor+activity	0.561613	#N/A	0.561613
BP_GO:0006423_cysteinyl-tRNA+aminoacylation	0.565482	0.257017	0.812157
MF_GO:0004817_cysteine-tRNA+ligase+activity	0.565482	0.257017	0.812157
BP_GO:0055075_potassium+ion+homeostasis	0.333672	#N/A	0.333672
BP_GO:0046960_sensitization	0.597783	#N/A	0.597783
BP_GO:0060292_long+term+synaptic+depression	0.597783	#N/A	0.597783
MF_GO:0001588_dopamine+receptor+coupled+via+Gs	0.597783	#N/A	0.597783
BP_GO:0001573_ganglioside+metabolic+process	0.402036	0.596921	0.275655
BP_GO:0021536_diencephalon+development	0.25993	0.25993	#N/A
BP_GO:0006638_neutral+lipid+metabolic+process	0.561217	#N/A	0.561217
BP_GO:0045741_positive+regulation+of+epidermal+growth+factor+recepto	0.0388382	#N/A	0.0388382
BP_GO:0002507_tolerance+induction	0.222089	0.222089	#N/A
CC_GO:0005914_spot+adherens+junction	0.644648	#N/A	0.644648
BP_GO:0043567_regulation+of+insulin-like+growth+factor+receptor+signali	0.621462	#N/A	0.621462
BP_GO:0060133_somatotropin+secreting+cell+development	0.621462	#N/A	0.621462

BP_GO:0042404_thyroid+hormone+catabolic+process	0.200167	0.725943	0.0367984
MF_GO:0001639_PLC+activating+metabotropic+glutamate+receptor+activit	0.260093	#N/A	0.260093
MF_GO:0005315_inorganic+phosphate+transmembrane+transporter+activit	0.343882	0.343882	#N/A
BP_GO:0050708_regulation+of+protein+secretion	0.973458	0.748272	0.980547
BP_GO:0046425_regulation+of+JAK-STAT+cascade	0.968086	#N/A	0.968086
BP_GO:0043558_regulation+of+translational+initiation+in+response+to+stre	0.476984	0.405162	0.563032
BP_GO:0032695_negative+regulation+of+interleukin-12+production	0.955312	#N/A	0.955312
MF_GO:0015276_ligand-gated+ion+channel+activity	0.468392	#N/A	0.468392
CC_GO:0032389_MutLalpha+complex	0.345268	0.381584	0.396978
MF_GO:0005131_growth+hormone+receptor+binding	0.998894	0.988244	0.980331
BP_GO:0045608_negative+regulation+of+auditory+receptor+cell+differentia	0.758543	0.60782	0.763722
MF_GO:0001716_L-amino-acid+oxidase+activity	0.709547	0.709547	#N/A
BP_GO:0021568_rhombomere+2+development	0.700064	0.700064	#N/A
CC_GO:0033270_paranode+region+of+axon	0.740901	#N/A	0.740901
BP_GO:0046427_positive+regulation+of+JAK-STAT+cascade	0.956881	#N/A	0.956881
MF_GO:0032183_SUMO+binding	0.000300376	#N/A	0.000300376
BP_GO:0050847_progesterone+receptor+signaling+pathway	0.31534	0.0226003	0.907161
BP_GO:0017145_stem+cell+division	0.46521	#N/A	0.46521
BP_GO:0007567_parturition	0.32174	#N/A	0.32174
MF_GO:0008743_L-threonine+3-dehydrogenase+activity	0.91768	0.747032	0.903076
MF_GO:0005432_calcium:sodium+antiporter+activity	0.090362	#N/A	0.090362
MF_GO:0004948_calcitonin+receptor+activity	0.748036	0.638673	0.7224
MF_GO:0016428_tRNA+(cytosine-5-)-methyltransferase+activity	0.391843	0.391843	#N/A
BP_GO:0045408_regulation+of+interleukin-6+biosynthetic+process	0.48696	0.23402	0.751655
CC_GO:0000214_tRNA-intron+endonuclease+complex	0.0871135	0.0109889	0.644475
MF_GO:0000213_tRNA-intron+endonuclease+activity	0.0871135	0.0109889	0.644475
CC_GO:0030880_RNA+polymerase+complex	0.000745783	0.000745783	#N/A
BP_GO:0043516_regulation+of+DNA+damage+response,+signal+transductio	0.985518	#N/A	0.985518
MF_GO:0004647_phosphoserine+phosphatase+activity	0.935052	0.535449	0.979915
MF_GO:0004366_glycerol-3-phosphate+O-acyltransferase+activity	0.340634	0.340634	#N/A
MF_GO:0005427_proton-dependent+oligopeptide+secondary+active+transr	0.533201	0.624711	0.420794
BP_GO:0021965_spinal+cord+ventral+commissure+morphogenesis	0.483096	0.678157	0.30076
MF_GO:0008486_diphosphoinositol-polyphosphate+diphosphatase+activity	0.383585	#N/A	0.383585
BP_GO:0051297_centrosome+organization	0.106004	0.106004	#N/A
CC_GO:0000930_gamma-tubulin+complex	0.106004	0.106004	#N/A
BP_GO:0006189_'de+novo'+IMP+biosynthetic+process	0.998141	0.999215	0.826291
BP_GO:0007418_ventral+midline+development	0.424767	#N/A	0.424767
BP_GO:0060032_notochord+regression	0.424767	#N/A	0.424767
BP_GO:0060259_regulation+of+feeding+behavior	0.873007	#N/A	0.873007
MF_GO:0001594_trace-amine+receptor+activity	0.953894	0.872045	0.893287
BP_GO:0050432_catecholamine+secretion	0.224712	0.224712	#N/A
BP_GO:0060126_somatotropin+secreting+cell+differentiation	0.536933	0.369855	0.678506
BP_GO:0006434_seryl-tRNA+aminoacylation	0.469735	0.469735	#N/A
MF_GO:0004828_serine-tRNA+ligase+activity	0.469735	0.469735	#N/A
BP_GO:0050916_sensory+perception+of+sweet+taste	0.728842	#N/A	0.728842
MF_GO:0003730_mRNA+3'-UTR+binding	0.483587	0.415726	0.561556
BP_GO:0048343_paraxial+mesodermal+cell+fate+commitment	0.150147	0.157116	0.323673

MF_GO:0004446_multiple+inositol-polyphosphate+phosphatase+activity	0.961535	0.961535	#N/A
MF_GO:0000702_oxidized+base+lesion+DNA+N-glycosylase+activity	0.376385	0.356091	0.469687
MF_GO:0005148_prolactin+receptor+binding	0.937081	#N/A	0.937081
BP_GO:0002443_leukocyte+mediated+immunity	0.24649	#N/A	0.24649
MF_GO:0001540_beta-amyloid+binding	0.323494	0.306521	0.443809
BP_GO:0022605_oogenesis+stage	0.573698	#N/A	0.573698
BP_GO:0045744_negative+regulation+of+G-protein+coupled+receptor+prot	0.856187	0.903284	0.58034
BP_GO:0001781_neutrophil+apoptosis	0.194764	#N/A	0.194764
MF_GO:0008494_translation+activator+activity	0.213174	0.213174	#N/A
CC_GO:0000779_condensed+chromosome,+centromeric+region	0.974958	0.894915	0.935304
CC_GO:0031618_nuclear+centromeric+heterochromatin	0.974958	0.894915	0.935304
BP_GO:0007549_dosage+compensation	0.752823	0.752823	#N/A
BP_GO:0007529_establishment+of+synaptic+specificity+at+neuromuscular+	0.958058	0.958058	#N/A
MF_GO:0004910_interleukin-1,+Type+II,+blocking+receptor+activity	0.0740335	0.357852	0.0464797
MF_GO:0001635_calcitonin+gene-related+polypeptide+receptor+activity	0.224074	0.224074	#N/A
BP_GO:0030327_prenylated+protein+catabolic+process	0.3654	0.3654	#N/A
BP_GO:0045746_negative+regulation+of+Notch+signaling+pathway	0.776423	0.776423	#N/A
MF_GO:0001671_ATPase+activator+activity	0.155057	0.155057	#N/A
MF_GO:0030189_chaperone+activator+activity	0.155057	0.155057	#N/A
BP_GO:0032011_ARF+protein+signal+transduction	0.649898	0.641016	0.572727
MF_GO:0047066_phospholipid-hydroperoxide+glutathione+peroxidase+acti	0.631536	0.631536	#N/A
CC_GO:0005787_signal+peptidase+complex	0.826716	0.125394	0.993415
BP_GO:0000432_positive+regulation+of+transcription+from+RNA+polymera	0.371012	0.371012	#N/A
MF_GO:0004813_alanine-tRNA+ligase+activity	0.68534	0.68534	#N/A
MF_GO:0030569_chymotrypsin+inhibitor+activity	0.40163	#N/A	0.40163
BP_GO:0010519_negative+regulation+of+phospholipase+activity	0.0349175	#N/A	0.0349175
MF_GO:0004454_ketohexokinase+activity	0.00334619	0.00334619	#N/A
CC_GO:0033018_sarcoplasmic+reticulum+lumen	0.397361	0.397361	#N/A
BP_GO:0010389_regulation+of+G2/M+transition+of+mitotic+cell+cycle	0.140979	#N/A	0.140979
MF_GO:0017174_glycine+N-methyltransferase+activity	0.980729	0.980729	#N/A
BP_GO:0008292_acetylcholine+biosynthetic+process	0.32979	#N/A	0.32979
MF_GO:0005307_choline:sodium+symporter+activity	0.32979	#N/A	0.32979
BP_GO:0046952_ketone+body+catabolic+process	0.090362	0.090362	#N/A
MF_GO:0008410_CoA-transferase+activity	0.090362	0.090362	#N/A
MF_GO:0008127_quercetin+2,3-dioxygenase+activity	0.148543	#N/A	0.148543
BP_GO:0046314_phosphocreatine+biosynthetic+process	0.127624	0.127624	#N/A
MF_GO:0003845_11-beta-hydroxysteroid+dehydrogenase+activity	0.296654	#N/A	0.296654
MF_GO:0000334_3-hydroxyanthranilate+3,4-dioxygenase+activity	0.603289	#N/A	0.603289
MF_GO:0004939_beta-adrenergic+receptor+activity	0.438301	0.438301	#N/A
MF_GO:0031699_beta-3+adrenergic+receptor+binding	0.438301	0.438301	#N/A
MF_GO:0004846_urate+oxidase+activity	0.0673314	0.0673314	#N/A
BP_GO:0006741_NADP+biosynthetic+process	0.0117782	#N/A	0.0117782
MF_GO:0051370_ZASP+binding	0.277955	0.277955	#N/A
BP_GO:0034370_triglyceride-rich+lipoprotein+particle+remodeling	0.512178	#N/A	0.512178
BP_GO:0060332_positive+regulation+of+response+to+interferon-gamma	0.333219	0.333219	#N/A
BP_GO:0060335_positive+regulation+of+interferon-gamma-mediated+signa	0.333219	0.333219	#N/A
MF_GO:0030429_kynureninase+activity	0.667695	#N/A	0.667695

BP_GO:0030832_regulation+of+actin+filament+length	0.88049	#N/A	0.88049
BP_GO:0015939_pantothenate+metabolic+process	0.429023	#N/A	0.429023
BP_GO:0006084_acetyl-CoA+metabolic+process	0.994531	0.994531	#N/A
MF_GO:0004996_thyroid-stimulating+hormone+receptor+activity	0.358544	#N/A	0.358544
BP_GO:0045721_negative+regulation+of+gluconeogenesis	0.0315437	#N/A	0.0315437
BP_GO:0045923_positive+regulation+of+fatty+acid+metabolic+process	0.0315437	#N/A	0.0315437
MF_GO:0004055_argininosuccinate+synthase+activity	0.902381	0.902381	#N/A
BP_GO:0042360_vitamin+E+metabolic+process	0.726022	0.726022	#N/A
MF_GO:0008431_vitamin+E+binding	0.726022	0.726022	#N/A
MF_GO:0003983_UTP:glucose-1-phosphate+uridylyltransferase+activity	0.407891	#N/A	0.407891
BP_GO:0032570_response+to+progesterone+stimulus	0.073383	0.073383	#N/A
BP_GO:0033484_nitric+oxide+homeostasis	0.073383	0.073383	#N/A
BP_GO:0042524_negative+regulation+of+tyrosine+phosphorylation+of+Stat	0.073383	0.073383	#N/A
BP_GO:0052547_regulation+of+peptidase+activity	0.073383	0.073383	#N/A
BP_GO:0051918_negative+regulation+of+fibrinolysis	0.611328	#N/A	0.611328
MF_GO:0051213_dioxygenase+activity	0.00861585	#N/A	0.00861585
MF_GO:0047130_saccharopine+dehydrogenase+(NADP+,+L-lysine-forming)+	0.354513	#N/A	0.354513
BP_GO:0042694_muscle+cell+fate+specification	0.0561516	0.0561516	#N/A
BP_GO:0001775_cell+activation	0.300378	#N/A	0.300378
BP_GO:0001959_regulation+of+cytokine+mediated+signaling+pathway	0.300378	#N/A	0.300378
BP_GO:0002523_leukocyte+migration+during+inflammatory+response	0.300378	#N/A	0.300378
BP_GO:0002842_positive+regulation+of+T+cell+mediated+immune+respons	0.300378	#N/A	0.300378
BP_GO:0032600_chemokine+receptor+transport+out+of+membrane+raft	0.300378	#N/A	0.300378
BP_GO:0032913_negative+regulation+of+transforming+growth+factor-beta3	0.300378	#N/A	0.300378
BP_GO:0033625_positive+regulation+of+integrin+activation	0.300378	#N/A	0.300378
BP_GO:0033630_positive+regulation+of+cell+adhesion+mediated+by+integr	0.300378	#N/A	0.300378
BP_GO:0033632_regulation+of+cell-cell+adhesion+mediated+by+integrin	0.300378	#N/A	0.300378
BP_GO:0033634_positive+regulation+of+cell-cell+adhesion+mediated+by+ir	0.300378	#N/A	0.300378
BP_GO:0034107_negative+regulation+of+erythrocyte+clearance	0.300378	#N/A	0.300378
BP_GO:0034119_negative+regulation+of+erythrocyte+aggregation	0.300378	#N/A	0.300378
BP_GO:0042103_positive+regulation+of+T+cell+homeostatic+proliferation	0.300378	#N/A	0.300378
BP_GO:0046014_negative+regulation+of+T+cell+homeostatic+proliferation	0.300378	#N/A	0.300378
CC_GO:0060170_cilium+membrane	0.300378	#N/A	0.300378
MF_GO:0016410_N-acyltransferase+activity	0.262274	#N/A	0.262274
MF_GO:0047963_glycine+N-choloyltransferase+activity	0.262274	#N/A	0.262274
BP_GO:0006067_ethanol+metabolic+process	0.681769	#N/A	0.681769
BP_GO:0042375_quinone+cofactor+metabolic+process	0.681769	#N/A	0.681769
BP_GO:0046164_alcohol+catabolic+process	0.681769	#N/A	0.681769
MF_GO:0004024_alcohol+dehydrogenase+activity,+zinc-dependent	0.681769	#N/A	0.681769
BP_GO:0051347_positive+regulation+of+transferase+activity	0.0810297	0.0810297	#N/A
BP_GO:0007284_spermatogonial+cell+division	0.61643	#N/A	0.61643
BP_GO:0034633_retinol+transport	0.61643	#N/A	0.61643
BP_GO:0060059_embryonic+retina+morphogenesis+in+camera-type+eye	0.61643	#N/A	0.61643
BP_GO:0060065_uterus+development	0.61643	#N/A	0.61643
MF_GO:0034632_retinol+transporter+activity	0.61643	#N/A	0.61643
MF_GO:0008747_N-acetylneuraminate+lyase+activity	0.0027369	#N/A	0.0027369
BP_GO:0006116_NADH+oxidation	0.0648304	0.0648304	#N/A

MF_GO:0030395_lactose+binding	0.742037	#N/A	0.742037
MF_GO:0008443_phosphofructokinase+activity	0.652143	0.652143	#N/A
BP_GO:0043501_skeletal+muscle+adaptation	0.270459	0.270459	#N/A
BP_GO:0055001_muscle+cell+development	0.270459	0.270459	#N/A
CC_GO:0009348_ornithine+carbamoyltransferase+complex	0.866703	0.866703	#N/A
MF_GO:0004585_ornithine+carbamoyltransferase+activity	0.866703	0.866703	#N/A
MF_GO:0016743_carboxyl-+or+carbamoyltransferase+activity	0.866703	0.866703	#N/A
MF_GO:0017129_triglyceride+binding	0.222769	0.222769	#N/A
BP_GO:0030241_muscle+thick+filament+assembly	0.0445389	#N/A	0.0445389
MF_GO:0047568_3-oxo-5-beta-steroid+4-dehydrogenase+activity	0.379121	#N/A	0.379121
MF_GO:0047787_delta4-3-oxosteroid+5beta-reductase+activity	0.379121	#N/A	0.379121
MF_GO:0033778_7alpha-hydroxycholest-4-en-3-one+12alpha-hydroxylase+a	0.626542	#N/A	0.626542
BP_GO:0046439_L-cysteine+metabolic+process	0.316282	#N/A	0.316282
MF_GO:0017172_cysteine+dioxygenase+activity	0.316282	#N/A	0.316282
CC_GO:0016014_dystrobrevin+complex	0.693768	#N/A	0.693768
MF_GO:0017166_vinculin+binding	0.590277	0.590277	#N/A
MF_GO:0051371_muscle+alpha-actinin+binding	0.590277	0.590277	#N/A
MF_GO:0047305_(R)-3-amino-2-methylpropionate-pyruvate+transaminase+	0.364672	0.364672	#N/A
MF_GO:0008458_carnitine+O-octanoyltransferase+activity	0.682527	0.682527	#N/A
MF_GO:0008119_thiopurine+S-methyltransferase+activity	0.0147534	#N/A	0.0147534
BP_GO:0032972_regulation+of+muscle+filament+sliding+speed	0.000309064	0.000309064	#N/A
MF_GO:0008320_protein+transmembrane+transporter+activity	0.298003	0.298003	#N/A
MF_GO:0048244_phytanoyl-CoA+dioxygenase+activity	0.493548	0.493548	#N/A
MF_GO:0001512_dihydronicotinamide+riboside+quinone+reductase+activit	0.173127	#N/A	0.173127
MF_GO:0043184_vascular+endothelial+growth+factor+receptor+2+binding	0.0466833	#N/A	0.0466833
BP_GO:0003009_skeletal+muscle+contraction	0.255897	#N/A	0.255897
BP_GO:0032236_positive+regulation+of+calcium+ion+transport+via+store-o	0.255897	#N/A	0.255897
BP_GO:0002544_chronic+inflammatory+response	0.346658	0.346658	#N/A
MF_GO:0034235_GPI+anchor+binding	0.346658	0.346658	#N/A
MF_GO:0008466_glycogenin+glucosyltransferase+activity	0.189192	0.189192	#N/A
MF_GO:0004648_phosphoserine+transaminase+activity	0.972364	#N/A	0.972364
BP_GO:0050482_arachidonic+acid+secretion	0.0776861	#N/A	0.0776861
MF_GO:0004608_phosphatidylethanolamine+N-methyltransferase+activity	0.925104	0.925104	#N/A
MF_GO:0051183_vitamin+transporter+activity	0.339298	#N/A	0.339298
MF_GO:0030339_fatty-acyl-ethyl-ester+synthase+activity	0.832479	0.832479	#N/A
BP_GO:0051651_maintenance+of+location+in+cell	0.946104	#N/A	0.946104
BP_GO:0042941_D-alanine+transport	0.9099	0.9099	#N/A
BP_GO:0042942_D-serine+transport	0.9099	0.9099	#N/A
BP_GO:0031585_regulation+of+inositol-1,4,5-triphosphate+receptor+activit	0.229218	#N/A	0.229218
BP_GO:0032252_secretory+granule+localization	0.229218	#N/A	0.229218
BP_GO:0051643_ER+localization	0.229218	#N/A	0.229218
CC_GO:0042641_actomyosin	0.229218	#N/A	0.229218
MF_GO:0047105_4-trimethylammoniobutyraldehyde+dehydrogenase+activi	0.287893	#N/A	0.287893
MF_GO:0005360_insulin-responsive+hydrogen:glucose+symporter+activity	0.0194117	0.0194117	#N/A
MF_GO:0004411_homogentisate+1,2-dioxygenase+activity	0.32645	0.32645	#N/A
CC_GO:0045098_type+III+intermediate+filament	0.600618	#N/A	0.600618
BP_GO:0018931_naphthalene+metabolic+process	0.791474	#N/A	0.791474

MF_GO:0016206_catechol+O-methyltransferase+activity	0.146494	0.146494	#N/A
MF_GO:0050124_N-acylneuraminate-9-phosphatase+activity	0.0396378	#N/A	0.0396378
MF_GO:0004086_carbamoyl-phosphate+synthase+activity	0.673336	#N/A	0.673336
MF_GO:0004087_carbamoyl-phosphate+synthase+(ammonia)+activity	0.673336	#N/A	0.673336
BP_GO:0034213_quinolinate+catabolic+process	0.903456	0.903456	#N/A
MF_GO:0004514_nicotinate-nucleotide+diphosphorylase+(carboxylating)+ac	0.903456	0.903456	#N/A
BP_GO:0015759_beta-glucoside+transport	0.827388	0.827388	#N/A
BP_GO:0060192_negative+regulation+of+lipase+activity	0.827388	0.827388	#N/A
BP_GO:0030070_insulin+processing	0.224945	0.224945	#N/A
BP_GO:0007525_somatic+muscle+development	0.0607042	0.0607042	#N/A
BP_GO:0060004_reflex	0.379388	#N/A	0.379388
BP_GO:0014829_vascular+smooth+muscle+contraction	0.277675	0.277675	#N/A
BP_GO:0032836_glomerular+basement+membrane+development	0.7628	#N/A	0.7628
MF_GO:0004502_kynurenine+3-monooxygenase+activity	0.0336353	#N/A	0.0336353
MF_GO:0047747_cholate-CoA+ligase+activity	0.628056	#N/A	0.628056
BP_GO:0003065_positive+regulation+of+heart+rate+by+epinephrine	0.826794	0.826794	#N/A
BP_GO:0001766_membrane+raft+polarization	0.700678	0.700678	#N/A
BP_GO:0046631_alpha-beta+T+cell+activation	0.700678	0.700678	#N/A
BP_GO:0046633_alpha-beta+T+cell+proliferation	0.700678	0.700678	#N/A
MF_GO:0047865_dimethylglycine+dehydrogenase+activity	0.749275	0.749275	#N/A
BP_GO:0045540_regulation+of+cholesterol+biosynthetic+process	0.792677	#N/A	0.792677
BP_GO:0032098_regulation+of+appetite	0.397884	#N/A	0.397884
BP_GO:0051608_histamine+transport	0.397884	#N/A	0.397884
BP_GO:0051615_histamine+uptake	0.397884	#N/A	0.397884
BP_GO:0001993_regulation+of+systemic+arterial+blood+pressure+by+norep	0.210015	#N/A	0.210015
MF_GO:0004941_beta2-adrenergic+receptor+activity	0.210015	#N/A	0.210015
MF_GO:0008179_adenylate+cyclase+binding	0.210015	#N/A	0.210015
MF_GO:0030171_voltage-gated+proton+channel+activity	0.735738	0.735738	#N/A
BP_GO:0032148_activation+of+protein+kinase+B+activity	0.183053	#N/A	0.183053
MF_GO:0043422_protein+kinase+B+binding	0.183053	#N/A	0.183053
MF_GO:0047757_chondroitin-glucuronate+5-epimerase+activity	0.0792526	#N/A	0.0792526
BP_GO:0002446_neutrophil+mediated+immunity	0.999552	#N/A	0.999552
BP_GO:0032956_regulation+of+actin+cytoskeleton+organization	0.999552	#N/A	0.999552
BP_GO:0045088_regulation+of+innate+immune+response	0.999552	#N/A	0.999552
BP_GO:0019318_hexose+metabolic+process	0.907628	#N/A	0.907628
MF_GO:0004034_aldose+1-epimerase+activity	0.907628	#N/A	0.907628
MF_GO:0033971_hydroxyisourate+hydrolase+activity	0.954563	#N/A	0.954563
MF_GO:0003837_beta-ureidopropionase+activity	0.680901	0.680901	#N/A
BP_GO:0006060_sorbitol+metabolic+process	0.195134	#N/A	0.195134
MF_GO:0003939_L-iditol+2-dehydrogenase+activity	0.195134	#N/A	0.195134
BP_GO:0033574_response+to+testosterone+stimulus	0.472152	#N/A	0.472152
MF_GO:0004716_receptor+signaling+protein+tyrosine+kinase+activity	0.728818	0.728818	#N/A
MF_GO:0004617_phosphoglycerate+dehydrogenase+activity	0.105027	0.105027	#N/A
MF_GO:0016619_malate+dehydrogenase+(oxaloacetate-decarboxylating)+a	0.920121	0.920121	#N/A
MF_GO:0016404_15-hydroxyprostaglandin+dehydrogenase+(NAD+)+activity	0.307208	#N/A	0.307208
BP_GO:0043648_dicarboxylic+acid+metabolic+process	0.169766	#N/A	0.169766
MF_GO:0030343_vitamin+D3+25-hydroxylase+activity	0.246209	#N/A	0.246209

CC_GO:0005586_collagen+type+III	0.731717	0.731717	#N/A
BP_GO:0046340_diacylglycerol+catabolic+process	0.374158	0.374158	#N/A
MF_GO:0033878_hormone-sensitive+lipase+activity	0.374158	0.374158	#N/A
MF_GO:0004741_[pyruvate+dehydrogenase+(lipoamide)]+phosphatase+act	0.613508	0.613508	#N/A
MF_GO:0015368_calcium:cation+antiporter+activity	0.530866	0.530866	#N/A
BP_GO:0002920_regulation+of+humoral+immune+response	0.180122	0.180122	#N/A
MF_GO:0003941_L-serine+ammonia-lyase+activity	0.157115	#N/A	0.157115
MF_GO:0003885_D-arabinono-1,4-lactone+oxidase+activity	0.811098	#N/A	0.811098
MF_GO:0016899_oxidoreductase+activity,+acting+on+the+CH-OH+group+of	0.811098	#N/A	0.811098
MF_GO:0050105_L-gulonolactone+oxidase+activity	0.811098	#N/A	0.811098
BP_GO:0032224_positive+regulation+of+synaptic+transmission,+cholinergic	0.69964	0.69964	#N/A
BP_GO:0009446_putrescine+biosynthetic+process	0.527301	0.527301	#N/A
MF_GO:0008783_agmatinase+activity	0.527301	0.527301	#N/A
MF_GO:0004924_oncostatin-M+receptor+activity	0.164001	#N/A	0.164001
MF_GO:0003991_acetylglutamate+kinase+activity	0.979498	0.979498	#N/A
MF_GO:0004042_amino-acid+N-acetyltransferase+activity	0.979498	0.979498	#N/A
BP_GO:0030814_regulation+of+cAMP+metabolic+process	0.762784	#N/A	0.762784
MF_GO:0004334_fumarylacetoacetase+activity	0.499882	0.499882	#N/A
BP_GO:0015810_aspartate+transport	0.392749	#N/A	0.392749
MF_GO:0003996_acyl-CoA+ligase+activity	0.987828	#N/A	0.987828
MF_GO:0050998_nitric-oxide+synthase+binding	0.56963	#N/A	0.56963
BP_GO:0017144_drug+metabolic+process	0.10501	#N/A	0.10501
BP_GO:0060262_negative+regulation+of+N-terminal+protein+palmitoylation	0.0941098	0.0941098	#N/A
BP_GO:0045834_positive+regulation+of+lipid+metabolic+process	0.559438	0.559438	#N/A
BP_GO:0051005_negative+regulation+of+lipoprotein+lipase+activity	0.559438	0.559438	#N/A
MF_GO:0004464_leukotriene-C4+synthase+activity	0.141469	#N/A	0.141469
MF_GO:0001875_lipopolysaccharide+receptor+activity	0.316436	0.316436	#N/A
MF_GO:0004992_platelet+activating+factor+receptor+activity	0.316436	0.316436	#N/A
BP_GO:0048170_positive+regulation+of+long-term+neuronal+synaptic+plas	0.339827	#N/A	0.339827
MF_GO:0050046_lathosterol+oxidase+activity	0.914901	#N/A	0.914901
BP_GO:0021768_nucleus+accumbens+development	0.134178	#N/A	0.134178
MF_GO:0004504_peptidylglycine+monooxygenase+activity	0.914654	#N/A	0.914654
MF_GO:0004598_peptidylamidoglycolate+lyase+activity	0.914654	#N/A	0.914654
MF_GO:0004796_thromboxane-A+synthase+activity	0.323158	0.323158	#N/A
MF_GO:0004096_catalase+activity	0.856222	0.856222	#N/A
MF_GO:0016684_oxidoreductase+activity,+acting+on+peroxide+as+accepto	0.856222	0.856222	#N/A
BP_GO:0006942_regulation+of+striated+muscle+contraction	0.0307204	0.0307204	#N/A
BP_GO:0045988_negative+regulation+of+striated+muscle+contraction	0.0307204	0.0307204	#N/A
BP_GO:0051481_reduction+of+cytosolic+calcium+ion+concentration	0.0307204	0.0307204	#N/A
MF_GO:0004920_interleukin-10+receptor+activity	0.505585	#N/A	0.505585
BP_GO:0002711_positive+regulation+of+T+cell+mediated+immunity	0.557645	#N/A	0.557645
BP_GO:0002923_regulation+of+humoral+immune+response+mediated+by+	0.557645	#N/A	0.557645
BP_GO:0034113_heterotypic+cell-cell+adhesion	0.557645	#N/A	0.557645
BP_GO:0050855_regulation+of+B+cell+receptor+signaling+pathway	0.557645	#N/A	0.557645
BP_GO:0050857_positive+regulation+of+antigen+receptor-mediated+signali	0.557645	#N/A	0.557645
BP_GO:0050677_positive+regulation+of+urothelial+cell+proliferation	0.52883	#N/A	0.52883
MF_GO:0030883_endogenous+lipid+antigen+binding	0.605352	0.605352	#N/A

MF_GO:0016034_maleylacetoacetate+isomerase+activity	0.660836	#N/A	0.660836
BP_GO:0032288_myelin+formation	0.614207	0.614207	#N/A
BP_GO:0001999_renal+response+to+blood+flow+during+renin-angiotensin+	0.804886	0.804886	#N/A
BP_GO:0048143_astrocyte+activation	0.804886	0.804886	#N/A
MF_GO:0031702_type+1+angiotensin+receptor+binding	0.804886	0.804886	#N/A
MF_GO:0031703_type+2+angiotensin+receptor+binding	0.804886	0.804886	#N/A
MF_GO:0030492_hemoglobin+binding	0.736564	0.736564	#N/A
BP_GO:0006127_glycerophosphate+shuttle	0.918431	#N/A	0.918431
BP_GO:0015794_glycerol-3-phosphate+transport	0.918431	#N/A	0.918431
MF_GO:0015169_glycerol-3-phosphate+transmembrane+transporter+activit	0.918431	#N/A	0.918431
MF_GO:0004361_glutaryl-CoA+dehydrogenase+activity	0.394275	0.394275	#N/A
BP_GO:0006069_ethanol+oxidation	0.53428	0.53428	#N/A
BP_GO:0046294_formaldehyde+catabolic+process	0.53428	0.53428	#N/A
BP_GO:0051409_response+to+nitrosative+stress	0.53428	0.53428	#N/A
MF_GO:0051903_S-(hydroxymethyl)glutathione+dehydrogenase+activity	0.53428	0.53428	#N/A
BP_GO:0002246_healing+during+inflammatory+response	0.0819674	#N/A	0.0819674
CC_GO:0030690_Noc1p-Noc2p+complex	0.132946	#N/A	0.132946
CC_GO:0005588_collagen+type+V	0.607423	#N/A	0.607423
MF_GO:0015291_secondary+active+transmembrane+transporter+activity	0.700145	0.700145	#N/A
MF_GO:0047536_2-aminoadipate+transaminase+activity	0.548356	#N/A	0.548356
BP_GO:0045329_carnitine+biosynthetic+process	0.0795779	#N/A	0.0795779
MF_GO:0008336_gamma-butyrobetaine+dioxygenase+activity	0.0795779	#N/A	0.0795779
MF_GO:0004990_oxytocin+receptor+activity	0.986602	#N/A	0.986602
BP_GO:0008300_isoprenoid+catabolic+process	0.00176973	0.00176973	#N/A
MF_GO:0008111_alpha-methylacyl-CoA+racemase+activity	0.00176973	0.00176973	#N/A
BP_GO:0032344_regulation+of+aldosterone+metabolic+process	0.398968	#N/A	0.398968
BP_GO:0045794_negative+regulation+of+cell+volume	0.398968	#N/A	0.398968
BP_GO:0060073_micturition	0.398968	#N/A	0.398968
BP_GO:0060082_eye+blink+reflex	0.398968	#N/A	0.398968
MF_GO:0060072_large+conductance+calcium-activated+potassium+channe	0.398968	#N/A	0.398968
MF_GO:0004520_endodeoxyribonuclease+activity	0.95082	#N/A	0.95082
MF_GO:0030228_lipoprotein+receptor+activity	0.80349	0.80349	#N/A
BP_GO:0016480_negative+regulation+of+transcription+from+RNA+polymera	0.723845	0.723845	#N/A
BP_GO:0030195_negative+regulation+of+blood+coagulation	0.378241	0.378241	#N/A
BP_GO:0032367_intracellular+cholesterol+transport	0.151608	0.151608	#N/A
MF_GO:0004123_cystathionine+gamma-lyase+activity	0.175884	0.175884	#N/A
BP_GO:0031639_plasminogen+activation	0.636238	#N/A	0.636238
MF_GO:0016628_oxidoreductase+activity,+acting+on+the+CH-CH+group+of	0.636238	#N/A	0.636238
BP_GO:0000147_actin+cortical+patch+assembly	0.198996	#N/A	0.198996
BP_GO:0007499_ectoderm+and+mesoderm+interaction	0.00445032	#N/A	0.00445032
BP_GO:0030859_polarized+epithelial+cell+differentiation	0.00445032	#N/A	0.00445032
BP_GO:0060197_cloacal+septation	0.00445032	#N/A	0.00445032
BP_GO:0006696_ergosterol+biosynthetic+process	0.339226	#N/A	0.339226
MF_GO:0030409_glutamate+formimidoyltransferase+activity	0.60562	#N/A	0.60562
MF_GO:0030412_formimidoyltetrahydrofolate+cyclodeaminase+activity	0.60562	#N/A	0.60562
BP_GO:0015761_mannose+transport	0.462273	0.462273	#N/A
CC_GO:0000323_lytic+vacuole	0.462273	0.462273	#N/A

MF_GO:0015578_mannose+transmembrane+transporter+activity	0.462273	0.462273	#N/A
BP_GO:0017014_protein+amino+acid+nitrosylation	0.456525	#N/A	0.456525
MF_GO:0004397_histidine+ammonia-lyase+activity	0.671426	#N/A	0.671426
MF_GO:0016211_ammonia+ligase+activity	0.671426	#N/A	0.671426
MF_GO:0033981_D-dopachrome+decarboxylase+activity	0.614158	0.614158	#N/A
MF_GO:0047323_[3-methyl-2-oxobutanoate+dehydrogenase+(acetyl-transfe	0.0300832	0.0300832	#N/A
BP_GO:0048677_axon+extension+involved+in+regeneration	0.865671	#N/A	0.865671
BP_GO:0009152_purine+ribonucleotide+biosynthetic+process	0.192862	0.192862	#N/A
MF_GO:0004018_adenylosuccinate+lyase+activity	0.192862	0.192862	#N/A
MF_GO:0031008_filamin-C+binding	0.348329	0.348329	#N/A
MF_GO:0004750_ribulose-phosphate+3-epimerase+activity	0.7054	0.7054	#N/A
BP_GO:0006106_fumarate+metabolic+process	0.26199	0.26199	#N/A
CC_GO:0045239_tricarboxylic+acid+cycle+enzyme+complex	0.26199	0.26199	#N/A
MF_GO:0004333_fumarate+hydratase+activity	0.26199	0.26199	#N/A
BP_GO:0009855_determination+of+bilateral+symmetry	0.142415	#N/A	0.142415
BP_GO:0048388_endosomal+lumen+acidification	0.748502	0.748502	#N/A
MF_GO:0033791_3alpha,7alpha,12alpha-trihydroxy-5beta-cholestanoyl-CoA	0.585965	0.585965	#N/A
BP_GO:0006543_glutamine+catabolic+process	0.709548	0.709548	#N/A
BP_GO:0050861_positive+regulation+of+B+cell+receptor+signaling+pathway	0.869442	0.869442	#N/A
BP_GO:0045908_negative+regulation+of+vasodilation	0.229697	0.229697	#N/A
MF_GO:0017020_myosin+phosphatase+regulator+activity	0.229697	0.229697	#N/A
CC_GO:0016942_insulin-like+growth+factor+binding+protein+complex	0.727781	#N/A	0.727781
MF_GO:0033783_25-hydroxycholesterol+7alpha-hydroxylase+activity	0.54184	#N/A	0.54184
MF_GO:0033867_Fas-activated+serine/threonine+kinase+activity	0.320385	0.320385	#N/A
MF_GO:0001851_complement+component+C3b+binding	0.105387	#N/A	0.105387
MF_GO:0004371_glycerone+kinase+activity	0.842122	#N/A	0.842122
MF_GO:0042171_lysophosphatidic+acid+acyltransferase+activity	0.974022	#N/A	0.974022
BP_GO:0019556_histidine+catabolic+process+to+glutamate+and+formamide	0.71548	0.71548	#N/A
MF_GO:0016812_hydrolase+activity,+acting+on+carbon-nitrogen+(but+not+	0.71548	0.71548	#N/A
MF_GO:0050480_imidazolonepropionase+activity	0.71548	0.71548	#N/A
MF_GO:0008420_CTD+phosphatase+activity	0.334457	0.334457	#N/A
BP_GO:0006208_pyrimidine+base+catabolic+process	0.531064	#N/A	0.531064
MF_GO:0017113_dihydropyrimidine+dehydrogenase+(NADP+)+activity	0.531064	#N/A	0.531064
MF_GO:0033961_cis-stilbene-oxide+hydrolase+activity	0.571812	#N/A	0.571812
BP_GO:0015976_carbon+utilization	0.00913004	0.00913004	#N/A
MF_GO:0008480_sarcosine+dehydrogenase+activity	0.726725	#N/A	0.726725
MF_GO:0016153_urocanate+hydratase+activity	0.578225	0.578225	#N/A
MF_GO:0030504_inorganic+diphosphate+transmembrane+transporter+activ	0.620603	0.620603	#N/A
MF_GO:0033735_aspartate+dehydrogenase+activity	0.58998	0.58998	#N/A
MF_GO:0004051_arachidonate+5-lipoxygenase+activity	0.253581	0.253581	#N/A
MF_GO:0004133_glycogen+debranching+enzyme+activity	0.86018	0.86018	#N/A
MF_GO:0031593_polyubiquitin+binding	0.86018	0.86018	#N/A
MF_GO:0042602_flavin+reductase+activity	0.0359424	0.0359424	#N/A
BP_GO:0019919_peptidyl-arginine+methylation,+to+asymmetrical-dimethyl	0.135652	0.135652	#N/A
BP_GO:0035247_peptidyl-arginine+omega-N-methylation	0.135652	0.135652	#N/A
MF_GO:0016275_[cytochrome+c]-arginine+N-methyltransferase+activity	0.135652	0.135652	#N/A
MF_GO:0035241_protein-arginine+omega-N+monomethyltransferase+activi	0.135652	0.135652	#N/A

MF_GO:0035242_protein-arginine+omega-N+asymmetric+methyltransfera	0.135652	0.135652	#N/A
MF_GO:0050121_N-acylglucosamine+2-epimerase+activity	0.559001	#N/A	0.559001
MF_GO:0008114_phosphogluconate+2-dehydrogenase+activity	0.994706	0.994706	#N/A
BP_GO:0009074_aromatic+amino+acid+family+catabolic+process	0.942725	0.942725	#N/A
MF_GO:0004838_tyrosine+transaminase+activity	0.942725	0.942725	#N/A
BP_GO:0031223_auditory+behavior	0.16412	#N/A	0.16412
MF_GO:0005314_high-affinity+glutamate+transmembrane+transporter+acti	0.16412	#N/A	0.16412
MF_GO:0016595_glutamate+binding	0.16412	#N/A	0.16412
BP_GO:0046654_tetrahydrofolate+biosynthetic+process	0.131558	0.131558	#N/A
MF_GO:0003934_GTP+cyclohydrolase+I+activity	0.131558	0.131558	#N/A
BP_GO:0006148_inosine+catabolic+process	0.19387	#N/A	0.19387
BP_GO:0006149_deoxyinosine+catabolic+process	0.19387	#N/A	0.19387
BP_GO:0006161_deoxyguanosine+catabolic+process	0.19387	#N/A	0.19387
BP_GO:0034418_urate+biosynthetic+process	0.19387	#N/A	0.19387
BP_GO:0042278_purine+nucleoside+metabolic+process	0.19387	#N/A	0.19387
BP_GO:0046115_guanosine+catabolic+process	0.19387	#N/A	0.19387
MF_GO:0004731_purine-nucleoside+phosphorylase+activity	0.19387	#N/A	0.19387
BP_GO:0042167_heme+catabolic+process	0.974028	#N/A	0.974028
CC_GO:0005715_late+recombination+nodule	0.892568	#N/A	0.892568
BP_GO:0032196_transposition	0.936188	0.936188	#N/A
BP_GO:0030853_negative+regulation+of+granulocyte+differentiation	0.269018	#N/A	0.269018
BP_GO:0045656_negative+regulation+of+monocyte+differentiation	0.269018	#N/A	0.269018
BP_GO:0045659_negative+regulation+of+neutrophil+differentiation	0.269018	#N/A	0.269018
BP_GO:0045844_positive+regulation+of+striated+muscle+development	0.238632	#N/A	0.238632
BP_GO:0000705_achiasmate+meiosis+I	0.837868	#N/A	0.837868
BP_GO:0051309_female+meiosis+chromosome+separation	0.837868	#N/A	0.837868
BP_GO:0051353_positive+regulation+of+oxidoreductase+activity	0.862884	0.862884	#N/A
BP_GO:0060265_positive+regulation+of+respiratory+burst+during+acute+in	0.9866	#N/A	0.9866
MF_GO:0051637_Gram-positive+bacterial+cell+surface+binding	0.9866	#N/A	0.9866
MF_GO:0030267_glyoxylate+reductase+(NADP)+activity	0.328656	0.328656	#N/A
MF_GO:0047273_galactosylgalactosylglucosylceramide+beta-D-acetylgalact	0.767452	#N/A	0.767452
BP_GO:0032964_collagen+biosynthetic+process	0.994385	#N/A	0.994385
BP_GO:0051127_positive+regulation+of+actin+nucleation	0.851722	0.851722	#N/A
MF_GO:0004978_adrenocorticotropin+receptor+activity	0.648043	#N/A	0.648043
BP_GO:0042347_negative+regulation+of+NF-kappaB+import+into+nucleus	0.78254	0.78254	#N/A
CC_GO:0043293_apoptosome	0.78254	0.78254	#N/A
MF_GO:0003976_UDP-N-acetylglucosamine-lysosomal-enzyme+N-acetylglu	0.494132	#N/A	0.494132
BP_GO:0043244_regulation+of+protein+complex+disassembly	0.0787836	#N/A	0.0787836
BP_GO:0019464_glycine+decarboxylation+via+glycine+cleavage+system	0.646017	#N/A	0.646017
MF_GO:0004375_glycine+dehydrogenase+(decarboxylating)+activity	0.646017	#N/A	0.646017
BP_GO:0010107_potassium+ion+import	0.0589449	0.0589449	#N/A
BP_GO:0006646_phosphatidylethanolamine+biosynthetic+process	0.729795	0.729795	#N/A
CC_GO:0000808_origin+recognition+complex	0.0142572	#N/A	0.0142572
BP_GO:0006760_folic+acid+and+derivative+metabolic+process	0.879732	0.879732	#N/A
MF_GO:0004905_type+I+interferon+receptor+activity	0.355655	#N/A	0.355655
BP_GO:0014853_regulation+of+excitatory+postsynaptic+membrane+potent	0.337966	0.337966	#N/A
MF_GO:0004884_ecdysteroid+hormone+receptor+activity	0.113768	0.113768	#N/A

BP_GO:0001922_B-1+B+cell+homeostasis	0.414319	0.414319	#N/A
BP_GO:0010812_negative+regulation+of+cell-substrate+adhesion	0.968123	#N/A	0.968123
BP_GO:0033345_asparagine+catabolic+process+via+L-aspartate	0.0534964	0.0534964	#N/A
MF_GO:0008123_cholesterol+7-alpha-monooxygenase+activity	0.834825	#N/A	0.834825
BP_GO:0002084_protein+depalmitoylation	0.23775	#N/A	0.23775
BP_GO:0032429_regulation+of+phospholipase+A2+activity	0.23775	#N/A	0.23775
BP_GO:0042371_vitamin+K+biosynthetic+process	0.374719	#N/A	0.374719
MF_GO:0047057_vitamin-K-epoxide+reductase+(warfarin-sensitive)+activity	0.374719	#N/A	0.374719
MF_GO:0050682_AF-2+domain+binding	0.0759658	0.0759658	#N/A
BP_GO:0046415_urate+metabolic+process	0.995898	0.995898	#N/A
BP_GO:0051156_glucose+6-phosphate+metabolic+process	0.995898	0.995898	#N/A
CC_GO:0030870_Mre11+complex	0.0926624	#N/A	0.0926624
MF_GO:0033613_transcription+activator+binding	0.956696	#N/A	0.956696
MF_GO:0004140_dephospho-CoA+kinase+activity	0.343669	0.343669	#N/A
MF_GO:0004595_pantetheine-phosphate+adenylyltransferase+activity	0.343669	0.343669	#N/A
BP_GO:0051823_regulation+of+synapse+structural+plasticity	0.22079	0.22079	#N/A
BP_GO:0001887_selenium+metabolic+process	0.893838	0.893838	#N/A
BP_GO:0030240_muscle+thin+filament+assembly	0.572272	#N/A	0.572272
BP_GO:0055008_cardiac+muscle+morphogenesis	0.572272	#N/A	0.572272
BP_GO:0019852_L-ascorbic+acid+metabolic+process	0.808186	#N/A	0.808186
MF_GO:0004357_glutamate-cysteine+ligase+activity	0.808186	#N/A	0.808186
BP_GO:0033993_response+to+lipid	0.533717	0.533717	#N/A
BP_GO:0048669_collateral+sprouting+in+the+absence+of+injury	0.988449	0.988449	#N/A
CC_GO:0051233_spindle+midzone	0.988449	0.988449	#N/A
MF_GO:0047499_calcium-independent+phospholipase+A2+activity	0.0678519	0.0678519	#N/A
BP_GO:0001923_B-1+B+cell+differentiation	0.538769	0.538769	#N/A
BP_GO:0006627_mitochondrial+protein+processing+during+import	0.897281	#N/A	0.897281
CC_GO:0042720_mitochondrial+inner+membrane+peptidase+complex	0.897281	#N/A	0.897281
MF_GO:0047134_protein-disulfide+reductase+activity	0.0917883	0.0917883	#N/A
BP_GO:0034332_adherens+junction+organization	0.369617	#N/A	0.369617
BP_GO:0051647_nucleus+localization	0.369617	#N/A	0.369617
CC_GO:0042585_germinal+vesicle	0.793575	#N/A	0.793575
MF_GO:0004770_sterol+carrier+protein+X-related+thiolase+activity	0.943189	#N/A	0.943189
MF_GO:0033814_propanoyl-CoA+C-acyltransferase+activity	0.943189	#N/A	0.943189
CC_GO:0030121_AP-1+adaptor+complex	0.227741	0.227741	#N/A
BP_GO:0014740_negative+regulation+of+muscle+hyperplasia	0.355404	0.355404	#N/A
BP_GO:0014806_smooth+muscle+hyperplasia	0.355404	0.355404	#N/A
BP_GO:0051549_positive+regulation+of+keratinocyte+migration	0.351843	0.351843	#N/A
BP_GO:0015817_histidine+transport	0.443394	0.443394	#N/A
MF_GO:0005290_L-histidine+transmembrane+transporter+activity	0.443394	0.443394	#N/A
MF_GO:0003978_UDP-glucose+4-epimerase+activity	0.461268	0.461268	#N/A
MF_GO:0004058_aromatic-L-amino-acid+decarboxylase+activity	0.693446	#N/A	0.693446
MF_GO:0030748_amine+N-methyltransferase+activity	0.146473	0.146473	#N/A
BP_GO:0033578_protein+amino+acid+glycosylation+in+Golgi	0.885512	#N/A	0.885512
BP_GO:0043112_receptor+metabolic+process	0.885512	#N/A	0.885512
CC_GO:0032580_Golgi+cisterna+membrane	0.885512	#N/A	0.885512
MF_GO:0008424_glycoprotein+6-alpha-L-fucosyltransferase+activity	0.885512	#N/A	0.885512

MF_GO:0046921_alpha(1,6)-fucosyltransferase+activity	0.885512	#N/A	0.885512
BP_GO:0060318_definitive+erythrocyte+differentiation	0.547788	0.547788	#N/A
BP_GO:0015798_myo-inositol+transport	0.888228	#N/A	0.888228
MF_GO:0050512_lactosylceramide+4-alpha-galactosyltransferase+activity	0.381991	#N/A	0.381991
BP_GO:0031129_inductive+cell-cell+signaling	0.836757	#N/A	0.836757
BP_GO:0006545_glycine+biosynthetic+process	0.564855	#N/A	0.564855
MF_GO:0004146_dihydrofolate+reductase+activity	0.564855	#N/A	0.564855
BP_GO:0042447_hormone+catabolic+process	0.689679	0.689679	#N/A
BP_GO:0045362_positive+regulation+of+interleukin-1+biosynthetic+process	0.916328	0.916328	#N/A
BP_GO:0045368_positive+regulation+of+interleukin-13+biosynthetic+proces	0.916328	0.916328	#N/A
CC_GO:0000221_vacuolar+proton-transporting+V-type+ATPase,+V1+domair	0.673031	#N/A	0.673031
MF_GO:0050613_delta14-sterol+reductase+activity	0.321728	0.321728	#N/A
MF_GO:0005157_macrophage+colony+stimulating+factor+receptor+binding	0.388649	0.388649	#N/A
BP_GO:0006636_unsaturated+fatty+acid+biosynthetic+process	0.524261	0.524261	#N/A
MF_GO:0016213_linoleoyl-CoA+desaturase+activity	0.524261	0.524261	#N/A
BP_GO:0006598_polyamine+catabolic+process	0.244304	0.244304	#N/A
CC_GO:0032311_angiogenin-PRI+complex	0.538298	#N/A	0.538298
BP_GO:0018158_protein+amino+acid+oxidation	0.221567	0.221567	#N/A
BP_GO:0051897_positive+regulation+of+protein+kinase+B+signaling+cascac	0.221567	0.221567	#N/A
MF_GO:0005534_galactose+binding	0.234894	0.234894	#N/A
MF_GO:0016936_galactoside+binding	0.234894	0.234894	#N/A
CC_GO:0045160_myosin+I+complex	0.699369	0.699369	#N/A
MF_GO:0004903_growth+hormone+receptor+activity	0.933045	0.933045	#N/A
MF_GO:0017046_peptide+hormone+binding	0.933045	0.933045	#N/A
BP_GO:0007019_microtubule+depolymerization	0.713745	#N/A	0.713745
BP_GO:0031115_negative+regulation+of+microtubule+polymerization	0.713745	#N/A	0.713745
MF_GO:0043890_N-acetylgalactosamine-6-sulfatase+activity	0.541863	#N/A	0.541863
BP_GO:0009132_nucleoside+diphosphate+metabolic+process	0.509204	0.509204	#N/A
BP_GO:0015938_coenzyme+A+catabolic+process	0.509204	0.509204	#N/A
BP_GO:0046356_acetyl-CoA+catabolic+process	0.509204	0.509204	#N/A
BP_GO:0006581_acetylcholine+catabolic+process	0.938099	#N/A	0.938099
MF_GO:0003990_acetylcholinesterase+activity	0.938099	#N/A	0.938099
MF_GO:0004304_estrone+sulfotransferase+activity	0.590184	#N/A	0.590184
MF_GO:0004508_steroid+17-alpha-monooxygenase+activity	0.920146	0.920146	#N/A
BP_GO:0021785_branchiomotor+neuron+axon+guidance	0.89735	#N/A	0.89735
BP_GO:0006535_cysteine+biosynthetic+process+from+serine	0.389856	0.389856	#N/A
BP_GO:0019343_cysteine+biosynthetic+process+via+cystathionine	0.389856	0.389856	#N/A
MF_GO:0004122_cystathionine+beta-synthase+activity	0.389856	0.389856	#N/A
MF_GO:0004423_iduronate-2-sulfatase+activity	0.922383	#N/A	0.922383
CC_GO:0032127_dense+core+granule+membrane	0.428277	0.428277	#N/A
BP_GO:0048017_inositol+lipid-mediated+signaling	0.725508	0.725508	#N/A
BP_GO:0050663_cytokine+secretion	0.616615	0.616615	#N/A
BP_GO:0007096_regulation+of+exit+from+mitosis	0.38767	#N/A	0.38767
BP_GO:0006653_lecithin+metabolic+process	0.421407	0.421407	#N/A
BP_GO:0006776_vitamin+A+metabolic+process	0.421407	0.421407	#N/A
MF_GO:0008374_O-acyltransferase+activity	0.421407	0.421407	#N/A
MF_GO:0047173_phosphatidylcholine-retinol+O-acyltransferase+activity	0.421407	0.421407	#N/A

MF_GO:0004856_xylulokinase+activity	0.69998	#N/A	0.69998
BP_GO:0001985_negative+regulation+of+heart+rate+in+baroreceptor+respo	0.00907392	#N/A	0.00907392
MF_GO:0051786_all-trans-retinol+13,14-reductase+activity	0.436219	0.436219	#N/A
BP_GO:0030204_chondroitin+sulfate+metabolic+process	0.815197	#N/A	0.815197
BP_GO:0033037_polysaccharide+localization	0.815197	#N/A	0.815197
BP_GO:0045637_regulation+of+myeloid+cell+differentiation	0.0110289	#N/A	0.0110289
MF_GO:0004001_adenosine+kinase+activity	0.45178	0.45178	#N/A
BP_GO:0002066_columnar/cuboidal+epithelial+cell+development	0.935999	#N/A	0.935999
BP_GO:0032943_mononuclear+cell+proliferation	0.767763	0.767763	#N/A
BP_GO:0042306_regulation+of+protein+import+into+nucleus	0.767763	0.767763	#N/A
BP_GO:0048298_positive+regulation+of+isotype+switching+to+IgA+isotypes	0.767763	0.767763	#N/A
BP_GO:0046587_positive+regulation+of+calcium-dependent+cell-cell+adhes	0.912669	0.912669	#N/A
CC_GO:0016939_kinesin+II+complex	0.912669	0.912669	#N/A
BP_GO:0046038_GMP+catabolic+process	0.396397	#N/A	0.396397
MF_GO:0042296_ISG15+ligase+activity	0.760566	0.760566	#N/A
BP_GO:0006949_syncytium+formation	0.829683	0.829683	#N/A
BP_GO:0046327_glycerol+biosynthetic+process+from+pyruvate	0.336626	#N/A	0.336626
MF_GO:0004155_6,7-dihydropteridine+reductase+activity	0.481125	0.481125	#N/A
BP_GO:0002651_positive+regulation+of+tolerance+induction+to+self+antige	0.639259	#N/A	0.639259
BP_GO:0002663_positive+regulation+of+B+cell+tolerance+induction	0.639259	#N/A	0.639259
CC_GO:0043235_receptor+complex	0.639259	#N/A	0.639259
BP_GO:0043124_negative+regulation+of+I-kappaB+kinase/NF-kappaB+casca	0.839971	0.839971	#N/A
MF_GO:0002113_interleukin-33+binding	0.839971	0.839971	#N/A
MF_GO:0002114_interleukin-33+receptor+activity	0.839971	0.839971	#N/A
BP_GO:0051001_negative+regulation+of+nitric-oxide+synthase+activity	0.020665	0.020665	#N/A
MF_GO:0033130_acetylcholine+receptor+binding	0.0930245	0.0930245	#N/A
MF_GO:0003958_NADPH-hemoprotein+reductase+activity	0.3936	0.3936	#N/A
BP_GO:0009124_nucleoside+monophosphate+biosynthetic+process	0.966666	0.966666	#N/A
BP_GO:0009133_nucleoside+diphosphate+biosynthetic+process	0.966666	0.966666	#N/A
MF_GO:0004343_glucosamine+6-phosphate+N-acetyltransferase+activity	0.28316	0.28316	#N/A
BP_GO:0007497_posterior+midgut+development	0.209315	#N/A	0.209315
MF_GO:0001600_endothelin-B+receptor+activity	0.209315	#N/A	0.209315
BP_GO:0002248_connective+tissue+replacement+during+inflammatory+res	0.528041	#N/A	0.528041
BP_GO:0010634_positive+regulation+of+epithelial+cell+migration	0.528041	#N/A	0.528041
BP_GO:0051541_elastin+metabolic+process	0.528041	#N/A	0.528041
BP_GO:0021903_rostrocaudal+neural+tube+patterning	0.237003	#N/A	0.237003
MF_GO:0046539_histamine+N-methyltransferase+activity	0.808594	0.808594	#N/A
MF_GO:0004766_spermidine+synthase+activity	0.58491	0.58491	#N/A
BP_GO:0034976_response+to+endoplasmic+reticulum+stress	0.678427	#N/A	0.678427
BP_GO:0006591_ornithine+metabolic+process	0.611994	0.611994	#N/A
MF_GO:0008792_arginine+decarboxylase+activity	0.611994	0.611994	#N/A
MF_GO:0005055_laminin+receptor+activity	0.744087	0.744087	#N/A
BP_GO:0033275_actin-myosin+filament+sliding	0.835918	#N/A	0.835918
CC_GO:0000138_Golgi+trans+cisterna	0.550725	0.550725	#N/A
BP_GO:0001909_leukocyte+mediated+cytotoxicity	0.758005	0.758005	#N/A
MF_GO:0008281_sulfonylurea+receptor+activity	0.749498	0.749498	#N/A
MF_GO:0046316_gluconokinase+activity	0.247662	#N/A	0.247662

BP_GO:0008611_ether+lipid+biosynthetic+process	0.645841	0.645841	#N/A
MF_GO:0016287_glycerone-phosphate+O-acyltransferase+activity	0.645841	0.645841	#N/A
MF_GO:0004312_fatty-acid+synthase+activity	0.525559	0.525559	#N/A
MF_GO:0004313_[acyl-carrier-protein]+S-acetyltransferase+activity	0.525559	0.525559	#N/A
MF_GO:0004316_3-oxoacyl-[acyl-carrier-protein]+reductase+activity	0.525559	0.525559	#N/A
MF_GO:0004317_3-hydroxypalmitoyl-[acyl-carrier-protein]+dehydratase+ac	0.525559	0.525559	#N/A
MF_GO:0004319_enoyl-[acyl-carrier-protein]+reductase+(NADPH,+B-specifi	0.525559	0.525559	#N/A
BP_GO:0015886_heme+transport	0.228858	#N/A	0.228858
MF_GO:0015232_heme+transporter+activity	0.228858	#N/A	0.228858
BP_GO:0006900_membrane+budding	0.552183	#N/A	0.552183
BP_GO:0014049_positive+regulation+of+glutamate+secretion	0.552183	#N/A	0.552183
BP_GO:0032308_positive+regulation+of+prostaglandin+secretion	0.552183	#N/A	0.552183
BP_GO:0032731_positive+regulation+of+interleukin-1+beta+production	0.552183	#N/A	0.552183
BP_GO:0034405_response+to+fluid+shear+stress	0.552183	#N/A	0.552183
BP_GO:0043132_NAD+transport	0.552183	#N/A	0.552183
BP_GO:0044254_multicellular+organismal+protein+catabolic+process	0.552183	#N/A	0.552183
BP_GO:0046931_pore+complex+biogenesis	0.552183	#N/A	0.552183
BP_GO:0051901_positive+regulation+of+mitochondrial+depolarization	0.552183	#N/A	0.552183
BP_GO:0009231_riboflavin+biosynthetic+process	0.188801	0.188801	#N/A
MF_GO:0008531_riboflavin+kinase+activity	0.188801	0.188801	#N/A
MF_GO:0045127_N-acetylglucosamine+kinase+activity	0.537878	#N/A	0.537878
BP_GO:0042276_error-prone+postreplication+DNA+repair	0.221519	#N/A	0.221519
MF_GO:0017125_deoxycytidyl+transferase+activity	0.221519	#N/A	0.221519
BP_GO:0042738_exogenous+drug+catabolic+process	0.950131	#N/A	0.950131
BP_GO:0035229_positive+regulation+of+glutamate-cysteine+ligase+activity	0.332057	0.332057	#N/A
MF_GO:0035226_glutamate-cysteine+ligase+catalytic+subunit+binding	0.332057	0.332057	#N/A
BP_GO:0006683_galactosylceramide+catabolic+process	0.346165	0.346165	#N/A
MF_GO:0004336_galactosylceramidase+activity	0.346165	0.346165	#N/A
MF_GO:0019782_ISG15+activating+enzyme+activity	0.649168	0.649168	#N/A
BP_GO:0042840_D-glucuronate+catabolic+process	0.621603	0.621603	#N/A
BP_GO:0046185_aldehyde+catabolic+process	0.621603	0.621603	#N/A
MF_GO:0008106_alcohol+dehydrogenase+(NADP+)+activity	0.621603	0.621603	#N/A
MF_GO:0047939_L-glucuronate+reductase+activity	0.621603	0.621603	#N/A
MF_GO:0045029_UDP-activated+nucleotide+receptor+activity	0.44938	#N/A	0.44938
BP_GO:0006684_sphingomyelin+metabolic+process	0.717413	#N/A	0.717413
CC_GO:0030288_outer+membrane-bounded+periplasmic+space	0.0219313	0.0219313	#N/A
BP_GO:0005997_xylulose+metabolic+process	0.906374	#N/A	0.906374
MF_GO:0050038_L-xylulose+reductase+activity	0.906374	#N/A	0.906374
CC_GO:0005895_interleukin-5+receptor+complex	0.350412	0.350412	#N/A
BP_GO:0055070_copper+ion+homeostasis	0.945492	#N/A	0.945492
BP_GO:0030389_fructosamine+metabolic+process	0.582671	0.582671	#N/A
MF_GO:0030387_fructosamine-3-kinase+activity	0.582671	0.582671	#N/A
MF_GO:0004736_pyruvate+carboxylase+activity	0.196252	0.196252	#N/A
MF_GO:0003979_UDP-glucose+6-dehydrogenase+activity	0.294283	#N/A	0.294283
MF_GO:0004075_biotin+carboxylase+activity	0.315622	#N/A	0.315622
BP_GO:0045075_regulation+of+interleukin-12+biosynthetic+process	0.894855	#N/A	0.894855
BP_GO:0015881_creatine+transport	0.145435	0.145435	#N/A

MF_GO:0005308_creatine+transporter+activity	0.145435	0.145435	#N/A
MF_GO:0005309_creatine:sodium+symporter+activity	0.145435	0.145435	#N/A
MF_GO:0033858_N-acetylgalactosamine+kinase+activity	0.762678	0.762678	#N/A
BP_GO:0016024_CDP-diacylglycerol+biosynthetic+process	0.233377	#N/A	0.233377
BP_GO:0002316_follicular+B+cell+differentiation	0.77546	#N/A	0.77546
BP_GO:0032237_activation+of+store-operated+calcium+channel+activity	0.77546	#N/A	0.77546
BP_GO:0030728_ovulation	0.216691	#N/A	0.216691
BP_GO:0019676_ammonia+assimilation+cycle	0.832082	#N/A	0.832082
BP_GO:0042450_arginine+biosynthetic+process+via+ornithine	0.832082	#N/A	0.832082
MF_GO:0004056_argininosuccinate+lyase+activity	0.832082	#N/A	0.832082
MF_GO:0004306_ethanolamine-phosphate+cytidylyltransferase+activity	0.100817	0.100817	#N/A
MF_GO:0032440_2-alkenal+reductase+activity	0.455416	#N/A	0.455416
BP_GO:0008653_lipopolysaccharide+metabolic+process	0.511571	#N/A	0.511571
MF_GO:0050528_acyloxyacyl+hydrolase+activity	0.511571	#N/A	0.511571
BP_GO:0046462_monoacylglycerol+metabolic+process	0.42094	#N/A	0.42094
MF_GO:0017120_polyphosphoinositide+phosphatase+activity	0.227873	#N/A	0.227873
BP_GO:0031638_zymogen+activation	0.890752	#N/A	0.890752
MF_GO:0047277_globoside+alpha-N-acetylgalactosaminyltransferase+activi	0.965867	0.965867	#N/A
MF_GO:0033884_phosphoethanolamine/phosphocholine+phosphatase+acti	0.101067	0.101067	#N/A
BP_GO:0010464_regulation+of+mesenchymal+cell+proliferation	0.0996829	#N/A	0.0996829
BP_GO:0051150_regulation+of+smooth+muscle+cell+differentiation	0.0996829	#N/A	0.0996829
MF_GO:0004168_dolichol+kinase+activity	0.959035	0.959035	#N/A
BP_GO:0006114_glycerol+biosynthetic+process	0.506109	0.506109	#N/A
BP_GO:0055089_fatty+acid+homeostasis	0.506109	0.506109	#N/A
BP_GO:0051170_nuclear+import	0.358944	0.358944	#N/A
BP_GO:0042489_negative+regulation+of+odontogenesis+of+dentine-contai	0.442052	0.442052	#N/A
MF_GO:0047015_3-hydroxy-2-methylbutyryl-CoA+dehydrogenase+activity	0.377697	0.377697	#N/A
MF_GO:0000210_NAD++diphosphatase+activity	0.169607	#N/A	0.169607
MF_GO:0008478_pyridoxal+kinase+activity	0.639566	0.639566	#N/A
MF_GO:0008460_dTDP-glucose+4,6-dehydratase+activity	0.677548	0.677548	#N/A
BP_GO:0046653_tetrahydrofolate+metabolic+process	0.573159	#N/A	0.573159
MF_GO:0008115_sarcosine+oxidase+activity	0.573159	#N/A	0.573159
MF_GO:0050031_L-pipecolate+oxidase+activity	0.573159	#N/A	0.573159
BP_GO:0050863_regulation+of+T+cell+activation	0.906689	#N/A	0.906689
MF_GO:0000250_lanosterol+synthase+activity	0.760517	0.760517	#N/A
MF_GO:0051538_3+iron,+4+sulfur+cluster+binding	0.00220752	0.00220752	#N/A
MF_GO:0004092_carnitine+O-acetyltransferase+activity	0.465949	0.465949	#N/A
BP_GO:0006507_GPI+anchor+release	0.570288	#N/A	0.570288
MF_GO:0004621_glycosylphosphatidylinositol+phospholipase+D+activity	0.570288	#N/A	0.570288
BP_GO:0035246_peptidyl-arginine+N-methylation	0.487933	0.487933	#N/A
BP_GO:0019402_galactitol+metabolic+process	0.422148	0.422148	#N/A
BP_GO:0002502_peptide+antigen+assembly+with+MHC+class+I+protein+co	0.895155	0.895155	#N/A
BP_GO:0010149_senescence	0.895155	0.895155	#N/A
CC_GO:0042824_MHC+class+I+peptide+loading+complex	0.895155	0.895155	#N/A
MF_GO:0004353_glutamate+dehydrogenase+[NAD(P)+]+activity	0.271347	0.271347	#N/A
MF_GO:0001571_non-tyrosine+kinase+fibroblast+growth+factor+receptor+	0.830425	#N/A	0.830425
MF_GO:0047012_sterol-4-alpha-carboxylate+3-dehydrogenase+(decarboxyl	0.114245	#N/A	0.114245

BP_GO:0060043_regulation+of+cardiac+muscle+cell+proliferation	0.330004	0.330004	#N/A
BP_GO:0060314_regulation+of+ryanodine-sensitive+calcium-release+chann	0.218143	0.218143	#N/A
MF_GO:0008144_drug+binding	0.218143	0.218143	#N/A
BP_GO:0007100_mitotic+centrosome+separation	0.797223	#N/A	0.797223
BP_GO:0030490_maturation+of+SSU-rRNA	0.370094	0.370094	#N/A
BP_GO:0048752_semicircular+canal+morphogenesis	0.027364	0.027364	#N/A
BP_GO:0060005_vestibular+reflex	0.027364	0.027364	#N/A
MF_GO:0043874_acireductone+synthase+activity	0.373845	0.373845	#N/A
MF_GO:0004340_glucokinase+activity	0.586897	0.586897	#N/A
BP_GO:0002001_renin+secretion+into+blood+stream	0.960408	#N/A	0.960408
MF_GO:0001596_angiotensin+type+I+receptor+activity	0.960408	#N/A	0.960408
BP_GO:0032957_inositol+trisphosphate+metabolic+process	0.376595	0.376595	#N/A
MF_GO:0035300_inositol-1,3,4-trisphosphate+5/6-kinase+activity	0.376595	0.376595	#N/A
MF_GO:0047325_inositol+tetrakisphosphate+1-kinase+activity	0.376595	0.376595	#N/A
MF_GO:0003955_NAD(P)H+dehydrogenase+(quinone)+activity	0.00852882	0.00852882	#N/A
MF_GO:0047391_alkylglycerophosphoethanolamine+phosphodiesterase+ac	0.940365	#N/A	0.940365
MF_GO:0016641_oxidoreductase+activity,+acting+on+the+CH-NH2+group+c	0.414205	0.414205	#N/A
CC_GO:0031092_platelet+alpha+granule+membrane	0.0804572	#N/A	0.0804572
BP_GO:0043681_protein+import+into+mitochondrion	0.894852	0.894852	#N/A
BP_GO:0001811_negative+regulation+of+type+I+hypersensitivity	0.825784	0.825784	#N/A
MF_GO:0015211_purine+nucleoside+transmembrane+transporter+activity	0.338659	0.338659	#N/A
MF_GO:0000014_single-stranded+DNA+specific+endodeoxyribonuclease+ac	0.524617	0.524617	#N/A
MF_GO:0004157_dihydropyrimidinase+activity	0.529704	0.529704	#N/A
BP_GO:0045909_positive+regulation+of+vasodilation	0.516989	#N/A	0.516989
BP_GO:0002036_regulation+of+L-glutamate+transport	0.355627	#N/A	0.355627
BP_GO:0002520_immune+system+development	0.89561	#N/A	0.89561
BP_GO:0010523_negative+regulation+of+calcium+ion+transport+into+cytos	0.89561	#N/A	0.89561
BP_GO:0010559_regulation+of+glycoprotein+biosynthetic+process	0.89561	#N/A	0.89561
BP_GO:0014042_positive+regulation+of+neuron+maturation	0.89561	#N/A	0.89561
BP_GO:0014911_positive+regulation+of+smooth+muscle+cell+migration	0.89561	#N/A	0.89561
BP_GO:0021747_cochlear+nucleus+development	0.89561	#N/A	0.89561
BP_GO:0032835_glomerulus+development	0.89561	#N/A	0.89561
BP_GO:0033033_negative+regulation+of+myeloid+cell+apoptosis	0.89561	#N/A	0.89561
BP_GO:0033689_negative+regulation+of+osteoblast+proliferation	0.89561	#N/A	0.89561
BP_GO:0043375_CD8-positive,+alpha-beta+T+cell+lineage+commitment	0.89561	#N/A	0.89561
BP_GO:0045069_regulation+of+viral+genome+replication	0.89561	#N/A	0.89561
BP_GO:0048743_positive+regulation+of+skeletal+muscle+fiber+developmer	0.89561	#N/A	0.89561
BP_GO:0048753_pigment+granule+organization	0.89561	#N/A	0.89561
BP_GO:0042264_peptidyl-aspartic+acid+hydroxylation	0.269014	#N/A	0.269014
MF_GO:0004496_mevalonate+kinase+activity	0.866499	0.866499	#N/A
BP_GO:0032640_tumor+necrosis+factor+production	0.510001	#N/A	0.510001
BP_GO:0007424_open+tracheal+system+development	0.760958	#N/A	0.760958
BP_GO:0009052_pentose-phosphate+shunt,+non-oxidative+branch	0.825716	#N/A	0.825716
MF_GO:0004751_ribose-5-phosphate+isomerase+activity	0.825716	#N/A	0.825716
BP_GO:0007632_visual+behavior	0.901071	0.901071	#N/A
BP_GO:0034341_response+to+interferon-gamma	0.369943	0.369943	#N/A
CC_GO:0031314_extrinsic+to+mitochondrial+inner+membrane	0.687543	#N/A	0.687543

MF_GO:0042624_ATPase+activity,+uncoupled	0.478137	#N/A	0.478137
MF_GO:0008321_Ral+guanyl-nucleotide+exchange+factor+activity	0.528103	0.528103	#N/A
BP_GO:0018344_protein+geranylgeranylation	0.230371	0.230371	#N/A
MF_GO:0008859_exoribonuclease+II+activity	0.0991784	#N/A	0.0991784
MF_GO:0047560_3-dehydrosphinganine+reductase+activity	0.360078	0.360078	#N/A
MF_GO:0008200_ion+channel+inhibitor+activity	0.0515533	0.0515533	#N/A
MF_GO:0030550_acetylcholine+receptor+inhibitor+activity	0.0515533	0.0515533	#N/A
MF_GO:0004662_CAAX-protein+geranylgeranyltransferase+activity	0.331629	0.331629	#N/A
BP_GO:0046477_glycosylceramide+catabolic+process	0.635159	0.635159	#N/A
MF_GO:0004557_alpha-galactosidase+activity	0.635159	0.635159	#N/A
BP_GO:0009203_ribonucleoside+triphosphate+catabolic+process	0.980901	#N/A	0.980901
MF_GO:0000215_tRNA+2'-phosphotransferase+activity	0.333213	0.333213	#N/A
MF_GO:0008665_2'-phosphotransferase+activity	0.333213	0.333213	#N/A
BP_GO:0015680_intracellular+copper+ion+transport	0.59338	#N/A	0.59338
MF_GO:0043682_copper-transporting+ATPase+activity	0.59338	#N/A	0.59338
MF_GO:0008117_sphinganine-1-phosphate+aldolase+activity	0.411596	#N/A	0.411596
BP_GO:0046487_glyoxylate+metabolic+process	0.715797	0.715797	#N/A
BP_GO:0046724_oxalic+acid+secretion	0.715797	0.715797	#N/A
MF_GO:0004760_serine-pyruvate+transaminase+activity	0.715797	0.715797	#N/A
BP_GO:0046381_CMP-N-acetylneuraminate+metabolic+process	0.636286	#N/A	0.636286
MF_GO:0030338_CMP-N-acetylneuraminate+monooxygenase+activity	0.636286	#N/A	0.636286
BP_GO:0051754_meiotic+sister+chromatid+cohesion,+centromeric	0.922676	0.922676	#N/A
MF_GO:0001874_zymosan+receptor+activity	0.380301	0.380301	#N/A
BP_GO:0006892_post-Golgi+vesicle-mediated+transport	0.286717	0.286717	#N/A
MF_GO:0004515_nicotinate-nucleotide+adenylyltransferase+activity	0.766311	#N/A	0.766311
BP_GO:0044236_multicellular+organismal+metabolic+process	0.718403	#N/A	0.718403
BP_GO:0051006_positive+regulation+of+lipoprotein+lipase+activity	0.351205	#N/A	0.351205
BP_GO:0043122_regulation+of+I-kappaB+kinase/NF-kappaB+cascade	0.287334	0.287334	#N/A
BP_GO:0045357_regulation+of+interferon-beta+biosynthetic+process	0.287334	0.287334	#N/A
CC_GO:0045323_interleukin-1+receptor+complex	0.287334	0.287334	#N/A
MF_GO:0005199_structural+constituent+of+cell+wall	0.633854	#N/A	0.633854
BP_GO:0016119_carotene+metabolic+process	0.602162	0.602162	#N/A
BP_GO:0006687_glycosphingolipid+metabolic+process	0.587285	0.587285	#N/A
BP_GO:0008049_male+courtship+behavior	0.587285	0.587285	#N/A
BP_GO:0031323_regulation+of+cellular+metabolic+process	0.587285	0.587285	#N/A
BP_GO:0016572_histone+phosphorylation	0.521951	0.521951	#N/A
BP_GO:0009229_thiamin+diphosphate+biosynthetic+process	0.89396	0.89396	#N/A
MF_GO:0004788_thiamin+diphosphokinase+activity	0.89396	0.89396	#N/A
BP_GO:0040019_positive+regulation+of+embryonic+development	0.163525	0.163525	#N/A
MF_GO:0009000_selenocysteine+lyase+activity	0.240758	0.240758	#N/A
BP_GO:0015722_canalicular+bile+acid+transport	0.977213	#N/A	0.977213
MF_GO:0042030_ATPase+inhibitor+activity	0.606307	0.606307	#N/A
MF_GO:0004368_glycerol-3-phosphate+dehydrogenase+activity	0.908415	0.908415	#N/A
BP_GO:0042495_detection+of+triacylated+bacterial+lipoprotein	0.386776	#N/A	0.386776
MF_GO:0042497_triacylated+lipoprotein+binding	0.386776	#N/A	0.386776
MF_GO:0048040_UDP-glucuronate+decarboxylase+activity	0.0255029	#N/A	0.0255029
BP_GO:0006401_RNA+catabolic+process	0.452831	#N/A	0.452831

BP_GO:0000096_sulfur+amino+acid+metabolic+process	0.993542	#N/A	0.993542
BP_GO:0021590_cerebellum+maturation	0.993542	#N/A	0.993542
BP_GO:0021679_cerebellar+molecular+layer+development	0.993542	#N/A	0.993542
BP_GO:0021750_vestibular+nucleus+development	0.993542	#N/A	0.993542
BP_GO:0032353_negative+regulation+of+hormone+biosynthetic+process	0.993542	#N/A	0.993542
BP_GO:0048791_calcium+ion-dependent+exocytosis+of+neurotransmitter	0.993542	#N/A	0.993542
BP_GO:0002726_positive+regulation+of+T+cell+cytokine+production	0.414229	0.414229	#N/A
BP_GO:0002821_positive+regulation+of+adaptive+immune+response	0.414229	0.414229	#N/A
BP_GO:0051251_positive+regulation+of+lymphocyte+activation	0.414229	0.414229	#N/A
MF_GO:0008456_alpha-N-acetylgalactosaminidase+activity	0.381742	#N/A	0.381742
BP_GO:0002761_regulation+of+myeloid+leukocyte+differentiation	0.361067	0.361067	#N/A
MF_GO:0045130_keratan+sulfotransferase+activity	0.238065	0.238065	#N/A
CC_GO:0030478_actin+cap	0.262469	#N/A	0.262469
BP_GO:0007522_visceral+muscle+development	0.00637966	0.00637966	#N/A
MF_GO:0008112_nicotinamide+N-methyltransferase+activity	0.108794	#N/A	0.108794
MF_GO:0047223_beta-1,3-galactosyl-O-glycosyl-glycoprotein+beta-1,3-N-ac	0.0803089	0.0803089	#N/A
BP_GO:0006167_AMP+biosynthetic+process	0.394533	#N/A	0.394533
BP_GO:0021660_rhombomere+3+formation	0.386082	0.386082	#N/A
BP_GO:0021666_rhombomere+5+formation	0.386082	0.386082	#N/A
CC_GO:0030485_smooth+muscle+contractile+fiber	0.947022	#N/A	0.947022
CC_GO:0016600_flotillin+complex	0.0608336	0.0608336	#N/A
BP_GO:0030913_paranodal+junction+assembly	0.803865	#N/A	0.803865
BP_GO:0006021_inositol+biosynthetic+process	0.688337	0.688337	#N/A
MF_GO:0004512_inositol-3-phosphate+synthase+activity	0.688337	0.688337	#N/A
BP_GO:0048790_maintenance+of+presynaptic+active+zone+structure	0.0293923	0.0293923	#N/A
BP_GO:0050975_sensory+perception+of+touch	0.0293923	0.0293923	#N/A
MF_GO:0050659_N-acetylgalactosamine+4-sulfate+6-O-sulfotransferase+ac	0.167221	#N/A	0.167221
BP_GO:0009890_negative+regulation+of+biosynthetic+process	0.7509	#N/A	0.7509
MF_GO:0016971_flavin-linked+sulfhydryl+oxidase+activity	0.302556	0.302556	#N/A
BP_GO:0006066_cellular+alcohol+metabolic+process	0.949777	#N/A	0.949777
BP_GO:0019285_glycine+betaine+biosynthetic+process+from+choline	0.949777	#N/A	0.949777
MF_GO:0008812_choline+dehydrogenase+activity	0.949777	#N/A	0.949777
BP_GO:0001880_Mullerian+duct+regression	0.457747	#N/A	0.457747
BP_GO:0048382_mesendoderm+development	0.457747	#N/A	0.457747
MF_GO:0030729_acetoacetate-CoA+ligase+activity	0.553565	0.553565	#N/A
BP_GO:0019673_GDP-mannose+metabolic+process	0.18856	#N/A	0.18856
MF_GO:0008446_GDP-mannose+4,6-dehydratase+activity	0.18856	#N/A	0.18856
BP_GO:0032287_myelin+maintenance+in+the+peripheral+nervous+system	0.436528	#N/A	0.436528
MF_GO:0055100_adiponectin+binding	0.437932	0.437932	#N/A
BP_GO:0043457_regulation+of+cellular+respiration	0.285999	0.285999	#N/A
BP_GO:0030201_heparan+sulfate+proteoglycan+metabolic+process	0.14913	0.14913	#N/A
MF_GO:0016250_N-sulfoglucosamine+sulfohydrolase+activity	0.14913	0.14913	#N/A
MF_GO:0004782_sulfinoalanine+decarboxylase+activity	0.457875	#N/A	0.457875
MF_GO:0033989_3alpha,7alpha,12alpha-trihydroxy-5beta-cholest-24-enoyl-	0.449547	0.449547	#N/A
BP_GO:0000266_mitochondrial+fission	0.147968	0.147968	#N/A
BP_GO:0006784_heme+a+biosynthetic+process	0.147968	0.147968	#N/A
BP_GO:0017004_cytochrome+complex+assembly	0.147968	0.147968	#N/A

BP_GO:0018347_protein+amino+acid+farnesylation	0.147968	0.147968	#N/A
MF_GO:0030161_calpain+inhibitor+activity	0.0727779	#N/A	0.0727779
BP_GO:0006178_guanine+salvage	0.781887	0.781887	#N/A
BP_GO:0045964_positive+regulation+of+dopamine+metabolic+process	0.781887	0.781887	#N/A
BP_GO:0046100_hypoxanthine+metabolic+process	0.781887	0.781887	#N/A
MF_GO:0004698_calcium-dependent+protein+kinase+C+activity	0.785825	#N/A	0.785825
BP_GO:0007208_serotonin+receptor,+phospholipase+C+activating+pathway	0.721626	#N/A	0.721626
BP_GO:0016560_protein+import+into+peroxisome+matrix,+docking	0.787537	#N/A	0.787537
MF_GO:0004031_aldehyde+oxidase+activity	0.631119	0.631119	#N/A
MF_GO:0004631_phosphomevalonate+kinase+activity	0.738121	0.738121	#N/A
BP_GO:0002479_antigen+processing+and+presentation+of+exogenous+pep	0.35145	0.35145	#N/A
BP_GO:0050823_peptide+antigen+stabilization	0.35145	0.35145	#N/A
BP_GO:0016444_somatic+cell+DNA+recombination	0.164404	0.164404	#N/A
MF_GO:0016844_strictosidine+synthase+activity	0.435507	0.435507	#N/A
BP_GO:0000075_cell+cycle+checkpoint	0.609918	0.609918	#N/A
BP_GO:0042892_chloramphenicol+transport	0.43003	0.43003	#N/A
MF_GO:0004139_deoxyribose-phosphate+aldolase+activity	0.377139	0.377139	#N/A
MF_GO:0008329_pattern+recognition+receptor+activity	0.725542	0.725542	#N/A
BP_GO:0006206_pyrimidine+base+metabolic+process	0.995479	0.995479	#N/A
BP_GO:0006213_pyrimidine+nucleoside+metabolic+process	0.995479	0.995479	#N/A
MF_GO:0009032_thymidine+phosphorylase+activity	0.995479	0.995479	#N/A
BP_GO:0001987_vasoconstriction+of+artery+involved+in+baroreceptor+res	0.798348	#N/A	0.798348
BP_GO:0045818_negative+regulation+of+glycogen+catabolic+process	0.798348	#N/A	0.798348
BP_GO:0045819_positive+regulation+of+glycogen+catabolic+process	0.798348	#N/A	0.798348
CC_GO:0005639_integral+to+nuclear+inner+membrane	0.193317	0.193317	#N/A
MF_GO:0004487_methylenetetrahydrofolate+dehydrogenase+(NAD+)+activ	0.643647	0.643647	#N/A
MF_GO:0004108_citrate+(Si)-synthase+activity	0.383588	0.383588	#N/A
BP_GO:0040001_establishment+of+mitotic+spindle+localization	0.889005	#N/A	0.889005
BP_GO:0001886_endothelial+cell+morphogenesis	0.911874	0.911874	#N/A
MF_GO:0030160_GKAP/Homer+scaffold+activity	0.0376913	0.0376913	#N/A
MF_GO:0004326_tetrahydrofolylpolyglutamate+synthase+activity	0.175913	0.175913	#N/A
BP_GO:0051694_pointed-end+actin+filament+capping	0.781455	#N/A	0.781455
MF_GO:0051011_microtubule+minus-end+binding	0.781455	#N/A	0.781455
MF_GO:0050104_L-gulonate+3-dehydrogenase+activity	0.0968849	0.0968849	#N/A
MF_GO:0008887_glycerate+kinase+activity	0.528337	0.528337	#N/A
BP_GO:0007225_patched+ligand+processing	0.821224	0.821224	#N/A
BP_GO:0045657_positive+regulation+of+monocyte+differentiation	0.762833	0.762833	#N/A
BP_GO:0019067_viral+assembly,+maturation,+egress,+and+release	0.535239	#N/A	0.535239
MF_GO:0015093_ferrous+iron+transmembrane+transporter+activity	0.809123	0.809123	#N/A
BP_GO:0048389_intermediate+mesoderm+development	0.191249	#N/A	0.191249
MF_GO:0004854_xanthine+dehydrogenase+activity	0.498019	#N/A	0.498019
MF_GO:0004855_xanthine+oxidase+activity	0.498019	#N/A	0.498019
MF_GO:0004801_transaldolase+activity	0.219077	0.219077	#N/A
MF_GO:0030731_guanidinoacetate+N-methyltransferase+activity	0.264958	0.264958	#N/A
CC_GO:0005796_Golgi+lumen	0.784671	0.784671	#N/A
BP_GO:0031110_regulation+of+microtubule+polymerization+or+depolymer	0.701507	#N/A	0.701507
BP_GO:0048549_positive+regulation+of+pinocytosis	0.217053	0.217053	#N/A

CC_GO:0031143_pseudopodium	0.217053	0.217053	#N/A
MF_GO:0004339_glucan+1,4-alpha-glucosidase+activity	0.937024	#N/A	0.937024
BP_GO:0002431_Fc+receptor+mediated+stimulatory+signaling+pathway	0.951109	#N/A	0.951109
BP_GO:0032765_positive+regulation+of+mast+cell+cytokine+production	0.951109	#N/A	0.951109
BP_GO:0033026_negative+regulation+of+mast+cell+apoptosis	0.951109	#N/A	0.951109
BP_GO:0009262_deoxyribonucleotide+metabolic+process	0.804231	#N/A	0.804231
BP_GO:0032872_regulation+of+stress-activated+MAPK+cascade	0.245624	0.245624	#N/A
BP_GO:0042345_regulation+of+NF-kappaB+import+into+nucleus	0.245624	0.245624	#N/A
CC_GO:0030891_VCB+complex	0.039487	0.039487	#N/A
BP_GO:0045841_negative+regulation+of+mitotic+metaphase/anaphase+tra	0.0385111	#N/A	0.0385111
CC_GO:0005816_spindle+pole+body	0.0385111	#N/A	0.0385111
MF_GO:0004458_D-lactate+dehydrogenase+(cytochrome)+activity	0.530786	0.530786	#N/A
MF_GO:0008720_D-lactate+dehydrogenase+activity	0.530786	0.530786	#N/A
BP_GO:0015822_ornithine+transport	0.71729	0.71729	#N/A
MF_GO:0000064_L-ornithine+transmembrane+transporter+activity	0.71729	0.71729	#N/A
MF_GO:0005289_high+affinity+arginine+transmembrane+transporter+activi	0.71729	0.71729	#N/A
MF_GO:0005020_stem+cell+factor+receptor+activity	0.500266	#N/A	0.500266
BP_GO:0005991_trehalose+metabolic+process	0.843896	0.843896	#N/A
MF_GO:0004555_alpha,alpha-trehalase+activity	0.843896	0.843896	#N/A
BP_GO:0032446_protein+modification+by+small+protein+conjugation	0.426352	0.426352	#N/A
MF_GO:0004839_ubiquitin+activating+enzyme+activity	0.426352	0.426352	#N/A
MF_GO:0004774_succinate-CoA+ligase+activity	0.494619	0.494619	#N/A
BP_GO:0045090_retroviral+genome+replication	0.410766	0.410766	#N/A
BP_GO:0000052_citrulline+metabolic+process	0.846023	#N/A	0.846023
BP_GO:0019244_lactate+biosynthetic+process+from+pyruvate	0.846023	#N/A	0.846023
BP_GO:0019805_quinolinate+biosynthetic+process	0.846023	#N/A	0.846023
BP_GO:0021988_olfactory+lobe+development	0.846023	#N/A	0.846023
BP_GO:0021990_neural+plate+formation	0.846023	#N/A	0.846023
MF_GO:0050809_diazepam+binding	0.846023	#N/A	0.846023
MF_GO:0008907_integrase+activity	0.666309	#N/A	0.666309
BP_GO:0050428_3'-phosphoadenosine+5'-phosphosulfate+biosynthetic+pro	0.698475	#N/A	0.698475
CC_GO:0009336_sulfate+adenylyltransferase+complex+(ATP)	0.698475	#N/A	0.698475
MF_GO:0004349_glutamate+5-kinase+activity	0.188176	#N/A	0.188176
MF_GO:0004350_glutamate-5-semialdehyde+dehydrogenase+activity	0.188176	#N/A	0.188176
CC_GO:0032593_insulin-responsive+compartment	0.92825	0.92825	#N/A
BP_GO:0015698_inorganic+anion+transport	0.455194	#N/A	0.455194
BP_GO:0043271_negative+regulation+of+ion+transport	0.455194	#N/A	0.455194
BP_GO:0015721_bile+acid+and+bile+salt+transport	0.76341	#N/A	0.76341
BP_GO:0019896_axon+transport+of+mitochondrion	0.0232381	0.0232381	#N/A
MF_GO:0047291_lactosylceramide+alpha-2,3-sialyltransferase+activity	0.979358	#N/A	0.979358
CC_GO:0005818_aster	0.9548	#N/A	0.9548
BP_GO:0031076_embryonic+camera-type+eye+development	0.736429	0.736429	#N/A
MF_GO:0045030_UTP-activated+nucleotide+receptor+activity	0.588599	0.588599	#N/A
BP_GO:0030382_sperm+mitochondrion+organization	0.245602	0.245602	#N/A
BP_GO:0006668_sphinganine-1-phosphate+metabolic+process	0.0964228	#N/A	0.0964228
MF_GO:0042392_sphingosine-1-phosphate+phosphatase+activity	0.0964228	#N/A	0.0964228
BP_GO:0048539_bone+marrow+development	0.69676	0.69676	#N/A

MF_GO:0047280_nicotinamide+phosphoribosyltransferase+activity	0.0685131	#N/A	0.0685131
BP_GO:0051300_spindle+pole+body+organization	0.980778	#N/A	0.980778
MF_GO:0000225_N-acetylglucosaminylphosphatidylinositol+deacetylase+ac	0.224787	#N/A	0.224787
BP_GO:0007402_ganglion+mother+cell+fate+determination	0.037553	0.037553	#N/A
BP_GO:0008052_sensory+organ+boundary+specification	0.037553	0.037553	#N/A
MF_GO:0031750_D3+dopamine+receptor+binding	0.452378	0.452378	#N/A
MF_GO:0004655_porphobilinogen+synthase+activity	0.137855	0.137855	#N/A
BP_GO:0006474_N-terminal+protein+amino+acid+acetylation	0.819728	0.819728	#N/A
BP_GO:0060323_head+morphogenesis	0.392149	0.392149	#N/A
MF_GO:0047933_glucose-1,6-bisphosphate+synthase+activity	0.987808	0.987808	#N/A
BP_GO:0030488_tRNA+methylation	0.603746	0.603746	#N/A
MF_GO:0016429_tRNA+(adenine-N1-)-methyltransferase+activity	0.603746	0.603746	#N/A
MF_GO:0008526_phosphatidylinositol+transporter+activity	0.746283	0.746283	#N/A
MF_GO:0004904_interferon+receptor+activity	0.790841	#N/A	0.790841
BP_GO:0050746_regulation+of+lipoprotein+metabolic+process	0.0884235	0.0884235	#N/A
MF_GO:0008970_phospholipase+A1+activity	0.0884235	0.0884235	#N/A
MF_GO:0001848_complement+binding	0.810981	#N/A	0.810981
BP_GO:0009311_oligosaccharide+metabolic+process	0.111966	0.111966	#N/A
MF_GO:0004573_mannosyl-oligosaccharide+glucosidase+activity	0.111966	0.111966	#N/A
BP_GO:0043461_proton-transporting+ATP+synthase+complex+assembly	0.666736	0.666736	#N/A
CC_GO:0005971_ribonucleoside-diphosphate+reductase+complex	0.670181	#N/A	0.670181
BP_GO:0019532_oxalate+transport	0.960526	#N/A	0.960526
MF_GO:0015116_sulfate+transmembrane+transporter+activity	0.960526	#N/A	0.960526
MF_GO:0019531_oxalate+transmembrane+transporter+activity	0.960526	#N/A	0.960526
BP_GO:0032474_otolith+morphogenesis	0.681507	0.681507	#N/A
BP_GO:0006203_dGTP+catabolic+process	0.400053	0.400053	#N/A
BP_GO:0006930_substrate-bound+cell+migration,+cell+extension	0.543249	0.543249	#N/A
BP_GO:0021592_fourth+ventricle+development	0.543249	0.543249	#N/A
BP_GO:0021678_third+ventricle+development	0.543249	0.543249	#N/A
BP_GO:0009069_serine+family+amino+acid+metabolic+process	0.12531	#N/A	0.12531
BP_GO:0070179_D-serine+biosynthetic+process	0.12531	#N/A	0.12531
MF_GO:0018114_threonine+racemase+activity	0.12531	#N/A	0.12531
MF_GO:0030378_serine+racemase+activity	0.12531	#N/A	0.12531
BP_GO:0009972_cytidine+deamination	0.00752861	0.00752861	#N/A
MF_GO:0030226_apolipoprotein+receptor+activity	0.884126	#N/A	0.884126
MF_GO:0019007_N-acetylneuraminic+acid+phosphate+synthase+activity	0.26972	0.26972	#N/A
BP_GO:0030174_regulation+of+DNA+replication+initiation	0.510436	0.510436	#N/A
MF_GO:0004408_holocytochrome-c+synthase+activity	0.252629	0.252629	#N/A
BP_GO:0009913_epidermal+cell+differentiation	0.155264	0.155264	#N/A
MF_GO:0030305_heparanase+activity	0.657993	0.657993	#N/A
CC_GO:0030014_CCR4-NOT+complex	0.116178	#N/A	0.116178
MF_GO:0004561_alpha-N-acetylglucosaminidase+activity	0.355667	0.355667	#N/A
MF_GO:0050277_sedoheptulokinase+activity	0.736139	0.736139	#N/A
BP_GO:0050983_spermidine+catabolic+process+to+deoxyhypusine,+using+c	0.178516	0.178516	#N/A
MF_GO:0034038_deoxyhypusine+synthase+activity	0.178516	0.178516	#N/A
BP_GO:0046874_quinolinate+metabolic+process	0.873234	#N/A	0.873234
MF_GO:0001760_aminocarboxymuconate-semialdehyde+decarboxylase+act	0.873234	#N/A	0.873234

BP_GO:0008054_cyclin+catabolic+process	0.798919	#N/A	0.798919
BP_GO:0002756_MyD88-independent+toll-like+receptor+signaling+pathway	0.235713	#N/A	0.235713
BP_GO:0032481_positive+regulation+of+type+I+interferon+production	0.235713	#N/A	0.235713
BP_GO:0006235_dTTP+biosynthetic+process	0.309446	#N/A	0.309446
BP_GO:0002769_natural+killer+cell+inhibitory+signaling+pathway	0.96661	0.96661	#N/A
BP_GO:0046886_positive+regulation+of+hormone+biosynthetic+process	0.305406	0.305406	#N/A
MF_GO:0043758_acetate-CoA+ligase+(ADP-forming)+activity	0.986439	#N/A	0.986439
BP_GO:0007231_osmosensory+signaling+pathway	0.261314	0.261314	#N/A
BP_GO:0030103_vasopressin+secretion	0.261314	0.261314	#N/A
BP_GO:0042538_hyperosmotic+salinity+response	0.261314	0.261314	#N/A
MF_GO:0005034_osmosensor+activity	0.261314	0.261314	#N/A
MF_GO:0004463_leukotriene-A4+hydrolase+activity	0.555494	0.555494	#N/A
BP_GO:0015918_sterol+transport	0.903073	#N/A	0.903073
BP_GO:0055092_sterol+homeostasis	0.903073	#N/A	0.903073
BP_GO:0046825_regulation+of+protein+export+from+nucleus	0.938091	#N/A	0.938091
BP_GO:0048609_reproductive+process+in+a+multicellular+organism	0.938091	#N/A	0.938091
BP_GO:0051463_negative+regulation+of+cortisol+secretion	0.938091	#N/A	0.938091
BP_GO:0060125_negative+regulation+of+growth+hormone+secretion	0.938091	#N/A	0.938091
MF_GO:0051428_peptide+hormone+receptor+binding	0.938091	#N/A	0.938091
BP_GO:0006842_tricarboxylic+acid+transport	0.612732	#N/A	0.612732
BP_GO:0015744_succinate+transport	0.612732	#N/A	0.612732
MF_GO:0015141_succinate+transmembrane+transporter+activity	0.612732	#N/A	0.612732
MF_GO:0015142_tricarboxylic+acid+transmembrane+transporter+activity	0.612732	#N/A	0.612732
BP_GO:0033605_positive+regulation+of+catecholamine+secretion	0.0137444	0.0137444	#N/A
BP_GO:0046007_negative+regulation+of+activated+T+cell+proliferation	0.623099	0.623099	#N/A
MF_GO:0019900_kinase+binding	0.93768	#N/A	0.93768
BP_GO:0002082_regulation+of+oxidative+phosphorylation	0.621354	#N/A	0.621354
BP_GO:0006568_tryptophan+metabolic+process	0.621354	#N/A	0.621354
BP_GO:0015677_copper+ion+import	0.621354	#N/A	0.621354
BP_GO:0018205_peptidyl-lysine+modification	0.621354	#N/A	0.621354
BP_GO:0042093_T-helper+cell+differentiation	0.621354	#N/A	0.621354
BP_GO:0042414_epinephrine+metabolic+process	0.621354	#N/A	0.621354
BP_GO:0048553_negative+regulation+of+metalloenzyme+activity	0.621354	#N/A	0.621354
BP_GO:0051542_elastin+biosynthetic+process	0.621354	#N/A	0.621354
BP_GO:0060003_copper+ion+export	0.621354	#N/A	0.621354
MF_GO:0016532_superoxide+dismutase+copper+chaperone+activity	0.621354	#N/A	0.621354
MF_GO:0047988_hydroxyacid-oxoacid+transhydrogenase+activity	0.477732	#N/A	0.477732
BP_GO:0031508_centromeric+heterochromatin+formation	0.168711	#N/A	0.168711
MF_GO:0008482_sulfite+oxidase+activity	0.439225	0.439225	#N/A
BP_GO:0002040_sprouting+angiogenesis	0.538632	0.538632	#N/A
BP_GO:0007440_foregut+morphogenesis	0.538632	0.538632	#N/A
BP_GO:0045618_positive+regulation+of+keratinocyte+differentiation	0.538632	0.538632	#N/A
BP_GO:0046533_negative+regulation+of+photoreceptor+cell+differentiation	0.538632	0.538632	#N/A
BP_GO:0033025_regulation+of+mast+cell+apoptosis	0.762758	#N/A	0.762758
BP_GO:0048870_cell+motility	0.762758	#N/A	0.762758
BP_GO:0051088_PMA-inducible+membrane+protein+ectodomain+proteolys	0.762758	#N/A	0.762758
MF_GO:0047598_7-dehydrocholesterol+reductase+activity	0.495179	#N/A	0.495179

MF_GO:0004733_pyridoxamine-phosphate+oxidase+activity	0.651425	0.651425	#N/A
MF_GO:0008609_alkylglycerone-phosphate+synthase+activity	0.755622	#N/A	0.755622
MF_GO:0008444_CDP-diacylglycerol-glycerol-3-phosphate+3-phosphatidyltr	0.0344263	0.0344263	#N/A
MF_GO:0004085_butyryl-CoA+dehydrogenase+activity	0.022616	0.022616	#N/A
BP_GO:0006027_glycosaminoglycan+catabolic+process	0.394028	#N/A	0.394028
MF_GO:0004363_glutathione+synthase+activity	0.436913	#N/A	0.436913
MF_GO:0003834_beta-carotene+15,15'-monooxygenase+activity	0.0793507	#N/A	0.0793507
MF_GO:0000254_C-4+methylsterol+oxidase+activity	0.0659281	#N/A	0.0659281
MF_GO:0016531_copper+chaperone+activity	0.986511	0.986511	#N/A
MF_GO:0050080_malonyl-CoA+decarboxylase+activity	0.0339606	0.0339606	#N/A
BP_GO:0030208_dermatan+sulfate+biosynthetic+process	0.126073	0.126073	#N/A
MF_GO:0004506_squalene+monooxygenase+activity	0.375268	#N/A	0.375268
MF_GO:0003943_N-acetylgalactosamine-4-sulfatase+activity	0.387694	#N/A	0.387694
MF_GO:0008116_prostaglandin-I+synthase+activity	0.753292	#N/A	0.753292
MF_GO:0008545_JUN+kinase+kinase+activity	0.404194	0.404194	#N/A
BP_GO:0043171_peptide+catabolic+process	0.979532	#N/A	0.979532
MF_GO:0008240_tripeptidyl-peptidase+activity	0.979532	#N/A	0.979532
MF_GO:0047708_biotinidase+activity	0.792127	#N/A	0.792127
CC_GO:0030665_clathrin+coated+vesicle+membrane	0.612226	#N/A	0.612226
CC_GO:0009288_flagellin-based+flagellum	0.200431	#N/A	0.200431
BP_GO:0021695_cerebellar+cortex+development	0.794789	0.794789	#N/A
BP_GO:0021697_cerebellar+cortex+formation	0.794789	0.794789	#N/A
BP_GO:0031914_negative+regulation+of+synaptic+plasticity	0.794789	0.794789	#N/A
BP_GO:0032801_receptor+catabolic+process	0.794789	0.794789	#N/A
BP_GO:0033136_serine+phosphorylation+of+STAT3+protein	0.794789	0.794789	#N/A
BP_GO:0045956_positive+regulation+of+calcium+ion-dependent+exocytosis	0.794789	0.794789	#N/A
BP_GO:0046826_negative+regulation+of+protein+export+from+nucleus	0.794789	0.794789	#N/A
MF_GO:0030549_acetylcholine+receptor+activator+activity	0.794789	0.794789	#N/A
MF_GO:0043125_ErbB-3+class+receptor+binding	0.794789	0.794789	#N/A
BP_GO:0051403_stress-activated+MAPK+cascade	0.504824	0.504824	#N/A
MF_GO:0042806_fucose+binding	0.859514	#N/A	0.859514
MF_GO:0033925_mannosyl-glycoprotein+endo-beta-N-acetylglucosaminida	0.593989	0.593989	#N/A
MF_GO:0034602_oxoglutarate+dehydrogenase+(NAD+)+activity	0.432667	0.432667	#N/A
BP_GO:0001971_negative+regulation+of+activation+of+membrane+attack+	0.694354	#N/A	0.694354
BP_GO:0045918_negative+regulation+of+cytolysis	0.694354	#N/A	0.694354
MF_GO:0050815_phosphoserine+binding	0.955198	#N/A	0.955198
MF_GO:0050816_phosphothreonine+binding	0.955198	#N/A	0.955198
MF_GO:0008761_UDP-N-acetylglucosamine+2-epimerase+activity	0.989428	#N/A	0.989428
MF_GO:0004382_guanosine-diphosphatase+activity	0.166028	0.166028	#N/A
MF_GO:0045134_uridine-diphosphatase+activity	0.166028	0.166028	#N/A
BP_GO:0006531_aspartate+metabolic+process	0.674437	0.674437	#N/A
MF_GO:0008445_D-aspartate+oxidase+activity	0.674437	0.674437	#N/A
CC_GO:0000407_pre-autophagosomal+structure	0.180343	0.180343	#N/A
BP_GO:0048242_epinephrine+secretion	0.436469	0.436469	#N/A
MF_GO:0004729_protoporphyrinogen+oxidase+activity	0.0123678	0.0123678	#N/A
MF_GO:0004061_arylformamidase+activity	0.766907	#N/A	0.766907
BP_GO:0030322_stabilization+of+membrane+potential	0.501094	#N/A	0.501094

BP_GO:0042592_homeostatic+process	0.39903	0.39903	#N/A
BP_GO:0032682_negative+regulation+of+chemokine+production	0.156821	0.156821	#N/A
BP_GO:0014067_negative+regulation+of+phosphoinositide+3-kinase+cascac	0.65931	0.65931	#N/A
BP_GO:0050918_positive+chemotaxis	0.65931	0.65931	#N/A
BP_GO:0045724_positive+regulation+of+flagellum+assembly	0.313767	#N/A	0.313767
MF_GO:0008890_glycine+C-acetyltransferase+activity	0.097744	0.097744	#N/A
BP_GO:0010046_response+to+mycotoxin	0.388614	0.388614	#N/A
MF_GO:0004572_mannosyl-oligosaccharide+1,3-1,6-alpha-mannosidase+act	0.533687	#N/A	0.533687
BP_GO:0045626_negative+regulation+of+T-helper+1+cell+differentiation	0.679109	0.679109	#N/A
BP_GO:0007260_tyrosine+phosphorylation+of+STAT+protein	0.420079	#N/A	0.420079
BP_GO:0042517_positive+regulation+of+tyrosine+phosphorylation+of+Stat3	0.420079	#N/A	0.420079
MF_GO:0004696_glycogen+synthase+kinase+3+activity	0.756046	#N/A	0.756046
BP_GO:0051685_maintenance+of+ER+location	0.24494	0.24494	#N/A
BP_GO:0042231_interleukin-13+biosynthetic+process	0.565782	0.565782	#N/A
BP_GO:0045906_negative+regulation+of+vasoconstriction	0.249821	#N/A	0.249821
BP_GO:0015724_formate+transport	0.0525026	0.0525026	#N/A
MF_GO:0015499_formate+transmembrane+transporter+activity	0.0525026	0.0525026	#N/A
CC_GO:0008352_katanin+complex	0.940064	#N/A	0.940064
BP_GO:0019682_glyceraldehyde-3-phosphate+metabolic+process	0.207007	0.207007	#N/A
MF_GO:0004807_triose-phosphate+isomerase+activity	0.207007	0.207007	#N/A
BP_GO:0010614_negative+regulation+of+cardiac+muscle+hypertrophy	0.986645	0.986645	#N/A
MF_GO:0004137_deoxycytidine+kinase+activity	0.581893	#N/A	0.581893
MF_GO:0016784_3-mercaptopyruvate+sulfurtransferase+activity	0.0663331	0.0663331	#N/A
MF_GO:0015362_high+affinity+sodium:dicarboxylate+symporter+activity	0.811597	#N/A	0.811597
BP_GO:0002029_desensitization+of+G-protein+coupled+receptor+protein+s	0.399242	0.399242	#N/A
BP_GO:0046856_phosphoinositide+dephosphorylation	0.69081	#N/A	0.69081
BP_GO:0051895_negative+regulation+of+focal+adhesion+formation	0.69081	#N/A	0.69081
MF_GO:0016314_phosphatidylinositol-3,4,5-trisphosphate+3-phosphatase+a	0.69081	#N/A	0.69081
MF_GO:0051800_phosphatidylinositol-3,4-bisphosphate+3-phosphatase+act	0.69081	#N/A	0.69081
MF_GO:0004738_pyruvate+dehydrogenase+activity	0.728607	0.728607	#N/A
MF_GO:0017106_activin+inhibitor+activity	0.911444	#N/A	0.911444
MF_GO:0016603_glutaminyl-peptide+cyclotransferase+activity	0.357045	0.357045	#N/A
BP_GO:0007296_vitellogenesis	0.757675	#N/A	0.757675
MF_GO:0016262_protein+N-acetylglucosaminyltransferase+activity	0.973982	0.973982	#N/A
MF_GO:0033872_[heparan+sulfate]-glucosamine+3-sulfotransferase+3+activ	0.631255	0.631255	#N/A
BP_GO:0002827_positive+regulation+of+T-helper+1+type+immune+respons	0.248887	0.248887	#N/A
MF_GO:0045509_interleukin-27+receptor+activity	0.248887	0.248887	#N/A
BP_GO:0021572_rhombomere+6+development	0.0912299	#N/A	0.0912299
BP_GO:0000055_ribosomal+large+subunit+export+from+nucleus	0.504402	#N/A	0.504402
MF_GO:0005139_interleukin-7+receptor+binding	0.623642	#N/A	0.623642
MF_GO:0042805_actinin+binding	0.850095	0.850095	#N/A
BP_GO:0001986_negative+regulation+of+the+force+of+heart+contraction+c	0.943985	0.943985	#N/A
CC_GO:0042599_lamellar+body	0.349277	0.349277	#N/A
BP_GO:0002457_T+cell+antigen+processing+and+presentation	0.359441	0.359441	#N/A
BP_GO:0009401_phosphoenolpyruvate-dependent+sugar+phosphotransfera	0.315039	#N/A	0.315039
BP_GO:0000046_autophagic+vacuole+fusion	0.937855	0.937855	#N/A
BP_GO:0016236_macroautophagy	0.937855	0.937855	#N/A

BP_GO:0051480_cytosolic+calcium+ion+homeostasis	0.937855	0.937855	#N/A
CC_GO:0044433_cytoplasmic+vesicle+part	0.937855	0.937855	#N/A
BP_GO:0043089_positive+regulation+of+Cdc42+GTPase+activity	0.584461	0.584461	#N/A
BP_GO:0045899_positive+regulation+of+transcriptional+preinitiation+comp	0.433829	#N/A	0.433829
CC_GO:0000506_glycosylphosphatidylinositol-N-acetylglucosaminyltransfera	0.322445	0.322445	#N/A
BP_GO:0046069_cGMP+catabolic+process	0.410211	#N/A	0.410211
MF_GO:0004112_cyclic-nucleotide+phosphodiesterase+activity	0.410211	#N/A	0.410211
MF_GO:0015278_calcium-release+channel+activity	0.964825	#N/A	0.964825
MF_GO:0004347_glucose-6-phosphate+isomerase+activity	0.389939	0.389939	#N/A
BP_GO:0018120_peptidyl-arginine+ADP-ribosylation	0.0826629	#N/A	0.0826629
CC_GO:0000275_mitochondrial+proton-transporting+ATP+synthase+comple	0.862408	0.862408	#N/A
MF_GO:0017047_adrenocorticotropin-releasing+hormone+binding	0.527383	#N/A	0.527383
BP_GO:0031062_positive+regulation+of+histone+methylation	0.167062	0.167062	#N/A
BP_GO:0051154_negative+regulation+of+striated+muscle+cell+differentiatio	0.241499	#N/A	0.241499
MF_GO:0001618_viral+receptor+activity	0.137509	0.137509	#N/A
MF_GO:0005175_CD27+receptor+binding	0.137509	0.137509	#N/A
BP_GO:0001550_ovarian+cumulus+expansion	0.634637	0.634637	#N/A
BP_GO:0018208_peptidyl-proline+modification	0.276948	#N/A	0.276948
BP_GO:0031274_positive+regulation+of+pseudopodium+formation	0.315823	#N/A	0.315823
BP_GO:0042483_negative+regulation+of+odontogenesis	0.315823	#N/A	0.315823
BP_GO:0051171_regulation+of+nitrogen+compound+metabolic+process	0.315823	#N/A	0.315823
BP_GO:0051988_regulation+of+attachment+of+spindle+microtubules+to+ki	0.315823	#N/A	0.315823
MF_GO:0042577_lipid+phosphatase+activity	0.567112	#N/A	0.567112
MF_GO:0001691_pseudophosphatase+activity	0.0995992	0.0995992	#N/A
BP_GO:0019255_glucose+1-phosphate+metabolic+process	0.311314	0.311314	#N/A
MF_GO:0004610_phosphoacetylglucosamine+mutase+activity	0.311314	0.311314	#N/A
MF_GO:0008520_L-ascorbate:sodium+symporter+activity	0.897421	#N/A	0.897421
BP_GO:0045185_maintenance+of+protein+location	0.145274	0.145274	#N/A
MF_GO:0050827_toxin+receptor+binding	0.986686	#N/A	0.986686
BP_GO:0046853_inositol+and+derivative+phosphorylation	0.752052	0.752052	#N/A
MF_GO:0000824_inositol+tetrakisphosphate+3-kinase+activity	0.752052	0.752052	#N/A
MF_GO:0004163_diphosphomevalonate+decarboxylase+activity	0.0966784	0.0966784	#N/A
MF_GO:0004156_dihydropteroate+synthase+activity	0.475568	#N/A	0.475568
MF_GO:0008705_methionine+synthase+activity	0.475568	#N/A	0.475568
MF_GO:0004633_phosphopantothenoylcysteine+decarboxylase+activity	0.117848	0.117848	#N/A
BP_GO:0016082_synaptic+vesicle+priming	0.970196	#N/A	0.970196
BP_GO:0015014_heparan+sulfate+proteoglycan+biosynthetic+process,+poly	0.753302	#N/A	0.753302
BP_GO:0030202_heparin+metabolic+process	0.753302	#N/A	0.753302
MF_GO:0004394_heparan+sulfate+2-O-sulfotransferase+activity	0.753302	#N/A	0.753302
BP_GO:0046087_cytidine+metabolic+process	0.33577	0.33577	#N/A
BP_GO:0033108_mitochondrial+respiratory+chain+complex+assembly	0.263838	0.263838	#N/A
BP_GO:0009200_deoxyribonucleoside+triphosphate+metabolic+process	0.709162	0.709162	#N/A
MF_GO:0005384_manganese+ion+transmembrane+transporter+activity	0.680762	#N/A	0.680762
MF_GO:0008398_sterol+14-demethylase+activity	0.560588	#N/A	0.560588
BP_GO:0042986_positive+regulation+of+amyloid+precursor+protein+biosyn	0.671313	#N/A	0.671313
CC_GO:0005675_holo+TFIIH+complex	0.948288	0.948288	#N/A
BP_GO:0032229_negative+regulation+of+synaptic+transmission,+GABAergic	0.41542	#N/A	0.41542

BP_GO:0015670_carbon+dioxide+transport	0.342883	0.342883	#N/A
BP_GO:0043308_eosinophil+degranulation	0.985463	#N/A	0.985463
CC_GO:0031902_late+endosome+membrane	0.985463	#N/A	0.985463
MF_GO:0005283_sodium:amino+acid+symporter+activity	0.733519	0.733519	#N/A
MF_GO:0004578_chitobiosyldiphosphodolichol+beta-mannosyltransferase+a	0.0225169	0.0225169	#N/A
MF_GO:0004395_hexaprenyldihydroxybenzoate+methyltransferase+activity	0.446321	#N/A	0.446321
MF_GO:0008425_2-polyprenyl-6-methoxy-1,4-benzoquinone+methyltransfe	0.446321	#N/A	0.446321
BP_GO:0043071_positive+regulation+of+non-apoptotic+programmed+cell+c	0.790273	0.790273	#N/A
BP_GO:0006766_vitamin+metabolic+process	0.0609385	#N/A	0.0609385
BP_GO:0016074_snoRNA+metabolic+process	0.0399844	0.0399844	#N/A
MF_GO:0003826_alpha-ketoacid+dehydrogenase+activity	0.242824	0.242824	#N/A
MF_GO:0004677_DNA-dependent+protein+kinase+activity	0.619003	#N/A	0.619003
MF_GO:0003947_(N-acetylneuraminyl)-galactosylglucosylceramide+N-acety	0.553614	0.553614	#N/A
BP_GO:0006041_glucosamine+metabolic+process	0.903832	#N/A	0.903832
BP_GO:0046370_fructose+biosynthetic+process	0.903832	#N/A	0.903832
BP_GO:0045026_plasma+membrane+fusion	0.163516	0.163516	#N/A
BP_GO:0006556_S-adenosylmethionine+biosynthetic+process	0.561522	#N/A	0.561522
BP_GO:0035083_cilium+axoneme+assembly	0.9005	0.9005	#N/A
BP_GO:0050658_RNA+transport	0.10461	0.10461	#N/A
BP_GO:0016553_base+conversion+or+substitution+editing	0.944653	#N/A	0.944653
MF_GO:0043843_ADP-specific+glucokinase+activity	0.336295	#N/A	0.336295
MF_GO:0030586_[methionine+synthase]+reductase+activity	0.0930202	0.0930202	#N/A
MF_GO:0047138_aquacobalamin+reductase+activity	0.0930202	0.0930202	#N/A
BP_GO:0055069_zinc+ion+homeostasis	0.288779	0.288779	#N/A
MF_GO:0004077_biotin-[acetyl-CoA-carboxylase]+ligase+activity	0.875062	0.875062	#N/A
MF_GO:0004078_biotin-[methylcrotonoyl-CoA-carboxylase]+ligase+activity	0.875062	0.875062	#N/A
MF_GO:0004079_biotin-[methylmalonyl-CoA-carboxytransferase]+ligase+ac	0.875062	0.875062	#N/A
MF_GO:0004080_biotin-[propionyl-CoA-carboxylase+(ATP-hydrolyzing)]+liga	0.875062	0.875062	#N/A
BP_GO:0045602_negative+regulation+of+endothelial+cell+differentiation	0.477891	0.477891	#N/A
BP_GO:0006931_substrate-bound+cell+migration,+cell+attachment+to+subs	0.0404812	0.0404812	#N/A
BP_GO:0045601_regulation+of+endothelial+cell+differentiation	0.0766602	0.0766602	#N/A
BP_GO:0051569_regulation+of+histone+H3-K4+methylation	0.852367	#N/A	0.852367
BP_GO:0000381_regulation+of+alternative+nuclear+mRNA+splicing,+via+sp	0.269483	0.269483	#N/A
BP_GO:0007518_myoblast+cell+fate+determination	0.269483	0.269483	#N/A
MF_GO:0003944_N-acetylglucosamine-1-phosphodiester+alpha-N-acetylglu	0.568179	0.568179	#N/A
MF_GO:0000104_succinate+dehydrogenase+activity	0.339658	0.339658	#N/A
BP_GO:0006220_pyrimidine+nucleotide+metabolic+process	0.722231	#N/A	0.722231
BP_GO:0046108_uridine+metabolic+process	0.722231	#N/A	0.722231
BP_GO:0021935_granule+cell+precursor+tangential+migration	0.767684	0.767684	#N/A
MF_GO:0004132_dCMP+deaminase+activity	0.142971	#N/A	0.142971
BP_GO:0007093_mitotic+cell+cycle+checkpoint	0.578575	0.578575	#N/A
MF_GO:0008455_alpha-1,6-mannosylglycoprotein+2-beta-N-acetylglucosam	0.572081	#N/A	0.572081
BP_GO:0051964_negative+regulation+of+synaptogenesis	0.270958	#N/A	0.270958
MF_GO:0004324_ferredoxin-NADP++reductase+activity	0.00942632	#N/A	0.00942632
MF_GO:0004493_methylmalonyl-CoA+epimerase+activity	0.963453	0.963453	#N/A
MF_GO:0008262_importin-alpha+export+receptor+activity	0.947188	#N/A	0.947188
BP_GO:0045083_negative+regulation+of+interleukin-12+biosynthetic+proce	0.660058	0.660058	#N/A

BP_GO:0007521_muscle+cell+fate+determination	0.754562	0.754562	#N/A
MF_GO:0004466_long-chain-acyl-CoA+dehydrogenase+activity	0.134082	#N/A	0.134082
MF_GO:0001735_prenylcysteine+oxidase+activity	0.626396	0.626396	#N/A
MF_GO:0003882_CDP-diacylglycerol-serine+O-phosphatidyltransferase+acti	0.942416	0.942416	#N/A
MF_GO:0030791_arsenite+methyltransferase+activity	0.402202	0.402202	#N/A
MF_GO:0033882_choloyl-CoA+hydrolase+activity	0.487608	0.487608	#N/A
MF_GO:0004567_beta-mannosidase+activity	0.200359	0.200359	#N/A
MF_GO:0046935_1-phosphatidylinositol-3-kinase+regulator+activity	0.999592	#N/A	0.999592
BP_GO:0002315_marginal+zone+B+cell+differentiation	0.345963	#N/A	0.345963
BP_GO:0019730_antimicrobial+humoral+response	0.345963	#N/A	0.345963
BP_GO:0042536_negative+regulation+of+tumor+necrosis+factor+biosynthet	0.345963	#N/A	0.345963
BP_GO:0045064_T-helper+2+cell+differentiation	0.345963	#N/A	0.345963
BP_GO:0045082_positive+regulation+of+interleukin-10+biosynthetic+proces	0.345963	#N/A	0.345963
MF_GO:0019002_GMP+binding	0.120516	#N/A	0.120516
BP_GO:0021759_globus+pallidus+development	0.891721	#N/A	0.891721
MF_GO:0008108_UDP-glucose:hexose-1-phosphate+uridylyltransferase+acti	0.0421391	0.0421391	#N/A
BP_GO:0006296_nucleotide-excision+repair,+DNA+incision,+5'-to+lesion	0.795282	0.795282	#N/A
MF_GO:0004098_cerebroside-sulfatase+activity	0.391988	0.391988	#N/A
MF_GO:0005006_epidermal+growth+factor+receptor+activity	0.649005	#N/A	0.649005
BP_GO:0001866_NK+T+cell+proliferation	0.0181809	0.0181809	#N/A
BP_GO:0045062_extrathymic+T+cell+selection	0.0181809	0.0181809	#N/A
BP_GO:0050691_regulation+of+defense+response+to+virus+by+host	0.0181809	0.0181809	#N/A
CC_GO:0016011_dystroglycan+complex	0.347943	0.347943	#N/A
BP_GO:0042026_protein+refolding	0.953513	#N/A	0.953513
BP_GO:0045585_positive+regulation+of+cytotoxic+T+cell+differentiation	0.953513	#N/A	0.953513
CC_GO:0005719_nuclear+euchromatin	0.888123	0.888123	#N/A
MF_GO:0047710_bis(5'-adenosyl)-triphosphatase+activity	0.255913	#N/A	0.255913
BP_GO:0051865_protein+autoubiquitination	0.870132	#N/A	0.870132
BP_GO:0006430_lysyl-tRNA+aminoacylation	0.874461	#N/A	0.874461
MF_GO:0004824_lysine-tRNA+ligase+activity	0.874461	#N/A	0.874461
MF_GO:0004588_orotate+phosphoribosyltransferase+activity	0.243505	0.243505	#N/A
MF_GO:0004590_orotidine-5'-phosphate+decarboxylase+activity	0.243505	0.243505	#N/A
BP_GO:0019428_allantoin+biosynthetic+process	0.743203	#N/A	0.743203
MF_GO:0042328_heparan+sulfate+N-acetylglucosaminyltransferase+activity	0.21955	0.21955	#N/A
MF_GO:0030331_estrogen+receptor+binding	0.689133	#N/A	0.689133
MF_GO:0046966_thyroid+hormone+receptor+binding	0.689133	#N/A	0.689133
MF_GO:0046975_histone+lysine+N-methyltransferase+activity+(H3-K36+spe	0.689133	#N/A	0.689133
MF_GO:0008924_malate+dehydrogenase+(acceptor)+activity	0.875191	0.875191	#N/A
BP_GO:0002314_germinal+center+B+cell+differentiation	0.770644	0.770644	#N/A
BP_GO:0002906_negative+regulation+of+mature+B+cell+apoptosis	0.770644	0.770644	#N/A
BP_GO:0006154_adenosine+catabolic+process	0.770644	0.770644	#N/A
BP_GO:0006157_deoxyadenosine+catabolic+process	0.770644	0.770644	#N/A
BP_GO:0010460_positive+regulation+of+heart+rate	0.770644	0.770644	#N/A
BP_GO:0046061_dATP+catabolic+process	0.770644	0.770644	#N/A
BP_GO:0046101_hypoxanthine+biosynthetic+process	0.770644	0.770644	#N/A
BP_GO:0046103_inosine+biosynthetic+process	0.770644	0.770644	#N/A
BP_GO:0046111_xanthine+biosynthetic+process	0.770644	0.770644	#N/A

BP_GO:0021870_Cajal-Retzius+cell+differentiation	0.795135	#N/A	0.795135
BP_GO:0051966_regulation+of+synaptic+transmission,+glutamatergic	0.795135	#N/A	0.795135
BP_GO:0060075_regulation+of+resting+membrane+potential	0.795135	#N/A	0.795135
MF_GO:0004676_3-phosphoinositide-dependent+protein+kinase+activity	0.831841	#N/A	0.831841
MF_GO:0004556_alpha-amylase+activity	0.27505	#N/A	0.27505
BP_GO:0019046_reactivation+of+latent+virus	0.263024	0.263024	#N/A
BP_GO:0007635_chemosensory+behavior	0.369572	#N/A	0.369572
MF_GO:0004699_calcium-independent+protein+kinase+C+activity	0.369572	#N/A	0.369572
BP_GO:0060319_primitive+erythrocyte+differentiation	0.113339	0.113339	#N/A
BP_GO:0048592_eye+morphogenesis	0.94476	#N/A	0.94476
MF_GO:0004494_methylmalonyl-CoA+mutase+activity	0.69308	#N/A	0.69308
MF_GO:0005521_lamin+binding	0.869013	#N/A	0.869013
MF_GO:0047545_2-hydroxyglutarate+dehydrogenase+activity	0.314763	#N/A	0.314763
BP_GO:0021960_anterior+commissure+morphogenesis	0.490497	#N/A	0.490497
BP_GO:0045226_extracellular+polysaccharide+biosynthetic+process	0.378833	#N/A	0.378833
MF_GO:0008831_dTDP-4-dehydrorhamnose+reductase+activity	0.378833	#N/A	0.378833
CC_GO:0043626_PCNA+complex	0.163519	0.163519	#N/A
MF_GO:0030337_DNA+polymerase+processivity+factor+activity	0.163519	0.163519	#N/A
MF_GO:0005035_death+receptor+activity	0.660149	#N/A	0.660149
MF_GO:0048406_nerve+growth+factor+binding	0.660149	#N/A	0.660149
BP_GO:0015755_fructose+transport	0.00238602	0.00238602	#N/A
BP_GO:0015757_galactose+transport	0.00238602	0.00238602	#N/A
MF_GO:0005354_galactose+transmembrane+transporter+activity	0.00238602	0.00238602	#N/A
MF_GO:0005536_glucose+binding	0.00238602	0.00238602	#N/A
MF_GO:0015284_fructose+uniporter+activity	0.00238602	0.00238602	#N/A
BP_GO:0048878_chemical+homeostasis	0.242559	0.242559	#N/A
MF_GO:0003940_L-iduronidase+activity	0.242559	0.242559	#N/A
BP_GO:0052565_response+to+defense-related+host+nitric+oxide+productic	0.595137	#N/A	0.595137
MF_GO:0015446_arsenite+transmembrane-transporting+ATPase+activity	0.716868	#N/A	0.716868
BP_GO:0000042_protein+targeting+to+Golgi	0.705678	0.705678	#N/A
MF_GO:0047936_glucose+1-dehydrogenase+activity	0.30743	0.30743	#N/A
BP_GO:0006662_glycerol+ether+metabolic+process	0.942038	#N/A	0.942038
BP_GO:0001830_trophectodermal+cell+fate+commitment	0.0687999	0.0687999	#N/A
MF_GO:0019785_ISG15-specific+protease+activity	0.223336	0.223336	#N/A
MF_GO:0004694_eukaryotic+translation+initiation+factor+2alpha+kinase+a	0.405162	0.405162	#N/A
MF_GO:0016229_steroid+dehydrogenase+activity	0.694329	#N/A	0.694329
MF_GO:0046899_nucleoside+triphosphate+adenylate+kinase+activity	0.528043	#N/A	0.528043
BP_GO:0042226_interleukin-6+biosynthetic+process	0.42447	0.42447	#N/A
BP_GO:0021897_forebrain+astrocyte+development	0.374784	#N/A	0.374784
BP_GO:0042992_negative+regulation+of+transcription+factor+import+into+	0.374784	#N/A	0.374784
BP_GO:0045685_regulation+of+glial+cell+differentiation	0.374784	#N/A	0.374784
BP_GO:0045762_positive+regulation+of+adenylate+cyclase+activity	0.374784	#N/A	0.374784
BP_GO:0046929_negative+regulation+of+neurotransmitter+secretion	0.374784	#N/A	0.374784
CC_GO:0005845_mRNA+cap+binding+complex	0.13356	#N/A	0.13356
MF_GO:0004482_mRNA+(guanine-N7-)-methyltransferase+activity	0.13356	#N/A	0.13356
MF_GO:0047800_cysteamine+dioxygenase+activity	0.874191	#N/A	0.874191
BP_GO:0015732_prostaglandin+transport	0.532809	#N/A	0.532809

MF_GO:0015132_prostaglandin+transmembrane+transporter+activity	0.532809	#N/A	0.532809
BP_GO:0007035_vacuolar+acidification	0.931451	0.931451	#N/A
BP_GO:0032799_low-density+lipoprotein+receptor+metabolic+process	0.443414	#N/A	0.443414
BP_GO:0032803_regulation+of+low-density+lipoprotein+receptor+catabolic	0.443414	#N/A	0.443414
BP_GO:0032764_negative+regulation+of+mast+cell+cytokine+production	0.578197	0.578197	#N/A
BP_GO:0042092_T-helper+2+type+immune+response	0.578197	0.578197	#N/A
BP_GO:0043380_regulation+of+memory+T+cell+differentiation	0.578197	0.578197	#N/A
MF_GO:0003874_6-pyruvoyltetrahydropterin+synthase+activity	0.81393	#N/A	0.81393
BP_GO:0034474_U2+snRNA+3'-end+processing	0.117648	0.117648	#N/A
BP_GO:0033371_T+cell+secretory+granule+organization	0.040753	#N/A	0.040753
BP_GO:0033373_maintenance+of+protease+location+in+mast+cell+secretor	0.040753	#N/A	0.040753
BP_GO:0033382_maintenance+of+granzyme+B+location+in+T+cell+secretor	0.040753	#N/A	0.040753
CC_GO:0042629_mast+cell+granule	0.040753	#N/A	0.040753
BP_GO:0001777_T+cell+homeostatic+proliferation	0.0579951	0.0579951	#N/A
BP_GO:0030264_nuclear+fragmentation+during+apoptosis	0.0579951	0.0579951	#N/A
BP_GO:0033599_regulation+of+mammary+gland+epithelial+cell+proliferatic	0.0579951	0.0579951	#N/A
MF_GO:0051434_BH3+domain+binding	0.0579951	0.0579951	#N/A
BP_GO:0030578_PML+body+organization	0.758059	0.758059	#N/A
BP_GO:0045343_regulation+of+MHC+class+I+biosynthetic+process	0.758059	0.758059	#N/A
MF_GO:0004991_parathyroid+hormone+receptor+activity	0.62091	0.62091	#N/A
BP_GO:0040032_post-embryonic+body+morphogenesis	0.234569	0.234569	#N/A
BP_GO:0002086_diaphragm+contraction	0.251553	0.251553	#N/A
BP_GO:0042461_photoreceptor+cell+development	0.198244	#N/A	0.198244
MF_GO:0004490_methylglutaconyl-CoA+hydratase+activity	0.279057	0.279057	#N/A
MF_GO:0001681_sialate+O-acetylesterase+activity	0.855424	0.855424	#N/A
BP_GO:0006650_glycerophospholipid+metabolic+process	0.255609	0.255609	#N/A
BP_GO:0006678_glucosylceramide+metabolic+process	0.255609	0.255609	#N/A
BP_GO:0006681_galactosylceramide+metabolic+process	0.255609	0.255609	#N/A
BP_GO:0009450_gamma-aminobutyric+acid+catabolic+process	0.255609	0.255609	#N/A
MF_GO:0004724_magnesium-dependent+protein+serine/threonine+phosph	0.714298	#N/A	0.714298
BP_GO:0006090_pyruvate+metabolic+process	0.215167	0.215167	#N/A
CC_GO:0005967_mitochondrial+pyruvate+dehydrogenase+complex	0.215167	0.215167	#N/A
CC_GO:0045254_pyruvate+dehydrogenase+complex	0.215167	0.215167	#N/A
MF_GO:0004742_dihydrolipoyllysine-residue+acetyltransferase+activity	0.215167	0.215167	#N/A
BP_GO:0006188_IMP+biosynthetic+process	0.249873	0.249873	#N/A
MF_GO:0003937_IMP+cyclohydrolase+activity	0.249873	0.249873	#N/A
MF_GO:0004643_phosphoribosylaminoimidazolecarboxamide+formyltransf	0.249873	0.249873	#N/A
BP_GO:0002369_T+cell+cytokine+production	0.707104	#N/A	0.707104
BP_GO:0042982_amyloid+precursor+protein+metabolic+process	0.707104	#N/A	0.707104
BP_GO:0048639_positive+regulation+of+developmental+growth	0.707104	#N/A	0.707104
BP_GO:0048729_tissue+morphogenesis	0.707104	#N/A	0.707104
BP_GO:0060022_hard+palate+development	0.707104	#N/A	0.707104
BP_GO:0006663_platelet+activating+factor+biosynthetic+process	0.215123	#N/A	0.215123
MF_GO:0047192_1-alkylglycerophosphocholine+O-acetyltransferase+activit	0.215123	#N/A	0.215123
BP_GO:0042492_gamma-delta+T+cell+differentiation	0.0155045	0.0155045	#N/A
BP_GO:0021512_spinal+cord+anterior/posterior+patterning	0.0725003	0.0725003	#N/A
BP_GO:0030704_vitelline+membrane+formation	0.663188	0.663188	#N/A

MF_GO:0008316_structural+constituent+of+vitelline+membrane	0.663188	0.663188	#N/A
MF_GO:0047316_glutamine-phenylpyruvate+transaminase+activity	0.351438	0.351438	#N/A
BP_GO:0010159_specification+of+organ+position	0.533496	0.533496	#N/A
MF_GO:0005171_hepatocyte+growth+factor+receptor+binding	0.786099	0.786099	#N/A
MF_GO:0008510_sodium:bicarbonate+symporter+activity	0.522765	#N/A	0.522765
BP_GO:0006680_glucosylceramide+catabolic+process	0.960805	#N/A	0.960805
MF_GO:0033781_cholesterol+24-hydroxylase+activity	0.814372	0.814372	#N/A
MF_GO:0045437_uridine+nucleosidase+activity	0.751715	0.751715	#N/A
BP_GO:0045833_negative+regulation+of+lipid+metabolic+process	0.181285	0.181285	#N/A
BP_GO:0050996_positive+regulation+of+lipid+catabolic+process	0.181285	0.181285	#N/A
MF_GO:0008448_N-acetylglucosamine-6-phosphate+deacetylase+activity	0.295267	0.295267	#N/A
BP_GO:0006622_protein+targeting+to+lysosome	0.703536	#N/A	0.703536
BP_GO:0048278_vesicle+docking	0.906518	0.906518	#N/A
BP_GO:0030950_establishment+or+maintenance+of+actin+cytoskeleton+pc	0.230664	#N/A	0.230664
BP_GO:0045175_basal+protein+localization	0.230664	#N/A	0.230664
BP_GO:0035308_negative+regulation+of+protein+amino+acid+dephosphory	0.75077	#N/A	0.75077
BP_GO:0046604_positive+regulation+of+mitotic+centrosome+separation	0.921605	#N/A	0.921605
BP_GO:0016598_protein+arginylation	0.594243	0.594243	#N/A
MF_GO:0004057_arginyltransferase+activity	0.594243	0.594243	#N/A
MF_GO:0046525_xylosylprotein+4-beta-galactosyltransferase+activity	0.493225	0.493225	#N/A
MF_GO:0003952_NAD++synthase+(glutamine-hydrolyzing)+activity	0.880922	0.880922	#N/A
MF_GO:0008795_NAD++synthase+activity	0.880922	0.880922	#N/A
BP_GO:0006978_DNA+damage+response,+signal+transduction+by+p53+clas	0.805738	0.805738	#N/A
BP_GO:0033205_cytokinesis+during+cell+cycle	0.805738	0.805738	#N/A
MF_GO:0017061_S-methyl-5-thioadenosine+phosphorylase+activity	0.91218	0.91218	#N/A
BP_GO:0030422_RNA+interference,+production+of+siRNA	0.856692	#N/A	0.856692
BP_GO:0032507_maintenance+of+protein+location+in+cell	0.66341	#N/A	0.66341
CC_GO:0000932_cytoplasmic+mRNA+processing+body	0.66341	#N/A	0.66341
BP_GO:0007207_muscarinic+acetylcholine+receptor,+phospholipase+C+acti	0.752359	#N/A	0.752359
MF_GO:0016997_alpha-sialidase+activity	0.815601	#N/A	0.815601
MF_GO:0030274_LIM+domain+binding	0.502987	0.502987	#N/A
MF_GO:0004489_methylenetetrahydrofolate+reductase+(NADPH)+activity	0.694878	0.694878	#N/A
BP_GO:0009120_deoxyribonucleoside+metabolic+process	0.102975	0.102975	#N/A
BP_GO:0019047_provirus+integration	0.352131	0.352131	#N/A
MF_GO:0047874_dolichyldiphosphatase+activity	0.32482	0.32482	#N/A
BP_GO:0030220_platelet+formation	0.454732	0.454732	#N/A
BP_GO:0060155_platelet+dense+granule+organization	0.716457	0.716457	#N/A
BP_GO:0018191_peptidyl-serine+octanoylation	0.445878	0.445878	#N/A
MF_GO:0016412_serine+O-acyltransferase+activity	0.445878	0.445878	#N/A
BP_GO:0001754_eye+photoreceptor+cell+differentiation	0.74988	#N/A	0.74988
BP_GO:0002287_alpha-beta+T+cell+activation+during+immune+response	0.414912	#N/A	0.414912
BP_GO:0002476_antigen+processing+and+presentation+of+endogenous+pe	0.414912	#N/A	0.414912
BP_GO:0002477_antigen+processing+and+presentation+of+exogenous+pep	0.414912	#N/A	0.414912
CC_GO:0032398_MHC+class+Ib+protein+complex	0.414912	#N/A	0.414912
BP_GO:0021544_subpallium+development	0.198059	#N/A	0.198059
BP_GO:0014707_branchiomeric+skeletal+muscle+development	0.688089	#N/A	0.688089
BP_GO:0048625_myoblast+cell+fate+commitment	0.688089	#N/A	0.688089

BP_GO:0031943_regulation+of+glucocorticoid+metabolic+process	0.0618762	0.0618762	#N/A
CC_GO:0031371_ubiquitin+conjugating+enzyme+complex	0.0618762	0.0618762	#N/A
MF_GO:0030191_Hsp70/Hsc70+protein+inhibitor+activity	0.0618762	0.0618762	#N/A
MF_GO:0030911_TPR+domain+binding	0.0618762	0.0618762	#N/A
BP_GO:0018144_RNA-protein+covalent+cross-linking	0.990108	#N/A	0.990108
BP_GO:0042149_cellular+response+to+glucose+starvation	0.990108	#N/A	0.990108
BP_GO:0043153_entrainment+of+circadian+clock+by+photoperiod	0.057145	0.057145	#N/A
MF_GO:0005459_UDP-galactose+transmembrane+transporter+activity	0.193872	0.193872	#N/A
BP_GO:0031583_G-protein+signaling,+phospholipase+D+activating+pathwa	0.370184	#N/A	0.370184
MF_GO:0031707_endothelin+A+receptor+binding	0.370184	#N/A	0.370184
BP_GO:0005513_detection+of+calcium+ion	0.968321	0.968321	#N/A
BP_GO:0032781_positive+regulation+of+ATPase+activity	0.968321	0.968321	#N/A
MF_GO:0008242_omega+peptidase+activity	0.42664	#N/A	0.42664
MF_GO:0034722_gamma-glutamyl-peptidase+activity	0.42664	#N/A	0.42664
BP_GO:0042713_sperm+ejaculation	0.178859	#N/A	0.178859
BP_GO:0043084_penile+erection	0.178859	#N/A	0.178859
BP_GO:0050999_regulation+of+nitric-oxide+synthase+activity	0.178859	#N/A	0.178859
MF_GO:0048186_inhibin+beta-A+binding	0.178859	#N/A	0.178859
BP_GO:0021511_spinal+cord+patterning	0.995373	#N/A	0.995373
BP_GO:0051057_positive+regulation+of+small+GTPase+mediated+signal+tr	0.995373	#N/A	0.995373
BP_GO:0032388_positive+regulation+of+intracellular+transport	0.827057	0.827057	#N/A
MF_GO:0005047_signal+recognition+particle+binding	0.679941	#N/A	0.679941
BP_GO:0015739_sialic+acid+transport	0.94683	0.94683	#N/A
MF_GO:0015136_sialic+acid+transmembrane+transporter+activity	0.94683	0.94683	#N/A
BP_GO:0009056_catabolic+process	0.439591	#N/A	0.439591
BP_GO:0007057_spindle+assembly+involved+in+female+meiosis+I	0.736353	0.736353	#N/A
BP_GO:0046785_microtubule+polymerization	0.736353	0.736353	#N/A
BP_GO:0051442_negative+regulation+of+ubiquitin-protein+ligase+activity+	0.736353	0.736353	#N/A
BP_GO:0009062_fatty+acid+catabolic+process	0.1208	0.1208	#N/A
MF_GO:0016402_pristanoyl-CoA+oxidase+activity	0.417328	0.417328	#N/A
BP_GO:0021771_lateral+geniculate+nucleus+development	0.854831	0.854831	#N/A
BP_GO:0042320_regulation+of+circadian+sleep/wake+cycle,+REM+sleep	0.854831	0.854831	#N/A
BP_GO:0045188_regulation+of+circadian+sleep/wake+cycle,+non-REM+slee	0.854831	0.854831	#N/A
BP_GO:0045759_negative+regulation+of+action+potential	0.854831	0.854831	#N/A
BP_GO:0006421_asparaginyl-tRNA+aminoacylation	0.52308	#N/A	0.52308
BP_GO:0009589_detection+of+UV	0.510938	0.510938	#N/A
MF_GO:0004325_ferrochelatase+activity	0.510938	0.510938	#N/A
CC_GO:0000176_nuclear+exosome+(RNase+complex)	0.539417	0.539417	#N/A
BP_GO:0048319_axial+mesoderm+morphogenesis	0.477133	#N/A	0.477133
CC_GO:0019908_nuclear+cyclin-dependent+protein+kinase+holoenzyme+co	0.468938	#N/A	0.468938
BP_GO:0019310_inositol+catabolic+process	0.191894	0.191894	#N/A
MF_GO:0050113_inositol+oxygenase+activity	0.191894	0.191894	#N/A
BP_GO:0046850_regulation+of+bone+remodeling	0.852467	#N/A	0.852467
BP_GO:0046831_regulation+of+RNA+export+from+nucleus	0.331178	0.331178	#N/A
BP_GO:0015936_coenzyme+A+metabolic+process	0.456539	#N/A	0.456539
MF_GO:0004420_hydroxymethylglutaryl-CoA+reductase+(NADPH)+activity	0.456539	#N/A	0.456539
BP_GO:0021920_regulation+of+transcription+from+RNA+polymerase+II+pro	0.0412294	0.0412294	#N/A

MF_GO:0030229_very-low-density+lipoprotein+receptor+activity	0.97651	0.97651	#N/A
MF_GO:0005008_hepatocyte+growth+factor+receptor+activity	0.470338	#N/A	0.470338
MF_GO:0004138_deoxyguanosine+kinase+activity	0.12135	0.12135	#N/A
BP_GO:0000917_barrier+septum+formation	0.307133	0.307133	#N/A
BP_GO:0001762_beta-alanine+transport	0.440019	0.440019	#N/A
BP_GO:0015734_taurine+transport	0.440019	0.440019	#N/A
MF_GO:0001761_beta-alanine+transmembrane+transporter+activity	0.440019	0.440019	#N/A
MF_GO:0005368_taurine+transmembrane+transporter+activity	0.440019	0.440019	#N/A
MF_GO:0005369_taurine:sodium+symporter+activity	0.440019	0.440019	#N/A
BP_GO:0042534_regulation+of+tumor+necrosis+factor+biosynthetic+proces	0.751655	#N/A	0.751655
CC_GO:0031234_extrinsic+to+internal+side+of+plasma+membrane	0.368521	#N/A	0.368521
MF_GO:0005127_ciliary+neurotrophic+factor+receptor+binding	0.800427	0.800427	#N/A
BP_GO:0042745_circadian+sleep/wake+cycle	0.441589	0.441589	#N/A
MF_GO:0004853_uroporphyrinogen+decarboxylase+activity	0.2005	0.2005	#N/A
BP_GO:0032968_positive+regulation+of+RNA+elongation+from+RNA+polym	0.838767	0.838767	#N/A
CC_GO:0045252_oxoglutarate+dehydrogenase+complex	0.464637	0.464637	#N/A
MF_GO:0004149_dihydrolipoyllysine-residue+succinyltransferase+activity	0.464637	0.464637	#N/A
MF_GO:0051435_BH4+domain+binding	0.301289	0.301289	#N/A
BP_GO:0042990_regulation+of+transcription+factor+import+into+nucleus	0.566274	#N/A	0.566274
BP_GO:0048822_enucleate+erythrocyte+development	0.378495	0.378495	#N/A
MF_GO:0004587_ornithine-oxo-acid+transaminase+activity	0.327806	0.327806	#N/A
MF_GO:0005219_ryanodine-sensitive+calcium-release+channel+activity	0.896659	0.896659	#N/A
MF_GO:0050333_thiamin-triphosphatase+activity	0.233553	0.233553	#N/A
CC_GO:0030482_actin+cable	0.594782	0.594782	#N/A
BP_GO:0035026_leading+edge+cell+differentiation	0.277794	0.277794	#N/A
BP_GO:0051223_regulation+of+protein+transport	0.609448	#N/A	0.609448
MF_GO:0032794_GTPase+activating+protein+binding	0.609448	#N/A	0.609448
BP_GO:0000018_regulation+of+DNA+recombination	0.327087	#N/A	0.327087
MF_GO:0030629_U6+snRNA+3'-end+binding	0.953561	#N/A	0.953561
MF_GO:0008387_steroid+7-alpha-hydroxylase+activity	0.371547	#N/A	0.371547
MF_GO:0033782_24-hydroxycholesterol+7alpha-hydroxylase+activity	0.371547	#N/A	0.371547
MF_GO:0004988_mu-opioid+receptor+activity	0.235798	#N/A	0.235798
MF_GO:0016941_natriuretic+peptide+receptor+activity	0.234856	#N/A	0.234856
BP_GO:0016246_RNA+interference	0.479426	0.479426	#N/A
CC_GO:0016442_RNA-induced+silencing+complex	0.479426	0.479426	#N/A
MF_GO:0032556_pyrimidine+deoxyribonucleotide+binding	0.0295899	0.0295899	#N/A
BP_GO:0018916_nitrobenzene+metabolic+process	0.909454	0.909454	#N/A
BP_GO:0042178_xenobiotic+catabolic+process	0.909454	0.909454	#N/A
BP_GO:0046483_heterocycle+metabolic+process	0.829633	0.829633	#N/A
MF_GO:0004070_aspartate+carbamoyltransferase+activity	0.281728	0.281728	#N/A
MF_GO:0003842_1-pyrroline-5-carboxylate+dehydrogenase+activity	0.0847603	0.0847603	#N/A
MF_GO:0004418_hydroxymethylbilane+synthase+activity	0.82034	#N/A	0.82034
BP_GO:0045768_positive+regulation+of+anti-apoptosis	0.179871	0.179871	#N/A
BP_GO:0046898_response+to+cycloheximide	0.179871	0.179871	#N/A
CC_GO:0044429_mitochondrial+part	0.179871	0.179871	#N/A
MF_GO:0050290_sphingomyelin+phosphodiesterase+D+activity	0.780229	#N/A	0.780229
BP_GO:0001949_sebaceous+gland+cell+differentiation	0.444684	0.444684	#N/A

MF_GO:0043140_ATP-dependent+3'-5'+DNA+helicase+activity	0.697611	#N/A	0.697611
BP_GO:0019725_cellular+homeostasis	0.802428	0.802428	#N/A
BP_GO:0048871_multicellular+organismal+homeostasis	0.802428	0.802428	#N/A
MF_GO:0008330_protein+tyrosine/threonine+phosphatase+activity	0.584072	#N/A	0.584072
MF_GO:0008579_JUN+kinase+phosphatase+activity	0.584072	#N/A	0.584072
MF_GO:0004491_methylmalonate-semialdehyde+dehydrogenase+(acylating	0.3488	0.3488	#N/A
MF_GO:0018478_malonate-semialdehyde+dehydrogenase+(acetylating)+ac	0.3488	0.3488	#N/A
MF_GO:0017049_GTP-Rho+binding	0.746166	#N/A	0.746166
BP_GO:0006933_negative+regulation+of+cell+adhesion+involved+in+substra	0.267338	0.267338	#N/A
BP_GO:0051649_establishment+of+localization+in+cell	0.874201	0.874201	#N/A
CC_GO:0031226_intrinsic+to+plasma+membrane	0.874201	0.874201	#N/A
MF_GO:0043495_protein+anchor	0.874201	0.874201	#N/A
MF_GO:0015559_multidrug+efflux+pump+activity	0.340079	#N/A	0.340079
BP_GO:0006383_transcription+from+RNA+polymerase+III+promoter	0.08465	0.08465	#N/A
CC_GO:0005969_serine-pyruvate+aminotransferase+complex	0.767491	#N/A	0.767491
BP_GO:0032066_nucleolus+to+nucleoplasm+transport	0.312038	0.312038	#N/A
BP_GO:0019102_male+somatic+sex+determination	0.204996	#N/A	0.204996
MF_GO:0004882_androgen+receptor+activity	0.204996	#N/A	0.204996
BP_GO:0006682_galactosylceramide+biosynthetic+process	0.597004	#N/A	0.597004
BP_GO:0032275_luteinizing+hormone+secretion	0.506723	#N/A	0.506723
BP_GO:0032870_cellular+response+to+hormone+stimulus	0.506723	#N/A	0.506723
BP_GO:0046884_follicle-stimulating+hormone+secretion	0.506723	#N/A	0.506723
MF_GO:0030562_rRNA+2'-O-ribose+methylation+guide+activity	0.817664	#N/A	0.817664
MF_GO:0003860_3-hydroxyisobutyryl-CoA+hydrolase+activity	0.936463	#N/A	0.936463
BP_GO:0045927_positive+regulation+of+growth	0.202788	0.202788	#N/A
MF_GO:0016565_general+transcriptional+repressor+activity	0.202788	0.202788	#N/A
BP_GO:0006231_dTMP+biosynthetic+process	0.189481	0.189481	#N/A
MF_GO:0004799_thymidylate+synthase+activity	0.189481	0.189481	#N/A
BP_GO:0042789_mRNA+transcription+from+RNA+polymerase+II+promoter	0.270117	0.270117	#N/A
BP_GO:0006496_protein+amino+acid+terminal+N-glycosylation	0.432253	#N/A	0.432253
BP_GO:0051270_regulation+of+cell+motion	0.432253	#N/A	0.432253
BP_GO:0060046_regulation+of+acrosome+reaction	0.432253	#N/A	0.432253
BP_GO:0060054_positive+regulation+of+epithelial+cell+proliferation+involv	0.432253	#N/A	0.432253
MF_GO:0016433_rRNA+(adenine)+methyltransferase+activity	0.301317	#N/A	0.301317
BP_GO:0042273_ribosomal+large+subunit+biogenesis	0.108041	0.108041	#N/A
MF_GO:0047395_glycerophosphoinositol+glycerophosphodiesterase+activit	0.766553	#N/A	0.766553
MF_GO:0047115_trans-1,2-dihydrobenzene-1,2-diol+dehydrogenase+activit	0.872788	0.872788	#N/A
MF_GO:0047837_D-xylose+1-dehydrogenase+(NADP+)+activity	0.872788	0.872788	#N/A
MF_GO:0016519_gastric+inhibitory+peptide+receptor+activity	0.672629	#N/A	0.672629
BP_GO:0000105_histidine+biosynthetic+process	0.544922	0.544922	#N/A
CC_GO:0005847_mRNA+cleavage+and+polyadenylation+specificity+factor+c	0.811777	#N/A	0.811777
BP_GO:0006669_sphinganine-1-phosphate+biosynthetic+process	0.571067	0.571067	#N/A
MF_GO:0017050_D-erythro-sphingosine+kinase+activity	0.571067	0.571067	#N/A
BP_GO:0017085_response+to+insecticide	0.411562	#N/A	0.411562
BP_GO:0050684_regulation+of+mRNA+processing	0.242574	#N/A	0.242574
BP_GO:0006450_regulation+of+translational+fidelity	0.157316	#N/A	0.157316
BP_GO:0002071_glandular+epithelial+cell+maturation	0.653632	0.653632	#N/A

MF_GO:0008780_acyl-[acyl-carrier-protein]-UDP-N-acetylglucosamine+O-ac	0.653632	0.653632	#N/A
BP_GO:0007500_mesodermal+cell+fate+determination	0.979292	0.979292	#N/A
CC_GO:0005623_cell	0.27498	0.27498	#N/A
BP_GO:0019543_propionate+catabolic+process	0.384836	0.384836	#N/A
MF_GO:0047547_2-methylcitrate+dehydratase+activity	0.384836	0.384836	#N/A
BP_GO:0048219_inter-Golgi+cisterna+vesicle-mediated+transport	0.491811	#N/A	0.491811
BP_GO:0034504_protein+localization+in+nucleus	0.655893	0.655893	#N/A
BP_GO:0019695_choline+metabolic+process	0.926769	#N/A	0.926769
BP_GO:0017187_peptidyl-glutamic+acid+carboxylation	0.743874	#N/A	0.743874
MF_GO:0008488_gamma-glutamyl+carboxylase+activity	0.743874	#N/A	0.743874
BP_GO:0002017_regulation+of+blood+volume+by+renal+aldosterone	0.979217	#N/A	0.979217
BP_GO:0032342_aldosterone+biosynthetic+process	0.979217	#N/A	0.979217
MF_GO:0004507_steroid+11-beta-monooxygenase+activity	0.979217	#N/A	0.979217
CC_GO:0030122_AP-2+adaptor+complex	0.0284991	0.0284991	#N/A
BP_GO:0009236_cobalamin+biosynthetic+process	0.0450841	0.0450841	#N/A
MF_GO:0008817_cob(I)yrinic+acid+a,c-diamide+adenosyltransferase+activit	0.0450841	0.0450841	#N/A
MF_GO:0005129_granulocyte+macrophage+colony-stimulating+factor+rece	0.465366	#N/A	0.465366
BP_GO:0046471_phosphatidylglycerol+metabolic+process	0.422375	0.422375	#N/A
MF_GO:0017171_serine+hydrolase+activity	0.283219	0.283219	#N/A
BP_GO:0035090_maintenance+of+apical/basal+cell+polarity	0.0777914	0.0777914	#N/A
BP_GO:0009794_regulation+of+mitotic+cell+cycle,+embryonic	0.18972	0.18972	#N/A
MF_GO:0017080_sodium+channel+regulator+activity	0.721554	#N/A	0.721554
MF_GO:0004438_phosphatidylinositol-3-phosphatase+activity	0.608338	#N/A	0.608338
BP_GO:0034505_tooth+mineralization	0.684009	#N/A	0.684009
MF_GO:0043498_cell+surface+binding	0.684009	#N/A	0.684009
MF_GO:0046848_hydroxyapatite+binding	0.684009	#N/A	0.684009
BP_GO:0009113_purine+base+biosynthetic+process	0.826291	#N/A	0.826291
MF_GO:0004641_phosphoribosylformylglycinamidine+cyclo-ligase+activity	0.826291	#N/A	0.826291
MF_GO:0004644_phosphoribosylglycinamide+formyltransferase+activity	0.826291	#N/A	0.826291
BP_GO:0031946_regulation+of+glucocorticoid+biosynthetic+process	0.623932	#N/A	0.623932
BP_GO:0042921_glucocorticoid+receptor+signaling+pathway	0.623932	#N/A	0.623932
MF_GO:0004883_glucocorticoid+receptor+activity	0.623932	#N/A	0.623932
BP_GO:0019100_male+germ-line+sex+determination	0.893654	0.893654	#N/A
BP_GO:0060350_endochondral+bone+morphogenesis	0.893654	0.893654	#N/A
CC_GO:0042564_NLS-dependent+protein+nuclear+import+complex	0.207521	0.207521	#N/A
BP_GO:0032206_positive+regulation+of+telomere+maintenance	0.679095	#N/A	0.679095
BP_GO:0002121_inter-male+aggressive+behavior	0.522055	#N/A	0.522055
BP_GO:0002124_territorial+aggressive+behavior	0.522055	#N/A	0.522055
BP_GO:0008050_female+courtship+behavior	0.522055	#N/A	0.522055
BP_GO:0048047_mating+behavior,+sex+discrimination	0.522055	#N/A	0.522055
CC_GO:0032590_dendrite+membrane	0.522055	#N/A	0.522055
BP_GO:0009257_10-formyltetrahydrofolate+biosynthetic+process	0.136676	#N/A	0.136676
BP_GO:0030948_negative+regulation+of+vascular+endothelial+growth+fact	0.131546	#N/A	0.131546
MF_GO:0004798_thymidylate+kinase+activity	0.788521	#N/A	0.788521
BP_GO:0030320_cellular+monovalent+inorganic+anion+homeostasis	0.137259	#N/A	0.137259
BP_GO:0051044_positive+regulation+of+membrane+protein+ectodomain+p	0.512601	#N/A	0.512601
MF_GO:0003975_UDP-N-acetylglucosamine-dolichyl-phosphate+N-acetylglu	0.750977	#N/A	0.750977

MF_GO:0008963_phospho-N-acetylmuramoyl-pentapeptide-transferase+act	0.750977	#N/A	0.750977
BP_GO:0030035_microspike+assembly	0.403637	#N/A	0.403637
BP_GO:0045623_negative+regulation+of+T-helper+cell+differentiation	0.442476	#N/A	0.442476
BP_GO:0051896_regulation+of+protein+kinase+B+signaling+cascade	0.520277	#N/A	0.520277
BP_GO:0031664_regulation+of+lipopolysaccharide-mediated+signaling+path	0.563295	#N/A	0.563295
BP_GO:0001941_postsynaptic+membrane+organization	0.985008	0.985008	#N/A
MF_GO:0004148_dihydrolipoyl+dehydrogenase+activity	0.847411	0.847411	#N/A
BP_GO:0006348_chromatin+silencing+at+telomere	0.0208835	#N/A	0.0208835
BP_GO:0042073_intraflagellar+transport	0.393407	#N/A	0.393407
MF_GO:0008808_cardiolipin+synthase+activity	0.393407	#N/A	0.393407
MF_GO:0005412_glucose:sodium+symporter+activity	0.138786	#N/A	0.138786
BP_GO:0046822_regulation+of+nucleocytoplasmic+transport	0.0818516	#N/A	0.0818516
BP_GO:0051444_negative+regulation+of+ubiquitin-protein+ligase+activity	0.0818516	#N/A	0.0818516
MF_GO:0055105_ubiquitin-protein+ligase+inhibitor+activity	0.0818516	#N/A	0.0818516
MF_GO:0015019_heparan-alpha-glucosaminide+N-acetyltransferase+activity	0.767393	#N/A	0.767393
MF_GO:0043425_bHLH+transcription+factor+binding	0.045927	0.045927	#N/A
CC_GO:0001650_fibrillar+center	0.105872	0.105872	#N/A
MF_GO:0015051_X-opioid+receptor+activity	0.482974	0.482974	#N/A
BP_GO:0001555_oocyte+growth	0.260585	#N/A	0.260585
BP_GO:0032212_positive+regulation+of+telomere+maintenance+via+telome	0.451944	0.451944	#N/A
CC_GO:0005697_telomerase+holoenzyme+complex	0.451944	0.451944	#N/A
MF_GO:0000332_template+for+synthesis+of+G-rich+strand+of+telomere+D	0.451944	0.451944	#N/A
BP_GO:0048845_venous+blood+vessel+morphogenesis	0.596818	0.596818	#N/A
BP_GO:0000379_tRNA-type+intron+splice+site+recognition+and+cleavage	0.0109889	0.0109889	#N/A
BP_GO:0014813_satellite+cell+commitment	0.357611	0.357611	#N/A
BP_GO:0007197_muscarinic+acetylcholine+receptor,+adenylate+cyclase+inh	0.927868	0.927868	#N/A
BP_GO:0048627_myoblast+development	0.267729	#N/A	0.267729
MF_GO:0030942_endoplasmic+reticulum+signal+peptide+binding	0.107603	0.107603	#N/A
BP_GO:0008593_regulation+of+Notch+signaling+pathway	0.471034	#N/A	0.471034
BP_GO:0007158_neuron+adhesion	0.535338	#N/A	0.535338
CC_GO:0045180_basal+cortex	0.840739	#N/A	0.840739
BP_GO:0032808_lacrimal+gland+development	0.392262	#N/A	0.392262
MF_GO:0032810_sterol+response+element+binding	0.0174381	0.0174381	#N/A
MF_GO:0008418_protein+N-terminal+asparagine+amidohydrolase+activity	0.450789	0.450789	#N/A
MF_GO:0015054_gastrin+receptor+activity	0.61668	#N/A	0.61668
MF_GO:0005142_interleukin-11+receptor+binding	0.621793	0.621793	#N/A
MF_GO:0004951_cholecystokinin+receptor+activity	0.478102	#N/A	0.478102
CC_GO:0002111_BRCA2-BRAF35+complex	0.607373	0.607373	#N/A
MF_GO:0000400_four-way+junction+DNA+binding	0.607373	0.607373	#N/A
BP_GO:0046619_optic+placode+formation+involved+in+camera-type+eye	0.315264	0.315264	#N/A
BP_GO:0003097_renal+water+transport	0.964467	#N/A	0.964467
BP_GO:0042631_cellular+response+to+water+deprivation	0.964467	#N/A	0.964467
CC_GO:0031303_integral+to+endosome+membrane	0.964467	#N/A	0.964467
CC_GO:0055038_recycling+endosome+membrane	0.964467	#N/A	0.964467
MF_GO:0033871_[heparan+sulfate]-glucosamine+3-sulfotransferase+2+activ	0.332583	#N/A	0.332583
MF_GO:0019135_deoxyhypusine+monooxygenase+activity	0.0397194	0.0397194	#N/A
BP_GO:0002903_negative+regulation+of+B+cell+apoptosis	0.96492	#N/A	0.96492

BP_GO:0051935_glutamate+uptake+during+transmission+of+nerve+impulse	0.767746	0.767746	#N/A
BP_GO:0006798_polyphosphate+catabolic+process	0.973877	#N/A	0.973877
MF_GO:0015926_glucosidase+activity	0.884767	0.884767	#N/A
MF_GO:0033919_glucan+1,3-alpha-glucosidase+activity	0.884767	0.884767	#N/A
MF_GO:0005087_Ran+guanyl-nucleotide+exchange+factor+activity	0.684507	#N/A	0.684507
MF_GO:0004819_glutamine-tRNA+ligase+activity	0.392916	0.392916	#N/A
BP_GO:0015855_pyrimidine+transport	0.728958	0.728958	#N/A
MF_GO:0005350_pyrimidine+transmembrane+transporter+activity	0.728958	0.728958	#N/A
MF_GO:0016455_RNA+polymerase+II+transcription+mediator+activity	0.700996	0.700996	#N/A
BP_GO:0000291_nuclear-transcribed+mRNA+catabolic+process,+exonucleol	0.845013	#N/A	0.845013
BP_GO:0050773_regulation+of+dendrite+development	0.512008	#N/A	0.512008
MF_GO:0030116_glial+cell+line-derived+neurotrophic+factor+receptor+binc	0.439166	#N/A	0.439166
BP_GO:0043654_recognition+of+apoptotic+cell	0.125969	0.125969	#N/A
MF_GO:0043014_alpha-tubulin+binding	0.711417	#N/A	0.711417
BP_GO:0007024_alpha-tubulin+folding	0.44899	0.44899	#N/A
BP_GO:0014889_muscle+atrophy	0.44899	0.44899	#N/A
BP_GO:0048936_peripheral+nervous+system+neuron+axonogenesis	0.44899	0.44899	#N/A
BP_GO:0048137_spermatocyte+division	0.591101	0.591101	#N/A
BP_GO:0045046_protein+import+into+peroxisome+membrane	0.337017	#N/A	0.337017
MF_GO:0004530_deoxyribonuclease+I+activity	0.258998	#N/A	0.258998
BP_GO:0009628_response+to+abiotic+stimulus	0.879447	#N/A	0.879447
MF_GO:0047290_(alpha-N-acetylneuraminyl-2,3-beta-galactosyl-1,3)-N-acet	0.606848	0.606848	#N/A
MF_GO:0047677_arachidonate+8-lipoxygenase+activity	0.457634	#N/A	0.457634
MF_GO:0050473_arachidonate+15-lipoxygenase+activity	0.457634	#N/A	0.457634
BP_GO:0018343_protein+farnesylation	0.943429	#N/A	0.943429
BP_GO:0030823_regulation+of+cGMP+metabolic+process	0.943429	#N/A	0.943429
MF_GO:0001918_farnesylated+protein+binding	0.943429	#N/A	0.943429
BP_GO:0048246_macrophage+chemotaxis	0.725876	#N/A	0.725876
MF_GO:0031727_CCR2+chemokine+receptor+binding	0.725876	#N/A	0.725876
BP_GO:0042706_eye+photoreceptor+cell+fate+commitment	0.936995	0.936995	#N/A
MF_GO:0004852_uroporphyrinogen-III+synthase+activity	0.0792472	0.0792472	#N/A
MF_GO:0004728_receptor+signaling+protein+tyrosine+phosphatase+activity	0.749686	0.749686	#N/A
BP_GO:0006463_steroid+hormone+receptor+complex+assembly	0.254798	0.254798	#N/A
BP_GO:0046661_male+sex+differentiation	0.254798	0.254798	#N/A
MF_GO:0035259_glucocorticoid+receptor+binding	0.254798	0.254798	#N/A
BP_GO:0006551_leucine+metabolic+process	0.172319	0.172319	#N/A
BP_GO:0035148_lumen+formation	0.246873	0.246873	#N/A
MF_GO:0015106_bicarbonate+transmembrane+transporter+activity	0.99787	#N/A	0.99787
MF_GO:0015301_anion:anion+antiporter+activity	0.99787	#N/A	0.99787
MF_GO:0008139_nuclear+localization+sequence+binding	0.456131	0.456131	#N/A
MF_GO:0004980_melanocyte+stimulating+hormone+receptor+activity	0.401764	#N/A	0.401764
BP_GO:0043316_cytotoxic+T+cell+degranulation	0.625341	#N/A	0.625341
MF_GO:0000156_two-component+response+regulator+activity	0.166682	#N/A	0.166682
BP_GO:0009996_negative+regulation+of+cell+fate+specification	0.337135	#N/A	0.337135
BP_GO:0042660_positive+regulation+of+cell+fate+specification	0.337135	#N/A	0.337135
BP_GO:0021873_forebrain+neuroblast+division	0.594503	0.594503	#N/A
BP_GO:0045769_negative+regulation+of+asymmetric+cell+division	0.594503	0.594503	#N/A

BP_GO:0051661_maintenance+of+centrosome+location	0.594503	0.594503	#N/A
MF_GO:0016501_prostacyclin+receptor+activity	0.794265	0.794265	#N/A
MF_GO:0016709_oxidoreductase+activity,+acting+on+paired+donors,+with-	0.026948	0.026948	#N/A
BP_GO:0045780_positive+regulation+of+bone+resorption	0.630224	#N/A	0.630224
CC_GO:0030532_small+nuclear+ribonucleoprotein+complex	0.346692	0.346692	#N/A
BP_GO:0032525_somite+rostral/caudal+axis+specification	0.728682	#N/A	0.728682
BP_GO:0006576_biogenic+amine+metabolic+process	0.423758	0.423758	#N/A
BP_GO:0009405_pathogenesis	0.423758	0.423758	#N/A
BP_GO:0031061_negative+regulation+of+histone+methylation	0.423758	0.423758	#N/A
BP_GO:0032048_cardiolipin+metabolic+process	0.423758	0.423758	#N/A
BP_GO:0040029_regulation+of+gene+expression,+epigenetic	0.423758	0.423758	#N/A
MF_GO:0010385_double-stranded+methylated+DNA+binding	0.423758	0.423758	#N/A
BP_GO:0009214_cyclic+nucleotide+catabolic+process	0.0537241	0.0537241	#N/A
MF_GO:0004113_2',3'-cyclic-nucleotide+3'-phosphodiesterase+activity	0.0537241	0.0537241	#N/A
CC_GO:0045259_proton-transporting+ATP+synthase+complex	0.203073	0.203073	#N/A
CC_GO:0030868_smooth+endoplasmic+reticulum+membrane	0.610891	#N/A	0.610891
MF_GO:0005042_netrin+receptor+activity	0.932935	#N/A	0.932935
BP_GO:0051584_regulation+of+dopamine+uptake	0.780458	#N/A	0.780458
BP_GO:0051586_positive+regulation+of+dopamine+uptake	0.780458	#N/A	0.780458
MF_GO:0001591_dopamine+receptor+coupled+via+Gi/Go	0.780458	#N/A	0.780458
MF_GO:0001670_dopamine+D2+receptor+activity	0.780458	#N/A	0.780458
BP_GO:0006278_RNA-dependent+DNA+replication	0.475723	#N/A	0.475723
MF_GO:0003721_telomeric+template+RNA+reverse+transcriptase+activity	0.475723	#N/A	0.475723
BP_GO:0016340_calcium-dependent+cell-matrix+adhesion	0.244218	#N/A	0.244218
BP_GO:0008216_spermidine+metabolic+process	0.744963	#N/A	0.744963
BP_GO:0042628_mating+plug+formation	0.896208	#N/A	0.896208
BP_GO:0043480_pigment+accumulation+in+tissues	0.351296	#N/A	0.351296
BP_GO:0045113_regulation+of+integrin+biosynthetic+process	0.494972	#N/A	0.494972
CC_GO:0005674_transcription+factor+TFIIF+complex	0.0155502	0.0155502	#N/A
BP_GO:0047497_mitochondrion+transport+along+microtubule	0.914199	#N/A	0.914199
MF_GO:0019784_NEDD8-specific+protease+activity	0.69483	0.69483	#N/A
MF_GO:0017162_aryl+hydrocarbon+receptor+binding	0.486507	#N/A	0.486507
BP_GO:0016239_positive+regulation+of+macroautophagy	0.295658	0.295658	#N/A
MF_GO:0032403_protein+complex+binding	0.591676	0.591676	#N/A
BP_GO:0060349_bone+morphogenesis	0.902408	#N/A	0.902408
BP_GO:0006873_cellular+ion+homeostasis	0.483466	#N/A	0.483466
BP_GO:0030007_cellular+potassium+ion+homeostasis	0.483466	#N/A	0.483466
BP_GO:0006307_DNA+dealkylation	0.540936	0.540936	#N/A
MF_GO:0003908_methylated-DNA-[protein]-cysteine+S-methyltransferase+	0.540936	0.540936	#N/A
BP_GO:0016264_gap+junction+assembly	0.702467	0.702467	#N/A
BP_GO:0007400_neuroblast+fate+determination	0.987914	0.987914	#N/A
BP_GO:0021779_oligodendrocyte+cell+fate+commitment	0.987914	0.987914	#N/A
BP_GO:0060165_regulation+of+timing+of+subpallium+neuron+differentiatic	0.987914	0.987914	#N/A
MF_GO:0004484_mRNA+guanylyltransferase+activity	0.0261285	#N/A	0.0261285
MF_GO:0004651_polynucleotide+5'-phosphatase+activity	0.0261285	#N/A	0.0261285
MF_GO:0003844_1,4-alpha-glucan+branching+enzyme+activity	0.796877	#N/A	0.796877
BP_GO:0007495_visceral+mesoderm-endoderm+interaction+involved+in+m	0.855019	#N/A	0.855019

BP_GO:0021794_thalamus+development	0.0499359	#N/A	0.0499359
BP_GO:0009992_cellular+water+homeostasis	0.795813	#N/A	0.795813
BP_GO:0019858_cytosine+metabolic+process	0.795914	#N/A	0.795914
MF_GO:0016908_MAP+kinase+2+activity	0.795914	#N/A	0.795914
BP_GO:0000185_activation+of+MAPKKK+activity	0.0226547	0.0226547	#N/A
BP_GO:0070207_protein+homotrimerization	0.474582	#N/A	0.474582
MF_GO:0004081_bis(5'-nucleosyl)-tetraphosphatase+(asymmetrical)+activit	0.412222	0.412222	#N/A
MF_GO:0008796_bis(5'-nucleosyl)-tetraphosphatase+activity	0.412222	0.412222	#N/A
BP_GO:0010667_negative+regulation+of+cardiac+muscle+cell+apoptosis	0.679948	0.679948	#N/A
BP_GO:0010735_positive+regulation+of+transcription+via+serum+response	0.679948	0.679948	#N/A
BP_GO:0051929_positive+regulation+of+calcium+ion+transport+via+voltage	0.679948	0.679948	#N/A
BP_GO:0055117_regulation+of+cardiac+muscle+contraction	0.679948	0.679948	#N/A
BP_GO:0060261_positive+regulation+of+transcription+initiation+from+RNA	0.679948	0.679948	#N/A
MF_GO:0010736_serum+response+element+binding	0.679948	0.679948	#N/A
BP_GO:0046878_positive+regulation+of+saliva+secretion	0.489257	#N/A	0.489257
BP_GO:0043568_positive+regulation+of+insulin-like+growth+factor+recepto	0.330475	#N/A	0.330475
BP_GO:0021692_cerebellar+Purkinje+cell+layer+morphogenesis	0.984256	#N/A	0.984256
BP_GO:0045299_otolith+mineralization	0.984256	#N/A	0.984256
BP_GO:0051928_positive+regulation+of+calcium+ion+transport	0.984256	#N/A	0.984256
MF_GO:0015085_calcium+ion+transmembrane+transporter+activity	0.984256	#N/A	0.984256
MF_GO:0030899_calcium-dependent+ATPase+activity	0.984256	#N/A	0.984256
CC_GO:0016342_catenin+complex	0.394672	#N/A	0.394672
BP_GO:0002335_mature+B+cell+differentiation	0.833722	0.833722	#N/A
BP_GO:0002380_immunoglobulin+secretion+during+immune+response	0.833722	0.833722	#N/A
BP_GO:0060122_inner+ear+receptor+stereocilium+organization	0.526074	#N/A	0.526074
MF_GO:0034617_tetrahydrobiopterin+binding	0.43042	0.43042	#N/A
MF_GO:0034618_arginine+binding	0.43042	0.43042	#N/A
CC_GO:0005683_snRNP+U7	0.648252	0.648252	#N/A
MF_GO:0005471_ATP:ADP+antiporter+activity	0.115621	#N/A	0.115621
BP_GO:0045065_cytotoxic+T+cell+differentiation	0.776894	#N/A	0.776894
MF_GO:0001593_dopamine+D4+receptor+activity	0.203137	#N/A	0.203137
BP_GO:0002355_detection+of+tumor+cell	0.244928	#N/A	0.244928
BP_GO:0002860_positive+regulation+of+natural+killer+cell+mediated+cytot	0.244928	#N/A	0.244928
BP_GO:0002098_tRNA+wobble+uridine+modification	0.614757	#N/A	0.614757
MF_GO:0004730_pseudouridylate+synthase+activity	0.0970602	#N/A	0.0970602
BP_GO:0060161_positive+regulation+of+dopamine+receptor+signaling+path	0.814936	0.814936	#N/A
MF_GO:0001592_dopamine+D3+receptor+activity	0.814936	0.814936	#N/A
CC_GO:0032444_activin+responsive+factor+complex	0.691229	0.691229	#N/A
BP_GO:0043587_tongue+morphogenesis	0.962996	0.962996	#N/A
BP_GO:0042752_regulation+of+circadian+rhythm	0.509399	0.509399	#N/A
BP_GO:0051151_negative+regulation+of+smooth+muscle+cell+differentiatio	0.518764	0.518764	#N/A
MF_GO:0003881_CDP-diacylglycerol-inositol+3-phosphatidyltransferase+act	0.376972	0.376972	#N/A
CC_GO:0033268_node+of+Ranvier	0.413117	#N/A	0.413117
CC_GO:0043194_initial+segment	0.413117	#N/A	0.413117
BP_GO:0001988_positive+regulation+of+heart+rate+in+baroreceptor+respo	0.659575	#N/A	0.659575
BP_GO:0014061_regulation+of+norepinephrine+secretion	0.659575	#N/A	0.659575
BP_GO:0032691_negative+regulation+of+interleukin-1+beta+production	0.659575	#N/A	0.659575

BP_GO:0060112_generation+of+ovulation+cycle+rhythm	0.659575	#N/A	0.659575
CC_GO:0043203_axon+hillock	0.121645	#N/A	0.121645
BP_GO:0030299_cholesterol+absorption	0.415721	#N/A	0.415721
BP_GO:0051977_lysophospholipid+transport	0.844815	#N/A	0.844815
BP_GO:0042059_negative+regulation+of+epidermal+growth+factor+recepto	0.305513	0.305513	#N/A
MF_GO:0051425_PTB+domain+binding	0.305513	0.305513	#N/A
BP_GO:0007621_negative+regulation+of+female+receptivity	0.488351	#N/A	0.488351
BP_GO:0030157_pancreatic+juice+secretion	0.71813	#N/A	0.71813
MF_GO:0004380_glycoprotein-fucosylgalactoside+alpha-N-acetylgalactosam	0.296847	#N/A	0.296847
MF_GO:0004381_fucosylgalactoside+3-alpha-galactosyltransferase+activity	0.296847	#N/A	0.296847
CC_GO:0005861_troponin+complex	0.0899571	0.0899571	#N/A
MF_GO:0048256_flap+endonuclease+activity	0.631719	0.631719	#N/A
MF_GO:0050178_phenylpyruvate+tautomerase+activity	0.281174	0.281174	#N/A
BP_GO:0006473_protein+amino+acid+acetylation	0.296085	#N/A	0.296085
MF_GO:0005221_intracellular+cyclic+nucleotide+activated+cation+channel+	0.563713	0.563713	#N/A
BP_GO:0042271_susceptibility+to+natural+killer+cell+mediated+cytotoxicity	0.590326	#N/A	0.590326
BP_GO:0045058_T+cell+selection	0.594952	#N/A	0.594952
MF_GO:0008437_thyrotropin-releasing+hormone+activity	0.986241	#N/A	0.986241
MF_GO:0004498_calcidiol+1-monooxygenase+activity	0.746879	#N/A	0.746879
BP_GO:0010455_positive+regulation+of+cell+fate+commitment	0.910921	#N/A	0.910921
BP_GO:0045187_regulation+of+circadian+sleep/wake+cycle,+sleep	0.66146	#N/A	0.66146
BP_GO:0048387_negative+regulation+of+retinoic+acid+receptor+signaling+	0.552706	#N/A	0.552706
BP_GO:0008057_eye+pigment+granule+organization	0.419996	0.419996	#N/A
BP_GO:0030835_negative+regulation+of+actin+filament+depolymerization	0.419996	0.419996	#N/A
BP_GO:0032401_establishment+of+melanosome+localization	0.419996	0.419996	#N/A
BP_GO:0043482_cellular+pigment+accumulation	0.419996	0.419996	#N/A
BP_GO:0042664_negative+regulation+of+endodermal+cell+fate+specificatio	0.626639	#N/A	0.626639
BP_GO:0031536_positive+regulation+of+exit+from+mitosis	0.82659	#N/A	0.82659
BP_GO:0015811_L-cystine+transport	0.577019	#N/A	0.577019
MF_GO:0015184_L-cystine+transmembrane+transporter+activity	0.577019	#N/A	0.577019
BP_GO:0001510_RNA+methylation	0.586122	0.586122	#N/A
MF_GO:0001734_mRNA+(N6-adenosine)-methyltransferase+activity	0.586122	0.586122	#N/A
MF_GO:0016422_mRNA+(2'-O-methyladenosine-N6-)-methyltransferase+ac	0.586122	0.586122	#N/A
BP_GO:0030210_heparin+biosynthetic+process	0.355843	#N/A	0.355843
MF_GO:0050379_UDP-glucuronate+5'-epimerase+activity	0.355843	#N/A	0.355843
MF_GO:0008403_25-hydroxycholecalciferol-24-hydroxylase+activity	0.47375	0.47375	#N/A
MF_GO:0045545_syndecan+binding	0.674041	#N/A	0.674041
BP_GO:0050907_detection+of+chemical+stimulus+involved+in+sensory+per	0.948047	0.948047	#N/A
BP_GO:0050961_detection+of+temperature+stimulus+involved+in+sensory+	0.948047	0.948047	#N/A
BP_GO:0015747_urate+transport	0.430173	#N/A	0.430173
MF_GO:0015143_urate+transmembrane+transporter+activity	0.430173	#N/A	0.430173
BP_GO:0002023_reduction+of+food+intake+in+response+to+dietary+excess	0.991155	0.991155	#N/A
BP_GO:0008633_activation+of+pro-apoptotic+gene+products	0.843954	#N/A	0.843954
BP_GO:0043279_response+to+alkaloid	0.843954	#N/A	0.843954
MF_GO:0008768_UDP-sugar+diphosphatase+activity	0.0177508	0.0177508	#N/A
BP_GO:0030223_neutrophil+differentiation	0.171021	#N/A	0.171021
BP_GO:0032790_ribosome+disassembly	0.452666	0.452666	#N/A

MF_GO:0043024_ribosomal+small+subunit+binding	0.452666	0.452666	#N/A
BP_GO:0060272_embryonic+skeletal+joint+morphogenesis	0.0240571	0.0240571	#N/A
BP_GO:0060348_bone+development	0.839284	0.839284	#N/A
CC_GO:0005585_collagen+type+II	0.839284	0.839284	#N/A
BP_GO:0045407_positive+regulation+of+interleukin-5+biosynthetic+process	0.152664	#N/A	0.152664
BP_GO:0042506_tyrosine+phosphorylation+of+Stat5+protein	0.935869	#N/A	0.935869
BP_GO:0042508_tyrosine+phosphorylation+of+Stat1+protein	0.935869	#N/A	0.935869
BP_GO:0045875_negative+regulation+of+sister+chromatid+cohesion	0.742667	#N/A	0.742667
BP_GO:0015898_amiloride+transport	0.742951	0.742951	#N/A
BP_GO:0042518_negative+regulation+of+tyrosine+phosphorylation+of+Stat	0.301717	0.301717	#N/A
BP_GO:0046655_folic+acid+metabolic+process	0.980268	0.980268	#N/A
BP_GO:0007228_positive+regulation+of+hh+target+transcription+factor+act	0.696996	#N/A	0.696996
BP_GO:0046639_negative+regulation+of+alpha-beta+T+cell+differentiation	0.696996	#N/A	0.696996
BP_GO:0060020_Bergmann+glial+cell+differentiation	0.696996	#N/A	0.696996
MF_GO:0043237_laminin-1+binding	0.696996	#N/A	0.696996
MF_GO:0003977_UDP-N-acetylglucosamine+diphosphorylase+activity	0.447475	0.447475	#N/A
CC_GO:0005760_gamma+DNA+polymerase+complex	0.101363	0.101363	#N/A
BP_GO:0010039_response+to+iron+ion	0.0153637	#N/A	0.0153637
CC_GO:0005960_glycine+cleavage+complex	0.86555	#N/A	0.86555
BP_GO:0046834_lipid+phosphorylation	0.409472	0.409472	#N/A
MF_GO:0001727_lipid+kinase+activity	0.409472	0.409472	#N/A
MF_GO:0047620_acylglycerol+kinase+activity	0.409472	0.409472	#N/A
BP_GO:0045184_establishment+of+protein+localization	0.629144	0.629144	#N/A
BP_GO:0008355_olfactory+learning	0.361716	#N/A	0.361716
BP_GO:0010646_regulation+of+cell+communication	0.361716	#N/A	0.361716
BP_GO:0021586_pons+maturation	0.361716	#N/A	0.361716
BP_GO:0060179_male+mating+behavior	0.361716	#N/A	0.361716
MF_GO:0008176_tRNA+(guanine-N7-)-methyltransferase+activity	0.343694	0.343694	#N/A
BP_GO:0035036_sperm-egg+recognition	0.263302	#N/A	0.263302
MF_GO:0030273_melanin-concentrating+hormone+receptor+activity	0.993735	#N/A	0.993735
BP_GO:0015864_pyrimidine+nucleoside+transport	0.0829919	#N/A	0.0829919
MF_GO:0015389_pyrimidine-+and+adenine-specific:sodium+symporter+acti	0.0829919	#N/A	0.0829919
MF_GO:0015390_purine-specific+nucleoside:sodium+symporter+activity	0.0829919	#N/A	0.0829919
BP_GO:0051102_DNA+ligation+during+DNA+recombination	0.0748776	#N/A	0.0748776
BP_GO:0051103_DNA+ligation+during+DNA+repair	0.0748776	#N/A	0.0748776
CC_GO:0060077_inhibitory+synapse	0.606258	#N/A	0.606258
MF_GO:0015495_gamma-aminobutyric+acid:hydrogen+symporter+activity	0.606258	#N/A	0.606258
CC_GO:0000153_cytoplasmic+ubiquitin+ligase+complex	0.68354	0.68354	#N/A
BP_GO:0043248_proteasome+assembly	0.887316	0.887316	#N/A
BP_GO:0007065_male+meiosis+sister+chromatid+cohesion	0.295055	#N/A	0.295055
BP_GO:0034230_enkephalin+processing	0.52871	0.52871	#N/A
BP_GO:0045725_positive+regulation+of+glycogen+biosynthetic+process	0.463516	0.463516	#N/A
BP_GO:0046321_positive+regulation+of+fatty+acid+oxidation	0.463516	0.463516	#N/A
BP_GO:0035038_female+pronucleus+formation	0.972218	0.972218	#N/A
BP_GO:0035039_male+pronucleus+formation	0.972218	0.972218	#N/A
CC_GO:0033011_perinuclear+theca	0.972218	0.972218	#N/A
MF_GO:0004986_delta-opioid+receptor+activity	0.241102	#N/A	0.241102

MF_GO:0004744_retinal+isomerase+activity	0.955655	0.955655	#N/A
BP_GO:0007321_sperm+displacement	0.313948	0.313948	#N/A
MF_GO:0004723_calcium-dependent+protein+serine/threonine+phosphata:	0.313948	0.313948	#N/A
MF_GO:0004059_aralkylamine+N-acetyltransferase+activity	0.974482	#N/A	0.974482
MF_GO:0004167_dopachrome+isomerase+activity	0.662246	#N/A	0.662246
MF_GO:0035299_inositol+pentakisphosphate+2-kinase+activity	0.434571	0.434571	#N/A
CC_GO:0012510_trans-Golgi+network+transport+vesicle+membrane	0.414136	0.414136	#N/A
MF_GO:0033819_lipoyl(octanoyl)+transferase+activity	0.0690964	0.0690964	#N/A
MF_GO:0008897_holo-[acyl-carrier-protein]+synthase+activity	0.193352	0.193352	#N/A
BP_GO:0042297_vocal+learning	0.916283	#N/A	0.916283
MF_GO:0008507_sodium:iodide+symporter+activity	0.527869	0.527869	#N/A
BP_GO:0018146_keratan+sulfate+biosynthetic+process	0.463859	#N/A	0.463859
MF_GO:0001517_N-acetylglucosamine+6-O-sulfotransferase+activity	0.463859	#N/A	0.463859
BP_GO:0009629_response+to+gravity	0.247978	#N/A	0.247978
BP_GO:0048840_otolith+development	0.247978	#N/A	0.247978
CC_GO:0043020_NADPH+oxidase+complex	0.247978	#N/A	0.247978
CC_GO:0005775_vacuolar+lumen	0.601267	0.601267	#N/A
MF_GO:0015105_arsenite+transmembrane+transporter+activity	0.839947	#N/A	0.839947
MF_GO:0004891_glycine-inhibited+chloride+channel+activity	0.444978	#N/A	0.444978
MF_GO:0050694_galactose+3-O-sulfotransferase+activity	0.348367	#N/A	0.348367
MF_GO:0005132_interferon-alpha/beta+receptor+binding	0.699744	#N/A	0.699744
MF_GO:0015125_bile+acid+transmembrane+transporter+activity	0.884842	#N/A	0.884842
MF_GO:0004808_tRNA+(5-methylaminomethyl-2-thiouridylate)-methyltrans	0.46088	0.46088	#N/A
CC_GO:0032437_cuticular+plate	0.565164	0.565164	#N/A
CC_GO:0060091_kinocilium	0.565164	0.565164	#N/A
BP_GO:0042350_GDP-L-fucose+biosynthetic+process	0.644764	0.644764	#N/A
MF_GO:0047341_fucose-1-phosphate+guanylyltransferase+activity	0.644764	0.644764	#N/A
BP_GO:0009593_detection+of+chemical+stimulus	0.632759	#N/A	0.632759
BP_GO:0021559_trigeminal+nerve+development	0.632759	#N/A	0.632759
MF_GO:0005329_dopamine+transmembrane+transporter+activity	0.773672	#N/A	0.773672
MF_GO:0005330_dopamine:sodium+symporter+activity	0.773672	#N/A	0.773672
MF_GO:0000253_3-keto+sterol+reductase+activity	0.541968	0.541968	#N/A
MF_GO:0050576_3-keto-steroid+reductase+activity	0.541968	0.541968	#N/A
BP_GO:0042107_cytokine+metabolic+process	0.612398	#N/A	0.612398
BP_GO:0060008_Sertoli+cell+differentiation	0.025277	#N/A	0.025277
CC_GO:0030677_ribonuclease+P+complex	0.623122	#N/A	0.623122
BP_GO:0006368_RNA+elongation+from+RNA+polymerase+II+promoter	0.195834	#N/A	0.195834
BP_GO:0048133_male+germ-line+stem+cell+division	0.643403	0.643403	#N/A
MF_GO:0000217_DNA+secondary+structure+binding	0.29496	#N/A	0.29496
BP_GO:0042790_transcription+of+nuclear+rRNA+large+RNA+polymerase+I+	0.854379	0.854379	#N/A
BP_GO:0021891_olfactory+bulb+interneuron+development	0.374232	0.374232	#N/A
BP_GO:0035106_operant+conditioning	0.554616	#N/A	0.554616
BP_GO:0046323_glucose+import	0.554616	#N/A	0.554616
BP_GO:0051968_positive+regulation+of+synaptic+transmission,+glutamater	0.554616	#N/A	0.554616
MF_GO:0001590_dopamine+D1+receptor+activity	0.554616	#N/A	0.554616
BP_GO:0021521_ventral+spinal+cord+interneuron+specification	0.948221	#N/A	0.948221
BP_GO:0030954_spindle+astral+microtubule+nucleation	0.613751	#N/A	0.613751

MF_GO:0042163_interleukin-12+beta+subunit+binding	0.295295	0.295295	#N/A
BP_GO:0000712_resolution+of+meiotic+joint+molecules+as+recombinants	0.381584	0.381584	#N/A
BP_GO:0007060_male+meiosis+chromosome+segregation	0.381584	0.381584	#N/A
BP_GO:0043060_meiotic+metaphase+I+plate+congression	0.381584	0.381584	#N/A
BP_GO:0051257_spindle+midzone+assembly+involved+in+meiosis	0.381584	0.381584	#N/A
MF_GO:0045295_gamma-catenin+binding	0.899693	#N/A	0.899693
MF_GO:0051393_alpha-actinin+binding	0.899693	#N/A	0.899693
MF_GO:0070097_delta-catenin+binding	0.899693	#N/A	0.899693
BP_GO:0042137_sequestering+of+neurotransmitter	0.569909	0.569909	#N/A
CC_GO:0060076_excitatory+synapse	0.569909	0.569909	#N/A
MF_GO:0015319_sodium:inorganic+phosphate+symporter+activity	0.569909	0.569909	#N/A
CC_GO:0042571_immunoglobulin+complex,+circulating	0.489499	#N/A	0.489499
BP_GO:0032289_myelin+formation+in+the+central+nervous+system	0.209386	0.209386	#N/A
BP_GO:0031053_primary+microRNA+processing	0.285246	#N/A	0.285246
MF_GO:0000171_ribonuclease+MRP+activity	0.00425356	0.00425356	#N/A
BP_GO:0051639_actin+filament+network+formation	0.202893	#N/A	0.202893
BP_GO:0006844_acyl+carnitine+transport	0.795365	0.795365	#N/A
MF_GO:0015227_acyl+carnitine+transporter+activity	0.795365	0.795365	#N/A
BP_GO:0002729_positive+regulation+of+natural+killer+cell+cytokine+produ	0.564215	#N/A	0.564215
BP_GO:0032825_positive+regulation+of+natural+killer+cell+differentiation	0.564215	#N/A	0.564215
BP_GO:0046148_pigment+biosynthetic+process	0.588437	#N/A	0.588437
BP_GO:0048852_diencephalon+morphogenesis	0.588437	#N/A	0.588437
BP_GO:0033577_protein+amino+acid+glycosylation+in+endoplasmic+reticul	0.794242	0.794242	#N/A
BP_GO:0006895_Golgi+to+endosome+transport	0.268367	#N/A	0.268367
MF_GO:0030292_protein+tyrosine+kinase+inhibitor+activity	0.487906	#N/A	0.487906
BP_GO:0045994_positive+regulation+of+translational+initiation+by+iron	0.63955	0.63955	#N/A
BP_GO:0045004_DNA+replication+proofreading	0.732494	#N/A	0.732494
BP_GO:0046032_ADP+catabolic+process	0.945531	0.945531	#N/A
BP_GO:0046709_IDP+catabolic+process	0.945531	0.945531	#N/A
BP_GO:0001984_vasodilation+of+artery+during+baroreceptor+response+to+	0.355591	#N/A	0.355591
BP_GO:0031645_negative+regulation+of+neurological+system+process	0.355591	#N/A	0.355591
MF_GO:0031716_calcitonin+receptor+binding	0.355591	#N/A	0.355591
BP_GO:0060334_regulation+of+interferon-gamma-mediated+signaling+path	0.280623	0.280623	#N/A
BP_GO:0060338_regulation+of+type+I+interferon-mediated+signaling+pathw	0.280623	0.280623	#N/A
MF_GO:0004940_beta1-adrenergic+receptor+activity	0.213767	0.213767	#N/A
BP_GO:0006688_glycosphingolipid+biosynthetic+process	0.499703	#N/A	0.499703
MF_GO:0001962_alpha-1,3-galactosyltransferase+activity	0.499703	#N/A	0.499703
CC_GO:0035086_flagellar+axoneme	0.36607	#N/A	0.36607
BP_GO:0040013_negative+regulation+of+locomotion	0.869042	0.869042	#N/A
MF_GO:0016608_growth+hormone-releasing+hormone+activity	0.869042	0.869042	#N/A
BP_GO:0001819_positive+regulation+of+cytokine+production	0.0686472	#N/A	0.0686472
BP_GO:0045740_positive+regulation+of+DNA+replication	0.0686472	#N/A	0.0686472
BP_GO:0030222_eosinophil+differentiation	0.983412	#N/A	0.983412
MF_GO:0030380_interleukin-17E+receptor+binding	0.983412	#N/A	0.983412
BP_GO:0006782_protoporphyrinogen+IX+biosynthetic+process	0.112869	#N/A	0.112869
BP_GO:0001562_response+to+protozoan	0.518923	#N/A	0.518923
BP_GO:0001808_negative+regulation+of+type+IV+hypersensitivity	0.518923	#N/A	0.518923

BP_GO:0050688_regulation+of+defense+response+to+virus	0.518923	#N/A	0.518923
MF_GO:0008367_bacterial+binding	0.518923	#N/A	0.518923
BP_GO:0006408_snRNA+export+from+nucleus	0.665592	0.665592	#N/A
BP_GO:0021930_granule+cell+precursor+proliferation	0.404698	0.404698	#N/A
BP_GO:0048483_autonomic+nervous+system+development	0.404698	0.404698	#N/A
MF_GO:0000170_sphingosine+hydroxylase+activity	0.726034	#N/A	0.726034
BP_GO:0006007_glucose+catabolic+process	0.268061	0.268061	#N/A
BP_GO:0030449_regulation+of+complement+activation	0.917202	0.917202	#N/A
BP_GO:0018283_iron+incorporation+into+metallo-sulfur+cluster	0.266718	0.266718	#N/A
MF_GO:0031071_cysteine+desulfurase+activity	0.266718	0.266718	#N/A
MF_GO:0008569_minus-end-directed+microtubule+motor+activity	0.685798	0.685798	#N/A
MF_GO:0004581_dolichyl-phosphate+beta-glucosyltransferase+activity	0.667284	#N/A	0.667284
BP_GO:0033141_positive+regulation+of+peptidyl-serine+phosphorylation+o	0.307742	#N/A	0.307742
MF_GO:0005133_interferon-gamma+receptor+binding	0.307742	#N/A	0.307742
BP_GO:0006851_mitochondrial+calcium+ion+transport	0.664927	0.664927	#N/A
BP_GO:0048312_intracellular+distribution+of+mitochondria	0.664927	0.664927	#N/A
BP_GO:0002572_pro-T+cell+differentiation	0.564026	#N/A	0.564026
MF_GO:0043548_phosphoinositide+3-kinase+binding	0.564026	#N/A	0.564026
CC_GO:0042581_specific+granule	0.289983	#N/A	0.289983
BP_GO:0051573_negative+regulation+of+histone+H3-K9+methylation	0.339763	0.339763	#N/A
MF_GO:0004398_histidine+decarboxylase+activity	0.923129	0.923129	#N/A
BP_GO:0048284_organelle+fusion	0.9072	#N/A	0.9072
BP_GO:0010507_negative+regulation+of+autophagy	0.916002	0.916002	#N/A
BP_GO:0033028_myeloid+cell+apoptosis	0.916002	0.916002	#N/A
MF_GO:0005135_interleukin-3+receptor+binding	0.916002	0.916002	#N/A
BP_GO:0046986_negative+regulation+of+hemoglobin+biosynthetic+process	0.725133	0.725133	#N/A
MF_GO:0031770_growth+hormone-releasing+hormone+receptor+binding	0.827953	#N/A	0.827953
BP_GO:0021858_GABAergic+neuron+differentiation+in+the+basal+ganglia	0.992869	0.992869	#N/A
BP_GO:0001920_negative+regulation+of+receptor+recycling	0.263039	0.263039	#N/A
CC_GO:0009346_citrate+lyase+complex	0.106702	#N/A	0.106702
MF_GO:0008815_citrate+(pro-3S)-lyase+activity	0.106702	#N/A	0.106702
MF_GO:0016830_carbon-carbon+lyase+activity	0.106702	#N/A	0.106702
BP_GO:0021933_radial+glia+guided+migration+of+granule+cell	0.762732	0.762732	#N/A
BP_GO:0051386_regulation+of+nerve+growth+factor+receptor+signaling+pa	0.762732	0.762732	#N/A
CC_GO:0016507_fatty+acid+beta-oxidation+multienzyme+complex	0.542368	0.542368	#N/A
MF_GO:0016509_long-chain-3-hydroxyacyl-CoA+dehydrogenase+activity	0.542368	0.542368	#N/A
MF_GO:0004170_dUTP+diphosphatase+activity	0.110474	0.110474	#N/A
BP_GO:0000305_response+to+oxygen+radical	0.567676	0.567676	#N/A
MF_GO:0019534_toxin+transporter+activity	0.563571	#N/A	0.563571
BP_GO:0046324_regulation+of+glucose+import	0.626418	0.626418	#N/A
CC_GO:0031315_extrinsic+to+mitochondrial+outer+membrane	0.17187	0.17187	#N/A
MF_GO:0050254_rhodopsin+kinase+activity	0.362383	#N/A	0.362383
BP_GO:0043624_cellular+protein+complex+disassembly	0.176599	0.176599	#N/A
BP_GO:0009691_cytokinin+biosynthetic+process	0.286911	0.286911	#N/A
BP_GO:0045980_negative+regulation+of+nucleotide+metabolic+process	0.538247	0.538247	#N/A
BP_GO:0006304_DNA+modification	0.673681	0.673681	#N/A
CC_GO:0005726_perichromatin+fibrils	0.0771753	0.0771753	#N/A

BP_GO:0033326_cerebrospinal+fluid+secretion	0.856294	#N/A	0.856294
BP_GO:0048546_digestive+tract+morphogenesis	0.856294	#N/A	0.856294
BP_GO:0002508_central+tolerance+induction	0.495385	#N/A	0.495385
BP_GO:0002635_negative+regulation+of+germinal+center+formation	0.495385	#N/A	0.495385
BP_GO:0002897_positive+regulation+of+central+B+cell+tolerance+inductior	0.495385	#N/A	0.495385
BP_GO:0032862_activation+of+Rho+GTPase+activity	0.495385	#N/A	0.495385
BP_GO:0033085_negative+regulation+of+T+cell+differentiation+in+the+thyr	0.495385	#N/A	0.495385
MF_GO:0004603_phenylethanolamine+N-methyltransferase+activity	0.537389	0.537389	#N/A
BP_GO:0000072_M+phase+specific+microtubule+process	0.853573	#N/A	0.853573
CC_GO:0045111_intermediate+filament+cytoskeleton	0.848259	0.848259	#N/A
CC_GO:0032302_MutSbeta+complex	0.668417	#N/A	0.668417
BP_GO:0009105_lipoic+acid+biosynthetic+process	0.820634	#N/A	0.820634
MF_GO:0016992_lipoate+synthase+activity	0.820634	#N/A	0.820634
MF_GO:0017140_lipoic+acid+synthase+activity	0.820634	#N/A	0.820634
BP_GO:0002361_CD4-positive,+CD25-positive,+alpha-beta+regulatory+T+cel	0.419406	0.419406	#N/A
BP_GO:0002637_regulation+of+immunoglobulin+production	0.419406	0.419406	#N/A
BP_GO:0002677_negative+regulation+of+chronic+inflammatory+response	0.419406	0.419406	#N/A
BP_GO:0002851_positive+regulation+of+peripheral+T+cell+tolerance+induc	0.419406	0.419406	#N/A
BP_GO:0032693_negative+regulation+of+interleukin-10+production	0.419406	0.419406	#N/A
BP_GO:0032914_positive+regulation+of+transforming+growth+factor-beta1	0.419406	0.419406	#N/A
BP_GO:0035066_positive+regulation+of+histone+acetylation	0.419406	0.419406	#N/A
BP_GO:0045591_positive+regulation+of+regulatory+T+cell+differentiation	0.419406	0.419406	#N/A
BP_GO:0010569_regulation+of+double-strand+break+repair+via+homologou	0.606278	#N/A	0.606278
BP_GO:0034502_protein+localization+to+chromosome	0.606278	#N/A	0.606278
BP_GO:0045650_negative+regulation+of+macrophage+differentiation	0.975878	#N/A	0.975878
BP_GO:0007210_serotonin+receptor+signaling+pathway	0.811617	#N/A	0.811617
MF_GO:0005232_serotonin-activated+cation-selective+channel+activity	0.811617	#N/A	0.811617
BP_GO:0002253_activation+of+immune+response	0.918544	#N/A	0.918544
BP_GO:0002381_immunoglobulin+production+during+immune+response	0.918544	#N/A	0.918544
BP_GO:0006965_positive+regulation+of+biosynthetic+process+of+antibacte	0.918544	#N/A	0.918544
BP_GO:0032701_negative+regulation+of+interleukin-18+production	0.918544	#N/A	0.918544
BP_GO:0034136_negative+regulation+of+toll-like+receptor+2+signaling+pat	0.918544	#N/A	0.918544
BP_GO:0008616_queuosine+biosynthetic+process	0.411254	0.411254	#N/A
MF_GO:0008479_queuine+tRNA-ribosyltransferase+activity	0.411254	0.411254	#N/A
BP_GO:0046949_acyl-CoA+biosynthetic+process	0.931695	#N/A	0.931695
MF_GO:0043754_dihydrolipoyllysine-residue+(2-methylpropanoyl)transfera	0.931695	#N/A	0.931695
MF_GO:0008605_protein+kinase+CK2+regulator+activity	0.346352	0.346352	#N/A
BP_GO:0048855_adenohypophysis+morphogenesis	0.207894	#N/A	0.207894
MF_GO:0001972_retinoic+acid+binding	0.736883	#N/A	0.736883
BP_GO:0009649_entrainment+of+circadian+clock	0.816137	0.816137	#N/A
MF_GO:0008532_N-acetyllactosaminide+beta-1,3-N-acetylglucosaminyltran	0.283155	0.283155	#N/A
MF_GO:0031957_very-long-chain-fatty-acid-CoA+ligase+activity	0.824341	0.824341	#N/A
BP_GO:0032317_regulation+of+Rap+GTPase+activity	0.285685	#N/A	0.285685
BP_GO:0031034_myosin+filament+assembly	0.818761	#N/A	0.818761
MF_GO:0032036_myosin+heavy+chain+binding	0.818761	#N/A	0.818761
BP_GO:0042985_negative+regulation+of+amyloid+precursor+protein+biosyi	0.752414	#N/A	0.752414
MF_GO:0016019_peptidoglycan+receptor+activity	0.660821	0.660821	#N/A

MF_GO:0008097_5S+rRNA+binding	0.823466	#N/A	0.823466
BP_GO:0042309_homoiothermy	0.696862	#N/A	0.696862
BP_GO:0048714_positive+regulation+of+oligodendrocyte+differentiation	0.760276	#N/A	0.760276
MF_GO:0004997_thyrotropin-releasing+hormone+receptor+activity	0.778892	0.778892	#N/A
MF_GO:0047685_amine+sulfotransferase+activity	0.301083	#N/A	0.301083
BP_GO:0048857_neural+nucleus+development	0.544074	#N/A	0.544074
BP_GO:0007423_sensory+organ+development	0.305857	#N/A	0.305857
BP_GO:0010038_response+to+metal+ion	0.0918998	#N/A	0.0918998
BP_GO:0021589_cerebellum+structural+organization	0.725419	#N/A	0.725419
BP_GO:0021813_cell-cell+adhesion+involved+in+neuronal-glial+interactions	0.725419	#N/A	0.725419
BP_GO:0021942_radial+glia+guided+migration+of+Purkinje+cell	0.725419	#N/A	0.725419
BP_GO:0060018_astrocyte+fate+commitment	0.491878	0.491878	#N/A
MF_GO:0047045_testosterone+17-beta-dehydrogenase+(NADP+)+activity	0.86144	#N/A	0.86144
BP_GO:0032696_negative+regulation+of+interleukin-13+production	0.528194	#N/A	0.528194
BP_GO:0001682_tRNA+5'-leader+removal	0.240107	0.240107	#N/A
CC_GO:0030133_transport+vesicle	0.945953	0.945953	#N/A
CC_GO:0001651_dense+fibrillar+component	0.763137	0.763137	#N/A
BP_GO:0009134_nucleoside+diphosphate+catabolic+process	0.0646967	0.0646967	#N/A
BP_GO:0009143_nucleoside+triphosphate+catabolic+process	0.0646967	0.0646967	#N/A
BP_GO:0003029_detection+of+hypoxic+conditions+in+blood+by+carotid+bc	0.409445	#N/A	0.409445
BP_GO:0060064_Spemann+organizer+formation+at+the+anterior+end+of+t	0.712218	0.712218	#N/A
MF_GO:0003851_2-hydroxyacylsphingosine+1-beta-galactosyltransferase+a	0.782077	#N/A	0.782077
MF_GO:0050577_GDP-L-fucose+synthase+activity	0.0302287	0.0302287	#N/A
BP_GO:0046373_L-arabinose+metabolic+process	0.813377	#N/A	0.813377
MF_GO:0046556_alpha-N-arabinofuranosidase+activity	0.813377	#N/A	0.813377
BP_GO:0007168_receptor+guanylyl+cyclase+signaling+pathway	0.280276	#N/A	0.280276
BP_GO:0016344_meiotic+chromosome+movement+towards+spindle+pole	0.10621	0.10621	#N/A
BP_GO:0040038_polar+body+extrusion+after+meiotic+divisions	0.10621	0.10621	#N/A
BP_GO:0007506_gonadal+mesoderm+development	0.295542	#N/A	0.295542
BP_GO:0014808_release+of+sequestered+calcium+ion+into+cytosol+by+sar	0.984598	0.984598	#N/A
MF_GO:0001567_cholesterol+25-hydroxylase+activity	0.86063	#N/A	0.86063
MF_GO:0008395_steroid+hydroxylase+activity	0.86063	#N/A	0.86063
CC_GO:0009317_acetyl-CoA+carboxylase+complex	0.508507	0.508507	#N/A
CC_GO:0016235_aggresome	0.00526246	#N/A	0.00526246
BP_GO:0000256_allantoin+catabolic+process	0.748646	#N/A	0.748646
MF_GO:0004037_allantoicase+activity	0.748646	#N/A	0.748646
BP_GO:0030540_female+genitalia+development	0.404345	#N/A	0.404345
MF_GO:0001631_cysteinyl+leukotriene+receptor+activity	0.105613	0.105613	#N/A
BP_GO:0002003_angiotensin+maturation	0.32774	#N/A	0.32774
MF_GO:0042922_neuromedin+U+receptor+binding	0.43731	#N/A	0.43731
MF_GO:0016005_phospholipase+A2+activator+activity	0.503971	#N/A	0.503971
BP_GO:0002085_repression+of+premature+neural+plate+formation	0.657691	0.657691	#N/A
MF_GO:0003948_N4-(beta-N-acetylglucosaminyl)-L-asparaginase+activity	0.985789	0.985789	#N/A
BP_GO:0048681_negative+regulation+of+axon+regeneration	0.357756	0.357756	#N/A
BP_GO:0050807_regulation+of+synapse+organization	0.357756	0.357756	#N/A
BP_GO:0021889_olfactory+bulb+interneuron+differentiation	0.304183	#N/A	0.304183
BP_GO:0051026_chiasma+formation	0.49041	0.49041	#N/A

MF_GO:0047915_ganglioside+galactosyltransferase+activity	0.236507	0.236507	#N/A
MF_GO:0050681_androgen+receptor+binding	0.869945	0.869945	#N/A
MF_GO:0001604_urotensin+II+receptor+activity	0.0659562	#N/A	0.0659562
BP_GO:0021874_Wnt+receptor+signaling+pathway+in+forebrain+neuroblas	0.157116	0.157116	#N/A
BP_GO:0045839_negative+regulation+of+mitosis	0.336579	0.336579	#N/A
MF_GO:0004987_kappa-opioid+receptor+activity	0.410548	0.410548	#N/A
BP_GO:0045661_regulation+of+myoblast+differentiation	0.788225	#N/A	0.788225
MF_GO:0046527_glucosyltransferase+activity	0.59058	0.59058	#N/A
BP_GO:0018009_N-terminal+peptidyl-L-cysteine+N-palmitoylation	0.667646	#N/A	0.667646
MF_GO:0008757_S-adenosylmethionine-dependent+methyltransferase+acti	0.765986	0.765986	#N/A
MF_GO:0046403_polynucleotide+3'-phosphatase+activity	0.168297	0.168297	#N/A
CC_GO:0031231_intrinsic+to+peroxisomal+membrane	0.5505	#N/A	0.5505
MF_GO:0004811_tRNA+isopentenyltransferase+activity	0.318072	0.318072	#N/A
BP_GO:0042667_auditory+receptor+cell+fate+specification	0.639313	#N/A	0.639313
BP_GO:0045609_positive+regulation+of+auditory+receptor+cell+differentiat	0.639313	#N/A	0.639313
BP_GO:0021535_cell+migration+in+hindbrain	0.0528109	0.0528109	#N/A
BP_GO:0048934_peripheral+nervous+system+neuron+differentiation	0.0528109	0.0528109	#N/A
BP_GO:0051355_proprioception+during+equilibrioception	0.0528109	0.0528109	#N/A
BP_GO:0035260_internal+genitalia+morphogenesis	0.691929	0.691929	#N/A
BP_GO:0034339_regulation+of+transcription+from+RNA+polymerase+II+prc	0.999176	0.999176	#N/A
BP_GO:0002882_positive+regulation+of+chronic+inflammatory+response+tc	0.852483	#N/A	0.852483
BP_GO:0008038_neuron+recognition	0.656844	#N/A	0.656844
BP_GO:0055078_sodium+ion+homeostasis	0.187732	#N/A	0.187732
BP_GO:0043486_histone+exchange	0.352666	#N/A	0.352666
MF_GO:0030354_melanin-concentrating+hormone+activity	0.658662	#N/A	0.658662
BP_GO:0022004_midbrain-hindbrain+boundary+maturation+during+brain+c	0.625319	0.625319	#N/A
MF_GO:0042164_interleukin-12+alpha+subunit+binding	0.171518	#N/A	0.171518
BP_GO:0060231_mesenchymal+to+epithelial+transition	0.53756	0.53756	#N/A
BP_GO:0002432_granuloma+formation	0.846436	0.846436	#N/A
MF_GO:0000224_peptide-N4-(N-acetyl-beta-glucosaminyl)asparagine+amida	0.979607	0.979607	#N/A
MF_GO:0051267_CP2+mannose-ethanolamine+phosphotransferase+activity	0.768247	0.768247	#N/A
BP_GO:0001980_regulation+of+systemic+arterial+blood+pressure+by+ische	0.555132	0.555132	#N/A
CC_GO:0048476_Holliday+junction+resolvase+complex	0.490101	#N/A	0.490101
MF_GO:0008821_crossover+junction+endodeoxyribonuclease+activity	0.490101	#N/A	0.490101
MF_GO:0004521_endoribonuclease+activity	0.884488	0.884488	#N/A
MF_GO:0032542_sulfiredoxin+activity	0.61179	0.61179	#N/A
MF_GO:0019706_protein-cysteine+S-palmitoleyltransferase+activity	0.192464	0.192464	#N/A
BP_GO:0007614_short-term+memory	0.286773	#N/A	0.286773
BP_GO:0033555_multicellular+organismal+response+to+stress	0.286773	#N/A	0.286773
CC_GO:0048787_presynaptic+active+zone+membrane	0.286773	#N/A	0.286773
BP_GO:0001774_microglial+cell+activation	0.150508	#N/A	0.150508
BP_GO:0001956_positive+regulation+of+neurotransmitter+secretion	0.150508	#N/A	0.150508
BP_GO:0014048_regulation+of+glutamate+secretion	0.150508	#N/A	0.150508
BP_GO:0034599_cellular+response+to+oxidative+stress	0.150508	#N/A	0.150508
BP_GO:0042775_mitochondrial+ATP+synthesis+coupled+electron+transport	0.150508	#N/A	0.150508
BP_GO:0050812_regulation+of+acyl-CoA+biosynthetic+process	0.150508	#N/A	0.150508
MF_GO:0019855_calcium+channel+inhibitor+activity	0.988845	#N/A	0.988845

BP_GO:0006747_FAD+biosynthetic+process	0.525685	0.525685	#N/A
MF_GO:0003919_FMN+adenylyltransferase+activity	0.525685	0.525685	#N/A
MF_GO:0033699_DNA+5'-adenosine+monophosphate+hydrolase+activity	0.356113	0.356113	#N/A
BP_GO:0006515_misfolded+or+incompletely+synthesized+protein+catabolic	0.0129422	0.0129422	#N/A
BP_GO:0019932_second-messenger-mediated+signaling	0.880771	#N/A	0.880771
BP_GO:0002554_serotonin+secretion+by+platelet	0.675906	0.675906	#N/A
BP_GO:0003056_regulation+of+vascular+smooth+muscle+contraction	0.675906	0.675906	#N/A
CC_GO:0031240_external+side+of+cell+outer+membrane	0.675906	0.675906	#N/A
MF_GO:0008118_N-acetyllactosaminide+alpha-2,3-sialyltransferase+activity	0.706242	0.706242	#N/A
MF_GO:0004875_complement+receptor+activity	0.299538	0.299538	#N/A
BP_GO:0007062_sister+chromatid+cohesion	0.332279	#N/A	0.332279
BP_GO:0010042_response+to+manganese+ion	0.719846	0.719846	#N/A
BP_GO:0032025_response+to+cobalt+ion	0.719846	0.719846	#N/A
BP_GO:0032026_response+to+magnesium+ion	0.719846	0.719846	#N/A
MF_GO:0051990_(R)-2-hydroxyglutarate+dehydrogenase+activity	0.719846	0.719846	#N/A
MF_GO:0003830_beta-1,4-mannosylglycoprotein+4-beta-N-acetylglucosami	0.297833	#N/A	0.297833
BP_GO:0048050_post-embryonic+eye+morphogenesis	0.404606	0.404606	#N/A
BP_GO:0043438_acetoacetic+acid+metabolic+process	0.831575	#N/A	0.831575
CC_GO:0033162_melanosome+membrane	0.831575	#N/A	0.831575
MF_GO:0016716_oxidoreductase+activity,+acting+on+paired+donors,+with-	0.831575	#N/A	0.831575
BP_GO:0048250_mitochondrial+iron+ion+transport	0.581366	#N/A	0.581366
BP_GO:0006082_organic+acid+metabolic+process	0.311737	0.311737	#N/A
BP_GO:0021999_neural+plate+anterioposterior+pattern+formation	0.865192	0.865192	#N/A
BP_GO:0032583_regulation+of+gene-specific+transcription	0.865192	0.865192	#N/A
MF_GO:0032394_MHC+class+Ib+receptor+activity	0.663948	0.663948	#N/A
MF_GO:0042288_MHC+class+I+protein+binding	0.663948	0.663948	#N/A
BP_GO:0002536_respiratory+burst+during+acute+inflammatory+response	0.0809038	0.0809038	#N/A
BP_GO:0010310_regulation+of+hydrogen+peroxide+metabolic+process	0.0809038	0.0809038	#N/A
BP_GO:0010671_negative+regulation+of+oxygen+and+reactive+oxygen+spe	0.0809038	0.0809038	#N/A
BP_GO:0031665_negative+regulation+of+lipopolysaccharide-mediated+sign	0.0809038	0.0809038	#N/A
MF_GO:0008379_thioredoxin+peroxidase+activity	0.0809038	0.0809038	#N/A
BP_GO:0009249_protein+lipoylation	0.118693	#N/A	0.118693
BP_GO:0021761_limbic+system+development	0.941421	0.941421	#N/A
MF_GO:0005144_interleukin-13+receptor+binding	0.616678	#N/A	0.616678
MF_GO:0001621_platelet+ADP+receptor+activity	0.571817	0.571817	#N/A
BP_GO:0006337_nucleosome+disassembly	0.512159	#N/A	0.512159
MF_GO:0004509_steroid+21-monooxygenase+activity	0.315909	0.315909	#N/A
MF_GO:0005092_GDP-dissociation+inhibitor+activity	0.521393	#N/A	0.521393
BP_GO:0009187_cyclic+nucleotide+metabolic+process	0.610508	#N/A	0.610508
MF_GO:0005223_intracellular+cGMP+activated+cation+channel+activity	0.610508	#N/A	0.610508
MF_GO:0005502_11-cis+retinal+binding	0.915669	#N/A	0.915669
MF_GO:0003980_UDP-glucose:glycoprotein+glucosyltransferase+activity	0.956838	#N/A	0.956838
CC_GO:0032592_integral+to+mitochondrial+membrane	0.103395	0.103395	#N/A
MF_GO:0016846_carbon-sulfur+lyase+activity	0.01542	0.01542	#N/A
BP_GO:0002317_plasma+cell+differentiation	0.634406	#N/A	0.634406
BP_GO:0016333_morphogenesis+of+follicular+epithelium	0.634406	#N/A	0.634406
BP_GO:0048621_post-embryonic+gut+morphogenesis	0.634406	#N/A	0.634406

BP_GO:0010629_negative+regulation+of+gene+expression	0.63724	0.63724	#N/A
BP_GO:0034984_cellular+response+to+DNA+damage+stimulus	0.63724	0.63724	#N/A
BP_GO:0043046_DNA+methylation+during+gametogenesis	0.63724	0.63724	#N/A
MF_GO:0050429_calcium-dependent+phospholipase+C+activity	0.0983492	#N/A	0.0983492
MF_GO:0033798_thyroxine+5-deiodinase+activity	0.725943	0.725943	#N/A
BP_GO:0008626_induction+of+apoptosis+by+granzyme	0.997735	#N/A	0.997735
BP_GO:0048525_negative+regulation+of+viral+reproduction	0.7274	0.7274	#N/A
MF_GO:0005136_interleukin-4+receptor+binding	0.292241	#N/A	0.292241
BP_GO:0019101_female+somatic+sex+determination	0.0427237	0.0427237	#N/A
BP_GO:0060014_granulosa+cell+differentiation	0.0427237	0.0427237	#N/A
BP_GO:0003032_detection+of+oxygen	0.343993	0.343993	#N/A
BP_GO:0003069_vasodilation+by+acetylcholine+involved+in+regulation+of+	0.343993	0.343993	#N/A
BP_GO:0010260_organ+senescence	0.343993	0.343993	#N/A
BP_GO:0048773_erythrophore+differentiation	0.343993	0.343993	#N/A
BP_GO:0055093_response+to+hyperoxia	0.343993	0.343993	#N/A
MF_GO:0004027_alcohol+sulfotransferase+activity	0.301172	#N/A	0.301172
MF_GO:0004964_lutropin-choriogonadotropic+hormone+receptor+activity	0.319801	0.319801	#N/A
MF_GO:0004004_ATP-dependent+RNA+helicase+activity	0.203609	#N/A	0.203609
BP_GO:0051100_negative+regulation+of+binding	0.449985	0.449985	#N/A
MF_GO:0008252_nucleotidase+activity	0.398007	#N/A	0.398007
BP_GO:0006356_regulation+of+transcription+from+RNA+polymerase+I+pro	0.504472	0.504472	#N/A
CC_GO:0030685_nucleolar+preribosome	0.321897	#N/A	0.321897
BP_GO:0007113_endomitotic+cell+cycle	0.40161	#N/A	0.40161
MF_GO:0019781_NEDD8+activating+enzyme+activity	0.40161	#N/A	0.40161
MF_GO:0015379_potassium:chloride+symporter+activity	0.0439108	0.0439108	#N/A
MF_GO:0032558_adenyl+deoxyribonucleotide+binding	0.215438	0.215438	#N/A
BP_GO:0046578_regulation+of+Ras+protein+signal+transduction	0.212205	0.212205	#N/A
MF_GO:0004671_protein-S-isoprenylcysteine+O-methyltransferase+activity	0.212205	0.212205	#N/A
MF_GO:0008140_cAMP+response+element+binding+protein+binding	0.212205	0.212205	#N/A
BP_GO:0048691_positive+regulation+of+axon+extension+involved+in+reger	0.336614	#N/A	0.336614
BP_GO:0006585_dopamine+biosynthetic+process+from+tyrosine	0.697077	0.697077	#N/A
MF_GO:0004511_tyrosine+3-monooxygenase+activity	0.697077	0.697077	#N/A
BP_GO:0032962_positive+regulation+of+inositol+trisphosphate+biosyntheti	0.678506	#N/A	0.678506
BP_GO:0035092_sperm+chromatin+condensation	0.593538	0.593538	#N/A
BP_GO:0000027_ribosomal+large+subunit+assembly+and+maintenance	0.0398715	0.0398715	#N/A
BP_GO:0010458_exit+from+mitosis	0.0398715	0.0398715	#N/A
BP_GO:0021554_optic+nerve+development	0.0398715	0.0398715	#N/A
MF_GO:0005155_epidermal+growth+factor+receptor+activating+ligand+act	0.156812	#N/A	0.156812
MF_GO:0005335_serotonin:sodium+symporter+activity	0.349242	0.349242	#N/A
BP_GO:0001696_gastric+acid+secretion	0.224639	#N/A	0.224639
MF_GO:0005253_anion+channel+activity	0.224639	#N/A	0.224639
BP_GO:0000080_G1+phase+of+mitotic+cell+cycle	0.770701	0.770701	#N/A
BP_GO:0045924_regulation+of+female+receptivity	0.584301	#N/A	0.584301
MF_GO:0001589_dopamine+D5+receptor+activity	0.584301	#N/A	0.584301
BP_GO:0031947_negative+regulation+of+glucocorticoid+biosynthetic+proce	0.611521	0.611521	#N/A
BP_GO:0045823_positive+regulation+of+heart+contraction	0.611521	0.611521	#N/A
BP_GO:0045989_positive+regulation+of+striated+muscle+contraction	0.611521	0.611521	#N/A

MF_GO:0003869_4-nitrophenylphosphatase+activity	0.611521	0.611521	#N/A
MF_GO:0004362_glutathione-disulfide+reductase+activity	0.703566	0.703566	#N/A
MF_GO:0004503_monophenol+monooxygenase+activity	0.108312	#N/A	0.108312
BP_GO:0032863_activation+of+Rac+GTPase+activity	0.7873	0.7873	#N/A
BP_GO:0035020_regulation+of+Rac+protein+signal+transduction	0.7873	0.7873	#N/A
BP_GO:0021508_floor+plate+formation	0.30076	#N/A	0.30076
BP_GO:0021517_ventral+spinal+cord+development	0.30076	#N/A	0.30076
BP_GO:0030886_negative+regulation+of+myeloid+dendritic+cell+activation	0.842786	#N/A	0.842786
MF_GO:0005183_gonadotropin+hormone-releasing+hormone+activity	0.0663471	#N/A	0.0663471
BP_GO:0030655_beta-lactam+antibiotic+catabolic+process	0.554446	0.554446	#N/A
MF_GO:0008800_beta-lactamase+activity	0.554446	0.554446	#N/A
BP_GO:0016267_O-glycan+processing,+core+1	0.0162016	#N/A	0.0162016
MF_GO:0016263_glycoprotein-N-acetylgalactosamine+3-beta-galactosyltran	0.0162016	#N/A	0.0162016
BP_GO:0043487_regulation+of+RNA+stability	0.283632	0.283632	#N/A
BP_GO:0035262_gonad+morphogenesis	0.902238	#N/A	0.902238
BP_GO:0006624_vacuolar+protein+processing	0.0917573	#N/A	0.0917573
BP_GO:0043313_regulation+of+neutrophil+degranulation	0.436108	#N/A	0.436108
BP_GO:0048850_hypophysis+morphogenesis	0.369855	0.369855	#N/A
BP_GO:0051453_regulation+of+intracellular+pH	0.672677	#N/A	0.672677
MF_GO:0015386_potassium:hydrogen+antiporter+activity	0.672677	#N/A	0.672677
BP_GO:0021523_somatic+motor+neuron+differentiation	0.183651	0.183651	#N/A
BP_GO:0021703_locus+ceruleus+development	0.183651	0.183651	#N/A
BP_GO:0048486_parasympathetic+nervous+system+development	0.183651	0.183651	#N/A
BP_GO:0006984_ER-nuclear+signaling+pathway	0.276706	0.276706	#N/A
MF_GO:0003701_RNA+polymerase+I+transcription+factor+activity	0.678706	0.678706	#N/A
MF_GO:0004968_gonadotropin-releasing+hormone+receptor+activity	0.144778	#N/A	0.144778
BP_GO:0048597_post-embryonic+camera-type+eye+morphogenesis	0.0578872	0.0578872	#N/A
MF_GO:0008386_cholesterol+monooxygenase+(side-chain-cleaving)+activity	0.989578	#N/A	0.989578
MF_GO:0017065_single-strand+selective+uracil+DNA+N-glycosylase+activity	0.356091	0.356091	#N/A
BP_GO:0045347_negative+regulation+of+MHC+class+II+biosynthetic+proces	0.486145	#N/A	0.486145
MF_GO:0001883_purine+nucleoside+binding	0.362251	0.362251	#N/A
MF_GO:0004923_leukemia+inhibitory+factor+receptor+activity	0.524603	#N/A	0.524603
CC_GO:0005751_mitochondrial+respiratory+chain+complex+IV	0.176961	0.176961	#N/A
MF_GO:0050692_DBD+domain+binding	0.911029	#N/A	0.911029
BP_GO:0014012_axon+regeneration+in+the+peripheral+nervous+system	0.309309	0.309309	#N/A
MF_GO:0016536_cyclin-dependent+protein+kinase+5+activator+regulator+a	0.0163791	0.0163791	#N/A
MF_GO:0016746_transferase+activity,+transferring+acyl+groups	0.633135	#N/A	0.633135
BP_GO:0021658_rhombomere+3+morphogenesis	0.837181	0.837181	#N/A
BP_GO:0045210_FasL+biosynthetic+process	0.795171	0.795171	#N/A
MF_GO:0046625_sphingolipid+binding	0.301599	#N/A	0.301599
MF_GO:0008534_oxidized+purine+base+lesion+DNA+N-glycosylase+activity	0.469687	#N/A	0.469687
MF_GO:0034039_8-oxo-7,8-dihydroguanine+DNA+N-glycosylase+activity	0.469687	#N/A	0.469687
MF_GO:0015334_high+affinity+oligopeptide+transporter+activity	0.112023	#N/A	0.112023
BP_GO:0007382_specification+of+segmental+identity,+maxillary+segment	0.0477817	#N/A	0.0477817
BP_GO:0021532_neural+tube+patterning	0.0477817	#N/A	0.0477817
BP_GO:0042305_specification+of+segmental+identity,+mandibular+segmen	0.0477817	#N/A	0.0477817
BP_GO:0042527_negative+regulation+of+tyrosine+phosphorylation+of+Stat	0.139387	0.139387	#N/A

MF_GO:0004577_N-acetylglucosaminyldiphosphodolichol+N-acetylglucosam	0.645021	0.645021	#N/A
BP_GO:0033554_cellular+response+to+stress	0.980547	#N/A	0.980547
BP_GO:0001712_ectodermal+cell+fate+commitment	0.200878	0.200878	#N/A
BP_GO:0045955_negative+regulation+of+calcium+ion-dependent+exocytosi	0.200878	0.200878	#N/A
BP_GO:0006709_progesterone+catabolic+process	0.724582	#N/A	0.724582
MF_GO:0047006_20-alpha-hydroxysteroid+dehydrogenase+activity	0.724582	#N/A	0.724582
MF_GO:0005165_neurotrophin+receptor+binding	0.566943	#N/A	0.566943
MF_GO:0004584_dolichyl-phosphate-mannose-glycolipid+alpha-mannosyltr	0.422103	#N/A	0.422103
BP_GO:0051383_kinetochore+organization	0.133077	#N/A	0.133077
MF_GO:0043515_kinetochore+binding	0.133077	#N/A	0.133077
BP_GO:0010032_meiotic+chromosome+condensation	0.949709	#N/A	0.949709
BP_GO:0033315_meiotic+cell+cycle+DNA+replication+checkpoint	0.949709	#N/A	0.949709
BP_GO:0042138_meiotic+DNA+double-strand+break+formation	0.949709	#N/A	0.949709
MF_GO:0030280_structural+constituent+of+epidermis	0.0953339	#N/A	0.0953339
BP_GO:0050890_cognition	0.19631	#N/A	0.19631
BP_GO:0042256_mature+ribosome+assembly	0.196195	0.196195	#N/A
BP_GO:0000290_deadenylation-dependent+decapping+of+nuclear-transcrib	0.95003	0.95003	#N/A
MF_GO:0002055_adenine+binding	0.0529877	0.0529877	#N/A
MF_GO:0003999_adenine+phosphoribosyltransferase+activity	0.0529877	0.0529877	#N/A
MF_GO:0045517_interleukin-20+receptor+binding	0.641248	0.641248	#N/A
MF_GO:0050265_RNA+uridylyltransferase+activity	0.403564	0.403564	#N/A
BP_GO:0045836_positive+regulation+of+meiosis	0.415726	0.415726	#N/A
BP_GO:0045948_positive+regulation+of+translational+initiation	0.415726	0.415726	#N/A
MF_GO:0042156_zinc-mediated+transcriptional+activator+activity	0.638387	#N/A	0.638387
BP_GO:0022408_negative+regulation+of+cell-cell+adhesion	0.779164	#N/A	0.779164
MF_GO:0004102_choline+O-acetyltransferase+activity	0.988142	0.988142	#N/A
BP_GO:0031344_regulation+of+cell+projection+organization	0.816809	#N/A	0.816809
MF_GO:0004479_methionyl-tRNA+formyltransferase+activity	0.217724	#N/A	0.217724
CC_GO:0030061_mitochondrial+crista	0.527697	0.527697	#N/A
BP_GO:0032621_interleukin-18+production	0.852858	0.852858	#N/A
BP_GO:0050701_interleukin-1+secretion	0.852858	0.852858	#N/A
BP_GO:0030194_positive+regulation+of+blood+coagulation	0.133205	0.133205	#N/A
MF_GO:0015068_glycine+amidinotransferase+activity	0.701289	#N/A	0.701289
CC_GO:0032426_stereocilium+bundle+tip	0.606192	0.606192	#N/A
BP_GO:0046058_cAMP+metabolic+process	0.627481	#N/A	0.627481
MF_GO:0003905_alkylbase+DNA+N-glycosylase+activity	0.451817	0.451817	#N/A
BP_GO:0032727_positive+regulation+of+interferon-alpha+production	0.880164	0.880164	#N/A
BP_GO:0048280_vesicle+fusion+with+Golgi+apparatus	0.871593	#N/A	0.871593
MF_GO:0008251_tRNA-specific+adenosine+deaminase+activity	0.710554	0.710554	#N/A
BP_GO:0000271_polysaccharide+biosynthetic+process	0.417148	0.417148	#N/A
BP_GO:0048702_embryonic+neurocranium+morphogenesis	0.417148	0.417148	#N/A
CC_GO:0005797_Golgi+medial+cisterna	0.553909	#N/A	0.553909
MF_GO:0047220_galactosylxylosylprotein+3-beta-galactosyltransferase+acti	0.553909	#N/A	0.553909
BP_GO:0006896_Golgi+to+vacuole+transport	0.979915	#N/A	0.979915
BP_GO:0042268_regulation+of+cytolysis	0.186229	#N/A	0.186229
CC_GO:0043189_H4/H2A+histone+acetyltransferase+complex	0.319912	0.319912	#N/A
BP_GO:0000238_zygotene	0.833635	#N/A	0.833635

CC_GO:0044451_nucleoplasm+part	0.950999	#N/A	0.950999
MF_GO:0015326_cationic+amino+acid+transmembrane+transporter+activit	0.120136	#N/A	0.120136
CC_GO:0005831_steroid+hormone+aporeceptor+complex	0.487292	0.487292	#N/A
MF_GO:0047429_nucleoside-triphosphate+diphosphatase+activity	0.615835	#N/A	0.615835
MF_GO:0030021_extracellular+matrix+structural+constituent+conferring+cc	0.585333	#N/A	0.585333
MF_GO:0030345_structural+constituent+of+tooth+enamel	0.585333	#N/A	0.585333
BP_GO:0032637_interleukin-8+production	0.686565	0.686565	#N/A
BP_GO:0017183_peptidyl-diphthamide+biosynthetic+process+from+peptidy	0.695134	0.695134	#N/A
MF_GO:0004164_diphthine+synthase+activity	0.695134	0.695134	#N/A
MF_GO:0009019_tRNA+(guanine-N1-)-methyltransferase+activity	0.972375	#N/A	0.972375
MF_GO:0042019_interleukin-23+binding	0.0688786	#N/A	0.0688786
MF_GO:0042020_interleukin-23+receptor+activity	0.0688786	#N/A	0.0688786
BP_GO:0002678_positive+regulation+of+chronic+inflammatory+response	0.888417	0.888417	#N/A
BP_GO:0002830_positive+regulation+of+T-helper+2+type+immune+respons	0.888417	0.888417	#N/A
BP_GO:0045743_positive+regulation+of+fibroblast+growth+factor+receptor	0.457714	0.457714	#N/A
MF_GO:0005222_intracellular+cAMP+activated+cation+channel+activity	0.157073	#N/A	0.157073
BP_GO:0008535_respiratory+chain+complex+IV+assembly	0.134743	0.134743	#N/A
BP_GO:0043467_regulation+of+generation+of+precursor+metabolites+and+	0.243496	0.243496	#N/A
MF_GO:0004810_tRNA+adenylyltransferase+activity	0.38139	0.38139	#N/A
MF_GO:0009022_tRNA+nucleotidyltransferase+activity	0.38139	0.38139	#N/A
MF_GO:0008419_RNA+lariat+debranching+enzyme+activity	0.762881	0.762881	#N/A
CC_GO:0000439_core+TFIIH+complex	0.0501649	0.0501649	#N/A
BP_GO:0019731_antibacterial+humoral+response	0.384168	0.384168	#N/A
CC_GO:0002095_caveolar+macromolecular+signaling+complex	0.754034	0.754034	#N/A
BP_GO:0009956_radial+pattern+formation	0.940023	#N/A	0.940023
MF_GO:0008917_lipopolysaccharide+N-acetylglucosaminyltransferase+activ	0.198073	#N/A	0.198073
CC_GO:0009320_phosphoribosylaminoimidazole+carboxylase+complex	0.999215	0.999215	#N/A
MF_GO:0004638_phosphoribosylaminoimidazole+carboxylase+activity	0.999215	0.999215	#N/A
MF_GO:0004639_phosphoribosylaminoimidazolesuccinocarboxamide+synth	0.999215	0.999215	#N/A
CC_GO:0005850_eukaryotic+translation+initiation+factor+2+complex	0.563032	#N/A	0.563032
CC_GO:0017117_single-stranded+DNA-dependent+ATP-dependent+DNA+he	0.963615	0.963615	#N/A
MF_GO:0017116_single-stranded+DNA-dependent+ATP-dependent+DNA+h	0.963615	0.963615	#N/A
MF_GO:0005138_interleukin-6+receptor+binding	0.237691	#N/A	0.237691
BP_GO:0006055_CMP-N-acetylneuraminate+biosynthetic+process	0.592624	0.592624	#N/A
MF_GO:0008781_N-acylneuraminate+cytidylyltransferase+activity	0.592624	0.592624	#N/A
MF_GO:0008187_poly-pyrimidine+tract+binding	0.879871	#N/A	0.879871
CC_GO:0033588_Elongator+holoenzyme+complex	0.686927	0.686927	#N/A
MF_GO:0004574_oligo-1,6-glucosidase+activity	0.307052	#N/A	0.307052
MF_GO:0016937_short-branched-chain-acyl-CoA+dehydrogenase+activity	0.978666	#N/A	0.978666
MF_GO:0003921_GMP+synthase+activity	0.71779	#N/A	0.71779
MF_GO:0003922_GMP+synthase+(glutamine-hydrolyzing)+activity	0.71779	#N/A	0.71779
BP_GO:0006419_alanyl-tRNA+aminoacylation	0.778176	0.778176	#N/A
BP_GO:0034620_cellular+response+to+unfolded+protein	0.778176	0.778176	#N/A
MF_GO:0008470_isovaleryl-CoA+dehydrogenase+activity	0.114117	0.114117	#N/A
BP_GO:0021885_forebrain+cell+migration	0.575912	0.575912	#N/A
BP_GO:0050760_negative+regulation+of+thymidylate+synthase+biosyntheti	0.306521	0.306521	#N/A
BP_GO:0006222_UMP+biosynthetic+process	0.151698	0.151698	#N/A

Muscle

Genome-scale model(GSM)

#Feature	Reporter P-value No-direction	Down regulated	Up regulated
ATP	0.573482	0.0229891	0.996672
NADH	0.00479599	1.74475E-05	0.961686
NADPH	0.780763	0.323428	0.93063
Pyrophosphate	0.691116	0.248295	0.955078
ADP	0.0749567	0.00443637	0.821816
AMP	0.950839	0.760229	0.955538
Oxygen	0.975591	0.829381	0.964337
UDP	0.616232	0.48576	0.684772
NADP+	0.928229	0.693862	0.930158
Orthophosphate	0.656298	0.128076	0.964565
CoA	0.917176	0.779981	0.913758
Ubiquinone	0.000836733	1.54149E-05	0.980355
Ubiquinol	0.000802306	1.54149E-05	0.986546
NAD+	0.107255	0.319249	0.100804
Acetyl-CoA	0.886738	0.849041	0.736398
Benzo[a]pyrene-4,5-oxide	0.956185	0.272729	0.99757
Benzo[a]pyrene-7,8-diol	0.956185	0.272729	0.99757
GTP	0.112073	0.0103248	0.885556
CO2	0.83597	0.785442	0.717893
(5Z,8Z,11Z,14Z)-Icosatetraenoic_acid	0.981262	0.869359	0.960214
H2O	0.668262	0.619864	0.623692
UTP	0.375553	0.103634	0.901916
CTP	0.459504	0.134804	0.921565
3',5'-Cyclic_GMP	0.904791	0.980234	0.490265
(1R,2S)-Naphthalene_1,2-oxide	0.54236	0.091795	0.947818
(1S,2R)-Naphthalene_1,2-oxide	0.54236	0.091795	0.947818
1-Nitronaphthalene-5,6-oxide	0.599982	0.120615	0.947818
GDP	0.166915	0.0114191	0.818303
UDP-D-galactose	0.139176	0.37986	0.0971952
2-Amino-4-hydroxy-6-(erythro-1,2,3-trihydroxypropyl)dihydropteridine_tripl	0.570615	0.161714	0.878489
1-Nitronaphthalene-7,8-oxide	0.587805	0.120615	0.948665
3',5'-Cyclic_AMP	0.90082	0.962394	0.5513
2-Amino-4-hydroxy-6-(D-erythro-1,2,3-trihydroxypropyl)-7,8-dihydropteridin	0.649558	0.235393	0.878489
UDP-N-acetyl-D-glucosamine	0.636171	0.411271	0.753292
Trichloroethene	0.611077	0.0725082	0.979972
Bromobenzene-3,4-oxide	0.564365	0.0725082	0.969463
Bromobenzene-2,3-oxide	0.564365	0.0725082	0.969463
Isomaltose	0.691361	0.276259	0.873248
H+	0.927362	0.893688	0.814675
UDP-N-acetyl-D-galactosamine	0.869574	0.915364	0.529922

Dihydroneopterin_phosphate	0.727237	0.276259	0.90402
Linoleate	0.959185	0.939034	0.870624
CMP	0.624991	0.109369	0.935249
9(10)-EpOME	0.960789	0.960903	0.870624
12(13)-EpOME	0.960789	0.960903	0.870624
11,12-EET	0.982257	0.855955	0.965468
2,2-Dichloroacetaldehyde	0.558617	0.0254103	0.992878
1,1-DichloroethylenE_epoxide	0.558617	0.0254103	0.992878
Chloroacetyl_chloride	0.558617	0.0254103	0.992878
1,2-Dibromoethane	0.490706	0.0254103	0.989596
2-Bromoacetaldehyde	0.490706	0.0254103	0.989596
IMP	0.280305	0.0343783	0.740765
Palmitoyl-CoA	0.45436	0.479282	0.451707
Ubiquitin	0.793153	0.703112	0.73527
Benzo[a]pyrene-7,8-oxide	0.986156	0.97742	0.919831
RX	0.590212	0.0254103	0.997884
HX	0.590212	0.0254103	0.997884
7,8-Dihydro-7-hydroxy-8-S-glutathionyl-benzo[a]pyrene	0.590212	0.0254103	0.997884
2-(S-Glutathionyl)acetyl_chloride	0.590212	0.0254103	0.997884
S-(2-Chloroacetyl)glutathione	0.590212	0.0254103	0.997884
2-(S-Glutathionyl)acetyl_glutathione	0.590212	0.0254103	0.997884
S-(1,2-Dichlorovinyl)glutathione	0.590212	0.0254103	0.997884
GlutathionE_episulfonium_ion	0.590212	0.0254103	0.997884
S-(Formylmethyl)glutathione	0.590212	0.0254103	0.997884
Phosphatidylethanolamine	0.456238	0.784151	0.162573
14,15-EET	0.957414	0.855955	0.915483
8,9-EET	0.957414	0.855955	0.915483
5,6-EET	0.957414	0.855955	0.915483
Benzpyrene	0.986554	0.97742	0.919293
Benzo[a]pyrene-9,10-oxide	0.986554	0.97742	0.919293
Benzo[a]pyrene-7,8-dihydrodiol-9,10-oxide	0.986554	0.97742	0.919293
9-Hydroxybenzo[a]pyrene	0.986554	0.97742	0.919293
9-Hydroxybenzo[a]pyrene-4,5-oxide	0.986554	0.97742	0.919293
Pyruvate	0.0325231	0.0470621	0.218146
S-Adenosyl-L-homocysteine	0.613647	0.671863	0.443972
Ferricytochrome_c	0.766803	0.43137	0.956434
1-Organyl-2-lyso-sn-glycero-3-phosphocholine	0.569304	0.852708	0.241072
Methylglyoxal	0.214033	0.430824	0.0653136
2-Oxoglutarate	0.39845	0.172204	0.767056
2-Methoxyestradiol-17beta	0.244051	0.203596	0.502184
D-myo-Inositol_1,4,5-trisphosphate	0.924724	0.297498	0.989825
Phosphatidylcholine	0.337485	0.682891	0.13218
dGTP	0.0130858	0.00304418	0.488085
3-(2-Aminoethyl)-1H-indol-5-ol	0.324555	0.198037	0.649249
Acetate	0.755226	0.615472	0.761912
S-Adenosyl-L-methionine	0.590208	0.737442	0.25955
1-Phosphatidyl-D-myo-inositol_4,5-bisphosphate	0.640823	0.212799	0.858457

1-Acyl-sn-glycero-3-phosphocholine	0.269831	0.439281	0.225324
dATP	0.00893215	0.00304418	0.432815
GMP	0.380562	0.0144402	0.904475
dTTP	0.011121	0.00283694	0.504866
Trichloroethanol	0.704668	0.882159	0.392334
Testosterone	0.698389	0.941288	0.319274
3'-Phosphoadenylyl_sulfate	0.408092	0.814682	0.179457
3-Hydroxyanthranilate	0.337349	0.154809	0.761211
H2O2	0.851283	0.818544	0.69808
UMP	0.599776	0.0630911	0.888187
1-(1-Alkenyl)-sn-glycero-3-phosphoethanolamine	0.701065	0.663273	0.632059
O-1-Alk-1-enyl-2-acyl-sn-glycero-3-phosphoethanolamine	0.263109	0.604579	0.13218
NH3	0.369178	0.401765	0.408399
IDP	0.165263	0.0523896	0.842496
Tetrahydrofolate	0.37242	0.337279	0.483875
Succinate	0.192021	0.0997902	0.655327
L-Glutamine	0.968269	0.930097	0.874827
CMP-N-acetylneuraminate	0.583782	0.229982	0.82119
N-Acylsphingosine	0.167026	0.116918	0.348046
1-Acyl-sn-glycero-3-phosphoethanolamine	0.330314	0.553016	0.225324
dCTP	0.0119998	0.00753638	0.351769
L-Histidine	0.21113	0.101839	0.694497
Acetaldehyde	0.533008	0.49934	0.545615
Fatty_acid	0.0890885	0.202097	0.145145
L-Glutamate	0.845955	0.274622	0.974058
Naphthyl-2-oxomethyl-succinyl-CoA	0.953544	0.510633	0.988279
FADH2	0.274797	0.022063	0.992477
Lysophospholipid	0.394637	0.663273	0.225324
1-Radyl-2-acyl-sn-glycero-3-phosphocholine	0.394637	0.663273	0.225324
Glutathione	0.357081	0.171849	0.833256
16(R)-HETE	0.982206	0.886482	0.961038
(S)-Methylmalonate_semialdehyde	0.252724	0.258679	0.427109
Acceptor	0.903885	0.836328	0.809095
(S)-3-Hydroxybutanoyl-CoA	0.832255	0.431573	0.972151
N-Acetyl-D-mannosamine	0.038034	0.0082206	0.535649
Adenosine_3',5'-bisphosphate	0.417613	0.807396	0.192576
ITP	0.136517	0.0673911	0.654735
Succinyl-CoA	0.57101	0.366549	0.786053
2,6-Dimethyl-5-methylene-3-oxo-heptanoyl-CoA	0.906154	0.261196	0.988279
N(pi)-Methyl-L-histidine	0.162654	0.0768533	0.692805
N-Methyltyramine	0.162654	0.0768533	0.692805
Hordenine	0.162654	0.0768533	0.692805
2-Hydroxyestradiol-17beta	0.162654	0.0768533	0.692805
CH3-R	0.162654	0.0768533	0.692805
2-Hydroxypropylphosphonate	0.162654	0.0768533	0.692805
Methylcobalamin	0.162654	0.0768533	0.692805
1-Phenanthrol	0.162654	0.0768533	0.692805

1-Methoxyphenanthrene	0.162654	0.0768533	0.692805
Reduced_acceptor	0.815221	0.742222	0.744522
Octadecanoic_acid	0.328438	0.24448	0.610069
Tetradecanoyl-CoA	0.19171	0.382817	0.166834
Hexadecanoic_acid	0.930552	0.820641	0.892759
Hexanoyl-CoA	0.283274	0.153746	0.65526
beta-D-Fructose	0.021427	0.00686287	0.380084
Phosphatidate	0.34938	0.223605	0.602013
sn-Glycerol_3-phosphate	0.977316	0.856257	0.972151
L-Arginine	0.31112	0.0628914	0.767355
Glutaryl-CoA	0.844198	0.429779	0.972151
Androst-4-ene-3,17-dione	0.854114	0.93062	0.680304
Dolichyl_phosphate	0.154139	0.555028	0.0660028
Estradiol-17beta	0.394046	0.691696	0.149532
Estrone	0.394046	0.691696	0.149532
Indole-3-acetaldehyde	0.656157	0.816155	0.320102
1,2-Dihydronaphthalene-1,2-diol	0.562414	0.886507	0.162125
Naphthalene-1,2-diol	0.562414	0.886507	0.162125
3-Keto-beta-D-galactose	0.124806	0.36085	0.03117
beta-D-Fructose_6-phosphate	0.748137	0.432972	0.867569
beta-Alanine	0.564519	0.764059	0.312258
Adenosine	0.693741	0.542466	0.696081
1,2-Diacyl-sn-glycerol	0.165324	0.466515	0.0755056
Decanoyl-CoA	0.16223	0.382817	0.116326
alpha-D-Glucose	0.502315	0.663812	0.202317
Tetradecanoic_acid	0.800376	0.820641	0.554743
FAD	0.191761	0.0676588	0.755052
Xanthosine_5'-phosphate	0.620133	0.721985	0.519125
trans-Hex-2-enoyl-CoA	0.0906412	0.0154094	0.804535
Progesterone	0.943734	0.926206	0.828889
3-Isopropylbut-3-enoyl-CoA	0.992739	0.617014	0.998213
Aldehyde	0.589355	0.484419	0.632408
3alpha,7alpha-Dihydroxy-5beta-cholestan-26-al	0.589355	0.484419	0.632408
3-Chloroallyl_aldehyde	0.589355	0.484419	0.632408
cis-3-Chloroallyl_aldehyde	0.589355	0.484419	0.632408
Ecgonine_methyl_ester	0.90749	0.917991	0.628743
3,4-Dihydroxymandelaldehyde	0.530408	0.522535	0.521386
Parathion	0.849249	0.78414	0.749082
Paraoxon	0.849249	0.78414	0.749082
20-HETE	0.973125	0.859145	0.946585
Naphthalene	0.414867	0.782211	0.162125
1,2-Dihydroxy-3,4-epoxy-1,2,3,4-tetrahydronaphthalene	0.414867	0.782211	0.162125
beta-Naphthol	0.414867	0.782211	0.162125
Trichloroacetate	0.463174	0.74704	0.279848
UDP-D-galacturonate	0.10564	0.370848	0.0208226
dTDP-glucose	0.10564	0.370848	0.0208226
dTDP-D-glucuronate	0.10564	0.370848	0.0208226

2-Dehydro-3-deoxy-D-xylonate	0.10564	0.370848	0.0208226
(4S)-5-Hydroxy-2,4-dioxopentanoate	0.10564	0.370848	0.0208226
13(S)-HODE	0.10564	0.370848	0.0208226
13-OxoODE	0.10564	0.370848	0.0208226
beta-D-Galactose	0.143649	0.329206	0.025922
Ethylene_oxide	0.118791	0.285631	0.025922
4-Aminobutanoate	0.449139	0.737987	0.182952
Acyl-CoA	0.82039	0.686852	0.816039
Octanoyl-CoA	0.257617	0.382817	0.240107
Lauroyl-CoA	0.257617	0.382817	0.240107
Uridine	0.937266	0.342038	0.961555
N-Acetyl-D-mannosamine_6-phosphate	0.0375716	0.0073084	0.644424
trans-Hexadec-2-enoyl-CoA	0.0507421	0.0489938	0.352287
Cortisol	0.943828	0.782021	0.91692
L-Proline	0.493988	0.147379	0.927364
17alpha-Hydroxyprogesterone	0.961637	0.926206	0.873892
5-Hydroxyindoleacetaldehyde	0.460827	0.573345	0.344822
D-Glyceraldehyde	0.221096	0.21727	0.427109
1-Nitronaphthalene	0.521225	0.913111	0.162125
Formaldehyde	0.513014	0.392289	0.625385
Glycine	0.496738	0.293537	0.754318
Nicotinate_d-ribonucleotide	0.790513	0.903925	0.644986
4-Aminobutanal	0.696897	0.809835	0.385732
Imidazole-4-acetaldehyde	0.696897	0.809835	0.385732
beta-Aminopropion_aldehyde	0.696897	0.809835	0.385732
Hydroxyacetone	0.159895	0.370848	0.025922
Sorbitol_6-phosphate	0.159895	0.370848	0.025922
Sorbose_1-phosphate	0.159895	0.370848	0.025922
Butanal	0.159895	0.370848	0.025922
1-Butanol	0.159895	0.370848	0.025922
3alpha,7alpha,24-Trihydroxy-5beta-cholestanoyl-CoA	0.159895	0.370848	0.025922
3alpha,7alpha-Dihydroxy-5beta-24-oxocholestanoyl-CoA	0.159895	0.370848	0.025922
3alpha,7alpha,12alpha,24-Tetrahydroxy-5beta-cholestanoyl-CoA	0.159895	0.370848	0.025922
3alpha,7alpha,12alpha-Trihydroxy-5beta-24-oxocholestanoyl-CoA	0.159895	0.370848	0.025922
1-(4'-Hydroxyphenyl)ethanol	0.159895	0.370848	0.025922
4'-Hydroxyacetophenone	0.159895	0.370848	0.025922
L-Cysteine	0.211396	0.137556	0.539727
Deoxyadenosine	0.913465	0.991061	0.582108
4-Hydroxyphenylacetaldehyde	0.639387	0.857462	0.286375
Phenylacetaldehyde	0.639387	0.857462	0.286375
3,4-Dihydroxyphenylacetaldehyde	0.639387	0.857462	0.286375
Galactosylceramide	0.721391	0.612589	0.817222
Protein_lysine	0.173568	0.094378	0.564023
beta-D-Fructose_2-phosphate	0.0467171	0.00799304	0.644424
Thiamin	0.0467171	0.00799304	0.644424
Thiamin_monophosphate	0.0467171	0.00799304	0.644424
N1-(5-Phospho-alpha-D-ribosyl)-5,6-dimethylbenzimidazole	0.0467171	0.00799304	0.644424

alpha-Ribazole	0.0467171	0.00799304	0.644424
5-Amino-6-(5'-phosphoribitylamino)uracil	0.0467171	0.00799304	0.644424
4-(1-D-Ribitylamino)-5-amino-2,6-dihydroxypyrimidine	0.0467171	0.00799304	0.644424
2-Acyl-sn-glycerol_3-phosphate	0.969767	0.771243	0.972151
2-Naphthoyl-CoA	0.969767	0.771243	0.972151
3-Hydroxy-5-oxohexanoyl-CoA	0.969767	0.771243	0.972151
2-Naphthoic_acid	0.969767	0.771243	0.972151
trans-Oct-2-enoyl-CoA	0.0651588	0.0632102	0.352287
2-trans-Dodecenoyl-CoA	0.0651588	0.0632102	0.352287
trans-Tetradec-2-enoyl-CoA	0.0651588	0.0632102	0.352287
trans-Dec-2-enoyl-CoA	0.0651588	0.0632102	0.352287
L-Tyrosine	0.529119	0.819117	0.0757354
Benzoyl_acetyl-CoA	0.865661	0.497703	0.919861
S-Benzoate_coenzyme_a	0.865661	0.497703	0.919861
4-(2-Aminoethyl)-1,2-benzenediol	0.70645	0.915702	0.352408
6-Oxo-2-hydroxycyclohexane-1-carboxyl-CoA	0.95048	0.318937	0.992891
Propanoyl-CoA	0.295954	0.376866	0.246032
UDP-D-glucuronate	0.598616	0.194448	0.887843
4-Nitrophenyl_phosphate	0.715792	0.691341	0.620766
4-Nitrophenol	0.715792	0.691341	0.620766
3-Methylcrotonyl-CoA	0.11554	0.153635	0.246032
Pregnenolone	0.960753	0.926206	0.865438
17alpha-Hydroxypregnenolone	0.960753	0.926206	0.865438
GDP-L-fucose	0.552614	0.314462	0.705977
Glutathione_disulfide	0.76798	0.633379	0.879229
N4-Acetylaminobutanal	0.406892	0.513635	0.344822
Chloral_hydrate	0.562406	0.458506	0.599424
3-Oxotetradecanoyl-CoA	0.0674962	0.203477	0.0685755
dADP	0.296929	0.0602056	0.836156
dGDP	0.296929	0.0602056	0.836156
Nicotinate_d-ribonucleoside	0.644986	#N/A	0.644986
D-Galactose	0.139982	0.188184	0.2443
Deoxyguanosine	0.879835	0.99537	0.582108
Triacylglycerol	0.197579	0.655894	0.0389609
Inosine	0.682367	0.772886	0.582108
Octadecanoyl-[acyl-carrier_protein]	0.270754	0.153256	0.68254
5,10-Methylenetetrahydrofolate	0.484858	0.654118	0.141714
Mercaptopyruvate	0.0164471	0.00830327	0.588147
Tyramine	0.648991	0.915702	0.246475
Cytidine	0.860596	0.342038	0.909208
Deoxycytidine	0.860596	0.342038	0.909208
Formate	0.881323	0.781699	0.813334
Sphingosine_1-phosphate	0.118495	0.318465	0.123816
Sphingosine	0.118495	0.318465	0.123816
dTMP	0.596926	0.0680774	0.808628
L-Alanine	0.406075	0.406075	#N/A
L-Tryptophan	0.338991	0.880556	0.0583072

4-Hydroxyphenylacetyl-CoA	0.925349	0.497703	0.972151
4-Hydroxyphenylacetylglycine	0.925349	0.497703	0.972151
3-Oxopimeloyl-CoA	0.925349	0.497703	0.972151
Phenylacetyl-CoA	0.925349	0.497703	0.972151
Phenylacetylglycine	0.925349	0.497703	0.972151
trans-4-Hydroxy-L-proline	0.272584	0.109302	0.921382
1-Acyl-sn-glycerol_3-phosphate	0.730294	0.513033	0.816039
11-Deoxycortisol	0.893906	0.782021	0.837481
11-Deoxycorticosterone	0.893906	0.782021	0.837481
17alpha,21-Dihydroxypregnenolone	0.893906	0.782021	0.837481
Dolichyl_phosphate_d-mannose	0.241532	0.614884	0.118811
UDP-glucuronate	0.50427	0.792071	0.212335
Perillic_acid	0.300142	0.304342	0.427109
3-Phospho-D-glycerate	0.481695	0.188214	0.969083
3alpha,7alpha-Dihydroxy-5beta-cholestanate	0.483782	0.459345	0.517822
4-Trimethylammoniobutanoate	0.229014	0.459345	0.171631
D-Glycerate	0.362357	0.381535	0.427109
Methylmalonate	0.48509	0.533543	0.427109
5-Hydroxyindoleacetate	0.48509	0.533543	0.427109
TCE_epoxide	0.56835	0.838679	0.312773
Chloral	0.56835	0.838679	0.312773
Ferricytochrome_b5	0.393743	0.287323	0.628147
(S)-3-Hydroxyhexadecanoyl-CoA	0.127288	0.203477	0.174235
(S)-3-Hydroxytetradecanoyl-CoA	0.127288	0.203477	0.174235
Phosphoenolpyruvate	0.667811	0.624587	0.619019
Starch	0.704282	0.894039	0.201678
L-Phenylalanine	0.547135	0.89061	0.0624493
Poly-beta-hydroxybutyrate	0.783607	0.831732	0.500036
((R)-3-Hydroxybutanoyl)(n-2)	0.783607	0.831732	0.500036
Ecgonine	0.783607	0.831732	0.500036
Guanosine	0.582108	#N/A	0.582108
N-Ribosylnicotinamide	0.582108	#N/A	0.582108
Xanthosine	0.582108	#N/A	0.582108
Tryptamine	0.817916	0.915702	0.463076
Phenethylamine	0.817916	0.915702	0.463076
Lauric	0.882284	0.617153	0.916333
acid	0.882284	0.617153	0.916333
Thymidine	0.946537	0.99537	0.726998
1-Acylglycerol	0.0599471	0.330913	0.0389609
Nicotinamide_d-ribonucleotide	0.482161	#N/A	0.482161
CDP	0.454904	0.27483	0.742242
5,10-Methenyltetrahydrofolate	0.6896	0.729917	0.534873
Cholesterol	0.964593	0.883622	0.925958
dCMP	0.549763	#N/A	0.549763
L-Methionine	0.859802	0.696762	0.844292
Sphinganine	0.14141	0.217865	0.229651
L-Cysteate	0.880051	0.662721	0.973954

L-Aspartate	0.210616	0.100102	0.556919
2-Aminoacrylate	0.758061	0.818304	0.532565
10-Formyltetrahydrofolate	0.515261	0.465123	0.556384
UDP-glucose	0.279661	0.044716	0.547435
Oxaloacetate	0.216956	0.216956	#N/A
dGMP	0.750374	#N/A	0.750374
1D-myo-Inositol_1,4-bisphosphate	0.856739	0.468885	0.943645
1D-myo-Inositol_1,3,4-trisphosphate	0.856739	0.468885	0.943645
p-Benzoquinone	0.425539	0.157497	0.684491
5(S)-HETE	0.725366	0.552893	0.879229
(15S)-15-Hydroxy-5,8,11-cis-13-trans-eicosatetraenoate	0.725366	0.552893	0.879229
Tetrahydrobiopterin	0.631354	0.876532	0.145352
3beta-Hydroxyandrost-5-en-17-one	0.792927	0.902578	0.533576
16alpha-Hydroxydehydroepiandrosterone	0.792927	0.902578	0.533576
1D-myo-Inositol_1,3,4,5-tetrakisphosphate	0.92523	0.674736	0.943645
Dolichyl_diphosphate	0.325284	0.325284	#N/A
11beta,17alpha,21-Trihydroxypregnenolone	0.937726	0.941084	0.837481
Maleic_acid	0.708651	0.800737	0.293849
Maleamate	0.708651	0.800737	0.293849
N-Cyclohexylformamide	0.708651	0.800737	0.293849
Cyclohexylamine	0.708651	0.800737	0.293849
21-Hydroxypregnenolone	0.697822	0.782021	0.533576
Estrone_glucuronide	0.596916	0.792071	0.271461
Androsterone	0.596916	0.792071	0.271461
Androsterone_glucuronide	0.596916	0.792071	0.271461
Testosterone_glucuronide	0.596916	0.792071	0.271461
Estradiol-17beta_3-glucuronide	0.596916	0.792071	0.271461
3alpha-Hydroxy-5beta-androstan-17-one	0.596916	0.792071	0.271461
Etiocholan-3alpha-ol-17-one_3-glucuronide	0.596916	0.792071	0.271461
2-Methoxyestrone	0.596916	0.792071	0.271461
2-Methoxyestrone_3-glucuronide	0.596916	0.792071	0.271461
2-Methoxy-estradiol-17beta_3-glucuronide	0.596916	0.792071	0.271461
Trichloroethanol_glucuronide	0.596916	0.792071	0.271461
Ethanol	0.73949	0.616569	0.721369
Indole-3-acetate	0.422587	0.459345	0.427109
2-Propyn-1-al	0.422587	0.459345	0.427109
Propynoate	0.422587	0.459345	0.427109
D-Glucuronolactone	0.422587	0.459345	0.427109
D-Glucarate	0.422587	0.459345	0.427109
4-Trimethylammoniobutanal	0.422587	0.459345	0.427109
Imidazole-4-acetate	0.422587	0.459345	0.427109
4-Acetamidobutanoate	0.422587	0.459345	0.427109
3-Butyn-1-al	0.422587	0.459345	0.427109
3-Butynoate	0.422587	0.459345	0.427109
trans-3-Chloroacrylic_acid	0.422587	0.459345	0.427109
cis-3-Chloroacrylic_acid	0.422587	0.459345	0.427109
Perillyl_aldehyde	0.422587	0.459345	0.427109

3alpha,7alpha,26-Trihydroxy-5beta-cholestane	0.757229	0.6497	0.721369
Glycerol	0.092986	0.092986	#N/A
Leukotriene_b4	0.927297	0.916048	0.826202
20-OH-Leukotriene_b4	0.927297	0.916048	0.826202
Icosanoic_acid	0.0846654	0.0440646	0.632159
Docosanoic_acid	0.0846654	0.0440646	0.632159
Tetracosanoic_acid	0.0846654	0.0440646	0.632159
Hexacosanoic_acid	0.0846654	0.0440646	0.632159
19(S)-HETE	0.390749	0.557342	0.351188
D-Sorbitol	0.160473	0.244804	0.20091
Glycerone_phosphate	0.304352	0.304352	#N/A
Uracil	0.94534	0.99537	0.688152
Bromobenzene	0.429797	0.838679	0.150181
4-Bromophenol	0.429797	0.838679	0.150181
4-Bromophenol-2,3-epoxide	0.429797	0.838679	0.150181
Dihydroceramide	0.0498271	#N/A	0.0498271
3-Methoxy-4-hydroxyphenylacetaldehyde	0.346033	0.499777	0.304712
3-Methoxy-4-hydroxyphenylglycolaldehyde	0.346033	0.499777	0.304712
Methylimidazole_acetaldehyde	0.346033	0.499777	0.304712
Estriol	0.26581	0.458137	0.194699
16alpha-Hydroxyestrone	0.26581	0.458137	0.194699
Stearoyl-CoA	0.169454	0.118624	0.589185
4-Sulfolactone	0.623465	0.657747	0.500036
HSO3-	0.623465	0.657747	0.500036
Crotonoyl-CoA	0.0163005	0.0163005	#N/A
(S)-Malate	0.0247521	0.117996	0.02147
Butanoic_acid	0.930707	0.713589	0.950786
Hexanoic_acid	0.930707	0.713589	0.950786
Octanoic_acid	0.930707	0.713589	0.950786
Decanoic_acid	0.930707	0.713589	0.950786
(S)-3-Hydroxyisobutyrate	0.204684	0.204684	#N/A
3-Oxopalmitoyl-CoA	0.203477	0.203477	#N/A
(S)-3-Hydroxydodecanoyl-CoA	0.203477	0.203477	#N/A
3-Oxododecanoyl-CoA	0.203477	0.203477	#N/A
(S)-Hydroxydecanoyl-CoA	0.203477	0.203477	#N/A
3-Oxodecanoyl-CoA	0.203477	0.203477	#N/A
(S)-Hydroxyoctanoyl-CoA	0.203477	0.203477	#N/A
3-Oxooctanoyl-CoA	0.203477	0.203477	#N/A
(S)-Hydroxyhexanoyl-CoA	0.203477	0.203477	#N/A
3-Oxohexanoyl-CoA	0.203477	0.203477	#N/A
Putrescine	0.570372	0.900095	0.0985312
Guanine	0.512138	0.850241	0.110726
dAMP	0.726998	#N/A	0.726998
Corticosterone	0.890052	0.322191	0.945475
Aminoacetone	0.77762	0.915702	0.316522
Melatonin	0.401268	0.938302	0.0570031
Oleoyl-[acyl-carrier_protein]	0.58656	0.508619	0.672374

Dihydrobiopterin	0.401925	0.706034	0.145352
Malonyl-CoA	0.129074	0.3012	0.124941
(S)-1-Phenylethanol	0.631451	0.157497	0.895052
e-	0.631451	0.157497	0.895052
2,6-Dihydroxycyclohexane-1-carboxyl-CoA	0.631451	0.157497	0.895052
2-Hydroxy-4-isopropenylcyclohexane-1-carboxyl-CoA	0.631451	0.157497	0.895052
4-Isopropenyl-2-oxy-cyclohexanecarboxyl-CoA	0.631451	0.157497	0.895052
Myrtenol	0.631451	0.157497	0.895052
Myrtenal	0.631451	0.157497	0.895052
Pinocarvone	0.631451	0.157497	0.895052
3-Hydroxy-2,6-dimethyl-5-methylene-heptanoyl-CoA	0.631451	0.157497	0.895052
4,4'-Dihydroxy-alpha-methylstilbene	0.631451	0.157497	0.895052
9,10-Dihydroxyanthracene	0.631451	0.157497	0.895052
2-Acetyl-1-alkyl-sn-glycero-3-phosphocholine	0.777247	0.914324	0.447816
Sphinganine_1-phosphate	0.152934	0.237846	0.229651
Ceramide_1-phosphate	0.152934	0.237846	0.229651
dUDP	0.432275	0.164044	0.823387
dCDP	0.432275	0.164044	0.823387
Adenine	0.849123	0.942477	0.199634
G12336	0.216045	0.22849	0.393825
Fe3+	0.639744	0.604934	0.605753
Lauric_acid	0.814994	0.830636	0.536539
2-Methylprop-2-enoyl-CoA	0.0126876	0.0126876	#N/A
2-Methylbut-2-enoyl-CoA	0.0126876	0.0126876	#N/A
1H-Imidazole-4-ethanamine	0.956882	0.980254	0.576102
FMN	0.880087	0.875597	0.717932
Riboflavin	0.880087	0.875597	0.717932
4-Methyl-2-oxopentanoate	0.0599952	0.0196291	0.52621
3-Methyl-2-oxobutanoic_acid	0.0599952	0.0196291	0.52621
Protein_N6-methyl-L-lysine	0.22685	0.161084	0.511007
2-Oxobutanoate	0.145459	0.0150112	0.838286
Protein_asparagine	0.381704	0.381704	#N/A
Androst-5-ene-3beta,17beta-diol	0.780522	0.941084	0.533576
16alpha-Hydroxyandrost-4-ene-3,17-dione	0.780522	0.941084	0.533576
Sphingomyelin	0.454993	0.165762	0.741108
L-Serine	0.559949	0.559949	#N/A
Oxidized_thioredoxin	0.903348	0.70012	0.89467
Thioredoxin	0.903348	0.70012	0.89467
Butanoyl-CoA	0.48794	0.0639266	0.96544
Amylose	0.894039	0.894039	#N/A
Retinal	0.428673	0.619206	0.351029
Phenylpyruvate	0.48455	0.706805	0.153765
3-(4-Hydroxyphenyl)pyruvate	0.48455	0.706805	0.153765
L-Kynurenine	0.21373	0.357679	0.240097
3-Hydroxy-L-kynurenine	0.21373	0.357679	0.240097
Primary_alcohol	0.711411	0.539395	0.721369
3,4-Dihydroxyphenylethyleneglycol	0.711411	0.539395	0.721369

1-Hydroxymethylnaphthalene	0.711411	0.539395	0.721369
1-Naphthaldehyde	0.711411	0.539395	0.721369
(2-Naphthyl)methanol	0.711411	0.539395	0.721369
2-Naphthaldehyde	0.711411	0.539395	0.721369
Acetoacetate	0.795412	0.751317	0.682988
Isocitrate	0.744429	0.204941	0.960274
Cyromazine	0.675723	0.3631	0.76685
N-Cyclopropylammeline	0.675723	0.3631	0.76685
N-Cyclopropylammelide	0.675723	0.3631	0.76685
Cyanuric_acid	0.675723	0.3631	0.76685
Glycolate	0.159211	0.153923	0.347633
3-Oxostearoyl-[acp]	0.257998	0.714857	0.0685755
Xanthine	0.320064	0.783974	0.160321
L-Citrulline	0.887534	0.674356	0.950117
5-Methyl-3-oxo-4-hexenoyl-CoA	0.19132	0.270328	0.246032
Prostaglandin_F2alpha	0.0316412	0.0308267	0.316165
Prostaglandin_E2	0.0316412	0.0308267	0.316165
D-Glucono-1,5-lactone_6-phosphate	0.0799143	0.494264	0.0241754
Acetoacetyl-CoA	0.161897	0.161897	#N/A
(2S,3S)-3-Hydroxy-2-methylbutanoyl-CoA	0.161897	0.161897	#N/A
2-Methylacetoacetyl-CoA	0.161897	0.161897	#N/A
(3S)-3-Hydroxyadipyl-CoA	0.161897	0.161897	#N/A
3-Oxoadipyl-CoA	0.161897	0.161897	#N/A
Hypoxanthine	0.898932	0.991679	0.277681
Xylitol	0.417294	0.157831	0.906808
GM2	0.475603	0.628913	0.2443
G06780	0.475603	0.628913	0.2443
Glyoxylate	0.170708	0.169992	0.347633
myo-Inositol_hexakisphosphate	0.645517	0.297023	0.952654
1D-myo-Inositol_1,3,4,5,6-pentakisphosphate	0.645517	0.297023	0.952654
Biliverdin	0.725933	0.335005	0.97387
Glucosylceramide	0.646787	0.798046	0.2443
15H-11,12-EETA	0.541215	#N/A	0.541215
Ferrocytochrome_b5	0.564549	0.492233	0.640291
Nicotinamide	0.35926	#N/A	0.35926
p-Benzenediol	0.263165	0.486986	0.112821
L-erythro-4-Hydroxyglutamate	0.25873	0.25873	#N/A
G00011	0.300368	0.456754	0.263791
G00012	0.300368	0.456754	0.263791
1-Phosphatidyl-1D-myo-inositol_4-phosphate	0.231946	0.567236	0.114001
Selenomethionine	0.528924	0.314011	0.721505
3-Phospho-D-glyceroyl_phosphate	0.989017	0.887891	0.978624
Deoxyuridine	0.949178	0.939621	0.777733
Deamino-NAD+	0.46085	0.881668	0.212972
Methanol	0.467041	0.23946	0.855846
Dodecanoic_acid	0.703326	0.713589	0.536539
(S)-3-Methyl-2-oxopentanoic_acid	0.00651982	0.0196291	0.081273

G13043	0.294821	0.320105	0.393825
Hexadecanoyl-[acp]	0.516696	0.714857	0.305634
Acyl-carrier_protein	0.516696	0.714857	0.305634
cis-2-Methyl-5-isopropylhexa-2,5-dienoyl-CoA	0.0188171	0.0188171	#N/A
trans-2-Methyl-5-isopropylhexa-2,5-dienoyl-CoA	0.0188171	0.0188171	#N/A
L-Isoleucine	0.727107	0.214371	0.875816
D-Sedoheptulose_7-phosphate	0.445363	0.485382	0.437387
D-Erythrose_4-phosphate	0.445363	0.485382	0.437387
GM4	0.793094	0.66344	0.817222
(S)-Lactate	0.038569	0.0150112	0.588147
2-Hydroxybutanoic_acid	0.038569	0.0150112	0.588147
3-Mercaptolactate	0.038569	0.0150112	0.588147
L-Ornithine	0.291329	0.169785	0.570512
Octanoyl-[acp]	0.553627	0.353044	0.822185
L-1-Pyrroline-3-hydroxy-5-carboxylate	0.0459663	0.0459663	#N/A
Cys-Gly	0.481899	0.367878	0.556611
Peptide	0.585959	0.367878	0.672059
5-Hydroxy-L-tryptophan	0.687021	0.880919	0.312129
1D-myo-Inositol_3-phosphate	0.490238	0.12578	0.97372
myo-Inositol	0.102785	0.102785	#N/A
dTDP	0.0698677	0.0153549	0.78502
dUMP	0.0810629	0.0727735	0.300721
dUTP	0.0578077	0.0231049	0.620083
Diacylglycerol	0.711175	0.775155	0.512773
Cocaine	0.882524	0.853746	0.715479
3-Oxo-(7Z,10Z,13Z,16Z)-Docosatetraenoyl-CoA	0.718145	0.914664	0.174235
3-Oxo-(8Z,11Z,14Z)-Icosatrienoyl-CoA	0.718145	0.914664	0.174235
3-Oxo-(7Z,10Z,13Z,16Z,19Z)-Docosapentaenoyl-CoA	0.718145	0.914664	0.174235
Retinoate	0.256811	0.619206	0.15516
2,3-Bisphospho-D-glycerate	0.160549	0.160549	#N/A
D-Tagatose_6-phosphate	0.567075	0.567075	#N/A
D-Tagatose_1,6-bisphosphate	0.567075	0.567075	#N/A
11H-14,15-EETA	0.589429	#N/A	0.589429
3-Oxoicosanoyl-CoA	0.360261	0.58881	0.174235
3-Oxodocosanoyl-CoA	0.360261	0.58881	0.174235
3-Oxotetracosanoyl-CoA	0.360261	0.58881	0.174235
3-Oxostearoyl-CoA	0.12121	0.530756	0.0685755
Sarcosine	0.804669	0.75181	0.715992
S-Adenosylmethioninamine	0.514926	0.589506	0.45463
omega-Hydroxy_fatty_acid	0.916212	#N/A	0.916212
Fumarate	0.387114	0.150226	0.833476
11-Dehydrocorticosterone	0.750822	#N/A	0.750822
(2E)-Octadecenoyl-CoA	0.00217935	0.00985021	0.0325711
(2E)-Octadecenoyl-[acp]	0.00217935	0.00985021	0.0325711
Diethylthiophosphoric_acid	0.486986	0.486986	#N/A
Diethylphosphoric_acid	0.486986	0.486986	#N/A
4-Hydroxyphenyl_acetate	0.486986	0.486986	#N/A

Cholesta-5,7-dien-3beta-ol	0.841465	#N/A	0.841465
Leukotriene_a4	0.186575	0.36394	0.146584
5-Hydroxykynurenamine	0.575096	0.596982	0.523344
14-Demethyllanosterol	0.18294	0.328008	0.213947
Reduced_flavoprotein	0.154233	0.831061	0.0271199
6-Hydroxymelatonin	0.154233	0.831061	0.0271199
Oxidized_flavoprotein	0.154233	0.831061	0.0271199
OPC6-CoA	0.423925	0.423322	0.460871
OPC4-CoA	0.423925	0.423322	0.460871
Choline	0.86735	0.105418	0.987749
N-Acetyl-D-galactosamine	0.595924	0.691065	0.387896
Phenol	0.297023	0.297023	#N/A
Phenolic_phosphate	0.297023	0.297023	#N/A
7alpha,12alpha-Dihydroxycholest-4-en-3-one	0.533264	0.702214	0.392515
5'-Methylthioadenosine	0.774932	0.868358	0.392187
Procollagen_5-hydroxy-L-lysine	0.284852	0.193163	0.595023
G13032	0.340946	0.446124	0.342268
(S)-3-Hydroxy-3-methylglutaryl-CoA	0.976735	0.989401	0.791011
Galactitol	0.157831	0.157831	#N/A
D-Xylose	0.157831	0.157831	#N/A
L-Arabitol	0.157831	0.157831	#N/A
L-Arabinose	0.157831	0.157831	#N/A
Lactaldehyde	0.157831	0.157831	#N/A
Propane-1,2-diol	0.157831	0.157831	#N/A
beta-D-Galactosyl-1,4-beta-D-glucosylceramide	0.382051	0.54853	0.2443
Octadecanoyl-CoA	0.622944	0.58881	0.589185
Tetracosanoyl-CoA	0.622944	0.58881	0.589185
Se-Adenosylselenohomocysteine	0.579513	0.275134	0.747973
Deoxyinosine	0.926004	0.991061	0.199634
3-beta-D-Galactosyl-sn-glycerol	0.392683	0.561969	0.2443
L-Leucine	0.118618	0.322488	0.081273
Oleoyl-CoA	0.492233	0.492233	#N/A
Phenylacetic_acid	0.343227	0.402764	0.374332
4-Hydroxyphenylacetate	0.343227	0.402764	0.374332
3,4-Dihydroxyphenylacetate	0.343227	0.402764	0.374332
3,4-Dihydroxymandelate	0.343227	0.402764	0.374332
Homovanillate	0.343227	0.402764	0.374332
3-Methoxy-4-hydroxymandelate	0.343227	0.402764	0.374332
Methylimidazoleacetic_acid	0.343227	0.402764	0.374332
L-Asparagine	0.0881336	0.267971	0.071039
3-Hydroxy-5-methylhex-4-enoyl-CoA	0.270328	0.270328	#N/A
N-(omega)-Hydroxyarginine	0.743033	0.358069	0.950117
Nitric_oxide	0.743033	0.358069	0.950117
UDP-D-xylose	0.479036	0.944501	0.116882
L-Valine	0.126623	0.340001	0.081273
3-Dehydrosphinganine	0.906348	0.665531	0.953587
11beta-Hydroxyandrost-4-ene-3,17-dione	0.978261	#N/A	0.978261

3-Methyl-2-oxopentanoate	0.343481	0.214371	0.52621
beta-D-Fructose_2,6-bisphosphate	0.225337	0.16131	0.536716
D-Glucosamine_6-phosphate	0.660266	0.338315	0.904296
(2R)-2-Hydroxy-3-(phosphonooxy)-propanal	0.798957	0.625624	0.841181
Propenoyl-CoA	0.06996	0.06996	#N/A
5-Carboxy-2-pentenoyl-CoA	0.06996	0.06996	#N/A
L-Noradrenaline	0.577281	0.596982	0.526065
Sulfate	0.368112	0.445093	0.37639
2-Acyl-1-alkyl-sn-glycero-3-phosphate	0.316412	0.237846	0.571899
1-Alkyl-2-acylglycerol	0.316412	0.237846	0.571899
2-Oxoadipate	0.500588	0.46301	0.553333
Ethanolamine	0.0566355	0.131742	0.122474
trans,trans-Farnesyl_diphosphate	0.328	0.622487	0.221803
Pantetheine_4'-phosphate	0.176757	0.12255	0.37639
DWA-2	0.320105	0.320105	#N/A
sn-glycero-3-Phosphocholine	0.0343113	0.0343113	#N/A
Citrate	0.695644	0.76838	0.440235
Selenide	0.412259	0.279361	0.671169
Fe2+	0.446821	0.361938	0.605753
L-Glutamate_5-semialdehyde	0.0637687	0.1045	0.193885
Alcohol	0.580836	0.727625	0.307449
(2E,6Z,9Z,12Z,15Z,18Z,21Z)-Tetracosahepta-2,6,9,12,15,18,21-enoyl-CoA	0.607661	0.479023	0.708212
(2E,6Z,9Z,12Z,15Z,18Z)-Tetracosahexa-2,6,9,12,15,18-enoyl-CoA	0.607661	0.479023	0.708212
1,3-Diaminopropane	0.863172	0.93488	0.403874
N-Methylputrescine	0.863172	0.93488	0.403874
1-Methylpyrrolinium	0.863172	0.93488	0.403874
Cadaverine	0.863172	0.93488	0.403874
5-Aminopentanal	0.863172	0.93488	0.403874
G00144	0.454898	#N/A	0.454898
2-Oxoarginine	0.605696	0.420215	0.681137
D-Ribose_5-phosphate	0.609173	0.759141	0.437387
Aminofructose_6-phosphate	0.609173	0.759141	0.437387
Iminoerythrose_4-phosphate	0.609173	0.759141	0.437387
Dodecanoyl-[acyl-carrier_protein]	0.764497	0.714857	0.672374
Tetradecanoyl-[acp]	0.764497	0.714857	0.672374
11beta-Hydroxyprogesterone	0.957244	0.748178	0.979047
21-Deoxycortisol	0.957244	0.748178	0.979047
(S)-1-Pyrroline-5-carboxylate	0.121372	0.121372	#N/A
D-myo-Inositol_3,4-bisphosphate	0.867155	0.497426	0.97372
Pyridoxal	0.676078	0.676078	#N/A
3-Isopropylbut-3-enoic_acid	0.988152	0.727737	0.990433
5-Methylhex-4-enoyl-CoA	0.487974	0.673198	0.246032
Taurine	0.171838	#N/A	0.171838
Hypotaurine	0.171838	#N/A	0.171838
(5-L-Glutamyl)-peptide	0.538531	0.367878	0.639508
Leukotriene_c4	0.538531	0.367878	0.639508
Leukotriene_d4	0.538531	0.367878	0.639508

R-S-Glutathione	0.538531	0.367878	0.639508
R-S-Alanylglycine	0.538531	0.367878	0.639508
3-Cyano-L-alanine	0.538531	0.367878	0.639508
gamma-Glutamyl-beta-cyanoalanine	0.538531	0.367878	0.639508
gamma-Glutamyl-beta-aminopropiononitrile	0.538531	0.367878	0.639508
2-Methylpropanoyl-CoA	0.290622	0.0419483	0.931851
(S)-2-Methylbutanoyl-CoA	0.290622	0.0419483	0.931851
Dolichyl_d-glucosyl_phosphate	0.264907	0.595114	0.17368
Protein_N6-(lipoyl)lysine	0.164591	0.072613	0.434175
myo-Inositol_4-phosphate	0.112662	0.112662	#N/A
N6-D-Biotinyl-L-lysine	0.894405	0.130237	0.990021
Dextrin	0.167811	#N/A	0.167811
3-Methylbutanoyl-CoA	0.462055	0.121101	0.931851
N6,N6,N6-Trimethyl-L-lysine	0.576971	0.0656155	0.993283
Protein_N6,N6,N6-trimethyl-L-lysine	0.576971	0.0656155	0.993283
beta-D-Fructose_1,6-bisphosphate	0.886457	0.651167	0.906692
beta-D-Glucose_6-phosphate	0.0241754	#N/A	0.0241754
Homogentisate	0.152228	0.332727	0.153765
3-Oxo-(8Z,11Z,14Z,17Z)-Icosatetraenoyl-CoA	0.462593	0.789542	0.174235
3-Oxo-(11Z,14Z,17Z)-Icosatrienoyl-CoA	0.462593	0.789542	0.174235
(5Z,8Z,11Z,14Z)-Icosatetraenoyl-CoA	0.914664	0.914664	#N/A
(5Z,8Z,11Z,14Z,17Z)-Icosapentaenoyl-CoA	0.914664	0.914664	#N/A
3-Phosphonooxypyruvate	0.68582	0.109465	0.972212
1,1-Dichloroethylene	0.405492	#N/A	0.405492
Nucleoside_triphosphate	0.233186	0.0787361	0.649211
NDP	0.233186	0.0787361	0.649211
(9Z,12Z,15Z)-Octadecatrienoyl-CoA	0.887482	0.887482	#N/A
3-Oxohexadecanoyl-CoA	0.0685755	#N/A	0.0685755
3-Hydroxyoctadecanoyl-CoA	0.271524	0.530756	0.174235
3-Hydroxyoctadecanoyl-[acp]	0.271524	0.530756	0.174235
5alpha-Cholest-7-en-3beta-ol	0.889647	#N/A	0.889647
Urate	0.15083	0.0707942	0.503458
Urocanate	0.6803	0.582894	0.674994
(R)-3-Hydroxybutanoate	0.85419	0.845217	0.682988
S-Aminomethyldihydrolipoylprotein	0.747393	0.71151	0.649903
Bilirubin	0.547708	0.0382596	0.97387
Formylanthranilate	0.797785	#N/A	0.797785
L-Formylkynurenine	0.797785	#N/A	0.797785
D-Glutamine	0.775827	0.71262	0.6956
D-Glutamate	0.775827	0.71262	0.6956
Agmatine	0.605088	0.605088	#N/A
L-Cystathionine	0.203391	0.203391	#N/A
Selenocystathionine	0.203391	0.203391	#N/A
Oxalosuccinate	0.314514	0.110288	0.70596
Oxooctadecanoyl-CoA	0.207335	0.504587	0.121916
L-Lysine	0.712945	#N/A	0.712945
Selenocysteine	0.129899	0.129899	#N/A

5,6-Dihydrouracil	0.550127	0.534861	0.536209
5,6-Dihydrothymine	0.550127	0.534861	0.536209
1,7-Dimethylxanthine	0.194084	#N/A	0.194084
Vitamin_K	0.03028	0.00933677	0.380897
Icosanoyl-CoA	0.58881	0.58881	#N/A
Dcosanoyl-CoA	0.58881	0.58881	#N/A
3-Oxohexacosanoyl-CoA	0.58881	0.58881	#N/A
Hydroxypyruvate	0.542991	0.542991	#N/A
Thymine	0.971508	0.99537	0.536209
Phytoceramide	0.0376821	#N/A	0.0376821
L-Adrenaline	0.447526	0.596982	0.332853
Methylamine	0.447526	0.596982	0.332853
N-Acetylputrescine	0.447526	0.596982	0.332853
N-Methylhistamine	0.447526	0.596982	0.332853
3-Methoxytyramine	0.447526	0.596982	0.332853
L-Normetanephrine	0.447526	0.596982	0.332853
L-Metanephrine	0.447526	0.596982	0.332853
3-Hydroxykynurenamine	0.447526	0.596982	0.332853
4,8-Dihydroxyquinoline	0.447526	0.596982	0.332853
4,6-Dihydroxyquinoline	0.447526	0.596982	0.332853
Chitobiose	0.691065	0.691065	#N/A
GM3	0.691065	0.691065	#N/A
G13033	0.691065	0.691065	#N/A
G08421	0.691065	0.691065	#N/A
(7Z,10Z,13Z,16Z)-Docosatetraenoyl-CoA	0.83161	0.871543	0.589185
(7Z,10Z,13Z,16Z,19Z)-Docosapentaenoyl-CoA	0.83161	0.871543	0.589185
Carbamoyl_phosphate	0.652786	0.652786	#N/A
3-Sulfopyruvate	0.326706	0.326706	#N/A
3-Sulfino-L-alanine	0.326706	0.326706	#N/A
3-Sulfinylpyruvate	0.326706	0.326706	#N/A
D-4-Hydroxy-2-oxoglutarate	0.326706	0.326706	#N/A
Cortisone	0.849829	#N/A	0.849829
Adrenosterone	0.849829	#N/A	0.849829
Tetrahydrocorticosterone	0.849829	#N/A	0.849829
3alpha,21-Dihydroxy-5beta-pregnane-11,20-dione	0.849829	#N/A	0.849829
Lanosterol	0.644377	#N/A	0.644377
Digalactosylceramide	0.68064	0.68064	#N/A
Ethanolamine_phosphate	0.151158	0.105418	0.41762
Dimethylallyl_diphosphate	0.0932946	0.424121	0.0467567
Geranyl_diphosphate	0.0932946	0.424121	0.0467567
Pseudoecgonine	0.0416332	0.0416332	#N/A
Pseudoecgonyl-CoA	0.0416332	0.0416332	#N/A
4-(2-Aminophenyl)-2,4-dioxobutanoate	0.435233	0.357679	0.553333
4-(2-Amino-3-hydroxyphenyl)-2,4-dioxobutanoate	0.435233	0.357679	0.553333
cis-Aconitate	0.781782	0.826787	0.563427
alpha-L-Fucoside	0.727625	0.727625	#N/A
4,4-Dimethyl-5alpha-cholesta-8,14,24-trien-3beta-ol	0.421348	0.328008	0.565442

4alpha-Methyl-5alpha-ergosta-8,14,24(28)-trien-3beta-ol	0.421348	0.328008	0.565442
4alpha-Methylzymosterol-4-carboxylate	0.0299816	#N/A	0.0299816
Galactan	0.279168	0.446124	0.2443
Lactose	0.279168	0.446124	0.2443
Lactose_6-phosphate	0.279168	0.446124	0.2443
Galactose	0.279168	0.446124	0.2443
3-Ketolactose	0.279168	0.446124	0.2443
GM1	0.279168	0.446124	0.2443
N-Acetylneuraminate	0.433805	0.275938	0.640291
Protoporphyrinogen_IX	0.0586998	0.0134421	0.499194
D-Glucuronate	0.110298	0.110298	#N/A
O-Phospho-L-serine	0.922909	0.540553	0.972212
Acetyl_phosphate	0.977526	0.833886	0.969083
Benzoyl_phosphate	0.977526	0.833886	0.969083
Benzoate	0.977526	0.833886	0.969083
G13035	0.700555	0.24867	0.922636
G13042	0.700555	0.24867	0.922636
CDP-choline	0.844914	0.555679	0.902511
Dihydrolipoylprotein	0.869165	0.869165	#N/A
trans-2,3-didehydroacyl-CoA	0.555523	0.555523	#N/A
trans,trans-2,3,4,5-tetradehydroacyl-CoA	0.555523	0.555523	#N/A
G00013	0.283447	0.182076	0.538807
G00014	0.283447	0.182076	0.538807
Acetyl-[acyl-carrier_protein]	0.714857	0.714857	#N/A
Acetoacetyl-[acp]	0.714857	0.714857	#N/A
Butyryl-[acp]	0.714857	0.714857	#N/A
3-Oxodecanoyl-[acp]	0.714857	0.714857	#N/A
3-Oxooctanoyl-[acp]	0.714857	0.714857	#N/A
3-Oxohexadecanoyl-[acp]	0.714857	0.714857	#N/A
3-Oxotetradecanoyl-[acp]	0.714857	0.714857	#N/A
3-Oxohexanoyl-[acp]	0.714857	0.714857	#N/A
Hexanoyl-[acp]	0.714857	0.714857	#N/A
Decanoyl-[acp]	0.714857	0.714857	#N/A
3-Oxododecanoyl-[acp]	0.714857	0.714857	#N/A
N,N-Dimethylglycine	0.704086	0.75181	0.53131
OPC8-CoA	0.598957	0.423322	0.708212
trans-2-Enoyl-OPC8-CoA	0.598957	0.423322	0.708212
trans-2-Enoyl-OPC6-CoA	0.598957	0.423322	0.708212
trans-2-Enoyl-OPC4-CoA	0.598957	0.423322	0.708212
(6Z,9Z,12Z,15Z,18Z,21Z)-Tetracosahexaenoyl-CoA	0.598957	0.423322	0.708212
(6Z,9Z,12Z,15Z,18Z)-Tetracosapentaenoyl-CoA	0.598957	0.423322	0.708212
Dihydrofolate	0.314516	0.195202	0.569665
Succinate_semialdehyde	0.682025	0.682025	#N/A
Holo-[carboxylase]	0.354541	0.473949	0.321903
Melibiose	0.417608	0.417608	#N/A
Cholesterol_ester	0.710867	0.883622	0.342147
L-2-Aminoadipate_6-semialdehyde	0.772109	0.920997	0.360747

Coproporphyrinogen_III	0.280771	0.206324	0.499194
(R)-S-Lactoylglutathione	0.0947401	0.0947401	#N/A
Acetyl_adenylate	0.228916	0.228916	#N/A
Propinol_adenylate	0.228916	0.228916	#N/A
3-Oxopropanoate	0.745846	0.745846	#N/A
Dephospho-CoA	0.37639	#N/A	0.37639
5alpha-Androstane-3,17-dione	0.621149	0.702214	0.46249
17beta-Hydroxyandrostan-3-one	0.621149	0.702214	0.46249
7alpha,12alpha-Dihydroxy-5alpha-cholestan-3-one	0.621149	0.702214	0.46249
Porphobilinogen	0.459229	0.142912	0.821896
L-4-Hydroxyglutamate_semialdehyde	0.320386	0.320386	#N/A
N-Acetyl-L-aspartate	0.976034	0.800792	0.974663
N-Formyl-L-aspartate	0.976034	0.800792	0.974663
Protoporphyrin	0.062327	0.062327	#N/A
Hemoglobin	0.767295	0.767295	#N/A
Globin	0.767295	0.767295	#N/A
CO	0.767295	0.767295	#N/A
3-Keto-4-methylzymosterol	0.226305	0.547008	0.118876
5-Methyltetrahydrofolate	0.630596	0.698143	0.481187
5-Aminolevulinate	0.891414	#N/A	0.891414
Squalene	0.58162	#N/A	0.58162
ADP-ribose	0.809306	0.945556	0.357705
L-2-Aminoadipate	0.862699	0.920997	0.553333
Heparan_sulfate	0.82681	0.82681	#N/A
2-Acyl-1-(1-alkenyl)-sn-glycero-3-phosphate	0.145576	0.370445	0.122474
GDP-D-mannose	0.0142328	0.0241887	0.130189
2-Hexaprenyl-6-methoxy-1,4-benzoquinone	0.00634487	0.00634487	#N/A
2-Hexaprenyl-3-methyl-6-methoxy-1,4-benzoquinone	0.201167	0.201167	#N/A
2-Hexaprenyl-3-methyl-5-hydroxy-6-methoxy-1,4-benzoquinone	0.590303	0.671272	0.452144
GDP-4-dehydro-6-deoxy-D-mannose	0.0276943	0.0322868	0.194272
20alpha-Hydroxycholesterol	0.995658	0.920423	0.98942
17alpha,20alpha-Dihydroxycholesterol	0.995658	0.920423	0.98942
5-Phosphoribosylamine	0.957804	0.932732	0.827765
Selenophosphate	0.584023	0.443231	0.671169
S-Glutaryldihydrolipoamide	0.46301	0.46301	#N/A
3-Carboxy-1-hydroxypropyl-ThPP	0.46301	0.46301	#N/A
[Dihydrolipoyllysine-residue_succinyltransferase]_S-succinyldihydrolipoyllysi	0.46301	0.46301	#N/A
CMP-2-aminoethylphosphonate	0.878052	0.878052	#N/A
Ceramide_2-aminoethylphosphonate	0.878052	0.878052	#N/A
Phosphatidylserine	0.878052	0.878052	#N/A
5'-Phosphoribosyl-N-formylglycinamide	0.778945	#N/A	0.778945
Prostaglandin_H2	0.645554	0.800277	0.376666
Pyridoxal_phosphate	0.623768	0.623768	#N/A
Pyridoxine	0.623768	0.623768	#N/A
Pyridoxine_phosphate	0.623768	0.623768	#N/A
Pyridoxamine	0.623768	0.623768	#N/A
Pyridoxamine_phosphate	0.623768	0.623768	#N/A

2-Methyl-1-hydroxypropyl-ThPP	0.0246484	0.0246484	#N/A
[Dihydrolipoyllysine-residue_(2-methylpropanoyl)transferase]_S-(2-methylpı	0.0246484	0.0246484	#N/A
3-Methyl-1-hydroxybutyl-ThPP	0.0246484	0.0246484	#N/A
[Dihydrolipoyllysine-residue_(2-methylpropanoyl)transferase]_S-(3-methylbı	0.0246484	0.0246484	#N/A
2-Methyl-1-hydroxybutyl-ThPP	0.0246484	0.0246484	#N/A
[Dihydrolipoyllysine-residue_(2-methylpropanoyl)transferase]_S-(2-methylbı	0.0246484	0.0246484	#N/A
tRNA(Glu)	0.807173	0.649688	0.800407
L-Glutamyl-tRNA(Glu)	0.807173	0.649688	0.800407
Protein_N6-(octanoyl)lysine	0.353188	0.072613	0.822185
Lipoyl-[acp]	0.353188	0.072613	0.822185
GDP-mannose	0.571315	#N/A	0.571315
Chitin	0.590827	0.559769	0.569344
N-Acetyl-D-glucosamine	0.590827	0.559769	0.569344
N-Acetyl-D-glucosaminide	0.590827	0.559769	0.569344
6-Pyruvoyltetrahydropterin	0.946249	0.916014	0.815574
1L-myo-Inositol_1-phosphate	0.0746913	0.0746913	#N/A
L-Threonine	0.955475	#N/A	0.955475
XTP	0.52794	0.418242	0.620083
Sucrose	0.210809	0.257705	0.313332
3,4-Dihydroxy-L-phenylalanine	0.313765	0.700313	0.112821
3alpha,7alpha-Dihydroxy-5beta-cholestanoyl-CoA	0.673198	0.673198	#N/A
3alpha,7alpha-Dihydroxy-5beta-cholest-24-enoyl-CoA	0.673198	0.673198	#N/A
3alpha,7alpha,12alpha-Trihydroxy-5beta-cholestanoyl-CoA	0.673198	0.673198	#N/A
3alpha,7alpha,12alpha-Trihydroxy-5beta-cholest-24-enoyl-CoA	0.673198	0.673198	#N/A
Naphthyl-2-methylene-succinyl-CoA	0.673198	0.673198	#N/A
Citronellyl-CoA	0.673198	0.673198	#N/A
Geranoyl-CoA	0.673198	0.673198	#N/A
(2E)-5-Methylhexa-2,4-dienoyl-CoA	0.673198	0.673198	#N/A
Orotate	0.117141	0.117141	#N/A
(S)-Dihydroorotate	0.134115	0.134115	#N/A
tRNA(Asp)	0.887459	#N/A	0.887459
L-Aspartyl-tRNA(Asp)	0.887459	#N/A	0.887459
2-Hydroxyphenylacetate	0.153765	#N/A	0.153765
5-Hydroxyisourate	0.0707942	0.0707942	#N/A
1,3,7-Trimethyluric_acid	0.0707942	0.0707942	#N/A
3,6,8-Trimethylallantoin	0.0707942	0.0707942	#N/A
Anthranilate	0.67131	#N/A	0.67131
3-Oxooctadecanoyl-CoA	0.174235	#N/A	0.174235
(S)-3-Hydroxyoctadecanoyl-CoA	0.174235	#N/A	0.174235
(S)-3-Hydroxytetracosanoyl-CoA	0.174235	#N/A	0.174235
3-Oxohexacosanoyl	0.174235	#N/A	0.174235
(S)-3-Hydroxyhexacosanoyl-CoA	0.174235	#N/A	0.174235
(S)-3-Hydroxy(11Z,14Z,17Z)-Icosatrienoyl-CoA	0.174235	#N/A	0.174235
(S)-3-Hydroxy(7Z,10Z,13Z,16Z)-Docosatetraenoyl-CoA	0.174235	#N/A	0.174235
(S)-3-Hydroxy(7Z,10Z,13Z,16Z,19Z)-Docosapentaenoyl-CoA	0.174235	#N/A	0.174235
(S)-3-Hydroxyicosanoyl-CoA	0.174235	#N/A	0.174235
(S)-3-Hydroxydocosanoyl-CoA	0.174235	#N/A	0.174235

(S)-3-Hydroxy(8Z,11Z,14Z,17Z)-Icosatetraenoyl-CoA	0.174235	#N/A	0.174235
(S)-3-Hydroxy(8Z,11Z,14Z)-Icosatrienoyl-CoA	0.174235	#N/A	0.174235
N6-(L-1,3-Dicarboxypropyl)-L-lysine	0.360747	#N/A	0.360747
Perillyl-CoA	0.198298	0.198298	#N/A
(+)-7-Isojasmonic_acid_CoA	0.246032	#N/A	0.246032
7-Methyl-3-oxo-6-octenoyl-CoA	0.246032	#N/A	0.246032
5beta-Androstane-3,17-dione	0.385284	#N/A	0.385284
5beta-Pregnane-3,20-dione	0.385284	#N/A	0.385284
5beta-Dihydrotestosterone	0.385284	#N/A	0.385284
11beta,21-Dihydroxy-3,20-oxo-5beta-pregnan-18-al	0.385284	#N/A	0.385284
Aldosterone	0.385284	#N/A	0.385284
11beta,21-Dihydroxy-5beta-pregnane-3,20-dione	0.385284	#N/A	0.385284
7alpha-Hydroxy-5beta-cholestan-3-one	0.385284	#N/A	0.385284
7alpha-Hydroxycholest-4-en-3-one	0.385284	#N/A	0.385284
7alpha,12alpha-Dihydroxy-5beta-cholestan-3-one	0.385284	#N/A	0.385284
21-Hydroxy-5beta-pregnane-3,11,20-trione	0.385284	#N/A	0.385284
Pantetheine	0.352908	0.352908	#N/A
Pantothenate	0.352908	0.352908	#N/A
O-Phospho-4-hydroxy-L-threonine	0.972212	#N/A	0.972212
2-Oxo-3-hydroxy-4-phosphobutanoate	0.972212	#N/A	0.972212
(6Z,9Z,12Z,15Z)-Octadecateraenoyl-CoA	0.789542	0.789542	#N/A
(6Z,9Z,12Z)-Octadecatrienoyl-CoA	0.789542	0.789542	#N/A
Pyridine-2,3-dicarboxylate	0.903925	0.903925	#N/A
6Z,9Z,12Z)-Octadecatrienoyl-CoA	0.871543	0.871543	#N/A
3-Oxo-(9Z,12Z,15Z,18Z)-Tetracosatetraenoyl-CoA	0.871543	0.871543	#N/A
(9Z,12Z,15Z,18Z,21Z)-Tetracosapentaenoyl-CoA	0.871543	0.871543	#N/A
3alpha,7alpha,12alpha-Trihydroxy-5beta-cholanate	0.632159	#N/A	0.632159
Chenodeoxycholate	0.632159	#N/A	0.632159
3alpha,7alpha,12alpha-Trihydroxy-5beta-cholestanoate	0.632159	#N/A	0.632159
3-Ureidopropionate	0.684348	0.684348	#N/A
3-Ureidoisobutyrate	0.684348	0.684348	#N/A
3-Aminoisobutanoate	0.684348	0.684348	#N/A
L-Gulono-1,4-lactone	0.812781	#N/A	0.812781
L-xylo-Hexulonolactone	0.812781	#N/A	0.812781
4-Fumarylacetoacetate	0.505305	0.505305	#N/A
Butanoic	0.987664	#N/A	0.987664
Dodecanoic	0.987664	#N/A	0.987664
Tetradecanoic	0.987664	#N/A	0.987664
Caffeine	0.109448	#N/A	0.109448
Cinnavalininate	0.857291	0.857291	#N/A
Glutaconyl-1-CoA	0.400379	0.400379	#N/A
S-(Hydroxymethyl)glutathione	0.539395	0.539395	#N/A
S-Formylglutathione	0.539395	0.539395	#N/A
Carnitine	0.0833957	#N/A	0.0833957
Hydrogen_sulfide	0.181461	0.181461	#N/A
L-Cystine	0.181461	0.181461	#N/A
Desmosterol	0.640243	#N/A	0.640243

24,25-Dihydrolanosterol	0.640243	#N/A	0.640243
5alpha-Cholesta-7,24-dien-3beta-ol	0.640243	#N/A	0.640243
Isofucosterol	0.640243	#N/A	0.640243
Sitosterol	0.640243	#N/A	0.640243
24-Methylenecholesterol	0.640243	#N/A	0.640243
Campesterol	0.640243	#N/A	0.640243
Zymosterol	0.640243	#N/A	0.640243
5alpha-Cholest-8-en-3beta-ol	0.640243	#N/A	0.640243
4,4-Dimethyl-5alpha-cholesta-8-en-3beta-ol	0.640243	#N/A	0.640243
7-Dehydrodesmosterol	0.640243	#N/A	0.640243
5-Formiminotetrahydrofolate	0.609985	#N/A	0.609985
5-Formyltetrahydrofolate	0.609985	#N/A	0.609985
(25R)-3-alpha,7-alpha,12-alpha-trihydroxy-5-beta-cholestan-26-oyl-CoA	0.59055	0.59055	#N/A
4-Imidazolone-5-propanoate	0.718473	0.718473	#N/A
N-Formimino-L-glutamate	0.718473	0.718473	#N/A
6-Phospho-D-gluconate	0.994588	0.994588	#N/A
D-Ribulose_5-phosphate	0.994588	0.994588	#N/A
4-Methylthio-2-oxobutanoic_acid	0.942775	0.942775	#N/A
Bilirubin_beta-diglucuronide	0.188647	0.188647	#N/A
D-Urobilinogen	0.188647	0.188647	#N/A
G13040	0.188647	0.188647	#N/A
G09660	0.188647	0.188647	#N/A
Formamidopyrimidine_nucleoside_triphosphate	0.13651	0.13651	#N/A
2,5-Diaminopyrimidine_nucleoside_triphosphate	0.13651	0.13651	#N/A
Nicotinate	0.199634	#N/A	0.199634
Linoleoyl-CoA	0.81908	0.81908	#N/A
gamma-Linolenoyl-CoA	0.81908	0.81908	#N/A
(6Z,9Z,12Z)-Octadecatrienoic_acid	0.81908	0.81908	#N/A
(9Z,12Z,15Z)-Octadecatrienoic_acid	0.81908	0.81908	#N/A
Stearidonic_acid	0.81908	0.81908	#N/A
(6Z,9Z,12Z,15Z)-Octadecatetraenoyl-CoA	0.81908	0.81908	#N/A
(R)-Lactaldehyde	0.334931	0.334931	#N/A
7alpha-Hydroxycholesterol	0.836182	#N/A	0.836182
Oxidized_dithiothreitol	0.380897	#N/A	0.380897
Vitamin_K_epoxide	0.380897	#N/A	0.380897
alpha-D-Glucose_6-phosphate	0.995795	0.995795	#N/A
Folate	0.569665	#N/A	0.569665
4alpha-Methylfecosterol	0.328008	0.328008	#N/A
G13031	0.546905	#N/A	0.546905
Acetylcholine	0.938192	#N/A	0.938192
G13034	0.922636	#N/A	0.922636
G13041	0.922636	#N/A	0.922636
(4Z,7Z,10Z,13Z,16Z,19Z)-Docosahexaenoyl-CoA	0.837341	0.837341	#N/A
(4Z,7Z,10Z,13Z,16Z)-Docosapentaenoyl-CoA	0.837341	0.837341	#N/A
But-2-enoyl-[acyl-carrier_protein]	0.530756	0.530756	#N/A
(3R)-3-Hydroxybutanoyl-[acyl-carrier_protein]	0.530756	0.530756	#N/A
(3R)-3-Hydroxydecanoyl-[acyl-carrier_protein]	0.530756	0.530756	#N/A

trans-Dec-2-enoyl-[acp]	0.530756	0.530756	#N/A
(3R)-3-Hydroxyoctanoyl-[acyl-carrier_protein]	0.530756	0.530756	#N/A
trans-Oct-2-enoyl-[acp]	0.530756	0.530756	#N/A
(3R)-3-Hydroxypalmitoyl-[acyl-carrier_protein]	0.530756	0.530756	#N/A
trans-Hexadec-2-enoyl-[acp]	0.530756	0.530756	#N/A
(3R)-3-Hydroxytetradecanoyl-[acyl-carrier_protein]	0.530756	0.530756	#N/A
trans-Tetradec-2-enoyl-[acp]	0.530756	0.530756	#N/A
trans-Dodec-2-enoyl-[acp]	0.530756	0.530756	#N/A
(R)-3-Hydroxyhexanoyl-[acp]	0.530756	0.530756	#N/A
trans-Hex-2-enoyl-[acp]	0.530756	0.530756	#N/A
(R)-3-Hydroxydodecanoyl-[acp]	0.530756	0.530756	#N/A
6-Hydroxyhexanoic_acid	0.625783	0.625783	#N/A
Adipate_semialdehyde	0.625783	0.625783	#N/A
L-Xylulose	0.906808	#N/A	0.906808
Choline_phosphate	0.105418	0.105418	#N/A
dTDP-4-dehydro-6-deoxy-alpha-D-Glucose	0.681038	0.681038	#N/A
4,6-Dideoxy-4-oxo-dTDP-D-glucose	0.681038	0.681038	#N/A
L-Pipecolate	0.577882	#N/A	0.577882
2,3,4,5-Tetrahydropyridine-2-carboxylate	0.577882	#N/A	0.577882
6-(alpha-D-glucosaminyl)-1D-myo-inositol	0.575041	#N/A	0.575041
2-Phosphoglycolate	0.154737	0.154737	#N/A
Reduced_Vitamin_K	0.00933677	0.00933677	#N/A
1-(1-Alkenyl)-sn-glycero-3-phosphate	0.940437	#N/A	0.940437
R-S-Alanine	0.398066	#N/A	0.398066
Phosphatidylglycerol	0.882871	0.882871	#N/A
Cardiolipin	0.882871	0.882871	#N/A
Raffinose	0.639177	0.639177	#N/A
1-alpha-D-Galactosyl-myo-inositol	0.639177	0.639177	#N/A
Epimelibiose	0.639177	0.639177	#N/A
D-Mannose	0.639177	0.639177	#N/A
Melibiitol	0.639177	0.639177	#N/A
Stachyose	0.639177	0.639177	#N/A
Digalactosyl-diacylglycerol	0.639177	0.639177	#N/A
1,2-Diacyl-3-beta-D-galactosyl-sn-glycerol	0.639177	0.639177	#N/A
alpha-D-Galactose	0.639177	0.639177	#N/A
CMP-N-glycoloylneuraminate	0.640291	#N/A	0.640291
N-Glycoloyl-neuraminate	0.640291	#N/A	0.640291
Guanosine_3'-diphosphate_5'-triphosphate	0.580894	0.580894	#N/A
Guanosine_3',5'-bis(diphosphate)	0.580894	0.580894	#N/A
2,5-Dihydroxypyridine	0.606566	0.606566	#N/A
4-Hydroxybenzoate	0.606566	0.606566	#N/A
4-Hydroxybenzaldehyde	0.606566	0.606566	#N/A
Glutaminyl-tRNA	0.970528	#N/A	0.970528
L-Glutamyl-tRNA(Gln)	0.970528	#N/A	0.970528
L-Asparaginyl-tRNA(Asn)	0.970528	#N/A	0.970528
L-Aspartyl-tRNA(Asn)	0.970528	#N/A	0.970528
Betaine_aldehyde	0.949766	#N/A	0.949766

4-Pyridoxate	0.635186	0.635186	#N/A
2,5-Dihydroxybenzoate	0.635186	0.635186	#N/A
Gentisate_aldehyde	0.635186	0.635186	#N/A
1-Methylnicotinamide	0.635186	0.635186	#N/A
N1-Methyl-4-pyridone-5-carboxamide	0.635186	0.635186	#N/A
N1-Methyl-2-pyridone-5-carboxamide	0.635186	0.635186	#N/A
Dihydropteroate	0.181491	0.181491	#N/A
Tetrahydrofolyl-[Glu](n)	0.181491	0.181491	#N/A
1-Methylxanthine	0.503458	#N/A	0.503458
1-Methyluric_acid	0.503458	#N/A	0.503458
1,7-Dimethyluric_acid	0.503458	#N/A	0.503458
Theobromine	0.503458	#N/A	0.503458
3,7-Dimethyluric_acid	0.503458	#N/A	0.503458
7-Methylxanthine	0.503458	#N/A	0.503458
7-Methyluric_acid	0.503458	#N/A	0.503458
3alpha,7alpha-Dihydroxy-5beta-cholestane	0.671931	0.671931	#N/A
3alpha,7alpha,12alpha-Trihydroxy-5beta-cholestane	0.671931	0.671931	#N/A
3alpha,7alpha,12alpha,26-Tetrahydroxy-5beta-cholestane	0.671931	0.671931	#N/A
(R)-Lactate	0.535934	0.535934	#N/A
alpha,alpha-Trehalose	0.84513	0.84513	#N/A
Itaconate	0.500087	0.500087	#N/A
L-Glutamyl_5-phosphate	0.193885	#N/A	0.193885
G00143	0.230795	#N/A	0.230795
2-Deoxy-D-ribose_5-phosphate	0.505875	#N/A	0.505875
Deoxyribose	0.505875	#N/A	0.505875
Insulin	0.20495	#N/A	0.20495
Insulin_dithiol	0.20495	#N/A	0.20495
Protein_dithiol	0.20495	#N/A	0.20495
Protein_disulfide	0.20495	#N/A	0.20495
Retinol	0.780102	#N/A	0.780102
11-cis-Retinol	0.780102	#N/A	0.780102
11-cis-Retinal	0.780102	#N/A	0.780102
Apocytochrome_c	0.258786	0.258786	#N/A
N-Acetylneuraminate_9-phosphate	0.275938	0.275938	#N/A
Undecaprenyl-diphospho-N-acetylmuramoyl-(N-acetylglucosamine)-L-alanyl-	0.533741	0.533741	#N/A
Undecaprenyl-diphospho-N-acetylmuramoyl-(N-acetylglucosamine)-L-alanyl-	0.533741	0.533741	#N/A
Undecaprenyl-diphospho-N-acetylmuramoyl-(N-acetylglucosamine)-L-alanyl-	0.533741	0.533741	#N/A
D-4'-Phosphopantothenate	0.552079	#N/A	0.552079
Sulfite	0.445093	0.445093	#N/A
Acylglycerone_phosphate	0.758071	#N/A	0.758071
O-Alkylglycerone_phosphate	0.758071	#N/A	0.758071
gamma-L-Glutamyl-L-cysteine	0.442796	#N/A	0.442796
beta-Carotene	0.0831623	#N/A	0.0831623
(S)-2,3-Epoxysqualene	0.381444	#N/A	0.381444
Biotin	0.794072	#N/A	0.794072
D-Aspartate	0.677967	0.677967	#N/A
5-Hydroxy-N-formylkynurenine	0.7692	#N/A	0.7692

5-Hydroxykynurenine	0.7692	#N/A	0.7692
H2S2O3	0.069765	0.069765	#N/A
Phosphatidylinositol-3,4,5-trisphosphate	0.694129	#N/A	0.694129
N-Acetylornithine	0.706269	0.706269	#N/A
Isopentenyl_diphosphate	0.100929	0.100929	#N/A
Obtusifoliol	0.565442	#N/A	0.565442
Biotinyl-5'-AMP	0.875883	0.875883	#N/A
Adenylyl_sulfate	0.604856	0.604856	#N/A
UDP-N-acetyl-D-mannosamine	0.356828	0.356828	#N/A
alpha-D-Glucose_1-phosphate	0.044716	0.044716	#N/A
Sulfatide	0.398102	0.398102	#N/A
P1,P3-Bis(5'-adenosyl)_triphosphate	0.262083	#N/A	0.262083
Orotidine_5'-phosphate	0.24962	0.24962	#N/A
2-Hydroxyglutarate	0.321045	#N/A	0.321045
trans-Tetradeca-2-enoyl-CoA	0.589185	#N/A	0.589185
trans-Hexadeca-2-enoyl-CoA	0.589185	#N/A	0.589185
trans-Octadeca-2-enoyl	0.589185	#N/A	0.589185
trans-Icosa-2-enoyl-CoA	0.589185	#N/A	0.589185
Icosnoyl-CoA	0.589185	#N/A	0.589185
trans-Docosa-2-enoyl-CoA	0.589185	#N/A	0.589185
Docosanoyl-CoA	0.589185	#N/A	0.589185
trans-Tetracosa-2-enoyl-CoA	0.589185	#N/A	0.589185
trans-Hexacosa-2-enoyl-CoA	0.589185	#N/A	0.589185
Hexacosanoyl-CoA	0.589185	#N/A	0.589185
trans-(8Z,11Z,14Z,17Z)-Icosatetraene-2-enoyl-CoA	0.589185	#N/A	0.589185
(8Z,11Z,14Z,17Z)-Icosatetraenoyl-CoA	0.589185	#N/A	0.589185
trans-(8Z,11Z,14Z)-Icosatriene-2-enoyl-CoA	0.589185	#N/A	0.589185
(8Z,11Z,14Z)-Icosatrienoyl-CoA	0.589185	#N/A	0.589185
trans-(11Z,14Z,17Z)-Icosatriene-2-enoyl-CoA	0.589185	#N/A	0.589185
(11Z,14Z,17Z)-Icosatrienoyl-CoA	0.589185	#N/A	0.589185
trans-(7Z,10Z,13Z,16Z)-Docosatetraene-2-enoyl-CoA	0.589185	#N/A	0.589185
trans-(7Z,10Z,13Z,16Z,19Z)-Docosapentaene-2-enoyl-CoA	0.589185	#N/A	0.589185
G13036	0.24867	0.24867	#N/A
beta-D-Glucose	0.31371	0.31371	#N/A
D-Glucono-1,5-lactone	0.31371	0.31371	#N/A
Cysteamine	0.875023	#N/A	0.875023
trans,trans,cis-Geranylgeranyl_diphosphate	0.622487	0.622487	#N/A
Dehydrodolichol_diphosphate	0.622487	0.622487	#N/A
Maltose	0.257705	0.257705	#N/A
3-Methylglutaconyl-CoA	0.285299	0.285299	#N/A
all-trans-Hexaprenyl_diphosphate	0.141915	0.141915	#N/A
3-Hexaprenyl-4-hydroxybenzoate	0.141915	0.141915	#N/A
5-Phospho-alpha-D-ribose_1-diphosphate	0.932732	0.932732	#N/A
2-Acetolactate	0.87989	#N/A	0.87989
(S)-2-Acetolactate	0.87989	#N/A	0.87989
(S)-2-Aceto-2-hydroxybutanoate	0.87989	#N/A	0.87989
N-Acetyl-D-glucosamine_6-phosphate	0.301537	0.301537	#N/A

Poly-L-glutamate	0.432583	#N/A	0.432583
Heme	0.516266	0.516266	#N/A
(R)-Mevalonate	0.462295	#N/A	0.462295
Coproporphyrinogen_I	0.206324	0.206324	#N/A
Thiamin_triphosphate	0.239615	0.239615	#N/A
Thiamin_diphosphate	0.239615	0.239615	#N/A
HCO3-	0.287976	0.287976	#N/A
Hydroxymethylbilane	0.821896	#N/A	0.821896
3-Hydroxypropionyl-CoA	0.936572	#N/A	0.936572
3-Hydroxypropanoate	0.936572	#N/A	0.936572
(S)-3-Hydroxyisobutyryl-CoA	0.936572	#N/A	0.936572
1-(1-Alkenyl)-sn-glycerol	0.926976	#N/A	0.926976
GlA_protein	0.746483	#N/A	0.746483
GlA_protein_precursor	0.746483	#N/A	0.746483
Reduced_ferredoxin	0.979047	#N/A	0.979047
Oxidized_ferredoxin	0.979047	#N/A	0.979047
Reduced_adrenal_ferredoxin	0.979047	#N/A	0.979047
Oxidized_adrenal_ferredoxin	0.979047	#N/A	0.979047
18-Hydroxycorticosterone	0.979047	#N/A	0.979047
Cob(I)yrinate_a,c_diamide	0.0477778	0.0477778	#N/A
Adenosyl_cobyrinate_a,c_diamide	0.0477778	0.0477778	#N/A
Triphosphate	0.0477778	0.0477778	#N/A
5'-Phosphoribosylglycinamide	0.827765	#N/A	0.827765
Presqualene_diphosphate	0.723523	#N/A	0.723523
3,5-Diiodo-L-tyrosine	0.207239	#N/A	0.207239
O-(4-Hydroxy-3,5-diidophenyl)-3,5-diiodo-L-tyrosine	0.207239	#N/A	0.207239
HI	0.207239	#N/A	0.207239
Iodine	0.207239	#N/A	0.207239
3-Iodo-L-tyrosine	0.207239	#N/A	0.207239
Triiodothyronine	0.207239	#N/A	0.207239
Lipoylprotein	0.848597	0.848597	#N/A
D-Glucose	0.885464	0.885464	#N/A
Uroporphyrinogen_III	0.083056	0.083056	#N/A
D-Ornithine	0.177855	0.177855	#N/A
5-Amino-2-oxopentanoic_acid	0.177855	0.177855	#N/A
D-Proline	0.177855	0.177855	#N/A
1-Pyrroline-2-carboxylate	0.177855	0.177855	#N/A
D-Arginine	0.177855	0.177855	#N/A
5-Guanidino-2-oxopentanoate	0.177855	0.177855	#N/A
cis-4-Hydroxy-D-proline	0.177855	0.177855	#N/A
1-Pyrroline-4-hydroxy-2-carboxylate	0.177855	0.177855	#N/A
2-Hexaprenyl-6-methoxyphenol	0.0288475	0.0288475	#N/A
P1,P4-Bis(5'-adenosyl)_tetraphosphate	0.418242	0.418242	#N/A
P1,P4-Bis(5'-uridyl)_tetraphosphate	0.418242	0.418242	#N/A
P1,P4-Bis(5'-guanosyl)_tetraphosphate	0.418242	0.418242	#N/A
P1,P4-Bis(5'-xanthosyl)_tetraphosphate	0.418242	0.418242	#N/A
19-Hydroxyandrost-4-ene-3,17-dione	0.0667953	#N/A	0.0667953

19-Hydroxytestosterone	0.0667953	#N/A	0.0667953
(3R)-3-Isopropenyl-6-oxoheptanoate	0.0544972	0.0544972	#N/A
(3R)-3-Isopropenyl-6-oxoheptanoyl-CoA	0.0544972	0.0544972	#N/A
cis-2-Methyl-5-isopropylhexa-2,5-dienoic_acid	0.0544972	0.0544972	#N/A
trans-2-Methyl-5-isopropylhexa-2,5-dienoic_acid	0.0544972	0.0544972	#N/A
CDP-diacylglycerol	0.383143	0.383143	#N/A
2-(Formamido)-N1-(5'-phosphoribosyl)acetamidine	0.556567	#N/A	0.556567
D-Glucosamine	0.417418	0.417418	#N/A
D-Glucosaminide	0.417418	0.417418	#N/A
Pentosans	0.417418	0.417418	#N/A
Calcidiol	0.749448	#N/A	0.749448
Calcitriol	0.749448	#N/A	0.749448
5-(2'-Carboxyethyl)-4,6-dihydroxypicolinate	0.406293	0.406293	#N/A
3,4-Dihydroxybenzoate	0.406293	0.406293	#N/A
Benzene-1,2,4-triol	0.406293	0.406293	#N/A
1,1-Dichloro-2-(dihydroxy-4'-chlorophenyl)-2-(4'-chlorophenyl)ethylene	0.406293	0.406293	#N/A
2,3-Dihydroxy_1,1,1-Trichloro-2,2-bis(4'-chlorophenyl)ethane	0.406293	0.406293	#N/A
4-Sulfocatechol	0.406293	0.406293	#N/A
3,4-Dihydroxyfluorene	0.406293	0.406293	#N/A
2,2',3-Trihydroxybiphenyl	0.406293	0.406293	#N/A
1,2-Dihydroxyfluorene	0.406293	0.406293	#N/A
2,2',3-Trihydroxydiphenylether	0.406293	0.406293	#N/A
Carbazole	0.406293	0.406293	#N/A
2'-Aminobiphenyl-2,3-diol	0.406293	0.406293	#N/A
Styrene	0.406293	0.406293	#N/A
Styrene_cis-glycol	0.406293	0.406293	#N/A
Phenanthrene-3,4-diol	0.406293	0.406293	#N/A
Phenanthracene	0.406293	0.406293	#N/A
(+)-cis-3,4-Dihydrophenanthrene-3,4-diol	0.406293	0.406293	#N/A
CDP-ethanolamine	0.529107	0.529107	#N/A
4alpha-Methylzymosterol	0.547008	0.547008	#N/A
Carnosine	0.762555	#N/A	0.762555
beta-Alanyl-N(pi)-methyl-L-histidine	0.762555	#N/A	0.762555
Betaine	0.53131	#N/A	0.53131
C15811	0.272926	0.272926	#N/A
C15812	0.272926	0.272926	#N/A
(6Z,9Z,12Z,15Z,18Z,21Z)-3-Hydroxytetracosahexa-6,9,12,15,18,21-enoyl-CoA	0.547405	0.547405	#N/A
(6Z,9Z,12Z,15Z,18Z,21Z)-3-Oxotetracosahexa-6,9,12,15,18,21-enoyl-CoA	0.547405	0.547405	#N/A
(6Z,9Z,12Z,15Z,18Z)-3-Hydroxytetracosapenta-6,9,12,15,18-enoyl-CoA	0.547405	0.547405	#N/A
(6Z,9Z,12Z,15Z,18Z)-3-Oxotetracosapenta-6,9,12,15,18-enoyl-CoA	0.547405	0.547405	#N/A
G00005	0.3462	0.3462	#N/A
G10526	0.3462	0.3462	#N/A
G00006	0.3462	0.3462	#N/A
Sulfur	0.822185	#N/A	0.822185
Ascorbate	0.700101	#N/A	0.700101
Dehydroascorbate	0.700101	#N/A	0.700101
Allantoate	0.75119	#N/A	0.75119

(-)-Ureidoglycolate	0.75119	#N/A	0.75119
5-Oxoproline	0.0482985	0.0482985	#N/A
2-Amino-3-oxoadipate	0.998006	#N/A	0.998006
Lipoyl-AMP	0.123411	#N/A	0.123411
3-D-Glucosyl-1,2-diacylglycerol	0.956773	#N/A	0.956773
Diglucosyl-diacylglycerol	0.956773	#N/A	0.956773
Alkyl_sulfate	0.307449	#N/A	0.307449
2-Acyl-sn-glycero-3-phosphocholine	0.156314	0.156314	#N/A
2-Acyl-sn-glycero-3-phosphoethanolamine	0.156314	0.156314	#N/A
Dopaquinone	0.112821	#N/A	0.112821
4-Coumarate	0.112821	#N/A	0.112821
3,4-Dihydroxy-trans-cinnamate	0.112821	#N/A	0.112821
5,6-Dihydroxyindole	0.112821	#N/A	0.112821
(S)-N-Methylcoclaurine	0.112821	#N/A	0.112821
3'-Hydroxy-N-methyl-(S)-coclaurine	0.112821	#N/A	0.112821
Indole-5,6-quinone	0.112821	#N/A	0.112821
22beta-Hydroxycholesterol	0.98942	#N/A	0.98942
20alpha,22beta-Dihydroxycholesterol	0.98942	#N/A	0.98942
Ferredoxin	0.98942	#N/A	0.98942
4-Methylpentanal	0.98942	#N/A	0.98942
L-2-Aminoadipate_adenylate	0.920997	0.920997	#N/A
alpha-Aminoadipoyl-S-acyl_enzyme	0.920997	0.920997	#N/A
Holo-Lys2	0.920997	0.920997	#N/A
G00145	0.637177	#N/A	0.637177
20alpha-Hydroxy-4-pregnen-3-one	0.727453	#N/A	0.727453
17alpha,20alpha-Dihydroxypregn-4-en-3-one	0.727453	#N/A	0.727453
1-(5'-Phosphoribosyl)-5-amino-4-imidazolecarboxamide	0.0559758	0.0559758	#N/A
5-Amino-4-imidazolecarboxyamide	0.0559758	0.0559758	#N/A
L-2-Amino-3-oxobutanoic_acid	0.903549	#N/A	0.903549
L-Methionyl-tRNA	0.223685	#N/A	0.223685
dITP	0.620083	#N/A	0.620083
2'-Deoxyinosine_5'-phosphate	0.620083	#N/A	0.620083
RNA	0.0237395	#N/A	0.0237395
6-Lactoyl-5,6,7,8-tetrahydropterin	0.916014	0.916014	#N/A
Lipid_hydroperoxide	0.524622	0.524622	#N/A
Lipid	0.524622	0.524622	#N/A
Aminoimidazole_ribotide	0.999179	0.999179	#N/A
1-(5-Phospho-D-ribosyl)-5-amino-4-imidazolecarboxylate	0.999179	0.999179	#N/A

Muscle

Reactome

	Reporter P-value		
#Feature	**No-direction**	**Down regulated**	**Up regulated**
Olfactory_Receptor_-_G_Protein_olfactory_trimer_complex_formation	0.374742	0.984548	0.0381125
Cleavage_at_the_3-Splice_Site_and_Exon_Ligation	0.778833	0.199111	0.976147

Formation_of_Exon_Junction_Complex	0.778833	0.199111	0.976147
Formation_of_an_intermediate_Spliceosomal_C_complex	0.732066	0.22776	0.955284
Lariat_Formation_and_5-Splice_Site_Cleavage	0.758101	0.236984	0.960913
Formation_of_the_active_Spliceosomal_C_complex	0.741658	0.236984	0.955284
Recruitment_of_additional_gamma_tubulin/_gamma_TuRC_to_the_centros	0.758129	0.395035	0.911333
Formation_of_the_Spliceosomal_B_Complex	0.840688	0.182359	0.991446
Dissociation_of_Phospho-Nlp_from_the_centrosome	0.678105	0.329347	0.878097
Loss_of_C-Nap-1_from_centrosomes	0.678105	0.329347	0.878097
Plk1-mediated_phosphorylation_of_Nlp	0.678105	0.329347	0.878097
Recruitment_of_CDK11p58_to_the_centrosomes	0.678105	0.329347	0.878097
Recruitment_of_Plk1_to_centrosomes	0.678105	0.329347	0.878097
GPCRs_that_act_as_GEFs_for_G_alpha-i	0.637234	0.665686	0.527877
Kinetochore_capture_of_astral_microtubules	0.91404	0.980231	0.492769
Translocation_of_ribosome_by_3_bases_in_the_3_direction	1.41459E-07	6.34771E-08	0.154866
Aminoacyl-tRNA__binds_to_the_ribosome_at_the_A-site	2.46078E-07	1.16377E-07	0.154866
Hydrolysis_of_eEF1A:GTP	2.46078E-07	1.16377E-07	0.154866
Release_of_40S_and_60S_subunits_from_the_80S_ribosome_	6.72105E-07	4.51058E-07	0.128704
GTP_bound_eRF3:eRF1_complex_binds_the_peptidyl_tRNA:mRNA:80S_Ribo	3.13874E-06	3.03593E-06	0.118204
GTP_Hydrolysis_by_eRF3_bound_to_the_eRF1:mRNA:polypeptide:80S_Ribo	3.13874E-06	3.03593E-06	0.118204
Polypeptide_release_from_the_eRF3-GDP:eRF1:mRNA:80S_Ribosome_comp	3.13874E-06	3.03593E-06	0.118204
eIF5B:GTP_is_hydrolyzed_and_released	2.04158E-06	1.01096E-06	0.165011
The_60S_subunit_joins_the_translation_initiation_complex_	2.04158E-06	1.01096E-06	0.165011
Peptide_transfer_from_P-site_tRNA_to_the_A-site_tRNA	1.69079E-06	1.01096E-06	0.154866
GAPs_inactivate_Rho_GTPase:GTP_by_hydrolysis	0.7742	0.830949	0.569814
Association_of_phospho-L13a_with_GAIT_element_of_Ceruloplasmin_mRN	0.000219468	4.08605E-05	0.520886
Formation_of_translation_initiation_complexes_yielding_circularized_Cerulo	0.000215538	3.71464E-05	0.520886
eIF2:GTP_is_hydrolyzed,_eIFs_are_released_	0.000127589	3.71464E-05	0.421662
Start_codon_recognition	0.000127589	3.71464E-05	0.421662
Formation_of_translation_initiation_complexes_containing_mRNA_that_do	0.000127537	3.71464E-05	0.443027
Ribosomal_scanning_	0.000127537	3.71464E-05	0.443027
GEFs_activate_Rho_GTPase:GDP	0.772177	0.606036	0.785263
Formation_of_the_43S_pre-initiation_complex	0.000174293	0.000033032	0.352303
ATP_Hydrolysis_By_Myosin	0.00104241	0.00025087	0.384136
Calcium_Binds_Troponin-C	0.00104241	0.00025087	0.384136
Myosin_Binds_ATP	0.00104241	0.00025087	0.384136
Release_Of_ADP_From_Myosin	0.00104241	0.00025087	0.384136
Dissociation_of_L13a_from_the_60s_ribosomal_subunit	0.000740353	0.000102156	0.559496
GPCRs_that_act_as_GEFs_for_G_alpha-q/11	0.289374	0.618175	0.164347
GPCRs_that_act_as_GEFs_for_G_alpha-s	0.536845	0.468133	0.59012
eIF3_and_eIF1A_bind_to_the_40S_subunit	4.60129E-05	0.000102283	0.0767113
Addition_of_nucleotides_leads_to_transcript_elongation	0.736782	0.383822	0.897945
Recruitment_of_elongation_factors_to_form_elongation_complex	0.736782	0.383822	0.897945
ATAC_spliceosome_mediated_3_splice_site_cleavage,_exon_ligation	0.297207	0.0342264	0.911977
ATAC_spliceosome_mediated_Lariat_formation,5_splice_site_cleavage_	0.297207	0.0342264	0.911977
Formation_of_AT-AC_B_Complex	0.297207	0.0342264	0.911977
Formation_of_AT-AC_C_complex	0.297207	0.0342264	0.911977
Formation_of_pre-mRNPs	0.586556	0.145067	0.904015

Abortive_initiation_after_formation_of_the_first_phosphodiester_bond	0.0319139	0.0133634	0.325972
Abortive_Initiation_Before_Second_Transition	0.0319139	0.0133634	0.325972
Fall_Back_to_Closed_Pre-initiation_Complex	0.0319139	0.0133634	0.325972
Formation_of_the_closed_pre-initiation_complex	0.0319139	0.0133634	0.325972
Newly_Formed_Phosphodiester_Bond_Stabilized_and_PPi_Released	0.0319139	0.0133634	0.325972
NTP_Binds_Active_Site_of_RNA_Polymerase_II	0.0319139	0.0133634	0.325972
Nucleophillic_Attack_by_3-hydroxyl_Oxygen_of_nascent_transcript_on_the	0.0319139	0.0133634	0.325972
RNA_Polymerase_II_Promoter_Opening:_First_Transition	0.0319139	0.0133634	0.325972
Cleavage_of_mRNA_at_the_3-end	0.77575	0.433365	0.878958
Addition_of_Nucleotides_5_through_9_on_the_growing_Transcript	0.0183798	0.0133634	0.224854
NADH_enters_the_respiratory_chain_at_Complex_I	0.0139251	0.00273097	0.830231
Formation_of_AT-AC_A_complex	0.197642	0.00663351	0.942953
Actin/tubulin:prefoldin_complex_associates_with_CCT/TriC	0.227399	0.0777407	0.854535
2-4_nt.backtracking_of_Pol_II_complex_on_the_template_leading_to_elong	0.821963	0.547372	0.900368
7-14_nt._Backtracking_of_Pol_II_complex_on_the_template_leading_to_elo	0.821963	0.547372	0.900368
Abortive_termination_of_elongation_after_arrest	0.821963	0.547372	0.900368
Elongating_transcript_encounters_a_lesion_in_the_template	0.821963	0.547372	0.900368
Pol_II_elongation_complex_moves_on_the_template_as_transcript_elongat	0.821963	0.547372	0.900368
Resumption_of_elongation_after_recovery_from_pausing	0.821963	0.547372	0.900368
Separation_of_elongating_transcript_from_template	0.821963	0.547372	0.900368
Formation_of_NR-NCOR_Complex	0.0231176	0.0400677	0.140297
Binding_of_TFIIE_to_the_growing_preinitiation_complex	0.0280304	0.0224107	0.236885
mRNA_polyadenylation	0.84205	0.246249	0.964779
Crk_binding_to_p130cas	0.975746	0.787904	0.979054
Ubiquitination_of_Cyclin_B_by_phospho-APC/C:Cdc20_complex	0.559792	0.494361	0.589634
Assembly_of_repair_proteins_at_the_site_of_Pol_II_blockage	0.470577	0.128919	0.811247
Displacement_of_stalled_Pol_II__from_the_lesion_site_	0.470577	0.128919	0.811247
Phosphorylation_of_p130Cas_by_Src-FAK_complex	0.978341	0.800465	0.979054
Recruitment_of_p130Cas_to_FAK	0.978341	0.800465	0.979054
Interaction_of_SOS_with_Grb2_bound_to_FADK1	0.948596	0.694717	0.965671
Ubiquitination_of_cell_cycle_proteins_targeted_by_the_APC/C:Cdh1comple	0.458957	0.260562	0.713137
Translocation_of_Preproinsulin_to_Endoplasmic_Reticulum	0.161972	0.107722	0.479796
Recruitment_of_RNA_Polymerase_II_Holoenzyme_by_TFIIF_to_the_pol_II_p	0.0293463	0.0158359	0.290085
Recruitment_of_Grb2_to_pFADK1	0.955787	0.694717	0.974749
Ubiquitination_of_Cyclin_A_by_APC/C:Cdc20_complex	0.493975	0.544388	0.439003
Phosphorylation_of_PLC-gamma1	0.9732	0.910583	0.918505
Association_of_Cyclin_B:Cdc2_with_Cdc20:APC/C_complex	0.645856	0.556181	0.650741
Auto-phosphorylation_of_FADK1_at_Y397	0.9743	0.794012	0.974749
Phosphorylation_of_pFADK1_by_SRC	0.9743	0.794012	0.974749
Translocation_of_FADK1_to_Focal_complexes	0.9743	0.794012	0.974749
Orc1_is_phosphorylated_by_cyclin_A/CDK2	0.0465349	0.758627	0.00306816
DNA_Replication_Factor_A_(RPA)_associates_with_the_pre-replicative_com	0.196774	0.659929	0.0661502
Association_of_cell_cycle_proteins_with_the_APC/C:Cdh1_complex	0.54588	0.303701	0.774258
Free_APC/C_phosphorylated_by_Cyclin_B:Cdc2	0.620567	0.51575	0.650741
The_high_affinity_receptor_complex_binds_to_G-protein	0.093363	0.0840209	0.345271
The_receptor:G-protein_complex__binds_GTP	0.093363	0.0840209	0.345271
The_receptor:G-protein_complex_dissociates	0.093363	0.0840209	0.345271

The_receptor:G-protein_complex_releases_GDP	0.093363	0.0840209	0.345271
Hyperphosphorylation_(Ser2)_of_RNA_Pol_II_CTD_by_P-TEFb_complex	0.509439	0.209136	0.809592
Hypophosphorylation_of_RNA_Pol_II_CTD_by_FCP1P_protein	0.158619	0.0242167	0.660565
Capping_complex_formation	0.032687	0.0310319	0.209922
Dissociation_of__transcript_with_5-GMP_from_GT	0.032687	0.0310319	0.209922
Formation_of_the_CE:GMP_intermediate_complex	0.032687	0.0310319	0.209922
Hydrolysis_of_the_5-end_of_the_nascent_transcript_by_the_capping_enzyı	0.032687	0.0310319	0.209922
Methylation_of_GMP-cap_by_RNA_Methyltransferase	0.032687	0.0310319	0.209922
SPT5_subunit_of_Pol_II_binds_the_RNA_triphosphatase_(RTP)	0.032687	0.0310319	0.209922
Transfer_of_GMP_from_the_capping_enzyme_GT_site_to_5-end_of_mRNA	0.032687	0.0310319	0.209922
alphaIIbbeta3:c-Src_clustering	0.983766	0.794012	0.988226
Autophosphorylation_of_c-src	0.983766	0.794012	0.988226
Interaction_of_alphaIIbbeta3_with_Fibrinogen	0.983766	0.794012	0.988226
Release_of_Csk_from_Src	0.983766	0.794012	0.988226
Dephosphorylation_of_inactive_c-src_by_PTPB1	0.9677	0.776712	0.974993
Assembly_in_clathrin-coated_vesicles_(CCVs)	0.15972	0.1641	0.333833
Association_of_Cyclin_A_with_the_APC/C	0.58364	0.604765	0.504984
Activation_of_claspin	0.223823	0.793212	0.0554896
Recruitment_of_TAP_to_the_EJC	0.964736	0.31429	0.994709
Abortive_Initiation_After_Second_Transition	0.0601376	0.0183308	0.425717
Dissociation_of_Rho_GTP:GDP_from_GDI_complex	0.586913	0.634609	0.468765
GDIs_block_activation_of_Rho_GTPase:GDP	0.586913	0.634609	0.468765
unfolded_actin/tubulin_associates_with_prefoldin	0.389407	0.151125	0.911991
Vamp2_associated_trans-Golgi_Network_Derived_Vesicle_Uncoating	0.383791	0.0375741	0.878642
Loading_of_claspin_onto_DNA_during_replication_origin_firing	0.234667	0.83029	0.0554896
Multiubiquitination_of_Nek2A	0.366477	0.332398	0.490732
Activation_of_GT	0.0290852	0.0116883	0.29828
RNA_Polymerase_II_CTD_(phosphorylated)_binds_to_CE	0.0290852	0.0116883	0.29828
Vamp7_associated_trans-Golgi_Network_Derived_Vesicle_Uncoating	0.678815	0.0485949	0.976387
Vamp8_associated_trans-Golgi_Network_Derived_Vesicle_Uncoating	0.386551	0.0225975	0.924843
Cdc45_associates_with_the_pre-replicative_complex_at_the_origin	0.26617	0.83029	0.0661502
G-protein_beta-gamma_subunits_rebind_the_alpha-GDP_subunit	0.186119	0.0951893	0.599854
Abortive_termination_of_early_transcription_elongation_by_DSIF:NELF	0.426611	0.217811	0.718883
Formation_of_DSIF:NELF:early_elongation_complex	0.426611	0.217811	0.718883
Addition_of_nucleotides_10_and_11_on_the_growing_transcript:_Third_Tra	0.0638156	0.0116883	0.50337
Addition_of_nucleotides_between_position_+11_and_+30	0.0638156	0.0116883	0.50337
Extrusion_of_5-end_of_30_nt_long_transcript_through_the_pore_in_Pol_II_	0.0638156	0.0116883	0.50337
Phosphorylation_(Ser5)_of_RNA_pol_II_CTD	0.0638156	0.0116883	0.50337
PDGF_binds_to_extracellular_matrix_proteins	0.973362	0.751324	0.981541
Sprouty_sequesters_Cbl_away_from_active_EGFR	0.276523	0.43149	0.248853
CDK_and_DDK_associate_with_the_Mcm10:pre-replicative_complex	0.230265	0.83029	0.0457648
Association_of_Nek2A_with_MCC:APC/C	0.45443	0.386428	0.562906
Association_of_Cyclin_A:Cdk2_with_Cdh1	0.632351	0.342067	0.856651
Phosphorylation_of_Cdh1_by_Cyclin_A:Cdk2	0.632351	0.342067	0.856651
Cbl-mediated_ubiquitination_of_CIN85	0.185644	0.328823	0.20839
Phosphorylation_of_SLP-76	0.980856	0.841142	0.977694
G-protein_Gq_activation_by_PAR	0.260847	0.281333	0.382227

G12/13_activation_by_PAR	0.395139	0.238232	0.661249
Cleavage_of_Signal_Peptide_(Preproinsulin)	0.527782	0.243728	0.817996
3-_incision_of_DNA_by_XPG_in_GG-NER	0.784632	0.426379	0.934684
5-incision_of_DNA_by_ERCC1-XPF_in_GG-NER	0.784632	0.426379	0.934684
Binding_of_ERCC1-XPF_to_preincision_complex_	0.784632	0.426379	0.934684
Vamp2_associated_trans-Golgi_Network_Vesicle_Scission	0.298421	0.248038	0.449626
Activation_of_APC/C:Cdc20_by_dissociation_of_Cdc20:phospho-APC/C_fror	0.399491	0.313288	0.562906
Binding_of_the_MCC_complex_to_the_APC/C_complex	0.399491	0.313288	0.562906
Ubiquitination_of_Securin_by_phospho-APC/C:Cdc20_complex	0.403717	0.124608	0.847426
Dissociation_of_PTRF:Polymerase_I/Nascent_Pre_rRNA_Complex:TTF-I:Sal_I	0.279391	0.0942332	0.719943
PTRF_Binds_the_Polymerase_I_Transcription_Complex/Nascent_Pre_rRNA_	0.279391	0.0942332	0.719943
Interaction_of_integrin_alpha_3_beta_1_with_Laminin-5	0.795533	0.820647	0.591481
Interaction_of_integrin_alpha_6_beta_1_with_Laminin-1	0.795533	0.820647	0.591481
Interaction_of_integrin_alpha_7_beta_1_with_Laminin-1	0.795533	0.820647	0.591481
Interaction_of_integrin_alpha_7_beta_1_with_Laminin-2	0.795533	0.820647	0.591481
alphaIIbbeta3_activation	0.944343	0.552285	0.980704
EGFR_non-clathrin_mediated_endocytosis	0.246718	0.389848	0.248853
Vamp7_associated_trans-Golgi_Network_Vesicle_Scission	0.611054	0.315043	0.765062
Vamp8_associated_trans-Golgi_Network_Vesicle_Scission	0.298833	0.176121	0.539929
Free_APC/C_phosphorylated_by_Plk1	0.729146	0.361409	0.896101
Phosphorylation_of_TBSMs_in_LAT	0.987078	0.877819	0.977694
Multiubiquitination_of_APC/C-associated_Cdh1	0.34525	0.0810665	0.847426
Formation_of_open_bubble_structure_in_DNA_by_helicases	0.518702	0.320542	0.753976
Recruitment_of_repair_factors_to_form_preincision_complex	0.518702	0.320542	0.753976
Recruitment_of_RNA_Polymerase_III_to_TFIIIB:SNAPc:Type_3_Promoter_Cc	0.0732399	0.140857	0.161311
RNA_Polymerase_III_Abortive_Initiation_At_Type_3_Open_Promoters	0.0732399	0.140857	0.161311
RNA_Polymerase_III_Promoter_Opening_at_Type_3_Promoters	0.0732399	0.140857	0.161311
RNA_Polymerase_III_Simple_Start_Sequence_Initiation_At_Type_3_Promote	0.0732399	0.140857	0.161311
Loss_of_Rrn3_from_RNA_Polymerase_I_promoter_escape_complex	0.435667	0.218856	0.719943
Recruitment_of_Active_RNA_Polymerase_I_to_SL1:phos.UBF-1:rDNA_Prom	0.435667	0.218856	0.719943
Polymerase_I_Transcription_Complex/Nascent_Pre_rRNA_Complex_pauses_	0.385222	0.169815	0.719943
Interaction_of_alpha_2_beta_1_with_Lamini-5	0.869888	0.876051	0.697473
Interaction_of_integrin_alpha_1_beta_1_with_Laminin-1	0.869888	0.876051	0.697473
Vamp2_associated_trans-Golgi_Network_AP-1_Binding_And_Cargo_Capture	0.259779	0.433604	0.218152
Association_of_Securin_with_Cdc20:APC/C_complex	0.502021	0.1506	0.903774
DSIF_complex_binds_to_RNA_Pol_II_(hypophosphorylated)	0.332112	0.0970599	0.718883
Recruitment_of__RNA_polymerase_III_to_TFIIIB:TFIIIC:TFIIIA:Type_1_Promc	0.0177146	0.0812859	0.057496
RNA_Polymerase_III_Abortive_Initiation_At_Type_1_Open_Promoters	0.0177146	0.0812859	0.057496
RNA_Polymerase_III_Promoter_Opening_at_Type_1_Promoters	0.0177146	0.0812859	0.057496
RNA_Polymerase_III_Simple_Start_Sequence_Initiation_At_Type_1_Promote	0.0177146	0.0812859	0.057496
Elongation_of_pre-rRNA_transcript	0.302127	0.0942143	0.719943
Akt_binds_eNOS_complex_via_HSP90	0.688598	0.20585	0.9264
Akt_phosphorylates_eNOS	0.688598	0.20585	0.9264
NO_biosynthesis	0.688598	0.20585	0.9264
Change_of_PKC_theta_conformation	0.99588	0.936001	0.985861
Vamp7_associated_trans-Golgi_Network_AP-1_Binding_And_Cargo_Capture	0.582827	0.516251	0.603055
Vamp8_associated_trans-Golgi_Network_AP-1_Binding_And_Cargo_Capture	0.258846	0.34809	0.294326

Activation_of_ZAP-70	0.994788	0.949492	0.97679
Phosphorylation_of_ZAP-70_by_Lck	0.994788	0.949492	0.97679
Recruitment_of_ZAP-70_to_phosphorylated_ITAMs	0.994788	0.949492	0.97679
Association_of_Cdh1_with_the_APC/C	0.439846	0.098831	0.903774
Dissociation_of_phospho-Cdh1_from_the_APC/C_complex	0.439846	0.098831	0.903774
Dissociation_of_Cdc20_from_APC/C_complex	0.62858	0.253517	0.903774
Inactivation_of_Lck_by_Csk	0.999509	0.963328	0.997411
3_incision_of_the_lesioned_strand_of_DNA_in_TC-NER	0.860605	0.382619	0.951656
5_incision_leading_to_excision_of_DNA_fragment_with_lesion_in_TC-NER	0.860605	0.382619	0.951656
Cleavage_of_Intronless_Pre-mRNA_at_3-end	0.337865	0.589207	0.19806
Recruitment_of_RNA_Polymerase_III_to_the_TFIIIB:TFIIIC:_Type_2_Promot	0.0160374	0.0812859	0.0503715
RNA_Polymerase_III_Abortive_Initiation_At_Type_2_Open_Promoters	0.0160374	0.0812859	0.0503715
RNA_Polymerase_III_Promoter_Opening_at_Type_2_Promoters	0.0160374	0.0812859	0.0503715
RNA_Polymerase_III_Simple_Start_Sequence_Initiation_At_Type_2_Promot	0.0160374	0.0812859	0.0503715
Adhesion_via_alpha_2_beta_1_glycoprotein	0.899386	0.562575	0.932366
Interaction_of_integrin_alpha_1_beta_1_with_Collagen_IV	0.899386	0.562575	0.932366
Interaction_of_integrin_alpha_10_beta_1_with_Collagen-II	0.899386	0.562575	0.932366
Interaction_of_integrin_alpha_11_beta_1_with_Collagen-I	0.899386	0.562575	0.932366
Adenylate_cyclase_increases_the_GTPase_activity_of_G_alpha-olf	0.60734	0.535982	0.628419
Adenylate_cyclase_increases_the_GTPase_activity_of_Gi_alpha	0.60734	0.535982	0.628419
Dissociation_of_the_Gi_alpha:G_olf_complex	0.60734	0.535982	0.628419
G_alpha-olf:GTP_binds_to_Gi_alpha1:GTP:adenylate_cyclase_complex	0.60734	0.535982	0.628419
Sustained_activation_of_SRC_kinase_by_SHP2	0.947727	0.308139	0.997404
Cdt1_is_displaced_from_the_pre-replicative_complex.	0.0192671	0.477329	0.00702066
Mcm10_associates_with_the_pre-replicative_complex,_stabilizing_Mcm2-7	0.0192671	0.477329	0.00702066
Dephosphorylation_of_Lck-pY505_by_CD45	0.995731	0.963328	0.972872
Association_of_Cyclin_B/Cdk1_with_replicative_origin_inhibits_pre-RC_forn	0.0480859	0.912756	0.00341005
Internal_Methylation_of_mRNA	0.142427	0.027779	0.603973
Interaction_of_integrin_alpha_V_beta_3_with_Fibrillin	0.696896	0.752537	0.509711
Beta-tubulin:GTP:Cofactor_D:alpha-tubulin:GTP:Cofactor_E+_Cofactor_C->_	0.838791	0.427368	0.986674
Beta-tubulin:GTP:Cofactor_D:alpha-tubulin:GTP:Cofactor_E:Cofactor_C->_Be	0.838791	0.427368	0.986674
Mcm2-7_associates_with_the_Cdt1:CDC6:ORC:origin_complex,_forming_th	0.0253637	0.614264	0.00702066
Activation_of_Lck	0.996316	0.963328	0.97679
Phosphorylation_of_ITAM_motifs_in_CD3_complexes	0.996316	0.963328	0.97679
Autocatalytic_phosphorylation_of_FGFR4	0.615091	0.423587	0.703629
FGFR4_binds_to_FGF	0.615091	0.423587	0.703629
Autocatalytic_phosphorylation_of_FGFR2c	0.524575	0.423587	0.593611
Recognition_and_binding_of_the_mRNA_cap_by_the_cap-binding_complex	0.123124	0.0156018	0.603973
Formation_of_the_Cleavage_and_Polyadenylation_Complex	0.201102	0.589207	0.0548623
Interaction_of_integrin_alpha_3_beta_1_with_Thrombospondin-1	0.47624	0.671873	0.20766
Interaction_of_integrin_alpha_IIb_beta_3_with_Thrombospondin	0.800652	0.891923	0.402134
Gi_alpha1:GTP_binds_to_adenylate_cyclase	0.666324	0.611884	0.628419
Interaction_of_integrin_alpha_6_beta_4_with_Laminin-5	0.973199	0.948571	0.850556
Binding_of_SRC_tyrosine_kinase	0.999764	0.718799	0.999957
Src-mediated_phoshorylation_of_FcR1_gamma	0.999764	0.718799	0.999957
Electron_transfer_from_reduced_cytochrome_c_to_molecular_oxygen	0.579824	0.456022	0.709076
Beta-tubulin:GTP:Cofactor_D+alpha-tubulin:GTP:Cofactor_E->_Beta-tubulin:	0.815636	0.366855	0.986674

PI3K_catalytic_subunit_binds_to_Gab1:Grb2:PI3K:EGF:EGFR	0.955207	0.4125	0.99257
PI3K_converts_Phosphatidylinositol-4,5-bisphosphate_to_Phosphatidylinosit	0.955207	0.4125	0.99257
Cyclin_D:Cdk4/6_mediated_phosphorylation_of_Rb_and_dissociation_of_Rb	0.352723	0.538732	0.245334
Association_of_MRN_with_sites_of_DSB_	0.00589435	0.0223818	0.0554584
BRCA1_associates_with_53BP1at_the_site_of_DNA_double-strand_break	0.00703474	0.0040397	0.408656
Sos-mediated_nucleotide_exchange_of_Ras_(EGF:EGFR-Sos:Grb2:Shc)	0.763448	0.696771	0.712971
Autocatalytic_phosphorylation_of_FGFR1c	0.619773	0.423587	0.715633
FGFR1c_binds_to_FGF	0.619773	0.423587	0.715633
Autocatalytic_phosphorylation_of_FGFR3c	0.488282	0.356879	0.593611
FGFR3c_binds_to_FGF	0.488282	0.356879	0.593611
GAP_Recruitment_to_the_Coatomer:Arf1-GTP_Complex	0.193076	0.300566	0.244157
methionine_+_tRNA(Met)_+_ATP_=>_Met-tRNA(Met)_+_AMP_+_pyrophosp	0.672499	0.0959014	0.985684
Exchange_of_ADP_for_ATP_in_CCT/TriC:actin_complex	0.136037	0.0725343	0.598551
Hydrolysis_of_ATP_and_release_of_folded_actin_from_CCT/TriC	0.136037	0.0725343	0.598551
Multi-ubiquitination_of_phospho-beta-catenin_by_SCF-beta-TrCP1	0.118787	0.103184	0.416526
leucine_+_tRNA(Leu)_+_ATP_=>_Leu-tRNA(Leu)_+_AMP_+_pyrophosphate	0.703363	0.117046	0.985684
Binding_of_TFIIA_and_TFIIB_to_the_pol_II_promoter:TFIID_complex	0.154489	0.175882	0.306191
proline_+_tRNA(Pro)_+_ATP_=>_Pro-tRNA(Pro)_+_AMP_+_pyrophosphate	0.600477	0.0594478	0.985684
glutamate_+_tRNA(Glu)_+_ATP_=>_Glu-tRNA(Glu)_+_AMP_+_pyrophosphat	0.725704	0.13503	0.985684
aspartate_+_tRNA(Asp)_+_ATP_=>_Asp-tRNA(Asp)_+_AMP_+_pyrophosphat	0.769294	0.093706	0.990837
snRNP_nuclear_import_and_release	0.488937	0.316907	0.758189
snRNP:Snurportin_complex_formation	0.488937	0.316907	0.758189
snRNA_Cap_hypermethylation	0.503173	0.314749	0.74537
Syk-mediated_phosphorylation_of_Phospholipase_C_gamma_2	0.995612	0.76277	0.998971
ADP_and_Pi_bind_to_ATPase	0.312757	0.188222	0.903319
ATP_is_synthesized_from_ADP_and_Pi_by_ATPase	0.312757	0.188222	0.903319
Enzyme-bound_ATP_is_released	0.312757	0.188222	0.903319
NEXT1_is_cleaved_to_produce_NICD1	0.512921	0.410785	0.614498
NEXT2_is_cleaved_to_produce_NICD2	0.512921	0.410785	0.614498
NEXT3_is_cleaved_to_produce_NICD3	0.512921	0.410785	0.614498
NEXT4_is_cleaved_to_produce_NICD4	0.512921	0.410785	0.614498
GP_Ib-IX-V_binds_to_[vWF:Collagen]_complex	0.992908	0.787739	0.99697
Formation_of_CSL-NICD_coactivator_complex	0.552841	0.457483	0.600096
(ARMS)Rap1-GTP_binds_and_activates_B-Raf	0.300069	0.40018	0.309238
Activated_RAF_complex_binds_MEK	0.627051	0.739456	0.386135
RAF_phosphorylates_MEK1	0.627051	0.739456	0.386135
RAF_phosphorylates_MEK2	0.627051	0.739456	0.386135
phospho-ATM_(Serine_1981)_associates_with_DNA_at_the_site_of_double	0.0200361	0.00287028	0.779206
53BP1_associates_with_gamma_H2AX_at_nuclear_foci	0.0094166	0.0040397	0.588613
IKKbeta_phosphorylates_IkB_causing_NF-kB_to_dissociate	0.994303	0.808728	0.994348
Recruitment_of_Dna2_endonuclease	0.00296095	0.0203112	0.0295859
Removal_of_RNA_primer_and_dissociation_of_RPA_and_Dna2	0.00296095	0.0203112	0.0295859
Repair_synthesis_for_gap-filling__by_DNA_pol_delta__in_TC-NER	0.0528839	0.0063886	0.793648
Repair_synthesis_of_patch_~27-30_bases_long__by_DNA_Pol_Delta	#N/A	#N/A	#N/A
Coat_Complex_Formation	0.296925	0.506781	0.244157
arginine_+_tRNA(Arg)_+_ATP_=>_Arg-tRNA(Arg)_+_AMP_+_pyrophosphate	0.698513	0.093706	0.985684
glutamine_+_tRNA(Gln)_+_ATP_=>_Gln-tRNA(Gln)_+_AMP_+_pyrophosphat	0.698513	0.093706	0.985684

isoleucine_+_tRNA(Ile)_+_ATP_=>_Ile-tRNA(Ile)_+_AMP_+_pyrophosphate	0.698513	0.093706	0.985684
lysine_+_tRNA(Lys)_+_ATP_=>_Lys-tRNA(Lys)_+_AMP_+_pyrophosphate	0.698513	0.093706	0.985684
snRNP_complex_assembly	0.495042	0.314749	0.758189
Release_from_the_NPC_and_Disassembly_of_the_mRNP	0.777079	0.155901	0.922719
Aflatoxin_B1_oxidized_to_Aflatoxin-8,9-oxide	0.992364	0.87869	0.983197
CYP3A4_can_N-demethylate_loperaminde	0.992364	0.87869	0.983197
CYP3A43_catalyzes_the_6beta-hydroxylation_of_testosterone	0.992364	0.87869	0.983197
CYP3A5_oxidises_aflatoxin_B1_to_aflatoxin-8,9-oxide	0.992364	0.87869	0.983197
CYP3A7_can_6beta-hydroxylate_testosterone	0.992364	0.87869	0.983197
CYP4A11_omega-hydroxylates_laurate	0.992364	0.87869	0.983197
CYP4B1_can_12-hydroxylate_arachidonic_acid	0.992364	0.87869	0.983197
CYP4F11_omega-hydroxylates_3-hydroxypalmitate	0.992364	0.87869	0.983197
CYP4F12_hydroxylates_arachidonic_acid	0.992364	0.87869	0.983197
CYP4F2_omega-hydroxylate_leukotriene_B4,_thus_inactivating_it	0.992364	0.87869	0.983197
CYP4F8_hydroxylates_prostaglandin_H2	0.992364	0.87869	0.983197
Dehydrogenation_of_Testosterone_to_form_6-Dehydrotestosterone	0.992364	0.87869	0.983197
Thromboxane_synthase_(CYP5A1)_mediates_the_isomerization_of_prostag	0.992364	0.87869	0.983197
Adhesion_of_alpha_IIb_beta_3_integrin_to_fibrin_network	0.876811	0.957851	0.402134
Glutathione_conjugation_of_cytosolic_substrates	0.740213	0.0850641	0.995593
Binding_of_Syk_tyrosine_kinase	0.993727	0.67217	0.998971
Type_II_receptor_phosphorylates_type_I_receptor	0.700226	0.906965	0.280809
Assembly_of_EGFR_complex_in_clathrin-coated_vesicles	0.367496	0.168453	0.685032
Activated_Adenylate_cyclase_catalyses_cAMP_synthesis	0.552705	0.703989	0.29866
Adenylate_cyclase_converts_ATP_into_cyclic_AMP	0.552705	0.703989	0.29866
Adenylaye_cyclase_increases_the_GTPase_activity_of_G_alpha-olf	0.552705	0.703989	0.29866
G_alpha_(s)_activates_adenylate_cyclase	0.552705	0.703989	0.29866
Galpha-olf:GTP_binds_to_adenylate_cyclase_and_activates_it	0.552705	0.703989	0.29866
The_G_alpha-olf:GDP:Adenylate_cyclase_complex_dissociates	0.552705	0.703989	0.29866
Cbl_escapes_Cdc42-mediated_inhibition_by_down-regulating_the_adaptor_	0.42392	0.271559	0.631874
Interaction_of_integrin_alpha_8_beta_1_with_Tenascin-c	0.494061	0.687934	0.332188
Interaction_of_integrin_alpha_V_beta_3_with_Tenascin	0.494061	0.687934	0.332188
TORC2_(mTOR)_phosphorylates_AKT_at_S473	0.635918	0.306862	0.810962
ABCA1_tetramer_binds_apoA-I	0.614324	0.326108	0.805444
ABCA1-mediated_transport_of_intracellular_cholesterol_to_the_cell_surfac	0.614324	0.326108	0.805444
ABCA1-mediated_transport_of_intracellular_phospholipid_to_the_cell_surfa	0.614324	0.326108	0.805444
ABCA7:Apo1A-mediated_phospholipid_efflux	0.614324	0.326108	0.805444
CaMK_IV_phosphorylates_CREB	0.439782	0.571116	0.327567
Phosphorylation_of_histone_H2AX_at_Serine-139_by_ATM_at_the_site_of_	0.021334	0.00287028	0.886916
Sos-mediated_nucleotide_exchange_of_Ras_(EGF:EGFR-Sos:Grb2)	0.705621	0.617563	0.712971
MDC1/NFBD1associates_with_gamma_H2AX_at_nuclear_foci	0.0075416	0.00287028	0.588613
Association_of_gamma-H2AX_with_NBS1_	0.0016509	0.00287028	0.154474
Association_of_beta-catenin_with__the_SCF-beta-TrCP1_ubiquitin_ligase_co	0.169841	0.126218	0.529093
RPA_binds_to_the_Flap	0.0134829	0.0203112	0.190604
Ubiquitination_of_phospho-p27/p21	0.283716	0.436709	0.222343
Ca2+_influx_through_voltage_gated_Ca2+_channels	0.957226	0.743387	0.962116
CAK-mediated_phosphorylation_of_Cyclin_B1:Cdc2_complexes	0.455278	0.931438	0.117105
Phosphorylation_of_the_Emi1_DSGxxS_degron_by_Cyclin_B:Cdc2	0.494101	0.793039	0.201003

Wee1-_mediated_phosphorylation_of_Cyclin_B1:phospho-Cdc2_complexes	0.636838	0.92895	0.216742
The_signal_peptide_is_excised_from_pre-pro-NGF	0.646554	0.194336	0.857742
Recruitment_of_the_Rad9-Hus1-Rad1_complex_to_DNA	0.0688764	0.47542	0.0130604
Activation_of_IKK_complex	0.90758	0.625574	0.952886
Transport_of_the_export-competent_complex_through_the_NPC	0.78956	0.155901	0.934946
Interaction_of_integrin_alpha_D_beta_2_with_fibrin	0.616197	0.8604	0.278169
Interaction_of_integrin_alpha_X_beta_2_with_fibrin	0.616197	0.8604	0.278169
glycogen_phosphorylase,_liver_form,_tetramer_b_+_ATP_=>_glycogen_pho	0.650521	0.746155	0.366442
Interaction_of_integrin_alpha_4_beta-1_with_Thrombospondin	0.491927	0.722624	0.20766
betaPIX_pushes_CIN85_away_from_Cbl	0.570967	0.33915	0.732562
Electron_transfer_from_ubiquinol_to_cytochrome_c_of_complex_III	0.336138	0.284706	0.584908
PI3-kinase_binds_to_the_active_receptor	0.933218	0.522098	0.957688
PI3K_catalyses_the_phosphorylation_of_PIP2_to_PIP3	0.933218	0.522098	0.957688
TCR_complex_interacts_with_peptide_antigen-presenting_MHC_Class_I	0.501958	0.541252	0.448689
AKT_phosphorylates_IKKalpha	0.89923	0.1957	0.978433
Notch_1-ligand_complex_is_cleaved_to_produce_NEXT1	0.461334	0.387681	0.585632
Notch_2-ligand_complex_is_cleaved_to_produce_NEXT2	0.461334	0.387681	0.585632
Notch_3-ligand_complex_is_cleaved_to_produce_NEXT3	0.461334	0.387681	0.585632
Notch_4-ligand_complex_is_cleaved_to_produce_NEXT4	0.461334	0.387681	0.585632
AKT_phosphorylates_p21Cip1_and_p27Kip1	0.66727	0.351624	0.824529
Interaction_of_integrin_alpha_E_beta_7_with_E-cadherin	0.39334	0.618995	0.231814
Stabilisation_of_RAS:RAF_by_14-3-3	0.751805	0.88189	0.386135
Transient_dissociation_of_14-3-3_upon_Ras_binding	0.751805	0.88189	0.386135
Active_IRS_recruits_PI3K_to_the_plasma_membrane_and_activates_it	0.975864	0.498945	0.993365
Sos-mediated_nucleotide_exchange_of_Ras_(PDGF_receptor:Grb2:Sos)	0.427696	0.572387	0.293475
Endocytosis_(internalization)_of_clathrin-coated_vesicle	0.4247	0.402246	0.487614
Activation_of_S6K1	0.562652	0.19246	0.84293
AKT_can_phosphorylate_forkhead_box_transcription_factors	0.526656	0.0485256	0.909673
Calmodulin_binds_CaMK_IV	0.387304	0.571116	0.238405
CaMK_IV_autophosphorylation	0.387304	0.571116	0.238405
CamKIV_enters_the_nucleus	0.387304	0.571116	0.238405
Activated_type_I_receptor_phosphorylates_R-Smad1/5/8_directly	0.462332	0.859858	0.174831
An_anchoring_protein,_Endofin,_recruits_R-Smad1/5/8	0.462332	0.859858	0.174831
Phospho-R-Smad1/5/8_dissociates_from_the_receptor_complex	0.462332	0.859858	0.174831
Cbl_binds_and_ubiquitinates_Phospho-Sprouty	0.585336	0.363536	0.793429
Cbl_ubiquitinates_Sprouty	0.585336	0.363536	0.793429
Dephosphorylation_of_cyclin_B2:phospho-Cdc2_(Thr_14)_by_Cdc25	0.5452	0.867948	0.241174
Dephosphorylation_of_cytoplasmic_Cyclin_B1:phospho-Cdc2_(Thr_14,_Tyr_	0.5452	0.867948	0.241174
Dephosphorylation_of_nuclear_Cyclin_B1:phospho-Cdc2_(Thr_14,_Tyr15)_c	0.5452	0.867948	0.241174
Phosphorylation_of_the_SA2_Cohesion_Complex	0.73944	0.974714	0.327624
Phosphorylation_of_the_Scc1:Cohesion_Complex	0.73944	0.974714	0.327624
Repair_synthesis_for_gap-filling_by_DNA_pol_epsilon_in_TC-NER	0.286094	0.0879026	0.803451
Repair_synthesis_of_~27-30_bases_long_patch__by_DNA_Pol_Epsilon	#N/A	#N/A	#N/A
Recognition_and_Binding_of_Core_Promoter_Elements_by_TFIID	0.0817563	0.185349	0.142717
Activation_of_NF-kB_complex	0.985014	0.687435	0.990609
Interaction_between_SRP_and_SRP_Receptor	0.238047	0.0726341	0.705037
Formation_of_active_Pol_II_complex_with_lesioned_DNA_template	0.071654	0.0810178	0.240756

Resumption_of_transcription_after_TC-NER	0.071654	0.0810178	0.240756
RNA_Pol_II_is_blocked_by_the_lesion_leading_to_reduced_transcription	0.071654	0.0810178	0.240756
Binding_of_GP_VI:Fc_Epsilon_R1_gamma_receptor_complex_with_collagen	0.996285	0.675645	0.998971
STAT_binds_to_the_active_receptor	0.399239	0.408897	0.443791
Interaction_of_integrin_alpha_V_beta_3_with_vitronectin	0.582733	0.706397	0.402134
Interaction_of_integrin_alpha_V_beta_5_with_vitronectin	0.582733	0.706397	0.402134
Basigin_binds_to_integrins	0.127732	0.211268	0.20766
Heterophilic_interaction_of_PECAM-1_and_Integrin_alpha-v_beta-3	0.645064	0.790989	0.427938
Interaction_of_integrin_alpha_5_beta_1_with_Osteopontin	0.679971	0.790989	0.47676
Interaction_of_integrin_alpha_8_beta_1_with_Osteopontin	0.679971	0.790989	0.47676
Integrin_alpha_5_beta_1_binds_fibronectin	0.740088	0.790989	0.567024
Interaction_of_integrin_alpha_8_beta_1_with_Fibronectin	0.740088	0.790989	0.567024
Interaction_of_integrin_alpha_IIb_beta_3_with_Fibronectin	0.740088	0.790989	0.567024
Interaction_of_integrin_alpha_V_beta_1_with_Fibronectin	0.740088	0.790989	0.567024
Interaction_of_integrin_alpha_V_beta_3_with_Fibronectin	0.740088	0.790989	0.567024
Interaction_of_integrin_alpha_V_beta_6_with_Fibronectin	0.740088	0.790989	0.567024
Interaction_of_integrin_alpha_IIb_beta_3_with_van_Willebrand_factor	0.585393	0.709707	0.402134
Interaction_of_integrin_alpha_V_beta_3_with_von_Willbrand_Factor	0.585393	0.709707	0.402134
The_p75NTR:NgR:MDGI_complex_reduces_RHOA-GDI_activity,_displacing_I	0.886473	0.797961	0.812336
Adenylate_cyclase_produces_cAMP	0.621371	0.796786	0.29866
Dephosphorylation_of_PAG_by_SHP2	0.782818	0.300798	0.969526
AKT_phosphorylates_caspase-9	0.595951	0.224106	0.824529
Activation_of_cytosolic_AMPK_by_phosphorylation	0.0316953	0.0448381	0.156441
Phosphorylation_of_complexed_TSC2_by_PKB	0.606993	0.416171	0.724154
AKT_phosphorylates__TSC2,_inhibiting_it	0.648443	0.294586	0.824529
AKT_phosphorylates_GSK3	0.680926	0.1957	0.869363
Interaction_of_Tie2_and_p85_of_PI3K	0.965654	0.228128	0.996129
Interaction_of_integrin_alpha_V_beta_3_with_Bone_sialoprotein_2	0.760281	0.790989	0.599036
Notch_1_heterodimer_binds_with_a_Notch_ligand_in_the_extracellular_sp	0.572217	0.387681	0.778534
Notch_2_heterodimer_binds_with_a_Notch_ligand_in_the_extracellular_sp	0.572217	0.387681	0.778534
Notch_3_heterodimer_binds_with_a_Notch_ligand_in_the_extracellular_sp	0.572217	0.387681	0.778534
Notch_4_heterodimer_binds_with_a_Notch_ligand_in_the_extracellular_sp	0.572217	0.387681	0.778534
AKT_phosphorylates_MDM2	0.566468	0.1957	0.776816
Phosphorylation_of_proteins_involved_in_the_G1/S_transition_by_Cyclin_A	0.397004	0.748021	0.109216
AKT_phosphorylates_PRAS40	0.404084	0.0635591	0.824529
Phosphorylation_and_activation_of_eIF4G_by_activated_S6K1	0.669526	0.339212	0.817131
ICAMs_1-4_bind_to_Integrin_LFA-1	0.441864	0.632259	0.292999
Binding_of_phospho-p27/p21:Cdk2:Cyclin_E/A_to_the_SCF(Skp2):Cks1_com	0.391637	0.516795	0.28571
CTMP_and/or_TRB3_inhibit_AKT_phosphorylation	0.354699	0.200429	0.581316
PKB_attachment_to_plasma_membrane	0.354699	0.200429	0.581316
Caveolin-1_dissociates_from_eNOS:CaM:HSP90_complex	0.591467	0.175526	0.934081
HSP90_binds_eNOS:Caveolin-1:CaM_complex	0.591467	0.175526	0.934081
AKT_phosphorylates_BAD	0.522677	0.14635	0.824529
PI3K_activation	0.972407	0.401292	0.996298
Gab1:Grb2:PI3K_binds_to_EGF:Phospho-EGFR	0.931182	0.226272	0.997808
Notch_1_precursor_cleaved_to_form_a_heterodimer	0.743499	0.547505	0.812475
Notch_2_precursor_cleaved_to_form_a_heterodimer	0.743499	0.547505	0.812475

Notch_3_precursor_cleaved_to_form_a_heterodimer	0.743499	0.547505	0.812475
Notch_4_precursor_cleaved_to_form_a_heterodimer	0.743499	0.547505	0.812475
Axonal_transport_of_NGF:Trk_complexes	0.284356	0.280991	0.41073
Sos-mediated_nucleotide_exchange_of_Ras_(Tie2_receptor:Grb2:Sos)	0.522354	0.366163	0.733705
Phospho-IKK_Complex_phosphorylates_IkB_within_the_IkB:NFkB_Complex	0.991299	0.646406	0.997123
Removal_of_remaining_Flap	0.0290097	0.0454105	0.190604
Multiple_proteins_are_localized_at_replication_fork	0.625227	0.697144	0.517179
Loading_of_PCNA_-_Sliding_Clamp_Formation	0.210708	0.260591	0.310388
RFC_dissociates_after_sliding_clamp_formation	0.210708	0.260591	0.310388
Ubiquitination_of_stimulated_EGFR_(Cbl:Grb2)	0.424203	0.18003	0.793429
Cargo,_Sec31p:Sec13p,_and_v-SNARE_recruitment	0.895693	0.777962	0.869611
Vesicle_Budding	0.895693	0.777962	0.869611
Vesicle_Uncoating	0.895693	0.777962	0.869611
Cyclin_B/Cdk1_mediate_phosphorylation_of_NuMA	0.545523	0.886176	0.201003
Phosphorylation_of_Cdh1_by_Cyclin_B1:Cdc2	0.369957	0.691964	0.201003
I-Smad_binds_to_type_I_receptor,_preventing_Smad1/5/8_from_being_act	0.524756	0.815345	0.158384
Autocatalytic_phosphorylation_of_FGFR3b	0.325428	0.159338	0.591139
FGFR3b_binds_to_FGF	0.325428	0.159338	0.591139
Initiation_of_RNA_Polymerase_III_Productive_Transcription	0.0142737	0.0390356	0.0913417
Resumption_of_RNA_Polymerase_III_Productive_Transcription	0.0142737	0.0390356	0.0913417
RNA_Polymerase_III_Productive_Transcription	0.0142737	0.0390356	0.0913417
RNA_Polymerase_III_Retractive_RNase_Activity_at_U-tract_Pause_Sites	0.0142737	0.0390356	0.0913417
RNA_Polymerase_III_Termination_and_release_of_transcribed_mRNA	0.0142737	0.0390356	0.0913417
RNA_Polymerase_III_Transcriptional_Pause_at_Terminator_Sequence	0.0142737	0.0390356	0.0913417
chylomicron_=>_TG-depleted_chylomicron_+_50_long-chain_fatty_acids_+_	0.90929	0.624437	0.968815
LILRs_interact_with_MHC_Class_I	0.105381	0.367389	0.0458507
Platelet_glycoprotein_IV_[plasma_membrane]_GP_IV_:_Collagen_IV_compl	0.993272	0.823927	0.996138
MAC1_binds_JAM-C	0.144877	0.15858	0.278169
vWF_binds_to_collagen	0.974603	0.593111	0.996138
Integrin_alphaX_beta2_binds_JAM-C	0.297373	0.355151	0.342991
alpha-tubulin:GTP:Cofactor_B__+Cofactor_E_->__alpha-tubulin:GTP:_Cofact	0.572725	0.431485	0.761318
Association_of_Rb_with_Cyclin_E:Cdk2_complexes	0.635267	0.933613	0.260931
Cyclin_E:Cdk2-mediated_phosphorylation_of_Rb	0.635267	0.933613	0.260931
AKT_can_phosphorylate_NUR77	0.52955	0.289668	0.724154
Transport_(efflux)_of_bile_salts_by_ABCC3_(MRP3)	0.978946	0.936107	0.910471
Surface_deployment_of_alpha_IIb_beta_3_integrin	0.638941	0.790989	0.402134
Phosphorylation_and_inactivation_of_eEF2K_by_activated_S6K1	0.379115	0.129918	0.724154
Guanine_nucleotide_exchange_on_Ral	0.906633	0.69752	0.966352
Activation_of_SHP2_through_the_binding_to_phospho-Gab1	0.576288	0.300798	0.857811
Dephosphorylation_of_Gab1_by_SHP2	0.576288	0.300798	0.857811
SHP2_dephosphorylates_Tyr_992_on_EGFR	0.576288	0.300798	0.857811
Phosphorylation_of_TSC2_by_PKB	0.533349	0.294586	0.724154
PDK1_phosphorylates_AKT_at_T308	0.605112	0.1957	0.824529
Phosphorylation_of_PKB_by_PDK1	0.605112	0.1957	0.824529
PIP3_recruits_PDK1_and_AKT_to_the_membrane	0.605112	0.1957	0.824529
AKT_phosphorylates_CREB	0.535936	0.1957	0.763254
Autocatalytic_phosphorylation_of_FGFR2b	0.663512	0.0552235	0.908684

p130Cas_and_C3G_bind_PDGFR_bound_Crk	0.161686	0.226242	0.260946
Phosphorylation_and_activation_of_eIF4B_by_activated_S6K1	0.358969	0.11375	0.724154
Akt1_phosphorylates_BAD_protein	0.398182	0.14635	0.724154
CAK-mediated_phosphorylation_of_Cyclin_E:Cdk2	0.760405	0.931438	0.351482
Inactivation_of_PDC_by_phosphorylation_of_PDC_E1_alpha_component	0.761602	0.627262	0.887457
Phosphorylation_of_Ribosomal_protein_S6_by_activated_S6K1	0.511863	0.1957	0.739821
Interaction_of_integrin_alpha_9_beta_1_with_tenascin-c	0.137494	0.272245	0.182026
PI3K_produces_PIP3_and_other_phosphatidyl_inositides	0.973747	0.333478	0.996298
Phosphorylation_of_Cyclin_E:Cdk2_complexes	0.892758	0.92895	0.586809
Formation_of_Okazaki_fragments	0.0175015	0.0249101	0.190604
Formation_of_Processive_Complex	0.0175015	0.0249101	0.190604
Formation_of_the_Flap_Intermediate	0.0175015	0.0249101	0.190604
Phosphorylation_of_EGFR_by_SRC_kinase	0.926989	0.456547	0.989292
RFC_binding_displaces_Pol_Alpha	0.301287	0.410016	0.310388
RFC_binding_displaces_Pol_Alpha_on_the_C-strand_of_the_telomere	0.301287	0.410016	0.310388
Ubiquitination_of_stimulated_EGFR_(Cbl)	0.559343	0.297331	0.793429
Free_CDT1_associates_with_CDC6:ORC:origin_complexes	0.00463464	0.53756	0.00134939
Formation_of_Cyclin_B:Cdc2_complexes	0.573172	0.933613	0.201003
Myt-1_mediated_phosphorylation_of_Cyclin_B:Cdc2_complexes	0.573172	0.933613	0.201003
Phosphorylation_of_Cyclin_B1_in_the_CRS_domain	0.573172	0.933613	0.201003
Phosphorylation_of_M_phase_proteins_by_active_Cyclin_B1:Cdc2_complex	0.573172	0.933613	0.201003
Translocation_of_CRS_phosphorylated_Cyclin_B1:Cdc2_complexes	0.573172	0.933613	0.201003
Translocation_of_Cyclin_B1:phospho-Cdc2_complexes_to_the_nucleus	0.573172	0.933613	0.201003
Translocation_of_Cyclin_B1:phospho-Cdc2_to_the_cytoplasm	0.573172	0.933613	0.201003
Sos:Grb2_binds_to_Phospho-Shc:EGF:Phospho-EGFR	0.407342	0.371193	0.546779
Basigin_binds_Mannose-carrying_cell_recognition_molecules	0.318799	0.335168	0.400951
Recruitment_of_Dna2_endonuclease_to_the_C_strand	0.00306349	0.00646437	0.122813
Removal_of_RNA_primer_and_dissociation_of_RPA_and_Dna2_from_the_C	0.00306349	0.00646437	0.122813
SOS_mediated_nucleotide_exchange_of_RAS_(SHC)	0.835946	0.811324	0.663371
SOS_mediated_nucleotide_exchange_of_RAS_(IRS)	0.531626	0.445978	0.663371
Phoshorylation_of_PECAM-1_by_Fyn_or_Lyn_or_c-Src	0.990683	0.839354	0.98531
Sculpting_and_pinching-off_of_Golgi_vessicle	0.158046	0.0210935	0.480414
Melanocortin_receptors_bind_melanocortins	0.403703	0.135857	0.744197
eIF2_activation	0.467902	0.0732965	0.972059
Formation_of_eIF2:GDP:eIF2B_intermediate	0.467902	0.0732965	0.972059
Activation_of_TAK1-TAB2_complex	0.589592	0.560003	0.57077
Association_of_NuMA_with_microtubules	0.81919	0.64215	0.894625
Ubiquitination_of_Emi1_by_SCF-beta-TrCP	0.131116	0.150626	0.328792
Association_of_CCT/TriC_with_sphingosine_kinase_1	0.18977	0.18188	0.434699
KIR2DS1_interacting_with_HLA-C_group_2_(Cw3)	0.277653	0.519508	0.103345
NKG2A-CD94_heterdimer_interacts_with_HLA-E	0.330365	0.436147	0.305473
alpha-tubulin:GTP_+_Cofactor_B_->_alpha-tubulin:GTP:_Cofactor_B	0.595173	0.447191	0.761318
alpha-tubulin:GTP_+_Cofactor_E_->__alpha-tubulin:GTP:Cofactor_E	0.774363	0.676793	0.761318
PKA_phosphorylates_PDE4B	0.797778	0.568438	0.805834
Activated_type_I_receptor_phosphorylates_R-SMAD_directly	0.652855	0.449113	0.75276
An_anchoring_protein,_SARA,_recruits_R-SMAD	0.652855	0.449113	0.75276
Phospho-R-SMAD_dissociates_from_the_receptor_complex	0.652855	0.449113	0.75276

Activation_of_BAD_by_calcineurin	0.209706	0.461173	0.148311
LFA1_binds_JAM-A	0.332967	0.580949	0.20766
DARPP-32_is_dephosphorylated_on_Thr34_by_PP2B	0.200685	0.393308	0.153913
gamma-secretase_cleaves_the_p75NTR_transmembrane_domain	0.443187	0.300604	0.588739
Autocatalytic_phosphorylation_of_Klotho-bound_FGFR1c	0.413793	0.384491	0.49433
FGFR1c_binds_to_Klotho-bound_FGF23	0.413793	0.384491	0.49433
Localization_of_Cbl:Grb2_to_the_membrane	0.571009	0.226272	0.896346
Phosphorylation_of_Cbl_(EGFR:Cbl:Grb2)	0.571009	0.226272	0.896346
Calcium_Influx_through_Voltage-gated_Calcium_Channels	0.857073	0.698304	0.857806
AKT_can_phosphorylate_SRK	0.478266	0.1957	0.724154
AKT_translocates_to_the_nucleus	0.478266	0.1957	0.724154
PHLPP_dephosphorylates_S473_in_AKT	0.478266	0.1957	0.724154
Dephosphorylation_of_Cyclin_E:Cdk2_complexes_by_Cdc25A	0.846387	0.867948	0.627808
Mcm2-7_is_phosphorylated_by_DDK	0.854428	0.65519	0.847308
Activation_of_Src	0.747369	0.352057	0.872131
SH2_domain_of_Src_binds_to_the_active_receptor	0.747369	0.352057	0.872131
PI3K_phosphorylates_PIP2_to_PIP3	0.993756	0.638855	0.996298
Recruitment_of_PI3K_to_plasmamembrane	0.993756	0.638855	0.996298
(Frs2)Rap1-GTP_binds_to_and_activates_B-Raf	0.236422	0.38693	0.233533
Phosphorylation_of_Cyclin_D:Cdk4/6_complexes	0.355363	0.542616	0.177225
Dissociation_of_beta-catenin_from_Axin_and_association_of_beta_catenin_	0.278823	0.223362	0.557738
Phosphoryation_of_phospho-_(Ser45,_Thr41)_beta-catenin__at_Ser37_by_(	0.278823	0.223362	0.557738
Phosphorylation_of_APC_component_of_the_destruction_complex	0.278823	0.223362	0.557738
Phosphorylation_of_beta-catenin_at_Ser45_by_CK1_alpha	0.278823	0.223362	0.557738
Phosphorylation_of_phospho-(Ser45_)_at_Thr_41_by_GSK-3	0.278823	0.223362	0.557738
Phosphorylation_of_phospho-(Ser45,Thr41,Ser37)_at_Ser33_by_GSK-3	0.278823	0.223362	0.557738
3,5,3-triiodothyronine_+_PAPS_=>_3,5,3-triiodothyronine_4-sulfate_+_PAP	0.978399	0.889526	0.948758
Phosphorylation_of_Cyclin_E2:Cdk2_complexes_by_Myt1	0.959437	0.92895	0.829188
Phosphorylation_of_Cyclin_E1:Cdk2_complexes_by_Myt1	0.71657	0.92895	0.1685
L-Glutamate_loading_of_synaptic_vesicle	0.559519	0.417979	0.695434
Recruitment_of_ITK_to_SLP-76	0.295008	0.408721	0.280532
CDC6_association_with_ORC:origin_complexes_mediated_by_MCM8	0.00282571	0.544194	0.00134939
Association_of_Cyclin_A:phospho-Cdk2(Thr_160)_with_E2F1/E2F3	0.838832	0.888679	0.446872
Phosphorylation_of_E2F1/E2F3_by_Cyclin_A:phosph-Cdk2(Thr_160)	0.838832	0.888679	0.446872
RPA_binds_to_the_Flap_on_the_C-strand	0.0174992	0.00646437	0.729934
Proteolysis_of_the_IGF:IGFBP-3:ALS_Complex_by_Matrix_Metalloproteinase	0.0895399	0.141388	0.210113
Recruitment_of_Rad17-RFC_complex_to_DNA	0.0966473	0.159787	0.201011
Binding_of_IRS_to_insulin_receptor	0.655066	0.564698	0.696695
Dissociation_of_IRS-P_from_insulin_receptor	0.655066	0.564698	0.696695
Phosphorylation_of_IRS	0.655066	0.564698	0.696695
Neuropeptide_Y_receptors_can_bind_neuropeptide_Y-related_peptides	0.323011	0.742772	0.138731
Docking_of_the_Mature_intronless_derived_transcript_derived_mRNA,_TAI	0.761646	0.332676	0.891543
Trace_amine-associated_receptors_can_bind_trace_amines	0.946152	0.872665	0.871615
nascent_chylomicron_+_spherical_HDL:apoC-II:apoC-III:apoE_=>spherical__I	0.956964	0.743692	0.968815
TG-depleted_chylomicron_+_spherical_HDL_=>_chylomicron_remnant_+_sp	0.956964	0.743692	0.968815
Creation_of_the_Membrane_Attack_Complex_(MAC)	0.294239	0.466581	0.232989
Release_of_Tubulin_alpha-4A_chain	0.803683	0.712662	0.761318

Glutathione_conjugation_of_luminal_substrates	0.245295	0.118696	0.622355
MHC_Class_I_interacts_with_CD160	0.118838	0.288414	0.123413
KIR2DL1_interacting_with_HLA-C_group_2_(Cw3)	0.141016	0.318064	0.103345
KIR2DL2/3_interacting_with_HLA-C_group_1_(Cw4)	0.141016	0.318064	0.103345
KIR2DL4_interacting_with_HLA-G	0.141016	0.318064	0.103345
KIR3DL1_interacting_with_HLA_Bw4	0.141016	0.318064	0.103345
KIR3DL2_interacting_with_HLA-A3	0.141016	0.318064	0.103345
NGF_binding_to_p75NTR_inactivates_RHOA	0.297611	0.259563	0.473387
eNOS:Caveolin-1:NOSTRIN:dynamin-2_complex_binds_N-WASP	0.573311	0.124298	0.893196
Interaction_of_integrin_alpha_4_beta-1_with_Osteopontin	0.254853	0.355151	0.29296
Interaction_of_integrin_alpha_9_beta_1_with_Osteopontin	0.254853	0.355151	0.29296
Interaction_of_integrin_alpha_4_beta-1_with_Fibronectin	0.326709	0.355151	0.383599
eNOS:Caveolin-1_complex_binds_to_CaM	0.174377	0.0877425	0.65144
VLA-4_binds_JAM-B	0.282192	0.355151	0.327568
Interaction_of_integrin_alpha_9_beta_1_with_VCAM-1	0.223944	0.355151	0.253788
VCAM-1interacts_with_VLA-4	0.223944	0.355151	0.253788
LTC4_is_exported_from_the_cell	0.985127	0.936107	0.951039
VEGF-A,C,D,E_bind_to_VEGFR2_leading_to_receptor_dimerization	0.0339618	0.0546037	0.126049
ERKs_are_inactivated_by_dual-specific_phosphatases_(DUSPs)	0.448653	0.132817	0.935375
binding_of_Cbl_to_EGFR	0.722038	0.376786	0.896346
Phosphorylation_of_Cbl_(EGFR:Cbl)	0.722038	0.376786	0.896346
Ral-GDS_binds_to_Ras-GTP	0.946519	0.772842	0.966352
Association_of_DFF_with_alpha:beta_importin	0.493264	0.151907	0.807398
Translocation_of_DFF_to_the_nucleus	0.493264	0.151907	0.807398
MadCAM_interacts_with_Integrin_alpha-4_beta-7	0.26143	0.355151	0.301294
factor_XI:platelet_glycoprotein_(GP)_Ib:IX:V_complex_->_factor_XIa:platele	0.686416	0.617278	0.654223
Caspase-mediated_cleavage_of_gelsolin	0.173105	0.0755787	0.586788
factor_IX_->_factor_IXa_+_factor_IX_activation_peptide_(factor_XIa_catalys	0.852777	0.753626	0.785747
I-SMAD_competes_with_R-SMAD_for_type_I_receptor	0.621789	0.542587	0.659251
Activation_of_Rap1	0.658616	0.428066	0.93601
cAMP_induces_dissociation_of_inactive_PKA_tetramers	0.981657	0.765488	0.977924
Gab1_binds_phosphatidylinositol-3,4,5-trisphosphate	0.304549	0.226272	0.591346
Gab1_phosphorylation_by_EGFR_kinase	0.304549	0.226272	0.591346
Gab1:Grb2_binds_to_EGF:Phospho-EGFR	0.304549	0.226272	0.591346
factor_XI:platelet_glycoprotein_(GP)_Ib:IX:V_complex_->_factor_XIa:platele	0.843195	0.838525	0.654223
RIT/RIN-GTP_binds_B-Raf	0.666944	0.699241	0.492465
CAK-mediated_phosphorylation_of_Cyclin_A:Cdc2_complexes	0.678788	0.931438	0.156831
CAK-mediated_phosphorylation_of_Cyclin_A:Cdk2	0.678788	0.931438	0.156831
Shc_binds_to_the_phospho-receptor:ligand_complex	0.583773	0.529675	0.646675
Shc_phosphorylation_by_phospho-EGFR:EGF	0.583773	0.529675	0.646675
BMP2_binds_to_the_receptor_complex	0.562468	0.91856	0.158384
3,3-diiodothyronine_+_PAPS_=>_3,3-diiodothyronine_4-sulfate_+_PAP	0.948521	0.72693	0.948758
p75NTR_ICD_signals_to_NF-kB	0.978515	0.873129	0.952672
Basigin_binds_CD98_complex	0.716851	0.799152	0.512621
Association_of_MCM8_with_ORC:origin_complex	0.00324138	0.544194	0.00141592
C3G_stimulates_nucleotide_exchange_on_Rap1	0.387164	0.328402	0.565927
The_ligand_trap_binds_the_ligand_BMP2,_blocking_BMP_signalling	0.289875	0.429984	0.224798

(Frs2)C3G_stimulates_nucleotide_exchange_on_Rap1	0.287221	0.273347	0.496327
Sos:Grb2_complex_binds_to_EGF:EGFR_complex	0.283627	0.226272	0.546779
TRAF6:Phosho-TAK1:Tab1:PhosphoTAB2_mediated_phosphorylation_of_MA	0.239619	0.12292	0.695354
Myt-1_mediated_phosphorylation_of_Cyclin_A:Cdc2	0.85754	0.92895	0.374044
Phosphorylation_of_Cyclin_A:Cdk2_at_Tyr_15	0.85754	0.92895	0.374044
Wee1-mediated_phosphorylation_of_Cyclin_A:phospho-Cdc2_complexes	0.85754	0.92895	0.374044
SOS_phosphorylation_and_dissociation_(SHC)	0.292143	0.17891	0.666435
p62_recruits_an_atypical_PKC	0.820753	0.856276	0.568795
SOS_phosphorylation_and_dissociation_(IRS)	0.0587036	0.016821	0.666435
Coat_Assembly	0.857447	0.910644	0.320762
Loss_of_Sar1p_GTPase	0.857447	0.910644	0.320762
IKK-beta_is_recruited	0.962882	0.69625	0.972257
IKKbeta_is_activated	0.962882	0.69625	0.972257
Phosphorylated_Emi1_binds_the_beta-TrCP_in_the_SCF_complex	0.197316	0.187536	0.468466
Nrage_sequesters_Che1_in_the_cytoplasm	0.487208	0.112076	0.767764
Caspase-mediated_cleavage_of_Desmoglein_1	0.431741	0.0633348	0.933265
Caspase-mediated_cleavage_of_Desmoglein_2	0.431741	0.0633348	0.933265
Caspase-mediated_cleavage_of_Desmoglein_3	0.431741	0.0633348	0.933265
Phosphorylation_of_JNK_by_the_Activated_TRAF6:Phospho-TAK1:TAB1:Pho	0.372942	0.473342	0.338145
Myelin_components_can_interact_with_p75NTR:NgR:LINGO1	0.996348	0.991875	0.906548
TRAF6:Phosho-TAK1:Tab1:PhosphoTAB2_mediated_phosphorylation_of_the	0.874314	0.439717	0.972915
SOS_phosphorylation_and_dissociation_(IRS,_Crk)	0.116845	0.0463838	0.666435
Binding_of_SNAPc,_Oct-1,_and_Staf_to_Type_3_Promoter	0.608396	0.503405	0.625489
Beta-tubulin:GTP:_Cofactor_A+_Cofactor_D_->__Beta-tubulin:GTP:Cofactor_	0.592203	0.135857	0.947871
Granzyme-B_activates_BID_by_cleavage	0.996735	0.981289	0.971246
Binding_of_SHC_to_insulin_receptor	0.911854	0.898555	0.696695
Dissociation_of_SHC-P_from_insulin_receptor	0.911854	0.898555	0.696695
Phosphorylation_of_SHC	0.911854	0.898555	0.696695
Signal_Recognition_(Preproinsulin)	0.386138	0.119637	0.820333
Binding_of_Rrn3_to_RNA_Polymerase_I	0.680917	0.435111	0.81757
Acetylcholine_clearance_from_synaptic_cleft	0.817706	0.74656	0.731524
Exocytosis_of_alpha_1_antitrypsin	0.398041	#N/A	0.398041
Conversion_of_C4_into_C4a_and_C4b	0.835141	0.880093	0.579967
kallikrein_+_alpha2-macroglobulin_->_kallikrein:alpha2-macrogloulin	0.418409	0.463829	0.425435
CYP1B1_4-hydroxylates_estradiol-17beta	0.448676	0.730526	0.276653
CYP2D6_4-hydroxylates_debrisoquine	0.448676	0.730526	0.276653
CYP2W1_can_oxidize_indole	0.448676	0.730526	0.276653
Dehydrogenation_of_Sparteine_to_form_2-Dehydrosparteine	0.448676	0.730526	0.276653
Activation_of_C5	0.678944	0.834355	0.28466
Cleavage_of_C3_by_C3_convertase	0.678944	0.834355	0.28466
prekallikrein:kininogen:C1q_binding_protein_tetramer_->_kallikrein:kininog	0.39842	0.539686	0.321474
4-cholesten-7alpha,_12alpha-diol-3-one_is_reduced_to_5beta-cholesten-7a	0.298657	0.330009	0.378372
4-cholesten-7alpha,12alpha,24(S)-triol-3-one_is_reduced_to_5beta-cholesta	0.298657	0.330009	0.378372
4-cholesten-7alpha,12alpha,27-triol-3-one_is_reduced_to_5beta-cholestan-7	0.298657	0.330009	0.378372
4-cholesten-7alpha,24(S)-diol-3-one_is_reduced_to_5beta-cholestan-7alpha	0.298657	0.330009	0.378372
4-cholesten-7alpha,27-diol-3-one_is_reduced_to_5beta-cholestan-7alpha,27	0.298657	0.330009	0.378372
4-cholesten-7alpha-ol-3-one_is_reduced_to_5beta-cholestan-7alpha-ol-3-or	0.298657	0.330009	0.378372

Reduction_of_isocaproaldehyde_to_4-methylpentan-1-ol	0.298657	0.330009	0.378372
Release_of_Kappa-actin	0.298316	0.15532	0.697427
SHP2_binds_to_the_active_receptor	0.372498	0.355719	0.46421
p75NTR_and_RHOA-GDI_interact	0.284316	0.259563	0.469979
p75NTR_reduces_RHO-GDI_activity	0.284316	0.259563	0.469979
Homodimerization_of_VEGF_proteins	0.0237997	0.179212	0.029976
Replication_initiation_regulation_by_Rb1/E2F1	0.245334	#N/A	0.245334
ERK1/2/5_activate_RSK1/2/3	0.0462483	0.0273273	0.422642
Inactive_catalytic_PP2B_is_activated_by_the_binding_of_calmodulin	0.29712	0.585032	0.110237
Nck_binds_to_the_active_PDGF_receptor	0.206061	0.435488	0.117114
pAMPK_inactivates_ACC2_inhibiting_malonyl-CoA_synthesis	0.157866	0.365064	0.162502
Activation_of_PLC_beta-1/4	0.444559	0.84336	0.188257
Inactivation_of_PLC_beta	0.444559	0.84336	0.188257
PIP2_hydrolysis	0.444559	0.84336	0.188257
Formation_of_clathrin-coated_vesicle	0.482798	0.331882	0.645662
NICD1_trafficks_to_the_nucleus	0.647567	0.561388	0.668868
NICD2_trafficks_to_the_nucleus	0.647567	0.561388	0.668868
NICD3_trafficks_to_the_nucleus	0.647567	0.561388	0.668868
NICD4_trafficks_to_the_nucleus	0.647567	0.561388	0.668868
Notch_1_heterodimer_trafficks_to_the_plasma_membrane	0.647567	0.561388	0.668868
Notch_1_precursor_transport_to_golgi	0.647567	0.561388	0.668868
Notch_2_heterodimer_trafficks_to_the_plasma_membrane	0.647567	0.561388	0.668868
Notch_3_heterodimer_trafficks_to_the_plasma_membrane	0.647567	0.561388	0.668868
Notch_4_heterodimer_trafficks_to_the_plasma_membrane	0.647567	0.561388	0.668868
Proteolysis_of_the_IGF:IGFBP-3:ALS_Complex_by_Cathepsin_L	0.00226693	0.115677	0.000642544
Formation_of_Cyclin_E:Cdk2_complexes	0.894503	0.933613	0.60421
Translocation_of_Cyclin_E:Cdk2_complex__to_the_nucleus	0.894503	0.933613	0.60421
Phosphorylation_of_ChREBP_at_Serine_568_by_AMPK	0.204032	0.377909	0.195369
Cleavage_of_Procaspase-7_by_the_apoptosome	0.343518	0.348412	0.454046
Cleavage_of_ Procaspase-3_by_the_apoptosome	0.343518	0.348412	0.454046
Association_of_INK4A_with_Cdk4/6	0.0742706	0.0937603	0.262048
PEX-19_docks_ABCD1/ABCD3_to_peroximal_membrane	0.01768	0.0472779	0.100642
Caspase_mediated_cleavage_of_alpha-II-Fodrin	0.573248	0.0861585	0.947983
Creatine_transport_across_the_plasma_membrane	0.553026	0.45436	0.719606
SLC6A12_(BGT-1)-mediated_uptake_of_GABA_and_betaine	0.553026	0.45436	0.719606
SLC6A6-mediated_uptake_of_taurine_and_beta-alanine	0.553026	0.45436	0.719606
phospho-RAF_binds_14-3-3	0.322231	0.590657	0.233533
Active_PLC-gamma1_dissociates_from_EGFR	0.588653	0.530585	0.646675
EGFR_activates_PLC-gamma1_by_phosphorylation	0.588653	0.530585	0.646675
Phospholipase_C-gamma1_binds_to_the_activated_EGF_receptor	0.588653	0.530585	0.646675
14-3-3epsilon_attentuates_NADE-related_apoptosis	0.594103	0.947315	0.321062
MCM2-7_mediated_fork_unwinding	0.539443	0.624012	0.459177
Mcm4,6,7_trimer_forms_and_associates_with_the_replication_fork	0.539443	0.624012	0.459177
Fc_gamma_receptors_interact_with_antigen-bound_IgG	0.224505	0.823268	0.10658
p75NTR:NADE_promotes_caspase2/3_activation	0.525172	0.51793	0.517602
G_Protein_trimer_formation_(olfactory)	0.296259	0.249292	0.580033
Activation_of_Rac1	0.971596	0.852367	0.947085

Grb2/Sos1_complex_binds_to_the_active_receptor	0.0517157	0.113486	0.137153
Association_of_p21/p27_with_Cyclin_E/Cdk2_complexes	0.873833	0.926243	0.331893
Cyclin_E/A:Cdk2-mediated__phosphorylation_of_p27/p21	0.873833	0.926243	0.331893
Inactivation_of_Cyclin_A:Cdk2_complexes_by_p27/p21	0.873833	0.926243	0.331893
Inactivation_of_Cyclin_E:Cdk2_complexes_by_p27/p21	0.873833	0.926243	0.331893
LKB1_phosphorylates_the_alpha_subunit_of_AMPK_heterotrimer	0.0798632	0.129499	0.195369
Crk_binds_to_the_active_PDGF_receptor	0.114715	0.262346	0.14403
Cdc25A_mediated_dephosphorylation_of_Cyclin_A:phospho-Cdk2	0.792128	0.867948	0.432362
Dephosphorylation_of_nuclear_Cyclin_A:phosph-Cdc2_complexes	0.792128	0.867948	0.432362
Caspase-mediated_cleavage_of_Etk	0.207118	0.381831	0.161638
DNA_polymerase_alpha:primase_binds_at_the_origin	0.285536	0.513865	0.201741
The_primase_component_of_DNA_polymerase:primase_synthesizes_a_6-1(	0.285536	0.513865	0.201741
The_primase_component_of_DNA_polymerase:primase_synthesizes_a_6-1(	0.285536	0.513865	0.201741
Orc6_associates_with_Orc1:Orc4:Orc5:Orc3:Orc2:origin_complexes,_formin	0.00141592	#N/A	0.00141592
GTP-bound_RAC_contributes_to_JNK_activation	0.415197	0.74066	0.300211
Assembly_of_the_destruction_complex	0.469636	0.422246	0.557738
Association_of_beta-catenin_with_the_destruction_complex	0.469636	0.422246	0.557738
NRP-1_forms_a_ternary_complex_with_VEGF165_and_VEGFR1	0.32588	0.173008	0.619597
NRAGE_activates_JNK	0.576444	0.689867	0.454347
Caspase-mediated_cleavage_of_E-Cadherin	0.136173	0.115445	0.496242
POL_delta_associates_with_AP_site_displacing_POL_Beta	0.0785585	0.0229551	0.645478
p-nitrophenol_+_PAPS_=>_p-nitrophenol_sulfate_+_PAP	0.942429	0.889526	0.840317
Phosphorylation_of_4E-BP1_by_activated_mTORC1	0.321119	0.13225	0.780229
Vamp7_associated_Lysosomal_vesicle_docking_and_fusion	0.875278	0.410639	0.989599
Inactivation_of_MEK1_by_p34cdc2	0.670837	0.751429	0.331893
VEGF-A,B,PLGF_bind_to_VEGFR1_leading_to_receptor_dimerization	0.212655	0.0925068	0.689247
factor_XI_+_platelet_glycoprotein_(GP)_Ib:IX:V_complex_->_factor_XI:plate	0.778232	0.753626	0.654223
cytidine_+_ATP_=>_cytidine_5-monophosphate_(CMP)_+_ADP	0.00892036	0.00900396	0.224934
Ligation_of_DNA_at_sites_of_patch_replacement	0.0374137	0.00724017	0.645478
Phosphorylation_of_the_Emi1_DSGxxS_degron_by_Plk1	0.669334	0.644914	0.591947
Activation_and_Release_of_the_TRAF6:Phospho-Tak1:Tab1:Phospho-Tab2_(	0.259976	0.326234	0.323095
Formation_of_the_Viral_dsRNA:TLR3:TRIF:TRAF6:TAB1:TAB2_Complex	0.259976	0.326234	0.323095
2-(alpha-hydroxyethyl)-TPP_+_lipoamide_=>_S-acetyldihydrolipoamide_+_T	0.668816	0.473967	0.887457
dihydrolipoamide_+_FAD_=>_lipoamide_+_FADH2_[pyruvate_dehydrogena:	0.668816	0.473967	0.887457
FADH2_+_NAD+_=>_FAD_+_NADH_+_H+_[pyruvate_dehydrogenase]	0.668816	0.473967	0.887457
pyruvate_+_TPP_=>_2-(alpha-hydroxyethyl)-TPP_+_CO2	0.668816	0.473967	0.887457
S-acetyldihydrolipoamide_+_CoA_=>_acetyl-CoA_+_dihydrolipoamide	0.668816	0.473967	0.887457
G-protein_alpha_subunit_is_inactivated	0.398095	0.106458	0.905449
Removal_of_remaining_Flap_from_the_C-strand	0.0397446	0.0140753	0.729934
A_ligand:p75NTR_complex_binds_to_Nrage	0.378137	0.112076	0.684417
Loading_of_PCNA_-_Sliding_Clamp_Formation_on_the_C-strand_of_the_tel	0.364974	0.140222	0.722865
RFC_dissociates_after_sliding_clamp_formation_on_the_C-strand_of_the_t	0.364974	0.140222	0.722865
Phosphorylation_of_DLC1_by_MAPK_8	0.0827933	0.0832132	0.328751
Arf1_Activation_by_GBF1	0.599001	0.862735	0.0339061
trans-Golgi_Network_Coat_Activation	0.599001	0.862735	0.0339061
Cleavage_of__flap_structures	0.0824732	0.0247573	0.645478
Opioid_receptors_bind_opioid_peptides	0.0350169	0.318593	0.0297643

Inactivation_of_Myt1_kinase	0.82986	0.878714	0.570991
Inactivation_of_Wee1_kinase	0.82986	0.878714	0.570991
Activation_of_SRC_by_Ral-GTP	0.960578	0.518116	0.992616
Phosphorylation_of_DLC2_by_MAPK-8	0.0735908	0.114663	0.198634
Binding_of_TFIIIC_to_TFIIIA:Type_I_Promoter_complex_	0.52577	0.696314	0.336289
Beta-tubulin:GTP_+_Cofactor_D_->__Beta-tubulin:GTP:_Cofactor_D	0.692198	0.135857	0.992276
Docking_of_Mature_Replication_Dependent_Histone_mRNA_with_the_NPC	0.59963	0.185493	0.826887
Inhibitory_effect_of_Grb10_on_insulin_signalling	0.836441	0.798189	0.696695
phosphatidylinositol_+_UDP-N-acetyl-D-glucosamine_->_N-acetylglucosamir	0.950005	0.388437	0.986591
Beta-tubulin:GTP_+_Cofactor_A_->__Beta-tubulin:GTP:_Cofactor_A	0.266895	0.135857	0.683183
Somatostatin_receptors_bind_somatostatin	0.93585	0.87865	0.836454
Assembly_of_RNA_Polymerase_I_Holoenzyme_(mouse)	0.943453	0.333205	0.996208
cholesterol_ester_+_H2O_->_cholesterol_+_fatty_acid	0.602219	0.332084	0.826553
diacylglycerol_+_H2O_->_2-acylglycerol_+_fatty_acid	0.602219	0.332084	0.826553
phosphorylated_HSL_dimer_+_FABP4_->_phosphorylated_HSL_dimer:FABP4	0.602219	0.332084	0.826553
triacylglycerol_+_H2O_->_diacylglycerol_+_fatty_acid	0.602219	0.332084	0.826553
Factor_D_cleaves_C3(H2O)-bound_Factor_B	0.165387	0.214713	0.28466
Factor_D_cleaves_C3b-bound_Factor_B	0.165387	0.214713	0.28466
CYP2C18_initiates_bioactivation_of_phenytoin_by_4-hydroxylation	0.968804	0.552029	0.978172
CYP2C19_5-hydroxylates_omeprazole	0.968804	0.552029	0.978172
CYP2C8_inactivates_paclitaxel_by_6alpha-hydroxylation	0.968804	0.552029	0.978172
CYP2C9_inactivates_tolbutamide_by_4methyl-hydroxylation	0.968804	0.552029	0.978172
Formation_of_alternate_C5_convertase	0.573604	0.759325	0.28466
Properdin_stabilizes_C3b:Bb_bound_to_cell_surfaces	0.573604	0.759325	0.28466
ApoB-48_+_40_triacylglycerol_+_60_phospholipid_=>_ApoB-48:TG:PL_comp	0.497367	0.076147	0.760941
factor_XII_->_factor_XIIa	0.509538	0.647937	0.334588
cytosolic_GK:GKRP_complex_<=>_glucokinase_(GK)_+_glucokinase_regulato	0.139504	0.30677	0.079548
GK:GKRP_[cytosol]_=>_GK:GKRP_[nucleoplasm]	0.139504	0.30677	0.079548
glucokinase_(GK)_+_glucokinase_regulatory_protein_(GKRP)_<=>_GK:GKRP_	0.139504	0.30677	0.079548
nucleoplasmic_GK:GKRP_complex_=>_glucokinase_(GK)_+_glucokinase_regu	0.139504	0.30677	0.079548
hormone-sensitive_lipase_(HSL)_+_2_ATP_->_phosphorylated_HSL_+_2_AD	0.813377	0.573385	0.880973
Calmodulin_activates_Cam-PDE_1	0.537862	0.409261	0.587473
cAMP_hydrolysis_by_Cam-PDE_1	0.537862	0.409261	0.587473
cAMP_hydrolysis_by_PDE_4	0.537862	0.409261	0.587473
Type_II_receptor_recruits_type_I_receptor	0.734518	0.681909	0.659251
PKC_phosphorylates_GRK2	0.580651	0.312656	0.745831
Grb7_binds_to_the_active_PDGF_receptor	0.105625	0.260602	0.117114
chylomicron_remnant:apoE_complex_+_LDLR_=>_chylomicron_remnant:ap	0.997522	0.975592	0.984385
chylomicron_remnant:apoE:LDLR_complex_=>_chylomicron_remnant:apoE_	0.997522	0.975592	0.984385
chylomicron_remnant:apoE:LDLR_complex_[coated_vesicle_membrane]_=>	0.997522	0.975592	0.984385
chylomicron_remnant:apoE:LDLR_complex_[plasma_membrane]_=>_chylon	0.997522	0.975592	0.984385
eNOS:Caveolin-1_complex_binds_to_Nostrin	0.336718	0.124298	0.772351
eNOS:Caveolin-1:NOSTRIN_complex_binds_dynamin-2	0.336718	0.124298	0.772351
Exocytosis_of_thrombospondin	0.824764	0.824764	#N/A
PLC-gamma_binds_to_the_active_receptor	0.194503	0.444356	0.117114
PLC-gamma_hydrolyses_PIP2	0.194503	0.444356	0.117114
GAP_binds_to_PDGF-beta_receptors_only	0.10062	0.230817	0.147361

MAP_kinase_activates_MAPKAPK2,_MAPKAPK3_and_MSK1	0.459942	0.333149	0.6439
Cleavage_of_PAK-2_at_212	0.0303588	0.137643	0.0631369
Proteolysis_of_the_IGF:IGFBP-3:ALS_Complex_by_Thrombin	0.00731535	0.0639141	0.00799824
Interaction_of_Tie2_and_Shp2	0.497245	0.120926	0.922449
p38MAPK_phosphorylates_MSK1	0.523994	0.310474	0.75813
FMO1_N-oxidizes_the_anti-cancer_drug_tamoxifen	0.878625	0.698079	0.852173
FMO2_S-oxidizes_the_antithyroid_drug_methimazole	0.878625	0.698079	0.852173
FMO3_N-oxidizes_the_tertiary_amine_trimethylamine	0.878625	0.698079	0.852173
Nicotine_N-oxidised_to_Nicotine-N-oxide	0.878625	0.698079	0.852173
LCAT_+_discoidal_HDL_<=>_LCAT:discoidal_HDL_complex	0.193549	0.0756493	0.825072
LCAT_+_spherical_HDL_<=>_LCAT:spherical_HDL_complex	0.193549	0.0756493	0.825072
LCAT:discoidal_HDL_complex_<=>_LCAT_+_discoidal_HDL	0.193549	0.0756493	0.825072
LCAT:spherical_HDL_complex_<=>_LCAT_+_spherical_HDL	0.193549	0.0756493	0.825072
Phosphorylation_of_Platelet_Sec-1	0.56839	#N/A	0.56839
Proteolysis_of_the_IGF:IGFBP-3:ALS_Complex_by_Plasmin	0.0449209	0.245158	0.00799824
ERK5_activates_the_transcription_factor_MEF2	0.364585	0.0999242	0.725857
Proteolysis_of_the_IGF:IGFBP-5:ALS_Complex_by_PAPP-A2	0.259814	0.228912	0.411182
5,6-dihydrothymine_+_H2O_=>_beta-ureidoisobutyrate	0.619028	0.585695	0.59627
5,6-dihydrouracil_+_H2O_=>_beta-ureidopropionate	0.619028	0.585695	0.59627
ERK1/2_phosphorylates_MSK1	0.340624	0.0632178	0.889146
TBK1_is_Recruited_to_the_Viral_dsRNA:TLR3:TRIF_Complex	0.670171	#N/A	0.670171
VEGF-C,D_bind_to_VEGFR3_leading_to_receptor_dimerization	0.0812457	0.144937	0.151191
MERTK_receptor_binds_ligands_(Gas6_or_Protein_S)	0.542619	0.117188	0.762699
AMP_binds_to_gamma_subunit_of_AMP_kinase_heterotrimer	0.188491	0.365064	0.195369
adenosine_5-diphosphate_+_thioredoxin_(reduced)_=>_2-deoxyadenosine_	0.913314	0.555941	0.949667
cytidine_5-diphosphate_+_thioredoxin_(reduced)_=>_2-deoxycytidine_5-di	0.913314	0.555941	0.949667
guanosine_5-diphosphate_+_thioredoxin_(reduced)_=>_2-deoxyguanosine_	0.913314	0.555941	0.949667
uridine_5-diphosphate_+_thioredoxin_(reduced)_=>_2-deoxyuridine_5-diph	0.913314	0.555941	0.949667
Activation_of_Talin	0.490012	0.210423	0.939606
Phosphorylation_and_Release_of_IRF3	0.503939	#N/A	0.503939
Viral_dsRNA:TLR3:TRIF:TBK1_complex_recruits_IRF3	0.503939	#N/A	0.503939
Autocatalytic_phosphorylation_of_FGFR1b	0.946975	#N/A	0.946975
FGFR1b_binds_to_FGF	0.946975	#N/A	0.946975
Receptors_CCR3,_4_and_5_bind_CCL5_ligand	0.195104	0.360845	0.206728
Activation_of_PDC_by_dephosphorylation_of_phospho-E1_alpha_compone	0.676097	0.461392	0.887457
pro-prothrombin_->_prothrombin_+_prothrombin_propeptide	0.596688	0.362664	0.792932
eIF4F_binds_to_mRNP	0.372132	0.181896	0.724531
Formation_of_the_cap-binding_eIF4F_complex	0.372132	0.181896	0.724531
Caspase-mediated_cleavage_of_MASK	0.0627554	0.0156723	0.810278
STAT3_activation	0.595413	0.770058	0.460085
TNF_Mediated_Activation_of_Pro-caspase_8	0.014208	0.0110591	0.168888
TRADD:TRAF2:RIP1:FADD_complex_binds_Pro-Caspase_8__	0.014208	0.0110591	0.168888
EGFR_autophosphorylation	0.455341	0.376786	0.646675
EGFR_binds_EGF_ligand	0.455341	0.376786	0.646675
EGFR_dimerization	0.455341	0.376786	0.646675
pro-factor_IX_->_factor_IX_+_factor_IX_propeptide	0.818679	0.495222	0.881847
NF-kB_migrates_to_the_nucleus_and_turns_on_transcription	0.952661	0.657686	0.952001

Formation_of_Cyclin_D:Cdk4/6_complexes_	0.48098	0.48098	#N/A
Translocation_of_Cyclin_D:Cdk4/6_complexes_from_the_cytoplasm_to_the	0.48098	0.48098	#N/A
pro-factor_X_->_factor_X_+_factor_X_propeptide	0.800959	0.663917	0.792932
SKI_complexes_with_the_Smad_complex,_suppressing_BMP2_signalling	0.483231	0.282872	0.660845
uPAR_precursor_+_acyl-GPI_->_uPAR-acyl-GPI_+_uPAR_propeptide	0.0115528	0.00923084	0.357325
Basigin_binds_MCT1,_MCT2,_MCT3_or_MCT4	0.156398	0.0862274	0.530181
lactate_+_H+_[cytosol]_<=>_lactate_+_H+_[extracellular]	0.156398	0.0862274	0.530181
lactate_+_H+_[extracellular]_<=>_lactate_+_H+_[cytosol]	0.156398	0.0862274	0.530181
depalmitoylation_of_eNOS	0.386079	0.126951	0.948656
Regeneration_of_eEF1A:GTP_by_eEF1B_activity	0.19727	0.0165279	0.896933
Binding_of_PI3K_regulatory_alpha_subunit_to_Gab1:Grb2	0.986451	0.146848	0.998633
pro-protein_Z_->_protein_Z_+_protein_Z_propeptide	0.893377	0.82609	0.792932
N-hydroxy-2-acetylaminofluorene_+_PAPS_=>_2-acetylaminofluorene-N-sul	0.863952	0.72693	0.840317
Interaction_of_Tie2_and_Shc1	0.30597	0.209176	0.685656
Crks_SH3_domain_engages_C3G	0.525129	0.478309	0.565927
pro-protein_C_->_protein_C_+_protein_C_propeptide	0.644225	0.424627	0.792932
CDK5_phosphorylates_DARPP-32_on_Thr75	0.830394	0.933613	0.370399
Formation_of_a_heteromeric_BMP_receptor_complex	0.278628	0.609069	0.162656
adenosine_5-diphosphate_+_glutaredoxin_(reduced)_=>_2-deoxyadenosine	0.465855	0.706645	0.356407
cytidine_5-diphosphate_+_glutaredoxin_(reduced)_=>_2-deoxycytidine_5-di	0.465855	0.706645	0.356407
guanosine_5-diphosphate_+_glutaredoxin_(reduced)_=>_2-deoxyguanosine	0.465855	0.706645	0.356407
uridine_5-diphosphate_+_glutaredoxin_(reduced)_=>_2-deoxyuridine_5-dip	0.465855	0.706645	0.356407
Interaction_of_integrin_alpha_V_beta_8_with_vitronectin	0.839392	0.842259	0.632283
Phospho-Frs2:CrkL_engages_C3G	0.39666	0.386546	0.496327
Endothelin_receptors_bind_endothelin	0.522093	#N/A	0.522093
NRP-2_associates_with_VEGFR1_forming_complexes_on__cell_surface	0.518175	0.381769	0.619597
Latent_TGF-beta1_is_cleaved_by_furin	0.797713	0.658453	0.792932
Phosphorylation_of_PF2K-Pase_by_PKA_catalytic_subunit	0.911532	0.815877	0.843063
Antizyme_inhibitor_binds_to_OAZ_and_stablizes_ODC_complex	0.00407206	0.00174314	0.151961
Antizyme_OAZ_binds_to_Ornithine_decarboxylase	0.00407206	0.00174314	0.151961
pro-factor_VII_->_factor_VII_+_factor_VII_propeptide	0.721353	0.535873	0.792932
Exocytosis_of_TGF_beta	0.755071	0.664141	0.716274
MEK1_binds_ERK-1	0.175901	0.0734628	0.793339
MEK1_phosphorylates_ERK-1	0.175901	0.0734628	0.793339
MEK2_binds_ERK-2	0.175901	0.0734628	0.793339
MEK2_phosphorylates_ERK-2	0.175901	0.0734628	0.793339
TNF:TNF-R1_binds_TRADD,_TRAF2_and_RIP_Complex	0.367856	0.404806	0.392622
TRADD:TRAF2:RIP1_complex_dissociates_from_the_TNF-alpha:TNF-R1_com	0.367856	0.404806	0.392622
pro-GAS6_->_GAS6_+_GAS6_propeptide	0.490765	0.242883	0.792932
Orc1_associates_with_Orc4:Orc5:Orc3:Orc2:origin_complexes	0.000633142	#N/A	0.000633142
Translocation_of_TRAF6_to_CBM_complex	0.799446	0.677942	0.829344
pro-protein_S_->_protein_S_+_protein_S_propeptide	0.773676	0.495222	0.836132
ARMS_is_phosphorylated_by_active_TrkA_receptor	0.576711	0.545352	0.565927
Disassociation_of_Processive_Complex_and_Completed_Telomere_End	0.0192841	0.00440622	0.729934
Formation_of_C-strand_Okazaki_fragments	0.0192841	0.00440622	0.729934
Formation_of_Processive_Complex_on_the_C-strand_of_the_telomere	0.0192841	0.00440622	0.729934
Formation_of_the_Flap_Intermediate_on_the_C-strand	0.0192841	0.00440622	0.729934

IRAK_interacts_with_TRAF6	0.803518	0.839339	0.614862
MYD88_dissociates	0.803518	0.839339	0.614862
Interaction_of_SOS-1_to_Tie2_bound__Grb2	0.0757566	0.0222875	0.576676
Cdc6_protein_is_phosphorylated_by_CDK	0.755409	0.933613	0.227234
Megalin_internalizes_the_cubilin-DBP:Calcidiol_complex	0.691984	0.328609	0.783086
Luteinizing_hormone_receptor_can_bind_LH	0.301322	0.319546	0.401924
Thyroid-stimulating_hormone_receptor_can_bind_TSH	0.410113	0.463959	0.401924
c-src_associates_with_Cx43_in_gap_junctions	0.959346	0.649194	0.960498
Closure_of_gap_junction	0.959346	0.649194	0.960498
Phosphorylation_of_Cx43_by_c-src	0.959346	0.649194	0.960498
Receptor_CXCR2_binds_ligands_CXCL1_to_7	0.542769	0.467961	0.563651
Part_of_pro-beta-NGF_is_processed_to_mature_beta-NGF	0.693377	0.495222	0.745962
DNA_strand_displacement_synthesis	0.0475615	0.00697826	0.645478
Activation_of_the_Anaphase_Promoting_Complex_(APC)_by_Plk1	0.665837	0.460053	0.737166
Disassociation_of_PLC-gamma1_from_SLP-76	0.45221	0.370832	0.651772
Recruitment_of_PLC-gamma1_to_SLP-76	0.45221	0.370832	0.651772
Association_of__the_XRCC4:DNA_ligase_IV_complex_with_the_DNA-PK:DN/	0.305133	#N/A	0.305133
Removal_of_repair_proteins_and_ligation_of_the_processed_ends_of_the_	0.305133	#N/A	0.305133
Follicle-stimulating_hormone_receptor_can_bind_FSH	0.51256	0.596149	0.401924
Release_of_the_Mature_intronless_transcript_derived_Histone_mRNA:SLBI	0.337152	0.185493	0.574723
JNK_phosphorylates_BIM,_BAD_and_other_targets	0.282671	0.352055	0.328751
p62_is_recruited_and_forms_a_complex_with_TRAF6	0.71775	0.69625	0.610706
I-Smad_competes_with_Co-Smad_for_R-Smad1/5/8	0.218048	0.31409	0.261911
Binding_of_pro-NGF_to_p75NTR:sortilin	0.0938911	0.293139	0.115098
Binding_of_TFIIIC_to_Type_2_promoter	0.543434	0.696314	0.323883
Docking_of_Mature_Histone_mRNA_complex:TAP_at_the_NPC	0.743771	0.337566	0.826887
5-HT1_and_5A_receptors_can_bind_serotonin	0.598836	0.728467	0.430969
Proteinase-activated_receptors_can_bind_thrombin	0.344422	0.114596	0.679188
Autophosphorylation_of_insulin_receptor	0.891964	0.880085	0.696695
Dissociation_of_insulin_from_insulin_receptor	0.891964	0.880085	0.696695
Insulin_binding	0.891964	0.880085	0.696695
Internalisation_of_the_insulin_receptor	0.891964	0.880085	0.696695
thioredoxin,_oxidized_+_NADPH_+_H+_=>_thioredoxin,_reduced_+_NADP+	0.890823	0.579783	0.95522
fibrinogen_->_fibrin_monomer_+_2_fibrinopeptide_A_+_2_fibrinopeptide_B	0.880285	0.880285	#N/A
Spherical_HDL_binds_C_and_E_apolipoproteins	0.83777	0.585425	0.944562
glycogen-glycogenin-1_+_n_orthophosphate_=>_limit_dextrin-glycogenin-1_	0.595989	0.595989	#N/A
glycogen-glycogenin-1_+_n_orthophosphate_=>_limit_dextrin-glycogenin-1_	0.595989	0.595989	#N/A
glycogen-glycogenin-2_+_n_orthophosphate_=>_limit_dextrin-glycogenin-2_	0.595989	0.595989	#N/A
glycogen-glycogenin-2_+_n_orthophosphate_=>_limit_dextrin-glycogenin-2_	0.595989	0.595989	#N/A
poly{(1,4)-alpha-glucosyl}_glycogenin-1_+_n_orthophosphate_=>_glycogeni	0.595989	0.595989	#N/A
poly{(1,4)-alpha-glucosyl}_glycogenin-1_+_n_orthophosphate_=>_glycogeni	0.595989	0.595989	#N/A
poly{(1,4)-alpha-glucosyl}_glycogenin-2_+_n_orthophosphate_=>_glycogeni	0.595989	0.595989	#N/A
poly{(1,4)-alpha-glucosyl}_glycogenin-2_+_n_orthophosphate_=>_glycogeni	0.595989	0.595989	#N/A
ApoB-48:TG:PL_complex_+_100_triacylglycerols_+_ApoA-I_+_ApoA-IV_=>_r	0.721158	0.37264	0.875597
nascent_chylomicron_[endoplasmic_reticulum_lumen]_=>_nascent_chylom	0.721158	0.37264	0.875597
plasminogen:histidine-rich_glycoprotein_->_plasmin_+_histidine-rich_glycop	0.457507	0.388408	0.609158
plasminogen:histidine-rich_glycoprotein_->_plasmin_+_histidine-rich_glycop	0.457507	0.388408	0.609158

C3(H2O):Factor_Bb-mediated_C3_cleavage_leads_to_C3b_deposition_on_a	0.501561	0.717168	0.28466
Factor_B_binds_to_Complement_factor_3(H2O)_(C3(H2O))	0.501561	0.717168	0.28466
Factor_B_binds_to_surface-associated_C3b	0.501561	0.717168	0.28466
Exocytosis_of_alpha_2_macroglobulin	0.617245	0.605457	0.575177
C5a_receptor_binds_C5a_anaphylatoxin	0.449972	0.731792	0.307625
Exocytosis_of_Alpha_Actinins	0.1453	0.1453	#N/A
5-hydroxyindole_acetaldehyde_to_5-hydroxyindole_acetic_acid	0.0121894	0.0275031	0.0681752
Acetaldehyde_+_NAD+_<=>_Acetate_+_NADH_+_H+_[cytosolic]	0.0121894	0.0275031	0.0681752
Acetaldehyde_+_NAD+_<=>_Acetate_+_NADH_+_H+_[mitochondrial]	0.0121894	0.0275031	0.0681752
Conversion_of_C2_into_C2a_and_C2b	0.950289	0.891647	0.875575
Formation_of_C5b:C6_complex	0.668514	0.881952	0.28466
Extracellular_processing_of_novel_PDGFs	0.624451	0.583237	0.594033
Formation_of_C3_convertase_(C4b:C2a_complex)	0.605847	0.82853	0.28466
Formation_of_classic_C5_convertase	0.605847	0.82853	0.28466
kallikrein:kininogen:C1q_binding_protein_tetramer_->_kallikrein_+_activate	0.356637	0.463293	0.334588
prekallikrein_+_kininogen:C1q_binding_protein_tetramer_->_prekallikrein:k	0.356637	0.463293	0.334588
adenosine_5-diphosphate_(ADP)_+_ADP_<=>_adenosine_5-monophosphate	0.658188	0.658188	#N/A
adenosine_5-monophosphate_(AMP)_+_ATP_<=>_adenosine_5-diphosphate	0.658188	0.658188	#N/A
TFPI_+_TF:F7a_+_factor_Xa_->_TFPI:TF:F7a:factor_Xa	0.262974	0.742838	0.0607524
DAG_stimulates_protein_kinase_C-delta	0.693523	0.332642	0.797836
ATP_+_alpha-D-Glucose_=>_ADP_+_alpha-D-glucose_6-phosphate_[glucokir	0.30677	0.30677	#N/A
ATP_+_alpha-D-Glucose_=>_ADP_+_alpha-D-glucose_6-phosphate_[hexokin	0.30677	0.30677	#N/A
glucokinase_[nucleoplasm]_=>_glucokinase_[cytosol]	0.30677	0.30677	#N/A
PRDM4_inhibits_cyclin_E_transcription	0.240662	#N/A	0.240662
benzoate_+_Coenzyme_A_+_ATP_=>_benzoyl-CoA_+_AMP_+_pyrophospha	0.95644	0.783516	0.948806
phenylacetate_+_Coenzyme_A_+_ATP_=>_phenylacetyl-CoA_+_AMP_+_pyr	0.95644	0.783516	0.948806
salicylic_acid_+_Coenzyme_A_+_ATP_=>_salicylate-CoA_+_AMP_+_pyropho	0.95644	0.783516	0.948806
Formation_of_the_IGF:IGFBP-5:ALS_Complex	0.0760976	0.228912	0.100078
eNOS_associates_with_Caveolin-1	0.427702	0.124298	0.948656
Nucleotide_exchange_on_RHOA	0.539767	0.169645	0.863578
Exocytosis_of_VEGF	0.00823408	0.0751984	0.0255101
Autophosphorylation_of_PDGF_alpha_receptors	0.0865069	0.230817	0.117114
Autophosphorylation_of_PDGF_alpha/beta_receptors	0.0865069	0.230817	0.117114
Autophosphorylation_of_PDGF_beta_receptors	0.0865069	0.230817	0.117114
Caspase_3-mediated_cleavage_of_PKC_delta	0.30303	0.126905	0.827453
Caspase-mediated_cleavage_of_PKC_theta	0.30303	0.126905	0.827453
Translocation_of_PDGF_from_ER_to_Golgi	0.22028	0.0533336	0.516821
Mouse_pro-EGF_is_cleaved_by_ADAM_sheddases	0.203504	0.0868948	0.43209
phosphorylated_perilipin_+_H2O_->_perilipin_+_orthophosphate	0.111095	0.165475	0.225719
Caspase-mediated_cleavage_of_plectin-1	0.304495	0.188693	0.46799
Transport_(efflux)_of_bile_salts_by_ABCB11_(bile_salt_export_pump)	0.903215	0.649839	0.899408
NQO1_interaction_with_ODC	0.0315721	0.00878724	0.219888
Phosphorylation_of_CARMA1	0.817086	0.474588	0.910131
RSK1/2/3_phosphorylates_CREB_at_Serine_133	0.0885002	0.0758268	0.317655
Transport_(influx)_of_bile_salts_and_acids_by_OATP-A	0.904711	#N/A	0.904711
thymidine_5-monophosphate_(TMP)_+_H2O_=>_thymidine_+_orthophosph	0.0605728	0.0551549	0.276349
uridine_5-monophosphate_(UMP)_+_H2O_=>_uridine_+_orthophosphate	0.0605728	0.0551549	0.276349

LDL_+_LDLR_=>_LDL:LDLR_complex	0.990419	0.975592	0.941207
LDL:LDLR_complex_[plasma_membrane]_=>_LDL:LDLR_complex_[clathrin-co	0.990419	0.975592	0.941207
LDLR:LDL_complex_=>_LDLR_+_LDL	0.990419	0.975592	0.941207
LDLR:LDL_complex_[coated_vesicle_membrane]_=>_LDLR:LDL_complex_[er	0.990419	0.975592	0.941207
Conversion_of_pro-apoA-I_to_apoA-I	0.776607	0.65479	0.750702
Interaction_of_Tie2_and_Dok-2	0.144507	0.066348	0.685656
Interaction_of_Tie2_and_Grb7	0.156989	0.0746602	0.685656
P-selectin_binds_P-selectin_ligand	0.325326	0.447636	0.327524
SLC7A10-mediated_uptake_of_small_neutral_amino_acids	0.898595	0.973363	0.447087
SLC7A5-mediated_uptake_of_neutral_amino_acids	0.898595	0.973363	0.447087
SLC7A6_(y+LAT2)-mediated_exchange_of_extracellular_leucine_for_cytosoli	0.898595	0.973363	0.447087
SLC7A8-mediated_uptake_of_neutral_amino_acids	0.898595	0.973363	0.447087
Caspase-mediated_cleavage_of_FADK_1	0.314046	0.137643	0.657731
PLC_beta_1/4-mediated_hydrolysis	0.911293	0.84336	0.815282
guanosine_5-monophosphate_(GMP)_+_H2O_=>_guanosine_+_orthophosp	0.214289	0.0551549	0.683662
inosine_5-monophosphate_(IMP)_+_H2O_=>_inosine_+_orthophosphate	0.214289	0.0551549	0.683662
Formation_of_the_MCC_complex	0.14505	0.727342	0.0178678
2-deoxyadenosine_+_ATP_=>_2-deoxyadenosine_5-monophosphate_+_ADP	0.118984	0.180844	0.224934
SKI_complexes_with_the_SMAD_complex,_suppressing_TGF-beta_signaling	0.568534	0.124737	0.918563
ABCD1/ABCD3_mediates_long_chain_fatty_acid_transport_in_to_peroxison	0.0797813	0.159319	0.139223
Peroxisomal_uptake_of_very_long-chain_fatty_acyl_CoA	0.0797813	0.159319	0.139223
mannose_(a1-2)_mannose_(a1-6)_(ethanolamineP)_mannose_(a1-4)_glucos	0.483395	0.483395	#N/A
FAS_Mediated_Activation_of_Pro-caspase_8	0.106349	0.880197	0.0170721
FASL:FAS_Receptor_Trimer:FADD_complex_binds_pro-Caspase-8	0.106349	0.880197	0.0170721
Cleavage_of_Procaspase-9_to_Caspase-9	0.609755	0.651113	0.454046
Cytochrome_C:Apaf-1_binds_Procaspase-9	0.609755	0.651113	0.454046
TrkA_phosphorylates_PLCG1	0.699866	0.729109	0.496327
17-Hydroxypregnenolone_is_dehydrogenated_to_form_pregn-5-ene-3,20-d	0.599284	0.939397	0.2724
DHA_isomerizes_to_4-Androstene3,17-dione	0.599284	0.939397	0.2724
Pregn-5-ene-3,20-dione_isomerizes_to_progesterone	0.599284	0.939397	0.2724
Pregn-5-ene-3,20-dione-17-ol_isomerizes_to_17-hydroxyprogesterone	0.599284	0.939397	0.2724
Pregnenolone_is_dehydrogenated_to_form_pregn-5-ene-3,20-dione	0.599284	0.939397	0.2724
Caspase-mediated_cleavage_of_Rock-1	0.132626	0.137643	0.314363
Caspase-mediated_cleavage_of_Lamin_A	0.523238	0.279148	0.870538
Caspase-mediated_cleavage_of_Lamin_B1	0.523238	0.279148	0.870538
Regulation_of_MKLP-1_by_phosphorylation	0.715629	0.910759	0.452271
Association_of_Cks1_with_SCF(Skp2)_complex	0.0331803	0.0135762	0.349983
Angiotensin_II_binds_to_angiotensin_II_receptor_(types_1_and_2)	0.806369	0.802324	0.693801
Proteolysis_of_the_IGF:IGFBP-4_Complex_by_PAPP-A	0.1127	0.155082	0.242455
Interaction_of_Tie2_and_Grb2	0.0670873	0.0222875	0.685656
PKA_phosphorylates_CREB	0.90043	0.681873	0.886614
Formation_of_Cyclin_A:Cdc2_complexes	0.860967	0.933613	0.331893
Formation_of_Cyclin_A:Cdk2_complexes	0.860967	0.933613	0.331893
Translocation_of_Cyclin_A:Cdk2_complexes_to_the_nucleus	0.860967	0.933613	0.331893
Translocation_of_Cyclin_A:phospho-Cdc2_(Thr_14)_to_the_nucleus	0.860967	0.933613	0.331893
palmitoylation_of_eNOS	0.452642	0.348483	0.587507
eNOS_binds_NOSIP	0.767707	0.460482	0.948656

Phosphorylation_of_UBF-1:rDNA_Promoter	0.354602	0.183145	0.793339
Regulation_of_MKLP-2_by_phosphorylation	0.947772	0.910759	0.863556
Receptor_CXCR3_binds_ligands_CXCL9_to_11	0.966406	0.91247	0.904758
Basigin_binds_CyPA	0.270547	0.270547	#N/A
Formation_of_the_IGF:IGFBP-3:ALS_Complex	0.00748655	0.0783593	0.00799824
Phosphorylated_MAPK1_phosphorylates_ATF-2	0.200541	0.0999242	0.537931
cholesterol_+_phosphatidylcholine_(lecithin)_=>_cholesterol_ester_+_2-lyso	0.393913	0.197343	0.825072
Caspase_3-mediated_cleavage_of_DFF45_(117)	0.0699757	0.0424055	0.51349
Cleavage_of_DFF45_(224)_by_caspase-3	0.0699757	0.0424055	0.51349
Interaction_and_oligomerization_of_MALT1_to_Bcl10	0.630909	0.434757	0.829344
Phospho-Shc_dissociates_from_the_TrkA_receptor	0.779523	0.814539	0.496327
Shc_binds_to_the_activated_TrkA_receptor	0.779523	0.814539	0.496327
Shc,_complexed_with_TrkA,_is_tyrosine-phosphorylated	0.779523	0.814539	0.496327
Sequestration_of_BAD_protein_by_14-3-3	0.151915	0.268051	0.203432
DARPP-32_phosphorylated_on_T34_binds_to_PP1,_inhibiting_its_function	0.116003	0.0984987	0.263028
DARPP-32_phosphorylated_on_Thr75_binds_to_PKA,_inhibiting_its_functio	0.854381	0.681873	0.827629
PKA_phosphorylates_DARPP-32_on_Thr34	0.854381	0.681873	0.827629
Detection_of_damage_during_initiation_of__DNA_synthesis_in_S-phase	0.198359	0.620895	0.0660819
The_polymerase_component_of_DNA_polymerase_alpha:primase_synthesi	0.198359	0.620895	0.0660819
The_polymerase_component_of_DNA_polymerase_alpha:primase_synthesi	0.198359	0.620895	0.0660819
Caspase_mediated_cleavage_of_beta-catenin	0.0658591	0.0658591	#N/A
Dissociation_of_Caspase-3_from_SMAC:XIAP:Caspase-3	0.17224	0.17224	#N/A
Dissociation_of_Caspase-7_from_SMAC:XIAP:Caspase-7	0.17224	0.17224	#N/A
SMAC_binds_XIAP:Caspase-3	0.17224	0.17224	#N/A
SMAC_binds_XIAP:Caspase-7	0.17224	0.17224	#N/A
Phosphorylation_of_Bcl10	0.305013	0.394768	0.324918
Association_of_DFF40_with_chromatin	0.188757	#N/A	0.188757
Cleavage_of_DNA_by_DFF40	0.188757	#N/A	0.188757
Orc4_associates_with_Orc5:Orc3:Orc2:origin_complexes	0.000510004	#N/A	0.000510004
IRAK_is_activated	0.637054	0.577224	0.614862
p75NTR_interacts_with_IRAK:MYD88	0.637054	0.577224	0.614862
TrkA_recruits_RIT_and_RIN	0.661615	0.686674	0.496327
Vamp2_associated_secretory_vesicle_to_plasma_membrane_transport	0.119038	0.17948	0.220867
Phospho-R-Smad1/5/8_forms_a_complex_with_Co-Smad	0.591004	0.398145	0.660845
The_phospho-R-Smad1/5/8:Co-Smad_transfers_to_the_nucleus	0.591004	0.398145	0.660845
Succinate_<=>_Fumarate_(with_FAD_redox_reaction_on_enzyme)	0.0107112	0.0107112	#N/A
Transfer_of_electrons_through_the_succinate_dehydrogenase_complex	0.0107112	0.0107112	#N/A
Down_Regulation_of_Emi1_through_Phosphorylation_of_Emi1	0.875145	0.918333	0.591947
Caspase_mediated_cleavage_of_C-IAP1	0.220226	0.137643	0.50027
NRIF_and_TRAF6_may_activate_JNK	0.569501	0.886202	0.31016
ARMS:Crk_complex_binds_to_active_TrkA_receptor	0.676336	0.68451	0.565927
ERK5_is_activated	0.445895	0.264349	0.793339
TRADD:TRAF2:RIP1_complex_binds_FADD	0.0291064	0.0110591	0.348831
RIP1_phosphorylates_IKKs	0.965472	0.682962	0.965881
Phospho-Frs2_binds_CrkL	0.537511	0.545352	0.496327
Regulation_of_NudC_by_phosphorylation	0.673555	0.656484	0.591947
Dehydration_of_5-HpETE_to_leukotriene_A4	0.245854	0.232557	0.456354

Oxidation_of_arachidonic_acid_to_5-HpETE	0.245854	0.232557	0.456354
propionyl-CoA_+_CO2_+_ATP_<=>_D-methylmalonyl-CoA_+_ADP_+_orthop	0.620312	0.620312	#N/A
Association_of_RAD52_with_the_RPA_complex	0.0325724	0.220588	0.00936727
Formation_of_RAD52_heptameric_ring_structure_complexes_on_ssDNA	0.0325724	0.220588	0.00936727
beta-methylcrotonyl-CoA_+_ATP_+_CO2_<=>_beta-methylglutaconyl-CoA_+	0.525319	0.525319	#N/A
Interaction_of_Tie2_and_Grb14	0.227054	0.126366	0.685656
Recruitment_of_SLP-76_to_Gads	0.296693	0.199912	0.651772
Bombesin-like_receptors_bind_bombesin_homologues	0.448761	#N/A	0.448761
Release_of_the_Mature_intronless_derived_mRNA,_TAP,_and_Aly/Ref_fror	0.48143	0.337566	0.574723
Release_of_the_SLBP_independent_Histone_mRNA_from_the_NPC	0.48143	0.337566	0.574723
Oxidation_of_Cysteine_to_Cystine_in_Proinsulin	0.827584	0.464771	0.922669
SLC6A14-mediated_uptake_of_basic_and_neutral_amino_acids_and_of_bet	0.625989	0.777569	0.378218
Binding_of_ATR-ATRIP_to_the_RPA-ssDNA_complex	0.214213	0.214213	#N/A
pro-NGF_dimerizes	0.270593	0.0381477	0.625205
Formation_of_NELF_complex_	0.686548	0.686548	#N/A
p75NTR:NgR_complex_interacts_with_the_axonal_inhibitor_LINGO1	0.957918	0.960675	0.657777
Association_of_RAD51_with_the_RPA_complex	0.223166	0.220588	0.425233
2-methyl-1-hydroxybutyl-TPP_+_lipoamide_=>_S-(2-methylbutanoyl)-dihydr	0.434085	0.14813	0.929941
2-methyl-1-hydroxypropyl-TPP_+_lipoamide_=>_S-(isobutyryl)-dihydrolipoar	0.434085	0.14813	0.929941
3-methyl-1-hydroxybutyl-TPP_+_lipoamide_=>_S-isovaleryldihydrolipoamide	0.434085	0.14813	0.929941
alpha-keto-beta-methylvalerate_+_TPP_=>_2-methyl-1-hydroxybutyl-TPP_+_	0.434085	0.14813	0.929941
alpha-ketoisocaproate_+_TPP_=>_3-methyl-1-hydroxybutyl-TPP_+_CO2	0.434085	0.14813	0.929941
alpha-ketoisovalerate_+_TPP_=>_2-methyl-1-hydroxypropyl-TPP_+_CO2	0.434085	0.14813	0.929941
dihydrolipoamide_+_FAD_=>_lipoamide_+_FADH2_[branched-chain_ketoac	0.434085	0.14813	0.929941
FADH2_+_NAD+_=>_FAD_+_NADH_+_H+_[branched-chain_ketoacid_dehydı	0.434085	0.14813	0.929941
S-(2-methylbutanoyl)-dihydrolipoamide_+_CoA_=>_alpha-methylbutyryl-Co/	0.434085	0.14813	0.929941
S-(isobutyryl)-dihydrolipoamide_+_CoA_=>_isobutyryl-CoA_+_dihydrolipoan	0.434085	0.14813	0.929941
S-isovaleryldihydrolipoamide_+_CoA_=>_isovaleryl-CoA_+_dihydrolipoamide	0.434085	0.14813	0.929941
GRB2:SOS_binds_to_SHC-P	0.41059	0.444571	0.416676
GRB2:SOS_binds_IRS-P	0.0578912	0.0451376	0.416676
Surface_deployment_of_GP_Ib-IX-V_complex	0.931682	0.868603	0.837313
Transport_of_the_Mature_IntronlessTranscript_Derived_Histone_mRNA:SLE	0.326396	0.185493	0.602077
Phosphorylation_of_Wee1_kinase_by_Chk1	0.780973	0.817595	0.492302
Receptor_CCR7_binds_CCL19_and_21_ligands	0.979159	0.666328	0.992822
Galanin_receptors_can_bind_galanin	0.893443	0.668552	0.882021
2-deoxyadenosine_5-diphosphate_(dADP)_+_ATP_<=>_dATP_+_adenosine_	0.00631355	0.00631355	#N/A
2-deoxycytidine_5-diphosphate_(dCDP)_+_ATP_<=>_dCTP_+_adenosine_5-c	0.00631355	0.00631355	#N/A
2-deoxyguanosine_5-diphosphate_(dGDP)_+_ATP_<=>_dGTP_+_adenosine_	0.00631355	0.00631355	#N/A
2-deoxyuridine_5-diphosphate_(dUDP)_+_ATP_<=>_deoxyuridine_5-triphos	0.00631355	0.00631355	#N/A
2-deoxyuridine_5-diphosphate_(dUDP)_+_ATP_<=>_dUTP_+_adenosine_5-d	0.00631355	0.00631355	#N/A
2-deoxyuridine_5-triphosphate_(dUTP)_+_ADP_<=>_2-deoxyuridine_5-diph	0.00631355	0.00631355	#N/A
adenosine_5-diphosphate_(ADP)_+_CTP_<=>_ATP_+_cytidine_5-diphosphat	0.00631355	0.00631355	#N/A
adenosine_5-diphosphate_(ADP)_+_dATP_<=>_ATP_+_2-deoxyadenosine_5	0.00631355	0.00631355	#N/A
adenosine_5-diphosphate_(ADP)_+_dCTP_<=>_ATP_+_2-deoxycytidine_5-di	0.00631355	0.00631355	#N/A
adenosine_5-diphosphate_(ADP)_+_dGTP_<=>_ATP_+_2-deoxyguanosine_5	0.00631355	0.00631355	#N/A
adenosine_5-diphosphate_(ADP)_+_dUTP_<=>_ATP_+_2-deoxyuridine_5-di	0.00631355	0.00631355	#N/A
adenosine_5-diphosphate_(ADP)_+_GTP_<=>_ATP_+_guanosine_5-diphosp	0.00631355	0.00631355	#N/A

adenosine_5-diphosphate_(ADP)_+_TTP_<=>_ATP_+_thymidine_5-diphosph	0.00631355	0.00631355	#N/A
adenosine_5-diphosphate_(ADP)_+_UTP_<=>_ATP_+_uridine_5-diphosphat	0.00631355	0.00631355	#N/A
cytidine_5-diphosphate_(CDP)_+_ATP_<=>_CTP_+_adenosine_5-diphosphat	0.00631355	0.00631355	#N/A
guanosine_5-diphosphate_(GDP)_+_ATP_<=>_GTP_+_adenosine_5-diphospl	0.00631355	0.00631355	#N/A
thymidine_5-diphosphate_(TDP)_+_ATP_<=>_thymidine_5-triphosphate_(TT	0.00631355	0.00631355	#N/A
thymidine_5-diphosphate_(TDP)_+_ATP_<=>_TTP_+_adenosine_5-diphosph	0.00631355	0.00631355	#N/A
uridine_5-diphosphate_(UDP)_+_ATP_<=>_uridine_5-triphosphate_(UTP)_+_	0.00631355	0.00631355	#N/A
uridine_5-diphosphate_(UDP)_+_ATP_<=>_UTP_+_adenosine_5-diphosphat	0.00631355	0.00631355	#N/A
The_geminin_component_of_geminin:Cdt1_complexes_is_ubiquitinated,_re	0.230171	0.296006	0.292053
De_novo_formation_of_eIF2:GTP	0.936139	0.395076	0.972059
Met-tRNAi_binds_to_eIF2:GTP_to_form_the_ternary_complex	0.936139	0.395076	0.972059
Polyubiquitinated_NRIF_binds_to_p62_(Sequestosome)	0.256176	0.223651	0.434043
Polyubiquitinated_NRIF_migrates_to_the_nucleus	0.256176	0.223651	0.434043
factor_XIII_->_factor_XIII_cleaved_tetramer_+_2_factor_XIII_A_activation_p	0.583545	0.737597	0.296712
Exocytosis_of_fibrinogen	0.955392	0.955392	#N/A
n_fibrin_monomers_->_fibrin_multimer	0.955392	0.955392	#N/A
prothrombin_->_activated_thrombin_(factor_IIa)_+_thrombin_activation_p	0.538437	0.544496	0.503528
protein_C_->_activated_protein_C_+_protein_C_heavy_chain_activation_pe	0.142194	0.142194	#N/A
2-Phospho-D-glycerate_<=>_3-Phospho-D-glycerate	0.152397	0.152397	#N/A
3-Phospho-D-glycerate_<=>_2-Phospho-D-glycerate	0.152397	0.152397	#N/A
Activation_of_MBL	0.931181	0.841686	0.875575
MBL_binds_to_repetitive_carbohydrate_structures_on_the_surfaces_of_vir	0.931181	0.841686	0.875575
ATP_+_D-fructose_6-phosphate_=>_ADP_+_D-fructose_1,6-bisphosphate	0.555504	0.555504	#N/A
factor_X_->_factor_Xa_+_factor_X_activation_peptide_(VIIIa:IXa_catalyst)	0.989085	0.985161	0.814505
urokinase_plasminogen_activator_(one-chain):uPAR_->_urokinase_plasminc	0.388408	0.388408	#N/A
alpha-D-glucose_6-phosphate_+_NADP+_=>_D-glucono-1,5-lactone_6-phosp	0.0268483	0.307167	0.0224608
alpha-D-glucose_6-phosphate_+_NADP+_=>_D-glucono-1,5-lactone_6-phosp	0.0268483	0.307167	0.0224608
Spontaneous_hydrolysis_of_C3_thioester	0.457243	0.731792	0.28466
3,7,24THCA_is_conjugated_with_Coenzyme_A_(SLC27A2_VLCS)	0.140299	0.140299	#N/A
DHCA_is_conjugated_with_Coenzyme_A_(SLC27A2_VLCS)	0.140299	0.140299	#N/A
phytanate_+_CoA-SH_+_ATP_=>_phytanoyl-CoA_+_AMP_+_pyrophosphate	0.140299	0.140299	#N/A
pristanate_+_CoA-SH_+_ATP_=>_pristanoyl-CoA_+_AMP_+_pyrophosphate	0.140299	0.140299	#N/A
TetraHCA_is_conjugated_with_Coenzyme_A_(SLC27A2_VLCS)	0.140299	0.140299	#N/A
THCA_is_conjugated_with_Coenzyme_A_(SLC27A2_VLCS)	0.140299	0.140299	#N/A
Caspase_mediated_cleavage_of_BAP31	0.0824163	0.120336	0.192074
2-Phospho-D-glycerate_<=>_Phosphoenolpyruvate_+_H2O	0.839749	0.738117	0.793188
Phosphoenolpyruvate_+_H2O_<=>_2-Phospho-D-glycerate	0.839749	0.738117	0.793188
factor_IX_->_factor_IXa_+_factor_IX_activation_peptide_(TF:F7a_catalyst)	0.670496	0.568495	0.662033
D-fructose_1,6-bisphosphate_<=>_dihydroxyacetone_phosphate_+_D-glycei	0.130673	0.130673	#N/A
dihydroxyacetone_phosphate_+_D-glyceraldehyde_3-phosphate_<=>_D-fru	0.130673	0.130673	#N/A
Conversion_of_palmitic_acid_to_palmitoyl-CoA	0.105085	0.0112848	0.531752
factor_X_->_factor_Xa_+_factor_X_activation_peptide_(TF:F7_catalyst)	0.63947	0.742838	0.380556
factor_X_->_factor_Xa_+_factor_X_activation_peptide_(TF:F7a_catalyst)	0.63947	0.742838	0.380556
urokinase_plasminogen_activator_(two-chain):uPAR_+_plasminogen_activa	0.530062	0.530062	#N/A
ABCG1-mediated_transport_of_intracellular_cholesterol_to_the_cell_surfac	0.0908033	0.268507	0.115341
factor_VIII:von_Willibrand_factor_multimer_->_factor_VIIIa_+_factor_VIIIa_	0.782201	0.782201	#N/A
Beta_adrenoceptors_bind_catecholamines	0.15512	0.250618	0.210345

Formation_of_the_IGF:IGFBP-6_Complex	0.011395	0.149006	0.0201268
Formation_of_the_IGF:IGFBP-2_Complex	0.0253515	0.149006	0.0488837
kallikrein_+_C1Inh_->_kallikrein:C1Inh	0.447778	0.346076	0.547503
Cdc42_lures_Cbl_away_from_the_receptor	0.802339	#N/A	0.802339
PIP3_binds_to_RhoA_and_activates_it	0.140744	0.169645	0.303983
Transfer_of_LPS_onto_TLR4_from_CD14	0.86552	0.87796	0.604955
ADP_+_Orthophosphate_+_Succinyl-CoA_<=>_ATP_+_Succinate_+_CoA	0.210572	0.210572	#N/A
GDP_+_Orthophosphate_+_Succinyl-CoA_<=>_GTP_+_Succinate_+_CoA	0.210572	0.210572	#N/A
2-deoxyadenosine_5-diphosphate_(dADP)_+_ADP_<=>_2-deoxyadenosine_5	0.599452	0.599452	#N/A
2-deoxyadenosine_5-monophosphate_(dAMP)_+_ATP_<=>_2-deoxyadenosi	0.599452	0.599452	#N/A
cytidine_5-diphosphate_(CDP)_+_ADP_<=>_cytidine_5-monophosphate_(CN	0.599452	0.599452	#N/A
cytidine_5-monophosphate_(CMP)_+_ATP_<=>_cytidine_5-diphosphate_(CD	0.599452	0.599452	#N/A
Reduction_of_ferric_cytochrome_B5A_to_ferrous_cytochrome_B5A	0.390372	0.153125	0.832513
SAM_is_sythesized_from_methionines_reaction_with_ATP	0.610972	#N/A	0.610972
Phosphorylation_of_PKC_theta	0.801188	0.332642	0.910131
Autophosphorylation_of_PAK-2p34_in_the_activation_loop	0.0631369	#N/A	0.0631369
Partial_autophosphorylation_of_PAK-2_at__Ser-19,_Ser-20,_Ser-55,_Ser-19	0.0631369	#N/A	0.0631369
Proteolytic_PAK-2p34_fragment_translocates_to_the_nucleus	0.0631369	#N/A	0.0631369
Interaction_of_PECAM-1_and_SHP-1	0.880248	0.655489	0.876296
Interaction_of_PECAM-1_and_SHP-2	0.880248	0.655489	0.876296
2_acetyl-CoA_<=>_acetoacetyl-CoA+CoA	0.0255968	0.182104	0.0182561
acetoacetyl-CoA+CoA_<=>_2_acetyl-CoA	0.0255968	0.182104	0.0182561
alpha-methyl-acetoacetyl-CoA_+_CoA_<=>_propionyl-CoA_+_acetyl-CoA	0.0255968	0.182104	0.0182561
Caspase-mediated_cleavage_of_vimentin_at_DSVD_(85)	0.22757	0.137643	0.5985
perilipin_+_3_ATP_->_phosphorylated_perilipin_+_3_ADP	0.756529	0.608501	0.791983
Formation_of_the_IGF:IGFBP-4_Complex	0.0131628	0.155082	0.00799824
Release_of_Filamin-A	0.195365	0.195365	#N/A
Transport_(influx)_of_glycocholate_and_taurocholate_by_OATP-8	0.634231	#N/A	0.634231
Transport_(influx)_of_glycocholate_and_taurocholate_by_OATP-C	0.634231	#N/A	0.634231
2-deoxyuridine_5-monophosphate_(dUMP)_+_H2O_=>_2-deoxyuridine_+_c	0.0272855	0.0551549	0.142492
Caspase-mediated_cleavage_of_GAS2	0.244512	0.137643	0.634815
Ligand_bound_to_TLR6:TLR2	0.597154	0.693614	0.385975
L-Glutamine_transport_into_neurons	0.320905	0.219703	0.61323
Pp2a_mediated_localization_of_Rb_protein_in_chromatin	0.121102	#N/A	0.121102
Disassembly_of_SR-BI-bound_spherical_HDL	0.11146	0.11146	#N/A
spherical_HDL_and_SR-BI_receptor_form_a_complex_at_the_cell_surface	0.11146	0.11146	#N/A
Alpha-1_adrenoceptors_bind_catecholamines	0.511645	0.942409	0.140269
lactose_+_H2O_=>_D-glucose_+_D-galactose	0.459311	0.69019	0.316878
Viral_dsRNA:TLR3:TRIF_Complex_Recruits_RIP1	0.287361	#N/A	0.287361
Viral_dsRNA:TLR3:TRIF_Complex_Releases_Activated_RIP1	0.287361	#N/A	0.287361
Formation_of_the_IGF:IGFBP-1_Complex	0.105669	0.569233	0.00799824
By_PACAP_type_1_receptor	0.784468	0.760761	0.640737
Active_PLCG1_dissociates_from_TrkA_receptor	0.811472	0.861658	0.496327
Binding_of_PLCG1_to_active_TrkA_receptor	0.811472	0.861658	0.496327
MAPKAPK2_phosphorylates_CREB_at_Serine_133	0.532532	0.234827	0.729558
Cyclophosphamide_is_4-hydroxylated_by_CYP2B6	0.547249	0.810477	0.316593
CYP2J2_epoxygenates_arachidonic_acid	0.547249	0.810477	0.316593

Cbl_binds_to_Grb2	0.668765	0.146848	0.899254
CPT1_converts_palmitoyl-CoA_to_palmitoyl_carnitine	0.567559	0.567559	#N/A
Further_cleavage_of_LTD4_forms_LTE4	0.357494	0.305996	0.464852
2-deoxyguanosine_5-monophosphate_(dGMP)_+_H2O_=>_2-deoxyguanosir	0.147579	0.0551549	0.671942
2-deoxyinosine_5-monophosphate_(dIMP)_+_H2O_=>_2-deoxyinosine_+_oı	0.147579	0.0551549	0.671942
TRAIL_Mediated_Activation_of_Pro-caspase_8	0.119199	#N/A	0.119199
TRAIL:TRAIL-Receptor2_Trimer:FADD_complex_binds_Caspase-8	0.119199	#N/A	0.119199
Phosphorylation_of_Syntaxin-4	0.520446	0.455635	0.556268
Caspase-mediated_cleavage_of_Tau	0.0640129	0.0640129	#N/A
depalmitoylated_eNOS_translocates_from_plasma_membrane	0.734115	0.348483	0.948656
N-myristoylation_of_eNOS	0.734115	0.348483	0.948656
Caspase-mediated_cleavage_of_farnesyltransferase/geranyl-_geranyltransfe	0.12643	0.12643	#N/A
(S)-Lactate_+_NAD+_<=>_Pyruvate_+_NADH_+_H+	0.0136044	0.0136044	#N/A
Pyruvate_+_NADH_+_H+_<=>_(S)-Lactate_+_NAD+	0.0136044	0.0136044	#N/A
2-deoxyuridine_+_orthophosphate_<=>_uracil_+_2-deoxy-D-ribose_1-phosp	0.993467	0.995206	0.885971
uracil_+_2-deoxy-D-ribose_1-phosphate_<=>_2-deoxyuridine_+_orthophosp	0.993467	0.995206	0.885971
FAD_can_be_hydrolyzed_back_to_FMN	0.826717	#N/A	0.826717
Receptors_CXCR4_and_7_bind__CXCL12_ligand	0.235306	0.450769	0.141522
Dimerization_of_Tie2/Ang1_complex	0.130895	0.0430739	0.685656
Interaction_of_Tie2_with_Ang1	0.130895	0.0430739	0.685656
Caspase_mediated_cleavage_of_HIP-55	0.432446	0.432446	#N/A
MSK1_activates_CREB	0.613733	0.202561	0.827127
Dimerisation_of_phospho-ERK-1	0.282904	0.0999242	0.793339
Dimerisation_of_phospho-ERK-2	0.282904	0.0999242	0.793339
ERK5_translocates_to_the_nucleus	0.282904	0.0999242	0.793339
ERKs_are_inactivated_by_protein_phosphatase_2A	0.282904	0.0999242	0.793339
Nuclear_translocation_of_phospho-ERK-1_dimer	0.282904	0.0999242	0.793339
Nuclear_translocation_of_phospho-ERK-2_dimer	0.282904	0.0999242	0.793339
Phospho-R-SMAD_forms_a_complex_with_CO-SMAD	0.698187	0.141417	0.918563
The_phospho-R-SMAD:CO-SMAD_transfers_to_the_nucleus	0.698187	0.141417	0.918563
alpha-D-glucose_(extracellular)_<=>_alpha-D-glucose_(cytosol)_[GLUT1]	0.00149767	0.00149767	#N/A
alpha-D-glucose_(extracellular)_<=>_alpha-D-glucose_(cytosol)_[GLUT3]	0.00149767	0.00149767	#N/A
alpha-D-glucose_(extracellular)_<=>_alpha-D-glucose_(cytosol)_[GLUT4]	0.00149767	0.00149767	#N/A
Dehydroascorbate_transport_across_the_plasma_membrane	0.00149767	0.00149767	#N/A
PKA_catalytic_subunit_translocates_to_the_nucleus	0.892	0.681873	0.880973
Dissociation_of_phosphorylated_4EBP1_from_eIF4E	0.147561	0.104989	0.484117
Release_of_eIF4E_from_the_inactive_eIF4E:4E-BP_complex_	0.147561	0.104989	0.484117
Co-transport_(influx)_of_bile_salts_and_acids_and_sodium_ions_by_ASBT	0.797411	#N/A	0.797411
Cytochrome_C_Binds_to_Apaf-1	0.633097	0.690723	0.454046
Frs2_binds_to_active_TrkA_receptor	0.557049	0.572237	0.496327
Frs2_is_phosphorylated_by_active_TrkA_receptor	0.557049	0.572237	0.496327
urokinase_plasminogen_activator_(two-chain):uPAR_+_plasminogen_activa	0.101371	0.134377	0.260777
PRDM4_(SC1)_binds_to_p75NTR	0.470574	#N/A	0.470574
p75NTR_interacts_with_RIP2	0.24472	#N/A	0.24472
Caspase_mediated_cleavage_of_APC	0.121262	0.137643	0.315504
EIF5A_+_spermidine_<=>_EIF5A(Dhp)_+_1,3-diaminopropane	0.40001	0.179001	0.63315
EIF5A(Dhp)_+_1,3-diaminopropane_<=>_EIF5A_+_spermidine	0.40001	0.179001	0.63315

FA_anion_diffuses_laterally_to_UCP	0.38851	0.612387	0.185759
FA_anion_flip-flops_to_the_opposite_surface	0.38851	0.612387	0.185759
Protons_are_translocated_from_the_intermembrane_space_to_the_matrix	0.38851	0.612387	0.185759
The_FA_anion_diffuses_away_laterally_from_UCP	0.38851	0.612387	0.185759
2-deoxycytidine_+_ATP_=>_2-deoxycytidine_5-monophosphate_(dCMP)_+_	0.0984436	0.122037	0.224934
2-deoxycytidine_+_ATP_=>_2-deoxycytidine_5-monophosphate_+_ADP	0.0984436	0.122037	0.224934
2-deoxyguanosine_+_ATP_=>_2-deoxyguanosine_5-monophosphate_+_ADP	0.0984436	0.122037	0.224934
2-deoxyinosine_+_ATP_=>_2-deoxyinosine_5-monophosphate_+_ADP	0.0984436	0.122037	0.224934
2-deoxyuridine_+_ATP_=>_2-deoxyuridine_5-monophosphate_+_ADP	0.0984436	0.122037	0.224934
thymidine_+_ATP_=>_thymidine_5-monophosphate_+_ADP	0.0984436	0.122037	0.224934
Relocalization_of_nuclearly_localized_phospho-(T286):cyclin_D1:Cdk4_to_cy	0.334653	0.334653	#N/A
Orc5_associates_with_Orc3:Orc2:origin_complexes	0.00154644	#N/A	0.00154644
Phosphorylation_of_Cyclin_D1_on_Thr-286_by_GSK-3_beta	0.619291	0.454842	0.753461
LDLR_[endosome_membrane]_=>_LDLR_[plasma_membrane]	0.987373	0.975592	0.910669
BAD_displaces_tBID_from_BCL-2_sequestration	0.859848	0.670201	0.893469
Release_of_Bromodomain_and_PHD_finger-containing_protein_3	0.822969	0.530523	0.93253
ERK1/2_activates_ELK1	0.532395	0.0936958	0.849034
Phosphorylation_of_Cdc25A_at_Ser-123_by_Chk2	0.7216	0.719749	0.576837
Association_of_RAD50:MRE11_complex_with_NBS1_via_MRE11_interactior	0.212684	0.980976	0.00726578
Bradykinin_receptors_B1_and_B2_bind_to_bradykinin	0.212328	0.192746	0.358156
Nuclear_translocation_of_catalytic_domain_of_Mst3	0.138795	0.0255136	0.810278
cis-Aconitate_+_H2O_<=>_Isocitrate	0.467996	0.822783	0.225513
Citrate_<=>_cis-Aconitate_+_H2O	0.467996	0.822783	0.225513
G_alpha_13_activates_Rho_guanine_nucleotide_exchange_factor_1_(p115-	0.998674	0.852367	0.998351
Caspase-mediated_cleavage_of_Acinus	0.204938	0.204938	#N/A
Succinyl_CoA_and_glycine_condense_to_form_5-aminolevulinate_(ALA)	0.596986	0.0984692	0.887544
Dicer_cleaves_pre-miRNA_to_mature_miRNA	0.382691	0.133066	0.854295
MSK1_activates_ATF1	0.680711	0.472839	0.818246
Release_of_Serotransferrin	0.117505	0.195771	0.199387
Ligands_bind_L-selectin	0.825789	#N/A	0.825789
Calcium_binds_calmodulin	0.11127	0.233396	0.139611
Release_of_Calmodulin	0.11127	0.233396	0.139611
TRAF6_is_Recruited_to_the_Viral_dsRNA:TLR3:TRIF_Complex	0.615556	0.886202	0.310581
Activation_of_Cdc25C	0.833416	0.910759	0.591947
Dissociation_of_Caspase-9_from_SMAC:XIAP:Caspase-9	0.413483	0.413483	#N/A
SMAC_binds_XIAP:Caspase-9	0.413483	0.413483	#N/A
p75NTR_binds_to_NADE	0.878149	0.947315	0.610706
Translocation_of_RIAM_to_plasma_membrane	0.647227	0.26258	0.939606
NKG2D_homodimer_interacting_with_ligands	0.545273	0.661562	0.438107
Association_of_DNA-PKcs_with_Ku-bound_ends_of_DNA_double-strand_bre	0.918114	#N/A	0.918114
Removal_of_3-phosphoglycolate_(PG)__moiety_from_DSB_ends	0.918114	#N/A	0.918114
Synapsis,_or_interaction_between_two_DNA-PK:DNA_complexes_at_oppos	0.918114	#N/A	0.918114
Sar1p_Activation_And_Membrane_Binding	0.413217	0.413217	#N/A
Interaction_of_Tie2_with_Ang2	0.563399	0.441596	0.685656
Cholecystokinin_receptors_bind_cholecystokinin	0.62691	#N/A	0.62691
FasL:Fas_binds_FADD	0.212641	0.880197	0.0352783
Caspase-mediated_cleavage_of_claspin	0.114507	0.137643	0.294778

Binding_of_IP3_to_IP3_receptor	0.89814	0.597838	0.916296
Entry_of_Ca++_from_platelet_dense_tubular_system	0.89814	0.597838	0.916296
IP3_binds_with_the_IP3_receptor,_opening_the_Ca2+_channel	0.89814	0.597838	0.916296
Release_of_calcium_from_intracellular_stores_by_IP3_receptor_activation	0.89814	0.597838	0.916296
3-carboxy-1-hydroxypropyl-TPP_+_lipoamide_=>_S-succinyldihydrolipoamid	0.667865	0.667865	#N/A
4-carboxy-1-hydroxybutyl-TPP_+_lipoamide_=>_S-glutaryldihydrolipoamide_	0.667865	0.667865	#N/A
alpha-ketoadipate_+_TPP_=>_4-carboxy-1-hydroxybutyl-TPP_+_CO2	0.667865	0.667865	#N/A
alpha-ketoglutarate_+_TPP_=>_3-carboxy-1-hydroxypropyl-TPP_+_CO2	0.667865	0.667865	#N/A
dihydrolipoamide_+_FAD_=>_lipoamide_+_FADH2_[alpha-ketoglutarate_de	0.667865	0.667865	#N/A
FADH2_+_NAD+_=>_FAD_+_NADH_+_H+_[alpha-ketoglutarate_dehydrogen	0.667865	0.667865	#N/A
S-glutaryldihydrolipoamide_+_CoA_=>_glutaryl-CoA_+_dihydrolipoamide	0.667865	0.667865	#N/A
S-succinyldihydrolipoamide_+_CoA_=>_succinyl-CoA_+_dihydrolipoamide	0.667865	0.667865	#N/A
Pyruvate_+_CO2_+_ATP_=>_ADP_+_Orthophosphate_+_Oxaloacetate	0.634381	0.634381	#N/A
Transfer_of_electrons_from_ETF_to_ubiquinone_by_ETF-QO	0.137993	0.137993	#N/A
Thyroxine_is_deiodinated_to_triiodothyronine	0.736201	0.723393	0.638185
Vamp7_associated_Lysosome_to_Plasma_membrane_transport	0.761009	0.253516	0.984805
Formation_of_P-TEFb_complex	0.714955	0.414048	0.802004
Interaction_of_Bcl10_to_CARMA1	0.630092	0.394768	0.829344
Oligomerization_of_Bcl10	0.630092	0.394768	0.829344
Vamp8_associated_secretory_vesicle_to_plasma_membrane_transport	0.0926737	0.0926737	#N/A
Active_JNK_moves_to_the_nucleus_and_phosphorylates_different_transcri	0.504378	0.74066	0.328751
Formation_of_elongin_complex	0.919092	0.783936	0.904903
Myristoylation_of_tBID_by_NMT1	0.990122	0.950966	0.954928
Nuclear_export_of_snRNA_transcripts	0.868522	0.6632	0.858197
Rab5-mediated_recruitment_of_class_III_PI3K_to_TLR9	0.896022	0.916913	0.712822
Interaction_of_Tie2_and_Ang4	0.52818	0.164067	0.781837
DARC__is_a_non-specific_receptor_for_many_chemokines	0.230413	#N/A	0.230413
Phosphorylation_of_Cdc25A_at_Ser-123_by_Chk1	0.660307	0.643831	0.576837
Active_NTRK1_binds_IRS1/2	0.137347	0.0916234	0.496327
Recruitment_of_Acetylated_SL1_to_phosUBF-1:rDNA_Promoter	0.318455	0.433318	0.281142
Association_of_Mal_to_activated_TLR4	0.886103	0.901128	0.604955
Sphingomyelinase_is_activated_by_the_NGF:p75NTR_complex	0.275241	0.0763051	0.610706
IRAK1-P(n):TRAF6_binds_MEKK1	0.74814	0.74814	#N/A
Association_of_TFAM:mt_promoter_complex_with_POLRMT:TFB2M	0.357172	0.357172	#N/A
SLC7A1_(CAT-1)-mediated_uptake_of_cationic_amino_acids	0.702775	0.93059	0.120826
SLC7A2,_isoform_A_(CAT-2A)-mediated_uptake_of_cationic_amino_acids	0.702775	0.93059	0.120826
SLC7A3_(CAT-3)-mediated_uptake_of_cationic_amino_acids	0.702775	0.93059	0.120826
Acetylation_of_SL1	0.175697	0.232419	0.281142
Receptor_CXCR1_binds_CXCL6_and_CXCL8_ligands	0.479216	#N/A	0.479216
uridine_+_ATP_=>_uridine_5-monophosphate_(UMP)_+_ADP	0.0198272	0.0198272	#N/A
Neurotensin_receptors_can_bind_neurotensins	0.730068	0.481198	0.870131
5-HT2_receptor_can_bind_serotonin	0.691301	0.810205	0.495883
M1,_M3_and_M5_receptors_bind_acetylcholine	0.695588	#N/A	0.695588
Re-integration_of_insulin_receptor_into_plasma_membrane	0.714919	0.911473	0.177369
dehydroepiandrosterone_(DHEA)_+_PAPS_=>_DHEA_sulfate_+_PAP	0.779356	#N/A	0.779356
Disassociation_of_PLC-gamma1_from_LAT	0.489003	0.378315	0.651772
Recruitment_of_PLC-gamma1_to_LAT	0.489003	0.378315	0.651772

Association_of__RPA_complexes_with_ssDNA	0.220588	0.220588	#N/A
Stalling_of_DNA_replication_fork_and_RPA_binding	0.220588	0.220588	#N/A
Formation_of_eEF1B_complex	0.864929	0.548147	0.896933
Opioid_binds_MOR	0.0819853	0.330685	0.0816487
Opioid_dissociates_from_MOR	0.0819853	0.330685	0.0816487
phenylalanine_+_tRNA(Phe)_+_ATP_=>_Phe-tRNA(Phe)_+_AMP_+_pyropho	0.343725	0.684203	0.0847568
DIT_and_MIT_combine_to_form_triiodothyronine	0.227942	0.425248	0.217826
Iodide_is_organified	0.227942	0.425248	0.217826
Two_DITs_combine_to_form_thyroxine	0.227942	0.425248	0.217826
Tyrosine_is_diiodinated	0.227942	0.425248	0.217826
Tyrosine_is_monoiodinated	0.227942	0.425248	0.217826
Receptors_CCR1,_2_and_8_bind_CCL16_ligand	0.629005	0.629005	#N/A
Dissociation_of_LIG3:XRCC1_complex_from_site_of_BER	0.642239	0.586643	0.625889
DNA_ligation_via_the_single-nucleotide_replacement_pathway	0.642239	0.586643	0.625889
Recruitment__of__LIG3:XRRC1_complex_to_the_site_of_repair_by_POL_Be	0.642239	0.586643	0.625889
Resynthesis_of_excised_residue	0.642239	0.586643	0.625889
Vasopressin_receptor_type_1_bind_vasopressin	0.497276	0.823165	0.253092
Gonadotropin-releasing_hormone_receptor_can_bind_GnRH	0.229188	0.405311	0.230158
5-HT4,_6_and_7_receptors_can_bind_serotonin	0.718571	0.81708	0.527235
XPC_binds_to_HR23B_forming_a_heterodimeric_complex	0.655585	0.655585	#N/A
XPC:HR23B_complex_binds_to_damaged_DNA_site_with_lesion	0.655585	0.655585	#N/A
Recruitment_of_Gads_to_LAT	0.216354	0.10811	0.651772
Transport_of_the_Mature_Intronless_Transcript_Derived_Histone_mRNA:T/	0.487703	0.337566	0.602077
Transport_of_the_Mature_intronless_transcript_derived_mRNA:TAP:Aly/Re	0.487703	0.337566	0.602077
Basigin_binds_Matrix_metalloproteinase-1	0.399993	0.626334	0.185729
Association_of_HMGB1/HMGB2_with_chromatin	0.101808	#N/A	0.101808
Digestion_of_diacylglycerols_by_extracellular_PTL:colipase	0.270852	0.696659	0.133183
Digestion_of_triacylglycerols_by_extracellular_PTL:colipase	0.270852	0.696659	0.133183
Reuptake_of_dopamine_from_the_synaptic_cleft_into_neurons	0.810068	0.348697	0.911506
Receptor_CCR10_binds_CCL27_and_28_ligands	0.429903	#N/A	0.429903
IkB_is_ubiquitinated_and_degraded	0.396503	0.526311	0.292053
glutathione_(oxidized)_+_NADPH_+_H+_=>_2_glutathione_(reduced)_+_NA	0.812359	0.688094	0.799722
Phosphorylated_Orc1_is_ubiquitinated_while_still_associated_with_chroma	0.156748	0.287403	0.201285
Ubiquitinated_Orc1_enters_the_cytosol	0.156748	0.287403	0.201285
activated_thrombin_(factor_IIa)_+_antithrombin_III:heparin_->_thrombin:a	0.247349	0.277238	0.354181
thrombin:antithrombin_III:heparin_->_thrombin:cleaved_antithrombin_III:h	0.247349	0.277238	0.354181
thrombin:cleaved_antithrombin_III:heparin_->_thrombin:cleaved_antithrom	0.247349	0.277238	0.354181
2_phosphorylated_HSL_monomers_->_phosphorylated_HSL_dimer	0.456879	0.456879	#N/A
phosphorylated_HSL_+_H2O_->_HSL_+_orthophosphate	0.456879	0.456879	#N/A
Phosphorylated_HSL_dimer_translocates_from_the_cytosol_to_the_lipid_p	0.456879	0.456879	#N/A
D-fructose_1,6-bisphosphate_+_H2O_=>_D-fructose_6-phosphate_+_orthop	0.882445	0.644973	0.904213
prothrombin_->_activated_thrombin_(factor_IIa)_+_thrombin_activation_p	0.544496	0.544496	#N/A
histidine-rich_glycoprotein_+_plasminogen_<->_histidine-rich_glycoprotein:	0.830209	0.858209	0.609158
histidine-rich_glycoprotein:plasminogen_<->_histidine-rich_glycoprotein_+_	0.830209	0.858209	0.609158
plasminogen_+_histidine-rich_glycoprotein_->_plasminogen:histidine-rich_g	0.830209	0.858209	0.609158
Coumarin_is_7-hydroxylated_by_CYP2A13	0.185018	#N/A	0.185018
Coumarin_is_7-hydroxylated_by_CYP2A6	0.185018	#N/A	0.185018

creatine_+_ATP_=>_phosphocreatine_+_ADP_[CKB,CKM]	0.211411	0.211411	#N/A
ADP-ATP_translocase_maintains_a_high_ADP:ATP_ratio_in_the_matrix	0.451451	0.315135	0.621268
m_UDP-glucose_+_{(1,4)-alpha-D-glucosyl}n_glycogenin-1_=>_m_UDP_+_{(1	0.132222	0.132222	#N/A
m_UDP-glucose_+_{(1,4)-alpha-D-glucosyl}n_glycogenin-1_=>_m_UDP_+_{(1	0.132222	0.132222	#N/A
m_UDP-glucose_+_{(1,4)-alpha-D-glucosyl}n_glycogenin-2_=>_m_UDP_+_{(1	0.132222	0.132222	#N/A
m_UDP-glucose_+_{(1,4)-alpha-D-glucosyl}n_glycogenin-2_=>_m_UDP_+_{(1	0.132222	0.132222	#N/A
activated_thrombin_(factor_IIa)_+_thrombomodulin_->_activated_thrombin	0.137895	0.137895	#N/A
factor_V_->_factor_Va_+_factor_V_activation_peptide	0.340205	0.277238	0.503528
chylomicron_remnant_+_apoE_=>_chylomicron_remnant:apoE_complex	0.955353	#N/A	0.955353
factor_XIII_cleaved_tetramer_+_2_Ca++_->_factor_XIIIa_+_2_factor_XIII_B_	0.750613	0.931841	0.296712
alpha-2-antiplasmin_+_plasmin_->_alpha-2-antiplasmin:plasmin	0.861688	0.861688	#N/A
Caspase_mediated_cleavage_of_vimentin_at_IDVD_(259)	0.271427	#N/A	0.271427
ADP_+_Phosphoenolpyruvate_=>_ATP_+_Pyruvate_(pyruvate_kinase_M2)	0.225007	0.0757688	0.643
2-deoxycytidine_5-diphosphate_(dCDP)_+_ADP_<=>_deoxycytidine_5-monc	0.266063	0.266063	#N/A
2-deoxycytidine_5-monophosphate_(dCMP)_+_ATP_<=>_deoxycytidine_5-d	0.266063	0.266063	#N/A
uridine_5-diphosphate_(UDP)_+_ADP_<=>_uridine_5-monophosphate_(UM	0.266063	0.266063	#N/A
uridine_5-monophosphate_(UMP)_+_ATP_<=>_uridine_5-diphosphate_(UDI	0.266063	0.266063	#N/A
Co-transport_(influx)_of_bile_salts_and_sodium_ions_by_NTCP	0.420718	#N/A	0.420718
limit_dextrin-glycogenin-1_=>_{(1,6)-alpha-glucosyl}poly{(1,4)-alpha-glucosy	0.553856	0.553856	#N/A
limit_dextrin-glycogenin-2_=>_{(1,6)-alpha-glucosyl}poly{(1,4)-alpha-glucosy	0.553856	0.553856	#N/A
{(1,6)-alpha-glucosyl}poly{(1,4)-alpha-glucosyl}glycogenin-1_=>_poly{(1,4)-al	0.553856	0.553856	#N/A
{(1,6)-alpha-glucosyl}poly{(1,4)-alpha-glucosyl}glycogenin-2_=>_poly{(1,4)-al	0.553856	0.553856	#N/A
aspartate_[mitochondrial_matrix]_+_glutamate_[cytosol]_=>_aspartate_[cy	0.658332	0.802524	0.391907
crosslinked_fibrin_multimer:tissue_plasminogen_activator_(one-chain)_+_p	0.583237	0.583237	#N/A
crosslinked_fibrin_multimer:tissue_plasminogen_activator_(one-chain)_->_c	0.583237	0.583237	#N/A
crosslinked_fibrin_multimer:tissue_plasminogen_activator_(one-chain):plasi	0.583237	0.583237	#N/A
crosslinked_fibrin_multimer:tissue_plasminogen_activator_(two-chain)_+_p	0.583237	0.583237	#N/A
crosslinked_fibrin_multimer:tissue_plasminogen_activator_(two-chain):plasi	0.583237	0.583237	#N/A
3-ketohexacosanoyl-CoA_+_CoASH_=>_tetracosanoyl-CoA_+_acetyl-CoA	0.573625	0.833517	0.240097
Connexin_oligomerization_in__ER-Golgi-Intermediate_Compartment	0.938879	0.948766	0.709122
factor_Va_+_factor_Xa_->_Va:Xa_complex_(prothrombinase)	0.704118	0.773035	0.503528
lysophosphatic_acid_+_fatty_acyl_CoA__=>_phosphatidic_acid_+_CoA_(1)_	0.537739	0.602228	0.450116
By_adenosine_A2a_receptor	0.631775	0.770058	0.39606
S-Adenosyl_methionine_<=>_Decarboxylated-Adenosyl_methionine_+_CO2	0.571014	#N/A	0.571014
Ligand_binds_to_TLR7_or_TLR8	0.639142	#N/A	0.639142
Binding_of_APOBEC-1_to_form_editosome	0.864732	#N/A	0.864732
C4_deamination_of_cytidine	0.864732	#N/A	0.864732
perilipin:CGI-58_complex_->_perilipin_+_CGI-58	0.370442	0.46658	0.350647
factor_VIIIa_+_factor_IXa_->_factor_VIIIa:factor_IXa	0.988621	0.98993	0.814505
factor_VII_->_factor_VIIa	0.742838	0.742838	#N/A
factor_Va_->_factor_Vi	0.415091	0.377498	0.503528
Translocation_of_PKC_theta_to_plasma_membrane	0.641346	0.332642	0.827453
acetoacetyl-CoA+acetyl-CoA_=>_HMG-CoA	0.9936	0.989041	0.88987
Condensation_of_acetyl_CoA_with_acetoacetyl_CoA_to_form_HMG-CoA	0.9936	0.989041	0.88987
(2S)-pristanoyl-CoA_+_O2_=>_trans-2,3-dehydropristanoyl-CoA_+_H2O2_(A	0.700692	0.58392	0.702573
25(S)_DHCA-CoA_is_dehydrogenated_to_25(S)_3alpha,7alpha-dihydroxy-5b	0.700692	0.58392	0.702573
25(S)_THCA-CoA_is_dehydrogenated_to_25(S)_3alpha,7alpha,12alpha-trihy	0.700692	0.58392	0.702573

hexacosanoyl-CoA_+_O2_=>_trans-2,3-dehydrohexacosanoyl-CoA_+_H2O2	0.700692	0.58392	0.702573
urokinase_plasminogen_activator_+_urokinase_plasminogen_activator_rece	0.134377	0.134377	#N/A
isocitrate_+_NADP+_=>_2-oxoglutarate_+_CO2_+_NADPH_+_H+	0.305276	0.106549	0.700297
eEF1A_complexes_with_GTP	0.00351923	0.00351923	#N/A
eEF1A:GTP:aminoacyl_tRNA_ternary_complex_formation.	0.00351923	0.00351923	#N/A
Caspase-mediated_cleavage_of_vimentin_at_TNLD_(429)	0.414059	0.288962	0.5985
fibrin_multimer,_crosslinked:tissue_plasminogen_activator_(one-chain)_+_p	0.742008	0.742008	#N/A
fibrin_multimer,_crosslinked:tissue_plasminogen_activator_(two-chain)_+_p	0.742008	0.742008	#N/A
ADP_is_exchanged_for_ATP_in_the__ADP:CCT/TriC:tubulin_complex	0.869159	0.809903	0.760684
Hydrolysis_of_ATP_and_release_of_tubulin_folding_intermediate_from_CCT	0.869159	0.809903	0.760684
Isocitrate_+_NAD+_=>_alpha-ketoglutarate_+_CO2_+_NADH_+_H+	0.918183	0.516657	0.972984
Caspase-8_activates_BID_by_cleavage	0.537114	0.892705	0.133604
CYP2S1_can_deactivate_all-trans-retinoic_acid	0.17987	0.207304	0.316115
GPI-bound_CD14_binds_LPS	0.960032	0.609385	0.985979
Secreted_CD14_binds_LPS	0.960032	0.609385	0.985979
glycolate_+_O2_=>_glyoxylate_+_H2O2	0.338117	#N/A	0.338117
glyoxylate_+_O2_=>_oxalate_+_H2O2	0.338117	#N/A	0.338117
isoleucine_+_alpha-ketoglutarate_<=>_alpha-keto-beta-methylvalerate_+_g	0.0574918	0.208818	0.0782384
isoleucine_+_alpha-ketoglutarate_<=>_alpha-keto-beta-methylvalerate_+_g	0.0574918	0.208818	0.0782384
L-Glutamate_[cytosolic]_from_leucine_catabolism	0.0574918	0.208818	0.0782384
L-Glutamate_[mitochondrial]_from_leucine_catabolism	0.0574918	0.208818	0.0782384
valine_+_alpha-ketoglutarate_<=>_alpha-ketoisovalerate_+_glutamate_[cyto	0.0574918	0.208818	0.0782384
valine_+_alpha-ketoglutarate_<=>_alpha-ketoisovalerate_+_glutamate_[mit	0.0574918	0.208818	0.0782384
Orc3_associates_with_Orc2_constitutively_bound_at_origins_of_replication	0.000686666	#N/A	0.000686666
CD47_binds_SIRP	0.921099	0.963295	0.581488
Glutamate_and_cysteine_combine	0.618631	0.331629	0.805629
inosine_5-monophosphate_+_L-aspartate_+_GTP_=>_adenylosuccinate_+_g	0.225383	0.212982	0.393679
kininogen_+_C1q_binding_protein_tetramer_->_kininogen:C1q_binding_pro	0.602005	0.604723	0.539684
Release_of_novel_PDGFs_as_latent_factors	0.594033	#N/A	0.594033
pro-prothrombin,_uncarboxylated_+_10_CO2_+_10_O2_+_10_vitamin_K_hy	0.515923	0.277238	0.741299
Dimeric_TGF-beta1_binds_to_the_receptor	0.776274	0.765174	0.636968
Reduction_of_semidehydroascorbate_to_ascorbate	0.67153	0.368199	0.832513
Conversion_of_Serotonin_to_5-hydroxyindole_acetaldehyde	0.437645	0.590391	0.326185
Dietary_tyramine_is_oxidatively_deaminated_to_an_aldehyde_by_MAOB	0.437645	0.590391	0.326185
Metabolism_of_Noradrenaline	0.437645	0.590391	0.326185
Oxidation_of_3-Methoxytyramine_to_homovanillic_acid	0.437645	0.590391	0.326185
oxidation_of_dopamine_to_3,4dihydroxyphenylacetic_acid_(DOPAC)	0.437645	0.590391	0.326185
Oxidative_deamination_of_5-Hydroxytryptamine_by_MAOA	0.437645	0.590391	0.326185
Oxidative_deamination_of_Phenyethylamine_by_MAOB	0.437645	0.590391	0.326185
uridine_5-triphosphate_+_glutamine_+_ATP_+_H2O_=>_cytidine_5-triphosp	0.848403	0.848403	#N/A
CD200_binds_to_CD200R	0.681156	0.941101	0.184404
Caveolin-1_binds_Basigin	0.0268513	0.0268513	#N/A
Thrombin-mediated_activation_of_PAR3	0.37395	0.277238	0.554377
factor_XIIa_+_C1Inh_->_factor_XIIa:C1Inh	0.879836	0.784372	0.80843
tissue_factor_(TF)_+_activated_factor_VII_(F7a)_->_TF:F7a_complex	0.462975	0.568495	0.380556
tissue_factor_(TF)_+_factor_VII_(F7)_->_TF:F7_complex	0.462975	0.568495	0.380556
vitamin_K_epoxide_->_vitamin_K_hydroquinone	0.0841148	#N/A	0.0841148

OLR1_binds_to_oxidized_LDL	0.658115	0.408549	0.790099
pro-factor_IX,_uncarboxylated_+_12_CO2_+_12_O2_+_12_vitamin_K_hydro	0.862416	#N/A	0.862416
ATP_+_D-galactose_=>_ADP_+_D-galactose_1-phosphate	0.640291	0.640291	#N/A
Release_of_Talin-1	0.307742	0.307742	#N/A
arginine_=>_ornithine_+_urea	0.189042	0.173133	0.380605
Activation_of_PKC	0.556268	#N/A	0.556268
aspartate_+_alpha-ketoglutarate_<=>_oxaloacetate_+_glutamate_[cytosol]	0.317356	0.317356	#N/A
aspartate_+_alpha-ketoglutarate_<=>_oxaloacetate_+_glutamate_[mitochor	0.317356	0.317356	#N/A
oxaloacetate_+_glutamate_<=>_aspartate_+_alpha-ketoglutarate_[cytosolic]	0.317356	0.317356	#N/A
oxaloacetate_+_glutamate_<=>_aspartate_+_alpha-ketoglutarate_[mitochor	0.317356	0.317356	#N/A
cytidine_5-monophosphate_(CMP)_+_H2O_=>_cytidine_+_orthophosphate	0.276349	#N/A	0.276349
Exocytosis_of_protease_nexin_II_APP	0.973523	0.987891	0.684785
Binding_of_HREV1_to_lesioned_DNA_template	0.0196207	#N/A	0.0196207
Elongation_by_HREV1_protein	0.0196207	#N/A	0.0196207
Misinsertion_of__bases_opposite_to_the_lesion_by_HREV1	0.0196207	#N/A	0.0196207
Thrombin-mediated_activation_of_PAR4	0.380047	0.277238	0.563328
Sequestration_of_tBID_by_BCL-2	0.960747	0.892705	0.893469
D-ribose_5-phosphate_+_2-deoxyadenosine_5-triphosphate_(dATP)_=>_5-P	0.720798	0.720798	#N/A
D-ribose_5-phosphate_+_ATP_=>_5-phospho-alpha-D-ribose_1-diphosphate	0.720798	0.720798	#N/A
ABCG5:ABCG8-mediated_export_of_cholesterol_and_phytosterols	0.984172	0.959864	0.900998
Ligand_binds_to_TLR10	0.665587	0.81439	0.385975
1-palmitoylglycerone_phosphate_+_hexadecanol_=>_O-hexadecylglycerone	0.771712	0.643523	0.753037
palmitoyl-CoA_+_DHAP_=>_1-palmitoylglycerone_phosphate_+_CoASH	0.771712	0.643523	0.753037
pro-factor_X,_uncarboxylated_+_11_CO2_+_11_O2_+_11_vitamin_K_hydro	0.83845	0.773035	0.741299
(S)-3-Hydroxyhexadecanoyl-CoA+NAD<=>3-Oxopalmitoyl-CoA+NADH+H	0.211247	0.211247	#N/A
(S)-3-Hydroxytetradecanoyl-CoA+NAD<=>3-Oxotetradecanoyl-CoA+NADH+H	0.211247	0.211247	#N/A
3-Oxodecanoyl-CoA+CoA-SH<=>Octanoyl-CoA	0.211247	0.211247	#N/A
3-Oxododecanoyl-CoA+CoA-SH<=>Decanoyl-CoA	0.211247	0.211247	#N/A
3-Oxohexanoyl-CoA+CoA-SH<=>Butanoyl-CoA	0.211247	0.211247	#N/A
3-Oxooctanoyl-CoA+CoA-SH<=>Hexanoyl-CoA	0.211247	0.211247	#N/A
3-Oxopalmitoyl-CoA+CoA-SH<=>myristoyl-CoA	0.211247	0.211247	#N/A
3-Oxotetradecanoyl-CoA+CoA-SH<=>Lauroyl-CoA	0.211247	0.211247	#N/A
trans-Hexadec-2-enoyl-CoA+H2O<=>(S)-3-Hydroxyhexadecanoyl-CoA	0.211247	0.211247	#N/A
trans-Tetradec-2-enoyl-CoA+H2O<=>(S)-3-Hydroxytetradecanoyl-CoA	0.211247	0.211247	#N/A
beta-NGF_dimer_binds_to_TrkA_receptor	0.697262	0.770058	0.496327
The_bound_receptor_dimerizes	0.697262	0.770058	0.496327
TrkA_receptor_autophosphorylates	0.697262	0.770058	0.496327
Ethanol_+_NAD+_<=>_Acetaldehyde_+_NADH_+_H+_[class_IV,_mu_or_sign	0.678862	0.53252	0.717254
adenylyl_sulfate_(APS)_+_ATP_=>_PAPS_+_ADP	0.357417	#N/A	0.357417
ATP_+_sulfate_=>_adenylyl_sulfate_(APS)_+_pyrophosphate	0.357417	#N/A	0.357417
Trans-phosphorylation_of_Tie2	0.658049	#N/A	0.658049
poly{(1,4)-alpha-D-glucosyl}_glycogenin-1_=>_glycogen-glycogenin-1	0.483718	0.189628	0.794304
poly{(1,4)-alpha-D-glucosyl}_glycogenin-2_=>_glycogen-glycogenin-2	0.483718	0.189628	0.794304
Phosphorylation_of_PDE3B_by_AKT-1	0.253174	0.374481	0.267884
Viral_dsRNA_bound_TRL3_Recruits_TRIF	0.310581	#N/A	0.310581
Noradrenaline_clearance_from_the_synaptic_cleft	0.461424	#N/A	0.461424
adenosine_5-monophosphate_(AMP)_+_H2O_=>_inosine_5-monophosphate	0.627771	#N/A	0.627771

Hypoxanthine_+_H2O_+_O2_=>_Xanthine_+_H2O2	0.589611	0.628862	0.496479
Xanthine_+_H2O_+_O2_=>_Urate_+_H2O2	0.589611	0.628862	0.496479
Hydrolysis_of_cAMP_to_5_AMP_by_Phosphorylated_PDE3B	0.70233	0.914601	0.267884
Interaction_of_PECAM-1_and_PLC_gamma1	0.739549	0.787586	0.543394
pro-protein_Z,_uncarboxylated_+_13_CO2_+_13_O2_+_13_vitamin_K_hydr	0.947317	0.949598	0.741299
tBID_activates_BAK_protein	0.750662	0.750662	#N/A
tBID_binds_to_inactive_BAK_protein	0.750662	0.750662	#N/A
Formation_of_RAD50:MRE11_complex	0.703251	0.980976	0.0933909
Exocytosis_of_PDGF	0.0920939	0.0533336	0.396111
pro-protein_C,_uncarboxylated_+_8_CO2_+_8_O2_+_8_vitamin_K_hydroqu	0.593811	0.377498	0.741299
Interaction_of_Csk_with_PAG	0.988196	#N/A	0.988196
Reduction_of_androstenedione_to_testosterone	0.533617	#N/A	0.533617
apoA-I_binds_to_CUBN:AMN	0.434482	0.434482	#N/A
NAT1_acetylation	0.721695	#N/A	0.721695
NAT2_acetylation	0.721695	#N/A	0.721695
The_acetyl_group_from_acetyl-CoA_is_transferred_to_the_NAT1	0.721695	#N/A	0.721695
ACTH_specifically_binds_with_melanocortin_receptor_2	0.267436	#N/A	0.267436
Nicotinate_D-ribonucleotide_+_ATP_=>_deamino-NAD+_+_pyrophosphate_	0.299488	0.0719245	0.763722
Nicotinate_D-ribonucleotide_+_ATP_=>_deamino-NAD+_+_pyrophosphate_	0.299488	0.0719245	0.763722
Ascorbate_transport_across_the_plasma_membrane	0.860545	#N/A	0.860545
Reduction_of_2-trans-4-cis-decadienoyl-CoA_to_form_3-trans-decenoyl-CoA	0.545974	0.545974	#N/A
ADP_+_3-Phospho-D-glyceroyl_phosphate_<=>_ATP_+_3-Phospho-D-glycera	0.200688	#N/A	0.200688
ATP_+_3-Phospho-D-glycerate_<=>_ADP_+_1,3-bisphospho-D-glycerate	0.200688	#N/A	0.200688
factor_VIII_+_von_Willebrand_factor_multimer_->_factor_VIII:von_Willibrar	0.915063	0.915063	#N/A
Glutamine_transport_from_astrocytes	0.356767	#N/A	0.356767
SLC38A1_(ATA1)-mediated_uptake_of_neutral_amino_acids	0.356767	#N/A	0.356767
2-deoxyuridine_3-monophosphate_+_H2O_=>_2-deoxyuridine_+_orthophos	0.0551549	0.0551549	#N/A
uridine_2-monophosphate_+_H2O_=>_uridine_+_orthophosphate	0.0551549	0.0551549	#N/A
uridine_2-monophosphate_+_H2O_=>_uridine_+_orthophosphate_	0.0551549	0.0551549	#N/A
uridine_3-monophosphate_+_H2O_=>_uridine_+_orthophosphate	0.0551549	0.0551549	#N/A
Formation_of_ERCC1-XPF_heterodimeric_complex	0.991857	0.827178	0.992894
Pantothenate_is_phosphorylated_[PANK1;3;4]	0.795219	0.795219	#N/A
Pantothenate_is_phosphorylated_[PANK2]	0.795219	0.795219	#N/A
tBID_activates_BAX_protein	0.409127	0.409127	#N/A
tBID_binds_to_inactive_BAX_protein	0.409127	0.409127	#N/A
alanine_+_alpha-ketoglutarate_<=>_pyruvate_+_glutamate	0.25418	0.25418	#N/A
pyruvate_+_glutamate_<=>_alanine_+_alpha-ketoglutarate	0.25418	0.25418	#N/A
Formation_of_mChREBP:mMlx_complex	0.310163	0.310163	#N/A
uracil_+_D-ribose_1-phosphate_<=>_uridine_+_orthophosphate	0.885971	#N/A	0.885971
uridine_+_orthophosphate_<=>_uracil_+_D-ribose_1-phosphate	0.885971	#N/A	0.885971
phenylalanine_+_tetrahydrobiopterin_+_O2_=>_tyrosine_+_4a-hydroxytetra	0.408282	0.694589	0.201341
Tyrosine_is_hydroxylated_to_dopa	0.408282	0.694589	0.201341
Thrombin-mediated_activation_of_PAR1	0.114596	0.114596	#N/A
Phosphorylation_and_activation_of_CHK2_by_ATM	0.940896	0.883587	0.844853
thioredoxin,_oxidized_(nucleus)_<=>_thioredoxin,_oxidized_(cytosol)	0.804771	0.365074	0.940432
thioredoxin,_reduced_(cytosol)_<=>_thioredoxin,_reduced_(nucleus)	0.804771	0.365074	0.940432
Translocation_of_caspase-3_to_the_nucleus	0.137643	0.137643	#N/A

pro-factor_VII,_uncarboxylated_+_10_CO2_+_10_O2_+_10_vitamin_K_hydr	0.719136	0.568495	0.741299
creatine_+_ATP_=>_phosphocreatine_+_ADP_[CK_octamer]	0.699866	0.246533	0.923041
ATP_+_Acetate_+_CoA_<=>_AMP_+_Pyrophosphate_+_Acetyl-CoA	0.220808	0.220808	#N/A
Interaction_of_tBID_with_BCL-xl	0.591535	0.591535	#N/A
Excision_of__the_abasic_sugar_phosphate_(dRP)_residue_at_the_strand_b	0.55097	0.605014	0.465958
POL_Beta_mediated_incorporation_of_the_first_replacement_nucleotide	0.55097	0.605014	0.465958
Recruitment_of_POL_Beta_to_the_AP_site	0.55097	0.605014	0.465958
alpha-secretase_cleaves_the_p75NTR_extracellular_domain	0.784612	#N/A	0.784612
inosine_5-monophosphate_(IMP)_+_NAD+_+_H2O_=>_xanthosine_5-mono	0.0688576	0.511979	0.0166765
Formation_of_TSC1:TSC2_complex	0.762606	0.762606	#N/A
1,3-bisphospho-D-glycerate_+_NADH_+_H+_<=>_D-glyceraldehyde_3-phosp	0.887982	0.768576	0.837424
D-glyceraldehyde_3-phosphate_+_orthophosphate_+_NAD+_<=>_1,3-bisph	0.887982	0.768576	0.837424
JAM-B_binds_JAM-C	0.341758	0.113948	0.735507
pro-GAS6,_uncarboxylated_+_11_CO2_+_11_O2_+_11_vitamin_K_hydroqui	0.350747	0.117188	0.741299
Phosphorylation_of_mpChREBP_(Thr_666)_at_Ser(196)_by_mPKA	0.617493	0.617493	#N/A
Phosphorylation_of_mChREBP_at_Thr_(666)_residue_by_mPKA	0.617493	0.617493	#N/A
EIF5A(Dhp)_+_O2_=>_EIF5A(Hyp)	0.63315	#N/A	0.63315
10-formylTHF_polyglutamate_<=>_5,10-methenylTHF_polyglutamate_+_H2	0.630388	0.630388	#N/A
5,10-methenylTHF_polyglutamate_+_H2O_<=>_10-formylTHF_polyglutamat	0.630388	0.630388	#N/A
5,10-methenylTHF_polyglutamate_+_NADPH_+_H+_<=>_5,10-methyleneTH	0.630388	0.630388	#N/A
5,10-methyleneTHF_polyglutamate_+_NADP+_<=>_5,10-methenylTHF_poly	0.630388	0.630388	#N/A
THF_polyglutamate_+_formate_+_ATP_=>_10-formylTHF_polyglutamate_+_	0.630388	0.630388	#N/A
Interaction_of_BIM_with_BCL2_	0.654398	0.246994	0.893469
Cholesterol_translocates_to_the_inner_mitochondrial_membrane	0.805853	#N/A	0.805853
DNA_polymerase_epsilon_binds_at_the_origin	0.586086	0.353817	0.75246
Formation_of_DSIF_complex_	0.923387	0.836293	0.850566
Oligomerization_of_CARMA1	0.820104	0.633982	0.829344
Translocation_of_CARMA1_to_Plasma_membrane	0.820104	0.633982	0.829344
ornithine_=>_putrescine_+_CO2	0.389427	#N/A	0.389427
Phosphopantetheine_conjugation_of_the_ACP_domain_of_FAS	0.284667	0.284667	#N/A
Interaction_of_PUMA_and_Bcl-2	0.937463	#N/A	0.937463
Spermidine__=>_N-acetylated_spermidine	0.680915	#N/A	0.680915
Spermine__=>_N-acetylated_spermine	0.680915	#N/A	0.680915
oxaloacetate_+_GTP_=>_phosphoenolpyruvate_+_GDP_+_CO2_[cytosol]	0.459843	0.610386	0.336167
oxaloacetate_+_GTP_=>_phosphoenolpyruvate_+_GDP_+_CO2_[mitochond	0.459843	0.610386	0.336167
Interaction_of_NOXA_and_Bcl-2	0.198435	#N/A	0.198435
Digestion_of_branched_starch_(amylopectin)_by_extracellular_amylase	0.228348	#N/A	0.228348
Digestion_of_linear_starch_(amylose)_by_extracellular_amylase	0.228348	#N/A	0.228348
Inactivation_of_APC/C_via_CDC20_sequestration	0.162737	0.644838	0.0391121
TRAIL:TRAIL_receptor-2_Trimer_Binds_FADD	0.255161	#N/A	0.255161
FASL_binds_FAS_Receptor	0.217572	0.880197	0.0113343
Trimerization_of_the_FASL:FAS_receptor_complex	0.217572	0.880197	0.0113343
SLC7A9-mediated_exchange_of_extracellular_arginine,_lysine,_or_cystine_f	0.775516	#N/A	0.775516
pro-protein_S,_uncarboxylated_+_11_CO2_+_11_O2_+_11_vitamin_K_hydr	0.799421	#N/A	0.799421
MAD2_associates_with_the_Mad1_kinetochore_complex	0.327475	#N/A	0.327475
MAD2_converted__to_an_inhibitory_state_via_interaction_with_Mad1	0.327475	#N/A	0.327475
Release_of_activated_MAD2_from_kinetochores	0.327475	#N/A	0.327475

Binding_of_Grb2_to_Gab1	0.21818	0.146848	0.480047
Interaction_of_APE1_with_DNA_ligase_I	0.306336	0.264303	0.465958
Binding_of_Pol_zeta_to_lesioned_DNA_template	0.0434391	0.240368	0.0432181
Elongation_by_Pol_zeta_complex	0.0434391	0.240368	0.0432181
Formation_of_Pol_zeta_complex	0.0434391	0.240368	0.0432181
Phosphorylation_of_MDM2_at_serine-395_by_ATM_kinase	0.700879	#N/A	0.700879
Association_of_DFF40_with_DFF45	0.159878	0.0749333	0.51349
Cleaved_fragments_of_DFF45_dissociate_from__DFF40	0.159878	0.0749333	0.51349
Pannexin_1/Pannexin2_mediated_neuronal_gap_junction_communication	0.0268714	0.0268714	#N/A
SLC7A11-mediated_exchange_of_extracellular_cysteine_and_cytosolic_glut	0.721602	0.919722	0.283341
Formation_of_cap_binding_complex_(CBC)	0.858197	#N/A	0.858197
NRIF_binds_to_p75NTR	0.610706	#N/A	0.610706
NGF_homodimer_binds_to_p75NTR	0.610706	#N/A	0.610706
Interaction_of_BAD_with_Bcl-2	0.671263	0.268051	0.893469
Release_of_Cytochrome_c_from_mitochondria	0.355954	0.342165	0.454046
Phosphorylation_of_Cdc25A_at_Ser-123_in_response_to_DNA_damage	0.450246	0.355452	0.576837
Translocation_of_Cdc25_to_the_nucleus	0.450246	0.355452	0.576837
Translocation_of_Cdc25B_to_the_cytoplasm	0.450246	0.355452	0.576837
glycine_+_O2_=>_glyoxylate_+_H2O2_+_NH4+	0.362425	0.362425	#N/A
TNF_Binds_TNF-R1	0.974276	0.974276	#N/A
Association_of_Ku_heterodimer_with_ends_of_DNA_double-strand_break	0.932462	#N/A	0.932462
Akt_phosphorylates_Foxo1_in_the_presence_of_IRS2	0.348644	0.374481	0.409114
Tryptophan_is_hydroxylated	0.425615	0.66674	0.243182
pregnenolone_+_PAPS_=>_pregnenolone_sulfate_+_PAP	0.416264	#N/A	0.416264
Phosphorylation_of__BRCA1_at_multiple_sites_by_ATM	0.583623	#N/A	0.583623
Reducing_equivalents_from_beta-oxidation_of_fatty_acids_transfer_to_ETF	0.162201	0.162201	#N/A
Phosphorylation_of_p53_at_ser-15_by_ATM_kinase	0.990716	#N/A	0.990716
Microprocessor_complex_cleaves_pri-miRNA_to_pre-miRNA	0.540454	0.761486	0.285127
CXADR_binds_to_AMICA1	0.924618	#N/A	0.924618
Nectin_2_binds_CD226	0.265049	0.128529	0.597148
pyrophosphate_+_H2O_=>_2_orthophosphate	0.231432	0.231432	#N/A
estrone_+_PAPS_=>_estrone_3-sulfate_+_PAP	0.905191	#N/A	0.905191
Phosphorylation_of_MDC1/NFBD1_by_ATM_(within__2_c-term_BRCT_dom	0.67354	#N/A	0.67354
2-amino-3-carboxymuconate_semialdehyde_=>_2-aminomuconate_semiald	0.802004	#N/A	0.802004
Loading_of_dopamine_into_synaptic_veiscles	0.578918	0.643072	0.465965
loading_of_Serotonin_in_synaptic_vesicles	0.578918	0.643072	0.465965
CD48_binds_CD244	0.520855	0.572121	0.456998
Displacement_of_hNTH1_glycosylase_by_APE1_at_the_AP_site	0.697407	0.792707	0.465958
Formation_of_FACT_complex	0.893088	0.671824	0.905181
Epithelial_cadherin_binds_to_KLRG1_in_mice	0.264148	#N/A	0.264148
adenosine_5-monophosphate_(AMP)_+_H2O_=>_adenosine_+_orthophosp	0.761176	#N/A	0.761176
Connexin_45/Connexin_36_mediated_neuronal_gap_junction_communicati	0.811586	0.811586	#N/A
Receptor_CXCR6_binds_CXCL16_ligand	0.637641	0.281268	0.859173
palmitoyl-CoA_+_2_NADPH_+_2_H+_=>_hexadecanol_+_2_NADP+_[FAR1]	0.0106022	#N/A	0.0106022
p75NTR_interacts_with_the_NOGO_receptor	0.919059	0.941812	0.657777
Association_of_Emi1_with_Cdh1	0.24511	0.24511	#N/A
Phosphorylation_of__NBS1_by_ATM	0.214268	#N/A	0.214268

Association_of_Emi1_with_Cdc20	0.759501	0.759501	#N/A
Interaction_between_FEN1_and_PCNA	0.323455	0.323455	#N/A
Receptor_CCR6_binds_CCL20_ligand	0.316464	#N/A	0.316464
Dimerisation_of_Activated_Protein_1_(AP-1)_heterodimer	0.183823	0.277723	0.247105
Formation_of_Elongin_BC_complex	0.915757	0.737587	0.904903
Displacement_of_MBD4_glycosylase_by_APE1_at_the_AP_site	0.784128	#N/A	0.784128
Association_of_RAD51_with_RAD52:DNA_double-strand_break_ends	0.036185	#N/A	0.036185
Interaction_of_BIM_with_BCL-xl	0.12909	0.12909	#N/A
CRTAM_binds_to_NECL2	0.0984803	0.128529	0.24506
Interaction_of_PUMA_and_Bcl-XL	0.502352	0.18035	0.821795
Transport_of_palmitoyl_carnitine_into_mitochondria	0.239207	0.239207	#N/A
Formation_of_SL1	0.225474	0.313492	0.281142
Interaction_of_NOXA_and_Bcl-XL	0.00877084	0.18035	0.007291
Human_chorionic_gonadotropin_(hCG)_is_a_heterodimer	0.436294	0.405311	0.50513
Luteinizing_hormone_is_a_heterodimer	0.436294	0.405311	0.50513
Cdt1_associates_with_geminin	0.397732	0.397732	#N/A
Thyrotropin_is_a_heterodimer	0.619055	0.661003	0.50513
CyP60_chaperones_Basigin	0.288478	0.288478	#N/A
Isopentenyl_pyrophosphate_rearranges_to_dimethylallyl_pyrophosphate	0.898042	#N/A	0.898042
L-glutamate_gamma-semialdehyde_+_NAD+_=>_glutamate_+_NADH_+_H+	0.0519766	0.0854901	0.17648
Phosphorylation_and_activation_of_Chk1_by_ATM_kinase	0.911325	0.813689	0.844853
ATP_+_D-fructose_6-phosphate_=>_ADP_+_D-fructose_2,6-bisphosphate_[b	0.731732	0.787694	0.529832
D-fructose_2,6-bisphosphate_+_H2O_=>_D-fructose_6-phosphate_+_orthop	0.731732	0.787694	0.529832
Dephosphorylation_of_PF2K-Pase_by_PP2A_complex	0.731732	0.787694	0.529832
Fructose_2,6-bisphosphate_is_hydrolyzed_to_form_fructose-6-phosphate_a	0.731732	0.787694	0.529832
glutaredoxin_(oxidized)_+_glutathione_(reduced)_=>_glutaredoxin_(reduce	0.0767727	#N/A	0.0767727
Association_of_RAD51_with_BRCA2	0.680935	0.803177	0.425233
The_orphan_QRFP_receptor_can_bind_neuropeptide_QRFP	0.978242	#N/A	0.978242
Interaction__of_BAD_with_BCL-xl___	0.139066	0.139066	#N/A
D2-like_dopamine_receptors_bind_to_dopamine	0.878785	0.812393	0.77787
Arachidonic_acid_oxidised_to_PGG2	0.636825	0.795848	0.369785
Peroxidative_reduction_of_PGG2_to_PGH2	0.636825	0.795848	0.369785
Basigin_interacts_with_CD43	0.190661	0.100171	0.517255
Interaction_of_APE1_with_FEN1	0.568853	0.629459	0.465958
Corticotropin_cleavage_from_POMC	0.512975	#N/A	0.512975
Tachykinin_receptor_2_(NK2)_binds_to_nuerokinin_A	0.437113	#N/A	0.437113
asparagine_+_tRNA(Asn)_+_ATP_=>_Asn-tRNA(Asn)_+_AMP_+_pyrophosph	0.701453	0.755912	0.521386
lysophosphatidic_acid_+_fatty_acyl_CoA__=>_phosphatidic_acid_+_CoA_(3)	0.134	0.110284	0.366755
Prolactin-releasing_hormone_receptor_binds_PRH	0.56329	0.768867	0.304986
Recruitment_and_activation_of_Chk1	0.67534	0.67534	#N/A
glucosaminyl-acyl-PI_+_dolichol_phosphate_D-mannose_->_mannose(al1-4)	0.182773	#N/A	0.182773
Tachykinin_receptor_1_(NK1)_binds_to_substance_P	0.311206	#N/A	0.311206
threonine_+_tRNA(Thr)_+_ATP_=>_Thr-tRNA(Thr)_+_AMP_+_pyrophosphate	0.531803	0.1645	0.861873
Tachykinin_receptor_3_(NK3)_binds_to_neurokinin_B	0.181994	0.438913	0.129392
Orexin_2_receptor_can_bind_orexin-B_neuropeptide	0.700759	#N/A	0.700759
IRAK1-P(n)_combines_with_TRAF6	0.839339	0.839339	#N/A
Alpha-2_adrenoceptors_bind_catecholamines	0.897502	0.87526	0.738708

Exportin_complex_translocates_pre-miRNA_to_cytosol	0.815882	0.278925	0.968377
Exportin-5_recognizes_3_overhang_of_pre-miRNA_	0.815882	0.278925	0.968377
Nociceptin_receptor_binds_to_nociceptin	0.421948	0.421948	#N/A
D-galactose_1-phosphate_+_UDP-glucose_<=>_D-glucose_1-phosphate_+_U	0.223344	0.223344	#N/A
Binding_of_ADAR2_homodimer_to_dsRNA_duplex	0.115194	0.146381	0.260128
Deamination_at_C6_position_of_adenosine_in_Editosome_(ADAR2)	0.115194	0.146381	0.260128
Formation_of_ADAR2__homodimer	0.115194	0.146381	0.260128
Displacement_of_MYH_glycosylase_by_APE1_at_the_AP_site	0.469273	#N/A	0.469273
Follitropin_is_a_heterodimer	0.761678	0.839591	0.50513
Two_NPFF_receptors_can_bind_neuropeptide_FF	0.461152	0.94368	0.042318
SLC38A5-mediated_uptake_of_glutamine,_histidine,_asparagine,_and_serin	0.922058	0.867842	0.812993
Displacement_of_TDG_glycosylase_by_APE1_at_the_AP_site	0.0360536	#N/A	0.0360536
SLC6A15-mediated_amino_acid_uptake	0.785083	#N/A	0.785083
histidine_+_tRNA(His)_+_ATP_=>_His-tRNA(His)_+_AMP_+_pyrophosphate	0.603635	0.596115	0.550885
Orexin_1_receptor_can_bind_orexin-A_neuropeptide	0.793689	#N/A	0.793689
tryptophan_+_tRNA(Trp)_+_ATP_=>_Trp-tRNA(Trp)_+_AMP_+_pyrophospha	0.129302	0.639043	0.0254399
Vasopressin_receptor_type_2_bind_vasopressin	0.481457	0.378419	0.596305
Displacement_of_hSMUG1_glycosylase_by_APE1_at_the_AP_site	0.373519	0.355499	0.465958
Grb2_binds_Sos	0.18624	0.146848	0.416676
SLC6A18-mediated_glycine_uptake	0.905294	0.825386	0.820772
SLC6A19-mediated_uptake_of_neutral_amino_acids	0.905294	0.825386	0.820772
Displacement_of_hOGG1_glycosylase_by_APE1_at_the_AP_site	0.453589	#N/A	0.453589
NPC1L1_inactivation_by_ezetimibe	0.95028	#N/A	0.95028
NPC1L1-mediated_cholesterol_uptake	0.95028	#N/A	0.95028
D1-like_dopamine_receptors_bind_to_dopamine	0.595177	#N/A	0.595177
alpha-methylbutyryl-CoA_+_FAD_=>_tiglyl-CoA_+_FADH2	0.504704	0.0230826	0.977806
Butanoyl-CoA+FAD<=>Crotonoyl-CoA+FADH2	0.504704	0.0230826	0.977806
Hexanoyl-CoA+FAD<=>trans-Hex-2-enoyl-CoA+FADH2	0.504704	0.0230826	0.977806
Displacement_of__MPG_glycosylase_by_APE1_at_the_AP_site	0.441071	0.450576	0.465958
Digestion_of_triacylglycerols_by_extracellular_pancreatic_lipase-related_pr	0.133183	#N/A	0.133183
mannose_(a1-2)_mannose_(a1-6)_(ethanolamineP)_mannose_(a1-4)_glucos	0.631897	0.622588	0.564998
Receptor_CXC1_binds_to_the_ligands_lymphotactin_and_cytokine_SCM-1_	0.929199	#N/A	0.929199
H3_and_H4_receptors_binds_histamine	0.748174	0.722534	0.638536
CD40_interacting_with_CD40L	0.818447	#N/A	0.818447
serine_+_tRNA(Ser)_+_ATP_=>_Ser-tRNA(Ser)_+_AMP_+_pyrophosphate	0.467834	0.467834	#N/A
Conversion_of_Ran-GDP_to_Ran-GTP	0.200193	0.200193	#N/A
alanine_+_tRNA(Ala)_+_ATP_=>_Ala-tRNA(Ala)_+_AMP_+_pyrophosphate	0.682441	0.682441	#N/A
5Beta-cholestan-7alpha,12alpha,24(S)-triol-3-one_is_reduced_to_5beta-cho	0.623146	0.623146	#N/A
5Beta-cholestan-7alpha,12alpha,27-triol-3-one_is_reduced_to_5beta-choles	0.623146	0.623146	#N/A
5beta-cholestan-7alpha,24(S)-diol-3-one_is_reduced_to_5beta-cholestan-3a	0.623146	0.623146	#N/A
5beta-cholestan-7alpha,27-diol-3-one_is_reduced_to_5beta-cholestan-3alpl	0.623146	0.623146	#N/A
5beta-cholestan-7alpha-ol-3-one_is_reduced_to_5beta-cholestan-3alpha,_7	0.623146	0.623146	#N/A
5Beta-cholesten-7alpha,_12alpha-diol-3-one_is_reduced_to_5beta-cholesta	0.623146	0.623146	#N/A
ATP_+_beta-D-fructose_=>_ADP_+_D-fructose_1-phosphate	0.00348012	0.00348012	#N/A
Transport_of_Choline	0.329377	#N/A	0.329377
acetoacetate+succinyl-CoA_<=>_acetoacetyl-CoA+succinate	0.0910915	0.0910915	#N/A
Spermine_is_oxidized_to_spermidine	0.401464	0.401464	#N/A

Pro-prothrombin_is_transported_from_the_endoplasmic_reticulum_to_the_	0.277238	0.277238	#N/A
benzoyl-CoA_+_glycine_=>_benzoyl_glycine_(hippuric_acid)_+_Coenzyme_A	0.146369	0.146369	#N/A
salicylate-CoA_+_glycine_=>_salicyluric_acid_+_Coenzyme_A	0.146369	0.146369	#N/A
p-hydroxyphenylpyruvate_+_O2_=>_homogentisate_+_CO2	0.149151	#N/A	0.149151
tryptophan_+_O2_=>_N-formylkynurenine_[TDO]	0.296163	#N/A	0.296163
Oxidation_of_cortisol_to_yield_cortisone	0.296462	#N/A	0.296462
Exocytosis_of_kininogen	0.539684	#N/A	0.539684
3-hydroxyanthranilate_+_O2_=>_2-amino-3-carboxymuconate_semialdehyd	0.601158	#N/A	0.601158
Release_of_Titin	0.692904	#N/A	0.692904
3-hydroxykynurenine_+_H2O_=>_3-hydroxyanthranilate_+_alanine	0.665296	#N/A	0.665296
antithrombin_III_+_heparin_->_antithrombin_III:heparin	0.354181	#N/A	0.354181
4a-hydroxytetrahydrobiopterin_=>_q-dihydrobiopterin_+_H2O	0.733115	0.733115	#N/A
UTP_+_D-glucose_1-phosphate_<=>_pyrophosphate_+_UDP-glucose_[liver]	0.406946	#N/A	0.406946
UTP_+_D-glucose_1-phosphate_<=>_pyrophosphate_+_UDP-glucose_[musc	0.406946	#N/A	0.406946
Surface_deployment_of_LAMP2	0.906734	0.906734	#N/A
Exocytosis_of_histidine_rich_glycoprotein	0.609158	#N/A	0.609158
Pro-factor_IX_is_transported_from_the_endoplasmic_reticulum_to_the_Go	0.814505	#N/A	0.814505
lysine_+_alpha-ketoglutarate_+NADPH_+_H+_=>_saccharopine_+_NADP+_+	0.353932	#N/A	0.353932
saccharopine_+_NAD+_+_H2O_=>_alpha-aminoadipic_semialdehyde_+_glut	0.353932	#N/A	0.353932
Chenodeoxycholoyl_CoA_reacts_with_glycine_or_taurine_to_form_glycoche	0.262301	#N/A	0.262301
Choloyl_CoA_reacts_with_glycine_or_taurine_to_form_glycocholate_or_tau	0.262301	#N/A	0.262301
Cytosolic_chenodeoxycholoyl-CoA_or_choloyl-CoA_are_conjugated_with_gl	0.262301	#N/A	0.262301
Exocytosis_of_osteonectin	0.993218	#N/A	0.993218
Ethanol_+_NAD+_<=>_Acetaldehyde_+_NADH_+_H+_[class_II,_pi/pi]	0.679324	#N/A	0.679324
Apolipoprotein_A-I_binds_membrane-associated_cholesterol_and_phospho	0.0817583	0.0817583	#N/A
Discoidal_HDL_binds_membrane-associated_free_cholesterol	0.0817583	0.0817583	#N/A
pre-beta_HDL_binds_membrane-associated_cholesterol_and_phospholipid_	0.0817583	0.0817583	#N/A
Release_of_Apolipoprotein_A-I_	0.0817583	0.0817583	#N/A
Spherical_HDL_binds_membrane-associated_free_cholesterol_and_phospho	0.0817583	0.0817583	#N/A
7-hydroxycoumarin_can_form_an_O-glucuronide	0.300233	#N/A	0.300233
Formation_of_N-glucuronides	0.300233	#N/A	0.300233
Formation_of_O-glucuronides	0.300233	#N/A	0.300233
carbamoyl_phosphate_+_ornithine_=>_citrulline_+_orthophosphate	0.864359	0.864359	#N/A
4-Cholesten-7alpha,24(S)-diol-3-one_is_12alpha-hydroxylated_to_4-cholest	0.624304	#N/A	0.624304
4-Cholesten-7alpha,27-diol-3-one_is_12alpha-hydroxylated_to_4-Cholesten-	0.624304	#N/A	0.624304
4-Cholesten-7alpha-ol-3-one_is_12alpha-hydroxylated_to_7-alpha,12-alpha-	0.624304	#N/A	0.624304
HMG_CoA_=>_acetoacetic_acid+_acetyl_CoA	0.460075	0.460075	#N/A
4,8-dimethylnonanoyl-CoA_+_carnitine_=>_4,8-dimethylnonanoylcarnitine_	0.680081	0.680081	#N/A
6-mercaptopurine_can_be_S-methylated	0.0151192	#N/A	0.0151192
phytanoyl-CoA_+_2-oxoglutarate_+_O2_=>_2-hydroxyphytanoyl-CoA_+_suc	0.492037	0.492037	#N/A
Connexin_oligomerization_in_endoplasmic_reticulum_membrane	0.948766	0.948766	#N/A
Exocytosis_of_Fructose-bisphosphate_aldolase	0.280678	0.280678	#N/A
Exocytosis_of_Coagulation_factor_XIIIA	0.296712	#N/A	0.296712
Diiodinated_tyrosine_can_be_deiodinated	0.840625	#N/A	0.840625
Monoiodinated_tyrosine_can_be_deiodinated	0.840625	#N/A	0.840625
n_UDP-glucose_+_glycogenin-1_=>_n_UDP_+_{(1,4)-alpha-D-glucosyl}n_glyc	0.189628	0.189628	#N/A
n_UDP-glucose_+_glycogenin-2_=>_n_UDP_+_{(1,4)-alpha-D-glucosyl}n_glyc	0.189628	0.189628	#N/A

Exocytosis_of_plasminogen	0.858209	0.858209	#N/A
Calcidiol_binds_to_DBP_in_the_bloodstream_and_is_delivered_to_the_kidr	0.338821	#N/A	0.338821
DBP:Calcidiol_is_sequestered_by_cubilin_on_the_cell_surface	0.338821	#N/A	0.338821
DBP:Calcidiol_translocates_into_lysosomes	0.338821	#N/A	0.338821
Vitamin_D3_translocates_into_the_cytosol	0.338821	#N/A	0.338821
Vitamin_D3_translocates_to_the_blood_serum_and_is_transported_to_the_	0.338821	#N/A	0.338821
gamma-butyrobetaine_semialdehyde_+_NAD+_=>_gamma-butyrobetaine_+	0.287758	#N/A	0.287758
Exocytosis_of_Insulin-like_growth_factor	0.00799824	#N/A	0.00799824
S-adenoylhomocysteine_is_hydrolyzed	0.268904	0.268904	#N/A
homogentisate_+_O2_=>_maleylacetoacetate	0.32606	0.32606	#N/A
Zymosterol_is_isomerized_to_cholesta-7,24-dien-3beta-ol	0.516734	0.516734	#N/A
CYP2F1_dehydrogenates_3-methylindole	0.788894	#N/A	0.788894
3,4-dihydroxybenzoate_can_be_O-methylated	0.147109	0.147109	#N/A
Methylation_of_3,4-dihydroxypheylacetic_acid_to_homovanillic_acid	0.147109	0.147109	#N/A
methylation_of_Dopamine_to_form_3-Methoxytyramine	0.147109	0.147109	#N/A
2_ATP_+_NH4+_+_HCO3-_=>_2_ADP_+_orthophosphate_+_carbamoyl_pho	0.670919	#N/A	0.670919
carbamoyl_phosphate_+_L-aspartate_<=>_N-carbamoyl_L-aspartate_+_orth	0.670919	#N/A	0.670919
L-glutamine_+_2_ATP_+_HCO3-_+_H2O_=>_carbamoyl_phosphate_+_L-glut	0.670919	#N/A	0.670919
N-carbamoyl_L-aspartate_+_H+_<=>_(S)-dihydroorotate_+_H2O	0.670919	#N/A	0.670919
A_phospho-ribosyl_group_is_added_to_quinolinate	0.901386	0.901386	#N/A
Pro-factor_X_is_transported_from_the_endoplasmic_reticulum_to_the_Gol	0.773035	0.773035	#N/A
Surface_deployment_of_GpIV	0.878397	0.878397	#N/A
kynurenine_+_O2_+_NADPH_+_H+_=>_3-hydroxykynurenine_+_NADP+_+_I	0.0342024	#N/A	0.0342024
3,7,24THCA_is_conjugated_with_Coenzyme_A_(SLC27A5_BACS)	0.625811	#N/A	0.625811
Cytosolic_cholate_and_chenodeoxycholate_are_conjugated_with_Coenzym	0.625811	#N/A	0.625811
DHCA_is_conjugated_with_Coenzyme_A_(SLC27A5_BACS)	0.625811	#N/A	0.625811
TetraHCA_is_conjugated_with_Coenzyme_A_(SLC27A5_BACS)	0.625811	#N/A	0.625811
THCA_is_conjugated_with_Coenzyme_A_(SLC27A5_BACS)	0.625811	#N/A	0.625811
SLC38A4_(ATA3)-mediated_uptake_of_arginine_and_lysine	0.822072	#N/A	0.822072
Exocytosis_of_complement_factor_D	0.014667	0.014667	#N/A
phosphoenolpyruvate_[mitochondrial_matrix]_+_citrate_[cytosol]_=>_phos	0.580235	#N/A	0.580235
Acetaminophen_oxidised_to_N-acetylbenzoquinoneimine_(NAPQI)	0.119685	#N/A	0.119685
Benzene_is_hydroxylated_to_phenol	0.119685	#N/A	0.119685
Dehalogenation_of_carbon_tetrachloride_to_form_a_free_radical	0.119685	#N/A	0.119685
Dehalogenation_of_the_poly-halogenated_hydrocarbon_Halothane_to_forr	0.119685	#N/A	0.119685
MEOS_oxidizes_ethanol_to_acetaldehyde	0.119685	#N/A	0.119685
Vinyl_chloride_is_oxidized_to_2-Chloroethylene_oxide	0.119685	#N/A	0.119685
beta-ureidoisobutyrate_+_H2O_=>_beta-aminoisobutyrate_+_NH4+_+_CO2	0.67846	0.67846	#N/A
beta-ureidopropionate_+_H2O_=>_beta-alanine_+_NH4+_+_CO2	0.67846	0.67846	#N/A
Ethanol_+_NAD+_<=>_Acetaldehyde_+_NADH_+_H+_[class_I,_alpha/alpha]	0.470778	#N/A	0.470778
aspartate_+_glutamine_+_ATP_<=>_asparagine_+_glutamate_+_AMP_+_py	0.0682821	#N/A	0.0682821
5,10-methyleneTHF_polyglutamate_+_glycine_<=>_tetrahydrofolate_polyglu	0.670523	0.670523	#N/A
beta-hydroxy-trimethyllysine_=>_gamma-butyrobetaine_aldehyde_+_glycine	0.670523	0.670523	#N/A
Tetrahydrofolate_polyglutamate_(THF_polyglutamate)_+_serine_<=>_5,10-r	0.670523	0.670523	#N/A
25-Hydroxylation_of_vitamin_D3_in_liver	0.246334	#N/A	0.246334
D-beta_hydroxybutyrate+NAD+_<=>_acetoacetate+NADH+H+	0.84153	0.84153	#N/A
Reduction_of_Acetoacetate_to_beta-Hydroxybutyrate_	0.84153	0.84153	#N/A

glutamate_+_acetyl_CoA_=>_N-acetyl_glutamate_+_CoA	0.978661	0.978661	#N/A
Exocytosis_of_alpha_2_antiplasmin	0.679033	0.679033	#N/A
fumarylacetoacetate_=>_fumarate_+_acetoacetate	0.498331	0.498331	#N/A
Conversion_of_Diacylglycerol_to_Triacylglycerol	0.862645	0.862645	#N/A
FMN_can_be_hydrolyzed_back_to_riboflavin	0.877459	#N/A	0.877459
Cholesta-7,24-dien-3beta-ol_is_desaturated_to_form_cholesta-5,7,24-trien-	0.912946	#N/A	0.912946
2_H2O2_=>_O2_+_2_H2O	0.853823	0.853823	#N/A
2-hydroxyphytanoyl-CoA_=>_pristanal_+_formyl-CoA	0.870158	#N/A	0.870158
Binding_of_ACF_to_stem-looped_RNA	0.900881	#N/A	0.900881
Pro-protein_Z_is_transported_from_the_endoplasmic_reticulum_to_the_G	0.949598	0.949598	#N/A
dc-Adenosyl_methionine_+_Spermidine_=>_Spermine_+_5-methylthioaden	0.385253	#N/A	0.385253
glutaryl-CoA_+_FAD_=>_crotonyl-CoA_+_FADH2_+_CO2	0.393422	0.393422	#N/A
Exocytosis_of_low-affinity_platelet_factor_IV_	0.467961	0.467961	#N/A
Exocytosis_of_Platelet_factor_4	0.467961	0.467961	#N/A
Formation_of_Caspase-8_dimer	0.133604	#N/A	0.133604
Translocation_of_active_caspase-8_to_the_mitochondrial_membrane	0.133604	#N/A	0.133604
2-acylglycerol_+_H2O_->_glycerol_+_fatty_acid	0.309753	0.309753	#N/A
alpha-aminoadipate_+_alpha-ketoglutarate_=>_alpha-ketoadipate_+_glutan	0.546515	#N/A	0.546515
malate_[mitochondrial_matrix]_+_orthophosphate_[cytosol]_<=>_malate_[	0.69765	0.69765	#N/A
Isomerization_of_(2R)-pristanoyl-CoA_to_(2S)-pristanoyl-CoA	0.00185265	0.00185265	#N/A
Isomerization_of_25(R)_DHCA-CoA_to_25(S)_DHCA-CoA	0.00185265	0.00185265	#N/A
Isomerization_of_25(R)_TetraHCA-CoA_to_(24R,_25R)_3alpha,7alpha,12alp	0.00185265	0.00185265	#N/A
Isomerization_of_25(R)_THCA-CoA_to_25(S)_THCA-CoA	0.00185265	0.00185265	#N/A
Isomerization_of_3,7,24THCA-CoA_to_(24R,_25R)_3alpha,7alpha,24-trihydr	0.00185265	0.00185265	#N/A
Pro-protein_C_is_transported_from_the_endoplasmic_reticulum_to_the_G	0.377498	0.377498	#N/A
Reduction_of_desmosterol_to_cholesterol	0.633959	#N/A	0.633959
N-formiminoglutamate_+_tetrahydrofolate_=>_glutamate_+_5-formiminote	0.603477	#N/A	0.603477
histidine_=>_urocanate_+_NH4+	0.669015	#N/A	0.669015
5-phosphoribosyl-5-aminoimidazole-4-N-succinocarboxamide_(SAICAR)_<=>	0.19328	0.19328	#N/A
adenylosuccinate_=>_adenosine_5-monophosphate_+_fumarate	0.19328	0.19328	#N/A
D-ribulose_5-phosphate_<=>_xylulose_5-phosphate	0.702891	0.702891	#N/A
xylulose_5-phosphate_<=>_D-ribulose_5-phosphate	0.702891	0.702891	#N/A
Fumarate_+_H2O_<=>_(S)-Malate	0.262019	0.262019	#N/A
CD84_homodimerises	0.774299	0.774299	#N/A
alpha-D-glucose_(extracellular)_<=>_alpha-D-glucose_(cytosol)_[GLUT2]	0.501605	#N/A	0.501605
Transport_(efflux)_of_fructose,_galactose,_and_glucose_by_GLUT2	0.501605	#N/A	0.501605
25-hydroxycholesterol_is_7alpha-hydroxylated_by_CYP7B1	0.540036	#N/A	0.540036
27-hydroxycholesterol_is_7alpha-hydroxylated	0.540036	#N/A	0.540036
Guanine_+_H2O_=>_Xanthine_+_NH3	0.182408	#N/A	0.182408
PMP34-mediated_exchange_of_cytosolic_ATP_for_peroxisomal_AMP	0.80904	0.80904	#N/A
thymine_+_NADPH_+_H+_=>_5,6-dihydrothymine_+_NADP+	0.529323	#N/A	0.529323
uracil_+_NADPH_+_H+_=>_5,6-dihydrouracil_+_NADP+	0.529323	#N/A	0.529323
PS-GAP_interacts_ with_caspase-activated_PAK-2p34	0.955943	#N/A	0.955943
Interaction_of_PAK-2p34_with_PS-GAP_results_in_accumulation_of_PAK-2	0.955943	#N/A	0.955943
7alpha-hydroxycholesterol_is_oxidized_and_isomerized_to_4-cholesten-7al	0.154429	0.154429	#N/A
Cholest-5-ene-3beta,7alpha,24(S)-triol_is_oxidized_and_isomerized_to_4-ch	0.154429	0.154429	#N/A
Cholest-5-ene-3beta,7alpha,27-triol_is_oxidized_and_isomerized_to_4-chol	0.154429	0.154429	#N/A

urocanate_+_H2O_=>_4-imidazolone-5-propionate	0.576221	0.576221	#N/A
Conversion_of_Glycerol_to_Glycerol-3-phosphate	0.857625	#N/A	0.857625
2-deoxycytidine_5-monophosphate_(dCMP)_+_H2O_=>_2-deoxycytidine_+_	0.142492	#N/A	0.142492
6-phospho-D-gluconate_+_NADP+_=>_D-ribulose_5-phosphate_+_CO2_+_N	0.994397	0.994397	#N/A
tyrosine_+_alpha-ketoglutarate_=>_p-hydroxyphenylpyruvate_+_glutamate	0.94113	0.94113	#N/A
2-deoxyguanosine_+_orthophosphate_<=>_guanine_+_2-deoxy-D-ribose_1-	0.194283	#N/A	0.194283
2-deoxyinosine_+_orthophosphate_<=>_hypoxanthine_+_2-deoxy-D-ribose_	0.194283	#N/A	0.194283
guanine_+_2-deoxy-D-ribose_1-phosphate_<=>_2-deoxyguanosine_+_ortho	0.194283	#N/A	0.194283
guanine_+_D-ribose_1-phosphate_<=>_guanosine_+_orthophosphate	0.194283	#N/A	0.194283
guanosine_+_orthophosphate_<=>_guanine_+_D-ribose_1-phosphate	0.194283	#N/A	0.194283
hypoxanthine_+_2-deoxy-D-ribose_1-phosphate_<=>_2-deoxyinosine_+_ort	0.194283	#N/A	0.194283
hypoxanthine_+_D-ribose_1-phosphate_<=>_inosine_+_orthophosphate	0.194283	#N/A	0.194283
inosine_+_orthophosphate_<=>_hypoxanthine_+_D-ribose_1-phosphate	0.194283	#N/A	0.194283
Association_of_Cx43__with_ZO-1	0.238801	#N/A	0.238801
Association_of_Golgi__transport_vesicles_with_microtubules_and_transpor	0.238801	#N/A	0.238801
Budding_of_connexon-containing_transport_vesicles_from_the_Golgi	0.238801	#N/A	0.238801
Connexin_oligomerization_in_Trans-Golgi_Network_(TGN)	0.238801	#N/A	0.238801
Docking_of_connexons_into_junctional,_double-membrane_spanning_chan	0.238801	#N/A	0.238801
Insertion_of_connexons_into_the_plasma_membrane_resulting_in_the_for	0.238801	#N/A	0.238801
Transport_of_connexins_to_the_Trans-Golgi_Network_(TGN)	0.238801	#N/A	0.238801
Association_of_LBP_with_LPS	0.985979	#N/A	0.985979
glyoxylate_+_NADPH_+_H+_=>_glycolate_+_NADP+	0.328251	0.328251	#N/A
Cholesterol_is_hydroxylated_to_7alpha-hydroxycholesterol_by_CYP7A1	0.832338	#N/A	0.832338
NAD+_is_phosphorylated_to_NADP+	0.755091	#N/A	0.755091
Adenylation_of_phosphopantetheine	0.343163	0.343163	#N/A
Phosphorylation_of_dephospho-CoA_to_produce_CoA	0.343163	0.343163	#N/A
glutamine_+_H2O_=>_glutamate_+_NH4+_[kidney]	0.689826	#N/A	0.689826
L-Glutamine_conversion_to_L-Glutamate	0.689826	#N/A	0.689826
crosslinked_fibrin_multimer_+_tissue_plasminogen_activator_(one-chain)_-	0.219046	0.219046	#N/A
Exocytosis_of_EGF	0.0868948	0.0868948	#N/A
Exocytosis_of_factor_V	0.503528	#N/A	0.503528
Acetaminophen_can_form_an_O-_sulfate_conjugate	0.820545	0.820545	#N/A
Dopamine_can_form_an_O-sulfate_conjugate	0.820545	0.820545	#N/A
N-hydroxy-4-aminobiphenyl_can_form_a_sulfate_conjugate	0.820545	0.820545	#N/A
Phenol_can_form_a_sulfate_conjugate	0.820545	0.820545	#N/A
A_phosphoribosyl_group_is_added_to_nicotinate_to_form_nicotinate_mon	0.368295	0.368295	#N/A
3-ketopristanoyl-CoA_+_CoASH_=>_4,8,12-trimethyltridecanoyl-CoA_+_prop	0.941601	#N/A	0.941601
Thiolysis_of_3alpha,7alpha,12alpha-trihydroxy-5beta-cholan-24-one-CoA_yi	0.941601	#N/A	0.941601
Thiolysis_of_3alpha,7alpha-dihydroxy-5beta-cholan-24-one-CoA_yields_chei	0.941601	#N/A	0.941601
SLC38A3-mediated_uptake_of_glutamine,_histidine,_asparagine,_and_alani	0.442209	0.442209	#N/A
UDP-galactose_<=>_UDP-glucose	0.459964	0.459964	#N/A
Decarboxylation_of_5-hydroxytryptophan_forms_serotonin	0.690969	#N/A	0.690969
Dopa_is_decarboxylated_to_dopamine	0.690969	#N/A	0.690969
Addition_of_isopentenyl_pyrophosphate_to_DMAPP	0.0447348	#N/A	0.0447348
Another_isopentenyl_pyrophosphate_is_added_to_geranyl_pyrophosphate	0.0447348	#N/A	0.0447348
DHF_is_reduced_to_tetrahydrofolate_(THF)	0.562922	#N/A	0.562922
Folate_is_reduced_to_dihydrofolate_(DHF)	0.562922	#N/A	0.562922

Exocytosis_of_Metalloproteinase_inhibitor_1	0.887702	0.887702	#N/A
ornithine_(cytosolic)_+_citrulline_(mitochondrial)_=>_ornithine_(mitochond	0.490631	0.490631	#N/A
Microtubule-independent_trafficking_of_connexons_to_the_plasma_memb	0.709122	#N/A	0.709122
N-acetylspermidine_is_oxidized_to_putrescine	0.24444	0.24444	#N/A
N-acetylspermine_is_oxidised_to_spermidine	0.24444	0.24444	#N/A
Exocytosis_of_plasminogen_activator_inhibitor	0.954792	0.954792	#N/A
2_glutathione,_reduced_+_H2O2_=>_glutathione,_oxidized_+_2_H2O	0.221834	0.221834	#N/A
Release_of_Cofilin-1	0.973505	0.973505	#N/A
beta-estradiol_+_PAPS_=>_beta-estradiol_3-sulfate_+_PAP	0.588118	#N/A	0.588118
CYP2U1_can_omega-hydroxylate_arachidonate	0.918248	0.918248	#N/A
Ethylene_is_oxidized_to_Ethylene_oxide_by_CYP1A1	0.918248	0.918248	#N/A
Hydroxylation_of_pregnenolone_to_form_17alpha-hydroxypregnenolone	0.918248	0.918248	#N/A
Hydroxylation_of_progesterone_to_form_17alpha-hydroxyprogesterone	0.918248	0.918248	#N/A
N-atom_dealkylation_of_caffeine	0.918248	0.918248	#N/A
N-hydroxylation_of_4-aminobiphenyl	0.918248	0.918248	#N/A
S-atom_dealkylation_of_6-methylmercaptopurine	0.918248	0.918248	#N/A
Side_chain_cleavage_of_17alpha-hydroxyprenenolone_to_yield_DHA	0.918248	0.918248	#N/A
Side_chain_cleavage_of_17alpha-hydroxyprogesterone_to_form_4-Androst	0.918248	0.918248	#N/A
Exocytosis_of_albumin	0.382317	#N/A	0.382317
Serum_albumin_binds_2-lysophosphatidylcholine	0.382317	#N/A	0.382317
adenosine_+_ATP_=>_adenosine_5-monophosphate_(AMP)_+_ADP	0.450539	0.450539	#N/A
Secretion_and_activation_of_the_latent_large_complex_of_TGF-beta1	0.765174	0.765174	#N/A
q-dihydrobiopterin_+_NADH_+_H+_=>_tetrahydrobiopterin_+_NAD+	0.479692	0.479692	#N/A
2-oxoglutarate_[mitochondrial_matrix]_+_2-oxoadipate_[cytosol]_<=>_2-ox	0.336447	0.336447	#N/A
Putrescine_+_dc-Adenosyl_methionine__=>_Spermidine_+_5-methylthioad	0.58287	0.58287	#N/A
Arginine<=>Agmatine+CO2	0.609822	0.609822	#N/A
Acetyl-ACP(intermediate)__=>_Acetyl-synthase_	0.523851	0.523851	#N/A
Condensation_of_Malonyl-ACP_and_Acetate_on_different_monomers_of_F	0.523851	0.523851	#N/A
Conversion_of_Malonyl-CoA_to_Malonyl-ACP	0.523851	0.523851	#N/A
Dehydration_of_beta-Hydroxybutyryl-ACP_to_Crotonoyl-ACP_	0.523851	0.523851	#N/A
Formation_of_Acetyl-ACP(intermediate)	0.523851	0.523851	#N/A
Formation_of_fatty_acid_synthase_(FAS)_dimer	0.523851	0.523851	#N/A
Hydrolysis_of_Palmitoyl-ACP_to_Palmitic_Acid	0.523851	0.523851	#N/A
Reduction_of_Acetoacetyl-ACP_to_beta-Hydroxybutyryl-ACP	0.523851	0.523851	#N/A
Reduction_of_Crotonoyl-ACP_to_Butyrl-ACP	0.523851	0.523851	#N/A
Cytosolic_folate_export_across_the_plasma_membrane	0.229083	#N/A	0.229083
Extracellular_folate_import_across_the_plasma_membrane	0.229083	#N/A	0.229083
Riboflavin_is_phosphorylated_to_FMN	0.189238	0.189238	#N/A
uPAR-acyl-GPI_+_H2O_->_uPAR_+_long-chain_fatty_acid	0.0135357	0.0135357	#N/A
Exocytosis_of_multimerin	0.0363784	#N/A	0.0363784
Pro-factor_VII_is_transported_from_the_endoplasmic_reticulum_to_the_G	0.568495	0.568495	#N/A
malate_[mitochondrial_matrix]_+_alpha-ketoglutarate_[cytosol]_<=>_malat	0.280298	0.280298	#N/A
PRDM4_translocates_to_the_nucleus	0.29972	#N/A	0.29972
UDP-glucose_is_oxidised_to_UDP-glucuronate	0.294106	#N/A	0.294106
Formation_of_Malonyl-CoA_from_Acetyl-CoA_(liver)	0.315304	#N/A	0.315304
Generation_of_Cytoplasmic_Acetyl_CoA_from_Citrate	0.433212	#N/A	0.433212
D-glyceraldehyde_3-phosphate_+_D-fructose_6-phosphate___<=>_xylulose_	0.754121	0.754121	#N/A

D-glyceraldehyde_3-phosphate_+_sedoheptulose_7-phosphate<=>_xylulose	0.754121	0.754121	#N/A
ribose_5-phosphate_+_xylulose_5-phosphate_<=>_sedoheptulose_7-phospl	0.754121	0.754121	#N/A
xylulose_5-phosphate_+_D-erythrose_4-phosphate_<=>_D-glyceraldehyde_:	0.754121	0.754121	#N/A
argininosuccinate_<=>_fumarate_+_arginine	0.829585	#N/A	0.829585
alpha-methyl-beta-hydroxybutyryl-CoA_+_NAD+_<=>_alpha-methylacetoace	0.376958	0.376958	#N/A
sequestered_tissue_factor_->_tissue_factor	0.380556	#N/A	0.380556
Squalene_2,3-epoxide_cyclizes,_forming_lanosterol	0.757929	0.757929	#N/A
acetyl-CoA_+_carnitine_=>_acetylcarnitine_+_CoASH	0.464615	0.464615	#N/A
propionyl-CoA_+_carnitine_=>_propionylcarnitine_+_CoASH	0.464615	0.464615	#N/A
alpha-ketoglutarate_+_NH4+_+_NADPH_+_H+_=>_glutamate_+_NADP+	0.271317	0.271317	#N/A
glutamate_+_NAD+_=>_alpha-ketoglutarate_+_NH4+_+_NADH_+_H+	0.271317	0.271317	#N/A
Release_of_Adenylyl_cyclase-associated_protein_1	0.848008	#N/A	0.848008
4-carboxycholesta-8(9),24-dien-3beta-ol_is_decarboxylated_and_oxidized_t	0.114948	#N/A	0.114948
4-methyl,4-carboxycholesta-8(9),24-dien-3beta-ol_is_decarboxylated_and_c	0.114948	#N/A	0.114948
Surface_deployment_of_P-selectin	0.0811855	#N/A	0.0811855
Conversion_of_coproporphyrinogen_III_to_protoporphyrinogen_IX	0.492207	#N/A	0.492207
Association_of_BID_with_mitochondrial_membrane	0.892705	0.892705	#N/A
Translocation_of_tBID_to_mitochondria	0.892705	0.892705	#N/A
Mevalonate_is_phosphorylated_to_mevalonate-5-phosphate	0.864154	0.864154	#N/A
Viral_dsRNA_binds_the_Toll-Like_Receptor_3_(TLR3)	0.508388	#N/A	0.508388
D-ribulose_5-phosphate_<=>_ribose_5-phosphate	0.8232	#N/A	0.8232
ribose_5-phosphate_<=>_D-ribulose_5-phosphate	0.8232	#N/A	0.8232
Phosphorylation_of_Cdc25C_at_Ser_216_by_Chk2(Cds1)	0.883587	0.883587	#N/A
Hydrolysis_of_phosphatidylcholine	0.0422632	#N/A	0.0422632
Phospho-PLA2_translocates_to_membranes_when_intracellular_calcium_le	0.0422632	#N/A	0.0422632
Release_of_WD_repeat-containing_protein_1	0.700399	0.700399	#N/A
Surface_deployment_of_PECAM-1	0.543394	#N/A	0.543394
Trans-homophilic_interaction_of_PECAM-1	0.543394	#N/A	0.543394
glyoxylate_+_alanine_=>_glycine_+_pyruvate	0.713266	0.713266	#N/A
Pro-GAS6_is_transported_from_the_endoplasmic_reticulum_to_the_Golgi_	0.117188	0.117188	#N/A
Thiamin_is_pyrophosphorylated	0.891806	0.891806	#N/A
Gly-3-P+FAD->DHAP+FADH2_(catalyzed_by_mitochondrial_Gly-Phos_dehydı	0.906392	0.906392	#N/A
Surface_deployment_of_CD63	0.026376	#N/A	0.026376
Pyridine_can_be_N-methylated	0.109506	#N/A	0.109506
Surface_deployment_of_CD9	0.801302	#N/A	0.801302
Release_of_Superoxide_dismutase_[Cu-Zn]	0.435389	#N/A	0.435389
(24R,_25R)_3alpha,7alpha,12alpha,24-tetrahydroxy-5beta-cholestanoyl-CoA	0.448321	0.448321	#N/A
(24R,_25R)_3alpha,7alpha,24-trihydroxy-5beta-cholestanoyl-CoA_is_oxidize	0.448321	0.448321	#N/A
25(S)_3alpha,7alpha,12alpha-trihydroxy-5beta-cholest-24-enoyl-CoA_is_hyd	0.448321	0.448321	#N/A
25(S)_3alpha,7alpha-dihydroxy-5beta-cholest-24-enoyl-CoA_is_hydrated_to	0.448321	0.448321	#N/A
3-hydroxyhexacosanoyl-CoA_+_NAD+_=>_3-ketohexacosanoyl-CoA_+_NADH	0.448321	0.448321	#N/A
3-hydroxypristanoyl-CoA_+_NAD+_=>_3-ketoxypristanoyl-CoA_+_NADH_+_I	0.448321	0.448321	#N/A
trans-2,3-dehydrohexacosanoyl-CoA_+_H2O_=>_3-hydroxyhexacosanoyl-Co	0.448321	0.448321	#N/A
trans-2,3-dehydropristanoyl-CoA_+_H2O_=>_3-hydroxypristanoyl-CoA	0.448321	0.448321	#N/A
Guanine_+_PRPP_=>_GMP_+_PPi	0.779301	0.779301	#N/A
Hypoxanthine_+_PRPP_=>_IMP_+_PPi	0.779301	0.779301	#N/A
Mevalonate-5-phosphate_is_further_phosphorylated.	0.735553	0.735553	#N/A

thymidine_+_orthophosphate_<=>_thymine_+_2-deoxy-D-ribose_1-phospha	0.995206	0.995206	#N/A
thymine_+_2-deoxy-D-ribose_1-phosphate_<=>_thymidine_+_orthophospha	0.995206	0.995206	#N/A
alpha-D-Glucose_6-phosphate_<=>_D-Glucose_1-phosphate	0.775648	0.775648	#N/A
D-Glucose_1-phosphate_<=>_alpha-D-Glucose_6-phosphate	0.775648	0.775648	#N/A
Acetyl-CoA_+_H2O_+_Oxaloacetate_=>_Citrate_+_CoA	0.382809	0.382809	#N/A
Conversion_of_cytosolic_5-methyltetrahydrofolate_(5-methylTHF)_to_5-me	0.17641	0.17641	#N/A
Conversion_of_cytosolic_THF_to_THF-polyglutamate	0.17641	0.17641	#N/A
Conversion_of_mitochondrial_THF_to_THF-polyglutamate	0.17641	0.17641	#N/A
Ligation_of_newly_synthesized_repair_patch_to_incised_DNA_in_GG-NER	0.264303	0.264303	#N/A
Ligation_of_newly_synthesized_repair_patch_to_incised_DNA_in_TC-NER	0.264303	0.264303	#N/A
Exocytosis_of_Clusterin	0.507785	0.507785	#N/A
D-fructose_6-phosphate_+_D-erythrose_4-phosphate__<=>__sedoheptulose	0.219358	0.219358	#N/A
sedoheptulose_7-phosphate_+_D-glyceraldehyde_3-phosphate_<=>_D-erytl	0.219358	0.219358	#N/A
guanidinoacetate_+_S-adenosylmethionine_=>_creatine_+_S-adenosylhomc	0.264969	0.264969	#N/A
Heterdimerization_of_CEACAMs	0.758075	#N/A	0.758075
JAM-A_homodimerises	0.661401	0.661401	#N/A
CYP26A1_breaks_down_all-trans-retinoic_acid_by_4-hydroxylation	0.859902	#N/A	0.859902
3alpha,_7alpha-dihydroxy-5beta-cholestan-26-al_is_oxidized_to_3alpha,_7a	0.665923	0.665923	#N/A
3alpha,7alpha,12alpha,24(S)-tetrahydroxy-5beta-cholestan-27-al_is_oxidizec	0.665923	0.665923	#N/A
3alpha,7alpha,12alpha-trihydroxy-5beta-cholestan-27-al_is_oxidized_to_3al	0.665923	0.665923	#N/A
3alpha,7alpha,24(S)-trihydroxy-5beta-cholestan-27-al_is_oxidized_to_3alpha	0.665923	0.665923	#N/A
5beta-cholestan-3alpha,_7alpha,_12alpha-triol_is_hydroxylated_to_5beta-cl	0.665923	0.665923	#N/A
5beta-cholestan-3alpha,_7alpha,_26-triol_is_oxidized_to_3alpha,_7alpha-di	0.665923	0.665923	#N/A
5beta-cholestan-3alpha,_7alpha-diol_is_hydroxylated_to_5beta-cholestan-3	0.665923	0.665923	#N/A
5beta-cholestan-3alpha,7alpha,12alpha,24(S),27-pentol_is_oxidized_to_3alp	0.665923	0.665923	#N/A
5beta-cholestan-3alpha,7alpha,12alpha,24(S)-tetrol_is_hydroxylated_to_5be	0.665923	0.665923	#N/A
5beta-cholestan-3alpha,7alpha,12alpha,27-tetrol_is_oxidized_to_3alpha,7al	0.665923	0.665923	#N/A
5beta-cholestan-3alpha,7alpha,24(S),27-tetrol_is_oxidized_to_3alpha,7alpha	0.665923	0.665923	#N/A
5beta-cholestan-3alpha,7alpha,24(S)-triol_is_hydroxylated_to_5beta-choles	0.665923	0.665923	#N/A
Cholesterol_is_hydroxylated_to_27-hydroxycholesterol_by_CYP27	0.665923	0.665923	#N/A
Release_of_Vinculin	0.98239	#N/A	0.98239
trehalose_+_H2O_=>_2_D-glucose	0.841443	0.841443	#N/A
Exocytosis_of_factor_VIII	0.98993	0.98993	#N/A
Exocytosis_of_fibronectin	0.832909	#N/A	0.832909
Cleavage_of_the_gamma-glutamyl_bond_of_LTC4_forms_LTD4	0.361032	0.361032	#N/A
Binding_of_TFIIIA_To_type_1_Promoter	0.46587	#N/A	0.46587
Phosphatidic_acid_+_H2O_<=>_Diacylglycerol_+__phosphate_(3)	0.506811	0.506811	#N/A
Exocytosis_of_Proactivator_polypeptide	0.793922	#N/A	0.793922
Condensation_of_nicotinamide_to_nicotinamide_D-ribonucleotide_(NMN)	0.069228	#N/A	0.069228
N-acetylglucosaminyl-PI_+_H2O_->_glucosaminyl-PI_+_acetate	0.225036	#N/A	0.225036
ALAD_octamer_associates_with_Pb++,_forming_a_catalytically_inactive_coi	0.138498	0.138498	#N/A
Two_molecules_of_ALA_condense_to_form_porphobilinogen_(PBG)	0.138498	0.138498	#N/A
Exocytosis_of_C1_inhibitor	0.80843	#N/A	0.80843
proline_=>_L-1-pyrroline-5-carboxylate	0.749393	0.749393	#N/A
glutamate_+_NH4+_+_ATP_=>_glutamine_+_ADP_+_orthophosphate	0.526846	0.526846	#N/A
Glutamate_conversion_to_glutamine	0.526846	0.526846	#N/A
LTA4_is_hydrolyzed_to_LTB4	0.553613	0.553613	#N/A

Active_PLCG1_hydrolyses_PIP2	0.787586	0.787586	#N/A
PLC_gamma_2-mediated_hydrolysis	0.787586	0.787586	#N/A
PLC-gamma1_hydrolyses_PIP2	0.787586	0.787586	#N/A
Translocation_of_PLC-gamma1_to_PIP2	0.787586	0.787586	#N/A
Condensation_of_phosphopantothenate_with_cysteine	0.545255	#N/A	0.545255
Exocytosis_of_Protein_S	0.705038	#N/A	0.705038
Pro-protein_S_is_transported_from_the_endoplasmic_reticulum_to_the_Go	0.705038	#N/A	0.705038
Release_of_Calumenin_	0.375588	#N/A	0.375588
JAM-B_homodimerises	0.735507	#N/A	0.735507
Cholesta-5,7,24-trien-3beta-ol_is_reduced_to_desmosterol	0.493658	#N/A	0.493658
Release_of_Peptidyl-prolyl_cis-trans_isomerase_A	0.727909	0.727909	#N/A
Release_of_SMAC_from_mitochondria	0.214489	0.214489	#N/A
gamma-glutamylcysteine_combines_with_glycine_to_form_glutathione	0.435771	#N/A	0.435771
4,4-dimethylcholesta-8(9),24-dien-3beta-ol_is_oxidized_to_4-methyl,4-carbo	0.0666381	#N/A	0.0666381
4-methylcholesta-8(9),24-dien-3beta-ol_is_oxidized_to_4-carboxycholesta-8	0.0666381	#N/A	0.0666381
(S)-Malate_+_NAD+_<=>_Oxaloacetate_+_NADH_+_H+	0.267224	0.267224	#N/A
Oxaloacetate_+_NADH_+_H+_<=>_(S)-Malate_+_NAD+	0.267224	0.267224	#N/A
Oxidative_demethylation_of_1-EtA_damaged_DNA_By_ABH2	0.0412706	#N/A	0.0412706
Oxidative_demethylation_of_1-MeA_damaged_DNA_By_ABH2	0.0412706	#N/A	0.0412706
Squalene_is_oxidized_to_its_epoxide	0.374546	#N/A	0.374546
Prostacyclin_synthase_(CYP8A1)_mediates_the_isomerization_of_prostaglan	0.750708	#N/A	0.750708
Extracellular_5-methyltetrahydrofolate_import_across_the_plasma_membra	0.955079	#N/A	0.955079
TRAIL_Binds_TRAIL-Receptor2	0.256604	#N/A	0.256604
Trimerization_of_TRAIL:_TRAIL_receptor-2_complex	0.256604	#N/A	0.256604
glycine_+_tRNA(Gly)_+_ATP_=>_Gly-tRNA(Gly)_+_AMP_+_pyrophosphate	0.397729	0.397729	#N/A
Oxidation_of_protoporphyrinogen_IX_to_protoporphyrin_IX	0.0126962	0.0126962	#N/A
N-formylkynurenine_+_H2O_=>_kynurenine_+_formate_[mouse]	0.764317	#N/A	0.764317
Exocytosis_of_hepatocyte_growth_factor	0.549833	#N/A	0.549833
alpha-D-glucose_6-phosphate_[cytosol]_=>_alpha-D-glucose_6-phosphate_[	0.157398	0.157398	#N/A
Oligomerization__of_BAK_at_the_mitochondrial_membrane	0.3878	0.3878	#N/A
2-deoxyadenosine_5-monophosphate_(dAMP)_+_H2O_=>_deoxyadenosine_	0.590965	#N/A	0.590965
D-glyceraldehyde_3-phosphate_<=>_dihydroxyacetone_phosphate	0.207352	0.207352	#N/A
dihydroxyacetone_phosphate_<=>_D-glyceraldehyde_3-phosphate	0.207352	0.207352	#N/A
Exocytosis_of_vWF	0.350444	0.350444	#N/A
Inhibition_of_GRK2_by_calmodulin	0.398356	0.398356	#N/A
Hydrolysis_of_PIP3_to_PIP2	0.68834	#N/A	0.68834
PTEN_dephosphorylates_PIP3	0.68834	#N/A	0.68834
Engulfed_CpG_DNA_binds_to_endosomal_TLR9	0.169743	#N/A	0.169743
alpha-D-glucose_6-phosphate_<=>_D-fructose_6-phosphate	0.389116	0.389116	#N/A
D-fructose_6-phosphate_<=>_alpha-D-Glucose_6-phosphate	0.389116	0.389116	#N/A
Association_of_RAD52_with_ssDNA_at_resected_ends_of_double-strand_b	0.00936727	#N/A	0.00936727
Phosphatidic_acid_+_H2O_<=>_Diacylglycerol_+__phosphate_(2)	0.565167	#N/A	0.565167
Binding_of_Pol_eta_to_lesioned_DNA_template	0.280132	0.280132	#N/A
Elongation_by_Pol_eta_	0.280132	0.280132	#N/A
Insertion_of_correct_bases_opposite_to_the_lesion_by_Pol_eta	0.280132	0.280132	#N/A
thymidine_+_ATP_=>_thymidine_5-monophosphate_(dTMP)_+_ADP	0.850954	#N/A	0.850954
Mevalonate-5-pyrophosphate_is_decarboxylated	0.0974044	0.0974044	#N/A

Phosphopantothenoylcysteine_is_decarboxylated	0.118543	0.118543	#N/A
Relocalization_of_nuclearly_localized_Cyclin_D1_to_the_cytoplasm	0.731711	0.731711	#N/A
SLC36A1-mediated_uptake_of_glycine,_proline,_and_alanine	0.178434	0.178434	#N/A
SLC36A2-mediated_uptake_of_glycine_and_proline	0.178434	0.178434	#N/A
2-deoxycytidine_+_H2O_=>_2-deoxyuridine_+_NH4+	0.335317	0.335317	#N/A
cytidine_+_H2O_=>_uridine_+_NH4+	0.335317	0.335317	#N/A
TFAM_binds_to_mitochondrial_promoters	0.263855	0.263855	#N/A
Lanosterol_is_oxidatively_demethylated_to_4,4-dimethylcholesta-8(9),14,24	0.558679	#N/A	0.558679
(S)-3-Hydroxydodecanoyl-CoA+NAD<=>3-Oxododecanoyl-CoA+NADH+H	0.250933	0.250933	#N/A
(S)-Hydroxybutanoyl-CoA+NAD<=>Acetoacetyl-CoA+NADH+H	0.250933	0.250933	#N/A
(S)-Hydroxydecanoyl-CoA+NAD<=>3-Oxodecanoyl-CoA+NADH+H	0.250933	0.250933	#N/A
(S)-Hydroxyhexanoyl-CoA+NAD<=>3-Oxohexanoyl-CoA+NADH+H	0.250933	0.250933	#N/A
(S)-Hydroxyoctanoyl-CoA+NAD<=>3-Oxooctanoyl-CoA+NADH+H	0.250933	0.250933	#N/A
Translocation_of_p21_to_the_nucleus	0.787693	0.787693	#N/A
Release_of_Secretogranin-3	0.874426	0.874426	#N/A
Autophosphorylation_of_DNA-PKcs	0.616799	#N/A	0.616799
Release_of_78_kDa_glucose-regulated_protein	0.850832	0.850832	#N/A
Binding_of_ADAR1_homodimer_to_dsRNA_duplex	0.943088	#N/A	0.943088
Deamination_at_C6_position_of_adenosine_in_Editosome_(ADAR1)	0.943088	#N/A	0.943088
Formation_of_ADAR1_homodimer	0.943088	#N/A	0.943088
Adenosine_3,5-bisphosphate_(PAP)_+_H2O_=>_AMP_+_orthophosphate	0.598315	0.598315	#N/A
Phosphatidic_acid_+_H2O_<=>_Diacylglycerol_+__phosphate_(1)	0.143838	0.143838	#N/A
Isomerization_of_3-trans-decenoyl-CoA_to_form_trans-dec-2-enoyl-CoA	0.122224	0.122224	#N/A
Isomerization_of_cis,cis-3,6-Dodecadienoyl-CoA_to_form_trans,cis-Lauro-2,(	0.122224	0.122224	#N/A
palmitoyl-CoA+FAD<=>trans-Hexadec-2-enoyl-CoA+FADH2	0.256552	0.256552	#N/A
2-trans-Dodecenoyl-CoA+H2O<=>(S)-3-Hydroxydodecanoyl-CoA	0.0372115	0.0372115	#N/A
Crotonoyl-CoA+H2O<=>(S)-3-Hydroxybutanoyl-CoA	0.0372115	0.0372115	#N/A
trans-Dec-2-enoyl-CoA+H2O<=>(S)-Hydroxydecanoyl-CoA	0.0372115	0.0372115	#N/A
trans-Hex-2-enoyl-CoA+H2O<=>(S)-Hydroxyhexanoyl-CoA	0.0372115	0.0372115	#N/A
trans-Oct-2-enoyl-CoA+H2O<=>(S)-Hydroxyoctanoyl-CoA	0.0372115	0.0372115	#N/A
2-deoxycytidine_5-monophosphate_(dCMP)_+_H2O_=>_2-deoxyuridine_5-r	0.143598	#N/A	0.143598
mannose(a1-4)glucosaminyl-acyl-PI_+_phosphatidylethanolamine_->_(ethar	0.439145	#N/A	0.439145
D-methylmalonyl-CoA_<=>_L-methylmalonyl-CoA	0.96223	0.96223	#N/A
lysophosphatidic_acid_+_fatty_acyl_CoA__=>_phosphatidic_acid_+_CoA_(5)	0.273562	0.273562	#N/A
lauroyl-CoA+FAD<=>2-trans-Dodecenoyl-CoA+FADH2	0.134736	#N/A	0.134736
myristoyl-CoA+FAD<=>trans-Tetradec-2-enoyl-CoA+FADH2	0.134736	#N/A	0.134736
UDP-glucuronate_transport_from_the_cytosol_to_ER_lumen	0.70983	0.70983	#N/A
acetyl-CoA_+_H2O_=>_acetate_+_CoASH	0.486134	0.486134	#N/A
Hydrolysis_of_choloyl-CoA_to_cholate_and_CoASH	0.486134	0.486134	#N/A
Cleavage_of_cytosine_glycol_by_hNTH1_glycosylase	0.792707	0.792707	#N/A
Cleavage_of_dihydrouracil_by_hNTH1_glycosylase	0.792707	0.792707	#N/A
Cleavage_of_formamidopyrimidine__by_hNTH1_glycosylase_	0.792707	0.792707	#N/A
Cleavage_of_thymine_glycol__by_hNTH1_glycosylase__	0.792707	0.792707	#N/A
hNTH1_glycosylase_mediated_recognition_and_binding_of_cytosine_glycol	0.792707	0.792707	#N/A
hNTH1_glycosylase_mediated_recognition_and_binding_of_dihydrouracil_	0.792707	0.792707	#N/A
hNTH1_glycosylase_mediated_recognition_and_binding_of_formamidopyrii	0.792707	0.792707	#N/A
hNTH1_glycosylase_mediated_recognition_and_binding_of_thymine_glycol	0.792707	0.792707	#N/A

orotate_+_5-phospho-alpha-D-ribose_1-diphosphate_(PRPP)_<=>_orotidine_	0.243645	0.243645	#N/A
orotidine_5-monophosphate_=>_uridine_5-monophosphate_+_CO2	0.243645	0.243645	#N/A
Dephosphorylation_of_pChREBP_(Ser_196)_by_PP2A	0.479792	0.479792	#N/A
Dephosphorylation_of_pChREBP_(Ser_568)_by_PP2A	0.479792	0.479792	#N/A
Dephosphorylation_of_pChREBP_(Thr_666)_by_PP2A	0.479792	0.479792	#N/A
Nuclear_transport_of_pChREBP_(Thr_666)_protein	0.479792	0.479792	#N/A
PhosphoChREBP_(Thr_666)__is_exported_to_cytosol	0.479792	0.479792	#N/A
2-Deoxyadenosine_+_H2O_=>_2-Deoxyinosine_+_NH3	0.768054	0.768054	#N/A
Adenosine_+_H2O_=>_Inosine_+_NH3	0.768054	0.768054	#N/A
PDK1_attachment_to_plasma_membrane	0.829344	#N/A	0.829344
Translocation_of_PDK1_to_Plasma_membrane	0.829344	#N/A	0.829344
L-methylmalonyl-CoA_<=>_succinyl-CoA	0.690603	#N/A	0.690603
Oxidative_demethylation_of_1-EtA_damaged_DNA_By_ABH3	0.402792	#N/A	0.402792
Oxidative_demethylation_of_1-MeA_damaged_DNA_By_ABH3	0.402792	#N/A	0.402792
Oxidative_demethylation_of_3-MeC_damaged_DNA_By_ABH3	0.402792	#N/A	0.402792
Dab2_interacts_with_clathrin	0.826294	0.826294	#N/A
Dab2_is_recruited_to_the_junctional_plaques	0.826294	0.826294	#N/A
Dynamin_is_recruited_to_the_gap_junction_plaque	0.826294	0.826294	#N/A
Internalization_of_gap_junction_plaques	0.826294	0.826294	#N/A
Part_of_the_ICD_migrates_to_the_nucleus	0.657777	#N/A	0.657777
The_p75NTR_C-terminal_fragment_enters_endosomes	0.657777	#N/A	0.657777
L-1-pyrroline-5-carboxylate_+_NADPH_+_H+_=>_proline_+_NADP+	0.0207091	0.0207091	#N/A
Translocation_of_p27_to_the_nucleoplasm	0.43394	0.43394	#N/A
Dimerization_of_Phospho-IRF3	0.108695	#N/A	0.108695
Dimerized_Phospho-IRF3_is_Transported_To_The_Nucleus	0.108695	#N/A	0.108695
Exocytosis_of_SRGN	0.0413677	#N/A	0.0413677
JAM-C_homodimerises	0.113948	0.113948	#N/A
Oligomerization_of_BAX_at_the_mitochondrial_membrane	0.0586851	0.0586851	#N/A
Translocation_of_activated_BAX_to_the_mitochondria	0.0586851	0.0586851	#N/A
Biotin_transport_across_the_plasma_membrane	0.247696	0.247696	#N/A
Pantothenate_transport_across_the_plasma_membrane	0.247696	0.247696	#N/A
Dimerisation_of_CREB	0.664073	#N/A	0.664073
Digestion_of_1-6_linkages_of_limit_dextrins_to_yield_maltose,_maltotriose	0.251645	0.251645	#N/A
maltose_+_H2O_=>_2_D-glucose_(maltase-glucoamylase)	0.251645	0.251645	#N/A
maltose_+_H2O_=>_2_D-glucose_(sucrase-isomaltase)	0.251645	0.251645	#N/A
maltotriose_+_H2O_=>_maltose_+_D-glucose_(maltase-glucoamylase)	0.251645	0.251645	#N/A
maltotriose_+_H2O_=>_maltose_+_D-glucose_(sucrase-isomaltase)	0.251645	0.251645	#N/A
sucrose_+_H2O_=>_glucose_+_fructose	0.251645	0.251645	#N/A
beta-methylglutaconyl-CoA_+_H2O_<=>_beta-hydroxy-beta-methylglutaryl-	0.278978	0.278978	#N/A
5-phosphoribosyl-5-aminoimidazole-4-carboxamide_(AICAR)_+_10-formylte	0.249975	0.249975	#N/A
5-phosphoribosyl-5-formaminoimidazole-4-carboxamide_(FAICAR)_<=>_inos	0.249975	0.249975	#N/A
Dissociation_of_phosphoribosyl_pyrophosphate_amidotransferase_tetrame	0.930843	0.930843	#N/A
Formation_of_phosphoribosyl_pyrophosphate_amidotransferase_tetramer	0.930843	0.930843	#N/A
5-phospho-alpha-D-ribose_1-diphosphate_(PRPP)_+_H2O_+_L-glutamine_<=	0.930843	0.930843	#N/A
GTP_loading_by_Rheb	0.186135	0.186135	#N/A
Cholesterol_is_hydroxylated_to_24-hydroxycholesterol_by_CYP46A1	0.811828	0.811828	#N/A
Pro-beta-NGF_and_mature_beta-NGF_are_secreted	0.496327	#N/A	0.496327

pro-beta-NGF_homodimer_transits_to_the_golgi_apparatus	0.496327	#N/A	0.496327
Dissociation_of_dimeric_phospho-ATM_complexes	0.844853	#N/A	0.844853
Intermolecular_autophosphorylation_of_ATM_within_dimeric_ATM_comple	0.844853	#N/A	0.844853
Amidation_of_deamino-NAD+_to_NAD+	0.878669	0.878669	#N/A
Mitochondrial_recruitment_of_Drp1	0.531532	#N/A	0.531532
5,10-methyleneTHF_polyglutamate_+_NADPH_+_H+_=>_5-methylTHF_poly	0.692396	0.692396	#N/A
Release_of_Profilin-1	0.493921	0.493921	#N/A
(2S)-pristanoyl-CoA_+_O2_=>_trans-2,3-dehydropristanoyl-CoA_+_H2O2_(A	0.416318	0.416318	#N/A
Ferrous_iron_is_inserted_into_protoporphyrin_IX_to_form_heme	0.509318	0.509318	#N/A
Reduction_of_HMG-CoA_produces_mevalonate	0.455267	#N/A	0.455267
Cleavage_of__thymine_by_MBD4_glycosylase	0.88422	#N/A	0.88422
Cleavage_of_uracil_by_MBD4_glycosylase	0.88422	#N/A	0.88422
MBD4_glycosylase_mediated_recognition_and_binding_of_a_thymine_opp	0.88422	#N/A	0.88422
MBD4_glycosylase_mediated_recognition_and_binding_of_an_uracil_oppos	0.88422	#N/A	0.88422
Uroporphyrinogen_I_is_decarboxylated_to_form_coproporphyrinogen_I	0.20088	0.20088	#N/A
Uroporphyrinogen_III_is_decarboxylated_to_form_coproporphyrinogen_III	0.20088	0.20088	#N/A
glutamate_+_L-glutamate_gamma-semialdehyde_<=>_ornithine_+_alpha-ke	0.327406	0.327406	#N/A
ornithine_+_alpha-ketoglutarate_<=>_glutamate_+_L-glutamate_gamma-se	0.327406	0.327406	#N/A
24-hydroxycholesterol_is_7alpha-hydroxylated_to_yield_cholest-5-ene-3bet	0.37085	#N/A	0.37085
UBF-1_Binds_rDNA_Promoter	0.598136	0.598136	#N/A
Four_PBGs_combine_through_deamination_to_form_hydroxymethylbilane_	0.81781	#N/A	0.81781
2-deoxyguanosine_5-diphosphate_(dGDP)_+_ADP_<=>_2-deoxyguanosine_5	0.191862	0.191862	#N/A
2-deoxyguanosine_5-monophosphate_(dGMP)_+_ATP_<=>_2-deoxyguanosi	0.191862	0.191862	#N/A
guanosine_5-diphosphate_(GDP)_+_ADP_<=>_guanosine_5-monophosphate	0.191862	0.191862	#N/A
guanosine_5-monophosphate_(GMP)_+_ATP_<=>_guanosine_5-diphosphate	0.191862	0.191862	#N/A
beta-hydroxyisobutyryl-CoA_+_H2O_=>_beta-hydroxyisobutyrate_+_CoA	0.934775	#N/A	0.934775
Basigin_homodimerises	0.100171	0.100171	#N/A
2-deoxyuridine_5-monophosphate_(dUMP)_+_N5,N10-methylene_tetrahyd	0.189915	0.189915	#N/A
MCM8_mediated_fork_unwinding	0.544194	0.544194	#N/A
Exocytosis_of_Thymosin_beta-4	0.434551	0.434551	#N/A
Reduction_of_presqualene_diphosphate_to_form_squalene	0.727556	#N/A	0.727556
Two_FPP_molecules_dimerize_to_form_presqualene_diphosphate	0.727556	#N/A	0.727556
2-deoxycytidine_5-diphosphate_(dCDP)_+_ADP_<=>_2-deoxycytidine_5-mo	0.906346	0.906346	#N/A
2-deoxycytidine_5-monophosphate_(dCMP)_+_ATP_<=>_2-deoxycytidine_5	0.906346	0.906346	#N/A
11-deoxycorticosterone_is_oxidised_to_corticosterone_by_CYP11B2	0.978373	#N/A	0.978373
11-deoxycortisol_is_oxidised_to_cortisol_by_CYP11B1	0.978373	#N/A	0.978373
Conversion_of_18-hydroxycorticosterone_to_aldosterone	0.978373	#N/A	0.978373
Hydroxylation_of_corticosterone_to_form_18-hydroxycorticosterone	0.978373	#N/A	0.978373
4,4-dimethylcholesta-8(9),14,24-trien-3beta-ol_is_reduced_to_4,4-dimethyl	0.629583	#N/A	0.629583
4,4-dimethylcholesta-8(9),14,24-trien-3beta-ol_is_reduced_to_4,4-dimethyl	0.629583	#N/A	0.629583
5-phosphoribosylformylglycinamidine_(FGAM)_+_ATP_=>_5-phosphoribosyl	0.823777	#N/A	0.823777
5-phosphoribosylamine_+_glycine_+_ATP_<=>_5-phosphoribosylglycinamide	0.823777	#N/A	0.823777
5-phosphoribosylglycinamide_(GAR)_+_10-formyl-tetrahydrofolate_=>_5-ph	0.823777	#N/A	0.823777
2-deoxyuridine_5-diphosphate_(dUDP)_+_ADP_<=>_2deoxyuridine_5-mono	0.785939	#N/A	0.785939
2-deoxyuridine_5-monophosphate_(dUMP)_+_ATP_<=>_2-deoxyuridine_5-c	0.785939	#N/A	0.785939
thymidine_5-diphosphate_(TDP)_+_ADP_<=>_thymidine_5-monophosphate	0.785939	#N/A	0.785939
thymidine_5-monophosphate_(TMP)_+_ATP_<=>_thymidine_5-diphosphate	0.785939	#N/A	0.785939

SLC38A2_(ATA2)-mediated_uptake_of_neutral_amino_acids	0.70277	0.70277	#N/A
Dephosphorylation_of_phospho-Cdh1	0.0548582	0.0548582	#N/A
Receptor_CCR9__binds_CCL25_ligand	0.00987626	#N/A	0.00987626
Association_of_mTERF_with_the_termination_sequence	0.0457118	#N/A	0.0457118
M2_and_M4_receptors_bind_acetylcholine	0.531376	0.531376	#N/A
glutamine_+_tRNA(Glu)_+_ATP_=>_Glu-tRNA(Glu)_+_AMP_+_pyrophosphat	0.392073	0.392073	#N/A
SLC43A1_(LAT3)-mediated_uptake_of_large_neutral_amino_acids	0.339604	0.339604	#N/A
tyrosine_+_tRNA(Tyr)_+_ATP_=>Tyr-tRNA(Tyr)_+_AMP_+_pyrophosphate	0.756019	0.756019	#N/A
Conversion_of_HMB_to_uroporphyrinogen_III	0.0799748	0.0799748	#N/A
Conversion_of_Dihydroxyacetone_Phosphate_to_Glycerol_-3-_phosphate	0.270727	0.270727	#N/A
Nicotinate_D-ribonucleotide_+_ATP_=>_deamino-NAD+_+_pyrophosphate_	0.754855	0.754855	#N/A
Translocation_of_BIM_to_mitochondria	0.246994	0.246994	#N/A
Alignment_Of_The_RNA_Template_On_The_Telomeric_Chromosome_End	0.474325	#N/A	0.474325
Disassociation_of_Telomerase_RNP_and_the_Chromosome_End	0.474325	#N/A	0.474325
Elongation_of_Extended_Telomeric_Chromosome_End	0.474325	#N/A	0.474325
Elongation_Of_The_Telomeric_Chromosome_End	0.474325	#N/A	0.474325
Recruitment_of_Telomerase_RNP_to_the_Telomeric_Chromosome_End	0.474325	#N/A	0.474325
Translocation_Of_Telomerase_RNP_And_Alignment_Of_RNA_Template_(TE	0.474325	#N/A	0.474325
APE1_mediated_endonucleolytic_cleavage_at_the_5_side_of_the_base-free	0.465958	#N/A	0.465958
Displacement_of_UNG2_glycosylase_by__APE1_at_the_AP_site	0.465958	#N/A	0.465958
MGMT/hAGT_mediated_DNA_Damage_Reversal	0.539137	0.539137	#N/A
Translocation_of_PUMA_protein_to_mitochondria	0.821795	#N/A	0.821795
Homodimerization__of_DFF40	0.51349	#N/A	0.51349
methylmalonate_semialdehyde_+_NAD+_+_CoA_=>_propionyl-CoA_+_CO2_	0.485283	0.485283	#N/A
Phosphorylated_Foxo1_is_excluded_from_the_nucleus	0.409114	#N/A	0.409114
Phosphorylated_FOXO1A_is_excluded_from_the_nucleus	0.409114	#N/A	0.409114
Adrostenedione_is_converted_to_estrone_by_Aromatase_(CYP19A1)	0.0641587	#N/A	0.0641587
Testosterone_is_converted_to_estradiol	0.0641587	#N/A	0.0641587
cysteine_+_tRNA(Cys)_+_ATP_=>_Cys-tRNA(Cys)_+_AMP_+_pyrophosphate	0.809609	#N/A	0.809609
DARPP-32_is_dephosphorylated_on_Thr75_by_PP2A	0.486872	#N/A	0.486872
Further_hydroxylation_of_calcidiol_in_kidney_to_form_calcitriol	0.744301	#N/A	0.744301
Oxytocin_receptor_bind_oxytocin	0.417763	#N/A	0.417763
1,25-dihydroxyvitamin_D3_is_deactivated	0.472365	0.472365	#N/A
CYP24A1_catalyzes_tjhe_initial_step_in_the_deactivation_of_the_hormonal	0.472365	0.472365	#N/A
D-glucono-1,5-lactone_6-phosphate_+_H2O_=>_6-phospho-D-gluconate	0.67315	0.67315	#N/A
Connexin_62_mediated_neuronal_gap_junction_communication	0.847765	#N/A	0.847765
valine_+_tRNA(Val)_+_ATP_=>_Val-tRNA(Val)_+_AMP_+_pyrophosphate	0.575674	0.575674	#N/A
Receptor_CX3C1_binds_fractalkine	0.0584022	0.0584022	#N/A
CoA_transport_across_the_inner_mitochondrial_membrane	0.884029	0.884029	#N/A
N-acetylation_of_serotonin	0.973512	#N/A	0.973512
Iodide_is_taken_up_by_thyroid_epithelial_cells	0.526147	0.526147	#N/A
CYP26B1_also_deactivates_all-trans-retinoic_acid_by_4-hydroxylation	0.448966	#N/A	0.448966
CYP26C1_deactivates_9-cis-retinoic_acid_by_4-hydroxylation	0.448966	#N/A	0.448966
Protaglandin_E_synthase_isomerizes_PGH2_to_PGE2	0.368844	#N/A	0.368844
4-methylcholesta-8(9),24-dien-3-one_is_reduced_to_4-methylcholesta-8(9),	0.540163	0.540163	#N/A
Zymosterone_(cholesta-8(9),24-dien-3-one)_is_reduced_to_zymosterol_(ch	0.540163	0.540163	#N/A
GPCR_neuropeptide_receptor_binds_neuropeptides_B_and_W	0.976475	#N/A	0.976475

H2_receptor_binds_histamine	0.330443	#N/A	0.330443
GTP-binding_activates_eEF2	0.116039	0.116039	#N/A
LTA4_conjugates_with_glutathione_to_form_LTC4	0.631521	#N/A	0.631521
Translocation_of_activated_BAD_protein_to_mitochondria	0.268051	0.268051	#N/A
beta-hydroxyisobutyrate_+_NAD+_<=>_methylmalonyl_semialdehyde_+_NA	0.54435	0.54435	#N/A
hydrolysis_of__2-deoxyuridine_5-triphosphate_to_form__2-deoxyuridine_5	0.111183	0.111183	#N/A
Platelet-derived_TREM-1_ligand_binds_to_TREM-1	0.716744	#N/A	0.716744
H1_receptor_binds_histamine	0.198946	0.198946	#N/A
Noradrenaline_is_converted_to_adrenaline	0.535611	0.535611	#N/A
Cholesterol_is_released_into_the_inner_mitochondrial_membrane	0.447084	#N/A	0.447084
Phosphorylation_of_Cdc25C_at_Ser_216_by_Chk1	0.813689	0.813689	#N/A
TTF-I_binds_to_the_Sal_Box	0.822272	0.822272	#N/A
Association_of_RAD51_with_the_resected_ends_of_the_DNA_double-stran	0.425233	#N/A	0.425233
Dopamine_is_oxidised_to_noradrenaline	0.694375	#N/A	0.694375
Synthesis_of_noradrenaline	0.694375	#N/A	0.694375
Translocation_of_BMF_to_mitochondria	0.200991	#N/A	0.200991
Cholesterol_is_hydroxylated_to_25-hydroxycholesterol	0.858253	#N/A	0.858253
G_protein-coupled_estrogen_receptor_1_binds_estrogen_non-classically	0.911982	#N/A	0.911982
Digestion_of_cholesterol_esters_by_extracellular_CEL_(bile_salt-dependent	0.335425	#N/A	0.335425
Digestion_of_monoacylglycerols_by_extracellular_CEL_(bile_salt-dependent	0.335425	#N/A	0.335425
Digestion_of_triacylglycerols_by_extracellular_CEL_(bile_salt-dependent_lip	0.335425	#N/A	0.335425
Production_of_ceramide_which_can_activate_JNK_and_other_targets	0.0763051	0.0763051	#N/A
tyrosine_+_tRNA(Tyr)_+_ATP_=>_Tyr-tRNA(Tyr)_+_AMP_+_pyrophosphate	0.516665	0.516665	#N/A
SLC6A20-mediated_uptake_of_proline	0.793025	0.793025	#N/A
FMN_is_futher_phosphorylated_to_FAD	0.523976	0.523976	#N/A
Entry_of_Ca++_from_plasma	0.673481	0.673481	#N/A
Decanoyl-CoA+FAD<=>trans-Dec-2-enoyl-CoA+FADH2	0.311445	0.311445	#N/A
dehydrogenation_of_4-cis-decenoyl-CoA_to_form_2-trans-4-cis-decadienoy	0.311445	0.311445	#N/A
Octanoyl-CoA+FAD<=>trans-Oct-2-enoyl-CoA+FADH2	0.311445	0.311445	#N/A
Reduction_of_dehydroascorbate_to_ascorbate	0.891084	#N/A	0.891084
21-hydroxylation_of_progesterone_to_form_11-deoxycorticosterone	0.31559	0.31559	#N/A
Hydroxylation_of_17-hydroxyprogesterone_to_form_11-deoxycortisol	0.31559	0.31559	#N/A
Exocytosis_of_beta-thromboglobulin	0.659096	#N/A	0.659096
Exocytosis_of_neutrophil-activating_peptide_2	0.659096	#N/A	0.659096
Cleavage_8-oxo_guanine__by_MYH_glycosylase	0.490565	#N/A	0.490565
MYH_glycosylase_mediated_recognition_and_binding_of_an_adenine_oppo	0.490565	#N/A	0.490565
(ethanolamineP)_mannose_(a1-2)_mannose_(a1-6)_(ethanolamineP)_mann	0.564998	#N/A	0.564998
mannose_(a1-6)_(ethanolamineP)_mannose_(a1-4)_glucosaminyl-acyl-PI_+_	0.745924	0.745924	#N/A
27-hydroxycholesterol_+_PAPS_=>_27-hydroxycholesterol_3-sulfate_+_PAP	0.30095	#N/A	0.30095
cholesterol_+_PAPS_=>_cholesterol_sulfate_+_PAP	0.30095	#N/A	0.30095
Cleavage_of__thymine_by_TDG_glycosylase	0.00702071	#N/A	0.00702071
Cleavage_of_ethenocytosine_by_TDG_glycosylase_	0.00702071	#N/A	0.00702071
Cleavage_of_uracil_by_TDG_glycosylase	0.00702071	#N/A	0.00702071
TDG_glycosylase_mediated_recognition_and_binding_of_an_ethenocytosin	0.00702071	#N/A	0.00702071
TDG_glycosylase_mediated_recognition_and_binding_of_an_thymine_oppo	0.00702071	#N/A	0.00702071
TDG_glycosylase_mediated_recognition_and_binding_of_an_uracil_opposit	0.00702071	#N/A	0.00702071
Connexin_36_mediated_neuronal_gap_junction_communication	0.742917	0.742917	#N/A

alpha-D-Glucose_6-phosphate_+_H2O_=>_alpha-D-Glucose_+_Orthophosph	0.884628	0.884628	#N/A
lithocholate_+_PAPS_=>_lithocholate_sulfate_+_PAP	0.948537	#N/A	0.948537
taurolithocholate_+_PAPS_=>_taurolithocholate_sulfate_+_PAP	0.948537	#N/A	0.948537
20alpha,22beta-hydroxycholesterol_is_cleaved_by_CYP11A1_to_yield_pregı	0.989061	#N/A	0.989061
Oxidation_of_22beta-hydroxycholesterol_to_20alpha,22beta-hydroxycholes	0.989061	#N/A	0.989061
Oxidation_of_cholesterol_to_22beta-hydroxycholesterol	0.989061	#N/A	0.989061
Cleavage_of_uracil_by_hSMUG1_glycosylase	0.355499	0.355499	#N/A
hSMUG1_glycosylase_mediated_recognition_and_binding_of_uracil_within_	0.355499	0.355499	#N/A
Thiamin_transport_across_the_plasma_membrane	0.984062	#N/A	0.984062
malate_+_NAD+_<=>_oxaloacetate_+_NADH_+_H+	0.177506	0.177506	#N/A
glucosaminyl-PI_+_fatty_acyl-CoA_->_glucosaminyl-acyl-PI_+_CoA-SH	0.630868	#N/A	0.630868
Cleavage_of_8-oxo_guanine_by_hOGG1_glycosylase	0.468329	#N/A	0.468329
Cleavage_of_formamidopyrimidine_by_hOGG1_glycosylase	0.468329	#N/A	0.468329
hOGG1_glycosylase_mediated_recognition_and_binding_of_a_formamidopy	0.468329	#N/A	0.468329
hOGG1_glycosylase_mediated_recognition_and_binding_of_an_8-oxo_guan	0.468329	#N/A	0.468329
Conversion_of_glycerol-3-phosphate_to_lysophosphatidic_acid	0.716703	0.716703	#N/A
Adenine_+_PRPP_=>_AMP_+_PPi	0.0536607	0.0536607	#N/A
Synthesis_of_o-acetylcholine	0.987574	0.987574	#N/A
CPT2_converts_palmitoyl_carnitine_to_palmitoyl-CoA	0.465955	0.465955	#N/A
arginine_+_glycine_=>_ornithine_+_guanidoacetate	0.698791	#N/A	0.698791
Cleavage_of_3-methyladenine_by_MPG_glycosylase_	0.450576	0.450576	#N/A
Cleavage_of_ethenoadenine_by_MPG_glycosylase	0.450576	0.450576	#N/A
Cleavage_of_hypoxanthine_by_MPG_glycosylase	0.450576	0.450576	#N/A
MPG_glycosylase_mediated_recognition_and_binding_of_3-methyladenine_	0.450576	0.450576	#N/A
MPG_glycosylase_mediated_recognition_and_binding_of_ethenoadenine_	0.450576	0.450576	#N/A
MPG_glycosylase_mediated_recognition_and_binding_of_hypoxanthine	0.450576	0.450576	#N/A
Phosphorylated_Cdc6_is_exported_from_the_nucleus	0.266815	#N/A	0.266815
Cytosolic_tetrahydrofolate_import_across_the_inner_mitochondrial_memb	0.763445	#N/A	0.763445
Mitochondrial_tetrahydrofolate_export_across_the_inner_mitochondrial_m	0.763445	#N/A	0.763445
SLC16A10-mediated_uptake_of_aromatic_amino_acids	0.715803	0.715803	#N/A
HCO3-_transport_through_ion_channel	0.684106	0.684106	#N/A
tryptophan_+_O2_=>_N-formylkynurenine_[IDO]	0.886218	0.886218	#N/A
5-phosphoribosyl-5-aminoimidazole_(AIR)_+_CO2_<=>_5-phosphoribosyl-5-	0.999148	0.999148	#N/A
5-phosphoribosyl-5-aminoimidazole-4-carboxylate_(CAIR)_+_L-aspartate_+_	0.999148	0.999148	#N/A
isobutyryl-CoA_+_FAD_=>_methacrylyl-CoA_+_FADH2	0.834097	0.834097	#N/A
(ethanolamineP)_mannose_(a1-4)_glucosaminyl-acyl-PI_+_dolichol_phospha	0.240437	0.240437	#N/A
xanthosine_5-monophosphate_(XMP)_+_L-glutamine_+_ATP_+_H2O_=>_gu	0.715255	#N/A	0.715255
isovaleryl-CoA_+_FAD_=>_beta-methylcrotonyl-CoA_+_FADH2	0.114819	0.114819	#N/A
(S)-dihydroorotate_+_ubiquinone_=>_orotate_+_ubiquinol	0.152294	0.152294	#N/A

Adipose(WAT)

Gene ontology(GO)

#Feature	Reporter P-value<br>No-direction	 Down regulated	 Up regulated

CC_GO:0016020_membrane	1.06409E-05	0.519364	2.29468E-10
CC_GO:0016021_integral+to+membrane	6.6492E-07	0.0267036	7.80158E-07
CC_GO:0005634_nucleus	1	1	0.996529
MF_GO:0005515_protein+binding	0.967488	0.997197	0.415297
CC_GO:0005737_cytoplasm	0.999877	0.999969	0.785307
MF_GO:0004872_receptor+activity	0.0308294	0.0236078	0.186176
MF_GO:0046872_metal+ion+binding	0.997474	0.999898	0.487591
CC_GO:0005886_plasma+membrane	8.87916E-08	0.0182617	5.6103E-08
MF_GO:0008270_zinc+ion+binding	0.997395	0.999845	0.645118
BP_GO:0006355_regulation+of+transcription,+DNA-dependent	0.999951	0.998781	0.993324
BP_GO:0007186_G-protein+coupled+receptor+protein+signaling+pathway	0.336271	0.13522	0.57135
MF_GO:0003677_DNA+binding	0.92133	0.982382	0.474388
MF_GO:0000166_nucleotide+binding	0.999997	0.9999	0.998526
BP_GO:0006810_transport	0.809864	0.997153	0.0458746
BP_GO:0006350_transcription	0.999966	0.996525	0.998345
BP_GO:0008150_biological_process	0.772063	0.946918	0.269978
CC_GO:0005576_extracellular+region	1.53211E-14	1.21825E-09	1.38943E-06
CC_GO:0005575_cellular_component	0.385321	0.755448	0.123686
MF_GO:0003674_molecular_function	0.788562	0.935793	0.336069
MF_GO:0016787_hydrolase+activity	0.925482	0.974228	0.525414
MF_GO:0016740_transferase+activity	0.882896	0.775483	0.824612
CC_GO:0005622_intracellular	0.999981	0.999989	0.938637
MF_GO:0005524_ATP+binding	0.999743	0.995323	0.989574
BP_GO:0007608_sensory+perception+of+smell	0.941815	0.383706	0.974268
MF_GO:0004984_olfactory+receptor+activity	0.954377	0.443656	0.976954
BP_GO:0007165_signal+transduction	0.00343083	0.131401	0.00495414
CC_GO:0005739_mitochondrion	0.999998	0.999998	0.930564
BP_GO:0007275_multicellular+organismal+development	0.056892	0.679172	0.00364922
MF_GO:0005509_calcium+ion+binding	9.38396E-05	0.0203819	0.000606391
CC_GO:0005783_endoplasmic+reticulum	0.968321	0.975772	0.729653
MF_GO:0003676_nucleic+acid+binding	0.999999	0.999995	0.993368
MF_GO:0016301_kinase+activity	0.971364	0.775273	0.973908
MF_GO:0003700_transcription+factor+activity	0.196289	0.674232	0.0473113
MF_GO:0004871_signal+transducer+activity	0.00588827	0.0405945	0.0330398
MF_GO:0003824_catalytic+activity	0.0336466	0.265094	0.0165544
MF_GO:0005488_binding	0.0846976	0.35987	0.0533305
BP_GO:0008152_metabolic+process	0.0894569	0.614236	0.00681177
CC_GO:0005794_Golgi+apparatus	0.826973	0.976711	0.209164
BP_GO:0006468_protein+amino+acid+phosphorylation	0.994892	0.872916	0.992787
MF_GO:0016491_oxidoreductase+activity	0.0534335	0.345346	0.0236615
BP_GO:0006811_ion+transport	0.00192689	0.356358	0.000122884
BP_GO:0055114_oxidation+reduction	0.0303088	0.255346	0.0181521
MF_GO:0004672_protein+kinase+activity	0.997246	0.917415	0.993858
MF_GO:0003723_RNA+binding	1	0.999925	0.999909
MF_GO:0004930_G-protein+coupled+receptor+activity	0.00344329	0.0305976	0.0235566
BP_GO:0030154_cell+differentiation	0.0203755	0.491992	0.00218318
BP_GO:0015031_protein+transport	0.999999	0.999956	0.997945

BP_GO:0006511_ubiquitin-dependent+protein+catabolic+process	1	0.999606	0.999982
BP_GO:0045449_regulation+of+transcription	0.318352	0.712024	0.104972
MF_GO:0043565_sequence-specific+DNA+binding	0.266783	0.731318	0.0631352
MF_GO:0008233_peptidase+activity	0.609112	0.662017	0.491152
BP_GO:0007049_cell+cycle	0.485794	0.537008	0.444581
BP_GO:0007155_cell+adhesion	8.48708E-08	0.00028598	3.79057E-05
CC_GO:0005856_cytoskeleton	0.806399	0.959782	0.306324
MF_GO:0004674_protein+serine/threonine+kinase+activity	0.999425	0.976472	0.995413
BP_GO:0006508_proteolysis	0.06334	0.0780788	0.225572
BP_GO:0006915_apoptosis	0.823737	0.795016	0.682531
CC_GO:0030054_cell+junction	0.000486552	0.108991	0.00029466
MF_GO:0000287_magnesium+ion+binding	0.616717	0.571396	0.596177
CC_GO:0005615_extracellular+space	0.000982982	0.17702	0.000588408
CC_GO:0005887_integral+to+plasma+membrane	0.0932909	0.0554036	0.350197
CC_GO:0030529_ribonucleoprotein+complex	1	0.999977	0.999192
BP_GO:0006412_translation	1	0.999988	0.999963
MF_GO:0005525_GTP+binding	0.891501	0.945677	0.490436
BP_GO:0045944_positive+regulation+of+transcription+from+RNA+polymera	0.552375	0.286931	0.772015
MF_GO:0005216_ion+channel+activity	0.000685809	0.137321	0.000332696
CC_GO:0005829_cytosol	0.998593	0.996297	0.918062
CC_GO:0005743_mitochondrial+inner+membrane	0.999994	0.999993	0.90069
BP_GO:0006955_immune+response	0.0674399	0.303164	0.0647909
MF_GO:0005506_iron+ion+binding	0.504943	0.610291	0.394127
MF_GO:0016874_ligase+activity	0.996222	0.951033	0.985475
MF_GO:0003779_actin+binding	0.166602	0.375673	0.141638
CC_GO:0005578_proteinaceous+extracellular+matrix	0.000923389	0.000185	0.154608
BP_GO:0006397_mRNA+processing	0.999939	0.983825	0.999914
MF_GO:0005215_transporter+activity	0.00698645	0.0816411	0.0202973
BP_GO:0007242_intracellular+signaling+cascade	0.0259704	0.0105183	0.351101
CC_GO:0005840_ribosome	1	0.999949	0.999257
CC_GO:0045202_synapse	0.130606	0.334717	0.117912
BP_GO:0006629_lipid+metabolic+process	0.437844	0.227299	0.727347
CC_GO:0005624_membrane+fraction	0.361346	0.989109	0.000865266
BP_GO:0051301_cell+division	0.391999	0.545793	0.321176
MF_GO:0030528_transcription+regulator+activity	0.224016	0.662305	0.0528491
BP_GO:0050896_response+to+stimulus	0.528656	0.485834	0.54552
BP_GO:0007264_small+GTPase+mediated+signal+transduction	0.940602	0.994141	0.221225
CC_GO:0005874_microtubule	0.948391	0.90956	0.829214
CC_GO:0031410_cytoplasmic+vesicle	0.799181	0.556208	0.84389
BP_GO:0008380_RNA+splicing	0.999706	0.973345	0.998494
MF_GO:0016757_transferase+activity,+transferring+glycosyl+groups	0.0627118	0.210899	0.0837661
BP_GO:0007283_spermatogenesis	0.037578	0.201322	0.0515551
CC_GO:0005667_transcription+factor+complex	0.582704	0.538625	0.581581
BP_GO:0006974_response+to+DNA+damage+stimulus	0.805143	0.735485	0.721741
BP_GO:0000122_negative+regulation+of+transcription+from+RNA+polymer	0.930425	0.859216	0.844885
MF_GO:0003735_structural+constituent+of+ribosome	1	0.999947	0.999435
BP_GO:0006281_DNA+repair	0.879125	0.922471	0.60522

MF_GO:0009055_electron+carrier+activity	0.77772	0.769833	0.63447
BP_GO:0007399_nervous+system+development	0.214514	0.675488	0.0452525
CC_GO:0005768_endosome	0.535777	0.706992	0.305494
CC_GO:0042995_cell+projection	0.20244	0.781655	0.0320756
MF_GO:0005198_structural+molecule+activity	3.69469E-05	0.00725423	0.000886272
BP_GO:0006886_intracellular+protein+transport	0.999715	0.999683	0.904203
MF_GO:0005125_cytokine+activity	0.112561	0.662777	0.0252665
CC_GO:0005694_chromosome	0.412425	0.876918	0.0605751
MF_GO:0005096_GTPase+activator+activity	0.598999	0.698364	0.442415
BP_GO:0005975_carbohydrate+metabolic+process	0.190768	0.403914	0.134451
MF_GO:0008415_acyltransferase+activity	0.687836	0.927155	0.179176
MF_GO:0005529_sugar+binding	0.00153033	0.0353109	0.00921333
MF_GO:0008236_serine-type+peptidase+activity	0.171329	0.177892	0.340749
BP_GO:0006813_potassium+ion+transport	0.350645	0.357301	0.42806
MF_GO:0004713_protein+tyrosine+kinase+activity	0.556087	0.514675	0.561311
MF_GO:0008237_metallopeptidase+activity	0.040465	0.236299	0.0480861
BP_GO:0007067_mitosis	0.294797	0.447005	0.275645
CC_GO:0005792_microsome	0.508442	0.667021	0.360596
CC_GO:0005764_lysosome	0.259028	0.464447	0.174774
BP_GO:0016192_vesicle-mediated+transport	0.983653	0.938067	0.931149
MF_GO:0016563_transcription+activator+activity	0.711888	0.406927	0.849489
BP_GO:0016568_chromatin+modification	0.977145	0.785577	0.979974
BP_GO:0001701_in+utero+embryonic+development	0.962616	0.991226	0.596921
MF_GO:0004252_serine-type+endopeptidase+activity	0.131705	0.0479104	0.549849
CC_GO:0009897_external+side+of+plasma+membrane	0.00724615	0.22704	0.00420412
MF_GO:0008083_growth+factor+activity	0.00690169	0.414561	0.00106816
MF_GO:0030145_manganese+ion+binding	0.430991	0.716952	0.148924
BP_GO:0007507_heart+development	0.604836	0.680251	0.446063
MF_GO:0005244_voltage-gated+ion+channel+activity	0.0120314	0.155656	0.0140881
BP_GO:0008284_positive+regulation+of+cell+proliferation	0.185429	0.0707297	0.595491
BP_GO:0008283_cell+proliferation	0.438311	0.527891	0.387233
BP_GO:0006954_inflammatory+response	0.00117085	0.00294816	0.0567584
CC_GO:0005681_spliceosome	0.999822	0.966479	0.999435
CC_GO:0031225_anchored+to+membrane	0.010811	0.0530864	0.0509094
CC_GO:0045211_postsynaptic+membrane	0.165338	0.217553	0.276806
CC_GO:0005882_intermediate+filament	0.0782769	0.107931	0.180055
MF_GO:0004721_phosphoprotein+phosphatase+activity	0.986681	0.991337	0.741808
MF_GO:0042803_protein+homodimerization+activity	0.0358364	0.315341	0.0177192
MF_GO:0017111_nucleoside-triphosphatase+activity	0.98785	0.945561	0.943732
BP_GO:0008285_negative+regulation+of+cell+proliferation	0.823675	0.952452	0.316157
MF_GO:0003774_motor+activity	0.196078	0.0859703	0.653814
BP_GO:0016055_Wnt+receptor+signaling+pathway	0.784911	0.8415	0.519148
MF_GO:0005085_guanyl-nucleotide+exchange+factor+activity	0.141128	0.280126	0.166959
BP_GO:0006464_protein+modification+process	0.97456	0.97105	0.75902
MF_GO:0016829_lyase+activity	0.0497394	0.40522	0.0143097
BP_GO:0006814_sodium+ion+transport	0.00884578	0.121431	0.0164348
MF_GO:0008168_methyltransferase+activity	0.788853	0.638387	0.790465

MF_GO:0030955_potassium+ion+binding	0.443822	0.32921	0.590689
MF_GO:0008289_lipid+binding	0.00991492	0.0240989	0.0906131
MF_GO:0003924_GTPase+activity	0.847978	0.870645	0.594865
MF_GO:0005516_calmodulin+binding	0.281508	0.129959	0.63547
MF_GO:0003682_chromatin+binding	0.561214	0.483115	0.592972
MF_GO:0016853_isomerase+activity	0.74935	0.717135	0.645658
MF_GO:0016791_phosphatase+activity	0.977786	0.771329	0.980302
MF_GO:0016564_transcription+repressor+activity	0.80093	0.823054	0.596193
MF_GO:0015293_symporter+activity	0.0637531	0.0820721	0.224259
BP_GO:0006260_DNA+replication	0.805884	0.701241	0.754106
BP_GO:0006470_protein+amino+acid+dephosphorylation	0.994196	0.986662	0.907102
BP_GO:0042981_regulation+of+apoptosis	0.594644	0.862297	0.166723
MF_GO:0005102_receptor+binding	0.396222	0.536284	0.33174
MF_GO:0008234_cysteine-type+peptidase+activity	0.998479	0.870694	0.999035
BP_GO:0006816_calcium+ion+transport	0.2818	0.629901	0.120711
BP_GO:0006457_protein+folding	0.999153	0.999719	0.73889
BP_GO:0006897_endocytosis	0.739736	0.96971	0.173327
CC_GO:0005730_nucleolus	0.999105	0.997903	0.94449
MF_GO:0020037_heme+binding	0.904153	0.387166	0.97899
MF_GO:0004866_endopeptidase+inhibitor+activity	0.00847748	0.00426424	0.163819
BP_GO:0009887_organ+morphogenesis	0.907778	0.755077	0.878494
BP_GO:0006357_regulation+of+transcription+from+RNA+polymerase+II+prc	0.704123	0.393538	0.860335
BP_GO:0019236_response+to+pheromone	0.991372	0.612502	0.995791
BP_GO:0043066_negative+regulation+of+apoptosis	0.672577	0.178751	0.955043
MF_GO:0005179_hormone+activity	0.0531375	0.102694	0.135773
MF_GO:0031402_sodium+ion+binding	0.00565524	0.074341	0.0178655
MF_GO:0004222_metalloendopeptidase+activity	0.457964	0.268421	0.639642
BP_GO:0008610_lipid+biosynthetic+process	0.0117559	0.065406	0.044695
MF_GO:0016503_pheromone+receptor+activity	0.987681	0.605241	0.99354
CC_GO:0005777_peroxisome	0.0414849	0.0129013	0.50579
BP_GO:0009952_anterior/posterior+pattern+formation	0.157337	0.557313	0.0778061
MF_GO:0004386_helicase+activity	0.921388	0.789381	0.878796
BP_GO:0022900_electron+transport+chain	0.996791	0.995994	0.798133
MF_GO:0004725_protein+tyrosine+phosphatase+activity	0.95024	0.913289	0.840592
BP_GO:0001525_angiogenesis	0.00928537	0.0336559	0.0676337
BP_GO:0016311_dephosphorylation	0.988913	0.839217	0.985362
MF_GO:0004842_ubiquitin-protein+ligase+activity	0.996874	0.936099	0.991237
CC_GO:0043234_protein+complex	0.92542	0.949127	0.555868
MF_GO:0005550_pheromone+binding	0.929117	0.704703	0.919635
MF_GO:0004867_serine-type+endopeptidase+inhibitor+activity	0.029666	0.0100517	0.268448
BP_GO:0007601_visual+perception	0.0786271	0.238077	0.101677
MF_GO:0004518_nuclease+activity	0.511787	0.451163	0.565607
MF_GO:0004497_monooxygenase+activity	0.915947	0.697965	0.906827
BP_GO:0045941_positive+regulation+of+transcription	0.67399	0.777439	0.436814
BP_GO:0016042_lipid+catabolic+process	0.38073	0.456487	0.375395
BP_GO:0006952_defense+response	0.000729957	0.0736942	0.00164866
MF_GO:0008201_heparin+binding	0.0522986	0.0444591	0.317709

BP_GO:0042127_regulation+of+cell+proliferation	0.255182	0.321718	0.319264
BP_GO:0045087_innate+immune+response	0.026024	0.12866	0.0525557
BP_GO:0006950_response+to+stress	0.953773	0.959271	0.695976
BP_GO:0007154_cell+communication	0.63542	0.410813	0.755406
BP_GO:0006334_nucleosome+assembly	0.788586	0.979977	0.0599939
BP_GO:0045786_negative+regulation+of+cell+cycle	0.486357	0.398396	0.590845
BP_GO:0006935_chemotaxis	0.140324	0.448689	0.0990993
BP_GO:0006917_induction+of+apoptosis	0.977569	0.994616	0.516889
MF_GO:0005249_voltage-gated+potassium+channel+activity	0.15621	0.186853	0.291106
CC_GO:0000786_nucleosome	0.362566	0.893099	0.0140789
CC_GO:0005929_cilium	0.063343	0.304738	0.0570348
BP_GO:0042742_defense+response+to+bacterium	3.36615E-05	0.000286817	0.00362381
CC_GO:0009986_cell+surface	0.000340319	0.155329	0.000242189
CC_GO:0005923_tight+junction	4.39994E-07	0.00155724	2.42966E-05
BP_GO:0007169_transmembrane+receptor+protein+tyrosine+kinase+signali	0.0816044	0.0788442	0.285351
BP_GO:0007218_neuropeptide+signaling+pathway	0.0838845	0.585298	0.0280429
BP_GO:0006631_fatty+acid+metabolic+process	0.0477203	0.0552009	0.285109
BP_GO:0030198_extracellular+matrix+organization	0.132743	0.0844544	0.401977
CC_GO:0030424_axon	0.448814	0.391209	0.533189
BP_GO:0007229_integrin-mediated+signaling+pathway	0.207202	0.54719	0.12649
MF_GO:0046982_protein+heterodimerization+activity	0.290775	0.191936	0.56279
BP_GO:0007411_axon+guidance	0.057568	0.169239	0.104183
MF_GO:0046983_protein+dimerization+activity	0.54951	0.821933	0.206573
MF_GO:0016887_ATPase+activity	0.93441	0.892987	0.810944
CC_GO:0043025_cell+soma	0.8278	0.942704	0.456618
MF_GO:0042802_identical+protein+binding	0.129397	0.388078	0.0784724
BP_GO:0001558_regulation+of+cell+growth	0.611379	0.299197	0.873191
MF_GO:0005267_potassium+channel+activity	0.124674	0.24475	0.177514
MF_GO:0017124_SH3+domain+binding	0.743627	0.369372	0.881948
MF_GO:0016798_hydrolase+activity,+acting+on+glycosyl+bonds	0.243769	0.485545	0.143974
BP_GO:0030036_actin+cytoskeleton+organization	0.571038	0.520751	0.582619
BP_GO:0030324_lung+development	0.484708	0.560908	0.418424
CC_GO:0005741_mitochondrial+outer+membrane	0.891885	0.923275	0.563761
BP_GO:0007166_cell+surface+receptor+linked+signal+transduction	0.0481787	0.154752	0.0915146
BP_GO:0007018_microtubule-based+movement	0.224778	0.134623	0.558926
BP_GO:0007626_locomotory+behavior	0.0659877	0.292179	0.0683409
BP_GO:0007605_sensory+perception+of+sound	0.160063	0.298785	0.184648
MF_GO:0016779_nucleotidyltransferase+activity	0.908696	0.712223	0.920463
MF_GO:0031404_chloride+ion+binding	0.173875	0.470665	0.122167
CC_GO:0031012_extracellular+matrix	0.00237445	0.00199067	0.123426
CC_GO:0016324_apical+plasma+membrane	0.05535	0.362174	0.0275744
BP_GO:0007420_brain+development	0.0624598	0.0340752	0.351092
MF_GO:0003743_translation+initiation+factor+activity	0.997447	0.972398	0.979946
CC_GO:0005604_basement+membrane	0.0395977	0.00115487	0.674099
MF_GO:0008134_transcription+factor+binding	0.838827	0.294472	0.992566
BP_GO:0016481_negative+regulation+of+transcription	0.766839	0.790949	0.564958
BP_GO:0006364_rRNA+processing	0.762952	0.677324	0.709446

BP_GO:0043065_positive+regulation+of+apoptosis	0.919952	0.907661	0.714457
BP_GO:0001501_skeletal+system+development	0.788467	0.574286	0.826248
MF_GO:0003713_transcription+coactivator+activity	0.981822	0.803138	0.983799
BP_GO:0006916_anti-apoptosis	0.956962	0.850731	0.918898
BP_GO:0045893_positive+regulation+of+transcription,+DNA-dependent	0.809378	0.505435	0.880811
BP_GO:0065002_intracellular+protein+transmembrane+transport	0.98634	0.96735	0.894466
BP_GO:0030900_forebrain+development	0.950403	0.952239	0.749606
MF_GO:0051082_unfolded+protein+binding	0.997583	0.99949	0.573451
BP_GO:0007268_synaptic+transmission	0.605673	0.99646	0.00583368
BP_GO:0007156_homophilic+cell+adhesion	0.0910512	0.415084	0.054766
CC_GO:0005625_soluble+fraction	0.264701	0.497313	0.157597
MF_GO:0016702_oxidoreductase+activity,+acting+on+single+donors+with+i	0.417905	0.700386	0.193533
MF_GO:0003714_transcription+corepressor+activity	0.961236	0.685499	0.98455
BP_GO:0001764_neuron+migration	0.399153	0.357092	0.492167
MF_GO:0050660_FAD+binding	0.44919	0.850615	0.0803444
MF_GO:0005089_Rho+guanyl-nucleotide+exchange+factor+activity	0.176506	0.299055	0.206904
CC_GO:0019717_synaptosome	0.727504	0.426443	0.832899
CC_GO:0008076_voltage-gated+potassium+channel+complex	0.183675	0.254763	0.267414
MF_GO:0005262_calcium+channel+activity	0.174674	0.367573	0.161802
MF_GO:0005254_chloride+channel+activity	0.359185	0.60725	0.240938
MF_GO:0008565_protein+transporter+activity	0.910256	0.887723	0.748837
BP_GO:0001503_ossification	0.0525357	0.173867	0.091412
MF_GO:0019992_diacylglycerol+binding	0.228751	0.0824298	0.620597
BP_GO:0035023_regulation+of+Rho+protein+signal+transduction	0.145752	0.181371	0.28822
CC_GO:0015629_actin+cytoskeleton	0.184249	0.256662	0.267532
MF_GO:0003777_microtubule+motor+activity	0.441928	0.185201	0.785765
MF_GO:0008026_ATP-dependent+helicase+activity	0.992742	0.957839	0.959613
MF_GO:0004888_transmembrane+receptor+activity	0.00774319	0.00861803	0.142496
MF_GO:0004714_transmembrane+receptor+protein+tyrosine+kinase+activi	0.226296	0.0594241	0.613616
MF_GO:0005507_copper+ion+binding	0.045202	0.262822	0.0381133
BP_GO:0009790_embryonic+development	0.961354	0.917608	0.864976
BP_GO:0030326_embryonic+limb+morphogenesis	0.981651	0.994397	0.640872
BP_GO:0009791_post-embryonic+development	0.881304	0.799648	0.79696
MF_GO:0004519_endonuclease+activity	0.132976	0.28648	0.161669
BP_GO:0007409_axonogenesis	0.24442	0.527899	0.134552
BP_GO:0042254_ribosome+biogenesis	0.982146	0.965852	0.863972
BP_GO:0008033_tRNA+processing	0.629679	0.764252	0.359611
CC_GO:0005746_mitochondrial+respiratory+chain	0.999567	0.99762	0.977839
BP_GO:0007219_Notch+signaling+pathway	0.182124	0.0384685	0.730683
BP_GO:0042472_inner+ear+morphogenesis	0.164658	0.647779	0.035812
BP_GO:0045892_negative+regulation+of+transcription,+DNA-dependent	0.579201	0.655642	0.436512
BP_GO:0006694_steroid+biosynthetic+process	0.00582018	0.000462934	0.285297
BP_GO:0009058_biosynthetic+process	0.875356	0.784868	0.800329
BP_GO:0007389_pattern+specification+process	0.390732	0.788372	0.112485
BP_GO:0045454_cell+redox+homeostasis	0.997671	0.994893	0.910671
MF_GO:0004221_ubiquitin+thiolesterase+activity	0.997714	0.9575	0.98791
BP_GO:0030182_neuron+differentiation	0.723128	0.584453	0.737618

CC_GO:0030027_lamellipodium	0.921952	0.996032	0.258271
BP_GO:0051726_regulation+of+cell+cycle	0.586971	0.691797	0.38959
CC_GO:0008021_synaptic+vesicle	0.0929232	0.176656	0.170429
MF_GO:0030170_pyridoxal+phosphate+binding	0.378301	0.594711	0.213304
BP_GO:0006633_fatty+acid+biosynthetic+process	0.276839	0.402605	0.275591
BP_GO:0016337_cell-cell+adhesion	0.0623598	0.00430758	0.620321
BP_GO:0008104_protein+localization	0.111409	0.253011	0.13678
BP_GO:0010468_regulation+of+gene+expression	0.958846	0.201787	0.998784
BP_GO:0006417_regulation+of+translation	0.989678	0.825139	0.987329
BP_GO:0006486_protein+amino+acid+glycosylation	0.157992	0.234103	0.242684
BP_GO:0008202_steroid+metabolic+process	0.893545	0.586814	0.935328
CC_GO:0016459_myosin+complex	0.0134336	0.0229898	0.15957
BP_GO:0007519_skeletal+muscle+development	0.95043	0.954546	0.697045
MF_GO:0004197_cysteine-type+endopeptidase+activity	0.832889	0.491371	0.939853
MF_GO:0035091_phosphoinositide+binding	0.956558	0.810272	0.938574
BP_GO:0051246_regulation+of+protein+metabolic+process	0.993221	0.694464	0.998338
BP_GO:0006812_cation+transport	0.27857	0.879857	0.0297825
BP_GO:0006821_chloride+transport	0.794781	0.933781	0.407595
BP_GO:0015992_proton+transport	0.670814	0.786524	0.320969
MF_GO:0019787_small+conjugating+protein+ligase+activity	0.998603	0.889452	0.998755
CC_GO:0005643_nuclear+pore	0.920186	0.910447	0.715797
BP_GO:0043687_post-translational+protein+modification	0.998823	0.810715	0.999626
MF_GO:0003707_steroid+hormone+receptor+activity	0.77804	0.871874	0.426091
BP_GO:0006869_lipid+transport	0.090045	0.159574	0.180547
BP_GO:0007596_blood+coagulation	0.362995	0.715759	0.146858
BP_GO:0008203_cholesterol+metabolic+process	0.188106	0.157819	0.417178
BP_GO:0008360_regulation+of+cell+shape	0.257681	0.290092	0.350589
BP_GO:0007050_cell+cycle+arrest	0.0805759	0.375586	0.0491971
MF_GO:0004879_ligand-dependent+nuclear+receptor+activity	0.816724	0.871874	0.507022
CC_GO:0005875_microtubule+associated+complex	0.823862	0.262325	0.989589
BP_GO:0045165_cell+fate+commitment	0.179106	0.441976	0.121769
CC_GO:0005789_endoplasmic+reticulum+membrane	0.972335	0.744965	0.985121
CC_GO:0005654_nucleoplasm	0.971283	0.987624	0.691377
BP_GO:0007126_meiosis	0.919369	0.948132	0.701653
BP_GO:0050909_sensory+perception+of+taste	0.735805	0.89517	0.482906
BP_GO:0006754_ATP+biosynthetic+process	0.651279	0.759017	0.402315
BP_GO:0006979_response+to+oxidative+stress	0.444611	0.139668	0.865187
MF_GO:0015078_hydrogen+ion+transmembrane+transporter+activity	0.864236	0.896265	0.515277
CC_GO:0048471_perinuclear+region+of+cytoplasm	0.450169	0.2802	0.660971
BP_GO:0030097_hemopoiesis	0.421715	0.567937	0.312453
CC_GO:0000785_chromatin	0.607097	0.387897	0.744701
BP_GO:0007267_cell-cell+signaling	0.803691	0.657277	0.787973
BP_GO:0051028_mRNA+transport	0.880349	0.911838	0.590615
MF_GO:0008017_microtubule+binding	0.765437	0.854656	0.561554
CC_GO:0005635_nuclear+envelope	0.62697	0.568841	0.613951
CC_GO:0016023_cytoplasmic+membrane-bounded+vesicle	0.768163	0.753753	0.64564
BP_GO:0048704_embryonic+skeletal+system+morphogenesis	0.252966	0.540357	0.154244

BP_GO:0006887_exocytosis	0.679385	0.782387	0.434756
CC_GO:0000775_chromosome,+centromeric+region	0.0479408	0.457072	0.0154455
BP_GO:0009953_dorsal/ventral+pattern+formation	0.92763	0.973473	0.567123
MF_GO:0004812_aminoacyl-tRNA+ligase+activity	0.766935	0.767568	0.58768
BP_GO:0006096_glycolysis	0.981807	0.968098	0.839142
BP_GO:0006006_glucose+metabolic+process	0.443627	0.454464	0.465913
BP_GO:0046777_protein+amino+acid+autophosphorylation	0.915643	0.623135	0.935288
CC_GO:0045121_membrane+raft	0.122302	0.415309	0.0817467
MF_GO:0051536_iron-sulfur+cluster+binding	0.585373	0.260276	0.842431
CC_GO:0000139_Golgi+membrane	0.89003	0.80526	0.812422
CC_GO:0030425_dendrite	0.178934	0.212359	0.292023
MF_GO:0004527_exonuclease+activity	0.786274	0.824648	0.542807
BP_GO:0016477_cell+migration	0.47881	0.863249	0.078813
CC_GO:0005938_cell+cortex	0.803947	0.638834	0.836408
BP_GO:0001568_blood+vessel+development	0.0187073	0.0200952	0.163753
BP_GO:0006874_cellular+calcium+ion+homeostasis	0.95525	0.971116	0.726996
BP_GO:0001822_kidney+development	0.591264	0.87272	0.207898
BP_GO:0043524_negative+regulation+of+neuron+apoptosis	0.254669	0.459524	0.215839
CC_GO:0005813_centrosome	0.928001	0.750417	0.90934
BP_GO:0009615_response+to+virus	0.41703	0.987999	0.0208221
BP_GO:0006836_neurotransmitter+transport	0.167394	0.153368	0.353362
BP_GO:0007010_cytoskeleton+organization	0.0580783	0.306919	0.0356512
BP_GO:0006396_RNA+processing	0.976813	0.893135	0.951285
CC_GO:0030018_Z+disc	0.00424238	0.148868	0.000640074
BP_GO:0007243_protein+kinase+cascade	0.975937	0.934308	0.900685
CC_GO:0045177_apical+part+of+cell	0.017287	0.361024	0.00820197
BP_GO:0051216_cartilage+development	0.391284	0.671677	0.235706
MF_GO:0031072_heat+shock+protein+binding	0.999714	0.997238	0.979988
BP_GO:0019221_cytokine-mediated+signaling+pathway	0.492364	0.315011	0.662331
BP_GO:0009968_negative+regulation+of+signal+transduction	0.235805	0.0593751	0.641308
CC_GO:0005802_trans-Golgi+network	0.993855	0.840048	0.99729
CC_GO:0016323_basolateral+plasma+membrane	0.229152	0.128705	0.526515
MF_GO:0005097_Rab+GTPase+activator+activity	0.987641	0.979182	0.861072
BP_GO:0043010_camera-type+eye+development	0.0809162	0.68291	0.00941212
MF_GO:0003702_RNA+polymerase+II+transcription+factor+activity	0.804352	0.901965	0.446356
BP_GO:0007179_transforming+growth+factor+beta+receptor+signaling+pat	0.867635	0.926341	0.507733
BP_GO:0006139_nucleobase,+nucleoside,+nucleotide+and+nucleic+acid+me	0.0658169	0.271003	0.0535641
MF_GO:0008092_cytoskeletal+protein+binding	0.57578	0.904599	0.208631
MF_GO:0004715_non-membrane+spanning+protein+tyrosine+kinase+activit	0.611832	0.702516	0.467876
MF_GO:0008009_chemokine+activity	0.0976096	0.293727	0.113634
MF_GO:0004175_endopeptidase+activity	0.999039	0.971099	0.995206
BP_GO:0006310_DNA+recombination	0.674502	0.74016	0.524366
MF_GO:0005230_extracellular+ligand-gated+ion+channel+activity	0.678389	0.702583	0.548805
MF_GO:0030594_neurotransmitter+receptor+activity	0.678389	0.702583	0.548805
BP_GO:0007160_cell-matrix+adhesion	0.306838	0.866514	0.0310321
MF_GO:0016773_phosphotransferase+activity,+alcohol+group+as+acceptor	0.423674	0.58706	0.320702
BP_GO:0042593_glucose+homeostasis	0.944773	0.9751	0.641701

BP_GO:0008344_adult+locomotory+behavior	0.61684	0.223125	0.886468
BP_GO:0001570_vasculogenesis	0.00701279	0.00083085	0.445927
BP_GO:0042475_odontogenesis+of+dentine-containing+tooth	0.646028	0.860481	0.282391
BP_GO:0006418_tRNA+aminoacylation+for+protein+translation	0.489657	0.418172	0.58768
BP_GO:0051056_regulation+of+small+GTPase+mediated+signal+transductio	0.858523	0.740712	0.807113
MF_GO:0008138_protein+tyrosine/serine/threonine+phosphatase+activity	0.958563	0.901277	0.879466
BP_GO:0015986_ATP+synthesis+coupled+proton+transport	0.777625	0.948229	0.0979318
BP_GO:0008643_carbohydrate+transport	0.0444406	0.455478	0.0108155
BP_GO:0001756_somitogenesis	0.835979	0.661978	0.823107
MF_GO:0005351_sugar:hydrogen+symporter+activity	0.039279	0.455478	0.00869021
BP_GO:0008654_phospholipid+biosynthetic+process	0.446255	0.135166	0.907321
MF_GO:0019904_protein+domain+specific+binding	0.0361761	0.0389401	0.275763
BP_GO:0006865_amino+acid+transport	0.0113349	0.448316	0.000730344
BP_GO:0001889_liver+development	0.97801	0.259463	0.999763
BP_GO:0007368_determination+of+left/right+symmetry	0.945838	0.739851	0.948291
BP_GO:0007417_central+nervous+system+development	0.790202	0.958041	0.496789
MF_GO:0003755_peptidyl-prolyl+cis-trans+isomerase+activity	0.977274	0.933892	0.906321
BP_GO:0042391_regulation+of+membrane+potential	0.310045	0.492149	0.241303
CC_GO:0001726_ruffle	0.555225	0.146189	0.923262
BP_GO:0000902_cell+morphogenesis	0.654375	0.134152	0.92155
BP_GO:0032313_regulation+of+Rab+GTPase+activity	0.974753	0.923904	0.908758
CC_GO:0005834_heterotrimeric+G-protein+complex	0.753304	0.801528	0.517916
BP_GO:0007286_spermatid+development	0.75945	0.556888	0.781781
MF_GO:0003899_DNA-directed+RNA+polymerase+activity	0.709262	0.455808	0.843086
MF_GO:0004091_carboxylesterase+activity	0.0889354	0.265789	0.0922766
BP_GO:0006730_one-carbon+compound+metabolic+process	0.308895	0.621617	0.154608
MF_GO:0004180_carboxypeptidase+activity	0.000157212	0.0424439	0.000556923
MF_GO:0004896_cytokine+receptor+activity	0.0750394	0.510062	0.0417556
BP_GO:0000187_activation+of+MAPK+activity	0.330845	0.460602	0.308977
BP_GO:0000226_microtubule+cytoskeleton+organization	0.539987	0.644344	0.348384
MF_GO:0008146_sulfotransferase+activity	0.326057	0.708993	0.10701
BP_GO:0019882_antigen+processing+and+presentation	0.985046	0.99552	0.732146
BP_GO:0007517_muscle+development	0.219676	0.749769	0.0295756
BP_GO:0018108_peptidyl-tyrosine+phosphorylation	0.231344	0.0968688	0.575432
CC_GO:0030117_membrane+coat	0.891021	0.659473	0.894553
BP_GO:0007017_microtubule-based+process	0.31975	0.434644	0.280815
BP_GO:0016567_protein+ubiquitination	0.928294	0.968593	0.43487
BP_GO:0030216_keratinocyte+differentiation	0.120216	0.0693041	0.420396
MF_GO:0050381_unspecific+monooxygenase+activity	0.529657	0.208748	0.737347
MF_GO:0016820_hydrolase+activity,+acting+on+acid+anhydrides,+catalyzin	0.615166	0.655229	0.505539
BP_GO:0001843_neural+tube+closure	0.816248	0.960447	0.315033
BP_GO:0042493_response+to+drug	0.462214	0.347911	0.57895
BP_GO:0030163_protein+catabolic+process	0.916906	0.757085	0.903773
MF_GO:0051287_NAD+binding	0.915772	0.83104	0.850021
BP_GO:0030334_regulation+of+cell+migration	0.125378	0.410052	0.0890816
BP_GO:0000165_MAPKKK+cascade	0.511186	0.291422	0.800867
BP_GO:0050885_neuromuscular+process+controlling+balance	0.133144	0.386306	0.0964993

CC_GO:0030173_integral+to+Golgi+membrane	0.0241893	0.0774097	0.0849287
MF_GO:0004693_cyclin-dependent+protein+kinase+activity	0.692589	0.270985	0.859461
BP_GO:0030879_mammary+gland+development	0.151362	0.0735094	0.566095
CC_GO:0005925_focal+adhesion	0.499111	0.723716	0.215998
MF_GO:0043169_cation+binding	0.169988	0.276421	0.216526
CC_GO:0005905_coated+pit	0.108101	0.27524	0.115399
BP_GO:0003007_heart+morphogenesis	0.671715	0.654591	0.593381
BP_GO:0048706_embryonic+skeletal+system+development	0.925963	0.905274	0.793734
MF_GO:0008080_N-acetyltransferase+activity	0.843864	0.973387	0.335593
BP_GO:0043473_pigmentation	0.411858	0.231018	0.609661
CC_GO:0019898_extrinsic+to+membrane	0.663987	0.790726	0.50257
MF_GO:0003887_DNA-directed+DNA+polymerase+activity	0.590589	0.520345	0.62695
CC_GO:0030141_secretory+granule	0.998644	0.999801	0.727329
CC_GO:0019861_flagellum	0.112572	0.434063	0.0369008
BP_GO:0006506_GPI+anchor+biosynthetic+process	0.656442	0.353712	0.816448
BP_GO:0008544_epidermis+development	0.391871	0.108147	0.743376
BP_GO:0031424_keratinization	0.310104	0.452222	0.30336
MF_GO:0000155_two-component+sensor+activity	0.582767	0.574736	0.541766
BP_GO:0009987_cellular+process	0.989069	0.959302	0.932057
BP_GO:0030183_B+cell+differentiation	0.109085	0.0931245	0.319702
MF_GO:0019901_protein+kinase+binding	0.123964	0.633936	0.0217229
BP_GO:0035264_multicellular+organism+growth	0.218862	0.0857312	0.740324
BP_GO:0006413_translational+initiation	0.942674	0.948447	0.720331
BP_GO:0006366_transcription+from+RNA+polymerase+II+promoter	0.981208	0.926988	0.931746
MF_GO:0003746_translation+elongation+factor+activity	0.993826	0.993661	0.795583
MF_GO:0005319_lipid+transporter+activity	0.00787443	0.0257035	0.0654802
MF_GO:0016712_oxidoreductase+activity,+acting+on+paired+donors,+with-	0.35816	0.22568	0.530245
BP_GO:0001666_response+to+hypoxia	0.0298888	0.164791	0.0398228
MF_GO:0004722_protein+serine/threonine+phosphatase+activity	0.954415	0.989516	0.393299
MF_GO:0015662_ATPase+activity,+coupled+to+transmembrane+movement	0.268904	0.478371	0.198315
MF_GO:0042626_ATPase+activity,+coupled+to+transmembrane+movement	0.890298	0.959823	0.469732
CC_GO:0043005_neuron+projection	0.958678	0.920758	0.846929
BP_GO:0040007_growth	0.143832	0.577183	0.026122
BP_GO:0045596_negative+regulation+of+cell+differentiation	0.00226192	0.0837164	0.00469905
MF_GO:0004177_aminopeptidase+activity	0.91485	0.965062	0.596609
MF_GO:0005044_scavenger+receptor+activity	0.298175	0.0604798	0.812482
CC_GO:0000151_ubiquitin+ligase+complex	0.942707	0.84366	0.889212
BP_GO:0016358_dendrite+development	0.761278	0.558251	0.786242
MF_GO:0003684_damaged+DNA+binding	0.777746	0.764041	0.646056
MF_GO:0008430_selenium+binding	0.286677	0.151305	0.775419
CC_GO:0042383_sarcolemma	0.1367	0.159385	0.303044
MF_GO:0005200_structural+constituent+of+cytoskeleton	0.336648	0.570924	0.202183
BP_GO:0001702_gastrulation+with+mouth+forming+second	0.436336	0.81963	0.166835
BP_GO:0006605_protein+targeting	0.845006	0.721035	0.811637
BP_GO:0019538_protein+metabolic+process	0.381576	0.919795	0.0151124
BP_GO:0048705_skeletal+system+morphogenesis	0.858492	0.641388	0.893496
MF_GO:0005496_steroid+binding	0.592361	0.881126	0.165868

BP_GO:0048754_branching+morphogenesis+of+a+tube	0.144218	0.236081	0.216802
MF_GO:0016881_acid-amino+acid+ligase+activity	0.868791	0.779254	0.794052
MF_GO:0003705_RNA+polymerase+II+transcription+factor+activity,+enhanc	0.505228	0.690616	0.280659
BP_GO:0007281_germ+cell+development	0.371754	0.223793	0.623718
BP_GO:0040018_positive+regulation+of+multicellular+organism+growth	0.249104	0.379771	0.25458
BP_GO:0008542_visual+learning	0.465879	0.760961	0.124345
BP_GO:0045665_negative+regulation+of+neuron+differentiation	0.194288	0.245536	0.300741
BP_GO:0006333_chromatin+assembly+or+disassembly	0.904609	0.332759	0.971776
BP_GO:0001890_placenta+development	0.286491	0.151285	0.64015
CC_GO:0030286_dynein+complex	0.662373	0.72486	0.463621
CC_GO:0016529_sarcoplasmic+reticulum	0.007053	0.0546857	0.0212929
BP_GO:0006958_complement+activation,+classical+pathway	0.197471	0.223475	0.336638
MF_GO:0051015_actin+filament+binding	0.421514	0.265723	0.719325
CC_GO:0008305_integrin+complex	0.0441853	0.244727	0.0460191
BP_GO:0032496_response+to+lipopolysaccharide	0.332112	0.801332	0.0486934
BP_GO:0006606_protein+import+into+nucleus	0.505412	0.809852	0.137969
MF_GO:0045028_purinergic+nucleotide+receptor+activity,+G-protein+coupl	0.0680365	0.0460214	0.300803
BP_GO:0048469_cell+maturation	0.0353377	0.350146	0.00752382
BP_GO:0006338_chromatin+remodeling	0.735872	0.48088	0.816838
BP_GO:0007628_adult+walking+behavior	0.352891	0.69798	0.159672
CC_GO:0005921_gap+junction	0.0504953	0.342024	0.0240036
MF_GO:0003725_double-stranded+RNA+binding	0.90664	0.809955	0.838156
MF_GO:0003729_mRNA+binding	0.973091	0.972276	0.71265
BP_GO:0030317_sperm+motility	0.230793	0.497976	0.14968
BP_GO:0005977_glycogen+metabolic+process	0.996284	0.988923	0.917998
BP_GO:0010811_positive+regulation+of+cell-substrate+adhesion	0.420867	0.620711	0.334452
BP_GO:0016126_sterol+biosynthetic+process	0.000726355	0.00118679	0.0588097
BP_GO:0006826_iron+ion+transport	0.00452166	0.0879176	0.00907937
CC_GO:0005911_cell-cell+junction	0.22422	0.642209	0.120969
BP_GO:0001658_ureteric+bud+branching	0.742959	0.732469	0.636881
MF_GO:0016758_transferase+activity,+transferring+hexosyl+groups	0.35051	0.365842	0.419045
BP_GO:0001656_metanephros+development	0.528105	0.93006	0.112199
BP_GO:0006914_autophagy	0.978684	0.633846	0.997348
BP_GO:0030030_cell+projection+organization	0.613205	0.777852	0.333518
CC_GO:0001669_acrosome	0.33884	0.266201	0.488768
MF_GO:0004364_glutathione+transferase+activity	0.00455485	0.00490941	0.161485
MF_GO:0008483_transaminase+activity	0.638601	0.82133	0.217918
CC_GO:0030863_cortical+cytoskeleton	0.447668	0.556478	0.396513
BP_GO:0006953_acute-phase+response	0.0996888	0.0163839	0.511176
BP_GO:0001649_osteoblast+differentiation	0.941406	0.848693	0.88109
BP_GO:0008217_regulation+of+blood+pressure	0.721113	0.853372	0.368347
CC_GO:0005769_early+endosome	0.996612	0.989983	0.9196
BP_GO:0045444_fat+cell+differentiation	0.913766	0.942215	0.639098
BP_GO:0030073_insulin+secretion	0.174057	0.143162	0.409562
BP_GO:0009954_proximal/distal+pattern+formation	0.848292	0.981386	0.33984
MF_GO:0004601_peroxidase+activity	0.107403	0.251789	0.0781656
MF_GO:0015297_antiporter+activity	0.65744	0.419686	0.738659

BP_GO:0000160_two-component+signal+transduction+system+(phosphorel	0.641962	0.669558	0.541766
BP_GO:0050731_positive+regulation+of+peptidyl-tyrosine+phosphorylation	0.0557119	0.0349561	0.309206
BP_GO:0048663_neuron+fate+commitment	0.618954	0.817028	0.295787
BP_GO:0007266_Rho+protein+signal+transduction	0.934339	0.985356	0.422062
BP_GO:0006695_cholesterol+biosynthetic+process	0.000717887	0.000584579	0.110812
BP_GO:0007015_actin+filament+organization	0.191532	0.111375	0.463073
CC_GO:0005770_late+endosome	0.219868	0.210477	0.41687
BP_GO:0001755_neural+crest+cell+migration	0.00326175	0.0105497	0.0632225
BP_GO:0019835_cytolysis	0.0765533	0.0125229	0.565552
BP_GO:0009636_response+to+toxin	0.335862	0.1146	0.637503
BP_GO:0051258_protein+polymerization	0.195512	0.289656	0.242767
BP_GO:0009117_nucleotide+metabolic+process	0.372103	0.188909	0.652222
CC_GO:0031224_intrinsic+to+membrane	0.0109919	0.066731	0.0414905
MF_GO:0016788_hydrolase+activity,+acting+on+ester+bonds	0.0788837	0.253374	0.10272
BP_GO:0040014_regulation+of+multicellular+organism+growth	0.744543	0.870245	0.386219
BP_GO:0001569_patterning+of+blood+vessels	0.0558209	0.1935	0.0869811
MF_GO:0004553_hydrolase+activity,+hydrolyzing+O-glycosyl+compounds	0.892834	0.794373	0.825745
CC_GO:0030131_clathrin+adaptor+complex	0.57394	0.826802	0.237355
BP_GO:0030178_negative+regulation+of+Wnt+receptor+signaling+pathway	0.615634	0.40241	0.73986
BP_GO:0007189_G-protein+signaling,+adenylate+cyclase+activating+pathwa	0.0577121	0.399347	0.0387924
BP_GO:0007610_behavior	0.87785	0.661592	0.872284
BP_GO:0007059_chromosome+segregation	0.743788	0.542287	0.768261
BP_GO:0048666_neuron+development	0.811764	0.75405	0.719852
BP_GO:0009566_fertilization	0.678845	0.400468	0.799064
CC_GO:0045095_keratin+filament	0.228171	0.419362	0.22765
BP_GO:0042632_cholesterol+homeostasis	0.929342	0.875267	0.826565
MF_GO:0004857_enzyme+inhibitor+activity	0.332366	0.112765	0.81154
CC_GO:0030426_growth+cone	0.311773	0.135228	0.585582
MF_GO:0016772_transferase+activity,+transferring+phosphorus-containing-	0.244824	0.089916	0.769605
MF_GO:0004114_3',5'-cyclic-nucleotide+phosphodiesterase+activity	0.0273298	0.0533133	0.109851
BP_GO:0015758_glucose+transport	0.0316903	0.386487	0.0121354
BP_GO:0006879_cellular+iron+ion+homeostasis	0.458923	0.285188	0.617356
MF_GO:0016831_carboxy-lyase+activity	0.863233	0.733601	0.817377
BP_GO:0008286_insulin+receptor+signaling+pathway	0.276197	0.520284	0.197006
BP_GO:0006829_zinc+ion+transport	0.758171	0.866246	0.452789
BP_GO:0042552_myelination	0.112227	0.246302	0.124683
MF_GO:0005083_small+GTPase+regulator+activity	0.96373	0.961722	0.765467
BP_GO:0000082_G1/S+transition+of+mitotic+cell+cycle	0.816094	0.483784	0.905593
MF_GO:0018024_histone-lysine+N-methyltransferase+activity	0.529575	0.377505	0.609183
BP_GO:0006986_response+to+unfolded+protein	0.941879	0.91326	0.826559
BP_GO:0007369_gastrulation	0.345884	0.816612	0.119011
BP_GO:0006919_caspase+activation	0.787449	0.439348	0.962248
BP_GO:0006259_DNA+metabolic+process	0.33457	0.182874	0.558267
BP_GO:0008584_male+gonad+development	0.166273	0.377929	0.151516
BP_GO:0001892_embryonic+placenta+development	0.790582	0.785699	0.615265
CC_GO:0005839_proteasome+core+complex	0.990461	0.984796	0.830069
MF_GO:0004298_threonine-type+endopeptidase+activity	0.990461	0.984796	0.830069

MF_GO:0005272_sodium+channel+activity	0.000801369	0.270408	0.00023615
MF_GO:0004890_GABA-A+receptor+activity	0.863335	0.81321	0.753243
BP_GO:0008652_amino+acid+biosynthetic+process	0.347359	0.316037	0.489264
MF_GO:0016616_oxidoreductase+activity,+acting+on+the+CH-OH+group+of	0.325283	0.501899	0.17377
MF_GO:0005201_extracellular+matrix+structural+constituent	0.0319483	0.0671455	0.131913
BP_GO:0030335_positive+regulation+of+cell+migration	0.368774	0.154282	0.64484
BP_GO:0007265_Ras+protein+signal+transduction	0.356231	0.405186	0.377591
MF_GO:0004181_metallocarboxypeptidase+activity	6.18301E-05	0.0173064	0.00057195
BP_GO:0007005_mitochondrion+organization	0.851495	0.80423	0.72693
BP_GO:0006909_phagocytosis	0.141192	0.240388	0.206
CC_GO:0005913_cell-cell+adherens+junction	0.127533	0.508595	0.0470565
BP_GO:0021987_cerebral+cortex+development	0.982264	0.920962	0.940697
BP_GO:0001657_ureteric+bud+development	0.974049	0.893684	0.940829
BP_GO:0030855_epithelial+cell+differentiation	0.421316	0.105751	0.872556
MF_GO:0008373_sialyltransferase+activity	0.0643221	0.059162	0.270044
BP_GO:0043588_skin+development	0.212001	0.322448	0.25459
MF_GO:0016627_oxidoreductase+activity,+acting+on+the+CH-CH+group+of	0.542464	0.972105	0.0116804
BP_GO:0001707_mesoderm+formation	0.841769	0.949312	0.394864
CC_GO:0005740_mitochondrial+envelope	0.9426	0.979572	0.388321
BP_GO:0048468_cell+development	0.153779	0.384708	0.120351
BP_GO:0006913_nucleocytoplasmic+transport	0.866173	0.986395	0.11419
CC_GO:0005819_spindle	0.435227	0.508858	0.402216
BP_GO:0031175_neurite+development	0.0473693	0.413187	0.00752808
MF_GO:0003697_single-stranded+DNA+binding	0.883928	0.713798	0.856384
BP_GO:0048511_rhythmic+process	0.561589	0.595972	0.462022
BP_GO:0001541_ovarian+follicle+development	0.031265	0.083515	0.102086
CC_GO:0000119_mediator+complex	0.946335	0.90315	0.831347
BP_GO:0007223_Wnt+receptor+signaling+pathway,+calcium+modulating+p	0.911103	0.518936	0.959209
CC_GO:0009434_microtubule-based+flagellum	0.202754	0.399622	0.195123
BP_GO:0007606_sensory+perception+of+chemical+stimulus	0.9154	0.839471	0.882425
BP_GO:0034097_response+to+cytokine+stimulus	0.232725	0.280453	0.31952
BP_GO:0006941_striated+muscle+contraction	0.577969	0.309378	0.843799
MF_GO:0005520_insulin-like+growth+factor+binding	0.282487	0.582199	0.0852966
BP_GO:0006099_tricarboxylic+acid+cycle	0.984755	0.995751	0.335061
BP_GO:0051016_barbed-end+actin+filament+capping	0.158758	0.582843	0.100998
BP_GO:0030199_collagen+fibril+organization	0.425809	0.616713	0.300909
MF_GO:0004129_cytochrome-c+oxidase+activity	0.999116	0.999709	0.453493
MF_GO:0050662_coenzyme+binding	0.252762	0.350726	0.251444
BP_GO:0051260_protein+homooligomerization	0.0396048	0.0394034	0.282849
BP_GO:0032312_regulation+of+ARF+GTPase+activity	0.756781	0.613939	0.78276
MF_GO:0008060_ARF+GTPase+activator+activity	0.756781	0.613939	0.78276
MF_GO:0005178_integrin+binding	0.852627	0.789766	0.748554
BP_GO:0048536_spleen+development	0.799433	0.380027	0.885481
BP_GO:0048538_thymus+development	0.782756	0.63558	0.765957
BP_GO:0042102_positive+regulation+of+T+cell+proliferation	0.70814	0.88065	0.343175
MF_GO:0005245_voltage-gated+calcium+channel+activity	0.548624	0.890615	0.119272
BP_GO:0019233_sensory+perception+of+pain	0.0973656	0.676464	0.0152142

BP_GO:0007492_endoderm+development	0.20231	0.0665189	0.737983
BP_GO:0007612_learning	0.0973505	0.160679	0.200937
BP_GO:0042733_embryonic+digit+morphogenesis	0.655007	0.688496	0.545009
CC_GO:0001750_photoreceptor+outer+segment	0.797985	0.848859	0.57611
MF_GO:0004869_cysteine+protease+inhibitor+activity	0.0908707	0.0587777	0.396415
CC_GO:0005922_connexon+complex	0.00951357	0.109785	0.0182268
BP_GO:0007602_phototransduction	0.0646664	0.32755	0.0383759
BP_GO:0007613_memory	0.149989	0.127218	0.386999
BP_GO:0007623_circadian+rhythm	0.424465	0.546615	0.298607
BP_GO:0044267_cellular+protein+metabolic+process	0.806099	0.52424	0.947685
CC_GO:0001533_cornified+envelope	0.139668	0.0554001	0.431022
BP_GO:0006641_triacylglycerol+metabolic+process	0.745189	0.793445	0.537834
CC_GO:0001725_stress+fiber	0.0324393	0.102137	0.0834112
BP_GO:0006094_gluconeogenesis	0.722107	0.736314	0.535853
MF_GO:0019838_growth+factor+binding	0.59403	0.528236	0.606509
BP_GO:0030001_metal+ion+transport	0.488392	0.37969	0.610089
BP_GO:0006469_negative+regulation+of+protein+kinase+activity	0.908029	0.80764	0.844845
MF_GO:0005540_hyaluronic+acid+binding	0.00216063	0.00912969	0.0482514
BP_GO:0007157_heterophilic+cell+adhesion	0.337838	0.884695	0.0118662
BP_GO:0030218_erythrocyte+differentiation	0.211981	0.187726	0.409338
BP_GO:0030155_regulation+of+cell+adhesion	0.00168172	0.0102372	0.0281801
BP_GO:0009408_response+to+heat	0.441297	0.428061	0.505664
MF_GO:0004089_carbonate+dehydratase+activity	0.164414	0.515846	0.0601201
BP_GO:0030217_T+cell+differentiation	0.108517	0.067485	0.500625
BP_GO:0007569_cell+aging	0.226705	0.204912	0.424381
BP_GO:0006820_anion+transport	0.84132	0.715725	0.808481
MF_GO:0016849_phosphorus-oxygen+lyase+activity	0.0198799	0.112162	0.0482789
BP_GO:0007585_respiratory+gaseous+exchange	0.337482	0.567223	0.277432
CC_GO:0000118_histone+deacetylase+complex	0.33912	0.386203	0.382341
BP_GO:0043410_positive+regulation+of+MAPKKK+cascade	0.94514	0.974823	0.603168
MF_GO:0005544_calcium-dependent+phospholipid+binding	0.223733	0.0517058	0.651211
CC_GO:0016605_PML+body	0.693466	0.601454	0.668664
BP_GO:0009411_response+to+UV	0.361883	0.205359	0.614817
BP_GO:0048589_developmental+growth	0.380164	0.65678	0.275176
BP_GO:0030509_BMP+signaling+pathway	0.974969	0.965978	0.834136
BP_GO:0050679_positive+regulation+of+epithelial+cell+proliferation	0.24157	0.941388	0.0279146
MF_GO:0008378_galactosyltransferase+activity	0.0943253	0.161845	0.18999
BP_GO:0045597_positive+regulation+of+cell+differentiation	0.869742	0.896856	0.61891
MF_GO:0005328_neurotransmitter:sodium+symporter+activity	0.0343628	0.0293057	0.27748
BP_GO:0006289_nucleotide-excision+repair	0.838407	0.5728	0.892875
BP_GO:0035116_embryonic+hindlimb+morphogenesis	0.74555	0.618561	0.733649
MF_GO:0030414_protease+inhibitor+activity	0.0421887	0.914544	0.00127416
MF_GO:0019843_rRNA+binding	0.512425	0.73321	0.194142
MF_GO:0005126_hematopoietin/interferon-class+(D200-domain)+cytokine+	0.344087	0.934734	0.0851661
BP_GO:0006936_muscle+contraction	0.303289	0.27837	0.45347
BP_GO:0006749_glutathione+metabolic+process	0.21549	0.212025	0.375988
BP_GO:0016049_cell+growth	0.05587	0.0157547	0.506547

BP_GO:0018105_peptidyl-serine+phosphorylation	0.893396	0.441355	0.948298
BP_GO:0042110_T+cell+activation	0.565294	0.212971	0.847979
MF_GO:0016769_transferase+activity,+transferring+nitrogenous+groups	0.476405	0.395807	0.606468
BP_GO:0001932_regulation+of+protein+amino+acid+phosphorylation	0.457011	0.340175	0.601871
MF_GO:0031418_L-ascorbic+acid+binding	0.024354	0.20971	0.0114171
BP_GO:0048535_lymph+node+development	0.270616	0.0288845	0.885641
MF_GO:0051539_4+iron,+4+sulfur+cluster+binding	0.887615	0.260737	0.994428
MF_GO:0008324_cation+transmembrane+transporter+activity	0.781567	0.925241	0.402817
BP_GO:0002053_positive+regulation+of+mesenchymal+cell+proliferation	0.509074	0.823755	0.184437
BP_GO:0008016_regulation+of+heart+contraction	0.618614	0.776009	0.29061
BP_GO:0030890_positive+regulation+of+B+cell+proliferation	0.912748	0.764902	0.880214
BP_GO:0006644_phospholipid+metabolic+process	0.0711027	0.0701681	0.257047
CC_GO:0015630_microtubule+cytoskeleton	0.375879	0.606991	0.266906
BP_GO:0009190_cyclic+nucleotide+biosynthetic+process	0.0236613	0.112162	0.0581686
BP_GO:0007528_neuromuscular+junction+development	0.413776	0.480288	0.378689
BP_GO:0015711_organic+anion+transport	0.342801	0.0546357	0.696734
BP_GO:0007566_embryo+implantation	0.447225	0.638131	0.25548
BP_GO:0032526_response+to+retinoic+acid	0.231586	0.595117	0.128871
BP_GO:0046677_response+to+antibiotic	0.799032	0.698575	0.75026
BP_GO:0006885_regulation+of+pH	0.486506	0.37719	0.592281
CC_GO:0030176_integral+to+endoplasmic+reticulum+membrane	0.896059	0.932401	0.527249
BP_GO:0033077_T+cell+differentiation+in+the+thymus	0.109835	0.0953181	0.316215
MF_GO:0004402_histone+acetyltransferase+activity	0.787	0.898489	0.501132
BP_GO:0001947_heart+looping	0.537949	0.139323	0.888232
BP_GO:0021766_hippocampus+development	0.907788	0.830522	0.821741
MF_GO:0004623_phospholipase+A2+activity	0.0922753	0.169781	0.174253
BP_GO:0030901_midbrain+development	0.974798	0.956786	0.845816
BP_GO:0007276_gamete+generation	0.105688	0.485816	0.0735572
BP_GO:0060079_regulation+of+excitatory+postsynaptic+membrane+potent	0.275594	0.451893	0.223466
MF_GO:0005261_cation+channel+activity	0.606354	0.850621	0.224225
BP_GO:0048646_anatomical+structure+formation	0.609358	0.944897	0.114032
BP_GO:0007565_female+pregnancy	0.125114	0.0421753	0.538978
MF_GO:0050840_extracellular+matrix+binding	0.0295636	0.526413	0.00236858
BP_GO:0001816_cytokine+production	0.307238	0.227952	0.503685
MF_GO:0008137_NADH+dehydrogenase+(ubiquinone)+activity	0.842977	0.662326	0.932295
BP_GO:0006928_cell+motion	0.261787	0.30968	0.334525
BP_GO:0008299_isoprenoid+biosynthetic+process	0.0298578	0.0463589	0.167441
BP_GO:0051092_positive+regulation+of+NF-kappaB+transcription+factor+ac	0.355964	0.36542	0.413494
MF_GO:0016706_oxidoreductase+activity,+acting+on+paired+donors,+with-	0.153624	0.445389	0.0471514
MF_GO:0004709_MAP+kinase+kinase+kinase+activity	0.500893	0.299353	0.73618
MF_GO:0019001_guanyl+nucleotide+binding	0.685393	0.7085	0.527343
BP_GO:0007611_learning+or+memory	0.867712	0.741071	0.826514
BP_GO:0009653_anatomical+structure+morphogenesis	0.213198	0.659017	0.0552454
MF_GO:0004629_phospholipase+C+activity	0.366171	0.392431	0.413425
BP_GO:0051276_chromosome+organization	0.274035	0.424409	0.265977
BP_GO:0060021_palate+development	0.551142	0.88759	0.105309
CC_GO:0000793_condensed+chromosome	0.888157	0.796524	0.814015

BP_GO:0031069_hair+follicle+morphogenesis	0.0320377	0.119084	0.0714463
BP_GO:0007595_lactation	0.343272	0.290055	0.486277
MF_GO:0008013_beta-catenin+binding	0.57622	0.61243	0.504362
BP_GO:0042113_B+cell+activation	0.460273	0.622731	0.347331
BP_GO:0001942_hair+follicle+development	0.486945	0.843842	0.159026
MF_GO:0015171_amino+acid+transmembrane+transporter+activity	0.0474014	0.849251	0.00224742
MF_GO:0016493_C-C+chemokine+receptor+activity	0.150854	0.293558	0.181587
BP_GO:0007254_JNK+cascade	0.92404	0.955064	0.644487
CC_GO:0005669_transcription+factor+TFIID+complex	0.941145	0.849617	0.881202
BP_GO:0006888_ER+to+Golgi+vesicle-mediated+transport	0.927992	0.680472	0.975503
MF_GO:0003712_transcription+cofactor+activity	0.981037	0.996338	0.445501
BP_GO:0000184_nuclear-transcribed+mRNA+catabolic+process,+nonsense-r	0.966217	0.977966	0.637166
CC_GO:0000795_synaptonemal+complex	0.750133	0.510747	0.804268
CC_GO:0005762_mitochondrial+large+ribosomal+subunit	0.97151	0.952724	0.821298
BP_GO:0007269_neurotransmitter+secretion	0.799523	0.537342	0.919837
CC_GO:0001673_male+germ+cell+nucleus	0.266053	0.362966	0.300569
MF_GO:0017137_Rab+GTPase+binding	0.437496	0.425216	0.49162
MF_GO:0004970_ionotropic+glutamate+receptor+activity	0.00810709	0.10322	0.0150867
MF_GO:0005164_tumor+necrosis+factor+receptor+binding	0.899458	0.763277	0.85521
BP_GO:0007339_binding+of+sperm+to+zona+pellucida	0.266772	0.790039	0.0852641
BP_GO:0001580_detection+of+chemical+stimulus+involved+in+sensory+per	0.657175	0.687374	0.586957
CC_GO:0042612_MHC+class+I+protein+complex	0.677513	0.943684	0.216206
BP_GO:0001974_blood+vessel+remodeling	0.277067	0.149077	0.494571
CC_GO:0031594_neuromuscular+junction	0.830253	0.655184	0.829282
BP_GO:0032880_regulation+of+protein+localization	0.802313	0.725964	0.73235
BP_GO:0030032_lamellipodium+assembly	0.61536	0.806964	0.35425
BP_GO:0050776_regulation+of+immune+response	0.709039	0.633198	0.667265
BP_GO:0010332_response+to+gamma+radiation	0.117358	0.12347	0.285919
BP_GO:0030336_negative+regulation+of+cell+migration	0.00748797	0.00221053	0.381593
MF_GO:0004653_polypeptide+N-acetylgalactosaminyltransferase+activity	0.000676292	0.0151021	0.0058205
BP_GO:0042130_negative+regulation+of+T+cell+proliferation	0.00519382	0.00370219	0.171203
BP_GO:0007224_smoothened+signaling+pathway	0.900963	0.943578	0.655531
BP_GO:0030318_melanocyte+differentiation	0.418852	0.261143	0.61407
BP_GO:0000723_telomere+maintenance	0.347045	0.644365	0.104745
BP_GO:0007249_I-kappaB+kinase/NF-kappaB+cascade	0.101676	0.468067	0.0332635
MF_GO:0005543_phospholipid+binding	0.678616	0.205058	0.889488
BP_GO:0001502_cartilage+condensation	0.690329	0.460546	0.805913
BP_GO:0035108_limb+morphogenesis	0.487187	0.193187	0.79382
BP_GO:0001708_cell+fate+specification	0.775343	0.654131	0.756141
BP_GO:0042221_response+to+chemical+stimulus	0.718422	0.879432	0.396222
BP_GO:0030574_collagen+catabolic+process	0.28816	0.345297	0.342086
BP_GO:0000059_protein+import+into+nucleus,+docking	0.686443	0.771446	0.433461
CC_GO:0016363_nuclear+matrix	0.864251	0.53284	0.941703
BP_GO:0030308_negative+regulation+of+cell+growth	0.743605	0.238815	0.897465
MF_GO:0004889_nicotinic+acetylcholine-activated+cation-selective+channe	0.167477	0.492969	0.0888561
BP_GO:0008306_associative+learning	0.139335	0.245873	0.199168
CC_GO:0005932_microtubule+basal+body	0.228419	0.106682	0.696163

MF_GO:0005003_ephrin+receptor+activity	0.813869	0.356929	0.921044
BP_GO:0030534_adult+behavior	0.372718	0.722967	0.146371
MF_GO:0005234_extracellular-glutamate-gated+ion+channel+activity	0.00816868	0.109095	0.0150867
CC_GO:0015935_small+ribosomal+subunit	0.840389	0.783586	0.736798
CC_GO:0005863_striated+muscle+thick+filament	0.7953	0.622673	0.83816
BP_GO:0006520_amino+acid+metabolic+process	0.323517	0.254877	0.554807
MF_GO:0004697_protein+kinase+C+activity	0.586059	0.49498	0.621221
BP_GO:0007512_adult+heart+development	0.528138	0.565984	0.476214
MF_GO:0046914_transition+metal+ion+binding	0.181657	0.281526	0.223937
CC_GO:0005581_collagen	0.156369	0.155156	0.331717
BP_GO:0006637_acyl-CoA+metabolic+process	0.0721508	0.012985	0.661529
MF_GO:0051537_2+iron,+2+sulfur+cluster+binding	0.163208	0.11615	0.435832
BP_GO:0030301_cholesterol+transport	0.623072	0.40073	0.847109
BP_GO:0001934_positive+regulation+of+protein+amino+acid+phosphorylati	0.706434	0.520978	0.726356
MF_GO:0005539_glycosaminoglycan+binding	0.135597	0.192358	0.238038
MF_GO:0046873_metal+ion+transmembrane+transporter+activity	0.498804	0.363785	0.610089
BP_GO:0048286_alveolus+development	0.299835	0.403724	0.318575
BP_GO:0008219_cell+death	0.17799	0.240345	0.274979
BP_GO:0007040_lysosome+organization	0.118124	0.156048	0.264523
BP_GO:0044237_cellular+metabolic+process	0.068048	0.0742508	0.311054
BP_GO:0016525_negative+regulation+of+angiogenesis	0.542014	0.364029	0.749788
BP_GO:0048066_pigmentation+during+development	0.480662	0.835055	0.163863
BP_GO:0019370_leukotriene+biosynthetic+process	0.448597	0.933156	0.0205242
BP_GO:0042445_hormone+metabolic+process	0.243977	0.792097	0.0592776
MF_GO:0050661_NADP+binding	0.580488	0.36593	0.746305
BP_GO:0051789_response+to+protein+stimulus	0.0929054	0.0108695	0.630309
CC_GO:0005912_adherens+junction	0.27297	0.884967	0.0150472
BP_GO:0048873_homeostasis+of+number+of+cells+within+a+tissue	0.632439	0.287811	0.915837
CC_GO:0005903_brush+border	0.000306112	0.143931	0.000186349
MF_GO:0046961_proton-transporting+ATPase+activity,+rotational+mechani	0.983039	0.993537	0.494021
MF_GO:0004860_protein+kinase+inhibitor+activity	0.166519	0.322978	0.190446
BP_GO:0031016_pancreas+development	0.699347	0.540986	0.743563
MF_GO:0005484_SNAP+receptor+activity	0.992735	0.996739	0.650464
BP_GO:0016043_cellular+component+organization	0.615318	0.356221	0.819769
BP_GO:0060041_retina+development+in+camera-type+eye	0.812535	0.524661	0.907784
MF_GO:0005057_receptor+signaling+protein+activity	0.132363	0.409712	0.0941649
MF_GO:0016818_hydrolase+activity,+acting+on+acid+anhydrides,+in+phosp	0.666084	0.711562	0.525965
BP_GO:0042060_wound+healing	0.676718	0.796238	0.385811
BP_GO:0035115_embryonic+forelimb+morphogenesis	0.525125	0.679825	0.362568
BP_GO:0048593_camera-type+eye+morphogenesis	0.87584	0.710464	0.863676
CC_GO:0000776_kinetochore	0.254457	0.414456	0.248988
BP_GO:0006302_double-strand+break+repair	0.17738	0.113497	0.389389
BP_GO:0016998_cell+wall+catabolic+process	0.501261	0.237656	0.778412
MF_GO:0003995_acyl-CoA+dehydrogenase+activity	0.797456	0.970737	0.108472
BP_GO:0031290_retinal+ganglion+cell+axon+guidance	0.997143	0.923374	0.992856
BP_GO:0042384_cilium+assembly	0.944115	0.932624	0.752721
MF_GO:0016566_specific+transcriptional+repressor+activity	0.15924	0.562169	0.00540601

CC_GO:0000781_chromosome,+telomeric+region	0.810545	0.645656	0.803769
BP_GO:0030902_hindbrain+development	0.160312	0.159513	0.333689
BP_GO:0001824_blastocyst+development	0.182497	0.38088	0.143839
BP_GO:0007422_peripheral+nervous+system+development	0.825204	0.843541	0.631861
MF_GO:0004864_protein+phosphatase+inhibitor+activity	0.98935	0.989184	0.796386
BP_GO:0050680_negative+regulation+of+epithelial+cell+proliferation	0.548642	0.619465	0.428487
BP_GO:0048167_regulation+of+synaptic+plasticity	0.917391	0.777855	0.881976
BP_GO:0000398_nuclear+mRNA+splicing,+via+spliceosome	0.917227	0.842033	0.832625
MF_GO:0004190_aspartic-type+endopeptidase+activity	0.755117	0.951589	0.222259
BP_GO:0021983_pituitary+gland+development	0.243933	0.221722	0.393046
MF_GO:0005212_structural+constituent+of+eye+lens	0.00138214	0.00653823	0.0328149
BP_GO:0048839_inner+ear+development	0.878883	0.773054	0.81893
BP_GO:0001829_trophectodermal+cell+differentiation	0.431592	0.585036	0.272951
BP_GO:0007214_gamma-aminobutyric+acid+signaling+pathway	0.837662	0.942972	0.492601
CC_GO:0042734_presynaptic+membrane	0.411406	0.791795	0.0618454
MF_GO:0008227_amine+receptor+activity	0.423188	0.472934	0.419598
CC_GO:0022626_cytosolic+ribosome	0.384149	0.38385	0.477947
BP_GO:0042157_lipoprotein+metabolic+process	0.0938111	0.0629878	0.337195
BP_GO:0002474_antigen+processing+and+presentation+of+peptide+antigen	0.563197	0.897663	0.187136
CC_GO:0030017_sarcomere	0.195927	0.0867379	0.652402
BP_GO:0019886_antigen+processing+and+presentation+of+exogenous+pep	0.900074	0.996763	0.333743
BP_GO:0006839_mitochondrial+transport	0.919073	0.708579	0.915441
BP_GO:0006071_glycerol+metabolic+process	0.374354	0.581199	0.290049
BP_GO:0007178_transmembrane+receptor+protein+serine/threonine+kinas	0.74105	0.779877	0.55655
BP_GO:0006635_fatty+acid+beta-oxidation	0.192194	0.575924	0.0265889
CC_GO:0005778_peroxisomal+membrane	0.471908	0.835116	0.0469182
BP_GO:0048514_blood+vessel+morphogenesis	0.630624	0.121629	0.949272
BP_GO:0001759_induction+of+an+organ	0.237387	0.614829	0.0960524
BP_GO:0042098_T+cell+proliferation	0.307941	0.122249	0.764494
CC_GO:0008287_protein+serine/threonine+phosphatase+complex	0.867441	0.879611	0.563784
BP_GO:0016485_protein+processing	0.627062	0.599742	0.580244
BP_GO:0001958_endochondral+ossification	0.562291	0.10837	0.867565
BP_GO:0006471_protein+amino+acid+ADP-ribosylation	0.36346	0.390377	0.41548
MF_GO:0004707_MAP+kinase+activity	0.960849	0.995406	0.454427
MF_GO:0004437_inositol+or+phosphatidylinositol+phosphatase+activity	0.463201	0.724068	0.233706
MF_GO:0016747_transferase+activity,+transferring+acyl+groups+other+thar	0.595183	0.903423	0.168175
CC_GO:0005614_interstitial+matrix	0.13135	0.395562	0.123215
MF_GO:0004435_phosphoinositide+phospholipase+C+activity	0.342167	0.379669	0.389771
MF_GO:0005247_voltage-gated+chloride+channel+activity	0.0358184	0.34399	0.0158306
BP_GO:0051402_neuron+apoptosis	0.821355	0.86789	0.599032
MF_GO:0004198_calcium-dependent+cysteine-type+endopeptidase+activity	0.657483	0.474271	0.77782
MF_GO:0017048_Rho+GTPase+binding	0.279042	0.655203	0.130945
MF_GO:0008514_organic+anion+transmembrane+transporter+activity	0.548889	0.308871	0.677241
BP_GO:0006665_sphingolipid+metabolic+process	0.573415	0.62348	0.478575
MF_GO:0004835_tubulin-tyrosine+ligase+activity	0.972529	0.972236	0.833379
BP_GO:0006700_C21-steroid+hormone+biosynthetic+process	0.391169	0.398659	0.438704
BP_GO:0009409_response+to+cold	0.990201	0.961175	0.942727

MF_GO:0005242_inward+rectifier+potassium+channel+activity	0.766891	0.967019	0.264863
MF_GO:0005070_SH3/SH2+adaptor+activity	0.137235	0.113234	0.44667
BP_GO:0001782_B+cell+homeostasis	0.639997	0.581492	0.634816
BP_GO:0008624_induction+of+apoptosis+by+extracellular+signals	0.0274143	0.769034	0.000737816
BP_GO:0009966_regulation+of+signal+transduction	0.753234	0.859713	0.488476
BP_GO:0001662_behavioral+fear+response	0.326197	0.666339	0.166208
MF_GO:0008553_hydrogen-exporting+ATPase+activity,+phosphorylative+m	0.797948	0.916855	0.197612
MF_GO:0008022_protein+C-terminus+binding	0.00481546	0.024621	0.0334962
BP_GO:0030514_negative+regulation+of+BMP+signaling+pathway	0.707654	0.488445	0.772632
CC_GO:0019897_extrinsic+to+plasma+membrane	0.524074	0.743328	0.253432
BP_GO:0032012_regulation+of+ARF+protein+signal+transduction	0.341597	0.850914	0.12689
MF_GO:0005086_ARF+guanyl-nucleotide+exchange+factor+activity	0.341597	0.850914	0.12689
BP_GO:0001975_response+to+amphetamine	0.371648	0.175908	0.579247
BP_GO:0006284_base-excision+repair	0.375846	0.215439	0.664585
CC_GO:0014069_postsynaptic+density	0.135937	0.14336	0.281566
BP_GO:0042491_auditory+receptor+cell+differentiation	0.166953	0.596343	0.0257672
BP_GO:0035176_social+behavior	0.357498	0.433404	0.357335
BP_GO:0007338_single+fertilization	0.0212789	0.352543	0.0063428
BP_GO:0006414_translational+elongation	0.964287	0.871648	0.932652
MF_GO:0015380_anion+exchanger+activity	0.405358	0.169524	0.694906
BP_GO:0031018_endocrine+pancreas+development	0.468835	0.185362	0.715114
BP_GO:0048169_regulation+of+long-term+neuronal+synaptic+plasticity	0.671418	0.939153	0.225584
CC_GO:0030057_desmosome	0.851924	0.503182	0.87981
BP_GO:0002009_morphogenesis+of+an+epithelium	0.177984	0.162925	0.390731
BP_GO:0045214_sarcomere+organization	0.492872	0.498379	0.490101
BP_GO:0050766_positive+regulation+of+phagocytosis	0.310077	0.495536	0.236059
MF_GO:0005537_mannose+binding	0.48425	0.895614	0.0782316
BP_GO:0016044_membrane+organization	0.00771778	0.0503576	0.0362827
MF_GO:0003950_NAD++ADP-ribosyltransferase+activity	0.663354	0.600398	0.633443
BP_GO:0030168_platelet+activation	0.307335	0.368079	0.352493
BP_GO:0031668_cellular+response+to+extracellular+stimulus	0.304976	0.661123	0.163581
MF_GO:0016805_dipeptidase+activity	0.101689	0.296941	0.107154
CC_GO:0005626_insoluble+fraction	0.125802	0.18092	0.234406
BP_GO:0006898_receptor-mediated+endocytosis	0.518776	0.496801	0.526111
CC_GO:0030175_filopodium	0.608968	0.837174	0.33549
MF_GO:0004467_long-chain-fatty-acid-CoA+ligase+activity	0.0051675	0.000683297	0.464824
MF_GO:0005518_collagen+binding	0.561599	0.31127	0.7761
BP_GO:0030099_myeloid+cell+differentiation	0.942121	0.985762	0.479764
MF_GO:0030165_PDZ+domain+binding	0.0872805	0.00550195	0.691939
MF_GO:0008047_enzyme+activator+activity	0.098434	0.280683	0.117462
BP_GO:0030593_neutrophil+chemotaxis	0.618489	0.958606	0.213626
BP_GO:0007026_negative+regulation+of+microtubule+depolymerization	0.781706	0.546593	0.804153
MF_GO:0004407_histone+deacetylase+activity	0.692072	0.379338	0.85767
BP_GO:0006833_water+transport	0.272251	0.0286285	0.766912
BP_GO:0006171_cAMP+biosynthetic+process	0.206743	0.464752	0.113336
BP_GO:0007274_neuromuscular+synaptic+transmission	0.0906398	0.72107	0.00461653
CC_GO:0030315_T-tubule	0.282982	0.130979	0.688665

BP_GO:0002062_chondrocyte+differentiation	0.732466	0.632025	0.702871
BP_GO:0009611_response+to+wounding	0.0481942	0.268452	0.0285202
BP_GO:0007076_mitotic+chromosome+condensation	0.112845	0.331264	0.128801
MF_GO:0004143_diacylglycerol+kinase+activity	0.199123	0.440385	0.168601
BP_GO:0050900_leukocyte+migration	0.693515	0.60594	0.674993
BP_GO:0007031_peroxisome+organization	0.594259	0.799502	0.202857
CC_GO:0005891_voltage-gated+calcium+channel+complex	0.837913	0.966069	0.235579
BP_GO:0001836_release+of+cytochrome+c+from+mitochondria	0.89659	0.764334	0.853958
BP_GO:0050905_neuromuscular+process	0.249046	0.368435	0.269693
BP_GO:0008045_motor+axon+guidance	0.611533	0.700748	0.439384
BP_GO:0050880_regulation+of+blood+vessel+size	0.517166	0.525137	0.505542
BP_GO:0007405_neuroblast+proliferation	0.181359	0.713673	0.014481
BP_GO:0048856_anatomical+structure+development	0.182602	0.556731	0.0856958
BP_GO:0050808_synapse+organization	0.192531	0.22343	0.334698
MF_GO:0003993_acid+phosphatase+activity	0.562083	0.901385	0.163158
BP_GO:0044419_interspecies+interaction+between+organisms	0.769936	0.69224	0.709763
BP_GO:0008272_sulfate+transport	0.339452	0.231064	0.496557
BP_GO:0030282_bone+mineralization	0.755355	0.458541	0.870749
BP_GO:0015671_oxygen+transport	0.959909	0.936197	0.835068
MF_GO:0005344_oxygen+transporter+activity	0.959909	0.936197	0.835068
MF_GO:0030552_cAMP+binding	0.432587	0.486897	0.430003
BP_GO:0050852_T+cell+receptor+signaling+pathway	0.823887	0.178148	0.991004
BP_GO:0030878_thyroid+gland+development	0.557975	0.206354	0.898108
CC_GO:0000794_condensed+nuclear+chromosome	0.545278	0.76056	0.274769
BP_GO:0006367_transcription+initiation+from+RNA+polymerase+II+promot	0.733892	0.587962	0.72644
BP_GO:0048477_oogenesis	0.0272922	0.0110035	0.360039
BP_GO:0019827_stem+cell+maintenance	0.674217	0.473961	0.750247
BP_GO:0001654_eye+development	0.115146	0.451414	0.0637929
BP_GO:0042462_eye+photoreceptor+cell+development	0.225367	0.217471	0.349221
BP_GO:0001964_startle+response	0.209561	0.290493	0.272762
MF_GO:0005184_neuropeptide+hormone+activity	0.561504	0.347915	0.693382
BP_GO:0001709_cell+fate+determination	0.577709	0.601384	0.504056
CC_GO:0016607_nuclear+speck	0.69596	0.911364	0.33979
BP_GO:0035249_synaptic+transmission,+glutamatergic	0.344406	0.790566	0.0715652
BP_GO:0045666_positive+regulation+of+neuron+differentiation	0.668327	0.285876	0.841615
BP_GO:0043434_response+to+peptide+hormone+stimulus	0.260832	0.0992902	0.548767
BP_GO:0019915_sequestering+of+lipid	0.0481395	0.0187243	0.608693
CC_GO:0030016_myofibril	0.0726663	0.372642	0.00928042
CC_GO:0005811_lipid+particle	0.794282	0.818666	0.490194
BP_GO:0043029_T+cell+homeostasis	0.0247216	0.0816537	0.0811449
BP_GO:0009749_response+to+glucose+stimulus	0.329771	0.257811	0.561584
MF_GO:0004806_triacylglycerol+lipase+activity	0.812182	0.746224	0.724818
CC_GO:0005605_basal+lamina	0.0213925	0.026305	0.198188
BP_GO:0018107_peptidyl-threonine+phosphorylation	0.829156	0.566404	0.852214
BP_GO:0048844_artery+morphogenesis	0.735582	0.380984	0.859928
BP_GO:0016064_immunoglobulin+mediated+immune+response	0.888154	0.88963	0.690169
BP_GO:0043123_positive+regulation+of+I-kappaB+kinase/NF-kappaB+casca	0.920786	0.996635	0.15343

BP_GO:0050853_B+cell+receptor+signaling+pathway	0.385904	0.0758342	0.893641
BP_GO:0006541_glutamine+metabolic+process	0.513008	0.232907	0.710378
MF_GO:0004012_phospholipid-translocating+ATPase+activity	0.209495	0.731409	0.0390547
CC_GO:0030139_endocytic+vesicle	0.684293	0.961755	0.0468507
BP_GO:0043113_receptor+clustering	0.599477	0.898336	0.132199
BP_GO:0006479_protein+amino+acid+methylation	0.724803	0.406634	0.88578
BP_GO:0055010_ventricular+cardiac+muscle+morphogenesis	0.667561	0.882312	0.17636
BP_GO:0007204_elevation+of+cytosolic+calcium+ion+concentration	0.0277571	0.388437	0.0243043
BP_GO:0051085_chaperone+cofactor-dependent+protein+folding	0.592114	0.580281	0.547894
BP_GO:0035265_organ+growth	0.780218	0.987187	0.127343
BP_GO:0016331_morphogenesis+of+embryonic+epithelium	0.155376	0.154941	0.318887
CC_GO:0000792_heterochromatin	0.789042	0.39543	0.940381
MF_GO:0005158_insulin+receptor+binding	0.454413	0.497722	0.424008
BP_GO:0042474_middle+ear+morphogenesis	0.480825	0.671542	0.2738
BP_GO:0016571_histone+methylation	0.741294	0.629992	0.729016
BP_GO:0017148_negative+regulation+of+translation	0.294017	0.424948	0.22425
BP_GO:0006904_vesicle+docking+during+exocytosis	0.303868	0.306847	0.402518
CC_GO:0017053_transcriptional+repressor+complex	0.883653	0.949208	0.401754
CC_GO:0000159_protein+phosphatase+type+2A+complex	0.985821	0.993338	0.680331
BP_GO:0007498_mesoderm+development	0.791057	0.671706	0.780592
BP_GO:0045669_positive+regulation+of+osteoblast+differentiation	0.976172	0.976569	0.792186
BP_GO:0007259_JAK-STAT+cascade	0.516264	0.149356	0.901844
CC_GO:0005871_kinesin+complex	0.895749	0.916512	0.588533
BP_GO:0006493_protein+amino+acid+O-linked+glycosylation	0.481753	0.813275	0.0509136
BP_GO:0010001_glial+cell+differentiation	0.114651	0.286894	0.127197
BP_GO:0035050_embryonic+heart+tube+development	0.259476	0.108868	0.437678
BP_GO:0006164_purine+nucleotide+biosynthetic+process	0.703868	0.568755	0.733776
BP_GO:0046330_positive+regulation+of+JNK+cascade	0.712306	0.94864	0.200414
BP_GO:0043161_proteasomal+ubiquitin-dependent+protein+catabolic+proc	0.982281	0.97712	0.814795
BP_GO:0000209_protein+polyubiquitination	0.880092	0.425176	0.979559
MF_GO:0015450_P-P-bond-hydrolysis-driven+protein+transmembrane+tran	0.998205	0.997042	0.914263
BP_GO:0030968_endoplasmic+reticulum+unfolded+protein+response	0.524687	0.4834	0.557321
MF_GO:0005149_interleukin-1+receptor+binding	0.971184	0.783924	0.965801
BP_GO:0050850_positive+regulation+of+calcium-mediated+signaling	0.104435	0.312017	0.085436
BP_GO:0007205_activation+of+protein+kinase+C+activity	0.873222	0.553487	0.903719
CC_GO:0045120_pronucleus	0.272969	0.313571	0.355768
BP_GO:0045860_positive+regulation+of+protein+kinase+activity	0.929694	0.757446	0.909799
CC_GO:0005788_endoplasmic+reticulum+lumen	0.977141	0.503776	0.992665
MF_GO:0003690_double-stranded+DNA+binding	0.449245	0.54075	0.35675
BP_GO:0045190_isotype+switching	0.471801	0.124688	0.719686
BP_GO:0006298_mismatch+repair	0.532576	0.281483	0.72404
MF_GO:0000049_tRNA+binding	0.968268	0.836693	0.949957
BP_GO:0045595_regulation+of+cell+differentiation	0.690064	0.948656	0.175924
CC_GO:0044424_intracellular+part	0.0559623	0.377133	0.0180751
MF_GO:0004993_serotonin+receptor+activity	0.331831	0.625686	0.223606
CC_GO:0000178_exosome+(RNase+complex)	0.48472	0.378139	0.600698
CC_GO:0000300_peripheral+to+membrane+of+membrane+fraction	0.0480346	0.0532324	0.319327

MF_GO:0008527_taste+receptor+activity	0.799353	0.687374	0.753957
BP_GO:0030833_regulation+of+actin+filament+polymerization	0.846021	0.908033	0.522506
BP_GO:0006911_phagocytosis,+engulfment	0.140915	0.275101	0.184287
BP_GO:0006807_nitrogen+compound+metabolic+process	0.851017	0.861713	0.612705
MF_GO:0030674_protein+binding,+bridging	0.689258	0.489866	0.838156
CC_GO:0031941_filamentous+actin	0.474396	0.290746	0.631206
MF_GO:0008484_sulfuric+ester+hydrolase+activity	0.512416	0.831405	0.246063
BP_GO:0048738_cardiac+muscle+development	0.893599	0.0475634	0.999882
MF_GO:0010181_FMN+binding	0.34029	0.282118	0.531343
BP_GO:0050830_defense+response+to+Gram-positive+bacterium	0.0573514	0.246194	0.0775604
MF_GO:0048037_cofactor+binding	0.207301	0.192188	0.442935
BP_GO:0015914_phospholipid+transport	0.43068	0.773684	0.177993
BP_GO:0046716_muscle+maintenance	0.12898	0.669056	0.0400649
BP_GO:0042573_retinoic+acid+metabolic+process	0.0218525	0.029357	0.113143
BP_GO:0030048_actin+filament-based+movement	0.0170164	0.100494	0.0403747
BP_GO:0007162_negative+regulation+of+cell+adhesion	0.042632	0.73294	0.0226571
CC_GO:0000299_integral+to+membrane+of+membrane+fraction	0.936603	0.702407	0.953831
BP_GO:0051017_actin+filament+bundle+formation	0.442362	0.604723	0.182443
MF_GO:0015250_water+channel+activity	0.180332	0.0286285	0.69006
BP_GO:0031663_lipopolysaccharide-mediated+signaling+pathway	0.476213	0.654801	0.29944
MF_GO:0004675_transmembrane+receptor+protein+serine/threonine+kina	0.699433	0.639772	0.649084
MF_GO:0005024_transforming+growth+factor+beta+receptor+activity	0.699433	0.639772	0.649084
MF_GO:0004016_adenylate+cyclase+activity	0.0316365	0.185504	0.0287194
BP_GO:0043085_positive+regulation+of+catalytic+activity	0.994728	0.802103	0.994839
MF_GO:0017110_nucleoside-diphosphatase+activity	0.280905	0.365815	0.313326
MF_GO:0050897_cobalt+ion+binding	0.850278	0.538641	0.876361
BP_GO:0048598_embryonic+morphogenesis	0.697159	0.783437	0.549824
BP_GO:0007200_G-protein+signaling,+coupled+to+IP3+second+messenger+	0.847425	0.82867	0.699238
MF_GO:0008603_cAMP-dependent+protein+kinase+regulator+activity	0.395604	0.215453	0.680447
CC_GO:0033177_proton-transporting+two-sector+ATPase+complex,+proton	0.126681	0.261202	0.125981
BP_GO:0046847_filopodium+formation	0.655474	0.835884	0.406053
BP_GO:0042771_DNA+damage+response,+signal+transduction+by+p53+clas	0.748291	0.187229	0.934391
BP_GO:0051145_smooth+muscle+cell+differentiation	0.989852	0.978547	0.930937
MF_GO:0016790_thiolester+hydrolase+activity	0.733848	0.847677	0.463418
BP_GO:0032869_cellular+response+to+insulin+stimulus	0.927388	0.840935	0.857076
CC_GO:0031105_septin+complex	0.548592	0.312091	0.729611
BP_GO:0048015_phosphoinositide-mediated+signaling	0.698116	0.62432	0.660618
MF_GO:0004683_calmodulin-dependent+protein+kinase+activity	0.581746	0.259823	0.843927
MF_GO:0008235_metalloexopeptidase+activity	0.358044	0.672145	0.151651
BP_GO:0048701_embryonic+cranial+skeleton+morphogenesis	0.650841	0.732564	0.457924
CC_GO:0005942_phosphoinositide+3-kinase+complex	0.617942	0.829957	0.505294
MF_GO:0004926_non-G-protein+coupled+7TM+receptor+activity	0.409972	0.126135	0.872091
BP_GO:0007190_activation+of+adenylate+cyclase+activity	0.043626	0.0270895	0.333651
CC_GO:0005884_actin+filament	0.687119	0.561106	0.727365
BP_GO:0050728_negative+regulation+of+inflammatory+response	0.207063	0.0827867	0.620169
BP_GO:0048821_erythrocyte+development	0.168648	0.508228	0.127382
BP_GO:0001837_epithelial+to+mesenchymal+transition	0.806159	0.815433	0.574155

MF_GO:0046933_hydrogen+ion+transporting+ATP+synthase+activity,+rotat	0.965157	0.996214	0.0787285
MF_GO:0004428_inositol+or+phosphatidylinositol+kinase+activity	0.935988	0.905778	0.819677
BP_GO:0009888_tissue+development	0.386867	0.771835	0.0715577
CC_GO:0005902_microvillus	0.0275376	0.236238	0.0350505
BP_GO:0016197_endosome+transport	0.888798	0.896959	0.664493
BP_GO:0006809_nitric+oxide+biosynthetic+process	0.383986	0.17353	0.722827
BP_GO:0040015_negative+regulation+of+multicellular+organism+growth	0.608831	0.182439	0.809938
CC_GO:0031227_intrinsic+to+endoplasmic+reticulum+membrane	0.611554	0.797294	0.392011
MF_GO:0015269_calcium-activated+potassium+channel+activity	0.243139	0.630039	0.177026
BP_GO:0030539_male+genitalia+development	0.071757	0.81624	0.0205586
CC_GO:0005720_nuclear+heterochromatin	0.673545	0.29009	0.86718
BP_GO:0055007_cardiac+muscle+cell+differentiation	0.869016	0.831949	0.722489
MF_GO:0001619_lysosphingolipid+and+lysophosphatidic+acid+receptor+act	0.671859	0.717624	0.511448
BP_GO:0007340_acrosome+reaction	0.661568	0.997072	0.0593441
BP_GO:0010165_response+to+X-ray	0.137909	0.27756	0.178345
BP_GO:0008543_fibroblast+growth+factor+receptor+signaling+pathway	0.668979	0.707315	0.519947
BP_GO:0042417_dopamine+metabolic+process	0.155181	0.254281	0.219573
CC_GO:0043197_dendritic+spine	0.120271	0.317747	0.0698981
BP_GO:0018298_protein-chromophore+linkage	0.30292	0.421733	0.301591
BP_GO:0016070_RNA+metabolic+process	0.949757	0.881263	0.878289
BP_GO:0016573_histone+acetylation	0.875678	0.824731	0.770535
BP_GO:0006342_chromatin+silencing	0.660308	0.587989	0.66556
CC_GO:0030672_synaptic+vesicle+membrane	0.79102	0.585479	0.832637
BP_GO:0006968_cellular+defense+response	0.20556	0.0424417	0.747531
CC_GO:0005814_centriole	0.837194	0.850411	0.573943
BP_GO:0007530_sex+determination	0.000649125	0.067855	0.00136461
BP_GO:0007548_sex+differentiation	0.176397	0.574457	0.0365691
BP_GO:0002088_lens+development+in+camera-type+eye	0.11896	0.14529	0.275937
MF_GO:0019905_syntaxin+binding	0.749118	0.728467	0.648784
BP_GO:0007416_synaptogenesis	0.0632007	0.601592	0.0105367
CC_GO:0005758_mitochondrial+intermembrane+space	0.65654	0.549535	0.677531
BP_GO:0001522_pseudouridine+synthesis	0.231445	0.839627	0.0708768
MF_GO:0009982_pseudouridine+synthase+activity	0.231445	0.839627	0.0708768
BP_GO:0048265_response+to+pain	0.234838	0.0611382	0.471035
BP_GO:0031589_cell-substrate+adhesion	0.0100071	0.0116876	0.258209
BP_GO:0006957_complement+activation,+alternative+pathway	0.682633	0.416655	0.843407
MF_GO:0008307_structural+constituent+of+muscle	0.0501239	0.232814	0.0437934
BP_GO:0019363_pyridine+nucleotide+biosynthetic+process	0.713918	0.890533	0.269794
BP_GO:0048741_skeletal+muscle+fiber+development	0.107373	0.417373	0.0437447
BP_GO:0006937_regulation+of+muscle+contraction	0.508212	0.277796	0.889738
BP_GO:0006825_copper+ion+transport	0.0229648	0.332165	0.0130459
BP_GO:0007159_leukocyte+adhesion	0.458677	0.559265	0.383251
MF_GO:0016290_palmitoyl-CoA+hydrolase+activity	0.488295	0.685389	0.332553
BP_GO:0032760_positive+regulation+of+tumor+necrosis+factor+production	0.161665	0.574189	0.0764709
BP_GO:0043087_regulation+of+GTPase+activity	0.241623	0.461236	0.204115
CC_GO:0045263_proton-transporting+ATP+synthase+complex,+coupling+fac	0.947332	0.853798	0.975346
BP_GO:0009072_aromatic+amino+acid+family+metabolic+process	0.335246	0.227826	0.556823

MF_GO:0003954_NADH+dehydrogenase+activity	0.589894	0.473273	0.739538
BP_GO:0055085_transmembrane+transport	0.286643	0.444801	0.254875
MF_GO:0022891_substrate-specific+transmembrane+transporter+activity	0.286643	0.444801	0.254875
BP_GO:0001676_long-chain+fatty+acid+metabolic+process	0.0438188	0.012532	0.618645
BP_GO:0042744_hydrogen+peroxide+catabolic+process	0.215681	0.263845	0.30898
BP_GO:0048813_dendrite+morphogenesis	0.317896	0.385982	0.31507
MF_GO:0005355_glucose+transmembrane+transporter+activity	0.455009	0.554629	0.363825
MF_GO:0004935_adrenoceptor+activity	0.0699742	0.0265491	0.438173
MF_GO:0016209_antioxidant+activity	0.771515	0.86657	0.288063
BP_GO:0022008_neurogenesis	0.782722	0.560792	0.787215
BP_GO:0046676_negative+regulation+of+insulin+secretion	0.660528	0.839244	0.34214
CC_GO:0001772_immunological+synapse	0.497415	0.238248	0.85889
BP_GO:0009116_nucleoside+metabolic+process	0.677484	0.538495	0.755872
BP_GO:0042640_anagen	0.858845	0.872575	0.588331
MF_GO:0008143_poly(A)+binding	0.996998	0.985545	0.955663
CC_GO:0012506_vesicle+membrane	0.162632	0.352535	0.111116
BP_GO:0006691_leukotriene+metabolic+process	0.631638	0.933366	0.0953455
BP_GO:0042755_eating+behavior	0.299716	0.250031	0.47215
BP_GO:0006309_DNA+fragmentation+during+apoptosis	0.529838	0.66308	0.376021
BP_GO:0006783_heme+biosynthetic+process	0.838723	0.58539	0.881631
MF_GO:0016291_acyl-CoA+thioesterase+activity	0.572661	0.647295	0.452302
MF_GO:0015075_ion+transmembrane+transporter+activity	0.391465	0.627744	0.293442
MF_GO:0019903_protein+phosphatase+binding	0.23262	0.808606	0.0282489
BP_GO:0043330_response+to+exogenous+dsRNA	0.0769408	0.787109	0.0231334
BP_GO:0002026_regulation+of+the+force+of+heart+contraction	0.790696	0.572147	0.884777
MF_GO:0005385_zinc+ion+transmembrane+transporter+activity	0.574344	0.912579	0.137295
BP_GO:0051899_membrane+depolarization	0.254771	0.571618	0.132456
BP_GO:0030100_regulation+of+endocytosis	0.989758	0.995983	0.452308
BP_GO:0048010_vascular+endothelial+growth+factor+receptor+signaling+p	0.00972913	0.340824	0.00576513
CC_GO:0005761_mitochondrial+ribosome	0.56234	0.620767	0.46555
BP_GO:0001516_prostaglandin+biosynthetic+process	0.124626	0.748773	0.0346058
BP_GO:0046488_phosphatidylinositol+metabolic+process	0.299131	0.316492	0.410999
BP_GO:0019722_calcium-mediated+signaling	0.967544	0.963984	0.791669
BP_GO:0000045_autophagic+vacuole+formation	0.97077	0.504134	0.996167
BP_GO:0008630_DNA+damage+response,+signal+transduction+resulting+in	0.376584	0.0571155	0.811128
BP_GO:0050821_protein+stabilization	0.024938	0.0266163	0.264188
BP_GO:0010467_gene+expression	0.902077	0.182856	0.991981
BP_GO:0015672_monovalent+inorganic+cation+transport	0.190912	0.280904	0.237988
BP_GO:0006801_superoxide+metabolic+process	0.00233039	0.121101	0.00228582
BP_GO:0046928_regulation+of+neurotransmitter+secretion	0.0185271	0.246788	0.0232645
BP_GO:0006857_oligopeptide+transport	0.515978	0.279	0.782317
BP_GO:0009880_embryonic+pattern+specification	0.561679	0.834499	0.225595
CC_GO:0005876_spindle+microtubule	0.361861	0.661876	0.272765
BP_GO:0001833_inner+cell+mass+cell+proliferation	0.800406	0.824243	0.575966
BP_GO:0006352_transcription+initiation	0.5675	0.505137	0.599544
BP_GO:0001556_oocyte+maturation	0.0585459	0.041074	0.362724
CC_GO:0016604_nuclear+body	0.928781	0.946136	0.719305

BP_GO:0002052_positive+regulation+of+neuroblast+proliferation	0.0498118	0.422084	0.0163919
MF_GO:0004602_glutathione+peroxidase+activity	0.0019496	0.00990548	0.0160153
MF_GO:0031625_ubiquitin+protein+ligase+binding	0.995634	0.900961	0.992302
CC_GO:0032420_stereocilium	0.126558	0.294529	0.140508
BP_GO:0006446_regulation+of+translational+initiation	0.745642	0.375061	0.924339
CC_GO:0005657_replication+fork	0.655769	0.480189	0.756239
CC_GO:0005892_nicotinic+acetylcholine-gated+receptor-channel+complex	0.671937	0.779442	0.443856
BP_GO:0045494_photoreceptor+cell+maintenance	0.602167	0.649517	0.492598
MF_GO:0008509_anion+transmembrane+transporter+activity	0.381296	0.20347	0.612477
MF_GO:0001664_G-protein-coupled+receptor+binding	0.786292	0.99253	0.167945
BP_GO:0006183_GTP+biosynthetic+process	0.0685833	0.329698	0.0480808
MF_GO:0015035_protein+disulfide+oxidoreductase+activity	0.606474	0.552579	0.604282
BP_GO:0006182_cGMP+biosynthetic+process	0.687262	0.742722	0.586957
BP_GO:0007257_activation+of+JNK+activity	0.24178	0.455903	0.188977
BP_GO:0048566_embryonic+gut+development	0.900223	0.5633	0.93649
BP_GO:0019226_transmission+of+nerve+impulse	0.139955	0.471789	0.120271
BP_GO:0007631_feeding+behavior	0.461833	0.0636521	0.65753
BP_GO:0010212_response+to+ionizing+radiation	0.135072	0.435244	0.0981546
MF_GO:0043022_ribosome+binding	0.969621	0.777109	0.978938
BP_GO:0006402_mRNA+catabolic+process	0.532422	0.591609	0.437932
BP_GO:0060052_neurofilament+cytoskeleton+organization	0.00634357	0.0278647	0.0399104
BP_GO:0048266_behavioral+response+to+pain	0.0507978	0.547863	0.0208299
MF_GO:0004843_ubiquitin-specific+protease+activity	0.98715	0.954774	0.950373
MF_GO:0030983_mismatched+DNA+binding	0.644166	0.40709	0.72404
BP_GO:0021549_cerebellum+development	0.710858	0.617708	0.695566
BP_GO:0043523_regulation+of+neuron+apoptosis	0.765938	0.224404	0.913421
MF_GO:0008271_secondary+active+sulfate+transmembrane+transporter+a	0.582899	0.478884	0.602514
MF_GO:0004983_neuropeptide+Y+receptor+activity	0.957118	0.792073	0.939938
BP_GO:0016265_death	0.524986	0.167462	0.899636
BP_GO:0030238_male+sex+determination	0.473915	0.48073	0.481876
CC_GO:0015934_large+ribosomal+subunit	0.72789	0.787864	0.315048
BP_GO:0000050_urea+cycle	0.901476	0.868164	0.75348
BP_GO:0005978_glycogen+biosynthetic+process	0.88903	0.765154	0.947536
BP_GO:0016310_phosphorylation	0.0755098	0.0755098	#N/A
MF_GO:0048407_platelet-derived+growth+factor+binding	0.274764	0.365773	0.290019
BP_GO:0001916_positive+regulation+of+T+cell+mediated+cytotoxicity	0.384565	0.113115	0.68959
BP_GO:0050730_regulation+of+peptidyl-tyrosine+phosphorylation	0.780016	0.562561	0.797879
BP_GO:0000910_cytokinesis	0.493675	0.23399	0.688855
MF_GO:0016811_hydrolase+activity,+acting+on+carbon-nitrogen+(but+not+	0.751238	0.615447	0.741254
BP_GO:0042135_neurotransmitter+catabolic+process	0.333204	0.213321	0.716314
BP_GO:0006487_protein+amino+acid+N-linked+glycosylation	0.586836	0.638424	0.472958
BP_GO:0060048_cardiac+muscle+contraction	0.921075	0.822508	0.860974
MF_GO:0004017_adenylate+kinase+activity	0.123609	0.317927	0.0905484
MF_GO:0019205_nucleobase,+nucleoside,+nucleotide+kinase+activity	0.208247	0.519733	0.0343773
MF_GO:0017127_cholesterol+transporter+activity	0.455498	0.379936	0.548677
BP_GO:0045766_positive+regulation+of+angiogenesis	0.142598	0.131694	0.361416
MF_GO:0016651_oxidoreductase+activity,+acting+on+NADH+or+NADPH	0.236474	0.158015	0.752547

BP_GO:0035137_hindlimb+morphogenesis	0.42638	0.37364	0.515179
MF_GO:0005542_folic+acid+binding	0.414212	0.314475	0.555759
BP_GO:0046622_positive+regulation+of+organ+growth	0.568642	0.813883	0.167502
MF_GO:0016538_cyclin-dependent+protein+kinase+regulator+activity	0.137523	0.0367283	0.641361
BP_GO:0030512_negative+regulation+of+transforming+growth+factor+beta	0.243081	0.440376	0.211372
MF_GO:0003854_3-beta-hydroxy-delta5-steroid+dehydrogenase+activity	0.797352	0.43822	0.847595
BP_GO:0008632_apoptotic+program	0.861465	0.915351	0.590574
BP_GO:0043407_negative+regulation+of+MAP+kinase+activity	0.709006	0.640893	0.688725
BP_GO:0046641_positive+regulation+of+alpha-beta+T+cell+proliferation	0.00298108	0.110702	0.00610703
BP_GO:0045582_positive+regulation+of+T+cell+differentiation	0.994858	0.922321	0.988158
BP_GO:0035162_embryonic+hemopoiesis	0.245825	0.70365	0.179116
MF_GO:0005391_sodium:potassium-exchanging+ATPase+activity	0.232833	0.0847798	0.846552
BP_GO:0006270_DNA+replication+initiation	0.92511	0.78187	0.888238
MF_GO:0046332_SMAD+binding	0.577083	0.461995	0.722561
MF_GO:0008191_metalloendopeptidase+inhibitor+activity	0.410272	0.277688	0.670051
CC_GO:0005759_mitochondrial+matrix	0.808525	0.821607	0.582192
MF_GO:0019841_retinol+binding	0.00532618	0.000442362	0.60867
BP_GO:0006878_cellular+copper+ion+homeostasis	0.33644	0.191255	0.692381
MF_GO:0008253_5'-nucleotidase+activity	0.337873	0.0393546	0.843714
BP_GO:0006548_histidine+catabolic+process	0.831182	0.800746	0.689406
BP_GO:0045060_negative+thymic+T+cell+selection	0.0904934	0.60756	0.0103222
BP_GO:0006800_oxygen+and+reactive+oxygen+species+metabolic+process	0.0862934	0.065968	0.313945
CC_GO:0005771_multivesicular+body	0.912476	0.987023	0.324361
BP_GO:0009395_phospholipid+catabolic+process	0.0822981	0.452565	0.0526825
MF_GO:0004702_receptor+signaling+protein+serine/threonine+kinase+activ	0.93031	0.841696	0.864877
BP_GO:0045453_bone+resorption	0.917136	0.760435	0.890055
BP_GO:0030889_negative+regulation+of+B+cell+proliferation	0.386467	0.729833	0.256285
BP_GO:0006744_ubiquinone+biosynthetic+process	0.334148	0.387712	0.353019
MF_GO:0016303_1-phosphatidylinositol-3-kinase+activity	0.835281	0.829957	0.75722
BP_GO:0019752_carboxylic+acid+metabolic+process	0.852689	0.668971	0.835066
MF_GO:0008375_acetylglucosaminyltransferase+activity	0.0016132	0.0971026	0.00355067
BP_GO:0046854_phosphoinositide+phosphorylation	0.951887	0.9272	0.843486
BP_GO:0051607_defense+response+to+virus	0.0615873	0.239987	0.0751168
MF_GO:0008195_phosphatidate+phosphatase+activity	0.808967	0.918118	0.534309
CC_GO:0035253_ciliary+rootlet	0.886276	0.654974	0.936641
BP_GO:0021954_central+nervous+system+neuron+development	0.529664	0.486142	0.587803
MF_GO:0015198_oligopeptide+transporter+activity	0.655781	0.436351	0.782317
BP_GO:0017156_calcium+ion-dependent+exocytosis	0.994231	0.96096	0.965182
BP_GO:0021952_central+nervous+system+projection+neuron+axonogenesis	0.815149	0.297867	0.973738
CC_GO:0008180_signalosome	0.983355	0.987432	0.627859
MF_GO:0008276_protein+methyltransferase+activity	0.654699	0.560976	0.664531
MF_GO:0015485_cholesterol+binding	0.409704	0.698748	0.219718
CC_GO:0005776_autophagic+vacuole	0.892345	0.467149	0.974483
BP_GO:0006376_mRNA+splice+site+selection	0.573533	0.563578	0.537347
BP_GO:0042542_response+to+hydrogen+peroxide	0.811524	0.717834	0.749958
BP_GO:0008629_induction+of+apoptosis+by+intracellular+signals	0.519534	0.229825	0.673515
BP_GO:0016051_carbohydrate+biosynthetic+process	0.271117	0.876979	0.0316995

MF_GO:0003841_1-acylglycerol-3-phosphate+O-acyltransferase+activity	0.737749	0.406375	0.888615
BP_GO:0000186_activation+of+MAPKK+activity	0.813181	0.906941	0.442735
BP_GO:0007032_endosome+organization	0.961692	0.924858	0.893405
BP_GO:0000188_inactivation+of+MAPK+activity	0.0788174	0.177055	0.120502
MF_GO:0030234_enzyme+regulator+activity	0.991205	0.99473	0.81567
CC_GO:0042470_melanosome	0.0638756	0.251645	0.0739617
BP_GO:0030325_adrenal+gland+development	0.173269	0.311332	0.204818
BP_GO:0051209_release+of+sequestered+calcium+ion+into+cytosol	0.70856	0.250137	0.87459
BP_GO:0050673_epithelial+cell+proliferation	0.881104	0.0544237	0.99504
CC_GO:0005641_nuclear+envelope+lumen	0.542585	0.741018	0.286559
BP_GO:0031295_T+cell+costimulation	0.034643	0.716875	0.0165919
BP_GO:0030316_osteoclast+differentiation	0.815341	0.774089	0.714793
BP_GO:0033198_response+to+ATP	0.797275	0.938556	0.229056
BP_GO:0001967_suckling+behavior	0.405507	0.350659	0.490916
BP_GO:0045671_negative+regulation+of+osteoclast+differentiation	0.250053	0.360955	0.282471
BP_GO:0006306_DNA+methylation	0.351957	0.332602	0.450603
BP_GO:0045668_negative+regulation+of+osteoblast+differentiation	0.700675	0.494665	0.743284
CC_GO:0033178_proton-transporting+two-sector+ATPase+complex,+catalyt	0.779061	0.895717	0.327306
BP_GO:0043525_positive+regulation+of+neuron+apoptosis	0.403111	0.126739	0.629593
BP_GO:0048148_behavioral+response+to+cocaine	0.730481	0.784404	0.547654
BP_GO:0048872_homeostasis+of+number+of+cells	0.666721	0.637753	0.597595
BP_GO:0031047_gene+silencing+by+RNA	0.9296	0.807488	0.895316
MF_GO:0004003_ATP-dependent+DNA+helicase+activity	0.416552	0.253974	0.582704
BP_GO:0043616_keratinocyte+proliferation	0.175138	0.320563	0.201021
MF_GO:0016780_phosphotransferase+activity,+for+other+substituted+phos	0.890213	0.920152	0.554716
CC_GO:0000922_spindle+pole	0.765857	0.660985	0.841916
BP_GO:0050804_regulation+of+synaptic+transmission	0.637469	0.834112	0.288274
MF_GO:0019825_oxygen+binding	0.988263	0.98198	0.854419
BP_GO:0002087_regulation+of+respiratory+gaseous+exchange+by+neurolo	0.822381	0.685528	0.820668
MF_GO:0003796_lysozyme+activity	0.397099	0.371819	0.476606
BP_GO:0014032_neural+crest+cell+development	0.180939	0.107892	0.297733
BP_GO:0016601_Rac+protein+signal+transduction	0.676273	0.38969	0.841024
BP_GO:0000077_DNA+damage+checkpoint	0.717985	0.951355	0.239056
BP_GO:0001508_regulation+of+action+potential	0.510113	0.433377	0.610347
CC_GO:0043231_intracellular+membrane-bounded+organelle	0.789455	0.937095	0.385951
MF_GO:0015299_solute:hydrogen+antiporter+activity	0.690046	0.703482	0.574143
CC_GO:0043198_dendritic+shaft	0.99836	0.991266	0.965673
CC_GO:0031461_cullin-RING+ubiquitin+ligase+complex	0.989981	0.900961	0.979855
CC_GO:0030126_COPI+vesicle+coat	0.645325	0.325736	0.780845
BP_GO:0048146_positive+regulation+of+fibroblast+proliferation	0.145105	0.675663	0.0744414
MF_GO:0030553_cGMP+binding	0.134149	0.0787535	0.360067
CC_GO:0005680_anaphase-promoting+complex	0.86925	0.943506	0.465
CC_GO:0016272_prefoldin+complex	0.629021	0.604223	0.594606
MF_GO:0004383_guanylate+cyclase+activity	0.411376	0.410929	0.436962
CC_GO:0005844_polysome	0.921886	0.974546	0.47776
BP_GO:0021915_neural+tube+development	0.341593	0.6982	0.192759
MF_GO:0005452_inorganic+anion+exchanger+activity	0.455601	0.164727	0.764674

BP_GO:0007586_digestion	0.333503	0.355447	0.372165
BP_GO:0006959_humoral+immune+response	0.777177	0.41834	0.835068
BP_GO:0050877_neurological+system+process	0.296485	0.357161	0.346745
BP_GO:0016486_peptide+hormone+processing	0.762622	0.427263	0.842424
BP_GO:0016339_calcium-dependent+cell-cell+adhesion	0.205251	0.30915	0.248463
BP_GO:0050910_detection+of+mechanical+stimulus+involved+in+sensory+p	0.750717	0.951655	0.452241
BP_GO:0000724_double-strand+break+repair+via+homologous+recombinat	0.24987	0.530834	0.164506
MF_GO:0016494_C-X-C+chemokine+receptor+activity	0.814444	0.727385	0.747001
MF_GO:0009881_photoreceptor+activity	0.537089	0.421733	0.640396
BP_GO:0006228_UTP+biosynthetic+process	0.073062	0.329698	0.0455243
BP_GO:0006241_CTP+biosynthetic+process	0.073062	0.329698	0.0455243
MF_GO:0004550_nucleoside+diphosphate+kinase+activity	0.073062	0.329698	0.0455243
MF_GO:0004526_ribonuclease+P+activity	0.483411	0.46047	0.512752
MF_GO:0008417_fucosyltransferase+activity	0.467238	0.930162	0.0760572
CC_GO:0016585_chromatin+remodeling+complex	0.763422	0.542269	0.807054
BP_GO:0002076_osteoblast+development	0.38537	0.782108	0.0950967
BP_GO:0001542_ovulation+from+ovarian+follicle	0.140168	0.155026	0.331257
BP_GO:0006476_protein+amino+acid+deacetylation	0.964761	0.862242	0.963975
BP_GO:0007141_male+meiosis+I	0.163529	0.131462	0.303439
CC_GO:0005832_chaperonin-containing+T-complex	0.737282	0.635502	0.728907
CC_GO:0042788_polysomal+ribosome	0.00313122	0.021937	0.00606608
CC_GO:0031674_I+band	0.664689	0.700392	0.530019
CC_GO:0042627_chylomicron	0.0601147	0.154613	0.119018
MF_GO:0004303_estradiol+17-beta-dehydrogenase+activity	0.242352	0.0266221	0.827184
CC_GO:0043292_contractile+fiber	0.235269	0.235269	#N/A
CC_GO:0005901_caveola	0.514312	0.274265	0.866683
MF_GO:0015020_glucuronosyltransferase+activity	0.230323	0.41247	0.205005
MF_GO:0016298_lipase+activity	0.744103	0.888695	0.385057
MF_GO:0005080_protein+kinase+C+binding	0.901733	0.857781	0.767056
BP_GO:0045059_positive+thymic+T+cell+selection	0.49798	0.153283	0.960913
BP_GO:0009435_NAD+biosynthetic+process	0.553155	0.829364	0.155799
BP_GO:0042572_retinol+metabolic+process	0.382913	0.322915	0.514787
BP_GO:0050798_activated+T+cell+proliferation	0.185773	0.472667	0.17617
BP_GO:0043409_negative+regulation+of+MAPKKK+cascade	0.240536	0.382855	0.241935
BP_GO:0045576_mast+cell+activation	0.157972	0.447411	0.0708491
CC_GO:0030864_cortical+actin+cytoskeleton	0.11737	0.175551	0.226607
BP_GO:0030031_cell+projection+assembly	0.893436	0.785379	0.832134
BP_GO:0045579_positive+regulation+of+B+cell+differentiation	0.146879	0.526771	0.0356192
MF_GO:0043621_protein+self-association	0.650125	0.694443	0.489016
BP_GO:0009165_nucleotide+biosynthetic+process	0.917242	0.973117	0.410747
CC_GO:0005765_lysosomal+membrane	0.652045	0.314221	0.849903
BP_GO:0000060_protein+import+into+nucleus,+translocation	0.626177	0.142421	0.891459
BP_GO:0050796_regulation+of+insulin+secretion	0.812348	0.707014	0.771705
MF_GO:0004745_retinol+dehydrogenase+activity	0.296041	0.429893	0.280075
MF_GO:0008121_ubiquinol-cytochrome-c+reductase+activity	0.624493	0.644157	0.516005
BP_GO:0045086_positive+regulation+of+interleukin-2+biosynthetic+process	0.141709	0.181719	0.28077
BP_GO:0002504_antigen+processing+and+presentation+of+peptide+or+poly	0.911457	0.988616	0.477704

CC_GO:0042613_MHC+class+II+protein+complex	0.911457	0.988616	0.477704
BP_GO:0043011_myeloid+dendritic+cell+differentiation	0.0753139	0.114731	0.212367
BP_GO:0018345_protein+palmitoylation	0.941485	0.968911	0.461725
MF_GO:0016409_palmitoyltransferase+activity	0.941485	0.968911	0.461725
BP_GO:0042104_positive+regulation+of+activated+T+cell+proliferation	0.109826	0.00980238	0.724347
CC_GO:0046658_anchored+to+plasma+membrane	0.14503	0.440426	0.110536
MF_GO:0005021_vascular+endothelial+growth+factor+receptor+activity	0.0371103	0.385644	0.00548473
CC_GO:0005952_cAMP-dependent+protein+kinase+complex	0.679449	0.510475	0.715222
BP_GO:0007173_epidermal+growth+factor+receptor+signaling+pathway	0.771168	0.964552	0.224785
MF_GO:0030675_Rac+GTPase+activator+activity	0.0379423	0.547734	8.36038E-05
BP_GO:0045765_regulation+of+angiogenesis	0.101277	0.138897	0.250955
MF_GO:0051018_protein+kinase+A+binding	0.290303	0.28788	0.445333
CC_GO:0005881_cytoplasmic+microtubule	0.597334	0.788659	0.48424
BP_GO:0006835_dicarboxylic+acid+transport	0.285843	0.517395	0.163077
BP_GO:0030500_regulation+of+bone+mineralization	0.0507934	0.0165008	0.661555
BP_GO:0001933_negative+regulation+of+protein+amino+acid+phosphorylat	0.944966	0.634023	0.960457
BP_GO:0050870_positive+regulation+of+T+cell+activation	0.680452	0.736234	0.479813
MF_GO:0017017_MAP+kinase+tyrosine/serine/threonine+phosphatase+acti	0.12676	0.259567	0.150383
CC_GO:0043296_apical+junction+complex	0.496425	0.894543	0.163342
BP_GO:0001952_regulation+of+cell-matrix+adhesion	0.811496	0.540805	0.874246
MF_GO:0050750_low-density+lipoprotein+receptor+binding	0.540163	0.29379	0.753029
BP_GO:0001678_cellular+glucose+homeostasis	0.951524	0.961396	0.865945
MF_GO:0008199_ferric+iron+binding	0.147681	0.407866	0.0791921
BP_GO:0006996_organelle+organization	0.722769	0.642817	0.708112
MF_GO:0016307_phosphatidylinositol+phosphate+kinase+activity	0.132588	0.123445	0.410999
BP_GO:0006940_regulation+of+smooth+muscle+contraction	0.202383	0.138652	0.415244
BP_GO:0008637_apoptotic+mitochondrial+changes	0.608102	0.728069	0.307059
BP_GO:0016079_synaptic+vesicle+exocytosis	0.981509	0.991293	0.716694
BP_GO:0009267_cellular+response+to+starvation	0.460578	0.474289	0.46948
BP_GO:0050884_neuromuscular+process+controlling+posture	0.34136	0.170078	0.797861
MF_GO:0042277_peptide+binding	0.916629	0.954443	0.670342
BP_GO:0007188_G-protein+signaling,+coupled+to+cAMP+nucleotide+secon	0.370594	0.167969	0.689285
BP_GO:0006779_porphyrin+biosynthetic+process	0.48361	0.469515	0.519566
BP_GO:0006939_smooth+muscle+contraction	0.303446	0.202506	0.541251
BP_GO:0042100_B+cell+proliferation	0.589553	0.255339	0.786663
MF_GO:0016918_retinal+binding	0.0361549	0.00125135	0.832972
BP_GO:0044262_cellular+carbohydrate+metabolic+process	0.219323	0.342632	0.130932
BP_GO:0021702_cerebellar+Purkinje+cell+differentiation	0.549085	0.69815	0.402582
BP_GO:0042554_superoxide+release	0.00117495	0.06998	0.00108114
MF_GO:0015377_cation:chloride+symporter+activity	0.837174	0.710637	0.798056
MF_GO:0008536_Ran+GTPase+binding	0.347142	0.594958	0.114091
CC_GO:0016581_NuRD+complex	0.503295	0.564696	0.439514
BP_GO:0051605_protein+maturation+via+proteolysis	0.99446	0.976739	0.962875
BP_GO:0060216_definitive+hemopoiesis	0.665239	0.649705	0.595426
BP_GO:0000302_response+to+reactive+oxygen+species	0.460337	0.639525	0.206109
BP_GO:0001841_neural+tube+formation	0.844167	0.941946	0.525071
CC_GO:0031966_mitochondrial+membrane	0.710114	0.649103	0.709835

BP_GO:0050770_regulation+of+axonogenesis	0.0468814	0.256438	0.0531531
MF_GO:0048306_calcium-dependent+protein+binding	0.745793	0.933913	0.193558
BP_GO:0035136_forelimb+morphogenesis	0.368993	0.332365	0.578993
BP_GO:0019800_peptide+cross-linking+via+chondroitin+4-sulfate+glycosam	0.0615277	0.0902635	0.199301
MF_GO:0008889_glycerophosphodiester+phosphodiesterase+activity	0.514371	0.715311	0.386955
BP_GO:0046907_intracellular+transport	0.535281	0.421582	0.604251
BP_GO:0007193_G-protein+signaling,+adenylate+cyclase+inhibiting+pathwa	0.335986	0.912698	0.0561678
BP_GO:0007625_grooming+behavior	0.0774971	0.087379	0.283405
BP_GO:0007130_synaptonemal+complex+assembly	0.761334	0.585212	0.811202
MF_GO:0019894_kinesin+binding	0.904951	0.891275	0.707997
BP_GO:0021953_central+nervous+system+neuron+differentiation	0.343213	0.173423	0.58573
BP_GO:0006626_protein+targeting+to+mitochondrion	0.818212	0.673314	0.798329
BP_GO:0009755_hormone-mediated+signaling	0.262951	0.0839044	0.604241
CC_GO:0005815_microtubule+organizing+center	0.871174	0.702369	0.877569
CC_GO:0031519_PcG+protein+complex	0.401892	0.131724	0.709017
CC_GO:0009925_basal+plasma+membrane	0.149536	0.162976	0.312697
BP_GO:0021537_telencephalon+development	0.847241	0.951663	0.318491
BP_GO:0007191_dopamine+receptor,+adenylate+cyclase+activating+pathwa	0.183015	0.373295	0.169269
BP_GO:0042325_regulation+of+phosphorylation	0.201836	0.622659	0.0968747
BP_GO:0001659_temperature+homeostasis	0.182342	0.563194	0.127255
CC_GO:0005801_cis-Golgi+network	0.846333	0.44857	0.966703
BP_GO:0032729_positive+regulation+of+interferon-gamma+production	0.990071	0.654574	0.998117
BP_GO:0006983_ER+overload+response	0.804509	0.58313	0.807583
BP_GO:0008340_determination+of+adult+life+span	0.0995237	0.317505	0.11613
BP_GO:0030850_prostate+gland+development	0.588672	0.606559	0.52927
BP_GO:0002027_regulation+of+heart+rate	0.21754	0.348566	0.236882
BP_GO:0060113_inner+ear+receptor+cell+differentiation	0.888462	0.948721	0.347558
MF_GO:0047555_3',5'-cyclic-GMP+phosphodiesterase+activity	0.14837	0.618181	0.083892
BP_GO:0021904_dorsoventral+neural+tube+patterning	0.959028	0.944289	0.83348
MF_GO:0004945_angiotensin+type+II+receptor+activity	0.692523	0.521158	0.708724
BP_GO:0008361_regulation+of+cell+size	0.355299	0.0744561	0.741206
MF_GO:0005246_calcium+channel+regulator+activity	0.345648	0.658423	0.119522
BP_GO:0045176_apical+protein+localization	0.627675	0.810064	0.447888
CC_GO:0005763_mitochondrial+small+ribosomal+subunit	0.37242	0.600062	0.137295
BP_GO:0001655_urogenital+system+development	0.71744	0.812116	0.471038
MF_GO:0008601_protein+phosphatase+type+2A+regulator+activity	0.906552	0.947321	0.597663
MF_GO:0042393_histone+binding	0.354074	0.588877	0.12705
BP_GO:0030432_peristalsis	0.861533	0.971114	0.359483
BP_GO:0009617_response+to+bacterium	0.309396	0.306834	0.420631
MF_GO:0008094_DNA-dependent+ATPase+activity	0.495645	0.650214	0.406754
BP_GO:0007212_dopamine+receptor+signaling+pathway	0.546875	0.888996	0.145587
CC_GO:0000228_nuclear+chromosome	0.926006	0.904181	0.770499
BP_GO:0045471_response+to+ethanol	0.64518	0.464748	0.704992
BP_GO:0007129_synapsis	0.629816	0.632771	0.573923
BP_GO:0048168_regulation+of+neuronal+synaptic+plasticity	0.0370952	0.123439	0.0853935
MF_GO:0008158_hedgehog+receptor+activity	0.148905	0.183285	0.267843
CC_GO:0000790_nuclear+chromatin	0.530592	0.658427	0.381876

BP_GO:0042220_response+to+cocaine	0.644137	0.827896	0.395289
CC_GO:0005677_chromatin+silencing+complex	0.661464	0.62197	0.638859
CC_GO:0043209_myelin+sheath	0.423641	0.730178	0.187568
BP_GO:0048240_sperm+capacitation	0.389998	0.245881	0.570612
BP_GO:0006032_chitin+catabolic+process	0.424883	0.506747	0.399875
MF_GO:0004568_chitinase+activity	0.424883	0.506747	0.399875
BP_GO:0006611_protein+export+from+nucleus	0.726594	0.565965	0.820533
BP_GO:0042074_cell+migration+involved+in+gastrulation	0.938151	0.917517	0.764727
BP_GO:0007435_salivary+gland+morphogenesis	0.198547	0.310299	0.24533
BP_GO:0042438_melanin+biosynthetic+process	0.288134	0.21624	0.381367
BP_GO:0016579_protein+deubiquitination	0.983165	0.972934	0.8597
BP_GO:0015991_ATP+hydrolysis+coupled+proton+transport	0.57992	0.742691	0.123611
BP_GO:0016446_somatic+hypermutation+of+immunoglobulin+genes	0.412462	0.381766	0.467038
MF_GO:0050699_WW+domain+binding	0.00767167	0.148012	0.00850373
BP_GO:0001539_ciliary+or+flagellar+motility	0.437844	0.616875	0.33396
MF_GO:0004522_pancreatic+ribonuclease+activity	0.000127773	0.000396777	0.0705989
BP_GO:0007140_male+meiosis	0.355314	0.215856	0.555024
BP_GO:0045109_intermediate+filament+organization	0.666289	0.377349	0.750409
BP_GO:0007131_reciprocal+meiotic+recombination	0.359434	0.211698	0.518033
MF_GO:0005248_voltage-gated+sodium+channel+activity	0.0137684	0.024342	0.125408
CC_GO:0022625_cytosolic+large+ribosomal+subunit	0.869501	0.955819	0.0912925
CC_GO:0005885_Arp2/3+protein+complex	0.489346	0.602122	0.3666
MF_GO:0016810_hydrolase+activity,+acting+on+carbon-nitrogen+(but+not+	0.658042	0.736523	0.456435
BP_GO:0042311_vasodilation	0.854741	0.555911	0.877263
BP_GO:0006910_phagocytosis,+recognition	0.206263	0.316619	0.24113
BP_GO:0051259_protein+oligomerization	0.0388169	0.0553055	0.169748
BP_GO:0042692_muscle+cell+differentiation	0.280839	0.368479	0.316231
CC_GO:0046581_intercellular+canaliculus	0.514701	0.838087	0.280753
MF_GO:0004029_aldehyde+dehydrogenase+(NAD)+activity	0.787088	0.632341	0.795567
BP_GO:0006013_mannose+metabolic+process	0.60275	0.541049	0.609518
BP_GO:0008206_bile+acid+metabolic+process	0.770082	0.212877	0.915797
MF_GO:0008508_bile+acid:sodium+symporter+activity	0.648581	0.48371	0.705182
BP_GO:0006656_phosphatidylcholine+biosynthetic+process	0.225822	0.126454	0.497093
BP_GO:0007520_myoblast+fusion	0.733076	0.712614	0.606842
MF_GO:0004365_glyceraldehyde-3-phosphate+dehydrogenase+(phosphoryl	0.971558	0.78548	0.958558
MF_GO:0008943_glyceraldehyde-3-phosphate+dehydrogenase+activity	0.971558	0.78548	0.958558
BP_GO:0045785_positive+regulation+of+cell+adhesion	0.392289	0.265427	0.571365
BP_GO:0030866_cortical+actin+cytoskeleton+organization	0.476455	0.590981	0.390397
BP_GO:0007044_cell-substrate+junction+assembly	0.709516	0.740595	0.504645
BP_GO:0010259_multicellular+organismal+aging	0.279396	0.358626	0.300423
BP_GO:0008277_regulation+of+G-protein+coupled+receptor+protein+signal	0.708233	0.67275	0.638224
BP_GO:0009086_methionine+biosynthetic+process	0.401437	0.282778	0.565983
BP_GO:0046034_ATP+metabolic+process	0.948083	0.947188	0.686016
BP_GO:0030148_sphingolipid+biosynthetic+process	0.244309	0.632463	0.113046
MF_GO:0005100_Rho+GTPase+activator+activity	0.338738	0.659377	0.182189
MF_GO:0004630_phospholipase+D+activity	0.951313	0.645148	0.96932
BP_GO:0006108_malate+metabolic+process	0.0532364	0.430147	0.0117387

BP_GO:0043353_enucleate+erythrocyte+differentiation	0.446865	0.299594	0.656063
BP_GO:0006044_N-acetylglucosamine+metabolic+process	0.40017	0.61549	0.3136
BP_GO:0006805_xenobiotic+metabolic+process	0.502498	0.578553	0.412772
BP_GO:0030225_macrophage+differentiation	0.375846	0.456172	0.360307
BP_GO:0006672_ceramide+metabolic+process	0.850108	0.749873	0.789733
BP_GO:0042310_vasoconstriction	0.262691	0.45119	0.249861
BP_GO:0007250_activation+of+NF-kappaB-inducing+kinase+activity	0.559469	0.677845	0.325784
BP_GO:0006729_tetrahydrobiopterin+biosynthetic+process	0.52521	0.355226	0.859291
BP_GO:0050772_positive+regulation+of+axonogenesis	0.19194	0.528217	0.0405598
BP_GO:0016575_histone+deacetylation	0.655711	0.292807	0.89276
MF_GO:0051920_peroxiredoxin+activity	0.853098	0.749972	0.815374
BP_GO:0007270_nerve-nerve+synaptic+transmission	0.175519	0.536148	0.122639
BP_GO:0019222_regulation+of+metabolic+process	0.135944	0.0753565	0.491635
MF_GO:0005545_phosphatidylinositol+binding	0.978766	0.341132	0.996085
MF_GO:0017153_sodium:dicarboxylate+symporter+activity	0.426662	0.517395	0.338513
BP_GO:0006750_glutathione+biosynthetic+process	0.272429	0.220876	0.445778
BP_GO:0002011_morphogenesis+of+an+epithelial+sheet	0.47266	0.272495	0.796217
BP_GO:0006546_glycine+catabolic+process	0.618953	0.690974	0.411671
MF_GO:0045296_cadherin+binding	0.959127	0.939348	0.831861
MF_GO:0005501_retinoid+binding	0.00296013	0.000168516	0.9332
BP_GO:0030838_positive+regulation+of+actin+filament+polymerization	0.632981	0.484544	0.685209
BP_GO:0045843_negative+regulation+of+striated+muscle+development	0.278283	0.627651	0.100974
MF_GO:0003956_NAD(P)+-protein-arginine+ADP-ribosyltransferase+activity	0.415695	0.241582	0.761043
CC_GO:0045261_proton-transporting+ATP+synthase+complex,+catalytic+co	0.98639	0.998691	0.0627835
BP_GO:0048147_negative+regulation+of+fibroblast+proliferation	0.163163	0.109886	0.406082
BP_GO:0017157_regulation+of+exocytosis	0.960859	0.375404	0.998884
BP_GO:0046328_regulation+of+JNK+cascade	0.996712	0.996712	#N/A
BP_GO:0032755_positive+regulation+of+interleukin-6+production	0.442735	0.889127	0.172743
BP_GO:0021756_striatum+development	0.690496	0.682753	0.583166
BP_GO:0048854_brain+morphogenesis	0.938424	0.77043	0.933289
BP_GO:0006584_catecholamine+metabolic+process	0.83321	0.52442	0.920192
MF_GO:0031177_phosphopantetheine+binding	0.155547	0.17803	0.314326
MF_GO:0008528_peptide+receptor+activity,+G-protein+coupled	0.049719	0.0777045	0.189726
BP_GO:0001963_synaptic+transmission,+dopaminergic	0.246173	0.639583	0.149353
MF_GO:0017075_syntaxin-1+binding	0.88824	0.612697	0.884477
BP_GO:0002064_epithelial+cell+development	0.462844	0.492004	0.457382
BP_GO:0007413_axonal+fasciculation	0.186048	0.562285	0.060976
BP_GO:0006098_pentose-phosphate+shunt	0.898679	0.898679	#N/A
BP_GO:0007271_synaptic+transmission,+cholinergic	0.222002	0.630237	0.13203
MF_GO:0004931_ATP-gated+cation+channel+activity	0.827739	0.975432	0.203725
MF_GO:0015385_sodium:hydrogen+antiporter+activity	0.665386	0.780992	0.40371
BP_GO:0048070_regulation+of+pigmentation+during+development	0.657731	0.558584	0.658806
BP_GO:0045410_positive+regulation+of+interleukin-6+biosynthetic+process	0.885806	0.881467	0.6825
BP_GO:0031032_actomyosin+structure+organization	0.131681	0.104285	0.398007
BP_GO:0045084_positive+regulation+of+interleukin-12+biosynthetic+proces	0.852959	0.304357	0.941155
BP_GO:0009416_response+to+light+stimulus	0.374141	0.875201	0.0774431
MF_GO:0004622_lysophospholipase+activity	0.798514	0.73255	0.715235

BP_GO:0060088_auditory+receptor+cell+stereocilium+organization	0.569715	0.923837	0.156796
BP_GO:0008625_induction+of+apoptosis+via+death+domain+receptors	0.045795	0.405835	0.0026838
BP_GO:0002063_chondrocyte+development	0.525882	0.305471	0.654515
BP_GO:0043281_regulation+of+caspase+activity	0.367361	0.535159	0.219008
BP_GO:0009314_response+to+radiation	0.873294	0.951886	0.528121
BP_GO:0042551_neuron+maturation	0.918909	0.8748	0.787554
CC_GO:0000780_condensed+nuclear+chromosome,+centromeric+region	0.308943	0.483297	0.237207
BP_GO:0030279_negative+regulation+of+ossification	0.382222	0.755545	0.213964
MF_GO:0008440_inositol+trisphosphate+3-kinase+activity	0.853981	0.876898	0.478543
BP_GO:0035239_tube+morphogenesis	0.863184	0.776085	0.786925
BP_GO:0006817_phosphate+transport	0.318491	0.433302	0.298571
BP_GO:0002360_T+cell+lineage+commitment	0.389061	0.156079	0.542595
MF_GO:0005160_transforming+growth+factor+beta+receptor+binding	0.512091	0.343825	0.694845
BP_GO:0006924_activated+T+cell+apoptosis	0.10561	0.379481	0.0822609
MF_GO:0015077_monovalent+inorganic+cation+transmembrane+transporte	0.0174601	0.0325814	0.150654
MF_GO:0001609_adenosine+receptor+activity,+G-protein+coupled	0.137302	0.716399	0.0260475
BP_GO:0045880_positive+regulation+of+smoothened+signaling+pathway	0.921502	0.925985	0.579515
BP_GO:0040011_locomotion	0.930743	0.582443	0.992737
MF_GO:0005041_low-density+lipoprotein+receptor+activity	0.356993	0.127687	0.691008
CC_GO:0016328_lateral+plasma+membrane	0.645565	0.48502	0.730094
BP_GO:0010033_response+to+organic+substance	0.756903	0.918176	0.292764
BP_GO:0043249_erythrocyte+maturation	0.231136	0.265352	0.344298
BP_GO:0014059_regulation+of+dopamine+secretion	0.876442	0.925244	0.541256
MF_GO:0003727_single-stranded+RNA+binding	0.629882	0.734626	0.540665
MF_GO:0003678_DNA+helicase+activity	0.760949	0.709174	0.677576
BP_GO:0006268_DNA+unwinding+during+replication	0.790773	0.416731	0.862088
BP_GO:0043491_protein+kinase+B+signaling+cascade	0.774915	0.575637	0.824594
BP_GO:0060080_regulation+of+inhibitory+postsynaptic+membrane+potenti	0.341052	0.806018	0.0984368
BP_GO:0060037_pharyngeal+system+development	0.228726	0.47845	0.141279
MF_GO:0016165_lipoxygenase+activity	0.900684	0.97729	0.223723
BP_GO:0043488_regulation+of+mRNA+stability	0.473255	0.819595	0.123204
CC_GO:0000800_lateral+element	0.755297	0.947789	0.417345
MF_GO:0016500_protein-hormone+receptor+activity	0.159542	0.94032	0.00381408
MF_GO:0000149_SNARE+binding	0.993919	0.966775	0.958074
MF_GO:0004579_dolichyl-diphosphooligosaccharide-protein+glycotransfera	0.728708	0.740104	0.548031
BP_GO:0006349_genetic+imprinting	0.532549	0.52518	0.520273
BP_GO:0008585_female+gonad+development	0.103588	0.303741	0.121178
MF_GO:0004767_sphingomyelin+phosphodiesterase+activity	0.71807	0.353814	0.861622
CC_GO:0015030_Cajal+body	0.285822	0.287828	0.430589
MF_GO:0015280_amiloride-sensitive+sodium+channel+activity	0.0113333	0.821223	0.00051984
BP_GO:0006303_double-strand+break+repair+via+nonhomologous+end+joi	0.344439	0.259648	0.473119
CC_GO:0001739_sex+chromatin	0.452053	0.44519	0.483622
BP_GO:0048304_positive+regulation+of+isotype+switching+to+IgG+isotypes	0.595082	#N/A	0.595082
CC_GO:0042105_alpha-beta+T+cell+receptor+complex	0.0146995	0.0152926	0.255004
MF_GO:0003906_DNA-(apurinic+or+apyrimidinic+site)+lyase+activity	0.0523698	0.0927732	0.170531
CC_GO:0000242_pericentriolar+material	0.479384	0.558442	0.42205
MF_GO:0017136_NAD-dependent+histone+deacetylase+activity	0.807892	0.786576	0.638859

BP_GO:0060134_prepulse+inhibition	0.111418	0.239209	0.151845
BP_GO:0007616_long-term+memory	0.0967313	0.291516	0.0877079
MF_GO:0008188_neuropeptide+receptor+activity	0.355194	0.26892	0.516343
MF_GO:0003711_transcription+elongation+regulator+activity	0.902036	0.978322	0.360366
BP_GO:0009948_anterior/posterior+axis+specification	0.652102	0.93935	0.204737
MF_GO:0050733_RS+domain+binding	0.807741	0.922521	0.467599
CC_GO:0032391_photoreceptor+connecting+cilium	0.935096	0.868796	0.84621
BP_GO:0019229_regulation+of+vasoconstriction	0.33995	0.740125	0.134715
CC_GO:0031526_brush+border+membrane	0.826008	0.617102	0.837282
MF_GO:0004000_adenosine+deaminase+activity	0.748961	0.488349	0.855152
BP_GO:0007342_fusion+of+sperm+to+egg+plasma+membrane	0.0886141	0.303525	0.101535
BP_GO:0042176_regulation+of+protein+catabolic+process	0.812572	0.923792	0.473942
BP_GO:0060136_embryonic+process+involved+in+female+pregnancy	0.745655	0.623247	0.770257
CC_GO:0005786_signal+recognition+particle,+endoplasmic+reticulum+targe	0.763718	0.596989	0.79201
BP_GO:0032259_methylation	0.54403	0.751657	0.328867
MF_GO:0016251_general+RNA+polymerase+II+transcription+factor+activity	0.921131	0.771393	0.889759
BP_GO:0032355_response+to+estradiol+stimulus	0.0467655	0.159758	0.0874403
BP_GO:0046620_regulation+of+organ+growth	0.456205	0.74841	0.172965
BP_GO:0045954_positive+regulation+of+natural+killer+cell+mediated+cytot	0.070085	0.0238274	0.310194
BP_GO:0006559_L-phenylalanine+catabolic+process	0.261421	0.347138	0.304263
CC_GO:0005859_muscle+myosin+complex	0.284458	0.32805	0.350453
BP_GO:0042158_lipoprotein+biosynthetic+process	0.748757	0.287698	0.934234
CC_GO:0016012_sarcoglycan+complex	0.242674	0.0788364	0.66442
MF_GO:0008035_high-density+lipoprotein+binding	0.176884	0.176884	#N/A
BP_GO:0055003_cardiac+myofibril+assembly	0.176839	0.18614	0.336767
BP_GO:0006956_complement+activation	0.892664	0.840818	0.769792
BP_GO:0042574_retinal+metabolic+process	0.547844	0.295041	0.9332
MF_GO:0016868_intramolecular+transferase+activity,+phosphotransferases	0.504431	0.504431	#N/A
MF_GO:0005523_tropomyosin+binding	0.600297	0.465071	0.645227
BP_GO:0042953_lipoprotein+transport	0.135815	0.133335	0.286784
BP_GO:0002237_response+to+molecule+of+bacterial+origin	0.10445	0.181446	0.192315
BP_GO:0006693_prostaglandin+metabolic+process	0.157331	0.643613	0.102643
BP_GO:0046513_ceramide+biosynthetic+process	0.38077	0.737167	0.14342
MF_GO:0043499_eukaryotic+cell+surface+binding	0.186102	0.790717	0.0478805
MF_GO:0030151_molybdenum+ion+binding	0.116604	0.272742	0.112633
BP_GO:0002021_response+to+dietary+excess	0.199748	0.0377849	0.588078
BP_GO:0043406_positive+regulation+of+MAP+kinase+activity	0.337283	0.307044	0.436852
BP_GO:0060347_heart+trabecula+formation	0.954743	0.606114	0.994656
BP_GO:0030041_actin+filament+polymerization	0.0160804	0.049058	0.0839863
BP_GO:0010765_positive+regulation+of+sodium+ion+transport	0.771206	0.664971	0.807057
BP_GO:0032868_response+to+insulin+stimulus	0.328424	0.111586	0.721877
MF_GO:0003756_protein+disulfide+isomerase+activity	0.975753	0.984427	0.737573
MF_GO:0004115_3',5'-cyclic-AMP+phosphodiesterase+activity	0.0545753	0.00649797	0.769196
MF_GO:0017114_wide-spectrum+protease+inhibitor+activity	0.520442	0.67943	0.439369
BP_GO:0008015_blood+circulation	0.810578	0.684972	0.769451
BP_GO:0017015_regulation+of+transforming+growth+factor+beta+receptor	0.117935	0.137186	0.305163
BP_GO:0051604_protein+maturation	0.806006	0.709864	0.747824

MF_GO:0008308_voltage-gated+anion+channel+activity	0.743971	0.842776	0.259331
MF_GO:0016813_hydrolase+activity,+acting+on+carbon-nitrogen+(but+not+	0.318519	0.319232	0.421178
BP_GO:0048041_focal+adhesion+formation	0.682371	0.736237	0.515342
BP_GO:0050860_negative+regulation+of+T+cell+receptor+signaling+pathwa	0.788967	0.0881766	0.930932
BP_GO:0030856_regulation+of+epithelial+cell+differentiation	0.109008	0.111289	0.299967
BP_GO:0015813_L-glutamate+transport	0.852601	0.582859	0.935648
MF_GO:0017154_semaphorin+receptor+activity	0.477428	0.416616	0.629787
MF_GO:0030246_carbohydrate+binding	0.203225	0.238683	0.331774
MF_GO:0048185_activin+binding	0.599929	0.723076	0.456608
BP_GO:0009312_oligosaccharide+biosynthetic+process	0.190495	0.133695	0.320891
BP_GO:0048009_insulin-like+growth+factor+receptor+signaling+pathway	0.536236	0.38264	0.718715
BP_GO:0045445_myoblast+differentiation	0.291677	0.383298	0.297323
MF_GO:0016814_hydrolase+activity,+acting+on+carbon-nitrogen+(but+not+	0.174793	0.408001	0.162939
BP_GO:0007588_excretion	0.528179	0.597374	0.44136
MF_GO:0042605_peptide+antigen+binding	0.894079	0.967686	0.467323
BP_GO:0006882_cellular+zinc+ion+homeostasis	0.627259	0.699687	0.510751
CC_GO:0033017_sarcoplasmic+reticulum+membrane	0.0884503	0.178978	0.104564
BP_GO:0050790_regulation+of+catalytic+activity	0.331896	0.200708	0.523737
CC_GO:0030130_clathrin+coat+of+trans-Golgi+network+vesicle	0.00552247	0.0829759	0.0163603
BP_GO:0030098_lymphocyte+differentiation	0.257309	0.240888	0.381264
MF_GO:0001784_phosphotyrosine+binding	0.385396	0.276804	0.630044
MF_GO:0004047_aminomethyltransferase+activity	0.683463	0.690974	0.521291
BP_GO:0046902_regulation+of+mitochondrial+membrane+permeability	0.624979	0.724627	0.487193
MF_GO:0004571_mannosyl-oligosaccharide+1,2-alpha-mannosidase+activity	0.547379	0.646184	0.417777
CC_GO:0030496_midbody	0.816931	0.406972	0.898445
MF_GO:0005388_calcium-transporting+ATPase+activity	0.761488	0.883645	0.425396
BP_GO:0045885_positive+regulation+of+survival+gene+product+expression	0.0203949	0.0207248	0.195752
CC_GO:0000307_cyclin-dependent+protein+kinase+holoenzyme+complex	0.344144	0.211886	0.789773
MF_GO:0004396_hexokinase+activity	0.94887	0.972325	0.388511
MF_GO:0005154_epidermal+growth+factor+receptor+binding	0.961745	0.93852	0.812512
BP_GO:0006122_mitochondrial+electron+transport,+ubiquinol+to+cytochro	0.982918	0.959472	0.90127
CC_GO:0005954_calcium-+and+calmodulin-dependent+protein+kinase+com	0.190018	0.0480449	0.797227
BP_GO:0045599_negative+regulation+of+fat+cell+differentiation	0.274282	0.263141	0.414387
BP_GO:0048048_embryonic+eye+morphogenesis	0.46449	0.831704	0.0648177
BP_GO:0006790_sulfur+metabolic+process	0.649265	0.444633	0.714721
BP_GO:0045664_regulation+of+neuron+differentiation	0.199838	0.553147	0.129846
BP_GO:0016338_calcium-independent+cell-cell+adhesion	0.00249305	0.120999	0.00531524
BP_GO:0048103_somatic+stem+cell+division	0.825347	0.321827	0.962956
BP_GO:0006353_transcription+termination	0.617648	0.359398	0.847868
BP_GO:0006970_response+to+osmotic+stress	0.710196	0.856382	0.152588
BP_GO:0031647_regulation+of+protein+stability	0.977946	0.876997	0.954262
CC_GO:0044444_cytoplasmic+part	0.171725	0.0278185	0.716408
BP_GO:0030517_negative+regulation+of+axon+extension	0.431881	0.470988	0.436589
BP_GO:0022904_respiratory+electron+transport+chain	0.731707	0.745952	0.55321
MF_GO:0016847_1-aminocyclopropane-1-carboxylate+synthase+activity	0.870821	0.870821	#N/A
BP_GO:0009396_folic+acid+and+derivative+biosynthetic+process	0.761349	0.645033	0.81842
MF_GO:0008331_high+voltage-gated+calcium+channel+activity	0.173373	0.234204	0.260667

BP_GO:0019953_sexual+reproduction	0.0205513	0.00953304	0.410942
BP_GO:0043388_positive+regulation+of+DNA+binding	0.884977	0.480183	0.933817
BP_GO:0006573_valine+metabolic+process	0.86613	0.86613	#N/A
MF_GO:0004540_ribonuclease+activity	0.0749728	0.172896	0.123631
BP_GO:0006072_glycerol-3-phosphate+metabolic+process	0.164993	0.409421	0.0859588
BP_GO:0050930_induction+of+positive+chemotaxis	0.174115	0.10154	0.477825
BP_GO:0018149_peptide+cross-linking	0.581823	0.460868	0.606258
MF_GO:0003810_protein-glutamine+gamma-glutamyltransferase+activity	0.581823	0.460868	0.606258
BP_GO:0042743_hydrogen+peroxide+metabolic+process	0.944071	0.648929	0.986595
CC_GO:0005587_collagen+type+IV	0.0373612	0.0176069	0.455791
BP_GO:0008354_germ+cell+migration	0.214659	0.25975	0.317267
BP_GO:0048745_smooth+muscle+development	0.554161	0.489304	0.573375
BP_GO:0001578_microtubule+bundle+formation	0.984142	0.920972	0.948133
BP_GO:0035121_tail+morphogenesis	0.641837	0.197043	0.781737
CC_GO:0033179_proton-transporting+V-type+ATPase,+V0+domain	0.15392	0.15392	#N/A
BP_GO:0045732_positive+regulation+of+protein+catabolic+process	0.612659	0.521287	0.637094
BP_GO:0051592_response+to+calcium+ion	0.627574	0.757193	0.462219
MF_GO:0016175_superoxide-generating+NADPH+oxidase+activity	0.000821974	0.0868007	0.000204896
BP_GO:0051924_regulation+of+calcium+ion+transport	0.504145	0.542274	0.463165
MF_GO:0000062_acyl-CoA+binding	0.416405	0.562574	0.32375
BP_GO:0006461_protein+complex+assembly	0.988582	0.641239	0.99787
MF_GO:0008239_dipeptidyl-peptidase+activity	0.142759	0.43567	0.0522159
MF_GO:0004128_cytochrome-b5+reductase+activity	0.316376	0.384512	0.350643
BP_GO:0043526_neuroprotection	0.948208	0.952777	0.735666
BP_GO:0045648_positive+regulation+of+erythrocyte+differentiation	0.564189	0.802155	0.419641
MF_GO:0004703_G-protein+coupled+receptor+kinase+activity	0.0162996	0.0483186	0.074217
MF_GO:0016407_acetyltransferase+activity	0.241074	#N/A	0.241074
BP_GO:0045995_regulation+of+embryonic+development	0.0232508	0.0504945	0.112532
MF_GO:0008046_axon+guidance+receptor+activity	0.749417	0.436146	0.825598
BP_GO:0043154_negative+regulation+of+caspase+activity	0.925147	0.925147	#N/A
BP_GO:0042415_norepinephrine+metabolic+process	0.511198	0.352867	0.719665
BP_GO:0007163_establishment+or+maintenance+of+cell+polarity	0.756222	0.925802	0.199694
BP_GO:0016925_protein+sumoylation	0.89213	0.948478	0.435717
MF_GO:0019789_SUMO+ligase+activity	0.89213	0.948478	0.435717
BP_GO:0000038_very-long-chain+fatty+acid+metabolic+process	0.441154	0.406794	0.564924
MF_GO:0017022_myosin+binding	0.49164	0.629566	0.358913
MF_GO:0001948_glycoprotein+binding	0.324644	0.629561	0.164722
MF_GO:0004659_prenyltransferase+activity	0.451077	0.274042	0.851317
BP_GO:0007406_negative+regulation+of+neuroblast+proliferation	0.903634	0.924633	0.718926
BP_GO:0042446_hormone+biosynthetic+process	0.336759	0.296112	0.444903
BP_GO:0002262_myeloid+cell+homeostasis	0.506269	0.563045	0.375172
BP_GO:0001776_leukocyte+homeostasis	0.765292	0.596855	0.817567
BP_GO:0030203_glycosaminoglycan+metabolic+process	0.0435192	0.146121	0.0697391
BP_GO:0045039_protein+import+into+mitochondrial+inner+membrane	0.73592	0.673314	0.676183
CC_GO:0042719_mitochondrial+intermembrane+space+protein+transporter	0.73592	0.673314	0.676183
BP_GO:0050829_defense+response+to+Gram-negative+bacterium	0.293843	0.263673	0.377365
MF_GO:0016308_1-phosphatidylinositol-4-phosphate+5-kinase+activity	0.168403	0.154182	0.410999

BP_GO:0006221_pyrimidine+nucleotide+biosynthetic+process	0.383146	0.383146	#N/A
BP_GO:0007568_aging	0.816024	0.620415	0.869917
BP_GO:0060351_cartilage+development+involved+in+endochondral+bone+	0.336579	0.29193	0.516996
BP_GO:0007398_ectoderm+development	0.691113	0.808188	0.356106
MF_GO:0005112_Notch+binding	0.0614612	0.104844	0.176288
BP_GO:0016188_synaptic+vesicle+maturation	0.824452	0.839679	0.583226
BP_GO:0045778_positive+regulation+of+ossification	0.914276	0.808588	0.854981
BP_GO:0006612_protein+targeting+to+membrane	0.926857	0.467024	0.983727
MF_GO:0030374_ligand-dependent+nuclear+receptor+transcription+coactiv	0.382941	0.719906	0.157462
BP_GO:0048568_embryonic+organ+development	0.926602	0.536025	0.939276
BP_GO:0045598_regulation+of+fat+cell+differentiation	0.96887	0.855417	0.942658
BP_GO:0008343_adult+feeding+behavior	0.200925	0.062588	0.522178
BP_GO:0045670_regulation+of+osteoclast+differentiation	0.9315	0.971651	0.452404
BP_GO:0043583_ear+development	0.54879	0.454951	0.709835
BP_GO:0045840_positive+regulation+of+mitosis	0.0400845	0.509123	0.0269368
MF_GO:0004977_melanocortin+receptor+activity	0.0519045	#N/A	0.0519045
BP_GO:0043278_response+to+morphine	0.741273	0.922373	0.306345
BP_GO:0006561_proline+biosynthetic+process	0.492109	0.336362	0.713044
BP_GO:0021846_cell+proliferation+in+forebrain	0.27015	0.823593	0.079614
BP_GO:0019933_cAMP-mediated+signaling	0.837238	0.694125	0.800969
CC_GO:0000123_histone+acetyltransferase+complex	0.817722	0.681938	0.781055
BP_GO:0018279_protein+amino+acid+N-linked+glycosylation+via+asparagin	0.66142	0.508344	0.714731
BP_GO:0006406_mRNA+export+from+nucleus	0.619309	0.669905	0.495643
BP_GO:0060070_Wnt+receptor+signaling+pathway+through+beta-catenin	0.177548	0.174279	0.432072
CC_GO:0035097_histone+methyltransferase+complex	0.386085	0.599261	0.296931
CC_GO:0043195_terminal+button	0.613334	0.825247	0.298414
BP_GO:0015904_tetracycline+transport	0.783741	0.789036	0.588324
MF_GO:0015520_tetracycline:hydrogen+antiporter+activity	0.783741	0.789036	0.588324
MF_GO:0031202_RNA+splicing+factor+activity,+transesterification+mechan	0.865418	0.8592	0.686507
CC_GO:0033180_proton-transporting+V-type+ATPase,+V1+domain	0.237059	0.452704	0.141392
BP_GO:0015807_L-amino+acid+transport	0.0170002	0.458181	0.0018887
MF_GO:0015179_L-amino+acid+transmembrane+transporter+activity	0.0170002	0.458181	0.0018887
BP_GO:0007346_regulation+of+mitotic+cell+cycle	0.247439	0.175403	0.486475
MF_GO:0008408_3'-5'+exonuclease+activity	0.0123361	0.0401436	0.0776375
BP_GO:0045429_positive+regulation+of+nitric+oxide+biosynthetic+process	0.0363059	0.0225512	0.29534
MF_GO:0004908_interleukin-1+receptor+activity	0.127836	0.241361	0.18473
BP_GO:0048667_cell+morphogenesis+involved+in+neuron+differentiation	0.0950298	0.252115	0.0920715
BP_GO:0035058_sensory+cilium+assembly	0.822761	0.652913	0.820201
BP_GO:0007617_mating+behavior	0.649345	0.341739	0.775542
CC_GO:0042101_T+cell+receptor+complex	0.0310885	0.0310885	#N/A
BP_GO:0060017_parathyroid+gland+development	0.813548	0.554149	0.869451
BP_GO:0040016_embryonic+cleavage	0.847898	0.315681	0.909775
BP_GO:0030111_regulation+of+Wnt+receptor+signaling+pathway	0.391056	0.400803	0.435605
BP_GO:0000278_mitotic+cell+cycle	0.855421	#N/A	0.855421
BP_GO:0033365_protein+localization+in+organelle	0.613071	0.269932	0.783598
CC_GO:0016514_SWI/SNF+complex	0.918797	0.465151	0.961856
BP_GO:0032088_negative+regulation+of+NF-kappaB+transcription+factor+a	0.327988	0.662754	0.146536

BP_GO:0048599_oocyte+development	0.587883	0.525033	0.58976
BP_GO:0006998_nuclear+envelope+organization	0.36733	0.56307	0.117318
BP_GO:0048596_embryonic+camera-type+eye+morphogenesis	0.670342	0.497736	0.779718
BP_GO:0006020_inositol+metabolic+process	0.247926	0.339461	0.275522
BP_GO:0001763_morphogenesis+of+a+branching+structure	0.395903	0.191102	0.615336
BP_GO:0019227_action+potential+propagation	0.21273	0.231312	0.323386
BP_GO:0009266_response+to+temperature+stimulus	0.822494	0.812495	0.663028
BP_GO:0035019_somatic+stem+cell+maintenance	0.341998	0.167392	0.493677
CC_GO:0001939_female+pronucleus	0.594148	0.659315	0.530714
BP_GO:0009103_lipopolysaccharide+biosynthetic+process	0.811779	0.846085	0.641403
BP_GO:0040008_regulation+of+growth	0.722101	0.656662	0.666138
BP_GO:0006378_mRNA+polyadenylation	0.900169	0.868554	0.738313
MF_GO:0004415_hyalurononglucosaminidase+activity	0.772823	0.836406	0.531074
BP_GO:0001783_B+cell+apoptosis	0.633934	0.529706	0.658676
CC_GO:0014704_intercalated+disc	0.250322	0.37334	0.264005
BP_GO:0044255_cellular+lipid+metabolic+process	0.412515	0.618039	0.111918
CC_GO:0009898_internal+side+of+plasma+membrane	0.188283	0.272608	0.248813
BP_GO:0042490_mechanoreceptor+differentiation	0.487346	0.0306453	0.966037
BP_GO:0021798_forebrain+dorsal/ventral+pattern+formation	0.301962	0.693147	0.160322
BP_GO:0006583_melanin+biosynthetic+process+from+tyrosine	0.27909	0.683885	0.196039
MF_GO:0043236_laminin+binding	0.183393	0.114893	0.55309
BP_GO:0048305_immunoglobulin+secretion	0.947673	0.416834	0.969331
MF_GO:0016799_hydrolase+activity,+hydrolyzing+N-glycosyl+compounds	0.594948	0.445374	0.65197
MF_GO:0031683_G-protein+beta/gamma-subunit+binding	0.852481	0.789789	0.749919
BP_GO:0016447_somatic+recombination+of+immunoglobulin+gene+segme	0.620873	0.381766	0.722021
CC_GO:0005833_hemoglobin+complex	0.987215	0.977344	0.876323
MF_GO:0051219_phosphoprotein+binding	0.765177	0.9664	0.209511
CC_GO:0000502_proteasome+complex	0.885462	0.971664	0.269997
MF_GO:0051378_serotonin+binding	0.465757	0.758371	0.273744
MF_GO:0031490_chromatin+DNA+binding	0.0821765	0.210299	0.1016
MF_GO:0051087_chaperone+binding	0.88542	0.887553	0.686912
CC_GO:0005858_axonemal+dynein+complex	0.88718	0.686521	0.890212
BP_GO:0046888_negative+regulation+of+hormone+secretion	0.701535	0.692352	0.596703
MF_GO:0008641_small+protein+activating+enzyme+activity	0.433635	0.343922	0.609353
BP_GO:0021984_adenohypophysis+development	0.468103	#N/A	0.468103
BP_GO:0050966_detection+of+mechanical+stimulus+involved+in+sensory+p	0.373296	0.399572	0.419242
MF_GO:0042043_neurexin+binding	0.141003	0.363854	0.141657
CC_GO:0016471_vacuolar+proton-transporting+V-type+ATPase+complex	0.572061	0.667648	0.382233
CC_GO:0010369_chromocenter	0.0463501	0.27459	0.0192884
BP_GO:0007128_meiotic+prophase+I	0.041766	0.0537984	0.23435
MF_GO:0004972_N-methyl-D-aspartate+selective+glutamate+receptor+acti	0.202994	0.150584	0.386549
BP_GO:0006614_SRP-dependent+cotranslational+protein+targeting+to+me	0.689608	0.454502	0.79201
BP_GO:0007341_penetration+of+zona+pellucida	0.010305	0.565641	0.00156812
BP_GO:0021799_cerebral+cortex+radially+oriented+cell+migration	0.493127	0.323477	0.667352
CC_GO:0005732_small+nucleolar+ribonucleoprotein+complex	0.0900357	0.728051	0.00612298
BP_GO:0032438_melanosome+organization	0.478786	0.625821	0.292054
BP_GO:0042596_fear+response	0.894222	0.788927	0.832126

CC_GO:0001518_voltage-gated+sodium+channel+complex	0.0222934	0.0741216	0.0811182
BP_GO:0006325_establishment+or+maintenance+of+chromatin+architectur	0.529472	0.0518084	0.992456
BP_GO:0043039_tRNA+aminoacylation	0.814715	0.824485	0.542658
MF_GO:0016876_ligase+activity,+forming+aminoacyl-tRNA+and+related+co	0.814715	0.824485	0.542658
BP_GO:0045104_intermediate+filament+cytoskeleton+organization	0.0919718	0.167194	0.179833
BP_GO:0021675_nerve+development	0.357989	0.0385309	0.894735
BP_GO:0060013_righting+reflex	0.425365	0.573386	0.325521
CC_GO:0022627_cytosolic+small+ribosomal+subunit	0.979039	0.979039	#N/A
CC_GO:0005883_neurofilament	0.341873	0.444698	0.304794
MF_GO:0000175_3'-5'-exoribonuclease+activity	0.544933	0.388303	0.724434
CC_GO:0005579_membrane+attack+complex	0.500828	0.155547	0.893109
BP_GO:0019433_triacylglycerol+catabolic+process	0.817141	0.607183	0.930518
BP_GO:0006572_tyrosine+catabolic+process	0.340253	0.347138	0.432051
CC_GO:0031430_M+band	0.629915	0.724533	0.476355
BP_GO:0045577_regulation+of+B+cell+differentiation	0.339275	0.723521	0.0831914
BP_GO:0032024_positive+regulation+of+insulin+secretion	0.566408	0.566408	#N/A
BP_GO:0030212_hyaluronan+metabolic+process	0.937812	0.349976	0.989231
MF_GO:0004013_adenosylhomocysteinase+activity	0.735837	0.815835	0.458013
MF_GO:0004033_aldo-keto+reductase+activity	0.617628	0.0931847	0.981757
BP_GO:0031103_axon+regeneration	0.817454	0.373965	0.966069
BP_GO:0046835_carbohydrate+phosphorylation	0.991946	0.98207	0.882047
BP_GO:0019530_taurine+metabolic+process	0.11905	0.0171312	0.765982
MF_GO:0016597_amino+acid+binding	0.392573	0.704253	0.214459
MF_GO:0004032_aldehyde+reductase+activity	0.0651928	0.0321355	0.624281
BP_GO:0030239_myofibril+assembly	0.0259085	0.112595	0.0269634
CC_GO:0043034_costamere	0.345482	0.628385	0.217182
CC_GO:0030055_cell-substrate+junction	0.406267	0.726023	0.0413128
BP_GO:0018106_peptidyl-histidine+phosphorylation	0.383902	0.383902	#N/A
MF_GO:0004673_protein+histidine+kinase+activity	0.383902	0.383902	#N/A
MF_GO:0004768_stearoyl-CoA+9-desaturase+activity	0.511707	0.41252	0.693968
MF_GO:0050291_sphingosine+N-acyltransferase+activity	0.324265	0.836572	0.0822846
BP_GO:0002028_regulation+of+sodium+ion+transport	0.524846	0.0567768	0.914834
MF_GO:0019864_IgG+binding	0.692796	0.887336	0.367002
BP_GO:0015937_coenzyme+A+biosynthetic+process	0.0799971	0.104961	0.262022
CC_GO:0005916_fascia+adherens	0.354397	0.537235	0.239891
BP_GO:0050729_positive+regulation+of+inflammatory+response	0.400668	0.0802985	0.906543
BP_GO:0006107_oxaloacetate+metabolic+process	0.0247732	0.0247732	#N/A
BP_GO:0042168_heme+metabolic+process	0.0883682	0.0592295	0.410611
BP_GO:0043086_negative+regulation+of+catalytic+activity	0.115957	0.161342	0.230324
BP_GO:0051272_positive+regulation+of+cell+motion	0.242972	0.145794	0.484014
BP_GO:0007029_endoplasmic+reticulum+organization	0.507623	0.506157	0.504079
BP_GO:0009168_purine+ribonucleoside+monophosphate+biosynthetic+pro	0.599038	0.536192	0.598306
MF_GO:0019239_deaminase+activity	0.599038	0.536192	0.598306
BP_GO:0008631_induction+of+apoptosis+by+oxidative+stress	0.834869	0.610796	0.946896
BP_GO:0043552_positive+regulation+of+phosphoinositide+3-kinase+activity	0.884089	0.998631	0.436658
BP_GO:0042759_long-chain+fatty+acid+biosynthetic+process	0.146336	0.218027	0.238215
BP_GO:0006004_fucose+metabolic+process	0.49341	0.609017	0.276743

BP_GO:0019395_fatty+acid+oxidation	0.418441	0.331168	0.582434
MF_GO:0016776_phosphotransferase+activity,+phosphate+group+as+accep	0.583353	0.580576	0.52503
MF_GO:0019201_nucleotide+kinase+activity	0.583353	0.580576	0.52503
MF_GO:0001968_fibronectin+binding	0.691608	0.275659	0.871122
BP_GO:0045588_positive+regulation+of+gamma-delta+T+cell+differentiatio	0.207013	0.0336078	0.828608
BP_GO:0030177_positive+regulation+of+Wnt+receptor+signaling+pathway	0.576528	0.17699	0.842533
BP_GO:0051091_positive+regulation+of+transcription+factor+activity	0.184792	0.0543863	0.559276
BP_GO:0016202_regulation+of+striated+muscle+development	0.15826	0.291491	0.198632
MF_GO:0000146_microfilament+motor+activity	0.160696	0.305836	0.114052
MF_GO:0005522_profilin+binding	0.225853	0.369332	0.241763
MF_GO:0004559_alpha-mannosidase+activity	0.864722	0.730231	0.839107
BP_GO:0008088_axon+cargo+transport	0.419055	0.551043	0.23706
MF_GO:0047760_butyrate-CoA+ligase+activity	0.261674	0.335138	0.312283
MF_GO:0004499_flavin-containing+monooxygenase+activity	0.727602	0.888252	0.527384
BP_GO:0006012_galactose+metabolic+process	0.178956	0.350204	0.098591
BP_GO:0030278_regulation+of+ossification	0.312306	0.227538	0.490726
BP_GO:0043408_regulation+of+MAPKKK+cascade	0.237031	0.876083	0.0308282
CC_GO:0000145_exocyst	0.360182	0.448145	0.360593
MF_GO:0003857_3-hydroxyacyl-CoA+dehydrogenase+activity	0.0360704	0.0360704	#N/A
BP_GO:0048011_nerve+growth+factor+receptor+signaling+pathway	0.734409	0.990169	0.136536
MF_GO:0004679_AMP-activated+protein+kinase+activity	0.937266	0.510308	0.974829
MF_GO:0003847_1-alkyl-2-acetylglycerophosphocholine+esterase+activity	0.823682	0.907436	0.646937
BP_GO:0000904_cell+morphogenesis+involved+in+differentiation	0.554734	0.659426	0.387156
MF_GO:0016504_protease+activator+activity	0.16508	0.0144894	0.871489
BP_GO:0050873_brown+fat+cell+differentiation	0.409427	0.16313	0.926711
BP_GO:0006884_cell+volume+homeostasis	0.791546	0.802779	0.637797
BP_GO:0000070_mitotic+sister+chromatid+segregation	0.344556	0.621374	0.220679
MF_GO:0004565_beta-galactosidase+activity	0.0509431	0.704667	0.00534456
MF_GO:0004720_protein-lysine+6-oxidase+activity	0.497099	0.196959	0.848946
BP_GO:0032060_bleb+formation	0.991191	0.991875	0.865053
BP_GO:0045931_positive+regulation+of+mitotic+cell+cycle	0.0977489	0.0198947	0.888192
BP_GO:0030595_leukocyte+chemotaxis	0.514516	0.731061	0.323949
MF_GO:0008131_amine+oxidase+activity	0.787144	0.798922	0.592113
BP_GO:0007088_regulation+of+mitosis	0.293571	0.135537	0.687256
BP_GO:0008211_glucocorticoid+metabolic+process	0.430472	0.721184	0.159446
BP_GO:0051098_regulation+of+binding	0.0709872	0.291491	0.0735992
CC_GO:0000276_mitochondrial+proton-transporting+ATP+synthase+comple	0.93957	0.93957	#N/A
BP_GO:0008635_caspase+activation+via+cytochrome+c	0.680349	0.680349	#N/A
BP_GO:0045351_type+I+interferon+biosynthetic+process	0.878623	0.710382	0.848199
BP_GO:0045727_positive+regulation+of+translation	0.570219	0.667722	0.449749
BP_GO:0055074_calcium+ion+homeostasis	0.153926	0.164621	0.300936
BP_GO:0050771_negative+regulation+of+axonogenesis	0.867787	0.876083	0.690521
MF_GO:0005066_transmembrane+receptor+protein+tyrosine+kinase+signal	0.246084	0.3228	0.268106
CC_GO:0005795_Golgi+stack	0.335192	0.0882137	0.578642
MF_GO:0008093_cytoskeletal+adaptor+activity	0.457258	0.559592	0.422475
CC_GO:0005744_mitochondrial+inner+membrane+presequence+translocase	0.929549	0.977383	0.237604
BP_GO:0045792_negative+regulation+of+cell+size	0.753498	0.753498	#N/A

BP_GO:0048008_platelet-derived+growth+factor+receptor+signaling+pathw	0.387134	0.460623	0.327945
MF_GO:0008266_poly(U)+binding	0.867169	0.844804	0.728694
MF_GO:0019899_enzyme+binding	0.155115	0.112061	0.374722
BP_GO:0051289_protein+homotetramerization	0.645246	0.265516	0.912437
BP_GO:0045080_positive+regulation+of+chemokine+biosynthetic+process	0.55491	0.935448	0.272895
CC_GO:0005606_laminin-1+complex	0.0272576	0.0611757	0.112532
BP_GO:0046579_positive+regulation+of+Ras+protein+signal+transduction	0.183085	0.351178	0.103877
BP_GO:0001832_blastocyst+growth	0.945166	0.950382	0.764159
BP_GO:0006308_DNA+catabolic+process	0.287454	0.323552	0.362551
MF_GO:0030276_clathrin+binding	0.993316	0.869675	0.988584
MF_GO:0008499_UDP-galactose:beta-N-acetylglucosamine+beta-1,3-galacto	0.181187	0.133695	0.32082
MF_GO:0030295_protein+kinase+activator+activity	0.548912	0.340485	0.757143
MF_GO:0004563_beta-N-acetylhexosaminidase+activity	0.579989	0.452173	0.755349
BP_GO:0014047_glutamate+secretion	0.569401	0.349288	0.773387
BP_GO:0045773_positive+regulation+of+axon+extension	0.217095	0.29261	0.284339
BP_GO:0048384_retinoic+acid+receptor+signaling+pathway	0.527121	0.602448	0.450269
BP_GO:0030511_positive+regulation+of+transforming+growth+factor+beta+	0.871142	0.852849	0.770753
MF_GO:0005498_sterol+carrier+activity	0.338572	0.151286	0.870935
BP_GO:0042756_drinking+behavior	0.386898	0.773994	0.242713
MF_GO:0042153_RPTP-like+protein+binding	0.0532442	0.428526	0.0262638
BP_GO:0022409_positive+regulation+of+cell-cell+adhesion	0.408402	0.797165	0.082795
BP_GO:0030433_ER-associated+protein+catabolic+process	0.672749	0.672749	#N/A
MF_GO:0004445_inositol-polyphosphate+5-phosphatase+activity	0.193551	0.33631	0.197087
BP_GO:0010552_positive+regulation+of+specific+transcription+from+RNA+p	0.813814	0.792087	0.643147
BP_GO:0007600_sensory+perception	0.286219	0.775496	0.0342316
MF_GO:0003828_alpha-N-acetylneuraminate+alpha-2,8-sialyltransferase+ac	0.320402	0.066472	0.864668
BP_GO:0046548_retinal+rod+cell+development	0.591426	0.0636521	0.845975
MF_GO:0004861_cyclin-dependent+protein+kinase+inhibitor+activity	0.754809	0.679323	0.698342
MF_GO:0035257_nuclear+hormone+receptor+binding	0.790897	0.621517	0.785857
BP_GO:0019430_removal+of+superoxide+radicals	0.547688	0.371057	0.722968
BP_GO:0002089_lens+morphogenesis+in+camera-type+eye	0.723237	0.880175	0.422556
BP_GO:0051384_response+to+glucocorticoid+stimulus	0.539498	0.825827	0.26166
MF_GO:0008467_[heparan+sulfate]-glucosamine+3-sulfotransferase+1+activ	0.12204	0.134745	0.311515
MF_GO:0001515_opioid+peptide+activity	0.155089	0.423046	0.0296943
BP_GO:0042088_T-helper+1+type+immune+response	0.425427	0.203491	0.763408
CC_GO:0001931_uropod	0.130648	0.242347	0.131869
BP_GO:0002467_germinal+center+formation	0.420412	0.0993209	0.784897
CC_GO:0005851_eukaryotic+translation+initiation+factor+2B+complex	0.403045	0.403045	#N/A
BP_GO:0055015_ventricular+cardiac+muscle+cell+development	0.609523	0.302598	0.858211
BP_GO:0015701_bicarbonate+transport	0.724272	0.66605	0.674051
BP_GO:0048644_muscle+morphogenesis	0.340048	0.340048	#N/A
BP_GO:0047496_vesicle+transport+along+microtubule	0.484517	0.815312	0.122549
BP_GO:0007127_meiosis+I	0.424985	0.716047	0.238426
BP_GO:0040020_regulation+of+meiosis	0.266553	0.266553	#N/A
MF_GO:0005173_stem+cell+factor+receptor+binding	0.266942	0.198156	0.39252
BP_GO:0009306_protein+secretion	0.448117	0.249596	0.732522
BP_GO:0060271_cilium+morphogenesis	0.891565	0.784074	0.882993

BP_GO:0048339_paraxial+mesoderm+development	0.445929	0.514234	0.397441
BP_GO:0021631_optic+nerve+morphogenesis	0.747567	0.301659	0.900523
CC_GO:0030659_cytoplasmic+vesicle+membrane	0.692683	0.62102	0.654639
MF_GO:0008517_folic+acid+transporter+activity	0.951221	0.770153	0.937683
BP_GO:0001779_natural+killer+cell+differentiation	0.125215	0.125215	#N/A
BP_GO:0009101_glycoprotein+biosynthetic+process	0.00433292	0.613889	0.00103286
BP_GO:0048562_embryonic+organ+morphogenesis	0.472541	0.181559	0.646705
CC_GO:0044431_Golgi+apparatus+part	0.880851	0.602593	0.939
BP_GO:0045787_positive+regulation+of+cell+cycle	0.837173	0.905866	0.577212
MF_GO:0004726_non-membrane+spanning+protein+tyrosine+phosphatase+	0.991414	0.997222	0.64529
BP_GO:0000245_spliceosome+assembly	0.182336	0.116724	0.337469
CC_GO:0030008_TRAPP+complex	0.956021	0.956021	#N/A
BP_GO:0033014_tetrapyrrole+biosynthetic+process	0.793793	0.478961	0.864517
BP_GO:0048812_neurite+morphogenesis	0.499177	0.281142	0.87585
BP_GO:0043666_regulation+of+phosphoprotein+phosphatase+activity	0.934524	0.893523	0.812425
BP_GO:0006261_DNA-dependent+DNA+replication	0.0737304	0.0957169	0.264612
BP_GO:0009650_UV+protection	0.401208	0.0649188	0.819173
BP_GO:0009620_response+to+fungus	0.141773	0.113948	0.412622
MF_GO:0008190_eukaryotic+initiation+factor+4E+binding	0.145223	0.165796	0.335646
BP_GO:0006590_thyroid+hormone+generation	0.264967	0.0647523	0.805768
CC_GO:0005662_DNA+replication+factor+A+complex	0.651708	0.753785	0.307567
CC_GO:0005869_dynactin+complex	0.592656	0.541652	0.623282
BP_GO:0048762_mesenchymal+cell+differentiation	0.412418	0.360971	0.501467
BP_GO:0006346_methylation-dependent+chromatin+silencing	0.870081	0.553716	0.947012
BP_GO:0048699_generation+of+neurons	0.715069	0.244108	0.902994
CC_GO:0019031_viral+envelope	0.0273101	0.0191519	0.307004
BP_GO:0009083_branched+chain+family+amino+acid+catabolic+process	0.804264	0.93326	0.314394
MF_GO:0003704_specific+RNA+polymerase+II+transcription+factor+activity	0.268957	0.268957	#N/A
CC_GO:0019005_SCF+ubiquitin+ligase+complex	0.83491	0.928546	0.524345
BP_GO:0008366_axon+ensheathment	0.392673	0.340854	0.528057
BP_GO:0050727_regulation+of+inflammatory+response	0.0911682	0.254056	0.0966795
BP_GO:0051225_spindle+assembly	0.302927	0.194556	0.59387
BP_GO:0000154_rRNA+modification	0.577272	0.669655	0.408865
BP_GO:0050892_intestinal+absorption	0.494648	0.10289	0.7311
BP_GO:0048489_synaptic+vesicle+transport	0.479765	0.790651	0.141811
MF_GO:0017040_ceramidase+activity	0.637096	0.677219	0.530563
BP_GO:0007379_segment+specification	0.528215	0.81104	0.26463
MF_GO:0004620_phospholipase+activity	0.432126	0.676043	0.276107
BP_GO:0016574_histone+ubiquitination	0.508157	0.313409	0.663614
BP_GO:0050908_detection+of+light+stimulus+involved+in+visual+perceptio	0.768992	0.416628	0.868846
BP_GO:0048515_spermatid+differentiation	0.246826	0.621722	0.0715738
BP_GO:0007030_Golgi+organization	0.56718	0.869955	0.241486
MF_GO:0042169_SH2+domain+binding	0.689601	0.0881766	0.890225
MF_GO:0017016_Ras+GTPase+binding	0.0580947	0.113297	0.157286
BP_GO:0045198_establishment+of+epithelial+cell+apical/basal+polarity	0.358797	0.11265	0.947061
BP_GO:0006415_translational+termination	0.967041	0.978471	0.765189
MF_GO:0003747_translation+release+factor+activity	0.967041	0.978471	0.765189

MF_GO:0002039_p53+binding	0.903452	0.937916	0.664659
BP_GO:0009792_embryonic+development+ending+in+birth+or+egg+hatchir	0.131344	#N/A	0.131344
BP_GO:0001957_intramembranous+ossification	0.318977	0.172225	0.564717
BP_GO:0002070_epithelial+cell+maturation	0.494153	0.280709	0.75403
MF_GO:0004960_thromboxane+receptor+activity	0.233854	0.556592	0.145699
CC_GO:0005637_nuclear+inner+membrane	0.514048	0.702775	0.275031
MF_GO:0031419_cobalamin+binding	0.382247	0.344857	0.445787
BP_GO:0021513_spinal+cord+dorsal/ventral+patterning	0.713502	0.967291	0.385778
MF_GO:0005487_nucleocytoplasmic+transporter+activity	0.878526	0.469782	0.941692
MF_GO:0035014_phosphoinositide+3-kinase+regulator+activity	0.335868	0.931265	0.111953
BP_GO:0045879_negative+regulation+of+smoothened+signaling+pathway	0.75395	0.678586	0.694238
MF_GO:0004708_MAP+kinase+kinase+activity	0.654512	0.705498	0.486451
BP_GO:0015012_heparan+sulfate+proteoglycan+biosynthetic+process	0.526343	0.588022	0.382535
MF_GO:0019863_IgE+binding	0.0448864	0.180534	0.0746739
BP_GO:0001101_response+to+acid	0.462049	0.632284	0.285737
BP_GO:0021516_dorsal+spinal+cord+development	0.550932	0.847647	0.145469
BP_GO:0046621_negative+regulation+of+organ+growth	0.498602	0.640796	0.426759
BP_GO:0048541_Peyer's+patch+development	0.199286	0.0276322	0.844103
MF_GO:0031405_lipoic+acid+binding	0.756343	0.859907	0.410979
MF_GO:0017091_AU-rich+element+binding	0.376296	0.571168	0.328985
BP_GO:0006265_DNA+topological+change	0.893363	#N/A	0.893363
MF_GO:0003916_DNA+topoisomerase+activity	0.893363	#N/A	0.893363
MF_GO:0050220_prostaglandin-E+synthase+activity	0.00586683	0.460854	0.00280559
BP_GO:0002328_pro-B+cell+differentiation	0.165028	0.0827161	0.345596
BP_GO:0009725_response+to+hormone+stimulus	0.0179727	0.00684717	0.594109
BP_GO:0000288_nuclear-transcribed+mRNA+catabolic+process,+deadenylat	0.385081	0.721859	0.11814
BP_GO:0043576_regulation+of+respiratory+gaseous+exchange	0.849854	0.521849	0.870824
BP_GO:0030071_regulation+of+mitotic+metaphase/anaphase+transition	0.690281	0.729122	0.556686
BP_GO:0048341_paraxial+mesoderm+formation	0.222015	0.381965	0.239901
BP_GO:0019217_regulation+of+fatty+acid+metabolic+process	0.788681	0.504965	0.961593
BP_GO:0021680_cerebellar+Purkinje+cell+layer+development	0.246672	0.659757	0.0559552
BP_GO:0018101_peptidyl-citrulline+biosynthetic+process+from+peptidyl-arg	0.15762	0.15762	#N/A
MF_GO:0004668_protein-arginine+deiminase+activity	0.15762	0.15762	#N/A
BP_GO:0009950_dorsal/ventral+axis+specification	0.456897	0.563262	0.356612
BP_GO:0046580_negative+regulation+of+Ras+protein+signal+transduction	0.897579	0.552364	0.936901
BP_GO:0030851_granulocyte+differentiation	0.179972	0.179972	#N/A
MF_GO:0042054_histone+methyltransferase+activity	0.321264	0.537597	0.249057
BP_GO:0043193_positive+regulation+of+gene-specific+transcription	0.964623	0.973251	0.775521
BP_GO:0050769_positive+regulation+of+neurogenesis	0.286775	#N/A	0.286775
MF_GO:0043021_ribonucleoprotein+binding	0.889089	0.567107	0.95775
BP_GO:0007638_mechanosensory+behavior	0.350425	#N/A	0.350425
MF_GO:0016922_ligand-dependent+nuclear+receptor+binding	0.245906	0.333642	0.286965
BP_GO:0048485_sympathetic+nervous+system+development	0.576059	0.521849	0.57397
BP_GO:0046627_negative+regulation+of+insulin+receptor+signaling+pathwa	0.811755	0.678167	0.777517
BP_GO:0016226_iron-sulfur+cluster+assembly	0.331499	0.623918	0.140599
BP_GO:0060042_retina+morphogenesis+in+camera-type+eye	0.202647	0.547897	0.16075
BP_GO:0032094_response+to+food	0.411809	0.593409	0.315018

MF_GO:0004947_bradykinin+receptor+activity	0.162968	0.224133	0.235606
BP_GO:0007004_telomere+maintenance+via+telomerase	0.715164	0.813054	0.424317
MF_GO:0016624_oxidoreductase+activity,+acting+on+the+aldehyde+or+oxo	0.667341	0.931751	0.021985
BP_GO:0045078_positive+regulation+of+interferon-gamma+biosynthetic+pr	0.283215	0.16231	0.524934
BP_GO:0042136_neurotransmitter+biosynthetic+process	0.148406	0.33678	0.157544
MF_GO:0034450_ubiquitin-ubiquitin+ligase+activity	0.625799	0.548415	0.682021
BP_GO:0060040_retinal+bipolar+neuron+differentiation	0.0992398	0.695816	0.0187627
BP_GO:0030307_positive+regulation+of+cell+growth	0.329001	0.240658	0.564009
BP_GO:0032331_negative+regulation+of+chondrocyte+differentiation	0.622906	0.676363	0.414343
MF_GO:0005000_vasopressin+receptor+activity	0.671133	0.584497	0.654374
MF_GO:0017176_phosphatidylinositol+N-acetylglucosaminyltransferase+act	0.964714	0.796368	0.951366
CC_GO:0001940_male+pronucleus	0.493528	0.659315	0.411446
BP_GO:0006777_Mo-molybdopterin+cofactor+biosynthetic+process	0.925576	0.948426	0.487148
BP_GO:0030858_positive+regulation+of+epithelial+cell+differentiation	0.266634	0.603485	0.153921
MF_GO:0004981_muscarinic+acetylcholine+receptor+activity	0.0646178	0.0246892	0.360775
BP_GO:0006516_glycoprotein+catabolic+process	0.663515	0.635444	0.598857
BP_GO:0035095_behavioral+response+to+nicotine	0.480772	0.513715	0.46365
BP_GO:0007006_mitochondrial+membrane+organization	0.429859	0.273006	0.676858
BP_GO:0046686_response+to+cadmium+ion	0.447694	0.782178	0.295643
CC_GO:0042589_zymogen+granule+membrane	0.429597	0.458402	0.442445
BP_GO:0042053_regulation+of+dopamine+metabolic+process	0.814348	0.322692	0.890446
CC_GO:0046540_U4/U6+x+U5+tri-snRNP+complex	0.886561	0.885135	0.617791
MF_GO:0004982_N-formyl+peptide+receptor+activity	0.819699	0.885723	0.578148
BP_GO:0032314_regulation+of+Rac+GTPase+activity	0.255841	0.385903	0.27034
BP_GO:0048665_neuron+fate+specification	0.682923	#N/A	0.682923
BP_GO:0042428_serotonin+metabolic+process	0.56379	0.178927	0.916139
CC_GO:0012505_endomembrane+system	0.839343	0.839343	#N/A
BP_GO:0010224_response+to+UV-B	0.416307	0.128984	0.741956
CC_GO:0000784_nuclear+chromosome,+telomeric+region	0.56883	0.611906	0.49642
BP_GO:0042423_catecholamine+biosynthetic+process	0.401598	0.204778	0.730695
BP_GO:0000239_pachytene	0.231384	0.252006	0.36525
BP_GO:0007431_salivary+gland+development	0.0849549	0.137851	0.18618
BP_GO:0014044_Schwann+cell+development	0.768332	0.732563	0.655091
BP_GO:0006685_sphingomyelin+catabolic+process	0.733959	0.478919	0.80214
BP_GO:0048715_negative+regulation+of+oligodendrocyte+differentiation	0.763835	0.753984	0.615778
BP_GO:0033151_V(D)J+recombination	0.0991111	0.156079	0.174955
CC_GO:0030137_COPI-coated+vesicle	0.148582	0.0549974	0.620668
BP_GO:0045638_negative+regulation+of+myeloid+cell+differentiation	0.279339	0.800073	0.0251123
BP_GO:0035094_response+to+nicotine	0.116126	0.513715	0.0579862
BP_GO:0048484_enteric+nervous+system+development	0.780382	0.723559	0.689996
BP_GO:0021527_spinal+cord+association+neuron+differentiation	0.779331	0.872507	0.525707
BP_GO:0050678_regulation+of+epithelial+cell+proliferation	0.016723	0.016723	#N/A
BP_GO:0048709_oligodendrocyte+differentiation	0.286528	0.438649	0.240788
CC_GO:0044464_cell+part	0.139292	0.0657758	0.432678
BP_GO:0060045_positive+regulation+of+cardiac+muscle+cell+proliferation	0.961662	0.886131	0.939149
BP_GO:0060135_maternal+process+involved+in+pregnancy	0.462961	0.370968	0.673612
BP_GO:0000012_single+strand+break+repair	0.799915	0.76919	0.658545

MF_GO:0004952_dopamine+receptor+activity	0.707443	0.805566	0.571254
MF_GO:0004826_phenylalanine-tRNA+ligase+activity	0.998561	0.980386	0.98572
BP_GO:0048703_embryonic+viscerocranium+morphogenesis	0.585941	0.754092	0.308622
BP_GO:0033138_positive+regulation+of+peptidyl-serine+phosphorylation	0.697091	0.323634	0.850619
BP_GO:0045776_negative+regulation+of+blood+pressure	0.941844	0.805566	0.907456
MF_GO:0004994_somatostatin+receptor+activity	0.0909934	0.534145	0.0132703
MF_GO:0001601_peptide+YY+receptor+activity	0.93044	0.666525	0.940414
MF_GO:0003831_beta-N-acetylglucosaminylglycopeptide+beta-1,4-galactos	0.856596	0.847109	0.705057
CC_GO:0017146_N-methyl-D-aspartate+selective+glutamate+receptor+com	0.30644	0.288738	0.386562
BP_GO:0006354_RNA+elongation	0.639968	0.863364	0.218795
MF_GO:0004652_polynucleotide+adenylyltransferase+activity	0.402571	0.20042	0.642874
BP_GO:0009253_peptidoglycan+catabolic+process	0.812145	0.821357	0.607517
BP_GO:0030819_positive+regulation+of+cAMP+biosynthetic+process	0.137585	0.163444	0.232271
BP_GO:0060038_cardiac+muscle+cell+proliferation	0.389099	0.748413	0.102554
BP_GO:0051023_regulation+of+immunoglobulin+secretion	0.975482	0.962184	0.825619
MF_GO:0030235_nitric-oxide+synthase+regulator+activity	0.416443	0.492275	0.37779
BP_GO:0045861_negative+regulation+of+proteolysis	0.0202757	0.00966139	0.230564
BP_GO:0046697_decidualization	0.333798	0.623112	0.143434
BP_GO:0042035_regulation+of+cytokine+biosynthetic+process	0.385779	0.502801	0.351366
BP_GO:0031575_G1/S+transition+checkpoint	0.616112	0.771577	0.410294
CC_GO:0001740_Barr+body	0.797037	0.797037	#N/A
CC_GO:0005865_striated+muscle+thin+filament	0.202549	0.176521	0.476752
BP_GO:0030300_regulation+of+cholesterol+absorption	0.0341441	0.123217	0.0502322
BP_GO:0006526_arginine+biosynthetic+process	0.761915	0.748668	0.603862
MF_GO:0001758_retinal+dehydrogenase+activity	0.238583	0.386966	0.235841
BP_GO:0006597_spermine+biosynthetic+process	0.214583	#N/A	0.214583
CC_GO:0002102_podosome	0.359885	0.0866407	0.803177
BP_GO:0006547_histidine+metabolic+process	0.649832	0.773909	0.417557
MF_GO:0004645_phosphorylase+activity	0.947219	0.947219	#N/A
BP_GO:0008295_spermidine+biosynthetic+process	0.237809	0.0926492	0.623276
MF_GO:0004022_alcohol+dehydrogenase+activity	0.286819	0.305573	0.360392
MF_GO:0004470_malic+enzyme+activity	0.114318	0.50134	0.0438162
BP_GO:0002639_positive+regulation+of+immunoglobulin+production	0.687364	0.687364	#N/A
BP_GO:0014823_response+to+activity	0.580872	0.51014	0.641866
CC_GO:0005826_contractile+ring	0.312547	0.31844	0.384819
BP_GO:0060047_heart+contraction	0.754557	0.402007	0.889153
MF_GO:0003846_2-acylglycerol+O-acyltransferase+activity	0.385767	0.623782	0.12931
MF_GO:0004111_creatine+kinase+activity	0.0519501	0.0399624	0.412235
BP_GO:0033089_positive+regulation+of+T+cell+differentiation+in+the+thym	0.328451	0.328451	#N/A
BP_GO:0043534_blood+vessel+endothelial+cell+migration	0.362587	0.362587	#N/A
CC_GO:0009341_beta-galactosidase+complex	0.378133	0.704667	0.164008
BP_GO:0042590_antigen+processing+and+presentation+of+exogenous+pep	0.355389	0.438537	0.367002
BP_GO:0001798_positive+regulation+of+type+IIa+hypersensitivity	0.388452	#N/A	0.388452
BP_GO:0043149_stress+fiber+formation	0.410156	0.495016	0.3783
CC_GO:0010008_endosome+membrane	0.479556	#N/A	0.479556
MF_GO:0008967_phosphoglycolate+phosphatase+activity	0.534492	0.71503	0.20812
CC_GO:0031672_A+band	0.308252	0.579682	0.243483

BP_GO:0020027_hemoglobin+metabolic+process	0.349793	0.254042	0.545677
BP_GO:0006707_cholesterol+catabolic+process	0.9556	0.319422	0.987308
MF_GO:0004028_3-chloroallyl+aldehyde+dehydrogenase+activity	0.067183	#N/A	0.067183
BP_GO:0006699_bile+acid+biosynthetic+process	0.978991	#N/A	0.978991
MF_GO:0051010_microtubule+plus-end+binding	0.127134	0.0903739	0.292694
BP_GO:0006081_cellular+aldehyde+metabolic+process	0.810231	0.894174	0.497409
BP_GO:0050832_defense+response+to+fungus	0.183679	0.0586024	0.614076
MF_GO:0016717_oxidoreductase+activity,+acting+on+paired+donors,+with-	0.102573	0.131085	0.276088
BP_GO:0046638_positive+regulation+of+alpha-beta+T+cell+differentiation	0.306204	0.348986	0.36579
MF_GO:0004740_pyruvate+dehydrogenase+(acetyl-transferring)+kinase+act	0.515053	0.515053	#N/A
BP_GO:0002526_acute+inflammatory+response	0.0935056	0.296863	0.0909715
MF_GO:0004749_ribose+phosphate+diphosphokinase+activity	0.937937	0.937937	#N/A
BP_GO:0006101_citrate+metabolic+process	0.0469869	0.0469869	#N/A
MF_GO:0004062_aryl+sulfotransferase+activity	0.902018	0.654542	0.971218
BP_GO:0021940_positive+regulation+of+granule+cell+precursor+proliferatic	0.275799	0.801074	0.119796
CC_GO:0016010_dystrophin-associated+glycoprotein+complex	0.000329186	0.00141826	0.0331386
BP_GO:0042159_lipoprotein+catabolic+process	0.823183	0.145208	0.953558
CC_GO:0030140_trans-Golgi+network+transport+vesicle	0.798309	0.798309	#N/A
BP_GO:0009404_toxin+metabolic+process	0.294457	0.846329	0.112438
BP_GO:0007167_enzyme+linked+receptor+protein+signaling+pathway	0.520298	0.114733	0.898449
BP_GO:0007009_plasma+membrane+organization	0.418961	0.496238	0.389338
BP_GO:0051346_negative+regulation+of+hydrolase+activity	0.168599	0.0372742	0.664253
MF_GO:0004030_aldehyde+dehydrogenase+[NAD(P)+]+activity	0.457841	0.894174	0.0807762
BP_GO:0022027_interkinetic+nuclear+migration	0.691763	0.780107	0.552586
BP_GO:0032886_regulation+of+microtubule-based+process	0.691763	0.780107	0.552586
BP_GO:0006361_transcription+initiation+from+RNA+polymerase+I+promote	0.932162	0.932162	#N/A
BP_GO:0048771_tissue+remodeling	0.33009	0.173927	0.772144
BP_GO:0006742_NADP+catabolic+process	0.00367841	0.0596153	0.00382196
MF_GO:0015175_neutral+amino+acid+transmembrane+transporter+activity	0.8291	0.8291	#N/A
BP_GO:0001523_retinoid+metabolic+process	0.777154	0.572931	0.814436
BP_GO:0051138_positive+regulation+of+NK+T+cell+differentiation	0.417704	0.151553	0.768759
BP_GO:0006497_protein+amino+acid+lipidation	0.243973	0.4893	0.089519
BP_GO:0045123_cellular+extravasation	0.26975	0.462333	0.219249
BP_GO:0006596_polyamine+biosynthetic+process	0.345012	0.164966	0.658678
BP_GO:0033344_cholesterol+efflux	0.676037	0.245807	0.908727
BP_GO:0050715_positive+regulation+of+cytokine+secretion	0.393316	0.642612	0.226774
BP_GO:0045647_negative+regulation+of+erythrocyte+differentiation	0.195156	0.195156	#N/A
BP_GO:0032963_collagen+metabolic+process	0.458094	0.660819	0.286163
BP_GO:0002318_myeloid+progenitor+cell+differentiation	0.930553	0.968892	0.590383
MF_GO:0003997_acyl-CoA+oxidase+activity	0.291608	0.950387	0.00763096
BP_GO:0007176_regulation+of+epidermal+growth+factor+receptor+activity	0.987959	0.74992	0.994083
BP_GO:0006105_succinate+metabolic+process	0.0649983	0.0649983	#N/A
MF_GO:0004957_prostaglandin+E+receptor+activity	0.545411	0.788149	0.261221
BP_GO:0070059_apoptosis+in+response+to+endoplasmic+reticulum+stress	0.279667	0.248537	0.441129
BP_GO:0050718_positive+regulation+of+interleukin-1+beta+secretion	0.717388	0.642612	0.672704
BP_GO:0042541_hemoglobin+biosynthetic+process	0.294129	0.197513	0.533246
CC_GO:0005664_nuclear+origin+of+replication+recognition+complex	0.202047	0.264052	0.274082

BP_GO:0002024_diet+induced+thermogenesis	0.39688	0.199282	0.681973
BP_GO:0016556_mRNA+modification	0.752286	0.90389	0.184839
BP_GO:0000103_sulfate+assimilation	0.950456	#N/A	0.950456
BP_GO:0007288_sperm+axoneme+assembly	0.419607	0.419607	#N/A
BP_GO:0045636_positive+regulation+of+melanocyte+differentiation	0.319101	0.0537204	0.827257
BP_GO:0001778_plasma+membrane+repair	0.0423573	0.0318482	0.27882
BP_GO:0042307_positive+regulation+of+protein+import+into+nucleus	0.0701044	0.32049	0.0523991
BP_GO:0035235_ionotropic+glutamate+receptor+signaling+pathway	0.136238	0.138049	0.378089
BP_GO:0002925_positive+regulation+of+humoral+immune+response+medi	0.0455321	0.0305402	0.301738
BP_GO:0014037_Schwann+cell+differentiation	0.140925	0.146368	0.369881
BP_GO:0046326_positive+regulation+of+glucose+import	0.649512	0.612813	0.6067
BP_GO:0046836_glycolipid+transport	0.5646	0.67313	0.471465
MF_GO:0017089_glycolipid+transporter+activity	0.5646	0.67313	0.471465
MF_GO:0051861_glycolipid+binding	0.5646	0.67313	0.471465
MF_GO:0015923_mannosidase+activity	0.863495	0.71206	0.839107
MF_GO:0004614_phosphoglucomutase+activity	0.319783	0.319783	#N/A
MF_GO:0004551_nucleotide+diphosphatase+activity	0.398464	#N/A	0.398464
MF_GO:0001530_lipopolysaccharide+binding	0.152515	0.210118	0.258975
CC_GO:0001527_microfibril	0.525592	0.0974345	0.794197
MF_GO:0015101_organic+cation+transmembrane+transporter+activity	0.0262486	0.159385	0.0479244
BP_GO:0045651_positive+regulation+of+macrophage+differentiation	0.166671	0.176144	0.372272
MF_GO:0004046_aminoacylase+activity	0.620867	0.62745	0.520489
MF_GO:0030976_thiamin+pyrophosphate+binding	0.906853	0.939965	0.623539
BP_GO:0042116_macrophage+activation	0.858478	0.887668	0.517266
BP_GO:0000281_cytokinesis+after+mitosis	0.0492339	0.254993	0.0464381
BP_GO:0015816_glycine+transport	0.00712903	0.0704391	0.0229371
MF_GO:0015187_glycine+transmembrane+transporter+activity	0.00712903	0.0704391	0.0229371
BP_GO:0007091_mitotic+metaphase/anaphase+transition	0.921439	#N/A	0.921439
BP_GO:0032020_ISG15-protein+conjugation	0.0837073	0.0837073	#N/A
BP_GO:0043277_apoptotic+cell+clearance	0.657427	0.714164	0.432528
CC_GO:0043227_membrane-bounded+organelle	0.992588	0.721134	0.997888
BP_GO:0001667_ameboidal+cell+migration	0.42318	0.0743125	0.728585
BP_GO:0042535_positive+regulation+of+tumor+necrosis+factor+biosyntheti	0.195286	0.933079	0.0316542
BP_GO:0021955_central+nervous+system+neuron+axonogenesis	0.00260717	0.00357307	0.175781
MF_GO:0043023_ribosomal+large+subunit+binding	0.991772	0.994837	0.638877
MF_GO:0004718_Janus+kinase+activity	0.744499	0.365563	0.898449
BP_GO:0009166_nucleotide+catabolic+process	0.580409	#N/A	0.580409
BP_GO:0030501_positive+regulation+of+bone+mineralization	0.996644	0.977929	0.975546
BP_GO:0001838_embryonic+epithelial+tube+formation	0.425706	0.579192	0.234885
BP_GO:0007216_metabotropic+glutamate+receptor+signaling+pathway	0.427858	0.258901	0.651147
BP_GO:0001991_regulation+of+systemic+arterial+blood+pressure+by+circul	0.467746	0.773994	0.298762
BP_GO:0002829_negative+regulation+of+T-helper+2+type+immune+respon	0.468606	0.13057	0.963218
BP_GO:0050919_negative+chemotaxis	0.0161082	0.00633423	0.294987
MF_GO:0030898_actin-dependent+ATPase+activity	0.448076	0.271253	0.786203
BP_GO:0050793_regulation+of+developmental+process	0.0397121	0.0397121	#N/A
BP_GO:0050774_negative+regulation+of+dendrite+morphogenesis	0.295313	0.070821	0.929181
BP_GO:0009437_carnitine+metabolic+process	0.951596	0.845138	0.908745

BP_GO:0001835_blastocyst+hatching	0.639403	0.210714	0.80925
BP_GO:0000255_allantoin+metabolic+process	0.0484695	0.0171312	0.635008
BP_GO:0019441_tryptophan+catabolic+process+to+kynurenine	0.159081	0.0118393	0.560043
BP_GO:0043403_skeletal+muscle+regeneration	0.770287	0.970342	0.200471
BP_GO:0046651_lymphocyte+proliferation	0.592752	0.633937	0.495504
CC_GO:0008290_F-actin+capping+protein+complex	0.328733	#N/A	0.328733
BP_GO:0007094_mitotic+cell+cycle+spindle+assembly+checkpoint	0.396501	0.750155	0.244119
BP_GO:0045332_phospholipid+translocation	0.365996	0.386202	0.422138
BP_GO:0045213_neurotransmitter+receptor+metabolic+process	0.160118	0.441276	0.0413128
BP_GO:0035196_gene+silencing+by+miRNA,+production+of+miRNAs	0.994622	0.994837	0.745243
BP_GO:0043542_endothelial+cell+migration	0.617036	0.852849	0.396765
MF_GO:0004104_cholinesterase+activity	0.691468	#N/A	0.691468
BP_GO:0015908_fatty+acid+transport	0.482919	0.248352	0.86246
MF_GO:0046934_phosphatidylinositol-4,5-bisphosphate+3-kinase+activity	0.919276	0.829957	0.856861
BP_GO:0001660_fever	0.666377	0.935448	0.352175
CC_GO:0005793_ER-Golgi+intermediate+compartment	0.876901	0.830478	0.753006
MF_GO:0004126_cytidine+deaminase+activity	0.868517	0.941244	0.507105
MF_GO:0004769_steroid+delta-isomerase+activity	0.930006	0.700712	0.919281
MF_GO:0045499_chemorepellent+activity	0.158439	0.119504	0.40519
BP_GO:0043009_chordate+embryonic+development	0.382101	0.318896	0.51806
MF_GO:0004185_serine-type+carboxypeptidase+activity	0.255839	0.25184	0.397066
CC_GO:0031982_vesicle	0.802412	0.878334	0.373961
BP_GO:0051271_negative+regulation+of+cell+motion	0.857717	0.666885	0.860381
BP_GO:0015833_peptide+transport	0.738898	0.538881	0.866731
MF_GO:0015197_peptide+transporter+activity	0.738898	0.538881	0.866731
BP_GO:0046849_bone+remodeling	0.0846208	0.163269	0.16722
BP_GO:0008053_mitochondrial+fusion	0.55344	0.427705	0.720306
BP_GO:0032733_positive+regulation+of+interleukin-10+production	0.515123	0.966056	0.0381066
BP_GO:0015871_choline+transport	0.54233	#N/A	0.54233
MF_GO:0016462_pyrophosphatase+activity	0.519795	0.519795	#N/A
BP_GO:0042473_outer+ear+morphogenesis	0.800094	0.804006	0.579515
CC_GO:0030132_clathrin+coat+of+coated+pit	0.277366	0.111696	0.648268
BP_GO:0002035_brain+renin-angiotensin+system	0.423781	0.773994	0.255748
BP_GO:0048843_negative+regulation+of+axon+extension+involved+in+axon	0.00998383	0.019847	0.136786
BP_GO:0051262_protein+tetramerization	0.770817	#N/A	0.770817
MF_GO:0005332_gamma-aminobutyric+acid:sodium+symporter+activity	0.442722	0.319192	0.700229
BP_GO:0042420_dopamine+catabolic+process	0.127065	0.0356919	0.574653
BP_GO:0050768_negative+regulation+of+neurogenesis	0.0579436	0.768143	0.0125671
MF_GO:0008113_peptide-methionine-(S)-S-oxide+reductase+activity	0.227769	0.227769	#N/A
BP_GO:0012501_programmed+cell+death	0.248521	0.173196	0.491827
BP_GO:0050803_regulation+of+synapse+structure+and+activity	0.920091	0.920091	#N/A
MF_GO:0015355_secondary+active+monocarboxylate+transmembrane+tran	0.357753	0.226725	0.592077
MF_GO:0004300_enoyl-CoA+hydratase+activity	0.232423	0.232423	#N/A
BP_GO:0050778_positive+regulation+of+immune+response	0.78431	0.57366	0.788427
BP_GO:0030384_phosphoinositide+metabolic+process	0.424954	0.673813	0.235989
CC_GO:0031252_cell+leading+edge	0.768674	0.691653	0.704661
CC_GO:0016469_proton-transporting+two-sector+ATPase+complex	0.860142	0.935583	0.319273

CC_GO:0031253_cell+projection+membrane	0.807609	0.805441	0.693716
MF_GO:0004427_inorganic+diphosphatase+activity	0.718288	0.718288	#N/A
BP_GO:0006351_transcription,+DNA-dependent	0.0946592	0.869517	0.015189
BP_GO:0030146_diuresis	0.0986043	#N/A	0.0986043
BP_GO:0045061_thymic+T+cell+selection	0.91707	0.91707	#N/A
BP_GO:0045930_negative+regulation+of+mitotic+cell+cycle	0.591254	#N/A	0.591254
BP_GO:0048255_mRNA+stabilization	0.621158	0.668492	0.499939
BP_GO:0010172_embryonic+body+morphogenesis	0.526635	0.503039	0.534238
BP_GO:0030261_chromosome+condensation	0.158484	0.182816	0.262787
MF_GO:0009374_biotin+binding	0.635669	0.765599	0.286857
BP_GO:0001938_positive+regulation+of+endothelial+cell+proliferation	0.251386	0.146001	0.685714
BP_GO:0006927_transformed+cell+apoptosis	0.430962	0.660329	0.254468
CC_GO:0019815_B+cell+receptor+complex	0.461473	0.299998	0.762474
BP_GO:0000303_response+to+superoxide	0.771071	0.648971	0.74762
MF_GO:0046965_retinoid+X+receptor+binding	0.744918	0.866623	0.428737
CC_GO:0005658_alpha+DNA+polymerase:primase+complex	0.261923	0.235642	0.427532
CC_GO:0005868_cytoplasmic+dynein+complex	0.803602	0.777501	0.650184
BP_GO:0050435_beta-amyloid+metabolic+process	0.996401	0.941651	0.996102
BP_GO:0030818_negative+regulation+of+cAMP+biosynthetic+process	0.165467	0.233997	0.25749
BP_GO:0000089_mitotic+metaphase	0.365323	0.392144	0.414466
MF_GO:0004656_procollagen-proline+4-dioxygenase+activity	0.686284	0.872982	0.156986
BP_GO:0021545_cranial+nerve+development	0.942576	0.459104	0.990177
BP_GO:0002051_osteoblast+fate+commitment	0.662734	0.624075	0.609134
MF_GO:0004308_exo-alpha-sialidase+activity	0.54646	0.515351	0.565827
BP_GO:0032816_positive+regulation+of+natural+killer+cell+activation	0.536962	0.566568	0.485104
BP_GO:0033299_secretion+of+lysosomal+enzymes	0.536962	0.566568	0.485104
BP_GO:0045736_negative+regulation+of+cyclin-dependent+protein+kinase+	0.912765	0.679323	0.971973
BP_GO:0001736_establishment+of+planar+polarity	0.442022	0.296355	0.555408
MF_GO:0050431_transforming+growth+factor+beta+binding	0.310722	0.411048	0.274151
MF_GO:0004536_deoxyribonuclease+activity	0.446711	0.633128	0.362551
BP_GO:0045333_cellular+respiration	0.0581234	0.0677992	0.169992
MF_GO:0042801_polo+kinase+kinase+activity	0.315481	0.414507	0.321025
BP_GO:0009967_positive+regulation+of+signal+transduction	0.404411	#N/A	0.404411
BP_GO:0045779_negative+regulation+of+bone+resorption	0.0042079	#N/A	0.0042079
MF_GO:0030332_cyclin+binding	0.541917	0.318252	0.652997
BP_GO:0051881_regulation+of+mitochondrial+membrane+potential	0.233239	0.179534	0.454463
BP_GO:0048617_embryonic+foregut+morphogenesis	0.165902	0.268987	0.224077
BP_GO:0006906_vesicle+fusion	0.89602	#N/A	0.89602
BP_GO:0019885_antigen+processing+and+presentation+of+endogenous+pe	0.172099	0.380335	0.179421
BP_GO:0002326_B+cell+lineage+commitment	0.504231	0.156079	0.723895
CC_GO:0005652_nuclear+lamina	0.766204	0.947878	0.275031
BP_GO:0006269_DNA+replication,+synthesis+of+RNA+primer	0.243141	0.160977	0.407631
MF_GO:0004095_carnitine+O-palmitoyltransferase+activity	0.224006	0.224006	#N/A
BP_GO:0002904_positive+regulation+of+B+cell+apoptosis	0.250291	0.236603	0.357546
CC_GO:0005721_centromeric+heterochromatin	0.681022	0.659315	0.620185
BP_GO:0060296_regulation+of+cilium+beat+frequency+involved+in+ciliary+	0.825188	0.652913	0.882993
BP_GO:0015074_DNA+integration	0.266213	0.74309	0.136021

MF_GO:0000179_rRNA+(adenine-N6,N6-)-dimethyltransferase+activity	0.517163	0.669655	0.249468
MF_GO:0008649_rRNA+methyltransferase+activity	0.517163	0.669655	0.249468
CC_GO:0008091_spectrin	0.36273	0.47782	0.354108
BP_GO:0006610_ribosomal+protein+import+into+nucleus	0.555335	0.295414	0.768388
BP_GO:0046632_alpha-beta+T+cell+differentiation	0.0404837	0.0599334	0.180035
BP_GO:0048013_ephrin+receptor+signaling+pathway	0.130858	0.485624	0.0143935
BP_GO:0048558_embryonic+gut+morphogenesis	0.129098	0.186096	0.236283
MF_GO:0005313_L-glutamate+transmembrane+transporter+activity	0.206949	0.214766	0.356715
BP_GO:0007386_compartment+specification	0.213582	0.223846	0.357393
MF_GO:0005005_transmembrane-ephrin+receptor+activity	0.912666	0.436146	0.981264
BP_GO:0007028_cytoplasm+organization	0.985752	0.985752	#N/A
MF_GO:0043015_gamma-tubulin+binding	0.473017	0.378067	0.539911
BP_GO:0001817_regulation+of+cytokine+production	0.729906	0.422454	0.855752
BP_GO:0009308_cellular+amine+metabolic+process	0.937815	0.958173	0.530616
MF_GO:0015631_tubulin+binding	0.427415	0.581257	0.320955
CC_GO:0005853_eukaryotic+translation+elongation+factor+1+complex	0.925092	0.806797	0.87918
MF_GO:0048365_Rac+GTPase+binding	0.0568388	0.188718	0.0873982
MF_GO:0016614_oxidoreductase+activity,+acting+on+CH-OH+group+of+do	0.627579	0.727423	0.442164
BP_GO:0050965_detection+of+temperature+stimulus+involved+in+sensory+	0.783938	0.671342	0.788941
BP_GO:0051146_striated+muscle+cell+differentiation	0.142082	0.431739	0.0893628
BP_GO:0055072_iron+ion+homeostasis	0.0660268	0.280411	0.0800424
BP_GO:0001895_retina+homeostasis	0.392819	0.588991	0.328303
BP_GO:0048149_behavioral+response+to+ethanol	0.428967	#N/A	0.428967
BP_GO:0051898_negative+regulation+of+protein+kinase+B+signaling+casca	0.723573	0.859071	0.406215
BP_GO:0060158_dopamine+receptor,+phospholipase+C+activating+pathwa	0.441546	0.667475	0.260418
MF_GO:0005337_nucleoside+transmembrane+transporter+activity	0.187049	0.293102	0.23747
BP_GO:0007599_hemostasis	0.156608	0.0959239	0.33987
BP_GO:0032713_negative+regulation+of+interleukin-4+production	0.256714	0.506416	0.0904698
BP_GO:0048608_reproductive+structure+development	0.815193	0.282704	0.967371
BP_GO:0031119_tRNA+pseudouridine+synthesis	0.00843257	#N/A	0.00843257
BP_GO:0050810_regulation+of+steroid+biosynthetic+process	0.477072	0.28193	0.689606
BP_GO:0001825_blastocyst+formation	0.53843	0.808259	0.0935692
MF_GO:0004439_phosphatidylinositol-4,5-bisphosphate+5-phosphatase+ac	0.932155	0.990781	0.135781
BP_GO:0034109_homotypic+cell-cell+adhesion	0.734881	0.94121	0.248944
MF_GO:0042562_hormone+binding	0.407509	0.0641532	0.984885
BP_GO:0045822_negative+regulation+of+heart+contraction	0.598619	0.598619	#N/A
MF_GO:0004500_dopamine+beta-monooxygenase+activity	0.950817	0.923859	0.817653
BP_GO:0030166_proteoglycan+biosynthetic+process	0.0919327	0.18886	0.128567
BP_GO:0043129_surfactant+homeostasis	0.0393642	0.155914	0.0697594
BP_GO:0043069_negative+regulation+of+programmed+cell+death	0.128536	0.421599	0.115914
BP_GO:0045745_positive+regulation+of+G-protein+coupled+receptor+prote	0.307895	0.550629	0.200901
BP_GO:0060124_positive+regulation+of+growth+hormone+secretion	0.214293	0.357427	0.240612
BP_GO:0042787_protein+ubiquitination+during+ubiquitin-dependent+prote	0.701065	0.631375	0.682021
MF_GO:0017147_Wnt-protein+binding	0.662721	0.680272	0.54964
BP_GO:0019228_regulation+of+action+potential+in+neuron	0.512342	0.899858	0.240823
CC_GO:0030673_axolemma	0.551935	0.720112	0.344787
MF_GO:0005381_iron+ion+transmembrane+transporter+activity	0.234633	0.33291	0.27652

BP_GO:0030010_establishment+of+cell+polarity	0.0343925	0.0694815	0.140034
MF_GO:0001730_2'-5'-oligoadenylate+synthetase+activity	0.137596	0.0476664	0.54884
BP_GO:0045580_regulation+of+T+cell+differentiation	0.954295	0.940186	0.751973
BP_GO:0042403_thyroid+hormone+metabolic+process	0.694272	#N/A	0.694272
MF_GO:0042623_ATPase+activity,+coupled	0.0756581	0.0250127	0.298832
BP_GO:0033152_immunoglobulin+V(D)J+recombination	0.412196	0.885344	0.170965
BP_GO:0001514_selenocysteine+incorporation	0.89153	0.916904	0.641222
BP_GO:0006091_generation+of+precursor+metabolites+and+energy	0.0132915	0.0881484	0.0369726
BP_GO:0030949_positive+regulation+of+vascular+endothelial+growth+factc	0.849435	0.993782	0.402059
BP_GO:0031122_cytoplasmic+microtubule+organization	0.0478556	0.182823	0.0729758
MF_GO:0016742_hydroxymethyl-,+formyl-+and+related+transferase+activit	0.522341	#N/A	0.522341
CC_GO:0005663_DNA+replication+factor+C+complex	0.683542	0.262964	0.82036
MF_GO:0003689_DNA+clamp+loader+activity	0.683542	0.262964	0.82036
BP_GO:0048675_axon+extension	0.519562	0.519562	#N/A
MF_GO:0005088_Ras+guanyl-nucleotide+exchange+factor+activity	0.603121	#N/A	0.603121
BP_GO:0048565_gut+development	0.778909	0.827846	0.556011
BP_GO:0015780_nucleotide-sugar+transport	0.757668	0.634145	0.789232
MF_GO:0005338_nucleotide-sugar+transmembrane+transporter+activity	0.757668	0.634145	0.789232
BP_GO:0021854_hypothalamus+development	0.256277	0.236603	0.365642
BP_GO:0046642_negative+regulation+of+alpha-beta+T+cell+proliferation	0.378012	0.607814	0.302435
BP_GO:0021795_cerebral+cortex+cell+migration	0.997452	0.850426	0.995799
BP_GO:0021696_cerebellar+cortex+morphogenesis	0.838371	0.8702	0.60623
CC_GO:0070044_synaptobrevin+2-SNAP-25-syntaxin-1a+complex	0.989153	0.672403	0.991651
CC_GO:0005638_lamin+filament	0.877048	0.762863	0.86034
BP_GO:0015879_carnitine+transport	0.476138	0.434201	0.531877
MF_GO:0015226_carnitine+transporter+activity	0.476138	0.434201	0.531877
BP_GO:0021884_forebrain+neuron+development	0.513533	0.729631	0.286142
BP_GO:0006875_cellular+metal+ion+homeostasis	0.548062	#N/A	0.548062
BP_GO:0042476_odontogenesis	0.666956	0.867185	0.307298
BP_GO:0006595_polyamine+metabolic+process	0.264188	0.264407	0.396116
BP_GO:0006287_base-excision+repair,+gap-filling	0.327084	0.627481	0.0718413
CC_GO:0005930_axoneme	0.162001	0.219241	0.244144
BP_GO:0031532_actin+cytoskeleton+reorganization	0.937813	0.803629	0.906406
BP_GO:0001945_lymph+vessel+development	0.0490435	0.0215476	0.228226
BP_GO:0060011_Sertoli+cell+proliferation	0.403303	0.183771	0.857878
MF_GO:0003840_gamma-glutamyltransferase+activity	0.108207	0.124854	0.27451
BP_GO:0035021_negative+regulation+of+Rac+protein+signal+transduction	0.0357381	0.0133872	0.36809
MF_GO:0003923_GPI-anchor+transamidase+activity	0.27024	0.449589	0.2295
BP_GO:0030033_microvillus+assembly	0.496778	0.369885	0.572019
BP_GO:0045806_negative+regulation+of+endocytosis	0.561223	0.546304	0.542046
MF_GO:0004060_arylamine+N-acetyltransferase+activity	0.369179	#N/A	0.369179
BP_GO:0043303_mast+cell+degranulation	0.337964	0.203954	0.49751
BP_GO:0050882_voluntary+musculoskeletal+movement	0.427205	0.142959	0.930763
MF_GO:0004784_superoxide+dismutase+activity	0.0699148	0.351965	0.0436023
BP_GO:0046887_positive+regulation+of+hormone+secretion	0.309134	#N/A	0.309134
BP_GO:0008406_gonad+development	0.862902	#N/A	0.862902
BP_GO:0043200_response+to+amino+acid+stimulus	0.711637	0.82619	0.304359

MF_GO:0004735_pyrroline-5-carboxylate+reductase+activity	0.459364	0.367266	0.6489
BP_GO:0021612_facial+nerve+structural+organization	0.0466679	0.274917	0.0554678
BP_GO:0007143_female+meiosis	0.566967	0.606437	0.487085
MF_GO:0004974_leukotriene+receptor+activity	0.053483	0.35642	0.0494393
BP_GO:0042416_dopamine+biosynthetic+process	0.789109	#N/A	0.789109
CC_GO:0043204_perikaryon	0.188464	0.232187	0.274495
BP_GO:0055088_lipid+homeostasis	0.98442	0.90617	0.978823
BP_GO:0060173_limb+development	0.159897	0.947934	0.0184382
CC_GO:0008250_oligosaccharyltransferase+complex	0.608379	0.605499	0.548031
CC_GO:0005958_DNA-dependent+protein+kinase+complex	0.178971	#N/A	0.178971
MF_GO:0005499_vitamin+D+binding	0.318633	0.338714	0.400403
BP_GO:0015844_monoamine+transport	0.10685	0.0812157	0.26394
MF_GO:0000030_mannosyltransferase+activity	0.600413	0.312012	0.912783
MF_GO:0005243_gap+junction+channel+activity	0.166335	0.179733	0.27722
BP_GO:0032495_response+to+muramyl+dipeptide	0.399444	0.781816	0.127163
MF_GO:0046703_natural+killer+cell+lectin-like+receptor+binding	0.598483	0.326349	0.788718
CC_GO:0031513_nonmotile+primary+cilium	0.469425	0.405142	0.603085
BP_GO:0002669_positive+regulation+of+T+cell+anergy	0.349308	0.217697	0.717605
MF_GO:0008353_RNA+polymerase+subunit+kinase+activity	0.841209	0.958867	0.56018
BP_GO:0006510_ATP-dependent+proteolysis	0.985736	0.870299	0.992428
MF_GO:0004176_ATP-dependent+peptidase+activity	0.985736	0.870299	0.992428
BP_GO:0016322_neuron+remodeling	0.867269	0.7356	0.87218
CC_GO:0070032_synaptobrevin+2-SNAP-25-syntaxin-1a-complexin+I+compl	0.986644	0.612697	0.991651
MF_GO:0005152_interleukin-1+receptor+antagonist+activity	0.5752	0.450913	0.61388
CC_GO:0032281_alpha-amino-3-hydroxy-5-methyl-4-isoxazolepropionic+aci	0.0107255	0.173876	0.0171871
CC_GO:0035098_ESC/E(Z)+complex	0.392274	0.537597	0.314803
BP_GO:0051798_positive+regulation+of+hair+follicle+development	0.746262	0.934482	0.283281
CC_GO:0005798_Golgi-associated+vesicle	0.811596	0.191114	0.983143
BP_GO:0021938_smoothened+signaling+pathway+involved+in+regulation+c	0.320494	0.602727	0.245248
BP_GO:0048025_negative+regulation+of+nuclear+mRNA+splicing,+via+splic	0.10769	0.807413	0.0266468
MF_GO:0016594_glycine+binding	0.828842	0.708848	0.781965
BP_GO:0048814_regulation+of+dendrite+morphogenesis	0.475511	0.322692	0.576811
BP_GO:0032720_negative+regulation+of+tumor+necrosis+factor+productio	0.316239	0.39646	0.307195
MF_GO:0004999_vasoactive+intestinal+polypeptide+receptor+activity	0.110918	0.0924458	0.343114
BP_GO:0007066_female+meiosis+sister+chromatid+cohesion	0.933021	#N/A	0.933021
BP_GO:0045672_positive+regulation+of+osteoclast+differentiation	0.480692	0.477216	0.500342
BP_GO:0045987_positive+regulation+of+smooth+muscle+contraction	0.104168	#N/A	0.104168
BP_GO:0060235_lens+induction+in+camera-type+eye	0.969095	0.942087	0.857567
BP_GO:0050828_regulation+of+liquid+surface+tension	0.131795	0.174691	0.225951
BP_GO:0007292_female+gamete+generation	0.636831	0.905007	0.36228
BP_GO:0030259_lipid+glycosylation	0.119687	0.432992	0.0194696
CC_GO:0005956_protein+kinase+CK2+complex	0.855749	0.748616	0.832083
BP_GO:0021853_cerebral+cortex+GABAergic+interneuron+migration	0.0772554	0.484701	0.0522855
MF_GO:0008301_DNA+bending+activity	0.0284949	0.260607	0.020032
MF_GO:0008853_exodeoxyribonuclease+III+activity	0.967301	0.967301	#N/A
BP_GO:0021707_cerebellar+granule+cell+differentiation	0.34643	0.199122	0.612105
BP_GO:0032689_negative+regulation+of+interferon-gamma+production	0.0650119	0.338383	0.0421148

CC_GO:0045179_apical+cortex	0.397497	0.357591	0.498484
BP_GO:0021569_rhombomere+3+development	0.161245	0.421607	0.114431
MF_GO:0004985_opioid+receptor+activity	0.172103	0.193217	0.317942
BP_GO:0000289_nuclear-transcribed+mRNA+poly(A)+tail+shortening	0.163811	0.276491	0.213886
MF_GO:0004791_thioredoxin-disulfide+reductase+activity	0.919675	0.964265	0.375608
BP_GO:0006824_cobalt+ion+transport	0.96472	0.999096	0.286984
BP_GO:0021772_olfactory+bulb+development	0.409669	0.734626	0.265634
MF_GO:0035254_glutamate+receptor+binding	0.019626	0.019626	#N/A
BP_GO:0050953_sensory+perception+of+light+stimulus	0.507687	0.551027	0.426597
CC_GO:0001917_photoreceptor+inner+segment	0.188341	0.470879	0.0499901
MF_GO:0016208_AMP+binding	0.0156993	0.0156993	#N/A
BP_GO:0048505_regulation+of+timing+of+cell+differentiation	0.720677	0.654049	0.685416
MF_GO:0015467_G-protein+activated+inward+rectifier+potassium+channel	0.786719	0.798202	0.613686
BP_GO:0001823_mesonephros+development	0.540197	0.721296	0.328206
BP_GO:0032332_positive+regulation+of+chondrocyte+differentiation	0.655188	0.643519	0.5778
BP_GO:0014051_gamma-aminobutyric+acid+secretion	0.240132	0.421713	0.241306
MF_GO:0030519_snoRNP+binding	0.771558	0.882936	0.445138
CC_GO:0043186_P+granule	0.182011	0.229619	0.296025
BP_GO:0051967_negative+regulation+of+synaptic+transmission,+glutamate	0.43665	0.899858	0.177851
BP_GO:0006432_phenylalanyl-tRNA+aminoacylation	0.995908	0.939634	0.98572
BP_GO:0008156_negative+regulation+of+DNA+replication	0.809344	0.508628	0.888064
CC_GO:0043159_acrosomal+matrix	0.958977	0.985661	0.607166
MF_GO:0017150_tRNA+dihydrouridine+synthase+activity	0.515542	0.414538	0.674039
BP_GO:0001661_conditioned+taste+aversion	0.298409	0.322692	0.364561
BP_GO:0033327_Leydig+cell+differentiation	0.859098	0.973825	0.337911
BP_GO:0042177_negative+regulation+of+protein+catabolic+process	0.43896	0.288738	0.556914
MF_GO:0004052_arachidonate+12-lipoxygenase+activity	0.595786	0.622402	0.477543
BP_GO:0045862_positive+regulation+of+proteolysis	0.6139	0.288462	0.833118
BP_GO:0021879_forebrain+neuron+differentiation	0.298939	0.39849	0.322196
BP_GO:0050868_negative+regulation+of+T+cell+activation	0.000253105	#N/A	0.000253105
MF_GO:0004950_chemokine+receptor+activity	0.660985	#N/A	0.660985
MF_GO:0046920_alpha(1,3)-fucosyltransferase+activity	0.869792	0.939763	0.330048
CC_GO:0032045_guanyl-nucleotide+exchange+factor+complex	0.274841	0.731525	0.147365
BP_GO:0000079_regulation+of+cyclin-dependent+protein+kinase+activity	0.559396	0.335122	0.737704
BP_GO:0014003_oligodendrocyte+development	0.104684	0.39041	0.0985013
BP_GO:0043623_cellular+protein+complex+assembly	0.294441	0.684454	0.106379
CC_GO:0000801_central+element	0.442586	0.483198	0.435164
MF_GO:0045182_translation+regulator+activity	0.516625	0.617496	0.450272
BP_GO:0019216_regulation+of+lipid+metabolic+process	0.987683	0.805566	0.982035
MF_GO:0003945_N-acetyllactosamine+synthase+activity	0.452239	0.72723	0.098591
MF_GO:0042826_histone+deacetylase+binding	0.423136	0.692349	0.303457
BP_GO:0051930_regulation+of+sensory+perception+of+pain	0.12634	0.216231	0.175944
BP_GO:0022011_myelination+in+the+peripheral+nervous+system	0.580072	0.454563	0.655091
CC_GO:0035085_cilium+axoneme	0.323893	#N/A	0.323893
CC_GO:0000120_RNA+polymerase+I+transcription+factor+complex	0.622743	0.85634	0.2668
BP_GO:0014832_urinary+bladder+smooth+muscle+contraction	0.484735	0.924764	0.0677147
MF_GO:0001642_group+III+metabotropic+glutamate+receptor+activity	0.0597857	0.11108	0.136871

MF_GO:0015016_[heparan+sulfate]-glucosamine+N-sulfotransferase+activit	0.950829	0.978638	0.418976
MF_GO:0008504_monoamine+transmembrane+transporter+activity	0.410483	0.480128	0.393032
CC_GO:0048500_signal+recognition+particle	0.549556	0.454502	0.672317
MF_GO:0008312_7S+RNA+binding	0.549556	0.454502	0.672317
BP_GO:0021797_forebrain+anterior/posterior+pattern+formation	0.354045	0.476058	0.258829
CC_GO:0032983_kainate+selective+glutamate+receptor+complex	0.375188	0.603201	0.137411
MF_GO:0019104_DNA+N-glycosylase+activity	0.2454	0.082515	0.659926
MF_GO:0015279_store-operated+calcium+channel+activity	0.0961357	0.219787	0.144355
CC_GO:0005736_DNA-directed+RNA+polymerase+I+complex	0.797973	0.797973	#N/A
CC_GO:0001652_granular+component	0.105429	0.0667605	0.280771
CC_GO:0005773_vacuole	0.648275	0.648275	#N/A
BP_GO:0016973_poly(A)++mRNA+export+from+nucleus	0.795625	0.72735	0.713356
MF_GO:0004969_histamine+receptor+activity	0.566561	0.75949	0.31973
CC_GO:0033391_chromatoid+body	0.36967	0.442005	0.372223
BP_GO:0021979_hypothalamus+cell+differentiation	0.429918	0.400967	0.531897
BP_GO:0030252_growth+hormone+secretion	0.816547	0.98092	0.438703
BP_GO:0055012_ventricular+cardiac+muscle+cell+differentiation	0.472613	0.472613	#N/A
BP_GO:0050913_sensory+perception+of+bitter+taste	0.20877	#N/A	0.20877
BP_GO:0006465_signal+peptide+processing	0.586988	0.579285	0.554787
MF_GO:0016934_extracellular-glycine-gated+chloride+channel+activity	0.871561	0.708848	0.839304
BP_GO:0021542_dentate+gyrus+development	0.539764	0.313257	0.734644
BP_GO:0009303_rRNA+transcription	0.71497	0.953809	0.0373872
MF_GO:0015057_thrombin+receptor+activity	0.223383	0.278149	0.294548
BP_GO:0006400_tRNA+modification	0.746072	0.746072	#N/A
BP_GO:0045604_regulation+of+epidermal+cell+differentiation	0.826004	0.932965	0.235903
MF_GO:0050327_testosterone+17-beta-dehydrogenase+activity	0.448709	0.453097	0.469903
MF_GO:0047961_glycine+N-acyltransferase+activity	0.383186	0.21085	0.580394
BP_GO:0019510_S-adenosylhomocysteine+catabolic+process	0.815835	0.815835	#N/A
BP_GO:0001960_negative+regulation+of+cytokine+mediated+signaling+patl	0.198275	0.0965587	0.644515
MF_GO:0004082_bisphosphoglycerate+mutase+activity	0.795808	0.795808	#N/A
MF_GO:0004083_bisphosphoglycerate+2-phosphatase+activity	0.795808	0.795808	#N/A
MF_GO:0004619_phosphoglycerate+mutase+activity	0.795808	0.795808	#N/A
BP_GO:0043056_forward+locomotion	0.35269	0.579682	0.272212
BP_GO:0055002_striated+muscle+cell+development	0.35269	0.579682	0.272212
MF_GO:0008034_lipoprotein+binding	0.515471	0.0825974	0.978823
MF_GO:0005504_fatty+acid+binding	0.20055	0.352837	0.222819
BP_GO:0016125_sterol+metabolic+process	0.00184612	0.016968	0.020985
CC_GO:0005945_6-phosphofructokinase+complex	0.339269	0.339269	#N/A
MF_GO:0003872_6-phosphofructokinase+activity	0.339269	0.339269	#N/A
BP_GO:0030193_regulation+of+blood+coagulation	0.578883	0.578883	#N/A
BP_GO:0015909_long-chain+fatty+acid+transport	0.538532	0.538532	#N/A
MF_GO:0004345_glucose-6-phosphate+dehydrogenase+activity	0.207796	0.388528	0.212385
MF_GO:0004090_carbonyl+reductase+(NADPH)+activity	0.0481511	0.0481511	#N/A
BP_GO:0001937_negative+regulation+of+endothelial+cell+proliferation	0.293439	0.156632	0.685714
BP_GO:0043496_regulation+of+protein+homodimerization+activity	0.450776	0.436216	0.484476
BP_GO:0006144_purine+base+metabolic+process	0.527269	0.385866	0.613323
BP_GO:0001915_negative+regulation+of+T+cell+mediated+cytotoxicity	0.168561	0.0751149	0.644515

MF_GO:0004332_fructose-bisphosphate+aldolase+activity	0.578956	0.578956	#N/A
BP_GO:0035025_positive+regulation+of+Rho+protein+signal+transduction	0.155939	0.0530485	0.460988
BP_GO:0045777_positive+regulation+of+blood+pressure	0.107971	0.305573	0.123511
BP_GO:0005980_glycogen+catabolic+process	0.411205	0.411205	#N/A
MF_GO:0004944_C5a+anaphylatoxin+receptor+activity	0.42111	0.898822	0.015761
MF_GO:0003876_AMP+deaminase+activity	0.598306	#N/A	0.598306
MF_GO:0004144_diacylglycerol+O-acyltransferase+activity	0.623782	0.623782	#N/A
CC_GO:0016234_inclusion+body	0.836298	0.955894	0.23706
BP_GO:0008154_actin+polymerization+or+depolymerization	0.0185329	0.461018	0.00645423
MF_GO:0004505_phenylalanine+4-monooxygenase+activity	0.205713	0.29116	0.258815
CC_GO:0032154_cleavage+furrow	0.764603	0.803021	0.517113
MF_GO:0004064_arylesterase+activity	0.762653	0.762653	#N/A
BP_GO:0050717_positive+regulation+of+interleukin-1+alpha+secretion	0.646643	0.642612	0.553326
BP_GO:0006509_membrane+protein+ectodomain+proteolysis	0.903981	#N/A	0.903981
MF_GO:0008449_N-acetylglucosamine-6-sulfatase+activity	0.533936	#N/A	0.533936
MF_GO:0004473_malate+dehydrogenase+(oxaloacetate-decarboxylating)+(	0.0823959	0.50294	0.0438162
BP_GO:0050732_negative+regulation+of+peptidyl-tyrosine+phosphorylation	0.257433	0.144428	0.644515
BP_GO:0030857_negative+regulation+of+epithelial+cell+differentiation	0.205075	0.0966717	0.659657
BP_GO:0002025_vasodilation+by+norepinephrine-epinephrine+involved+in+	0.559203	0.199282	0.926711
BP_GO:0031649_heat+generation	0.559203	0.199282	0.926711
MF_GO:0004065_arylsulfatase+activity	0.428879	#N/A	0.428879
BP_GO:0045600_positive+regulation+of+fat+cell+differentiation	0.354336	0.354336	#N/A
BP_GO:0043691_reverse+cholesterol+transport	0.803715	0.596348	0.809032
MF_GO:0016212_kynurenine-oxoglutarate+transaminase+activity	0.954586	0.928406	0.804846
BP_GO:0060325_face+morphogenesis	0.403004	0.0998118	0.727292
BP_GO:0048251_elastic+fiber+assembly	0.742011	0.577094	0.744602
BP_GO:0006689_ganglioside+catabolic+process	0.0921788	0.0921788	#N/A
BP_GO:0014070_response+to+organic+cyclic+substance	0.657058	0.84367	0.413417
BP_GO:0018894_dibenzo-p-dioxin+metabolic+process	0.127876	0.846329	0.017231
MF_GO:0004594_pantothenate+kinase+activity	0.404468	0.560855	0.262022
MF_GO:0003985_acetyl-CoA+C-acetyltransferase+activity	0.580676	0.830261	0.158689
MF_GO:0042578_phosphoric+ester+hydrolase+activity	0.371765	0.346625	0.495908
BP_GO:0006642_triacylglycerol+mobilization	0.863437	0.616493	0.930518
BP_GO:0006536_glutamate+metabolic+process	0.752804	0.884569	0.304359
BP_GO:0042359_vitamin+D+metabolic+process	0.894196	0.788142	0.832564
BP_GO:0030029_actin+filament-based+process	0.839617	0.780669	0.748062
BP_GO:0045621_positive+regulation+of+lymphocyte+differentiation	0.0224132	0.0238505	0.248966
BP_GO:0006564_L-serine+biosynthetic+process	0.0108771	0.0439284	0.0588699
BP_GO:0045103_intermediate+filament-based+process	0.495621	#N/A	0.495621
BP_GO:0015760_glucose-6-phosphate+transport	0.965063	0.965063	#N/A
CC_GO:0031362_anchored+to+external+side+of+plasma+membrane	0.0114776	#N/A	0.0114776
BP_GO:0001805_positive+regulation+of+type+III+hypersensitivity	0.367002	#N/A	0.367002
MF_GO:0008469_histone-arginine+N-methyltransferase+activity	0.289357	0.289357	#N/A
BP_GO:0009060_aerobic+respiration	0.374221	0.657178	0.129234
BP_GO:0046541_saliva+secretion	0.0817586	0.219787	0.12266
BP_GO:0015697_quaternary+ammonium+group+transport	0.457864	0.776824	0.251877
MF_GO:0030247_polysaccharide+binding	0.632384	0.632384	#N/A

MF_GO:0003716_RNA+polymerase+I+transcription+termination+factor+acti	0.653397	0.653397	#N/A
BP_GO:0006103_2-oxoglutarate+metabolic+process	0.0171312	0.0171312	#N/A
BP_GO:0006549_isoleucine+metabolic+process	0.0171312	0.0171312	#N/A
BP_GO:0006600_creatine+metabolic+process	0.0171312	0.0171312	#N/A
BP_GO:0046449_creatinine+metabolic+process	0.0171312	0.0171312	#N/A
CC_GO:0043259_laminin-10+complex	0.0523249	0.0621855	0.183233
MF_GO:0004775_succinate-CoA+ligase+(ADP-forming)+activity	0.510574	0.795173	0.130932
BP_GO:0006868_glutamine+transport	0.22214	0.461267	0.117037
MF_GO:0015186_L-glutamine+transmembrane+transporter+activity	0.22214	0.461267	0.117037
BP_GO:0045907_positive+regulation+of+vasoconstriction	0.586004	0.420528	0.745163
BP_GO:0008228_opsonization	0.0150009	0.0275768	0.146866
MF_GO:0030676_Rac+guanyl-nucleotide+exchange+factor+activity	0.0526118	0.118009	0.125412
CC_GO:0060053_neurofilament+cytoskeleton	0.127378	0.267313	0.136307
MF_GO:0047238_glucuronosyl-N-acetylgalactosaminyl-proteoglycan+4-beta	0.689802	0.887196	0.401189
MF_GO:0050510_N-acetylgalactosaminyl-proteoglycan+3-beta-glucuronosyl	0.689802	0.887196	0.401189
MF_GO:0004792_thiosulfate+sulfurtransferase+activity	0.106238	0.161413	0.222492
BP_GO:0045663_positive+regulation+of+myoblast+differentiation	0.717766	#N/A	0.717766
MF_GO:0004165_dodecenoyl-CoA+delta-isomerase+activity	0.0288811	0.0288811	#N/A
BP_GO:0008215_spermine+metabolic+process	0.316076	#N/A	0.316076
BP_GO:0043392_negative+regulation+of+DNA+binding	0.892807	0.602727	0.909286
BP_GO:0031397_negative+regulation+of+protein+ubiquitination	0.989955	0.891835	0.975792
MF_GO:0004859_phospholipase+inhibitor+activity	0.507347	#N/A	0.507347
BP_GO:0060157_urinary+bladder+development	0.910932	#N/A	0.910932
BP_GO:0043374_CD8-positive,+alpha-beta+T+cell+differentiation	0.977048	#N/A	0.977048
BP_GO:0043393_regulation+of+protein+binding	0.659479	#N/A	0.659479
BP_GO:0060068_vagina+development	0.622315	0.248537	0.9332
MF_GO:0008475_procollagen-lysine+5-dioxygenase+activity	0.49673	0.806241	0.107813
MF_GO:0016884_carbon-nitrogen+ligase+activity,+with+glutamine+as+amic	0.768034	0.827271	0.473525
MF_GO:0004955_prostaglandin+receptor+activity	0.403116	0.683901	0.261221
BP_GO:0002281_macrophage+activation+during+immune+response	0.155545	0.318419	0.181868
BP_GO:0002238_response+to+molecule+of+fungal+origin	0.0426624	0.0250257	0.416281
CC_GO:0005640_nuclear+outer+membrane	0.813562	0.197134	0.954767
MF_GO:0004117_calmodulin-dependent+cyclic-nucleotide+phosphodiestera	0.0255185	#N/A	0.0255185
MF_GO:0048101_calcium-+and+calmodulin-regulated+3',5'-cyclic-GMP+pho	0.0255185	#N/A	0.0255185
BP_GO:0030953_spindle+astral+microtubule+organization	0.807269	0.780107	0.697277
BP_GO:0050820_positive+regulation+of+coagulation	0.95061	0.787988	0.927416
BP_GO:0001955_blood+vessel+maturation	0.0559992	0.1979	0.0599646
BP_GO:0060087_relaxation+of+vascular+smooth+muscle	0.2056	#N/A	0.2056
BP_GO:0042133_neurotransmitter+metabolic+process	0.0404657	0.179178	0.0680929
BP_GO:0042246_tissue+regeneration	0.0907072	0.0944445	0.32312
BP_GO:0008347_glial+cell+migration	0.59953	0.735082	0.445962
BP_GO:0003016_respiratory+system+process	0.112887	0.112887	#N/A
BP_GO:0045926_negative+regulation+of+growth	0.670398	#N/A	0.670398
BP_GO:0003014_renal+system+process	0.28692	0.69332	0.147562
BP_GO:0006725_cellular+aromatic+compound+metabolic+process	0.316757	#N/A	0.316757
BP_GO:0007182_common-partner+SMAD+protein+phosphorylation	0.268537	0.597212	0.175923
MF_GO:0001786_phosphatidylserine+binding	0.194514	0.423388	0.111001

MF_GO:0033829_O-fucosylpeptide+3-beta-N-acetylglucosaminyltransferase	0.0887498	0.376786	0.0290468
BP_GO:0050819_negative+regulation+of+coagulation	0.525424	0.525424	#N/A
BP_GO:0006651_diacylglycerol+biosynthetic+process	0.69451	0.922295	0.12931
MF_GO:0000036_acyl+carrier+activity	0.24833	0.24833	#N/A
BP_GO:0050665_hydrogen+peroxide+biosynthetic+process	0.477478	0.846329	0.214359
MF_GO:0004937_alpha1-adrenergic+receptor+activity	0.135637	0.0451121	0.439783
BP_GO:0048007_antigen+processing+and+presentation,+exogenous+lipid+a	0.469387	0.151553	0.907074
CC_GO:0000015_phosphopyruvate+hydratase+complex	0.235291	0.819171	0.0630739
MF_GO:0004634_phosphopyruvate+hydratase+activity	0.235291	0.819171	0.0630739
MF_GO:0008559_xenobiotic-transporting+ATPase+activity	0.452715	0.844671	0.19412
BP_GO:0035051_cardiac+cell+differentiation	0.335148	0.183925	0.545336
MF_GO:0030169_low-density+lipoprotein+binding	0.0366653	0.0366653	#N/A
BP_GO:0043267_negative+regulation+of+potassium+ion+transport	0.497052	0.0366143	0.895443
BP_GO:0045986_negative+regulation+of+smooth+muscle+contraction	0.799874	0.212379	0.944513
BP_GO:0019941_modification-dependent+protein+catabolic+process	0.0832503	0.0832503	#N/A
BP_GO:0002377_immunoglobulin+production	0.0393113	0.0786764	0.123752
BP_GO:0007097_nuclear+migration	0.327681	0.268143	0.455945
BP_GO:0051707_response+to+other+organism	0.910558	0.632118	0.96798
BP_GO:0002481_antigen+processing+and+presentation+of+exogenous+prot	0.687086	0.509109	0.719197
MF_GO:0016763_transferase+activity,+transferring+pentosyl+groups	0.878134	0.952521	0.36579
MF_GO:0001872_zymosan+binding	0.118969	0.118969	#N/A
BP_GO:0019432_triacylglycerol+biosynthetic+process	0.496905	0.784633	0.12931
BP_GO:0009410_response+to+xenobiotic+stimulus	0.829059	0.782186	0.72999
BP_GO:0002682_regulation+of+immune+system+process	0.323804	0.121814	0.60375
BP_GO:0006926_virus-infected+cell+apoptosis	0.356451	#N/A	0.356451
BP_GO:0016458_gene+silencing	0.361195	0.306022	0.469023
BP_GO:0001820_serotonin+secretion	0.0114504	0.35476	0.00578381
BP_GO:0009605_response+to+external+stimulus	0.204187	0.318392	0.248219
MF_GO:0017160_Ral+GTPase+binding	0.752498	0.743881	0.645097
MF_GO:0005375_copper+ion+transmembrane+transporter+activity	0.839204	0.89637	0.626432
MF_GO:0004311_farnesyltranstransferase+activity	0.156698	0.495395	0.0415688
MF_GO:0030506_ankyrin+binding	0.383454	0.591405	0.199825
MF_GO:0003987_acetate-CoA+ligase+activity	0.0377122	0.0558231	0.20257
BP_GO:0006519_cellular+amino+acid+and+derivative+metabolic+process	0.624066	0.594166	0.583103
MF_GO:0046875_ephrin+receptor+binding	0.0252625	0.197185	0.0143935
BP_GO:0019987_negative+regulation+of+anti-apoptosis	0.254171	0.236603	0.380429
CC_GO:0005955_calcineurin+complex	0.545481	0.400694	0.709835
BP_GO:0048935_peripheral+nervous+system+neuron+development	0.875641	0.898237	0.578993
MF_GO:0004517_nitric-oxide+synthase+activity	0.292997	0.0297789	0.957346
BP_GO:0045404_positive+regulation+of+interleukin-4+biosynthetic+process	0.574262	0.0245047	0.947342
MF_GO:0042301_phosphate+binding	0.00269251	0.00269251	#N/A
BP_GO:0002573_myeloid+leukocyte+differentiation	0.954688	0.787988	0.93415
BP_GO:0010043_response+to+zinc+ion	0.02697	0.00700626	0.553326
BP_GO:0030104_water+homeostasis	0.829681	#N/A	0.829681
BP_GO:0032436_positive+regulation+of+proteasomal+ubiquitin-dependent-	0.664069	0.496548	0.772022
CC_GO:0005774_vacuolar+membrane	0.870077	0.784328	0.794926
BP_GO:0006085_acetyl-CoA+biosynthetic+process	0.0258962	0.0558231	0.130932

BP_GO:0007184_SMAD+protein+nuclear+translocation	0.637403	0.597212	0.600999
MF_GO:0016309_1-phosphatidylinositol-5-phosphate+4-kinase+activity	0.256155	0.256155	#N/A
MF_GO:0004862_cAMP-dependent+protein+kinase+inhibitor+activity	0.128899	0.559571	0.0676918
BP_GO:0050869_negative+regulation+of+B+cell+activation	0.0217552	#N/A	0.0217552
BP_GO:0006582_melanin+metabolic+process	0.41316	#N/A	0.41316
MF_GO:0016972_thiol+oxidase+activity	0.941344	0.657375	0.983852
BP_GO:0019642_anaerobic+glycolysis	0.294586	0.294586	#N/A
MF_GO:0004459_L-lactate+dehydrogenase+activity	0.294586	0.294586	#N/A
CC_GO:0005790_smooth+endoplasmic+reticulum	0.445496	0.734958	0.129802
MF_GO:0004667_prostaglandin-D+synthase+activity	0.0138313	#N/A	0.0138313
BP_GO:0014068_positive+regulation+of+phosphoinositide+3-kinase+cascad	0.949641	0.799638	0.951094
MF_GO:0042166_acetylcholine+binding	0.32903	0.416102	0.319633
BP_GO:0006527_arginine+catabolic+process	0.0969913	0.0969913	#N/A
BP_GO:0030323_respiratory+tube+development	0.3821	0.0709499	0.748422
MF_GO:0004528_phosphodiesterase+I+activity	0.098801	#N/A	0.098801
BP_GO:0046685_response+to+arsenic	0.692889	0.230051	0.872592
CC_GO:0005779_integral+to+peroxisomal+membrane	0.506467	0.186421	0.741756
BP_GO:0035088_establishment+or+maintenance+of+apical/basal+cell+pola	0.252792	0.0363835	0.674163
BP_GO:0030321_transepithelial+chloride+transport	0.487726	0.483215	0.496494
BP_GO:0046339_diacylglycerol+metabolic+process	0.191132	0.556867	0.0428532
MF_GO:0004370_glycerol+kinase+activity	0.292322	0.0458065	0.699
BP_GO:0060044_negative+regulation+of+cardiac+muscle+cell+proliferation	0.934804	0.724965	0.923649
BP_GO:0009263_deoxyribonucleotide+biosynthetic+process	0.400668	0.69332	0.252651
MF_GO:0004748_ribonucleoside-diphosphate+reductase+activity	0.400668	0.69332	0.252651
BP_GO:0051450_myoblast+proliferation	0.972713	0.782186	0.96434
MF_GO:0008384_IkappaB+kinase+activity	0.974471	#N/A	0.974471
BP_GO:0010273_detoxification+of+copper+ion	0.929025	0.480048	0.966661
MF_GO:0016671_oxidoreductase+activity,+acting+on+sulfur+group+of+don	0.978514	0.984369	0.677334
BP_GO:0050872_white+fat+cell+differentiation	0.542032	0.542032	#N/A
BP_GO:0048340_paraxial+mesoderm+morphogenesis	0.16037	0.370819	0.162455
MF_GO:0003988_acetyl-CoA+C-acyltransferase+activity	0.417288	0.417288	#N/A
BP_GO:0006620_posttranslational+protein+targeting+to+membrane	0.449128	0.770153	0.102194
CC_GO:0005782_peroxisomal+matrix	0.610282	0.610282	#N/A
MF_GO:0003865_3-oxo-5-alpha-steroid+4-dehydrogenase+activity	0.0394123	0.347582	0.00631646
MF_GO:0047184_1-acylglycerophosphocholine+O-acyltransferase+activity	0.0395172	0.0349204	0.315333
BP_GO:0002862_negative+regulation+of+inflammatory+response+to+antige	0.6879	0.6879	#N/A
MF_GO:0016986_transcription+initiation+factor+activity	0.177395	0.177395	#N/A
BP_GO:0032400_melanosome+localization	0.069072	0.0903739	0.19169
CC_GO:0000940_outer+kinetochore+of+condensed+chromosome	0.550756	#N/A	0.550756
BP_GO:0019724_B+cell+mediated+immunity	0.00928818	0.0872652	0.0270889
BP_GO:0051891_positive+regulation+of+cardioblast+differentiation	0.164499	0.164499	#N/A
BP_GO:0046325_negative+regulation+of+glucose+import	0.853077	0.786713	0.756082
BP_GO:0045662_negative+regulation+of+myoblast+differentiation	0.800262	0.800262	#N/A
MF_GO:0015238_drug+transporter+activity	0.29641	0.412389	0.269266
BP_GO:0048545_response+to+steroid+hormone+stimulus	0.965981	#N/A	0.965981
MF_GO:0019797_procollagen-proline+3-dioxygenase+activity	0.0205271	0.0205271	#N/A
BP_GO:0006560_proline+metabolic+process	0.734348	0.734348	#N/A

MF_GO:0004700_atypical+protein+kinase+C+activity	0.41115	0.131019	0.697271
MF_GO:0004346_glucose-6-phosphatase+activity	0.661608	0.661608	#N/A
BP_GO:0045475_locomotor+rhythm	0.0765007	0.458632	0.046648
BP_GO:0051496_positive+regulation+of+stress+fiber+formation	0.618298	0.665517	0.466398
BP_GO:0031558_induction+of+apoptosis+in+response+to+chemical+stimulu	0.0337011	0.782178	0.0026143
BP_GO:0021860_pyramidal+neuron+development	0.684334	0.409544	0.87585
BP_GO:0006972_hyperosmotic+response	0.874626	0.918915	0.662251
BP_GO:0018401_peptidyl-proline+hydroxylation+to+4-hydroxy-L-proline	0.872982	0.872982	#N/A
MF_GO:0004663_Rab-protein+geranylgeranyltransferase+activity	0.0841382	0.294537	0.0955781
MF_GO:0004069_aspartate+transaminase+activity	0.0668545	0.0668545	#N/A
MF_GO:0008898_homocysteine+S-methyltransferase+activity	0.732454	#N/A	0.732454
MF_GO:0016866_intramolecular+transferase+activity	0.0840437	0.0293978	0.361567
MF_GO:0016286_small+conductance+calcium-activated+potassium+channe	0.583593	0.630039	0.458215
CC_GO:0005747_mitochondrial+respiratory+chain+complex+I	0.721954	0.721954	#N/A
MF_GO:0019976_interleukin-2+binding	0.36665	0.446571	0.343892
BP_GO:0009100_glycoprotein+metabolic+process	0.76194	0.826057	0.462517
BP_GO:0010575_positive+regulation+vascular+endothelial+growth+factor+p	0.746042	0.62156	0.76074
BP_GO:0006686_sphingomyelin+biosynthetic+process	0.640849	0.438528	0.800443
BP_GO:0043306_positive+regulation+of+mast+cell+degranulation	0.102454	0.35476	0.0983873
BP_GO:0030049_muscle+filament+sliding	0.082449	0.082449	#N/A
CC_GO:0016461_unconventional+myosin+complex	0.082449	0.082449	#N/A
BP_GO:0007195_dopamine+receptor,+adenylate+cyclase+inhibiting+pathwa	0.231534	#N/A	0.231534
BP_GO:0001996_positive+regulation+of+heart+rate+by+epinephrine-norepi	0.7935	0.948726	0.439783
BP_GO:0001997_positive+regulation+of+the+force+of+heart+contraction+b	0.7935	0.948726	0.439783
MF_GO:0016155_formyltetrahydrofolate+dehydrogenase+activity	0.281938	0.241698	0.415804
BP_GO:0016559_peroxisome+fission	0.238114	0.752287	0.0138666
BP_GO:0000320_re-entry+into+mitotic+cell+cycle	0.384349	0.180069	0.612376
MF_GO:0045502_dynein+binding	0.557948	0.563138	0.510635
BP_GO:0007213_acetylcholine+receptor+signaling,+muscarinic+pathway	0.192651	0.156615	0.36246
BP_GO:0016117_carotenoid+biosynthetic+process	0.873151	0.859218	0.675094
MF_GO:0019209_kinase+activator+activity	0.94864	0.94864	#N/A
BP_GO:0045216_cell-cell+junction+organization	0.957226	0.958756	0.699392
BP_GO:0001844_protein+insertion+into+mitochondrial+membrane+during+	0.167818	0.236603	0.250271
BP_GO:0007095_mitotic+cell+cycle+G2/M+transition+DNA+damage+checkp	0.227202	0.622651	0.12752
BP_GO:0043068_positive+regulation+of+programmed+cell+death	0.959638	0.959638	#N/A
BP_GO:0045617_negative+regulation+of+keratinocyte+differentiation	0.805643	#N/A	0.805643
MF_GO:0017076_purine+nucleotide+binding	0.7599	0.705226	0.685424
BP_GO:0019884_antigen+processing+and+presentation+of+exogenous+anti	0.652249	0.182892	0.974844
BP_GO:0035234_germ+cell+programmed+cell+death	0.12219	0.236603	0.178721
BP_GO:0007016_cytoskeletal+anchoring+at+plasma+membrane	0.0490415	0.0490415	#N/A
BP_GO:0045022_early+endosome+to+late+endosome+transport	0.805892	0.733409	0.731055
BP_GO:0032469_endoplasmic+reticulum+calcium+ion+homeostasis	0.9171	0.208082	0.988447
MF_GO:0051020_GTPase+binding	0.676296	#N/A	0.676296
BP_GO:0048268_clathrin+coat+assembly	0.984666	0.783576	0.981827
MF_GO:0008502_melatonin+receptor+activity	0.592409	0.792957	0.226125
MF_GO:0004712_protein+serine/threonine/tyrosine+kinase+activity	0.541717	0.329621	0.789442
BP_GO:0001553_luteinization	0.0317542	0.0317542	#N/A

MF_GO:0019208_phosphatase+regulator+activity	0.868887	#N/A	0.868887
BP_GO:0007090_regulation+of+S+phase+of+mitotic+cell+cycle	0.184759	0.184759	#N/A
MF_GO:0030371_translation+repressor+activity	0.908117	0.489518	0.990377
BP_GO:0006929_substrate-bound+cell+migration	0.271699	0.362219	0.289135
MF_GO:0032947_protein+complex+scaffold	0.929448	#N/A	0.929448
MF_GO:0016151_nickel+ion+binding	0.192714	0.767843	0.0569009
CC_GO:0042598_vesicular+fraction	0.435079	#N/A	0.435079
BP_GO:0007034_vacuolar+transport	0.94874	0.823216	0.910541
CC_GO:0030904_retromer+complex	0.94874	0.823216	0.910541
MF_GO:0003896_DNA+primase+activity	0.407631	#N/A	0.407631
BP_GO:0001812_positive+regulation+of+type+I+hypersensitivity	0.0109668	#N/A	0.0109668
CC_GO:0030118_clathrin+coat	0.858782	#N/A	0.858782
MF_GO:0000293_ferric-chelate+reductase+activity	0.266165	0.0282821	0.719521
CC_GO:0001891_phagocytic+cup	0.497172	0.497172	#N/A
BP_GO:0048488_synaptic+vesicle+endocytosis	0.186728	0.659705	0.0834571
BP_GO:0009448_gamma-aminobutyric+acid+metabolic+process	0.526243	0.526243	#N/A
MF_GO:0016817_hydrolase+activity,+acting+on+acid+anhydrides	0.672173	#N/A	0.672173
BP_GO:0001675_acrosome+formation	0.712773	0.723713	0.60544
BP_GO:0042989_sequestering+of+actin+monomers	0.65947	0.65947	#N/A
BP_GO:0009181_purine+ribonucleoside+diphosphate+catabolic+process	0.656875	0.543818	0.661414
MF_GO:0008454_alpha-1,3-mannosylglycoprotein+4-beta-N-acetylglucosam	0.486903	0.616393	0.316676
BP_GO:0030502_negative+regulation+of+bone+mineralization	0.315196	0.0582405	0.917113
MF_GO:0000309_nicotinamide-nucleotide+adenylyltransferase+activity	0.980657	0.992872	0.545961
MF_GO:0019871_sodium+channel+inhibitor+activity	0.885235	0.9081	0.579747
MF_GO:0004597_peptide-aspartate+beta-dioxygenase+activity	0.628612	0.225615	0.824751
BP_GO:0030510_regulation+of+BMP+signaling+pathway	0.476253	0.509123	0.464158
BP_GO:0002320_lymphoid+progenitor+cell+differentiation	0.693603	#N/A	0.693603
BP_GO:0008090_retrograde+axon+cargo+transport	0.453789	0.73636	0.136307
BP_GO:0033209_tumor+necrosis+factor-mediated+signaling+pathway	0.12492	0.12492	#N/A
BP_GO:0051090_regulation+of+transcription+factor+activity	0.232404	0.139605	0.603641
MF_GO:0005094_Rho+GDP-dissociation+inhibitor+activity	0.475214	0.694335	0.203805
BP_GO:0060009_Sertoli+cell+development	0.884766	0.90452	0.589918
BP_GO:0030518_steroid+hormone+receptor+signaling+pathway	0.840093	0.843026	0.617423
MF_GO:0004849_uridine+kinase+activity	0.126108	0.395693	0.0534725
MF_GO:0004558_alpha-glucosidase+activity	0.723087	0.84679	0.336503
MF_GO:0001882_nucleoside+binding	0.001013	0.00427403	0.0514212
BP_GO:0033143_regulation+of+steroid+hormone+receptor+signaling+pathv	0.724352	0.80999	0.543336
CC_GO:0030136_clathrin-coated+vesicle	0.965439	0.783576	0.952729
BP_GO:0045055_regulated+secretory+pathway	0.493458	0.731886	0.182758
MF_GO:0043560_insulin+receptor+substrate+binding	0.620085	#N/A	0.620085
BP_GO:0030091_protein+repair	0.0293274	0.0293274	#N/A
MF_GO:0000318_protein-methionine-R-oxide+reductase+activity	0.0293274	0.0293274	#N/A
BP_GO:0001953_negative+regulation+of+cell-matrix+adhesion	0.322186	0.14295	0.574453
BP_GO:0030224_monocyte+differentiation	0.463544	0.495806	0.442372
BP_GO:0006670_sphingosine+metabolic+process	0.263769	0.814949	0.00901689
MF_GO:0050508_glucuronosyl-N-acetylglucosaminyl-proteoglycan+4-alpha-	0.484173	0.484173	#N/A
MF_GO:0042799_histone+lysine+N-methyltransferase+activity+(H4-K20+spe	0.445671	0.323762	0.561543

MF_GO:0004385_guanylate+kinase+activity	0.357075	0.583878	0.274488
BP_GO:0040012_regulation+of+locomotion	0.237762	#N/A	0.237762
BP_GO:0002636_positive+regulation+of+germinal+center+formation	0.557496	0.514656	0.578315
BP_GO:0001842_neural+fold+formation	0.368277	0.368277	#N/A
MF_GO:0015027_coreceptor,+soluble+ligand+activity	0.319019	0.319019	#N/A
BP_GO:0032147_activation+of+protein+kinase+activity	0.79726	0.582383	0.808237
MF_GO:0016505_apoptotic+protease+activator+activity	0.131218	0.131218	#N/A
MF_GO:0003726_double-stranded+RNA+adenosine+deaminase+activity	0.422184	0.191236	0.814438
BP_GO:0018125_peptidyl-cysteine+methylation	0.972562	0.585615	0.986046
MF_GO:0005548_phospholipid+transporter+activity	0.0775306	0.111574	0.228872
BP_GO:0006855_multidrug+transport	0.532583	0.289625	0.688347
MF_GO:0047631_ADP-ribose+diphosphatase+activity	0.184578	0.672338	0.0783045
MF_GO:0004322_ferroxidase+activity	0.570357	0.531104	0.577603
BP_GO:0060056_mammary+gland+involution	0.0473072	0.03507	0.368229
MF_GO:0008409_5'-3'+exonuclease+activity	0.434896	0.640769	0.213141
BP_GO:0048820_hair+follicle+maturation	0.15174	0.0544882	0.685416
BP_GO:0044249_cellular+biosynthetic+process	0.766881	0.766881	#N/A
BP_GO:0006542_glutamine+biosynthetic+process	0.824213	0.824213	#N/A
MF_GO:0004356_glutamate-ammonia+ligase+activity	0.824213	0.824213	#N/A
MF_GO:0004430_1-phosphatidylinositol+4-kinase+activity	0.868225	0.83665	0.708633
BP_GO:0007089_traversing+start+control+point+of+mitotic+cell+cycle	0.401181	0.730607	0.229097
BP_GO:0007252_I-kappaB+phosphorylation	0.81222	#N/A	0.81222
BP_GO:0016584_nucleosome+positioning	0.399528	0.871265	0.132869
MF_GO:0005031_tumor+necrosis+factor+receptor+activity	0.343213	0.634283	0.117645
CC_GO:0031512_motile+primary+cilium	0.472666	0.472666	#N/A
MF_GO:0035197_siRNA+binding	0.135065	0.466412	0.0364143
BP_GO:0007144_female+meiosis+I	0.277624	0.363205	0.298462
MF_GO:0016149_translation+release+factor+activity,+codon+specific	0.765189	#N/A	0.765189
BP_GO:0006808_regulation+of+nitrogen+utilization	0.524671	0.376108	0.709835
BP_GO:0006290_pyrimidine+dimer+repair	0.487014	0.175702	0.896364
BP_GO:0044265_cellular+macromolecule+catabolic+process	0.127438	0.090886	0.465874
BP_GO:0030513_positive+regulation+of+BMP+signaling+pathway	0.854495	#N/A	0.854495
MF_GO:0008173_RNA+methyltransferase+activity	0.482194	0.326841	0.60315
BP_GO:0006891_intra-Golgi+vesicle-mediated+transport	0.590415	0.446609	0.720782
MF_GO:0005528_FK506+binding	0.145502	0.40609	0.0673631
BP_GO:0048302_regulation+of+isotype+switching+to+IgG+isotypes	0.222851	0.222851	#N/A
BP_GO:0001757_somite+specification	0.0503029	#N/A	0.0503029
BP_GO:0006166_purine+ribonucleoside+salvage	0.0236755	0.0236755	#N/A
MF_GO:0003886_DNA+(cytosine-5-)-methyltransferase+activity	0.357047	0.357047	#N/A
MF_GO:0005229_intracellular+calcium+activated+chloride+channel+activity	0.0471069	0.497584	0.0203272
BP_GO:0040037_negative+regulation+of+fibroblast+growth+factor+recepto	0.260732	0.771383	0.094823
BP_GO:0006000_fructose+metabolic+process	0.667597	0.338343	0.909907
BP_GO:0006003_fructose+2,6-bisphosphate+metabolic+process	0.667597	0.338343	0.909907
MF_GO:0003873_6-phosphofructo-2-kinase+activity	0.667597	0.338343	0.909907
MF_GO:0004331_fructose-2,6-bisphosphate+2-phosphatase+activity	0.667597	0.338343	0.909907
BP_GO:0046549_retinal+cone+cell+development	0.10137	0.170502	0.1871
MF_GO:0004965_GABA-B+receptor+activity	0.771431	0.42981	0.849707

BP_GO:0051155_positive+regulation+of+striated+muscle+cell+differentiatio	0.678909	0.678909	#N/A
MF_GO:0048038_quinone+binding	0.882073	0.918834	0.530616
MF_GO:0008568_microtubule-severing+ATPase+activity	0.708031	0.636802	0.674785
BP_GO:0031000_response+to+caffeine	0.0590951	0.0590951	#N/A
BP_GO:0001994_norepinephrine-epinephrine+vasoconstriction+involved+in	0.188935	0.0451121	0.546155
BP_GO:0043367_CD4-positive,+alpha+beta+T+cell+differentiation	0.980587	#N/A	0.980587
MF_GO:0003918_DNA+topoisomerase+(ATP-hydrolyzing)+activity	0.852873	#N/A	0.852873
BP_GO:0034101_erythrocyte+homeostasis	0.57287	0.0763819	0.990377
BP_GO:0021522_spinal+cord+motor+neuron+differentiation	0.0553601	0.0778765	0.17057
BP_GO:0050806_positive+regulation+of+synaptic+transmission	0.413315	0.708443	0.255585
BP_GO:0048664_neuron+fate+determination	0.806455	0.719254	0.750725
MF_GO:0005220_inositol+1,4,5-triphosphate-sensitive+calcium-release+cha	0.286971	0.208082	0.454166
MF_GO:0008095_inositol-1,4,5-triphosphate+receptor+activity	0.286971	0.208082	0.454166
BP_GO:0045446_endothelial+cell+differentiation	0.204835	0.477321	0.165826
MF_GO:0016891_endoribonuclease+activity,+producing+5'-phosphomonoes	0.642481	0.600478	0.60511
CC_GO:0008537_proteasome+activator+complex	0.338958	0.338958	#N/A
MF_GO:0008538_proteasome+activator+activity	0.338958	0.338958	#N/A
MF_GO:0004616_phosphogluconate+dehydrogenase+(decarboxylating)+act	0.993807	0.993807	#N/A
BP_GO:0030643_cellular+phosphate+ion+homeostasis	0.875255	0.780715	0.80586
BP_GO:0007603_phototransduction,+visible+light	0.38288	0.905115	0.0981014
BP_GO:0006491_N-glycan+processing	0.913413	0.941986	0.55422
BP_GO:0048294_negative+regulation+of+isotype+switching+to+IgE+isotype:	0.118657	0.118657	#N/A
BP_GO:0042698_ovulation+cycle	0.584427	0.774119	0.393076
MF_GO:0004802_transketolase+activity	0.506216	0.71676	0.349447
BP_GO:0032402_melanosome+transport	0.122312	0.21624	0.19169
BP_GO:0009607_response+to+biotic+stimulus	0.936601	0.798989	0.899323
MF_GO:0008073_ornithine+decarboxylase+inhibitor+activity	0.0518141	0.264407	0.0266953
BP_GO:0006435_threonyl-tRNA+aminoacylation	0.391435	0.339371	0.542658
MF_GO:0004829_threonine-tRNA+ligase+activity	0.391435	0.339371	0.542658
BP_GO:0040023_establishment+of+nucleus+localization	0.179376	0.474351	0.140116
BP_GO:0046903_secretion	0.605362	0.605362	#N/A
BP_GO:0007220_Notch+receptor+processing	0.877141	#N/A	0.877141
MF_GO:0003953_NAD++nucleosidase+activity	0.462239	0.199969	0.683616
BP_GO:0009612_response+to+mechanical+stimulus	0.796425	0.821223	0.553326
BP_GO:0031116_positive+regulation+of+microtubule+polymerization	0.801496	0.849828	0.619828
CC_GO:0042405_nuclear+inclusion+body	0.681286	0.58352	0.665521
BP_GO:0007412_axon+target+recognition	0.71809	0.170192	0.916215
CC_GO:0017133_mitochondrial+electron+transfer+flavoprotein+complex	0.716119	0.716119	#N/A
MF_GO:0004800_thyroxine+5'-deiodinase+activity	0.587289	0.49054	0.612631
MF_GO:0004758_serine+C-palmitoyltransferase+activity	0.309671	0.798781	0.114847
MF_GO:0004566_beta-glucuronidase+activity	0.0820099	0.257982	0.0674723
BP_GO:0021895_cerebral+cortex+neuron+differentiation	0.883028	0.883028	#N/A
BP_GO:0007215_glutamate+signaling+pathway	0.935139	0.935139	#N/A
BP_GO:0045747_positive+regulation+of+Notch+signaling+pathway	0.340137	0.307106	0.499065
BP_GO:0045629_negative+regulation+of+T-helper+2+cell+differentiation	0.230322	0.230322	#N/A
BP_GO:0015695_organic+cation+transport	0.0632804	0.159385	0.121363
MF_GO:0003724_RNA+helicase+activity	0.940377	0.943454	0.676469

BP_GO:0045217_cell-cell+junction+maintenance	0.498864	0.498864	#N/A
BP_GO:0007000_nucleolus+organization	0.990685	0.990685	#N/A
CC_GO:0000109_nucleotide-excision+repair+complex	0.198747	0.0479798	0.55524
BP_GO:0030644_cellular+chloride+ion+homeostasis	0.0538541	0.242629	0.0696323
CC_GO:0032421_stereocilium+bundle	0.265443	0.855447	0.0645431
CC_GO:0000802_transverse+filament	0.956265	0.713735	0.954882
BP_GO:0050779_RNA+destabilization	0.126906	0.571168	0.0635418
BP_GO:0045884_regulation+of+survival+gene+product+expression	0.658786	0.658786	#N/A
BP_GO:0048676_axon+extension+involved+in+development	0.912378	0.908474	0.763982
BP_GO:0031623_receptor+internalization	0.539396	0.346563	0.766928
CC_GO:0030285_integral+to+synaptic+vesicle+membrane	0.446562	#N/A	0.446562
BP_GO:0035284_brain+segmentation	0.382775	0.421607	0.405819
CC_GO:0001741_XY+body	0.207141	0.17095	0.471428
BP_GO:0007199_G-protein+signaling,+coupled+to+cGMP+nucleotide+secon	0.69363	0.69363	#N/A
BP_GO:0010025_wax+biosynthetic+process	0.966945	0.927132	0.889283
BP_GO:0048678_response+to+axon+injury	0.175824	0.179534	0.375172
BP_GO:0030521_androgen+receptor+signaling+pathway	0.665112	0.524613	0.742436
MF_GO:0004705_JUN+kinase+activity	0.719558	0.782178	0.56387
BP_GO:0051493_regulation+of+cytoskeleton+organization	0.0350355	0.208146	0.0498993
BP_GO:0021575_hindbrain+morphogenesis	0.665572	0.665572	#N/A
BP_GO:0000087_M+phase+of+mitotic+cell+cycle	0.0143217	0.162979	0.0234111
MF_GO:0005104_fibroblast+growth+factor+receptor+binding	0.769586	0.780715	0.638845
BP_GO:0042326_negative+regulation+of+phosphorylation	0.341638	0.58352	0.257928
BP_GO:0048513_organ+development	0.857679	0.857679	#N/A
MF_GO:0008081_phosphoric+diester+hydrolase+activity	0.866551	0.866551	#N/A
BP_GO:0007256_activation+of+JNKK+activity	0.759592	#N/A	0.759592
MF_GO:0050811_GABA+receptor+binding	0.881247	0.476584	0.931617
BP_GO:0006467_protein+thiol-disulfide+exchange	0.951005	0.896913	0.871433
CC_GO:0031965_nuclear+membrane	0.713965	0.618185	0.709835
MF_GO:0004687_myosin+light+chain+kinase+activity	0.141339	0.141339	#N/A
BP_GO:0021571_rhombomere+5+development	0.0717073	#N/A	0.0717073
BP_GO:0006420_arginyl-tRNA+aminoacylation	0.271828	0.309194	0.347201
MF_GO:0004814_arginine-tRNA+ligase+activity	0.271828	0.309194	0.347201
BP_GO:0031529_ruffle+organization	0.77154	0.655959	0.734655
BP_GO:0051298_centrosome+duplication	0.809514	0.745566	0.727669
BP_GO:0006264_mitochondrial+DNA+replication	0.899292	0.899292	#N/A
MF_GO:0005172_vascular+endothelial+growth+factor+receptor+binding	0.574372	0.313515	0.844132
BP_GO:0045947_negative+regulation+of+translational+initiation	0.0924125	0.0924125	#N/A
BP_GO:0000380_alternative+nuclear+mRNA+splicing,+via+spliceosome	0.585993	0.639561	0.448243
BP_GO:0021819_layer+formation+in+the+cerebral+cortex	0.949506	0.902176	0.844046
BP_GO:0051457_maintenance+of+protein+location+in+nucleus	0.556852	0.452366	0.661342
BP_GO:0048557_embryonic+digestive+tract+morphogenesis	0.295954	0.664477	0.169094
MF_GO:0042162_telomeric+DNA+binding	0.49642	#N/A	0.49642
BP_GO:0045197_establishment+or+maintenance+of+epithelial+cell+apical/l	0.793571	#N/A	0.793571
BP_GO:0017158_regulation+of+calcium+ion-dependent+exocytosis	0.485176	0.894659	0.0331105
BP_GO:0031077_post-embryonic+camera-type+eye+development	0.184992	0.230011	0.281916
MF_GO:0005078_MAP-kinase+scaffold+activity	0.902884	0.902884	#N/A

BP_GO:0030072_peptide+hormone+secretion	0.47458	#N/A	0.47458
MF_GO:0004887_thyroid+hormone+receptor+activity	0.413251	0.655559	0.29037
BP_GO:0006120_mitochondrial+electron+transport,+NADH+to+ubiquinone	0.95906	0.95906	#N/A
MF_GO:0003708_retinoic+acid+receptor+activity	0.619313	0.619313	#N/A
MF_GO:0003910_DNA+ligase+(ATP)+activity	0.90177	0.98769	0.4975
BP_GO:0030219_megakaryocyte+differentiation	0.649378	0.495806	0.751393
BP_GO:0031398_positive+regulation+of+protein+ubiquitination	0.593896	0.616556	0.496506
BP_GO:0060218_hemopoietic+stem+cell+differentiation	0.877181	0.59032	0.954358
BP_GO:0042633_hair+cycle	0.445694	0.461404	0.459853
BP_GO:0051567_histone+H3-K9+methylation	0.21406	0.20752	0.412283
MF_GO:0008413_8-oxo-7,8-dihydroguanine+triphosphatase+activity	0.935058	0.935058	#N/A
BP_GO:0008595_determination+of+anterior/posterior+axis,+embryo	0.434999	0.428838	0.470257
BP_GO:0048645_organ+formation	0.710396	0.36495	0.926318
MF_GO:0004739_pyruvate+dehydrogenase+(acetyl-transferring)+activity	0.671724	0.975434	0.021985
BP_GO:0050856_regulation+of+T+cell+receptor+signaling+pathway	0.306908	0.0959076	0.834213
CC_GO:0031932_TORC2+complex	0.569193	0.655959	0.471724
MF_GO:0016160_amylase+activity	0.545754	#N/A	0.545754
MF_GO:0005275_amine+transmembrane+transporter+activity	0.0314535	0.159385	0.0576257
MF_GO:0004946_bombesin+receptor+activity	0.54276	#N/A	0.54276
BP_GO:0021800_cerebral+cortex+tangential+migration	0.255718	0.484701	0.218225
MF_GO:0019237_centromeric+DNA+binding	0.547697	0.686559	0.421716
BP_GO:0006997_nucleus+organization	0.631588	0.985237	0.129868
CC_GO:0031931_TORC1+complex	0.417745	0.655959	0.29496
BP_GO:0031167_rRNA+methylation	0.752839	0.752839	#N/A
BP_GO:0006323_DNA+packaging	0.221019	0.129746	0.441997
BP_GO:0001831_trophectodermal+cellular+morphogenesis	0.942364	0.854856	0.891217
BP_GO:0002455_humoral+immune+response+mediated+by+circulating+imr	0.668045	#N/A	0.668045
MF_GO:0001537_N-acetylgalactosamine+4-O-sulfotransferase+activity	0.523149	0.797748	0.140032
MF_GO:0051019_mitogen-activated+protein+kinase+binding	0.0170707	0.0989601	0.0320017
BP_GO:0048012_hepatocyte+growth+factor+receptor+signaling+pathway	0.94796	#N/A	0.94796
CC_GO:0009331_glycerol-3-phosphate+dehydrogenase+complex	0.760431	0.760431	#N/A
BP_GO:0032609_interferon-gamma+production	0.804723	0.804723	#N/A
BP_GO:0015809_arginine+transport	0.465679	0.750925	0.133686
MF_GO:0015181_arginine+transmembrane+transporter+activity	0.465679	0.750925	0.133686
BP_GO:0000086_G2/M+transition+of+mitotic+cell+cycle	0.226861	0.30607	0.287567
BP_GO:0048661_positive+regulation+of+smooth+muscle+cell+proliferation	0.896368	0.910975	0.610085
BP_GO:0050767_regulation+of+neurogenesis	0.109656	0.147168	0.258829
BP_GO:0030538_embryonic+genitalia+morphogenesis	0.681751	#N/A	0.681751
BP_GO:0048853_forebrain+morphogenesis	0.0315426	#N/A	0.0315426
BP_GO:0006704_glucocorticoid+biosynthetic+process	0.143367	0.143367	#N/A
BP_GO:0046470_phosphatidylcholine+metabolic+process	0.599391	0.599391	#N/A
BP_GO:0033153_T+cell+receptor+V(D)J+recombination	0.391657	#N/A	0.391657
BP_GO:0042523_positive+regulation+of+tyrosine+phosphorylation+of+Stat5	0.963669	#N/A	0.963669
BP_GO:0060078_regulation+of+postsynaptic+membrane+potential	0.393964	0.68464	0.125514
MF_GO:0008107_galactoside+2-alpha-L-fucosyltransferase+activity	0.134445	#N/A	0.134445
BP_GO:0060084_synaptic+transmission+involved+in+micturition	0.473658	0.322692	0.596152
BP_GO:0051963_regulation+of+synaptogenesis	0.362054	0.322692	0.456954

BP_GO:0032228_regulation+of+synaptic+transmission,+GABAergic	0.408537	0.464252	0.412614
BP_GO:0008594_photoreceptor+cell+morphogenesis	0.0304172	0.588991	0.00701114
MF_GO:0017069_snRNA+binding	0.779292	0.938861	0.196763
CC_GO:0005672_transcription+factor+TFIIA+complex	0.560535	0.687986	0.333345
BP_GO:0060083_smooth+muscle+contraction+involved+in+micturition	0.513847	0.98092	0.0773306
BP_GO:0042637_catagen	0.404817	0.42434	0.435988
BP_GO:0051101_regulation+of+DNA+binding	0.307302	0.328065	0.403586
BP_GO:0000212_meiotic+spindle+organization	0.551853	0.723117	0.2699
CC_GO:0044445_cytosolic+part	0.646473	0.646473	#N/A
BP_GO:0045807_positive+regulation+of+endocytosis	0.712579	0.538671	0.731656
BP_GO:0001574_ganglioside+biosynthetic+process	0.514016	0.34713	0.73106
BP_GO:0030903_notochord+development	0.340122	#N/A	0.340122
BP_GO:0006623_protein+targeting+to+vacuole	0.97912	0.725712	0.996317
MF_GO:0004938_alpha2-adrenergic+receptor+activity	0.0765329	0.153558	0.150756
BP_GO:0048563_post-embryonic+organ+morphogenesis	0.78057	0.78057	#N/A
MF_GO:0005098_Ran+GTPase+activator+activity	0.697507	0.827471	0.329771
BP_GO:0050871_positive+regulation+of+B+cell+activation	0.69049	#N/A	0.69049
MF_GO:0008656_caspase+activator+activity	0.0222615	0.0222615	#N/A
BP_GO:0048712_negative+regulation+of+astrocyte+differentiation	0.855417	0.830466	0.685714
BP_GO:0006426_glycyl-tRNA+aminoacylation	0.134022	0.134022	#N/A
MF_GO:0004820_glycine-tRNA+ligase+activity	0.134022	0.134022	#N/A
BP_GO:0000002_mitochondrial+genome+maintenance	0.831888	0.747444	0.765173
BP_GO:0017000_antibiotic+biosynthetic+process	0.800892	0.704227	0.759359
BP_GO:0042471_ear+morphogenesis	0.799187	0.829782	0.541408
BP_GO:0007132_meiotic+metaphase+I	0.825208	0.825208	#N/A
MF_GO:0008020_G-protein+coupled+photoreceptor+activity	0.0985115	0.182883	0.169013
BP_GO:0007217_tachykinin+signaling+pathway	0.593385	0.730902	0.441832
BP_GO:0001706_endoderm+formation	0.84512	0.84512	#N/A
BP_GO:0060164_regulation+of+timing+of+neuron+differentiation	0.374633	0.374633	#N/A
MF_GO:0005227_calcium+activated+cation+channel+activity	0.753796	0.770254	0.624594
BP_GO:0010553_negative+regulation+of+specific+transcription+from+RNA+	0.088328	#N/A	0.088328
CC_GO:0034706_sodium+channel+complex	0.00163876	#N/A	0.00163876
BP_GO:0050672_negative+regulation+of+lymphocyte+proliferation	0.38076	0.129141	0.858228
MF_GO:0015321_sodium-dependent+phosphate+transmembrane+transpor	0.0845486	0.0623124	0.274095
BP_GO:0006207_'de+novo'+pyrimidine+base+biosynthetic+process	0.586485	0.544832	0.586414
MF_GO:0015267_channel+activity	0.519752	#N/A	0.519752
CC_GO:0008023_transcription+elongation+factor+complex	0.228268	0.124836	0.460322
BP_GO:0051965_positive+regulation+of+synaptogenesis	0.202049	0.202049	#N/A
MF_GO:0031403_lithium+ion+binding	0.583403	0.299007	0.866529
BP_GO:0010825_positive+regulation+of+centrosome+duplication	0.0571315	#N/A	0.0571315
BP_GO:0006388_tRNA+splicing,+via+endonucleolytic+cleavage+and+ligatior	0.159192	0.0879628	0.573026
MF_GO:0008241_peptidyl-dipeptidase+activity	0.191886	0.28601	0.251863
BP_GO:0007442_hindgut+morphogenesis	0.191737	#N/A	0.191737
BP_GO:0030718_germ-line+stem+cell+maintenance	0.710865	0.718037	0.607396
BP_GO:0042421_norepinephrine+biosynthetic+process	0.886364	#N/A	0.886364
MF_GO:0050780_dopamine+receptor+binding	0.739018	0.871036	0.311395
MF_GO:0005519_cytoskeletal+regulatory+protein+binding	0.935145	#N/A	0.935145

BP_GO:0021913_regulation+of+transcription+from+RNA+polymerase+II+prc	0.26419	0.447124	0.248452
BP_GO:0001710_mesodermal+cell+fate+commitment	0.861144	0.680516	0.888192
BP_GO:0001976_regulation+of+systemic+arterial+blood+pressure+by+neurc	0.457068	0.42284	0.501836
MF_GO:0004305_ethanolamine+kinase+activity	0.63865	0.666567	0.501969
BP_GO:0042832_defense+response+to+protozoan	0.428608	#N/A	0.428608
BP_GO:0021543_pallium+development	0.135897	0.31174	0.158569
BP_GO:0031642_negative+regulation+of+myelination	0.615349	0.235465	0.807297
BP_GO:0031503_protein+complex+localization	0.884337	0.901869	0.596928
MF_GO:0001602_pancreatic+polypeptide+receptor+activity	0.937878	0.666574	0.942761
BP_GO:0042448_progesterone+metabolic+process	0.00661514	0.288837	0.00411051
MF_GO:0004461_lactose+synthase+activity	0.243747	0.123457	0.486724
MF_GO:0015087_cobalt+ion+transmembrane+transporter+activity	0.613488	0.902258	0.286984
MF_GO:0032137_guanine/thymine+mispair+binding	0.165684	0.153876	0.319102
BP_GO:0045089_positive+regulation+of+innate+immune+response	0.221103	#N/A	0.221103
BP_GO:0043586_tongue+development	0.245355	0.253119	0.353641
BP_GO:0032651_regulation+of+interleukin-1+beta+production	0.828362	0.688228	0.828287
BP_GO:0002666_positive+regulation+of+T+cell+tolerance+induction	0.0425518	0.0280916	0.388143
BP_GO:0016080_synaptic+vesicle+targeting	0.982706	#N/A	0.982706
CC_GO:0032040_small-subunit+processome	0.659581	#N/A	0.659581
BP_GO:0006883_cellular+sodium+ion+homeostasis	0.373851	#N/A	0.373851
MF_GO:0001733_galactosylceramide+sulfotransferase+activity	0.266701	0.527801	0.208051
BP_GO:0042711_maternal+behavior	0.885826	0.52963	0.922569
BP_GO:0001894_tissue+homeostasis	0.385364	0.618504	0.284008
MF_GO:0004035_alkaline+phosphatase+activity	0.362184	0.141633	0.817502
MF_GO:0051879_Hsp90+protein+binding	0.637089	0.902887	0.109305
BP_GO:0035067_negative+regulation+of+histone+acetylation	0.168053	0.168053	#N/A
BP_GO:0021570_rhombomere+4+development	0.0554678	#N/A	0.0554678
BP_GO:0006405_RNA+export+from+nucleus	0.401249	0.109001	0.713356
BP_GO:0016242_negative+regulation+of+macroautophagy	0.351818	0.357684	0.442467
BP_GO:0046649_lymphocyte+activation	0.0777996	0.183265	0.117925
MF_GO:0003917_DNA+topoisomerase+type+I+activity	0.824501	#N/A	0.824501
MF_GO:0017025_TATA-binding+protein+binding	0.514284	0.514284	#N/A
CC_GO:0042765_GPI-anchor+transamidase+complex	0.314964	0.583678	0.2295
BP_GO:0048755_branching+morphogenesis+of+a+nerve	0.632337	0.734626	0.488187
CC_GO:0044442_microtubule-based+flagellum+part	0.136713	0.44945	0.105119
MF_GO:0016615_malate+dehydrogenase+activity	0.140437	0.399968	0.0652359
MF_GO:0004719_protein-L-isoaspartate+(D-aspartate)+O-methyltransferase	0.753276	0.430958	0.831762
BP_GO:0009247_glycolipid+biosynthetic+process	0.174981	0.5143	0.120734
BP_GO:0006024_glycosaminoglycan+biosynthetic+process	0.696265	0.9262	0.346527
BP_GO:0045859_regulation+of+protein+kinase+activity	0.880634	0.880634	#N/A
BP_GO:0006177_GMP+biosynthetic+process	0.616368	0.584855	0.583293
BP_GO:0006112_energy+reserve+metabolic+process	0.456404	0.456404	#N/A
MF_GO:0008934_inositol-1(or+4)-monophosphatase+activity	0.424932	0.424932	#N/A
BP_GO:0021529_spinal+cord+oligodendrocyte+cell+differentiation	0.345418	0.461736	0.28951
BP_GO:0021530_spinal+cord+oligodendrocyte+cell+fate+specification	0.345418	0.461736	0.28951
CC_GO:0001520_outer+dense+fiber	0.0548933	0.0419456	0.230055
BP_GO:0015872_dopamine+transport	0.241952	#N/A	0.241952

BP_GO:0006360_transcription+from+RNA+polymerase+I+promoter	0.570304	0.85634	0.115117
BP_GO:0032715_negative+regulation+of+interleukin-6+production	0.252513	0.322206	0.307195
BP_GO:0042274_ribosomal+small+subunit+biogenesis	0.308564	0.498545	0.270678
MF_GO:0048487_beta-tubulin+binding	0.0726185	0.0726185	#N/A
BP_GO:0051603_proteolysis+involved+in+cellular+protein+catabolic+proces	0.387496	0.0903022	0.918933
BP_GO:0060039_pericardium+development	0.213601	#N/A	0.213601
MF_GO:0008900_hydrogen:potassium-exchanging+ATPase+activity	0.38237	0.385737	0.456592
MF_GO:0004966_galanin+receptor+activity	0.156651	0.0385976	0.505006
BP_GO:0060291_long-term+synaptic+potentiation	0.338198	0.42284	0.353949
BP_GO:0045749_negative+regulation+of+S+phase+of+mitotic+cell+cycle	0.133982	0.0668251	0.579515
BP_GO:0009048_dosage+compensation,+by+inactivation+of+X+chromosom	0.411959	0.0884084	0.752051
MF_GO:0005549_odorant+binding	0.906257	#N/A	0.906257
MF_GO:0015026_coreceptor+activity	0.165078	0.385996	0.161289
CC_GO:0001674_female+germ+cell+nucleus	0.439619	0.597912	0.358605
CC_GO:0005655_nucleolar+ribonuclease+P+complex	0.484025	0.325261	0.715828
BP_GO:0006266_DNA+ligation	0.288821	0.4632	0.268357
MF_GO:0004995_tachykinin+receptor+activity	0.610756	#N/A	0.610756
CC_GO:0048786_presynaptic+active+zone	0.195322	0.195322	#N/A
BP_GO:0021555_midbrain-hindbrain+boundary+morphogenesis	0.373849	0.373849	#N/A
BP_GO:0045578_negative+regulation+of+B+cell+differentiation	0.134226	#N/A	0.134226
BP_GO:0042147_retrograde+transport,+endosome+to+Golgi	0.952364	0.892004	0.872936
MF_GO:0003964_RNA-directed+DNA+polymerase+activity	0.481028	0.449383	0.538389
BP_GO:0031133_regulation+of+axon+diameter	0.443744	0.443744	#N/A
MF_GO:0016527_brain-specific+angiogenesis+inhibitor+activity	0.277887	0.277887	#N/A
BP_GO:0016539_intein-mediated+protein+splicing	0.575461	#N/A	0.575461
BP_GO:0006481_C-terminal+protein+amino+acid+methylation	0.77597	0.77597	#N/A
BP_GO:0010824_regulation+of+centrosome+duplication	0.0635411	0.299455	0.067005
MF_GO:0004865_type+1+serine/threonine+specific+protein+phosphatase+i	0.797485	0.60206	0.798585
BP_GO:0008589_regulation+of+smoothened+signaling+pathway	0.903	#N/A	0.903
MF_GO:0015277_kainate+selective+glutamate+receptor+activity	0.219425	0.429505	0.137411
BP_GO:0007262_STAT+protein+nuclear+translocation	0.884267	0.185177	0.982059
BP_GO:0009743_response+to+carbohydrate+stimulus	0.657886	0.924764	0.0916291
BP_GO:0045110_intermediate+filament+bundle+assembly	0.517302	0.517302	#N/A
MF_GO:0004406_H3/H4+histone+acetyltransferase+activity	0.618024	0.688245	0.508065
MF_GO:0050501_hyaluronan+synthase+activity	0.392667	0.528311	0.350217
BP_GO:0045085_negative+regulation+of+interleukin-2+biosynthetic+proces	0.489816	0.329582	0.610097
BP_GO:0050710_negative+regulation+of+cytokine+secretion	0.295307	0.546396	0.228955
BP_GO:0042668_auditory+receptor+cell+fate+determination	0.132403	0.529657	0.020681
BP_GO:0021985_neurohypophysis+development	0.405398	0.362906	0.531897
BP_GO:0006283_transcription-coupled+nucleotide-excision+repair	0.143878	0.0077231	0.658369
BP_GO:0045953_negative+regulation+of+natural+killer+cell+mediated+cyto	0.0804902	0.0804902	#N/A
BP_GO:0050955_thermoception	0.610084	#N/A	0.610084
MF_GO:0008327_methyl-CpG+binding	0.937524	0.466412	0.997268
BP_GO:0001992_regulation+of+systemic+arterial+blood+pressure+by+vasop	0.622363	0.374889	0.727887
BP_GO:0050968_detection+of+chemical+stimulus+involved+in+sensory+per	0.543486	0.846325	0.278148
MF_GO:0035035_histone+acetyltransferase+binding	0.121986	0.0668251	0.540308
MF_GO:0048029_monosaccharide+binding	0.291045	0.10474	0.583841

BP_GO:0048747_muscle+fiber+development	0.934106	0.911434	0.758649
MF_GO:0008745_N-acetylmuramoyl-L-alanine+amidase+activity	0.898482	0.864563	0.741438
BP_GO:0002309_T+cell+proliferation+during+immune+response	0.588345	#N/A	0.588345
CC_GO:0030127_COPII+vesicle+coat	0.727711	0.200432	0.987396
BP_GO:0050917_sensory+perception+of+umami+taste	0.395266	0.743203	0.215382
BP_GO:0021615_glossopharyngeal+nerve+morphogenesis	0.724249	0.355583	0.940071
MF_GO:0015018_galactosylgalactosylxylosylprotein+3-beta-glucuronosyltra	0.281449	0.615419	0.179559
BP_GO:0014002_astrocyte+development	0.625705	0.721007	0.491173
MF_GO:0008061_chitin+binding	0.738305	0.506247	0.779335
BP_GO:0045348_positive+regulation+of+MHC+class+II+biosynthetic+proces	0.904809	#N/A	0.904809
BP_GO:0007041_lysosomal+transport	0.2735	0.262237	0.442467
BP_GO:0045900_negative+regulation+of+translational+elongation	0.743859	0.686746	0.672317
BP_GO:0006505_GPI+anchor+metabolic+process	0.38184	0.876769	0.11738
MF_GO:0016654_oxidoreductase+activity,+acting+on+NADH+or+NADPH,+di	0.814752	0.906655	0.375608
BP_GO:0006422_aspartyl-tRNA+aminoacylation	0.381299	#N/A	0.381299
MF_GO:0004815_aspartate-tRNA+ligase+activity	0.381299	#N/A	0.381299
BP_GO:0043631_RNA+polyadenylation	0.300501	0.155176	0.530018
BP_GO:0021869_forebrain+ventricular+zone+progenitor+cell+division	0.348375	0.296371	0.531897
BP_GO:0043584_nose+development	0.105892	#N/A	0.105892
BP_GO:0051205_protein+insertion+into+membrane	0.525867	0.567068	0.44919
BP_GO:0016050_vesicle+organization	0.0231966	0.0231966	#N/A
BP_GO:0043270_positive+regulation+of+ion+transport	0.665385	0.665385	#N/A
CC_GO:0033269_internode+region+of+axon	0.805618	0.816408	0.585915
MF_GO:0032549_ribonucleoside+binding	0.987165	#N/A	0.987165
BP_GO:0030101_natural+killer+cell+activation	0.888995	0.117581	0.99021
MF_GO:0030304_trypsin+inhibitor+activity	0.0541345	0.00835888	0.390641
CC_GO:0005577_fibrinogen+complex	0.711959	0.711959	#N/A
BP_GO:0006603_phosphocreatine+metabolic+process	0.140667	0.140667	#N/A
MF_GO:0042132_fructose+1,6-bisphosphate+1-phosphatase+activity	0.189457	0.108242	0.495908
BP_GO:0043627_response+to+estrogen+stimulus	0.0107077	0.177055	0.00990313
CC_GO:0005862_muscle+thin+filament+tropomyosin	0.720025	0.720025	#N/A
MF_GO:0017159_pantetheine+hydrolase+activity	0.464216	0.529909	0.419509
MF_GO:0047131_saccharopine+dehydrogenase+(NAD+,+L-glutamate-formi	0.539243	0.954671	0.0601498
MF_GO:0003960_NADPH:quinone+reductase+activity	0.674464	0.626379	0.624281
BP_GO:0001998_angiotensin+mediated+vasoconstriction+involved+in+regu	0.265458	0.773994	0.0505222
MF_GO:0008184_glycogen+phosphorylase+activity	0.321618	0.321618	#N/A
BP_GO:0019322_pentose+biosynthetic+process	0.127276	0.510551	0.0505222
MF_GO:0042134_rRNA+primary+transcript+binding	0.66046	0.66046	#N/A
MF_GO:0030296_protein+tyrosine+kinase+activator+activity	0.0682396	#N/A	0.0682396
MF_GO:0017067_tyrosine-ester+sulfotransferase+activity	0.978449	0.832085	0.971218
MF_GO:0043395_heparan+sulfate+proteoglycan+binding	0.733319	#N/A	0.733319
MF_GO:0046592_polyamine+oxidase+activity	0.281407	0.281407	#N/A
BP_GO:0048807_female+genitalia+morphogenesis	0.822624	#N/A	0.822624
MF_GO:0004942_anaphylatoxin+receptor+activity	0.00458105	#N/A	0.00458105
MF_GO:0004607_phosphatidylcholine-sterol+O-acyltransferase+activity	0.623865	0.623865	#N/A
CC_GO:0005924_cell-substrate+adherens+junction	0.323822	#N/A	0.323822
BP_GO:0001973_adenosine+receptor+signaling+pathway	0.00106156	#N/A	0.00106156

MF_GO:0015645_fatty-acid+ligase+activity	0.312283	#N/A	0.312283
BP_GO:0048193_Golgi+vesicle+transport	0.61075	0.5847	0.572641
MF_GO:0003986_acetyl-CoA+hydrolase+activity	0.00748117	0.00748117	#N/A
BP_GO:0032613_interleukin-10+production	0.034774	0.176328	0.0505222
BP_GO:0032615_interleukin-12+production	0.034774	0.176328	0.0505222
MF_GO:0004743_pyruvate+kinase+activity	0.829758	0.307136	0.96798
BP_GO:0042730_fibrinolysis	0.387429	0.292909	0.555327
MF_GO:0000247_C-8+sterol+isomerase+activity	0.0138689	0.025809	0.121125
BP_GO:0002072_optic+cup+morphogenesis+involved+in+camera-type+eye+	0.0648177	#N/A	0.0648177
BP_GO:0046208_spermine+catabolic+process	0.784896	0.69817	0.724465
BP_GO:0043490_malate-aspartate+shuttle	0.201512	0.35915	0.205066
MF_GO:0004063_aryldialkylphosphatase+activity	0.760349	0.760349	#N/A
BP_GO:0045639_positive+regulation+of+myeloid+cell+differentiation	0.0990689	0.0990689	#N/A
MF_GO:0047750_cholestenol+delta-isomerase+activity	0.016968	0.016968	#N/A
BP_GO:0042308_negative+regulation+of+protein+import+into+nucleus	0.188369	0.559571	0.0804935
MF_GO:0004373_glycogen+(starch)+synthase+activity	0.901619	0.901619	#N/A
MF_GO:0016768_spermine+synthase+activity	0.0757946	#N/A	0.0757946
MF_GO:0004772_sterol+O-acyltransferase+activity	0.0574439	0.0574439	#N/A
MF_GO:0034736_cholesterol+O-acyltransferase+activity	0.0574439	0.0574439	#N/A
MF_GO:0034737_ergosterol+O-acyltransferase+activity	0.0574439	0.0574439	#N/A
MF_GO:0034738_lanosterol+O-acyltransferase+activity	0.0574439	0.0574439	#N/A
BP_GO:0019509_methionine+salvage	0.375717	0.61588	0.22846
BP_GO:0001935_endothelial+cell+proliferation	0.197944	0.197944	#N/A
MF_GO:0019955_cytokine+binding	0.21026	#N/A	0.21026
BP_GO:0006544_glycine+metabolic+process	0.245118	0.125179	0.56833
MF_GO:0019003_GDP+binding	0.230652	0.229727	0.380643
MF_GO:0008170_N-methyltransferase+activity	0.341647	0.341647	#N/A
BP_GO:0006907_pinocytosis	0.1799	#N/A	0.1799
BP_GO:0006529_asparagine+biosynthetic+process	0.781818	#N/A	0.781818
MF_GO:0004066_asparagine+synthase+(glutamine-hydrolyzing)+activity	0.781818	#N/A	0.781818
BP_GO:0016554_cytidine+to+uridine+editing	0.598473	#N/A	0.598473
MF_GO:0004014_adenosylmethionine+decarboxylase+activity	0.623276	#N/A	0.623276
BP_GO:0006778_porphyrin+metabolic+process	0.569954	#N/A	0.569954
MF_GO:0043185_vascular+endothelial+growth+factor+receptor+3+binding	0.0806056	#N/A	0.0806056
BP_GO:0030497_fatty+acid+elongation	0.0108102	0.074817	0.0350928
MF_GO:0003920_GMP+reductase+activity	0.577208	0.662595	0.442372
MF_GO:0003858_3-hydroxybutyrate+dehydrogenase+activity	0.0573787	0.118547	0.146715
MF_GO:0001846_opsonin+binding	0.173813	0.032883	0.694941
MF_GO:0004478_methionine+adenosyltransferase+activity	0.907177	#N/A	0.907177
BP_GO:0045019_negative+regulation+of+nitric+oxide+biosynthetic+process	0.285922	0.285922	#N/A
BP_GO:0007527_adult+somatic+muscle+development	0.0405102	0.0541574	0.193706
MF_GO:0008453_alanine-glyoxylate+transaminase+activity	0.364853	0.364853	#N/A
BP_GO:0014909_smooth+muscle+cell+migration	0.102967	0.102967	#N/A
MF_GO:0004074_biliverdin+reductase+activity	0.88661	0.807989	0.799252
BP_GO:0006569_tryptophan+catabolic+process	0.338216	#N/A	0.338216
BP_GO:0043462_regulation+of+ATPase+activity	0.598997	0.598997	#N/A
BP_GO:0019452_L-cysteine+catabolic+process+to+taurine	0.765982	#N/A	0.765982

MF_GO:0004359_glutaminase+activity	0.996557	0.991159	0.926594
BP_GO:0048554_positive+regulation+of+metalloenzyme+activity	0.564235	0.177055	0.87585
MF_GO:0015114_phosphate+transmembrane+transporter+activity	0.514542	0.547114	0.47298
BP_GO:0001561_fatty+acid+alpha-oxidation	0.304237	0.065254	0.784121
BP_GO:0019853_L-ascorbic+acid+biosynthetic+process	0.628651	0.551999	0.630374
BP_GO:0002074_extraocular+skeletal+muscle+development	0.494335	0.494335	#N/A
MF_GO:0005547_phosphatidylinositol-3,4,5-triphosphate+binding	0.217487	0.55727	0.105674
BP_GO:0019344_cysteine+biosynthetic+process	0.456679	0.456679	#N/A
CC_GO:0005927_muscle+tendon+junction	0.061925	0.061925	#N/A
BP_GO:0048260_positive+regulation+of+receptor-mediated+endocytosis	0.676525	0.783576	0.445437
MF_GO:0018685_alkane+1-monooxygenase+activity	0.930389	#N/A	0.930389
MF_GO:0047756_chondroitin+4-sulfotransferase+activity	0.0714436	#N/A	0.0714436
MF_GO:0004465_lipoprotein+lipase+activity	0.649345	0.649345	#N/A
BP_GO:0002329_pre-B+cell+differentiation	0.212932	0.885344	0.00990313
MF_GO:0005099_Ras+GTPase+activator+activity	0.727952	#N/A	0.727952
MF_GO:0004421_hydroxymethylglutaryl-CoA+synthase+activity	0.161418	0.569825	0.0575124
MF_GO:0004476_mannose-6-phosphate+isomerase+activity	0.295711	0.563775	0.178482
BP_GO:0018119_peptidyl-cysteine+S-nitrosylation	0.804371	0.305573	0.957346
BP_GO:0032959_inositol+trisphosphate+biosynthetic+process	0.0696043	0.0294304	0.419019
BP_GO:0045730_respiratory+burst	0.545379	0.993618	0.00990313
BP_GO:0015808_L-alanine+transport	0.0199483	0.137026	0.0347558
BP_GO:0015824_proline+transport	0.0199483	0.137026	0.0347558
MF_GO:0005280_hydrogen:amino+acid+symporter+activity	0.0199483	0.137026	0.0347558
MF_GO:0005302_L-tyrosine+transmembrane+transporter+activity	0.0199483	0.137026	0.0347558
MF_GO:0015180_L-alanine+transmembrane+transporter+activity	0.0199483	0.137026	0.0347558
MF_GO:0015193_L-proline+transmembrane+transporter+activity	0.0199483	0.137026	0.0347558
BP_GO:0001543_ovarian+follicle+rupture	0.758171	0.773994	0.594112
BP_GO:0006097_glyoxylate+cycle	0.68479	0.762488	0.486255
BP_GO:0006102_isocitrate+metabolic+process	0.68479	0.762488	0.486255
MF_GO:0004450_isocitrate+dehydrogenase+(NADP+)+activity	0.68479	0.762488	0.486255
CC_GO:0005584_collagen+type+I	0.627005	#N/A	0.627005
BP_GO:0043507_positive+regulation+of+JNK+activity	0.338647	0.109007	0.73953
BP_GO:0051451_myoblast+migration	0.075342	0.221946	0.102204
MF_GO:0004776_succinate-CoA+ligase+(GDP-forming)+activity	0.94346	0.94346	#N/A
MF_GO:0003951_NAD++kinase+activity	0.50726	0.325531	0.683331
BP_GO:0006555_methionine+metabolic+process	0.105154	0.105154	#N/A
BP_GO:0002018_renin-angiotensin+regulation+of+aldosterone+production	0.598002	0.773994	0.343891
BP_GO:0002019_regulation+of+renal+output+by+angiotensin	0.598002	0.773994	0.343891
MF_GO:0004531_deoxyribonuclease+II+activity	0.292223	0.161334	0.584743
MF_GO:0004449_isocitrate+dehydrogenase+(NAD+)+activity	0.54932	0.820467	0.228815
BP_GO:0060346_bone+trabecula+formation	0.193791	0.0998118	0.523494
BP_GO:0001885_endothelial+cell+development	0.936425	0.782186	0.915914
BP_GO:0019439_aromatic+compound+catabolic+process	0.376031	0.498634	0.328284
MF_GO:0004301_epoxide+hydrolase+activity	0.376031	0.498634	0.328284
BP_GO:0006570_tyrosine+metabolic+process	0.671202	0.298661	0.87585
MF_GO:0047749_cholestanetriol+26-monooxygenase+activity	0.852926	0.723324	0.813487
MF_GO:0003963_RNA-3'-phosphate+cyclase+activity	0.510573	#N/A	0.510573

MF_GO:0004067_asparaginase+activity	0.621171	0.0911964	0.961559
BP_GO:0030206_chondroitin+sulfate+biosynthetic+process	0.0706155	#N/A	0.0706155
MF_GO:0018738_S-formylglutathione+hydrolase+activity	0.303723	#N/A	0.303723
MF_GO:0003973_(S)-2-hydroxy-acid+oxidase+activity	0.171617	#N/A	0.171617
BP_GO:0021520_spinal+cord+motor+neuron+cell+fate+specification	0.538402	#N/A	0.538402
BP_GO:0009081_branched+chain+family+amino+acid+metabolic+process	0.556056	0.745678	0.321877
BP_GO:0009082_branched+chain+family+amino+acid+biosynthetic+process	0.556056	0.745678	0.321877
MF_GO:0004084_branched-chain-amino-acid+transaminase+activity	0.556056	0.745678	0.321877
BP_GO:0001788_antibody-dependent+cellular+cytotoxicity	0.446779	#N/A	0.446779
MF_GO:0019770_IgG+receptor+activity	0.446779	#N/A	0.446779
BP_GO:0051900_regulation+of+mitochondrial+depolarization	0.768915	0.615618	0.772022
CC_GO:0017109_glutamate-cysteine+ligase+complex	0.768915	0.615618	0.772022
BP_GO:0046626_regulation+of+insulin+receptor+signaling+pathway	0.461901	0.745447	0.212845
MF_GO:0050051_leukotriene-B4+20-monooxygenase+activity	0.983197	#N/A	0.983197
BP_GO:0051926_negative+regulation+of+calcium+ion+transport	0.480355	0.0366143	0.957346
BP_GO:0022407_regulation+of+cell-cell+adhesion	0.0451115	0.472667	0.00990313
BP_GO:0006563_L-serine+metabolic+process	0.349609	0.125179	0.726313
BP_GO:0001768_establishment+of+T+cell+polarity	0.271999	0.271999	#N/A
MF_GO:0004019_adenylosuccinate+synthase+activity	0.828577	0.614129	0.853425
BP_GO:0050777_negative+regulation+of+immune+response	0.484893	0.733404	0.248966
MF_GO:0005161_platelet-derived+growth+factor+receptor+binding	0.43663	#N/A	0.43663
BP_GO:0046879_hormone+secretion	0.179133	0.348181	0.181194
MF_GO:0030215_semaphorin+receptor+binding	0.0075112	0.0555544	0.0322171
MF_GO:0004998_transferrin+receptor+activity	0.0630776	0.102065	0.185258
BP_GO:0042531_positive+regulation+of+tyrosine+phosphorylation+of+STAT	0.0945731	0.0945731	#N/A
BP_GO:0043506_regulation+of+JNK+activity	0.708595	0.708595	#N/A
MF_GO:0004586_ornithine+decarboxylase+activity	0.0632174	0.118328	0.163446
BP_GO:0033627_cell+adhesion+mediated+by+integrin	0.12937	0.12937	#N/A
BP_GO:0006518_peptide+metabolic+process	0.141521	0.553855	0.0489034
MF_GO:0003878_ATP+citrate+synthase+activity	0.124451	0.304784	0.130932
BP_GO:0042732_D-xylose+metabolic+process	0.34244	#N/A	0.34244
MF_GO:0004471_malate+dehydrogenase+(decarboxylating)+activity	0.0438162	#N/A	0.0438162
BP_GO:0055013_cardiac+muscle+cell+development	0.723773	0.723773	#N/A
BP_GO:0032099_negative+regulation+of+appetite	0.0218399	0.082869	0.0708453
MF_GO:0003883_CTP+synthase+activity	0.0890832	0.0890832	#N/A
BP_GO:0016558_protein+import+into+peroxisome+matrix	0.232206	0.232206	#N/A
BP_GO:0050901_leukocyte+tethering+or+rolling	0.120452	#N/A	0.120452
BP_GO:0009313_oligosaccharide+catabolic+process	0.118883	0.118883	#N/A
BP_GO:0006991_response+to+sterol+depletion	0.678856	0.435143	0.793785
BP_GO:0032796_uropod+organization	0.432876	0.514023	0.391504
MF_GO:0004372_glycine+hydroxymethyltransferase+activity	0.497839	0.497839	#N/A
BP_GO:0000083_regulation+of+transcription+of+G1/S-phase+of+mitotic+ce	0.451349	0.628827	0.307567
BP_GO:0051938_L-glutamate+import	0.193016	#N/A	0.193016
BP_GO:0043312_neutrophil+degranulation	0.338696	#N/A	0.338696
BP_GO:0048002_antigen+processing+and+presentation+of+peptide+antiger	0.991165	0.991165	#N/A
MF_GO:0008429_phosphatidylethanolamine+binding	0.611406	0.611406	#N/A
CC_GO:0031088_platelet+dense+granule+membrane	0.490254	0.089364	0.90475

BP_GO:0050714_positive+regulation+of+protein+secretion	0.422138	#N/A	0.422138
MF_GO:0008238_exopeptidase+activity	0.24365	0.625543	0.0961089
BP_GO:0042373_vitamin+K+metabolic+process	0.352171	#N/A	0.352171
CC_GO:0008247_1-alkyl-2-acetylglycerophosphocholine+esterase+complex	0.338447	0.907436	0.027693
BP_GO:0046329_negative+regulation+of+JNK+cascade	0.963235	0.990642	0.571531
MF_GO:0019166_trans-2-enoyl-CoA+reductase+(NADPH)+activity	0.100164	0.100164	#N/A
MF_GO:0016403_dimethylargininase+activity	0.347573	0.317413	0.467869
MF_GO:0004335_galactokinase+activity	0.86271	0.86271	#N/A
MF_GO:0030188_chaperone+regulator+activity	0.281822	#N/A	0.281822
CC_GO:0005964_phosphorylase+kinase+complex	0.353301	0.353301	#N/A
MF_GO:0004689_phosphorylase+kinase+activity	0.353301	0.353301	#N/A
BP_GO:0042994_cytoplasmic+sequestering+of+transcription+factor	0.793494	0.967291	0.246686
MF_GO:0050262_ribosylnicotinamide+kinase+activity	0.846127	0.820607	0.700103
BP_GO:0006739_NADP+metabolic+process	0.822602	0.961396	0.32324
BP_GO:0006111_regulation+of+gluconeogenesis	0.13208	0.082869	0.422643
MF_GO:0016620_oxidoreductase+activity,+acting+on+the+aldehyde+or+oxo	0.974396	0.737955	0.983042
BP_GO:0030865_cortical+cytoskeleton+organization	0.485407	0.485407	#N/A
BP_GO:0008210_estrogen+metabolic+process	0.335495	0.453097	0.314087
MF_GO:0030375_thyroid+hormone+receptor+coactivator+activity	0.575442	0.487833	0.617423
BP_GO:0006525_arginine+metabolic+process	0.741104	0.442136	0.855381
MF_GO:0004053_arginase+activity	0.741104	0.442136	0.855381
BP_GO:0006275_regulation+of+DNA+replication	0.682946	0.793501	0.441922
CC_GO:0031528_microvillus+membrane	0.149823	0.805441	0.00990313
BP_GO:0045619_regulation+of+lymphocyte+differentiation	0.0342821	0.293605	0.020901
BP_GO:0030573_bile+acid+catabolic+process	0.983794	#N/A	0.983794
MF_GO:0017130_poly(rC)+binding	0.844804	0.844804	#N/A
BP_GO:0043366_beta+selection	0.254579	0.254579	#N/A
MF_GO:0019902_phosphatase+binding	0.729847	#N/A	0.729847
BP_GO:0002537_production+of+nitric+oxide+during+acute+inflammatory+r	0.917283	0.917283	#N/A
CC_GO:0019867_outer+membrane	0.950214	0.950214	#N/A
BP_GO:0006532_aspartate+biosynthetic+process	0.303213	0.303213	#N/A
BP_GO:0019550_glutamate+catabolic+process+to+aspartate	0.303213	0.303213	#N/A
BP_GO:0019551_glutamate+catabolic+process+to+2-oxoglutarate	0.303213	0.303213	#N/A
BP_GO:0051725_protein+amino+acid+de-ADP-ribosylation	0.571415	0.571415	#N/A
MF_GO:0003875_ADP-ribosylarginine+hydrolase+activity	0.571415	0.571415	#N/A
BP_GO:0051583_dopamine+uptake	0.840889	#N/A	0.840889
BP_GO:0001845_phagolysosome+formation	0.763094	0.810184	0.553326
BP_GO:0001771_formation+of+immunological+synapse	0.669991	0.185436	0.935283
MF_GO:0030023_extracellular+matrix+constituent+conferring+elasticity	0.993494	#N/A	0.993494
BP_GO:0006198_cAMP+catabolic+process	0.899022	#N/A	0.899022
BP_GO:0014916_regulation+of+lung+blood+pressure	0.585271	0.593256	0.527059
MF_GO:0004962_endothelin+receptor+activity	0.470392	#N/A	0.470392
BP_GO:0051602_response+to+electrical+stimulus	0.33407	0.22912	0.553326
MF_GO:0047804_cysteine-S-conjugate+beta-lyase+activity	0.928406	0.928406	#N/A
BP_GO:0030836_positive+regulation+of+actin+filament+depolymerization	0.288946	0.288946	#N/A
BP_GO:0009264_deoxyribonucleotide+catabolic+process	0.390053	0.390053	#N/A
BP_GO:0046459_short-chain+fatty+acid+metabolic+process	0.9392	0.737955	0.939753

MF_GO:0003785_actin+monomer+binding	0.873041	0.471702	0.953957
MF_GO:0000900_translation+repressor+activity,+nucleic+acid+binding	0.888948	0.888948	#N/A
MF_GO:0042056_chemoattractant+activity	0.428673	0.452961	0.445437
BP_GO:0045723_positive+regulation+of+fatty+acid+biosynthetic+process	0.942149	0.942149	#N/A
BP_GO:0051024_positive+regulation+of+immunoglobulin+secretion	0.965507	#N/A	0.965507
MF_GO:0005159_insulin-like+growth+factor+receptor+binding	0.620241	0.634218	0.535516
BP_GO:0008582_regulation+of+synaptic+growth+at+neuromuscular+junctic	0.734365	0.559993	0.768531
MF_GO:0046912_transferase+activity,+transferring+acyl+groups,+acyl+grou	0.40693	0.784592	0.130932
BP_GO:0009156_ribonucleoside+monophosphate+biosynthetic+process	0.940633	0.940633	#N/A
BP_GO:0051291_protein+heterooligomerization	0.29116	0.29116	#N/A
MF_GO:0008124_4-alpha-hydroxytetrahydrobiopterin+dehydratase+activity	0.29116	0.29116	#N/A
BP_GO:0018346_protein+amino+acid+prenylation	0.192794	0.192794	#N/A
MF_GO:0008318_protein+prenyltransferase+activity	0.192794	0.192794	#N/A
BP_GO:0006068_ethanol+catabolic+process	0.253807	#N/A	0.253807
BP_GO:0042987_amyloid+precursor+protein+catabolic+process	0.671399	#N/A	0.671399
BP_GO:0045212_neurotransmitter+receptor+biosynthetic+process	0.330121	0.0882137	0.766928
BP_GO:0050650_chondroitin+sulfate+proteoglycan+biosynthetic+process	0.489253	#N/A	0.489253
BP_GO:0043280_positive+regulation+of+caspase+activity	0.346222	0.346222	#N/A
BP_GO:0019218_regulation+of+steroid+metabolic+process	0.0238505	0.0238505	#N/A
BP_GO:0046543_development+of+secondary+female+sexual+characteristics	0.0238505	0.0238505	#N/A
BP_GO:0046544_development+of+secondary+male+sexual+characteristics	0.0238505	0.0238505	#N/A
BP_GO:0000085_G2+phase+of+mitotic+cell+cycle	0.918023	0.561066	0.965241
MF_GO:0047237_glucuronylgalactosylproteoglycan+4-beta-N-acetylgalactos	0.422198	#N/A	0.422198
BP_GO:0002755_MyD88-dependent+toll-like+receptor+signaling+pathway	0.209349	0.175155	0.416281
MF_GO:0004447_iodide+peroxidase+activity	0.199314	#N/A	0.199314
BP_GO:0043619_regulation+of+transcription+from+RNA+polymerase+II+prc	0.166903	0.331926	0.175125
MF_GO:0004161_dimethylallyltranstransferase+activity	0.0115228	0.0688522	0.0415688
MF_GO:0004337_geranyltranstransferase+activity	0.0115228	0.0688522	0.0415688
MF_GO:0019103_pyrimidine+nucleotide+binding	0.222508	0.483215	0.149232
BP_GO:0001834_trophectodermal+cell+proliferation	0.894058	0.942636	0.574705
BP_GO:0001919_regulation+of+receptor+recycling	0.74886	0.586928	0.766928
MF_GO:0005068_transmembrane+receptor+protein+tyrosine+kinase+adapt	0.181177	0.181177	#N/A
BP_GO:0046426_negative+regulation+of+JAK-STAT+cascade	0.102995	0.102995	#N/A
BP_GO:0006086_acetyl-CoA+biosynthetic+process+from+pyruvate	0.541946	0.541946	#N/A
CC_GO:0030314_junctional+membrane+complex	0.0699861	0.202244	0.104564
BP_GO:0042511_positive+regulation+of+tyrosine+phosphorylation+of+Stat1	0.190632	0.0410961	0.690775
BP_GO:0007172_signal+complex+assembly	0.478589	#N/A	0.478589
MF_GO:0015272_ATP-activated+inward+rectifier+potassium+channel+activi	0.264817	0.820546	0.0353064
BP_GO:0031098_stress-activated+protein+kinase+signaling+pathway	0.150726	0.137141	0.355672
MF_GO:0005372_water+transporter+activity	0.779715	#N/A	0.779715
BP_GO:0035166_post-embryonic+hemopoiesis	0.720417	0.720417	#N/A
BP_GO:0032743_positive+regulation+of+interleukin-2+production	0.966056	0.966056	#N/A
BP_GO:0043372_positive+regulation+of+CD4-positive,+alpha+beta+T+cell+d	0.966056	0.966056	#N/A
BP_GO:0002033_vasodilation+by+angiotensin+involved+in+regulation+of+sy	0.0952134	#N/A	0.0952134
BP_GO:0043415_positive+regulation+of+skeletal+muscle+regeneration	0.311209	0.311209	#N/A
BP_GO:0006089_lactate+metabolic+process	0.864421	0.993618	0.175125
BP_GO:0035313_wound+healing,+spreading+of+epidermal+cells	0.636056	0.636056	#N/A

BP_GO:0002752_cell+surface+pattern+recognition+receptor+signaling+path	0.0250257	0.0250257	#N/A
BP_GO:0001879_detection+of+yeast	0.405095	0.405095	#N/A
BP_GO:0008064_regulation+of+actin+polymerization+or+depolymerization	0.455426	0.546155	0.391504
MF_GO:0003994_aconitate+hydratase+activity	0.891785	0.62372	0.924064
MF_GO:0009384_N-acylmannosamine+kinase+activity	0.538109	#N/A	0.538109
MF_GO:0004020_adenylylsulfate+kinase+activity	0.596446	#N/A	0.596446
MF_GO:0004781_sulfate+adenylyltransferase+(ATP)+activity	0.596446	#N/A	0.596446
BP_GO:0042094_interleukin-2+biosynthetic+process	0.666521	0.620946	0.617911
MF_GO:0004560_alpha-L-fucosidase+activity	0.778564	0.778564	#N/A
BP_GO:0001738_morphogenesis+of+a+polarized+epithelium	0.11114	0.0621855	0.424075
BP_GO:0008615_pyridoxine+biosynthetic+process	0.987013	0.987013	#N/A
BP_GO:0002347_response+to+tumor+cell	0.773046	#N/A	0.773046
BP_GO:0033194_response+to+hydroperoxide	0.455524	0.782186	0.173942
MF_GO:0004419_hydroxymethylglutaryl-CoA+lyase+activity	0.916559	0.916559	#N/A
BP_GO:0030262_apoptotic+nuclear+changes	0.0467382	#N/A	0.0467382
BP_GO:0006537_glutamate+biosynthetic+process	0.828781	0.828781	#N/A
BP_GO:0006562_proline+catabolic+process	0.828781	0.828781	#N/A
MF_GO:0004657_proline+dehydrogenase+activity	0.828781	0.828781	#N/A
MF_GO:0004943_C3a+anaphylatoxin+receptor+activity	0.0488192	#N/A	0.0488192
BP_GO:0002863_positive+regulation+of+inflammatory+response+to+antiger	0.0015524	#N/A	0.0015524
BP_GO:0046855_inositol+phosphate+dephosphorylation	0.598582	0.456952	0.677399
BP_GO:0032092_positive+regulation+of+protein+binding	0.739028	#N/A	0.739028
BP_GO:0031953_negative+regulation+of+protein+amino+acid+autophospho	0.676321	0.60864	0.644515
BP_GO:0051563_smooth+endoplasmic+reticulum+calcium+ion+homeostasis	0.97668	0.561066	0.996102
MF_GO:0015464_acetylcholine+receptor+activity	0.371931	0.434047	0.383113
BP_GO:0045359_positive+regulation+of+interferon-beta+biosynthetic+proc	0.144772	#N/A	0.144772
MF_GO:0004777_succinate-semialdehyde+dehydrogenase+activity	0.991702	0.991702	#N/A
MF_GO:0016361_activin+receptor+activity,+type+I	0.226925	0.322522	0.274151
BP_GO:0048739_cardiac+muscle+fiber+development	0.654277	0.515156	0.699392
BP_GO:0006048_UDP-N-acetylglucosamine+biosynthetic+process	0.849866	0.849866	#N/A
MF_GO:0008476_protein-tyrosine+sulfotransferase+activity	0.4597	0.4597	#N/A
MF_GO:0004342_glucosamine-6-phosphate+deaminase+activity	0.474919	0.652907	0.314472
CC_GO:0031616_spindle+pole+centrosome	0.512033	0.745566	0.267985
MF_GO:0004008_copper-exporting+ATPase+activity	0.491582	#N/A	0.491582
MF_GO:0005007_fibroblast+growth+factor+receptor+activity	0.195497	0.195497	#N/A
BP_GO:0001878_response+to+yeast	0.00610323	0.00610323	#N/A
BP_GO:0006163_purine+nucleotide+metabolic+process	0.638852	0.638852	#N/A
BP_GO:0009258_10-formyltetrahydrofolate+catabolic+process	0.415804	#N/A	0.415804
BP_GO:0002268_follicular+dendritic+cell+differentiation	0.802812	#N/A	0.802812
BP_GO:0001302_replicative+cell+aging	0.0588765	0.0479798	0.291192
CC_GO:0031304_intrinsic+to+mitochondrial+inner+membrane	0.681818	0.518132	0.733223
MF_GO:0008396_oxysterol+7-alpha-hydroxylase+activity	0.683158	#N/A	0.683158
BP_GO:0009191_ribonucleoside+diphosphate+catabolic+process	0.13678	0.412611	0.091824
MF_GO:0030159_receptor+signaling+complex+scaffold+activity	0.335619	0.335619	#N/A
BP_GO:0031987_locomotion+during+locomotory+behavior	0.118221	0.318761	0.114052
BP_GO:0046070_dGTP+metabolic+process	0.466409	0.588124	0.36579
MF_GO:0033188_sphingomyelin+synthase+activity	0.644546	0.374594	0.800443

MF_GO:0047493_ceramide+cholinephosphotransferase+activity	0.644546	0.374594	0.800443
MF_GO:0019870_potassium+channel+inhibitor+activity	0.798318	0.869874	0.522221
CC_GO:0000939_inner+kinetochore+of+condensed+chromosome	0.65103	0.753978	0.444785
BP_GO:0043589_skin+morphogenesis	0.105969	0.0575157	0.424075
BP_GO:0042089_cytokine+biosynthetic+process	0.755636	0.192075	0.967772
BP_GO:0050851_antigen+receptor-mediated+signaling+pathway	0.164038	0.420904	0.117925
MF_GO:0015651_quaternary+ammonium+group+transmembrane+transpor	0.514364	0.776824	0.238371
BP_GO:0001505_regulation+of+neurotransmitter+levels	0.220791	0.220791	#N/A
MF_GO:0008273_calcium,+potassium:sodium+antiporter+activity	0.801595	#N/A	0.801595
BP_GO:0001826_inner+cell+mass+cell+differentiation	0.0145642	0.0145642	#N/A
MF_GO:0008670_2,4-dienoyl-CoA+reductase+(NADPH)+activity	0.225805	0.225805	#N/A
BP_GO:0010035_response+to+inorganic+substance	0.8975	#N/A	0.8975
MF_GO:0004686_elongation+factor-2+kinase+activity	0.585252	0.68927	0.424639
MF_GO:0004618_phosphoglycerate+kinase+activity	0.980074	#N/A	0.980074
BP_GO:0060166_olfactory+pit+development	0.0860539	0.39041	0.0489549
BP_GO:0045682_regulation+of+epidermis+development	0.116274	0.0871904	0.369881
BP_GO:0009268_response+to+pH	0.00156637	0.000338367	0.216796
MF_GO:0030507_spectrin+binding	0.315306	#N/A	0.315306
BP_GO:0031579_membrane+raft+organization	0.830315	0.434353	0.935283
BP_GO:0002244_hemopoietic+progenitor+cell+differentiation	0.992775	0.787988	0.996102
BP_GO:0002286_T+cell+activation+during+immune+response	0.992775	0.787988	0.996102
BP_GO:0051491_positive+regulation+of+filopodium+formation	0.568971	0.568971	#N/A
BP_GO:0009957_epidermal+cell+fate+specification	0.17813	0.0367758	0.685416
BP_GO:0048733_sebaceous+gland+development	0.17813	0.0367758	0.685416
BP_GO:0042770_DNA+damage+response,+signal+transduction	0.0128676	0.0128676	#N/A
BP_GO:0009651_response+to+salt+stress	0.619837	0.773994	0.373961
CC_GO:0033267_axon+part	0.418094	0.451245	0.432072
BP_GO:0032703_negative+regulation+of+interleukin-2+production	0.0477183	0.0477183	#N/A
BP_GO:0002763_positive+regulation+of+myeloid+leukocyte+differentiation	0.131847	0.131847	#N/A
BP_GO:0051124_synaptic+growth+at+neuromuscular+junction	0.61817	0.561066	0.606736
BP_GO:0006002_fructose+6-phosphate+metabolic+process	0.52692	0.718291	0.314472
MF_GO:0030544_Hsp70+protein+binding	0.500183	0.702354	0.297522
CC_GO:0016460_myosin+II+complex	0.220411	0.220411	#N/A
MF_GO:0043531_ADP+binding	0.340367	0.340367	#N/A
MF_GO:0004692_cGMP-dependent+protein+kinase+activity	0.577877	0.372779	0.726253
MF_GO:0003688_DNA+replication+origin+binding	0.293486	0.264052	0.444785
CC_GO:0045335_phagocytic+vesicle	0.635556	#N/A	0.635556
MF_GO:0004797_thymidine+kinase+activity	0.543363	#N/A	0.543363
MF_GO:0005093_Rab+GDP-dissociation+inhibitor+activity	0.293854	0.537314	0.194369
MF_GO:0004315_3-oxoacyl-[acyl-carrier-protein]+synthase+activity	0.462672	0.462672	#N/A
BP_GO:0006654_phosphatidic+acid+biosynthetic+process	0.573534	#N/A	0.573534
BP_GO:0051290_protein+heterotetramerization	0.716088	0.500995	0.789564
MF_GO:0000010_trans-hexaprenyltranstransferase+activity	0.716088	0.500995	0.789564
MF_GO:0050347_trans-octaprenyltranstransferase+activity	0.716088	0.500995	0.789564
BP_GO:0015804_neutral+amino+acid+transport	0.597363	0.597363	#N/A
MF_GO:0033857_diphosphoinositol-pentakisphosphate+kinase+activity	0.0705036	0.0640052	0.287001
MF_GO:0019834_phospholipase+A2+inhibitor+activity	0.20916	#N/A	0.20916

MF_GO:0004609_phosphatidylserine+decarboxylase+activity	0.666979	0.666979	#N/A
BP_GO:0045921_positive+regulation+of+exocytosis	0.664648	0.603327	0.632588
CC_GO:0005753_mitochondrial+proton-transporting+ATP+synthase+comple	0.922693	0.922693	#N/A
BP_GO:0045634_regulation+of+melanocyte+differentiation	0.667475	0.667475	#N/A
BP_GO:0034613_cellular+protein+localization	0.861598	0.805441	0.750725
MF_GO:0043130_ubiquitin+binding	0.622388	0.170192	0.918278
MF_GO:0015108_chloride+transmembrane+transporter+activity	0.8249	0.66605	0.8138
MF_GO:0046922_peptide-O-fucosyltransferase+activity	0.353195	0.353195	#N/A
BP_GO:0007263_nitric+oxide+mediated+signal+transduction	0.837115	0.480048	0.925015
BP_GO:0060055_angiogenesis+involved+in+wound+healing	0.359538	0.782186	0.098591
BP_GO:0051925_regulation+of+calcium+ion+transport+via+voltage-gated+c	0.167641	0.434353	0.11522
BP_GO:0051135_positive+regulation+of+NK+T+cell+activation	0.109187	0.0245047	0.589269
MF_GO:0030548_acetylcholine+receptor+regulator+activity	0.352247	0.352247	#N/A
MF_GO:0030158_protein+xylosyltransferase+activity	0.252577	0.371845	0.268588
MF_GO:0045569_TRAIL+binding	0.00218475	0.00218475	#N/A
BP_GO:0035022_positive+regulation+of+Rac+protein+signal+transduction	0.243519	0.243519	#N/A
MF_GO:0003868_4-hydroxyphenylpyruvate+dioxygenase+activity	0.888994	#N/A	0.888994
BP_GO:0009186_deoxyribonucleoside+diphosphate+metabolic+process	0.332483	0.69332	0.13154
BP_GO:0015893_drug+transport	0.410022	0.594895	0.286688
BP_GO:0048087_positive+regulation+of+pigmentation+during+developmen	0.454049	0.236603	0.709835
BP_GO:0044257_cellular+protein+catabolic+process	0.143915	0.0778765	0.465874
MF_GO:0004021_alanine+transaminase+activity	0.154175	0.154175	#N/A
BP_GO:0030050_vesicle+transport+along+actin+filament	0.0506417	#N/A	0.0506417
MF_GO:0002020_protease+binding	0.201041	0.201041	#N/A
MF_GO:0005137_interleukin-5+receptor+binding	0.166535	0.18094	0.323118
MF_GO:0004967_glucagon+receptor+activity	0.617159	0.617159	#N/A
MF_GO:0047150_betaine-homocysteine+S-methyltransferase+activity	0.546379	#N/A	0.546379
MF_GO:0004850_uridine+phosphorylase+activity	0.42943	#N/A	0.42943
BP_GO:0014850_response+to+muscle+activity	0.350935	0.652245	0.175125
CC_GO:0043205_fibril	0.146078	0.163589	0.304359
MF_GO:0008109_N-acetyllactosaminide+beta-1,6-N-acetylglucosaminyltran	0.0571217	#N/A	0.0571217
BP_GO:0006649_phospholipid+transfer+to+membrane	0.333859	#N/A	0.333859
MF_GO:0016316_phosphatidylinositol-3,4-bisphosphate+4-phosphatase+act	0.374206	0.150637	0.718644
MF_GO:0034597_phosphatidyl-inositol-4,5-bisphosphate+4-phosphatase+ac	0.374206	0.150637	0.718644
CC_GO:0042175_nuclear+envelope-endoplasmic+reticulum+network	0.485582	0.258515	0.724335
BP_GO:0046022_positive+regulation+of+transcription+from+RNA+polymera	0.919512	0.957118	0.60432
BP_GO:0051014_actin+filament+severing	0.99858	0.99858	#N/A
MF_GO:0005069_transmembrane+receptor+protein+tyrosine+kinase+docki	0.0951795	0.0951795	#N/A
MF_GO:0017112_Rab+guanyl-nucleotide+exchange+factor+activity	0.120799	0.182668	0.225758
MF_GO:0004771_sterol+esterase+activity	0.258882	0.0433386	0.787239
BP_GO:0007194_negative+regulation+of+adenylate+cyclase+activity	0.537456	0.239301	0.799716
CC_GO:0005583_fibrillar+collagen	0.335613	0.577094	0.212973
BP_GO:0046827_positive+regulation+of+protein+export+from+nucleus	0.996113	0.991317	0.91726
BP_GO:0014902_myotube+differentiation	0.827184	0.782186	0.710212
BP_GO:0042488_positive+regulation+of+odontogenesis+of+dentine-contain	0.130096	0.322731	0.128337
BP_GO:0010766_negative+regulation+of+sodium+ion+transport	0.853751	0.901023	0.579747
BP_GO:0030916_otic+vesicle+formation	0.0327258	0.629611	0.00164774

MF_GO:0004925_prolactin+receptor+activity	0.47817	0.753662	0.222273
BP_GO:0001893_maternal+placenta+development	0.952638	0.986097	0.564998
MF_GO:0004516_nicotinate+phosphoribosyltransferase+activity	0.0633625	0.319966	0.045101
BP_GO:0000084_S+phase+of+mitotic+cell+cycle	0.836931	0.845634	0.644556
MF_GO:0003867_4-aminobutyrate+transaminase+activity	0.355564	0.355564	#N/A
MF_GO:0047298_(S)-3-amino-2-methylpropionate+transaminase+activity	0.355564	0.355564	#N/A
MF_GO:0047696_beta-adrenergic+receptor+kinase+activity	0.529909	0.282223	0.752202
MF_GO:0001729_ceramide+kinase+activity	0.364917	0.364917	#N/A
BP_GO:0008333_endosome+to+lysosome+transport	0.681261	0.586928	0.672272
MF_GO:0004660_protein+farnesyltransferase+activity	0.368224	0.368224	#N/A
BP_GO:0032230_positive+regulation+of+synaptic+transmission,+GABAergic	0.00117939	#N/A	0.00117939
BP_GO:0060081_membrane+hyperpolarization	0.770844	0.243507	0.959383
MF_GO:0030742_GTP-dependent+protein+binding	0.236442	0.0451746	0.750725
BP_GO:0007051_spindle+organization	0.963971	0.821203	0.947867
BP_GO:0031401_positive+regulation+of+protein+modification+process	0.554368	0.554368	#N/A
BP_GO:0045542_positive+regulation+of+cholesterol+biosynthetic+process	0.336167	0.596348	0.199492
CC_GO:0016528_sarcoplasm	0.986558	0.918071	0.958961
CC_GO:0044428_nuclear+part	0.213028	0.232958	0.34522
BP_GO:0035024_negative+regulation+of+Rho+protein+signal+transduction	0.00832191	0.00832191	#N/A
MF_GO:0004591_oxoglutarate+dehydrogenase+(succinyl-transferring)+activ	0.939965	0.939965	#N/A
MF_GO:0004127_cytidylate+kinase+activity	0.763092	0.763092	#N/A
BP_GO:0016199_axon+midline+choice+point+recognition	0.465737	0.465737	#N/A
CC_GO:0042587_glycogen+granule	0.871023	0.423218	0.963556
BP_GO:0031648_protein+destabilization	0.118434	0.184003	0.219262
MF_GO:0004605_phosphatidate+cytidylyltransferase+activity	0.886223	0.718682	0.870274
MF_GO:0016857_racemase+and+epimerase+activity,+acting+on+carbohydra	0.0518585	0.0224954	0.382535
BP_GO:0006702_androgen+biosynthetic+process	0.00750762	0.171391	0.00631646
MF_GO:0016705_oxidoreductase+activity,+acting+on+paired+donors,+with-	0.575735	#N/A	0.575735
MF_GO:0004109_coproporphyrinogen+oxidase+activity	0.234635	0.250764	0.362042
MF_GO:0004348_glucosylceramidase+activity	0.436154	0.833906	0.115424
BP_GO:0033700_phospholipid+efflux	0.111574	0.111574	#N/A
BP_GO:0006772_thiamin+metabolic+process	0.676561	0.676561	#N/A
BP_GO:0008065_establishment+of+blood-nerve+barrier	0.575711	0.773994	0.314592
MF_GO:0004142_diacylglycerol+cholinephosphotransferase+activity	0.841521	0.698794	0.8144
BP_GO:0016482_cytoplasmic+transport	0.39907	0.807413	0.109098
BP_GO:0051781_positive+regulation+of+cell+division	0.55354	0.640796	0.432072
MF_GO:0010302_2-oxoglutarate-dependent+dioxygenase+activity	0.313972	#N/A	0.313972
MF_GO:0051747_DNA+demethylase+activity	0.313972	#N/A	0.313972
BP_GO:0021831_embryonic+olfactory+bulb+interneuron+precursor+migrati	0.0478134	#N/A	0.0478134
MF_GO:0003938_IMP+dehydrogenase+activity	0.506524	0.584855	0.423795
MF_GO:0047276_N-acetyllactosaminide+3-alpha-galactosyltransferase+activ	0.48797	0.137	0.853189
BP_GO:0033364_mast+cell+secretory+granule+organization	0.0998651	#N/A	0.0998651
BP_GO:0051084_'de+novo'+posttranslational+protein+folding	0.79666	#N/A	0.79666
BP_GO:0008608_attachment+of+spindle+microtubules+to+kinetochore	0.479761	0.884379	0.102043
MF_GO:0008474_palmitoyl-(protein)+hydrolase+activity	0.14601	0.0797895	0.465874
MF_GO:0004958_prostaglandin+F+receptor+activity	0.695991	0.960241	0.151817
BP_GO:0002924_negative+regulation+of+humoral+immune+response+medi	0.0652158	0.0881766	0.214999

CC_GO:0030125_clathrin+vesicle+coat	0.883431	0.935074	0.568121
BP_GO:0007171_activation+of+transmembrane+receptor+protein+tyrosine-	0.662765	#N/A	0.662765
BP_GO:0060152_microtubule-based+peroxisome+localization	0.518169	0.234926	0.784121
MF_GO:0005025_transforming+growth+factor+beta+receptor+activity,+type	0.543099	0.774119	0.274151
BP_GO:0045409_negative+regulation+of+interleukin-6+biosynthetic+proces	0.150126	#N/A	0.150126
BP_GO:0043206_fibril+organization	0.289703	0.289703	#N/A
BP_GO:0007185_transmembrane+receptor+protein+tyrosine+phosphatase+	0.765892	#N/A	0.765892
BP_GO:0014065_phosphoinositide+3-kinase+cascade	0.790769	0.745447	0.685714
BP_GO:0050982_detection+of+mechanical+stimulus	0.306948	0.306948	#N/A
BP_GO:0048016_inositol+phosphate-mediated+signaling	0.717578	0.717578	#N/A
BP_GO:0016045_detection+of+bacterium	0.969132	0.969132	#N/A
MF_GO:0030144_alpha-1,6-mannosyl-glycoprotein+6-beta-N-acetylglucosar	0.157668	0.249961	0.227437
BP_GO:0002352_B+cell+negative+selection	0.248537	0.248537	#N/A
BP_GO:0010524_positive+regulation+of+calcium+ion+transport+into+cytosc	0.248537	0.248537	#N/A
BP_GO:0032471_reduction+of+endoplasmic+reticulum+calcium+ion+concer	0.248537	0.248537	#N/A
BP_GO:0033137_negative+regulation+of+peptidyl-serine+phosphorylation	0.248537	0.248537	#N/A
BP_GO:0033081_regulation+of+T+cell+differentiation+in+the+thymus	0.82487	0.818411	0.659657
MF_GO:0004833_tryptophan+2,3-dioxygenase+activity	0.00831092	0.0118393	0.129679
MF_GO:0004691_cAMP-dependent+protein+kinase+activity	0.83104	0.991317	0.152975
BP_GO:0009609_response+to+symbiotic+bacterium	0.563194	0.563194	#N/A
BP_GO:0051702_interaction+with+symbiont	0.563194	0.563194	#N/A
BP_GO:0043266_regulation+of+potassium+ion+transport	0.753665	0.961396	0.212655
BP_GO:0051642_centrosome+localization	0.0258735	0.0258735	#N/A
MF_GO:0004477_methenyltetrahydrofolate+cyclohydrolase+activity	0.446589	0.446589	#N/A
MF_GO:0004488_methylenetetrahydrofolate+dehydrogenase+(NADP+)+act	0.446589	0.446589	#N/A
MF_GO:0004727_prenylated+protein+tyrosine+phosphatase+activity	0.750773	#N/A	0.750773
BP_GO:0045401_positive+regulation+of+interleukin-3+biosynthetic+process	0.101924	0.35476	0.0768376
BP_GO:0045425_positive+regulation+of+granulocyte+macrophage+colony-s	0.101924	0.35476	0.0768376
BP_GO:0002260_lymphocyte+homeostasis	0.877181	0.86194	0.709835
BP_GO:0015884_folic+acid+transport	0.975855	#N/A	0.975855
BP_GO:0032226_positive+regulation+of+synaptic+transmission,+dopaminer	0.618732	0.618732	#N/A
BP_GO:0033603_positive+regulation+of+dopamine+secretion	0.618732	0.618732	#N/A
BP_GO:0030828_positive+regulation+of+cGMP+biosynthetic+process	0.164829	#N/A	0.164829
MF_GO:0004704_NF-kappaB-inducing+kinase+activity	0.854	0.854	#N/A
MF_GO:0003692_left-handed+Z-DNA+binding	0.518184	0.203029	0.814438
BP_GO:0031396_regulation+of+protein+ubiquitination	0.711102	0.711102	#N/A
MF_GO:0001614_purinergic+nucleotide+receptor+activity	0.082289	0.386558	0.0465257
BP_GO:0045010_actin+nucleation	0.20344	#N/A	0.20344
MF_GO:0042978_ornithine+decarboxylase+activator+activity	0.604653	0.118328	0.94038
BP_GO:0031296_B+cell+costimulation	0.707347	0.716875	0.578315
BP_GO:0035054_embryonic+heart+tube+anterior/posterior+pattern+forma	0.235323	0.235323	#N/A
MF_GO:0005128_erythropoietin+receptor+binding	0.197513	0.197513	#N/A
MF_GO:0004145_diamine+N-acetyltransferase+activity	0.897705	#N/A	0.897705
BP_GO:0060024_rhythmic+synaptic+transmission	0.409675	0.934826	0.0331105
MF_GO:0004611_phosphoenolpyruvate+carboxykinase+activity	0.770409	0.705226	0.693916
MF_GO:0004613_phosphoenolpyruvate+carboxykinase+(GTP)+activity	0.770409	0.705226	0.693916
BP_GO:0001954_positive+regulation+of+cell-matrix+adhesion	0.278712	0.322731	0.355262

MF_GO:0019807_aspartoacylase+activity	0.301812	0.215605	0.520489
BP_GO:0009590_detection+of+gravity	0.0152417	#N/A	0.0152417
BP_GO:0002227_innate+immune+response+in+mucosa	0.0260687	#N/A	0.0260687
BP_GO:0048318_axial+mesoderm+development	0.789106	0.789106	#N/A
BP_GO:0043547_positive+regulation+of+GTPase+activity	0.645506	0.745447	0.447063
CC_GO:0031091_platelet+alpha+granule	0.680016	#N/A	0.680016
BP_GO:0051128_regulation+of+cellular+component+organization	0.617733	0.117745	0.946215
BP_GO:0021591_ventricular+system+development	0.403009	0.42284	0.438851
MF_GO:0001888_glucuronyl-galactosyl-proteoglycan+4-alpha-N-acetylgluco	0.28029	0.28029	#N/A
BP_GO:0050794_regulation+of+cellular+process	0.269182	0.242629	0.430989
CC_GO:0005968_Rab-protein+geranylgeranyltransferase+complex	0.0955781	#N/A	0.0955781
BP_GO:0007033_vacuole+organization	0.561198	#N/A	0.561198
BP_GO:0006925_inflammatory+cell+apoptosis	0.138803	#N/A	0.138803
CC_GO:0000805_X+chromosome	0.223403	0.223403	#N/A
CC_GO:0000806_Y+chromosome	0.223403	0.223403	#N/A
MF_GO:0035005_phosphatidylinositol-4-phosphate+3-kinase+activity	0.51774	#N/A	0.51774
BP_GO:0051875_pigment+granule+localization	0.726029	0.810184	0.488312
BP_GO:0051904_pigment+granule+transport	0.726029	0.810184	0.488312
BP_GO:0001780_neutrophil+homeostasis	0.754574	0.993618	0.0647152
BP_GO:0006828_manganese+ion+transport	0.164987	0.329648	0.173942
MF_GO:0004765_shikimate+kinase+activity	0.364336	0.600566	0.227616
MF_GO:0050509_N-acetylglucosaminyl-proteoglycan+4-beta-glucuronosyltr	0.453669	0.453669	#N/A
BP_GO:0048478_replication+fork+protection	0.117709	0.070601	0.417318
MF_GO:0004576_oligosaccharyl+transferase+activity	0.707868	0.626584	0.673821
MF_GO:0050839_cell+adhesion+molecule+binding	0.117544	0.31854	0.113237
MF_GO:0004637_phosphoribosylamine-glycine+ligase+activity	0.951694	#N/A	0.951694
BP_GO:0007098_centrosome+cycle	0.844837	0.784694	0.741042
BP_GO:0014054_positive+regulation+of+gamma-aminobutyric+acid+secreti	0.249449	#N/A	0.249449
MF_GO:0003829_beta-1,3-galactosyl-O-glycosyl-glycoprotein+beta-1,6-N-ac	0.059129	#N/A	0.059129
BP_GO:0006788_heme+oxidation	0.557558	0.557558	#N/A
MF_GO:0004392_heme+oxygenase+(decyclizing)+activity	0.557558	0.557558	#N/A
BP_GO:0006534_cysteine+metabolic+process	0.278759	0.278759	#N/A
BP_GO:0050883_musculoskeletal+movement,+spinal+reflex+action	0.245586	0.805566	0.0331105
BP_GO:0002513_tolerance+induction+to+self+antigen	0.265087	0.265087	#N/A
BP_GO:0045066_regulatory+T+cell+differentiation	0.265087	0.265087	#N/A
BP_GO:0006029_proteoglycan+metabolic+process	0.635354	0.618504	0.574237
BP_GO:0051799_negative+regulation+of+hair+follicle+development	0.13878	0.343365	0.128337
MF_GO:0005026_transforming+growth+factor+beta+receptor+activity,+type	0.176655	0.151421	0.388143
MF_GO:0050321_tau-protein+kinase+activity	0.789653	0.401918	0.91726
BP_GO:0015860_purine+nucleoside+transport	0.000326435	0.00070285	0.0514212
MF_GO:0005415_nucleoside:sodium+symporter+activity	0.000326435	0.00070285	0.0514212
BP_GO:0045739_positive+regulation+of+DNA+repair	0.49981	#N/A	0.49981
BP_GO:0022010_myelination+in+the+central+nervous+system	0.378658	0.385756	0.441212
BP_GO:0033092_positive+regulation+of+immature+T+cell+proliferation+in+	0.671965	0.329582	0.857755
MF_GO:0003870_5-aminolevulinate+synthase+activity	0.853171	#N/A	0.853171
BP_GO:0048730_epidermis+morphogenesis	0.834137	0.230011	0.982631
MF_GO:0050544_arachidonic+acid+binding	0.446664	0.590216	0.337656

MF_GO:0030306_ADP-ribosylation+factor+binding	0.601506	0.601506	#N/A
BP_GO:0019230_proprioception	0.174805	0.42284	0.129234
MF_GO:0031386_protein+tag	0.806764	0.265544	0.967908
MF_GO:0004963_follicle-stimulating+hormone+receptor+activity	0.87599	0.510148	0.946113
BP_GO:0032831_positive+regulation+of+CD4-positive,+CD25-positive,+alpha	0.645606	0.645606	#N/A
BP_GO:0006625_protein+targeting+to+peroxisome	0.736247	0.736247	#N/A
MF_GO:0003827_alpha-1,3-mannosylglycoprotein+2-beta-N-acetylglucosam	0.954065	0.954065	#N/A
BP_GO:0019348_dolichol+metabolic+process	0.919071	0.925278	0.704386
CC_GO:0033185_dolichol-phosphate-mannose+synthase+complex	0.919071	0.925278	0.704386
MF_GO:0004582_dolichyl-phosphate+beta-D-mannosyltransferase+activity	0.919071	0.925278	0.704386
BP_GO:0008612_peptidyl-lysine+modification+to+hypusine	0.771824	0.771824	#N/A
BP_GO:0048199_vesicle+targeting,+to,+from+or+within+Golgi	0.288327	0.545325	0.182758
BP_GO:0008209_androgen+metabolic+process	0.115516	0.453097	0.0572731
BP_GO:0006621_protein+retention+in+ER+lumen	0.568852	0.568852	#N/A
MF_GO:0046923_ER+retention+sequence+binding	0.568852	0.568852	#N/A
CC_GO:0005665_DNA-directed+RNA+polymerase+II,+core+complex	0.809757	0.617884	0.826428
BP_GO:0035089_establishment+of+apical/basal+cell+polarity	0.416006	#N/A	0.416006
BP_GO:0001946_lymphangiogenesis	0.856717	#N/A	0.856717
BP_GO:0016926_protein+desumoylation	0.431314	0.414501	0.488079
BP_GO:0009059_macromolecule+biosynthetic+process	0.782768	0.782768	#N/A
BP_GO:0014056_regulation+of+acetylcholine+secretion	0.286664	#N/A	0.286664
MF_GO:0000268_peroxisome+targeting+sequence+binding	0.901466	0.850426	0.784121
MF_GO:0004911_interleukin-2+receptor+activity	0.135066	0.123086	0.343892
BP_GO:0043044_ATP-dependent+chromatin+remodeling	0.552808	#N/A	0.552808
MF_GO:0031491_nucleosome+binding	0.552808	#N/A	0.552808
BP_GO:0046668_regulation+of+retinal+cell+programmed+cell+death	0.802189	#N/A	0.802189
BP_GO:0002224_toll-like+receptor+signaling+pathway	0.153213	#N/A	0.153213
MF_GO:0019215_intermediate+filament+binding	0.79461	0.509813	0.872518
BP_GO:0019307_mannose+biosynthetic+process	0.950864	0.733392	0.956875
MF_GO:0004615_phosphomannomutase+activity	0.950864	0.733392	0.956875
BP_GO:0014031_mesenchymal+cell+development	0.873137	0.855712	0.709835
CC_GO:0030892_mitotic+cohesin+complex	0.785419	0.785419	#N/A
MF_GO:0003989_acetyl-CoA+carboxylase+activity	0.352583	0.510434	0.286857
BP_GO:0042036_negative+regulation+of+cytokine+biosynthetic+process	0.00944051	0.00298554	0.282915
MF_GO:0015056_corticotrophin-releasing+factor+receptor+activity	0.649411	0.607554	0.606064
MF_GO:0045309_protein+phosphorylated+amino+acid+binding	0.482298	0.83306	0.15147
MF_GO:0000774_adenyl-nucleotide+exchange+factor+activity	0.421108	0.401989	0.486239
BP_GO:0002790_peptide+secretion	0.23483	0.404725	0.216796
BP_GO:0035282_segmentation	0.997281	0.898339	0.996102
BP_GO:0045667_regulation+of+osteoblast+differentiation	0.68736	0.805441	0.432072
BP_GO:0031017_exocrine+pancreas+development	0.0654115	0.0654115	#N/A
BP_GO:0043433_negative+regulation+of+transcription+factor+activity	0.308197	0.146563	0.633577
BP_GO:0048537_mucosal-associated+lymphoid+tissue+development	0.124248	0.00907647	0.766814
MF_GO:0003884_D-amino-acid+oxidase+activity	0.313683	0.313683	#N/A
BP_GO:0032435_negative+regulation+of+proteasomal+ubiquitin-dependent	0.354443	0.852849	0.0572731
MF_GO:0005353_fructose+transmembrane+transporter+activity	0.480634	0.680041	0.295447
BP_GO:0006587_serotonin+biosynthetic+process+from+tryptophan	0.374353	0.405352	0.414953

BP_GO:0042427_serotonin+biosynthetic+process	0.374353	0.405352	0.414953
MF_GO:0004510_tryptophan+5-monooxygenase+activity	0.374353	0.405352	0.414953
BP_GO:0046883_regulation+of+hormone+secretion	0.678277	#N/A	0.678277
MF_GO:0004452_isopentenyl-diphosphate+delta-isomerase+activity	0.813265	0.789426	0.675094
BP_GO:0034644_cellular+response+to+UV	0.64705	0.236603	0.894404
MF_GO:0019911_structural+constituent+of+myelin+sheath	0.620681	0.620681	#N/A
BP_GO:0043508_negative+regulation+of+JNK+activity	0.489326	#N/A	0.489326
MF_GO:0030272_5-formyltetrahydrofolate+cyclo-ligase+activity	0.705747	0.705747	#N/A
BP_GO:0007258_JUN+phosphorylation	0.383186	0.625205	0.229451
BP_GO:0030328_prenylcysteine+catabolic+process	0.0739476	0.198101	0.115013
MF_GO:0016670_oxidoreductase+activity,+acting+on+sulfur+group+of+don	0.0739476	0.198101	0.115013
BP_GO:0048619_embryonic+hindgut+morphogenesis	0.169094	#N/A	0.169094
BP_GO:0001504_neurotransmitter+uptake	0.397708	0.251151	0.619033
BP_GO:0006488_dolichol-linked+oligosaccharide+biosynthetic+process	0.289603	0.195819	0.528255
BP_GO:0016577_histone+demethylation	0.493522	0.493522	#N/A
BP_GO:0048711_positive+regulation+of+astrocyte+differentiation	0.633955	0.386011	0.780295
BP_GO:0034612_response+to+tumor+necrosis+factor	0.0824841	0.420754	0.0386948
BP_GO:0045143_homologous+chromosome+segregation	0.24966	0.365461	0.2699
MF_GO:0004475_mannose-1-phosphate+guanylyltransferase+activity	0.598029	0.598029	#N/A
BP_GO:0042095_interferon-gamma+biosynthetic+process	0.659245	0.659245	#N/A
MF_GO:0043790_dimethyladenosine+transferase+activity	0.821366	0.821366	#N/A
MF_GO:0015036_disulfide+oxidoreductase+activity	0.0545644	#N/A	0.0545644
MF_GO:0004314_[acyl-carrier-protein]+S-malonyltransferase+activity	0.236118	0.236118	#N/A
CC_GO:0002080_acrosomal+membrane	0.671439	0.746445	0.485567
MF_GO:0004320_oleoyl-[acyl-carrier-protein]+hydrolase+activity	0.800211	0.423218	0.916934
CC_GO:0000137_Golgi+cis+cisterna	0.840391	#N/A	0.840391
CC_GO:0005915_zonula+adherens	0.764563	0.764563	#N/A
MF_GO:0030350_iron-responsive+element+binding	0.996178	#N/A	0.996178
MF_GO:0016888_endodeoxyribonuclease+activity,+producing+5'-phosphom	0.358836	0.147132	0.704067
MF_GO:0070052_collagen+V+binding	0.415704	#N/A	0.415704
BP_GO:0045063_T-helper+1+cell+differentiation	0.851666	0.902212	0.572045
MF_GO:0001566_non-kinase+phorbol+ester+receptor+activity	0.35305	#N/A	0.35305
CC_GO:0042588_zymogen+granule	0.140489	#N/A	0.140489
CC_GO:0031233_intrinsic+to+external+side+of+plasma+membrane	0.473911	0.3201	0.645724
BP_GO:0032319_regulation+of+Rho+GTPase+activity	0.369299	0.369299	#N/A
MF_GO:0001965_G-protein+alpha-subunit+binding	0.952317	0.952317	#N/A
MF_GO:0042813_Wnt+receptor+activity	0.680272	0.680272	#N/A
MF_GO:0019210_kinase+inhibitor+activity	0.286225	0.286225	#N/A
BP_GO:0006613_cotranslational+protein+targeting+to+membrane	0.944867	0.857696	0.882769
CC_GO:0005784_translocon+complex	0.944867	0.857696	0.882769
BP_GO:0009798_axis+specification	0.715389	#N/A	0.715389
MF_GO:0004360_glutamine-fructose-6-phosphate+transaminase+(isomerizi	0.238466	#N/A	0.238466
BP_GO:0055009_atrial+cardiac+muscle+morphogenesis	0.562989	0.562989	#N/A
MF_GO:0004422_hypoxanthine+phosphoribosyltransferase+activity	0.383266	0.606059	0.24502
BP_GO:0015746_citrate+transport	0.812178	#N/A	0.812178
MF_GO:0015137_citrate+transmembrane+transporter+activity	0.812178	#N/A	0.812178
BP_GO:0000076_DNA+replication+checkpoint	0.0444324	0.241524	0.0438797

BP_GO:0040009_regulation+of+growth+rate	0.393289	0.56582	0.291192
BP_GO:0009088_threonine+biosynthetic+process	0.22405	0.600566	0.0918708
MF_GO:0004795_threonine+synthase+activity	0.22405	0.600566	0.0918708
BP_GO:0048547_gut+morphogenesis	0.417399	#N/A	0.417399
BP_GO:0010592_positive+regulation+of+lamellipodium+assembly	0.485901	0.655959	0.325459
BP_GO:0045136_development+of+secondary+sexual+characteristics	0.0781757	0.236603	0.098591
BP_GO:0060058_positive+regulation+of+apoptosis+involved+in+mammary+	0.0781757	0.236603	0.098591
BP_GO:0043067_regulation+of+programmed+cell+death	0.59934	0.421599	0.709835
BP_GO:0018193_peptidyl-amino+acid+modification	0.824751	#N/A	0.824751
BP_GO:0043043_peptide+biosynthetic+process	0.910011	#N/A	0.910011
BP_GO:0006796_phosphate+metabolic+process	0.784466	0.784466	#N/A
BP_GO:0046950_cellular+ketone+body+metabolic+process	0.455518	0.455518	#N/A
MF_GO:0008260_3-oxoacid+CoA-transferase+activity	0.455518	0.455518	#N/A
BP_GO:0006119_oxidative+phosphorylation	0.0992969	#N/A	0.0992969
BP_GO:0042249_establishment+of+polarity+of+embryonic+epithelium	0.029307	0.35924	0.0102614
BP_GO:0002031_G-protein+coupled+receptor+internalization	0.923253	0.865585	0.819393
BP_GO:0046671_negative+regulation+of+retinal+cell+programmed+cell+dea	0.246179	0.0635684	0.709835
CC_GO:0016533_cyclin-dependent+protein+kinase+5+activator+complex	0.0310601	#N/A	0.0310601
MF_GO:0016534_cyclin-dependent+protein+kinase+5+activator+activity	0.0310601	#N/A	0.0310601
MF_GO:0034211_GTP-dependent+protein+kinase+activity	0.677626	#N/A	0.677626
MF_GO:0004534_5'-3'+exoribonuclease+activity	0.605091	0.460187	0.683019
BP_GO:0045627_positive+regulation+of+T-helper+1+cell+differentiation	0.440118	0.440118	#N/A
CC_GO:0030056_hemidesmosome	0.706567	0.968911	0.136307
MF_GO:0008177_succinate+dehydrogenase+(ubiquinone)+activity	0.825317	0.825317	#N/A
BP_GO:0021587_cerebellum+morphogenesis	0.722834	0.737314	0.579515
BP_GO:0007354_zygotic+determination+of+anterior/posterior+axis,+embry	0.716497	#N/A	0.716497
BP_GO:0001705_ectoderm+formation	0.555041	0.539164	0.53845
BP_GO:0015696_ammonium+transport	0.0246091	0.0246091	#N/A
MF_GO:0005143_interleukin-12+receptor+binding	0.860151	0.44955	0.951094
BP_GO:0045581_negative+regulation+of+T+cell+differentiation	0.601359	0.601359	#N/A
BP_GO:0000720_pyrimidine+dimer+repair+via+nucleotide-excision+repair	0.00194467	0.00194467	#N/A
MF_GO:0004909_interleukin-1,+Type+I,+activating+receptor+activity	0.708035	#N/A	0.708035
BP_GO:0010216_maintenance+of+DNA+methylation	0.636043	#N/A	0.636043
BP_GO:0030431_sleep	0.184638	0.184638	#N/A
BP_GO:0022612_gland+morphogenesis	0.817567	#N/A	0.817567
BP_GO:0045916_negative+regulation+of+complement+activation	0.0381206	0.517696	0.0053199
BP_GO:0006200_ATP+catabolic+process	0.661414	#N/A	0.661414
MF_GO:0016492_neurotensin+receptor+activity,+G-protein+coupled	0.192604	0.192604	#N/A
MF_GO:0005123_death+receptor+binding	0.494436	0.494436	#N/A
BP_GO:0015888_thiamin+transport	0.940618	0.752358	0.936328
MF_GO:0015234_thiamin+transmembrane+transporter+activity	0.940618	0.752358	0.936328
BP_GO:0007052_mitotic+spindle+organization	0.310644	0.788659	0.0665619
MF_GO:0004043_L-aminoadipate-semialdehyde+dehydrogenase+activity	0.422538	0.422538	#N/A
BP_GO:0006893_Golgi+to+plasma+membrane+transport	0.132407	0.20618	0.223849
MF_GO:0015270_dihydropyridine-sensitive+calcium+channel+activity	0.777436	0.777436	#N/A
BP_GO:0045821_positive+regulation+of+glycolysis	0.945253	0.945253	#N/A
MF_GO:0005176_ErbB-2+class+receptor+binding	0.858094	0.858094	#N/A

BP_GO:0006944_membrane+fusion	0.998583	#N/A	0.998583
MF_GO:0008432_JUN+kinase+binding	0.107617	0.410842	0.0630817
MF_GO:0042809_vitamin+D+receptor+binding	0.732484	0.218062	0.951101
MF_GO:0045523_interleukin-27+receptor+binding	0.0818533	#N/A	0.0818533
MF_GO:0050062_long-chain-fatty-acyl-CoA+reductase+activity	0.889283	#N/A	0.889283
BP_GO:0060060_post-embryonic+retina+morphogenesis+in+camera-type+e	0.0066137	0.012968	0.100279
MF_GO:0070064_proline-rich+region+binding	0.811325	0.852454	0.579747
BP_GO:0006370_mRNA+capping	0.998568	#N/A	0.998568
BP_GO:0008089_anterograde+axon+cargo+transport	0.621496	#N/A	0.621496
BP_GO:0048295_positive+regulation+of+isotype+switching+to+IgE+isotypes	0.937618	#N/A	0.937618
MF_GO:0005134_interleukin-2+receptor+binding	0.935351	0.858407	0.858228
MF_GO:0015501_glutamate:sodium+symporter+activity	0.377003	0.377003	#N/A
BP_GO:0031848_protection+from+non-homologous+end+joining+at+telome	0.776949	0.898339	0.422731
BP_GO:0015889_cobalamin+transport	0.286984	#N/A	0.286984
BP_GO:0042048_olfactory+behavior	0.589507	0.588991	0.537548
CC_GO:0017177_alpha-glucosidase+II+complex	0.941986	0.941986	#N/A
BP_GO:0031630_regulation+of+synaptic+vesicle+fusion+to+presynaptic+me	0.0540472	0.0540472	#N/A
MF_GO:0004307_ethanolaminephosphotransferase+activity	0.809222	0.634223	0.8144
BP_GO:0051295_establishment+of+meiotic+spindle+localization	0.459924	0.459924	#N/A
BP_GO:0032330_regulation+of+chondrocyte+differentiation	0.547293	0.547293	#N/A
BP_GO:0006398_histone+mRNA+3'-end+processing	0.870664	0.870664	#N/A
BP_GO:0032753_positive+regulation+of+interleukin-4+production	0.904621	0.942143	0.609286
BP_GO:0047484_regulation+of+response+to+osmotic+stress	0.226317	0.226317	#N/A
MF_GO:0042800_histone+lysine+N-methyltransferase+activity+(H3-K4+spec	0.479304	0.626958	0.34522
BP_GO:0042594_response+to+starvation	0.826044	0.826044	#N/A
BP_GO:0015858_nucleoside+transport	0.265855	0.427464	0.240967
MF_GO:0003823_antigen+binding	0.705989	#N/A	0.705989
BP_GO:0042267_natural+killer+cell+mediated+cytotoxicity	0.412365	0.255992	0.633577
BP_GO:0006659_phosphatidylserine+biosynthetic+process	0.96972	0.96972	#N/A
BP_GO:0006429_leucyl-tRNA+aminoacylation	0.272752	0.272752	#N/A
MF_GO:0004823_leucine-tRNA+ligase+activity	0.272752	0.272752	#N/A
BP_GO:0045742_positive+regulation+of+epidermal+growth+factor+recepto	0.0108314	0.0576948	0.0468628
MF_GO:0017057_6-phosphogluconolactonase+activity	0.558803	0.558803	#N/A
BP_GO:0030147_natriuresis	0.140356	#N/A	0.140356
MF_GO:0017077_oxidative+phosphorylation+uncoupler+activity	0.82725	0.82725	#N/A
MF_GO:0008746_NAD(P)+transhydrogenase+activity	0.528917	0.528917	#N/A
MF_GO:0008750_NAD(P)++transhydrogenase+(AB-specific)+activity	0.528917	0.528917	#N/A
BP_GO:0051302_regulation+of+cell+division	0.280554	0.280554	#N/A
BP_GO:0007042_lysosomal+lumen+acidification	0.0730198	0.0730198	#N/A
MF_GO:0008339_MP+kinase+activity	0.850854	0.850854	#N/A
BP_GO:0006301_postreplication+repair	0.240114	0.378373	0.24502
MF_GO:0051864_histone+demethylase+activity+(H3-K36+specific)	0.436615	0.436615	#N/A
BP_GO:0016540_protein+autoprocessing	0.594212	0.646975	0.483722
MF_GO:0004310_farnesyl-diphosphate+farnesyltransferase+activity	0.556406	#N/A	0.556406
MF_GO:0004329_formate-tetrahydrofolate+ligase+activity	0.103148	0.241698	0.138158
MF_GO:0031489_myosin+V+binding	0.16707	0.0903739	0.488312
BP_GO:0001839_neural+plate+morphogenesis	0.438896	#N/A	0.438896

MF_GO:0042289_MHC+class+II+protein+binding	0.549462	0.945965	0.0760677
BP_GO:0016048_detection+of+temperature+stimulus	0.129169	#N/A	0.129169
BP_GO:0050906_detection+of+stimulus+involved+in+sensory+perception	0.271711	#N/A	0.271711
BP_GO:0007589_body+fluid+secretion	0.822588	#N/A	0.822588
BP_GO:0042773_ATP+synthesis+coupled+electron+transport	0.927888	0.916654	0.752547
BP_GO:0035117_embryonic+arm+morphogenesis	0.539108	0.539108	#N/A
BP_GO:0009296_flagellum+assembly	0.772692	0.772692	#N/A
CC_GO:0031514_motile+secondary+cilium	0.772692	0.772692	#N/A
BP_GO:0030910_olfactory+placode+formation	0.857567	#N/A	0.857567
BP_GO:0043537_negative+regulation+of+blood+vessel+endothelial+cell+mi	0.338496	0.0373846	0.883245
BP_GO:0030330_DNA+damage+response,+signal+transduction+by+p53+clas	0.85922	0.6074	0.894404
BP_GO:0019478_D-amino+acid+catabolic+process	0.196697	0.196697	#N/A
BP_GO:0032225_regulation+of+synaptic+transmission,+dopaminergic	0.248095	0.322692	0.307195
CC_GO:0000164_protein+phosphatase+type+1+complex	0.422073	#N/A	0.422073
BP_GO:0002358_B+cell+homeostatic+proliferation	0.111165	0.111165	#N/A
BP_GO:0046013_regulation+of+T+cell+homeostatic+proliferation	0.475687	0.123086	0.858228
BP_GO:0021510_spinal+cord+development	0.501391	0.315681	0.685714
BP_GO:0007320_insemination	0.368668	0.368668	#N/A
MF_GO:0008171_O-methyltransferase+activity	0.15701	0.0213512	0.726017
MF_GO:0004535_poly(A)-specific+ribonuclease+activity	0.764906	0.730506	0.657859
MF_GO:0019767_IgE+receptor+activity	0.0983873	#N/A	0.0983873
BP_GO:0006083_acetate+metabolic+process	0.458055	0.458055	#N/A
BP_GO:0042129_regulation+of+T+cell+proliferation	0.381542	0.525471	0.311548
BP_GO:0048863_stem+cell+differentiation	0.852592	#N/A	0.852592
CC_GO:0045298_tubulin+complex	0.647953	0.647953	#N/A
BP_GO:0050862_positive+regulation+of+T+cell+receptor+signaling+pathwa	0.254609	0.254609	#N/A
BP_GO:0005989_lactose+biosynthetic+process	0.486724	#N/A	0.486724
BP_GO:0030516_regulation+of+axon+extension	0.973558	0.989172	0.670814
BP_GO:0032924_activin+receptor+signaling+pathway	0.817105	0.582383	0.857878
MF_GO:0017002_activin+receptor+activity	0.817105	0.582383	0.857878
MF_GO:0030515_snoRNA+binding	0.141168	0.141168	#N/A
BP_GO:0045124_regulation+of+bone+resorption	0.48812	#N/A	0.48812
MF_GO:0003835_beta-galactoside+alpha-2,6-sialyltransferase+activity	0.192084	#N/A	0.192084
MF_GO:0019207_kinase+regulator+activity	0.450297	#N/A	0.450297
BP_GO:0043569_negative+regulation+of+insulin-like+growth+factor+recept	0.526749	0.58352	0.453447
BP_GO:0045076_regulation+of+interleukin-2+biosynthetic+process	0.37722	0.525471	0.305889
CC_GO:0030893_meiotic+cohesin+complex	0.615968	#N/A	0.615968
BP_GO:0043297_apical+junction+assembly	0.691831	0.932042	0.216776
CC_GO:0005838_proteasome+regulatory+particle	0.261558	0.307346	0.344116
CC_GO:0032998_Fc-epsilon+receptor+I+complex	0.219011	#N/A	0.219011
MF_GO:0004918_interleukin-8+receptor+activity	0.374086	#N/A	0.374086
BP_GO:0042487_regulation+of+odontogenesis+of+dentine-containing+toot	0.815016	#N/A	0.815016
CC_GO:0000783_nuclear+telomere+cap+complex	0.801175	0.898339	0.469633
CC_GO:0005712_chiasma	0.353266	0.353266	#N/A
CC_GO:0031201_SNARE+complex	0.697898	0.612697	0.672272
BP_GO:0032788_saturated+monocarboxylic+acid+metabolic+process	0.706241	0.726795	0.564924
BP_GO:0032789_unsaturated+monocarboxylic+acid+metabolic+process	0.706241	0.726795	0.564924

BP_GO:0009063_amino+acid+catabolic+process	0.145656	0.145656	#N/A
BP_GO:0046666_retinal+cell+programmed+cell+death	0.314831	0.314831	#N/A
MF_GO:0004525_ribonuclease+III+activity	0.966617	0.973425	0.745243
MF_GO:0033842_N-acetyl-beta-glucosaminyl-glycoprotein+4-beta-N-acetylg	0.220816	0.143589	0.489813
BP_GO:0021912_regulation+of+transcription+from+RNA+polymerase+II+prc	0.421627	0.447124	0.441212
BP_GO:0045686_negative+regulation+of+glial+cell+differentiation	0.421627	0.447124	0.441212
BP_GO:0045687_positive+regulation+of+glial+cell+differentiation	0.421627	0.447124	0.441212
BP_GO:0006703_estrogen+biosynthetic+process	0.486326	0.486326	#N/A
BP_GO:0035129_post-embryonic+hindlimb+morphogenesis	0.329556	0.671974	0.142163
BP_GO:0050878_regulation+of+body+fluid+levels	0.641591	#N/A	0.641591
MF_GO:0003720_telomerase+activity	0.737458	0.788716	0.538389
BP_GO:0021524_visceral+motor+neuron+differentiation	0.724676	0.638596	0.687518
BP_GO:0050801_ion+homeostasis	0.306217	#N/A	0.306217
CC_GO:0005785_signal+recognition+particle+receptor+complex	0.922631	#N/A	0.922631
CC_GO:0016327_apicolateral+plasma+membrane	0.281548	0.296355	0.388054
BP_GO:0009912_auditory+receptor+cell+fate+commitment	0.427686	0.516273	0.382186
BP_GO:0060023_soft+palate+development	0.349805	0.349805	#N/A
MF_GO:0016299_regulator+of+G-protein+signaling+activity	0.298329	#N/A	0.298329
BP_GO:0046068_cGMP+metabolic+process	0.611046	#N/A	0.611046
[truncated: 1,131,615 more chars]
